# CpG Island Methylator Phenotype Modulates the Immune Response of the Tumor Microenvironment and Influences the Prognosis of Pancreatic Cancer Patients

**DOI:** 10.1155/2021/2715694

**Published:** 2021-11-28

**Authors:** Gang Ning, Yongqiang Li, Wenji Chen, Wenjuan Tang, Diwen Shou, Qingling Luo, Huiting Chen, Yongjian Zhou

**Affiliations:** Department of Gastroenterology and Hepatology, Guangzhou Digestive Diseases Center, Guangzhou First People's Hospital, South China University of Technology, Guangzhou, Guangdong Province, China

## Abstract

**Background:**

CpG island methylator phenotype (CIMP), featured with concurrent and widespread hypermethylation of a cluster of CpGs, has been reported to play an important role in carcinogenesis. Limited studies have investigated the role of CIMP in pancreatic cancer (PC). The aim of this study was to explore the CIMP in PC patients and its impact on the immune response of the tumor microenvironment and prognosis.

**Methods:**

DNA methylation, somatic mutation, mRNA, and corresponding clinical data of PC patients were downloaded from TCGA (184 patients) and the ICGC (264 patients). Univariate and multivariate regression analyses were used to identify prognosis-related CpGs. Consensus clustering analysis was used for identification of the CIMP in PC patients. ESTIMATE and CIBORORT were used for estimation of the tumor microenvironment (TME) in PC patients.

**Results:**

In the TCGA PC cohort, 22,450 differential CpGs, including 12,937 hypermethylated CpGs and 9,513 hypomethylated CpGs, were identified between 184 PC patients and 10 normal controls. Univariate and multivariate Cox analysis further screened out 72 OS-related CpGs, and three distinct CIMP groups with distinctly different prognosis and molecular features, including the CIMP-L subgroup, CIMP-M subgroup, and CIMP-H subgroup, were identified based on unsupervised consensus clustering analysis of these CpGs. Patients of the CIMP-H subgroup had poorer OS and RFS, while patients of the CIMP-L subgroup had better OS and RFS. The CIMP status was also an independent prognostic factor for OS and PFS. In molecular features, significantly higher somatic mutation burden and tumor mutational burden were found in patients of the CIMP-H subgroup compared to those of the CIMP-L subgroup. Besides, lower stromal score, immune score, and higher cancer stemness indices and tumor purity were also found in patients of the CIMP-H subgroup compared to those of the CIMP-L subgroup. Correspondingly, significant total T cells, total B cells, CD8 T cells, memory CD4 T cells, and higher regulatory T cells were found in patients of the CIMP-H subgroup. Moreover, significantly lower expression of immune checkpoint genes, such as PD-1, CTLA4, CD86, VTCN1, and LAG-3, was also found in patients of the CIMP-H subgroup compared to those of the CIMP-L subgroup. In the end, we validated the CIMP status in PC patients of the ICGC dataset.

**Conclusion:**

The CIMP may modulate the immune response of the tumor microenvironment and influence the prognosis of pancreatic cancer patients, which may help to make an assertion to provide specific and efficient treatment options for patients of different subtypes.

## 1. Introduction

Pancreatic cancer (PC) is the fifteenth most common cancer and the seventh leading cause of cancer-related death worldwide, which is reported to have poor prognosis and high mortality rate [1]. Lots of effort was put into understanding the underlying mechanisms of PC and new treatments were investigated, but marginal improvement of survival was observed in PC patients [2]. Neureiter et al. reported the median survival of PC patients to be 6 months and the 5-year survival rate to be as low as 6%, attributing the cause to the lack of reliable early diagnostic markers and the nature of high aggressiveness and drug resistance of the disease itself [3]. Of note, only about 10% of PC patients were reported with family histories [4], indicating the etiology of PC to be more likely sporadic than genetic and suggesting epigenetic alterations might take part in the development and progression of PC.

DNA methylation is one of the main epigenetic modifications and plays an important role in the development and progression of various sorts of malignant tumors [5]. During the process of oncogenesis, aberrant DNA methylation of CpG islands occurs in the promoter regions of genes, such as hypermethylation surrounding the promoters of tumor suppressor genes (TSGs) and hypomethylation of promoters of oncogenes, resulting in transcriptional silence of TSGs and overexpression of oncogenes [6]. Aberrant DNA methylation of nonpromoter genes also contributes to the formation of intratumoral heterogeneity [7]. To date, many gene mutations with deregulated DNA methylation have been identified in PC patients. Treatments targeting these genes were reported to significantly improve the overall survival in PC mice models [8, 9].

The CpG island methylator phenotype (CIMP), first discovered and validated in colorectal cancer, is featured with concurrent and widespread hypermethylation of a cluster of CpGs in distinct cancer subtypes. CIMP affects the intergenic regions of the whole genome and leads to chromosomal instability, which is thought to play an important role in carcinogenesis [10]. To date, the CIMP phenotype has been identified in many kinds of tumors, including PC [11]. Subtypes with different CIMP patterns showed distinct epidemiological, clinical-pathological, and genomic characteristics [12, 13]. However, the limitation of earlier studies on the CIMP of PC patients was obvious for the limited patient samples or CpG sites used in these studies [5, 14]. Few of these studies validated their findings in independent cohorts and further explored the association of CIMP status with the tumor microenvironment (TME) in PC patients [11]. In the present study, we identified and validated three distinct methylation subgroups of PC patients, termed the CIMP-L subgroup, CIMP-M subgroup, and CIMP-H subgroup, with data downloaded from The Cancer Genome Atlas (TCGA) and the International Cancer Genome Consortium (ICGC), and further investigated their impact on the immune response of the tumor microenvironment and prognosis. As identification of clinically relevant cancer subtypes based on DNA methylation patterns is an important computational problem in medicine, CIMP might help to make an assertion to provide specific and efficient treatment options for patients of different subtypes.

## 2. Methods and Materials

### 2.1. Ethics Statement

All the data analyzed in the present study were obtained from TCGA and the ICGC. Informed consent had already been obtained from the patients before the present study.

### 2.2. Data Acquisition from TCGA

Level-3 DNA methylation data of 184 PC patients and 10 normal controls were downloaded from TCGA (https://cancergenome.nih.gov/, 2020-04-20).

The somatic mutation data of 177 PC patients measured by whole-exome sequencing were downloaded from TCGA, and maftools package was used to analyze these data [15]. Patients harboring missense mutations, nonsense mutations, multiple hits, splice-site mutations, frameshift insertions, frameshift deletions, in-frame insertions, or in-frame deletions were considered as positive for mutation. Moreover, tumor mutational burden (TMB), regarded as a promising biomarker for immunotherapy responses, was also calculated with the method described in the previous study [16].

mRNA expression data of 178 PC patients were also obtained from TCGA. The average gene expression was adopted in case of duplicates, and the gene expression data were normalized with the scale method [17]. Besides, more than 20% of the samples with missing gene expression were removed.

Meanwhile, corresponding clinical-pathological data of 184 PC patients, including gender, age, T stage (T), N stage (N), M stage (M), TNM stage, overall survival (OS) status and time, and progression-free survival (PFS) status and time, were also downloaded.

### 2.3. Methylation Data Processing and Identification of the CIMP in PC Patients

*β* value for those CpG probes that were either mapped against chromosomes *X* and *Y* were removed so as to avoid gender biases, and more than 20% of samples with missing *β* value were also removed. Besides, those probes mapped to SNP within 10 bp of interrogated CpG sites were also removed [18]. The Wilcoxon test was performed to identify differential CpGs between PC patients and normal controls. With *P* < 0.05, a CpG would be considered differentially methylated between primary tumor and normal samples.

To evaluate the CIMP phenomenon in PC patients, first, OS-related CpGs were selected by univariate Cox regression analysis on the basis of the differential CpGs between 184 PC patients and 10 normal samples. Then, multivariate Cox regression analysis was further performed to identify these most OS-related CpGs. After that, consensus clustering analysis for the unsupervised class of 184 PC patients based on the expression similarity of OS-related CpGs was performed with the ConsensusClusterPlus packages. Besides, principal component analysis (PCA) was also performed to examine whether the clusters of PC patients were suitable with the “limma” package.

### 2.4. Estimation of Stromal and Immune Cells in Malignant Tumor Tissues Using Expression Data (ESTIMATE)

ESTIMATE was a tool used for predicting tumor purity, the presence of infiltrating stromal/immune cells in tumor tissues with mRNA expression data. Based on single sample Gene Set Enrichment Analysis (ssGSEA), ESTIMATE could generate three scores: stromal score (that captured the presence of stroma in tumor tissue), immune score (that represented the infiltration of immune cells in tumor tissue), and estimate score (that negatively correlated with tumor purity) [19].

### 2.5. Cancer Stemness Indices of PC Patients

Previously, Malta et al. extracted transcriptomic and epigenetic feature sets derived from nontransformed pluripotent stem cells and their differentiated progeny using an innovative one-class logistic regression machine learning algorithm (OCLR). As a result, they identified four cancer stemness indices, including mRNAsi, epigenetically regulated mRNAsi (EREG-mRNAsi), mDNAsi, and EREG-mDNAsi, for assessing the degree of oncogenic dedifferentiation [20]. Based on their study, we could attain the four stemness indices of each PC patient in the TCGA database.

### 2.6. CIBORORT

22 kinds of tumor-infiltrating immune cells of each PC patient were calculated with CIBERSORT (https://cibersort.stanford.edu), an online tool designed for estimating the abundances of tumor-infiltrating immune cells with transcriptomic data [21].

### 2.7. Gene Set Enrichment Analysis (GSEA)

To explore the underlying mechanism exploited by the CIMP status to influence the prognosis of PC patients, GSEA analysis (Version: 4.2; http://software.broadinstitute.org/gsea/index.jsp) was performed. As a result, the difference of KEGG pathways between PC patients with distinct CIMP status was identified [22].

### 2.8. Validation of CIMP Status in PC Patients of the ICGC Dataset

To independently test the CIMP status in PC patients, DNA methylation data of 264 PC patients, somatic mutation data of 264 PC patients, mRNA expression data of 175 PC patients, and clinical-pathological parameters of 264 PC patients were downloaded from the ICGC (https://dcc.icgc.org/, 2020-04-20). Processing of methylation data, somatic mutation data, and mRNA expression data were similar to those in TCGA.

### 2.9. Data Analysis Flow Chart

**Figure 1:**
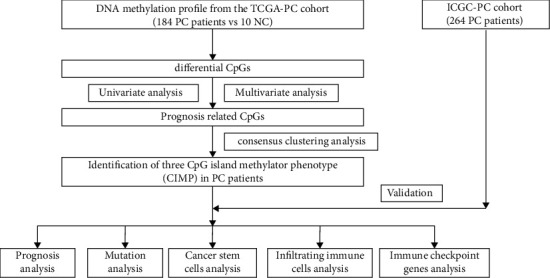
The workflow chart of the present study.

To make our study better understood, a workflow of the study is depicted and shown in Figure 1.

### 2.10. Statistical Analysis

GraphPad Prism 6 (GraphPad Software) and R software (version 3.5.1) were used for statistical analysis and plotting graphs. The association between CIMP status and clinical-pathological features was analyzed with the chi-square test. One-way ANOVA analysis was carried out to compare the difference of TMB, stromal score, immune score, estimate score, tumor-infiltrating immune cells, expression of chemokines, and immune checkpoint genes among PC patients with different CIMP statuses. Univariate and multivariate Cox regression analyses were performed to analyze the prognostic value of CIMP status. Kaplan–Meier analysis with a log-rank test was performed to analyze the difference of OS or PFS among patients with different CIMP statuses. *P* < 0.05 was considered as statistically significant.

## 3. Results

### 3.1. Identification of the CIMP in PC Patients

**Figure 2:**
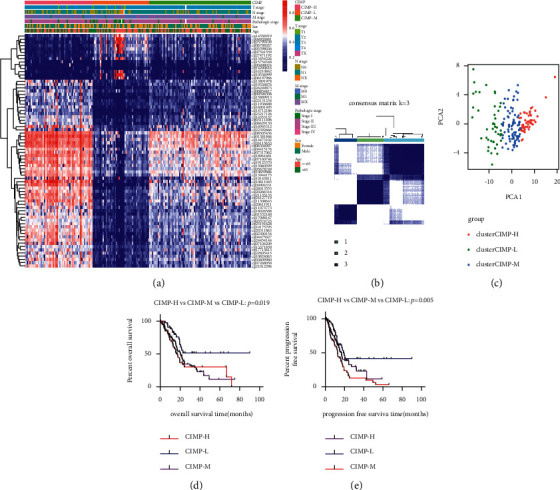
Identification of the CIMP in 184 PC patients. Heat map of expression of 72 overall survival-related CpGs in three methylation clusters (a); three methylation clusters (CIMP-L subgroup (*n* = 46), CIMP-M subgroup (*n* = 82), and CIMP-H subgroup (*n* = 56)) were generated via k-means consensus clustering (b); principal component analysis of 184 PC patients clustered as 3 subgroups (c); and Kaplan–Meier analysis of OS and PFS time among patients with different CIMP statuses (d-e).

To identify the CIMP in PC patients, we first screened out the differential CpGs between PC samples and normal samples with the DNA methylation data of 184 PC patients and 10 normal controls downloaded from TCGA. In total, 22,450 differential CpGs were identified (*P* < 0.05). Among these CpGs, 12,937 were hypermethylated CpGs (log2FC > 0), while 9,513 were hypomethylated CpGs (log2FC < 0) (Supplementary Material 1). The most significant 25 hypermethylated CpGs and the 25 most hypomethylated CpGs are shown in Supplementary Figure 1 between PC patients and normal controls. Next, 3102 CpGs were found to be related with OS in PC patients by univariate Cox analysis (*P* < 0.05, Supplementary Material 2). Among these CpGs, 2858 CpGs were found to be associated with worse OS of PC patients (HR > 1), while 244 were found to be associated with better OS of PC patients (HR < 1). The most significant 25 worse OS-related CpGs and the 25 better OS-related CpGs in PC patients are shown in Supplementary Figure 2. Then, in order to identify the most OS-related CpGs, only 1073 out of 3102 OS-related CpGs with *P* < 0.01 were used for multivariate Cox analysis, and 72 CpGs were found to be the most OS-related CpGs (Supplementary Material 3). Based on the unsupervised consensus clustering analysis, 184 PC patients were clustered into three distinct groups (Figures 2(a)–2(c)), namely, the CIMP-L subgroup (*n* = 46), CIMP-M subgroup (*n* = 82), and CIMP-H subgroup (*n* = 56). The methylation level of the CIMP-L subgroup was low, while patients of the CIMP-H subgroup had widespread hypermethylated CpGs.

Next, the associations between CIMP status and clinical characteristics were analyzed. As shown in Table 1, there were more patients with advanced T stage and TNM stage in the CIMP-M subgroup and CIMP-H subgroup compared to those in the CIMP-L subgroup (all *P* < 0.05, Table 1). Besides, Kaplan–Meier analysis showed that there were significant differences in OS and PFS among PC patients from different CIMP statuses. The patients of the CIMP-H subgroup had poorer OS and RFS, while the patients of the CIMP-L subgroup had better OS and RFS (all *P* < 0.05, Figures 2(d) and 2(e)). Moreover, univariate Cox analysis indicated that CIMP status was significantly related with OS and PFS, and multivariate Cox analysis also suggested that CIMP status was an independent prognostic factor for OS and PFS of PC patients after adjusting for gender, age, T state, N stage, M stage, and TNM stage (all *P* < 0.05, Table 2).

### 3.2. Mutational Landscapes of PC Patients with Different CIMP Statuses

**Figure 3:**
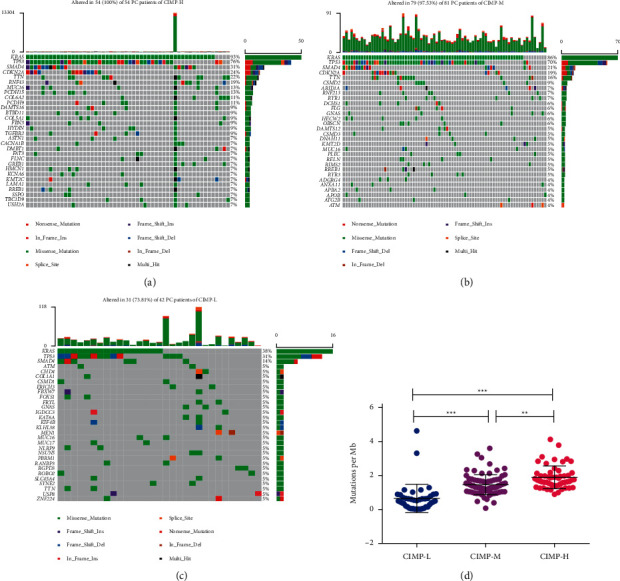
Mutational landscapes of PC patients with different CIMP statuses. 30 most significantly mutated genes in the PC patients from the CIMP-H subgroup (a), CIMP-M subgroup (b), and CIMP-L subgroup (c); expression of TMB among PC patients with different CIMP statuses (d).

A number of mutated genes with deregulated DNA methylation had been identified to play important roles in the development and progression of PC [8]. The association of CIMP status with gene mutations was analyzed with somatic mutation data of 177 PC patients downloaded from TCGA. As shown in Figure 3, there were significantly higher somatic mutation burdens among patients with different CIMP statuses. All the PC patients of the CIMP-H subgroup (*n* = 54) had gene mutation, and 79 out of 81 PC patients of the CIMP-M subgroup had gene mutation, while only 31 out of 41 PC patients of the CIMP-L subgroup had gene mutation. Significantly higher somatic mutation burdens in KRAS, TP53, SMAD4, CDKN2A, and TTN were observed in patients of the CIMP-H group (Figures 3(a)–3(c)), which had been shown to be major driver genes in PC [23]. Similarly, a significant difference of tumor mutational burden (TMB), serving as a biomarker of immunotherapy responses, was also found among patients with different CIMP statuses. Higher TMB was found in patients of the CIMP-H subgroup, while lower TMB was observed in patients with CIMP-L status (Figure 3(d)).

### 3.3. Landscape of TME in PC Patients with Distinct CIMP Status

**Figure 4:**
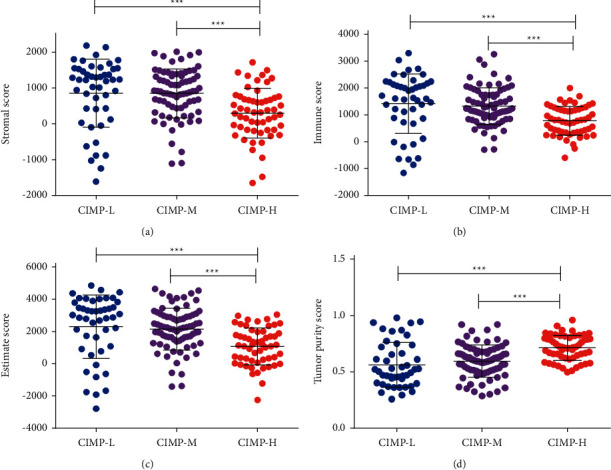
Association of CIMP status with the tumor microenvironment in PC patients with distinct CIMP status. Expression of stromal score (a), immune score (b), estimate score (c), and tumor purity (d) among PC patients with distinct CIMP status.

Consisting of cancer cells, stromal cells, and extracellular components, the TME has been demonstrated to play indispensable roles in tumorigenesis, progression, metastasis, recurrence, and drug resistance of PC [24]. The difference of TME in patients with distinct CIMP status was also analyzed. As shown in Figure 4, significantly lower stromal score, immune score, and estimate score were found in patients of the CIMP-H subgroup, while significantly higher stromal score, immune score, and estimate score were found in patients of the CIMP-L subgroup (Figures 4(a)–4(c)). Similarly, a significantly higher tumor purity score was also observed in patients of the CIMP-H subgroup (Figure 4(d)).

**Figure 5:**
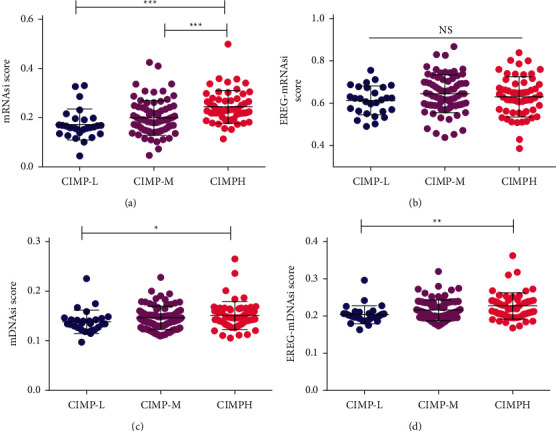
Association of CIMP status with cancer stemness indices of PC patients. Expression of mRNAsi (a), EREG-mRNAsi (b), mDNAsi (c), and EREG-mDNAsi (d) among PC patients with different CIMP status.

Cancer stem cells (CSCs) were cancer cells that possessed the ability to give rise to all tumor cell types, and CSCs were considered to be responsible for tumor growth, metastasis and recurrence, and resistance to chemotherapy and radiation therapy. The association of CIMP status with cancer stemness indices was explored. As expected, significantly higher tumor stemness indices, including mRNAsi score, mDNAsi score, and EREG-mDNAsi score, were found in patients of the CIMP-H subgroup compared to patients of the CIMP-L subgroup (Figures 5(a), 5(c), and 5(d)).

**Figure 6:**
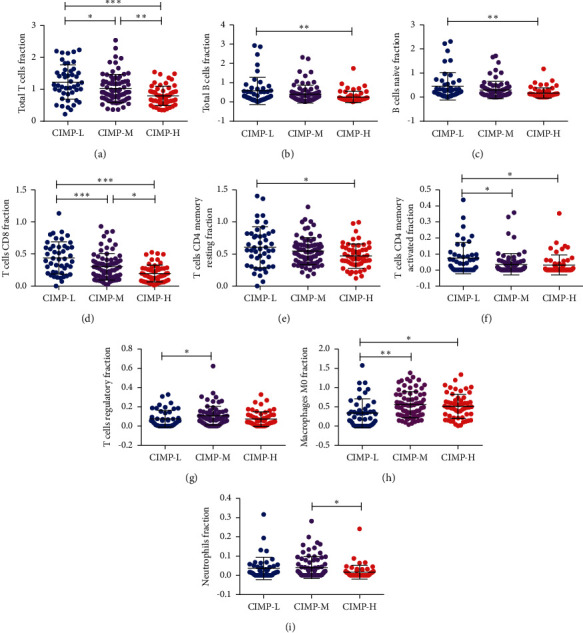
Landscapes of tumor-infiltrating immune cells in PC patients with different CIMP statuses. Expression of total T cells (a), total B cells (b), naive B cells (c), CD8 T cells (d), resting memory CD4 T cells (e), activated memory CD4 T cells (f), regulatory T cells (g), and M0 macrophages (h) among PC patients with different CIMP statuses.

Next, we analyzed the difference of tumor-infiltrating immune cells among patients with distinct CIMP statuses. As shown in Figure 6, significantly lower total T cells, total B cells, naive B cells, CD8 T cells, CD4 T cells, resting memory CD4 T cells, and activated memory CD4 T cells were found in patients of the CIMP-H subgroup compared to patients of the CIMP-L subgroup (Figures 6(a)–6(f)). Besides, significantly higher M0 macrophages were found in patients of the CIMP-H subgroup, and significantly higher regulatory T cells were found in patients of the CIMP-M subgroup compared to patients of the CIMP-L subgroup (Figures 6(g)–6(h)). It has been reported that different kinds of immune cell subsets are recruited into the TME via interactions between chemokines and their chemokine receptors [25]. We further analyzed the difference of expression of 58 kinds of chemokines among patients with different CIMP statuses. As summarized in Table 3, in line with the results of tumor-infiltrating immune cells, 31 kinds of chemokines, such as CCL2, XCL2, CCR2, CCL5, and CCR5, were found to be overexpressed in patients of the CIMP-L subgroup, while only 7 kinds of chemokines, such as CXCL14 and CXCL16, were found to be increased in patients of the CIMP-M and CIMP-H subgroup. Taken together, these results suggest that CIMP modulates the immune response of the tumor microenvironment of PC patients.

### 3.4. Expression of Immune Checkpoint Genes in PC Patients with Different CIMP Statuses

**Figure 7:**
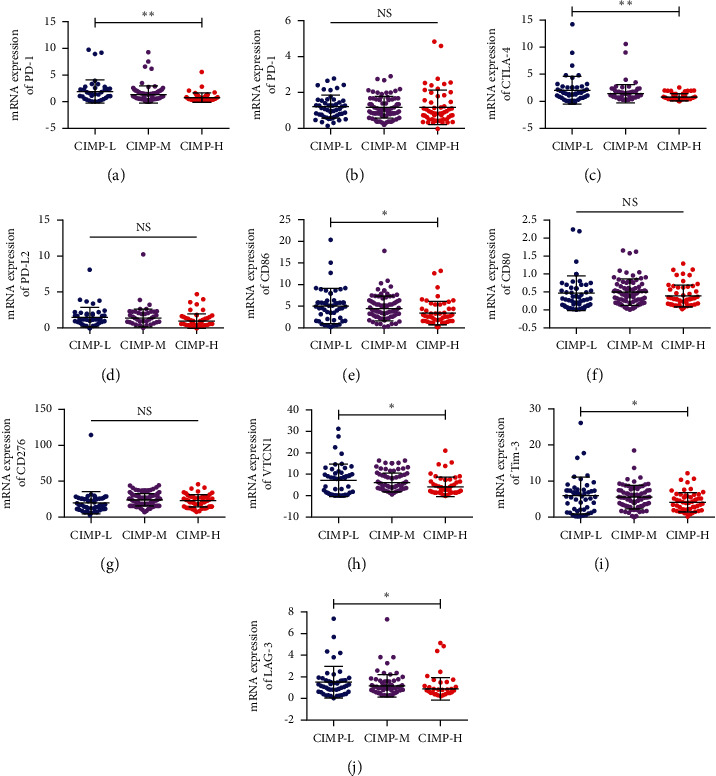
Expression of 10 immune checkpoint genes in PC patients with different CIMP statuses. Expression of PD-1 (a), PD-L1 (b), CTLA4 (c), PD-L2 (d), CD86 (e), CD80 (f), CD276 (g), VTCN1 (h), Tim-3 (i), and LAG-3 (j) in PC patients with distinct CIMP status.

The advent of immunotherapy, especially checkpoint inhibitor-based immunotherapy, has revolutionized cancer treatments, especially for patients with advanced tumors. These treatments functioned through the blockade of immunosuppressive checkpoints, so the expression of these immune checkpoint genes was necessary for checkpoint inhibitor immunotherapy [26]. The difference of expression of 10 immune checkpoint genes (including PD-1, PD-L1, CTLA4, PD-L2, CD86, CD80, CD276, VTCN1, Tim-3, and LAG-3) in PC patients with different CIMP status was further analyzed. As shown in Figure 7, the expression of PD-1, CTLA4, CD86, VTCN1, and LAG-3 of PC patients of the CIMP-H subgroup was significantly lower than that of patients of the CIMP-L subgroup (Figures 7(a), 7(c), 7(e), 7(h)–7(j)). These results may indicate that checkpoint inhibitor immunotherapy is less effective in patients of the CIMP-H subgroup as they showed less expression of immune checkpoint genes.

### 3.5. Potential Mechanism by Which CIMP Status Influences the Prognosis of PC Patients

**Figure 8:**
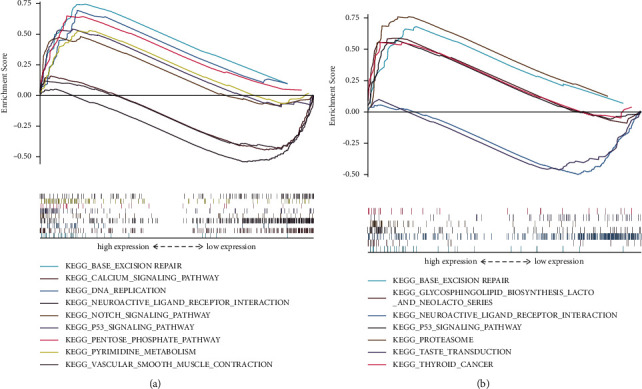
Potential mechanism by which CIMP status influences the prognosis of PC patients. Significant KEGG pathways enriched in PC patients of the CIMP-H subgroup (a); significant KEGG pathways enriched in PC patients of the CIMP-M subgroup (b).

GSEA analysis was performed to explore the underlying biological mechanism by which CIMP influenced the prognosis of PC patients. As shown in Figure 8, KEGG pathways, such as the “p53 signaling pathway,” “notch signaling pathway,” “calcium signaling pathway,” “DNA replication,” and “base excision repair,” were found to be significantly enriched in patients of the CIMP-H subgroup compared to patients of the CIMP-L patients (Figure 8(a)). Similarly, the “p53 signaling pathway,” “base excision repair,” and “proteasome” were found to be significantly enriched in patients of the CIMP-M subgroup compared to patients of the CIMP-L subgroup (Figure 8(b)). These results may suggest that the CIMP status may influence the prognosis of PC patients by regulating the aforementioned biological process.

### 3.6. Validation of CIMP Status in PC Patients of the ICGC Dataset

**Figure 9:**
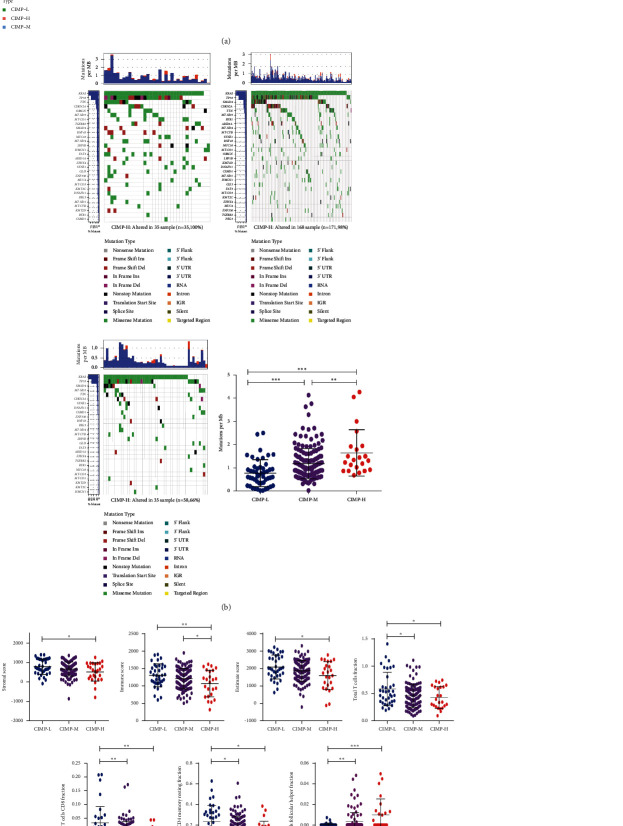
Validation of CIMP status in 264 PC patients of the ICGC dataset. Validation of three CIMP subgroups in PC patients of the ICGC dataset and the association of CIMP status with clinical-pathologic parameters and prognosis (a); mutational landscapes of three CIMP subgroups and expression of TMB among PC patients with different CIMP statuses (b); expression of stromal score, immune score, estimate score, and tumor-infiltrating immune cells among PC patients with distinct CIMP status (c); and expression of 4 immune checkpoint genes in PC patients with different CIMP statuses (d).

To independently test the CIMP status in PC patients, DNA methylation data of 264 PC patients, somatic mutation data of 264 PC patients, mRNA expression of 175 PC patients, and clinical-pathological parameters of 264 PC patients were downloaded from the ICGC (https://dcc.icgc.org/). Unsupervised consensus clustering analysis was also performed for 264 PC patients based on the expression of these 72 OS-related CpGs. Similarly, these 264 PC patients were also clustered into three distinct groups (Figure 9(a)). There were 58 PC patients in the CIMP-L subgroup, 171 PC patients in the CIMP-M subgroup, and 35 PC patients in the CIMP-H subgroup. Besides, significant associations between CIMP status and clinical-pathological characteristics, including age, T state, N stage, M stage, and TNM stage, were also observed (Table 4). Moreover, Kaplan–Meier analysis showed that there were significant differences in OS among PC patients with different CIMP statuses. Patients of the CIMP-H subgroup had poorer OS, while the patients of the CIMP-L subgroup had better OS (*P*=0.003, Figure 9(a)). Univariate and multivariate Cox analyses also suggested that the CIMP status was significantly related with OS and was also an independent prognostic factor for OS of PC patients after adjusting for gender, age, T stage, N stage, M stage, and TNM stage (*P*=0.012, Table 5).

The association of CIMP status with gene mutations was further analyzed. In line with the results in TCGA, all the 35 PC patients of the CIMP-H subgroup (100%) had gene mutation and 168 out of 171 PC patients of the CIMP-M subgroup (98%) had gene mutation, while only 35 out of 58 PC patients of the CIMP-L subgroup (58.66%) had gene mutation. Obviously, higher somatic mutation burdens in KRAS, TP53, CDKN2A, and TTN were found in patients of the CIMP-H group compared to patients of the CIMP-L group (Figure 9(b)). Besides, higher TMB was also found in patients of the CIMP-H subgroup, while lower TMB was observed in patients with CIMP-L status (Figure 9(b)).

The landscape of TME among PC patients with distinct CIMP status was also explored. As expected, significantly lower stromal score, immune score, and estimate score were found in patients of the CIMP-L subgroup. Meanwhile, significantly lower total T cells, total B cells, CD8 T cells, and memory CD4 T cells were found in patients of the CIMP-H subgroup, but significantly higher follicular helper T cells were found in patients of the CIMP-H subgroup (Figure 9(c)).

Finally, the association of CIMP status with immune checkpoint genes was also analyzed. Only expression of 4 immune checkpoint genes, including PD-1, PD-L1, CD86, and CD276, were available. Similarly, lower expression of PD-1, PD-L1, and CD86 were found in patients of the CIMP-H subgroup, but the difference was not statistically significant (Figure 9(d)).

## 4. Discussion

Identification of clinically relevant cancer subtypes based on the DNA methylation pattern is an important computational problem in medicine, which may help to provide specific and effective treatment options for patients with different subtypes. Previously, DNA methylation pattern analysis of PC patients has been performed but was limited to low sample size and a small number of CpG sites [5, 11, 14]. Sato et al. analyzed the genome-scale DNA methylation patterns in PC patients, but only 8 genes of methylation-specific sites were used [14]. Thompson et al. explored the association of DNA methylation patterns with the survival of PC patients, but the number of samples used for analysis was small, in which only 11 PC patients, 2 normal controls, and 3 chronic pancreatitis patients were included [2]. Recently, Nitish et al. identified three CIMP subtypes of PC patients with distinct clinical characteristics and gene mutation landscapes by clustering of differentially methylated sites using the genome-scale methylome data of PC patients from TCGA; however, they did not further explore the relationship among CIMP status, prognosis of PC patients, and TME. They also did not validate the three CIMP subtypes in independent PC cohorts [11]. In the present study, we identified and validated three distinct CIMP subgroups (termed CIMP-L, CIMP-M, and CIMP-H subgroup) in 448 PC patients with the data downloaded from TCGA and the ICGC. In agreement with previous studies, we observed that CIMP status was significantly associated with clinical characteristics, OS, and RFS in PC patients. There were more patients with advanced T stage and TNM stage in the CIMP-M subgroup and the CIMP-H subgroup. Patients of the CIMP-H subgroup had the worst OS and RFS. Moreover, CIMP status was also an independent prognostic factor for OS and DFS in PC patients.

Accumulation of somatic mutations in oncogenes and TSGs is common in the development and progression of cancer [27]. Based on the mutation analysis, significantly higher somatic mutation burdens in KRAS, TP53, SMAD4, and CDKN2A were observed in patients of the CIMP-H subgroup, which have been shown to be major driver genes in PC [23]. Encoding a small GTPase involved in cellular proliferation, motility, and cytoskeletal remodeling, KRAS was the most frequently mutated oncogene in PC. More than 90% of PC patients showed somatic mutations in KRAS [28]. CDKN2A encoded an essential cell-cycle regulator and was reported to be the most frequently mutated TSG in PC. Similarly, more than 90% of PC patients exhibited function defects of CDKN2A because of gene mutation [28]. Besides, studies have shown that somatic KRAS and CDKN2A mutations were early events of PC development as they were the earlier alteration genes in most low-grade pancreatic intraepithelial neoplasia [29]. TP53 played a vital role in the cellular stress response. Somatic mutations in TP53 were also observed frequently in a wide range of tumor types, including PC. SMAD4 was found to mainly mediate signaling downstream of the TGF*β* receptor and was inactivated in about 50% of PC patients. Alterations in TP53 and SMAD4 were late events in PC, as they often occurred in pancreatic patients with histologic grade 3 and high invasiveness [29]. Moreover, CIMP may also act as a tumor promoter in PC carcinogenesis by influencing mutation of major driver genes, and PC patients of different CIMP subtypes originated from precursor cells might have a distinct epigenetic background of the cell of origin.

KEGG pathways, such as the “p53 signaling pathway” and “base excision repair,” were all found to be significantly enriched in patients of the CIMP-H subgroup and CIMP-M subgroup. As a well-known tumor suppressor, p53 is also one of the most common mutant genes in PC. On one hand, it could block the cell cycle and maintain the genomic stability; increasing number of evidence have proved that mutation in p53 leads to the loss of tumor inhibition function of p53 and, thus, helps the tumor cells of PC to acquire the carcinogenic activity and promote the growth of tumors [30]. On the other hand, p53 could also regulate the immune microenvironment of PC. Textor et al. have also found that p53 could promote the antitumor activity of natural killer cells by transcriptional regulation of the expression of ULl6-binding protein 1 and ULl6-binding protein 2 [31]; Gasparini et al. found that p53 could increase the number of T cells by enhancing the ability of dendritic cells [32]. p53 could enhance the innate immune response by promoting the expression of toll-like receptors on the surface in tumor-associated macrophages and neutrophils [33]. Besides, Hayashi et al. have also proved that p53 could regulate TME by facilitating the secretion of vascular endothelial growth factors and activating fibroblasts to promote angiogenesis [34]. Thus, mutation of p53 no doubt plays an important role in the development and metastasis of PC. The base excision repair pathway has been recognized as a prognostic factor, therapeutic target, and therapeutic response predictor in a variety of cancers, including PC [35]. For example, Jiang et al. have observed that silence of the base excision repair pathway protein APE1 inhibits the proliferation/colony-forming ability and increases apoptosis of PC cells (Panc1 and MiaPaCa2) by increasing DNA damage [36]. Fishel et al. have also showed that inhibition of APE1 could reduce tumor growth in PC xenograft mouse models by reducing proliferation and migration of PC cells and cancer-associated endothelial cells and decreasing the transcription factor activity of NF*κ*B, AP-1, and HIF-1*α* [37]. Moreover, Cardoso et al. have showed that the DNA binding and transcriptional activity of STAT3 is regulated by APE1 and dual targeting of APE1 and STAT3 could synergistically inhibit the survival and migration of PC cells, which suggests that the synergistic effects may have been due to enhancement of STAT3 inhibition by inhibition of APE1 [38]. Taken together, these results may show that the CIMP status may influence the prognosis of PC patients by regulation of the “p53 signaling pathway” and “base excision repair.”

Consisting of cancer cells, stromal cells (mainly composed of fibroblasts and immune cells), and extracellular components, the TME has been found to promote tumor progression, metastasis niche formation, and therapeutic resistance in PC [24]. In the present study, we also found that CIMP status was significantly associated with TME. On one hand, significantly higher tumor purity and cancer stemness indices were found in patients of the CIMP-H subgroup. Research has proven that loss of differentiated phenotype and acquisition of progenitor and stem cell-like features are the main hallmarks of cancer progression. It was speculated that cancer cells might arise from a cell population with self-renewal ability, which was thought to be CSCs. Pancreatic cancer stem cells (PCSCs) have been first identified in 2007 and were reported to take part in the resistance to standard chemotherapy and radiation treatment as they could express multidrug resistant membrane transporters, aberrantly activate proliferation signaling pathways, and increase the capability of repairing DNA [39]. On the other hand, lower stromal score and immune score were found in PC patients of the CIMP-H subgroup. Consistently, lower proportions of total T cells, total B cells, naive B cells, CD8 T cells, CD4 T cells, memory CD4 T cells, and activated memory CD4 T cells were also found in patients of the CIMP-H subgroup, which indicated that patients of the CIMP-H subgroup had a distinct immune phenotype, characterized by less immune cell infiltration, lower cytotoxic potential, and immune activation. Moreover, many kinds of chemokines, such as CCL2, XCL2, CCR2, CCL5, and CCR5, were also found to be decreased in patients of the CIMP-H subgroup, which may in part account for the lower infiltration of immune cells as many different kinds of immune cell were mainly recruited into the TME via interactions between chemokines and their chemokine receptors [25]. Taken together, the higher tumor purity and cancer stemness indices and lower infiltration of immune cells in patients of the CIMP-H subgroup may contribute to their worse OS and DFS.

The advent of immunotherapy, especially checkpoint inhibitor-based immunotherapy, has revolutionized cancer treatments, especially for advanced cancer patients [26]. Currently, monoclonal antibodies targeted against PD-1 and its ligands have been successfully applied in clinical practice and have been approved for several cancers (such as melanoma, non-small-cell lung carcinoma, renal cancer, and bladder cancer) [40]. Besides, research has showed that a combination of anti-PD-1/PD-L1 antibody and CTLA-4 inhibitor can improve treatment effects of patients with advanced melanoma, which was approved by the FDA in treating BRAF V600 E wild-type patients with unresectable or metastatic melanoma [41, 42]. For example, a recent phase-II clinical trial proved that the combination of ipilimumab (a CTLA-4 inhibitor) and nivolumab (a PD-1 inhibitor) significantly improved treatment efficacy in advanced melanoma patients compared with monotherapy with ipilimumab [43]. In our study, a significant association of CIMP status with the expression of immune checkpoint genes was found. Lower expression of PD-1, CTLA4, CD86, VTCN1, and LAG-3 was found in PC patients of the CIMP-H subgroup. Considering the expression of immune checkpoint genes was necessary for checkpoint inhibitor immunotherapy. Effective immunotherapy of immune checkpoint inhibitors depends on the generation of neoantigen-specific T cells and their infiltration into the TME. Immunotherapies were less likely to be efficacious in patients with the CIMP-H phenotype as lower expression of immune checkpoint genes and T cell infiltration was found.

As a changeable and possibly heritable genetic alteration, epigenetic regulation may prove to be a promising clue for the treatment of various kinds of diseases, including cancers [44]. In vitro studies have shown that 5-Aza-CdR, a DNA methyltransferase 1 (DNMT1) inhibitor, could induce cell death and apoptosis of pancreatic cancer cells by reactivation of RASSF1A and upregulation of Bax genes [45]. Han et al. also observed the synergistic effects of the combination of 5-Aza-CdR and suberoylanilide hydroxamic acid on the anticancer property of PC [46]. More encouragingly, phase I/II clinical trials of DNMT1 inhibitors (azacitidine, decitabine, and guadecitabine) in PC patients are currently underway, the inhibitors which exhibited potential treatment outcomes [47]. There is a promising future in drug design for epigenetic targets, while CIMP may help to make an assertion to provide specific and efficient treatment options for patients with different CIMP statuses.

However, there were some limitations to be addressed in our study. First, our analysis was performed on the basis of single-omics (DNA methylation). Patients with the same CIMP status might have heterogeneity due to the different characteristics in terms of other omics data. Second, our analysis was performed on the basis of a retrospective cohort. Prospective studies with larger sample sizes should be performed to validate our findings. Finally, the biological functions and molecular mechanisms of CIMP status that influence the prognosis of PC patients should be further validated in in vitro experiments.

In conclusion, we identified and validated three distinct CIMP subgroups in PC patients. The CIMP may modulate the immune response of the tumor microenvironment and influence the prognosis of pancreatic cancer patients, which may help to make an assertion to provide specific and efficient treatment options for patients of different subtypes.

## Figures and Tables

**Table 1:** Demographic and clinical characteristics of PAAD patients with different CIMP statuses from TCGA.

Variables	CIMP-L	CIMP-M	CIMP-H	*P* value
Number of patients	46	82	56	
Gender (male/female)	21/25	48/34	32/24	0.343
Age (years, ≤65/>65)	27/19	43/39	26/29	0.466
T stage (T1/T2/T3/T4/NA)	5/10/30/0/1	0/8/70/3/1	2/5/48/1/0	0.008^*∗*^
N stage (N0/N1/NX + NA)	14/29/3	17/64/1	19/36/1	0.13
M stage (M0/M1/MX + NA)	22/1/23	34/2/46	28/2/26	0.829
TNM stage (I/II/III/IV/NA)	12/32/0/1/1	4/71/4/2/1	5/48/1/2/0	0.014^*∗*^

**Table 2:** Univariate and multivariate analyses of overall survival and progression-free survival in PAAD patients of the TCGA cohort.

Variables	OS						PFS					
	Univariate analysis			Multivariate analysis			Univariate analysis			Multivariate analysis		
	Hazard ratio	95% CI	*P* value	Hazard ratio	95% CI	*P* value	Hazard ratio	95% CI	*P* value	Hazard ratio	95% CI	*P* value
Gender												
(Male vs. female)	0.84	0.565–1.25	0.39				0.929	0.637–1.354	0.701			
Age												
(>65 vs. ≤ 65)	1.302	0.873–1.943	0.195				1.237	0.843–1.815	0.277			
T stage												
(T3 + T4 vs. T1 + T2)	2.28	1.179–4.407	0.014^*∗*^	1.584	0.796–3.15	0.19	2.651	1.406–4.999	0.003^*∗*^	2.037	1.053–3.942	0.035^*∗*^
N stage												
(N1 + Nx vs. N0)	2.117	1.278–3.508	0.004^*∗*^	1.828	1.096–3.049	0.021^*∗*^	1.603	1.035–2.482	0.034^*∗*^	1.48	0.946–2.316	0.086
M stage												
(M1 + Mx vs. M0)	0.844	0.567–1.257	0.404				0.58	0.396–0.851	0.005^*∗*^	0.725	0.491–1.0	0.105
TNM stage												
(stage III + IV vs. stage I + II)	1.0	0.31–3.225	1.0				0.994	0.461–2.154	0.994			
CIMP status												
CIMP-M vs. CIMP-L	2.039	1.142–3.642	0.016^*∗*^	1.72	0.948–3.122	0.074	1.585	0.934–2.688	0.088			
CIMP-H vs. CIMP-L	2.289	1.245–4.206	0.008^*∗*^	2.043	1.096–3.806	0.024^*∗*^	2.393	1.405–4.075	0.001^*∗*^	1.956	1.129–3.388	0.017^*∗*^

**Table 3:** Expression of 59 kinds of chemokine among PC patients with different CIMP statuses.

Gene	CIMP-L (*N* = 46)		CIMP-M (*N* = 76)		CIMP-H (*N* = 56)		*P* value
	Mean	Sd	Mean	Sd	Mean	Sd	
CCL1	0.019	0.034	0.021	0.037	0.020	0.045	0.941
CXCR6	2.238	1.739	1.344	1.406	0.669	0.483	<0.001^*∗∗∗*^
CCL2	35.294	33.754	19.973	17.815	11.123	9.482	<0.001^*∗∗∗*^
CCL19	89.272	118.604	52.134	78.701	16.316	27.712	<0.001^*∗∗∗*^
XCL2	1.092	0.904	0.696	0.675	0.446	0.439	<0.001^*∗∗∗*^
CX3CL1	11.107	6.235	14.391	10.037	15.302	17.829	0.207
CCR10	0.616	1.214	0.360	0.212	0.298	0.183	0.033^*∗*^
CCL25	0.224	0.518	2.914	14.716	0.225	1.103	0.188
CXCR4	86.490	125.355	57.819	71.923	31.478	18.115	0.003^*∗∗*^
CCL28	7.608	8.420	7.050	6.102	6.124	4.299	0.483
CXCL2	13.775	24.638	9.186	7.417	7.984	24.495	0.284
CCR2	2.095	1.697	1.318	1.272	0.603	0.563	<0.001^*∗∗∗*^
CCL5	32.342	26.573	23.419	21.406	13.148	10.918	<0.001^*∗∗∗*^
CCR9	0.185	0.186	0.248	0.333	0.248	0.225	0.402
XCR1	0.636	0.602	0.341	0.432	0.148	0.224	<0.001^*∗∗∗*^
CCL8	1.405	1.377	1.051	1.784	0.751	1.491	0.123
CXCL11	2.560	3.744	3.279	6.261	2.675	4.345	0.697
CXCL1	18.895	26.105	16.609	12.493	15.503	28.623	0.742
CXCL17	26.545	34.664	62.098	85.526	64.257	86.579	0.022^*∗*^
CXCR5	0.229	0.490	0.120	0.292	0.028	0.051	0.007^*∗∗*^
CCL16	0.141	0.109	0.084	0.065	0.038	0.041	<0.001^*∗∗∗*^
CCR5	3.709	2.898	2.485	2.357	1.251	0.993	<0.001^*∗∗∗*^
CXCL8	18.044	18.167	25.857	27.721	16.058	16.663	0.031^*∗*^
CXCL14	62.254	72.601	97.927	80.887	132.875	168.641	0.009^*∗∗*^
CCR8	0.536	0.583	0.446	0.435	0.307	0.366	0.040^*∗*^
CCL15	1.298	1.478	1.786	2.572	1.556	2.122	0.491
CCL21	108.128	193.077	72.864	195.196	24.227	43.603	0.034^*∗*^
CCL23	1.964	1.921	1.821	3.553	0.828	1.388	0.049^*∗*^
CCR7	8.640	15.561	4.779	12.140	1.401	1.492	0.006^*∗∗*^
CCL3	3.676	3.275	2.509	1.904	1.737	1.501	<0.001^*∗∗∗*^
CCL7	0.290	0.598	0.386	0.491	0.529	0.928	0.203
CXCL10	10.127	15.566	11.441	24.734	9.581	13.448	0.854
CCL24	3.600	3.986	23.088	85.332	20.610	94.433	0.372
CX3CR1	1.521	1.261	1.015	0.941	0.576	0.733	<0.001^*∗∗∗*^
CCL4	4.747	3.162	2.978	2.468	1.757	1.277	<0.001^*∗∗∗*^
CCL22	4.591	5.368	3.777	3.472	2.011	1.733	0.002^*∗∗*^
CXCL13	21.519	45.303	14.589	37.830	4.694	11.043	0.046^*∗*^
CXCR2	0.684	0.870	0.648	0.801	0.217	0.310	0.002^*∗∗*^
CXCL6	11.406	16.524	10.446	13.190	5.032	4.956	0.015^*∗*^
CCL14	1.066	0.998	0.577	0.508	0.162	0.145	<0.001^*∗∗∗*^
CCL26	3.888	9.430	3.928	16.757	1.956	3.101	0.606
CCR4	2.337	2.796	1.304	1.546	0.520	0.547	<0.001^*∗∗∗*^
CCL17	10.386	19.405	8.800	11.544	3.906	5.125	0.025^*∗*^
CXCR1	0.662	0.839	0.646	0.819	0.288	0.357	0.008^*∗∗*^
CCR1	5.030	3.568	3.859	3.112	2.588	1.995	<0.001^*∗∗∗*^
CXCL5	38.527	64.259	81.274	124.396	36.474	49.043	0.008^*∗∗*^
CXCL9	18.918	42.376	14.706	50.569	9.541	19.449	0.511
XCL1	0.452	0.345	0.409	0.472	0.377	0.674	0.766
CXCR3	2.298	2.170	2.379	2.067	1.941	1.557	0.424
CCL18	21.990	42.000	29.714	29.962	15.548	23.234	0.041^*∗*^
CCL13	4.006	5.276	4.984	4.601	4.791	7.459	0.655
CCR6	0.141	0.194	0.098	0.117	0.049	0.057	0.002^*∗∗*^
CCR3	0.086	0.101	0.110	0.159	0.147	0.449	0.522
CXCL16	38.590	19.162	48.610	16.384	49.334	14.780	0.002^*∗∗*^
CCL20	8.951	12.180	25.344	41.318	19.521	29.584	0.027^*∗*^
CCL11	3.908	3.675	3.800	3.876	1.647	1.317	<0.001^*∗∗∗*^
CCL27	0.000	0.003	0.000	0.000	0.001	0.005	0.300
CXCL3	4.555	9.367	5.720	6.384	5.472	13.359	0.811
CXCL12	25.525	16.445	12.292	10.336	4.826	3.670	<0.001^*∗∗∗*^

**Table 4:** Demographic and clinical characteristics of PAAD patients with different CIMP statuses from the ICGC.

Variables	CIMP-L	CIMP-M	CIMP-H	*P* value
Number of patients	58	171	35	
Gender (male/female/NA)	35/23/0	95/75/1	20/15	0.904
Age (years, ≤65/>65)	28/30/0	77/93/1	11/20/4	0.009^*∗∗*^
T stage (T1/T2/T3/T4/NA)	5/3/46/02/2	1/817/145/2/6	0/3/20/1/11	<0.001^*∗∗∗*^
N stage (N0/N1/NX + NA)	12/44/2	46/119/6	7/16/12	<0.001^*∗∗∗∗*^
M stage (M0/M1/MX + NA)	14/2/42	24/6/141	1/4/30	0.016^*∗*^
TNM stage (I/II/III/IV/NA)	6/48/1/2/1	8/150/2/6/5	1/20/0/4/10	<0.001^*∗∗∗*^

**Table 5:** Univariate and multivariate analyses of overall survival in PAAD patients of the ICGC cohort.

Variables	Univariate analysis			Multivariate analysis		
	Hazard ratio	95% CI	*P* value	Hazard ratio	95% CI	*P* value
Gender (male vs. female)	1.268	0.944–1.702	0.114			
Age (>65 vs. ≤65)	0.562	0.39–0.81	0.002^*∗*^	0.295	0.102–0.857	0.025^*∗*^
T stage (T3 + T4 vs. T1 + T2)	0.779	0.545–1.112	0.169			
N stage (N1 + Nx vs. N0)	0.796	0.61–1.038	0.092			
M stage (M1 + Mx vs. M0)	0.985	0.744–1.305	0.981			
TNM stage (stage III + IV vs. stage I + II)	0.917	0.642–1.310	0.633			
CIMP status						
CIMP-M vs. CIMP-L	1.016	0.717–1.439	0.929			
CIMP-H vs. CIMP-L	2.01	1.274–3.193	0.003^*∗*^	1.849	1.146–2.984	0.012^*∗*^

## Data Availability

The data of the study are available from the corresponding web page link, including the GDC Data Portal (https://cancergenome.nih.gov/) and ICGC Portal (https://dcc.icgc.org/).

## Abbreviations

PC: Pancreatic cancer
TSGs: Tumor suppressor genes
CIMP: CpG island methylator phenotype
TME: Tumor microenvironment
TCGA: The Cancer Genome Atlas
ICGC: International Cancer Genome Consortium
TMB: Tumor mutational burden
OS: Overall survival
PFS: Progression-free status
PCA: Principal component analysis
ESTIMATE: Estimation of STromal and Immune cells in MAlignant Tumor tissues using Expression data
ssGSEA: Single sample Gene Set Enrichment Analysis
OCLR: One-class logistic regression machine learning algorithm
GSEA: Gene set enrichment analysis
CSCs: Cancer stem cells
PCSCs: Pancreatic cancer stem cells
DNMT1: DNA methyltransferase 1.

## Supplementary Materials

Supplementary Materials Supplementary Figure 1: the most significant 25 hypermethylated CpGs and the most 25 hypomethylated CpGs between 184 PC patients and 10 normal controls by the Wilcoxon test. Supplementary Figure 2: the most significant 25 worse overall survival-related CpGs and the most 25 better overall survival-related CpGs in PC patients by univariate Cox analysis. The hazard ratio (HR) and the 95% confidence interval of the HR were log10-transformed. Supplementary Material 1: it summarizes the differential CpGs between 184 PC patients and 10 normal controls by the Wilcoxon test. In total, 22,450 differential CpGs were identified between 184 PC patients and 10 normal controls (*P* < 0.05). Among these CpGs, 12,937 were hypermethylated CpGs (log2FC > 0), while 9,513 were hypomethylated CpGs (log2FC < 0). Supplementary Material 2: it summarizes the overall survival- (OS-) related CpGs in PC patients by univariate Cox analysis. In total, 3102 CpGs were found to be related with OS in PC patients (*P* < 0.05). Among these CpGs, 2858 CpGs were found to be associated with worse OS of PC patients (HR > 1), while 244 were found to be associated with better OS of PC patients (HR < 1). Supplementary Material 3: it summarizes the most OS-related CpGs in PC patients by multivariate Cox analysis. In order to identify the most OS-related CpGs, only 1073 out of 3102 OS-related CpGs with *P* < 0.01 were used for multivariate Cox analysis, and 72 CpGs were found to be the most OS-related CpGs and they were finally selected for unsupervised consensus clustering analysis.
