# Supplementary material for: CpG Island Methylator Phenotype Modulates the Immune Response of the Tumor Microenvironment and Influences the Prognosis of Pancreatic Cancer Patients
Source: J Oncol. 2021 Nov 28;2021:2715694. doi: 10.1155/2021/2715694 (PMC8645373; doi:10.1155/2021/2715694)
Supplement: Supplementary Materials — Supplementary Figure 1: the most significant 25 hypermethylated CpGs and the most 25 hypomethylated CpGs between 184 PC patients and 10 normal controls by the Wilcoxon test. Supplementary Figure 2: the most significant 25 worse overall survival-related CpGs and the most 25 better overall survival-related CpGs in PC patients by univariate Cox analysis. The hazard ratio (HR) and the 95% confidence interval of the HR were log10-transformed. Supplementary Material 1: it summarizes the differential CpGs between 184 PC patients and 10 normal controls by the Wilcoxon test. In total, 22,450 differential CpGs were identified between 184 PC patients and 10 normal controls (P < 0.05). Among these CpGs, 12,937 were hypermethylated CpGs (log2FC > 0), while 9,513 were hypomethylated CpGs (log2FC < 0). Supplementary Material 2: it summarizes the overall survival- (OS-) related CpGs in PC patients by univariate Cox analysis. In total, 3102 CpGs were found to be related with OS in PC patients (P < 0.05). Among these CpGs, 2858 CpGs were found to be associated with worse OS of PC patients (HR > 1), while 244 were found to be associated with better OS of PC patients (HR < 1). Supplementary Material 3: it summarizes the most OS-related CpGs in PC patients by multivariate Cox analysis. In order to identify the most OS-related CpGs, only 1073 out of 3102 OS-related CpGs with P < 0.01 were used for multivariate Cox analysis, and 72 CpGs were found to be the most OS-related CpGs and they were finally selected for unsupervised consensus clustering analysis. [file 2715694.f1.zip › 2715694.f1/Supplementary material 1 (1).pdf]

Differential CpGs between 184 pancreatic cancer (PC) patients and 10 normal controls(NC)

CpG site	Mean expression of NC	Mean expression of PC	log2FC	pValue	Satus
cg01423964	0.01267	0.346253514	4.77234	8.72E-07	hypermethylated
cg01638592	0.01371	0.313350811	4.514478	2.56E-06	hypermethylated
cg26272220	0.01287	0.268971351	4.385369	3.95E-06	hypermethylated
cg13062406	0.01375	0.284086486	4.368827	1.56E-06	hypermethylated
cg23080354	0.01359	0.261954054	4.268696	4.12E-06	hypermethylated
cg07882671	0.01988	0.375895676	4.240943	2.10E-06	hypermethylated
cg02511231	0.01926	0.36332	4.237561	4.27E-07	hypermethylated
cg07009376	0.0147	0.27380973	4.219286	9.94E-07	hypermethylated
cg15202738	0.01421	0.263260541	4.211513	2.49E-06	hypermethylated
cg07252731	0.01565	0.285990811	4.191734	9.11E-07	hypermethylated
cg09568464	0.0155	0.280127568	4.175744	9.83E-05	hypermethylated
cg03315869	0.01464	0.261996757	4.161561	3.04E-06	hypermethylated
cg04638468	0.01573	0.278552973	4.146361	6.58E-07	hypermethylated
cg15760257	0.01694	0.297628108	4.135005	7.93E-06	hypermethylated
cg19001226	0.01604	0.281663784	4.134228	5.97E-06	hypermethylated
cg02279670	0.01537	0.269217838	4.130585	1.26E-06	hypermethylated
cg10863741	0.01407	0.245632432	4.125807	5.51E-07	hypermethylated
cg15565065	0.01728	0.296491351	4.100815	3.44E-06	hypermethylated
cg25875213	0.01741	0.297108649	4.093002	5.59E-07	hypermethylated
cg01307939	0.01275	0.217141622	4.090067	6.13E-06	hypermethylated
cg25884711	0.01751	0.29348	4.067011	1.47E-06	hypermethylated
cg14458834	0.01991	0.326183784	4.03412	4.86E-06	hypermethylated
cg10362542	0.01711	0.27732	4.01864	1.24E-06	hypermethylated
cg08384637	0.01718	0.278167027	4.017149	1.06E-05	hypermethylated
cg24403845	0.02159	0.347975135	4.010549	1.37E-06	hypermethylated
cg10096177	0.01667	0.267984324	4.006823	1.35E-06	hypermethylated
cg12477716	0.02377	0.375828649	3.982861	1.15E-06	hypermethylated
cg06947913	0.02316	0.363957838	3.974064	1.08E-05	hypermethylated
cg12799689	0.0177	0.277155676	3.968875	2.56E-06	hypermethylated
cg18607529	0.01435	0.22431027	3.966373	2.71E-05	hypermethylated
cg18579862	0.02283	0.354849189	3.958203	4.24E-06	hypermethylated
cg03394150	0.0188	0.291815676	3.956253	1.80E-05	hypermethylated
cg08452658	0.01794	0.276514054	3.946101	4.42E-06	hypermethylated
cg15072319	0.01981	0.305056216	3.944774	2.83E-06	hypermethylated
cg16443866	0.01772	0.271557297	3.937806	9.69E-06	hypermethylated
cg21546671	0.01931	0.291012432	3.913661	3.21E-06	hypermethylated
cg08089301	0.02028	0.302202162	3.897384	9.57E-06	hypermethylated
cg18328894	0.01579	0.234931351	3.895156	3.12E-06	hypermethylated
cg14496450	0.01648	0.243832432	3.887102	2.07E-06	hypermethylated
cg16964348	0.02227	0.329095676	3.885334	7.52E-07	hypermethylated
cg16732616	0.02892	0.426784324	3.883368	1.37E-06	hypermethylated
cg08985029	0.01832	0.268096216	3.871259	2.62E-07	hypermethylated
cg04353095	0.01803	0.262303243	3.862764	5.07E-06	hypermethylated
cg06952671	0.02005	0.28817027	3.845247	2.32E-06	hypermethylated
cg01088410	0.01944	0.278001081	3.83799	1.12E-06	hypermethylated
cg27633530	0.01818	0.257548108	3.824418	5.12E-05	hypermethylated
cg13135595	0.01549	0.219320541	3.823632	2.20E-06	hypermethylated
cg02012576	0.01524	0.214828649	3.817252	7.20E-07	hypermethylated
cg04557544	0.02765	0.389655676	3.816848	1.20E-06	hypermethylated
cg12074025	0.01951	0.274367568	3.813824	1.71E-05	hypermethylated
cg04342092	0.0144	0.200571892	3.799979	7.30E-07	hypermethylated
cg04904331	0.02771	0.384717838	3.795322	1.60E-06	hypermethylated
cg01614020	0.02328	0.322650811	3.792811	2.68E-06	hypermethylated
cg13457172	0.01933	0.263666486	3.769801	6.58E-07	hypermethylated
cg12824796	0.02541	0.345737297	3.766208	8.10E-07	hypermethylated
cg18680788	0.02087	0.283420541	3.763442	1.88E-06	hypermethylated

cg06392169	0.02776	0.376381622	3.761117	3.12E-06	hypermethylated
cg25999442	0.02909	0.394372432	3.760964	5.35E-06	hypermethylated
cg25079102	0.01594	0.215503243	3.756986	6.11E-07	hypermethylated
cg17277892	0.0199	0.268826486	3.755835	3.12E-06	hypermethylated
cg01642521	0.02687	0.362039459	3.752079	1.26E-06	hypermethylated
cg07900968	0.01785	0.240472432	3.751876	2.49E-06	hypermethylated
cg17228900	0.01818	0.242098378	3.735169	6.58E-07	hypermethylated
cg10332700	0.01663	0.220637838	3.72982	3.23E-05	hypermethylated
cg18485844	0.02624	0.347382162	3.726684	6.05E-06	hypermethylated
cg12417685	0.01861	0.245557297	3.72191	3.64E-06	hypermethylated
cg01410878	0.02003	0.263620541	3.718228	1.88E-06	hypermethylated
cg25662463	0.01675	0.22019027	3.716518	4.33E-07	hypermethylated
cg04106647	0.0168	0.219532973	3.707905	2.99E-06	hypermethylated
cg25778535	0.02182	0.284364324	3.704017	3.35E-06	hypermethylated
cg05336698	0.01666	0.217089189	3.703828	2.46E-06	hypermethylated
cg14409941	0.01822	0.236283784	3.696926	5.14E-06	hypermethylated
cg21384402	0.02115	0.273909189	3.694968	6.02E-07	hypermethylated
cg20463526	0.02199	0.284224865	3.692113	3.69E-06	hypermethylated
cg25958283	0.02741	0.352268108	3.6839	3.44E-06	hypermethylated
cg15927720	0.02237	0.285900541	3.675876	2.13E-05	hypermethylated
cg27331241	0.01824	0.232223243	3.670335	6.13E-06	hypermethylated
cg18009496	0.02425	0.307233514	3.663279	1.88E-06	hypermethylated
cg05720454	0.01596	0.202058378	3.66224	3.17E-06	hypermethylated
cg21790626	0.02503	0.315667027	3.656673	1.39E-06	hypermethylated
cg05099508	0.02286	0.285460541	3.642394	1.54E-06	hypermethylated
cg19288904	0.02598	0.321944324	3.631338	1.22E-06	hypermethylated
cg17331296	0.02407	0.297595135	3.628043	3.59E-06	hypermethylated
cg20870512	0.02312	0.285445405	3.626002	1.58E-05	hypermethylated
cg04543008	0.02814	0.34734	3.625654	1.58E-06	hypermethylated
cg20525183	0.02316	0.285569189	3.624133	1.60E-05	hypermethylated
cg22014661	0.019	0.233630811	3.620159	5.67E-07	hypermethylated
cg24773418	0.02129	0.261227568	3.617059	3.39E-06	hypermethylated
cg03703637	0.02641	0.323068649	3.612685	6.84E-06	hypermethylated
cg00505277	0.02335	0.285592973	3.612466	5.50E-06	hypermethylated
cg18115215	0.02008	0.245136757	3.609756	1.44E-05	hypermethylated
cg20978694	0.02411	0.293779459	3.60703	6.48E-06	hypermethylated
cg16918989	0.02011	0.244097838	3.601475	2.87E-06	hypermethylated
cg05157140	0.0256	0.308919459	3.593015	7.32E-06	hypermethylated
cg08586541	0.0227	0.273702162	3.591843	1.75E-06	hypermethylated
cg25208017	0.03131	0.375732973	3.585012	7.98E-07	hypermethylated
cg22898797	0.02476	0.297045946	3.584603	3.30E-06	hypermethylated
cg17208528	0.01907	0.228266486	3.581342	8.48E-06	hypermethylated
cg10461004	0.01942	0.231332973	3.574356	0.002699	hypermethylated
cg13445199	0.02327	0.276607568	3.571298	2.91E-06	hypermethylated
cg26802289	0.02186	0.259107568	3.567186	2.15E-05	hypermethylated
cg21097881	0.03132	0.368036757	3.554694	3.39E-06	hypermethylated
cg17448335	0.02198	0.256393514	3.544096	1.12E-05	hypermethylated
cg07017374	0.02652	0.308207568	3.53875	3.90E-06	hypermethylated
cg20817902	0.01995	0.230566486	3.530722	2.10E-05	hypermethylated
cg07438617	0.02174	0.25088973	3.52863	8.59E-06	hypermethylated
cg08862890	0.03001	0.344541081	3.521161	2.57E-05	hypermethylated
cg24761507	0.02522	0.288508649	3.515974	3.21E-06	hypermethylated
cg15967847	0.01753	0.199569189	3.508991	1.47E-06	hypermethylated
cg21277995	0.031	0.352556757	3.507515	9.52E-07	hypermethylated
cg10249375	0.0266	0.301764865	3.503927	1.31E-06	hypermethylated
cg01392544	0.02934	0.33058	3.494059	2.95E-06	hypermethylated
cg14564616	0.02359	0.264999459	3.489742	9.24E-07	hypermethylated
cg25963041	0.02538	0.284923243	3.488809	4.26E-05	hypermethylated

cg04741853	0.02353	0.26415027	3.488786	1.56E-05	hypermethylated
cg25324105	0.0342	0.383003784	3.48529	8.84E-07	hypermethylated
cg06241792	0.02655	0.295504324	3.476395	5.73E-06	hypermethylated
cg22346124	0.02831	0.314859459	3.475324	5.97E-06	hypermethylated
cg08217024	0.02871	0.319290811	3.475246	1.77E-06	hypermethylated
cg05347845	0.03109	0.345166486	3.47277	5.43E-06	hypermethylated
cg05661282	0.03545	0.393479459	3.472431	2.46E-06	hypermethylated
cg20334627	0.02451	0.271820541	3.471212	5.07E-06	hypermethylated
cg24876960	0.02449	0.271442162	3.47038	3.69E-06	hypermethylated
cg05028087	0.02387	0.26434	3.469124	1.12E-06	hypermethylated
cg17504999	0.0233	0.256626486	3.461268	1.10E-06	hypermethylated
cg05250768	0.02425	0.265523243	3.452782	1.02E-05	hypermethylated
cg17498296	0.02802	0.305802703	3.448072	1.72E-06	hypermethylated
cg13542964	0.0189	0.206240541	3.44787	1.51E-06	hypermethylated
cg01802258	0.0355	0.387256216	3.447397	1.58E-06	hypermethylated
cg26731327	0.02	0.217942162	3.445873	1.41E-06	hypermethylated
cg03304610	0.02565	0.279390811	3.445254	1.41E-06	hypermethylated
cg15278646	0.01752	0.19079027	3.444913	2.95E-06	hypermethylated
cg16457786	0.02324	0.25308	3.444912	2.64E-06	hypermethylated
cg22289581	0.01876	0.203959459	3.442551	8.98E-07	hypermethylated
cg01261798	0.02277	0.247496216	3.4422	6.75E-06	hypermethylated
cg16697214	0.02757	0.299652432	3.442119	8.95E-06	hypermethylated
cg05143887	0.02078	0.224552432	3.433785	4.30E-06	hypermethylated
cg00609966	0.0214	0.231077838	3.432696	1.04E-05	hypermethylated
cg07790615	0.02819	0.304311892	3.432295	6.99E-07	hypermethylated
cg00984146	0.02562	0.276049189	3.429583	2.15E-05	hypermethylated
cg26483578	0.0225	0.24193027	3.426594	7.52E-06	hypermethylated
cg17016394	0.03249	0.349257838	3.426225	3.49E-06	hypermethylated
cg19443257	0.02405	0.258229189	3.424543	6.57E-06	hypermethylated
cg00615473	0.02802	0.300819459	3.424369	0.000245	hypermethylated
cg14999001	0.02726	0.292623243	3.424187	0.000161	hypermethylated
cg09968723	0.02539	0.272037297	3.421472	3.90E-05	hypermethylated
cg24724633	0.02829	0.302824324	3.420117	8.60E-06	hypermethylated
cg18755296	0.02548	0.272731351	3.420043	3.30E-06	hypermethylated
cg17460447	0.02658	0.283644324	3.41567	1.37E-05	hypermethylated
cg12863545	0.02795	0.298158919	3.415161	2.99E-06	hypermethylated
cg11573679	0.02508	0.267084865	3.412689	1.12E-05	hypermethylated
cg18705773	0.03263	0.347087568	3.411029	6.20E-07	hypermethylated
cg01705052	0.02902	0.308453514	3.409934	1.31E-06	hypermethylated
cg27179533	0.02563	0.271440541	3.404731	7.72E-06	hypermethylated
cg08708684	0.03052	0.322623784	3.402026	6.48E-06	hypermethylated
cg14658804	0.03043	0.321236757	3.400071	1.31E-05	hypermethylated
cg01672943	0.02664	0.280907568	3.39843	4.24E-06	hypermethylated
cg09017619	0.02712	0.285867568	3.397918	1.45E-06	hypermethylated
cg00003298	0.02867	0.300827568	3.391323	1.04E-05	hypermethylated
cg16887264	0.02685	0.281262162	3.388921	5.03E-07	hypermethylated
cg14510812	0.02615	0.273750811	3.38798	1.35E-06	hypermethylated
cg27034819	0.01744	0.182458378	3.387095	1.85E-06	hypermethylated
cg26013553	0.03819	0.399498919	3.386925	3.44E-06	hypermethylated
cg03401096	0.02979	0.311243243	3.385142	0.001875	hypermethylated
cg07136998	0.0328	0.342604865	3.384778	1.17E-05	hypermethylated
cg14072515	0.03064	0.319942703	3.384325	3.21E-06	hypermethylated
cg05238769	0.02335	0.243651892	3.383327	1.54E-06	hypermethylated
cg23121993	0.04073	0.423527027	3.37829	1.37E-06	hypermethylated
cg14874750	0.02555	0.265676757	3.378277	8.71E-06	hypermethylated
cg20230721	0.03347	0.347775135	3.377214	1.85E-06	hypermethylated
cg15547669	0.0229	0.236867568	3.370661	2.95E-06	hypermethylated
cg00778995	0.02505	0.25858973	3.367782	3.96E-07	hypermethylated

cg07302069	0.03609	0.371013514	3.361801	7.09E-07	hypermethylated
cg21688264	0.02183	0.224151892	3.360093	3.49E-06	hypermethylated
cg21077559	0.02108	0.21608	3.357619	1.90E-06	hypermethylated
cg15386964	0.02613	0.267704324	3.356861	8.71E-06	hypermethylated
cg01543173	0.02404	0.245541622	3.352459	7.93E-06	hypermethylated
cg07143532	0.02549	0.259421622	3.347295	1.02E-05	hypermethylated
cg06445348	0.03396	0.344970811	3.344566	1.68E-05	hypermethylated
cg17105834	0.03353	0.340020541	3.342098	3.96E-06	hypermethylated
cg09528825	0.02925	0.296448649	3.341274	2.89E-05	hypermethylated
cg09570682	0.03218	0.325701081	3.339312	1.90E-06	hypermethylated
cg14507560	0.03839	0.388421622	3.338821	3.79E-06	hypermethylated
cg07832473	0.02298	0.232473514	3.338616	6.05E-06	hypermethylated
cg02869459	0.02167	0.218417838	3.33332	5.28E-06	hypermethylated
cg20435370	0.01765	0.177811351	3.332607	1.90E-06	hypermethylated
cg12243597	0.02974	0.298829189	3.328845	3.59E-06	hypermethylated
cg02767771	0.03104	0.31189027	3.328838	3.04E-06	hypermethylated
cg23727983	0.03007	0.301760541	3.327007	2.75E-06	hypermethylated
cg08111446	0.02771	0.276640541	3.319534	2.85E-05	hypermethylated
cg22978087	0.03273	0.326655135	3.319083	7.12E-06	hypermethylated
cg08151857	0.03044	0.303780541	3.318989	2.39E-06	hypermethylated
cg07230107	0.01671	0.16675027	3.318905	3.39E-06	hypermethylated
cg16504626	0.03064	0.305687568	3.31857	1.96E-06	hypermethylated
cg06759189	0.0212	0.211445946	3.318153	3.74E-06	hypermethylated
cg18856388	0.03314	0.32962973	3.314201	1.93E-06	hypermethylated
cg03278146	0.02835	0.281578378	3.312116	3.69E-06	hypermethylated
cg17839237	0.03844	0.381246486	3.310044	3.35E-06	hypermethylated
cg14492800	0.02701	0.267098919	3.305809	5.00E-06	hypermethylated
cg07589773	0.03412	0.336848649	3.303411	0.00012	hypermethylated
cg22286978	0.02939	0.289562703	3.300479	6.48E-06	hypermethylated
cg21913319	0.0245	0.241367027	3.300375	1.31E-05	hypermethylated
cg09236176	0.02965	0.291690811	3.298336	1.60E-06	hypermethylated
cg02595832	0.03171	0.31181027	3.297659	2.07E-06	hypermethylated
cg02746725	0.02199	0.21618	3.297314	5.34E-07	hypermethylated
cg08553437	0.02724	0.267428649	3.295355	1.60E-06	hypermethylated
cg23253569	0.02724	0.267285405	3.294582	3.04E-06	hypermethylated
cg00091285	0.0195	0.191087027	3.292684	2.13E-06	hypermethylated
cg13905586	0.02162	0.211535676	3.290463	1.44E-05	hypermethylated
cg10250663	0.03603	0.352174054	3.289018	4.18E-06	hypermethylated
cg04849541	0.03553	0.345750811	3.282623	5.58E-06	hypermethylated
cg01889143	0.03183	0.309351892	3.28079	8.59E-07	hypermethylated
cg10981909	0.02668	0.258844865	3.278257	6.01E-05	hypermethylated
cg26295057	0.03796	0.368180541	3.277861	1.39E-05	hypermethylated
cg23346462	0.02885	0.27937027	3.275535	3.12E-06	hypermethylated
cg01670677	0.02515	0.243328649	3.274276	6.39E-06	hypermethylated
cg21350575	0.03719	0.359465405	3.272866	3.21E-06	hypermethylated
cg09874822	0.03454	0.333435135	3.271066	4.01E-06	hypermethylated
cg11416384	0.03223	0.31088	3.269882	1.48E-05	hypermethylated
cg14214262	0.02086	0.201111351	3.269183	9.66E-07	hypermethylated
cg17847723	0.01965	0.189275135	3.267884	9.24E-07	hypermethylated
cg08675717	0.02702	0.260212973	3.267593	0.000174	hypermethylated
cg27490380	0.01701	0.163782162	3.267323	1.64E-05	hypermethylated
cg24446548	0.0295	0.283406486	3.264086	1.07E-06	hypermethylated
cg10922935	0.02785	0.267522162	3.263909	5.19E-05	hypermethylated
cg01535698	0.03239	0.310269189	3.2599	3.12E-06	hypermethylated
cg11732619	0.02235	0.214081081	3.259811	0.000138	hypermethylated
cg26246807	0.03941	0.376954054	3.257755	3.54E-06	hypermethylated
cg19859290	0.03345	0.319635676	3.25635	1.39E-06	hypermethylated
cg01939477	0.03586	0.342537297	3.255814	1.82E-06	hypermethylated

cg21938148	0.02841	0.270543784	3.251391	6.22E-06	hypermethylated
cg20872937	0.03212	0.305439459	3.249343	5.97E-06	hypermethylated
cg26170604	0.03111	0.295791892	3.249132	3.69E-06	hypermethylated
cg12974388	0.02792	0.26520973	3.247763	4.42E-06	hypermethylated
cg15867939	0.02171	0.205979459	3.246069	1.35E-05	hypermethylated
cg11017065	0.03643	0.344339459	3.240633	1.93E-06	hypermethylated
cg24454144	0.03737	0.352876757	3.239212	1.82E-06	hypermethylated
cg18130044	0.03588	0.337924324	3.235448	7.32E-06	hypermethylated
cg08516516	0.02682	0.25238	3.234216	3.04E-06	hypermethylated
cg08701047	0.02595	0.243943784	3.232742	1.93E-06	hypermethylated
cg02300154	0.03955	0.371658378	3.232228	2.42E-06	hypermethylated
cg17498773	0.02934	0.274924324	3.228094	8.26E-06	hypermethylated
cg00282249	0.03476	0.325707568	3.228077	1.77E-06	hypermethylated
cg27398263	0.03368	0.315384865	3.227149	5.00E-06	hypermethylated
cg05384102	0.02623	0.245605946	3.227056	5.14E-06	hypermethylated
cg17398252	0.027	0.25259027	3.225768	0.00012	hypermethylated
cg24873562	0.0268	0.250717297	3.225757	4.36E-06	hypermethylated
cg05166490	0.02621	0.245136757	3.225398	6.57E-06	hypermethylated
cg26232818	0.02058	0.192463243	3.225268	3.56E-07	hypermethylated
cg18404308	0.02075	0.19402973	3.225094	2.16E-06	hypermethylated
cg24884519	0.03046	0.284681081	3.224359	5.50E-06	hypermethylated
cg17772028	0.02848	0.265912432	3.22293	9.82E-06	hypermethylated
cg13222752	0.03958	0.369523784	3.222824	1.99E-06	hypermethylated
cg18568990	0.02304	0.214870811	3.221257	2.85E-05	hypermethylated
cg16543094	0.02648	0.246814595	3.220453	4.01E-06	hypermethylated
cg19380001	0.02105	0.196105405	3.219737	1.31E-06	hypermethylated
cg27376182	0.04039	0.375800541	3.217897	3.44E-06	hypermethylated
cg18991611	0.0339	0.315282162	3.217286	4.80E-06	hypermethylated
cg24347663	0.03271	0.303857838	3.215593	5.00E-06	hypermethylated
cg14650610	0.03405	0.316137297	3.214825	1.44E-05	hypermethylated
cg24663256	0.02755	0.255114595	3.211021	2.79E-06	hypermethylated
cg22882665	0.03126	0.288738919	3.207376	6.84E-06	hypermethylated
cg09551147	0.02112	0.194803784	3.20534	3.21E-06	hypermethylated
cg26067203	0.02864	0.263395135	3.201125	0.000117	hypermethylated
cg16616521	0.02873	0.264188108	3.200936	7.86E-07	hypermethylated
cg27357571	0.0317	0.291353514	3.200216	1.29E-06	hypermethylated
cg09682330	0.02546	0.233995135	3.200174	1.47E-06	hypermethylated
cg19074340	0.03782	0.347252973	3.198766	4.60E-06	hypermethylated
cg08495115	0.02672	0.244991351	3.196739	4.47E-05	hypermethylated
cg07573209	0.03257	0.298154595	3.194445	1.07E-06	hypermethylated
cg26998044	0.03407	0.31181027	3.194095	1.37E-05	hypermethylated
cg14416371	0.04562	0.417155135	3.192846	1.33E-06	hypermethylated
cg27513573	0.02445	0.223364324	3.191492	1.39E-06	hypermethylated
cg15146859	0.027	0.246424865	3.190117	4.36E-06	hypermethylated
cg02861380	0.02792	0.254741081	3.189661	4.24E-06	hypermethylated
cg25570913	0.04041	0.368477838	3.188794	1.17E-06	hypermethylated
cg10569606	0.02596	0.236128649	3.185211	2.79E-06	hypermethylated
cg22521696	0.02308	0.209724324	3.183779	2.46E-06	hypermethylated
cg04704053	0.03529	0.320646486	3.183652	1.22E-06	hypermethylated
cg05374412	0.02991	0.271518378	3.18235	3.26E-06	hypermethylated
cg14024461	0.02036	0.184636757	3.18088	7.20E-07	hypermethylated
cg21042456	0.03573	0.323929189	3.180471	5.03E-07	hypermethylated
cg25602684	0.02894	0.261983243	3.178338	4.80E-06	hypermethylated
cg10615414	0.02853	0.257969189	3.176647	2.29E-06	hypermethylated
cg03225817	0.02585	0.233706486	3.176462	1.77E-06	hypermethylated
cg00243313	0.02343	0.211290811	3.172801	2.05E-06	hypermethylated
cg21819468	0.02681	0.241604865	3.171806	3.21E-06	hypermethylated
cg00851770	0.03149	0.283762703	3.171719	1.50E-05	hypermethylated

cg14587524	0.04801	0.432516757	3.171349	2.10E-06	hypermethylated
cg24685755	0.03428	0.308725405	3.170885	2.92E-05	hypermethylated
cg05571581	0.03593	0.323417297	3.170136	8.59E-07	hypermethylated
cg21282630	0.03146	0.283157297	3.170013	1.82E-06	hypermethylated
cg13291704	0.026	0.233903784	3.169332	2.20E-06	hypermethylated
cg20980783	0.02175	0.195644865	3.16915	6.66E-06	hypermethylated
cg04281464	0.03245	0.291634054	3.167869	1.82E-06	hypermethylated
cg06223767	0.04105	0.368409189	3.165855	7.12E-06	hypermethylated
cg23003534	0.02834	0.254108108	3.164531	1.33E-05	hypermethylated
cg26998274	0.04357	0.390372432	3.163444	1.63E-06	hypermethylated
cg15708153	0.0359	0.321463784	3.1626	1.04E-05	hypermethylated
cg03217795	0.0312	0.279152973	3.161438	1.93E-06	hypermethylated
cg13939859	0.02862	0.25599027	3.160993	9.11E-07	hypermethylated
cg24767148	0.02963	0.264672973	3.15908	1.30E-05	hypermethylated
cg06082745	0.02517	0.224814054	3.158955	8.26E-06	hypermethylated
cg04672706	0.02845	0.253642162	3.156294	7.03E-06	hypermethylated
cg11601252	0.03819	0.340316216	3.155609	4.12E-06	hypermethylated
cg13933262	0.02807	0.250112432	3.155476	3.44E-06	hypermethylated
cg21707187	0.03823	0.340538919	3.155043	2.91E-06	hypermethylated
cg03387135	0.02688	0.239188108	3.153541	3.39E-06	hypermethylated
cg01283246	0.03655	0.32499027	3.152453	3.04E-06	hypermethylated
cg05020604	0.04082	0.362801081	3.151831	1.45E-06	hypermethylated
cg13142700	0.03432	0.304224324	3.148014	7.62E-06	hypermethylated
cg13512069	0.01614	0.142843243	3.14572	1.45E-06	hypermethylated
cg13912115	0.02757	0.243876216	3.144978	4.93E-06	hypermethylated
cg19971716	0.02514	0.222069189	3.142953	6.84E-06	hypermethylated
cg27111463	0.0248	0.219054054	3.142875	3.85E-06	hypermethylated
cg00339556	0.02585	0.22818973	3.141998	1.67E-06	hypermethylated
cg20219457	0.03366	0.296663784	3.139722	1.15E-05	hypermethylated
cg09774787	0.03387	0.297886486	3.136683	3.21E-06	hypermethylated
cg16277479	0.0288	0.253251892	3.136432	3.74E-06	hypermethylated
cg14192957	0.03373	0.295780541	3.132423	8.60E-06	hypermethylated
cg23623622	0.03358	0.294448108	3.132339	1.12E-06	hypermethylated
cg03112087	0.04264	0.373718378	3.131672	1.39E-05	hypermethylated
cg16326674	0.03084	0.270183243	3.131064	1.54E-05	hypermethylated
cg06060135	0.04413	0.386559459	3.130859	1.82E-06	hypermethylated
cg14123923	0.03026	0.264464324	3.127589	2.29E-06	hypermethylated
cg02471897	0.04187	0.365717838	3.126742	1.39E-05	hypermethylated
cg21678461	0.02466	0.21527027	3.125904	6.11E-07	hypermethylated
cg19651132	0.03565	0.310915135	3.124547	5.21E-06	hypermethylated
cg12072964	0.0341	0.297178919	3.123488	7.52E-06	hypermethylated
cg24129382	0.0259	0.225207568	3.120231	1.62E-05	hypermethylated
cg16166796	0.0381	0.331224324	3.119946	3.49E-06	hypermethylated
cg20014049	0.02962	0.257015676	3.117213	8.60E-06	hypermethylated
cg16206460	0.04201	0.36402973	3.115252	6.84E-06	hypermethylated
cg21678445	0.04128	0.357610811	3.114875	2.42E-06	hypermethylated
cg22512438	0.02732	0.23646	3.113567	2.99E-06	hypermethylated
cg07700514	0.03926	0.339701081	3.113134	1.70E-06	hypermethylated
cg21185289	0.04077	0.352010811	3.11004	2.87E-06	hypermethylated
cg10311806	0.03183	0.274763243	3.10973	8.71E-06	hypermethylated
cg20744625	0.03098	0.267392973	3.109553	1.02E-05	hypermethylated
cg06341513	0.02981	0.256932973	3.107524	1.20E-05	hypermethylated
cg21161253	0.03043	0.262237838	3.10731	8.59E-07	hypermethylated
cg27420520	0.02275	0.195908108	3.106239	9.07E-06	hypermethylated
cg10079740	0.03297	0.283822162	3.105761	2.45E-05	hypermethylated
cg19656577	0.04052	0.348264865	3.103479	3.59E-06	hypermethylated
cg25623768	0.03738	0.320787568	3.10128	1.46E-05	hypermethylated
cg10983544	0.03365	0.288542162	3.100104	2.10E-06	hypermethylated

cg05214690	0.03146	0.269714595	3.099843	6.48E-06	hypermethylated
cg22001496	0.03931	0.336695676	3.098477	6.84E-06	hypermethylated
cg07533148	0.04752	0.406791351	3.097682	1.41E-06	hypermethylated
cg06971129	0.03313	0.283555676	3.097422	2.64E-06	hypermethylated
cg09474331	0.04607	0.394243784	3.097189	2.56E-06	hypermethylated
cg03963198	0.0375	0.320755135	3.09651	2.42E-06	hypermethylated
cg01805540	0.04053	0.346645405	3.096399	1.70E-06	hypermethylated
cg04188273	0.02845	0.242947027	3.094141	6.22E-06	hypermethylated
cg06740600	0.03162	0.26983027	3.093143	4.36E-06	hypermethylated
cg26796679	0.03252	0.277399459	3.092566	4.58E-05	hypermethylated
cg04030615	0.03196	0.272322703	3.090978	1.33E-06	hypermethylated
cg05089090	0.03528	0.300384865	3.08989	2.10E-06	hypermethylated
cg25976257	0.02538	0.216070811	3.08974	1.28E-05	hypermethylated
cg01688536	0.04728	0.402276757	3.088886	9.11E-07	hypermethylated
cg02745211	0.03822	0.324796757	3.087138	7.93E-06	hypermethylated
cg18348647	0.04302	0.365534054	3.086926	2.32E-06	hypermethylated
cg21502786	0.03311	0.281180541	3.086158	7.72E-06	hypermethylated
cg16857858	0.03667	0.311410811	3.086147	5.45E-05	hypermethylated
cg20482521	0.0289	0.245236216	3.085031	5.11E-07	hypermethylated
cg26477488	0.0378	0.320751351	3.084997	9.69E-06	hypermethylated
cg02320740	0.02378	0.201583243	3.083555	1.41E-06	hypermethylated
cg25317585	0.04845	0.410639459	3.083304	7.93E-06	hypermethylated
cg13281139	0.03179	0.269424324	3.083235	4.24E-06	hypermethylated
cg09582952	0.02562	0.216942703	3.081972	1.50E-05	hypermethylated
cg13919438	0.02415	0.20432	3.080735	2.32E-05	hypermethylated
cg11595545	0.05104	0.431811892	3.080703	1.33E-06	hypermethylated
cg22199216	0.03994	0.33717027	3.077571	7.83E-06	hypermethylated
cg05224741	0.03055	0.257594054	3.075855	7.12E-06	hypermethylated
cg19696317	0.03269	0.275452432	3.074882	2.16E-06	hypermethylated
cg00213479	0.04679	0.394175676	3.074567	3.04E-06	hypermethylated
cg04390523	0.03297	0.277202162	3.071713	2.16E-06	hypermethylated
cg27200446	0.04528	0.380575135	3.071235	1.56E-06	hypermethylated
cg07495363	0.03113	0.261143784	3.068467	5.43E-06	hypermethylated
cg12662072	0.03557	0.298187568	3.067487	1.64E-05	hypermethylated
cg04575395	0.03429	0.286764324	3.064006	5.28E-06	hypermethylated
cg14168530	0.03753	0.313514054	3.062414	3.17E-06	hypermethylated
cg06818710	0.02897	0.241848649	3.061473	2.16E-06	hypermethylated
cg21858485	0.03141	0.261998378	3.060262	5.35E-06	hypermethylated
cg13699355	0.05122	0.42704973	3.059625	2.75E-06	hypermethylated
cg16571983	0.03072	0.25588	3.058217	2.23E-06	hypermethylated
cg09868336	0.03448	0.286969189	3.057064	8.04E-06	hypermethylated
cg12919520	0.02734	0.22704973	3.053923	1.02E-06	hypermethylated
cg26526374	0.02891	0.239958378	3.053144	9.82E-06	hypermethylated
cg27562682	0.03418	0.28329027	3.051057	3.79E-06	hypermethylated
cg27477373	0.03921	0.324892973	3.050671	1.96E-06	hypermethylated
cg01952234	0.0313	0.259071892	3.049118	1.73E-05	hypermethylated
cg07914084	0.03776	0.312424865	3.048579	5.50E-06	hypermethylated
cg19912142	0.04548	0.375888108	3.046999	1.12E-06	hypermethylated
cg20987924	0.03224	0.266428649	3.046826	5.50E-06	hypermethylated
cg10358533	0.04314	0.356244865	3.045771	2.42E-06	hypermethylated
cg18424634	0.02392	0.197436216	3.045097	1.82E-06	hypermethylated
cg23632875	0.04227	0.348827568	3.044808	3.90E-06	hypermethylated
cg01419831	0.03841	0.316847027	3.044233	2.36E-06	hypermethylated
cg03059131	0.03688	0.304204865	3.044133	7.63E-07	hypermethylated
cg09252495	0.02731	0.225022162	3.042566	1.54E-05	hypermethylated
cg19035107	0.02459	0.202145405	3.03925	2.92E-07	hypermethylated
cg07080358	0.034	0.279405946	3.038756	5.58E-06	hypermethylated
cg13356896	0.04676	0.383896216	3.03737	6.75E-06	hypermethylated

cg00421139	0.04022	0.330163784	3.037197	3.76E-05	hypermethylated
cg15836635	0.03565	0.292598919	3.03695	6.66E-06	hypermethylated
cg04293733	0.03565	0.292496757	3.036447	9.96E-06	hypermethylated
cg23448584	0.03283	0.269042162	3.034746	6.84E-06	hypermethylated
cg14712186	0.028	0.229303784	3.033761	3.49E-06	hypermethylated
cg12040830	0.05189	0.424717297	3.032974	3.49E-06	hypermethylated
cg09894698	0.03153	0.257829189	3.031619	5.89E-06	hypermethylated
cg18147485	0.04583	0.374309189	3.029866	8.94E-05	hypermethylated
cg07912789	0.04146	0.338483784	3.029295	3.85E-06	hypermethylated
cg08441170	0.03882	0.316307027	3.026454	3.67E-05	hypermethylated
cg03758150	0.04426	0.359932432	3.023651	2.20E-06	hypermethylated
cg09935282	0.03536	0.287548649	3.023616	5.72E-05	hypermethylated
cg20072171	0.03724	0.302639459	3.022675	1.72E-06	hypermethylated
cg07519873	0.03015	0.244727568	3.020947	1.58E-05	hypermethylated
cg21748223	0.03156	0.256078378	3.020416	2.95E-06	hypermethylated
cg24885417	0.0351	0.284570811	3.019245	8.04E-06	hypermethylated
cg01995480	0.03597	0.2914	3.018135	2.85E-05	hypermethylated
cg14487131	0.04399	0.356334595	3.017985	5.21E-06	hypermethylated
cg12700904	0.03109	0.251603243	3.016628	4.94E-05	hypermethylated
cg07193766	0.03643	0.294585946	3.01549	1.04E-05	hypermethylated
cg04510512	0.0285	0.230151351	3.013549	2.27E-05	hypermethylated
cg09360770	0.03973	0.320831892	3.013517	1.58E-05	hypermethylated
cg24464397	0.02554	0.206171351	3.013013	5.07E-06	hypermethylated
cg14562712	0.03466	0.279701622	3.012545	1.48E-05	hypermethylated
cg07547000	0.02727	0.219733514	3.010368	1.20E-05	hypermethylated
cg21239311	0.02723	0.219214595	3.009075	3.95E-06	hypermethylated
cg24031355	0.03418	0.275035676	3.008394	3.21E-06	hypermethylated
cg27260772	0.05721	0.460112432	3.007647	7.41E-07	hypermethylated
cg10188823	0.03708	0.297854054	3.005892	1.12E-05	hypermethylated
cg03132773	0.0279	0.224108108	3.005858	4.67E-06	hypermethylated
cg12877251	0.03528	0.28314973	3.004643	4.30E-06	hypermethylated
cg17535595	0.03803	0.304774595	3.002533	5.14E-06	hypermethylated
cg26682580	0.03075	0.246424865	3.00249	4.86E-06	hypermethylated
cg18630667	0.04923	0.394509189	3.002449	1.18E-06	hypermethylated
cg14038391	0.03904	0.312726486	3.001876	1.14E-05	hypermethylated
cg08466792	0.04737	0.379442703	3.001836	8.34E-07	hypermethylated
cg05135549	0.04321	0.345655135	2.999896	8.10E-07	hypermethylated
cg03159124	0.03169	0.25344973	2.9996	4.12E-06	hypermethylated
cg04123776	0.03172	0.253559459	2.998859	2.10E-06	hypermethylated
cg26521404	0.05372	0.429391351	2.998762	1.15E-06	hypermethylated
cg26809635	0.04977	0.397658919	2.998183	5.00E-06	hypermethylated
cg23180938	0.04213	0.336516216	2.997756	6.93E-06	hypermethylated
cg18884037	0.02806	0.223842162	2.995895	6.22E-06	hypermethylated
cg13611347	0.02698	0.214886486	2.993613	3.12E-06	hypermethylated
cg02383130	0.03556	0.282741622	2.991157	2.91E-06	hypermethylated
cg00557947	0.04086	0.324781622	2.990709	2.57E-06	hypermethylated
cg15048991	0.0411	0.326648108	2.990527	2.95E-06	hypermethylated
cg22603247	0.03965	0.314842162	2.989236	1.15E-05	hypermethylated
cg18646207	0.0329	0.261092432	2.988401	7.72E-06	hypermethylated
cg04669574	0.04049	0.321185405	2.987769	1.01E-05	hypermethylated
cg13962212	0.03109	0.24650973	2.987122	4.67E-06	hypermethylated
cg15630907	0.02616	0.207390811	2.986918	1.64E-05	hypermethylated
cg07931411	0.02862	0.226869189	2.986765	1.93E-06	hypermethylated
cg04784475	0.03512	0.278271892	2.98613	1.07E-06	hypermethylated
cg08235864	0.03967	0.314092973	2.985071	3.17E-06	hypermethylated
cg13794530	0.04833	0.382317838	2.983782	2.99E-06	hypermethylated
cg08283882	0.03175	0.250992973	2.982818	4.70E-05	hypermethylated
cg21779611	0.03537	0.279570811	2.982616	7.98E-07	hypermethylated

cg25645268	0.03349	0.264268649	2.980203	8.34E-07	hypermethylated
cg10800833	0.03536	0.278896216	2.979538	1.51E-06	hypermethylated
cg21541833	0.03444	0.271522162	2.978913	2.95E-06	hypermethylated
cg14394692	0.03307	0.260605946	2.978275	1.56E-06	hypermethylated
cg26477573	0.03308	0.260246486	2.975848	0.000251	hypermethylated
cg05151154	0.0457	0.358931351	2.973442	3.44E-06	hypermethylated
cg05917460	0.03718	0.291755676	2.972162	9.07E-06	hypermethylated
cg06785999	0.04251	0.333576757	2.972145	4.86E-06	hypermethylated
cg25691167	0.05002	0.392491892	2.972086	1.93E-06	hypermethylated
cg04846243	0.03238	0.253936757	2.971294	6.38E-05	hypermethylated
cg02467990	0.05254	0.411698378	2.9701	3.35E-06	hypermethylated
cg19619405	0.05335	0.417938378	2.96973	1.93E-06	hypermethylated
cg20810478	0.05197	0.406935676	2.96905	1.99E-06	hypermethylated
cg22049569	0.02526	0.197723243	2.968556	5.73E-06	hypermethylated
cg20618622	0.03233	0.252998919	2.968186	1.99E-05	hypermethylated
cg22773899	0.03364	0.26322	2.96802	2.87E-06	hypermethylated
cg27277463	0.05032	0.393731351	2.968008	5.00E-06	hypermethylated
cg25078150	0.03661	0.286446486	2.967956	1.40E-05	hypermethylated
cg02809707	0.0334	0.261321081	2.967904	1.09E-05	hypermethylated
cg19115272	0.02484	0.194277838	2.967384	3.54E-06	hypermethylated
cg24245418	0.03907	0.30554	2.967228	4.36E-05	hypermethylated
cg06382344	0.03446	0.269261622	2.966014	1.54E-06	hypermethylated
cg18578405	0.03948	0.308407568	2.965644	2.36E-06	hypermethylated
cg14485004	0.03117	0.243355676	2.964836	1.14E-05	hypermethylated
cg19484420	0.04536	0.35411027	2.964706	1.29E-06	hypermethylated
cg18063733	0.03566	0.278047027	2.96295	7.03E-06	hypermethylated
cg25116216	0.03979	0.310014595	2.961858	3.49E-06	hypermethylated
cg10152131	0.05281	0.411131351	2.960716	8.34E-07	hypermethylated
cg15987088	0.02144	0.166506486	2.957202	4.33E-07	hypermethylated
cg08575330	0.03467	0.269148649	2.956643	2.49E-06	hypermethylated
cg22356339	0.03611	0.280272432	2.95636	3.74E-06	hypermethylated
cg10293925	0.04133	0.320422703	2.954715	5.58E-06	hypermethylated
cg07978472	0.04032	0.312471892	2.954159	3.26E-06	hypermethylated
cg25973534	0.04186	0.324051892	2.952581	1.02E-05	hypermethylated
cg14324370	0.03817	0.295123243	2.950806	1.08E-05	hypermethylated
cg12219082	0.02974	0.229928649	2.95071	3.39E-06	hypermethylated
cg15126544	0.03516	0.27167027	2.94985	4.42E-06	hypermethylated
cg03315215	0.02745	0.212087568	2.949782	3.30E-06	hypermethylated
cg08567279	0.03302	0.255077838	2.949526	1.93E-06	hypermethylated
cg21885046	0.04278	0.330088649	2.947845	1.06E-05	hypermethylated
cg09524455	0.02976	0.229128108	2.944708	5.43E-06	hypermethylated
cg18932798	0.04046	0.311421622	2.944301	3.12E-06	hypermethylated
cg07881405	0.04357	0.33492973	2.942451	2.71E-06	hypermethylated
cg19497031	0.04261	0.326913514	2.939645	1.88E-06	hypermethylated
cg25723050	0.0377	0.288954595	2.938206	2.51E-05	hypermethylated
cg05888917	0.03051	0.233708649	2.937357	8.83E-06	hypermethylated
cg08857144	0.04001	0.306332432	2.936666	9.69E-06	hypermethylated
cg15556502	0.04425	0.338441081	2.935155	2.49E-06	hypermethylated
cg02237470	0.03994	0.30532973	2.934462	1.11E-05	hypermethylated
cg11501438	0.02565	0.196015676	2.933938	3.79E-06	hypermethylated
cg27528510	0.04134	0.315697838	2.932934	1.20E-05	hypermethylated
cg08228703	0.03159	0.24112	2.932211	2.10E-06	hypermethylated
cg17003293	0.03346	0.255147027	2.930819	8.15E-06	hypermethylated
cg09813525	0.04624	0.352534595	2.930552	3.59E-06	hypermethylated
cg19358877	0.03782	0.288067568	2.929186	1.31E-05	hypermethylated
cg03276408	0.03456	0.262781622	2.926689	1.93E-06	hypermethylated
cg07147449	0.04038	0.306410811	2.923754	1.84E-05	hypermethylated
cg05338433	0.03139	0.237983784	2.922486	2.29E-06	hypermethylated

cg21929943	0.03312	0.251075676	2.922348	7.22E-06	hypermethylated
cg01743841	0.0493	0.373670811	2.922108	1.20E-05	hypermethylated
cg24416513	0.06043	0.457792432	2.921357	1.01E-06	hypermethylated
cg09971314	0.05247	0.397407027	2.921053	2.46E-06	hypermethylated
cg26132774	0.05377	0.407224865	2.920952	1.58E-06	hypermethylated
cg07770968	0.04521	0.342394054	2.920944	7.72E-06	hypermethylated
cg15262242	0.03499	0.264660541	2.919129	3.35E-06	hypermethylated
cg00908291	0.03476	0.262794595	2.918436	1.44E-05	hypermethylated
cg26122980	0.04971	0.375630811	2.917707	3.44E-06	hypermethylated
cg11825621	0.03672	0.277167027	2.916118	0.000248	hypermethylated
cg15313459	0.03678	0.277603243	2.916031	1.87E-05	hypermethylated
cg22232327	0.03602	0.271744865	2.915383	8.37E-06	hypermethylated
cg26938597	0.02817	0.21236973	2.914347	3.49E-06	hypermethylated
cg03401357	0.03555	0.268003784	2.914332	2.92E-05	hypermethylated
cg22830113	0.04943	0.371808108	2.911099	2.57E-05	hypermethylated
cg19047292	0.03499	0.263185946	2.911068	3.69E-06	hypermethylated
cg20072442	0.03158	0.237379459	2.910112	7.52E-06	hypermethylated
cg05627639	0.05689	0.427450811	2.909511	3.49E-06	hypermethylated
cg23524195	0.03451	0.259200541	2.908982	3.30E-06	hypermethylated
cg16002355	0.04073	0.305846486	2.908644	8.71E-06	hypermethylated
cg08753373	0.03779	0.283541081	2.907481	7.03E-06	hypermethylated
cg11979589	0.03261	0.244559459	2.906799	2.74E-05	hypermethylated
cg14768785	0.03343	0.250374595	2.904873	9.66E-07	hypermethylated
cg02483484	0.02701	0.201768108	2.901133	3.79E-06	hypermethylated
cg07113642	0.05773	0.431088108	2.90059	3.21E-06	hypermethylated
cg24362812	0.04386	0.327264865	2.899481	4.24E-06	hypermethylated
cg18627360	0.03658	0.272935676	2.899434	7.83E-06	hypermethylated
cg03376794	0.0373	0.278296757	2.899377	1.39E-06	hypermethylated
cg03595755	0.03015	0.224936757	2.89929	2.10E-05	hypermethylated
cg14146100	0.02473	0.18443027	2.898741	3.17E-06	hypermethylated
cg23054189	0.05674	0.423013514	2.898266	2.87E-06	hypermethylated
cg05376374	0.05202	0.387792432	2.898146	3.39E-06	hypermethylated
cg08870743	0.04137	0.308294595	2.897653	3.49E-06	hypermethylated
cg09385093	0.03718	0.276811351	2.896304	1.21E-05	hypermethylated
cg13481132	0.03966	0.295104324	2.895468	5.14E-06	hypermethylated
cg12908522	0.02286	0.169964865	2.894339	2.46E-06	hypermethylated
cg08939095	0.04096	0.304413514	2.893745	2.39E-06	hypermethylated
cg07976064	0.05175	0.384470811	2.893243	1.67E-06	hypermethylated
cg08893692	0.05094	0.378226486	2.89238	5.34E-07	hypermethylated
cg20691140	0.02969	0.220282703	2.891307	3.59E-06	hypermethylated
cg21511365	0.03403	0.252428108	2.890994	1.75E-05	hypermethylated
cg26789779	0.03817	0.283005405	2.890319	1.42E-05	hypermethylated
cg05560435	0.04026	0.29800973	2.88794	3.30E-06	hypermethylated
cg13458645	0.03542	0.26172	2.885388	8.71E-06	hypermethylated
cg22435300	0.02256	0.166583243	2.884404	1.40E-05	hypermethylated
cg00596508	0.03345	0.24667027	2.882506	6.15E-05	hypermethylated
cg08004425	0.04066	0.299628649	2.881493	1.58E-06	hypermethylated
cg04658772	0.03058	0.225285946	2.881097	2.92E-05	hypermethylated
cg16021909	0.05529	0.407109189	2.880325	2.42E-06	hypermethylated
cg09238180	0.04752	0.349856757	2.880158	2.26E-06	hypermethylated
cg20803857	0.04204	0.309124324	2.878353	2.07E-05	hypermethylated
cg12973591	0.03813	0.2802	2.877459	1.42E-05	hypermethylated
cg09507526	0.03466	0.25452	2.876435	5.14E-06	hypermethylated
cg18206027	0.04824	0.354236757	2.876412	3.44E-06	hypermethylated
cg15034345	0.04472	0.328235135	2.875738	3.74E-06	hypermethylated
cg05901579	0.04924	0.361169189	2.874772	4.07E-06	hypermethylated
cg20279673	0.02796	0.204876216	2.873316	3.85E-06	hypermethylated
cg00001747	0.05107	0.374171351	2.873151	4.80E-06	hypermethylated

cg06463958	0.04369	0.319896216	2.872229	4.48E-06	hypermethylated
cg25936054	0.04572	0.334546486	2.871309	3.39E-06	hypermethylated
cg05663341	0.04079	0.298314054	2.870545	0.00013	hypermethylated
cg16295725	0.05138	0.375684865	2.870244	1.46E-05	hypermethylated
cg15051226	0.03103	0.226845405	2.869974	2.10E-06	hypermethylated
cg04865180	0.04956	0.36203027	2.868862	3.04E-06	hypermethylated
cg26814276	0.04111	0.300290811	2.868799	2.71E-06	hypermethylated
cg08313939	0.04439	0.324112432	2.868188	1.22E-06	hypermethylated
cg03714619	0.04883	0.356511892	2.868111	1.27E-06	hypermethylated
cg25366582	0.03724	0.271891892	2.868108	1.02E-05	hypermethylated
cg14287112	0.03437	0.250454054	2.865324	9.82E-06	hypermethylated
cg11664500	0.03585	0.261085405	2.864477	8.83E-06	hypermethylated
cg27199820	0.04463	0.325022162	2.864452	3.49E-06	hypermethylated
cg06288251	0.03909	0.284506486	2.86359	1.02E-05	hypermethylated
cg14306734	0.03728	0.271187568	2.862817	0.000283	hypermethylated
cg11334771	0.04387	0.318865946	2.861643	2.02E-06	hypermethylated
cg20185017	0.04415	0.320878919	2.861544	7.32E-06	hypermethylated
cg05809668	0.05459	0.396662162	2.861202	1.35E-06	hypermethylated
cg03940848	0.03582	0.260227027	2.860934	2.81E-05	hypermethylated
cg04347874	0.0607	0.44034	2.858849	2.02E-06	hypermethylated
cg21517947	0.0463	0.335619459	2.857742	1.35E-05	hypermethylated
cg05298922	0.03977	0.288272973	2.857683	1.28E-05	hypermethylated
cg01956420	0.05163	0.373916216	2.856434	9.31E-06	hypermethylated
cg05085230	0.03398	0.245962162	2.855679	1.39E-05	hypermethylated
cg09195198	0.03735	0.270116757	2.854403	2.61E-05	hypermethylated
cg11985360	0.03143	0.227227568	2.853924	4.36E-06	hypermethylated
cg21093192	0.04394	0.316724324	2.849621	4.54E-06	hypermethylated
cg15975865	0.04992	0.359716216	2.849169	2.56E-06	hypermethylated
cg06419761	0.03313	0.23868973	2.848926	1.39E-05	hypermethylated
cg03307465	0.03701	0.266493514	2.848113	8.37E-06	hypermethylated
cg18786873	0.04465	0.321227568	2.846864	1.41E-06	hypermethylated
cg23048481	0.0312	0.224311351	2.845885	2.83E-06	hypermethylated
cg16172814	0.03686	0.264946486	2.845573	5.43E-06	hypermethylated
cg19679633	0.04566	0.328113514	2.845192	1.99E-06	hypermethylated
cg13088368	0.02721	0.195454054	2.844621	7.62E-06	hypermethylated
cg16856286	0.05066	0.363881622	2.84455	3.54E-06	hypermethylated
cg17741501	0.05003	0.359264865	2.844182	2.95E-06	hypermethylated
cg22409854	0.03404	0.244395135	2.843913	5.43E-06	hypermethylated
cg14473327	0.03433	0.24642973	2.843635	1.52E-05	hypermethylated
cg14441976	0.04473	0.320783243	2.842284	7.52E-06	hypermethylated
cg07926691	0.04629	0.331957297	2.842225	3.21E-06	hypermethylated
cg23778596	0.03675	0.263292432	2.84085	1.42E-05	hypermethylated
cg13445796	0.05827	0.417221622	2.839989	1.77E-06	hypermethylated
cg19492047	0.03659	0.261884865	2.839411	8.04E-06	hypermethylated
cg05663573	0.04064	0.290642162	2.838272	3.35E-06	hypermethylated
cg23847381	0.03282	0.234646486	2.837842	2.96E-05	hypermethylated
cg01638213	0.04956	0.354203784	2.837332	3.59E-06	hypermethylated
cg19513834	0.05065	0.361947568	2.837147	1.15E-06	hypermethylated
cg09797577	0.04914	0.351063243	2.836761	2.83E-06	hypermethylated
cg23243867	0.04865	0.347517297	2.836573	1.04E-05	hypermethylated
cg18507379	0.05044	0.360178378	2.836071	4.54E-06	hypermethylated
cg04568492	0.03954	0.282264324	2.835662	4.80E-06	hypermethylated
cg14216285	0.03055	0.217931892	2.834633	4.01E-06	hypermethylated
cg12412079	0.03515	0.250735676	2.834571	2.24E-05	hypermethylated
cg19875547	0.05103	0.363954595	2.834341	6.57E-06	hypermethylated
cg24820783	0.03826	0.272846486	2.834181	6.39E-06	hypermethylated
cg13539545	0.06287	0.448302162	2.834028	1.12E-06	hypermethylated
cg05523056	0.03051	0.217552432	2.834009	1.08E-05	hypermethylated

cg20935165	0.04644	0.330705405	2.832107	4.12E-06	hypermethylated
cg02087954	0.04154	0.29558973	2.831023	2.38E-05	hypermethylated
cg05446629	0.056	0.398099459	2.82963	4.36E-06	hypermethylated
cg03063639	0.05964	0.423968108	2.829604	3.12E-06	hypermethylated
cg14649650	0.04419	0.313718378	2.827678	4.80E-06	hypermethylated
cg11357746	0.03184	0.225995135	2.827379	4.48E-06	hypermethylated
cg25950112	0.04303	0.305224865	2.826458	1.15E-06	hypermethylated
cg25474372	0.04181	0.296457838	2.825907	5.28E-06	hypermethylated
cg27058257	0.06026	0.427028649	2.82506	5.14E-06	hypermethylated
cg06118384	0.03296	0.233527568	2.824805	7.32E-06	hypermethylated
cg13912117	0.05109	0.36194	2.824638	6.48E-06	hypermethylated
cg26650846	0.03769	0.266973514	2.824443	1.28E-05	hypermethylated
cg02081266	0.04413	0.312207568	2.822674	8.60E-06	hypermethylated
cg06585708	0.04278	0.302514595	2.821996	1.12E-06	hypermethylated
cg18725867	0.04993	0.353073514	2.82199	1.05E-06	hypermethylated
cg06094615	0.03653	0.258254595	2.82164	3.96E-06	hypermethylated
cg17386093	0.0277	0.195547027	2.819558	7.12E-06	hypermethylated
cg05766140	0.04308	0.304038919	2.819166	3.74E-06	hypermethylated
cg05593641	0.04781	0.337231351	2.818354	5.14E-06	hypermethylated
cg02881570	0.04405	0.31046	2.817193	1.31E-05	hypermethylated
cg08696727	0.03912	0.275666486	2.816946	0.000454	hypermethylated
cg27655158	0.03608	0.254148108	2.816398	3.04E-05	hypermethylated
cg15584445	0.02609	0.18376973	2.81633	2.56E-06	hypermethylated
cg03951219	0.04733	0.333215135	2.815627	3.12E-06	hypermethylated
cg19909349	0.05135	0.361492432	2.815529	3.54E-06	hypermethylated
cg27501878	0.05141	0.361816216	2.815136	8.71E-06	hypermethylated
cg26079753	0.04452	0.313217297	2.814638	1.01E-05	hypermethylated
cg03779241	0.04038	0.284002162	2.814189	3.90E-06	hypermethylated
cg21331088	0.0417	0.29315027	2.813521	1.48E-05	hypermethylated
cg06829686	0.04975	0.34961027	2.812979	5.00E-06	hypermethylated
cg10715265	0.03689	0.259214595	2.812845	0.000135	hypermethylated
cg18318649	0.04481	0.314772973	2.812419	4.12E-06	hypermethylated
cg08063125	0.04114	0.28876973	2.811306	8.37E-06	hypermethylated
cg22954449	0.05789	0.406337297	2.811292	7.52E-06	hypermethylated
cg01360618	0.05453	0.382629189	2.810825	4.36E-06	hypermethylated
cg14230666	0.04564	0.320202703	2.810615	3.21E-06	hypermethylated
cg10903903	0.0628	0.440592432	2.810608	3.85E-06	hypermethylated
cg02023345	0.04036	0.28312973	2.810465	5.00E-06	hypermethylated
cg04012266	0.03543	0.248534054	2.8104	1.84E-05	hypermethylated
cg17754510	0.04744	0.332716216	2.810116	2.99E-06	hypermethylated
cg22759823	0.06305	0.441708108	2.808525	1.63E-06	hypermethylated
cg18403551	0.03876	0.271477838	2.808194	2.42E-06	hypermethylated
cg03227184	0.04069	0.284964324	2.808035	1.02E-06	hypermethylated
cg26492446	0.0588	0.411760541	2.807918	5.73E-06	hypermethylated
cg16364121	0.04569	0.319722703	2.806871	1.94E-05	hypermethylated
cg00530925	0.03669	0.256738378	2.80684	1.18E-05	hypermethylated
cg23318063	0.04633	0.324152432	2.806654	7.22E-06	hypermethylated
cg13759674	0.04486	0.313769189	2.806202	0.00032	hypermethylated
cg19456540	0.04935	0.344897838	2.805047	3.49E-06	hypermethylated
cg07972135	0.04113	0.287251892	2.804053	1.02E-05	hypermethylated
cg01893212	0.06167	0.430684324	2.80399	7.42E-06	hypermethylated
cg13601435	0.05374	0.374987568	2.802775	2.02E-05	hypermethylated
cg23291301	0.05208	0.363361622	2.802605	2.71E-06	hypermethylated
cg04205107	0.02783	0.194137297	2.802364	8.83E-06	hypermethylated
cg14603098	0.05256	0.366501081	2.80178	2.95E-06	hypermethylated
cg09022422	0.04195	0.292477297	2.801582	6.93E-06	hypermethylated
cg20474271	0.03424	0.238721081	2.801571	1.46E-05	hypermethylated
cg24407243	0.02424	0.168982703	2.801414	7.41E-07	hypermethylated

cg22871668	0.03602	0.251055135	2.801134	1.58E-05	hypermethylated
cg20276585	0.03582	0.249561622	2.800559	5.65E-05	hypermethylated
cg21956337	0.03923	0.273049189	2.799132	1.75E-06	hypermethylated
cg05923687	0.03616	0.251658919	2.799003	3.85E-06	hypermethylated
cg03238797	0.04118	0.286534054	2.798691	6.30E-06	hypermethylated
cg14492293	0.03145	0.218565405	2.796933	5.97E-06	hypermethylated
cg14060496	0.05212	0.362098919	2.796475	4.48E-06	hypermethylated
cg04184836	0.04957	0.344265405	2.795982	7.12E-06	hypermethylated
cg15808943	0.04996	0.34680973	2.795299	1.90E-06	hypermethylated
cg14944647	0.04598	0.319118378	2.795013	3.96E-06	hypermethylated
cg15672437	0.05289	0.366681622	2.793461	3.49E-06	hypermethylated
cg06746118	0.04499	0.311884324	2.793335	6.08E-05	hypermethylated
cg04332534	0.04714	0.326712973	2.793	1.93E-06	hypermethylated
cg20718350	0.04791	0.331803784	2.791932	1.44E-05	hypermethylated
cg17429382	0.03983	0.275828649	2.791845	9.19E-06	hypermethylated
cg11260904	0.02906	0.201144324	2.791124	1.42E-05	hypermethylated
cg14765959	0.04844	0.335226486	2.790865	6.66E-06	hypermethylated
cg00268840	0.04198	0.290402703	2.790281	6.01E-05	hypermethylated
cg17541528	0.05453	0.377077838	2.78974	1.12E-05	hypermethylated
cg10334767	0.049	0.338802703	2.789592	1.52E-05	hypermethylated
cg05630016	0.04074	0.281680541	2.789542	6.48E-06	hypermethylated
cg11878331	0.04846	0.33498973	2.789251	1.22E-06	hypermethylated
cg08722774	0.02949	0.203682162	2.788022	2.71E-05	hypermethylated
cg05310764	0.03439	0.237396216	2.787236	2.02E-06	hypermethylated
cg22295787	0.03575	0.246558378	2.785914	4.18E-06	hypermethylated
cg27600205	0.04581	0.315883243	2.785657	7.32E-06	hypermethylated
cg07188591	0.04106	0.282947568	2.784729	1.05E-05	hypermethylated
cg25113237	0.05157	0.355304865	2.784453	6.99E-07	hypermethylated
cg04475027	0.05089	0.350292432	2.783106	3.30E-06	hypermethylated
cg26419728	0.03277	0.225435135	2.782265	5.89E-06	hypermethylated
cg15228928	0.04971	0.341662703	2.780965	3.69E-06	hypermethylated
cg04917181	0.04204	0.288933514	2.780903	9.95E-05	hypermethylated
cg06715976	0.04224	0.290171892	2.780226	0.000147	hypermethylated
cg01052879	0.03636	0.249693514	2.779734	9.19E-06	hypermethylated
cg08806408	0.04052	0.278127027	2.779038	4.36E-06	hypermethylated
cg16376000	0.04224	0.289925405	2.779	5.86E-05	hypermethylated
cg13839457	0.05474	0.375716216	2.778976	2.30E-05	hypermethylated
cg23383871	0.03927	0.26938973	2.778195	1.04E-05	hypermethylated
cg15031661	0.06249	0.428596757	2.777924	5.81E-06	hypermethylated
cg26134895	0.05675	0.388614054	2.775646	1.60E-05	hypermethylated
cg06122635	0.0442	0.302611351	2.775348	5.28E-06	hypermethylated
cg25039722	0.04849	0.331979459	2.775335	1.09E-05	hypermethylated
cg02214096	0.02849	0.195044324	2.775274	2.99E-06	hypermethylated
cg04021697	0.05538	0.379034054	2.774891	4.12E-06	hypermethylated
cg07068756	0.05105	0.349357838	2.774723	8.83E-06	hypermethylated
cg03709091	0.03947	0.270102703	2.77468	6.95E-05	hypermethylated
cg13267264	0.06226	0.425493514	2.77276	4.12E-06	hypermethylated
cg20648847	0.02837	0.193870811	2.772658	4.82E-05	hypermethylated
cg24266670	0.05475	0.374095676	2.772476	1.04E-05	hypermethylated
cg16987305	0.05104	0.348741081	2.772456	1.23E-05	hypermethylated
cg19570244	0.05154	0.352071351	2.772103	5.89E-06	hypermethylated
cg02012703	0.06481	0.442557297	2.771576	3.54E-06	hypermethylated
cg05874732	0.03777	0.257911892	2.771566	1.45E-06	hypermethylated
cg16435571	0.0371	0.253259459	2.771125	6.57E-06	hypermethylated
cg22868282	0.04229	0.288611351	2.77074	9.31E-06	hypermethylated
cg24980653	0.07002	0.477807027	2.770589	4.01E-06	hypermethylated
cg18673954	0.04176	0.284963243	2.770582	7.83E-06	hypermethylated
cg16150752	0.04635	0.316120541	2.769834	6.22E-06	hypermethylated

cg19939997	0.02969	0.202414595	2.769264	1.62E-05	hypermethylated
cg18621091	0.03188	0.217321622	2.769108	7.62E-06	hypermethylated
cg19144684	0.03263	0.222338919	2.76849	3.04E-06	hypermethylated
cg08876932	0.03845	0.261796216	2.767389	4.15E-05	hypermethylated
cg17059658	0.06205	0.422365405	2.766989	1.33E-06	hypermethylated
cg15409013	0.04061	0.276224865	2.765936	1.14E-05	hypermethylated
cg10541517	0.03306	0.224624865	2.764359	5.89E-06	hypermethylated
cg17380661	0.04818	0.326843784	2.762095	1.30E-05	hypermethylated
cg22517656	0.03594	0.243760541	2.761802	8.15E-06	hypermethylated
cg05206884	0.05837	0.395816216	2.761532	4.01E-06	hypermethylated
cg20980055	0.02805	0.190195676	2.761412	7.32E-06	hypermethylated
cg12892506	0.04687	0.317708108	2.760965	2.16E-06	hypermethylated
cg20146541	0.06456	0.437543784	2.760715	1.90E-06	hypermethylated
cg01200640	0.04218	0.285833514	2.760544	1.41E-06	hypermethylated
cg08182446	0.05006	0.339055676	2.759792	2.95E-06	hypermethylated
cg16993043	0.03946	0.267116757	2.759008	1.75E-05	hypermethylated
cg10658542	0.04379	0.296367568	2.758714	8.04E-06	hypermethylated
cg17391928	0.04881	0.330317838	2.758606	3.90E-06	hypermethylated
cg19579167	0.03853	0.260694054	2.758304	8.03E-05	hypermethylated
cg03700449	0.04475	0.302725946	2.758053	5.14E-06	hypermethylated
cg05223720	0.04994	0.337785405	2.757839	7.12E-06	hypermethylated
cg13764991	0.03677	0.248697838	2.757793	8.48E-06	hypermethylated
cg12300724	0.04168	0.281894595	2.757729	2.32E-05	hypermethylated
cg25987923	0.03155	0.213376757	2.757691	1.02E-05	hypermethylated
cg24847829	0.05442	0.367744865	2.756496	5.14E-06	hypermethylated
cg12162138	0.0507	0.342576216	2.756367	4.36E-06	hypermethylated
cg13139972	0.0464	0.312935676	2.753669	5.51E-05	hypermethylated
cg08066943	0.03999	0.269617297	2.753202	1.96E-06	hypermethylated
cg22763718	0.04909	0.330834054	2.752607	3.49E-06	hypermethylated
cg08190858	0.05291	0.356522703	2.752382	3.30E-06	hypermethylated
cg10036918	0.02864	0.192948108	2.75211	3.62E-05	hypermethylated
cg25644556	0.05223	0.351751351	2.751605	1.94E-05	hypermethylated
cg06616729	0.05617	0.378175135	2.751183	3.69E-06	hypermethylated
cg23235241	0.04606	0.310059459	2.750959	3.12E-06	hypermethylated
cg17509220	0.04871	0.327636216	2.749805	5.50E-06	hypermethylated
cg06653699	0.03774	0.253542703	2.748062	8.37E-06	hypermethylated
cg26272270	0.03458	0.23231027	2.748043	6.39E-06	hypermethylated
cg04454951	0.05165	0.346812973	2.747318	2.71E-06	hypermethylated
cg20340508	0.05217	0.350166486	2.746749	4.67E-06	hypermethylated
cg13692446	0.05135	0.344662162	2.746747	1.26E-06	hypermethylated
cg26777883	0.04576	0.306772432	2.74501	8.26E-06	hypermethylated
cg15509687	0.03574	0.239559459	2.744772	1.77E-05	hypermethylated
cg23462956	0.02674	0.179144865	2.744055	4.01E-06	hypermethylated
cg19369022	0.04468	0.299025946	2.74257	3.90E-06	hypermethylated
cg06338562	0.04129	0.276308108	2.742414	2.38E-05	hypermethylated
cg14007067	0.03994	0.267083243	2.741383	2.64E-06	hypermethylated
cg24928391	0.04114	0.274993514	2.740784	5.58E-05	hypermethylated
cg01870995	0.03527	0.235627027	2.739992	1.87E-05	hypermethylated
cg11328303	0.04575	0.305573514	2.739676	4.60E-06	hypermethylated
cg09118932	0.05322	0.355399459	2.739401	3.64E-06	hypermethylated
cg09969277	0.05796	0.386924865	2.738924	1.65E-06	hypermethylated
cg23089825	0.05656	0.377111351	2.737137	1.72E-06	hypermethylated
cg18082337	0.05654	0.376940541	2.736993	1.88E-06	hypermethylated
cg09537620	0.04709	0.313677297	2.735788	1.93E-06	hypermethylated
cg00058329	0.04537	0.30219027	2.735647	1.54E-05	hypermethylated
cg26692294	0.04367	0.290751351	2.735071	6.31E-05	hypermethylated
cg03611452	0.05091	0.338530811	2.733266	2.57E-06	hypermethylated
cg20979852	0.045	0.299221081	2.733215	6.05E-06	hypermethylated

cg18380175	0.03963	0.263452973	2.732881	6.13E-06	hypermethylated
cg17290701	0.04224	0.280615676	2.731914	3.27E-05	hypermethylated
cg26460092	0.05934	0.393770811	2.730279	1.77E-05	hypermethylated
cg04209911	0.03685	0.244496216	2.730076	1.09E-05	hypermethylated
cg04245645	0.0344	0.228228649	2.729999	6.39E-06	hypermethylated
cg09123431	0.03371	0.223575135	2.729511	5.65E-06	hypermethylated
cg02400740	0.03712	0.246082162	2.728871	1.54E-06	hypermethylated
cg08322102	0.05125	0.339701081	2.728642	1.04E-05	hypermethylated
cg14507337	0.04541	0.300924324	2.728319	1.62E-05	hypermethylated
cg26551913	0.04553	0.301538378	2.727452	1.18E-05	hypermethylated
cg12505170	0.05462	0.361622162	2.726982	3.85E-06	hypermethylated
cg16521917	0.04723	0.312651351	2.726779	6.75E-06	hypermethylated
cg09728607	0.02551	0.168815676	2.726314	2.53E-06	hypermethylated
cg10448808	0.04587	0.303484865	2.726002	2.83E-06	hypermethylated
cg10168149	0.05383	0.356108108	2.725833	1.63E-06	hypermethylated
cg24109980	0.04393	0.290604324	2.725778	4.36E-06	hypermethylated
cg14470895	0.04479	0.295963243	2.724169	1.23E-05	hypermethylated
cg02478448	0.06898	0.455555676	2.723377	1.72E-06	hypermethylated
cg17087479	0.0488	0.32215027	2.722781	4.94E-05	hypermethylated
cg07821427	0.04852	0.320277297	2.72267	1.73E-05	hypermethylated
cg23809442	0.0445	0.293351892	2.720755	4.73E-06	hypermethylated
cg22661893	0.04517	0.297631351	2.72009	8.83E-06	hypermethylated
cg24961583	0.0343	0.22593027	2.719597	4.24E-06	hypermethylated
cg14055896	0.04699	0.309365946	2.718889	3.17E-06	hypermethylated
cg21995919	0.04239	0.279031892	2.718634	2.32E-05	hypermethylated
cg05694245	0.03735	0.245728649	2.717886	7.65E-05	hypermethylated
cg21463349	0.03274	0.215395135	2.717859	2.46E-06	hypermethylated
cg22753340	0.04694	0.308336216	2.715615	5.58E-06	hypermethylated
cg21479226	0.05483	0.360043784	2.715135	6.93E-06	hypermethylated
cg18888464	0.04114	0.270070811	2.714724	2.23E-06	hypermethylated
cg08346159	0.04316	0.28330973	2.714613	5.65E-05	hypermethylated
cg05984554	0.03304	0.216865405	2.714514	3.35E-06	hypermethylated
cg26654934	0.04861	0.318780541	2.713239	4.93E-06	hypermethylated
cg25886284	0.03946	0.258687027	2.712745	5.25E-05	hypermethylated
cg04600170	0.03308	0.216791892	2.71228	3.35E-06	hypermethylated
cg12781700	0.05601	0.367064324	2.712277	5.81E-06	hypermethylated
cg13950603	0.0454	0.297402162	2.711651	6.75E-06	hypermethylated
cg03145999	0.04113	0.269424865	2.71162	9.31E-06	hypermethylated
cg21908235	0.05959	0.390347027	2.711615	3.69E-06	hypermethylated
cg01350077	0.04672	0.305884324	2.710874	9.19E-06	hypermethylated
cg27510182	0.04606	0.3015	2.710572	1.01E-05	hypermethylated
cg14073722	0.05518	0.361172432	2.71047	5.35E-06	hypermethylated
cg13962186	0.04086	0.267425405	2.710375	3.96E-06	hypermethylated
cg10109500	0.03222	0.210659459	2.708884	2.56E-06	hypermethylated
cg03306374	0.07634	0.499109189	2.708844	3.12E-06	hypermethylated
cg19715410	0.04752	0.310623243	2.708559	0.000163	hypermethylated
cg23248357	0.03411	0.222912973	2.708214	7.20E-07	hypermethylated
cg12523924	0.04353	0.284373514	2.707705	1.01E-05	hypermethylated
cg25267072	0.04107	0.268298919	2.707684	3.64E-06	hypermethylated
cg23663774	0.04903	0.320204324	2.707256	5.43E-06	hypermethylated
cg04612444	0.04844	0.3163	2.707023	1.99E-06	hypermethylated
cg17373442	0.04587	0.299518378	2.707022	3.76E-05	hypermethylated
cg05783915	0.05105	0.333256757	2.706651	1.09E-05	hypermethylated
cg15607538	0.05191	0.338758378	2.706172	7.93E-06	hypermethylated
cg18331515	0.04804	0.313483784	2.706083	5.58E-05	hypermethylated
cg16405026	0.05213	0.340144865	2.705964	2.87E-06	hypermethylated
cg00043819	0.0281	0.183222162	2.704952	2.64E-05	hypermethylated
cg02264082	0.04158	0.271108649	2.704909	6.05E-06	hypermethylated

cg04342955	0.05137	0.334927027	2.704849	1.21E-05	hypermethylated
cg15042811	0.03958	0.258041081	2.704757	0.000103	hypermethylated
cg19937061	0.05787	0.377090811	2.704024	3.69E-06	hypermethylated
cg24198558	0.04192	0.273107568	2.703759	2.15E-05	hypermethylated
cg00986824	0.04577	0.298165405	2.703639	2.56E-06	hypermethylated
cg18780412	0.03553	0.23144973	2.703589	1.93E-06	hypermethylated
cg24610236	0.04783	0.311448108	2.703004	1.62E-05	hypermethylated
cg24236409	0.03278	0.213338919	2.702259	4.93E-06	hypermethylated
cg25683325	0.0648	0.421683784	2.702096	1.62E-05	hypermethylated
cg12353207	0.03395	0.220523784	2.699451	2.64E-05	hypermethylated
cg22541735	0.06081	0.394831351	2.698856	2.42E-06	hypermethylated
cg17741689	0.03975	0.258040541	2.698571	2.56E-06	hypermethylated
cg19944763	0.04028	0.261438378	2.698335	9.31E-06	hypermethylated
cg13879483	0.06227	0.404158378	2.698312	2.05E-06	hypermethylated
cg04591032	0.04105	0.266160541	2.696843	6.57E-06	hypermethylated
cg21635854	0.04341	0.281408108	2.696565	1.21E-05	hypermethylated
cg20567847	0.04576	0.296579459	2.69626	3.44E-05	hypermethylated
cg16707405	0.04305	0.278827568	2.695288	5.07E-06	hypermethylated
cg01436128	0.03736	0.241935676	2.695057	3.49E-06	hypermethylated
cg20148575	0.07295	0.471713514	2.692931	4.86E-06	hypermethylated
cg10808783	0.03746	0.242177838	2.692644	1.25E-05	hypermethylated
cg23302682	0.04543	0.293686486	2.69256	6.66E-06	hypermethylated
cg00516513	0.04783	0.309076216	2.691975	1.28E-05	hypermethylated
cg11437784	0.03999	0.258410811	2.691955	1.88E-06	hypermethylated
cg22769941	0.04021	0.259782162	2.691676	8.95E-06	hypermethylated
cg22916722	0.04536	0.292713514	2.689997	5.14E-06	hypermethylated
cg25463470	0.03134	0.202225946	2.689891	2.16E-06	hypermethylated
cg08149193	0.0556	0.358467568	2.688686	5.00E-06	hypermethylated
cg14045872	0.05672	0.365636757	2.688482	4.86E-06	hypermethylated
cg18529845	0.04966	0.320074054	2.68825	1.11E-05	hypermethylated
cg27464184	0.04932	0.317815676	2.687946	2.42E-05	hypermethylated
cg19694010	0.04368	0.281464865	2.68791	1.87E-05	hypermethylated
cg16580499	0.04656	0.299814054	2.686905	3.85E-06	hypermethylated
cg18777119	0.04604	0.296317838	2.686186	3.85E-06	hypermethylated
cg00690148	0.0452	0.290732432	2.685297	1.75E-05	hypermethylated
cg06083330	0.04879	0.313413514	2.68341	1.72E-06	hypermethylated
cg24531255	0.04524	0.290604865	2.683388	4.42E-05	hypermethylated
cg10741153	0.03581	0.229950811	2.682891	3.04E-06	hypermethylated
cg20973720	0.0506	0.324543784	2.681204	5.31E-05	hypermethylated
cg21884231	0.06487	0.415822162	2.680343	3.49E-06	hypermethylated
cg03483150	0.04536	0.290400541	2.678552	4.60E-06	hypermethylated
cg26615813	0.03972	0.254215135	2.678112	1.84E-05	hypermethylated
cg27039662	0.03269	0.209212432	2.678047	3.74E-06	hypermethylated
cg01447112	0.05865	0.375247027	2.677638	4.54E-06	hypermethylated
cg13671919	0.02822	0.180424865	2.676608	3.59E-06	hypermethylated
cg17422774	0.04238	0.270861081	2.676098	6.93E-06	hypermethylated
cg06611922	0.03882	0.248095135	2.676021	3.69E-06	hypermethylated
cg02809746	0.04671	0.298464865	2.675758	3.17E-06	hypermethylated
cg01246835	0.03156	0.201612432	2.675415	3.44E-06	hypermethylated
cg03044249	0.06555	0.418604865	2.674921	4.36E-06	hypermethylated
cg26381364	0.05786	0.369411351	2.67459	1.84E-05	hypermethylated
cg25019648	0.04027	0.256954054	2.673733	0.000102	hypermethylated
cg24657817	0.05666	0.361482703	2.673524	1.31E-05	hypermethylated
cg06572465	0.04977	0.317470811	2.673276	2.10E-06	hypermethylated
cg02728595	0.04177	0.265928649	2.6705	0.000445	hypermethylated
cg24202123	0.04909	0.31243027	2.670033	3.44E-05	hypermethylated
cg00153110	0.03793	0.241327568	2.669581	9.44E-06	hypermethylated
cg07519816	0.0458	0.291250811	2.668843	1.15E-05	hypermethylated

cg11074814	0.05218	0.331626486	2.66799	5.89E-06	hypermethylated
cg07072722	0.03181	0.202082162	2.66739	4.80E-06	hypermethylated
cg14700304	0.04215	0.267699459	2.66701	7.12E-05	hypermethylated
cg23528400	0.04773	0.302816757	2.665477	1.99E-05	hypermethylated
cg23906738	0.07437	0.471822162	2.665451	1.99E-06	hypermethylated
cg16519587	0.04893	0.310390811	2.665295	1.68E-05	hypermethylated
cg03149560	0.04377	0.277548108	2.664724	4.36E-05	hypermethylated
cg23934404	0.04982	0.315851351	2.664449	6.05E-06	hypermethylated
cg25923609	0.07312	0.463496216	2.66422	6.78E-07	hypermethylated
cg15015920	0.03254	0.206208649	2.663819	2.10E-06	hypermethylated
cg12356890	0.05609	0.355191351	2.662781	1.99E-05	hypermethylated
cg10437806	0.04013	0.253939459	2.661732	5.43E-06	hypermethylated
cg16712637	0.02864	0.181224324	2.661673	8.60E-06	hypermethylated
cg13495205	0.07351	0.465096757	2.661518	1.09E-06	hypermethylated
cg25090499	0.03824	0.241719459	2.660179	5.81E-06	hypermethylated
cg25191628	0.05621	0.355306486	2.660165	2.29E-06	hypermethylated
cg14271531	0.03629	0.229347568	2.659892	4.48E-06	hypermethylated
cg08911275	0.05575	0.352255135	2.659577	8.32E-05	hypermethylated
cg13464448	0.03683	0.232708108	2.659568	3.85E-05	hypermethylated
cg27553667	0.04484	0.283145946	2.658688	3.17E-06	hypermethylated
cg13557668	0.04096	0.258597838	2.658423	2.15E-05	hypermethylated
cg00926400	0.04418	0.278910811	2.658339	5.73E-06	hypermethylated
cg09493505	0.0657	0.414587027	2.65771	8.83E-06	hypermethylated
cg11414560	0.05216	0.329008108	2.657107	7.93E-06	hypermethylated
cg08204280	0.04808	0.303263784	2.657064	7.22E-06	hypermethylated
cg23766591	0.04331	0.273042703	2.656355	1.54E-05	hypermethylated
cg23156916	0.04	0.25202973	2.655522	7.93E-06	hypermethylated
cg26142412	0.05209	0.327931351	2.654315	1.20E-05	hypermethylated
cg26565021	0.05521	0.347475135	2.653908	3.32E-05	hypermethylated
cg00290506	0.03997	0.251428649	2.65316	1.09E-05	hypermethylated
cg12012932	0.04357	0.274053514	2.653051	1.01E-05	hypermethylated
cg11398452	0.05172	0.325234595	2.652687	9.05E-05	hypermethylated
cg16007456	0.05226	0.328605946	2.65258	8.71E-06	hypermethylated
cg26259537	0.0472	0.296772432	2.652498	4.26E-05	hypermethylated
cg03502002	0.07546	0.474445946	2.65246	3.96E-06	hypermethylated
cg15316843	0.06709	0.421524865	2.651448	2.57E-06	hypermethylated
cg19725343	0.04908	0.308320541	2.651224	2.74E-05	hypermethylated
cg20600210	0.03731	0.234247027	2.650396	3.39E-06	hypermethylated
cg01464835	0.06438	0.40412	2.650099	5.65E-06	hypermethylated
cg18375860	0.05877	0.368542162	2.648678	4.12E-06	hypermethylated
cg03131298	0.02497	0.156558919	2.648438	1.05E-06	hypermethylated
cg23858040	0.05261	0.329845946	2.648383	1.73E-05	hypermethylated
cg04945331	0.05383	0.337434595	2.648126	3.69E-06	hypermethylated
cg16426537	0.05174	0.324179459	2.647441	2.92E-05	hypermethylated
cg16896847	0.04197	0.262748108	2.64625	1.17E-05	hypermethylated
cg17302155	0.05524	0.345810811	2.646198	2.29E-06	hypermethylated
cg02401454	0.05215	0.326456216	2.64615	2.79E-06	hypermethylated
cg22702772	0.05678	0.355392973	2.64596	6.57E-06	hypermethylated
cg25181651	0.06124	0.382631892	2.643411	5.81E-06	hypermethylated
cg13314145	0.06201	0.387282703	2.642814	4.42E-06	hypermethylated
cg14252519	0.05623	0.351147568	2.642665	6.08E-05	hypermethylated
cg01240056	0.0621	0.38774	2.642424	1.08E-05	hypermethylated
cg19126300	0.06086	0.379850811	2.641867	4.01E-06	hypermethylated
cg16190732	0.06416	0.400445405	2.64186	1.31E-05	hypermethylated
cg23495748	0.06117	0.381747027	2.641721	9.31E-06	hypermethylated
cg07147166	0.04331	0.270091351	2.640675	4.12E-06	hypermethylated
cg23989821	0.0604	0.376265946	2.639132	4.01E-06	hypermethylated
cg22158650	0.06075	0.378404865	2.638974	2.79E-06	hypermethylated

cg12060744	0.0612	0.381198919	2.63894	2.91E-06	hypermethylated
cg18716164	0.06526	0.406316216	2.638332	5.21E-06	hypermethylated
cg18092028	0.05661	0.352427568	2.638198	9.44E-06	hypermethylated
cg02574509	0.04946	0.307660541	2.637005	6.93E-06	hypermethylated
cg15427906	0.04117	0.256073514	2.636893	1.89E-05	hypermethylated
cg09009536	0.04061	0.252474595	2.636231	7.42E-06	hypermethylated
cg05040472	0.04057	0.252217838	2.636185	3.04E-06	hypermethylated
cg21393713	0.05847	0.363310811	2.635436	2.42E-05	hypermethylated
cg02002583	0.04568	0.283608649	2.634267	1.97E-05	hypermethylated
cg10297491	0.05233	0.32470973	2.63344	3.62E-05	hypermethylated
cg09527126	0.05345	0.331560541	2.63301	7.93E-06	hypermethylated
cg22333960	0.0445	0.275910811	2.632325	4.00E-05	hypermethylated
cg09979478	0.0447	0.277074054	2.631925	1.46E-05	hypermethylated
cg11338643	0.04499	0.27883027	2.631711	1.73E-05	hypermethylated
cg17203063	0.04132	0.256012973	2.631305	7.52E-06	hypermethylated
cg03637815	0.04779	0.296048108	2.631051	5.73E-06	hypermethylated
cg17712694	0.05247	0.324907568	2.630465	5.28E-06	hypermethylated
cg20302133	0.0769	0.475952432	2.629762	7.72E-06	hypermethylated
cg01815671	0.05546	0.342975676	2.628587	7.72E-06	hypermethylated
cg13438893	0.06726	0.415751892	2.627902	1.88E-06	hypermethylated
cg09371439	0.0201	0.124216757	2.627592	5.21E-06	hypermethylated
cg15132565	0.06846	0.422497297	2.625609	2.64E-06	hypermethylated
cg25185173	0.06073	0.374669189	2.625136	7.12E-06	hypermethylated
cg04023150	0.04989	0.307678919	2.624603	4.05E-05	hypermethylated
cg02318926	0.05562	0.343010811	2.624578	3.69E-06	hypermethylated
cg04138185	0.05721	0.352811351	2.624558	1.18E-05	hypermethylated
cg18402615	0.0334	0.205802703	2.623342	3.76E-05	hypermethylated
cg16304215	0.04267	0.262921081	2.623336	7.83E-06	hypermethylated
cg18617005	0.05884	0.362378919	2.62263	6.75E-06	hypermethylated
cg21282549	0.05305	0.326515135	2.621725	1.33E-05	hypermethylated
cg05513983	0.02657	0.163455135	2.621025	1.20E-05	hypermethylated
cg09010671	0.05642	0.346914054	2.6203	1.44E-05	hypermethylated
cg27555582	0.06044	0.371548108	2.619973	1.15E-06	hypermethylated
cg16334314	0.04603	0.28293027	2.6198	2.04E-05	hypermethylated
cg05821789	0.05607	0.344584865	2.619558	2.10E-06	hypermethylated
cg10770742	0.052	0.319563243	2.619518	3.07E-05	hypermethylated
cg05686497	0.05296	0.325351892	2.619026	2.02E-05	hypermethylated
cg23632985	0.05622	0.345288649	2.618648	3.44E-06	hypermethylated
cg23144681	0.04252	0.260985405	2.617756	5.86E-05	hypermethylated
cg11342468	0.04769	0.292655676	2.617446	8.15E-06	hypermethylated
cg25798792	0.0456	0.27973027	2.616931	3.00E-05	hypermethylated
cg12940822	0.04581	0.280944324	2.61655	7.12E-06	hypermethylated
cg22290744	0.0424	0.260000541	2.616378	1.17E-05	hypermethylated
cg04673825	0.04278	0.262302703	2.616224	5.58E-06	hypermethylated
cg04136610	0.0578	0.354318378	2.615905	8.95E-06	hypermethylated
cg03976877	0.04839	0.296515676	2.615328	3.04E-05	hypermethylated
cg10303487	0.05495	0.336670811	2.615147	8.83E-06	hypermethylated
cg02761480	0.05146	0.315229189	2.614878	3.26E-06	hypermethylated
cg10636403	0.05355	0.32792	2.614385	0.000132	hypermethylated
cg24150172	0.0585	0.358209189	2.614294	1.35E-05	hypermethylated
cg11835068	0.03878	0.237435135	2.614149	9.94E-07	hypermethylated
cg19226007	0.03373	0.20646973	2.613826	1.14E-05	hypermethylated
cg00836482	0.06076	0.371795135	2.613314	2.71E-05	hypermethylated
cg07780095	0.05144	0.314764324	2.613309	4.58E-05	hypermethylated
cg18764577	0.04167	0.254951892	2.613144	4.48E-06	hypermethylated
cg23300659	0.0357	0.218415135	2.613077	8.15E-06	hypermethylated
cg17509967	0.05673	0.346888108	2.612287	4.42E-06	hypermethylated
cg27237300	0.05272	0.322335135	2.612139	8.83E-06	hypermethylated

cg09765089	0.06479	0.395336216	2.609237	1.50E-05	hypermethylated
cg18098400	0.04975	0.303538919	2.609113	1.31E-05	hypermethylated
cg16234557	0.0488	0.297381081	2.60736	1.14E-05	hypermethylated
cg27116912	0.0435	0.265055135	2.607205	3.49E-06	hypermethylated
cg22434409	0.07333	0.446790811	2.607124	2.57E-06	hypermethylated
cg22762492	0.04105	0.250027568	2.606633	2.99E-06	hypermethylated
cg26387689	0.03724	0.22681027	2.606561	5.86E-05	hypermethylated
cg17305436	0.04143	0.252080541	2.605137	2.79E-06	hypermethylated
cg16966815	0.0304	0.184874054	2.6044	1.60E-05	hypermethylated
cg09548051	0.0445	0.270580541	2.604181	1.62E-05	hypermethylated
cg05884032	0.03765	0.228661081	2.602489	3.30E-06	hypermethylated
cg25051341	0.05269	0.320001622	2.602478	1.66E-05	hypermethylated
cg01791874	0.03665	0.222585946	2.602477	7.52E-06	hypermethylated
cg19068510	0.04318	0.262198919	2.602227	6.31E-05	hypermethylated
cg20453394	0.04036	0.245024865	2.60193	9.31E-06	hypermethylated
cg03175305	0.03241	0.196714054	2.601589	4.36E-06	hypermethylated
cg19054524	0.04387	0.266166486	2.601022	1.30E-05	hypermethylated
cg13021333	0.05663	0.343503243	2.600685	1.58E-06	hypermethylated
cg00240432	0.04782	0.290014054	2.600437	4.42E-06	hypermethylated
cg09644707	0.04823	0.292456216	2.600218	6.05E-06	hypermethylated
cg24725789	0.04183	0.253564865	2.599745	5.00E-06	hypermethylated
cg11014373	0.05055	0.306421081	2.599733	4.07E-06	hypermethylated
cg06482019	0.04467	0.270749189	2.599579	3.04E-06	hypermethylated
cg04401986	0.04062	0.24564973	2.596341	4.01E-06	hypermethylated
cg14369938	0.04667	0.282215676	2.596231	9.96E-06	hypermethylated
cg20392607	0.0461	0.278612432	2.595421	1.71E-05	hypermethylated
cg12812583	0.04095	0.247351351	2.594626	3.00E-05	hypermethylated
cg01519253	0.04433	0.267696757	2.594244	6.75E-06	hypermethylated
cg12869912	0.04377	0.264239459	2.593832	1.92E-05	hypermethylated
cg05659097	0.05386	0.325125946	2.593713	1.72E-06	hypermethylated
cg13486532	0.03873	0.233754595	2.593471	5.93E-05	hypermethylated
cg08074851	0.05351	0.322940541	2.593388	7.12E-06	hypermethylated
cg01454487	0.0413	0.249116757	2.592608	1.06E-05	hypermethylated
cg03804213	0.05365	0.32357027	2.592429	1.02E-05	hypermethylated
cg02340083	0.043	0.259313514	2.592289	3.49E-06	hypermethylated
cg26705960	0.04406	0.26561027	2.59177	2.85E-05	hypermethylated
cg23619365	0.07844	0.472813514	2.59161	2.10E-06	hypermethylated
cg03109827	0.06361	0.383378919	2.591446	5.28E-06	hypermethylated
cg18693395	0.0522	0.314593514	2.591367	1.29E-06	hypermethylated
cg05495949	0.05399	0.325313514	2.591067	2.15E-05	hypermethylated
cg20398486	0.05423	0.326734054	2.590954	2.49E-06	hypermethylated
cg11630154	0.03605	0.217167568	2.590737	2.46E-06	hypermethylated
cg27252696	0.06116	0.368422703	2.590702	2.92E-05	hypermethylated
cg17131044	0.03117	0.187751351	2.590593	2.91E-06	hypermethylated
cg16703956	0.06238	0.375677838	2.590341	5.73E-06	hypermethylated
cg00304520	0.03286	0.197867027	2.590127	1.26E-06	hypermethylated
cg10356613	0.0571	0.343824324	2.590109	5.19E-05	hypermethylated
cg24934063	0.03261	0.196277838	2.589511	1.58E-05	hypermethylated
cg03425110	0.04176	0.251280541	2.589105	4.70E-05	hypermethylated
cg05309948	0.0484	0.29109027	2.588388	1.97E-05	hypermethylated
cg07678904	0.03851	0.231513514	2.587791	0.004863	hypermethylated
cg13096208	0.04302	0.258542162	2.58732	2.32E-05	hypermethylated
cg10435849	0.05409	0.325069189	2.587313	1.87E-05	hypermethylated
cg04701034	0.05364	0.322345405	2.587226	0.000551	hypermethylated
cg03646189	0.05103	0.306544324	2.586678	3.45E-07	hypermethylated
cg26328510	0.0504	0.302574054	2.585793	8.94E-05	hypermethylated
cg18944047	0.04285	0.257212973	2.585596	5.00E-06	hypermethylated
cg10512875	0.04839	0.290359459	2.585059	2.27E-05	hypermethylated

cg08464190	0.04696	0.281635135	2.584323	6.01E-05	hypermethylated
cg04688351	0.05835	0.349716757	2.583382	4.88E-07	hypermethylated
cg03092191	0.04268	0.255666486	2.582631	8.60E-06	hypermethylated
cg26309134	0.05568	0.33350973	2.582498	7.83E-06	hypermethylated
cg13211683	0.05332	0.319291351	2.582125	1.42E-05	hypermethylated
cg12746059	0.0412	0.246655676	2.581782	2.85E-05	hypermethylated
cg11071231	0.05754	0.344463243	2.581713	4.01E-06	hypermethylated
cg16642284	0.07847	0.469313514	2.580339	1.94E-05	hypermethylated
cg14696396	0.06408	0.383217838	2.580219	1.39E-05	hypermethylated
cg17284804	0.05063	0.302777838	2.580195	9.57E-06	hypermethylated
cg24472231	0.05589	0.334092973	2.579588	1.52E-05	hypermethylated
cg08526074	0.05505	0.328948108	2.579046	1.05E-05	hypermethylated
cg19098763	0.06259	0.373776757	2.578173	4.73E-06	hypermethylated
cg09671258	0.07242	0.432435135	2.578024	6.22E-06	hypermethylated
cg24872782	0.04701	0.280625946	2.577609	1.71E-05	hypermethylated
cg23244790	0.05345	0.318914054	2.576906	1.49E-06	hypermethylated
cg03264601	0.05279	0.314956757	2.576817	5.58E-06	hypermethylated
cg13755795	0.06309	0.376162703	2.575874	7.93E-06	hypermethylated
cg17168456	0.05104	0.304316757	2.575874	1.01E-05	hypermethylated
cg10273340	0.0481	0.286776216	2.575817	2.74E-05	hypermethylated
cg20673829	0.05415	0.322827568	2.575731	6.93E-06	hypermethylated
cg17300353	0.0506	0.301624324	2.575543	5.89E-06	hypermethylated
cg08015447	0.0419	0.249697297	2.575158	7.62E-06	hypermethylated
cg25397945	0.07869	0.468639459	2.574226	2.79E-06	hypermethylated
cg06554200	0.03405	0.202784324	2.574219	1.63E-06	hypermethylated
cg08430009	0.06015	0.358167568	2.573998	1.46E-05	hypermethylated
cg26195583	0.03729	0.221825946	2.572567	1.89E-05	hypermethylated
cg26091021	0.06417	0.381617838	2.572158	3.79E-06	hypermethylated
cg06546806	0.05465	0.324771351	2.571131	1.52E-05	hypermethylated
cg06682197	0.03416	0.202707568	2.56902	1.06E-05	hypermethylated
cg14015706	0.04119	0.244343243	2.568543	2.38E-05	hypermethylated
cg13407456	0.05415	0.321140541	2.568172	9.07E-06	hypermethylated
cg00046625	0.04481	0.265732973	2.568085	1.82E-05	hypermethylated
cg24393316	0.04041	0.239602162	2.567857	3.64E-06	hypermethylated
cg21844856	0.03618	0.214444324	2.567339	3.57E-05	hypermethylated
cg07033372	0.05608	0.332210811	2.566541	6.39E-06	hypermethylated
cg27360326	0.07056	0.417692973	2.56552	3.12E-06	hypermethylated
cg20443254	0.0737	0.436170811	2.565157	2.36E-06	hypermethylated
cg18627235	0.0559	0.330677297	2.564504	1.71E-05	hypermethylated
cg19717586	0.08932	0.527794595	2.562921	3.04E-06	hypermethylated
cg01461410	0.04034	0.238345405	2.562771	3.49E-05	hypermethylated
cg03224572	0.06273	0.370627027	2.562741	4.01E-06	hypermethylated
cg19852958	0.07053	0.41661027	2.562389	5.14E-06	hypermethylated
cg16640855	0.05782	0.341348108	2.561603	4.54E-06	hypermethylated
cg12483476	0.05795	0.3421	2.561538	3.39E-06	hypermethylated
cg27264049	0.04096	0.241576757	2.560194	7.32E-06	hypermethylated
cg22660147	0.03779	0.22284973	2.559995	2.21E-05	hypermethylated
cg11857997	0.04449	0.262297838	2.559653	8.83E-06	hypermethylated
cg12314713	0.05798	0.341642162	2.558859	2.64E-05	hypermethylated
cg20948997	0.0357	0.210206486	2.557811	1.42E-05	hypermethylated
cg15861196	0.03861	0.227170811	2.556731	3.49E-06	hypermethylated
cg17538572	0.06185	0.363691892	2.555871	2.64E-05	hypermethylated
cg16635314	0.04506	0.264863243	2.555328	6.57E-06	hypermethylated
cg00017437	0.0534	0.313743243	2.554673	3.59E-06	hypermethylated
cg16630482	0.06596	0.387518919	2.554603	4.48E-06	hypermethylated
cg20539752	0.04856	0.285144324	2.553852	5.89E-06	hypermethylated
cg01070209	0.04682	0.274831892	2.553353	1.71E-05	hypermethylated
cg14256587	0.04588	0.269300541	2.55328	7.42E-06	hypermethylated

cg15032142	0.04822	0.283023784	2.55322	9.44E-06	hypermethylated
cg18229521	0.06953	0.407678378	2.551724	4.42E-06	hypermethylated
cg18070676	0.05045	0.295566486	2.550557	1.68E-05	hypermethylated
cg20249252	0.05425	0.317670811	2.549837	4.30E-06	hypermethylated
cg21667878	0.05202	0.304580541	2.549685	0.000148	hypermethylated
cg15822765	0.07458	0.436472432	2.54903	3.15E-05	hypermethylated
cg16479539	0.03596	0.21044	2.548944	3.85E-05	hypermethylated
cg19861117	0.03796	0.222137838	2.548903	1.12E-05	hypermethylated
cg25189564	0.07628	0.44635027	2.5488	6.13E-06	hypermethylated
cg18787401	0.05225	0.305587568	2.548083	3.00E-05	hypermethylated
cg24369185	0.0562	0.328542162	2.547436	2.95E-06	hypermethylated
cg14872657	0.04783	0.279586486	2.547307	6.22E-06	hypermethylated
cg19864758	0.05638	0.32942	2.546673	1.68E-05	hypermethylated
cg22531183	0.05373	0.313904865	2.546528	0.000681	hypermethylated
cg14657517	0.05109	0.298427568	2.546268	1.08E-05	hypermethylated
cg24812837	0.05156	0.301165405	2.546232	3.00E-05	hypermethylated
cg26198463	0.02873	0.167752973	2.545708	4.93E-06	hypermethylated
cg16405019	0.04083	0.238359459	2.545437	2.16E-06	hypermethylated
cg07374509	0.04634	0.270408649	2.544811	3.07E-05	hypermethylated
cg03506640	0.04792	0.279564865	2.544483	2.21E-05	hypermethylated
cg08964780	0.04658	0.271707568	2.544272	0.000232	hypermethylated
cg06543087	0.0392	0.228634595	2.544118	9.19E-06	hypermethylated
cg20174066	0.04649	0.271047568	2.543554	1.18E-05	hypermethylated
cg06310816	0.06951	0.405135676	2.543113	1.94E-05	hypermethylated
cg04077662	0.04259	0.248174054	2.542766	1.44E-05	hypermethylated
cg08492619	0.05237	0.305118919	2.542559	5.06E-05	hypermethylated
cg02978421	0.04385	0.255474054	2.542528	0.000159	hypermethylated
cg02860732	0.05281	0.307653514	2.542423	3.90E-06	hypermethylated
cg27533288	0.05648	0.328917838	2.541915	0.0002	hypermethylated
cg08079580	0.033	0.192119459	2.541466	4.24E-06	hypermethylated
cg01777643	0.061	0.354972973	2.540828	6.75E-06	hypermethylated
cg04198308	0.0395	0.229793514	2.540414	7.74E-05	hypermethylated
cg24427504	0.02918	0.169688649	2.539838	3.85E-06	hypermethylated
cg23065934	0.04181	0.243129189	2.539803	7.52E-06	hypermethylated
cg15415452	0.06852	0.398091351	2.538502	2.91E-06	hypermethylated
cg04159903	0.03423	0.198835135	2.53824	8.37E-06	hypermethylated
cg26990102	0.04602	0.267096757	2.53703	2.95E-06	hypermethylated
cg24903887	0.05166	0.299823243	2.536993	1.21E-05	hypermethylated
cg02305377	0.05476	0.317783784	2.536851	3.12E-06	hypermethylated
cg27504802	0.04891	0.283731351	2.536324	4.05E-05	hypermethylated
cg05134015	0.04216	0.244416757	2.535396	4.36E-06	hypermethylated
cg02026062	0.03855	0.223477297	2.535326	1.52E-05	hypermethylated
cg20312205	0.06806	0.394148108	2.533859	6.93E-06	hypermethylated
cg00853103	0.04037	0.233697297	2.533286	3.96E-06	hypermethylated
cg12860686	0.05311	0.307418378	2.533148	2.71E-06	hypermethylated
cg24595510	0.06425	0.371844865	2.532932	1.08E-05	hypermethylated
cg21114773	0.05195	0.300604865	2.532673	1.62E-05	hypermethylated
cg25950235	0.05662	0.327527027	2.53223	3.64E-06	hypermethylated
cg18759960	0.04617	0.266867568	2.531096	3.44E-05	hypermethylated
cg27515369	0.06513	0.376348649	2.530676	1.04E-05	hypermethylated
cg25756435	0.04066	0.234879459	2.530238	1.05E-05	hypermethylated
cg27618240	0.07161	0.413588108	2.529962	8.60E-06	hypermethylated
cg05255522	0.04735	0.273413514	2.529648	2.10E-06	hypermethylated
cg11373604	0.02756	0.159111351	2.529389	1.12E-05	hypermethylated
cg09193347	0.04367	0.251974054	2.528561	2.51E-05	hypermethylated
cg02253760	0.07258	0.418778378	2.528543	4.73E-06	hypermethylated
cg23954465	0.04626	0.266715135	2.527463	8.83E-06	hypermethylated
cg08712932	0.04587	0.264434054	2.527285	1.62E-05	hypermethylated

cg13796823	0.05904	0.340300541	2.527045	9.31E-06	hypermethylated
cg22491927	0.07144	0.411751892	2.526971	7.32E-06	hypermethylated
cg05494604	0.0601	0.346323784	2.526685	4.30E-06	hypermethylated
cg20293594	0.05081	0.292751351	2.526491	2.36E-06	hypermethylated
cg19861741	0.04671	0.269002703	2.525817	3.85E-06	hypermethylated
cg05783139	0.04541	0.261274595	2.524485	5.06E-05	hypermethylated
cg14209299	0.04043	0.232313514	2.522575	5.28E-06	hypermethylated
cg17911318	0.05309	0.305044865	2.522509	1.80E-05	hypermethylated
cg18326021	0.05554	0.319045946	2.522165	3.54E-06	hypermethylated
cg06304097	0.08814	0.505990811	2.521242	6.39E-06	hypermethylated
cg09426834	0.06319	0.362618919	2.520686	1.46E-05	hypermethylated
cg21385821	0.0532	0.305284865	2.520658	6.02E-07	hypermethylated
cg24675150	0.054	0.309786486	2.520243	3.44E-05	hypermethylated
cg01532168	0.05723	0.327910811	2.51846	8.13E-05	hypermethylated
cg08279075	0.04368	0.249801622	2.515738	6.54E-05	hypermethylated
cg12904880	0.08514	0.486887027	2.515678	6.99E-07	hypermethylated
cg12143789	0.05224	0.298735135	2.51564	1.25E-05	hypermethylated
cg04842426	0.05135	0.293540541	2.515124	6.39E-06	hypermethylated
cg10386483	0.0566	0.323531892	2.515034	2.02E-05	hypermethylated
cg26673012	0.06031	0.344737297	2.515028	5.43E-06	hypermethylated
cg24511738	0.0436	0.249089189	2.514262	7.12E-06	hypermethylated
cg09642925	0.04028	0.230115676	2.514224	1.33E-06	hypermethylated
cg13078140	0.05476	0.312604865	2.513146	5.58E-06	hypermethylated
cg06159486	0.04775	0.272584865	2.513133	1.12E-05	hypermethylated
cg18639233	0.04795	0.273715135	2.513072	6.22E-06	hypermethylated
cg26612735	0.03801	0.216958919	2.512971	1.39E-05	hypermethylated
cg07167946	0.03908	0.222907568	2.511943	2.36E-06	hypermethylated
cg24425021	0.04531	0.258355676	2.511457	1.26E-05	hypermethylated
cg06750832	0.05466	0.311645405	2.511348	7.52E-06	hypermethylated
cg23076299	0.04254	0.242534054	2.511295	0.000143	hypermethylated
cg12513880	0.05042	0.287381622	2.5109	1.42E-05	hypermethylated
cg21629500	0.07331	0.417806486	2.510753	1.37E-06	hypermethylated
cg13239420	0.05806	0.330784865	2.510277	2.45E-05	hypermethylated
cg19712603	0.06818	0.38791027	2.508302	6.57E-06	hypermethylated
cg05994094	0.04116	0.234147027	2.5081	1.92E-05	hypermethylated
cg17787134	0.05771	0.328254595	2.507922	1.94E-05	hypermethylated
cg16357388	0.05204	0.295989189	2.507852	5.97E-06	hypermethylated
cg25092838	0.06963	0.39603027	2.50783	2.68E-06	hypermethylated
cg12882697	0.03874	0.220244865	2.507213	1.02E-05	hypermethylated
cg19384531	0.03852	0.21883027	2.506133	1.37E-06	hypermethylated
cg00295794	0.07187	0.408108108	2.50549	2.05E-06	hypermethylated
cg04184232	0.05352	0.303870811	2.505308	2.91E-06	hypermethylated
cg06183338	0.08216	0.466435135	2.505168	1.72E-06	hypermethylated
cg05375728	0.05178	0.293947027	2.505089	2.45E-05	hypermethylated
cg02886408	0.05126	0.290823784	2.50424	3.17E-06	hypermethylated
cg08065231	0.07257	0.411646486	2.503961	4.60E-06	hypermethylated
cg07915921	0.09079	0.514837838	2.503513	2.91E-06	hypermethylated
cg15790820	0.06143	0.348199459	2.502899	1.84E-05	hypermethylated
cg12768681	0.06602	0.374037838	2.502209	1.06E-05	hypermethylated
cg24896649	0.05241	0.296695676	2.50107	4.60E-06	hypermethylated
cg09053680	0.07326	0.414676216	2.500888	2.15E-05	hypermethylated
cg21026830	0.0589	0.33335027	2.500699	5.51E-07	hypermethylated
cg27298506	0.04573	0.258805405	2.500655	5.35E-06	hypermethylated
cg11389172	0.0585	0.331039459	2.500495	1.68E-05	hypermethylated
cg26715952	0.05229	0.295811892	2.500073	3.40E-05	hypermethylated
cg13823136	0.05451	0.308306486	2.499772	1.28E-05	hypermethylated
cg22989103	0.03988	0.225441081	2.499013	3.23E-05	hypermethylated
cg20764887	0.06556	0.370592973	2.498948	2.49E-06	hypermethylated

cg07013734	0.04369	0.246950811	2.498849	3.26E-06	hypermethylated
cg03943218	0.07477	0.422405405	2.498097	2.57E-06	hypermethylated
cg06424097	0.03139	0.177324865	2.498018	5.86E-05	hypermethylated
cg02230017	0.05416	0.305944865	2.497972	1.82E-05	hypermethylated
cg14353137	0.05181	0.292637838	2.497814	1.44E-05	hypermethylated
cg01307130	0.06431	0.362943243	2.496629	6.66E-06	hypermethylated
cg24946597	0.05332	0.300843784	2.496266	2.04E-05	hypermethylated
cg02874376	0.05849	0.329768649	2.495192	5.73E-06	hypermethylated
cg03994318	0.06071	0.342221081	2.494923	3.04E-05	hypermethylated
cg08301572	0.05031	0.283488649	2.494374	1.70E-06	hypermethylated
cg10237419	0.03905	0.219994595	2.494074	6.95E-05	hypermethylated
cg00179217	0.05326	0.300033514	2.493999	6.22E-06	hypermethylated
cg03146949	0.06626	0.372993514	2.49294	2.15E-05	hypermethylated
cg09515947	0.03771	0.212263243	2.492836	7.72E-06	hypermethylated
cg14932016	0.05856	0.329518378	2.492371	2.45E-05	hypermethylated
cg00097146	0.06745	0.379474595	2.492113	8.34E-07	hypermethylated
cg02842227	0.04203	0.236396757	2.491719	7.42E-06	hypermethylated
cg18249173	0.06715	0.377671351	2.491672	1.54E-05	hypermethylated
cg11452391	0.0423	0.237897297	2.491609	3.64E-06	hypermethylated
cg05452406	0.06067	0.341167027	2.491423	2.23E-06	hypermethylated
cg02671880	0.04313	0.242494595	2.491189	3.59E-06	hypermethylated
cg02866628	0.04582	0.257598919	2.491077	1.31E-05	hypermethylated
cg18035399	0.05315	0.298783784	2.49096	4.76E-05	hypermethylated
cg06873316	0.04888	0.274753514	2.490822	1.26E-05	hypermethylated
cg10224098	0.07187	0.403865946	2.490415	5.73E-06	hypermethylated
cg08347047	0.05239	0.294298919	2.489919	3.96E-06	hypermethylated
cg20100910	0.05073	0.284956216	2.489829	7.47E-05	hypermethylated
cg25970832	0.03474	0.195090811	2.489476	4.36E-05	hypermethylated
cg13909534	0.0402	0.225702703	2.489156	3.90E-05	hypermethylated
cg12456799	0.04866	0.273166486	2.488972	8.15E-06	hypermethylated
cg14189141	0.06831	0.383207027	2.487955	1.09E-06	hypermethylated
cg01590753	0.04232	0.237215135	2.486785	3.23E-05	hypermethylated
cg08501426	0.04288	0.240346486	2.486739	1.52E-05	hypermethylated
cg02943578	0.0512	0.286775135	2.485704	7.12E-06	hypermethylated
cg22455914	0.06625	0.370920541	2.485118	5.45E-05	hypermethylated
cg19595835	0.03141	0.175814595	2.484759	9.57E-06	hypermethylated
cg24637364	0.06571	0.36764973	2.484147	1.01E-05	hypermethylated
cg10662314	0.05596	0.312942162	2.483428	8.42E-05	hypermethylated
cg01972751	0.06874	0.384395135	2.483368	2.71E-06	hypermethylated
cg12453014	0.06343	0.354583243	2.482887	5.58E-06	hypermethylated
cg24847685	0.06574	0.367417838	2.482578	4.60E-06	hypermethylated
cg03659519	0.05108	0.285367027	2.481988	1.84E-05	hypermethylated
cg01405040	0.07106	0.396876757	2.481581	1.33E-06	hypermethylated
cg19019537	0.06226	0.347572432	2.480936	9.96E-06	hypermethylated
cg18897632	0.08249	0.460482162	2.480854	4.73E-06	hypermethylated
cg20172627	0.05839	0.325664865	2.479595	7.75E-05	hypermethylated
cg14627348	0.04889	0.272468108	2.478476	2.81E-05	hypermethylated
cg14031402	0.03833	0.213578378	2.47822	2.27E-05	hypermethylated
cg08594218	0.06073	0.338191351	2.477358	8.26E-06	hypermethylated
cg13829550	0.06285	0.349884865	2.476896	5.58E-06	hypermethylated
cg20377305	0.07372	0.41033027	2.476658	1.93E-06	hypermethylated
cg24284973	0.04905	0.272936216	2.476239	3.49E-06	hypermethylated
cg01452847	0.08256	0.459249189	2.475762	1.88E-06	hypermethylated
cg01645753	0.06067	0.337403243	2.475419	1.26E-05	hypermethylated
cg05311410	0.0593	0.329698919	2.475045	5.32E-05	hypermethylated
cg27254482	0.06777	0.376788108	2.475035	4.48E-06	hypermethylated
cg04370314	0.05048	0.280585946	2.474659	1.25E-05	hypermethylated
cg08620044	0.04022	0.223514595	2.474384	7.22E-06	hypermethylated

cg17307442	0.05634	0.312992973	2.473899	7.29E-05	hypermethylated
cg23989963	0.05296	0.294172973	2.47369	1.33E-06	hypermethylated
cg15611413	0.04844	0.268964324	2.473144	4.10E-05	hypermethylated
cg19304150	0.05551	0.308212973	2.473108	1.01E-05	hypermethylated
cg17045801	0.05282	0.293274595	2.473096	9.31E-06	hypermethylated
cg16897114	0.04786	0.265646486	2.472615	2.35E-05	hypermethylated
cg14916150	0.04102	0.227651351	2.472427	5.14E-06	hypermethylated
cg14763548	0.05985	0.331931351	2.471462	2.71E-05	hypermethylated
cg02250846	0.05804	0.321867027	2.471345	9.38E-07	hypermethylated
cg17390350	0.07195	0.398904865	2.470978	1.04E-05	hypermethylated
cg23244289	0.05199	0.288062703	2.470077	1.18E-05	hypermethylated
cg10325478	0.04243	0.234991351	2.469451	1.20E-05	hypermethylated
cg14248715	0.05096	0.282099459	2.468767	1.46E-05	hypermethylated
cg18770350	0.07533	0.416893514	2.468382	1.99E-05	hypermethylated
cg21306240	0.07103	0.392977297	2.467946	5.28E-06	hypermethylated
cg21529323	0.03599	0.199063243	2.467559	1.72E-06	hypermethylated
cg27094244	0.06303	0.348474054	2.466941	1.58E-05	hypermethylated
cg02344833	0.07374	0.407649189	2.466809	4.48E-06	hypermethylated
cg14058647	0.03634	0.200855135	2.466525	5.14E-06	hypermethylated
cg19952303	0.04642	0.256497838	2.466128	1.21E-05	hypermethylated
cg06610849	0.07647	0.422504324	2.466	4.07E-06	hypermethylated
cg16767801	0.05155	0.284574595	2.464763	3.71E-05	hypermethylated
cg18249580	0.05339	0.29470973	2.464653	7.52E-06	hypermethylated
cg05221057	0.06082	0.335687568	2.464501	2.87E-06	hypermethylated
cg08095196	0.04718	0.260335135	2.464123	1.58E-05	hypermethylated
cg25732462	0.04976	0.274458378	2.463529	6.70E-05	hypermethylated
cg16332256	0.04867	0.268416757	2.46337	2.71E-05	hypermethylated
cg01878345	0.07839	0.431948649	2.462118	2.05E-06	hypermethylated
cg13405887	0.06216	0.342474054	2.461936	0.000113	hypermethylated
cg12825070	0.07303	0.402249189	2.461528	3.07E-05	hypermethylated
cg25024074	0.06261	0.344854054	2.461521	1.64E-05	hypermethylated
cg25633678	0.06495	0.357704865	2.461368	8.71E-06	hypermethylated
cg26210445	0.07005	0.385712973	2.461071	5.58E-06	hypermethylated
cg10731700	0.05564	0.30631027	2.460799	3.32E-05	hypermethylated
cg08146483	0.06725	0.370218378	2.46077	3.49E-06	hypermethylated
cg22821908	0.04215	0.231867568	2.459697	6.39E-06	hypermethylated
cg13596833	0.05441	0.299248108	2.459398	1.42E-05	hypermethylated
cg06248560	0.05303	0.291647568	2.459345	1.09E-05	hypermethylated
cg26993198	0.04552	0.250247027	2.45878	1.09E-05	hypermethylated
cg20800509	0.06404	0.352032432	2.458663	9.57E-06	hypermethylated
cg10795666	0.05536	0.304213514	2.458168	0.000198	hypermethylated
cg24792289	0.05696	0.312588649	2.456244	5.14E-06	hypermethylated
cg05196969	0.04939	0.270987027	2.455933	6.75E-06	hypermethylated
cg24387472	0.04919	0.269810811	2.455511	2.71E-05	hypermethylated
cg23881278	0.07011	0.384182703	2.4541	6.75E-06	hypermethylated
cg10755235	0.05567	0.305024865	2.453955	2.05E-06	hypermethylated
cg12075445	0.05543	0.303627568	2.453564	6.70E-05	hypermethylated
cg26049726	0.04925	0.269736757	2.453356	2.32E-06	hypermethylated
cg03230154	0.06242	0.341825405	2.453179	1.93E-06	hypermethylated
cg04764012	0.03591	0.196581081	2.452667	3.69E-06	hypermethylated
cg08236767	0.05801	0.317447568	2.452145	6.38E-05	hypermethylated
cg23170850	0.03749	0.20507027	2.451541	2.95E-06	hypermethylated
cg13046832	0.05786	0.316460541	2.451387	5.73E-06	hypermethylated
cg16409261	0.05712	0.312407568	2.451362	5.81E-06	hypermethylated
cg18172877	0.05936	0.324553514	2.450893	5.28E-06	hypermethylated
cg14662355	0.04498	0.245900541	2.450719	4.24E-06	hypermethylated
cg20008332	0.03695	0.201987568	2.45062	7.12E-06	hypermethylated
cg17397592	0.06091	0.332890811	2.450298	3.90E-06	hypermethylated

cg11337945	0.05249	0.286801081	2.449936	1.88E-06	hypermethylated
cg02457680	0.0705	0.385193514	2.449888	2.64E-06	hypermethylated
cg26818735	0.07026	0.383570811	2.448717	4.86E-06	hypermethylated
cg03813164	0.05556	0.303272973	2.448498	4.64E-05	hypermethylated
cg04534765	0.07417	0.404738378	2.448082	3.35E-06	hypermethylated
cg25400229	0.04564	0.249051351	2.448073	4.94E-05	hypermethylated
cg21646598	0.05052	0.275667027	2.448	4.18E-06	hypermethylated
cg18213472	0.04865	0.26539027	2.447604	2.21E-05	hypermethylated
cg18863333	0.02603	0.141828108	2.445896	2.95E-06	hypermethylated
cg16729415	0.0919	0.500727027	2.445888	6.57E-06	hypermethylated
cg14267095	0.05886	0.320475676	2.444855	1.23E-05	hypermethylated
cg10784386	0.0524	0.285269189	2.444685	6.30E-06	hypermethylated
cg15745900	0.05034	0.274047027	2.444646	6.86E-05	hypermethylated
cg14409023	0.05095	0.277352432	2.444566	2.81E-05	hypermethylated
cg23771603	0.05493	0.298954054	2.444258	5.86E-05	hypermethylated
cg03024760	0.06248	0.340005405	2.444091	4.30E-06	hypermethylated
cg12248614	0.0583	0.317138919	2.443547	0.000189	hypermethylated
cg04035209	0.04422	0.240508108	2.443315	6.66E-06	hypermethylated
cg24221648	0.09653	0.524672973	2.442369	2.95E-06	hypermethylated
cg23813156	0.04529	0.246081081	2.441869	1.02E-06	hypermethylated
cg06831576	0.07112	0.386011351	2.440316	6.39E-06	hypermethylated
cg21039708	0.09206	0.499528108	2.43992	4.12E-06	hypermethylated
cg16848624	0.04257	0.230976757	2.439839	8.22E-05	hypermethylated
cg21264189	0.05976	0.324236216	2.439793	2.42E-05	hypermethylated
cg13696942	0.05411	0.293567568	2.439725	3.49E-06	hypermethylated
cg00582971	0.08886	0.482008649	2.439453	4.12E-06	hypermethylated
cg14625175	0.06708	0.363836757	2.439337	6.01E-05	hypermethylated
cg16896079	0.04874	0.264284324	2.438913	6.95E-05	hypermethylated
cg10439765	0.06301	0.341646486	2.438852	1.63E-06	hypermethylated
cg01783070	0.05988	0.324620541	2.438608	1.50E-05	hypermethylated
cg19186145	0.062	0.335954595	2.437926	0.00049	hypermethylated
cg03524083	0.04973	0.269465946	2.437915	4.18E-06	hypermethylated
cg14502484	0.07319	0.396572973	2.437868	7.52E-06	hypermethylated
cg23891360	0.0591	0.319883784	2.436318	6.30E-06	hypermethylated
cg10739556	0.06571	0.355656757	2.436301	5.89E-06	hypermethylated
cg17039236	0.0586	0.317121081	2.436061	5.43E-06	hypermethylated
cg20927661	0.07805	0.42234	2.435934	3.79E-06	hypermethylated
cg16827475	0.08101	0.438228649	2.435512	1.37E-06	hypermethylated
cg25975712	0.05364	0.290048108	2.434911	9.95E-05	hypermethylated
cg19810598	0.04744	0.256492973	2.434743	2.39E-05	hypermethylated
cg10812708	0.0434	0.234550811	2.434134	4.01E-06	hypermethylated
cg13210260	0.03531	0.190707027	2.433209	1.04E-05	hypermethylated
cg21383810	0.06267	0.338476216	2.433208	5.32E-05	hypermethylated
cg20985450	0.06788	0.366484324	2.432693	1.68E-05	hypermethylated
cg05997350	0.04731	0.255401081	2.432548	1.54E-05	hypermethylated
cg17926940	0.0446	0.240658378	2.431871	4.67E-06	hypermethylated
cg14373923	0.04868	0.262615676	2.431552	1.84E-05	hypermethylated
cg13437581	0.05362	0.289031892	2.430386	5.28E-06	hypermethylated
cg25081106	0.06254	0.337032973	2.430039	7.84E-05	hypermethylated
cg27134816	0.04482	0.241505946	2.429844	1.04E-05	hypermethylated
cg22167515	0.07059	0.380279459	2.429524	5.89E-06	hypermethylated
cg12676149	0.06172	0.332491351	2.429507	1.22E-06	hypermethylated
cg10184889	0.04425	0.238219459	2.428542	5.43E-06	hypermethylated
cg20664238	0.05989	0.321864324	2.426066	1.82E-05	hypermethylated
cg08572734	0.04268	0.229290811	2.425546	7.41E-06	hypermethylated
cg07213060	0.04481	0.240656757	2.425084	6.39E-06	hypermethylated
cg17240815	0.04342	0.233163784	2.424912	2.81E-05	hypermethylated
cg13703871	0.07499	0.402637297	2.424711	3.49E-06	hypermethylated

cg16348470	0.06525	0.350289189	2.424497	3.64E-06	hypermethylated
cg23774356	0.0731	0.392346486	2.424185	7.20E-05	hypermethylated
cg13092806	0.07681	0.412244324	2.424134	1.30E-05	hypermethylated
cg03234732	0.0529	0.283870811	2.423895	3.30E-06	hypermethylated
cg08768852	0.08713	0.467498378	2.42372	8.37E-06	hypermethylated
cg02043159	0.06258	0.335495135	2.422518	1.35E-05	hypermethylated
cg15489250	0.05411	0.290023243	2.422201	4.70E-05	hypermethylated
cg26630231	0.06127	0.328342162	2.421947	3.90E-06	hypermethylated
cg22665696	0.04852	0.259957838	2.421626	9.44E-06	hypermethylated
cg00159100	0.05367	0.287475676	2.421252	1.87E-05	hypermethylated
cg02119363	0.05245	0.280896757	2.421025	1.21E-05	hypermethylated
cg16422098	0.05813	0.3113	2.420951	1.94E-05	hypermethylated
cg21818713	0.05007	0.268064324	2.420561	5.28E-06	hypermethylated
cg15173134	0.06996	0.374392973	2.419951	4.53E-05	hypermethylated
cg18856581	0.07086	0.379206486	2.41994	5.00E-06	hypermethylated
cg09513990	0.0624	0.333921622	2.419892	1.22E-06	hypermethylated
cg12918457	0.07824	0.418630811	2.4197	8.71E-06	hypermethylated
cg03505117	0.06307	0.337405946	2.41946	5.73E-06	hypermethylated
cg02587316	0.06959	0.372262162	2.419367	4.24E-06	hypermethylated
cg20634967	0.05775	0.308812432	2.418838	6.93E-06	hypermethylated
cg00400832	0.06156	0.329119459	2.418546	7.86E-07	hypermethylated
cg10196720	0.06359	0.339872973	2.418124	7.12E-06	hypermethylated
cg27084746	0.06003	0.320622162	2.417119	1.21E-05	hypermethylated
cg17218713	0.0598	0.319356216	2.416949	0.000113	hypermethylated
cg03169018	0.0467	0.249394595	2.416936	5.43E-06	hypermethylated
cg08358166	0.06225	0.332293514	2.416312	2.15E-05	hypermethylated
cg17079833	0.05296	0.282547027	2.415516	3.76E-05	hypermethylated
cg24886257	0.05925	0.316099459	2.415492	6.05E-06	hypermethylated
cg23217126	0.04274	0.227933514	2.414954	3.04E-06	hypermethylated
cg22052659	0.05707	0.304274054	2.414567	8.60E-06	hypermethylated
cg07808555	0.05584	0.297655676	2.414274	7.41E-06	hypermethylated
cg03563308	0.04108	0.218888649	2.413689	2.74E-05	hypermethylated
cg25260137	0.06126	0.326403784	2.413641	6.75E-06	hypermethylated
cg02172150	0.06514	0.347014595	2.413381	2.87E-06	hypermethylated
cg25339566	0.06984	0.371822703	2.412489	9.82E-06	hypermethylated
cg21578219	0.06225	0.331201622	2.411564	1.52E-05	hypermethylated
cg03062549	0.05711	0.303827568	2.411437	2.79E-06	hypermethylated
cg08627720	0.0342	0.181856216	2.41073	1.06E-05	hypermethylated
cg15679813	0.06313	0.33527027	2.408927	4.53E-05	hypermethylated
cg00495860	0.06947	0.368848108	2.408565	3.21E-06	hypermethylated
cg20052718	0.03255	0.172803784	2.408405	1.73E-05	hypermethylated
cg08969532	0.04906	0.260420541	2.408224	7.20E-05	hypermethylated
cg17526573	0.08579	0.454998919	2.406982	1.93E-06	hypermethylated
cg17629929	0.05789	0.306892432	2.406347	2.04E-05	hypermethylated
cg27323784	0.0608	0.32228973	2.406215	1.12E-05	hypermethylated
cg06962944	0.06664	0.353209189	2.406063	2.96E-05	hypermethylated
cg27652464	0.04261	0.225795676	2.405754	7.32E-06	hypermethylated
cg24599434	0.04399	0.233037297	2.405313	1.93E-06	hypermethylated
cg08279008	0.06135	0.324916757	2.404935	5.89E-06	hypermethylated
cg11638181	0.06058	0.320705946	2.404338	1.15E-05	hypermethylated
cg14861089	0.06489	0.343387027	2.403767	1.35E-05	hypermethylated
cg17621718	0.06709	0.354804324	2.402854	3.04E-06	hypermethylated
cg00963169	0.06507	0.344034054	2.402487	1.12E-05	hypermethylated
cg15692992	0.05525	0.291831351	2.401089	4.01E-06	hypermethylated
cg12317601	0.05619	0.296787027	2.401043	4.01E-06	hypermethylated
cg27528660	0.04172	0.220280541	2.400531	4.53E-05	hypermethylated
cg01882870	0.05294	0.279348108	2.399634	5.19E-07	hypermethylated
cg23720732	0.05305	0.27986	2.399281	0.000324	hypermethylated

cg16783279	0.07095	0.374222162	2.39902	4.48E-06	hypermethylated
cg24203741	0.05329	0.280899459	2.398117	6.23E-05	hypermethylated
cg03530754	0.08339	0.439316757	2.397315	3.69E-06	hypermethylated
cg07090701	0.04569	0.240643784	2.396949	2.15E-05	hypermethylated
cg12374721	0.08593	0.45246	2.396556	4.24E-06	hypermethylated
cg06558014	0.06147	0.323626486	2.396375	1.21E-05	hypermethylated
cg23913421	0.0629	0.331088108	2.396083	2.10E-06	hypermethylated
cg17803868	0.04546	0.239161622	2.395316	7.29E-05	hypermethylated
cg24720580	0.02359	0.123932432	2.393306	7.52E-06	hypermethylated
cg00970313	0.05838	0.30668	2.393188	3.19E-05	hypermethylated
cg00795341	0.05251	0.275700541	2.392438	7.03E-06	hypermethylated
cg24912438	0.04741	0.248861622	2.39208	2.13E-05	hypermethylated
cg24483247	0.05171	0.271368649	2.391739	4.70E-05	hypermethylated
cg07529754	0.05104	0.267723784	2.391045	2.64E-06	hypermethylated
cg06962177	0.08455	0.443441081	2.390866	3.08E-06	hypermethylated
cg13982529	0.066	0.346121622	2.390741	3.90E-06	hypermethylated
cg17264670	0.0615	0.322416216	2.390266	0.000116	hypermethylated
cg09016242	0.08325	0.436358919	2.389993	4.18E-06	hypermethylated
cg23208513	0.05569	0.291869189	2.389832	3.39E-06	hypermethylated
cg23229395	0.05003	0.262077297	2.389127	1.56E-05	hypermethylated
cg05928342	0.0598	0.313141081	2.388595	4.48E-06	hypermethylated
cg13201808	0.07084	0.370594595	2.387206	1.50E-05	hypermethylated
cg17316316	0.05836	0.305270811	2.387038	3.59E-06	hypermethylated
cg09022943	0.04102	0.214498919	2.386571	2.48E-05	hypermethylated
cg03140968	0.05032	0.263127568	2.386559	1.28E-05	hypermethylated
cg25405145	0.04412	0.230703784	2.386537	1.11E-05	hypermethylated
cg08315202	0.09129	0.477283243	2.386317	5.00E-06	hypermethylated
cg15104644	0.05229	0.273364324	2.386218	5.72E-05	hypermethylated
cg26858704	0.05541	0.289543784	2.385563	4.26E-05	hypermethylated
cg01729827	0.07184	0.375245946	2.384977	7.32E-06	hypermethylated
cg08383315	0.06603	0.344718919	2.384227	5.14E-06	hypermethylated
cg03602280	0.08345	0.435651892	2.384192	8.37E-06	hypermethylated
cg25652029	0.05068	0.26454973	2.384051	0.000109	hypermethylated
cg08272731	0.08473	0.442218919	2.383816	3.30E-06	hypermethylated
cg11267630	0.06343	0.331049189	2.383808	1.14E-05	hypermethylated
cg13461241	0.0518	0.270323243	2.383662	3.69E-06	hypermethylated
cg22634891	0.03726	0.194284865	2.382474	4.88E-05	hypermethylated
cg26429655	0.06087	0.3173	2.382044	1.52E-05	hypermethylated
cg20846447	0.04092	0.213251892	2.38168	2.60E-06	hypermethylated
cg06204922	0.05072	0.264224324	2.381137	1.80E-05	hypermethylated
cg19727439	0.05693	0.296454054	2.380548	2.27E-05	hypermethylated
cg05743346	0.06141	0.319747027	2.380385	1.14E-05	hypermethylated
cg07071978	0.04583	0.238551351	2.379936	9.69E-06	hypermethylated
cg12395205	0.06257	0.325505405	2.379138	1.09E-05	hypermethylated
cg12079322	0.06011	0.312667027	2.37895	2.38E-05	hypermethylated
cg05417162	0.04902	0.254956757	2.37881	1.54E-06	hypermethylated
cg02970836	0.08952	0.465479459	2.378436	4.12E-06	hypermethylated
cg25655234	0.04778	0.248098919	2.376437	0.000207	hypermethylated
cg18401367	0.06829	0.354577838	2.376356	4.36E-05	hypermethylated
cg17424999	0.05185	0.269186486	2.37619	9.07E-06	hypermethylated
cg23965689	0.04456	0.231234054	2.375533	5.89E-06	hypermethylated
cg13661740	0.05746	0.297942703	2.374405	6.70E-05	hypermethylated
cg27062795	0.06662	0.345376216	2.374141	3.23E-05	hypermethylated
cg01445580	0.07245	0.375468108	2.373633	1.84E-05	hypermethylated
cg14654886	0.08304	0.430218378	2.373191	2.81E-05	hypermethylated
cg10831607	0.0616	0.318990811	2.372513	5.35E-06	hypermethylated
cg04243827	0.04947	0.256134595	2.372276	3.30E-06	hypermethylated
cg14667871	0.05523	0.285948649	2.372232	2.61E-05	hypermethylated

cg21200129	0.03703	0.191686486	2.371982	4.10E-05	hypermethylated
cg03184290	0.07974	0.412745946	2.371879	1.77E-06	hypermethylated
cg05396987	0.05437	0.28138973	2.371687	4.46E-07	hypermethylated
cg06273376	0.06283	0.325032432	2.371058	1.46E-05	hypermethylated
cg01729717	0.06016	0.311204324	2.370986	9.69E-06	hypermethylated
cg16086620	0.07822	0.404377297	2.370093	1.23E-05	hypermethylated
cg21545862	0.07624	0.394132432	2.37006	3.21E-06	hypermethylated
cg10617909	0.07868	0.40662	2.369612	8.15E-06	hypermethylated
cg22623133	0.04943	0.25536973	2.369129	5.73E-06	hypermethylated
cg09165441	0.07633	0.394254595	2.368805	1.11E-05	hypermethylated
cg13324546	0.05866	0.30296973	2.368725	1.54E-05	hypermethylated
cg01054478	0.06823	0.352331351	2.368455	7.32E-06	hypermethylated
cg06872705	0.05294	0.27330973	2.368107	2.49E-06	hypermethylated
cg15885337	0.0661	0.341060541	2.367306	1.08E-05	hypermethylated
cg08378830	0.05304	0.273576757	2.366793	1.12E-05	hypermethylated
cg05471296	0.06722	0.346674595	2.36662	2.10E-06	hypermethylated
cg11667020	0.03509	0.180914595	2.366177	6.39E-06	hypermethylated
cg08002883	0.06523	0.336282162	2.366065	0.000176	hypermethylated
cg19764599	0.05163	0.266117297	2.365781	3.44E-05	hypermethylated
cg00790098	0.05122	0.263953514	2.365505	3.15E-05	hypermethylated
cg23797411	0.07473	0.384974595	2.365004	2.79E-06	hypermethylated
cg25480336	0.05647	0.290808108	2.364511	0.000474	hypermethylated
cg18349911	0.04271	0.219792973	2.363499	2.35E-05	hypermethylated
cg25104105	0.07269	0.374030811	2.363328	3.40E-05	hypermethylated
cg19406736	0.04805	0.247236757	2.363285	3.12E-06	hypermethylated
cg18652900	0.07439	0.38275027	2.363223	6.93E-06	hypermethylated
cg15554678	0.05265	0.270644324	2.361893	1.35E-05	hypermethylated
cg17985646	0.09214	0.473637297	2.361883	9.57E-06	hypermethylated
cg08190291	0.0555	0.285254054	2.361688	1.89E-05	hypermethylated
cg19871940	0.06139	0.315385946	2.361043	1.84E-05	hypermethylated
cg10021878	0.05091	0.261351892	2.359973	1.06E-05	hypermethylated
cg06215569	0.07501	0.384987568	2.359657	1.43E-06	hypermethylated
cg24604013	0.07998	0.410389189	2.359282	1.65E-06	hypermethylated
cg16433156	0.04139	0.212325946	2.358927	1.72E-06	hypermethylated
cg13320291	0.06363	0.326268108	2.358279	0.000109	hypermethylated
cg03354113	0.06268	0.321311892	2.357897	4.88E-05	hypermethylated
cg17214381	0.07427	0.380654054	2.357629	6.39E-06	hypermethylated
cg00663077	0.08077	0.413543243	2.356147	5.07E-06	hypermethylated
cg01843946	0.0532	0.272384324	2.356146	1.48E-05	hypermethylated
cg20417527	0.04599	0.23531027	2.355172	1.26E-05	hypermethylated
cg02825373	0.06558	0.335517838	2.355062	2.42E-06	hypermethylated
cg10023272	0.03807	0.194757838	2.354955	4.24E-06	hypermethylated
cg01815529	0.05487	0.280664865	2.354759	5.93E-05	hypermethylated
cg18023283	0.08244	0.421283243	2.353374	5.65E-06	hypermethylated
cg03357798	0.07303	0.37305027	2.352809	2.39E-05	hypermethylated
cg06457317	0.05732	0.292712973	2.352376	1.52E-05	hypermethylated
cg09558195	0.04593	0.234548108	2.352375	6.66E-06	hypermethylated
cg15680755	0.04988	0.254675676	2.352128	1.87E-05	hypermethylated
cg23244488	0.07604	0.388237297	2.352108	2.42E-06	hypermethylated
cg20527270	0.06184	0.315733514	2.352095	2.15E-05	hypermethylated
cg02456226	0.07486	0.382102703	2.351694	6.84E-06	hypermethylated
cg08475953	0.08804	0.449335135	2.351561	2.36E-06	hypermethylated
cg10192893	0.06976	0.356023243	2.351499	2.46E-06	hypermethylated
cg03048654	0.06896	0.351845946	2.351112	5.89E-06	hypermethylated
cg12072560	0.06756	0.344658378	2.350926	1.58E-05	hypermethylated
cg13567542	0.07568	0.386047027	2.350793	1.15E-06	hypermethylated
cg02256049	0.06898	0.351796757	2.350492	1.50E-05	hypermethylated
cg05178576	0.06138	0.312963784	2.350155	2.61E-05	hypermethylated

cg08377819	0.05399	0.275182703	2.349626	3.15E-05	hypermethylated
cg13019491	0.07433	0.378641081	2.348814	1.28E-05	hypermethylated
cg25784220	0.06423	0.327154054	2.348651	0.0002	hypermethylated
cg26715571	0.04335	0.220752973	2.348329	0.000237	hypermethylated
cg01593190	0.04981	0.253592432	2.348004	1.54E-05	hypermethylated
cg16787600	0.06526	0.332249189	2.347995	7.83E-06	hypermethylated
cg10972973	0.04704	0.239475676	2.347919	1.23E-05	hypermethylated
cg18552380	0.05206	0.265013514	2.347819	2.29E-06	hypermethylated
cg10680051	0.05299	0.269704324	2.347587	7.29E-05	hypermethylated
cg04118306	0.04269	0.217254054	2.347413	1.02E-05	hypermethylated
cg14627172	0.06892	0.350727027	2.347354	3.04E-06	hypermethylated
cg07612562	0.0606	0.308364865	2.347249	1.42E-05	hypermethylated
cg20495645	0.05462	0.277874054	2.34693	9.16E-05	hypermethylated
cg12511214	0.05968	0.303495676	2.346357	1.89E-05	hypermethylated
cg13544006	0.05844	0.297168108	2.346251	2.15E-05	hypermethylated
cg26296488	0.10184	0.517798919	2.346088	3.74E-06	hypermethylated
cg09835225	0.07349	0.373463784	2.345348	1.99E-05	hypermethylated
cg14669274	0.08413	0.427363784	2.344772	4.12E-06	hypermethylated
cg24430140	0.07723	0.392198919	2.344352	5.43E-06	hypermethylated
cg00983904	0.07676	0.38977027	2.344197	1.77E-05	hypermethylated
cg13724788	0.0575	0.291685405	2.342779	7.75E-05	hypermethylated
cg09149648	0.05574	0.282735135	2.342666	5.45E-05	hypermethylated
cg08850461	0.07609	0.385937297	2.342588	5.07E-06	hypermethylated
cg10333808	0.06736	0.341290811	2.341038	6.48E-06	hypermethylated
cg12951849	0.05889	0.298372432	2.34102	1.39E-05	hypermethylated
cg08448701	0.06048	0.30635027	2.340652	2.04E-05	hypermethylated
cg24686074	0.05848	0.296205946	2.340585	8.63E-05	hypermethylated
cg27641522	0.02958	0.149823243	2.340568	1.31E-05	hypermethylated
cg04330449	0.07636	0.386724865	2.340419	2.04E-05	hypermethylated
cg23642130	0.09081	0.459814054	2.340127	1.54E-05	hypermethylated
cg11842610	0.06083	0.307785405	2.33907	2.57E-05	hypermethylated
cg02430692	0.05228	0.264249189	2.337568	4.12E-06	hypermethylated
cg12175729	0.07418	0.374750811	2.336829	8.32E-05	hypermethylated
cg16403344	0.05917	0.298854054	2.336503	9.27E-05	hypermethylated
cg22327646	0.0585	0.295285405	2.335602	1.99E-06	hypermethylated
cg17480760	0.07617	0.384475135	2.335595	1.02E-06	hypermethylated
cg10410142	0.05662	0.285407568	2.33364	3.15E-05	hypermethylated
cg07027251	0.04623	0.233011892	2.333502	3.26E-06	hypermethylated
cg12028548	0.03835	0.193282162	2.33341	1.09E-05	hypermethylated
cg27207308	0.05871	0.295877297	2.333321	2.92E-05	hypermethylated
cg06652199	0.04758	0.239687568	2.332728	3.85E-06	hypermethylated
cg03450948	0.07085	0.35686	2.332518	0.000108	hypermethylated
cg13930892	0.06876	0.346273514	2.332271	2.71E-05	hypermethylated
cg04434339	0.05504	0.277174054	2.33224	4.05E-05	hypermethylated
cg04673590	0.04895	0.2463	2.331036	5.45E-05	hypermethylated
cg20844851	0.0659	0.331412973	2.33028	1.73E-05	hypermethylated
cg07142797	0.05909	0.297088649	2.329908	1.99E-05	hypermethylated
cg07502439	0.04911	0.246892432	2.329794	6.22E-06	hypermethylated
cg05994320	0.0358	0.179958919	2.329636	2.02E-05	hypermethylated
cg15230781	0.06132	0.308205405	2.329463	1.15E-05	hypermethylated
cg23068913	0.06127	0.307871351	2.329075	7.52E-06	hypermethylated
cg13279673	0.05989	0.300683243	2.327857	2.49E-06	hypermethylated
cg18129621	0.05611	0.281705405	2.327857	3.36E-05	hypermethylated
cg02027945	0.08204	0.411707568	2.327221	2.42E-05	hypermethylated
cg18530551	0.06251	0.313646486	2.32698	1.08E-05	hypermethylated
cg21229268	0.06366	0.319181081	2.325916	1.31E-05	hypermethylated
cg13689003	0.06211	0.311342703	2.325606	0.0002	hypermethylated
cg13678973	0.07874	0.394561081	2.32508	1.56E-05	hypermethylated

cg23737737	0.07419	0.371419459	2.323753	2.21E-05	hypermethylated
cg25188395	0.07402	0.370385405	2.32304	1.54E-06	hypermethylated
cg06204735	0.07387	0.36963027	2.323022	3.49E-05	hypermethylated
cg19156875	0.07344	0.36746973	2.322987	4.48E-06	hypermethylated
cg00459623	0.06414	0.320778378	2.322281	2.89E-05	hypermethylated
cg22153181	0.08117	0.405725405	2.321485	2.71E-06	hypermethylated
cg02623991	0.06752	0.337351892	2.320867	3.85E-05	hypermethylated
cg11002404	0.05281	0.263847568	2.320822	0.000187	hypermethylated
cg02281208	0.0733	0.365821081	2.319253	2.49E-06	hypermethylated
cg14186641	0.06774	0.338067568	2.319232	6.46E-05	hypermethylated
cg26639039	0.0331	0.165169189	2.319041	1.85E-06	hypermethylated
cg08640634	0.06643	0.331449189	2.318881	1.94E-05	hypermethylated
cg15186181	0.0765	0.381652432	2.318728	1.54E-05	hypermethylated
cg17307479	0.05419	0.270309189	2.318512	0.000259	hypermethylated
cg02858606	0.05376	0.268123784	2.318294	1.73E-05	hypermethylated
cg23754772	0.04425	0.220590811	2.317623	1.82E-05	hypermethylated
cg16709232	0.07508	0.374253514	2.317515	4.36E-06	hypermethylated
cg18795809	0.07426	0.369977297	2.31678	8.73E-05	hypermethylated
cg15928093	0.05935	0.295615135	2.3164	5.72E-05	hypermethylated
cg10864319	0.05636	0.280695676	2.316263	0.000187	hypermethylated
cg01791371	0.06701	0.333646486	2.315872	2.07E-06	hypermethylated
cg11390378	0.08062	0.401364865	2.315705	1.02E-05	hypermethylated
cg11723848	0.04614	0.229589189	2.314965	0.000124	hypermethylated
cg14002960	0.07	0.348277838	2.314812	6.57E-06	hypermethylated
cg00711088	0.05372	0.267154595	2.314144	1.44E-05	hypermethylated
cg01369750	0.05433	0.270131351	2.31384	8.71E-06	hypermethylated
cg11183632	0.0562	0.279377297	2.313573	2.89E-05	hypermethylated
cg14238959	0.06708	0.333425946	2.313412	2.74E-05	hypermethylated
cg20930366	0.03627	0.180199459	2.312746	2.04E-05	hypermethylated
cg20482698	0.07626	0.378875676	2.312726	1.75E-05	hypermethylated
cg23645199	0.04852	0.241055676	2.312715	6.86E-05	hypermethylated
cg23353952	0.06525	0.324159459	2.312654	1.93E-06	hypermethylated
cg13347257	0.06428	0.319170811	2.311887	1.80E-05	hypermethylated
cg07860213	0.09462	0.469665946	2.311418	2.23E-06	hypermethylated
cg15063116	0.04897	0.24304	2.311224	2.51E-05	hypermethylated
cg06008912	0.05901	0.292823784	2.311001	0.001587	hypermethylated
cg24843380	0.06766	0.335568649	2.310233	2.18E-05	hypermethylated
cg18134312	0.04337	0.215070811	2.310042	2.30E-05	hypermethylated
cg22982368	0.04928	0.244170811	2.308817	1.23E-05	hypermethylated
cg22717014	0.06614	0.32768973	2.308736	4.36E-06	hypermethylated
cg11942956	0.05847	0.289608108	2.308333	2.32E-05	hypermethylated
cg26476852	0.08225	0.407273514	2.30791	1.75E-06	hypermethylated
cg24643706	0.05489	0.271647027	2.307118	6.05E-06	hypermethylated
cg05241461	0.07251	0.358698919	2.306522	5.73E-06	hypermethylated
cg22747746	0.07116	0.351969189	2.306311	9.07E-06	hypermethylated
cg09090724	0.05124	0.253435135	2.306274	1.99E-05	hypermethylated
cg20743280	0.04956	0.245092973	2.306081	3.11E-05	hypermethylated
cg19819285	0.04257	0.210478378	2.305763	6.30E-06	hypermethylated
cg00866976	0.05315	0.26272	2.305384	1.54E-05	hypermethylated
cg00532157	0.06301	0.311395135	2.305094	4.80E-06	hypermethylated
cg23536473	0.08658	0.427776757	2.304752	4.86E-06	hypermethylated
cg11267829	0.04942	0.244172973	2.304737	0.000749	hypermethylated
cg03445694	0.04103	0.202690811	2.30453	4.86E-06	hypermethylated
cg21200539	0.05736	0.283331892	2.304376	5.14E-06	hypermethylated
cg25825488	0.05812	0.287083243	2.304363	1.18E-05	hypermethylated
cg26205771	0.06783	0.335013514	2.304224	1.66E-05	hypermethylated
cg04597433	0.08478	0.418729189	2.304222	2.05E-06	hypermethylated
cg25951981	0.04441	0.219302162	2.303964	1.08E-05	hypermethylated

cg12396325	0.07287	0.359627568	2.303107	2.07E-05	hypermethylated
cg20386316	0.06183	0.304952973	2.302208	1.24E-06	hypermethylated
cg22757824	0.07347	0.362357297	2.302186	8.15E-06	hypermethylated
cg04954559	0.05615	0.276844865	2.30172	1.80E-05	hypermethylated
cg00287312	0.07425	0.366068108	2.301649	1.15E-05	hypermethylated
cg23207990	0.06851	0.337762703	2.301624	1.73E-05	hypermethylated
cg00674365	0.0821	0.404755135	2.301595	2.04E-05	hypermethylated
cg07034660	0.05646	0.278314054	2.301413	3.07E-05	hypermethylated
cg10527010	0.06984	0.344160541	2.300956	4.01E-06	hypermethylated
cg23290344	0.08689	0.427754595	2.299521	4.48E-06	hypermethylated
cg11469061	0.04515	0.222264865	2.299482	3.08E-06	hypermethylated
cg09780241	0.06976	0.34341027	2.299461	1.89E-05	hypermethylated
cg11381539	0.06712	0.330394595	2.299375	4.30E-06	hypermethylated
cg14930075	0.06364	0.313155135	2.298872	0.000145	hypermethylated
cg09234518	0.04592	0.225787027	2.297768	1.08E-05	hypermethylated
cg19909787	0.0613	0.301336757	2.297418	5.19E-05	hypermethylated
cg19099050	0.06661	0.327382162	2.297165	5.73E-06	hypermethylated
cg17349389	0.05677	0.278771892	2.295884	2.18E-05	hypermethylated
cg02246645	0.04578	0.22476	2.295596	1.62E-05	hypermethylated
cg26963797	0.06612	0.324599459	2.295502	1.58E-05	hypermethylated
cg21426003	0.07101	0.348546486	2.295257	1.99E-05	hypermethylated
cg08276289	0.06205	0.304491892	2.294901	2.92E-05	hypermethylated
cg04098866	0.05524	0.271024865	2.29464	5.35E-06	hypermethylated
cg05941108	0.0727	0.356588649	2.294234	6.20E-07	hypermethylated
cg17466857	0.06125	0.300427027	2.294233	4.58E-05	hypermethylated
cg12850078	0.04649	0.227996216	2.294018	0.000161	hypermethylated
cg23699196	0.05648	0.276916216	2.293638	1.04E-05	hypermethylated
cg17294725	0.06452	0.316259459	2.29329	6.05E-06	hypermethylated
cg18143243	0.06246	0.306084324	2.292925	0.000126	hypermethylated
cg10387551	0.06164	0.302011892	2.292667	1.44E-05	hypermethylated
cg06132028	0.0513	0.251182162	2.291703	1.50E-05	hypermethylated
cg12668482	0.0482	0.235918378	2.291183	1.46E-05	hypermethylated
cg06113354	0.05488	0.268409189	2.290082	2.42E-06	hypermethylated
cg15617814	0.09488	0.463971351	2.28986	9.31E-06	hypermethylated
cg09321400	0.08217	0.401555135	2.288914	3.30E-06	hypermethylated
cg12995800	0.05833	0.285039459	2.288852	4.58E-05	hypermethylated
cg10364040	0.08821	0.430895135	2.288323	5.89E-06	hypermethylated
cg18249634	0.07637	0.373044865	2.288271	2.99E-06	hypermethylated
cg10821845	0.06499	0.317283243	2.287482	3.76E-05	hypermethylated
cg02071005	0.05399	0.26345027	2.286767	2.71E-05	hypermethylated
cg22557662	0.07162	0.349379459	2.28636	1.25E-05	hypermethylated
cg20884887	0.09269	0.452129189	2.286249	3.90E-06	hypermethylated
cg05525743	0.05153	0.251250811	2.285644	6.93E-06	hypermethylated
cg26650638	0.08986	0.438127568	2.2856	1.09E-05	hypermethylated
cg14304817	0.05345	0.260573514	2.285429	3.62E-05	hypermethylated
cg23952663	0.04929	0.240190811	2.284814	2.87E-06	hypermethylated
cg07455757	0.06048	0.294701081	2.284722	7.29E-05	hypermethylated
cg18325439	0.04459	0.21716973	2.284031	6.01E-05	hypermethylated
cg26595278	0.07468	0.363509189	2.283198	9.07E-06	hypermethylated
cg02700891	0.07085	0.344820541	2.283006	1.28E-05	hypermethylated
cg14511946	0.05779	0.281246486	2.282943	5.00E-05	hypermethylated
cg04970570	0.07494	0.364533514	2.282244	5.00E-06	hypermethylated
cg17303756	0.05046	0.245408649	2.281974	1.94E-05	hypermethylated
cg07204550	0.0483	0.234807027	2.28138	1.84E-05	hypermethylated
cg02653557	0.06207	0.301742162	2.281348	1.62E-05	hypermethylated
cg18255353	0.06884	0.334568649	2.280983	4.36E-06	hypermethylated
cg00266286	0.04203	0.204248649	2.280835	4.30E-06	hypermethylated
cg07015911	0.06352	0.308471351	2.279854	0.000283	hypermethylated

cg02332982	0.06623	0.321601081	2.279715	1.39E-05	hypermethylated
cg19142026	0.08553	0.415245946	2.279464	3.59E-06	hypermethylated
cg17322443	0.06123	0.297173514	2.278995	7.83E-06	hypermethylated
cg11855516	0.03955	0.191907568	2.278662	0.000122	hypermethylated
cg22711111	0.07932	0.384803243	2.278364	9.11E-07	hypermethylated
cg24884703	0.0578	0.280208649	2.27736	6.66E-06	hypermethylated
cg07149609	0.08523	0.413123784	2.277141	2.29E-06	hypermethylated
cg23500059	0.05288	0.256263243	2.276832	3.67E-05	hypermethylated
cg06913501	0.0538	0.260623784	2.276291	1.75E-05	hypermethylated
cg12521353	0.06418	0.310798919	2.275786	0.000287	hypermethylated
cg24556441	0.08441	0.408525405	2.27494	6.57E-06	hypermethylated
cg00128702	0.04761	0.230358919	2.274547	1.80E-06	hypermethylated
cg05870739	0.05886	0.284781622	2.274497	5.00E-06	hypermethylated
cg15797110	0.06437	0.31142973	2.274446	1.82E-06	hypermethylated
cg02758612	0.07499	0.362503243	2.273224	1.01E-05	hypermethylated
cg07907386	0.06884	0.332684865	2.272837	1.49E-06	hypermethylated
cg26813646	0.07173	0.346537838	2.272364	3.39E-06	hypermethylated
cg19030579	0.05823	0.281299459	2.272272	3.40E-07	hypermethylated
cg21200408	0.07603	0.367041081	2.271301	4.80E-06	hypermethylated
cg08832906	0.0794	0.383234595	2.271017	2.67E-05	hypermethylated
cg22310279	0.04681	0.225890811	2.270737	8.60E-06	hypermethylated
cg17030173	0.07761	0.374482162	2.270583	4.36E-06	hypermethylated
cg08431563	0.07224	0.348288649	2.269414	2.04E-05	hypermethylated
cg17495912	0.10792	0.520276757	2.269317	6.75E-06	hypermethylated
cg04963514	0.05468	0.263575676	2.269132	0.00035	hypermethylated
cg17934871	0.07732	0.372675676	2.269007	5.43E-06	hypermethylated
cg14752336	0.04355	0.209892973	2.268909	4.86E-06	hypermethylated
cg03349020	0.05529	0.266422162	2.268624	9.60E-05	hypermethylated
cg13012916	0.07639	0.367832973	2.267595	3.79E-06	hypermethylated
cg23806894	0.08232	0.396372432	2.267542	4.60E-06	hypermethylated
cg10453420	0.05621	0.270542162	2.266955	2.74E-05	hypermethylated
cg00765783	0.08153	0.392157297	2.26603	6.57E-06	hypermethylated
cg07115542	0.06468	0.311061081	2.265806	0.000232	hypermethylated
cg05724110	0.05408	0.260022703	2.265471	7.93E-06	hypermethylated
cg25720804	0.08773	0.421798919	2.265413	1.63E-06	hypermethylated
cg01477985	0.08029	0.385998378	2.265303	1.39E-05	hypermethylated
cg09729848	0.05982	0.287507568	2.2649	7.52E-06	hypermethylated
cg03774803	0.07063	0.339433514	2.264776	9.82E-06	hypermethylated
cg26947626	0.08205	0.394298378	2.264713	2.87E-06	hypermethylated
cg25403721	0.0751	0.360774054	2.264211	3.49E-06	hypermethylated
cg02801786	0.08235	0.395461622	2.263697	7.83E-06	hypermethylated
cg08249988	0.07649	0.36729027	2.263578	3.57E-05	hypermethylated
cg02742906	0.07532	0.361484324	2.262828	6.39E-06	hypermethylated
cg08560111	0.05054	0.242411351	2.26196	7.52E-06	hypermethylated
cg27301032	0.05121	0.245615676	2.261905	6.93E-06	hypermethylated
cg24073466	0.04777	0.229005946	2.261208	2.56E-06	hypermethylated
cg15811515	0.11908	0.570521622	2.26035	2.49E-06	hypermethylated
cg12127472	0.0807	0.386569189	2.260086	8.60E-06	hypermethylated
cg23736843	0.07525	0.360393514	2.25981	2.51E-05	hypermethylated
cg00090936	0.04632	0.221827027	2.259728	6.93E-06	hypermethylated
cg19275261	0.05153	0.246603784	2.25871	0.000193	hypermethylated
cg09656848	0.07226	0.345692432	2.25822	0.000764	hypermethylated
cg21532325	0.06027	0.28832973	2.258208	0.000327	hypermethylated
cg13063344	0.06388	0.305521622	2.257838	2.21E-05	hypermethylated
cg07124687	0.08532	0.407995135	2.257596	6.08E-05	hypermethylated
cg17689721	0.05908	0.282484865	2.257432	8.60E-06	hypermethylated
cg13031171	0.05244	0.250651351	2.256942	2.74E-05	hypermethylated
cg16055026	0.05361	0.256198378	2.256687	1.97E-05	hypermethylated

cg14885748	0.08039	0.384037838	2.256161	3.21E-06	hypermethylated
cg18180569	0.0601	0.287070811	2.25597	2.39E-05	hypermethylated
cg21135135	0.09035	0.431431351	2.255535	2.29E-06	hypermethylated
cg00661970	0.08136	0.388343243	2.254941	3.21E-06	hypermethylated
cg07180307	0.07903	0.377153514	2.25468	1.08E-05	hypermethylated
cg12926938	0.06362	0.303609189	2.254663	0.000335	hypermethylated
cg22802813	0.06204	0.295863784	2.253663	1.46E-05	hypermethylated
cg16690757	0.05734	0.273421081	2.253511	5.00E-06	hypermethylated
cg05500840	0.07224	0.344336216	2.252948	1.99E-06	hypermethylated
cg21159778	0.04832	0.230290811	2.252764	5.38E-05	hypermethylated
cg10811045	0.08881	0.423235676	2.252667	4.24E-06	hypermethylated
cg22043168	0.0671	0.319631892	2.252027	2.15E-05	hypermethylated
cg13473356	0.06396	0.304558378	2.251477	2.13E-05	hypermethylated
cg11224582	0.09089	0.432696757	2.251163	1.75E-05	hypermethylated
cg14817655	0.07086	0.337332973	2.25113	8.15E-06	hypermethylated
cg13023870	0.08268	0.393405946	2.250408	2.79E-06	hypermethylated
cg27347269	0.05504	0.261873514	2.250318	9.07E-06	hypermethylated
cg24524352	0.05864	0.278982703	2.250219	8.03E-05	hypermethylated
cg04360049	0.0533	0.253563243	2.250138	9.60E-05	hypermethylated
cg01947130	0.05667	0.26949027	2.249576	4.26E-05	hypermethylated
cg09194159	0.06299	0.299496757	2.249346	0.000159	hypermethylated
cg08958294	0.07429	0.353080541	2.248757	1.30E-05	hypermethylated
cg22869726	0.09284	0.441014595	2.248008	1.37E-06	hypermethylated
cg13874817	0.05688	0.270096757	2.247483	3.07E-05	hypermethylated
cg10723962	0.04481	0.21278	2.24747	0.000145	hypermethylated
cg27423760	0.08648	0.410616757	2.247354	1.64E-05	hypermethylated
cg12563644	0.05315	0.252336757	2.247209	0.000857	hypermethylated
cg20438306	0.0552	0.262001081	2.246833	3.69E-06	hypermethylated
cg09683350	0.06943	0.329522703	2.246747	1.25E-05	hypermethylated
cg25984671	0.08598	0.408041081	2.246641	4.48E-06	hypermethylated
cg14096889	0.0566	0.268562703	2.246385	1.15E-05	hypermethylated
cg23650423	0.06211	0.294692432	2.246313	4.30E-06	hypermethylated
cg15736169	0.05663	0.268585946	2.245745	1.62E-05	hypermethylated
cg07963234	0.08743	0.414424865	2.24491	3.69E-06	hypermethylated
cg12682684	0.07111	0.337063243	2.244895	2.57E-05	hypermethylated
cg09124223	0.07188	0.340557838	2.244238	5.50E-06	hypermethylated
cg23205387	0.06808	0.322475676	2.243887	1.18E-05	hypermethylated
cg05732832	0.06514	0.308511892	2.24371	1.88E-06	hypermethylated
cg20585530	0.08861	0.419619459	2.24354	3.12E-06	hypermethylated
cg23554956	0.05476	0.259242703	2.243109	1.84E-05	hypermethylated
cg19472098	0.06887	0.325810811	2.242087	2.89E-05	hypermethylated
cg09227138	0.04044	0.191311351	2.242068	0.000102	hypermethylated
cg13575339	0.05036	0.238218919	2.241938	8.15E-06	hypermethylated
cg20129213	0.08758	0.414102162	2.241313	0.000101	hypermethylated
cg02076785	0.05338	0.252231892	2.240379	2.32E-05	hypermethylated
cg06506966	0.06493	0.306764324	2.240174	8.73E-05	hypermethylated
cg10034890	0.06678	0.315360541	2.239514	3.00E-05	hypermethylated
cg22674699	0.11636	0.549412973	2.239296	3.79E-06	hypermethylated
cg14473102	0.11216	0.529562162	2.239242	1.63E-06	hypermethylated
cg05860723	0.06779	0.319663784	2.237411	3.90E-06	hypermethylated
cg03638172	0.06559	0.309098919	2.236521	2.45E-05	hypermethylated
cg24569447	0.07208	0.339608649	2.236202	6.84E-06	hypermethylated
cg17583413	0.04742	0.223411892	2.236138	1.15E-05	hypermethylated
cg14625113	0.07298	0.343768108	2.235863	3.19E-05	hypermethylated
cg02900995	0.05799	0.273137297	2.23575	2.07E-05	hypermethylated
cg06401021	0.08118	0.382224865	2.235225	5.12E-05	hypermethylated
cg03078363	0.05725	0.269518378	2.235036	3.53E-05	hypermethylated
cg06003986	0.05597	0.263301081	2.233988	1.04E-05	hypermethylated

cg26162582	0.07228	0.340014595	2.233928	1.18E-05	hypermethylated
cg08858649	0.10011	0.470872432	2.23375	1.02E-06	hypermethylated
cg14644752	0.0577	0.271388108	2.233714	7.12E-05	hypermethylated
cg03900143	0.09412	0.442205946	2.232145	1.88E-06	hypermethylated
cg14094983	0.06339	0.29746973	2.230416	1.25E-05	hypermethylated
cg05404010	0.05376	0.252257838	2.230294	1.63E-06	hypermethylated
cg00334063	0.1085	0.509108108	2.230277	1.93E-06	hypermethylated
cg04290346	0.08585	0.402807568	2.230201	6.05E-06	hypermethylated
cg02828023	0.06429	0.301585405	2.2299	4.64E-05	hypermethylated
cg11178863	0.09053	0.424508649	2.229326	6.05E-06	hypermethylated
cg21718267	0.06421	0.301017838	2.228979	1.75E-05	hypermethylated
cg06528306	0.09526	0.446497838	2.228711	1.25E-05	hypermethylated
cg12271981	0.08055	0.377537838	2.228665	1.82E-06	hypermethylated
cg00619126	0.06807	0.318961622	2.228292	2.71E-06	hypermethylated
cg10671668	0.08165	0.38249027	2.227898	4.48E-06	hypermethylated
cg19870512	0.09275	0.434264865	2.227156	5.89E-06	hypermethylated
cg06651311	0.06614	0.309524865	2.22646	3.00E-05	hypermethylated
cg18803104	0.08854	0.41422	2.225996	6.38E-05	hypermethylated
cg22574825	0.06602	0.308823784	2.225809	5.72E-05	hypermethylated
cg19384289	0.08288	0.387662703	2.225706	5.00E-06	hypermethylated
cg04812556	0.07868	0.367972432	2.225529	9.49E-05	hypermethylated
cg19206040	0.07356	0.343980541	2.225334	2.57E-05	hypermethylated
cg24368848	0.07879	0.368355135	2.225013	4.12E-06	hypermethylated
cg22604316	0.08007	0.374335135	2.224997	6.22E-06	hypermethylated
cg17393267	0.0553	0.258488649	2.22475	0.000115	hypermethylated
cg23715830	0.08846	0.413448108	2.224609	7.22E-06	hypermethylated
cg11539780	0.06822	0.318758378	2.224197	3.90E-05	hypermethylated
cg12456714	0.0864	0.403422703	2.223189	5.35E-06	hypermethylated
cg19024969	0.05999	0.28009027	2.223098	3.62E-05	hypermethylated
cg23474501	0.08592	0.401128108	2.222997	1.09E-05	hypermethylated
cg20395967	0.0874	0.407968649	2.222753	5.89E-06	hypermethylated
cg13589108	0.06604	0.30815027	2.222222	1.84E-05	hypermethylated
cg26541867	0.06369	0.297152973	2.222067	7.03E-05	hypermethylated
cg16801887	0.07509	0.350212973	2.22154	1.89E-05	hypermethylated
cg14630357	0.04759	0.221936757	2.221418	3.53E-05	hypermethylated
cg18921771	0.08201	0.382452432	2.221409	1.18E-06	hypermethylated
cg06389697	0.06182	0.288251351	2.221182	1.54E-05	hypermethylated
cg15617155	0.07506	0.34994973	2.221031	5.65E-06	hypermethylated
cg23358612	0.0833	0.388064865	2.219909	1.72E-06	hypermethylated
cg11592503	0.07193	0.335065405	2.219777	6.23E-05	hypermethylated
cg03011535	0.07421	0.345630811	2.219546	5.51E-05	hypermethylated
cg05269218	0.07394	0.344152432	2.218621	7.72E-06	hypermethylated
cg19423543	0.05109	0.237795135	2.218606	1.52E-05	hypermethylated
cg25088758	0.05527	0.257225946	2.218468	4.64E-05	hypermethylated
cg10615842	0.0674	0.313457297	2.217448	9.94E-07	hypermethylated
cg21684012	0.0577	0.268322162	2.217323	3.67E-05	hypermethylated
cg16654801	0.05092	0.236563243	2.215922	9.96E-06	hypermethylated
cg11064034	0.06285	0.291952973	2.215751	3.90E-06	hypermethylated
cg05488770	0.07438	0.345451351	2.215496	1.17E-05	hypermethylated
cg18443359	0.07535	0.349856757	2.215085	4.12E-06	hypermethylated
cg14428146	0.06817	0.31648973	2.21495	1.62E-05	hypermethylated
cg00418216	0.09063	0.420636757	2.214514	5.81E-06	hypermethylated
cg00910518	0.07408	0.343747027	2.214191	2.99E-06	hypermethylated
cg07065111	0.08703	0.403821081	2.214132	1.29E-06	hypermethylated
cg13826890	0.05304	0.246104865	2.21412	1.51E-06	hypermethylated
cg05258261	0.06394	0.296678378	2.214109	1.84E-05	hypermethylated
cg18118685	0.07176	0.332833514	2.213549	3.40E-05	hypermethylated
cg10375890	0.06832	0.316591892	2.212244	0.000117	hypermethylated

cg15396686	0.09453	0.438009189	2.212117	4.01E-06	hypermethylated
cg23174932	0.0597	0.276611892	2.21206	2.57E-05	hypermethylated
cg11108269	0.05958	0.276051351	2.212037	0.000174	hypermethylated
cg05164933	0.07881	0.365102162	2.21185	1.18E-05	hypermethylated
cg07995179	0.0647	0.299709189	2.211726	0.000152	hypermethylated
cg12065366	0.05765	0.267022162	2.211567	0.000259	hypermethylated
cg07014523	0.05325	0.246638378	2.211544	0.000245	hypermethylated
cg15920654	0.06595	0.305329189	2.210921	6.05E-06	hypermethylated
cg04974290	0.06686	0.309256757	2.20959	3.59E-06	hypermethylated
cg26759954	0.05917	0.273669189	2.209495	8.04E-06	hypermethylated
cg10116864	0.06159	0.28473027	2.208828	4.70E-05	hypermethylated
cg01866955	0.04816	0.222581622	2.208427	1.09E-05	hypermethylated
cg10789281	0.05615	0.259458378	2.208145	2.51E-05	hypermethylated
cg01184975	0.08318	0.384346486	2.208099	1.44E-05	hypermethylated
cg18035255	0.04253	0.196511892	2.208064	2.67E-05	hypermethylated
cg05874561	0.06703	0.309709189	2.208035	4.07E-06	hypermethylated
cg02387803	0.07511	0.347032432	2.207994	6.57E-06	hypermethylated
cg12853633	0.05844	0.269896757	2.20738	1.31E-05	hypermethylated
cg06399302	0.08529	0.393777297	2.206931	4.60E-06	hypermethylated
cg00812438	0.08589	0.396543243	2.206916	6.75E-06	hypermethylated
cg03927893	0.04944	0.22819027	2.206487	2.02E-05	hypermethylated
cg16675507	0.09077	0.418808649	2.206004	7.93E-06	hypermethylated
cg03217253	0.06481	0.299014054	2.205925	0.000193	hypermethylated
cg22284043	0.05027	0.231858919	2.205478	0.000159	hypermethylated
cg15543616	0.05011	0.231015676	2.20482	3.26E-06	hypermethylated
cg24011260	0.06516	0.300397838	2.204816	2.21E-05	hypermethylated
cg09175749	0.04916	0.226516216	2.204058	5.28E-06	hypermethylated
cg02155398	0.0804	0.370433514	2.203947	1.39E-05	hypermethylated
cg20422099	0.07009	0.322868108	2.203664	1.54E-05	hypermethylated
cg10866298	0.06367	0.293231892	2.203356	8.15E-06	hypermethylated
cg04072323	0.06462	0.29758	2.203225	8.83E-06	hypermethylated
cg00343633	0.0761	0.350355135	2.20285	9.19E-06	hypermethylated
cg11312896	0.07578	0.348732973	2.202234	2.87E-06	hypermethylated
cg16288089	0.07746	0.356434595	2.202114	4.82E-05	hypermethylated
cg04415798	0.0953	0.43821027	2.201075	4.36E-06	hypermethylated
cg23827572	0.06568	0.301900541	2.200547	4.07E-06	hypermethylated
cg04248446	0.05221	0.239976216	2.200493	7.03E-05	hypermethylated
cg15603568	0.08397	0.385909189	2.200316	1.40E-05	hypermethylated
cg04510788	0.06619	0.304113514	2.199925	1.17E-05	hypermethylated
cg09851951	0.07737	0.355394054	2.199573	5.43E-06	hypermethylated
cg07212778	0.05907	0.271215135	2.19894	5.79E-05	hypermethylated
cg26019112	0.0694	0.318618919	2.198824	6.05E-06	hypermethylated
cg05583608	0.05498	0.252381081	2.198625	4.58E-05	hypermethylated
cg05230054	0.06175	0.283354054	2.198095	2.27E-05	hypermethylated
cg12601945	0.06044	0.277301622	2.19788	3.40E-05	hypermethylated
cg18789663	0.08025	0.368188108	2.19787	2.57E-05	hypermethylated
cg05057720	0.10251	0.470278378	2.19775	1.31E-05	hypermethylated
cg11260046	0.05678	0.260355676	2.197029	0.000667	hypermethylated
cg12113984	0.06354	0.291192973	2.196239	4.48E-06	hypermethylated
cg06496344	0.07582	0.347390811	2.195909	1.04E-05	hypermethylated
cg06598836	0.09854	0.451354054	2.195478	9.57E-06	hypermethylated
cg03679755	0.07352	0.336723243	2.195355	1.68E-05	hypermethylated
cg14836602	0.09014	0.412817838	2.195266	1.77E-06	hypermethylated
cg26836696	0.05604	0.256544324	2.194679	1.71E-05	hypermethylated
cg16418810	0.06531	0.298954595	2.194551	0.000109	hypermethylated
cg25307168	0.09176	0.419951892	2.194287	6.75E-06	hypermethylated
cg10453719	0.08053	0.368524865	2.194164	6.93E-06	hypermethylated
cg11907729	0.07466	0.341626486	2.194012	2.61E-05	hypermethylated

cg09556826	0.07484	0.342444865	2.19399	8.63E-05	hypermethylated
cg26822097	0.06885	0.314933514	2.193519	5.14E-06	hypermethylated
cg14699728	0.11378	0.520407027	2.193393	3.39E-06	hypermethylated
cg13172637	0.05713	0.26128973	2.19333	1.33E-05	hypermethylated
cg07568194	0.06023	0.275265405	2.192269	4.86E-06	hypermethylated
cg14507868	0.07111	0.324974595	2.192203	3.53E-05	hypermethylated
cg17222500	0.04871	0.222564324	2.191932	9.57E-06	hypermethylated
cg16719466	0.05821	0.265714595	2.190539	3.12E-06	hypermethylated
cg10652393	0.06899	0.314784865	2.189907	1.64E-05	hypermethylated
cg26398467	0.06567	0.299547568	2.189479	8.84E-07	hypermethylated
cg10304637	0.07325	0.334113514	2.189438	6.78E-07	hypermethylated
cg04285275	0.06036	0.275297838	2.189329	1.09E-05	hypermethylated
cg26831119	0.09776	0.44582973	2.189177	1.22E-06	hypermethylated
cg06159352	0.08107	0.369644324	2.188898	1.94E-05	hypermethylated
cg13060997	0.06829	0.311237297	2.188269	2.30E-05	hypermethylated
cg21488538	0.07144	0.325593514	2.188268	2.24E-05	hypermethylated
cg19754554	0.05491	0.250189189	2.187879	1.64E-05	hypermethylated
cg08856946	0.06193	0.282094595	2.187469	5.93E-05	hypermethylated
cg10143811	0.07578	0.345086486	2.187069	9.07E-06	hypermethylated
cg07462540	0.06978	0.317694595	2.186755	6.70E-05	hypermethylated
cg26983710	0.06663	0.303318378	2.186589	0.000102	hypermethylated
cg26034516	0.05914	0.269210811	2.18653	9.38E-05	hypermethylated
cg04525757	0.06163	0.280488649	2.186238	1.04E-05	hypermethylated
cg26365534	0.05624	0.255937838	2.186125	3.44E-05	hypermethylated
cg23036804	0.05056	0.230051892	2.185891	2.57E-06	hypermethylated
cg07846220	0.08275	0.376334054	2.185183	1.99E-05	hypermethylated
cg22784954	0.12471	0.567152432	2.185159	7.12E-06	hypermethylated
cg26744375	0.05416	0.246305405	2.185149	0.000168	hypermethylated
cg04249522	0.06012	0.273403784	2.185116	2.99E-06	hypermethylated
cg02387326	0.0612	0.278315135	2.185116	1.04E-05	hypermethylated
cg13539424	0.0622	0.282853514	2.185069	1.58E-05	hypermethylated
cg10016380	0.05676	0.258062162	2.184772	1.21E-05	hypermethylated
cg25020286	0.06809	0.309481081	2.184336	3.19E-05	hypermethylated
cg06629130	0.06654	0.30243027	2.184309	9.31E-06	hypermethylated
cg03929977	0.06414	0.291511892	2.184258	1.46E-05	hypermethylated
cg12500879	0.08966	0.407467568	2.184149	1.58E-05	hypermethylated
cg20667845	0.03133	0.142367568	2.184004	5.43E-06	hypermethylated
cg02523640	0.08436	0.383289189	2.183802	4.01E-06	hypermethylated
cg03205584	0.09021	0.409854054	2.183751	8.37E-06	hypermethylated
cg13834844	0.05723	0.260013514	2.183743	7.12E-05	hypermethylated
cg09142313	0.09614	0.436525946	2.182859	2.71E-06	hypermethylated
cg24439256	0.05174	0.234911351	2.182764	4.10E-05	hypermethylated
cg03807298	0.08088	0.367103243	2.182331	1.46E-05	hypermethylated
cg18372607	0.09964	0.452172432	2.182076	2.36E-06	hypermethylated
cg07682600	0.09651	0.437802162	2.181529	1.08E-05	hypermethylated
cg15126975	0.06783	0.307547568	2.180814	0.000178	hypermethylated
cg22510337	0.06766	0.306190811	2.178056	0.000245	hypermethylated
cg24039697	0.08271	0.374228108	2.177784	7.32E-06	hypermethylated
cg00515457	0.0761	0.344288649	2.17765	1.15E-05	hypermethylated
cg11739758	0.06043	0.273387027	2.177608	4.54E-06	hypermethylated
cg04865110	0.0653	0.295372973	2.177383	0.000193	hypermethylated
cg08086720	0.08743	0.395421081	2.177189	2.42E-06	hypermethylated
cg24846199	0.06893	0.311713514	2.177017	7.93E-05	hypermethylated
cg22920300	0.06495	0.293643784	2.176666	3.30E-06	hypermethylated
cg03120091	0.05495	0.24840973	2.17653	8.83E-06	hypermethylated
cg05929882	0.05468	0.247174054	2.176442	3.12E-06	hypermethylated
cg09316122	0.05714	0.258286486	2.176399	1.35E-05	hypermethylated
cg06610484	0.05774	0.260961081	2.176192	8.48E-06	hypermethylated

cg23420260	0.07877	0.356001081	2.176163	0.000306	hypermethylated
cg07536910	0.09162	0.413907568	2.175574	2.64E-06	hypermethylated
cg02647941	0.04823	0.217850811	2.175338	6.02E-07	hypermethylated
cg09754845	0.08765	0.395686486	2.174532	1.94E-05	hypermethylated
cg22152728	0.08125	0.366771892	2.174443	3.21E-06	hypermethylated
cg15711902	0.07472	0.337283243	2.174394	5.97E-06	hypermethylated
cg12612118	0.07209	0.325395135	2.174322	2.87E-06	hypermethylated
cg05619892	0.07861	0.354688108	2.173766	2.45E-05	hypermethylated
cg07792478	0.1087	0.490448649	2.17375	3.69E-06	hypermethylated
cg18428516	0.05173	0.233316757	2.173217	6.48E-06	hypermethylated
cg16015712	0.06571	0.296182703	2.172303	7.32E-06	hypermethylated
cg27393010	0.10643	0.479668649	2.172133	4.12E-06	hypermethylated
cg00904966	0.0851	0.383402703	2.171629	3.49E-05	hypermethylated
cg08814405	0.07028	0.316623784	2.171584	5.58E-06	hypermethylated
cg20058043	0.0661	0.297790811	2.171577	8.42E-05	hypermethylated
cg18724565	0.05444	0.244975676	2.1699	1.28E-05	hypermethylated
cg24632241	0.06357	0.285808649	2.168632	2.81E-05	hypermethylated
cg24398479	0.08462	0.380421622	2.168529	1.06E-05	hypermethylated
cg15778457	0.06376	0.286614054	2.168386	0.000256	hypermethylated
cg25266629	0.07658	0.343828649	2.16665	2.35E-05	hypermethylated
cg00177451	0.07964	0.357179459	2.165084	3.21E-06	hypermethylated
cg16727201	0.06433	0.288506486	2.16504	2.89E-05	hypermethylated
cg13163930	0.08426	0.377797297	2.164693	1.01E-06	hypermethylated
cg07696699	0.06088	0.272965405	2.164678	1.66E-05	hypermethylated
cg05534710	0.07067	0.31683027	2.16454	6.75E-06	hypermethylated
cg26848718	0.0526	0.23580973	2.164489	5.28E-06	hypermethylated
cg25640822	0.07266	0.325690811	2.16427	7.65E-05	hypermethylated
cg27058486	0.08077	0.362035135	2.164238	3.07E-05	hypermethylated
cg16317181	0.07075	0.317076216	2.164028	7.47E-05	hypermethylated
cg06425919	0.08534	0.382352432	2.163609	1.58E-05	hypermethylated
cg05437823	0.06101	0.273208649	2.162886	1.46E-05	hypermethylated
cg14835443	0.0605	0.27083027	2.162382	1.84E-05	hypermethylated
cg07215504	0.04723	0.211411351	2.162277	0.000157	hypermethylated
cg24060451	0.06186	0.27688	2.162182	9.82E-06	hypermethylated
cg27328797	0.0771	0.345016216	2.161861	6.15E-05	hypermethylated
cg01085837	0.05783	0.258779459	2.161833	8.48E-06	hypermethylated
cg07037371	0.04886	0.2186	2.161568	3.76E-05	hypermethylated
cg15054725	0.08352	0.373651351	2.161499	1.09E-05	hypermethylated
cg26894523	0.06745	0.301722703	2.161333	2.26E-06	hypermethylated
cg09467508	0.06964	0.311494595	2.161219	1.17E-05	hypermethylated
cg14985989	0.07208	0.322344324	2.160932	1.21E-05	hypermethylated
cg12810096	0.06532	0.292108649	2.160908	2.64E-05	hypermethylated
cg13703049	0.08198	0.366516216	2.160533	0.0017	hypermethylated
cg26664161	0.06403	0.286252432	2.160468	2.57E-05	hypermethylated
cg13252583	0.06447	0.288068108	2.15971	9.84E-05	hypermethylated
cg26354128	0.0895	0.399872432	2.15958	2.04E-05	hypermethylated
cg25360861	0.0667	0.298	2.159554	1.09E-05	hypermethylated
cg09914742	0.05301	0.236764324	2.159115	3.39E-06	hypermethylated
cg10416527	0.0726	0.323974595	2.157839	2.78E-05	hypermethylated
cg00175709	0.05523	0.246338919	2.157121	7.12E-06	hypermethylated
cg11171221	0.08554	0.381387027	2.156585	2.83E-06	hypermethylated
cg06779449	0.08297	0.369868649	2.156351	8.15E-06	hypermethylated
cg01995743	0.06947	0.309560541	2.15576	6.01E-05	hypermethylated
cg21573200	0.08825	0.393190811	2.155561	1.88E-06	hypermethylated
cg19127283	0.05884	0.262135135	2.155442	0.000374	hypermethylated
cg10624122	0.06805	0.303112973	2.155189	1.15E-05	hypermethylated
cg15215612	0.06299	0.280522162	2.15492	6.62E-05	hypermethylated
cg12403137	0.05139	0.228809189	2.154585	7.65E-05	hypermethylated

cg10038009	0.07172	0.319275135	2.154353	0.000172	hypermethylated
cg14493920	0.05321	0.236835135	2.154114	0.000165	hypermethylated
cg02463418	0.07192	0.319984865	2.153539	5.86E-05	hypermethylated
cg21266559	0.06038	0.268635676	2.153508	3.85E-06	hypermethylated
cg25306611	0.05542	0.246506486	2.153147	3.67E-05	hypermethylated
cg06374079	0.05778	0.256918378	2.152668	1.46E-05	hypermethylated
cg18276638	0.07548	0.335405946	2.151742	3.85E-05	hypermethylated
cg02631468	0.06666	0.296180541	2.151584	3.15E-05	hypermethylated
cg20061010	0.06312	0.280322162	2.150917	5.07E-06	hypermethylated
cg05137358	0.08022	0.356138378	2.150404	1.01E-05	hypermethylated
cg10931190	0.06852	0.30419027	2.150377	4.24E-06	hypermethylated
cg21534423	0.06537	0.290205405	2.150374	4.64E-05	hypermethylated
cg06374962	0.05636	0.25013027	2.149936	8.22E-05	hypermethylated
cg14270857	0.05001	0.221935135	2.14985	4.82E-05	hypermethylated
cg18322569	0.109	0.483592973	2.149465	2.57E-06	hypermethylated
cg00028935	0.07696	0.341182703	2.148364	2.29E-06	hypermethylated
cg24319902	0.07831	0.347117838	2.148157	8.60E-06	hypermethylated
cg09736669	0.05089	0.225536216	2.147905	6.22E-06	hypermethylated
cg22758454	0.0703	0.311395676	2.147152	2.21E-05	hypermethylated
cg18952796	0.11779	0.521748649	2.147138	3.79E-06	hypermethylated
cg00699993	0.05535	0.245109189	2.146769	5.72E-05	hypermethylated
cg16966315	0.05861	0.259331892	2.145581	7.52E-06	hypermethylated
cg01570424	0.07797	0.344958378	2.145431	1.82E-05	hypermethylated
cg22337128	0.05994	0.265114595	2.145025	1.04E-05	hypermethylated
cg22488797	0.09085	0.401791892	2.14489	2.26E-06	hypermethylated
cg18408326	0.06572	0.290579459	2.144528	1.88E-06	hypermethylated
cg02482218	0.07781	0.343994054	2.144356	1.33E-05	hypermethylated
cg07428323	0.05269	0.232911892	2.144183	4.36E-05	hypermethylated
cg25444386	0.09927	0.438735676	2.143922	4.73E-06	hypermethylated
cg19079845	0.05592	0.247032432	2.143264	1.94E-05	hypermethylated
cg15207742	0.0658	0.290637297	2.14306	0.000122	hypermethylated
cg24150623	0.09541	0.421387568	2.142935	6.84E-06	hypermethylated
cg00082664	0.07958	0.351364865	2.142492	1.28E-05	hypermethylated
cg09073398	0.07242	0.319642162	2.141998	1.39E-05	hypermethylated
cg09411719	0.05932	0.261791892	2.14183	3.96E-06	hypermethylated
cg23509896	0.05422	0.239241081	2.141568	0.000262	hypermethylated
cg11787346	0.06662	0.293952973	2.141558	4.88E-05	hypermethylated
cg24680320	0.05197	0.229256216	2.14121	1.44E-05	hypermethylated
cg18023096	0.06291	0.277305405	2.140114	6.05E-06	hypermethylated
cg26371061	0.06056	0.266707027	2.138819	7.12E-06	hypermethylated
cg12250896	0.07479	0.329263243	2.138324	0.000178	hypermethylated
cg27120816	0.07194	0.316657297	2.138056	1.26E-05	hypermethylated
cg02325540	0.05364	0.236058378	2.137763	1.51E-06	hypermethylated
cg24624901	0.08556	0.376478919	2.137561	2.79E-06	hypermethylated
cg10512745	0.08091	0.355921622	2.13717	0.000575	hypermethylated
cg02378847	0.07206	0.316865946	2.136602	3.85E-05	hypermethylated
cg18710462	0.04622	0.203212432	2.1364	3.12E-06	hypermethylated
cg00549566	0.08608	0.378455135	2.136372	2.10E-05	hypermethylated
cg18734428	0.0863	0.379005946	2.134788	2.95E-06	hypermethylated
cg11597131	0.07318	0.321315676	2.13447	5.00E-06	hypermethylated
cg19157034	0.07084	0.310863243	2.133644	5.45E-05	hypermethylated
cg02179652	0.05924	0.259948649	2.133583	1.11E-05	hypermethylated
cg26156448	0.05123	0.224707027	2.132984	1.06E-05	hypermethylated
cg02503874	0.0604	0.264916216	2.132916	5.65E-06	hypermethylated
cg24561661	0.0935	0.409985405	2.132534	1.45E-06	hypermethylated
cg27621340	0.07362	0.322703243	2.132038	3.85E-06	hypermethylated
cg25763393	0.07931	0.347358919	2.130852	5.28E-06	hypermethylated
cg21859781	0.06534	0.28612973	2.130631	0.000445	hypermethylated

cg07014673	0.10408	0.455628649	2.130166	5.89E-06	hypermethylated
cg19961480	0.0509	0.222821622	2.130152	3.54E-06	hypermethylated
cg23559689	0.08374	0.366531351	2.129948	1.50E-05	hypermethylated
cg04756279	0.05624	0.246047568	2.129269	9.69E-06	hypermethylated
cg03577655	0.07583	0.33175027	2.129257	2.27E-05	hypermethylated
cg23484951	0.06164	0.269608108	2.128925	5.81E-06	hypermethylated
cg06685968	0.07397	0.323471892	2.128628	1.99E-05	hypermethylated
cg23161999	0.08305	0.363087568	2.128265	5.28E-06	hypermethylated
cg08000065	0.06648	0.290416216	2.12713	2.89E-05	hypermethylated
cg00812833	0.08277	0.361465946	2.12668	2.21E-05	hypermethylated
cg26410450	0.06283	0.274335676	2.126417	0.000159	hypermethylated
cg27037551	0.06312	0.275551892	2.126155	4.26E-05	hypermethylated
cg05829782	0.06295	0.274808649	2.126149	1.09E-05	hypermethylated
cg03382304	0.05828	0.254341622	2.125695	3.11E-05	hypermethylated
cg12071888	0.08831	0.385297838	2.125325	1.33E-06	hypermethylated
cg00129651	0.06397	0.279076216	2.125192	0.00094	hypermethylated
cg13899017	0.08652	0.377224865	2.124319	5.58E-06	hypermethylated
cg12111758	0.05065	0.220826486	2.124279	0.000104	hypermethylated
cg00816224	0.07737	0.33731027	2.12423	3.44E-06	hypermethylated
cg03854198	0.0371	0.161738378	2.124171	4.73E-06	hypermethylated
cg04167903	0.06134	0.267412973	2.124169	9.72E-05	hypermethylated
cg04344565	0.07572	0.330067568	2.124015	0.000212	hypermethylated
cg06179765	0.05284	0.230294595	2.123778	1.15E-05	hypermethylated
cg10454766	0.05704	0.248565405	2.12358	1.52E-05	hypermethylated
cg26763877	0.07702	0.335629189	2.123563	5.97E-06	hypermethylated
cg24041541	0.05174	0.225311892	2.122572	2.71E-05	hypermethylated
cg08886973	0.06068	0.264154595	2.12209	2.04E-05	hypermethylated
cg11885396	0.06931	0.301566486	2.121341	8.84E-05	hypermethylated
cg22809871	0.08203	0.356896757	2.121283	2.48E-05	hypermethylated
cg02388378	0.06977	0.3034	2.120542	2.39E-05	hypermethylated
cg08612137	0.07086	0.308137838	2.120532	4.94E-05	hypermethylated
cg24061141	0.078	0.339125946	2.120275	1.82E-05	hypermethylated
cg11940285	0.10008	0.435103784	2.120206	2.95E-06	hypermethylated
cg02760031	0.07729	0.336004865	2.120128	6.93E-06	hypermethylated
cg01269620	0.07549	0.328063243	2.119617	2.15E-05	hypermethylated
cg21389753	0.06118	0.265844324	2.11945	3.23E-05	hypermethylated
cg21229570	0.07095	0.308139459	2.118709	1.66E-05	hypermethylated
cg05417127	0.08323	0.361399459	2.118419	0.000152	hypermethylated
cg04088969	0.06611	0.286922162	2.117719	5.31E-05	hypermethylated
cg23528705	0.10606	0.460108649	2.117094	1.15E-06	hypermethylated
cg19773855	0.0603	0.261583784	2.117043	3.30E-06	hypermethylated
cg20634442	0.07411	0.321427027	2.116751	3.36E-05	hypermethylated
cg13128937	0.05843	0.253341081	2.1163	3.62E-05	hypermethylated
cg19115393	0.06879	0.298222703	2.116119	6.46E-05	hypermethylated
cg08604097	0.07599	0.329402162	2.115969	1.09E-05	hypermethylated
cg26718878	0.07722	0.334730811	2.115955	5.50E-06	hypermethylated
cg06164660	0.05804	0.251544865	2.115696	1.12E-05	hypermethylated
cg01578017	0.08679	0.375876757	2.114659	2.96E-05	hypermethylated
cg26128079	0.05764	0.249562703	2.11426	1.05E-05	hypermethylated
cg15382538	0.07484	0.323874595	2.113554	5.14E-06	hypermethylated
cg24740218	0.04671	0.202052973	2.11293	3.67E-05	hypermethylated
cg10530851	0.07665	0.331562703	2.112924	8.13E-05	hypermethylated
cg02643054	0.07959	0.344271351	2.112887	1.77E-06	hypermethylated
cg13570101	0.0595	0.257280541	2.112381	1.25E-05	hypermethylated
cg12478381	0.07477	0.323073514	2.111331	1.42E-05	hypermethylated
cg26371320	0.11234	0.485347568	2.111147	3.21E-06	hypermethylated
cg13352750	0.08082	0.348864324	2.109882	3.76E-05	hypermethylated
cg24390913	0.06485	0.279743784	2.108928	3.44E-06	hypermethylated

cg00089798	0.07062	0.304581081	2.108678	1.66E-05	hypermethylated
cg19806642	0.10441	0.450087027	2.107944	4.73E-06	hypermethylated
cg21998983	0.06001	0.258684324	2.107918	1.33E-05	hypermethylated
cg04819499	0.07724	0.332932973	2.107812	2.57E-05	hypermethylated
cg13921352	0.06606	0.284718378	2.107687	4.05E-05	hypermethylated
cg04402007	0.09894	0.426341622	2.107384	1.25E-05	hypermethylated
cg24892510	0.05858	0.252263243	2.10645	4.36E-05	hypermethylated
cg14871414	0.05773	0.248539459	2.106082	0.001063	hypermethylated
cg15889913	0.05181	0.22301027	2.105808	1.44E-05	hypermethylated
cg12338417	0.09767	0.420357297	2.105629	2.71E-06	hypermethylated
cg08234308	0.07974	0.343103243	2.105267	0.000839	hypermethylated
cg22638505	0.08772	0.377427568	2.105222	1.72E-06	hypermethylated
cg07126525	0.08613	0.370415135	2.104555	2.29E-06	hypermethylated
cg21540621	0.06889	0.296266486	2.104529	1.11E-05	hypermethylated
cg21331821	0.06506	0.279741081	2.104249	3.30E-06	hypermethylated
cg07401230	0.06813	0.292834595	2.103724	3.81E-05	hypermethylated
cg22601348	0.07116	0.305758378	2.103254	7.83E-06	hypermethylated
cg06577205	0.09954	0.427667027	2.10314	9.44E-06	hypermethylated
cg10659805	0.10009	0.429990811	2.103008	9.11E-07	hypermethylated
cg25640519	0.06424	0.275785405	2.102002	4.01E-06	hypermethylated
cg16256390	0.07991	0.343052973	2.101983	1.26E-05	hypermethylated
cg25915838	0.08238	0.353615676	2.101816	1.77E-05	hypermethylated
cg23217097	0.06485	0.278362703	2.101787	9.19E-06	hypermethylated
cg17438849	0.0732	0.314148108	2.101529	7.93E-06	hypermethylated
cg27532847	0.05806	0.249030811	2.100708	4.00E-05	hypermethylated
cg11523290	0.05504	0.235964865	2.10002	1.37E-05	hypermethylated
cg15044957	0.08688	0.372302703	2.09938	1.68E-05	hypermethylated
cg26708319	0.06587	0.282231351	2.099185	2.57E-06	hypermethylated
cg12773394	0.07048	0.301837297	2.098485	1.49E-06	hypermethylated
cg10541864	0.08159	0.349194054	2.097565	0.000116	hypermethylated
cg03336086	0.06224	0.266331351	2.097308	1.63E-06	hypermethylated
cg17320707	0.0918	0.392788649	2.097187	7.32E-06	hypermethylated
cg24727311	0.07504	0.32106	2.097111	2.64E-05	hypermethylated
cg08490115	0.08307	0.355273514	2.096531	1.87E-05	hypermethylated
cg10741025	0.08798	0.376128108	2.095977	6.30E-06	hypermethylated
cg01924880	0.04892	0.209064324	2.095451	2.61E-05	hypermethylated
cg14251870	0.0632	0.269992432	2.094923	5.19E-05	hypermethylated
cg14780632	0.11316	0.483253514	2.094416	9.82E-06	hypermethylated
cg01408654	0.06765	0.28886973	2.094257	2.18E-05	hypermethylated
cg26059468	0.09535	0.407091351	2.094048	1.94E-05	hypermethylated
cg20311863	0.09072	0.387318919	2.094029	4.48E-06	hypermethylated
cg15180867	0.04942	0.210979459	2.093936	3.04E-05	hypermethylated
cg27154343	0.0833	0.355415676	2.093119	1.42E-05	hypermethylated
cg19237294	0.06641	0.283319459	2.092957	1.01E-05	hypermethylated
cg21627409	0.08876	0.378642162	2.092853	3.85E-06	hypermethylated
cg00397479	0.09478	0.404322162	2.092851	4.48E-06	hypermethylated
cg21001487	0.07542	0.321701622	2.092704	7.32E-06	hypermethylated
cg05292954	0.06951	0.296272973	2.091635	2.78E-05	hypermethylated
cg19084726	0.06375	0.271581622	2.090889	0.000104	hypermethylated
cg22060073	0.05248	0.223441622	2.090058	9.07E-06	hypermethylated
cg05415020	0.07617	0.32422973	2.089722	0.000469	hypermethylated
cg01942962	0.07534	0.320694595	2.089712	1.25E-05	hypermethylated
cg14200170	0.07759	0.330225946	2.089511	8.60E-06	hypermethylated
cg24224020	0.07941	0.337964324	2.089478	1.39E-05	hypermethylated
cg01836687	0.08678	0.369327027	2.089464	6.14E-06	hypermethylated
cg14559259	0.08003	0.340583243	2.089395	3.59E-06	hypermethylated
cg26249873	0.04912	0.209027027	2.089307	6.46E-05	hypermethylated
cg23500122	0.062	0.26382973	2.089267	2.78E-05	hypermethylated

cg15775138	0.06907	0.293702162	2.088223	0.001556	hypermethylated
cg11310950	0.05733	0.243685946	2.087661	1.21E-05	hypermethylated
cg05502701	0.05267	0.223877297	2.087655	5.72E-05	hypermethylated
cg04562217	0.07284	0.309556757	2.087401	1.15E-05	hypermethylated
cg19607165	0.06185	0.262804865	2.087146	2.10E-06	hypermethylated
cg05506365	0.07203	0.305842162	2.086117	8.15E-06	hypermethylated
cg06469345	0.07767	0.329649189	2.085502	5.43E-06	hypermethylated
cg23316253	0.079	0.335290811	2.085488	6.14E-06	hypermethylated
cg26332560	0.07785	0.330337838	2.085173	1.37E-05	hypermethylated
cg19267861	0.08903	0.377720541	2.084956	5.81E-06	hypermethylated
cg01615424	0.07918	0.335838378	2.084559	1.18E-05	hypermethylated
cg24415208	0.10751	0.455917838	2.084303	2.79E-06	hypermethylated
cg07565505	0.11159	0.473215135	2.084288	1.47E-06	hypermethylated
cg22660578	0.08711	0.369271351	2.083771	5.06E-05	hypermethylated
cg15634398	0.0523	0.221631892	2.083283	4.36E-05	hypermethylated
cg22235018	0.07569	0.320716216	2.083123	9.19E-06	hypermethylated
cg11823511	0.10154	0.430224324	2.083041	1.72E-06	hypermethylated
cg07152216	0.09649	0.408704324	2.082606	3.69E-06	hypermethylated
cg04541474	0.08629	0.365475135	2.082508	3.26E-06	hypermethylated
cg16696270	0.05416	0.229363243	2.082335	6.39E-06	hypermethylated
cg07557260	0.0806	0.341332973	2.082328	1.15E-05	hypermethylated
cg24328125	0.05632	0.238338378	2.081292	4.53E-05	hypermethylated
cg18177414	0.10249	0.433662703	2.08109	1.28E-05	hypermethylated
cg11372090	0.08268	0.349648108	2.080293	0.000123	hypermethylated
cg06724588	0.08785	0.371478919	2.080166	6.57E-06	hypermethylated
cg18415687	0.07565	0.319862703	2.080041	1.15E-05	hypermethylated
cg18323912	0.07094	0.299935135	2.079979	0.000119	hypermethylated
cg21530453	0.06148	0.259827027	2.079362	0.000287	hypermethylated
cg15852891	0.05177	0.218775676	2.079264	1.54E-05	hypermethylated
cg15092168	0.07227	0.305393514	2.079201	3.15E-05	hypermethylated
cg21549195	0.08001	0.338061622	2.079034	1.06E-05	hypermethylated
cg07379434	0.05444	0.229984324	2.078797	1.31E-05	hypermethylated
cg08862035	0.07286	0.307777838	2.07869	4.36E-05	hypermethylated
cg12393318	0.08496	0.35877027	2.078205	4.24E-06	hypermethylated
cg11850773	0.08011	0.338187027	2.077767	8.60E-06	hypermethylated
cg19650157	0.0726	0.306458919	2.077652	7.12E-06	hypermethylated
cg10703826	0.10059	0.424497838	2.07727	2.05E-06	hypermethylated
cg05604079	0.07998	0.337480541	2.077093	7.03E-05	hypermethylated
cg21195256	0.10692	0.45110973	2.076947	2.64E-06	hypermethylated
cg17352975	0.0671	0.283089189	2.076872	2.87E-06	hypermethylated
cg07962143	0.10798	0.455375135	2.076291	5.14E-06	hypermethylated
cg15985191	0.07687	0.324135676	2.076105	2.71E-05	hypermethylated
cg11601523	0.07269	0.306505946	2.076086	3.79E-06	hypermethylated
cg25916711	0.07544	0.317944324	2.075373	6.39E-06	hypermethylated
cg04908789	0.05395	0.227294054	2.074865	3.62E-05	hypermethylated
cg17774559	0.12738	0.536657838	2.074864	7.32E-06	hypermethylated
cg24621437	0.08861	0.373270811	2.074681	2.64E-05	hypermethylated
cg07746943	0.07708	0.324511351	2.07384	0.000287	hypermethylated
cg21347353	0.06505	0.273665946	2.072795	2.39E-06	hypermethylated
cg27296293	0.08578	0.360771892	2.072374	4.60E-06	hypermethylated
cg21126583	0.08276	0.347838919	2.071414	0.000115	hypermethylated
cg04733537	0.07204	0.302723784	2.071132	1.01E-05	hypermethylated
cg17924936	0.0945	0.396985405	2.0707	2.78E-05	hypermethylated
cg23676682	0.06026	0.25313027	2.070607	6.13E-06	hypermethylated
cg11120927	0.07372	0.30956	2.070091	0.00291	hypermethylated
cg02228111	0.07513	0.315425946	2.06984	3.04E-05	hypermethylated
cg02610222	0.0632	0.265331351	2.069799	8.95E-06	hypermethylated
cg02637318	0.09216	0.38682	2.06945	0.000113	hypermethylated

cg04389897	0.09048	0.379734595	2.069321	1.44E-05	hypermethylated
cg20631014	0.05238	0.219806486	2.069146	0.000129	hypermethylated
cg04276723	0.05631	0.236231892	2.068741	4.12E-06	hypermethylated
cg00040312	0.07562	0.317214054	2.068617	2.18E-05	hypermethylated
cg15140703	0.07476	0.313576757	2.06848	1.75E-05	hypermethylated
cg24848035	0.08771	0.367711892	2.067763	1.89E-05	hypermethylated
cg00483562	0.07755	0.325076757	2.067582	1.54E-06	hypermethylated
cg05495351	0.07353	0.308215676	2.067535	9.57E-06	hypermethylated
cg15854847	0.08328	0.34906973	2.067473	1.42E-05	hypermethylated
cg19594305	0.08573	0.359261081	2.067161	2.78E-05	hypermethylated
cg22793136	0.06499	0.272341622	2.067128	4.26E-05	hypermethylated
cg07498624	0.0479	0.200725405	2.067126	7.03E-06	hypermethylated
cg07489502	0.07697	0.322533514	2.067081	5.89E-06	hypermethylated
cg00355281	0.0813	0.340647568	2.066953	2.51E-05	hypermethylated
cg16291880	0.06164	0.258248108	2.066819	8.60E-06	hypermethylated
cg10762626	0.08728	0.365631351	2.066667	1.25E-05	hypermethylated
cg05769349	0.06707	0.280964324	2.066647	5.43E-06	hypermethylated
cg01792403	0.06988	0.292593514	2.065946	2.45E-05	hypermethylated
cg03843000	0.07682	0.321589189	2.065665	3.21E-06	hypermethylated
cg02268225	0.06444	0.269674595	2.065191	0.001176	hypermethylated
cg06660530	0.06326	0.264521622	2.06402	7.22E-06	hypermethylated
cg08304190	0.0917	0.383306486	2.063505	2.87E-06	hypermethylated
cg26123605	0.07501	0.313510811	2.06336	2.36E-06	hypermethylated
cg24032666	0.11383	0.475702703	2.063179	9.69E-06	hypermethylated
cg23363202	0.06209	0.259473514	2.063154	8.60E-06	hypermethylated
cg10730712	0.0857	0.358031351	2.062719	1.21E-05	hypermethylated
cg02315940	0.07057	0.294808108	2.062649	6.38E-05	hypermethylated
cg21127068	0.06227	0.259996757	2.061884	5.06E-05	hypermethylated
cg26843074	0.07388	0.308220541	2.060707	9.27E-05	hypermethylated
cg16138150	0.06188	0.258117297	2.060482	6.57E-06	hypermethylated
cg09221867	0.10094	0.420998378	2.060317	1.90E-06	hypermethylated
cg08487063	0.05012	0.209030811	2.060257	2.51E-05	hypermethylated
cg15408073	0.06373	0.265784324	2.060211	1.39E-05	hypermethylated
cg01718742	0.08446	0.352225946	2.060161	7.22E-06	hypermethylated
cg20855565	0.0663	0.276456757	2.059973	2.49E-06	hypermethylated
cg10253847	0.05382	0.224352973	2.059556	2.96E-05	hypermethylated
cg05347927	0.07824	0.326114054	2.059398	3.07E-05	hypermethylated
cg09686443	0.08333	0.347258919	2.059104	3.15E-05	hypermethylated
cg24127719	0.09682	0.403427027	2.058931	2.36E-06	hypermethylated
cg08013557	0.08382	0.349169189	2.05856	1.12E-05	hypermethylated
cg01500945	0.09431	0.392784865	2.058257	7.42E-06	hypermethylated
cg03078269	0.12287	0.511715135	2.058208	4.24E-06	hypermethylated
cg27271486	0.074	0.308145946	2.058017	1.75E-05	hypermethylated
cg16619425	0.08533	0.355322703	2.058005	7.83E-06	hypermethylated
cg03983645	0.08214	0.341972432	2.057723	1.39E-05	hypermethylated
cg21649258	0.07634	0.317717838	2.057235	0.000202	hypermethylated
cg10397440	0.04716	0.196238378	2.056972	1.05E-05	hypermethylated
cg19923650	0.09568	0.397680541	2.055321	4.24E-06	hypermethylated
cg03036592	0.06895	0.28652	2.055013	6.05E-06	hypermethylated
cg15759937	0.06825	0.283597297	2.054943	9.72E-05	hypermethylated
cg02428119	0.08993	0.373677838	2.054921	3.64E-06	hypermethylated
cg19870698	0.06302	0.261829189	2.054744	7.56E-05	hypermethylated
cg19414591	0.06293	0.261403784	2.05446	1.15E-06	hypermethylated
cg20059140	0.05792	0.240544865	2.054172	3.69E-06	hypermethylated
cg16509851	0.09123	0.37874	2.053628	5.43E-06	hypermethylated
cg20755170	0.07317	0.303543784	2.05258	8.60E-06	hypermethylated
cg10698355	0.04849	0.201140541	2.052445	4.12E-06	hypermethylated
cg15922174	0.08263	0.342752973	2.052432	0.000187	hypermethylated

cg26115633	0.12391	0.513952973	2.052344	2.68E-06	hypermethylated
cg16589299	0.07242	0.300378378	2.052321	6.57E-06	hypermethylated
cg04047221	0.07428	0.308056757	2.05215	4.12E-06	hypermethylated
cg05893614	0.06057	0.251178378	2.052037	2.96E-05	hypermethylated
cg08711858	0.08202	0.339952973	2.051288	8.03E-05	hypermethylated
cg22849427	0.06019	0.249431351	2.051047	4.80E-06	hypermethylated
cg17816908	0.08361	0.346472432	2.050993	1.89E-05	hypermethylated
cg24437715	0.06264	0.259416216	2.050113	0.000185	hypermethylated
cg19091036	0.07975	0.330091892	2.049311	1.18E-05	hypermethylated
cg16104915	0.06344	0.262556216	2.049162	3.81E-05	hypermethylated
cg27376707	0.09234	0.382121081	2.049002	5.00E-06	hypermethylated
cg16230141	0.06262	0.259069189	2.048642	0.000165	hypermethylated
cg27019126	0.05585	0.230995676	2.048237	3.85E-05	hypermethylated
cg08528231	0.06419	0.265366486	2.047566	9.96E-06	hypermethylated
cg18961681	0.11646	0.481423243	2.047471	6.22E-06	hypermethylated
cg22523852	0.0609	0.25164973	2.046903	0.000166	hypermethylated
cg23432345	0.06375	0.263425405	2.046897	3.39E-06	hypermethylated
cg00814733	0.06497	0.268418919	2.046641	5.86E-05	hypermethylated
cg25377802	0.06179	0.255098919	2.045612	1.66E-05	hypermethylated
cg15564098	0.11457	0.472912973	2.045345	7.12E-06	hypermethylated
cg04154276	0.0585	0.241402162	2.04493	7.72E-06	hypermethylated
cg09786701	0.07185	0.296145405	2.043246	1.21E-05	hypermethylated
cg04894619	0.07613	0.313665946	2.042692	2.78E-05	hypermethylated
cg03574723	0.08562	0.352721081	2.042508	3.44E-06	hypermethylated
cg11965976	0.09011	0.371206486	2.042463	0.000111	hypermethylated
cg12258042	0.08871	0.365310811	2.041956	2.04E-05	hypermethylated
cg22749589	0.0695	0.286084324	2.041356	5.25E-05	hypermethylated
cg23890008	0.04845	0.199356757	2.040784	1.09E-05	hypermethylated
cg18894648	0.07915	0.325668649	2.040744	7.32E-06	hypermethylated
cg14776201	0.07753	0.318934595	2.040434	2.38E-05	hypermethylated
cg07849944	0.07947	0.326884865	2.0403	2.10E-05	hypermethylated
cg13721404	0.07519	0.309231892	2.040076	5.79E-05	hypermethylated
cg24496841	0.09273	0.38128	2.039743	1.94E-05	hypermethylated
cg04666975	0.05572	0.229051892	2.039407	1.29E-06	hypermethylated
cg15916399	0.08056	0.331026486	2.038811	1.62E-05	hypermethylated
cg16027761	0.08968	0.36846	2.03865	6.22E-06	hypermethylated
cg01739725	0.08171	0.335700541	2.03859	7.03E-06	hypermethylated
cg12581627	0.08196	0.33668973	2.038428	3.76E-05	hypermethylated
cg19591056	0.06875	0.282333514	2.037969	3.40E-05	hypermethylated
cg09410234	0.0778	0.319415676	2.037593	3.26E-06	hypermethylated
cg14573411	0.06856	0.281434054	2.037358	0.000137	hypermethylated
cg13663793	0.06863	0.281595135	2.036711	6.01E-05	hypermethylated
cg03156893	0.09227	0.378576757	2.036652	1.71E-05	hypermethylated
cg15282819	0.07135	0.292658378	2.036232	9.69E-06	hypermethylated
cg25254825	0.049	0.200962703	2.036074	1.54E-05	hypermethylated
cg12836959	0.08616	0.353215676	2.035459	6.57E-06	hypermethylated
cg02656891	0.09827	0.402763243	2.035109	9.07E-06	hypermethylated
cg15219228	0.09664	0.396028108	2.03491	1.94E-05	hypermethylated
cg00956199	0.09135	0.374243243	2.0345	6.22E-06	hypermethylated
cg18325622	0.07605	0.311491351	2.034172	1.94E-05	hypermethylated
cg15475851	0.05926	0.242536216	2.03307	6.31E-05	hypermethylated
cg14658067	0.09325	0.38163027	2.033	8.60E-06	hypermethylated
cg16330450	0.074	0.302846486	2.032989	7.32E-06	hypermethylated
cg04425920	0.11129	0.455422162	2.032881	4.60E-07	hypermethylated
cg23082454	0.0641	0.262218378	2.032373	9.49E-05	hypermethylated
cg04398581	0.07722	0.315657297	2.031313	3.39E-06	hypermethylated
cg00294096	0.05757	0.235228108	2.030671	2.10E-05	hypermethylated
cg17768491	0.08189	0.334463784	2.030091	0.0002	hypermethylated

cg18146843	0.0741	0.302637838	2.030047	5.00E-05	hypermethylated
cg00152008	0.08025	0.327725405	2.029914	6.57E-06	hypermethylated
cg05547777	0.0758	0.309484865	2.029599	3.30E-06	hypermethylated
cg24280540	0.06924	0.282684865	2.029517	0.000124	hypermethylated
cg17802600	0.08917	0.364012432	2.029357	3.11E-05	hypermethylated
cg07857243	0.06761	0.275953514	2.029117	1.31E-05	hypermethylated
cg17147045	0.07035	0.287035676	2.028608	0.000216	hypermethylated
cg27615388	0.09377	0.382552432	2.028459	3.79E-06	hypermethylated
cg19081437	0.05116	0.208643243	2.02795	0.00044	hypermethylated
cg05295557	0.08056	0.328515135	2.027824	0.000703	hypermethylated
cg02909136	0.08445	0.344302162	2.027506	3.40E-05	hypermethylated
cg00926215	0.06856	0.279382162	2.026801	1.04E-05	hypermethylated
cg03323696	0.07724	0.314652973	2.026342	9.57E-06	hypermethylated
cg10126205	0.10804	0.43994	2.025741	2.57E-06	hypermethylated
cg12973118	0.05192	0.211345946	2.025244	4.64E-05	hypermethylated
cg20289688	0.08519	0.346756757	2.025168	1.14E-05	hypermethylated
cg03002848	0.07101	0.289005405	2.025002	5.06E-05	hypermethylated
cg23883696	0.09659	0.393023784	2.024671	3.49E-06	hypermethylated
cg20464804	0.06915	0.281284324	2.024228	3.95E-05	hypermethylated
cg09735723	0.0691	0.281068649	2.024165	7.93E-06	hypermethylated
cg24708471	0.1043	0.424154595	2.023851	8.83E-06	hypermethylated
cg15353031	0.06915	0.281185405	2.023721	8.83E-06	hypermethylated
cg07198552	0.09486	0.385711892	2.023652	1.82E-06	hypermethylated
cg06639332	0.07284	0.296129189	2.023424	2.15E-05	hypermethylated
cg15384598	0.0782	0.317897838	2.023323	4.47E-05	hypermethylated
cg14123942	0.07321	0.297578378	2.023157	6.78E-05	hypermethylated
cg12091642	0.07588	0.308424324	2.023125	4.26E-05	hypermethylated
cg20383155	0.06696	0.272152973	2.023046	2.10E-05	hypermethylated
cg10877086	0.05045	0.204888108	2.02191	4.93E-06	hypermethylated
cg05660670	0.05611	0.227788108	2.021363	7.56E-05	hypermethylated
cg01108118	0.06188	0.251142703	2.020962	2.51E-05	hypermethylated
cg07339783	0.06402	0.259741622	2.020483	2.27E-05	hypermethylated
cg03781266	0.07108	0.288322162	2.020166	7.72E-06	hypermethylated
cg11142705	0.0714	0.289311892	2.01863	6.93E-06	hypermethylated
cg10216615	0.07901	0.320067568	2.018269	5.89E-06	hypermethylated
cg26157386	0.07547	0.305589189	2.017618	1.46E-05	hypermethylated
cg03289872	0.09972	0.403610811	2.01701	7.12E-06	hypermethylated
cg00027083	0.08229	0.333027027	2.01685	3.85E-05	hypermethylated
cg27125044	0.06276	0.253934595	2.01654	7.52E-06	hypermethylated
cg16407471	0.09127	0.369272973	2.016475	5.43E-06	hypermethylated
cg06256858	0.05686	0.230048649	2.016453	8.84E-05	hypermethylated
cg24129977	0.08962	0.362583784	2.016422	3.04E-06	hypermethylated
cg14590098	0.06728	0.272139459	2.016097	5.00E-06	hypermethylated
cg08744177	0.0696	0.281508108	2.016017	4.05E-05	hypermethylated
cg07847863	0.09294	0.375755135	2.015421	2.10E-06	hypermethylated
cg05161082	0.07664	0.309756757	2.014966	1.25E-05	hypermethylated
cg26337312	0.0552	0.223094054	2.014912	2.96E-05	hypermethylated
cg17898329	0.07072	0.285807027	2.014851	5.06E-05	hypermethylated
cg12436377	0.08489	0.343016757	2.014613	5.86E-05	hypermethylated
cg18363192	0.07653	0.309028108	2.013641	3.40E-05	hypermethylated
cg12744820	0.09527	0.384495676	2.012873	4.24E-06	hypermethylated
cg12786548	0.06097	0.245927027	2.012059	1.25E-05	hypermethylated
cg10776919	0.0613	0.247247568	2.011997	1.30E-05	hypermethylated
cg17255450	0.08851	0.356867027	2.011474	1.42E-05	hypermethylated
cg08512142	0.06301	0.253874054	2.01046	1.80E-05	hypermethylated
cg09907936	0.11473	0.462180541	2.010214	2.42E-05	hypermethylated
cg25724283	0.07433	0.299408108	2.010097	0.000122	hypermethylated
cg03751030	0.06713	0.270366486	2.009887	4.73E-06	hypermethylated

cg12420104	0.06813	0.274387027	2.00985	2.15E-05	hypermethylated
cg07039180	0.07128	0.287032432	2.009645	6.22E-06	hypermethylated
cg16370491	0.07737	0.311554054	2.009636	7.72E-06	hypermethylated
cg14118515	0.09487	0.381922703	2.009257	7.83E-06	hypermethylated
cg10290764	0.05544	0.223134054	2.008912	3.32E-05	hypermethylated
cg01436998	0.07687	0.309355676	2.008774	7.72E-06	hypermethylated
cg04207385	0.06628	0.266728108	2.008724	2.32E-05	hypermethylated
cg23712342	0.04889	0.196745405	2.008719	2.85E-05	hypermethylated
cg02743222	0.08865	0.35668973	2.008477	4.01E-06	hypermethylated
cg07150669	0.06228	0.250558378	2.008306	7.72E-06	hypermethylated
cg08497530	0.06427	0.258481622	2.007844	3.76E-05	hypermethylated
cg18406033	0.09546	0.383826486	2.007486	8.15E-06	hypermethylated
cg21045464	0.08106	0.325924865	2.007477	8.04E-06	hypermethylated
cg02182795	0.07183	0.288787027	2.007348	3.76E-05	hypermethylated
cg26466587	0.09317	0.374188649	2.005828	1.09E-05	hypermethylated
cg16880856	0.05934	0.238318919	2.005817	5.73E-06	hypermethylated
cg21319323	0.06247	0.250861081	2.005653	7.20E-05	hypermethylated
cg06906472	0.10254	0.411738919	2.005543	1.25E-05	hypermethylated
cg07442479	0.07816	0.313712973	2.004943	2.27E-05	hypermethylated
cg06550340	0.06268	0.251561622	2.004835	1.67E-06	hypermethylated
cg13482308	0.08361	0.335424324	2.00424	3.23E-05	hypermethylated
cg05649108	0.07952	0.318960541	2.003988	8.63E-05	hypermethylated
cg01975483	0.08262	0.331394595	2.003987	3.81E-05	hypermethylated
cg22685966	0.08549	0.342675135	2.003014	6.93E-06	hypermethylated
cg11119746	0.07988	0.320158919	2.002882	4.94E-05	hypermethylated
cg18921980	0.07016	0.281107568	2.002402	4.47E-05	hypermethylated
cg16318271	0.07807	0.312745405	2.002149	2.71E-06	hypermethylated
cg12860391	0.0775	0.310134054	2.000624	6.57E-06	hypermethylated
cg11531579	0.05093	0.203741081	2.000149	1.71E-05	hypermethylated
cg05835105	0.12098	0.483871351	1.999855	3.39E-06	hypermethylated
cg15959715	0.0874	0.34952973	1.99971	0.000155	hypermethylated
cg16420199	0.07209	0.288293514	1.999667	0.000135	hypermethylated
cg22702328	0.08626	0.344865946	1.999272	2.10E-05	hypermethylated
cg23331421	0.10526	0.420637838	1.998621	5.50E-06	hypermethylated
cg12751432	0.08735	0.349057838	1.998586	1.05E-05	hypermethylated
cg02721665	0.08192	0.327278919	1.998233	9.07E-06	hypermethylated
cg24408656	0.06647	0.265532973	1.998116	5.06E-05	hypermethylated
cg08156066	0.07156	0.285783243	1.997696	8.83E-06	hypermethylated
cg16137995	0.05067	0.202276757	1.997127	2.96E-05	hypermethylated
cg27264249	0.06559	0.261828108	1.997072	3.49E-06	hypermethylated
cg22029275	0.08945	0.357047027	1.996961	7.65E-05	hypermethylated
cg16865446	0.10214	0.407461081	1.996114	1.71E-05	hypermethylated
cg23130131	0.0885	0.353046486	1.996109	6.84E-06	hypermethylated
cg03057083	0.08875	0.354035135	1.996074	5.06E-05	hypermethylated
cg16927040	0.09227	0.367915676	1.995442	5.58E-06	hypermethylated
cg08330224	0.07346	0.292764865	1.994712	4.05E-05	hypermethylated
cg14391419	0.10077	0.401425946	1.994068	7.12E-06	hypermethylated
cg12776966	0.08605	0.342629189	1.993401	9.07E-06	hypermethylated
cg14445507	0.08582	0.341710811	1.99339	3.85E-05	hypermethylated
cg03496122	0.08018	0.319246486	1.993356	6.31E-05	hypermethylated
cg01501009	0.07178	0.285789189	1.993298	2.16E-06	hypermethylated
cg11891579	0.08434	0.335707027	1.992914	0.000789	hypermethylated
cg20354141	0.07079	0.281764865	1.992874	2.13E-05	hypermethylated
cg15091407	0.07076	0.281603784	1.992661	2.71E-06	hypermethylated
cg26339504	0.0561	0.223210811	1.992334	4.54E-06	hypermethylated
cg11205072	0.09804	0.390027568	1.992134	8.60E-06	hypermethylated
cg20405893	0.07432	0.295591892	1.991784	8.60E-06	hypermethylated
cg09792881	0.07884	0.313516216	1.99154	0.000921	hypermethylated

cg14349667	0.08836	0.351184324	1.990763	2.95E-06	hypermethylated
cg01559663	0.08313	0.330372432	1.990652	7.32E-06	hypermethylated
cg18382893	0.08191	0.325507568	1.99058	1.22E-06	hypermethylated
cg01256089	0.05936	0.235845405	1.990278	9.82E-06	hypermethylated
cg03200166	0.10144	0.402967568	1.990037	1.99E-05	hypermethylated
cg18448949	0.09032	0.358777838	1.989973	8.60E-06	hypermethylated
cg12570712	0.08863	0.352040541	1.989875	1.46E-05	hypermethylated
cg24909548	0.09071	0.360300541	1.989867	4.60E-06	hypermethylated
cg14451382	0.11569	0.459443243	1.989622	2.29E-06	hypermethylated
cg06151165	0.08435	0.334942703	1.989454	4.26E-05	hypermethylated
cg11630404	0.08186	0.325005946	1.989236	9.44E-06	hypermethylated
cg03444245	0.06812	0.270316216	1.988498	5.06E-05	hypermethylated
cg02621130	0.06634	0.263181081	1.988105	6.22E-06	hypermethylated
cg16033053	0.07287	0.288983784	1.987592	0.000756	hypermethylated
cg19885761	0.10406	0.412634054	1.987447	2.64E-05	hypermethylated
cg04150495	0.10037	0.397954595	1.987276	5.89E-06	hypermethylated
cg01981601	0.06727	0.26661027	1.986697	2.57E-05	hypermethylated
cg04545136	0.06085	0.241145405	1.986574	4.70E-05	hypermethylated
cg01382137	0.08767	0.347384865	1.98638	3.04E-06	hypermethylated
cg17757602	0.09287	0.367868108	1.985904	8.83E-06	hypermethylated
cg07924874	0.06026	0.238648649	1.985616	0.000403	hypermethylated
cg09359114	0.11188	0.44290973	1.985061	2.57E-06	hypermethylated
cg20286200	0.08611	0.340848108	1.984876	3.19E-05	hypermethylated
cg09747169	0.05484	0.217036757	1.984639	8.13E-05	hypermethylated
cg24723331	0.07323	0.28972	1.984153	1.12E-05	hypermethylated
cg22721334	0.08105	0.320639459	1.984068	0.000412	hypermethylated
cg05854644	0.059	0.233405405	1.984051	0.000277	hypermethylated
cg20337384	0.09076	0.358952432	1.983664	6.39E-06	hypermethylated
cg18435449	0.08335	0.329604865	1.983483	1.92E-05	hypermethylated
cg09354309	0.06963	0.275337838	1.983422	7.29E-05	hypermethylated
cg17800654	0.10885	0.430401081	1.98334	5.67E-07	hypermethylated
cg14676825	0.0833	0.329321081	1.983106	0.000303	hypermethylated
cg06940614	0.08607	0.340261081	1.98306	4.36E-06	hypermethylated
cg11657808	0.09844	0.389144324	1.982989	6.57E-06	hypermethylated
cg13940693	0.07453	0.29457027	1.982719	1.09E-05	hypermethylated
cg18416576	0.07874	0.310981081	1.981658	4.47E-05	hypermethylated
cg00252282	0.07981	0.315171351	1.981495	1.25E-05	hypermethylated
cg09793121	0.10913	0.430868649	1.9812	2.05E-06	hypermethylated
cg14614901	0.06137	0.242298919	1.981182	8.95E-06	hypermethylated
cg11831238	0.07528	0.297148649	1.980846	6.57E-06	hypermethylated
cg16664405	0.11257	0.444323784	1.980789	9.57E-06	hypermethylated
cg12618988	0.04412	0.174139459	1.980738	3.59E-06	hypermethylated
cg16476975	0.09548	0.376791351	1.980495	0.000399	hypermethylated
cg23906459	0.06599	0.260408649	1.980458	7.84E-05	hypermethylated
cg05855917	0.08545	0.336976216	1.979494	9.82E-06	hypermethylated
cg18863595	0.11145	0.439502162	1.979474	8.37E-06	hypermethylated
cg16922279	0.08097	0.319143243	1.978745	6.84E-06	hypermethylated
cg12238343	0.07744	0.305155676	1.978395	2.61E-05	hypermethylated
cg05080154	0.08518	0.335629189	1.978282	5.89E-06	hypermethylated
cg01718116	0.07535	0.296853514	1.978072	0.0002	hypermethylated
cg12847373	0.067	0.263916757	1.97785	1.87E-05	hypermethylated
cg27344859	0.06826	0.268849189	1.977685	4.76E-05	hypermethylated
cg19290410	0.07364	0.289970811	1.977346	4.48E-06	hypermethylated
cg21851351	0.07644	0.300950811	1.977128	2.74E-05	hypermethylated
cg03365311	0.06991	0.275151351	1.976655	7.12E-06	hypermethylated
cg19665362	0.08534	0.335745405	1.976074	0.000274	hypermethylated
cg22108469	0.08668	0.340991351	1.975964	5.28E-06	hypermethylated
cg17138769	0.0914	0.359537297	1.975875	8.60E-06	hypermethylated

cg05542338	0.10696	0.420587027	1.975333	2.39E-05	hypermethylated
cg13273396	0.08991	0.353397838	1.97474	5.97E-06	hypermethylated
cg05949020	0.06238	0.245142162	1.974463	1.67E-06	hypermethylated
cg14510333	0.05731	0.225021622	1.973205	4.70E-05	hypermethylated
cg16956426	0.05574	0.218734054	1.972393	0.000335	hypermethylated
cg24669528	0.05969	0.23418	1.972057	1.58E-05	hypermethylated
cg24213719	0.0821	0.322098919	1.97205	9.49E-05	hypermethylated
cg11413039	0.07903	0.31004973	1.972027	8.15E-06	hypermethylated
cg25740565	0.09775	0.383430811	1.971798	2.57E-05	hypermethylated
cg21852408	0.09136	0.358281081	1.971457	1.94E-05	hypermethylated
cg02380531	0.07278	0.285233514	1.97053	3.07E-05	hypermethylated
cg04025150	0.08967	0.351292432	1.969975	1.77E-05	hypermethylated
cg10989897	0.08799	0.344617838	1.969586	9.07E-06	hypermethylated
cg22282405	0.10127	0.396623784	1.969564	4.48E-06	hypermethylated
cg27663938	0.07861	0.307845946	1.969424	2.74E-05	hypermethylated
cg00076307	0.06792	0.265973514	1.969374	3.69E-06	hypermethylated
cg00257047	0.06248	0.244662162	1.969325	1.75E-05	hypermethylated
cg08315174	0.08736	0.341818919	1.968187	2.56E-06	hypermethylated
cg03858756	0.07269	0.2844	1.968093	1.15E-05	hypermethylated
cg03202804	0.07496	0.293266486	1.968019	1.42E-05	hypermethylated
cg22614239	0.07826	0.306137297	1.967832	2.79E-06	hypermethylated
cg17384380	0.06714	0.262618919	1.967726	3.96E-06	hypermethylated
cg07349208	0.08136	0.318152973	1.967329	7.93E-06	hypermethylated
cg05167251	0.10147	0.396765405	1.967233	4.48E-06	hypermethylated
cg16587616	0.10719	0.419036216	1.966905	1.28E-05	hypermethylated
cg02657401	0.12406	0.48482	1.966411	4.30E-06	hypermethylated
cg24791025	0.05469	0.213721081	1.96638	9.82E-06	hypermethylated
cg11294513	0.1115	0.435622703	1.966035	3.39E-06	hypermethylated
cg15221604	0.09212	0.359765946	1.965472	6.62E-05	hypermethylated
cg24101492	0.08608	0.336094595	1.965117	8.32E-05	hypermethylated
cg12544951	0.0684	0.266967568	1.964596	8.37E-06	hypermethylated
cg11689813	0.07895	0.308015676	1.963993	8.52E-05	hypermethylated
cg09685182	0.08045	0.313857297	1.963944	1.50E-05	hypermethylated
cg12993163	0.06772	0.264158919	1.963752	5.72E-05	hypermethylated
cg21800232	0.06329	0.246858378	1.963634	0.000204	hypermethylated
cg11213690	0.08995	0.350693514	1.963016	1.58E-05	hypermethylated
cg08084616	0.08071	0.314654595	1.96295	2.48E-05	hypermethylated
cg25898550	0.06655	0.259388649	1.962605	1.28E-05	hypermethylated
cg02009585	0.07918	0.308602703	1.962543	3.39E-06	hypermethylated
cg15585794	0.08074	0.314609189	1.962205	1.77E-05	hypermethylated
cg19317211	0.07367	0.28704	1.962103	0.000111	hypermethylated
cg08545287	0.07648	0.297987027	1.962095	9.31E-06	hypermethylated
cg25738714	0.08165	0.318056216	1.961757	0.000327	hypermethylated
cg05653707	0.07171	0.279324865	1.961698	5.38E-05	hypermethylated
cg01104724	0.10597	0.412743243	1.961589	1.28E-05	hypermethylated
cg19796981	0.07961	0.310014595	1.961315	1.21E-05	hypermethylated
cg01735384	0.10986	0.427702162	1.96094	1.35E-05	hypermethylated
cg21512644	0.10978	0.427336757	1.960758	1.88E-06	hypermethylated
cg03704308	0.09914	0.385881081	1.960617	7.72E-06	hypermethylated
cg21172458	0.06748	0.26258973	1.960279	2.56E-06	hypermethylated
cg11546137	0.12349	0.480342703	1.95967	2.16E-06	hypermethylated
cg23976499	0.06526	0.253823243	1.959553	7.29E-05	hypermethylated
cg08498254	0.07898	0.307183243	1.95954	8.22E-05	hypermethylated
cg02980079	0.07777	0.302421622	1.959276	8.22E-05	hypermethylated
cg05736768	0.09797	0.380918378	1.95907	5.07E-06	hypermethylated
cg08668790	0.10928	0.424883784	1.959039	4.36E-06	hypermethylated
cg14317285	0.08479	0.329644324	1.958944	3.95E-05	hypermethylated
cg09618102	0.09851	0.382902703	1.958636	1.18E-05	hypermethylated

cg26449787	0.06951	0.270142162	1.958426	4.94E-05	hypermethylated
cg05276469	0.11058	0.429656757	1.958094	3.79E-06	hypermethylated
cg21865150	0.10893	0.423224324	1.958021	1.67E-06	hypermethylated
cg26622320	0.09752	0.378723243	1.957374	3.79E-06	hypermethylated
cg22248052	0.08289	0.321802162	1.956904	6.75E-06	hypermethylated
cg11439596	0.10492	0.40678973	1.954994	1.71E-05	hypermethylated
cg26158180	0.07102	0.275317838	1.954801	4.15E-05	hypermethylated
cg23990012	0.0634	0.245569189	1.953575	9.82E-06	hypermethylated
cg25763036	0.07087	0.274464324	1.953372	1.75E-05	hypermethylated
cg12467749	0.07403	0.286679459	1.953257	7.52E-06	hypermethylated
cg08417728	0.08443	0.326825946	1.952695	6.48E-06	hypermethylated
cg20750832	0.12155	0.470514054	1.952688	6.39E-06	hypermethylated
cg17746625	0.05309	0.205357297	1.951624	6.48E-06	hypermethylated
cg07095995	0.07062	0.273057838	1.951058	8.22E-05	hypermethylated
cg18384778	0.1238	0.478502162	1.950514	3.12E-06	hypermethylated
cg05142982	0.11179	0.431892973	1.949883	7.47E-05	hypermethylated
cg23572908	0.12345	0.476852973	1.949618	1.05E-05	hypermethylated
cg07402669	0.08716	0.336663784	1.94957	1.80E-05	hypermethylated
cg08747377	0.10022	0.38705027	1.949351	2.15E-05	hypermethylated
cg02583418	0.07141	0.27576	1.949215	4.70E-05	hypermethylated
cg09534159	0.099	0.382061622	1.948305	3.23E-05	hypermethylated
cg22848598	0.09624	0.371378378	1.948181	0.000245	hypermethylated
cg05349062	0.08215	0.316918919	1.947781	9.83E-05	hypermethylated
cg03970036	0.11897	0.458461622	1.946203	2.64E-05	hypermethylated
cg22036988	0.09545	0.36774973	1.945907	1.18E-05	hypermethylated
cg21697851	0.08413	0.324102703	1.945759	1.28E-05	hypermethylated
cg26014391	0.06124	0.235892432	1.945583	8.94E-05	hypermethylated
cg10281977	0.08265	0.318315135	1.945369	5.06E-05	hypermethylated
cg21651328	0.06743	0.259673514	1.945236	5.31E-05	hypermethylated
cg00651523	0.07934	0.305462162	1.944873	2.91E-06	hypermethylated
cg04111071	0.11098	0.42710973	1.944307	8.83E-06	hypermethylated
cg21460081	0.05296	0.203800541	1.944183	0.000115	hypermethylated
cg15953602	0.07474	0.287507027	1.943645	0.000112	hypermethylated
cg04606210	0.07006	0.269437838	1.94329	3.62E-05	hypermethylated
cg06850283	0.08791	0.338071892	1.943231	7.65E-05	hypermethylated
cg12388007	0.06132	0.235808108	1.943184	5.00E-06	hypermethylated
cg03437186	0.08195	0.315086486	1.942932	6.01E-05	hypermethylated
cg05634149	0.07957	0.30584973	1.942526	6.46E-05	hypermethylated
cg04130185	0.05877	0.225871892	1.942353	3.57E-05	hypermethylated
cg01485075	0.12678	0.487224865	1.942261	1.18E-05	hypermethylated
cg22471726	0.09297	0.356976757	1.940993	1.73E-05	hypermethylated
cg04904385	0.07347	0.282092973	1.940944	2.42E-05	hypermethylated
cg18197392	0.09183	0.352549189	1.940787	0.000111	hypermethylated
cg15416279	0.09431	0.362047027	1.940694	8.10E-07	hypermethylated
cg26857670	0.07659	0.293965946	1.940421	4.01E-06	hypermethylated
cg13434308	0.09154	0.351245405	1.940005	2.32E-05	hypermethylated
cg25846723	0.06881	0.264012432	1.939916	2.15E-05	hypermethylated
cg18924324	0.09342	0.358321622	1.939452	4.70E-05	hypermethylated
cg21883598	0.10817	0.414868649	1.939354	6.57E-06	hypermethylated
cg20130213	0.09386	0.359967027	1.939282	5.14E-06	hypermethylated
cg14568830	0.09111	0.34928	1.938703	6.57E-06	hypermethylated
cg09578475	0.13955	0.534696216	1.937937	3.21E-06	hypermethylated
cg21154627	0.07895	0.302371892	1.937313	7.12E-06	hypermethylated
cg24199834	0.06476	0.247999459	1.937162	7.52E-06	hypermethylated
cg01959730	0.10074	0.385781081	1.937146	3.39E-06	hypermethylated
cg07969619	0.06475	0.247905946	1.936841	1.26E-05	hypermethylated
cg00966398	0.05082	0.194545405	1.936639	1.48E-05	hypermethylated
cg13242070	0.10034	0.384044324	1.936376	2.21E-05	hypermethylated

cg18081940	0.11288	0.432022162	1.936315	6.93E-06	hypermethylated
cg13267931	0.07489	0.286605405	1.936221	0.000112	hypermethylated
cg18202449	0.08842	0.338347027	1.936059	3.15E-05	hypermethylated
cg22851691	0.11158	0.426916216	1.935875	9.11E-07	hypermethylated
cg00768993	0.07861	0.300585405	1.93499	1.92E-05	hypermethylated
cg03949391	0.07262	0.277638378	1.934768	0.000207	hypermethylated
cg18430555	0.0805	0.307754595	1.93472	1.46E-05	hypermethylated
cg21745612	0.07881	0.301226486	1.934398	3.23E-05	hypermethylated
cg04307561	0.1027	0.392514595	1.93431	8.26E-06	hypermethylated
cg10024876	0.06346	0.242527568	1.934229	2.78E-05	hypermethylated
cg12034383	0.09028	0.344962703	1.933962	0.00028	hypermethylated
cg20646280	0.07793	0.297672432	1.933475	4.26E-05	hypermethylated
cg04027805	0.04861	0.185531892	1.932342	3.69E-06	hypermethylated
cg26983177	0.06959	0.265596757	1.932286	3.95E-05	hypermethylated
cg08018585	0.09585	0.365717297	1.931879	2.92E-05	hypermethylated
cg09676860	0.0662	0.252576216	1.931816	7.56E-05	hypermethylated
cg23690166	0.11501	0.438742162	1.931614	1.04E-05	hypermethylated
cg20011562	0.05378	0.205115676	1.931296	3.57E-05	hypermethylated
cg00418282	0.0932	0.35540973	1.931081	3.49E-06	hypermethylated
cg25717994	0.04847	0.184802703	1.930822	9.05E-05	hypermethylated
cg04495995	0.08635	0.329193514	1.930668	1.12E-05	hypermethylated
cg26860935	0.10798	0.411575676	1.930394	9.57E-06	hypermethylated
cg20764116	0.05797	0.220941622	1.930287	2.27E-05	hypermethylated
cg24934400	0.08707	0.331771892	1.929944	4.70E-05	hypermethylated
cg00924004	0.07471	0.284667027	1.929902	3.19E-05	hypermethylated
cg12161228	0.12257	0.466992973	1.929795	1.06E-05	hypermethylated
cg23495279	0.10103	0.384915676	1.929759	1.31E-05	hypermethylated
cg26609631	0.09507	0.362123243	1.929419	8.03E-05	hypermethylated
cg04622777	0.07524	0.28646	1.928762	1.25E-05	hypermethylated
cg04917226	0.10121	0.385317297	1.928695	1.18E-06	hypermethylated
cg00937271	0.05942	0.226102703	1.927958	1.30E-05	hypermethylated
cg26560222	0.08381	0.318884324	1.927839	7.12E-06	hypermethylated
cg19542816	0.08805	0.3349	1.927335	0.0006	hypermethylated
cg02582387	0.08378	0.318650811	1.927299	0.000166	hypermethylated
cg06804433	0.05708	0.217016216	1.926746	2.15E-05	hypermethylated
cg10445315	0.08427	0.320335676	1.926493	2.32E-05	hypermethylated
cg16508480	0.10073	0.382801622	1.926104	1.50E-05	hypermethylated
cg01603847	0.09556	0.363122703	1.925978	1.62E-05	hypermethylated
cg13576883	0.0707	0.268617838	1.925773	3.40E-05	hypermethylated
cg04630292	0.08727	0.331428649	1.925141	1.99E-06	hypermethylated
cg00950846	0.07479	0.284004324	1.924996	2.15E-05	hypermethylated
cg18491080	0.08367	0.317661081	1.924706	1.17E-05	hypermethylated
cg11670651	0.08164	0.309821081	1.924087	6.62E-05	hypermethylated
cg10443049	0.09002	0.341542162	1.923746	5.89E-06	hypermethylated
cg06954658	0.05622	0.21326	1.923458	1.67E-06	hypermethylated
cg16001495	0.07273	0.275868108	1.923356	1.28E-05	hypermethylated
cg11994674	0.09647	0.365819459	1.92298	2.53E-06	hypermethylated
cg09918296	0.07494	0.284142162	1.922805	1.31E-05	hypermethylated
cg07103493	0.08924	0.338317297	1.922615	2.64E-06	hypermethylated
cg02164046	0.10375	0.393294054	1.922497	7.93E-06	hypermethylated
cg07104706	0.12298	0.46608973	1.922184	5.89E-06	hypermethylated
cg03307893	0.0667	0.252741622	1.921905	0.000189	hypermethylated
cg22399133	0.13183	0.499462703	1.921698	3.32E-05	hypermethylated
cg17357285	0.08335	0.31555027	1.920616	1.35E-05	hypermethylated
cg19254119	0.08081	0.305918919	1.920544	1.31E-05	hypermethylated
cg07447922	0.09258	0.350427568	1.920344	3.07E-05	hypermethylated
cg19050990	0.06425	0.243162162	1.92015	7.65E-05	hypermethylated
cg15843567	0.09395	0.355448108	1.919674	0.000195	hypermethylated

cg09341793	0.12996	0.491660541	1.919595	5.73E-06	hypermethylated
cg03544320	0.14717	0.556671351	1.919342	2.42E-06	hypermethylated
cg22131691	0.07974	0.30158973	1.919212	3.71E-05	hypermethylated
cg21781988	0.06136	0.232070811	1.919195	4.01E-06	hypermethylated
cg00310215	0.09685	0.366161622	1.918657	6.39E-06	hypermethylated
cg06912966	0.07047	0.266363784	1.918317	3.53E-05	hypermethylated
cg11667258	0.10214	0.385971892	1.917948	1.08E-05	hypermethylated
cg13459498	0.09137	0.345272973	1.917945	9.95E-05	hypermethylated
cg25308508	0.11828	0.446881081	1.917685	1.26E-06	hypermethylated
cg05398903	0.10249	0.387097297	1.917213	6.22E-06	hypermethylated
cg05743885	0.0789	0.297993514	1.917184	7.72E-06	hypermethylated
cg14537713	0.07609	0.287275676	1.916657	1.31E-05	hypermethylated
cg15712559	0.10215	0.385535135	1.916173	4.82E-05	hypermethylated
cg25209842	0.09958	0.375654595	1.915479	3.11E-05	hypermethylated
cg01921432	0.08942	0.33730973	1.915404	8.15E-06	hypermethylated
cg05026135	0.07788	0.293754054	1.915284	5.21E-06	hypermethylated
cg23217946	0.08688	0.327638378	1.915008	4.15E-05	hypermethylated
cg11763094	0.07203	0.271632973	1.914989	1.26E-05	hypermethylated
cg06321998	0.07796	0.293741622	1.913742	2.13E-05	hypermethylated
cg04543938	0.11055	0.416215135	1.91263	6.62E-05	hypermethylated
cg24748769	0.08514	0.320396757	1.911951	6.22E-06	hypermethylated
cg02583633	0.07969	0.29974	1.911241	0.000209	hypermethylated
cg20011402	0.08908	0.335051892	1.911211	0.000287	hypermethylated
cg01268824	0.10479	0.393903243	1.91034	1.25E-05	hypermethylated
cg24154839	0.07623	0.286514054	1.910175	2.57E-05	hypermethylated
cg05991454	0.1048	0.393838919	1.909967	3.59E-06	hypermethylated
cg07175883	0.07641	0.287062162	1.90953	3.15E-05	hypermethylated
cg00131983	0.08179	0.30722973	1.909321	1.20E-05	hypermethylated
cg17877704	0.07576	0.284575676	1.909304	1.46E-05	hypermethylated
cg20066716	0.09887	0.371342162	1.909144	8.15E-06	hypermethylated
cg24633978	0.10181	0.382331892	1.908946	1.82E-06	hypermethylated
cg02166450	0.07655	0.287339459	1.908282	1.89E-05	hypermethylated
cg15272362	0.07651	0.287144865	1.908059	0.00014	hypermethylated
cg23894086	0.08248	0.309534054	1.907982	7.12E-06	hypermethylated
cg26956874	0.08769	0.329026486	1.907719	9.31E-06	hypermethylated
cg08875705	0.12057	0.452384865	1.90768	2.45E-05	hypermethylated
cg09605164	0.08051	0.301972432	1.907177	7.93E-06	hypermethylated
cg01159623	0.07647	0.286818378	1.907172	4.07E-06	hypermethylated
cg20034792	0.08745	0.327945946	1.906928	0.00062	hypermethylated
cg18722841	0.10238	0.383671892	1.905939	4.18E-06	hypermethylated
cg11523712	0.06566	0.246056216	1.905901	1.21E-05	hypermethylated
cg12565585	0.09709	0.363735135	1.905494	1.99E-05	hypermethylated
cg12840502	0.07838	0.293584324	1.905217	9.05E-05	hypermethylated
cg24351410	0.06276	0.235010811	1.90481	2.04E-05	hypermethylated
cg16605327	0.0882	0.330251351	1.904714	8.15E-06	hypermethylated
cg03692651	0.16569	0.62038	1.904666	7.72E-06	hypermethylated
cg02317400	0.07807	0.292294054	1.90458	9.05E-05	hypermethylated
cg21484228	0.08574	0.321001622	1.90454	0.00054	hypermethylated
cg17957186	0.06809	0.254841081	1.904083	3.00E-05	hypermethylated
cg26437826	0.0815	0.304963243	1.903763	2.85E-05	hypermethylated
cg23460843	0.11562	0.432635135	1.90376	0.000182	hypermethylated
cg19084589	0.07102	0.26568973	1.903445	3.07E-05	hypermethylated
cg11315153	0.06501	0.243151892	1.903124	1.33E-05	hypermethylated
cg12807794	0.08053	0.301154595	1.902906	6.75E-06	hypermethylated
cg18629535	0.08732	0.32648	1.902611	1.68E-05	hypermethylated
cg19761848	0.11486	0.429055676	1.901288	2.64E-05	hypermethylated
cg18948722	0.0938	0.350335676	1.901078	1.80E-05	hypermethylated
cg20787173	0.09429	0.352091892	1.900775	2.42E-05	hypermethylated

cg14956197	0.06762	0.252467568	1.900576	3.23E-05	hypermethylated
cg20495183	0.05889	0.219698378	1.89943	6.23E-05	hypermethylated
cg24852548	0.09965	0.371650811	1.899006	2.15E-05	hypermethylated
cg16458494	0.0896	0.333721622	1.897075	1.26E-05	hypermethylated
cg14970666	0.07855	0.292543784	1.896969	3.61E-07	hypermethylated
cg03782202	0.10442	0.388883243	1.896939	1.09E-05	hypermethylated
cg24722073	0.08743	0.325605946	1.896927	8.15E-06	hypermethylated
cg10575261	0.07128	0.265459459	1.896922	1.42E-05	hypermethylated
cg16195091	0.09701	0.361165946	1.896456	1.84E-05	hypermethylated
cg05313771	0.06462	0.240566486	1.896383	6.05E-06	hypermethylated
cg25532099	0.08116	0.302095676	1.896165	0.000129	hypermethylated
cg21158633	0.10047	0.373944324	1.896059	3.67E-05	hypermethylated
cg14781189	0.07527	0.280103784	1.895815	3.00E-05	hypermethylated
cg07028914	0.0786	0.292494054	1.895806	0.00028	hypermethylated
cg14011639	0.07001	0.260478378	1.895531	1.92E-05	hypermethylated
cg15985184	0.10316	0.383776757	1.895384	1.71E-05	hypermethylated
cg08795964	0.10929	0.406564324	1.895322	4.12E-06	hypermethylated
cg25540824	0.09797	0.364440541	1.895272	8.84E-07	hypermethylated
cg07351192	0.09128	0.339544324	1.895229	8.37E-06	hypermethylated
cg15484532	0.09341	0.347402162	1.894958	6.13E-06	hypermethylated
cg09229683	0.05893	0.219154595	1.894875	3.21E-06	hypermethylated
cg23208176	0.07258	0.269866486	1.894602	1.73E-05	hypermethylated
cg07780543	0.09684	0.360028108	1.894435	9.31E-06	hypermethylated
cg21145524	0.09831	0.36548973	1.894421	8.63E-05	hypermethylated
cg27444994	0.09197	0.341622162	1.893166	1.62E-05	hypermethylated
cg22499720	0.07817	0.290332973	1.893021	2.15E-05	hypermethylated
cg21370856	0.09908	0.367915135	1.892707	1.60E-05	hypermethylated
cg03032214	0.07213	0.267774054	1.892345	4.94E-05	hypermethylated
cg04739647	0.08804	0.326724324	1.891843	5.14E-06	hypermethylated
cg10427452	0.08057	0.298976757	1.891719	3.00E-05	hypermethylated
cg24613080	0.13078	0.48516	1.891319	7.32E-06	hypermethylated
cg00590139	0.08984	0.333221622	1.891052	3.76E-05	hypermethylated
cg06360427	0.10361	0.384283243	1.891007	8.60E-06	hypermethylated
cg23129478	0.07538	0.279561622	1.890913	2.15E-05	hypermethylated
cg26537209	0.06565	0.243429189	1.890635	1.15E-05	hypermethylated
cg14305278	0.07022	0.260288108	1.890155	6.57E-06	hypermethylated
cg16768018	0.12304	0.456039459	1.890031	2.64E-06	hypermethylated
cg14037665	0.08474	0.313855676	1.888986	1.31E-05	hypermethylated
cg15908006	0.0627	0.232174595	1.888673	4.05E-05	hypermethylated
cg17121178	0.07763	0.287383243	1.88829	1.02E-05	hypermethylated
cg20014398	0.12223	0.452348649	1.887837	1.18E-05	hypermethylated
cg06675190	0.10497	0.387994054	1.886057	2.51E-05	hypermethylated
cg07382920	0.10685	0.394843784	1.885695	3.59E-06	hypermethylated
cg11097433	0.11663	0.430811351	1.885117	1.50E-05	hypermethylated
cg15680288	0.08117	0.299767568	1.884826	2.92E-05	hypermethylated
cg10243196	0.08132	0.300312432	1.884782	8.52E-05	hypermethylated
cg08318726	0.1042	0.384785405	1.884699	3.85E-05	hypermethylated
cg21041775	0.08981	0.331635135	1.884649	1.84E-05	hypermethylated
cg01048962	0.08225	0.303681081	1.884469	0.000287	hypermethylated
cg09434193	0.09784	0.360903243	1.883116	3.81E-05	hypermethylated
cg16935065	0.10545	0.388947568	1.883017	1.94E-05	hypermethylated
cg23758305	0.09455	0.348715676	1.882902	5.43E-06	hypermethylated
cg24073122	0.08342	0.307638378	1.88277	2.78E-05	hypermethylated
cg21858380	0.0807	0.297501622	1.882257	5.58E-05	hypermethylated
cg27467929	0.0843	0.310724865	1.882033	9.27E-05	hypermethylated
cg06370771	0.08054	0.296841622	1.881916	1.75E-05	hypermethylated
cg00340850	0.06955	0.256335135	1.881909	2.95E-06	hypermethylated
cg20685897	0.06797	0.250487568	1.881769	2.61E-05	hypermethylated

cg22125805	0.12512	0.461055135	1.881627	9.82E-06	hypermethylated
cg00522018	0.07723	0.284542703	1.881412	7.83E-06	hypermethylated
cg20510724	0.09376	0.345441622	1.881397	4.73E-06	hypermethylated
cg04115680	0.12083	0.445138378	1.881275	4.82E-05	hypermethylated
cg27353143	0.10608	0.390779459	1.881202	5.14E-06	hypermethylated
cg08421685	0.07147	0.263272973	1.88115	0.00024	hypermethylated
cg12159189	0.10024	0.36922	1.881022	1.35E-05	hypermethylated
cg20271517	0.07916	0.291550811	1.880904	1.20E-05	hypermethylated
cg16073378	0.10733	0.395281622	1.880827	1.04E-05	hypermethylated
cg15301316	0.09765	0.359391351	1.879864	1.14E-05	hypermethylated
cg21762788	0.10642	0.391608649	1.879643	6.93E-06	hypermethylated
cg22473637	0.06942	0.255438378	1.879552	1.11E-05	hypermethylated
cg11326255	0.08682	0.319436757	1.879431	6.54E-05	hypermethylated
cg12838303	0.10629	0.391064324	1.8794	4.48E-06	hypermethylated
cg20449685	0.09832	0.361645405	1.879019	9.07E-06	hypermethylated
cg00848728	0.06264	0.230269189	1.878165	1.12E-05	hypermethylated
cg03181248	0.10312	0.378955676	1.877705	2.21E-05	hypermethylated
cg11946503	0.09097	0.334285946	1.87762	3.49E-06	hypermethylated
cg13344169	0.06557	0.240917838	1.877433	0.000338	hypermethylated
cg04495670	0.09442	0.346776757	1.876843	8.03E-05	hypermethylated
cg18324707	0.10285	0.377703784	1.876713	3.67E-05	hypermethylated
cg08125821	0.08393	0.30806	1.875953	1.23E-05	hypermethylated
cg23141855	0.0775	0.284448108	1.875897	3.23E-05	hypermethylated
cg23847712	0.12531	0.459728108	1.875279	2.95E-06	hypermethylated
cg03243226	0.11781	0.432155135	1.875087	2.27E-05	hypermethylated
cg22097425	0.09363	0.343443784	1.875031	6.57E-06	hypermethylated
cg01689438	0.08857	0.324772432	1.874539	5.35E-06	hypermethylated
cg10601582	0.11584	0.424756757	1.874503	6.93E-06	hypermethylated
cg14730445	0.1034	0.379108108	1.874373	2.45E-05	hypermethylated
cg08535373	0.09334	0.342076216	1.87375	5.43E-06	hypermethylated
cg00939495	0.11594	0.424897838	1.873738	7.52E-06	hypermethylated
cg08453021	0.07617	0.279043784	1.873197	4.64E-05	hypermethylated
cg18335068	0.07837	0.287100541	1.873183	8.04E-06	hypermethylated
cg11706835	0.05331	0.19526973	1.87299	3.85E-06	hypermethylated
cg00344443	0.11232	0.411385405	1.872876	1.39E-05	hypermethylated
cg05945059	0.09426	0.345194595	1.872692	1.31E-05	hypermethylated
cg08572611	0.1163	0.425712973	1.87203	1.21E-05	hypermethylated
cg09619271	0.08117	0.297048649	1.871681	0.000214	hypermethylated
cg14270292	0.07458	0.27288	1.871406	6.15E-05	hypermethylated
cg07160277	0.0723	0.264520541	1.871312	1.23E-05	hypermethylated
cg23596233	0.10397	0.380163784	1.870454	3.49E-05	hypermethylated
cg04017533	0.0772	0.282110811	1.869589	4.82E-05	hypermethylated
cg05422029	0.10002	0.365497838	1.869574	2.79E-06	hypermethylated
cg17554604	0.07604	0.27778	1.869112	0.001188	hypermethylated
cg04902729	0.09726	0.355271351	1.869003	8.60E-06	hypermethylated
cg14058383	0.0601	0.219379459	1.867992	6.39E-06	hypermethylated
cg13784080	0.08188	0.298850811	1.867842	8.60E-06	hypermethylated
cg11676902	0.06808	0.248474054	1.867792	0.000209	hypermethylated
cg13390867	0.09547	0.348357838	1.867451	4.26E-05	hypermethylated
cg11898486	0.07384	0.26942	1.867382	6.01E-05	hypermethylated
cg04958794	0.08754	0.319309189	1.86694	3.90E-06	hypermethylated
cg12560987	0.13066	0.47654	1.86678	2.36E-06	hypermethylated
cg17633592	0.06016	0.219391351	1.86663	1.84E-05	hypermethylated
cg27247697	0.08214	0.299537838	1.866581	6.38E-05	hypermethylated
cg26990587	0.10868	0.396221622	1.866221	3.62E-05	hypermethylated
cg24019371	0.07654	0.279026486	1.866116	4.26E-05	hypermethylated
cg00824018	0.09785	0.356682162	1.865995	1.62E-05	hypermethylated
cg03192598	0.10461	0.381271351	1.865797	0.000306	hypermethylated

cg03495868	0.06233	0.227166486	1.865751	7.65E-05	hypermethylated
cg12646649	0.11114	0.404798378	1.864825	2.92E-05	hypermethylated
cg20387341	0.0674	0.245414054	1.864397	3.00E-05	hypermethylated
cg13187764	0.09398	0.342180541	1.864332	5.73E-06	hypermethylated
cg14062050	0.111	0.404125405	1.864243	2.79E-06	hypermethylated
cg09772661	0.10244	0.37292973	1.864125	6.38E-05	hypermethylated
cg11612786	0.07075	0.257551351	1.864058	1.17E-05	hypermethylated
cg03072621	0.10838	0.394423243	1.863646	1.71E-05	hypermethylated
cg27347290	0.07724	0.281071892	1.863519	4.82E-05	hypermethylated
cg24937747	0.11092	0.403597838	1.863399	3.90E-06	hypermethylated
cg19945554	0.09482	0.344998919	1.863329	8.42E-05	hypermethylated
cg22130262	0.08655	0.314791351	1.86279	2.39E-05	hypermethylated
cg10514097	0.09505	0.345612432	1.862397	2.32E-05	hypermethylated
cg08602190	0.08098	0.294435135	1.862312	1.71E-05	hypermethylated
cg08812189	0.10521	0.382475676	1.862096	3.39E-06	hypermethylated
cg18534345	0.07723	0.280681622	1.861701	8.83E-06	hypermethylated
cg19822251	0.08171	0.296951351	1.861642	1.80E-05	hypermethylated
cg26590537	0.08918	0.324046486	1.861409	1.39E-05	hypermethylated
cg27642554	0.07896	0.286685946	1.860277	3.62E-05	hypermethylated
cg00589371	0.07285	0.264498378	1.860258	4.05E-05	hypermethylated
cg08271804	0.09252	0.335882162	1.860118	3.69E-06	hypermethylated
cg00018128	0.08564	0.310855135	1.859886	2.04E-05	hypermethylated
cg04804618	0.09365	0.339888649	1.859711	3.15E-05	hypermethylated
cg05210258	0.10305	0.373985946	1.85964	1.46E-05	hypermethylated
cg08323651	0.07968	0.28909027	1.859231	1.71E-05	hypermethylated
cg13465477	0.1013	0.367520541	1.859191	1.99E-05	hypermethylated
cg22178613	0.09346	0.338976757	1.858765	2.45E-05	hypermethylated
cg00548708	0.09136	0.331294595	1.85848	5.14E-06	hypermethylated
cg14685796	0.07943	0.288001081	1.858318	1.89E-05	hypermethylated
cg14400886	0.08143	0.295215135	1.858134	3.23E-05	hypermethylated
cg13525197	0.07492	0.27156	1.857848	1.89E-05	hypermethylated
cg10959353	0.09867	0.35759027	1.857624	1.46E-05	hypermethylated
cg08776356	0.08543	0.309482703	1.857044	1.28E-05	hypermethylated
cg07204280	0.08685	0.314576216	1.856812	0.000365	hypermethylated
cg22299741	0.06436	0.233097838	1.856699	1.28E-05	hypermethylated
cg12877723	0.10261	0.371555676	1.856407	3.69E-06	hypermethylated
cg11659501	0.07435	0.269136757	1.855935	1.01E-05	hypermethylated
cg25632105	0.07416	0.268418378	1.85577	1.62E-05	hypermethylated
cg06369992	0.08961	0.324314054	1.85566	9.19E-06	hypermethylated
cg09799658	0.0877	0.316926486	1.853499	1.21E-05	hypermethylated
cg01486814	0.0736	0.265963784	1.853452	1.54E-05	hypermethylated
cg03532926	0.13057	0.471721081	1.853111	1.42E-05	hypermethylated
cg03964958	0.11111	0.401313514	1.852741	2.10E-06	hypermethylated
cg06806958	0.09824	0.354824324	1.852722	2.39E-05	hypermethylated
cg01284666	0.07728	0.279065405	1.852436	7.12E-05	hypermethylated
cg09048251	0.1037	0.374453514	1.852371	2.21E-05	hypermethylated
cg02561482	0.09958	0.359540541	1.852227	2.79E-06	hypermethylated
cg19632594	0.05889	0.212618378	1.852172	3.81E-05	hypermethylated
cg04201750	0.06185	0.223299459	1.852134	6.05E-06	hypermethylated
cg00991875	0.12245	0.441975676	1.851774	1.15E-05	hypermethylated
cg15183083	0.0806	0.290875676	1.851551	4.47E-05	hypermethylated
cg15132673	0.06297	0.227166486	1.851013	0.000338	hypermethylated
cg10058779	0.07091	0.255677297	1.850263	0.000132	hypermethylated
cg04961466	0.09395	0.338738919	1.850209	1.15E-05	hypermethylated
cg04227922	0.11801	0.425485405	1.850201	4.01E-06	hypermethylated
cg05667348	0.1095	0.394753514	1.850021	7.32E-06	hypermethylated
cg02864757	0.09004	0.32458973	1.849979	1.04E-05	hypermethylated
cg18103859	0.10484	0.377908108	1.849846	0.000109	hypermethylated

cg20778451	0.09089	0.327557297	1.849554	7.93E-06	hypermethylated
cg27603283	0.07397	0.266563243	1.849466	8.42E-05	hypermethylated
cg01939453	0.10676	0.384501081	1.848616	9.57E-06	hypermethylated
cg19096825	0.10492	0.377869189	1.848597	1.06E-05	hypermethylated
cg23683588	0.08839	0.318318378	1.848515	1.58E-05	hypermethylated
cg05983315	0.13563	0.488319459	1.848149	6.05E-06	hypermethylated
cg03610852	0.08884	0.319704324	1.847457	7.52E-06	hypermethylated
cg08039116	0.10778	0.387797297	1.847213	4.73E-06	hypermethylated
cg13843613	0.06713	0.241489189	1.846929	7.29E-05	hypermethylated
cg05328547	0.08092	0.291054054	1.846719	7.12E-06	hypermethylated
cg07592963	0.10665	0.38351027	1.846381	1.37E-05	hypermethylated
cg04415176	0.12598	0.452985946	1.846272	1.15E-05	hypermethylated
cg16876315	0.07133	0.256397838	1.845803	2.67E-05	hypermethylated
cg10507508	0.09306	0.334394595	1.845318	5.86E-05	hypermethylated
cg11359133	0.10013	0.359742162	1.845089	5.65E-06	hypermethylated
cg14510359	0.07342	0.263755676	1.844957	9.05E-05	hypermethylated
cg19792599	0.08628	0.309855676	1.844498	2.71E-05	hypermethylated
cg26282384	0.10353	0.371703243	1.844102	4.80E-06	hypermethylated
cg17714025	0.09141	0.328182703	1.844075	1.71E-05	hypermethylated
cg10300684	0.11507	0.413074595	1.843891	1.04E-05	hypermethylated
cg01354961	0.07148	0.256583784	1.843818	0.000358	hypermethylated
cg27041794	0.07632	0.273932432	1.843687	0.000112	hypermethylated
cg00885682	0.06391	0.229374054	1.843589	3.85E-06	hypermethylated
cg20103758	0.087	0.312031351	1.842604	6.57E-06	hypermethylated
cg00497967	0.06994	0.25082	1.842463	2.64E-05	hypermethylated
cg25729445	0.08603	0.308466486	1.842202	8.26E-06	hypermethylated
cg25950625	0.10316	0.369784865	1.841802	0.000245	hypermethylated
cg03352106	0.15461	0.554197297	1.841766	1.13E-06	hypermethylated
cg12791492	0.06243	0.223766486	1.841683	1.87E-05	hypermethylated
cg09788416	0.09676	0.346661622	1.841045	2.10E-05	hypermethylated
cg14508508	0.08973	0.321375135	1.840596	1.94E-05	hypermethylated
cg27434509	0.12504	0.447788649	1.840428	4.58E-05	hypermethylated
cg18723560	0.0868	0.310709189	1.839798	3.67E-05	hypermethylated
cg19430967	0.08701	0.311414595	1.839583	4.36E-05	hypermethylated
cg18267049	0.1034	0.369958378	1.839127	5.58E-05	hypermethylated
cg07918545	0.12903	0.461465946	1.838518	5.73E-06	hypermethylated
cg01427575	0.07947	0.284160541	1.838224	3.04E-05	hypermethylated
cg07983905	0.09148	0.327094595	1.83818	0.000126	hypermethylated
cg16413030	0.06194	0.221360541	1.837455	0.000126	hypermethylated
cg25078444	0.1179	0.421216216	1.836997	1.89E-05	hypermethylated
cg25517810	0.12924	0.461708108	1.836928	6.39E-06	hypermethylated
cg11070069	0.09535	0.340530811	1.83648	6.57E-06	hypermethylated
cg08701621	0.12287	0.438547568	1.835601	4.05E-05	hypermethylated
cg11645631	0.07459	0.266221622	1.835574	5.58E-05	hypermethylated
cg04738965	0.12551	0.44778973	1.835019	2.36E-06	hypermethylated
cg04784672	0.10497	0.374286486	1.834166	3.39E-06	hypermethylated
cg07536847	0.08085	0.288238378	1.833943	0.000116	hypermethylated
cg01962676	0.07112	0.253522703	1.833788	5.31E-05	hypermethylated
cg17922359	0.09384	0.334465946	1.833584	1.94E-05	hypermethylated
cg01518607	0.1413	0.503602703	1.833525	8.10E-07	hypermethylated
cg20670075	0.09987	0.355925946	1.833454	5.81E-06	hypermethylated
cg26923490	0.09991	0.355876216	1.832675	1.04E-05	hypermethylated
cg23665381	0.08654	0.308208108	1.832466	1.66E-05	hypermethylated
cg02886522	0.06855	0.244132432	1.832435	9.38E-05	hypermethylated
cg14976646	0.06716	0.239172973	1.83238	5.81E-06	hypermethylated
cg08158952	0.11186	0.398354595	1.832359	3.30E-06	hypermethylated
cg15285250	0.10998	0.391601081	1.832144	9.82E-06	hypermethylated
cg13188011	0.06773	0.241100541	1.831768	6.14E-06	hypermethylated

cg06022179	0.085	0.302507027	1.831434	1.50E-05	hypermethylated
cg04584301	0.09365	0.333158919	1.83086	0.000277	hypermethylated
cg10095242	0.08764	0.311745405	1.830707	0.000132	hypermethylated
cg13944175	0.12901	0.458640541	1.829881	2.83E-06	hypermethylated
cg09019936	0.07864	0.279509189	1.82956	3.81E-05	hypermethylated
cg11321433	0.07169	0.254715135	1.829041	1.12E-05	hypermethylated
cg26530498	0.09618	0.341522162	1.82817	4.12E-06	hypermethylated
cg22861116	0.11496	0.408105946	1.827812	1.75E-05	hypermethylated
cg17525406	0.10944	0.388484865	1.827718	2.51E-05	hypermethylated
cg23805360	0.07281	0.258448649	1.827669	0.000123	hypermethylated
cg06507579	0.11351	0.402885946	1.827552	6.22E-06	hypermethylated
cg22881914	0.10749	0.381477297	1.827395	0.000884	hypermethylated
cg10159630	0.09665	0.342991892	1.827333	9.31E-06	hypermethylated
cg18273840	0.09791	0.347410811	1.827115	0.0003	hypermethylated
cg23697546	0.1006	0.356945946	1.827075	6.15E-05	hypermethylated
cg25285090	0.12307	0.436554595	1.826683	5.43E-06	hypermethylated
cg02155658	0.08166	0.289510811	1.825916	5.19E-05	hypermethylated
cg07681938	0.10657	0.377807027	1.825848	2.79E-06	hypermethylated
cg08130144	0.06921	0.245345946	1.825765	2.39E-05	hypermethylated
cg09492451	0.12143	0.430439459	1.825685	2.04E-05	hypermethylated
cg20449590	0.128	0.453701622	1.8256	3.39E-06	hypermethylated
cg08482436	0.11159	0.395530811	1.825582	2.71E-06	hypermethylated
cg07831417	0.0736	0.260792973	1.825127	1.33E-05	hypermethylated
cg19707040	0.08151	0.288735676	1.8247	8.42E-05	hypermethylated
cg00800512	0.0986	0.34916	1.824229	1.62E-05	hypermethylated
cg19651694	0.10504	0.371855676	1.823804	2.42E-06	hypermethylated
cg24080247	0.10551	0.373461081	1.823578	1.18E-05	hypermethylated
cg08160350	0.09235	0.326844865	1.823422	0.000154	hypermethylated
cg18657094	0.10408	0.368342162	1.823354	8.95E-06	hypermethylated
cg23244910	0.08926	0.315884324	1.823311	7.74E-05	hypermethylated
cg19628148	0.10342	0.365993514	1.823303	2.07E-05	hypermethylated
cg18243760	0.10494	0.371347568	1.823205	3.90E-05	hypermethylated
cg19741167	0.10645	0.376617297	1.822923	6.57E-06	hypermethylated
cg06062984	0.12709	0.449625405	1.822873	6.05E-06	hypermethylated
cg19019849	0.0677	0.239466486	1.822596	1.28E-05	hypermethylated
cg02093732	0.1016	0.359368649	1.822564	3.79E-06	hypermethylated
cg03625287	0.10419	0.368479459	1.822367	7.41E-06	hypermethylated
cg10390058	0.08466	0.299283243	1.821759	1.52E-05	hypermethylated
cg07850527	0.131	0.462961622	1.821326	0.0002	hypermethylated
cg14676407	0.08136	0.287496216	1.821151	9.82E-06	hypermethylated
cg22797031	0.17841	0.630378919	1.821023	2.79E-06	hypermethylated
cg05255275	0.09828	0.347212432	1.820849	4.36E-06	hypermethylated
cg20881910	0.08148	0.287857297	1.820836	3.95E-05	hypermethylated
cg22983083	0.0949	0.335264324	1.820819	1.39E-05	hypermethylated
cg21292008	0.07904	0.279174054	1.82051	7.65E-05	hypermethylated
cg19677607	0.07126	0.251672973	1.820386	6.78E-05	hypermethylated
cg14035771	0.06502	0.229623784	1.820317	4.26E-05	hypermethylated
cg14523847	0.13453	0.474844324	1.819527	7.72E-06	hypermethylated
cg22336004	0.08645	0.305134595	1.819508	3.76E-05	hypermethylated
cg02072400	0.06797	0.239877838	1.81933	7.84E-05	hypermethylated
cg21099470	0.10049	0.354576757	1.819046	3.39E-06	hypermethylated
cg04980590	0.08756	0.308945946	1.819011	2.95E-06	hypermethylated
cg01939562	0.1049	0.37006973	1.818782	5.58E-06	hypermethylated
cg14531663	0.10604	0.373984324	1.818369	9.07E-06	hypermethylated
cg00491180	0.08348	0.294418919	1.818368	8.03E-05	hypermethylated
cg07636117	0.08548	0.301393514	1.81799	7.65E-05	hypermethylated
cg07104660	0.07085	0.249800541	1.817937	1.25E-05	hypermethylated
cg04279973	0.0904	0.318675676	1.817694	3.23E-05	hypermethylated

cg05099387	0.11137	0.392498378	1.817326	0.000563	hypermethylated
cg25903158	0.07001	0.246711892	1.817194	0.000107	hypermethylated
cg11965370	0.09191	0.323756757	1.816617	1.35E-05	hypermethylated
cg04838147	0.08092	0.285000541	1.816396	2.96E-05	hypermethylated
cg20121142	0.08388	0.295348649	1.81602	1.26E-05	hypermethylated
cg13628577	0.11542	0.406204865	1.815314	7.52E-06	hypermethylated
cg10732215	0.12348	0.434532973	1.815188	0.000575	hypermethylated
cg00618450	0.09214	0.324125946	1.814655	2.13E-05	hypermethylated
cg01049205	0.07358	0.258831892	1.81463	2.85E-05	hypermethylated
cg23442672	0.09088	0.319664324	1.814523	2.04E-05	hypermethylated
cg04514249	0.09333	0.328144865	1.81392	4.47E-05	hypermethylated
cg06122871	0.11993	0.421645405	1.813838	2.79E-06	hypermethylated
cg01618130	0.08976	0.315442703	1.813233	2.42E-05	hypermethylated
cg02432280	0.10663	0.374723784	1.813214	9.57E-06	hypermethylated
cg08676975	0.1039	0.365062703	1.812949	1.18E-05	hypermethylated
cg11996428	0.09259	0.325315135	1.81291	2.74E-05	hypermethylated
cg27005794	0.10453	0.367225946	1.812751	9.27E-05	hypermethylated
cg01664666	0.0867	0.304541622	1.812536	8.42E-05	hypermethylated
cg17495719	0.07824	0.274792973	1.812367	0.000123	hypermethylated
cg10366093	0.09742	0.342143243	1.812311	2.10E-05	hypermethylated
cg04671611	0.11288	0.396392432	1.81214	6.66E-06	hypermethylated
cg01046104	0.10771	0.378151892	1.811814	4.70E-05	hypermethylated
cg07477792	0.06727	0.236151351	1.811677	7.12E-05	hypermethylated
cg26024843	0.09971	0.349957297	1.811369	1.66E-05	hypermethylated
cg11277087	0.09919	0.348127027	1.811347	2.13E-06	hypermethylated
cg18106312	0.0969	0.340047568	1.811168	3.95E-05	hypermethylated
cg17840719	0.12574	0.441210811	1.811024	1.71E-05	hypermethylated
cg09222749	0.08083	0.28360973	1.810944	1.44E-05	hypermethylated
cg13358636	0.10915	0.382925405	1.810751	1.09E-05	hypermethylated
cg27606499	0.11533	0.404557838	1.810578	3.69E-06	hypermethylated
cg02530022	0.11687	0.409942703	1.810518	1.82E-06	hypermethylated
cg11642106	0.12042	0.422327027	1.810286	1.99E-05	hypermethylated
cg01916088	0.12001	0.420876757	1.810243	2.15E-05	hypermethylated
cg02991338	0.0817	0.286462162	1.809937	1.35E-05	hypermethylated
cg04774711	0.08245	0.289069189	1.809823	1.46E-05	hypermethylated
cg21459340	0.11413	0.400083784	1.809624	5.00E-06	hypermethylated
cg09524907	0.1211	0.424440541	1.809364	1.80E-05	hypermethylated
cg12180703	0.12464	0.436834595	1.80932	1.04E-05	hypermethylated
cg17283453	0.10763	0.377214054	1.809303	3.21E-06	hypermethylated
cg27278953	0.0788	0.276037297	1.808596	1.28E-05	hypermethylated
cg20754261	0.0986	0.345387027	1.808554	1.44E-05	hypermethylated
cg06617456	0.1008	0.352984865	1.808111	6.62E-05	hypermethylated
cg24863335	0.09794	0.342969189	1.808109	8.83E-06	hypermethylated
cg17241776	0.12847	0.449815135	1.807901	6.66E-06	hypermethylated
cg18343437	0.11432	0.400176216	1.807558	7.72E-06	hypermethylated
cg19714279	0.08025	0.280893514	1.80745	2.32E-05	hypermethylated
cg02914422	0.09648	0.337678378	1.807348	8.63E-05	hypermethylated
cg22634378	0.08265	0.289232973	1.807145	0.000195	hypermethylated
cg09693004	0.0593	0.20746	1.806729	5.28E-06	hypermethylated
cg08605326	0.10894	0.38104	1.806409	4.15E-05	hypermethylated
cg12764034	0.1238	0.432961081	1.806226	5.00E-06	hypermethylated
cg24104241	0.08623	0.301531351	1.806046	9.31E-06	hypermethylated
cg04141813	0.08261	0.288862703	1.805996	0.000313	hypermethylated
cg01944044	0.08013	0.280187568	1.805979	3.00E-05	hypermethylated
cg03609960	0.1125	0.393346486	1.805876	1.42E-05	hypermethylated
cg23505303	0.07255	0.253630811	1.805682	1.39E-05	hypermethylated
cg27318318	0.05254	0.183665405	1.805592	1.31E-05	hypermethylated
cg00480389	0.08989	0.314225946	1.80557	1.18E-05	hypermethylated

cg09054633	0.11797	0.412328649	1.805375	3.59E-06	hypermethylated
cg10524033	0.08668	0.302921622	1.805173	4.36E-05	hypermethylated
cg12754421	0.11527	0.402826486	1.805141	1.14E-05	hypermethylated
cg22891500	0.08331	0.291041622	1.804664	5.32E-05	hypermethylated
cg08319991	0.10041	0.35071027	1.804377	0.000142	hypermethylated
cg12212311	0.11506	0.401841081	1.804239	3.90E-06	hypermethylated
cg13454407	0.07114	0.248358378	1.803691	8.42E-05	hypermethylated
cg23726526	0.07007	0.244515135	1.803055	0.000123	hypermethylated
cg24234899	0.09482	0.330871892	1.803009	0.000335	hypermethylated
cg11557636	0.08982	0.313387568	1.802839	7.29E-05	hypermethylated
cg20375342	0.08015	0.279577297	1.802473	6.01E-05	hypermethylated
cg06288154	0.06099	0.212675135	1.802007	0.000354	hypermethylated
cg14419975	0.11618	0.404961081	1.801422	0.000268	hypermethylated
cg25017620	0.10194	0.355230811	1.801036	1.39E-05	hypermethylated
cg14638883	0.09147	0.318598378	1.800368	0.000313	hypermethylated
cg05522774	0.08406	0.292769189	1.800272	0.00017	hypermethylated
cg14991487	0.10257	0.357197838	1.800115	2.32E-06	hypermethylated
cg17384889	0.07709	0.268283784	1.799144	2.42E-06	hypermethylated
cg26263234	0.11628	0.404651351	1.799076	1.33E-05	hypermethylated
cg08806153	0.05908	0.20558	1.798958	1.73E-05	hypermethylated
cg17305181	0.07278	0.253187027	1.79859	4.88E-05	hypermethylated
cg11651237	0.10038	0.349132973	1.798305	3.32E-05	hypermethylated
cg19370054	0.09877	0.343436216	1.797897	5.28E-06	hypermethylated
cg14526718	0.09544	0.331796216	1.797631	3.40E-05	hypermethylated
cg20406460	0.10579	0.367751351	1.797527	5.58E-06	hypermethylated
cg27630311	0.06287	0.218536216	1.797429	0.000234	hypermethylated
cg23475625	0.0837	0.290771351	1.796586	2.30E-05	hypermethylated
cg13834623	0.07642	0.265427568	1.796296	0.000174	hypermethylated
cg02878244	0.12201	0.423699459	1.796042	8.15E-06	hypermethylated
cg17713613	0.10321	0.358367568	1.795857	4.80E-06	hypermethylated
cg20113824	0.11323	0.392916757	1.794967	2.60E-06	hypermethylated
cg18018027	0.06797	0.235842162	1.794852	9.57E-06	hypermethylated
cg26984343	0.07811	0.270985946	1.794639	1.31E-05	hypermethylated
cg15768103	0.10665	0.3699	1.794251	1.42E-05	hypermethylated
cg12128017	0.08651	0.299982703	1.793941	5.58E-05	hypermethylated
cg07675169	0.06907	0.239457297	1.793637	1.62E-05	hypermethylated
cg05181041	0.10402	0.360519459	1.793216	1.46E-05	hypermethylated
cg26035366	0.09598	0.332638919	1.793151	2.92E-05	hypermethylated
cg05680531	0.07839	0.271635676	1.792931	9.27E-05	hypermethylated
cg22367191	0.1031	0.357077297	1.792192	1.93E-06	hypermethylated
cg07291445	0.11233	0.389017838	1.792093	4.42E-06	hypermethylated
cg19922137	0.13114	0.454135676	1.792016	7.42E-06	hypermethylated
cg06274159	0.12238	0.423755676	1.791865	3.07E-05	hypermethylated
cg12133498	0.09308	0.322295135	1.791839	4.86E-06	hypermethylated
cg21054521	0.11649	0.40328	1.791576	1.09E-05	hypermethylated
cg24042242	0.10918	0.377895676	1.791279	3.40E-05	hypermethylated
cg00074145	0.11755	0.406861622	1.791264	4.36E-05	hypermethylated
cg11016563	0.07793	0.269667568	1.790931	3.04E-05	hypermethylated
cg00017221	0.1054	0.364644865	1.790617	8.22E-05	hypermethylated
cg10067970	0.094	0.325201622	1.790602	0.000132	hypermethylated
cg27167224	0.06271	0.216936216	1.790503	0.000115	hypermethylated
cg09516476	0.0749	0.259082703	1.790375	7.72E-06	hypermethylated
cg00379720	0.12107	0.418741081	1.790217	1.12E-05	hypermethylated
cg06808751	0.09848	0.340552432	1.789974	0.000193	hypermethylated
cg06658468	0.10178	0.35184	1.789465	3.00E-05	hypermethylated
cg01972418	0.10561	0.364956216	1.788977	1.04E-05	hypermethylated
cg13952556	0.07212	0.249220541	1.788952	1.21E-05	hypermethylated
cg14979301	0.1061	0.36664	1.78894	0.000102	hypermethylated

cg21699044	0.10866	0.37545027	1.788801	6.05E-06	hypermethylated
cg12595013	0.11669	0.403176216	1.78873	2.29E-06	hypermethylated
cg10486998	0.08426	0.29108	1.788496	3.67E-05	hypermethylated
cg05369857	0.12839	0.443482703	1.788345	4.94E-05	hypermethylated
cg07183447	0.07572	0.26153027	1.788232	4.36E-06	hypermethylated
cg11147786	0.06986	0.241277838	1.788157	3.04E-06	hypermethylated
cg11384848	0.08128	0.2806	1.787543	2.61E-05	hypermethylated
cg13824555	0.06334	0.218641622	1.787379	2.54E-05	hypermethylated
cg14384532	0.11255	0.388508108	1.787379	6.05E-06	hypermethylated
cg02954212	0.08232	0.284065946	1.786911	0.000293	hypermethylated
cg00024280	0.07914	0.272994595	1.786393	2.85E-05	hypermethylated
cg02019774	0.07303	0.251902703	1.786305	6.01E-05	hypermethylated
cg23695133	0.10648	0.36718973	1.785943	5.06E-05	hypermethylated
cg02266732	0.08522	0.293872432	1.785926	4.24E-06	hypermethylated
cg04763554	0.09927	0.342188649	1.785362	0.000142	hypermethylated
cg02937674	0.08495	0.292815676	1.785307	3.95E-05	hypermethylated
cg03012170	0.05331	0.183678919	1.784708	7.65E-05	hypermethylated
cg02119792	0.1453	0.500422703	1.784113	1.54E-05	hypermethylated
cg19269666	0.07852	0.270418378	1.784061	4.31E-05	hypermethylated
cg03730428	0.11811	0.406722162	1.783912	1.39E-05	hypermethylated
cg07733257	0.09903	0.341010811	1.78388	2.32E-05	hypermethylated
cg09420439	0.09653	0.332310811	1.783484	0.000725	hypermethylated
cg13519035	0.11779	0.405431351	1.783241	2.21E-05	hypermethylated
cg07573727	0.10059	0.346152973	1.782923	0.000195	hypermethylated
cg23348270	0.12215	0.420117838	1.78214	1.39E-05	hypermethylated
cg07651242	0.08975	0.308634054	1.781913	8.63E-05	hypermethylated
cg24892966	0.11295	0.388396216	1.781845	1.31E-05	hypermethylated
cg17987176	0.08897	0.305927568	1.781799	6.13E-06	hypermethylated
cg22876812	0.09618	0.330593514	1.78125	8.63E-05	hypermethylated
cg06201642	0.06974	0.23968973	1.78111	6.62E-05	hypermethylated
cg03882242	0.11355	0.390073514	1.780418	1.73E-05	hypermethylated
cg05327864	0.09783	0.335958378	1.779934	2.48E-05	hypermethylated
cg26836233	0.10265	0.352431892	1.779611	8.83E-06	hypermethylated
cg01082843	0.07983	0.274028649	1.779324	9.49E-05	hypermethylated
cg10836101	0.10369	0.355523243	1.777667	8.37E-06	hypermethylated
cg13974192	0.12675	0.434545946	1.777523	2.64E-06	hypermethylated
cg11980129	0.09134	0.313130811	1.777447	1.66E-05	hypermethylated
cg25514273	0.11088	0.380061622	1.777234	1.54E-05	hypermethylated
cg05031521	0.07695	0.263615135	1.77644	0.00032	hypermethylated
cg05864326	0.12356	0.423247027	1.776288	1.56E-05	hypermethylated
cg18290624	0.10128	0.346798919	1.77575	9.96E-06	hypermethylated
cg24845274	0.06343	0.217191892	1.775733	1.11E-05	hypermethylated
cg03738025	0.14754	0.505092973	1.775443	3.12E-06	hypermethylated
cg21384645	0.06728	0.230300541	1.775268	5.72E-05	hypermethylated
cg01627823	0.11301	0.38682973	1.775248	3.15E-05	hypermethylated
cg01388649	0.07837	0.26804	1.774075	3.15E-05	hypermethylated
cg09968620	0.07672	0.262229189	1.773154	1.23E-05	hypermethylated
cg02986266	0.08538	0.291809189	1.773055	1.02E-05	hypermethylated
cg17371081	0.12709	0.434349189	1.773005	3.85E-05	hypermethylated
cg16494192	0.10306	0.352129189	1.77262	4.86E-06	hypermethylated
cg07150062	0.11677	0.398783243	1.771935	1.71E-05	hypermethylated
cg22289434	0.08816	0.301029189	1.771707	9.07E-06	hypermethylated
cg06619938	0.06383	0.217945946	1.771664	5.45E-05	hypermethylated
cg13612083	0.05231	0.178488649	1.770674	7.38E-05	hypermethylated
cg09418063	0.08776	0.299404865	1.770462	0.00012	hypermethylated
cg16332936	0.06877	0.234555135	1.770076	0.000137	hypermethylated
cg09515953	0.10323	0.352004324	1.769731	3.90E-05	hypermethylated
cg24978630	0.09792	0.333783784	1.769238	1.94E-05	hypermethylated

cg03699182	0.10415	0.35492	1.768831	0.001684	hypermethylated
cg05669418	0.0733	0.249735135	1.768514	8.15E-06	hypermethylated
cg18877506	0.0957	0.325966486	1.768133	7.72E-06	hypermethylated
cg09382096	0.07913	0.269511892	1.768052	1.94E-05	hypermethylated
cg20585869	0.07716	0.26276973	1.767874	3.00E-05	hypermethylated
cg23097534	0.08293	0.282371351	1.767628	4.82E-05	hypermethylated
cg00332021	0.07598	0.258619459	1.767139	2.10E-05	hypermethylated
cg26344227	0.13696	0.46606	1.766761	9.57E-06	hypermethylated
cg21899596	0.16172	0.550252432	1.766596	4.01E-06	hypermethylated
cg26925231	0.10494	0.35690973	1.765995	1.46E-05	hypermethylated
cg26104297	0.0891	0.302869189	1.765197	9.07E-06	hypermethylated
cg15427886	0.13854	0.47082973	1.764903	3.79E-06	hypermethylated
cg07960450	0.08319	0.282683243	1.764704	0.0002	hypermethylated
cg04453180	0.08488	0.288352973	1.764339	8.52E-05	hypermethylated
cg16529477	0.13599	0.461954054	1.764249	5.43E-06	hypermethylated
cg07197785	0.12132	0.412062162	1.764045	4.05E-05	hypermethylated
cg04062391	0.08199	0.278438919	1.763841	2.21E-05	hypermethylated
cg15950068	0.10341	0.351131892	1.763637	4.10E-05	hypermethylated
cg12306195	0.09609	0.326244865	1.763497	7.12E-06	hypermethylated
cg26701242	0.09086	0.308438919	1.763268	4.24E-06	hypermethylated
cg04131898	0.10686	0.362742162	1.763223	9.57E-06	hypermethylated
cg10056132	0.09777	0.331816757	1.762923	8.37E-06	hypermethylated
cg20649017	0.10997	0.372882703	1.761612	3.53E-05	hypermethylated
cg02578368	0.09543	0.323580541	1.76161	0.003773	hypermethylated
cg26468007	0.08727	0.295884865	1.761478	8.73E-05	hypermethylated
cg08034077	0.10505	0.356158919	1.761445	2.32E-05	hypermethylated
cg04773818	0.11479	0.38913027	1.761256	0.000113	hypermethylated
cg25684999	0.12173	0.412578919	1.760985	9.57E-06	hypermethylated
cg07246273	0.12097	0.409923784	1.760706	4.60E-06	hypermethylated
cg22620221	0.12453	0.421937838	1.760537	6.75E-06	hypermethylated
cg17486097	0.09815	0.332474054	1.760182	1.37E-05	hypermethylated
cg00209038	0.10138	0.343263243	1.759542	0.000109	hypermethylated
cg17336584	0.091	0.308058378	1.759265	2.85E-05	hypermethylated
cg14750277	0.09639	0.326277838	1.759146	9.49E-05	hypermethylated
cg26124318	0.08697	0.29439027	1.75914	0.000124	hypermethylated
cg11877129	0.09001	0.304669189	1.759086	1.75E-05	hypermethylated
cg18587340	0.08436	0.285461081	1.758663	2.92E-05	hypermethylated
cg16459364	0.09768	0.330317297	1.757717	6.75E-06	hypermethylated
cg19811761	0.09747	0.329578378	1.757591	7.29E-05	hypermethylated
cg12122146	0.13836	0.467716216	1.757207	9.19E-06	hypermethylated
cg14442421	0.07663	0.259016216	1.757061	1.94E-05	hypermethylated
cg18217632	0.06118	0.206788108	1.757021	1.75E-05	hypermethylated
cg06793562	0.1155	0.390314595	1.756745	1.27E-06	hypermethylated
cg14823763	0.06997	0.236441622	1.756676	4.24E-06	hypermethylated
cg22837767	0.09546	0.322540541	1.756512	5.65E-05	hypermethylated
cg19993589	0.08374	0.282931351	1.756463	3.67E-05	hypermethylated
cg15874092	0.1163	0.392848649	1.756123	1.75E-05	hypermethylated
cg17123690	0.11995	0.405141081	1.755991	8.10E-07	hypermethylated
cg19760241	0.15645	0.528302703	1.755663	1.54E-06	hypermethylated
cg08826251	0.08949	0.302182703	1.755623	6.86E-05	hypermethylated
cg17796010	0.08373	0.282626486	1.75508	2.51E-05	hypermethylated
cg12307484	0.13534	0.456814595	1.75502	3.12E-06	hypermethylated
cg11412466	0.07907	0.266860541	1.754884	2.13E-05	hypermethylated
cg02091605	0.10258	0.346192432	1.754825	2.92E-05	hypermethylated
cg00235367	0.08429	0.284322162	1.754093	1.05E-05	hypermethylated
cg13954457	0.12043	0.406114054	1.75369	3.23E-05	hypermethylated
cg09913956	0.07604	0.256247027	1.752705	1.84E-05	hypermethylated
cg12591770	0.09357	0.315311351	1.752659	4.36E-06	hypermethylated

cg06991300	0.12059	0.406166486	1.751961	2.81E-05	hypermethylated
cg02348151	0.08038	0.270669189	1.751622	3.32E-05	hypermethylated
cg13117164	0.08755	0.294677297	1.750957	5.25E-05	hypermethylated
cg10996058	0.07564	0.254565405	1.750815	0.000159	hypermethylated
cg09797337	0.13042	0.438918378	1.750788	2.79E-06	hypermethylated
cg01775414	0.08581	0.288777297	1.75074	0.000112	hypermethylated
cg20168230	0.11441	0.385002162	1.750653	9.07E-06	hypermethylated
cg03229780	0.12066	0.405976216	1.750448	5.00E-06	hypermethylated
cg16711792	0.10749	0.361580541	1.750115	2.32E-05	hypermethylated
cg09477453	0.08801	0.296016216	1.749937	7.72E-06	hypermethylated
cg16018474	0.09103	0.306172973	1.749933	4.36E-05	hypermethylated
cg07788369	0.12685	0.426629189	1.749859	1.67E-06	hypermethylated
cg13842237	0.07364	0.247553514	1.749179	8.95E-06	hypermethylated
cg07800665	0.12425	0.417623243	1.748956	1.50E-05	hypermethylated
cg24856726	0.11519	0.38694973	1.748131	1.58E-05	hypermethylated
cg26132320	0.12041	0.404337838	1.747606	1.25E-05	hypermethylated
cg17910121	0.10899	0.365969189	1.747526	2.85E-05	hypermethylated
cg18468354	0.13361	0.448561081	1.747276	5.14E-06	hypermethylated
cg18148997	0.07	0.234990811	1.747178	3.95E-05	hypermethylated
cg07217924	0.06835	0.229407027	1.746896	1.97E-05	hypermethylated
cg10791959	0.11932	0.400445405	1.74677	1.37E-05	hypermethylated
cg01818151	0.07164	0.240416757	1.7467	1.77E-05	hypermethylated
cg26239984	0.09524	0.319589189	1.746579	1.64E-05	hypermethylated
cg25478600	0.07635	0.256121622	1.746129	1.04E-05	hypermethylated
cg05438320	0.09391	0.315001622	1.746009	6.01E-05	hypermethylated
cg21433231	0.0925	0.310202162	1.745683	5.58E-06	hypermethylated
cg07850592	0.0739	0.247775676	1.745388	5.79E-05	hypermethylated
cg14845689	0.07869	0.263779459	1.74508	6.39E-06	hypermethylated
cg15161394	0.07865	0.263572432	1.744681	1.29E-06	hypermethylated
cg04224064	0.1499	0.502328108	1.74463	2.36E-06	hypermethylated
cg04223420	0.13514	0.452783784	1.744368	3.54E-06	hypermethylated
cg22572159	0.09781	0.327543784	1.743634	9.95E-05	hypermethylated
cg07744841	0.10031	0.335906486	1.743594	3.36E-05	hypermethylated
cg21776417	0.09665	0.323555135	1.74317	1.89E-05	hypermethylated
cg11235498	0.09155	0.306471351	1.74312	0.000335	hypermethylated
cg20000562	0.08229	0.275361622	1.742538	5.72E-05	hypermethylated
cg02155884	0.07699	0.257619459	1.742499	3.67E-05	hypermethylated
cg17362052	0.08556	0.286280541	1.742421	2.27E-05	hypermethylated
cg02066682	0.06287	0.210359459	1.742413	1.54E-05	hypermethylated
cg13959523	0.08493	0.284107568	1.742091	1.01E-05	hypermethylated
cg18096722	0.09537	0.319028649	1.742079	5.21E-06	hypermethylated
cg00376022	0.06266	0.209577838	1.741869	2.35E-05	hypermethylated
cg11856542	0.08269	0.276528649	1.741644	1.39E-05	hypermethylated
cg02571816	0.12247	0.409477297	1.741355	1.66E-05	hypermethylated
cg07103129	0.12252	0.409637838	1.741332	1.01E-05	hypermethylated
cg00998387	0.09779	0.32693027	1.741224	6.01E-05	hypermethylated
cg02969141	0.12782	0.427216216	1.740853	9.31E-06	hypermethylated
cg11818631	0.10503	0.350948108	1.740456	2.64E-05	hypermethylated
cg24008884	0.06977	0.233037297	1.739882	4.36E-05	hypermethylated
cg23294090	0.09915	0.331027568	1.739267	0.000265	hypermethylated
cg07691152	0.12783	0.426615676	1.738711	1.08E-05	hypermethylated
cg20945566	0.1293	0.431391351	1.738275	2.48E-05	hypermethylated
cg12300353	0.093	0.310234054	1.738054	0.000245	hypermethylated
cg16945312	0.11443	0.381673514	1.737874	1.42E-05	hypermethylated
cg16670554	0.12565	0.419087027	1.737839	1.25E-05	hypermethylated
cg04550052	0.11952	0.39846973	1.737218	2.39E-05	hypermethylated
cg00839579	0.14296	0.476565946	1.737064	1.01E-05	hypermethylated
cg03847373	0.09943	0.331436216	1.736978	1.31E-05	hypermethylated

cg03430846	0.10479	0.34923027	1.736678	0.000129	hypermethylated
cg27659787	0.08866	0.295434595	1.736484	1.21E-05	hypermethylated
cg05379302	0.09321	0.310577297	1.736396	1.77E-05	hypermethylated
cg08364561	0.16309	0.543357838	1.736234	1.29E-06	hypermethylated
cg22974982	0.08415	0.280349189	1.73619	2.39E-05	hypermethylated
cg13304274	0.09561	0.318517838	1.736141	5.32E-05	hypermethylated
cg21095561	0.11038	0.367584324	1.735596	1.09E-05	hypermethylated
cg25349154	0.09219	0.306783243	1.734538	3.07E-05	hypermethylated
cg23651826	0.13763	0.457981622	1.734495	5.14E-06	hypermethylated
cg05310990	0.1037	0.34507027	1.734474	2.74E-05	hypermethylated
cg26649384	0.10298	0.34265027	1.734373	0.000256	hypermethylated
cg03045635	0.14303	0.475887027	1.734301	6.05E-06	hypermethylated
cg00963378	0.12141	0.403947568	1.734281	3.15E-05	hypermethylated
cg24660009	0.07229	0.24041027	1.733631	0.000159	hypermethylated
cg24632756	0.08151	0.271067568	1.733604	9.07E-06	hypermethylated
cg23004758	0.12471	0.414603243	1.733154	2.39E-05	hypermethylated
cg11215976	0.13413	0.445864865	1.732975	3.00E-05	hypermethylated
cg09355771	0.06727	0.223564324	1.732655	1.73E-05	hypermethylated
cg01024168	0.08586	0.28534	1.732624	5.79E-05	hypermethylated
cg04486019	0.09668	0.321281081	1.732547	9.82E-06	hypermethylated
cg19560710	0.07705	0.256047027	1.732542	0.000126	hypermethylated
cg05212802	0.06572	0.218363784	1.732329	7.84E-05	hypermethylated
cg26560110	0.06235	0.207166486	1.732329	2.32E-05	hypermethylated
cg08676249	0.08942	0.297035676	1.731967	9.69E-06	hypermethylated
cg05940984	0.09131	0.303311892	1.731957	8.03E-05	hypermethylated
cg11727383	0.10977	0.364537838	1.731585	5.31E-05	hypermethylated
cg02933679	0.08249	0.273936216	1.731549	9.07E-06	hypermethylated
cg10603275	0.14214	0.47201027	1.731506	5.72E-05	hypermethylated
cg12071328	0.12137	0.402995135	1.731351	6.46E-05	hypermethylated
cg26730369	0.1012	0.336021081	1.731342	2.15E-05	hypermethylated
cg19811087	0.06989	0.231861081	1.730103	7.72E-06	hypermethylated
cg08116462	0.09914	0.328805405	1.729695	5.73E-06	hypermethylated
cg12306414	0.09542	0.316414595	1.729453	0.000229	hypermethylated
cg01874697	0.09907	0.328499459	1.729371	8.71E-06	hypermethylated
cg05475524	0.10827	0.358985405	1.729292	0.000123	hypermethylated
cg09887059	0.1484	0.492030811	1.729258	5.58E-06	hypermethylated
cg25664338	0.07345	0.243503243	1.729107	0.000195	hypermethylated
cg19461621	0.10802	0.358022162	1.72875	3.95E-05	hypermethylated
cg03171770	0.14205	0.470761081	1.728596	7.93E-06	hypermethylated
cg20924470	0.084	0.278243784	1.727888	2.61E-05	hypermethylated
cg27454136	0.10646	0.352615135	1.727783	0.000165	hypermethylated
cg13713830	0.08944	0.296154054	1.727356	2.51E-05	hypermethylated
cg05484788	0.08331	0.275842703	1.727284	5.25E-05	hypermethylated
cg21183256	0.13478	0.445918378	1.726173	2.32E-05	hypermethylated
cg01610488	0.09406	0.311160541	1.726006	7.65E-05	hypermethylated
cg14977066	0.09241	0.305671892	1.725863	1.97E-05	hypermethylated
cg13718418	0.06735	0.22266	1.725093	6.62E-05	hypermethylated
cg02942845	0.08683	0.287042703	1.725	5.45E-05	hypermethylated
cg00912625	0.09961	0.329260541	1.724867	1.73E-05	hypermethylated
cg15980539	0.09636	0.318407568	1.724368	7.84E-05	hypermethylated
cg25103492	0.07503	0.247888108	1.72415	8.22E-05	hypermethylated
cg14345497	0.08164	0.269721622	1.724123	1.87E-05	hypermethylated
cg22557091	0.09385	0.310015676	1.723913	1.92E-05	hypermethylated
cg23799901	0.09239	0.305182703	1.723865	8.22E-05	hypermethylated
cg23291280	0.12502	0.412934595	1.723754	3.04E-06	hypermethylated
cg04131583	0.08764	0.289463243	1.723719	2.71E-05	hypermethylated
cg02990869	0.09369	0.309442162	1.723703	2.78E-05	hypermethylated
cg09579310	0.08938	0.295204324	1.72369	2.21E-05	hypermethylated

cg23153481	0.08204	0.270878378	1.723246	1.04E-05	hypermethylated
cg10151685	0.1065	0.351575676	1.722982	2.45E-05	hypermethylated
cg17403609	0.12173	0.401698378	1.722428	3.79E-06	hypermethylated
cg26074603	0.13756	0.453797297	1.721987	1.94E-05	hypermethylated
cg17152757	0.09558	0.31525027	1.721717	6.05E-06	hypermethylated
cg09465698	0.11958	0.394339459	1.721462	1.12E-05	hypermethylated
cg09430118	0.10976	0.361874595	1.721137	3.49E-06	hypermethylated
cg11073558	0.11877	0.39148973	1.720804	2.51E-05	hypermethylated
cg00162231	0.11068	0.364794054	1.720688	2.10E-05	hypermethylated
cg25308742	0.08101	0.267000541	1.720671	3.69E-06	hypermethylated
cg13492692	0.07485	0.246685405	1.720598	1.39E-05	hypermethylated
cg26736812	0.09965	0.328340541	1.720251	4.36E-06	hypermethylated
cg26422458	0.13232	0.435970811	1.7202	1.75E-05	hypermethylated
cg17093995	0.08868	0.292164865	1.720102	0.000253	hypermethylated
cg17474338	0.08692	0.286276216	1.719648	5.45E-05	hypermethylated
cg03232620	0.11293	0.371732432	1.718836	2.92E-05	hypermethylated
cg24226973	0.08291	0.272905946	1.718786	4.82E-05	hypermethylated
cg05062333	0.08724	0.287038919	1.718185	4.47E-05	hypermethylated
cg02355885	0.09349	0.307602162	1.718182	7.12E-06	hypermethylated
cg10751726	0.0739	0.243138919	1.718135	0.000262	hypermethylated
cg15515258	0.09868	0.324595676	1.717814	0.000101	hypermethylated
cg18557131	0.09829	0.323069189	1.716727	1.09E-05	hypermethylated
cg05295006	0.10456	0.343653514	1.716624	1.31E-05	hypermethylated
cg00292701	0.0718	0.235862162	1.715888	6.84E-06	hypermethylated
cg17827020	0.08025	0.263588649	1.715715	9.82E-06	hypermethylated
cg11841394	0.08849	0.290628649	1.715591	5.65E-05	hypermethylated
cg23657185	0.09409	0.308933514	1.715183	7.84E-05	hypermethylated
cg21124497	0.10385	0.340936216	1.715001	3.62E-05	hypermethylated
cg17966167	0.0845	0.277386486	1.714874	4.70E-05	hypermethylated
cg23300372	0.08852	0.290557838	1.71475	4.82E-05	hypermethylated
cg01461840	0.07597	0.249289189	1.714319	4.73E-06	hypermethylated
cg05048377	0.11427	0.374755135	1.713502	3.67E-05	hypermethylated
cg11144641	0.09509	0.311787027	1.713195	1.80E-05	hypermethylated
cg17891759	0.09421	0.308814054	1.712786	1.28E-05	hypermethylated
cg04910921	0.11206	0.367315676	1.712749	3.12E-06	hypermethylated
cg03775422	0.08766	0.287319459	1.712665	8.03E-05	hypermethylated
cg24645214	0.09736	0.31895027	1.71193	0.000271	hypermethylated
cg04922810	0.08139	0.266601081	1.711759	2.53E-06	hypermethylated
cg01448276	0.09308	0.304877838	1.711688	1.58E-05	hypermethylated
cg24611446	0.10791	0.353400541	1.711476	8.15E-06	hypermethylated
cg07224726	0.0955	0.312658378	1.711015	1.42E-05	hypermethylated
cg02388865	0.11934	0.39066973	1.710872	9.66E-07	hypermethylated
cg17400476	0.10671	0.349267027	1.710635	8.22E-05	hypermethylated
cg08718490	0.1404	0.459477838	1.710452	1.22E-06	hypermethylated
cg20611911	0.1113	0.363995676	1.709468	2.32E-05	hypermethylated
cg08499046	0.1413	0.461839459	1.70863	1.64E-05	hypermethylated
cg05575614	0.1234	0.403282162	1.708447	4.48E-06	hypermethylated
cg10591652	0.06352	0.207567568	1.708298	6.95E-05	hypermethylated
cg04617948	0.09415	0.307536757	1.707726	1.04E-05	hypermethylated
cg12917718	0.0803	0.262257838	1.707514	3.90E-05	hypermethylated
cg01612730	0.06634	0.216661622	1.707493	3.23E-05	hypermethylated
cg02345991	0.08821	0.288023243	1.707171	0.000204	hypermethylated
cg09014354	0.10134	0.330892973	1.707161	9.05E-05	hypermethylated
cg27473895	0.10083	0.329182703	1.706964	3.95E-05	hypermethylated
cg26593267	0.08649	0.282354054	1.7069	7.56E-05	hypermethylated
cg04490714	0.09545	0.311563243	1.706708	2.45E-05	hypermethylated
cg22795470	0.09771	0.318917838	1.706607	4.67E-06	hypermethylated
cg21816308	0.10358	0.338073514	1.706592	0.000117	hypermethylated

cg27170782	0.10842	0.35382973	1.706424	5.65E-05	hypermethylated
cg10526277	0.12004	0.391715676	1.706292	5.00E-06	hypermethylated
cg23696752	0.09862	0.321741622	1.70595	1.48E-05	hypermethylated
cg12525474	0.09212	0.30047027	1.705636	3.76E-05	hypermethylated
cg11325970	0.09155	0.298466486	1.704937	1.99E-05	hypermethylated
cg27416372	0.10688	0.348356757	1.704574	6.84E-06	hypermethylated
cg11873482	0.10983	0.357939459	1.704443	0.000129	hypermethylated
cg24189904	0.10293	0.335437838	1.704382	2.16E-06	hypermethylated
cg18034737	0.12989	0.423288649	1.704351	1.28E-05	hypermethylated
cg24889512	0.09921	0.323284865	1.704249	1.25E-05	hypermethylated
cg04431946	0.12367	0.402824865	1.703657	5.93E-05	hypermethylated
cg13033990	0.08059	0.262487027	1.703573	3.07E-05	hypermethylated
cg08448589	0.09048	0.294695676	1.703555	2.78E-05	hypermethylated
cg06272611	0.0889	0.289517297	1.703394	1.99E-05	hypermethylated
cg10552126	0.10817	0.352234054	1.703234	1.52E-05	hypermethylated
cg22946150	0.09705	0.315982162	1.703043	0.00064	hypermethylated
cg12880658	0.09961	0.324259459	1.702786	1.15E-05	hypermethylated
cg14602530	0.10581	0.344431351	1.70274	9.31E-06	hypermethylated
cg04499648	0.08625	0.280654054	1.702197	7.93E-05	hypermethylated
cg06759374	0.06869	0.223445946	1.701754	1.39E-05	hypermethylated
cg09614415	0.12809	0.416311351	1.700505	6.57E-06	hypermethylated
cg16065021	0.1302	0.423141622	1.700411	1.50E-05	hypermethylated
cg11701471	0.10639	0.345627568	1.699856	0.000517	hypermethylated
cg19974854	0.07729	0.25108973	1.699849	5.97E-06	hypermethylated
cg15638709	0.14554	0.472704865	1.699524	1.46E-05	hypermethylated
cg23684973	0.08793	0.285572432	1.699429	9.05E-05	hypermethylated
cg25567594	0.12401	0.402695676	1.699234	1.06E-05	hypermethylated
cg24116870	0.09448	0.306684865	1.698676	5.35E-06	hypermethylated
cg07171111	0.10055	0.326343784	1.698479	4.01E-06	hypermethylated
cg07850418	0.09882	0.320591351	1.697861	5.58E-06	hypermethylated
cg00396667	0.11412	0.370068108	1.697239	1.09E-05	hypermethylated
cg08211028	0.11088	0.35954973	1.697192	4.58E-05	hypermethylated
cg03175653	0.09475	0.307241622	1.697176	3.69E-06	hypermethylated
cg20574415	0.06913	0.224134054	1.696978	6.31E-05	hypermethylated
cg24563570	0.0643	0.208425946	1.696644	3.76E-05	hypermethylated
cg11580948	0.08004	0.259424865	1.696524	2.89E-05	hypermethylated
cg04534926	0.07692	0.249304324	1.696477	9.05E-05	hypermethylated
cg03790250	0.11682	0.378522162	1.69609	1.01E-05	hypermethylated
cg09311683	0.08488	0.275012973	1.696003	3.44E-05	hypermethylated
cg02759846	0.08226	0.266516757	1.695963	2.02E-05	hypermethylated
cg18698788	0.11624	0.376499459	1.695541	0.000805	hypermethylated
cg10362591	0.11412	0.369554054	1.695234	4.58E-05	hypermethylated
cg21548155	0.11925	0.386112432	1.695032	1.28E-05	hypermethylated
cg25987194	0.07918	0.256347568	1.694893	0.000283	hypermethylated
cg19047707	0.10856	0.351354054	1.694433	2.15E-05	hypermethylated
cg18239753	0.10388	0.336115676	1.69404	3.85E-05	hypermethylated
cg01366419	0.10467	0.338660541	1.693992	1.21E-05	hypermethylated
cg25371634	0.09036	0.292336757	1.693875	2.85E-05	hypermethylated
cg13566648	0.0776	0.251051351	1.693854	2.27E-05	hypermethylated
cg27032232	0.13965	0.451788649	1.693832	1.58E-05	hypermethylated
cg12268575	0.08627	0.279036216	1.693522	2.24E-05	hypermethylated
cg14001664	0.14789	0.478294595	1.693375	2.29E-06	hypermethylated
cg19771465	0.07475	0.241724324	1.693217	1.46E-05	hypermethylated
cg07920503	0.13773	0.445381081	1.693197	0.000159	hypermethylated
cg19623360	0.0683	0.22082	1.692913	1.77E-05	hypermethylated
cg04553838	0.07835	0.253288649	1.692777	2.51E-05	hypermethylated
cg14583673	0.0861	0.278318919	1.692654	3.40E-05	hypermethylated
cg00157668	0.12094	0.390935676	1.69264	2.71E-05	hypermethylated

cg26390889	0.09332	0.301650811	1.692621	4.58E-05	hypermethylated
cg27634876	0.07723	0.249640541	1.692619	2.92E-05	hypermethylated
cg09936426	0.124	0.400581081	1.691754	9.96E-06	hypermethylated
cg01210554	0.07089	0.229002703	1.691711	1.46E-05	hypermethylated
cg00582316	0.08462	0.273352432	1.691692	0.000245	hypermethylated
cg22643811	0.10692	0.345380541	1.691655	9.07E-06	hypermethylated
cg17727929	0.08645	0.27922973	1.691515	1.20E-05	hypermethylated
cg14875171	0.13954	0.450663243	1.691371	3.44E-06	hypermethylated
cg07819944	0.12826	0.413751892	1.689695	6.46E-05	hypermethylated
cg08159291	0.11375	0.366896216	1.689505	5.28E-06	hypermethylated
cg04651781	0.10919	0.352127027	1.689255	6.75E-06	hypermethylated
cg14714797	0.10754	0.34676973	1.689105	2.57E-05	hypermethylated
cg03981954	0.09655	0.311212973	1.688554	9.95E-05	hypermethylated
cg10172669	0.0899	0.289680541	1.68807	3.39E-06	hypermethylated
cg11308840	0.06548	0.210911892	1.687514	1.46E-05	hypermethylated
cg00663972	0.08824	0.284176216	1.687281	6.39E-06	hypermethylated
cg25406138	0.09092	0.292705946	1.686782	1.64E-05	hypermethylated
cg26656135	0.0967	0.311267027	1.686565	6.31E-05	hypermethylated
cg15092343	0.16706	0.537655676	1.686316	4.86E-06	hypermethylated
cg27032146	0.1327	0.426743243	1.6852	6.13E-06	hypermethylated
cg10778441	0.08697	0.279655676	1.685062	9.82E-06	hypermethylated
cg08430329	0.08829	0.28389027	1.685011	0.000135	hypermethylated
cg04206351	0.09932	0.319307027	1.684788	2.45E-05	hypermethylated
cg24809973	0.14476	0.465387568	1.68477	1.82E-06	hypermethylated
cg08959216	0.10524	0.338314054	1.68468	6.62E-05	hypermethylated
cg27011060	0.10153	0.326292432	1.684259	0.000354	hypermethylated
cg26911220	0.09955	0.319908649	1.684167	0.000106	hypermethylated
cg04328762	0.10127	0.325379459	1.683916	0.000667	hypermethylated
cg24089600	0.12347	0.396619459	1.683595	2.92E-05	hypermethylated
cg03893271	0.1151	0.369703243	1.68348	1.09E-05	hypermethylated
cg08165221	0.09351	0.300290811	1.683168	7.72E-06	hypermethylated
cg16269733	0.13319	0.427548649	1.682603	1.04E-05	hypermethylated
cg18940047	0.12345	0.396251892	1.682491	6.66E-06	hypermethylated
cg14754787	0.16962	0.544285405	1.682057	1.93E-06	hypermethylated
cg09336320	0.0997	0.319858378	1.681768	3.85E-05	hypermethylated
cg24436715	0.13306	0.426708108	1.681173	1.15E-06	hypermethylated
cg25537993	0.11487	0.368369189	1.68115	2.21E-05	hypermethylated
cg01105140	0.09757	0.312813514	1.680793	1.62E-05	hypermethylated
cg05992357	0.07423	0.237965946	1.680681	5.00E-05	hypermethylated
cg07382554	0.08761	0.280811892	1.680437	3.49E-06	hypermethylated
cg06679720	0.10669	0.341954054	1.680378	6.93E-06	hypermethylated
cg20095233	0.09484	0.303928649	1.680165	6.95E-05	hypermethylated
cg06213468	0.07592	0.243228649	1.679761	1.99E-05	hypermethylated
cg01495122	0.11648	0.373109189	1.679516	3.85E-05	hypermethylated
cg15699226	0.10483	0.335762162	1.679388	3.59E-06	hypermethylated
cg26931862	0.11928	0.382044324	1.679388	4.60E-06	hypermethylated
cg15343119	0.0967	0.309680541	1.679193	2.15E-05	hypermethylated
cg25476766	0.08412	0.269389189	1.679171	1.18E-05	hypermethylated
cg01749436	0.08223	0.263302162	1.678983	2.89E-05	hypermethylated
cg22345692	0.07226	0.231363784	1.678894	9.07E-06	hypermethylated
cg26974111	0.07257	0.232343243	1.678813	9.82E-06	hypermethylated
cg02216246	0.10418	0.333512973	1.678665	2.92E-05	hypermethylated
cg16590237	0.0905	0.289673514	1.678438	0.000148	hypermethylated
cg06324785	0.07268	0.232537838	1.677835	1.58E-06	hypermethylated
cg02622052	0.08349	0.267094054	1.677673	0.00017	hypermethylated
cg20498685	0.11295	0.361335676	1.677655	5.06E-05	hypermethylated
cg11554266	0.06857	0.219296757	1.677235	9.07E-06	hypermethylated
cg05350411	0.08599	0.274995135	1.677165	1.43E-06	hypermethylated

cg25976242	0.08616	0.275504324	1.676985	7.84E-05	hypermethylated
cg14131755	0.09843	0.314727568	1.676934	1.02E-05	hypermethylated
cg17524637	0.1204	0.384957297	1.676863	1.09E-05	hypermethylated
cg00296182	0.07801	0.249394054	1.676696	0.000126	hypermethylated
cg20457796	0.09441	0.301741622	1.676302	7.93E-06	hypermethylated
cg07647726	0.07704	0.246158378	1.675907	3.81E-05	hypermethylated
cg02694427	0.11699	0.373762162	1.675735	2.29E-06	hypermethylated
cg07962128	0.08591	0.274314054	1.674931	2.48E-05	hypermethylated
cg18347642	0.11323	0.361442162	1.674509	8.37E-06	hypermethylated
cg03817667	0.12212	0.389732432	1.674184	4.70E-05	hypermethylated
cg10055501	0.13167	0.420150811	1.673981	8.37E-06	hypermethylated
cg02370605	0.0777	0.247888649	1.673706	5.58E-05	hypermethylated
cg26149275	0.11722	0.37391027	1.673473	2.32E-06	hypermethylated
cg11469391	0.0958	0.305567568	1.673394	6.84E-06	hypermethylated
cg25324047	0.09712	0.309762162	1.673321	4.42E-05	hypermethylated
cg25585712	0.0969	0.308847568	1.672326	3.23E-05	hypermethylated
cg11717507	0.11724	0.373664324	1.672278	3.49E-05	hypermethylated
cg05771518	0.07014	0.223510811	1.672035	1.38E-05	hypermethylated
cg13875518	0.08797	0.2803	1.671888	4.15E-05	hypermethylated
cg17312004	0.09259	0.294979459	1.671686	0.000324	hypermethylated
cg05839235	0.0917	0.292131351	1.671624	4.93E-06	hypermethylated
cg23054883	0.09116	0.29022973	1.670722	9.07E-06	hypermethylated
cg21982950	0.11053	0.351730811	1.670034	1.33E-06	hypermethylated
cg26116950	0.12948	0.412012432	1.669959	3.15E-05	hypermethylated
cg26567423	0.12987	0.413234595	1.669893	1.31E-05	hypermethylated
cg05317090	0.14057	0.447135676	1.669424	4.60E-06	hypermethylated
cg07012926	0.08909	0.283338378	1.669191	3.15E-05	hypermethylated
cg09487611	0.08402	0.26720973	1.669168	9.31E-06	hypermethylated
cg04861929	0.11348	0.360835135	1.668902	2.21E-05	hypermethylated
cg23325963	0.11763	0.374003784	1.668797	8.84E-05	hypermethylated
cg04574459	0.1029	0.327135135	1.668644	0.000216	hypermethylated
cg19109538	0.09221	0.293136216	1.668576	8.13E-05	hypermethylated
cg19742055	0.07992	0.254045946	1.668461	5.72E-05	hypermethylated
cg08726446	0.06763	0.21494973	1.668264	1.75E-05	hypermethylated
cg25527090	0.16631	0.528527027	1.668102	2.71E-06	hypermethylated
cg06838365	0.08324	0.264532973	1.668099	4.58E-05	hypermethylated
cg10177394	0.08137	0.258527027	1.667746	9.31E-06	hypermethylated
cg04406454	0.09044	0.287334595	1.667699	4.80E-06	hypermethylated
cg26595643	0.10294	0.327031351	1.667625	1.31E-05	hypermethylated
cg02318567	0.10862	0.345066486	1.667585	9.07E-06	hypermethylated
cg11124364	0.1092	0.346908649	1.667583	8.15E-06	hypermethylated
cg21106486	0.1091	0.346588649	1.667573	1.15E-05	hypermethylated
cg02187214	0.09442	0.299931351	1.667468	8.15E-06	hypermethylated
cg11500797	0.12349	0.392158378	1.667042	4.01E-06	hypermethylated
cg02640041	0.10453	0.331902703	1.666843	1.09E-05	hypermethylated
cg02409351	0.08923	0.283289189	1.666675	2.18E-05	hypermethylated
cg10520887	0.09466	0.300506486	1.666569	8.84E-05	hypermethylated
cg10029842	0.07955	0.252457838	1.666109	1.99E-05	hypermethylated
cg23786625	0.11343	0.35997027	1.666076	1.09E-05	hypermethylated
cg04988476	0.10102	0.320516757	1.665759	4.94E-05	hypermethylated
cg11789740	0.09006	0.285694595	1.665515	2.64E-05	hypermethylated
cg00384539	0.13566	0.430098919	1.664673	5.19E-05	hypermethylated
cg10406295	0.11307	0.358467027	1.664624	6.15E-05	hypermethylated
cg05030680	0.11579	0.367071892	1.664552	4.12E-06	hypermethylated
cg22488256	0.12758	0.404403784	1.664394	1.50E-05	hypermethylated
cg00674679	0.10925	0.346247568	1.664171	2.85E-05	hypermethylated
cg12907702	0.06531	0.206873514	1.663373	2.15E-05	hypermethylated
cg10158541	0.13761	0.435867568	1.663305	7.72E-06	hypermethylated

cg26832142	0.06041	0.19132	1.663128	2.24E-05	hypermethylated
cg25164624	0.10648	0.337103784	1.66261	0.000718	hypermethylated
cg23322933	0.13942	0.44127027	1.662225	6.93E-06	hypermethylated
cg04531425	0.07335	0.232121622	1.662012	2.32E-05	hypermethylated
cg16428251	0.1116	0.353154595	1.661963	6.22E-06	hypermethylated
cg09768178	0.09606	0.303788108	1.661058	1.23E-05	hypermethylated
cg27190946	0.10262	0.324474595	1.660794	4.58E-05	hypermethylated
cg10047595	0.09092	0.287461622	1.6607	0.000165	hypermethylated
cg09639151	0.15083	0.47687027	1.660673	3.90E-06	hypermethylated
cg06719900	0.12623	0.398988649	1.660293	6.39E-06	hypermethylated
cg19623717	0.10704	0.338265946	1.660008	1.99E-05	hypermethylated
cg10477621	0.15228	0.481215135	1.659956	2.49E-06	hypermethylated
cg11824639	0.07607	0.240376757	1.659898	6.23E-05	hypermethylated
cg18106668	0.10964	0.346354595	1.659476	2.53E-06	hypermethylated
cg20253468	0.09705	0.306543243	1.65929	7.93E-06	hypermethylated
cg15428435	0.09152	0.289027027	1.659045	0.00015	hypermethylated
cg25496678	0.11065	0.349437838	1.659032	1.94E-05	hypermethylated
cg12419135	0.13076	0.412929189	1.658973	0.000365	hypermethylated
cg04598517	0.11737	0.370612973	1.65885	1.21E-05	hypermethylated
cg00504705	0.105	0.331498378	1.658612	1.35E-05	hypermethylated
cg23042510	0.09479	0.299143784	1.658032	5.89E-06	hypermethylated
cg05622465	0.08545	0.26962	1.657775	2.13E-05	hypermethylated
cg18479593	0.11527	0.363563784	1.657191	2.78E-05	hypermethylated
cg01834022	0.13813	0.435618378	1.657038	7.47E-05	hypermethylated
cg00586644	0.09427	0.297277297	1.656939	2.04E-05	hypermethylated
cg10767336	0.07854	0.247645405	1.656776	6.48E-06	hypermethylated
cg08248516	0.12995	0.409641081	1.656404	1.94E-05	hypermethylated
cg13964105	0.09015	0.284118919	1.656096	2.10E-05	hypermethylated
cg24827660	0.10088	0.317900541	1.655935	0.000445	hypermethylated
cg12493218	0.07929	0.249846486	1.655831	1.47E-06	hypermethylated
cg20552282	0.08243	0.259700541	1.655608	1.25E-05	hypermethylated
cg21306006	0.08293	0.261258378	1.655511	3.07E-05	hypermethylated
cg06704093	0.09657	0.304144324	1.655109	4.36E-05	hypermethylated
cg23165899	0.10245	0.322505946	1.654406	3.44E-05	hypermethylated
cg21169914	0.10502	0.330575676	1.654316	1.94E-05	hypermethylated
cg20635409	0.09044	0.28468	1.654308	6.62E-05	hypermethylated
cg01873311	0.09937	0.312784324	1.654286	4.82E-05	hypermethylated
cg12114834	0.13259	0.417172973	1.653674	1.46E-05	hypermethylated
cg09354241	0.13081	0.411545946	1.653581	1.44E-05	hypermethylated
cg06163371	0.10328	0.324886486	1.653375	3.19E-05	hypermethylated
cg03383508	0.07785	0.24488973	1.653363	0.00035	hypermethylated
cg20732478	0.10358	0.325764324	1.653083	3.04E-06	hypermethylated
cg09943716	0.08849	0.278276757	1.652934	1.75E-05	hypermethylated
cg05733135	0.08436	0.265255135	1.65275	0.00062	hypermethylated
cg24711649	0.07933	0.249434595	1.652723	3.49E-05	hypermethylated
cg03556497	0.08035	0.252631892	1.652667	1.58E-05	hypermethylated
cg25397922	0.11321	0.355921622	1.652558	0.000195	hypermethylated
cg00254133	0.0912	0.286687027	1.652371	0.000271	hypermethylated
cg13078381	0.08241	0.259014054	1.652139	1.31E-05	hypermethylated
cg16793187	0.0761	0.239175135	1.652099	1.77E-05	hypermethylated
cg00185566	0.08033	0.252425946	1.651849	9.95E-05	hypermethylated
cg04304705	0.1396	0.438648649	1.651767	3.12E-06	hypermethylated
cg16331929	0.08499	0.266994054	1.651443	0.000117	hypermethylated
cg11021995	0.10007	0.314281081	1.651046	4.70E-05	hypermethylated
cg26186727	0.10822	0.339744865	1.650485	6.22E-06	hypermethylated
cg19098710	0.11681	0.366708649	1.650471	6.75E-06	hypermethylated
cg24914355	0.10685	0.335307027	1.649896	4.05E-05	hypermethylated
cg01919632	0.09088	0.285154054	1.649707	1.99E-05	hypermethylated

cg07272711	0.08388	0.263132973	1.649393	5.14E-06	hypermethylated
cg09123188	0.11371	0.356676216	1.649256	2.71E-05	hypermethylated
cg22686881	0.11812	0.370469189	1.6491	2.07E-06	hypermethylated
cg04511041	0.09676	0.30344973	1.648975	3.49E-05	hypermethylated
cg14236662	0.13809	0.432958378	1.648619	4.36E-06	hypermethylated
cg05095591	0.11785	0.36944973	1.648426	3.90E-06	hypermethylated
cg24333629	0.10774	0.337754595	1.648421	2.45E-05	hypermethylated
cg13403815	0.08831	0.276787568	1.64813	1.46E-05	hypermethylated
cg23302649	0.12246	0.383814595	1.648099	2.15E-05	hypermethylated
cg03326762	0.1034	0.324076216	1.648097	7.52E-06	hypermethylated
cg04552470	0.07839	0.245665405	1.647953	2.30E-05	hypermethylated
cg19343464	0.12985	0.406921622	1.647905	2.42E-05	hypermethylated
cg07870999	0.12903	0.404325946	1.647812	6.22E-06	hypermethylated
cg08833577	0.12091	0.378835676	1.647639	1.89E-05	hypermethylated
cg07434271	0.10887	0.341056216	1.647403	3.90E-06	hypermethylated
cg05732979	0.09662	0.302664324	1.647325	1.21E-05	hypermethylated
cg03290530	0.1339	0.419417297	1.64723	1.41E-06	hypermethylated
cg04546041	0.11455	0.358801081	1.647207	4.05E-05	hypermethylated
cg12001304	0.14367	0.449979459	1.6471	1.62E-05	hypermethylated
cg06854015	0.04721	0.147863243	1.647099	3.00E-05	hypermethylated
cg01245966	0.09032	0.282852973	1.646935	4.64E-05	hypermethylated
cg12848065	0.10844	0.339571892	1.64682	5.58E-05	hypermethylated
cg23916167	0.10322	0.323104865	1.64628	3.67E-05	hypermethylated
cg10530883	0.15677	0.490683784	1.646144	3.30E-06	hypermethylated
cg22191326	0.0971	0.303843784	1.645787	1.89E-05	hypermethylated
cg03167683	0.09368	0.293131892	1.645737	0.000189	hypermethylated
cg04761722	0.06559	0.205184865	1.645377	2.10E-06	hypermethylated
cg00397635	0.10258	0.320888108	1.645321	5.38E-05	hypermethylated
cg27072996	0.14425	0.450995135	1.644541	2.15E-05	hypermethylated
cg09624466	0.16034	0.501267568	1.644447	3.21E-06	hypermethylated
cg19134945	0.10299	0.321943784	1.644305	0.000667	hypermethylated
cg07203423	0.13781	0.430655676	1.643854	8.95E-06	hypermethylated
cg20189244	0.12308	0.384575676	1.643671	9.66E-07	hypermethylated
cg02013838	0.12548	0.391885405	1.642974	2.85E-05	hypermethylated
cg14359292	0.11388	0.355592973	1.642712	2.24E-05	hypermethylated
cg00127167	0.12381	0.386569189	1.642599	1.28E-05	hypermethylated
cg11973479	0.07132	0.222643243	1.642355	1.25E-05	hypermethylated
cg26419265	0.11672	0.364331892	1.642201	2.21E-05	hypermethylated
cg13337047	0.09562	0.298433514	1.642025	0.00017	hypermethylated
cg23756251	0.08725	0.27226	1.641758	1.46E-05	hypermethylated
cg19793376	0.1054	0.328852973	1.641568	2.04E-05	hypermethylated
cg20712263	0.09764	0.304610811	1.641423	0.000268	hypermethylated
cg06010588	0.13861	0.432317297	1.641059	5.19E-05	hypermethylated
cg13125157	0.15148	0.472454054	1.641047	5.06E-05	hypermethylated
cg02824386	0.09272	0.289097297	1.640603	6.01E-05	hypermethylated
cg11141380	0.11367	0.354391892	1.640494	1.09E-05	hypermethylated
cg07765621	0.11191	0.348862703	1.64032	2.71E-05	hypermethylated
cg09970569	0.09273	0.289064324	1.640283	2.57E-05	hypermethylated
cg15873149	0.12539	0.390773514	1.63991	6.14E-06	hypermethylated
cg02259324	0.11684	0.36404973	1.639601	6.95E-05	hypermethylated
cg12118843	0.10754	0.335055676	1.639527	4.86E-06	hypermethylated
cg16991765	0.13433	0.41852	1.639515	8.60E-06	hypermethylated
cg00022911	0.09133	0.284542162	1.639482	0.000147	hypermethylated
cg15310492	0.11752	0.366009189	1.638974	4.15E-05	hypermethylated
cg12527175	0.07193	0.223868649	1.637987	3.67E-05	hypermethylated
cg12684209	0.10955	0.340877297	1.637663	5.73E-06	hypermethylated
cg03226000	0.12987	0.404039459	1.637428	1.52E-05	hypermethylated
cg02728050	0.0743	0.231105405	1.637117	2.51E-05	hypermethylated

cg01663016	0.11242	0.349666486	1.637081	1.09E-05	hypermethylated
cg23168531	0.09015	0.280284865	1.636494	9.31E-06	hypermethylated
cg04404540	0.0792	0.246217297	1.63636	4.26E-05	hypermethylated
cg19385628	0.09732	0.302513514	1.636191	8.42E-05	hypermethylated
cg06419750	0.12348	0.383667027	1.635577	0.00018	hypermethylated
cg18679410	0.10437	0.324275676	1.635514	4.20E-05	hypermethylated
cg08079908	0.14159	0.4399	1.635456	1.62E-05	hypermethylated
cg11841771	0.11932	0.370672432	1.635309	7.72E-06	hypermethylated
cg00970396	0.13047	0.405208649	1.634947	9.05E-05	hypermethylated
cg22047387	0.13038	0.404835676	1.634614	1.80E-05	hypermethylated
cg16794506	0.12124	0.376388108	1.634355	1.62E-05	hypermethylated
cg21290042	0.09778	0.303522162	1.634191	0.000474	hypermethylated
cg11657203	0.07899	0.245191351	1.634166	0.000157	hypermethylated
cg00153106	0.10643	0.33031027	1.633917	1.50E-05	hypermethylated
cg11115235	0.10957	0.340039459	1.633849	2.85E-05	hypermethylated
cg07003542	0.10441	0.324005946	1.63376	0.000124	hypermethylated
cg00872726	0.14151	0.439118378	1.633706	5.58E-06	hypermethylated
cg00472801	0.08876	0.275384324	1.633465	5.32E-05	hypermethylated
cg15089219	0.09053	0.280859459	1.633381	1.37E-05	hypermethylated
cg14328457	0.10836	0.336136216	1.633214	3.95E-05	hypermethylated
cg23121156	0.1124	0.348668108	1.633212	2.71E-05	hypermethylated
cg09853371	0.09636	0.298890811	1.633112	0.000224	hypermethylated
cg03164275	0.11193	0.347177297	1.633076	0.000102	hypermethylated
cg22748799	0.09146	0.283665405	1.632977	4.94E-05	hypermethylated
cg09980058	0.13865	0.429996757	1.632878	1.84E-05	hypermethylated
cg14695492	0.11895	0.36884	1.63264	9.31E-06	hypermethylated
cg08737296	0.10321	0.32003027	1.632626	5.58E-05	hypermethylated
cg20172500	0.07631	0.23660973	1.632565	1.66E-05	hypermethylated
cg25090514	0.13614	0.422105405	1.632512	2.79E-06	hypermethylated
cg15992284	0.08824	0.273587568	1.632498	2.92E-05	hypermethylated
cg25682299	0.07739	0.239835676	1.631827	5.86E-05	hypermethylated
cg25970377	0.06633	0.205551351	1.631765	5.45E-05	hypermethylated
cg07401378	0.07922	0.24546973	1.631609	3.00E-05	hypermethylated
cg13777717	0.103	0.319049189	1.631135	4.36E-06	hypermethylated
cg02798576	0.14243	0.441182703	1.631123	2.79E-06	hypermethylated
cg16646743	0.07387	0.228813514	1.631112	6.31E-05	hypermethylated
cg01519784	0.09887	0.30622	1.630964	5.86E-05	hypermethylated
cg13262687	0.10782	0.333879459	1.630703	9.82E-06	hypermethylated
cg09086835	0.12449	0.385461622	1.630557	1.09E-06	hypermethylated
cg12103265	0.09913	0.306837297	1.63008	5.00E-05	hypermethylated
cg19433697	0.11093	0.34324973	1.629609	3.00E-05	hypermethylated
cg19582316	0.07869	0.243469189	1.629487	2.21E-05	hypermethylated
cg23448729	0.11129	0.344236757	1.629077	3.49E-05	hypermethylated
cg05044185	0.09621	0.297574595	1.628993	2.85E-05	hypermethylated
cg25328184	0.07805	0.241358378	1.628706	2.27E-05	hypermethylated
cg24190603	0.15207	0.470135676	1.628342	2.51E-05	hypermethylated
cg14449051	0.09397	0.290508649	1.628309	9.72E-05	hypermethylated
cg22105145	0.09476	0.292885946	1.627989	4.93E-06	hypermethylated
cg11708136	0.09532	0.294421622	1.627033	1.37E-05	hypermethylated
cg07099331	0.13883	0.428681622	1.626587	6.93E-06	hypermethylated
cg06454760	0.16707	0.515804865	1.626373	2.92E-05	hypermethylated
cg20170533	0.11178	0.345053514	1.626158	0.000117	hypermethylated
cg14448169	0.11284	0.348217838	1.625712	7.84E-05	hypermethylated
cg02441747	0.08711	0.268791892	1.625579	9.49E-05	hypermethylated
cg14558529	0.16158	0.4985	1.625345	3.12E-06	hypermethylated
cg09799983	0.16769	0.51724973	1.625064	8.37E-06	hypermethylated
cg02172312	0.12039	0.371092432	1.624063	1.71E-05	hypermethylated
cg06847624	0.11298	0.348219459	1.623929	5.19E-05	hypermethylated

cg04457979	0.10516	0.32407027	1.623721	3.90E-06	hypermethylated
cg22274539	0.1046	0.322343784	1.623717	0.000138	hypermethylated
cg02767613	0.10875	0.335074054	1.623465	4.15E-05	hypermethylated
cg04217778	0.12596	0.388060541	1.623316	8.84E-05	hypermethylated
cg02681173	0.09396	0.289438378	1.623138	0.000143	hypermethylated
cg06518628	0.09704	0.298835676	1.622701	4.05E-05	hypermethylated
cg17397150	0.09487	0.292122703	1.622551	2.81E-05	hypermethylated
cg26588943	0.081	0.249361622	1.622246	4.15E-05	hypermethylated
cg02058267	0.07902	0.24317027	1.621677	1.99E-05	hypermethylated
cg22548438	0.08147	0.250655135	1.621363	3.85E-05	hypermethylated
cg12352960	0.10438	0.321135676	1.621338	0.000129	hypermethylated
cg23254031	0.08097	0.249078919	1.621144	1.06E-05	hypermethylated
cg17285663	0.09249	0.284502162	1.62107	0.000185	hypermethylated
cg22471517	0.07791	0.239652973	1.621066	1.31E-05	hypermethylated
cg13491462	0.07988	0.245708108	1.621039	1.80E-05	hypermethylated
cg24176563	0.10068	0.309622703	1.620734	4.00E-05	hypermethylated
cg04711162	0.15169	0.466467568	1.620651	3.79E-06	hypermethylated
cg13655674	0.10701	0.328976216	1.620238	1.89E-05	hypermethylated
cg15312264	0.13939	0.42842973	1.619932	4.24E-06	hypermethylated
cg22679728	0.11499	0.353264865	1.619242	1.84E-05	hypermethylated
cg12160317	0.10896	0.334734595	1.619219	5.58E-06	hypermethylated
cg25741487	0.09379	0.288104865	1.619088	6.66E-06	hypermethylated
cg08566455	0.19767	0.607168649	1.619003	2.29E-06	hypermethylated
cg26348902	0.09863	0.30289027	1.618697	3.49E-06	hypermethylated
cg16937268	0.14815	0.454944324	1.618631	3.90E-06	hypermethylated
cg13161658	0.08055	0.247317838	1.61841	1.80E-05	hypermethylated
cg22476848	0.06467	0.198556757	1.618383	0.000506	hypermethylated
cg14691529	0.08793	0.269968649	1.618365	0.000229	hypermethylated
cg08621473	0.11651	0.357683784	1.618231	9.07E-06	hypermethylated
cg04360793	0.17458	0.535924324	1.618141	9.57E-06	hypermethylated
cg06158650	0.12354	0.379221622	1.618063	3.49E-06	hypermethylated
cg07514158	0.11283	0.346318919	1.61795	3.15E-05	hypermethylated
cg07184578	0.12842	0.39416973	1.617947	0.000187	hypermethylated
cg03986829	0.09426	0.289205405	1.617377	0.000166	hypermethylated
cg14361458	0.11579	0.355242703	1.617294	3.79E-06	hypermethylated
cg03585912	0.1278	0.392049189	1.617147	1.45E-06	hypermethylated
cg12552771	0.11395	0.34954	1.617057	6.39E-06	hypermethylated
cg21528204	0.0855	0.262256757	1.616984	2.45E-05	hypermethylated
cg07676859	0.15064	0.461992432	1.616764	6.95E-05	hypermethylated
cg15562220	0.12573	0.38555027	1.61659	6.78E-05	hypermethylated
cg06574716	0.12984	0.398069189	1.616284	4.73E-06	hypermethylated
cg00502665	0.09493	0.291008649	1.616126	1.06E-05	hypermethylated
cg16024950	0.13856	0.424732432	1.616043	8.83E-06	hypermethylated
cg15731655	0.08862	0.271637297	1.615977	0.000135	hypermethylated
cg12087643	0.08085	0.247702703	1.61529	0.000138	hypermethylated
cg05732750	0.16272	0.498439459	1.615027	1.58E-06	hypermethylated
cg13929627	0.08068	0.24713027	1.614989	8.60E-06	hypermethylated
cg07143083	0.14685	0.449776757	1.614866	6.22E-06	hypermethylated
cg03640756	0.09977	0.305575676	1.614852	1.62E-05	hypermethylated
cg17811778	0.09984	0.305764324	1.61473	8.52E-05	hypermethylated
cg14564076	0.13729	0.420228649	1.613948	1.75E-05	hypermethylated
cg05844247	0.1095	0.335133514	1.613805	5.14E-06	hypermethylated
cg18710784	0.12673	0.387812432	1.613601	5.45E-05	hypermethylated
cg12508624	0.10987	0.336203243	1.613536	4.31E-05	hypermethylated
cg17300736	0.11329	0.346547027	1.613031	1.54E-05	hypermethylated
cg05511722	0.0967	0.295755676	1.612818	1.71E-05	hypermethylated
cg04599297	0.11584	0.354211892	1.612479	1.35E-05	hypermethylated
cg24575234	0.14361	0.439115135	1.612443	8.15E-06	hypermethylated

cg22931725	0.07746	0.236842703	1.612406	1.12E-05	hypermethylated
cg21725954	0.09224	0.282012432	1.612294	3.76E-05	hypermethylated
cg24693551	0.09284	0.283803784	1.612075	8.94E-05	hypermethylated
cg02252323	0.10152	0.310334054	1.612058	6.75E-06	hypermethylated
cg14799209	0.05987	0.182944865	1.611504	1.80E-05	hypermethylated
cg25164649	0.09867	0.301424865	1.611115	9.05E-05	hypermethylated
cg04559779	0.14381	0.439253514	1.61089	1.21E-05	hypermethylated
cg23998071	0.10903	0.333016757	1.61087	7.65E-05	hypermethylated
cg20989855	0.09881	0.301725405	1.610507	5.58E-05	hypermethylated
cg05756489	0.1018	0.31085027	1.610482	2.18E-05	hypermethylated
cg22374233	0.07237	0.220961081	1.610329	5.93E-05	hypermethylated
cg26872137	0.11656	0.355801081	1.609998	5.31E-05	hypermethylated
cg24981593	0.10052	0.306769189	1.609671	6.15E-05	hypermethylated
cg03281661	0.1029	0.313982162	1.60944	1.25E-05	hypermethylated
cg12093775	0.11241	0.342955135	1.609249	8.83E-06	hypermethylated
cg22989843	0.11759	0.358664324	1.608869	8.83E-06	hypermethylated
cg02036261	0.12741	0.388563243	1.608671	7.03E-06	hypermethylated
cg23242052	0.10528	0.321071351	1.608663	7.62E-06	hypermethylated
cg12919006	0.13109	0.399758919	1.608573	2.27E-05	hypermethylated
cg24693341	0.11121	0.338952973	1.607799	1.12E-05	hypermethylated
cg14750948	0.16177	0.492908108	1.607375	2.10E-06	hypermethylated
cg19122901	0.07602	0.231552973	1.606891	2.48E-05	hypermethylated
cg18173235	0.09628	0.293176216	1.60646	0.000165	hypermethylated
cg09699039	0.09389	0.285835135	1.60614	9.05E-05	hypermethylated
cg15690342	0.13746	0.418297297	1.605517	0.000132	hypermethylated
cg19147218	0.11539	0.350988649	1.604906	0.000152	hypermethylated
cg02983163	0.13257	0.40324	1.604884	2.10E-06	hypermethylated
cg08005774	0.10216	0.310725946	1.604812	1.50E-05	hypermethylated
cg24650501	0.08814	0.268043784	1.6046	1.06E-05	hypermethylated
cg12296772	0.16459	0.500520541	1.604553	3.07E-05	hypermethylated
cg21533331	0.08601	0.261503784	1.604256	1.54E-05	hypermethylated
cg00467420	0.0884	0.268761081	1.604206	9.05E-05	hypermethylated
cg17236169	0.12495	0.379793514	1.603864	1.75E-05	hypermethylated
cg08404009	0.09528	0.289594054	1.603787	1.21E-05	hypermethylated
cg01482645	0.10775	0.327484865	1.60374	6.78E-05	hypermethylated
cg15237494	0.12369	0.375893514	1.603595	2.85E-05	hypermethylated
cg05387399	0.1061	0.322340541	1.603161	1.09E-05	hypermethylated
cg18685561	0.12296	0.373543243	1.603086	8.60E-06	hypermethylated
cg25549459	0.09906	0.300907027	1.602943	1.75E-05	hypermethylated
cg10023530	0.11068	0.336131892	1.602633	5.65E-06	hypermethylated
cg04479219	0.11837	0.359462703	1.602539	5.14E-06	hypermethylated
cg07073391	0.11548	0.35062	1.602265	2.64E-05	hypermethylated
cg01630690	0.13131	0.39868	1.602254	8.71E-06	hypermethylated
cg20881942	0.12876	0.390913514	1.602165	2.96E-05	hypermethylated
cg19595505	0.11779	0.357583243	1.602062	7.52E-06	hypermethylated
cg19512268	0.11163	0.338761081	1.601543	9.72E-05	hypermethylated
cg03323462	0.1247	0.378378919	1.60137	1.42E-05	hypermethylated
cg00035316	0.12525	0.379983243	1.601125	4.60E-06	hypermethylated
cg08428452	0.08289	0.251443784	1.600966	3.11E-05	hypermethylated
cg06330691	0.093	0.282094054	1.600874	2.07E-05	hypermethylated
cg15964309	0.09732	0.295109189	1.600441	5.19E-05	hypermethylated
cg03031609	0.08365	0.253587027	1.600043	2.35E-05	hypermethylated
cg17657179	0.12301	0.372897838	1.600005	3.23E-05	hypermethylated
cg04853843	0.11206	0.339698378	1.599983	9.19E-06	hypermethylated
cg20897830	0.13972	0.423428108	1.599578	3.69E-06	hypermethylated
cg20519121	0.08476	0.256844865	1.599442	5.58E-05	hypermethylated
cg18867480	0.09082	0.275179459	1.599291	1.94E-05	hypermethylated
cg06372303	0.07819	0.236907027	1.599265	1.17E-05	hypermethylated

cg12756396	0.13591	0.411758378	1.599146	2.64E-06	hypermethylated
cg11751707	0.10623	0.321811351	1.599024	6.23E-05	hypermethylated
cg03882167	0.1181	0.357621622	1.598425	6.15E-05	hypermethylated
cg05016408	0.11918	0.360808649	1.598092	0.000107	hypermethylated
cg03171361	0.0959	0.290284324	1.597864	4.58E-05	hypermethylated
cg25563456	0.09795	0.296356757	1.597218	3.64E-06	hypermethylated
cg12997720	0.13377	0.404681081	1.597031	3.67E-05	hypermethylated
cg00181125	0.1142	0.345440541	1.596875	2.32E-05	hypermethylated
cg11891395	0.1171	0.354203784	1.596839	2.51E-05	hypermethylated
cg11710851	0.10785	0.326176216	1.596625	0.000152	hypermethylated
cg00332937	0.12972	0.39229027	1.596521	7.52E-06	hypermethylated
cg16415870	0.10443	0.315792432	1.59644	5.06E-05	hypermethylated
cg24608684	0.09128	0.275991892	1.596255	0.000143	hypermethylated
cg19020830	0.08318	0.25146973	1.596076	6.75E-06	hypermethylated
cg09526758	0.10178	0.307502162	1.595142	1.84E-05	hypermethylated
cg24837370	0.18356	0.554572973	1.595126	5.89E-06	hypermethylated
cg02541778	0.12127	0.366368649	1.595073	0.000126	hypermethylated
cg11998703	0.09399	0.283922162	1.594916	9.82E-06	hypermethylated
cg12818557	0.14351	0.433508649	1.59491	0.000256	hypermethylated
cg13574121	0.09486	0.286511351	1.594721	2.27E-05	hypermethylated
cg01033865	0.09728	0.293654595	1.593905	3.57E-05	hypermethylated
cg22648135	0.11842	0.357384324	1.593564	3.79E-06	hypermethylated
cg03876413	0.09816	0.296054595	1.592656	2.85E-05	hypermethylated
cg23839136	0.12702	0.383082162	1.592598	3.69E-06	hypermethylated
cg04222358	0.13333	0.402106486	1.592576	1.21E-05	hypermethylated
cg08107689	0.12305	0.371037838	1.592322	4.18E-06	hypermethylated
cg02164225	0.09348	0.281822703	1.592058	2.45E-05	hypermethylated
cg22717227	0.08502	0.256154595	1.591141	8.60E-06	hypermethylated
cg07142201	0.10479	0.315635676	1.590759	1.77E-05	hypermethylated
cg01577751	0.1006	0.303010811	1.590739	1.25E-05	hypermethylated
cg15628917	0.09662	0.291013514	1.590692	2.74E-05	hypermethylated
cg26239391	0.09803	0.295213514	1.590464	1.01E-05	hypermethylated
cg03442425	0.12435	0.374430811	1.590293	6.46E-05	hypermethylated
cg15412759	0.12249	0.368729189	1.589898	1.35E-05	hypermethylated
cg22335876	0.14363	0.432362703	1.589885	1.42E-05	hypermethylated
cg04032066	0.11829	0.356054595	1.58977	1.31E-05	hypermethylated
cg06549216	0.07586	0.228335135	1.589742	2.81E-05	hypermethylated
cg01796166	0.14568	0.438468108	1.589669	2.87E-06	hypermethylated
cg11793269	0.10922	0.328643243	1.589285	7.32E-06	hypermethylated
cg03936663	0.14338	0.43142	1.589249	2.10E-06	hypermethylated
cg15160263	0.08794	0.264594054	1.589189	4.58E-05	hypermethylated
cg17029062	0.08478	0.255076757	1.589136	9.38E-05	hypermethylated
cg26300652	0.11267	0.338842162	1.58851	8.95E-06	hypermethylated
cg02674384	0.10863	0.326513514	1.58772	0.000187	hypermethylated
cg06419850	0.12546	0.377077838	1.587635	2.71E-06	hypermethylated
cg11366849	0.15716	0.472296757	1.58746	8.60E-06	hypermethylated
cg02430935	0.12125	0.364146486	1.586534	2.21E-05	hypermethylated
cg04859466	0.10534	0.316357838	1.586504	1.87E-05	hypermethylated
cg16783744	0.14616	0.438929189	1.58644	3.21E-06	hypermethylated
cg08091192	0.11951	0.358885405	1.586392	1.94E-05	hypermethylated
cg02442509	0.08102	0.243268108	1.586197	1.01E-05	hypermethylated
cg06212175	0.09638	0.28936973	1.586108	0.000216	hypermethylated
cg18297437	0.12702	0.381345946	1.586045	9.31E-06	hypermethylated
cg00481629	0.09837	0.295298378	1.585883	0.000145	hypermethylated
cg08620154	0.12239	0.367354054	1.585686	4.47E-05	hypermethylated
cg01598250	0.0836	0.250924324	1.585677	1.93E-06	hypermethylated
cg19329160	0.12764	0.383048108	1.585445	1.09E-05	hypermethylated
cg20347882	0.10887	0.326712432	1.585415	3.79E-06	hypermethylated

cg09229912	0.112	0.336056216	1.585204	4.15E-05	hypermethylated
cg14265823	0.1222	0.366652973	1.585171	4.47E-05	hypermethylated
cg00919971	0.11382	0.341504865	1.585152	1.28E-05	hypermethylated
cg01851378	0.16397	0.491962162	1.585115	4.80E-06	hypermethylated
cg08889797	0.11206	0.336191892	1.585014	4.42E-06	hypermethylated
cg17892178	0.12343	0.370276757	1.584911	1.58E-05	hypermethylated
cg20462512	0.11167	0.33494973	1.584703	8.83E-06	hypermethylated
cg12378187	0.13569	0.406995676	1.584699	6.05E-06	hypermethylated
cg24309428	0.09731	0.291814595	1.584392	3.85E-05	hypermethylated
cg23877720	0.096	0.287883243	1.584378	6.75E-06	hypermethylated
cg05186311	0.10529	0.315717838	1.584267	3.26E-06	hypermethylated
cg24000873	0.10628	0.318655135	1.584126	1.71E-05	hypermethylated
cg02730156	0.08772	0.262988108	1.58402	5.65E-06	hypermethylated
cg21546184	0.10958	0.328475676	1.583802	1.54E-05	hypermethylated
cg20486569	0.10694	0.320531892	1.583666	1.06E-05	hypermethylated
cg21769640	0.14065	0.421513514	1.583469	5.89E-06	hypermethylated
cg01381846	0.18366	0.550408649	1.583466	2.10E-06	hypermethylated
cg11750165	0.11936	0.357624865	1.583128	5.58E-06	hypermethylated
cg26660002	0.0966	0.289396216	1.582951	9.72E-05	hypermethylated
cg15855970	0.09055	0.271215676	1.582654	1.46E-05	hypermethylated
cg00364016	0.10354	0.31008973	1.582498	8.15E-06	hypermethylated
cg24133115	0.10745	0.32176973	1.582363	0.00035	hypermethylated
cg19258062	0.14014	0.419633514	1.582261	7.93E-06	hypermethylated
cg00100012	0.10802	0.323408649	1.58206	9.69E-06	hypermethylated
cg16249035	0.06828	0.204425405	1.58204	7.93E-06	hypermethylated
cg25750389	0.07626	0.228283243	1.581827	3.54E-06	hypermethylated
cg11611600	0.0816	0.244137838	1.581055	1.20E-05	hypermethylated
cg27112565	0.10538	0.315261622	1.580948	9.72E-05	hypermethylated
cg08462988	0.12264	0.366874595	1.580857	1.06E-05	hypermethylated
cg24047377	0.07875	0.235575676	1.580839	0.000119	hypermethylated
cg26950557	0.09344	0.279446486	1.58046	5.93E-05	hypermethylated
cg05474726	0.12851	0.384168649	1.579859	5.72E-05	hypermethylated
cg15060012	0.1108	0.331204865	1.579766	2.89E-05	hypermethylated
cg25084878	0.10766	0.321813514	1.579743	4.15E-05	hypermethylated
cg22190437	0.11234	0.335725946	1.579412	1.89E-05	hypermethylated
cg27042584	0.11961	0.357445946	1.579387	1.01E-05	hypermethylated
cg07211140	0.08352	0.249563243	1.579212	2.81E-05	hypermethylated
cg13572145	0.08691	0.259677297	1.579126	7.72E-06	hypermethylated
cg08445263	0.12907	0.385616216	1.579012	1.04E-05	hypermethylated
cg17078427	0.0814	0.243091351	1.578398	1.25E-05	hypermethylated
cg26421310	0.11028	0.329275135	1.578122	0.00221	hypermethylated
cg03810428	0.09665	0.28846973	1.577578	4.05E-05	hypermethylated
cg01060089	0.06056	0.180746486	1.57753	6.54E-05	hypermethylated
cg15836660	0.08123	0.242405405	1.577337	1.46E-05	hypermethylated
cg02466815	0.13808	0.412047568	1.577307	1.15E-05	hypermethylated
cg01933308	0.10581	0.31572	1.57717	0.000195	hypermethylated
cg05863502	0.15554	0.464044324	1.576977	5.58E-06	hypermethylated
cg23288059	0.09944	0.296586486	1.576555	3.07E-05	hypermethylated
cg16823083	0.14901	0.444430811	1.57655	8.15E-06	hypermethylated
cg14582763	0.12782	0.381080541	1.575982	2.92E-05	hypermethylated
cg19206146	0.15757	0.469702162	1.575753	3.04E-06	hypermethylated
cg15590989	0.11024	0.328396216	1.57479	9.57E-06	hypermethylated
cg14983606	0.11164	0.332542162	1.574683	5.86E-05	hypermethylated
cg19517718	0.10919	0.325212432	1.574542	2.64E-06	hypermethylated
cg15397374	0.10204	0.303883243	1.574382	7.65E-05	hypermethylated
cg17459204	0.11988	0.357005946	1.574357	0.000224	hypermethylated
cg04954680	0.10199	0.303695135	1.574196	4.05E-05	hypermethylated
cg21444693	0.10513	0.313028649	1.57412	7.84E-05	hypermethylated

cg17199800	0.12677	0.377422162	1.573966	1.51E-06	hypermethylated
cg24871414	0.13228	0.393821081	1.573945	9.82E-06	hypermethylated
cg22687748	0.07933	0.236045946	1.573129	6.46E-05	hypermethylated
cg12109455	0.08405	0.250023784	1.572746	6.93E-06	hypermethylated
cg00792424	0.12713	0.378158919	1.572688	5.28E-06	hypermethylated
cg12431879	0.12566	0.373750811	1.572551	1.46E-05	hypermethylated
cg25865467	0.07546	0.224440541	1.572549	5.79E-05	hypermethylated
cg01146232	0.13686	0.40704	1.57247	5.73E-06	hypermethylated
cg20076842	0.09761	0.290232973	1.572111	8.15E-06	hypermethylated
cg24778248	0.09235	0.27458973	1.572094	2.10E-05	hypermethylated
cg04386206	0.09127	0.27134973	1.571941	4.70E-05	hypermethylated
cg18603028	0.1197	0.355860541	1.571889	3.30E-06	hypermethylated
cg21012296	0.11804	0.350891892	1.571751	6.86E-05	hypermethylated
cg01328892	0.1169	0.347458378	1.571565	0.000103	hypermethylated
cg12189551	0.10606	0.315205405	1.571412	4.15E-05	hypermethylated
cg11362010	0.11139	0.330992432	1.571179	5.72E-05	hypermethylated
cg19469447	0.10872	0.323018919	1.571001	0.000112	hypermethylated
cg13893543	0.1026	0.304791892	1.570794	2.64E-06	hypermethylated
cg22961278	0.1095	0.325221622	1.570492	0.000142	hypermethylated
cg02589791	0.13099	0.389008649	1.570346	3.04E-05	hypermethylated
cg03442378	0.11047	0.328	1.570041	1.35E-05	hypermethylated
cg19949955	0.13069	0.387995676	1.569892	1.31E-05	hypermethylated
cg20717123	0.13493	0.400530811	1.569702	8.15E-06	hypermethylated
cg08252579	0.09913	0.294226486	1.569533	1.62E-05	hypermethylated
cg25415246	0.14204	0.42150973	1.569269	6.05E-06	hypermethylated
cg13021619	0.09174	0.272222162	1.569162	7.12E-05	hypermethylated
cg27006650	0.07591	0.225185946	1.568755	7.52E-06	hypermethylated
cg04181150	0.09588	0.284378378	1.56851	1.56E-05	hypermethylated
cg21784917	0.0887	0.263064865	1.568413	0.000137	hypermethylated
cg08495770	0.09938	0.294652432	1.567987	3.57E-05	hypermethylated
cg26667205	0.10949	0.324556216	1.567669	9.16E-05	hypermethylated
cg04227961	0.16473	0.488141622	1.567196	1.12E-05	hypermethylated
cg23191956	0.12162	0.360347568	1.567009	4.73E-06	hypermethylated
cg19988449	0.12282	0.363677838	1.566116	8.10E-07	hypermethylated
cg20890210	0.10508	0.311063243	1.56572	9.72E-05	hypermethylated
cg02571636	0.13376	0.395943784	1.565649	5.93E-05	hypermethylated
cg15393937	0.13081	0.387102162	1.565242	8.03E-05	hypermethylated
cg08432727	0.10636	0.314685405	1.564955	3.90E-06	hypermethylated
cg05976714	0.11635	0.344231351	1.564907	0.000108	hypermethylated
cg04748834	0.10989	0.325059459	1.564644	4.05E-05	hypermethylated
cg11859607	0.13377	0.395456757	1.563765	5.89E-06	hypermethylated
cg01583131	0.10787	0.318865946	1.563656	1.18E-05	hypermethylated
cg01371799	0.12004	0.354835676	1.563636	4.36E-05	hypermethylated
cg17437939	0.13661	0.403798378	1.563572	5.86E-05	hypermethylated
cg04018622	0.08424	0.248973514	1.563415	4.80E-06	hypermethylated
cg11596580	0.07923	0.234115676	1.563103	2.32E-05	hypermethylated
cg01568998	0.10365	0.306096757	1.562268	1.82E-05	hypermethylated
cg21869586	0.09258	0.273382703	1.562149	1.42E-05	hypermethylated
cg15814504	0.12642	0.373271351	1.562	5.86E-05	hypermethylated
cg13818654	0.08484	0.250478378	1.56187	5.45E-05	hypermethylated
cg14576896	0.09429	0.27829027	1.561414	2.71E-05	hypermethylated
cg02773433	0.08014	0.236509189	1.561302	0.000142	hypermethylated
cg13596497	0.11099	0.327525405	1.561177	1.52E-05	hypermethylated
cg20581321	0.1171	0.345532432	1.56108	1.46E-05	hypermethylated
cg22195627	0.13276	0.391682162	1.560863	0.000293	hypermethylated
cg22016949	0.13067	0.385513514	1.560853	4.01E-06	hypermethylated
cg17165841	0.12029	0.354633514	1.559812	7.72E-06	hypermethylated
cg04251368	0.10491	0.309236757	1.55956	3.07E-05	hypermethylated

cg05733181	0.10628	0.313216216	1.559289	3.44E-05	hypermethylated
cg10074775	0.10583	0.311823243	1.55898	4.64E-05	hypermethylated
cg17772342	0.10064	0.296521081	1.558931	3.67E-05	hypermethylated
cg21485303	0.12468	0.36731027	1.558769	8.37E-06	hypermethylated
cg06459847	0.09392	0.276685405	1.558742	1.33E-05	hypermethylated
cg01078989	0.08498	0.250257297	1.558217	2.35E-05	hypermethylated
cg20962117	0.08899	0.262043784	1.558093	5.00E-05	hypermethylated
cg08734647	0.12128	0.357121622	1.558074	5.25E-05	hypermethylated
cg07731304	0.09092	0.267722162	1.558067	3.44E-05	hypermethylated
cg17319774	0.08681	0.255569189	1.557781	7.22E-06	hypermethylated
cg11311843	0.15342	0.451663243	1.557761	0.00012	hypermethylated
cg22457238	0.10222	0.300914054	1.557674	1.12E-05	hypermethylated
cg15466862	0.11481	0.337968108	1.557639	8.15E-06	hypermethylated
cg18247055	0.15041	0.442644865	1.557249	7.93E-06	hypermethylated
cg05209584	0.13949	0.41036973	1.556763	8.37E-06	hypermethylated
cg15784124	0.08334	0.24516	1.556643	1.12E-05	hypermethylated
cg11012061	0.12112	0.35627027	1.556535	1.07E-06	hypermethylated
cg07274716	0.16399	0.482029189	1.555513	1.45E-06	hypermethylated
cg21609106	0.11279	0.331105405	1.553651	5.86E-05	hypermethylated
cg07760204	0.08739	0.256463784	1.553215	1.28E-05	hypermethylated
cg17012160	0.12175	0.357273514	1.553107	2.27E-05	hypermethylated
cg03881775	0.12932	0.379447027	1.552953	6.75E-06	hypermethylated
cg21543987	0.09514	0.279128649	1.552806	9.95E-05	hypermethylated
cg22881652	0.09531	0.279609189	1.552712	2.96E-05	hypermethylated
cg00123055	0.13669	0.400941622	1.552484	2.36E-06	hypermethylated
cg06722633	0.10651	0.312385405	1.552338	4.73E-06	hypermethylated
cg22732749	0.11436	0.335387027	1.552244	1.58E-05	hypermethylated
cg14486338	0.18457	0.541183784	1.551951	2.16E-06	hypermethylated
cg13647155	0.11527	0.337738919	1.550891	0.000132	hypermethylated
cg13786089	0.12215	0.357851351	1.550707	2.15E-05	hypermethylated
cg11945824	0.16694	0.489048108	1.550647	1.65E-06	hypermethylated
cg02064106	0.10257	0.300405946	1.550305	0.000268	hypermethylated
cg10320659	0.13208	0.386727568	1.549906	4.82E-05	hypermethylated
cg01597641	0.13614	0.398588649	1.54981	5.45E-05	hypermethylated
cg11667086	0.11593	0.339362162	1.549572	3.79E-06	hypermethylated
cg21547690	0.13439	0.393331892	1.549321	1.58E-05	hypermethylated
cg02792792	0.11792	0.345065946	1.549064	3.76E-05	hypermethylated
cg24333192	0.09951	0.290983243	1.548023	5.19E-05	hypermethylated
cg15237923	0.09323	0.272608108	1.547962	7.52E-06	hypermethylated
cg21434530	0.1242	0.363148649	1.547895	6.01E-05	hypermethylated
cg04698114	0.14798	0.4325	1.547298	1.80E-05	hypermethylated
cg02662277	0.10232	0.299046486	1.547282	0.000159	hypermethylated
cg27443071	0.13363	0.390528649	1.547184	1.15E-05	hypermethylated
cg02613418	0.09562	0.279410811	1.547004	1.84E-05	hypermethylated
cg04180086	0.12912	0.377276216	1.546909	3.74E-06	hypermethylated
cg15762032	0.1025	0.299473514	1.546805	4.36E-05	hypermethylated
cg20266316	0.14101	0.411981622	1.546783	2.51E-05	hypermethylated
cg02895639	0.12495	0.365039459	1.546702	0.000224	hypermethylated
cg20277905	0.11983	0.349991892	1.546332	2.95E-06	hypermethylated
cg15549927	0.14454	0.422152432	1.546295	4.36E-06	hypermethylated
cg25901381	0.10715	0.312923784	1.546179	5.58E-06	hypermethylated
cg26385172	0.12919	0.377235676	1.545972	1.12E-05	hypermethylated
cg20676716	0.12719	0.371321081	1.545682	5.58E-05	hypermethylated
cg13791254	0.17436	0.508875135	1.545243	3.15E-05	hypermethylated
cg08097882	0.1153	0.336458919	1.545038	7.65E-05	hypermethylated
cg12514789	0.11761	0.342934595	1.543923	1.70E-06	hypermethylated
cg16023545	0.10329	0.301079459	1.543444	0.000335	hypermethylated
cg01019587	0.15299	0.445877297	1.543209	7.93E-06	hypermethylated

cg12520549	0.09911	0.288835135	1.543144	4.48E-06	hypermethylated
cg14907769	0.14725	0.429100541	1.543048	1.73E-05	hypermethylated
cg15175143	0.12186	0.355099459	1.542999	0.000109	hypermethylated
cg13526007	0.10712	0.312139459	1.542963	6.78E-05	hypermethylated
cg02825211	0.1395	0.406294595	1.542261	5.19E-05	hypermethylated
cg14262681	0.16463	0.479456757	1.542173	0.000667	hypermethylated
cg20405017	0.16062	0.467722162	1.542	8.37E-06	hypermethylated
cg15060599	0.16196	0.471507027	1.541642	4.36E-06	hypermethylated
cg03570636	0.09338	0.271848649	1.541618	5.00E-06	hypermethylated
cg11324832	0.12043	0.350476216	1.541122	1.58E-05	hypermethylated
cg06757662	0.09936	0.28914	1.541031	5.72E-05	hypermethylated
cg20504856	0.07166	0.208524324	1.540976	6.62E-05	hypermethylated
cg12741420	0.11067	0.321934054	1.540501	3.85E-05	hypermethylated
cg17269633	0.17211	0.500653514	1.540482	2.07E-06	hypermethylated
cg12004787	0.12949	0.376612432	1.54024	1.40E-05	hypermethylated
cg26327666	0.16648	0.484105405	1.539972	9.96E-06	hypermethylated
cg21179088	0.18627	0.541643784	1.539949	6.22E-06	hypermethylated
cg01910869	0.08974	0.260934054	1.539862	1.23E-05	hypermethylated
cg22546168	0.11782	0.342567568	1.539804	0.000132	hypermethylated
cg19149681	0.107	0.311092973	1.539735	2.15E-05	hypermethylated
cg14782672	0.10885	0.316447568	1.539625	4.01E-06	hypermethylated
cg23842255	0.12341	0.358696216	1.539303	2.56E-06	hypermethylated
cg15235798	0.13585	0.394824865	1.539198	1.39E-05	hypermethylated
cg06943420	0.12223	0.355114054	1.538684	0.000653	hypermethylated
cg21186708	0.11523	0.334728649	1.538476	1.99E-05	hypermethylated
cg26517714	0.12829	0.372614595	1.538275	1.63E-06	hypermethylated
cg11626496	0.1318	0.382804324	1.538257	4.48E-06	hypermethylated
cg10406124	0.12993	0.377320541	1.538056	4.86E-06	hypermethylated
cg20892260	0.13914	0.404028108	1.537918	3.07E-05	hypermethylated
cg15347189	0.12899	0.374552432	1.537908	6.05E-06	hypermethylated
cg26170569	0.14206	0.412373514	1.537451	8.26E-06	hypermethylated
cg15841063	0.14219	0.412744324	1.537428	0.000219	hypermethylated
cg17252960	0.11553	0.335187027	1.536699	1.99E-05	hypermethylated
cg10358981	0.12959	0.375961081	1.536629	6.46E-05	hypermethylated
cg07531268	0.09991	0.289771351	1.536214	9.38E-05	hypermethylated
cg08259925	0.08289	0.240374595	1.536014	5.19E-05	hypermethylated
cg08383399	0.12021	0.348591892	1.535982	7.29E-05	hypermethylated
cg26720125	0.10419	0.302115676	1.535884	4.47E-05	hypermethylated
cg09626193	0.09082	0.263229189	1.535238	9.05E-05	hypermethylated
cg22325703	0.07154	0.207294054	1.534857	0.00017	hypermethylated
cg27595860	0.13196	0.382340541	1.534757	1.62E-05	hypermethylated
cg19326876	0.10111	0.292931892	1.53464	8.52E-05	hypermethylated
cg27403635	0.13743	0.3981	1.534434	0.000112	hypermethylated
cg12640000	0.10568	0.306092973	1.534268	5.43E-06	hypermethylated
cg13272644	0.11946	0.345997838	1.534235	3.79E-06	hypermethylated
cg20051292	0.12123	0.351106486	1.534162	1.80E-05	hypermethylated
cg03166753	0.0987	0.285843243	1.534102	0.000667	hypermethylated
cg07341290	0.09799	0.283783784	1.534086	0.000178	hypermethylated
cg06424065	0.15824	0.458263784	1.534064	1.12E-05	hypermethylated
cg03707776	0.07166	0.207512432	1.533958	4.86E-06	hypermethylated
cg06531379	0.11915	0.345027568	1.533933	2.71E-05	hypermethylated
cg16203491	0.09426	0.272946486	1.533901	3.32E-05	hypermethylated
cg07837260	0.12796	0.370527027	1.533886	4.82E-05	hypermethylated
cg19555331	0.10497	0.303929189	1.533758	9.82E-06	hypermethylated
cg10700564	0.11085	0.320898378	1.533508	3.19E-05	hypermethylated
cg01625087	0.12074	0.349474595	1.533284	4.47E-05	hypermethylated
cg21127286	0.08604	0.249016216	1.53316	2.04E-05	hypermethylated
cg06545143	0.08853	0.256221622	1.533154	1.21E-05	hypermethylated

cg12164321	0.12628	0.365354054	1.532669	1.84E-05	hypermethylated
cg06369522	0.10069	0.291258378	1.532379	0.000277	hypermethylated
cg00960395	0.10941	0.316421622	1.532104	8.04E-06	hypermethylated
cg25037595	0.07876	0.227771351	1.532051	1.18E-06	hypermethylated
cg22851295	0.10265	0.296826486	1.531886	1.12E-05	hypermethylated
cg01527159	0.11443	0.330801622	1.531501	8.60E-06	hypermethylated
cg06905692	0.10387	0.300240541	1.53134	0.001141	hypermethylated
cg18252309	0.13126	0.379386486	1.531241	9.82E-06	hypermethylated
cg14482308	0.12514	0.361525405	1.530554	0.000234	hypermethylated
cg00745725	0.13139	0.379558378	1.530466	1.66E-05	hypermethylated
cg26163578	0.15408	0.445071351	1.530357	8.37E-06	hypermethylated
cg22897615	0.19017	0.5493	1.530305	6.39E-06	hypermethylated
cg23018092	0.09138	0.263871892	1.529887	0.000501	hypermethylated
cg26751356	0.14027	0.405004324	1.529731	8.15E-06	hypermethylated
cg22203547	0.09386	0.270970811	1.529555	2.64E-05	hypermethylated
cg25617340	0.08694	0.250987568	1.529524	8.48E-06	hypermethylated
cg23263937	0.17281	0.498727568	1.529065	9.31E-06	hypermethylated
cg02547394	0.15701	0.452991351	1.528627	2.57E-06	hypermethylated
cg04123385	0.08535	0.246231892	1.528555	6.31E-05	hypermethylated
cg03924566	0.09479	0.273464324	1.528546	6.70E-05	hypermethylated
cg11747183	0.11723	0.338184324	1.528468	5.72E-05	hypermethylated
cg20698924	0.12102	0.349095135	1.528375	3.95E-05	hypermethylated
cg06594404	0.1385	0.399512973	1.528356	1.62E-05	hypermethylated
cg01130127	0.13119	0.378392432	1.528225	2.48E-05	hypermethylated
cg16731240	0.13448	0.387868108	1.528175	0.000152	hypermethylated
cg03356595	0.16242	0.468424865	1.528088	1.21E-05	hypermethylated
cg11668923	0.12491	0.360243784	1.528085	1.99E-05	hypermethylated
cg26620530	0.11808	0.340541081	1.528064	0.000159	hypermethylated
cg00319545	0.07772	0.224141081	1.528049	3.23E-05	hypermethylated
cg23421023	0.10576	0.305000541	1.528018	5.86E-05	hypermethylated
cg25249539	0.09607	0.277028649	1.527877	2.27E-05	hypermethylated
cg01047586	0.16897	0.487203784	1.527758	3.67E-05	hypermethylated
cg22518208	0.08261	0.238195135	1.527756	2.10E-05	hypermethylated
cg15133351	0.14055	0.405204865	1.527568	3.00E-05	hypermethylated
cg01794426	0.10629	0.306383243	1.527332	1.18E-05	hypermethylated
cg05050657	0.13886	0.400248649	1.527265	5.58E-06	hypermethylated
cg12921750	0.09263	0.266994595	1.527259	2.64E-05	hypermethylated
cg01614102	0.17198	0.49570973	1.527255	2.49E-06	hypermethylated
cg00945238	0.08261	0.238108108	1.527228	1.58E-05	hypermethylated
cg01611862	0.07753	0.22344973	1.527124	1.75E-05	hypermethylated
cg05442408	0.0954	0.274942162	1.527067	1.73E-05	hypermethylated
cg25590492	0.0853	0.245807568	1.526912	3.15E-05	hypermethylated
cg08875948	0.11264	0.324586486	1.526884	4.36E-05	hypermethylated
cg02421985	0.13519	0.389557297	1.526847	8.60E-06	hypermethylated
cg04495336	0.085	0.244889189	1.526594	0.000123	hypermethylated
cg24298311	0.10299	0.296715676	1.526577	0.000104	hypermethylated
cg00824767	0.12832	0.369565405	1.526084	1.71E-05	hypermethylated
cg02157467	0.09047	0.260492432	1.52573	1.75E-05	hypermethylated
cg14642432	0.13784	0.396810811	1.525457	1.05E-05	hypermethylated
cg13891181	0.07345	0.211443784	1.52544	4.47E-05	hypermethylated
cg27319123	0.13627	0.392225946	1.525217	0.000109	hypermethylated
cg23266743	0.10991	0.316285946	1.524907	1.58E-05	hypermethylated
cg16606320	0.16834	0.484427027	1.524901	8.83E-06	hypermethylated
cg23760687	0.13439	0.386552432	1.524238	1.06E-05	hypermethylated
cg12568898	0.11441	0.32902973	1.524005	4.60E-06	hypermethylated
cg18135771	0.09753	0.28044	1.523774	1.60E-05	hypermethylated
cg15834072	0.16425	0.472112432	1.523237	2.71E-06	hypermethylated
cg13706544	0.09224	0.265074054	1.522931	5.45E-05	hypermethylated

cg27546237	0.10462	0.300554595	1.522468	7.84E-05	hypermethylated
cg21230745	0.12623	0.362627568	1.522434	1.25E-05	hypermethylated
cg14714046	0.11689	0.335696757	1.522007	1.67E-06	hypermethylated
cg05085500	0.13067	0.375230811	1.52185	1.99E-05	hypermethylated
cg11500467	0.10425	0.299344324	1.521759	3.76E-05	hypermethylated
cg16599703	0.13494	0.387467568	1.521757	4.24E-06	hypermethylated
cg16758813	0.09613	0.275984865	1.521531	1.94E-05	hypermethylated
cg01728554	0.08945	0.256792973	1.521452	1.35E-05	hypermethylated
cg03262554	0.14942	0.428765405	1.520815	1.85E-06	hypermethylated
cg02062480	0.11582	0.332324865	1.52071	3.11E-05	hypermethylated
cg06749715	0.11193	0.320994595	1.519952	2.92E-05	hypermethylated
cg20266154	0.11492	0.329457297	1.519462	9.60E-05	hypermethylated
cg16280141	0.10243	0.293601081	1.519219	1.12E-05	hypermethylated
cg10414975	0.11252	0.322500541	1.51912	3.96E-06	hypermethylated
cg18022662	0.08234	0.235978919	1.518993	6.75E-06	hypermethylated
cg02765859	0.09086	0.26036	1.518791	5.06E-05	hypermethylated
cg14780416	0.09411	0.269658378	1.518713	0.000741	hypermethylated
cg21802055	0.16744	0.479715135	1.518534	5.43E-06	hypermethylated
cg15604467	0.08831	0.252984324	1.518399	0.000123	hypermethylated
cg08445802	0.15971	0.457518378	1.518375	4.36E-06	hypermethylated
cg13866527	0.12703	0.363842703	1.518146	8.63E-05	hypermethylated
cg17078116	0.15368	0.440123243	1.517978	5.73E-06	hypermethylated
cg17871749	0.09319	0.266856757	1.517818	1.21E-05	hypermethylated
cg24736989	0.12379	0.354353514	1.517295	4.48E-06	hypermethylated
cg23556443	0.11048	0.316229189	1.517185	2.71E-05	hypermethylated
cg25372296	0.1434	0.410407027	1.51701	5.00E-06	hypermethylated
cg10239098	0.12136	0.347319459	1.51697	6.46E-05	hypermethylated
cg01994205	0.11064	0.316638378	1.516963	0.000202	hypermethylated
cg00806490	0.10873	0.311117838	1.516711	6.57E-06	hypermethylated
cg25201511	0.10666	0.304983784	1.515713	8.98E-07	hypermethylated
cg08087868	0.15431	0.441227568	1.515691	3.21E-06	hypermethylated
cg07095230	0.15205	0.434684865	1.515424	3.15E-05	hypermethylated
cg25922637	0.07619	0.217814054	1.515423	1.99E-05	hypermethylated
cg01185626	0.10372	0.296504324	1.515359	8.83E-06	hypermethylated
cg21063758	0.08548	0.244358919	1.515343	0.000129	hypermethylated
cg27313642	0.11374	0.325111351	1.515194	1.15E-05	hypermethylated
cg08453036	0.0924	0.264107027	1.515158	8.15E-06	hypermethylated
cg19547319	0.12894	0.368525946	1.515066	1.25E-05	hypermethylated
cg17537177	0.11196	0.319915135	1.514706	0.000102	hypermethylated
cg04168853	0.13831	0.395183243	1.514616	5.73E-06	hypermethylated
cg07030506	0.07431	0.212314595	1.514575	6.66E-06	hypermethylated
cg17029354	0.12122	0.346167568	1.513843	2.71E-05	hypermethylated
cg08662665	0.07733	0.220793514	1.513598	4.54E-06	hypermethylated
cg20631104	0.11568	0.330276216	1.513534	6.15E-05	hypermethylated
cg17101296	0.14102	0.402558919	1.5133	0.000214	hypermethylated
cg03843972	0.10415	0.297272432	1.513123	3.40E-05	hypermethylated
cg13338350	0.07802	0.222671892	1.513004	3.95E-05	hypermethylated
cg09323727	0.15309	0.436825946	1.512679	0.00024	hypermethylated
cg10217445	0.1029	0.293571892	1.512471	8.37E-06	hypermethylated
cg23938300	0.08724	0.248850811	1.512219	9.83E-05	hypermethylated
cg01396387	0.10083	0.287548108	1.511878	4.26E-05	hypermethylated
cg07549278	0.10231	0.291724324	1.511659	9.83E-05	hypermethylated
cg27403265	0.10379	0.295824865	1.511076	2.67E-05	hypermethylated
cg16142306	0.12809	0.365042703	1.510907	4.70E-05	hypermethylated
cg17542408	0.16958	0.48296973	1.509967	1.82E-05	hypermethylated
cg18877285	0.10408	0.296353514	1.509626	1.12E-05	hypermethylated
cg06883126	0.1447	0.412009189	1.509612	2.49E-06	hypermethylated
cg13434411	0.13239	0.376894054	1.509365	9.96E-06	hypermethylated

cg03245632	0.08675	0.24692	1.509108	2.57E-05	hypermethylated
cg14231297	0.14584	0.415040541	1.508866	1.28E-05	hypermethylated
cg04178787	0.15993	0.455036216	1.508541	3.15E-05	hypermethylated
cg09549813	0.11023	0.313621081	1.508506	0.000219	hypermethylated
cg21553182	0.0973	0.276815676	1.508414	1.84E-05	hypermethylated
cg13000912	0.09538	0.271291351	1.508084	0.000112	hypermethylated
cg06581175	0.13689	0.389195676	1.507479	4.93E-06	hypermethylated
cg05227131	0.11582	0.329268108	1.507378	6.95E-05	hypermethylated
cg02279719	0.10528	0.29900973	1.505961	9.95E-05	hypermethylated
cg26351229	0.12535	0.356001081	1.50592	1.05E-05	hypermethylated
cg11552072	0.14	0.39751027	1.505565	2.45E-05	hypermethylated
cg08361126	0.12951	0.367702703	1.505476	1.12E-05	hypermethylated
cg15877129	0.09476	0.269010811	1.505314	7.12E-05	hypermethylated
cg03576469	0.134	0.380378919	1.505204	6.54E-05	hypermethylated
cg22131234	0.09153	0.259791351	1.505037	0.000129	hypermethylated
cg26157385	0.13448	0.381668108	1.504927	1.58E-06	hypermethylated
cg14884793	0.08334	0.236503243	1.504779	0.000174	hypermethylated
cg09562322	0.11024	0.312823243	1.5047	7.65E-05	hypermethylated
cg23820885	0.08365	0.237366486	1.504679	1.06E-05	hypermethylated
cg18699744	0.11624	0.329713514	1.504106	0.000234	hypermethylated
cg21701379	0.12388	0.351375135	1.504069	2.27E-05	hypermethylated
cg11296759	0.11863	0.336418378	1.503788	1.33E-06	hypermethylated
cg10542584	0.08003	0.226937838	1.503684	2.32E-06	hypermethylated
cg03215105	0.10585	0.300050811	1.503186	5.86E-05	hypermethylated
cg01715455	0.09726	0.275676216	1.503056	0.00062	hypermethylated
cg26608174	0.12082	0.342388108	1.502773	1.46E-05	hypermethylated
cg03237606	0.10544	0.298775135	1.502638	1.89E-05	hypermethylated
cg07060551	0.13723	0.388817297	1.502496	9.31E-06	hypermethylated
cg23288973	0.11926	0.337870811	1.502361	3.40E-05	hypermethylated
cg01002529	0.12435	0.352278378	1.502309	5.00E-05	hypermethylated
cg00460235	0.09068	0.256890811	1.502299	2.10E-05	hypermethylated
cg11686427	0.15857	0.448914595	1.501321	8.15E-06	hypermethylated
cg04641171	0.11453	0.323920541	1.499914	5.00E-06	hypermethylated
cg06057566	0.09289	0.262696757	1.499803	5.00E-05	hypermethylated
cg03234186	0.13289	0.375699459	1.499346	6.31E-05	hypermethylated
cg23490887	0.09637	0.272441081	1.499288	3.07E-05	hypermethylated
cg05718036	0.10149	0.286911351	1.499267	0.00035	hypermethylated
cg17603132	0.10186	0.287937838	1.49917	1.21E-05	hypermethylated
cg23424003	0.14884	0.420644865	1.49884	0.000112	hypermethylated
cg04469880	0.09333	0.263762162	1.498825	1.23E-05	hypermethylated
cg07777790	0.13391	0.37826973	1.498152	2.57E-06	hypermethylated
cg17677030	0.13119	0.370583784	1.498142	1.42E-05	hypermethylated
cg05214685	0.13169	0.371967027	1.498029	4.24E-06	hypermethylated
cg02106850	0.10333	0.291763243	1.497539	0.000287	hypermethylated
cg15930596	0.09927	0.28026973	1.497386	2.51E-05	hypermethylated
cg20027133	0.11554	0.326178378	1.497269	6.31E-05	hypermethylated
cg18956933	0.15652	0.441763243	1.496926	4.01E-06	hypermethylated
cg02452944	0.15558	0.439053514	1.49674	1.04E-05	hypermethylated
cg09884341	0.12571	0.354748649	1.496698	9.82E-06	hypermethylated
cg12600018	0.08052	0.227203243	1.496564	0.000112	hypermethylated
cg14179581	0.10548	0.297614595	1.496476	4.42E-05	hypermethylated
cg11323198	0.1295	0.365367568	1.496396	1.01E-05	hypermethylated
cg10384245	0.14172	0.399772973	1.496138	2.71E-06	hypermethylated
cg08780398	0.0849	0.239436216	1.495805	4.88E-05	hypermethylated
cg11751806	0.09501	0.267945405	1.495788	2.71E-05	hypermethylated
cg09674215	0.11249	0.317237297	1.495766	4.86E-06	hypermethylated
cg14882700	0.1361	0.383775135	1.495594	6.95E-05	hypermethylated
cg22697574	0.11113	0.313291351	1.495257	0.000126	hypermethylated

cg18201679	0.11466	0.323104324	1.494638	1.75E-05	hypermethylated
cg15949044	0.12447	0.350621622	1.494117	1.42E-05	hypermethylated
cg25645064	0.12653	0.356365405	1.493878	3.32E-05	hypermethylated
cg14414464	0.12274	0.345547027	1.493277	5.43E-06	hypermethylated
cg00840332	0.1527	0.429875676	1.493219	4.94E-05	hypermethylated
cg11502555	0.12974	0.365177297	1.492974	3.07E-05	hypermethylated
cg25953239	0.1559	0.438808649	1.492971	2.51E-05	hypermethylated
cg12980692	0.09077	0.255487568	1.492966	3.00E-05	hypermethylated
cg24954895	0.15573	0.43826973	1.492772	1.37E-06	hypermethylated
cg16254309	0.12094	0.340306486	1.492543	9.95E-05	hypermethylated
cg01410472	0.09015	0.253603243	1.492174	7.38E-05	hypermethylated
cg09382127	0.14381	0.404547568	1.492145	2.57E-06	hypermethylated
cg11716272	0.11285	0.317454595	1.492144	1.18E-05	hypermethylated
cg05137466	0.07509	0.211221081	1.492061	0.000104	hypermethylated
cg05121480	0.11059	0.311073514	1.492035	3.07E-05	hypermethylated
cg17078686	0.14112	0.396914595	1.491906	1.66E-05	hypermethylated
cg09423283	0.10037	0.282177838	1.491277	6.75E-06	hypermethylated
cg21319932	0.09886	0.2779	1.491107	8.42E-05	hypermethylated
cg03834286	0.10788	0.303196216	1.490824	2.16E-06	hypermethylated
cg02391713	0.22735	0.638500541	1.489773	5.00E-06	hypermethylated
cg09042277	0.12953	0.363769189	1.489737	1.01E-05	hypermethylated
cg03755123	0.10029	0.281639459	1.489672	9.57E-06	hypermethylated
cg15333426	0.09773	0.274444865	1.489643	8.04E-06	hypermethylated
cg14186066	0.11867	0.333228108	1.489555	9.07E-06	hypermethylated
cg05658487	0.1238	0.347539459	1.489165	3.39E-06	hypermethylated
cg15795787	0.09608	0.269666486	1.488868	2.39E-05	hypermethylated
cg04931256	0.11868	0.333003243	1.488459	2.07E-05	hypermethylated
cg07035961	0.16583	0.465275135	1.488379	1.63E-06	hypermethylated
cg21000072	0.10512	0.294889189	1.488136	7.03E-06	hypermethylated
cg16884902	0.1085	0.304320541	1.487897	6.78E-05	hypermethylated
cg11870560	0.10903	0.305801081	1.487868	4.36E-05	hypermethylated
cg16733705	0.12506	0.35075027	1.487824	2.51E-05	hypermethylated
cg09972259	0.1049	0.294123243	1.487406	9.96E-06	hypermethylated
cg25636665	0.12902	0.361747027	1.487386	2.78E-05	hypermethylated
cg23130254	0.10939	0.306649189	1.487108	7.72E-06	hypermethylated
cg16355785	0.12079	0.338554595	1.486887	7.12E-05	hypermethylated
cg19968840	0.15207	0.426170811	1.486696	1.66E-05	hypermethylated
cg22746058	0.12373	0.346731892	1.486625	4.15E-05	hypermethylated
cg12597389	0.14042	0.39345027	1.486433	3.90E-06	hypermethylated
cg10084644	0.14863	0.416450811	1.486421	1.80E-05	hypermethylated
cg08146323	0.16824	0.471373514	1.48635	3.54E-06	hypermethylated
cg01370541	0.09725	0.272417297	1.486048	2.92E-05	hypermethylated
cg03816707	0.17624	0.493663784	1.485987	1.41E-06	hypermethylated
cg15325658	0.08335	0.233469189	1.485978	2.51E-05	hypermethylated
cg00239870	0.09703	0.271776216	1.485916	0.002432	hypermethylated
cg11229862	0.11457	0.320883784	1.485822	9.95E-05	hypermethylated
cg25247290	0.20622	0.577552432	1.485768	1.39E-05	hypermethylated
cg20099830	0.14017	0.392537838	1.485654	5.89E-06	hypermethylated
cg18582824	0.12868	0.360338378	1.485564	1.06E-05	hypermethylated
cg00777627	0.09777	0.273723784	1.485257	0.000733	hypermethylated
cg02049629	0.10668	0.29858973	1.484875	2.10E-05	hypermethylated
cg03733219	0.12687	0.355072973	1.484765	3.85E-05	hypermethylated
cg00378730	0.09671	0.270648108	1.484681	8.63E-05	hypermethylated
cg01405404	0.13797	0.386075676	1.484529	2.39E-05	hypermethylated
cg27211284	0.13755	0.384832432	1.484274	9.82E-06	hypermethylated
cg03700462	0.08085	0.226174595	1.484117	0.000219	hypermethylated
cg06674731	0.12599	0.352380541	1.483825	0.000155	hypermethylated
cg15594205	0.14309	0.400188649	1.483757	4.26E-05	hypermethylated

cg08426444	0.10594	0.296281622	1.483722	9.16E-05	hypermethylated
cg17156743	0.08689	0.243001622	1.483704	2.71E-05	hypermethylated
cg13906811	0.14232	0.397921081	1.483344	5.00E-06	hypermethylated
cg22070991	0.14622	0.408797297	1.483245	2.45E-05	hypermethylated
cg25559313	0.10981	0.306837297	1.482464	2.96E-05	hypermethylated
cg00854817	0.10019	0.27995027	1.482432	5.97E-06	hypermethylated
cg18862481	0.17144	0.478862703	1.481908	6.22E-06	hypermethylated
cg16498913	0.12634	0.352882703	1.481877	4.94E-05	hypermethylated
cg03769383	0.12217	0.341159459	1.481556	2.45E-05	hypermethylated
cg15965134	0.10854	0.30309027	1.481521	1.50E-05	hypermethylated
cg24534135	0.09413	0.262754054	1.480987	4.70E-05	hypermethylated
cg00452257	0.12268	0.342437838	1.480942	0.000166	hypermethylated
cg09578028	0.17352	0.484133514	1.480303	6.66E-06	hypermethylated
cg24496475	0.18307	0.51075027	1.480223	3.90E-06	hypermethylated
cg02841000	0.1482	0.413068108	1.478834	9.57E-06	hypermethylated
cg00282510	0.12759	0.355537838	1.478488	4.53E-05	hypermethylated
cg08278741	0.11004	0.306616216	1.478406	7.65E-05	hypermethylated
cg14223293	0.1267	0.353029189	1.478371	2.23E-06	hypermethylated
cg15520279	0.14862	0.414077838	1.478274	4.80E-06	hypermethylated
cg18844382	0.12381	0.34491027	1.478093	0.000805	hypermethylated
cg09904497	0.08128	0.226425946	1.478067	5.97E-06	hypermethylated
cg25205699	0.10526	0.293208108	1.477968	3.90E-05	hypermethylated
cg21751684	0.12649	0.352333514	1.477918	1.75E-05	hypermethylated
cg06066137	0.0983	0.273783784	1.477774	6.66E-06	hypermethylated
cg08280383	0.11123	0.309793514	1.477761	1.75E-05	hypermethylated
cg13906669	0.09198	0.256165946	1.477687	6.46E-05	hypermethylated
cg12233363	0.12272	0.341735135	1.477508	1.06E-05	hypermethylated
cg10966580	0.15811	0.440236216	1.477349	8.37E-06	hypermethylated
cg17971015	0.12563	0.34979027	1.477309	0.000207	hypermethylated
cg06721255	0.10134	0.282122162	1.477116	4.76E-05	hypermethylated
cg22304612	0.13217	0.367913514	1.476972	4.20E-05	hypermethylated
cg11788523	0.11242	0.312915676	1.476875	8.37E-06	hypermethylated
cg06243400	0.10407	0.289555135	1.476284	1.99E-05	hypermethylated
cg18090004	0.11725	0.326157297	1.47598	0.000124	hypermethylated
cg06713632	0.09803	0.272617297	1.475582	0.000454	hypermethylated
cg09521703	0.15122	0.420524324	1.47554	9.96E-06	hypermethylated
cg24005685	0.14539	0.404221081	1.475217	9.07E-06	hypermethylated
cg12550866	0.09078	0.252385946	1.475185	3.32E-05	hypermethylated
cg20000718	0.09408	0.261422703	1.474424	7.03E-05	hypermethylated
cg19267252	0.1537	0.427087568	1.474415	9.82E-06	hypermethylated
cg22864077	0.14006	0.389178919	1.474389	7.93E-06	hypermethylated
cg10908460	0.1046	0.290639459	1.474348	0.000354	hypermethylated
cg09960109	0.10105	0.280770811	1.474324	6.30E-06	hypermethylated
cg11950383	0.12397	0.344398378	1.474087	2.81E-05	hypermethylated
cg13720108	0.10076	0.279900541	1.473991	4.15E-05	hypermethylated
cg22278296	0.1031	0.286395676	1.473965	0.000588	hypermethylated
cg19672997	0.14725	0.409013514	1.473881	3.49E-06	hypermethylated
cg24620761	0.12891	0.358041622	1.473763	1.89E-05	hypermethylated
cg20332645	0.1317	0.365757838	1.473633	5.43E-06	hypermethylated
cg25784308	0.10171	0.282456216	1.473566	0.000117	hypermethylated
cg02455820	0.15789	0.438245946	1.472821	1.01E-05	hypermethylated
cg23689712	0.10735	0.297921622	1.472611	5.72E-05	hypermethylated
cg11784623	0.08642	0.23981027	1.472456	6.54E-05	hypermethylated
cg05588496	0.09165	0.254322703	1.472453	3.15E-05	hypermethylated
cg20044410	0.13438	0.372860541	1.472318	7.32E-06	hypermethylated
cg19585597	0.11032	0.305984865	1.471766	2.64E-05	hypermethylated
cg02111032	0.15181	0.421054054	1.471739	6.75E-06	hypermethylated
cg16803520	0.12139	0.336644865	1.471578	3.49E-05	hypermethylated

cg09792204	0.12779	0.35436973	1.47148	0.000172	hypermethylated
cg05667379	0.12762	0.353894595	1.471465	1.99E-05	hypermethylated
cg08152546	0.15543	0.430998378	1.471417	3.49E-06	hypermethylated
cg22206608	0.12646	0.350631351	1.471274	8.37E-06	hypermethylated
cg14056110	0.11958	0.331476757	1.470932	3.57E-05	hypermethylated
cg24721899	0.1545	0.428267027	1.470904	1.42E-05	hypermethylated
cg27365825	0.12429	0.344456216	1.47061	5.86E-05	hypermethylated
cg25809841	0.11458	0.317532973	1.470551	3.27E-05	hypermethylated
cg07138551	0.09714	0.269138919	1.470214	1.75E-05	hypermethylated
cg12118269	0.11698	0.324064865	1.470021	0.000358	hypermethylated
cg22271921	0.09759	0.270349189	1.470019	7.12E-05	hypermethylated
cg25681339	0.13381	0.370564865	1.46954	3.85E-05	hypermethylated
cg07647164	0.11625	0.321923784	1.469488	5.12E-05	hypermethylated
cg16845394	0.14074	0.389683784	1.469271	6.15E-05	hypermethylated
cg14095438	0.11682	0.323328649	1.468714	8.63E-05	hypermethylated
cg14074285	0.10354	0.286487568	1.468284	7.38E-05	hypermethylated
cg01883425	0.12478	0.345175676	1.467944	1.01E-05	hypermethylated
cg06984025	0.09556	0.264330811	1.467866	3.12E-06	hypermethylated
cg09554951	0.16322	0.451485946	1.467863	6.22E-06	hypermethylated
cg04150055	0.11256	0.31134973	1.467842	0.000209	hypermethylated
cg14614811	0.10681	0.295398919	1.467618	2.75E-06	hypermethylated
cg05470523	0.10849	0.299942703	1.467125	1.84E-05	hypermethylated
cg17328472	0.09482	0.26212973	1.467018	5.51E-05	hypermethylated
cg05788437	0.05766	0.159388108	1.466901	5.06E-05	hypermethylated
cg05052450	0.11763	0.325098919	1.466623	6.95E-05	hypermethylated
cg22344727	0.11727	0.324086486	1.466545	4.01E-06	hypermethylated
cg23262036	0.14672	0.405462162	1.466502	2.39E-05	hypermethylated
cg21158087	0.14958	0.413341622	1.466417	3.59E-06	hypermethylated
cg24874180	0.12416	0.342968108	1.465874	2.15E-05	hypermethylated
cg16206813	0.16083	0.444244865	1.465819	4.12E-06	hypermethylated
cg01189783	0.08545	0.236022703	1.465773	3.95E-05	hypermethylated
cg26620710	0.09827	0.271378378	1.465483	1.25E-05	hypermethylated
cg03794801	0.10603	0.292774595	1.465318	6.46E-05	hypermethylated
cg24583624	0.11333	0.312885946	1.465107	3.07E-05	hypermethylated
cg03209854	0.13234	0.365349189	1.465027	7.52E-06	hypermethylated
cg09322259	0.12626	0.348556757	1.464996	7.29E-05	hypermethylated
cg21647227	0.13664	0.377177838	1.464865	3.35E-06	hypermethylated
cg00339488	0.11486	0.317038919	1.464783	1.71E-05	hypermethylated
cg08291024	0.11771	0.324889189	1.464711	1.58E-05	hypermethylated
cg08977390	0.13036	0.359751351	1.464499	1.63E-06	hypermethylated
cg03464573	0.102	0.281486486	1.464497	8.71E-06	hypermethylated
cg05422683	0.09481	0.261553514	1.463995	5.93E-05	hypermethylated
cg04140663	0.1557	0.429495135	1.463873	1.89E-05	hypermethylated
cg07595776	0.13681	0.377327027	1.463642	8.15E-06	hypermethylated
cg23189410	0.19533	0.538682703	1.463522	5.73E-06	hypermethylated
cg21726372	0.13481	0.371723243	1.463301	5.72E-05	hypermethylated
cg10049708	0.10967	0.302368108	1.463137	4.86E-06	hypermethylated
cg04079301	0.11748	0.323846486	1.462895	7.93E-06	hypermethylated
cg27638727	0.1459	0.402176216	1.462848	5.00E-06	hypermethylated
cg03985727	0.17089	0.471031892	1.462757	2.10E-05	hypermethylated
cg19925849	0.1342	0.369868649	1.462628	1.58E-05	hypermethylated
cg15446990	0.08685	0.239332973	1.462421	1.17E-05	hypermethylated
cg27147819	0.0748	0.206114054	1.462333	0.000112	hypermethylated
cg06544111	0.16955	0.467142162	1.462151	2.27E-05	hypermethylated
cg20401252	0.10736	0.295755676	1.461949	9.82E-06	hypermethylated
cg10379890	0.10951	0.301556216	1.461364	9.27E-05	hypermethylated
cg14009098	0.11148	0.30696	1.461266	5.25E-05	hypermethylated
cg09393254	0.11044	0.304067027	1.461127	3.32E-05	hypermethylated

cg00924143	0.07104	0.195581622	1.461067	3.57E-05	hypermethylated
cg04613834	0.11353	0.312489189	1.460733	1.82E-05	hypermethylated
cg13378628	0.07865	0.216450811	1.460521	0.000115	hypermethylated
cg19095600	0.1143	0.314547027	1.46045	2.10E-05	hypermethylated
cg26452056	0.1087	0.299087568	1.460216	9.82E-06	hypermethylated
cg13342435	0.13069	0.359558378	1.460077	2.21E-05	hypermethylated
cg23437459	0.10943	0.301022162	1.459861	5.31E-05	hypermethylated
cg08528626	0.13823	0.380154054	1.459513	5.86E-05	hypermethylated
cg27180153	0.16201	0.44541027	1.459052	1.96E-06	hypermethylated
cg04138502	0.1068	0.293608108	1.45898	0.00097	hypermethylated
cg10068300	0.11256	0.309441081	1.458971	9.96E-06	hypermethylated
cg01175812	0.08339	0.229225946	1.458824	3.04E-05	hypermethylated
cg01560873	0.16877	0.463832973	1.458547	1.04E-05	hypermethylated
cg08286181	0.14455	0.397127568	1.458034	6.57E-06	hypermethylated
cg09986292	0.11194	0.307520541	1.457957	1.40E-05	hypermethylated
cg03369269	0.10483	0.287959459	1.457814	0.000534	hypermethylated
cg03698009	0.12775	0.350878378	1.457648	1.08E-05	hypermethylated
cg21575295	0.09625	0.264352432	1.457604	1.50E-05	hypermethylated
cg21606928	0.19796	0.54360973	1.457362	8.83E-06	hypermethylated
cg26195356	0.15202	0.417352432	1.457005	1.58E-05	hypermethylated
cg22055728	0.09805	0.269172973	1.456944	1.28E-05	hypermethylated
cg00470955	0.08807	0.241765946	1.456888	2.51E-05	hypermethylated
cg03469054	0.139	0.381536216	1.456735	2.78E-05	hypermethylated
cg23967544	0.13705	0.376123243	1.456503	2.57E-06	hypermethylated
cg01916115	0.1162	0.318817297	1.45612	1.33E-05	hypermethylated
cg03265671	0.10979	0.301207027	1.456009	3.00E-05	hypermethylated
cg00441573	0.13464	0.369325405	1.455785	2.64E-05	hypermethylated
cg14631386	0.11027	0.302465405	1.45573	0.000303	hypermethylated
cg09655666	0.16994	0.466136757	1.455728	2.78E-05	hypermethylated
cg00263760	0.13546	0.371550811	1.455693	0.00012	hypermethylated
cg07664198	0.15412	0.422719459	1.455646	9.57E-06	hypermethylated
cg07466705	0.12767	0.350104865	1.455368	8.63E-05	hypermethylated
cg21831931	0.11827	0.324308108	1.455281	7.72E-06	hypermethylated
cg07102705	0.11378	0.311976216	1.455189	9.49E-05	hypermethylated
cg15086113	0.11804	0.323641622	1.455121	6.62E-05	hypermethylated
cg08638184	0.12449	0.341325946	1.45512	2.39E-05	hypermethylated
cg13816321	0.14186	0.388934595	1.45506	7.42E-06	hypermethylated
cg21754400	0.1067	0.292522162	1.454986	3.85E-05	hypermethylated
cg18628094	0.12749	0.34947027	1.454786	1.39E-05	hypermethylated
cg10915263	0.11129	0.305009189	1.454529	9.69E-06	hypermethylated
cg16377881	0.11926	0.326765946	1.454147	0.000449	hypermethylated
cg15829088	0.14716	0.403194054	1.454089	5.58E-06	hypermethylated
cg06550462	0.09588	0.262646486	1.45382	2.21E-05	hypermethylated
cg14283758	0.111	0.304025946	1.453635	0.000126	hypermethylated
cg07897690	0.10243	0.280488649	1.453304	2.64E-06	hypermethylated
cg09899185	0.14663	0.40142	1.452932	2.21E-05	hypermethylated
cg11308277	0.10681	0.29236973	1.452747	3.04E-05	hypermethylated
cg12105941	0.16725	0.457743243	1.452532	2.27E-05	hypermethylated
cg23381749	0.10378	0.283978919	1.452255	4.76E-05	hypermethylated
cg05576262	0.16399	0.448702162	1.45215	1.94E-05	hypermethylated
cg26071016	0.13415	0.367049189	1.452126	8.04E-06	hypermethylated
cg07919443	0.12007	0.32850973	1.45206	7.65E-05	hypermethylated
cg18115040	0.16048	0.439065405	1.452042	5.73E-06	hypermethylated
cg18907180	0.12103	0.331091351	1.451865	1.92E-05	hypermethylated
cg02509825	0.10324	0.282405405	1.451766	4.60E-06	hypermethylated
cg09486778	0.14003	0.382939459	1.45138	2.96E-05	hypermethylated
cg03608712	0.08813	0.241003243	1.451347	3.76E-05	hypermethylated
cg06567373	0.13912	0.380214054	1.450482	1.58E-06	hypermethylated

cg04127342	0.1696	0.463448108	1.450272	1.18E-05	hypermethylated
cg17863912	0.13323	0.364048649	1.450212	7.32E-06	hypermethylated
cg10281002	0.13113	0.35812	1.449445	2.35E-05	hypermethylated
cg05107500	0.11392	0.311044324	1.449099	7.52E-06	hypermethylated
cg02631462	0.10733	0.293005946	1.448877	0.000152	hypermethylated
cg26312150	0.11787	0.321745946	1.448725	1.66E-05	hypermethylated
cg13398291	0.11019	0.300759459	1.448617	1.58E-05	hypermethylated
cg05697849	0.17699	0.482994054	1.448338	1.04E-05	hypermethylated
cg23618440	0.11691	0.318957838	1.447967	0.000166	hypermethylated
cg07788092	0.15597	0.425481622	1.447828	5.73E-06	hypermethylated
cg16795307	0.15374	0.419317838	1.447552	2.51E-05	hypermethylated
cg14852384	0.12957	0.353266486	1.447025	5.73E-06	hypermethylated
cg23668184	0.15616	0.425729189	1.446911	2.18E-05	hypermethylated
cg24923694	0.14915	0.406614054	1.446896	1.15E-05	hypermethylated
cg12973941	0.15369	0.418977297	1.446849	5.07E-06	hypermethylated
cg22885558	0.11243	0.306497297	1.446847	1.62E-05	hypermethylated
cg20950011	0.10424	0.284112973	1.446556	1.50E-05	hypermethylated
cg10334354	0.13283	0.362006486	1.446435	3.40E-05	hypermethylated
cg04413320	0.13447	0.366473514	1.446425	2.04E-05	hypermethylated
cg11060276	0.09531	0.259717297	1.446243	3.36E-05	hypermethylated
cg05640128	0.13169	0.358824324	1.446132	3.95E-05	hypermethylated
cg06015218	0.09874	0.268996757	1.445882	0.000107	hypermethylated
cg08132837	0.07916	0.215631351	1.445723	2.51E-05	hypermethylated
cg27338487	0.09827	0.267673514	1.445651	7.72E-06	hypermethylated
cg18912209	0.11682	0.318117297	1.445272	0.000155	hypermethylated
cg20981086	0.18302	0.498385946	1.445262	4.86E-06	hypermethylated
cg21639114	0.11337	0.308712973	1.445227	3.49E-05	hypermethylated
cg02115911	0.14038	0.382257838	1.445209	4.82E-05	hypermethylated
cg03152288	0.08641	0.235295676	1.445205	3.00E-05	hypermethylated
cg23676551	0.12024	0.327388649	1.445087	0.000374	hypermethylated
cg03041738	0.11073	0.301474595	1.44499	0.0002	hypermethylated
cg11743349	0.10726	0.29201027	1.444907	1.02E-05	hypermethylated
cg06812991	0.10182	0.277157297	1.444684	2.32E-05	hypermethylated
cg18026588	0.11924	0.324536216	1.444511	9.95E-05	hypermethylated
cg03672342	0.13566	0.369207027	1.444435	7.84E-05	hypermethylated
cg15267232	0.14579	0.396611892	1.443836	0.000335	hypermethylated
cg12166610	0.14183	0.385757838	1.443533	3.15E-05	hypermethylated
cg16419354	0.1395	0.379307027	1.443101	7.47E-05	hypermethylated
cg17918002	0.11534	0.313554595	1.442824	1.04E-05	hypermethylated
cg10633658	0.11309	0.307331892	1.442326	3.21E-06	hypermethylated
cg00714740	0.14103	0.383243784	1.44226	1.75E-05	hypermethylated
cg22605303	0.10324	0.28053027	1.442154	6.05E-06	hypermethylated
cg16764637	0.13078	0.355350811	1.442102	8.37E-06	hypermethylated
cg26568372	0.15493	0.42092	1.44193	5.73E-06	hypermethylated
cg00840960	0.11964	0.325015676	1.441809	0.000219	hypermethylated
cg06737561	0.16373	0.444744324	1.441658	6.39E-06	hypermethylated
cg06842954	0.13687	0.37178	1.441643	3.00E-05	hypermethylated
cg04122873	0.12752	0.346322703	1.441393	0.000426	hypermethylated
cg09831105	0.09749	0.264761622	1.441368	0.00017	hypermethylated
cg06922606	0.12047	0.327155676	1.441303	3.67E-05	hypermethylated
cg13066983	0.12707	0.345017838	1.441047	0.000117	hypermethylated
cg08053686	0.13245	0.359623784	1.441041	5.43E-06	hypermethylated
cg21210758	0.13074	0.354977838	1.441028	4.15E-05	hypermethylated
cg23954787	0.12659	0.343663784	1.440834	1.09E-05	hypermethylated
cg03656899	0.10534	0.285945405	1.440686	2.15E-05	hypermethylated
cg03599078	0.17037	0.462463243	1.440667	1.93E-06	hypermethylated
cg16240162	0.13871	0.376364324	1.440058	1.94E-05	hypermethylated
cg16804284	0.14194	0.385116757	1.440015	5.65E-05	hypermethylated

cg11248413	0.11271	0.305738378	1.439682	0.000132	hypermethylated
cg01650904	0.12262	0.332566486	1.439448	6.75E-06	hypermethylated
cg02078690	0.16453	0.446030811	1.438793	8.15E-06	hypermethylated
cg10414985	0.12806	0.347155135	1.438761	1.09E-05	hypermethylated
cg22048474	0.10205	0.276636216	1.438714	5.72E-05	hypermethylated
cg16404157	0.16242	0.440268649	1.438655	3.00E-05	hypermethylated
cg16206659	0.16002	0.433659459	1.43831	1.94E-05	hypermethylated
cg16940012	0.13757	0.372802703	1.438246	9.82E-06	hypermethylated
cg09471659	0.13265	0.35944973	1.438165	3.23E-05	hypermethylated
cg06444755	0.11345	0.307311892	1.437647	2.57E-05	hypermethylated
cg03764518	0.13632	0.369174054	1.437304	1.39E-05	hypermethylated
cg11755405	0.1307	0.353927027	1.437193	4.58E-05	hypermethylated
cg25136495	0.16836	0.455884324	1.437118	1.21E-05	hypermethylated
cg10679301	0.08842	0.239398919	1.436972	6.78E-05	hypermethylated
cg11600596	0.13979	0.378389189	1.43661	6.46E-05	hypermethylated
cg10972873	0.12484	0.337894595	1.436493	2.10E-05	hypermethylated
cg07135732	0.10868	0.294056216	1.436006	5.58E-06	hypermethylated
cg05977002	0.16643	0.450297297	1.435962	1.18E-05	hypermethylated
cg15662768	0.13526	0.365952432	1.435921	0.000129	hypermethylated
cg12614105	0.12402	0.335447027	1.435512	1.92E-05	hypermethylated
cg24884142	0.14319	0.387295676	1.435505	3.69E-06	hypermethylated
cg26752526	0.09563	0.258639459	1.435407	0.000155	hypermethylated
cg05756220	0.1303	0.352328108	1.435082	2.71E-05	hypermethylated
cg13787438	0.09642	0.2607	1.434986	4.94E-05	hypermethylated
cg00070680	0.10588	0.286275135	1.434972	0.000135	hypermethylated
cg17500587	0.10816	0.292416757	1.434859	5.19E-05	hypermethylated
cg26055770	0.10694	0.288944324	1.43399	0.000268	hypermethylated
cg18044383	0.11094	0.299751892	1.433989	6.54E-05	hypermethylated
cg08305436	0.20579	0.556028108	1.433985	5.28E-06	hypermethylated
cg21230493	0.12548	0.339007568	1.43386	1.73E-05	hypermethylated
cg12016437	0.10657	0.287918378	1.433859	8.63E-05	hypermethylated
cg23967169	0.10628	0.287125946	1.433814	5.45E-05	hypermethylated
cg04596004	0.12009	0.324430811	1.433795	5.73E-06	hypermethylated
cg22650617	0.14588	0.393978919	1.433336	5.19E-05	hypermethylated
cg08720517	0.1286	0.34731027	1.433334	9.05E-05	hypermethylated
cg26963356	0.12694	0.342793514	1.433193	0.000178	hypermethylated
cg01082512	0.09983	0.269538919	1.432948	5.58E-05	hypermethylated
cg01075271	0.12341	0.333180541	1.432845	7.84E-05	hypermethylated
cg07906632	0.1176	0.317450811	1.432645	0.000154	hypermethylated
cg13207326	0.14061	0.379561081	1.432633	6.57E-06	hypermethylated
cg11427855	0.11587	0.31277027	1.432596	3.76E-05	hypermethylated
cg05892568	0.12531	0.338238919	1.432541	1.35E-05	hypermethylated
cg05215925	0.11379	0.307138378	1.432515	2.16E-06	hypermethylated
cg06495961	0.16884	0.455675135	1.432349	2.39E-05	hypermethylated
cg23154526	0.11376	0.307014054	1.432311	1.31E-05	hypermethylated
cg18295183	0.15082	0.406968649	1.43209	1.99E-05	hypermethylated
cg01498609	0.1358	0.366416216	1.432	6.31E-05	hypermethylated
cg19349861	0.08379	0.226037838	1.431714	6.39E-06	hypermethylated
cg03480337	0.13827	0.372869189	1.431181	5.32E-05	hypermethylated
cg18341512	0.09807	0.264462162	1.431178	1.26E-06	hypermethylated
cg03401738	0.10984	0.296167568	1.43101	6.15E-05	hypermethylated
cg11148130	0.11104	0.299393514	1.430963	0.0003	hypermethylated
cg26517663	0.145	0.390934054	1.430872	1.60E-05	hypermethylated
cg14866863	0.19016	0.512625946	1.430693	1.06E-05	hypermethylated
cg27291304	0.16238	0.437635135	1.430355	9.57E-06	hypermethylated
cg16389399	0.13323	0.359021622	1.430152	7.72E-06	hypermethylated
cg26405376	0.11397	0.307086486	1.429991	2.57E-05	hypermethylated
cg01283289	0.09777	0.263414595	1.429872	1.20E-05	hypermethylated

cg18430128	0.1349	0.363423784	1.429762	6.78E-05	hypermethylated
cg04059773	0.14277	0.384498378	1.429285	3.44E-05	hypermethylated
cg14304469	0.12468	0.335754595	1.429177	9.05E-05	hypermethylated
cg02232208	0.14027	0.377724865	1.429129	1.21E-05	hypermethylated
cg16242629	0.17824	0.479964324	1.429106	1.58E-05	hypermethylated
cg24085946	0.12228	0.329248108	1.428987	0.00134	hypermethylated
cg09469566	0.16515	0.444670811	1.428961	8.83E-06	hypermethylated
cg10243939	0.12067	0.324879459	1.428837	1.01E-05	hypermethylated
cg26492514	0.12468	0.335652973	1.42874	2.71E-05	hypermethylated
cg18173058	0.1604	0.431814054	1.428736	1.50E-05	hypermethylated
cg23487201	0.14149	0.380779459	1.428256	6.75E-06	hypermethylated
cg01002253	0.14755	0.39706	1.428153	1.35E-05	hypermethylated
cg00497232	0.09807	0.263904865	1.428134	0.000313	hypermethylated
cg14188639	0.13699	0.368596757	1.427973	2.27E-05	hypermethylated
cg03181478	0.09856	0.265178919	1.427892	0.000168	hypermethylated
cg10119075	0.14477	0.389477297	1.427777	8.37E-06	hypermethylated
cg18482268	0.11367	0.305772973	1.427609	7.38E-05	hypermethylated
cg26082257	0.11224	0.301888649	1.42743	0.000117	hypermethylated
cg20928234	0.10957	0.294668108	1.427238	1.64E-05	hypermethylated
cg16265906	0.09826	0.264246486	1.427208	2.21E-05	hypermethylated
cg05184456	0.15504	0.416906486	1.427083	1.71E-05	hypermethylated
cg18774195	0.12412	0.333684324	1.426748	1.01E-05	hypermethylated
cg26118906	0.12125	0.325895676	1.426425	2.45E-05	hypermethylated
cg02011374	0.12162	0.326768108	1.425887	1.46E-05	hypermethylated
cg13644317	0.15233	0.409238919	1.425743	4.48E-06	hypermethylated
cg25102370	0.12239	0.328802162	1.425734	1.71E-05	hypermethylated
cg00109302	0.12888	0.346152432	1.425379	2.21E-05	hypermethylated
cg21242918	0.1588	0.426448108	1.425159	4.36E-05	hypermethylated
cg22428147	0.14144	0.379825405	1.425146	4.05E-05	hypermethylated
cg07936950	0.16938	0.454834595	1.425078	4.94E-05	hypermethylated
cg10683251	0.08402	0.225617838	1.425076	1.62E-05	hypermethylated
cg27638196	0.13784	0.370108108	1.424952	6.95E-05	hypermethylated
cg01800521	0.1308	0.351195135	1.42491	0.000112	hypermethylated
cg01181415	0.15386	0.413046486	1.424686	3.90E-05	hypermethylated
cg00529958	0.14857	0.398778919	1.424446	3.54E-06	hypermethylated
cg08534653	0.16642	0.446654054	1.424329	2.32E-05	hypermethylated
cg15199678	0.12212	0.327755676	1.424321	7.32E-06	hypermethylated
cg00922781	0.10703	0.287213514	1.424108	1.75E-05	hypermethylated
cg17525102	0.14111	0.378570811	1.423743	8.60E-06	hypermethylated
cg03462380	0.16109	0.432141622	1.423637	3.57E-05	hypermethylated
cg23234640	0.12168	0.326403243	1.423563	1.25E-05	hypermethylated
cg11563844	0.13778	0.369568108	1.423474	6.46E-05	hypermethylated
cg21590264	0.1298	0.34805027	1.423005	1.67E-06	hypermethylated
cg22534145	0.15513	0.415872432	1.422663	9.16E-05	hypermethylated
cg04996873	0.13166	0.352935676	1.422588	7.84E-05	hypermethylated
cg11286436	0.09769	0.261870811	1.422572	4.47E-05	hypermethylated
cg10422777	0.13802	0.369949189	1.42245	5.28E-06	hypermethylated
cg14972743	0.14951	0.400703784	1.422294	0.000145	hypermethylated
cg14258623	0.11485	0.307802162	1.422253	3.49E-05	hypermethylated
cg10421002	0.09509	0.254815676	1.422088	1.09E-05	hypermethylated
cg16043357	0.15834	0.424301081	1.422063	2.10E-06	hypermethylated
cg01708273	0.16078	0.430819459	1.421995	1.09E-05	hypermethylated
cg07893575	0.09278	0.248608108	1.421988	1.09E-05	hypermethylated
cg02835822	0.11564	0.309837838	1.421873	5.58E-05	hypermethylated
cg16791619	0.13587	0.364032432	1.42184	9.72E-05	hypermethylated
cg04974913	0.07666	0.205355135	1.421575	4.26E-05	hypermethylated
cg27420236	0.10927	0.292666486	1.42136	0.000268	hypermethylated
cg25561762	0.1522	0.40763027	1.421293	2.87E-06	hypermethylated

cg04949206	0.1059	0.283614054	1.421226	2.32E-05	hypermethylated
cg00475820	0.09345	0.250255676	1.421136	8.83E-06	hypermethylated
cg04064644	0.1293	0.34624	1.42105	2.49E-06	hypermethylated
cg23306526	0.08757	0.23446973	1.420893	6.05E-06	hypermethylated
cg19801921	0.15062	0.403265405	1.420816	6.05E-06	hypermethylated
cg17429000	0.14876	0.398235135	1.420634	5.32E-05	hypermethylated
cg10157251	0.10059	0.269280541	1.420623	1.06E-05	hypermethylated
cg26756625	0.11779	0.315294054	1.420481	1.18E-05	hypermethylated
cg19610529	0.16157	0.43248	1.420474	1.84E-05	hypermethylated
cg07703401	0.13169	0.352457838	1.420305	6.46E-05	hypermethylated
cg20585038	0.11596	0.310290811	1.419994	4.05E-05	hypermethylated
cg24642065	0.10354	0.277046486	1.41994	5.79E-05	hypermethylated
cg00279001	0.12311	0.329399459	1.41989	1.28E-05	hypermethylated
cg13481638	0.09566	0.25592	1.419705	3.85E-05	hypermethylated
cg09789636	0.10579	0.283012432	1.419662	1.09E-05	hypermethylated
cg16294566	0.14768	0.395052973	1.419572	2.21E-05	hypermethylated
cg23901857	0.10214	0.273221081	1.419521	2.13E-05	hypermethylated
cg26527487	0.10873	0.290823243	1.419393	1.84E-05	hypermethylated
cg21609640	0.11003	0.294295135	1.419367	9.05E-05	hypermethylated
cg25340966	0.12921	0.345576216	1.419286	0.000365	hypermethylated
cg09451457	0.15597	0.417140541	1.419265	9.07E-06	hypermethylated
cg24161501	0.10639	0.284529189	1.419214	4.47E-05	hypermethylated
cg22162281	0.1294	0.346060541	1.419187	0.000191	hypermethylated
cg10534507	0.12821	0.342874054	1.41917	7.12E-06	hypermethylated
cg13352836	0.12004	0.320998919	1.419053	9.60E-05	hypermethylated
cg01901262	0.17502	0.468003243	1.418999	8.42E-05	hypermethylated
cg13923530	0.13201	0.352989189	1.418977	2.64E-05	hypermethylated
cg03496114	0.12924	0.345507568	1.418665	3.76E-05	hypermethylated
cg01926238	0.12029	0.321514054	1.418365	5.45E-05	hypermethylated
cg09275869	0.11515	0.307683784	1.417934	0.000166	hypermethylated
cg05523911	0.1813	0.484332973	1.41762	6.57E-06	hypermethylated
cg09543255	0.13865	0.370384865	1.417578	4.15E-05	hypermethylated
cg22286936	0.15525	0.414654054	1.417315	2.15E-05	hypermethylated
cg05589784	0.19725	0.526634595	1.416777	9.82E-06	hypermethylated
cg26292150	0.14666	0.391491892	1.416507	0.000229	hypermethylated
cg16359550	0.13664	0.364722162	1.416418	4.15E-05	hypermethylated
cg09159022	0.14969	0.399484865	1.416163	1.84E-05	hypermethylated
cg02167020	0.10003	0.266927027	1.416013	0.000296	hypermethylated
cg03531247	0.15145	0.404100541	1.415873	1.50E-05	hypermethylated
cg16851417	0.15687	0.418516757	1.415716	9.57E-06	hypermethylated
cg15775921	0.13314	0.355192432	1.415657	1.75E-05	hypermethylated
cg05943996	0.11543	0.30791027	1.415492	7.75E-05	hypermethylated
cg03182917	0.10207	0.272234054	1.415289	1.99E-05	hypermethylated
cg13865810	0.11166	0.29770973	1.414794	3.49E-05	hypermethylated
cg08273957	0.17103	0.455925946	1.41455	1.46E-05	hypermethylated
cg00546005	0.12219	0.32572	1.414506	2.64E-05	hypermethylated
cg18521914	0.0969	0.258263784	1.414277	9.96E-06	hypermethylated
cg14123543	0.13686	0.364678378	1.413924	1.84E-05	hypermethylated
cg07414571	0.08619	0.229657297	1.41389	2.45E-05	hypermethylated
cg10806639	0.13987	0.372614054	1.413596	6.15E-05	hypermethylated
cg07268058	0.12706	0.338482162	1.41357	3.32E-05	hypermethylated
cg02446647	0.14505	0.386402703	1.413555	2.78E-05	hypermethylated
cg24838010	0.08783	0.233969189	1.413533	0.000176	hypermethylated
cg11849281	0.10595	0.282150811	1.413083	2.15E-05	hypermethylated
cg10521851	0.10891	0.289958919	1.412712	0.000403	hypermethylated
cg23279117	0.09633	0.256430811	1.412513	2.45E-05	hypermethylated
cg00626856	0.16094	0.428383784	1.412381	2.49E-06	hypermethylated
cg21591742	0.14367	0.382381081	1.412252	6.15E-05	hypermethylated

cg13260012	0.10615	0.282419459	1.411735	2.36E-06	hypermethylated
cg02469871	0.12321	0.327756216	1.411504	2.92E-05	hypermethylated
cg13502741	0.15521	0.412839459	1.411359	1.99E-05	hypermethylated
cg07411620	0.14506	0.38566	1.41068	2.64E-05	hypermethylated
cg24089118	0.10738	0.285458378	1.410555	0.000303	hypermethylated
cg10127275	0.12504	0.332387027	1.410474	2.57E-05	hypermethylated
cg12433277	0.11736	0.311927027	1.410268	6.54E-05	hypermethylated
cg01396176	0.1196	0.317838378	1.410076	6.39E-06	hypermethylated
cg20434529	0.10433	0.277225405	1.409905	0.000138	hypermethylated
cg02423064	0.13818	0.367144324	1.409798	9.82E-06	hypermethylated
cg06829299	0.14535	0.386005405	1.40909	9.57E-06	hypermethylated
cg09233651	0.16428	0.436241622	1.408971	1.12E-05	hypermethylated
cg05571970	0.13192	0.35031027	1.40897	1.02E-05	hypermethylated
cg17941572	0.17607	0.467527027	1.408901	2.42E-06	hypermethylated
cg04261138	0.11782	0.312727568	1.408322	9.07E-06	hypermethylated
cg22848150	0.07508	0.199213514	1.407815	1.35E-05	hypermethylated
cg11117878	0.10783	0.286021081	1.407363	1.20E-05	hypermethylated
cg07811110	0.15758	0.417902162	1.407081	8.94E-05	hypermethylated
cg25938646	0.13186	0.349501622	1.406292	3.76E-05	hypermethylated
cg14570291	0.09458	0.250651351	1.406075	9.95E-05	hypermethylated
cg25574024	0.14319	0.379464324	1.406034	8.84E-05	hypermethylated
cg06511917	0.11362	0.301043784	1.405757	6.39E-06	hypermethylated
cg18301583	0.15325	0.405884324	1.405182	9.05E-05	hypermethylated
cg13610307	0.16589	0.439345946	1.40513	0.000135	hypermethylated
cg03696599	0.14208	0.376195135	1.404778	2.04E-05	hypermethylated
cg22730047	0.13907	0.368151351	1.404488	3.39E-06	hypermethylated
cg09388605	0.14788	0.391386486	1.404167	1.66E-05	hypermethylated
cg00174428	0.13703	0.362619459	1.403965	6.54E-05	hypermethylated
cg11312408	0.12793	0.338484324	1.403734	1.28E-05	hypermethylated
cg26727693	0.1589	0.420397297	1.403634	3.57E-05	hypermethylated
cg25988034	0.11701	0.309560541	1.40359	4.05E-05	hypermethylated
cg23003783	0.15439	0.408441622	1.403551	2.71E-05	hypermethylated
cg17713376	0.10919	0.288858378	1.403522	4.70E-05	hypermethylated
cg18507138	0.12148	0.321352432	1.403438	6.01E-05	hypermethylated
cg00326719	0.09669	0.255676216	1.402879	5.72E-05	hypermethylated
cg21806015	0.094	0.248526486	1.402667	5.86E-05	hypermethylated
cg11418477	0.11272	0.297976757	1.402456	1.75E-05	hypermethylated
cg09896622	0.14199	0.375325405	1.402353	4.94E-05	hypermethylated
cg06462347	0.12106	0.319994595	1.402325	0.000138	hypermethylated
cg10443187	0.09057	0.239398919	1.402311	3.07E-05	hypermethylated
cg27242132	0.15466	0.408669189	1.401833	6.62E-05	hypermethylated
cg18824585	0.12998	0.343392973	1.401571	5.38E-05	hypermethylated
cg24456365	0.13971	0.369090811	1.401541	2.48E-05	hypermethylated
cg05455720	0.1437	0.379605946	1.401443	9.31E-06	hypermethylated
cg01765111	0.13573	0.358526486	1.40134	7.12E-05	hypermethylated
cg09294156	0.10894	0.287737838	1.401221	9.69E-06	hypermethylated
cg07312654	0.11731	0.309759459	1.400822	4.26E-05	hypermethylated
cg11438428	0.13603	0.359124324	1.400559	6.22E-06	hypermethylated
cg20019985	0.16229	0.428365946	1.40027	1.38E-05	hypermethylated
cg25717239	0.11397	0.300792432	1.400114	7.20E-05	hypermethylated
cg04721934	0.07647	0.201755676	1.399643	1.09E-05	hypermethylated
cg13208922	0.18304	0.482923243	1.399635	5.28E-06	hypermethylated
cg05093169	0.14361	0.37884	1.399432	3.12E-06	hypermethylated
cg08065657	0.15741	0.415236216	1.399405	2.79E-06	hypermethylated
cg06354392	0.1262	0.332823784	1.399047	9.72E-05	hypermethylated
cg12454814	0.10677	0.281571351	1.398994	4.26E-05	hypermethylated
cg00982919	0.10039	0.264735135	1.398934	3.27E-05	hypermethylated
cg03250019	0.15055	0.396940541	1.39868	1.89E-05	hypermethylated

cg09330153	0.11083	0.292155676	1.398389	6.15E-05	hypermethylated
cg25259711	0.11011	0.290198919	1.398097	9.82E-06	hypermethylated
cg05128922	0.11625	0.306375676	1.398071	8.60E-06	hypermethylated
cg05890785	0.1153	0.303737838	1.397434	8.32E-05	hypermethylated
cg17555825	0.1547	0.407381081	1.396906	2.95E-06	hypermethylated
cg20892990	0.11501	0.302811892	1.396663	4.70E-05	hypermethylated
cg08587845	0.12461	0.328069189	1.39658	6.05E-06	hypermethylated
cg19787532	0.10343	0.272275135	1.396411	1.62E-05	hypermethylated
cg09127607	0.1642	0.431991892	1.39555	5.89E-06	hypermethylated
cg01181227	0.10888	0.286331351	1.394947	3.85E-05	hypermethylated
cg02084669	0.08821	0.231949189	1.394795	1.73E-05	hypermethylated
cg05513806	0.16689	0.438805405	1.394684	2.51E-05	hypermethylated
cg10634619	0.1176	0.309098919	1.394181	0.000117	hypermethylated
cg25459450	0.07551	0.198448649	1.394026	2.21E-05	hypermethylated
cg25622154	0.14749	0.38761027	1.39399	8.52E-05	hypermethylated
cg14431006	0.11035	0.289946486	1.3937	3.53E-05	hypermethylated
cg21900624	0.12676	0.333058919	1.393678	6.62E-05	hypermethylated
cg06624154	0.12179	0.319978919	1.393581	2.89E-05	hypermethylated
cg06000994	0.14427	0.378781622	1.392595	5.14E-06	hypermethylated
cg15982972	0.09061	0.237884865	1.392521	7.42E-06	hypermethylated
cg13300273	0.19742	0.518285405	1.392479	1.99E-05	hypermethylated
cg10149225	0.1162	0.305037297	1.392376	1.99E-05	hypermethylated
cg24830738	0.09082	0.238398919	1.392296	0.000191	hypermethylated
cg17713539	0.12224	0.320866486	1.392257	7.32E-06	hypermethylated
cg26953640	0.1375	0.360861081	1.392012	6.86E-05	hypermethylated
cg03874199	0.12842	0.337026486	1.391992	7.12E-06	hypermethylated
cg08652487	0.11393	0.298977297	1.391888	0.000189	hypermethylated
cg07777008	0.13574	0.356200541	1.391844	2.74E-05	hypermethylated
cg18274480	0.17837	0.468054595	1.391804	2.10E-05	hypermethylated
cg06329574	0.13491	0.353992973	1.391723	6.31E-05	hypermethylated
cg04651603	0.11239	0.294901622	1.39172	9.57E-06	hypermethylated
cg16294013	0.1311	0.343982162	1.391666	3.57E-05	hypermethylated
cg18008019	0.10642	0.279211892	1.391591	5.32E-05	hypermethylated
cg10761315	0.14017	0.367721622	1.391436	1.99E-05	hypermethylated
cg00614182	0.14165	0.37152973	1.391147	3.04E-05	hypermethylated
cg00196414	0.10117	0.265305405	1.390873	7.52E-06	hypermethylated
cg17696160	0.09798	0.256931892	1.390827	2.15E-05	hypermethylated
cg26309457	0.12103	0.317362703	1.390768	5.25E-05	hypermethylated
cg08811309	0.1201	0.314906486	1.390687	1.46E-05	hypermethylated
cg03287574	0.17327	0.454257838	1.39049	5.00E-06	hypermethylated
cg05825073	0.12607	0.330501622	1.390432	0.000613	hypermethylated
cg08411068	0.11465	0.300427027	1.389778	2.79E-06	hypermethylated
cg20404387	0.11066	0.289931351	1.389578	5.65E-05	hypermethylated
cg08683938	0.11474	0.300533514	1.389157	0.000104	hypermethylated
cg06936155	0.1141	0.298854054	1.389142	1.71E-05	hypermethylated
cg24989962	0.13383	0.350525946	1.38912	0.000135	hypermethylated
cg06749053	0.13846	0.362643784	1.389084	4.15E-05	hypermethylated
cg16399049	0.13264	0.347383784	1.389014	2.57E-05	hypermethylated
cg04130427	0.09916	0.259674054	1.388872	4.47E-05	hypermethylated
cg15311814	0.15448	0.404464865	1.388594	7.12E-06	hypermethylated
cg18454863	0.11815	0.30922973	1.388059	2.57E-05	hypermethylated
cg26296364	0.12746	0.333582162	1.387998	1.58E-05	hypermethylated
cg15910662	0.11016	0.288222703	1.387584	2.30E-05	hypermethylated
cg18799217	0.1392	0.364174595	1.387471	3.49E-06	hypermethylated
cg08455099	0.13976	0.365597297	1.387304	1.50E-05	hypermethylated
cg11718030	0.12627	0.330296757	1.387251	5.06E-05	hypermethylated
cg05306472	0.12155	0.317936216	1.387187	9.27E-05	hypermethylated
cg22586653	0.11746	0.307228649	1.387143	4.15E-05	hypermethylated

cg18839750	0.11622	0.303905946	1.386767	3.32E-05	hypermethylated
cg05785155	0.12709	0.332180541	1.386117	2.23E-06	hypermethylated
cg24975834	0.11889	0.310702703	1.385907	2.15E-05	hypermethylated
cg05467160	0.14877	0.388781622	1.385876	2.05E-06	hypermethylated
cg21215550	0.18525	0.484081622	1.385777	0.000145	hypermethylated
cg24911113	0.20378	0.532365946	1.385406	9.07E-06	hypermethylated
cg17653824	0.11082	0.289411351	1.384903	1.42E-05	hypermethylated
cg23549358	0.09964	0.260202703	1.384839	2.64E-05	hypermethylated
cg13677149	0.15969	0.41699027	1.38474	6.95E-05	hypermethylated
cg16633951	0.13192	0.344422703	1.384517	0.000274	hypermethylated
cg02639634	0.14085	0.367721622	1.384454	1.96E-06	hypermethylated
cg15447540	0.11355	0.296380541	1.384123	2.64E-05	hypermethylated
cg14579864	0.11923	0.311143784	1.383834	2.85E-05	hypermethylated
cg02961004	0.19126	0.499055135	1.383664	1.66E-05	hypermethylated
cg08258526	0.12721	0.331857838	1.383353	0.000155	hypermethylated
cg09363735	0.10907	0.284528649	1.38332	0.000165	hypermethylated
cg13338137	0.10435	0.272194595	1.383208	4.36E-05	hypermethylated
cg27131891	0.17309	0.451487568	1.383164	7.65E-05	hypermethylated
cg01281157	0.16251	0.423869189	1.383091	5.32E-05	hypermethylated
cg01997272	0.15382	0.401203243	1.38309	2.15E-05	hypermethylated
cg08991339	0.10713	0.279391351	1.382925	8.03E-05	hypermethylated
cg15084543	0.2096	0.546283243	1.38201	1.58E-05	hypermethylated
cg26504021	0.17279	0.450309189	1.381896	1.82E-05	hypermethylated
cg23166389	0.11681	0.304354054	1.381587	2.64E-05	hypermethylated
cg01791587	0.10715	0.279177838	1.381553	2.54E-05	hypermethylated
cg17361203	0.10242	0.266835135	1.381451	0.00095	hypermethylated
cg22694818	0.17916	0.466765946	1.381451	5.43E-06	hypermethylated
cg13378934	0.10531	0.274309189	1.381161	6.62E-05	hypermethylated
cg12653796	0.11313	0.294638919	1.380966	3.59E-06	hypermethylated
cg18692678	0.13622	0.354728108	1.380775	3.15E-05	hypermethylated
cg08276295	0.13522	0.352096216	1.380661	0.000132	hypermethylated
cg25993718	0.13609	0.354296216	1.380395	9.57E-06	hypermethylated
cg17793621	0.11086	0.288601081	1.380338	0.000204	hypermethylated
cg17306747	0.16238	0.422703243	1.380271	6.75E-06	hypermethylated
cg14859460	0.17717	0.461090811	1.379917	1.50E-05	hypermethylated
cg12281565	0.09973	0.259419459	1.379187	3.90E-06	hypermethylated
cg19048251	0.11696	0.304230811	1.379151	1.71E-05	hypermethylated
cg09636202	0.127	0.330290811	1.378908	0.000293	hypermethylated
cg27324426	0.12064	0.313722703	1.378782	4.05E-05	hypermethylated
cg04972745	0.1563	0.406455676	1.37878	3.57E-05	hypermethylated
cg23416788	0.12064	0.313663784	1.378511	4.82E-05	hypermethylated
cg10384919	0.09962	0.258995676	1.378421	0.00035	hypermethylated
cg22370006	0.12752	0.331252432	1.377208	2.32E-05	hypermethylated
cg00423153	0.20612	0.535395135	1.37712	7.93E-05	hypermethylated
cg01657574	0.14397	0.373913514	1.376936	6.93E-06	hypermethylated
cg13564533	0.10884	0.282672973	1.376925	1.02E-05	hypermethylated
cg19884262	0.14723	0.38229027	1.376597	1.73E-05	hypermethylated
cg00571033	0.13003	0.337601622	1.376477	1.28E-05	hypermethylated
cg26485937	0.18179	0.471818919	1.37596	2.42E-06	hypermethylated
cg20023231	0.10583	0.274471892	1.37491	5.32E-05	hypermethylated
cg18791205	0.093	0.241191892	1.374879	6.93E-06	hypermethylated
cg10000952	0.11751	0.304748649	1.374836	6.01E-05	hypermethylated
cg19153763	0.10023	0.259796757	1.374069	4.60E-06	hypermethylated
cg16391955	0.15341	0.397611892	1.373968	2.51E-05	hypermethylated
cg12744722	0.1052	0.272635135	1.373837	8.13E-05	hypermethylated
cg03625109	0.16206	0.419992432	1.373835	3.32E-05	hypermethylated
cg11052202	0.14461	0.374690811	1.373533	4.36E-05	hypermethylated
cg11125104	0.12241	0.317160541	1.373492	1.62E-05	hypermethylated

cg13791131	0.138	0.357541622	1.373443	0.000172	hypermethylated
cg18365865	0.14369	0.372215676	1.373179	9.57E-06	hypermethylated
cg02165355	0.13745	0.355928649	1.372681	2.57E-05	hypermethylated
cg03757784	0.14925	0.386472973	1.372637	1.50E-05	hypermethylated
cg12259537	0.12116	0.31372	1.372564	1.11E-05	hypermethylated
cg13066658	0.09985	0.258536216	1.372532	8.03E-05	hypermethylated
cg00736523	0.12612	0.326435135	1.371999	3.12E-06	hypermethylated
cg12338097	0.11661	0.301803784	1.371919	2.92E-05	hypermethylated
cg18875371	0.13055	0.337755135	1.371375	3.07E-05	hypermethylated
cg22685409	0.1287	0.332896757	1.371063	2.71E-05	hypermethylated
cg06010032	0.17552	0.453912973	1.37078	2.51E-05	hypermethylated
cg04620418	0.10562	0.273084865	1.370466	4.05E-05	hypermethylated
cg15805568	0.10211	0.264005405	1.370443	7.52E-06	hypermethylated
cg12304520	0.21909	0.566321622	1.370098	9.82E-06	hypermethylated
cg12851526	0.095	0.245420541	1.369257	6.05E-06	hypermethylated
cg17410650	0.12201	0.315182703	1.369189	2.32E-05	hypermethylated
cg18115064	0.103	0.266045405	1.369028	0.000124	hypermethylated
cg09147777	0.1344	0.347137297	1.368973	4.15E-05	hypermethylated
cg05229355	0.09741	0.251573514	1.368838	2.71E-05	hypermethylated
cg04323427	0.12232	0.315898919	1.368803	5.32E-05	hypermethylated
cg23676439	0.10363	0.267604865	1.368663	0.00012	hypermethylated
cg01009664	0.15335	0.395887568	1.368263	7.12E-05	hypermethylated
cg07317062	0.18244	0.470957297	1.368174	1.18E-05	hypermethylated
cg19814981	0.20962	0.541101622	1.368123	1.99E-06	hypermethylated
cg21375294	0.10599	0.273564865	1.367955	1.58E-05	hypermethylated
cg13209481	0.16274	0.419994054	1.3678	1.12E-05	hypermethylated
cg14418226	0.12609	0.325302162	1.367327	0.000822	hypermethylated
cg09230996	0.10888	0.280847568	1.367048	0.000109	hypermethylated
cg25137711	0.1376	0.354911892	1.36698	1.92E-05	hypermethylated
cg12325536	0.16008	0.412817297	1.36671	8.15E-06	hypermethylated
cg09199698	0.0938	0.241884865	1.366661	1.42E-05	hypermethylated
cg14547644	0.11343	0.292489189	1.366581	4.73E-06	hypermethylated
cg16553435	0.12373	0.319024865	1.366474	0.000109	hypermethylated
cg12164282	0.18051	0.46538973	1.366361	3.00E-05	hypermethylated
cg02347074	0.15774	0.406598378	1.366056	1.06E-05	hypermethylated
cg15191648	0.13899	0.358257838	1.366017	7.12E-06	hypermethylated
cg11615755	0.12602	0.324754595	1.365697	2.21E-05	hypermethylated
cg09698471	0.14962	0.385519459	1.365501	1.80E-05	hypermethylated
cg10824063	0.13381	0.34464973	1.364945	4.36E-05	hypermethylated
cg10963061	0.16407	0.422586486	1.364935	0.000435	hypermethylated
cg27578811	0.20174	0.519535676	1.364726	1.54E-05	hypermethylated
cg21053529	0.13794	0.355209189	1.364628	6.95E-05	hypermethylated
cg03940684	0.13529	0.348223243	1.363957	5.19E-05	hypermethylated
cg03712816	0.12051	0.310128108	1.363711	4.60E-06	hypermethylated
cg09338032	0.17442	0.448817838	1.363565	1.11E-05	hypermethylated
cg25146557	0.14297	0.367877297	1.363512	7.52E-06	hypermethylated
cg00090261	0.15704	0.404078919	1.363505	4.15E-05	hypermethylated
cg15339231	0.12886	0.33142	1.362856	1.04E-05	hypermethylated
cg01162672	0.10487	0.269652432	1.362499	0.000137	hypermethylated
cg09825093	0.1055	0.271222703	1.362235	2.64E-05	hypermethylated
cg01382110	0.11797	0.303257838	1.362125	8.42E-05	hypermethylated
cg16766249	0.12796	0.328904865	1.361977	0.000117	hypermethylated
cg12782992	0.11186	0.287492973	1.361832	0.000148	hypermethylated
cg25825506	0.13831	0.355469189	1.361819	5.32E-05	hypermethylated
cg23035602	0.10492	0.269629189	1.361687	0.00012	hypermethylated
cg11530960	0.10512	0.270104865	1.361482	1.15E-05	hypermethylated
cg24438644	0.11183	0.287329189	1.361397	2.78E-05	hypermethylated
cg12573849	0.13961	0.358650811	1.361178	7.42E-06	hypermethylated

cg20055426	0.12446	0.319685405	1.360971	2.85E-05	hypermethylated
cg21187352	0.1193	0.306371892	1.36069	1.99E-05	hypermethylated
cg08688773	0.09976	0.256117297	1.360271	3.57E-05	hypermethylated
cg22489321	0.17946	0.460632973	1.359955	7.42E-06	hypermethylated
cg14530304	0.1184	0.303858378	1.35973	8.60E-06	hypermethylated
cg19596110	0.15097	0.387399459	1.35956	1.58E-05	hypermethylated
cg24747764	0.11653	0.299015135	1.359517	5.19E-05	hypermethylated
cg00688962	0.16923	0.434192973	1.359351	2.51E-05	hypermethylated
cg16182986	0.12154	0.311752973	1.358972	8.60E-06	hypermethylated
cg08962087	0.09275	0.23785027	1.358634	3.49E-05	hypermethylated
cg15107670	0.1398	0.358504865	1.358628	6.08E-05	hypermethylated
cg09798888	0.16279	0.417460541	1.358628	0.000342	hypermethylated
cg18007959	0.10924	0.280071892	1.358296	5.58E-05	hypermethylated
cg09907509	0.21899	0.56141027	1.35819	1.08E-05	hypermethylated
cg27362525	0.15697	0.402377297	1.35806	5.32E-05	hypermethylated
cg23597629	0.15199	0.389557297	1.357859	7.52E-06	hypermethylated
cg06973667	0.0939	0.240655676	1.357773	3.79E-06	hypermethylated
cg21300373	0.16496	0.422769189	1.357754	1.28E-05	hypermethylated
cg10851698	0.12954	0.331869189	1.357217	1.50E-05	hypermethylated
cg25903072	0.08693	0.222689189	1.357105	2.07E-05	hypermethylated
cg05842855	0.15059	0.385765946	1.3571	5.00E-06	hypermethylated
cg19459876	0.12046	0.308578378	1.357083	6.23E-05	hypermethylated
cg14757228	0.20046	0.513359459	1.356655	8.04E-06	hypermethylated
cg19355929	0.11257	0.288278378	1.35664	3.95E-05	hypermethylated
cg06987672	0.09673	0.247711351	1.356625	3.15E-05	hypermethylated
cg03004999	0.07651	0.195899459	1.356393	1.28E-05	hypermethylated
cg10367023	0.12121	0.31034	1.356341	1.11E-05	hypermethylated
cg19078576	0.14788	0.378597838	1.356239	0.001288	hypermethylated
cg07005778	0.15842	0.405555676	1.356146	7.12E-06	hypermethylated
cg15165122	0.14479	0.370616757	1.355966	0.000594	hypermethylated
cg13667638	0.13075	0.334634595	1.355776	8.83E-06	hypermethylated
cg17777676	0.16862	0.431520541	1.355654	3.90E-06	hypermethylated
cg16234978	0.21558	0.551544865	1.355255	4.82E-05	hypermethylated
cg06005396	0.09518	0.243426486	1.354756	2.13E-05	hypermethylated
cg18451114	0.1072	0.274161081	1.354719	0.000132	hypermethylated
cg22070406	0.11662	0.298227568	1.354598	2.71E-05	hypermethylated
cg26444528	0.17813	0.455507568	1.354545	1.18E-05	hypermethylated
cg08840847	0.10626	0.27172	1.354522	3.08E-06	hypermethylated
cg15810758	0.10942	0.279735135	1.354185	0.000224	hypermethylated
cg06335867	0.21518	0.550029189	1.353964	4.80E-06	hypermethylated
cg01567634	0.11731	0.299838919	1.353862	1.80E-05	hypermethylated
cg02630214	0.18971	0.484816216	1.353642	1.46E-05	hypermethylated
cg06946708	0.14621	0.373598919	1.353448	7.12E-06	hypermethylated
cg04273811	0.13704	0.350164865	1.353437	0.000117	hypermethylated
cg03997176	0.10948	0.27974	1.353419	0.000361	hypermethylated
cg24304919	0.12124	0.309758919	1.35328	4.15E-05	hypermethylated
cg15638055	0.10581	0.270332973	1.353262	2.15E-05	hypermethylated
cg20640433	0.14273	0.364648649	1.353218	0.000245	hypermethylated
cg00605982	0.10972	0.280310811	1.353201	3.95E-05	hypermethylated
cg19803052	0.17364	0.443528649	1.352928	2.10E-06	hypermethylated
cg22562942	0.16534	0.422327027	1.352925	1.18E-05	hypermethylated
cg07964848	0.1587	0.405342703	1.35284	6.84E-06	hypermethylated
cg21907579	0.13364	0.341315676	1.352755	3.76E-05	hypermethylated
cg02438781	0.16763	0.428124865	1.352751	1.25E-05	hypermethylated
cg16564033	0.13804	0.352490811	1.352499	0.000115	hypermethylated
cg03909500	0.10625	0.27128973	1.352372	1.71E-05	hypermethylated
cg04897683	0.13753	0.351143784	1.352316	2.10E-05	hypermethylated
cg16562275	0.14406	0.367809189	1.352288	2.92E-05	hypermethylated

cg17180678	0.11515	0.293971351	1.352161	0.000224	hypermethylated
cg15377518	0.1469	0.375016757	1.352121	0.0002	hypermethylated
cg07903370	0.11097	0.283262162	1.351968	2.23E-06	hypermethylated
cg26675654	0.17539	0.447652432	1.351813	9.07E-06	hypermethylated
cg19947104	0.14818	0.378198919	1.351795	0.000115	hypermethylated
cg25362630	0.12209	0.311550811	1.351522	2.64E-05	hypermethylated
cg05900347	0.08702	0.22204	1.351401	3.08E-06	hypermethylated
cg21711132	0.16199	0.413327027	1.351379	6.93E-06	hypermethylated
cg11764900	0.13695	0.349419459	1.351311	5.32E-05	hypermethylated
cg18447790	0.09418	0.240237838	1.350971	2.85E-05	hypermethylated
cg00030508	0.12894	0.328815676	1.350579	3.08E-06	hypermethylated
cg01345315	0.14743	0.375925946	1.350418	7.32E-06	hypermethylated
cg18221021	0.1069	0.272512973	1.350063	4.36E-05	hypermethylated
cg01718447	0.14852	0.378552432	1.349836	0.000822	hypermethylated
cg19497501	0.11494	0.292945405	1.349751	3.57E-05	hypermethylated
cg13674174	0.10603	0.270208108	1.349598	7.52E-06	hypermethylated
cg11301556	0.1181	0.300809189	1.34884	1.04E-05	hypermethylated
cg05812269	0.09934	0.253005405	1.348722	1.02E-06	hypermethylated
cg08044097	0.16567	0.421824865	1.348332	1.21E-05	hypermethylated
cg25830182	0.16925	0.430908108	1.348224	0.000117	hypermethylated
cg22950163	0.10723	0.272908108	1.347707	1.25E-05	hypermethylated
cg03840594	0.14286	0.363584324	1.347688	3.59E-06	hypermethylated
cg09834794	0.16558	0.421251892	1.347155	2.13E-05	hypermethylated
cg00312921	0.12993	0.330485405	1.346852	1.54E-05	hypermethylated
cg17011276	0.1349	0.343114595	1.3468	1.87E-05	hypermethylated
cg27405791	0.12749	0.324047027	1.345819	5.38E-05	hypermethylated
cg16113681	0.13134	0.333723784	1.345348	0.000417	hypermethylated
cg19827780	0.10801	0.274415135	1.345195	0.001751	hypermethylated
cg07489048	0.21119	0.536243784	1.344347	1.41E-06	hypermethylated
cg02409815	0.12321	0.312827027	1.344246	1.71E-05	hypermethylated
cg01442601	0.14754	0.374541081	1.344018	4.05E-05	hypermethylated
cg22418909	0.14444	0.366657838	1.343964	1.31E-05	hypermethylated
cg23284720	0.12445	0.31588	1.34381	2.36E-06	hypermethylated
cg06635832	0.13076	0.331850811	1.343614	1.58E-05	hypermethylated
cg06672560	0.1291	0.327627568	1.343568	2.32E-05	hypermethylated
cg20661138	0.19052	0.48347027	1.343485	2.27E-05	hypermethylated
cg19822343	0.13343	0.338551892	1.343294	1.18E-05	hypermethylated
cg25839023	0.09541	0.24202973	1.342972	3.07E-05	hypermethylated
cg21016855	0.12837	0.325568108	1.342651	8.84E-05	hypermethylated
cg18836626	0.15334	0.388814595	1.342348	2.78E-05	hypermethylated
cg18309817	0.11953	0.303081622	1.342334	8.83E-06	hypermethylated
cg24196693	0.1421	0.360303243	1.342305	2.85E-05	hypermethylated
cg25399352	0.14124	0.358017838	1.341883	6.95E-05	hypermethylated
cg10345920	0.09596	0.243164865	1.34143	4.26E-05	hypermethylated
cg12377139	0.18135	0.459531351	1.341387	8.26E-06	hypermethylated
cg15727287	0.14324	0.362875676	1.341041	0.000232	hypermethylated
cg19064258	0.15258	0.38652973	1.341014	1.71E-05	hypermethylated
cg05956679	0.11036	0.279529189	1.340782	0.000142	hypermethylated
cg14968543	0.12141	0.307505405	1.340725	2.10E-05	hypermethylated
cg25033990	0.14048	0.355765405	1.340561	8.60E-06	hypermethylated
cg02367144	0.12363	0.313068108	1.340448	3.07E-05	hypermethylated
cg17295225	0.13621	0.344863784	1.340194	3.26E-06	hypermethylated
cg00842299	0.09569	0.24227027	1.340177	1.71E-05	hypermethylated
cg01309744	0.12627	0.31968	1.340117	3.95E-05	hypermethylated
cg04548856	0.13726	0.347489189	1.340057	3.95E-05	hypermethylated
cg16379885	0.09618	0.243469189	1.33993	4.47E-05	hypermethylated
cg04282694	0.12481	0.31592	1.339826	2.45E-05	hypermethylated
cg02990291	0.1256	0.317885405	1.33967	3.15E-05	hypermethylated

cg08857994	0.19119	0.483860541	1.339584	1.82E-06	hypermethylated
cg11213369	0.17268	0.436987027	1.339489	9.82E-06	hypermethylated
cg18781988	0.1631	0.412725946	1.339427	4.70E-05	hypermethylated
cg05036656	0.18964	0.479874595	1.339394	1.51E-06	hypermethylated
cg00073543	0.0995	0.251758919	1.339274	3.85E-05	hypermethylated
cg26444951	0.13894	0.351534054	1.339202	8.84E-05	hypermethylated
cg14457782	0.16166	0.408981081	1.339071	8.22E-05	hypermethylated
cg08526991	0.2116	0.535275676	1.338942	9.66E-07	hypermethylated
cg08141424	0.10714	0.270978378	1.338681	0.000191	hypermethylated
cg11220663	0.16004	0.404756757	1.338623	1.80E-05	hypermethylated
cg10176110	0.16114	0.407509189	1.338518	9.72E-05	hypermethylated
cg13927566	0.13506	0.341525946	1.338395	1.72E-06	hypermethylated
cg27122536	0.13466	0.340457297	1.338152	2.21E-05	hypermethylated
cg12213062	0.18139	0.458565405	1.338033	2.45E-05	hypermethylated
cg07990736	0.10319	0.26082973	1.337805	1.66E-05	hypermethylated
cg24156796	0.10578	0.267372973	1.337787	7.32E-06	hypermethylated
cg05996052	0.13137	0.332045405	1.337745	2.71E-05	hypermethylated
cg19759671	0.17453	0.440998378	1.337298	1.89E-05	hypermethylated
cg02773086	0.16347	0.413032432	1.337229	4.94E-05	hypermethylated
cg21187554	0.10629	0.268557297	1.337224	2.85E-05	hypermethylated
cg04891086	0.12647	0.319534595	1.337177	0.000296	hypermethylated
cg23842170	0.14062	0.355205405	1.336852	2.39E-05	hypermethylated
cg25733708	0.10513	0.265538919	1.336749	0.000435	hypermethylated
cg15540820	0.14443	0.364777297	1.336646	9.82E-06	hypermethylated
cg27315191	0.10321	0.260630811	1.336425	7.72E-06	hypermethylated
cg18497508	0.11858	0.299377297	1.336104	0.000234	hypermethylated
cg19403014	0.13608	0.343511351	1.335903	0.000159	hypermethylated
cg22374057	0.16061	0.40539027	1.33575	4.24E-06	hypermethylated
cg11566394	0.18978	0.478743784	1.334926	3.76E-05	hypermethylated
cg24513224	0.12006	0.302802162	1.33462	8.37E-06	hypermethylated
cg24041917	0.11269	0.284207568	1.334585	9.19E-06	hypermethylated
cg04900080	0.13349	0.336594054	1.334278	4.05E-05	hypermethylated
cg02621287	0.22543	0.568403784	1.334237	3.95E-05	hypermethylated
cg13073186	0.10673	0.269095135	1.334151	5.43E-06	hypermethylated
cg06951245	0.11299	0.284877297	1.334146	0.000303	hypermethylated
cg19030607	0.11017	0.277750811	1.33406	3.30E-06	hypermethylated
cg08390877	0.14413	0.363356757	1.334016	9.82E-06	hypermethylated
cg07442409	0.11279	0.284294595	1.333748	6.57E-06	hypermethylated
cg02081006	0.23128	0.58294	1.333707	6.66E-06	hypermethylated
cg02739346	0.15604	0.393101622	1.332986	2.23E-06	hypermethylated
cg18279094	0.2301	0.579628108	1.332867	2.64E-06	hypermethylated
cg17473600	0.09973	0.251215135	1.332824	2.02E-06	hypermethylated
cg14659193	0.13717	0.34536973	1.332177	8.83E-06	hypermethylated
cg01610231	0.13538	0.340808108	1.331945	1.62E-05	hypermethylated
cg02712224	0.11703	0.294599459	1.331876	1.94E-05	hypermethylated
cg18653451	0.13062	0.328782162	1.331756	3.32E-05	hypermethylated
cg03111498	0.12176	0.30639027	1.33133	5.72E-05	hypermethylated
cg22974236	0.14502	0.364862162	1.3311	2.15E-05	hypermethylated
cg03694713	0.15291	0.384707027	1.331077	7.12E-05	hypermethylated
cg03355524	0.15275	0.384224324	1.330776	7.65E-05	hypermethylated
cg15448975	0.16609	0.417736757	1.330629	7.52E-06	hypermethylated
cg06482428	0.15292	0.384589189	1.330541	2.04E-05	hypermethylated
cg19656282	0.14983	0.376771351	1.330363	9.27E-05	hypermethylated
cg12215340	0.11576	0.291084865	1.330303	0.000187	hypermethylated
cg10476112	0.12094	0.304107027	1.330288	0.0003	hypermethylated
cg12506930	0.17133	0.430798919	1.330237	5.45E-05	hypermethylated
cg14621053	0.15373	0.38651027	1.330108	2.02E-06	hypermethylated
cg16123202	0.18388	0.462197297	1.329744	1.25E-05	hypermethylated

cg14909464	0.09015	0.22654973	1.329428	2.71E-06	hypermethylated
cg10354244	0.13056	0.328084865	1.329356	1.84E-05	hypermethylated
cg22203219	0.14475	0.363688108	1.329138	1.99E-05	hypermethylated
cg10722226	0.12591	0.316304324	1.32892	5.45E-05	hypermethylated
cg10269365	0.16735	0.420400541	1.328896	8.48E-06	hypermethylated
cg25684640	0.10891	0.273575135	1.328801	3.53E-05	hypermethylated
cg25486757	0.1126	0.282828649	1.328721	0.000229	hypermethylated
cg20475322	0.16934	0.425286486	1.328512	8.03E-05	hypermethylated
cg07376535	0.12768	0.320656216	1.328495	9.07E-06	hypermethylated
cg22875872	0.14285	0.358745405	1.328459	0.000607	hypermethylated
cg07212852	0.12925	0.324488108	1.328001	7.74E-05	hypermethylated
cg08553284	0.1218	0.305747027	1.327824	9.05E-05	hypermethylated
cg18255166	0.12624	0.316847027	1.327617	0.000296	hypermethylated
cg09703679	0.16182	0.406124865	1.327533	4.73E-06	hypermethylated
cg01718322	0.15	0.376443243	1.32747	0.000259	hypermethylated
cg17023770	0.1469	0.368611892	1.327268	2.10E-05	hypermethylated
cg20959866	0.14283	0.358394595	1.32725	2.57E-06	hypermethylated
cg13891768	0.18369	0.460839459	1.326991	1.48E-05	hypermethylated
cg00446413	0.1709	0.428705946	1.326836	1.89E-05	hypermethylated
cg05677041	0.11337	0.284372432	1.326743	8.94E-05	hypermethylated
cg05091238	0.15639	0.392256757	1.32665	3.40E-05	hypermethylated
cg25094569	0.13647	0.342275676	1.326575	0.000126	hypermethylated
cg04178316	0.12695	0.318384324	1.326509	4.42E-06	hypermethylated
cg15634980	0.11912	0.298740541	1.326477	0.000306	hypermethylated
cg18022224	0.09996	0.250683784	1.326446	9.07E-06	hypermethylated
cg14844194	0.14078	0.353048649	1.326425	1.92E-05	hypermethylated
cg03506489	0.12129	0.304168649	1.326411	2.35E-05	hypermethylated
cg24945701	0.17092	0.428491892	1.325947	3.12E-06	hypermethylated
cg19370653	0.11644	0.291877838	1.325778	2.74E-05	hypermethylated
cg25651783	0.12432	0.311442703	1.324908	2.39E-05	hypermethylated
cg26666835	0.15064	0.377316757	1.324671	3.04E-06	hypermethylated
cg10242602	0.19551	0.489407027	1.323792	1.21E-05	hypermethylated
cg25339054	0.22622	0.566277838	1.323784	8.60E-06	hypermethylated
cg10175795	0.14768	0.369637297	1.323636	1.18E-05	hypermethylated
cg01414185	0.15675	0.392270811	1.323385	0.000112	hypermethylated
cg01024444	0.19303	0.482978919	1.323135	6.01E-05	hypermethylated
cg00957516	0.13419	0.335745946	1.323093	1.66E-05	hypermethylated
cg10214421	0.13074	0.327047568	1.3228	3.90E-05	hypermethylated
cg22873539	0.11926	0.298232432	1.322327	3.40E-05	hypermethylated
cg08581953	0.13502	0.337642162	1.322322	6.93E-06	hypermethylated
cg12110911	0.12231	0.305774595	1.321926	8.60E-06	hypermethylated
cg22426570	0.10927	0.273148108	1.321786	0.000166	hypermethylated
cg19509330	0.1105	0.276082162	1.321051	1.62E-05	hypermethylated
cg03356900	0.12576	0.314203243	1.321025	6.62E-05	hypermethylated
cg16041686	0.1179	0.294547568	1.320937	7.52E-06	hypermethylated
cg09854003	0.15183	0.379249189	1.320689	2.95E-06	hypermethylated
cg20429172	0.16579	0.414102703	1.320632	9.31E-06	hypermethylated
cg21451110	0.10982	0.274277297	1.320494	8.84E-05	hypermethylated
cg06659727	0.15233	0.380421081	1.320397	4.58E-05	hypermethylated
cg10709593	0.13394	0.334465946	1.320272	5.21E-06	hypermethylated
cg20156659	0.15042	0.375593514	1.320176	4.36E-05	hypermethylated
cg23638252	0.12026	0.300260541	1.320058	3.12E-06	hypermethylated
cg20575761	0.19118	0.477228108	1.319747	3.59E-06	hypermethylated
cg22762091	0.17842	0.445358919	1.319691	9.57E-06	hypermethylated
cg00192136	0.12754	0.318244324	1.319185	0.000165	hypermethylated
cg15929698	0.14563	0.363368108	1.319124	7.29E-05	hypermethylated
cg06110297	0.14966	0.373405405	1.319054	1.08E-05	hypermethylated
cg16939364	0.14358	0.358227027	1.319019	1.42E-05	hypermethylated

cg11241627	0.12085	0.301479459	1.318842	6.23E-05	hypermethylated
cg15461335	0.10383	0.259010811	1.318789	0.000117	hypermethylated
cg22571664	0.16555	0.412851351	1.318355	7.12E-06	hypermethylated
cg00272971	0.14552	0.362882162	1.318284	2.32E-05	hypermethylated
cg02000318	0.12421	0.309716216	1.318166	4.10E-05	hypermethylated
cg20132590	0.14396	0.358854054	1.317729	1.54E-05	hypermethylated
cg03671700	0.11517	0.287083784	1.317707	9.05E-05	hypermethylated
cg03631561	0.13887	0.346158378	1.317697	6.31E-05	hypermethylated
cg10647513	0.10665	0.265824324	1.317589	0.000237	hypermethylated
cg23400446	0.12819	0.319423784	1.317188	0.001074	hypermethylated
cg08736638	0.12989	0.323542703	1.316666	9.60E-05	hypermethylated
cg02970384	0.12684	0.315772432	1.315875	2.95E-06	hypermethylated
cg02582957	0.1333	0.331834054	1.315785	5.00E-06	hypermethylated
cg16080876	0.11995	0.298554054	1.315559	1.66E-05	hypermethylated
cg26517171	0.10088	0.251081081	1.315513	4.24E-06	hypermethylated
cg15028514	0.11827	0.294355135	1.315474	8.63E-05	hypermethylated
cg25818402	0.12383	0.308168649	1.315359	1.28E-05	hypermethylated
cg11308643	0.1739	0.432709189	1.31514	1.89E-05	hypermethylated
cg01787574	0.14537	0.361660541	1.314907	7.62E-06	hypermethylated
cg26084511	0.1137	0.282808108	1.314591	1.68E-05	hypermethylated
cg08351336	0.11219	0.279044865	1.314553	0.000104	hypermethylated
cg13411015	0.09706	0.241398919	1.31447	5.06E-05	hypermethylated
cg13462129	0.17855	0.444065946	1.314446	1.04E-05	hypermethylated
cg03706436	0.12231	0.304188649	1.314424	5.12E-05	hypermethylated
cg02781618	0.15305	0.380560541	1.314123	9.31E-06	hypermethylated
cg06622528	0.08787	0.218483784	1.314084	4.70E-05	hypermethylated
cg02860602	0.16254	0.403978919	1.313485	3.49E-05	hypermethylated
cg02127215	0.1397	0.347159459	1.313266	1.04E-05	hypermethylated
cg10649903	0.1913	0.475165405	1.312593	2.10E-06	hypermethylated
cg11480800	0.13746	0.341407568	1.312483	0.000193	hypermethylated
cg04560225	0.1817	0.451256216	1.312388	5.14E-06	hypermethylated
cg07428136	0.16394	0.407145405	1.312376	1.66E-05	hypermethylated
cg07990546	0.16172	0.401592973	1.312236	8.63E-05	hypermethylated
cg17694795	0.15202	0.377453514	1.312038	3.85E-05	hypermethylated
cg17632372	0.12358	0.306785405	1.311785	8.73E-05	hypermethylated
cg17299899	0.15785	0.391814595	1.311617	2.79E-06	hypermethylated
cg18082638	0.15008	0.372457838	1.311345	5.28E-06	hypermethylated
cg00847277	0.13208	0.327756757	1.311213	2.92E-05	hypermethylated
cg23893898	0.1707	0.423538378	1.31103	1.04E-05	hypermethylated
cg07033624	0.1337	0.331718919	1.310962	1.84E-05	hypermethylated
cg04670857	0.15521	0.384992432	1.310609	9.72E-05	hypermethylated
cg06187770	0.15027	0.372732973	1.310585	0.000111	hypermethylated
cg10982364	0.1573	0.390133514	1.310449	9.27E-05	hypermethylated
cg25482900	0.14631	0.362818919	1.310221	9.27E-05	hypermethylated
cg01969701	0.25799	0.639610811	1.309879	7.32E-06	hypermethylated
cg13652336	0.13173	0.326464324	1.309341	0.000374	hypermethylated
cg12286573	0.10664	0.264247027	1.309139	5.58E-05	hypermethylated
cg14950072	0.15325	0.379736216	1.309111	4.82E-05	hypermethylated
cg19858749	0.10368	0.256863784	1.308866	2.78E-05	hypermethylated
cg00500705	0.11883	0.294396757	1.308863	8.83E-06	hypermethylated
cg22983282	0.13274	0.328746486	1.308372	0.000256	hypermethylated
cg01093363	0.12992	0.321745946	1.308298	1.01E-06	hypermethylated
cg14290904	0.13951	0.345471351	1.308198	1.50E-05	hypermethylated
cg10721149	0.14124	0.349672432	1.307855	0.000182	hypermethylated
cg23034757	0.1593	0.394255676	1.307385	7.93E-06	hypermethylated
cg01342746	0.12278	0.30384973	1.307282	4.05E-05	hypermethylated
cg17471467	0.09094	0.225048649	1.30725	1.73E-05	hypermethylated
cg01610605	0.14792	0.366004865	1.307046	1.35E-05	hypermethylated

cg09659227	0.12255	0.303205946	1.306928	6.05E-06	hypermethylated
cg22799141	0.14733	0.364494595	1.306846	0.00017	hypermethylated
cg07858908	0.15087	0.37324	1.306798	2.45E-05	hypermethylated
cg14015441	0.17383	0.42995027	1.306493	9.57E-06	hypermethylated
cg08287265	0.15524	0.383961081	1.30646	0.001052	hypermethylated
cg11475550	0.1218	0.301230811	1.306355	8.42E-05	hypermethylated
cg01301138	0.08428	0.20843027	1.306303	1.21E-05	hypermethylated
cg21854408	0.1149	0.284149189	1.30627	0.001188	hypermethylated
cg00043788	0.12059	0.298217297	1.306254	0.000129	hypermethylated
cg00333226	0.15485	0.382842703	1.30588	7.32E-06	hypermethylated
cg13246235	0.19524	0.482692432	1.305856	3.23E-05	hypermethylated
cg23895340	0.13345	0.329875135	1.305621	0.000104	hypermethylated
cg10904109	0.15886	0.392613514	1.305354	9.82E-06	hypermethylated
cg03075534	0.1427	0.352661081	1.305297	0.000626	hypermethylated
cg07871202	0.12063	0.29806973	1.305061	6.30E-06	hypermethylated
cg25201047	0.1714	0.423425946	1.304743	8.22E-05	hypermethylated
cg24676817	0.18294	0.451925405	1.304714	0.000426	hypermethylated
cg06838985	0.135	0.333446486	1.304496	2.10E-05	hypermethylated
cg05139788	0.18047	0.445678378	1.304244	7.52E-06	hypermethylated
cg25062778	0.1233	0.304475135	1.304152	1.25E-05	hypermethylated
cg22828989	0.11884	0.293248108	1.303101	2.75E-06	hypermethylated
cg12690369	0.13451	0.331866486	1.30289	0.000123	hypermethylated
cg23625660	0.16439	0.405574595	1.302845	1.37E-06	hypermethylated
cg27272235	0.16198	0.399558378	1.302591	9.31E-06	hypermethylated
cg02091109	0.1369	0.337680541	1.302537	3.49E-05	hypermethylated
cg07068674	0.1608	0.396582162	1.302352	1.04E-05	hypermethylated
cg14871601	0.12018	0.296358919	1.302149	6.01E-05	hypermethylated
cg14156751	0.21282	0.524771892	1.302057	1.63E-06	hypermethylated
cg11254700	0.22237	0.548254054	1.301882	6.38E-05	hypermethylated
cg20605061	0.13319	0.328354595	1.301769	6.01E-05	hypermethylated
cg09413950	0.16042	0.39542973	1.301567	5.06E-05	hypermethylated
cg26003934	0.15567	0.383682162	1.301421	2.71E-05	hypermethylated
cg18953784	0.12497	0.308001081	1.301354	0.000234	hypermethylated
cg11473001	0.1522	0.375096216	1.301292	1.42E-05	hypermethylated
cg12214908	0.13817	0.34043027	1.300915	1.21E-05	hypermethylated
cg06717565	0.13531	0.333347568	1.300759	1.06E-05	hypermethylated
cg00933210	0.08442	0.207951892	1.300593	8.22E-05	hypermethylated
cg22677715	0.15632	0.385002162	1.300364	1.43E-06	hypermethylated
cg11367354	0.16522	0.406855135	1.300127	2.87E-06	hypermethylated
cg09567439	0.15992	0.393793514	1.300089	9.27E-05	hypermethylated
cg04200224	0.10833	0.266746486	1.300036	0.000403	hypermethylated
cg27142536	0.17382	0.428004324	1.300031	1.39E-05	hypermethylated
cg03313945	0.15951	0.392658378	1.299628	0.000588	hypermethylated
cg27288226	0.22674	0.558105405	1.299499	9.07E-06	hypermethylated
cg19851909	0.12415	0.305563784	1.299389	0.000229	hypermethylated
cg06086731	0.14212	0.349746486	1.2992	4.15E-05	hypermethylated
cg09422450	0.21339	0.52513027	1.299183	7.03E-06	hypermethylated
cg13023623	0.22581	0.555693514	1.29918	3.26E-06	hypermethylated
cg10571987	0.14622	0.359747568	1.298844	7.93E-05	hypermethylated
cg11228052	0.14099	0.346834595	1.298655	8.42E-05	hypermethylated
cg09885735	0.1494	0.367521081	1.298647	1.64E-05	hypermethylated
cg26844246	0.18893	0.4647	1.298448	1.09E-06	hypermethylated
cg05353133	0.14144	0.347849189	1.298272	2.79E-06	hypermethylated
cg01566235	0.12811	0.315034595	1.298127	1.99E-05	hypermethylated
cg19841369	0.18889	0.464470811	1.298042	1.09E-05	hypermethylated
cg22319784	0.15043	0.369880541	1.297967	0.000155	hypermethylated
cg01150683	0.17359	0.426723243	1.297617	1.46E-05	hypermethylated
cg21231400	0.15191	0.373403243	1.297518	1.42E-05	hypermethylated

cg04145134	0.14746	0.362374054	1.297156	2.57E-05	hypermethylated
cg14370314	0.14258	0.350355676	1.297049	0.0006	hypermethylated
cg19678828	0.16449	0.404081081	1.296645	1.01E-06	hypermethylated
cg23746497	0.22634	0.555973514	1.296525	5.73E-06	hypermethylated
cg08448812	0.18392	0.451762703	1.296487	1.99E-06	hypermethylated
cg17453460	0.15877	0.389861622	1.296024	4.26E-05	hypermethylated
cg23091758	0.13313	0.326835676	1.29573	0.001452	hypermethylated
cg01859340	0.10433	0.256117297	1.295651	5.65E-05	hypermethylated
cg02273398	0.14512	0.356233514	1.295577	5.97E-06	hypermethylated
cg02124383	0.14136	0.346994054	1.295537	3.27E-05	hypermethylated
cg01202666	0.13412	0.329165405	1.295288	1.93E-06	hypermethylated
cg03319497	0.13574	0.333098378	1.295102	3.35E-06	hypermethylated
cg04694812	0.12078	0.296348649	1.294914	1.99E-05	hypermethylated
cg21399832	0.1946	0.477444324	1.294821	2.39E-05	hypermethylated
cg19443075	0.13684	0.335702703	1.294694	0.000154	hypermethylated
cg24074033	0.18975	0.465294595	1.294045	1.30E-05	hypermethylated
cg07695835	0.1452	0.356015135	1.293897	0.00024	hypermethylated
cg08934785	0.18025	0.441944865	1.293867	4.12E-06	hypermethylated
cg03205252	0.13485	0.330596757	1.293717	1.39E-05	hypermethylated
cg05221167	0.14429	0.353684324	1.293491	4.15E-05	hypermethylated
cg03478199	0.12636	0.309654595	1.29312	0.000155	hypermethylated
cg16598600	0.14352	0.351644865	1.292867	1.58E-05	hypermethylated
cg01295203	0.17222	0.421955135	1.292837	3.30E-06	hypermethylated
cg24761366	0.10228	0.250527568	1.292445	1.84E-05	hypermethylated
cg03754403	0.13816	0.338402703	1.292401	3.57E-05	hypermethylated
cg13658899	0.13318	0.326196757	1.292365	3.32E-05	hypermethylated
cg05701791	0.11256	0.275691351	1.29236	1.62E-05	hypermethylated
cg00891278	0.12882	0.31547027	1.292147	2.10E-05	hypermethylated
cg24346905	0.16393	0.401370811	1.291856	0.000172	hypermethylated
cg24881420	0.14197	0.347587027	1.291788	3.49E-06	hypermethylated
cg10945079	0.12913	0.316034595	1.291258	6.05E-06	hypermethylated
cg10373086	0.12602	0.308308108	1.29072	4.24E-06	hypermethylated
cg08046044	0.1138	0.278408649	1.290703	0.00054	hypermethylated
cg05676400	0.12184	0.298029189	1.290466	2.10E-05	hypermethylated
cg12356951	0.13576	0.332058919	1.290381	5.58E-06	hypermethylated
cg08668316	0.17477	0.427425405	1.290215	7.93E-06	hypermethylated
cg03942855	0.14339	0.350507568	1.289501	0.000245	hypermethylated
cg11510523	0.14155	0.346004324	1.289478	0.000248	hypermethylated
cg26089160	0.13166	0.321784865	1.289279	0.000224	hypermethylated
cg19620724	0.15119	0.369447027	1.289005	2.64E-06	hypermethylated
cg03263618	0.12511	0.305707568	1.288955	1.99E-05	hypermethylated
cg03815358	0.11933	0.291523243	1.288654	5.38E-05	hypermethylated
cg14459130	0.14694	0.358952432	1.288565	3.04E-05	hypermethylated
cg18653282	0.13763	0.336201081	1.288529	0.0002	hypermethylated
cg02186542	0.12606	0.307926486	1.288475	7.52E-06	hypermethylated
cg18030960	0.14132	0.345191892	1.288433	1.35E-05	hypermethylated
cg09003833	0.14124	0.344988649	1.2884	1.94E-05	hypermethylated
cg06607919	0.14451	0.352955135	1.288315	9.31E-06	hypermethylated
cg22600043	0.12877	0.314503784	1.288281	9.60E-05	hypermethylated
cg01730970	0.18888	0.461236757	1.288038	2.10E-05	hypermethylated
cg25112312	0.12358	0.301756216	1.287938	4.36E-05	hypermethylated
cg04991805	0.1585	0.386929189	1.287587	2.54E-05	hypermethylated
cg27605748	0.11422	0.278815676	1.287496	4.47E-05	hypermethylated
cg17514558	0.20627	0.503476216	1.28739	9.31E-06	hypermethylated
cg21188820	0.10664	0.260246486	1.28713	2.71E-05	hypermethylated
cg19125370	0.19857	0.484591892	1.287123	2.71E-06	hypermethylated
cg07160746	0.1845	0.450245946	1.287092	9.31E-06	hypermethylated
cg17508300	0.1192	0.290877838	1.287029	0.000495	hypermethylated

cg17781313	0.12517	0.305401081	1.286816	6.95E-05	hypermethylated
cg23346622	0.12893	0.314562703	1.28676	3.85E-05	hypermethylated
cg19962990	0.15505	0.378289189	1.286756	1.26E-05	hypermethylated
cg00393837	0.17067	0.416396757	1.286749	4.60E-06	hypermethylated
cg20629239	0.11444	0.279152973	1.286465	3.44E-05	hypermethylated
cg17007640	0.18624	0.454276757	1.286409	5.00E-06	hypermethylated
cg17861230	0.19306	0.470876216	1.286299	3.85E-05	hypermethylated
cg02885007	0.14052	0.342727568	1.286287	2.71E-06	hypermethylated
cg23708361	0.16033	0.391023784	1.286212	0.000195	hypermethylated
cg25234732	0.15446	0.376701081	1.286187	0.000234	hypermethylated
cg14196225	0.14802	0.360982162	1.286135	8.84E-05	hypermethylated
cg14289542	0.17881	0.435913514	1.285615	4.54E-06	hypermethylated
cg20157525	0.14046	0.342397297	1.285512	0.000132	hypermethylated
cg08874512	0.13598	0.331472973	1.285497	7.29E-05	hypermethylated
cg04028570	0.15533	0.378544324	1.285126	9.49E-05	hypermethylated
cg18599790	0.18759	0.457041081	1.284741	8.22E-05	hypermethylated
cg08496953	0.19294	0.47006973	1.284723	9.44E-06	hypermethylated
cg18538958	0.13067	0.318288649	1.284408	0.000613	hypermethylated
cg08538258	0.17378	0.423261081	1.284286	0.000132	hypermethylated
cg10778288	0.0915	0.222853514	1.284252	1.77E-05	hypermethylated
cg20912770	0.13938	0.339462703	1.28423	0.000229	hypermethylated
cg22355517	0.11473	0.279381622	1.283994	7.47E-05	hypermethylated
cg20049415	0.18964	0.461727027	1.283777	1.66E-05	hypermethylated
cg17062829	0.13726	0.334156216	1.283611	2.78E-05	hypermethylated
cg14336011	0.13256	0.322691892	1.283512	7.65E-05	hypermethylated
cg01085362	0.14958	0.364108108	1.28345	1.25E-05	hypermethylated
cg16859420	0.15161	0.369011892	1.283302	1.46E-05	hypermethylated
cg03789420	0.13472	0.327766486	1.282704	4.70E-05	hypermethylated
cg16739441	0.1758	0.427520541	1.282059	8.37E-06	hypermethylated
cg01656244	0.16485	0.400780541	1.281659	2.60E-06	hypermethylated
cg19505136	0.20127	0.489275676	1.281515	3.67E-05	hypermethylated
cg10428345	0.12668	0.307848649	1.281032	5.32E-05	hypermethylated
cg24913868	0.18693	0.454258378	1.281015	2.57E-06	hypermethylated
cg08417620	0.12931	0.314223243	1.280956	0.000135	hypermethylated
cg15912800	0.16693	0.405612432	1.280859	0.000123	hypermethylated
cg24066316	0.13924	0.338322703	1.280826	2.35E-05	hypermethylated
cg19630629	0.1514	0.367825946	1.280658	6.95E-05	hypermethylated
cg05043349	0.16727	0.40625027	1.28019	6.15E-05	hypermethylated
cg04857033	0.20382	0.494982162	1.280081	1.22E-06	hypermethylated
cg15505412	0.11642	0.282723784	1.280054	1.09E-05	hypermethylated
cg22057720	0.1306	0.317129189	1.279916	7.65E-05	hypermethylated
cg10863075	0.12174	0.295604324	1.279864	5.14E-06	hypermethylated
cg05591716	0.12216	0.29652	1.279357	2.92E-05	hypermethylated
cg05936441	0.14312	0.347338919	1.279119	4.42E-06	hypermethylated
cg14401466	0.11437	0.277422703	1.278377	5.58E-05	hypermethylated
cg01637233	0.10412	0.252544865	1.278292	1.06E-05	hypermethylated
cg13140167	0.12207	0.29605027	1.278133	1.35E-05	hypermethylated
cg15108727	0.17819	0.432130811	1.278052	1.01E-05	hypermethylated
cg01574673	0.11982	0.290547568	1.277906	0.000104	hypermethylated
cg08443038	0.10498	0.25454973	1.277833	2.78E-05	hypermethylated
cg03634479	0.10235	0.248159459	1.277756	2.24E-05	hypermethylated
cg18161327	0.21088	0.511231351	1.277554	5.14E-06	hypermethylated
cg04556126	0.19527	0.473375135	1.277514	8.83E-06	hypermethylated
cg26735846	0.13915	0.337244324	1.277153	0.000626	hypermethylated
cg26667946	0.18905	0.458102162	1.276902	0.000234	hypermethylated
cg20662725	0.13071	0.316625405	1.276408	0.000209	hypermethylated
cg22976218	0.15719	0.380747027	1.276323	0.000287	hypermethylated
cg08582356	0.13991	0.338844324	1.276124	3.26E-06	hypermethylated

cg05646686	0.15261	0.36958973	1.276075	1.80E-05	hypermethylated
cg11214140	0.21079	0.510452432	1.27597	9.31E-06	hypermethylated
cg00942219	0.16297	0.394643243	1.275943	3.62E-05	hypermethylated
cg20695433	0.12809	0.310042703	1.275309	9.57E-06	hypermethylated
cg03411507	0.087	0.210492432	1.274681	7.65E-05	hypermethylated
cg09557462	0.16306	0.394485946	1.274571	7.12E-06	hypermethylated
cg25345178	0.13919	0.336729189	1.274533	0.00024	hypermethylated
cg12530864	0.24378	0.589731892	1.274479	6.57E-06	hypermethylated
cg21678813	0.18564	0.449048649	1.274364	2.07E-05	hypermethylated
cg02569613	0.10968	0.265296216	1.274304	0.000106	hypermethylated
cg05213609	0.12258	0.296498378	1.274301	7.20E-05	hypermethylated
cg21052660	0.10796	0.261135135	1.2743	2.71E-05	hypermethylated
cg09231171	0.1486	0.359416757	1.274224	0.000138	hypermethylated
cg20779964	0.1926	0.46578	1.274041	1.35E-05	hypermethylated
cg20339230	0.10808	0.261364865	1.273966	0.000178	hypermethylated
cg17507671	0.12085	0.292213514	1.273805	1.21E-05	hypermethylated
cg20432507	0.1332	0.321932973	1.273166	2.81E-05	hypermethylated
cg17398677	0.11604	0.280384324	1.272783	1.09E-05	hypermethylated
cg19350360	0.19526	0.471762162	1.272663	3.19E-05	hypermethylated
cg05826596	0.14386	0.347550811	1.272558	3.32E-05	hypermethylated
cg19082560	0.1299	0.313809189	1.272486	2.87E-06	hypermethylated
cg10682299	0.14856	0.358817838	1.272206	4.82E-05	hypermethylated
cg13944468	0.19691	0.475597838	1.272206	7.84E-05	hypermethylated
cg26078977	0.17256	0.416743784	1.272063	5.86E-05	hypermethylated
cg07006091	0.12669	0.30590973	1.271803	2.21E-05	hypermethylated
cg15543551	0.11565	0.279235135	1.271715	0.000245	hypermethylated
cg26053480	0.19151	0.46234	1.271534	4.24E-06	hypermethylated
cg18198999	0.14725	0.355482703	1.271512	7.93E-05	hypermethylated
cg21982455	0.16603	0.400741622	1.271228	3.74E-06	hypermethylated
cg18417954	0.18653	0.450142703	1.270975	1.18E-05	hypermethylated
cg11891393	0.16763	0.40446973	1.270751	7.29E-05	hypermethylated
cg08491188	0.17253	0.416193514	1.270407	7.93E-06	hypermethylated
cg27314569	0.18465	0.44534	1.270114	5.58E-06	hypermethylated
cg03540175	0.13892	0.33501027	1.269951	0.000506	hypermethylated
cg21817858	0.22641	0.545914595	1.269738	2.95E-06	hypermethylated
cg21452281	0.16197	0.390517838	1.269662	2.87E-06	hypermethylated
cg14941291	0.14246	0.343477297	1.269658	1.54E-05	hypermethylated
cg19193956	0.17486	0.421578919	1.269602	6.62E-05	hypermethylated
cg03638905	0.15846	0.38190973	1.269113	4.80E-06	hypermethylated
cg23201513	0.10932	0.263447027	1.268956	6.62E-05	hypermethylated
cg17270257	0.1559	0.37555027	1.268385	6.62E-05	hypermethylated
cg01582980	0.14942	0.359767027	1.26769	0.000159	hypermethylated
cg23929344	0.14525	0.349709189	1.267618	0.000234	hypermethylated
cg19107469	0.14983	0.360692432	1.267443	1.75E-05	hypermethylated
cg20894686	0.10319	0.248403243	1.267381	2.96E-05	hypermethylated
cg13400168	0.14155	0.340741081	1.267364	1.84E-05	hypermethylated
cg18291941	0.15595	0.375392973	1.267318	9.31E-06	hypermethylated
cg06636427	0.15336	0.36906973	1.266971	4.82E-05	hypermethylated
cg05076914	0.16001	0.385042703	1.266856	0.000122	hypermethylated
cg02805665	0.11819	0.28432	1.266408	0.000214	hypermethylated
cg27223047	0.20629	0.496207568	1.26627	5.45E-05	hypermethylated
cg14186937	0.1327	0.319158919	1.266107	9.49E-05	hypermethylated
cg11786193	0.09396	0.225951892	1.265897	0.000262	hypermethylated
cg05099185	0.12738	0.306272973	1.265679	1.60E-05	hypermethylated
cg26173630	0.1134	0.272528649	1.264987	1.71E-05	hypermethylated
cg18164784	0.1536	0.369074054	1.264732	5.45E-05	hypermethylated
cg16762735	0.11211	0.269281081	1.264198	2.64E-05	hypermethylated
cg10567810	0.1274	0.305953514	1.263947	6.15E-05	hypermethylated

cg26128092	0.15838	0.380327027	1.26385	1.66E-05	hypermethylated
cg00250430	0.16477	0.395512973	1.263271	0.00018	hypermethylated
cg10675138	0.12619	0.302874054	1.26312	0.000696	hypermethylated
cg10298052	0.16901	0.405646486	1.263114	8.63E-05	hypermethylated
cg17939805	0.13862	0.332696757	1.263072	4.12E-06	hypermethylated
cg03934713	0.11882	0.285127027	1.262827	3.32E-05	hypermethylated
cg25888561	0.2185	0.524282703	1.262712	1.52E-05	hypermethylated
cg08832603	0.1716	0.411668649	1.262434	5.12E-05	hypermethylated
cg23493016	0.16527	0.396481622	1.262429	3.95E-05	hypermethylated
cg14741939	0.13712	0.328855676	1.262016	2.32E-05	hypermethylated
cg16472050	0.18229	0.437100541	1.26173	3.21E-06	hypermethylated
cg08829841	0.19653	0.471095676	1.261271	1.50E-05	hypermethylated
cg15506157	0.23901	0.572904324	1.261223	0.003316	hypermethylated
cg21656751	0.16232	0.389002703	1.260939	1.25E-05	hypermethylated
cg07923686	0.16687	0.399859459	1.260768	1.97E-05	hypermethylated
cg25756635	0.12969	0.310694054	1.260427	4.36E-05	hypermethylated
cg13778709	0.12648	0.302961081	1.260223	1.39E-05	hypermethylated
cg26492368	0.20093	0.481276757	1.260174	9.07E-06	hypermethylated
cg23243038	0.12077	0.289235135	1.259981	2.30E-05	hypermethylated
cg03340466	0.13526	0.323932432	1.259958	0.000445	hypermethylated
cg23037403	0.12882	0.308388649	1.259393	2.32E-05	hypermethylated
cg10767141	0.12761	0.305490811	1.259388	9.83E-05	hypermethylated
cg23317501	0.16642	0.398330811	1.259138	3.21E-06	hypermethylated
cg00412749	0.11235	0.268900541	1.259073	2.45E-05	hypermethylated
cg14565725	0.20433	0.489033514	1.259032	4.24E-06	hypermethylated
cg03688699	0.13306	0.31842	1.258854	8.42E-05	hypermethylated
cg04178266	0.14229	0.340496757	1.258807	1.20E-05	hypermethylated
cg06522054	0.12179	0.291375135	1.258482	9.05E-05	hypermethylated
cg01586959	0.13566	0.324556216	1.258473	0.000178	hypermethylated
cg01455568	0.12939	0.309442162	1.257944	7.47E-05	hypermethylated
cg12021814	0.17055	0.407866486	1.257902	1.99E-05	hypermethylated
cg11930477	0.13893	0.332235676	1.257849	5.35E-06	hypermethylated
cg03348397	0.15572	0.372378378	1.257815	1.01E-05	hypermethylated
cg07715201	0.11918	0.284989189	1.257765	4.82E-05	hypermethylated
cg13974632	0.136	0.325168108	1.257579	0.000681	hypermethylated
cg15345206	0.13756	0.328895135	1.257567	1.52E-05	hypermethylated
cg16086559	0.17956	0.429227027	1.257275	9.38E-05	hypermethylated
cg15268136	0.11063	0.264378378	1.256862	0.00018	hypermethylated
cg01574663	0.14762	0.352690811	1.256516	1.71E-05	hypermethylated
cg10991599	0.11032	0.263572973	1.256508	2.15E-05	hypermethylated
cg07281879	0.18179	0.434323243	1.256496	7.93E-06	hypermethylated
cg15621260	0.16874	0.403057297	1.256183	7.84E-05	hypermethylated
cg02649597	0.1383	0.330338919	1.256146	4.42E-06	hypermethylated
cg23479922	0.22288	0.532308649	1.255996	9.31E-06	hypermethylated
cg07244354	0.12964	0.30954973	1.25566	7.93E-06	hypermethylated
cg20977794	0.16548	0.394933514	1.254953	2.15E-05	hypermethylated
cg01190024	0.16237	0.387423243	1.254625	2.10E-05	hypermethylated
cg18745131	0.11639	0.277677297	1.254442	9.44E-06	hypermethylated
cg13234863	0.14479	0.345381081	1.254227	2.57E-05	hypermethylated
cg10638780	0.10507	0.25062973	1.254207	2.15E-05	hypermethylated
cg27282264	0.19912	0.474916757	1.254037	3.15E-05	hypermethylated
cg02593932	0.12997	0.309908649	1.253664	0.00024	hypermethylated
cg26014036	0.1892	0.451087027	1.253494	2.74E-05	hypermethylated
cg11856949	0.09782	0.233161622	1.253129	1.35E-05	hypermethylated
cg07173635	0.15105	0.36002973	1.25309	1.35E-05	hypermethylated
cg13298841	0.1793	0.427359459	1.253075	6.62E-05	hypermethylated
cg10741333	0.12236	0.291600541	1.252861	0.000195	hypermethylated
cg13844591	0.151	0.359803784	1.252662	5.93E-05	hypermethylated

cg05937737	0.20847	0.496741081	1.252654	1.50E-05	hypermethylated
cg09283154	0.131	0.312139459	1.252624	1.62E-05	hypermethylated
cg06516124	0.1655	0.394321622	1.252542	3.67E-05	hypermethylated
cg09366312	0.11389	0.271355135	1.252541	0.000229	hypermethylated
cg11548303	0.13034	0.310544324	1.252519	0.000445	hypermethylated
cg26980244	0.1958	0.4665	1.252496	7.32E-06	hypermethylated
cg09251429	0.14605	0.347919459	1.252291	0.000696	hypermethylated
cg14515453	0.1372	0.326827027	1.252247	1.35E-05	hypermethylated
cg00177388	0.19048	0.453727568	1.252187	2.23E-06	hypermethylated
cg00089091	0.15685	0.373596216	1.252094	0.000426	hypermethylated
cg02473540	0.19051	0.453751351	1.252035	2.89E-05	hypermethylated
cg10506882	0.14487	0.345046486	1.252032	9.60E-05	hypermethylated
cg22070855	0.13441	0.320115135	1.25195	8.42E-05	hypermethylated
cg04324666	0.14175	0.337589189	1.25192	1.28E-05	hypermethylated
cg24286765	0.18159	0.432438919	1.251812	3.04E-06	hypermethylated
cg19355079	0.14227	0.338678919	1.251287	9.44E-06	hypermethylated
cg09617993	0.14312	0.340671892	1.251158	4.82E-05	hypermethylated
cg17848763	0.13409	0.319108108	1.250844	7.74E-05	hypermethylated
cg03108139	0.13345	0.317554054	1.250703	9.72E-05	hypermethylated
cg13951074	0.12735	0.30296973	1.250375	1.62E-05	hypermethylated
cg04591034	0.15805	0.375974595	1.250254	4.36E-06	hypermethylated
cg23951961	0.16934	0.402817838	1.250205	1.06E-05	hypermethylated
cg23618344	0.13392	0.318464865	1.249763	7.29E-05	hypermethylated
cg14626309	0.20174	0.47968	1.249575	1.12E-05	hypermethylated
cg10308833	0.11449	0.272223243	1.249569	0.000107	hypermethylated
cg27635069	0.13888	0.330212432	1.249556	1.68E-05	hypermethylated
cg04209913	0.27731	0.659326486	1.249493	3.90E-06	hypermethylated
cg07104209	0.14554	0.346019459	1.249437	0.000102	hypermethylated
cg18451814	0.19217	0.456881081	1.249436	6.57E-06	hypermethylated
cg02681442	0.19384	0.46074973	1.249117	5.35E-06	hypermethylated
cg23900203	0.15975	0.379713514	1.249095	1.99E-05	hypermethylated
cg25739875	0.13478	0.320310811	1.248866	0.000268	hypermethylated
cg00592781	0.14272	0.339118919	1.248604	0.000421	hypermethylated
cg09643544	0.16608	0.394616216	1.248572	1.99E-06	hypermethylated
cg09071889	0.16604	0.394504865	1.248512	1.99E-05	hypermethylated
cg16638540	0.16418	0.38994	1.247974	2.10E-05	hypermethylated
cg07681935	0.19326	0.458976757	1.247878	0.000166	hypermethylated
cg16002818	0.16581	0.39375027	1.24775	1.89E-05	hypermethylated
cg11750592	0.1454	0.345276757	1.247726	6.01E-05	hypermethylated
cg21714266	0.17313	0.41107027	1.247529	2.64E-05	hypermethylated
cg04926361	0.12424	0.294967568	1.247427	0.000259	hypermethylated
cg07448060	0.13507	0.320623243	1.247172	0.00039	hypermethylated
cg10298815	0.12772	0.303146486	1.247031	9.27E-05	hypermethylated
cg07949597	0.19746	0.468644865	1.246935	6.01E-05	hypermethylated
cg08393041	0.18807	0.446334595	1.246856	1.12E-05	hypermethylated
cg21376883	0.1497	0.355269189	1.246838	1.37E-05	hypermethylated
cg12196573	0.13392	0.317804865	1.24677	1.12E-05	hypermethylated
cg21535606	0.14014	0.332562703	1.246758	1.58E-05	hypermethylated
cg10238972	0.16287	0.386375676	1.246283	1.04E-05	hypermethylated
cg01003961	0.20355	0.482854054	1.246204	1.12E-05	hypermethylated
cg03391684	0.13593	0.32244	1.246167	0.000112	hypermethylated
cg08470764	0.10871	0.257816757	1.245861	2.13E-05	hypermethylated
cg09155219	0.10569	0.250602703	1.245563	4.60E-06	hypermethylated
cg06765217	0.17537	0.415770811	1.245387	3.57E-05	hypermethylated
cg17062109	0.13854	0.328451351	1.245377	0.000485	hypermethylated
cg14659346	0.17533	0.415662162	1.245339	0.000191	hypermethylated
cg18315960	0.16898	0.4006	1.24531	1.80E-05	hypermethylated
cg09208611	0.11097	0.263024865	1.245029	0.000102	hypermethylated

cg01104047	0.12114	0.287076216	1.244758	4.26E-05	hypermethylated
cg05749717	0.1354	0.320695135	1.243975	0.00024	hypermethylated
cg12424785	0.13865	0.328367568	1.243864	0.000166	hypermethylated
cg00073837	0.17494	0.414245405	1.243625	2.79E-06	hypermethylated
cg16524049	0.19378	0.458725946	1.243213	2.89E-05	hypermethylated
cg05771261	0.11827	0.279921081	1.242936	2.39E-05	hypermethylated
cg10648197	0.14084	0.333317838	1.242841	0.00012	hypermethylated
cg21091227	0.12465	0.294932432	1.242502	2.04E-05	hypermethylated
cg25508679	0.18516	0.438103784	1.2425	0.000511	hypermethylated
cg12158272	0.1014	0.239912432	1.24245	4.10E-05	hypermethylated
cg05658491	0.13762	0.325571351	1.242284	5.58E-05	hypermethylated
cg10528576	0.2472	0.584743243	1.242125	0.000135	hypermethylated
cg08278487	0.11841	0.280091892	1.242109	0.000237	hypermethylated
cg23016129	0.16187	0.382785946	1.241702	1.12E-05	hypermethylated
cg14795752	0.11556	0.273245946	1.241558	6.46E-05	hypermethylated
cg05037662	0.14346	0.33916	1.241317	0.000104	hypermethylated
cg26335633	0.13949	0.329655135	1.240796	7.12E-05	hypermethylated
cg03748376	0.19161	0.452802703	1.24071	0.000174	hypermethylated
cg27391267	0.16691	0.394357838	1.240435	3.36E-05	hypermethylated
cg24871714	0.17877	0.422355676	1.240354	5.45E-05	hypermethylated
cg09184467	0.07162	0.1692	1.240295	7.93E-05	hypermethylated
cg16973098	0.18238	0.430858919	1.240268	5.06E-05	hypermethylated
cg09257824	0.13524	0.319494054	1.240267	2.48E-05	hypermethylated
cg06460717	0.13854	0.327287568	1.240256	0.000117	hypermethylated
cg18445088	0.16555	0.391083243	1.240209	1.29E-06	hypermethylated
cg09820150	0.12451	0.294058378	1.239841	2.71E-05	hypermethylated
cg18073471	0.14771	0.348828649	1.239751	6.01E-05	hypermethylated
cg05722918	0.09705	0.229175676	1.239654	0.0002	hypermethylated
cg16147305	0.10165	0.240036216	1.239642	2.45E-05	hypermethylated
cg14901205	0.19409	0.458277297	1.239495	5.51E-05	hypermethylated
cg15829006	0.10156	0.239775135	1.23935	7.47E-05	hypermethylated
cg25537434	0.1285	0.30336	1.239263	0.000346	hypermethylated
cg09782540	0.12515	0.295434595	1.23918	1.66E-05	hypermethylated
cg07304536	0.14177	0.334595676	1.238867	1.52E-05	hypermethylated
cg09651145	0.15108	0.356498378	1.238583	0.000204	hypermethylated
cg12810548	0.08017	0.18916973	1.238547	4.15E-05	hypermethylated
cg13929328	0.17086	0.403058378	1.238174	3.59E-06	hypermethylated
cg07713929	0.12405	0.292612432	1.238069	1.31E-05	hypermethylated
cg12426652	0.16627	0.392123243	1.237779	3.85E-05	hypermethylated
cg20859731	0.18957	0.447029189	1.237638	8.15E-06	hypermethylated
cg02657836	0.17194	0.40544973	1.237618	6.93E-06	hypermethylated
cg25775322	0.13371	0.315288649	1.237566	0.000109	hypermethylated
cg18016181	0.16456	0.387934054	1.237198	5.58E-05	hypermethylated
cg00875511	0.12924	0.304669189	1.237191	0.000274	hypermethylated
cg13688769	0.10736	0.253080541	1.23714	0.000234	hypermethylated
cg14230397	0.14477	0.341184865	1.236791	4.20E-05	hypermethylated
cg21297493	0.20773	0.489544865	1.236732	2.05E-06	hypermethylated
cg08475096	0.12329	0.290541081	1.236686	0.000259	hypermethylated
cg11899535	0.16976	0.400014054	1.236554	2.85E-05	hypermethylated
cg26509691	0.15498	0.365151892	1.236415	5.58E-06	hypermethylated
cg27100227	0.1693	0.398842162	1.236236	8.32E-05	hypermethylated
cg13691003	0.11895	0.280194054	1.236071	0.000191	hypermethylated
cg06014958	0.1356	0.319342162	1.235746	1.42E-05	hypermethylated
cg00486340	0.18144	0.427289189	1.23572	5.43E-06	hypermethylated
cg20009101	0.12757	0.300415676	1.235671	1.84E-05	hypermethylated
cg23409403	0.137	0.322589189	1.235522	4.05E-05	hypermethylated
cg06535308	0.13514	0.318173514	1.235359	3.57E-05	hypermethylated
cg06228507	0.15831	0.372654054	1.235085	6.39E-06	hypermethylated

cg00779065	0.17652	0.41551027	1.235052	4.47E-05	hypermethylated
cg12298745	0.17117	0.402805405	1.234653	2.04E-05	hypermethylated
cg10319893	0.11705	0.275431351	1.234568	0.000161	hypermethylated
cg10290276	0.15625	0.367488649	1.233843	5.19E-05	hypermethylated
cg25542878	0.21663	0.509405405	1.233581	4.26E-05	hypermethylated
cg08263708	0.14583	0.342824865	1.233184	4.12E-06	hypermethylated
cg21100077	0.17734	0.416858919	1.233041	1.01E-05	hypermethylated
cg07921625	0.14989	0.352332432	1.233033	3.69E-06	hypermethylated
cg24642236	0.09859	0.231600541	1.232125	3.15E-05	hypermethylated
cg04194073	0.13532	0.317839459	1.231923	3.15E-05	hypermethylated
cg01462545	0.12219	0.286876757	1.231305	5.65E-05	hypermethylated
cg25701114	0.16773	0.393738378	1.231097	0.0002	hypermethylated
cg24653263	0.1562	0.366671892	1.231095	0.000132	hypermethylated
cg03329976	0.17638	0.414021081	1.231017	1.71E-05	hypermethylated
cg08529882	0.16017	0.375936757	1.230886	5.58E-06	hypermethylated
cg12052765	0.13855	0.325176216	1.230815	2.27E-05	hypermethylated
cg17051321	0.12373	0.290374054	1.230717	5.43E-06	hypermethylated
cg25905812	0.12466	0.292491351	1.230395	0.0003	hypermethylated
cg07552803	0.17513	0.410892432	1.230335	9.07E-06	hypermethylated
cg21117210	0.14353	0.336705946	1.230137	4.36E-05	hypermethylated
cg22187630	0.17284	0.405420541	1.229982	7.22E-06	hypermethylated
cg15425280	0.12768	0.299480541	1.22993	9.49E-05	hypermethylated
cg04022561	0.20189	0.473480541	1.229736	3.79E-06	hypermethylated
cg25335557	0.11684	0.273982162	1.229548	6.23E-05	hypermethylated
cg13581422	0.14149	0.331756757	1.229426	0.000212	hypermethylated
cg14016236	0.24063	0.564176216	1.229329	3.67E-05	hypermethylated
cg07949989	0.14064	0.329717838	1.229225	1.58E-06	hypermethylated
cg04317962	0.20593	0.48264	1.228793	3.53E-05	hypermethylated
cg26425256	0.12932	0.303084865	1.228776	8.94E-05	hypermethylated
cg24035245	0.1954	0.457892432	1.228578	1.75E-05	hypermethylated
cg07531228	0.10914	0.255652432	1.228004	1.71E-05	hypermethylated
cg04043571	0.11357	0.266016216	1.227932	4.60E-06	hypermethylated
cg16108230	0.18525	0.433910811	1.227925	9.31E-06	hypermethylated
cg18750960	0.19004	0.445073514	1.227741	2.02E-05	hypermethylated
cg17078253	0.17777	0.416302703	1.227621	1.21E-05	hypermethylated
cg21088983	0.15571	0.364632973	1.227583	0.000108	hypermethylated
cg18185958	0.1214	0.284274054	1.227514	1.94E-05	hypermethylated
cg13540960	0.15404	0.360663243	1.227347	0.000346	hypermethylated
cg06470822	0.13658	0.319716216	1.227046	5.58E-05	hypermethylated
cg16705627	0.18234	0.426820541	1.226999	3.40E-05	hypermethylated
cg22491141	0.16505	0.386181622	1.226376	1.31E-05	hypermethylated
cg07675184	0.12791	0.299147568	1.225728	3.67E-05	hypermethylated
cg01695225	0.16315	0.381539459	1.225633	0.000124	hypermethylated
cg12089570	0.1458	0.340960541	1.225614	6.57E-06	hypermethylated
cg14647451	0.14085	0.329376757	1.225579	8.94E-05	hypermethylated
cg27294268	0.11342	0.265176757	1.225279	0.00024	hypermethylated
cg18275316	0.17556	0.410316216	1.224772	5.84E-07	hypermethylated
cg25617725	0.11652	0.27230973	1.224671	3.76E-05	hypermethylated
cg01315916	0.17038	0.398151892	1.224563	3.59E-06	hypermethylated
cg13371839	0.1538	0.35939027	1.224496	1.50E-05	hypermethylated
cg08551532	0.17497	0.408851892	1.224471	9.72E-05	hypermethylated
cg26709285	0.18974	0.443128108	1.2237	3.30E-06	hypermethylated
cg21778348	0.13441	0.3139	1.223665	4.47E-05	hypermethylated
cg16701059	0.14626	0.341542703	1.223531	4.60E-06	hypermethylated
cg18736063	0.14529	0.339241081	1.223375	2.54E-05	hypermethylated
cg14780132	0.13659	0.318824324	1.22291	0.000109	hypermethylated
cg16198087	0.1264	0.295003243	1.222734	0.000172	hypermethylated
cg12380743	0.21177	0.494118919	1.22236	1.66E-05	hypermethylated

cg26988423	0.13847	0.323085946	1.222345	2.39E-05	hypermethylated
cg10943359	0.16856	0.393265946	1.222243	1.06E-05	hypermethylated
cg16536329	0.14307	0.33379027	1.222221	3.40E-05	hypermethylated
cg18539122	0.18495	0.431464865	1.222108	3.69E-06	hypermethylated
cg03255182	0.16468	0.384161622	1.222048	6.57E-06	hypermethylated
cg19300741	0.0993	0.231631892	1.221968	1.26E-05	hypermethylated
cg16907558	0.18222	0.424897838	1.221435	9.38E-05	hypermethylated
cg06128028	0.12065	0.281256216	1.221057	4.73E-06	hypermethylated
cg06497668	0.12995	0.302927568	1.221016	1.71E-05	hypermethylated
cg20599066	0.10936	0.254888649	1.220782	2.57E-05	hypermethylated
cg01142635	0.2001	0.466335135	1.220646	9.82E-06	hypermethylated
cg15732768	0.16501	0.384543243	1.220592	1.50E-05	hypermethylated
cg23572163	0.09276	0.216155676	1.220496	7.12E-06	hypermethylated
cg09066326	0.14967	0.348656757	1.220022	7.65E-05	hypermethylated
cg00903099	0.15232	0.354785405	1.219841	1.99E-05	hypermethylated
cg16913789	0.12272	0.285817297	1.219723	6.31E-05	hypermethylated
cg07405021	0.1066	0.248238378	1.219519	5.43E-06	hypermethylated
cg17087640	0.13446	0.313081622	1.219362	0.000124	hypermethylated
cg13397057	0.1482	0.345057297	1.21929	1.99E-05	hypermethylated
cg15039102	0.1747	0.406734054	1.219206	1.62E-05	hypermethylated
cg08012278	0.14835	0.34538	1.21918	1.35E-05	hypermethylated
cg11938455	0.15453	0.359711892	1.218955	9.57E-06	hypermethylated
cg23125492	0.1743	0.405616757	1.218545	5.43E-06	hypermethylated
cg10018167	0.16402	0.38166973	1.218453	1.99E-05	hypermethylated
cg01142710	0.15139	0.351964865	1.217162	4.15E-05	hypermethylated
cg00842325	0.16892	0.392702162	1.217095	9.95E-05	hypermethylated
cg00860808	0.15886	0.369285405	1.21698	6.31E-05	hypermethylated
cg03099208	0.15438	0.358841081	1.216859	1.14E-05	hypermethylated
cg09714379	0.15679	0.364377838	1.216602	0.000187	hypermethylated
cg01685883	0.15028	0.349108108	1.216021	7.47E-05	hypermethylated
cg01296889	0.16184	0.375957838	1.216003	4.05E-05	hypermethylated
cg18693673	0.17584	0.408461081	1.215935	1.99E-05	hypermethylated
cg15830509	0.11303	0.262543243	1.215849	1.71E-05	hypermethylated
cg16158681	0.12535	0.291145946	1.215781	0.000303	hypermethylated
cg22836229	0.14913	0.346355135	1.215682	0.000214	hypermethylated
cg08204023	0.16331	0.379286486	1.215675	2.39E-05	hypermethylated
cg18912520	0.15187	0.352714054	1.215662	2.32E-05	hypermethylated
cg17857974	0.15758	0.365951892	1.21557	7.38E-05	hypermethylated
cg14785479	0.10135	0.235356757	1.215503	1.46E-05	hypermethylated
cg17093795	0.13434	0.31186973	1.215055	2.27E-05	hypermethylated
cg09926747	0.13687	0.317702162	1.214869	0.000426	hypermethylated
cg24932457	0.22657	0.525901081	1.214835	2.27E-05	hypermethylated
cg00044796	0.17195	0.398987027	1.214353	0.000182	hypermethylated
cg10241484	0.13543	0.314211892	1.21419	2.04E-05	hypermethylated
cg09017434	0.21983	0.509956757	1.213987	6.13E-06	hypermethylated
cg14452947	0.11303	0.26216973	1.213795	0.000378	hypermethylated
cg12355110	0.17668	0.409748649	1.2136	1.04E-05	hypermethylated
cg18425434	0.18416	0.427075135	1.21353	0.000117	hypermethylated
cg06428620	0.20034	0.464552432	1.213391	0.000178	hypermethylated
cg03087607	0.12796	0.296657297	1.213104	3.95E-05	hypermethylated
cg18761549	0.17556	0.406970811	1.212961	1.84E-05	hypermethylated
cg20038996	0.11317	0.262334054	1.212914	5.86E-05	hypermethylated
cg18930354	0.11785	0.273172973	1.212863	2.57E-05	hypermethylated
cg18293662	0.15722	0.364429189	1.212854	2.32E-05	hypermethylated
cg16415058	0.16449	0.381220541	1.212626	1.15E-05	hypermethylated
cg05678100	0.12186	0.282301081	1.21201	6.95E-05	hypermethylated
cg10141715	0.1883	0.436112973	1.211669	1.62E-05	hypermethylated
cg03526459	0.21116	0.489017297	1.211549	1.35E-05	hypermethylated

cg02069715	0.186	0.430416757	1.210432	6.39E-06	hypermethylated
cg05129325	0.19442	0.449872432	1.210339	2.42E-05	hypermethylated
cg19718882	0.19172	0.443621622	1.210329	1.71E-05	hypermethylated
cg04326198	0.1277	0.295479459	1.210299	2.10E-05	hypermethylated
cg22945387	0.10326	0.23890973	1.210184	6.95E-05	hypermethylated
cg08251037	0.14573	0.337168108	1.21017	8.37E-06	hypermethylated
cg24680632	0.21413	0.495325405	1.20989	0.00012	hypermethylated
cg05336395	0.23466	0.542683243	1.209538	2.05E-06	hypermethylated
cg05710997	0.09994	0.231071892	1.209208	0.000327	hypermethylated
cg11100386	0.13923	0.321911892	1.209196	4.26E-05	hypermethylated
cg26597242	0.17596	0.406802703	1.209082	0.000287	hypermethylated
cg03631131	0.14557	0.336516757	1.208965	3.49E-06	hypermethylated
cg22813950	0.17359	0.401220541	1.208712	2.51E-05	hypermethylated
cg07350501	0.14862	0.343486486	1.208625	1.12E-06	hypermethylated
cg18236477	0.19597	0.452863243	1.208443	1.09E-05	hypermethylated
cg01366595	0.13552	0.313106486	1.208148	5.00E-05	hypermethylated
cg15149095	0.1595	0.368494595	1.208087	1.99E-05	hypermethylated
cg07811198	0.19731	0.455762162	1.207817	1.01E-05	hypermethylated
cg13563725	0.16336	0.377324865	1.207752	3.07E-05	hypermethylated
cg01720455	0.15349	0.354463243	1.207491	4.86E-06	hypermethylated
cg20116804	0.15692	0.362343784	1.20733	0.000115	hypermethylated
cg00849713	0.13818	0.319043243	1.207203	6.62E-05	hypermethylated
cg00733728	0.14702	0.339417838	1.20705	9.27E-05	hypermethylated
cg15482122	0.14124	0.326062703	1.207001	2.21E-05	hypermethylated
cg00348762	0.18906	0.436450811	1.206975	1.62E-05	hypermethylated
cg14224762	0.11781	0.271963243	1.20695	0.000102	hypermethylated
cg12230289	0.13851	0.319748108	1.206946	7.29E-05	hypermethylated
cg10409302	0.12601	0.290853514	1.206755	0.000142	hypermethylated
cg13623495	0.12895	0.297632973	1.206723	2.56E-06	hypermethylated
cg24495585	0.17969	0.414591892	1.206182	0.000317	hypermethylated
cg25562664	0.13518	0.311876216	1.206092	6.46E-05	hypermethylated
cg04364726	0.08366	0.192988108	1.205902	0.000485	hypermethylated
cg06845853	0.20058	0.462664865	1.20579	3.32E-05	hypermethylated
cg10601940	0.17229	0.397363784	1.205621	5.58E-06	hypermethylated
cg02919615	0.1855	0.427752973	1.205359	3.95E-05	hypermethylated
cg18158990	0.1321	0.30458	1.205191	0.000293	hypermethylated
cg19502744	0.15043	0.346826486	1.205122	8.22E-05	hypermethylated
cg23075337	0.21474	0.495062703	1.20502	5.19E-05	hypermethylated
cg17555373	0.1955	0.450589189	1.204644	1.09E-05	hypermethylated
cg25319067	0.1742	0.401469189	1.204545	7.29E-05	hypermethylated
cg01914621	0.16218	0.373754595	1.204495	3.15E-05	hypermethylated
cg11344566	0.19219	0.442880541	1.204384	1.80E-05	hypermethylated
cg04005969	0.17246	0.397378378	1.204252	3.15E-05	hypermethylated
cg19716643	0.16567	0.38171027	1.204166	1.37E-05	hypermethylated
cg25019989	0.14955	0.344545405	1.204066	2.92E-05	hypermethylated
cg24361162	0.15252	0.351371892	1.204	3.00E-05	hypermethylated
cg21875330	0.15348	0.353515135	1.203721	1.09E-05	hypermethylated
cg13547644	0.14646	0.337255135	1.203334	5.73E-06	hypermethylated
cg20084504	0.15915	0.366423784	1.203126	8.60E-06	hypermethylated
cg25746778	0.26212	0.603483784	1.203088	3.90E-06	hypermethylated
cg15703632	0.12189	0.28062	1.203038	0.000237	hypermethylated
cg09143673	0.15711	0.361693514	1.202993	4.94E-05	hypermethylated
cg08189801	0.14175	0.326331351	1.202989	7.12E-05	hypermethylated
cg26659079	0.16572	0.381471892	1.202829	2.95E-06	hypermethylated
cg19509393	0.17021	0.391760541	1.202656	1.35E-05	hypermethylated
cg24507762	0.13091	0.301267027	1.202467	6.15E-05	hypermethylated
cg08790440	0.16766	0.385794595	1.202294	3.07E-05	hypermethylated
cg17486860	0.14221	0.327122703	1.201809	8.94E-05	hypermethylated

cg20674635	0.12925	0.297267027	1.201595	2.15E-05	hypermethylated
cg19848629	0.12616	0.29010973	1.201344	0.000113	hypermethylated
cg21728840	0.17462	0.401506486	1.201204	3.15E-05	hypermethylated
cg04352272	0.14666	0.337184324	1.201062	0.00012	hypermethylated
cg12146673	0.12562	0.288790811	1.200959	0.000268	hypermethylated
cg10752158	0.17429	0.400642703	1.200826	2.48E-05	hypermethylated
cg07124117	0.14869	0.341767568	1.200708	6.70E-05	hypermethylated
cg13758543	0.18119	0.416457297	1.200665	4.26E-05	hypermethylated
cg00945234	0.12808	0.294355135	1.200513	2.96E-05	hypermethylated
cg04101351	0.11367	0.261236216	1.200503	9.05E-05	hypermethylated
cg20576153	0.1343	0.308574595	1.20016	5.45E-05	hypermethylated
cg24431667	0.11938	0.274287027	1.200125	0.00012	hypermethylated
cg20653075	0.147	0.337631892	1.199635	1.18E-05	hypermethylated
cg18267374	0.1408	0.323331892	1.199368	8.60E-06	hypermethylated
cg07871947	0.13973	0.320741081	1.198767	3.67E-05	hypermethylated
cg00629514	0.15813	0.362861622	1.198308	3.49E-05	hypermethylated
cg10157715	0.14809	0.339780541	1.198129	7.52E-06	hypermethylated
cg00534492	0.1269	0.29112973	1.19797	3.15E-05	hypermethylated
cg03887163	0.12712	0.291622703	1.197912	2.51E-05	hypermethylated
cg05827312	0.15421	0.353728108	1.197745	7.72E-06	hypermethylated
cg24292761	0.19741	0.452747568	1.197512	2.42E-05	hypermethylated
cg24947764	0.12828	0.294116216	1.19709	0.000147	hypermethylated
cg23602974	0.11519	0.264080541	1.196963	8.15E-06	hypermethylated
cg22418829	0.12916	0.296094595	1.196899	3.04E-05	hypermethylated
cg27079680	0.14954	0.342741622	1.19659	2.85E-05	hypermethylated
cg05895545	0.14009	0.321060541	1.196491	9.07E-06	hypermethylated
cg10132208	0.19379	0.444022162	1.196138	8.83E-06	hypermethylated
cg00630958	0.14088	0.322779459	1.196082	0.000115	hypermethylated
cg21305834	0.13537	0.310138919	1.196007	1.77E-05	hypermethylated
cg09919570	0.19715	0.451555135	1.195608	0.000214	hypermethylated
cg20822372	0.11566	0.264898919	1.195552	0.000109	hypermethylated
cg27136844	0.1996	0.457094595	1.195381	5.32E-05	hypermethylated
cg13080379	0.15863	0.363108649	1.194736	3.04E-06	hypermethylated
cg14089714	0.16486	0.377358919	1.194696	1.89E-05	hypermethylated
cg15984718	0.12247	0.280266486	1.194371	0.000306	hypermethylated
cg11469098	0.14745	0.337429189	1.194359	0.000921	hypermethylated
cg24811864	0.15198	0.347787027	1.194323	3.15E-05	hypermethylated
cg02623400	0.16663	0.381289189	1.194237	1.89E-05	hypermethylated
cg11573566	0.15776	0.360931351	1.193993	0.000129	hypermethylated
cg04081201	0.13268	0.303456757	1.19354	6.31E-05	hypermethylated
cg01352175	0.15112	0.345590811	1.19337	4.86E-06	hypermethylated
cg07751641	0.09735	0.222574595	1.193036	2.79E-06	hypermethylated
cg05477514	0.15358	0.351121081	1.192978	2.64E-05	hypermethylated
cg17282395	0.16258	0.371682703	1.192922	0.000207	hypermethylated
cg23008606	0.13266	0.303251892	1.192783	4.94E-05	hypermethylated
cg06966113	0.18338	0.419183243	1.192745	3.04E-06	hypermethylated
cg14617562	0.10811	0.247101081	1.192601	0.000789	hypermethylated
cg16197717	0.16457	0.376089189	1.192373	1.09E-05	hypermethylated
cg23704517	0.1608	0.367470811	1.192362	0.000219	hypermethylated
cg03914922	0.11561	0.264161622	1.192155	3.67E-05	hypermethylated
cg25027798	0.18236	0.416641081	1.192016	6.93E-06	hypermethylated
cg25708328	0.1361	0.310858919	1.191593	1.58E-05	hypermethylated
cg19951663	0.12136	0.277172973	1.191494	6.78E-05	hypermethylated
cg08575537	0.1747	0.398945405	1.191312	0.000931	hypermethylated
cg24509810	0.1872	0.427476757	1.191266	5.73E-06	hypermethylated
cg02677946	0.11718	0.267545946	1.19106	0.000106	hypermethylated
cg06340987	0.20604	0.470376757	1.190892	1.62E-05	hypermethylated
cg21035875	0.15234	0.347780541	1.190882	4.88E-05	hypermethylated

cg02362103	0.19464	0.444347027	1.190879	1.94E-05	hypermethylated
cg04104463	0.15517	0.354079459	1.190223	2.71E-05	hypermethylated
cg00505045	0.16263	0.371093514	1.190189	4.12E-06	hypermethylated
cg01066602	0.15552	0.354708108	1.189532	1.62E-05	hypermethylated
cg10434261	0.14377	0.327888108	1.189441	0.000154	hypermethylated
cg26721877	0.20424	0.465731351	1.189233	1.01E-05	hypermethylated
cg17023149	0.12152	0.277081081	1.189114	0.000161	hypermethylated
cg16211684	0.12646	0.288341622	1.189098	0.000119	hypermethylated
cg04895345	0.17533	0.399710811	1.188884	3.53E-05	hypermethylated
cg03338754	0.19838	0.45221027	1.188727	1.58E-05	hypermethylated
cg08582701	0.14503	0.330579459	1.188646	1.01E-05	hypermethylated
cg21926708	0.12553	0.286126486	1.188621	0.000132	hypermethylated
cg21694941	0.18972	0.432417838	1.188554	6.39E-06	hypermethylated
cg19414741	0.18467	0.420876757	1.188448	5.21E-06	hypermethylated
cg06263341	0.14321	0.32632	1.188155	5.06E-05	hypermethylated
cg02039267	0.18363	0.418035135	1.186822	1.62E-05	hypermethylated
cg18101138	0.13424	0.305558378	1.186633	0.000187	hypermethylated
cg16043144	0.1231	0.280062703	1.185919	0.000219	hypermethylated
cg10832108	0.14338	0.326052973	1.185263	1.73E-05	hypermethylated
cg00017489	0.19204	0.436630811	1.185007	5.58E-05	hypermethylated
cg00336320	0.15699	0.356836757	1.184592	5.43E-06	hypermethylated
cg09290600	0.15252	0.346635135	1.184419	4.58E-05	hypermethylated
cg17862113	0.1555	0.353294595	1.183957	9.07E-06	hypermethylated
cg00347369	0.13597	0.308903784	1.183869	8.84E-05	hypermethylated
cg22354782	0.14337	0.325672432	1.183678	0.00012	hypermethylated
cg14134497	0.15323	0.34806973	1.183678	0.000104	hypermethylated
cg18944010	0.17133	0.389168108	1.183616	9.57E-06	hypermethylated
cg02531437	0.15391	0.349593514	1.183591	1.50E-05	hypermethylated
cg01297721	0.15064	0.342075135	1.183208	0.000646	hypermethylated
cg04819096	0.12111	0.275014054	1.183187	2.32E-05	hypermethylated
cg24880701	0.17444	0.396037838	1.182907	6.15E-05	hypermethylated
cg00816770	0.16685	0.378805405	1.182905	5.73E-06	hypermethylated
cg10902717	0.13067	0.296651351	1.18284	7.20E-05	hypermethylated
cg15634877	0.16183	0.367384865	1.182813	5.32E-05	hypermethylated
cg26332310	0.17326	0.393268108	1.182575	4.53E-05	hypermethylated
cg04837104	0.15582	0.353588108	1.182189	1.58E-05	hypermethylated
cg19526659	0.14484	0.328636216	1.182031	1.75E-05	hypermethylated
cg19846314	0.18884	0.428447027	1.181952	7.65E-05	hypermethylated
cg10678266	0.189	0.428803784	1.181931	0.000129	hypermethylated
cg17076890	0.18448	0.418514595	1.181814	1.42E-05	hypermethylated
cg08195247	0.1489	0.337786486	1.181768	0.000327	hypermethylated
cg15871127	0.1323	0.300114054	1.181698	5.00E-05	hypermethylated
cg04365452	0.14588	0.330675676	1.180635	2.10E-05	hypermethylated
cg07498275	0.14023	0.317862162	1.180606	1.28E-05	hypermethylated
cg07338917	0.12526	0.283876757	1.180339	4.58E-05	hypermethylated
cg11379081	0.23928	0.542258378	1.180281	1.80E-05	hypermethylated
cg04971534	0.18833	0.426718919	1.180023	3.27E-05	hypermethylated
cg17951138	0.19435	0.440274595	1.179746	6.05E-06	hypermethylated
cg08983097	0.12385	0.280555135	1.17969	2.92E-05	hypermethylated
cg07664005	0.09273	0.210001081	1.179289	1.87E-05	hypermethylated
cg03593833	0.09595	0.217276216	1.179176	7.84E-05	hypermethylated
cg03285577	0.13187	0.298426486	1.178259	5.28E-06	hypermethylated
cg00741609	0.15515	0.351104865	1.178238	2.57E-05	hypermethylated
cg04453140	0.13486	0.305162703	1.178116	8.83E-06	hypermethylated
cg10981651	0.18663	0.422287027	1.178043	6.93E-06	hypermethylated
cg22536150	0.1763	0.398911892	1.178038	9.07E-06	hypermethylated
cg19728226	0.14245	0.322276216	1.177842	4.05E-05	hypermethylated
cg16005494	0.18878	0.426997838	1.177523	8.60E-06	hypermethylated

cg14871932	0.17903	0.404925946	1.177457	4.73E-06	hypermethylated
cg00247557	0.17566	0.397271892	1.177341	2.27E-05	hypermethylated
cg21346043	0.20913	0.472925405	1.177213	2.56E-06	hypermethylated
cg03190266	0.15653	0.353964324	1.177165	0.000232	hypermethylated
cg20229496	0.12282	0.277711892	1.177043	0.00028	hypermethylated
cg15069295	0.15719	0.355420541	1.177018	2.04E-05	hypermethylated
cg16081281	0.15622	0.353195676	1.176889	1.31E-05	hypermethylated
cg16301890	0.18686	0.422383243	1.176595	8.52E-05	hypermethylated
cg18556676	0.18616	0.420772432	1.176497	1.50E-05	hypermethylated
cg17098147	0.17246	0.389765946	1.176346	2.45E-05	hypermethylated
cg24643282	0.14111	0.318911892	1.176338	3.85E-05	hypermethylated
cg18264657	0.12963	0.292937297	1.176192	1.58E-05	hypermethylated
cg16092786	0.13455	0.304032973	1.176085	5.38E-05	hypermethylated
cg03671597	0.17258	0.389902162	1.175847	2.10E-05	hypermethylated
cg10140638	0.16592	0.374843784	1.175802	3.57E-05	hypermethylated
cg09409539	0.14239	0.321515676	1.175041	1.99E-05	hypermethylated
cg04023483	0.14158	0.319678919	1.175006	0.00012	hypermethylated
cg22995692	0.1721	0.388561622	1.174896	0.000142	hypermethylated
cg09302895	0.18071	0.407983243	1.174834	5.14E-06	hypermethylated
cg03740167	0.15372	0.347014054	1.174689	7.52E-06	hypermethylated
cg13544851	0.10244	0.231210811	1.17443	0.00028	hypermethylated
cg09049251	0.19187	0.433048649	1.1744	1.31E-05	hypermethylated
cg23683254	0.18104	0.40857027	1.174276	5.45E-05	hypermethylated
cg02940165	0.18255	0.411857838	1.173855	6.93E-06	hypermethylated
cg25024717	0.15848	0.357344324	1.173014	1.58E-05	hypermethylated
cg14189571	0.22127	0.498774054	1.172579	1.28E-05	hypermethylated
cg19122389	0.13997	0.315500541	1.172525	1.42E-05	hypermethylated
cg16440561	0.28311	0.638122162	1.17247	1.14E-05	hypermethylated
cg10327400	0.15862	0.357494595	1.172347	3.07E-05	hypermethylated
cg18366919	0.17002	0.383058378	1.17186	2.02E-05	hypermethylated
cg00916536	0.18619	0.419373514	1.17146	0.000338	hypermethylated
cg06498267	0.13836	0.31162	1.171361	0.000268	hypermethylated
cg18110483	0.18191	0.409622703	1.171071	4.58E-05	hypermethylated
cg17015844	0.12818	0.288587568	1.170838	1.50E-05	hypermethylated
cg09384610	0.19282	0.434112973	1.170816	5.38E-05	hypermethylated
cg18239431	0.20793	0.468128108	1.170805	2.15E-05	hypermethylated
cg00378510	0.17763	0.399765405	1.170278	0.000313	hypermethylated
cg16202340	0.15306	0.344400541	1.16999	3.95E-05	hypermethylated
cg01819137	0.1027	0.231005405	1.16949	6.30E-06	hypermethylated
cg04986616	0.10368	0.233147027	1.169102	0.00017	hypermethylated
cg23271831	0.13986	0.314415676	1.16869	0.000148	hypermethylated
cg11410718	0.19117	0.429756757	1.168664	4.15E-05	hypermethylated
cg16316519	0.14073	0.31630973	1.168408	7.32E-06	hypermethylated
cg09497201	0.18403	0.413566486	1.168178	2.85E-05	hypermethylated
cg08648727	0.17537	0.394012432	1.167839	2.92E-05	hypermethylated
cg12547166	0.17026	0.382521622	1.167802	1.75E-05	hypermethylated
cg17046577	0.13339	0.299680541	1.167775	0.000219	hypermethylated
cg15688551	0.12069	0.271143784	1.167752	2.74E-05	hypermethylated
cg08813062	0.17072	0.383536757	1.167733	1.84E-05	hypermethylated
cg02245378	0.14845	0.333490811	1.16767	1.09E-05	hypermethylated
cg21760402	0.14277	0.320662703	1.167364	0.000145	hypermethylated
cg25203481	0.21903	0.491887027	1.167199	1.07E-06	hypermethylated
cg10689404	0.22871	0.513589189	1.167095	7.93E-06	hypermethylated
cg02750098	0.11326	0.254302703	1.166908	4.64E-05	hypermethylated
cg07135032	0.1583	0.35542	1.166864	0.001	hypermethylated
cg05158615	0.1622	0.364169189	1.166835	4.47E-05	hypermethylated
cg04207084	0.19463	0.43697027	1.166801	4.01E-06	hypermethylated
cg02109405	0.13276	0.29804973	1.166733	0.000115	hypermethylated

cg16334250	0.11175	0.250870811	1.16667	6.62E-05	hypermethylated
cg02086467	0.15325	0.344021081	1.16661	5.89E-06	hypermethylated
cg07463541	0.26062	0.584997297	1.166482	9.11E-07	hypermethylated
cg11889068	0.11987	0.269059459	1.166454	2.67E-05	hypermethylated
cg04299439	0.17537	0.393621081	1.166406	1.41E-06	hypermethylated
cg13891978	0.13311	0.298738919	1.166266	0.000142	hypermethylated
cg12610471	0.19847	0.445379459	1.166114	1.28E-05	hypermethylated
cg04367345	0.13673	0.306814595	1.166037	5.79E-05	hypermethylated
cg24136205	0.18249	0.409490811	1.166014	3.04E-06	hypermethylated
cg17607298	0.20628	0.46286973	1.166002	3.39E-06	hypermethylated
cg26503073	0.18832	0.422552432	1.165944	4.48E-06	hypermethylated
cg24740868	0.21958	0.492617297	1.165721	2.95E-06	hypermethylated
cg09816507	0.13782	0.309166486	1.165599	9.16E-05	hypermethylated
cg27361370	0.19361	0.434304865	1.165555	6.93E-06	hypermethylated
cg00767010	0.18112	0.406214054	1.165294	0.000219	hypermethylated
cg05589845	0.13111	0.294011351	1.165094	0.000214	hypermethylated
cg08333333	0.18724	0.419874595	1.16507	4.24E-06	hypermethylated
cg11969556	0.13872	0.311035135	1.164902	1.94E-05	hypermethylated
cg23646776	0.10732	0.240621622	1.164847	0.000112	hypermethylated
cg27316886	0.17921	0.401702162	1.164475	1.60E-05	hypermethylated
cg07687119	0.1647	0.369128649	1.164283	2.51E-05	hypermethylated
cg19813025	0.15886	0.356036216	1.164268	9.82E-06	hypermethylated
cg04317399	0.17914	0.401335135	1.16372	4.73E-06	hypermethylated
cg23891996	0.12732	0.285238919	1.163712	6.78E-05	hypermethylated
cg00345443	0.16247	0.363941081	1.163532	0.000287	hypermethylated
cg11930400	0.11983	0.268422162	1.163515	4.31E-05	hypermethylated
cg18652346	0.20541	0.45999027	1.163097	1.89E-05	hypermethylated
cg21545390	0.1631	0.365214595	1.162988	1.40E-05	hypermethylated
cg02720618	0.13609	0.304644324	1.162565	0.001031	hypermethylated
cg16137147	0.18294	0.409491892	1.162464	0.000474	hypermethylated
cg05872306	0.13985	0.313030811	1.162424	1.75E-05	hypermethylated
cg17880014	0.15058	0.337020541	1.162306	2.18E-05	hypermethylated
cg10676084	0.1831	0.409623784	1.161668	2.85E-05	hypermethylated
cg10189695	0.16092	0.359960541	1.161495	0.00012	hypermethylated
cg22041640	0.16686	0.373137297	1.161068	2.91E-06	hypermethylated
cg04024095	0.12207	0.272952432	1.160941	0.000219	hypermethylated
cg07563793	0.1383	0.309131351	1.160419	1.68E-05	hypermethylated
cg16117799	0.17896	0.400001622	1.160369	2.10E-05	hypermethylated
cg11142389	0.22554	0.504074595	1.160254	2.85E-05	hypermethylated
cg10828337	0.134	0.299445946	1.160063	9.19E-06	hypermethylated
cg20303399	0.16394	0.36635027	1.160056	7.72E-06	hypermethylated
cg17750959	0.12026	0.268720541	1.15995	0.000417	hypermethylated
cg02350677	0.13661	0.305252432	1.15994	6.15E-05	hypermethylated
cg18694169	0.12997	0.290383243	1.15978	4.47E-05	hypermethylated
cg20747266	0.16119	0.35998	1.159155	1.73E-05	hypermethylated
cg11199046	0.16825	0.375692973	1.158948	1.35E-05	hypermethylated
cg06092815	0.16906	0.377390811	1.158524	6.01E-05	hypermethylated
cg10997479	0.16118	0.359708649	1.158156	4.80E-06	hypermethylated
cg14098847	0.20613	0.460011892	1.158117	1.35E-05	hypermethylated
cg18988498	0.22677	0.506062703	1.158086	5.14E-06	hypermethylated
cg21475402	0.20161	0.449883243	1.157983	1.54E-05	hypermethylated
cg03563630	0.16325	0.364249189	1.157843	6.38E-05	hypermethylated
cg02519751	0.27831	0.620951892	1.157789	7.12E-06	hypermethylated
cg07544187	0.18666	0.416452432	1.157739	0.000109	hypermethylated
cg09187933	0.13583	0.302997838	1.157505	0.000229	hypermethylated
cg22274813	0.18854	0.420534595	1.157354	1.75E-05	hypermethylated
cg22640819	0.15204	0.339027027	1.156949	0.000132	hypermethylated
cg06410057	0.16663	0.371530811	1.156834	6.62E-05	hypermethylated

cg05413872	0.11643	0.259598919	1.156822	6.78E-05	hypermethylated
cg11501236	0.1308	0.29163027	1.156778	0.000195	hypermethylated
cg18021368	0.15709	0.350224324	1.156688	4.05E-05	hypermethylated
cg26446499	0.16935	0.377550811	1.156663	1.99E-05	hypermethylated
cg13722123	0.1194	0.266171892	1.156555	3.67E-05	hypermethylated
cg06717850	0.20377	0.4542	1.156386	5.14E-06	hypermethylated
cg22795586	0.28338	0.631646486	1.156379	6.22E-06	hypermethylated
cg03951603	0.20279	0.451976757	1.156262	4.82E-05	hypermethylated
cg16651126	0.19527	0.435207568	1.156233	1.15E-05	hypermethylated
cg25106913	0.10727	0.239071351	1.156195	5.19E-05	hypermethylated
cg04598121	0.1787	0.398252432	1.156144	9.69E-06	hypermethylated
cg04611203	0.15336	0.341774595	1.156123	4.70E-05	hypermethylated
cg03193589	0.18896	0.42109027	1.156049	3.23E-05	hypermethylated
cg01532436	0.19381	0.431850811	1.15589	0.000931	hypermethylated
cg22812955	0.13572	0.302298919	1.155342	1.67E-06	hypermethylated
cg24924958	0.17751	0.395376757	1.155328	4.05E-05	hypermethylated
cg03998104	0.13548	0.301757297	1.155309	4.67E-06	hypermethylated
cg24594454	0.15737	0.350495676	1.155236	0.000327	hypermethylated
cg01497613	0.14281	0.318057838	1.155192	1.39E-05	hypermethylated
cg11199713	0.15676	0.349080541	1.155002	8.15E-06	hypermethylated
cg12382153	0.15698	0.349567568	1.154991	0.000163	hypermethylated
cg01856162	0.20609	0.458917838	1.154961	6.31E-05	hypermethylated
cg25353171	0.1838	0.409258919	1.154877	1.73E-05	hypermethylated
cg00563824	0.21486	0.478361081	1.154703	0.000174	hypermethylated
cg03828874	0.15739	0.350407568	1.15469	7.41E-06	hypermethylated
cg05965288	0.15847	0.352799459	1.154639	3.67E-05	hypermethylated
cg02976699	0.10807	0.240574054	1.154515	0.000174	hypermethylated
cg24249411	0.1539	0.342583243	1.154461	1.06E-05	hypermethylated
cg17477162	0.16439	0.36592	1.154406	0.000219	hypermethylated
cg10588470	0.10856	0.241602703	1.154144	8.42E-05	hypermethylated
cg01944655	0.1716	0.381824865	1.153862	0.000142	hypermethylated
cg19851594	0.14085	0.313389189	1.153796	0.000221	hypermethylated
cg04778012	0.22399	0.498371351	1.153787	1.54E-05	hypermethylated
cg10097295	0.12304	0.273727568	1.153613	0.000265	hypermethylated
cg21564242	0.09341	0.207798378	1.153535	1.35E-05	hypermethylated
cg26257822	0.13748	0.305753514	1.153147	2.32E-05	hypermethylated
cg04851268	0.18741	0.416791351	1.153127	5.86E-05	hypermethylated
cg00914726	0.18398	0.408966486	1.152434	4.64E-05	hypermethylated
cg09866983	0.16094	0.357747027	1.152417	1.58E-05	hypermethylated
cg27234067	0.20771	0.461642162	1.152204	1.46E-05	hypermethylated
cg21355828	0.22178	0.492883243	1.152117	4.48E-06	hypermethylated
cg00197517	0.15483	0.344	1.151724	0.000262	hypermethylated
cg25744613	0.16536	0.367362162	1.151593	0.000245	hypermethylated
cg10603004	0.16439	0.365174054	1.151462	1.15E-05	hypermethylated
cg12544191	0.19596	0.435098378	1.150782	3.69E-06	hypermethylated
cg23619399	0.21282	0.472529189	1.15077	3.81E-05	hypermethylated
cg07950000	0.12174	0.270285946	1.150683	0.0002	hypermethylated
cg14421192	0.14803	0.328638378	1.150611	1.25E-05	hypermethylated
cg16821446	0.13752	0.305304324	1.150607	5.35E-06	hypermethylated
cg10717463	0.15169	0.336698919	1.150333	6.57E-06	hypermethylated
cg23096689	0.14748	0.327255135	1.149897	4.82E-05	hypermethylated
cg12385599	0.13582	0.301370811	1.149844	0.000408	hypermethylated
cg04088697	0.16007	0.355166486	1.149793	1.04E-05	hypermethylated
cg16650901	0.13708	0.304131351	1.149676	6.15E-05	hypermethylated
cg08196032	0.15493	0.343686486	1.149477	7.65E-05	hypermethylated
cg10841552	0.15358	0.340651351	1.149306	1.58E-05	hypermethylated
cg27302539	0.14263	0.316302703	1.149028	0.000219	hypermethylated
cg01106926	0.17972	0.398413514	1.148516	6.22E-06	hypermethylated

cg12173558	0.16289	0.36109027	1.148462	3.90E-05	hypermethylated
cg16498194	0.20999	0.465412973	1.148191	3.12E-06	hypermethylated
cg23954268	0.12857	0.284947027	1.14814	2.10E-05	hypermethylated
cg00745389	0.14468	0.320558378	1.147722	5.72E-05	hypermethylated
cg18063312	0.19897	0.440838378	1.147699	3.00E-05	hypermethylated
cg25097497	0.13359	0.295981622	1.147696	4.15E-05	hypermethylated
cg01295392	0.20316	0.450054595	1.147484	5.00E-06	hypermethylated
cg14512346	0.13094	0.29006	1.147445	8.63E-05	hypermethylated
cg25928819	0.18039	0.399418378	1.146781	1.64E-05	hypermethylated
cg16419235	0.15824	0.350345946	1.146666	2.78E-05	hypermethylated
cg04996277	0.14896	0.329781622	1.146586	6.01E-05	hypermethylated
cg27659049	0.26037	0.57636973	1.146431	8.37E-06	hypermethylated
cg10785263	0.17044	0.377283243	1.146384	4.70E-05	hypermethylated
cg24100636	0.17686	0.391352432	1.145861	0.000313	hypermethylated
cg22392666	0.15045	0.332890811	1.145765	0.000506	hypermethylated
cg00258597	0.1264	0.279614054	1.14544	3.19E-05	hypermethylated
cg18673377	0.16755	0.370628108	1.145381	1.71E-05	hypermethylated
cg14425564	0.17195	0.380263243	1.145009	0.000417	hypermethylated
cg22633280	0.15717	0.347555676	1.144918	0.000626	hypermethylated
cg24061208	0.16753	0.370452432	1.144869	0.000154	hypermethylated
cg11044575	0.15719	0.347547027	1.144699	4.05E-05	hypermethylated
cg01213381	0.15615	0.345091351	1.144046	0.000102	hypermethylated
cg06634914	0.18131	0.400643784	1.143862	7.29E-05	hypermethylated
cg06001519	0.20976	0.463476216	1.143756	1.66E-05	hypermethylated
cg09403666	0.21214	0.468650811	1.143497	3.00E-05	hypermethylated
cg04630271	0.12676	0.280028649	1.143475	2.05E-06	hypermethylated
cg09565079	0.11889	0.262628108	1.143394	7.65E-05	hypermethylated
cg19819837	0.13325	0.294331351	1.143306	7.62E-06	hypermethylated
cg24719321	0.19139	0.422754054	1.143303	3.49E-06	hypermethylated
cg26465391	0.18777	0.414720541	1.143173	1.66E-05	hypermethylated
cg07315858	0.13619	0.300765946	1.14302	1.35E-05	hypermethylated
cg04445871	0.13935	0.307463243	1.141701	1.89E-05	hypermethylated
cg04553690	0.18263	0.402897297	1.141488	2.23E-06	hypermethylated
cg08136772	0.19032	0.419857297	1.141472	1.12E-05	hypermethylated
cg03053579	0.14708	0.324371892	1.141048	3.57E-05	hypermethylated
cg09415754	0.18405	0.405840541	1.140815	1.45E-06	hypermethylated
cg16325502	0.14608	0.322111892	1.140803	1.39E-05	hypermethylated
cg14654613	0.18647	0.411165946	1.140777	6.95E-05	hypermethylated
cg11228126	0.14312	0.315512973	1.140474	1.89E-05	hypermethylated
cg09731996	0.15392	0.339304324	1.140399	6.54E-05	hypermethylated
cg13946538	0.14889	0.328196757	1.140314	1.54E-05	hypermethylated
cg09614300	0.16813	0.370543784	1.140067	2.32E-05	hypermethylated
cg26520012	0.16325	0.359745405	1.139893	0.00035	hypermethylated
cg03355526	0.16538	0.364430811	1.13986	1.33E-05	hypermethylated
cg21269934	0.12156	0.26784973	1.139755	0.000464	hypermethylated
cg16151151	0.13472	0.296841081	1.139727	5.79E-05	hypermethylated
cg00511674	0.15609	0.343907568	1.139643	0.000147	hypermethylated
cg22511262	0.11091	0.244331892	1.139453	3.67E-05	hypermethylated
cg13478928	0.17436	0.384051892	1.139232	2.13E-05	hypermethylated
cg01914153	0.11037	0.243043784	1.138868	1.64E-05	hypermethylated
cg00794881	0.11574	0.254838919	1.138698	7.65E-05	hypermethylated
cg01573321	0.2108	0.464098378	1.138556	0.000588	hypermethylated
cg18470295	0.14156	0.311636216	1.138449	0.00017	hypermethylated
cg07099161	0.12017	0.264544865	1.138436	1.63E-06	hypermethylated
cg03400421	0.16721	0.368095676	1.13842	2.10E-05	hypermethylated
cg20403938	0.17038	0.375035135	1.13827	5.06E-05	hypermethylated
cg04571163	0.13952	0.307094054	1.138209	1.17E-05	hypermethylated
cg08332074	0.19284	0.424446486	1.138178	6.46E-05	hypermethylated

cg24549277	0.19851	0.436772973	1.137672	1.09E-05	hypermethylated
cg02723395	0.16329	0.359247568	1.137542	2.10E-05	hypermethylated
cg09180848	0.156	0.343180541	1.137422	2.21E-05	hypermethylated
cg06410537	0.18531	0.407655676	1.13741	4.73E-06	hypermethylated
cg04339613	0.16344	0.359531351	1.137356	1.09E-05	hypermethylated
cg16135698	0.13854	0.304737838	1.137266	3.40E-05	hypermethylated
cg10237044	0.11953	0.262917297	1.137236	1.64E-05	hypermethylated
cg19224201	0.15178	0.333825946	1.137114	0.000142	hypermethylated
cg15431544	0.14447	0.317735135	1.137055	7.62E-06	hypermethylated
cg06697536	0.16058	0.353111892	1.136833	0.000517	hypermethylated
cg21914290	0.23851	0.524464865	1.136796	3.67E-05	hypermethylated
cg00893471	0.1399	0.307625946	1.136781	9.60E-05	hypermethylated
cg00831710	0.20936	0.460218919	1.136334	1.14E-05	hypermethylated
cg14894848	0.18542	0.407585405	1.136306	8.26E-06	hypermethylated
cg14269973	0.15687	0.344785405	1.136129	0.000166	hypermethylated
cg19411025	0.14407	0.316607568	1.135926	2.21E-05	hypermethylated
cg00500229	0.17538	0.385366486	1.135747	8.84E-05	hypermethylated
cg27232145	0.17442	0.383214054	1.135585	1.48E-05	hypermethylated
cg14810013	0.12349	0.271295135	1.135469	0.000145	hypermethylated
cg08620606	0.11839	0.260088108	1.135453	3.95E-05	hypermethylated
cg00911351	0.17885	0.392824865	1.135136	1.33E-06	hypermethylated
cg18335607	0.12197	0.267858919	1.134947	0.000224	hypermethylated
cg12665460	0.15857	0.348091351	1.134346	1.52E-05	hypermethylated
cg22287492	0.1591	0.349237838	1.134276	0.000191	hypermethylated
cg12699371	0.1315	0.28865027	1.13426	7.74E-05	hypermethylated
cg13193455	0.15577	0.341865405	1.134011	3.32E-05	hypermethylated
cg03711485	0.25409	0.557596216	1.133881	3.49E-06	hypermethylated
cg25271672	0.1192	0.261555135	1.133731	2.10E-06	hypermethylated
cg13365752	0.15199	0.333403784	1.133294	0.00012	hypermethylated
cg06108782	0.13211	0.289788649	1.133261	7.83E-06	hypermethylated
cg23917057	0.14728	0.322912432	1.132581	1.40E-05	hypermethylated
cg15007156	0.18351	0.402294595	1.132394	8.22E-05	hypermethylated
cg24842733	0.15069	0.330312432	1.132248	0.000142	hypermethylated
cg16038120	0.21142	0.463358378	1.132017	0.000174	hypermethylated
cg08400962	0.18881	0.413797297	1.131989	1.97E-05	hypermethylated
cg27112264	0.14708	0.322254054	1.131597	0.000109	hypermethylated
cg12432010	0.16893	0.370005405	1.131121	8.63E-05	hypermethylated
cg05842480	0.13889	0.304205946	1.131106	0.000207	hypermethylated
cg20792062	0.17686	0.387331351	1.13096	5.32E-05	hypermethylated
cg12545687	0.16656	0.364722162	1.130756	3.15E-05	hypermethylated
cg14161359	0.17946	0.392961622	1.130726	6.54E-05	hypermethylated
cg21328033	0.19836	0.434345946	1.130723	2.39E-05	hypermethylated
cg26425305	0.18284	0.400177838	1.13006	3.35E-06	hypermethylated
cg08697503	0.13031	0.285103243	1.129537	4.47E-05	hypermethylated
cg04542030	0.13303	0.291028108	1.129407	7.20E-05	hypermethylated
cg20270188	0.18611	0.407138378	1.129364	7.52E-06	hypermethylated
cg02497758	0.14651	0.320494054	1.129298	0.000226	hypermethylated
cg09169953	0.12177	0.266352973	1.129181	1.26E-05	hypermethylated
cg27659903	0.15859	0.34689027	1.129178	8.03E-05	hypermethylated
cg19215671	0.15628	0.341825946	1.129129	3.67E-05	hypermethylated
cg08239858	0.21228	0.4642	1.128778	6.39E-06	hypermethylated
cg05143633	0.14755	0.322638378	1.128714	3.15E-05	hypermethylated
cg12857945	0.16456	0.359825405	1.128683	2.10E-05	hypermethylated
cg02757432	0.17203	0.376040541	1.128228	1.68E-05	hypermethylated
cg03511041	0.15404	0.336664865	1.128008	8.84E-05	hypermethylated
cg14112075	0.13324	0.291175135	1.12786	0.000117	hypermethylated
cg15444648	0.14201	0.310322703	1.127777	4.58E-05	hypermethylated
cg18298920	0.20307	0.443713514	1.127651	4.36E-06	hypermethylated

cg23397578	0.11454	0.250271351	1.127642	0.00017	hypermethylated
cg06151243	0.16862	0.368372973	1.127392	1.25E-05	hypermethylated
cg20703997	0.20002	0.436903784	1.127171	1.66E-05	hypermethylated
cg10397765	0.18683	0.407970811	1.12674	0.00064	hypermethylated
cg13794993	0.14371	0.313803243	1.1267	1.15E-05	hypermethylated
cg14323675	0.18003	0.393071892	1.126556	2.39E-05	hypermethylated
cg12440258	0.17742	0.38732973	1.126394	2.27E-05	hypermethylated
cg24813176	0.14988	0.327197838	1.126355	9.72E-05	hypermethylated
cg10900455	0.17138	0.374048108	1.126025	1.18E-05	hypermethylated
cg25214093	0.12783	0.278925946	1.125656	8.84E-05	hypermethylated
cg11220245	0.19862	0.433362162	1.125562	8.60E-06	hypermethylated
cg20711812	0.2067	0.450984865	1.125541	8.60E-06	hypermethylated
cg14872952	0.17842	0.389227568	1.125337	0.000382	hypermethylated
cg07423205	0.1621	0.353608108	1.125267	8.95E-06	hypermethylated
cg20359994	0.15893	0.346615676	1.124945	2.32E-05	hypermethylated
cg18592174	0.16518	0.360223784	1.124854	3.30E-06	hypermethylated
cg13102079	0.14539	0.317008649	1.124594	8.84E-05	hypermethylated
cg10286380	0.17828	0.388703784	1.124526	3.23E-05	hypermethylated
cg27265170	0.17356	0.378350811	1.12429	1.75E-05	hypermethylated
cg04874129	0.14492	0.315908649	1.124251	3.15E-05	hypermethylated
cg06551997	0.16641	0.362719459	1.124112	0.000148	hypermethylated
cg08475379	0.1615	0.352001081	1.124046	1.42E-05	hypermethylated
cg08815081	0.17903	0.390202703	1.124022	8.60E-06	hypermethylated
cg26647617	0.14677	0.31989027	1.12402	1.06E-05	hypermethylated
cg07441518	0.23387	0.509685405	1.1239	7.20E-07	hypermethylated
cg05639205	0.18542	0.403848649	1.123018	1.45E-06	hypermethylated
cg19248676	0.1227	0.267237297	1.122986	6.62E-05	hypermethylated
cg20300343	0.22931	0.499390811	1.12287	1.09E-05	hypermethylated
cg10456990	0.14158	0.308329189	1.122854	8.71E-06	hypermethylated
cg21908557	0.15202	0.331026486	1.122686	0.000182	hypermethylated
cg05652533	0.16464	0.358432973	1.122388	2.27E-05	hypermethylated
cg16424078	0.15671	0.341141622	1.122274	2.15E-05	hypermethylated
cg05678749	0.19557	0.425718378	1.122214	3.04E-05	hypermethylated
cg13186884	0.14369	0.312775676	1.122169	3.44E-05	hypermethylated
cg13802013	0.19032	0.414262162	1.122117	2.79E-06	hypermethylated
cg04606861	0.1711	0.372396757	1.122001	1.62E-05	hypermethylated
cg20885782	0.17196	0.374248649	1.121924	1.39E-05	hypermethylated
cg17446371	0.10498	0.228438919	1.121694	4.20E-05	hypermethylated
cg15837212	0.1653	0.359687027	1.121655	8.15E-06	hypermethylated
cg21287054	0.1732	0.376872973	1.121639	0.000733	hypermethylated
cg23475371	0.11037	0.24015027	1.121589	5.38E-05	hypermethylated
cg24767540	0.16691	0.363128649	1.12141	0.000107	hypermethylated
cg24080008	0.22857	0.497125405	1.120974	4.48E-06	hypermethylated
cg23897749	0.16106	0.350258378	1.120821	3.76E-05	hypermethylated
cg19101547	0.14932	0.324639459	1.120431	0.000172	hypermethylated
cg15145341	0.23494	0.510716757	1.120231	1.11E-05	hypermethylated
cg04974130	0.1305	0.283641081	1.120017	1.38E-05	hypermethylated
cg02161900	0.1549	0.336659459	1.119953	3.36E-05	hypermethylated
cg06563089	0.23057	0.501118378	1.119947	2.05E-06	hypermethylated
cg06959142	0.13957	0.303304324	1.119777	1.54E-05	hypermethylated
cg11571585	0.16055	0.348856757	1.119612	0.000931	hypermethylated
cg09071155	0.16534	0.359257838	1.119584	2.15E-05	hypermethylated
cg09234616	0.20387	0.442857297	1.119192	0.000109	hypermethylated
cg03586879	0.13746	0.298578919	1.1191	3.57E-05	hypermethylated
cg08078137	0.10053	0.218277838	1.11854	6.95E-05	hypermethylated
cg25318296	0.14216	0.308666486	1.118533	0.000112	hypermethylated
cg24603408	0.20797	0.451504324	1.118364	9.27E-05	hypermethylated
cg15105206	0.12344	0.267987027	1.118353	1.40E-05	hypermethylated

cg14794043	0.15818	0.343346486	1.118098	9.05E-05	hypermethylated
cg08837627	0.1944	0.421933514	1.117987	4.70E-05	hypermethylated
cg20402783	0.21149	0.458971892	1.117816	0.000115	hypermethylated
cg18111119	0.14756	0.320229189	1.117803	3.00E-05	hypermethylated
cg18277979	0.16271	0.353090811	1.117736	5.06E-05	hypermethylated
cg04355159	0.12764	0.276972973	1.117665	3.49E-05	hypermethylated
cg06163425	0.18789	0.407697838	1.117612	4.70E-05	hypermethylated
cg05131483	0.19498	0.423072973	1.11758	1.46E-05	hypermethylated
cg06383163	0.19926	0.432318919	1.117444	4.70E-05	hypermethylated
cg09141965	0.19294	0.418581081	1.117355	8.37E-06	hypermethylated
cg24072202	0.16939	0.367365405	1.116867	9.82E-06	hypermethylated
cg09168808	0.2198	0.476631892	1.116684	5.89E-06	hypermethylated
cg11169641	0.1493	0.323715135	1.116511	2.18E-05	hypermethylated
cg20887073	0.15519	0.336453514	1.116372	8.60E-06	hypermethylated
cg14664621	0.13707	0.297151351	1.116285	5.00E-05	hypermethylated
cg00811065	0.20349	0.441090811	1.116118	1.62E-05	hypermethylated
cg09498572	0.1632	0.353695676	1.115868	4.64E-05	hypermethylated
cg19034038	0.15685	0.339885405	1.115663	6.01E-05	hypermethylated
cg20731937	0.1914	0.414734054	1.115596	0.000262	hypermethylated
cg01294808	0.26359	0.571041622	1.1153	9.66E-07	hypermethylated
cg26267388	0.1614	0.349622162	1.115156	8.83E-06	hypermethylated
cg07388347	0.1927	0.417385946	1.115025	2.27E-05	hypermethylated
cg02722596	0.24264	0.525443243	1.114718	3.00E-05	hypermethylated
cg04745161	0.13838	0.299501622	1.113928	9.95E-05	hypermethylated
cg03697918	0.20697	0.447768649	1.113332	4.58E-05	hypermethylated
cg14724471	0.16945	0.366539459	1.113109	9.31E-06	hypermethylated
cg14554491	0.15542	0.336177838	1.113052	0.000268	hypermethylated
cg00457403	0.203	0.439091351	1.113041	6.78E-05	hypermethylated
cg03943081	0.14071	0.304342703	1.112972	2.92E-05	hypermethylated
cg03086857	0.1267	0.274038919	1.112964	0.000256	hypermethylated
cg26106948	0.17218	0.37236	1.112781	0.000126	hypermethylated
cg26195829	0.18412	0.398073514	1.112389	0.000283	hypermethylated
cg11511795	0.15415	0.33325027	1.112271	9.60E-05	hypermethylated
cg09784934	0.14491	0.313222703	1.112032	1.73E-05	hypermethylated
cg21277243	0.15349	0.331764324	1.112014	1.52E-05	hypermethylated
cg14270687	0.20855	0.450557297	1.111317	1.46E-05	hypermethylated
cg06079106	0.16441	0.35516973	1.111211	1.87E-05	hypermethylated
cg11638298	0.1703	0.367892432	1.111206	1.18E-05	hypermethylated
cg15473084	0.14495	0.31307027	1.110931	1.89E-05	hypermethylated
cg01503065	0.14972	0.323314054	1.110669	7.72E-06	hypermethylated
cg12884406	0.11069	0.239010811	1.110551	8.63E-05	hypermethylated
cg09275667	0.13041	0.281571892	1.110449	0.0003	hypermethylated
cg06132283	0.11156	0.240864324	1.110401	0.000104	hypermethylated
cg22752533	0.20888	0.450971351	1.110361	3.00E-05	hypermethylated
cg05433039	0.10801	0.233169189	1.110212	0.000204	hypermethylated
cg06365303	0.21319	0.460193514	1.110101	9.57E-06	hypermethylated
cg14395298	0.24266	0.523772432	1.110004	9.16E-05	hypermethylated
cg23250574	0.16422	0.354455135	1.109973	0.000123	hypermethylated
cg08036309	0.21528	0.464623243	1.109847	9.82E-06	hypermethylated
cg25121513	0.17953	0.387443784	1.109762	1.71E-05	hypermethylated
cg04549162	0.17322	0.373795676	1.109644	4.26E-05	hypermethylated
cg05942128	0.14528	0.313447568	1.109388	5.07E-06	hypermethylated
cg17617843	0.19171	0.413603243	1.109322	0.000234	hypermethylated
cg11909748	0.21121	0.455671351	1.109316	8.37E-06	hypermethylated
cg05843457	0.19496	0.420588649	1.109232	0.000426	hypermethylated
cg23395449	0.15049	0.324623243	1.109099	7.12E-05	hypermethylated
cg09408098	0.13786	0.297262162	1.108532	0.000142	hypermethylated
cg08677617	0.18895	0.407374595	1.108351	0.000147	hypermethylated

cg17833265	0.18486	0.398508649	1.108178	9.82E-06	hypermethylated
cg07116997	0.14773	0.318461622	1.108157	5.58E-05	hypermethylated
cg03314029	0.20738	0.446972973	1.107911	0.001052	hypermethylated
cg07867924	0.08054	0.173557838	1.107639	1.94E-05	hypermethylated
cg14958635	0.1784	0.384424324	1.107584	4.58E-05	hypermethylated
cg05422049	0.18584	0.400449189	1.107558	5.25E-05	hypermethylated
cg06275859	0.18594	0.400608649	1.107356	7.93E-06	hypermethylated
cg20725013	0.16332	0.351869189	1.107338	3.15E-05	hypermethylated
cg08977371	0.13729	0.295720541	1.107008	1.12E-05	hypermethylated
cg02006615	0.16148	0.347782703	1.106831	9.49E-05	hypermethylated
cg01007828	0.14952	0.322014595	1.106788	1.46E-05	hypermethylated
cg21643178	0.19324	0.416030811	1.106297	1.93E-06	hypermethylated
cg26236177	0.1988	0.427968108	1.106186	0.000126	hypermethylated
cg13777292	0.1887	0.40618	1.106025	0.000174	hypermethylated
cg08813349	0.13651	0.293834054	1.105995	7.93E-05	hypermethylated
cg00553149	0.17222	0.370648108	1.105797	9.19E-06	hypermethylated
cg13197216	0.13785	0.296665405	1.105737	6.15E-05	hypermethylated
cg07138452	0.13191	0.283835135	1.105499	0.000268	hypermethylated
cg13705391	0.14847	0.319456216	1.105447	0.000176	hypermethylated
cg00319168	0.15388	0.331042162	1.105209	4.76E-05	hypermethylated
cg11111460	0.17769	0.382148108	1.104769	7.84E-05	hypermethylated
cg12718339	0.13489	0.290085405	1.104694	2.54E-05	hypermethylated
cg07066550	0.17517	0.376657838	1.104499	0.000135	hypermethylated
cg14273116	0.16037	0.344822703	1.10445	4.60E-06	hypermethylated
cg09896412	0.14359	0.308741622	1.104445	0.000268	hypermethylated
cg18799510	0.12175	0.261752973	1.104284	2.10E-05	hypermethylated
cg14994060	0.14816	0.318498378	1.10413	4.15E-05	hypermethylated
cg06695663	0.16785	0.36079027	1.103988	0.000163	hypermethylated
cg03160135	0.1298	0.278997838	1.103964	2.32E-05	hypermethylated
cg02800810	0.19249	0.413706486	1.103824	9.31E-06	hypermethylated
cg18232816	0.13976	0.300308108	1.103492	6.78E-05	hypermethylated
cg19063061	0.1869	0.401431351	1.102887	0.00039	hypermethylated
cg17795158	0.1758	0.377531351	1.102661	0.000143	hypermethylated
cg18181201	0.17432	0.374252432	1.102274	7.65E-05	hypermethylated
cg15989068	0.18003	0.386468649	1.102114	1.06E-05	hypermethylated
cg01287975	0.1938	0.415963784	1.101889	0.000224	hypermethylated
cg04849842	0.2105	0.451767568	1.10176	0.000306	hypermethylated
cg13413955	0.21753	0.466697297	1.101273	4.48E-06	hypermethylated
cg03609666	0.16903	0.362634054	1.101235	0.000435	hypermethylated
cg02332525	0.17431	0.373934595	1.101131	7.62E-06	hypermethylated
cg14552379	0.14657	0.314418378	1.101096	0.000129	hypermethylated
cg16642791	0.14095	0.302329189	1.100937	0.000129	hypermethylated
cg04694035	0.14108	0.302553514	1.100677	1.12E-05	hypermethylated
cg15724256	0.19001	0.407445946	1.100533	3.32E-05	hypermethylated
cg20139706	0.22135	0.474589189	1.10035	2.18E-05	hypermethylated
cg08550101	0.16276	0.348720541	1.099325	3.59E-06	hypermethylated
cg07362168	0.16043	0.343623243	1.098884	0.000293	hypermethylated
cg08260398	0.12321	0.263893514	1.098837	5.65E-05	hypermethylated
cg22861561	0.16009	0.342863784	1.098752	0.000207	hypermethylated
cg10103266	0.15194	0.325374595	1.0986	5.58E-05	hypermethylated
cg21424940	0.18996	0.406682162	1.098206	6.05E-06	hypermethylated
cg01874877	0.17505	0.374750811	1.098165	2.10E-05	hypermethylated
cg10095577	0.1217	0.260519459	1.098062	9.72E-05	hypermethylated
cg14228146	0.19693	0.421475676	1.097766	5.35E-06	hypermethylated
cg02341815	0.13284	0.284285946	1.097653	1.21E-05	hypermethylated
cg02915593	0.13551	0.289988108	1.097594	8.42E-05	hypermethylated
cg21851672	0.22422	0.479768649	1.097424	6.39E-06	hypermethylated
cg01270593	0.1427	0.305298919	1.097237	4.20E-05	hypermethylated

cg13884568	0.13698	0.293001081	1.096941	0.0002	hypermethylated
cg24277791	0.16938	0.362281622	1.096848	3.32E-05	hypermethylated
cg10659886	0.22935	0.490362703	1.096298	5.00E-06	hypermethylated
cg03149432	0.16901	0.361324324	1.096186	2.78E-05	hypermethylated
cg03476673	0.1482	0.316767568	1.095879	2.27E-05	hypermethylated
cg02431597	0.14417	0.308124324	1.095742	1.52E-05	hypermethylated
cg22459630	0.18859	0.403040541	1.095672	0.000495	hypermethylated
cg14582400	0.23859	0.509888649	1.095649	5.28E-06	hypermethylated
cg01031101	0.15779	0.337175135	1.095492	9.60E-05	hypermethylated
cg03386144	0.11772	0.251549189	1.095481	0.000607	hypermethylated
cg13338798	0.18261	0.390130811	1.095192	1.37E-06	hypermethylated
cg26220673	0.22821	0.487477297	1.094973	1.35E-05	hypermethylated
cg14473924	0.19602	0.418515676	1.094281	0.000251	hypermethylated
cg05965863	0.13874	0.29615027	1.093946	2.51E-05	hypermethylated
cg17780246	0.22813	0.486947027	1.093909	9.07E-06	hypermethylated
cg13844402	0.1657	0.353674054	1.093847	6.05E-06	hypermethylated
cg16105687	0.2081	0.444155135	1.093787	1.75E-05	hypermethylated
cg05312084	0.12607	0.26904973	1.093648	5.73E-06	hypermethylated
cg00182639	0.23077	0.492402162	1.093381	1.37E-06	hypermethylated
cg20963741	0.19824	0.422870811	1.092969	9.49E-05	hypermethylated
cg18752880	0.14284	0.304694054	1.092961	8.22E-05	hypermethylated
cg01866606	0.13531	0.288591892	1.092762	0.000613	hypermethylated
cg20430077	0.13879	0.296013514	1.092759	2.51E-05	hypermethylated
cg02316596	0.18071	0.385348108	1.092486	5.45E-05	hypermethylated
cg13188098	0.17059	0.363677838	1.092128	6.08E-05	hypermethylated
cg10672201	0.17631	0.375857838	1.092073	4.82E-05	hypermethylated
cg19179801	0.1536	0.32744	1.092052	0.000204	hypermethylated
cg21477176	0.23509	0.500949189	1.091451	1.42E-05	hypermethylated
cg23303408	0.1214	0.258603784	1.090975	1.40E-05	hypermethylated
cg21942490	0.14317	0.304858919	1.090413	1.58E-05	hypermethylated
cg11080192	0.07275	0.154874054	1.090076	0.000232	hypermethylated
cg27599792	0.18931	0.403003784	1.090043	5.73E-06	hypermethylated
cg03940047	0.19128	0.407178919	1.089977	1.33E-05	hypermethylated
cg20260127	0.20584	0.438089189	1.089701	1.54E-05	hypermethylated
cg10783469	0.17423	0.370734595	1.089394	0.000335	hypermethylated
cg07450037	0.18552	0.394756757	1.089389	0.000104	hypermethylated
cg26130488	0.19579	0.416492973	1.088985	6.62E-05	hypermethylated
cg12743978	0.16808	0.357511351	1.088841	3.76E-05	hypermethylated
cg06437464	0.1427	0.303520541	1.088809	6.15E-05	hypermethylated
cg08610862	0.21741	0.462418919	1.088782	0.000408	hypermethylated
cg17099568	0.16203	0.344627568	1.088777	3.32E-05	hypermethylated
cg10102762	0.11427	0.243028108	1.088676	3.32E-05	hypermethylated
cg19305488	0.1323	0.281333514	1.088468	0.000646	hypermethylated
cg02761376	0.108	0.229652432	1.088421	1.28E-05	hypermethylated
cg25116269	0.07649	0.162619459	1.088157	6.62E-05	hypermethylated
cg14262937	0.16048	0.341166486	1.088082	1.66E-05	hypermethylated
cg03541338	0.19892	0.422802703	1.087796	5.58E-05	hypermethylated
cg00901320	0.15932	0.338578919	1.087565	0.000129	hypermethylated
cg00792849	0.15737	0.334397838	1.087405	2.54E-05	hypermethylated
cg13019143	0.17216	0.36582	1.087384	6.57E-06	hypermethylated
cg09767822	0.22941	0.48731027	1.086912	1.09E-05	hypermethylated
cg01427458	0.16345	0.347101081	1.086506	4.76E-05	hypermethylated
cg27259320	0.17161	0.364324865	1.086092	6.93E-06	hypermethylated
cg26330116	0.1872	0.397403784	1.086025	0.000242	hypermethylated
cg17586860	0.17596	0.373473514	1.085758	0.000445	hypermethylated
cg27050153	0.21737	0.461305405	1.085569	1.84E-05	hypermethylated
cg15698842	0.15864	0.33665027	1.085494	0.00039	hypermethylated
cg08109815	0.16052	0.340623243	1.085424	2.64E-05	hypermethylated

cg03612357	0.25696	0.545253514	1.085383	0.000107	hypermethylated
cg27634344	0.18342	0.389161081	1.085216	1.99E-05	hypermethylated
cg19029904	0.1794	0.38062973	1.085208	7.12E-05	hypermethylated
cg00489401	0.23713	0.503096757	1.085158	1.31E-05	hypermethylated
cg26473844	0.11049	0.23441027	1.08512	8.95E-06	hypermethylated
cg04735310	0.16178	0.343205946	1.085041	1.58E-06	hypermethylated
cg21153898	0.1735	0.368013514	1.084823	0.000506	hypermethylated
cg07336617	0.18971	0.402341081	1.084623	3.00E-05	hypermethylated
cg02320543	0.19442	0.412321622	1.084593	3.00E-05	hypermethylated
cg06667761	0.16982	0.360140541	1.084554	1.62E-05	hypermethylated
cg07609862	0.18356	0.389264865	1.0845	2.10E-05	hypermethylated
cg03972665	0.16279	0.345197297	1.084409	1.84E-05	hypermethylated
cg24801123	0.15346	0.325402703	1.084364	3.76E-05	hypermethylated
cg21113740	0.21211	0.449708649	1.084178	7.93E-06	hypermethylated
cg10640845	0.19765	0.418928649	1.083757	1.38E-05	hypermethylated
cg17070988	0.19398	0.411072973	1.083487	1.31E-05	hypermethylated
cg06650419	0.11673	0.247367568	1.083481	0.000126	hypermethylated
cg15508809	0.17496	0.370691351	1.083193	3.76E-05	hypermethylated
cg21413325	0.13244	0.280569189	1.083018	8.22E-05	hypermethylated
cg15618978	0.18699	0.396121081	1.08298	5.58E-06	hypermethylated
cg15032098	0.16498	0.349479459	1.082917	8.63E-05	hypermethylated
cg06193578	0.17272	0.365845405	1.082799	8.63E-05	hypermethylated
cg18938204	0.19002	0.402481081	1.08277	0.000111	hypermethylated
cg06694734	0.16492	0.349303243	1.082714	0.000182	hypermethylated
cg01886514	0.12829	0.271671351	1.082454	4.82E-05	hypermethylated
cg07665387	0.17169	0.363556757	1.082375	2.39E-05	hypermethylated
cg10522607	0.14868	0.314722162	1.081868	3.11E-05	hypermethylated
cg19544372	0.16544	0.350132973	1.081595	2.24E-05	hypermethylated
cg01175020	0.17528	0.370956757	1.08159	0.000117	hypermethylated
cg14135731	0.14355	0.303790811	1.081525	4.42E-05	hypermethylated
cg03422911	0.23515	0.497624324	1.081476	4.93E-06	hypermethylated
cg06301178	0.22422	0.474459459	1.08137	7.47E-05	hypermethylated
cg17210933	0.17664	0.373763784	1.081315	0.000142	hypermethylated
cg10807260	0.1193	0.2524	1.081118	0.000172	hypermethylated
cg00579520	0.16943	0.358414595	1.08094	1.04E-05	hypermethylated
cg14047094	0.20361	0.430702703	1.080884	4.70E-05	hypermethylated
cg05314394	0.19446	0.411333514	1.080835	1.77E-05	hypermethylated
cg14054928	0.21268	0.449842703	1.080736	3.07E-05	hypermethylated
cg07271264	0.21935	0.463830811	1.080364	0.000176	hypermethylated
cg26865446	0.1757	0.371500541	1.08025	3.40E-05	hypermethylated
cg00699029	0.14272	0.301743784	1.080137	4.36E-05	hypermethylated
cg15598120	0.11867	0.250843243	1.079831	4.10E-05	hypermethylated
cg02604503	0.16631	0.351540541	1.079816	8.03E-05	hypermethylated
cg11806672	0.14248	0.301165946	1.079799	0.0002	hypermethylated
cg04878701	0.14075	0.297503784	1.079773	8.04E-06	hypermethylated
cg23615741	0.25452	0.537972973	1.079755	4.82E-05	hypermethylated
cg21299542	0.15498	0.327567027	1.079708	3.95E-05	hypermethylated
cg15683295	0.15583	0.329342162	1.079614	2.67E-05	hypermethylated
cg16954341	0.15839	0.334725946	1.079499	0.00032	hypermethylated
cg19378133	0.1694	0.357944865	1.079304	0.000274	hypermethylated
cg23444265	0.15569	0.328965405	1.07926	0.000129	hypermethylated
cg16792842	0.16748	0.353791892	1.078912	2.51E-05	hypermethylated
cg08409113	0.22926	0.484122703	1.078388	9.05E-05	hypermethylated
cg11893763	0.1879	0.396662162	1.077946	3.32E-05	hypermethylated
cg14489474	0.19858	0.41917027	1.077816	8.83E-06	hypermethylated
cg08120263	0.21275	0.449068649	1.077777	1.14E-05	hypermethylated
cg02925601	0.19757	0.41696973	1.077579	8.63E-05	hypermethylated
cg16876790	0.16127	0.34033027	1.077457	2.89E-05	hypermethylated

cg16167240	0.18302	0.386214054	1.077399	1.84E-05	hypermethylated
cg21480165	0.16975	0.358188108	1.077306	0.000112	hypermethylated
cg06825039	0.17056	0.359854595	1.077135	5.58E-05	hypermethylated
cg00107772	0.16321	0.344334054	1.077079	1.80E-05	hypermethylated
cg06493386	0.14769	0.311515135	1.07673	3.00E-05	hypermethylated
cg22115076	0.23865	0.503251892	1.076384	3.17E-06	hypermethylated
cg01100175	0.15776	0.332654054	1.076291	2.71E-05	hypermethylated
cg18950778	0.15679	0.330555676	1.07606	6.05E-06	hypermethylated
cg24621972	0.16116	0.339747027	1.075967	3.76E-05	hypermethylated
cg12337525	0.19572	0.412567568	1.075839	1.99E-05	hypermethylated
cg21096915	0.17421	0.367217297	1.075807	0.000119	hypermethylated
cg27621129	0.18295	0.385583784	1.075595	6.13E-06	hypermethylated
cg00741624	0.1882	0.396638378	1.075558	4.26E-05	hypermethylated
cg07961015	0.19945	0.420248649	1.075216	1.89E-05	hypermethylated
cg22914729	0.17663	0.372132432	1.075086	4.05E-05	hypermethylated
cg12176709	0.1586	0.33413027	1.075018	0.000115	hypermethylated
cg12285988	0.22074	0.464807568	1.074285	1.15E-05	hypermethylated
cg22052948	0.22834	0.480757297	1.074125	1.46E-05	hypermethylated
cg14146740	0.12624	0.265716757	1.07372	0.000129	hypermethylated
cg17085352	0.16023	0.337247027	1.073661	2.71E-05	hypermethylated
cg22871653	0.14895	0.313483243	1.07356	0.000274	hypermethylated
cg05787556	0.18323	0.385603784	1.073463	1.09E-06	hypermethylated
cg05043886	0.13205	0.277886486	1.073411	1.39E-05	hypermethylated
cg27471124	0.18691	0.39332	1.07336	1.14E-05	hypermethylated
cg13169011	0.1846	0.388402162	1.073149	0.000102	hypermethylated
cg04950301	0.16667	0.350648649	1.073032	7.32E-06	hypermethylated
cg23010048	0.18225	0.383418919	1.073003	0.000417	hypermethylated
cg16042149	0.19038	0.400498378	1.072914	6.62E-05	hypermethylated
cg06786064	0.16254	0.341927027	1.072894	7.03E-06	hypermethylated
cg12199221	0.19537	0.410889189	1.07254	0.000191	hypermethylated
cg02895156	0.15221	0.320066486	1.072308	0.000187	hypermethylated
cg15402529	0.18168	0.381984324	1.072114	1.28E-05	hypermethylated
cg18128969	0.13565	0.285189189	1.07203	1.66E-05	hypermethylated
cg00683332	0.26678	0.560825405	1.071901	1.18E-05	hypermethylated
cg09615505	0.20998	0.441406486	1.071856	1.04E-05	hypermethylated
cg02920216	0.23507	0.494085405	1.07167	6.62E-05	hypermethylated
cg16666329	0.15712	0.330159459	1.071296	1.54E-05	hypermethylated
cg25359908	0.12384	0.260179459	1.07103	0.000134	hypermethylated
cg12294208	0.16845	0.353883784	1.070955	4.58E-05	hypermethylated
cg08260406	0.21609	0.453932973	1.070847	3.32E-05	hypermethylated
cg23262897	0.21984	0.46178973	1.070782	6.05E-06	hypermethylated
cg17018096	0.17144	0.360121081	1.070778	8.60E-06	hypermethylated
cg07745624	0.17822	0.374246486	1.07033	2.78E-05	hypermethylated
cg22855255	0.19547	0.410465405	1.070313	0.000166	hypermethylated
cg06871074	0.15477	0.324955135	1.070115	1.71E-05	hypermethylated
cg01581084	0.25663	0.538740541	1.069901	1.50E-05	hypermethylated
cg16423682	0.12531	0.263059459	1.069887	0.000135	hypermethylated
cg12895779	0.12949	0.271818378	1.069802	3.67E-05	hypermethylated
cg14202850	0.173	0.363128649	1.069709	4.70E-05	hypermethylated
cg22740492	0.13062	0.273965405	1.068618	2.92E-05	hypermethylated
cg12464561	0.13917	0.291876216	1.068508	1.25E-05	hypermethylated
cg08301299	0.10979	0.230207027	1.068185	0.000168	hypermethylated
cg11565299	0.15155	0.317763784	1.068161	9.07E-06	hypermethylated
cg17102963	0.14559	0.305187568	1.067785	0.000135	hypermethylated
cg26199241	0.19512	0.408965405	1.067617	0.001667	hypermethylated
cg16981024	0.15263	0.319903243	1.067597	0.000229	hypermethylated
cg08280468	0.17372	0.364012432	1.067224	9.82E-06	hypermethylated
cg08690634	0.15456	0.323842703	1.067126	5.51E-05	hypermethylated

cg17834752	0.1432	0.299996757	1.066915	0.001052	hypermethylated
cg06668555	0.15905	0.333163243	1.066749	0.000132	hypermethylated
cg11089489	0.17215	0.3606	1.066733	1.42E-05	hypermethylated
cg11101926	0.18443	0.386308108	1.066679	0.000159	hypermethylated
cg11325267	0.16748	0.350591892	1.065804	2.15E-05	hypermethylated
cg14526953	0.14354	0.300468649	1.065762	6.38E-05	hypermethylated
cg14089267	0.24682	0.516647568	1.065721	6.70E-05	hypermethylated
cg02249074	0.12815	0.268211892	1.06554	1.80E-05	hypermethylated
cg01228134	0.19244	0.402718378	1.065363	0.000112	hypermethylated
cg00413617	0.18582	0.388828649	1.065229	2.89E-05	hypermethylated
cg14463412	0.16052	0.335842162	1.06503	0.000313	hypermethylated
cg03109101	0.1549	0.324075676	1.064994	2.04E-05	hypermethylated
cg13702996	0.14023	0.293345405	1.064805	7.84E-05	hypermethylated
cg08810842	0.28353	0.593094054	1.06476	6.57E-06	hypermethylated
cg01687680	0.1854	0.387807027	1.064698	3.19E-05	hypermethylated
cg02034102	0.16162	0.338007568	1.06445	6.15E-05	hypermethylated
cg26309511	0.17631	0.368707568	1.064363	3.85E-05	hypermethylated
cg04263186	0.13893	0.29044973	1.06393	3.23E-05	hypermethylated
cg23542968	0.17414	0.364048108	1.063881	4.94E-05	hypermethylated
cg15672768	0.24712	0.516608649	1.06386	1.54E-05	hypermethylated
cg08172065	0.12231	0.255683784	1.063818	2.10E-05	hypermethylated
cg21106407	0.17794	0.371967568	1.063786	4.15E-05	hypermethylated
cg11414921	0.15748	0.329149189	1.063573	0.000306	hypermethylated
cg02658693	0.14801	0.30933027	1.063453	4.58E-05	hypermethylated
cg11150585	0.15207	0.317775135	1.063271	0.000287	hypermethylated
cg25309759	0.15595	0.325827568	1.063025	5.19E-05	hypermethylated
cg15085086	0.18083	0.377733514	1.062735	0.000178	hypermethylated
cg03231647	0.18385	0.383945405	1.062372	9.82E-06	hypermethylated
cg04054313	0.17039	0.355814595	1.062285	0.000207	hypermethylated
cg24842086	0.18616	0.388695676	1.062098	1.92E-05	hypermethylated
cg10029186	0.1373	0.286651351	1.061965	1.99E-05	hypermethylated
cg26783679	0.1143	0.238597297	1.061752	4.47E-05	hypermethylated
cg22151446	0.17616	0.367706486	1.061668	3.95E-05	hypermethylated
cg13302823	0.22377	0.467067568	1.061615	1.66E-05	hypermethylated
cg03158581	0.12146	0.253518919	1.061612	1.26E-05	hypermethylated
cg24106020	0.14846	0.309761622	1.061084	8.26E-06	hypermethylated
cg02849693	0.2489	0.519260541	1.060892	1.14E-05	hypermethylated
cg09307530	0.20112	0.419498378	1.060609	3.17E-06	hypermethylated
cg12510458	0.1502	0.313199459	1.060197	2.92E-05	hypermethylated
cg17340519	0.216	0.450383784	1.060124	4.54E-06	hypermethylated
cg00007644	0.12053	0.251316757	1.060115	2.27E-05	hypermethylated
cg06679334	0.28368	0.591387027	1.059838	7.93E-06	hypermethylated
cg21609339	0.15186	0.316575135	1.059806	6.95E-05	hypermethylated
cg16218721	0.18483	0.385191892	1.059378	2.75E-06	hypermethylated
cg25761626	0.13037	0.27168973	1.059348	0.000688	hypermethylated
cg02488338	0.2367	0.493231892	1.059206	2.21E-05	hypermethylated
cg14720763	0.13066	0.272246486	1.059096	4.53E-05	hypermethylated
cg03662064	0.15251	0.317742162	1.058953	0.000148	hypermethylated
cg16713687	0.14394	0.299859459	1.058819	0.000485	hypermethylated
cg17392573	0.17772	0.370194595	1.058678	4.42E-05	hypermethylated
cg19751300	0.13031	0.271416216	1.058559	0.00012	hypermethylated
cg05903444	0.2256	0.469880541	1.058527	1.62E-05	hypermethylated
cg18869404	0.18264	0.380383243	1.058451	6.22E-06	hypermethylated
cg18175809	0.19334	0.402548108	1.058021	2.85E-05	hypermethylated
cg19893751	0.20753	0.432062703	1.057921	0.000117	hypermethylated
cg17518215	0.20986	0.436789189	1.05751	8.15E-06	hypermethylated
cg14319235	0.20231	0.420969189	1.057147	1.68E-05	hypermethylated
cg03867465	0.2807	0.584002703	1.056946	7.52E-06	hypermethylated

cg01161204	0.21092	0.438713514	1.056583	4.47E-05	hypermethylated
cg23714408	0.14852	0.308902162	1.056493	7.12E-05	hypermethylated
cg23448486	0.16664	0.346584865	1.056474	7.32E-06	hypermethylated
cg20916535	0.16253	0.33801027	1.056361	7.12E-05	hypermethylated
cg04020079	0.22683	0.471671351	1.056171	5.58E-05	hypermethylated
cg03091551	0.20161	0.419203784	1.056085	1.89E-05	hypermethylated
cg16816603	0.17773	0.369488108	1.055841	0.000112	hypermethylated
cg00397673	0.19334	0.401896757	1.055685	8.83E-06	hypermethylated
cg01541645	0.17965	0.373362162	1.055387	3.57E-05	hypermethylated
cg06980387	0.14532	0.30195027	1.055078	5.45E-05	hypermethylated
cg13822303	0.15024	0.312161622	1.055024	9.49E-05	hypermethylated
cg03774463	0.18638	0.387242703	1.054991	0.000166	hypermethylated
cg03020554	0.16095	0.334403243	1.054976	6.78E-05	hypermethylated
cg14381623	0.15356	0.318939459	1.05448	0.000681	hypermethylated
cg21431690	0.12514	0.259900541	1.054417	2.71E-05	hypermethylated
cg15532640	0.26832	0.557261622	1.0544	0.000726	hypermethylated
cg05462543	0.18387	0.381845946	1.054305	0.000681	hypermethylated
cg02447304	0.22151	0.459913514	1.053991	6.57E-06	hypermethylated
cg12432236	0.21771	0.451805946	1.053296	9.27E-05	hypermethylated
cg08061040	0.12795	0.265521622	1.053249	1.35E-05	hypermethylated
cg13928649	0.16593	0.344284324	1.053026	1.50E-05	hypermethylated
cg14005246	0.17284	0.358561622	1.052784	0.000178	hypermethylated
cg01243371	0.27373	0.567828108	1.052701	9.05E-05	hypermethylated
cg26979339	0.12086	0.250675135	1.052482	6.31E-05	hypermethylated
cg17352540	0.16445	0.340952973	1.051924	4.82E-05	hypermethylated
cg13640145	0.20101	0.416735676	1.051865	2.45E-05	hypermethylated
cg15373592	0.26255	0.544225946	1.051614	1.04E-05	hypermethylated
cg24396745	0.12127	0.251362703	1.051548	0.000107	hypermethylated
cg26972272	0.13928	0.28860973	1.051132	9.16E-05	hypermethylated
cg11936688	0.12313	0.255134595	1.051076	1.66E-05	hypermethylated
cg22850587	0.19236	0.398493514	1.050747	1.15E-05	hypermethylated
cg03237648	0.14779	0.306107027	1.050488	6.01E-05	hypermethylated
cg17003736	0.16735	0.346589189	1.050358	9.57E-06	hypermethylated
cg22910449	0.17356	0.359403243	1.050169	5.19E-05	hypermethylated
cg07622493	0.18141	0.375636757	1.050084	0.000274	hypermethylated
cg11073773	0.16891	0.349730811	1.04999	0.000122	hypermethylated
cg00899090	0.13749	0.28453027	1.049255	1.72E-06	hypermethylated
cg21165221	0.16055	0.332224324	1.049135	1.21E-05	hypermethylated
cg15490703	0.17027	0.3523	1.04898	1.94E-05	hypermethylated
cg22533573	0.14427	0.298383243	1.048395	9.82E-06	hypermethylated
cg22605415	0.16692	0.345213514	1.048332	0.000142	hypermethylated
cg22400703	0.22685	0.469070811	1.048067	0.000313	hypermethylated
cg11909912	0.16336	0.337756757	1.04793	3.95E-05	hypermethylated
cg14186187	0.13694	0.283065946	1.047594	0.000232	hypermethylated
cg23337620	0.17239	0.356297297	1.047405	3.90E-06	hypermethylated
cg22605919	0.22749	0.470172432	1.047387	4.93E-06	hypermethylated
cg05079049	0.21963	0.453814595	1.047028	6.22E-06	hypermethylated
cg07892413	0.13943	0.288083784	1.046947	0.000145	hypermethylated
cg03679521	0.14481	0.299196216	1.046931	0.00049	hypermethylated
cg09989644	0.15157	0.313158919	1.046911	9.57E-06	hypermethylated
cg27553162	0.16449	0.339830811	1.046817	0.000653	hypermethylated
cg15600488	0.1713	0.353882162	1.046744	4.82E-05	hypermethylated
cg13962664	0.15768	0.325711351	1.046594	1.54E-05	hypermethylated
cg04902302	0.15743	0.325083243	1.046099	1.52E-05	hypermethylated
cg09806671	0.21512	0.444202703	1.046077	3.79E-06	hypermethylated
cg08492173	0.21209	0.437860541	1.045795	1.66E-05	hypermethylated
cg01664241	0.18122	0.374014595	1.045352	1.04E-05	hypermethylated
cg05081498	0.23496	0.484918919	1.045328	9.95E-05	hypermethylated

cg13436155	0.18251	0.376534595	1.044807	1.30E-05	hypermethylated
cg25307665	0.17758	0.366322703	1.044646	0.00032	hypermethylated
cg25558103	0.23209	0.478726486	1.044517	0.000127	hypermethylated
cg17282004	0.17493	0.360795676	1.044404	4.58E-05	hypermethylated
cg27067781	0.24806	0.511518378	1.044097	1.84E-05	hypermethylated
cg14006181	0.19131	0.394483784	1.044054	2.78E-05	hypermethylated
cg18097850	0.14152	0.291798919	1.043969	1.84E-05	hypermethylated
cg22095604	0.18907	0.389841081	1.043966	0.000226	hypermethylated
cg19161124	0.22707	0.46813027	1.043773	1.50E-05	hypermethylated
cg15270892	0.20536	0.423356216	1.043717	5.45E-05	hypermethylated
cg17074396	0.17781	0.36655027	1.043675	0.000327	hypermethylated
cg11293190	0.18382	0.378878378	1.043441	3.23E-05	hypermethylated
cg23815646	0.17431	0.359272973	1.043425	4.58E-05	hypermethylated
cg00582524	0.25203	0.519405946	1.043267	2.39E-05	hypermethylated
cg18100702	0.18621	0.383739459	1.043197	1.12E-06	hypermethylated
cg06686226	0.15624	0.321976216	1.04319	0.000224	hypermethylated
cg13231921	0.17441	0.359295135	1.042687	8.42E-05	hypermethylated
cg19416570	0.23173	0.477326486	1.042532	7.65E-05	hypermethylated
cg11319389	0.17632	0.363156757	1.042396	0.000126	hypermethylated
cg04330683	0.18155	0.373862162	1.04214	8.37E-06	hypermethylated
cg10978753	0.19455	0.400604865	1.042039	7.29E-05	hypermethylated
cg02471153	0.20988	0.43212973	1.0419	4.30E-06	hypermethylated
cg26131286	0.18406	0.378948649	1.041826	0.000857	hypermethylated
cg03445516	0.18485	0.380480541	1.041468	6.22E-06	hypermethylated
cg03008707	0.14908	0.306848649	1.041441	0.000293	hypermethylated
cg26151467	0.2232	0.459388649	1.041378	3.85E-05	hypermethylated
cg09189772	0.16504	0.339622703	1.041117	0.000189	hypermethylated
cg13318787	0.17691	0.364004865	1.040942	2.95E-06	hypermethylated
cg09731694	0.15918	0.327484324	1.040767	0.000166	hypermethylated
cg21322436	0.22185	0.456374054	1.040632	0.000117	hypermethylated
cg07084163	0.15345	0.315651351	1.040563	1.66E-05	hypermethylated
cg19593767	0.27047	0.556315676	1.040435	6.14E-06	hypermethylated
cg14383828	0.16191	0.332957297	1.040145	8.03E-05	hypermethylated
cg09660365	0.19136	0.393478919	1.039997	1.66E-05	hypermethylated
cg04881814	0.20457	0.420570811	1.039754	1.12E-05	hypermethylated
cg18143296	0.19435	0.399534595	1.039663	1.06E-05	hypermethylated
cg27334919	0.21827	0.448694054	1.039618	2.39E-05	hypermethylated
cg02554345	0.18749	0.385408649	1.039575	1.40E-05	hypermethylated
cg27086874	0.19074	0.392085946	1.039563	5.51E-05	hypermethylated
cg12626956	0.19277	0.396225946	1.039443	1.37E-05	hypermethylated
cg02257793	0.14526	0.298563243	1.039399	0.000104	hypermethylated
cg26499363	0.17202	0.353505946	1.039158	4.05E-05	hypermethylated
cg12460133	0.27301	0.560951892	1.038923	3.67E-05	hypermethylated
cg18181607	0.17984	0.369504865	1.038879	3.23E-05	hypermethylated
cg17459298	0.18097	0.371802162	1.038785	6.15E-05	hypermethylated
cg06760710	0.18547	0.381032973	1.03873	2.67E-05	hypermethylated
cg27295832	0.16241	0.333648649	1.038689	0.000191	hypermethylated
cg27549878	0.19281	0.395996757	1.038309	3.57E-05	hypermethylated
cg08664487	0.20061	0.41196973	1.038145	1.94E-05	hypermethylated
cg25657713	0.2107	0.432608108	1.03787	3.30E-06	hypermethylated
cg19267596	0.17688	0.363126486	1.037701	0.0003	hypermethylated
cg17864046	0.15519	0.318584324	1.03764	8.63E-05	hypermethylated
cg04754315	0.182	0.373534595	1.037303	0.00017	hypermethylated
cg24954590	0.16511	0.33877027	1.03688	3.32E-05	hypermethylated
cg24865270	0.17157	0.352021622	1.036867	6.22E-06	hypermethylated
cg07440398	0.21116	0.433248108	1.036857	1.46E-05	hypermethylated
cg18358723	0.19693	0.404050811	1.036854	1.52E-05	hypermethylated
cg02575712	0.13374	0.274362703	1.036653	0.000154	hypermethylated

cg14959729	0.15727	0.322631892	1.036646	6.75E-06	hypermethylated
cg10721834	0.28629	0.587270811	1.036549	1.71E-05	hypermethylated
cg05039548	0.17273	0.354246486	1.036235	2.10E-05	hypermethylated
cg18492126	0.15198	0.311684865	1.036207	7.22E-06	hypermethylated
cg04605987	0.17717	0.363305946	1.036051	2.57E-05	hypermethylated
cg13347071	0.13678	0.28046973	1.035988	5.31E-05	hypermethylated
cg12055515	0.25008	0.512776216	1.03594	2.71E-05	hypermethylated
cg26820037	0.16151	0.331164324	1.035924	0.000155	hypermethylated
cg23292160	0.16546	0.33919027	1.035612	3.00E-05	hypermethylated
cg17966192	0.16794	0.344209189	1.03534	0.000262	hypermethylated
cg22879667	0.18753	0.384352973	1.03531	6.22E-06	hypermethylated
cg13702053	0.17076	0.34994973	1.035178	3.67E-05	hypermethylated
cg24071582	0.19703	0.403764324	1.035098	0.000277	hypermethylated
cg13315147	0.2133	0.437092432	1.035054	0.000204	hypermethylated
cg15491461	0.14014	0.287144324	1.034907	0.00024	hypermethylated
cg21214293	0.19085	0.391018919	1.034799	1.09E-05	hypermethylated
cg04930919	0.1175	0.240687027	1.034498	1.01E-05	hypermethylated
cg10965575	0.16133	0.330347568	1.03397	0.000588	hypermethylated
cg13307880	0.21358	0.437263784	1.033727	1.28E-05	hypermethylated
cg04235768	0.11223	0.229764324	1.033696	0.00029	hypermethylated
cg18525486	0.19303	0.395178919	1.033681	0.000109	hypermethylated
cg19857737	0.09899	0.202653514	1.03366	3.23E-05	hypermethylated
cg09988738	0.16189	0.331377838	1.033463	6.78E-05	hypermethylated
cg00321709	0.21053	0.43092973	1.033427	0.000726	hypermethylated
cg24139737	0.18524	0.379163243	1.033423	3.85E-05	hypermethylated
cg02222728	0.16255	0.332713514	1.033397	8.15E-06	hypermethylated
cg04427003	0.1276	0.261164865	1.033332	1.21E-05	hypermethylated
cg18813020	0.19908	0.407462703	1.03332	2.85E-05	hypermethylated
cg20507276	0.19988	0.408962162	1.032833	5.58E-05	hypermethylated
cg24645221	0.14293	0.292398378	1.032627	1.42E-05	hypermethylated
cg15541630	0.24935	0.510022703	1.032389	2.49E-06	hypermethylated
cg02052905	0.18263	0.373543243	1.032351	7.03E-05	hypermethylated
cg13985817	0.18807	0.384663784	1.032328	1.35E-05	hypermethylated
cg18784435	0.19404	0.396844865	1.032221	4.15E-05	hypermethylated
cg00723994	0.18358	0.375325946	1.031735	0.000161	hypermethylated
cg19828791	0.10933	0.223520541	1.031718	5.19E-05	hypermethylated
cg01083633	0.16223	0.331662703	1.031676	0.000403	hypermethylated
cg03587978	0.167	0.341383784	1.031546	1.01E-05	hypermethylated
cg00637687	0.17452	0.356739459	1.031478	4.58E-05	hypermethylated
cg00064261	0.14465	0.295642703	1.031288	0.000129	hypermethylated
cg21587238	0.25653	0.524295676	1.031253	0.000454	hypermethylated
cg00084338	0.22319	0.456124865	1.031156	0.001368	hypermethylated
cg04267526	0.1995	0.40764973	1.030941	2.21E-05	hypermethylated
cg15786900	0.19273	0.393722703	1.030599	1.05E-05	hypermethylated
cg22637890	0.15725	0.32124	1.030592	0.000109	hypermethylated
cg05924485	0.25202	0.514756757	1.030353	2.64E-06	hypermethylated
cg02485566	0.17738	0.36226973	1.030221	1.02E-05	hypermethylated
cg09225457	0.21644	0.441980541	1.030016	0.000185	hypermethylated
cg06558502	0.18381	0.375332432	1.029954	2.21E-05	hypermethylated
cg18065811	0.15973	0.326157838	1.029935	0.000163	hypermethylated
cg00123762	0.2564	0.52344	1.029628	0.000912	hypermethylated
cg03058660	0.25594	0.522401622	1.029354	2.48E-05	hypermethylated
cg26949694	0.18625	0.380106486	1.029163	2.10E-05	hypermethylated
cg00831126	0.21363	0.435939459	1.029014	2.15E-05	hypermethylated
cg13891702	0.13796	0.281515676	1.028965	0.000132	hypermethylated
cg21580428	0.15096	0.307999459	1.028761	6.31E-05	hypermethylated
cg06756919	0.18471	0.376815676	1.028597	2.42E-05	hypermethylated
cg10108296	0.21373	0.435988108	1.028499	2.29E-06	hypermethylated

cg09559950	0.13613	0.277625405	1.028155	0.00032	hypermethylated
cg10502121	0.25696	0.524015676	1.028066	4.24E-06	hypermethylated
cg21538208	0.27916	0.569152432	1.027723	7.12E-06	hypermethylated
cg02996801	0.20224	0.41232	1.027696	1.48E-05	hypermethylated
cg13016408	0.14423	0.294026486	1.027575	7.84E-05	hypermethylated
cg05184377	0.22885	0.466463784	1.027363	4.36E-06	hypermethylated
cg06095270	0.11744	0.239358919	1.027252	1.09E-05	hypermethylated
cg09746494	0.14769	0.300987568	1.027132	2.64E-05	hypermethylated
cg07035165	0.22157	0.451477297	1.026891	0.000142	hypermethylated
cg12627071	0.16302	0.332145405	1.026766	0.000209	hypermethylated
cg11268834	0.22577	0.459738378	1.025959	4.70E-05	hypermethylated
cg08482531	0.17597	0.358234595	1.025575	2.36E-06	hypermethylated
cg00584026	0.28826	0.58666	1.025154	0.000563	hypermethylated
cg19556343	0.15908	0.323711351	1.024955	1.62E-05	hypermethylated
cg03972398	0.13572	0.276146486	1.0248	0.00035	hypermethylated
cg11605835	0.18656	0.379518919	1.024532	1.80E-05	hypermethylated
cg25442600	0.229	0.465847568	1.02451	1.35E-05	hypermethylated
cg06364757	0.1379	0.280463784	1.024192	7.84E-05	hypermethylated
cg05501996	0.27437	0.558008108	1.024163	5.43E-06	hypermethylated
cg12700788	0.15676	0.31871027	1.023688	0.000358	hypermethylated
cg15174552	0.19506	0.396504865	1.023421	3.76E-05	hypermethylated
cg07300060	0.15688	0.318884324	1.023372	2.74E-05	hypermethylated
cg09839170	0.17755	0.360875135	1.023274	0.000202	hypermethylated
cg04541368	0.29748	0.604567027	1.02311	0.00024	hypermethylated
cg01211065	0.16777	0.340932973	1.023003	8.84E-05	hypermethylated
cg04316624	0.19193	0.38996973	1.022782	3.90E-06	hypermethylated
cg03890222	0.14509	0.294786486	1.022722	0.000112	hypermethylated
cg01759562	0.173	0.351416757	1.022411	0.000101	hypermethylated
cg00297721	0.13294	0.269996216	1.022164	1.39E-05	hypermethylated
cg22340508	0.23819	0.483738378	1.022114	4.42E-05	hypermethylated
cg20026939	0.20344	0.413164324	1.022112	7.52E-06	hypermethylated
cg05241015	0.16524	0.335558378	1.022001	8.26E-06	hypermethylated
cg14737286	0.15749	0.319805405	1.021934	3.19E-05	hypermethylated
cg09802066	0.18521	0.376094595	1.021934	2.54E-05	hypermethylated
cg02478907	0.17642	0.358119459	1.021427	6.46E-05	hypermethylated
cg20896728	0.12716	0.258088649	1.021222	7.56E-05	hypermethylated
cg15979173	0.25152	0.510426486	1.02103	5.28E-06	hypermethylated
cg13205528	0.13843	0.280917838	1.020992	6.15E-05	hypermethylated
cg19786627	0.1679	0.340679459	1.020813	1.71E-05	hypermethylated
cg01429449	0.21913	0.444594595	1.020703	4.47E-05	hypermethylated
cg26509715	0.17453	0.354080541	1.020603	0.000191	hypermethylated
cg24129390	0.28314	0.574311892	1.020319	1.94E-05	hypermethylated
cg13924432	0.19621	0.397968649	1.020256	7.29E-05	hypermethylated
cg02508651	0.20103	0.407730811	1.020206	2.51E-05	hypermethylated
cg11346837	0.17079	0.346381622	1.020139	4.36E-05	hypermethylated
cg00105628	0.16899	0.342660541	1.019842	9.27E-05	hypermethylated
cg02150988	0.14738	0.298798378	1.019632	0.000517	hypermethylated
cg01580681	0.19509	0.39549027	1.019502	2.85E-05	hypermethylated
cg26917694	0.19705	0.399425405	1.019364	1.99E-05	hypermethylated
cg22123464	0.22679	0.459511892	1.018745	0.000726	hypermethylated
cg00803827	0.20978	0.425031892	1.018694	1.50E-05	hypermethylated
cg04008601	0.21971	0.445114054	1.018575	0.000142	hypermethylated
cg08567644	0.16201	0.328110811	1.0181	0.00043	hypermethylated
cg15014975	0.20128	0.407568649	1.017839	0.000403	hypermethylated
cg16945186	0.2733	0.553344865	1.017693	1.25E-05	hypermethylated
cg11488401	0.11344	0.229602162	1.017207	2.15E-05	hypermethylated
cg20997792	0.12993	0.262970811	1.017168	0.00012	hypermethylated
cg08784129	0.16378	0.331456757	1.017061	0.000214	hypermethylated

cg14870229	0.14236	0.288103784	1.017045	2.85E-05	hypermethylated
cg11824921	0.16291	0.329634054	1.01679	0.000104	hypermethylated
cg24469980	0.24232	0.490305405	1.016767	7.52E-06	hypermethylated
cg18156592	0.22038	0.445899459	1.016725	4.26E-05	hypermethylated
cg22450968	0.19149	0.387434595	1.016684	0.000104	hypermethylated
cg23401445	0.20341	0.411541081	1.016646	3.59E-06	hypermethylated
cg07380705	0.16406	0.331872973	1.016408	2.36E-06	hypermethylated
cg15377283	0.24152	0.488481081	1.01616	4.94E-05	hypermethylated
cg10420161	0.24574	0.496982162	1.016061	9.31E-06	hypermethylated
cg05176667	0.17264	0.349087027	1.01582	0.000115	hypermethylated
cg12983394	0.16092	0.325347027	1.015636	0.000166	hypermethylated
cg06218338	0.1797	0.363316216	1.015635	0.000265	hypermethylated
cg22538780	0.20835	0.421137838	1.015283	0.000174	hypermethylated
cg17080163	0.21825	0.441144324	1.015269	0.00024	hypermethylated
cg25913172	0.12484	0.252267027	1.014871	1.21E-05	hypermethylated
cg04068980	0.22858	0.46184973	1.014724	4.58E-05	hypermethylated
cg22268510	0.26893	0.543364324	1.014689	7.03E-06	hypermethylated
cg21836358	0.25946	0.524184865	1.014564	6.39E-06	hypermethylated
cg14380586	0.16574	0.334812973	1.014434	5.32E-05	hypermethylated
cg08414570	0.1586	0.320388108	1.014428	1.94E-05	hypermethylated
cg23588462	0.208	0.420098919	1.014146	1.64E-05	hypermethylated
cg09410389	0.2162	0.436503784	1.013628	8.22E-05	hypermethylated
cg07027430	0.21542	0.434881081	1.013469	4.26E-05	hypermethylated
cg08721908	0.27261	0.550291351	1.013357	0.0002	hypermethylated
cg16835233	0.1668	0.336687027	1.013289	3.95E-05	hypermethylated
cg06544405	0.15006	0.302827027	1.012955	5.06E-05	hypermethylated
cg14855972	0.16547	0.333913514	1.012905	0.000104	hypermethylated
cg17722675	0.16476	0.332478919	1.012897	6.31E-05	hypermethylated
cg07329360	0.25422	0.513000541	1.012883	1.42E-05	hypermethylated
cg05581451	0.18058	0.364336216	1.012632	2.79E-06	hypermethylated
cg09813610	0.13087	0.263998919	1.012398	3.54E-06	hypermethylated
cg26343001	0.27046	0.545499459	1.012163	1.66E-05	hypermethylated
cg10484958	0.19141	0.386048108	1.012114	0.000174	hypermethylated
cg26879813	0.17186	0.346615676	1.012103	1.92E-05	hypermethylated
cg08938584	0.19783	0.398802162	1.011412	2.15E-05	hypermethylated
cg08363794	0.21631	0.436027568	1.011319	2.49E-06	hypermethylated
cg23329272	0.13377	0.26964	1.01128	0.001635	hypermethylated
cg02837536	0.2471	0.498036757	1.011157	0.000342	hypermethylated
cg04046669	0.21441	0.43214	1.011127	5.28E-06	hypermethylated
cg14511698	0.18613	0.375106486	1.01099	4.01E-06	hypermethylated
cg08379987	0.25536	0.514604324	1.010931	2.92E-05	hypermethylated
cg09521719	0.1877	0.378235135	1.010855	2.92E-05	hypermethylated
cg27304406	0.13608	0.274194595	1.010745	0.000138	hypermethylated
cg11874762	0.20255	0.408124324	1.010731	6.15E-05	hypermethylated
cg11229185	0.17605	0.354681622	1.010539	0.000674	hypermethylated
cg19407095	0.18449	0.371675676	1.010502	2.27E-05	hypermethylated
cg23404248	0.1753	0.353159459	1.010494	6.78E-05	hypermethylated
cg14514813	0.21096	0.424971892	1.010398	7.52E-06	hypermethylated
cg12602633	0.24973	0.503008108	1.010213	5.12E-05	hypermethylated
cg09522056	0.25401	0.511583243	1.010084	6.13E-06	hypermethylated
cg05380019	0.22488	0.452869189	1.009939	7.93E-06	hypermethylated
cg08143343	0.22684	0.456793514	1.009867	0.000187	hypermethylated
cg06003656	0.13781	0.277483784	1.009723	0.000575	hypermethylated
cg19119032	0.12187	0.245384324	1.0097	0.000209	hypermethylated
cg02020829	0.14919	0.30038973	1.009685	3.40E-05	hypermethylated
cg14008883	0.18741	0.377291351	1.009481	7.42E-06	hypermethylated
cg10906284	0.18774	0.377862703	1.009126	7.20E-05	hypermethylated
cg10862468	0.25492	0.513071351	1.009115	0.00098	hypermethylated

cg11671688	0.25384	0.510871351	1.009041	1.50E-05	hypermethylated
cg05342515	0.18288	0.368043243	1.008978	2.64E-05	hypermethylated
cg24236591	0.13755	0.276784865	1.008809	0.000454	hypermethylated
cg14738806	0.2727	0.548641081	1.008548	4.26E-05	hypermethylated
cg10967350	0.18484	0.371858378	1.008476	1.80E-05	hypermethylated
cg01610632	0.21868	0.439924324	1.008434	1.52E-05	hypermethylated
cg24054190	0.17375	0.349527027	1.008391	0.000132	hypermethylated
cg13051450	0.24254	0.487742162	1.007896	1.42E-05	hypermethylated
cg19389953	0.1652	0.332212432	1.007892	2.10E-05	hypermethylated
cg07301944	0.15157	0.304747027	1.007628	5.65E-05	hypermethylated
cg11097541	0.19097	0.383898919	1.00738	4.73E-06	hypermethylated
cg18369516	0.22024	0.442677838	1.007181	0.000135	hypermethylated
cg23188704	0.2162	0.434537297	1.007113	3.35E-06	hypermethylated
cg06284231	0.19437	0.390538378	1.006659	6.01E-05	hypermethylated
cg26994225	0.16472	0.330938919	1.006549	0.000112	hypermethylated
cg18720113	0.16323	0.327934054	1.006499	4.36E-05	hypermethylated
cg04002608	0.16135	0.324137838	1.006414	4.94E-05	hypermethylated
cg11575929	0.1602	0.321827027	1.006411	3.69E-06	hypermethylated
cg23905216	0.17617	0.353880541	1.006294	0.000166	hypermethylated
cg11412853	0.20288	0.407514054	1.006223	0.000287	hypermethylated
cg00890363	0.21762	0.437075676	1.006072	3.85E-05	hypermethylated
cg11700800	0.20217	0.406034595	1.006034	1.25E-05	hypermethylated
cg06951626	0.20934	0.420384324	1.005861	8.42E-05	hypermethylated
cg08523865	0.20792	0.417487027	1.005703	1.39E-05	hypermethylated
cg18646365	0.17193	0.345194054	1.005586	0.000234	hypermethylated
cg19188855	0.20761	0.416784324	1.005425	1.30E-05	hypermethylated
cg12371933	0.15383	0.308748649	1.005096	7.84E-05	hypermethylated
cg16902509	0.15126	0.303561622	1.004959	0.000109	hypermethylated
cg00503302	0.21409	0.429592432	1.004751	1.56E-05	hypermethylated
cg05457480	0.25537	0.512395135	1.004668	2.71E-05	hypermethylated
cg05314679	0.17087	0.342836757	1.004623	5.51E-05	hypermethylated
cg18805066	0.26846	0.538616216	1.004551	0.000399	hypermethylated
cg00339682	0.18157	0.364273514	1.004496	2.71E-05	hypermethylated
cg27626299	0.14082	0.282491351	1.004354	0.000155	hypermethylated
cg01305913	0.16289	0.326750811	1.004293	0.000195	hypermethylated
cg16600634	0.18348	0.368004324	1.0041	2.92E-05	hypermethylated
cg24099067	0.20124	0.403615135	1.004063	3.04E-06	hypermethylated
cg27277532	0.19383	0.388726486	1.003964	3.23E-05	hypermethylated
cg13067553	0.18935	0.37973027	1.00392	0.001949	hypermethylated
cg24468070	0.2146	0.430295135	1.003676	0.000256	hypermethylated
cg17054060	0.32167	0.644898378	1.00349	4.24E-06	hypermethylated
cg12351433	0.21865	0.438347027	1.00345	0.000198	hypermethylated
cg05022688	0.15329	0.307296216	1.003366	2.38E-05	hypermethylated
cg07803375	0.2701	0.541354054	1.003079	0.001031	hypermethylated
cg09417809	0.23304	0.467025405	1.002923	1.50E-05	hypermethylated
cg14783814	0.17103	0.342741622	1.002872	0.000182	hypermethylated
cg00528572	0.21041	0.421642703	1.002818	0.000191	hypermethylated
cg22639011	0.18	0.360603243	1.002415	5.19E-05	hypermethylated
cg07928083	0.18789	0.376401081	1.002382	2.71E-05	hypermethylated
cg03227021	0.19901	0.398676216	1.002377	1.06E-05	hypermethylated
cg16051195	0.19831	0.397221622	1.002187	1.21E-05	hypermethylated
cg12277278	0.15639	0.313241081	1.002125	0.000155	hypermethylated
cg06921573	0.13949	0.279369189	1.002011	7.93E-05	hypermethylated
cg26196480	0.20835	0.41725027	1.001904	0.000185	hypermethylated
cg13586599	0.20248	0.405435676	1.001694	0.00029	hypermethylated
cg13488201	0.21684	0.434175135	1.001646	6.14E-06	hypermethylated
cg05722981	0.19006	0.380353514	1.000886	5.38E-05	hypermethylated
cg15909981	0.18761	0.375445405	1.000866	3.00E-05	hypermethylated

cg04461228	0.19375	0.387728649	1.000851	1.42E-05	hypermethylated
cg11464895	0.22457	0.449266486	1.000406	2.85E-05	hypermethylated
cg03826642	0.26571	0.53150973	1.000244	0.000191	hypermethylated
cg06263495	0.18727	0.374568108	1.000108	0.000142	hypermethylated
cg13266435	0.19478	0.389584324	1.00009	7.29E-05	hypermethylated
cg14141458	0.13189	0.263795135	1.000083	0.000234	hypermethylated
cg09267483	0.13959	0.27916973	0.999947	0.000506	hypermethylated
cg24394856	0.2151	0.43012	0.999732	7.65E-05	hypermethylated
cg02249292	0.17998	0.359890811	0.999723	2.48E-05	hypermethylated
cg01811325	0.16887	0.337584865	0.999337	5.73E-06	hypermethylated
cg03515663	0.21634	0.432442703	0.999209	6.75E-06	hypermethylated
cg21233722	0.20143	0.402614054	0.999119	2.04E-05	hypermethylated
cg09524946	0.20401	0.407743243	0.999021	0.000126	hypermethylated
cg17450733	0.23228	0.464208649	0.998908	8.03E-05	hypermethylated
cg21591624	0.17358	0.346897297	0.998908	9.57E-06	hypermethylated
cg25148589	0.17882	0.357341622	0.998796	0.000132	hypermethylated
cg16135989	0.25958	0.518706486	0.998739	6.31E-05	hypermethylated
cg20707023	0.13475	0.269218378	0.998492	8.42E-05	hypermethylated
cg16615154	0.22185	0.443183784	0.998321	8.37E-06	hypermethylated
cg04938966	0.17641	0.352363243	0.998131	1.02E-05	hypermethylated
cg10050816	0.17967	0.358781081	0.997754	5.86E-05	hypermethylated
cg16918905	0.21451	0.428308108	0.997604	8.22E-05	hypermethylated
cg04105250	0.15633	0.31213027	0.997554	6.62E-05	hypermethylated
cg22967396	0.17537	0.349929189	0.996661	7.03E-06	hypermethylated
cg19094243	0.17343	0.346036216	0.99657	0.000195	hypermethylated
cg03585447	0.19031	0.379675676	0.996416	6.95E-05	hypermethylated
cg09876519	0.20369	0.406365405	0.996402	1.92E-05	hypermethylated
cg11826638	0.14628	0.291811892	0.996306	1.84E-05	hypermethylated
cg15174623	0.19285	0.384685946	0.996202	9.69E-06	hypermethylated
cg12962191	0.17203	0.343138378	0.99613	0.000224	hypermethylated
cg08988179	0.13124	0.261774054	0.996115	8.03E-05	hypermethylated
cg09032973	0.2438	0.486236757	0.995961	1.09E-06	hypermethylated
cg10245879	0.18093	0.360838919	0.995923	8.84E-05	hypermethylated
cg02749463	0.21356	0.425869189	0.995769	0.001395	hypermethylated
cg04308769	0.26302	0.524412973	0.995531	1.28E-05	hypermethylated
cg12613383	0.16541	0.329785405	0.995481	9.49E-05	hypermethylated
cg05981335	0.21059	0.419862162	0.995479	2.64E-06	hypermethylated
cg20756891	0.20831	0.415267027	0.995307	7.12E-06	hypermethylated
cg00604202	0.17699	0.352818919	0.99526	1.50E-05	hypermethylated
cg19830270	0.18947	0.377607027	0.994916	2.71E-05	hypermethylated
cg11400953	0.20473	0.408005405	0.994866	0.00039	hypermethylated
cg11842415	0.20676	0.412041622	0.994833	0.0002	hypermethylated
cg26365545	0.24462	0.487396216	0.994553	4.24E-06	hypermethylated
cg17611193	0.18316	0.364923784	0.994491	8.94E-05	hypermethylated
cg14434870	0.15104	0.300923243	0.994465	2.21E-05	hypermethylated
cg24935332	0.25013	0.498230811	0.994136	6.02E-07	hypermethylated
cg02867763	0.17777	0.354091351	0.99411	0.000104	hypermethylated
cg21051580	0.19992	0.398098919	0.993704	1.09E-05	hypermethylated
cg07150445	0.13811	0.274904865	0.993115	2.78E-05	hypermethylated
cg18299578	0.15962	0.317635676	0.992732	5.58E-06	hypermethylated
cg05223604	0.11901	0.236808649	0.992639	2.04E-05	hypermethylated
cg01624414	0.18948	0.377004865	0.992538	0.000335	hypermethylated
cg17017189	0.24034	0.478192973	0.992516	1.23E-05	hypermethylated
cg13908315	0.1629	0.324031351	0.992147	0.000259	hypermethylated
cg02844545	0.16796	0.334077838	0.992067	1.42E-05	hypermethylated
cg02128112	0.18249	0.362944865	0.991933	1.14E-05	hypermethylated
cg04181546	0.16925	0.336592432	0.991847	1.54E-05	hypermethylated
cg01337391	0.16821	0.334464324	0.991589	2.35E-05	hypermethylated

cg06911487	0.18264	0.363145405	0.991545	0.000152	hypermethylated
cg18182111	0.20216	0.401923243	0.991422	2.96E-05	hypermethylated
cg04365721	0.18445	0.36666	0.991213	3.49E-06	hypermethylated
cg14028400	0.25662	0.510054054	0.991017	4.01E-06	hypermethylated
cg11280525	0.15502	0.308074054	0.990823	3.00E-05	hypermethylated
cg11248896	0.22537	0.447878378	0.990812	8.83E-06	hypermethylated
cg27117509	0.24171	0.480316757	0.990709	4.88E-05	hypermethylated
cg18731327	0.1841	0.365802703	0.990576	9.31E-06	hypermethylated
cg24115032	0.16883	0.335418919	0.990393	6.93E-06	hypermethylated
cg11881313	0.12767	0.253631351	0.990314	0.000126	hypermethylated
cg09232937	0.19407	0.385428649	0.989887	1.01E-05	hypermethylated
cg22859289	0.21198	0.420957297	0.989746	0.000313	hypermethylated
cg00157572	0.20794	0.412865405	0.989504	2.91E-06	hypermethylated
cg17018946	0.20413	0.405221622	0.989223	6.01E-05	hypermethylated
cg10018615	0.13646	0.270880541	0.989179	4.47E-05	hypermethylated
cg05237641	0.22456	0.445645946	0.988797	5.28E-06	hypermethylated
cg14832406	0.17468	0.346608649	0.988593	5.58E-05	hypermethylated
cg27125849	0.17738	0.351954054	0.988544	0.000242	hypermethylated
cg17807479	0.17223	0.341629189	0.988095	0.000822	hypermethylated
cg04834436	0.23695	0.469983784	0.988028	3.85E-05	hypermethylated
cg11201710	0.16873	0.334656757	0.987966	0.000224	hypermethylated
cg23341223	0.17838	0.353768649	0.987852	3.90E-05	hypermethylated
cg02285263	0.12669	0.251251351	0.987829	0.001481	hypermethylated
cg09083947	0.22205	0.440239459	0.987404	4.15E-05	hypermethylated
cg00107187	0.14906	0.295527568	0.9874	9.72E-05	hypermethylated
cg17918544	0.18884	0.374358378	0.987256	6.31E-05	hypermethylated
cg04645914	0.25785	0.511153514	0.987225	2.32E-05	hypermethylated
cg01449136	0.21056	0.417362703	0.98707	6.22E-06	hypermethylated
cg20748533	0.24685	0.489285405	0.987042	0.00012	hypermethylated
cg22517995	0.17207	0.341018378	0.986854	1.75E-05	hypermethylated
cg26324366	0.12216	0.242096216	0.986809	4.15E-05	hypermethylated
cg15713103	0.15551	0.308182162	0.986776	5.45E-05	hypermethylated
cg07569216	0.2061	0.408422703	0.986719	0.000214	hypermethylated
cg27639030	0.15229	0.301782703	0.986689	1.56E-05	hypermethylated
cg13321077	0.24134	0.478225946	0.986625	4.60E-06	hypermethylated
cg15808426	0.21491	0.425843784	0.986592	1.18E-05	hypermethylated
cg06117072	0.21427	0.424573514	0.986585	1.04E-05	hypermethylated
cg04751631	0.19352	0.383450811	0.986559	0.00064	hypermethylated
cg14750066	0.19258	0.381541081	0.986381	3.95E-05	hypermethylated
cg14300730	0.19333	0.382982162	0.986212	6.31E-05	hypermethylated
cg10441628	0.17006	0.336868108	0.98614	0.000209	hypermethylated
cg08713073	0.1876	0.371598919	0.986086	4.76E-05	hypermethylated
cg16747564	0.1583	0.313504865	0.985827	2.02E-05	hypermethylated
cg07883457	0.23178	0.459023784	0.985813	1.29E-06	hypermethylated
cg26261502	0.1872	0.370732973	0.9858	2.15E-05	hypermethylated
cg13558371	0.19743	0.390858378	0.985305	0.000287	hypermethylated
cg09661370	0.22911	0.453558919	0.98525	1.18E-05	hypermethylated
cg13167730	0.17761	0.351572973	0.985111	7.41E-06	hypermethylated
cg07915884	0.29143	0.576821081	0.984974	1.26E-05	hypermethylated
cg09191036	0.22297	0.441239459	0.984712	2.92E-05	hypermethylated
cg16347279	0.18588	0.367834054	0.984684	4.88E-05	hypermethylated
cg13740815	0.21421	0.423888649	0.98466	3.67E-05	hypermethylated
cg14385245	0.16824	0.332776216	0.984032	8.83E-06	hypermethylated
cg00049047	0.19943	0.394417838	0.983842	6.46E-05	hypermethylated
cg04167239	0.20052	0.396566486	0.983817	7.72E-06	hypermethylated
cg06649410	0.1733	0.342711351	0.983722	5.86E-05	hypermethylated
cg08552214	0.18035	0.356633514	0.983643	0.000626	hypermethylated
cg04166294	0.20895	0.413140541	0.983475	3.85E-05	hypermethylated

cg00918522	0.20958	0.414336757	0.983303	5.19E-05	hypermethylated
cg24907814	0.17772	0.351306486	0.983124	3.39E-06	hypermethylated
cg24931520	0.16468	0.325498919	0.982987	4.47E-05	hypermethylated
cg00868523	0.20563	0.406398378	0.982844	4.82E-05	hypermethylated
cg20171396	0.22957	0.453710811	0.982839	2.32E-05	hypermethylated
cg23815900	0.14998	0.296397297	0.982762	0.000107	hypermethylated
cg07546916	0.17358	0.342965405	0.982462	0.000122	hypermethylated
cg13777681	0.15827	0.31268	0.982299	8.03E-05	hypermethylated
cg14289511	0.17783	0.351247568	0.98199	0.001275	hypermethylated
cg07398767	0.16142	0.318826486	0.981952	0.000204	hypermethylated
cg26365925	0.23583	0.465723243	0.981726	1.01E-05	hypermethylated
cg07750560	0.19754	0.390054054	0.981529	5.45E-05	hypermethylated
cg16700282	0.18815	0.371491892	0.981447	5.73E-06	hypermethylated
cg01059449	0.19345	0.38194973	0.981422	8.37E-06	hypermethylated
cg05022059	0.18864	0.372374054	0.981117	0.000174	hypermethylated
cg18089569	0.14152	0.279262703	0.980617	0.000107	hypermethylated
cg01815567	0.25792	0.50892	0.980515	0.000172	hypermethylated
cg16006349	0.23024	0.454212973	0.98023	3.62E-05	hypermethylated
cg16557178	0.20299	0.400416757	0.980094	1.39E-05	hypermethylated
cg17882660	0.19742	0.389412973	0.980033	6.08E-05	hypermethylated
cg18824990	0.1803	0.355632432	0.979988	0.0002	hypermethylated
cg20256245	0.13582	0.267851892	0.97974	2.18E-05	hypermethylated
cg22978515	0.24241	0.47797027	0.979472	0.000893	hypermethylated
cg04112787	0.17995	0.354806486	0.979436	7.47E-05	hypermethylated
cg03894892	0.24482	0.482656216	0.979275	2.15E-05	hypermethylated
cg11015251	0.2504	0.493508108	0.978839	1.12E-05	hypermethylated
cg08076125	0.19838	0.390981081	0.978832	0.000287	hypermethylated
cg18952647	0.18782	0.370147568	0.97875	0.000268	hypermethylated
cg17554126	0.15843	0.312208649	0.978665	1.44E-05	hypermethylated
cg01106338	0.17829	0.351338919	0.978638	1.99E-05	hypermethylated
cg02928664	0.23546	0.463815676	0.97807	0.000219	hypermethylated
cg01016119	0.20579	0.405313514	0.977865	0.000382	hypermethylated
cg13965328	0.13705	0.269855676	0.977486	0.000154	hypermethylated
cg25751787	0.16778	0.330335135	0.97736	9.82E-06	hypermethylated
cg04543289	0.20443	0.402479459	0.977308	1.84E-05	hypermethylated
cg04290586	0.22981	0.452404324	0.977171	1.09E-05	hypermethylated
cg20399871	0.17392	0.342374595	0.977152	1.25E-05	hypermethylated
cg12065138	0.20705	0.407546486	0.976985	0.000161	hypermethylated
cg01220680	0.16836	0.331347568	0.976796	0.000155	hypermethylated
cg00946245	0.12822	0.252344324	0.976772	9.49E-05	hypermethylated
cg11391201	0.1718	0.338098378	0.976713	8.15E-06	hypermethylated
cg13561879	0.26795	0.527216757	0.976432	0.000135	hypermethylated
cg07997434	0.20135	0.396161081	0.976382	0.000667	hypermethylated
cg13368923	0.19488	0.383406486	0.976289	9.05E-05	hypermethylated
cg25942450	0.22354	0.439758919	0.97618	1.72E-06	hypermethylated
cg00409356	0.15381	0.302577297	0.976154	0.000127	hypermethylated
cg00495775	0.17894	0.35197027	0.975978	3.44E-06	hypermethylated
cg23061046	0.16428	0.323128108	0.975949	4.58E-05	hypermethylated
cg01033938	0.14163	0.278533514	0.975724	4.82E-05	hypermethylated
cg16072688	0.16289	0.320336216	0.975689	0.000115	hypermethylated
cg08064488	0.18261	0.359092973	0.975592	2.27E-05	hypermethylated
cg18581378	0.19384	0.381101622	0.975309	8.22E-05	hypermethylated
cg01530605	0.20614	0.405270811	0.975262	7.75E-05	hypermethylated
cg07119829	0.16973	0.333684865	0.975245	4.15E-05	hypermethylated
cg18524739	0.15901	0.312444865	0.974484	3.85E-05	hypermethylated
cg26574334	0.17932	0.35228	0.974186	5.19E-05	hypermethylated
cg07603511	0.23512	0.461771351	0.973781	4.24E-06	hypermethylated
cg00705059	0.1899	0.372958378	0.973775	9.05E-05	hypermethylated

cg20381975	0.17544	0.344452432	0.973327	6.30E-06	hypermethylated
cg00662556	0.18103	0.35526	0.972646	6.22E-06	hypermethylated
cg26214742	0.26864	0.527110811	0.972432	7.12E-06	hypermethylated
cg06701529	0.23919	0.469268649	0.972257	3.23E-05	hypermethylated
cg11703632	0.17194	0.337236216	0.971854	9.72E-05	hypermethylated
cg05547902	0.17159	0.336503784	0.971657	5.00E-05	hypermethylated
cg03144922	0.23659	0.463965405	0.971628	1.66E-05	hypermethylated
cg02184008	0.17886	0.350623243	0.971091	2.96E-05	hypermethylated
cg25987208	0.22199	0.435156757	0.971041	6.15E-05	hypermethylated
cg24283289	0.25049	0.491010811	0.971002	1.94E-05	hypermethylated
cg13647052	0.15055	0.295069189	0.970811	7.65E-05	hypermethylated
cg17457637	0.18588	0.364300541	0.970758	1.62E-05	hypermethylated
cg15794859	0.19402	0.380233514	0.97068	4.42E-05	hypermethylated
cg17991695	0.1905	0.37320973	0.970196	1.46E-05	hypermethylated
cg14422827	0.18169	0.355917838	0.970065	0.000191	hypermethylated
cg06566775	0.17217	0.337265405	0.970051	6.46E-05	hypermethylated
cg14144025	0.18351	0.359471892	0.97002	4.70E-05	hypermethylated
cg02415779	0.33195	0.650141081	0.969787	0.000107	hypermethylated
cg27016494	0.21995	0.430768108	0.969736	4.48E-06	hypermethylated
cg22488849	0.1815	0.355347568	0.969261	4.24E-06	hypermethylated
cg18612627	0.19587	0.383478919	0.969251	2.10E-05	hypermethylated
cg07899681	0.23311	0.456315135	0.96902	2.04E-05	hypermethylated
cg03127416	0.20531	0.401830811	0.968784	1.30E-05	hypermethylated
cg16236679	0.11073	0.216712432	0.968736	0.000107	hypermethylated
cg06525987	0.19632	0.384158378	0.968494	0.000195	hypermethylated
cg20587394	0.15662	0.306467568	0.968466	7.03E-05	hypermethylated
cg09368188	0.19213	0.375931892	0.968389	3.95E-05	hypermethylated
cg01460202	0.216	0.422623784	0.968343	4.36E-06	hypermethylated
cg06382559	0.18201	0.356007027	0.967888	5.86E-05	hypermethylated
cg13974394	0.17749	0.347133514	0.967753	1.30E-05	hypermethylated
cg18391323	0.16912	0.330751351	0.9677	6.39E-06	hypermethylated
cg16179952	0.22574	0.44146	0.967621	4.86E-06	hypermethylated
cg03682719	0.22848	0.446811351	0.967598	1.75E-05	hypermethylated
cg05715492	0.1891	0.369752973	0.967412	0.004691	hypermethylated
cg16497340	0.15606	0.305137297	0.967358	8.37E-06	hypermethylated
cg06987468	0.16052	0.313848649	0.967316	0.000342	hypermethylated
cg08380411	0.16163	0.316018378	0.967313	0.000161	hypermethylated
cg01486146	0.16571	0.323798919	0.966438	5.72E-05	hypermethylated
cg19064523	0.22254	0.434836757	0.966409	6.22E-06	hypermethylated
cg22513901	0.18289	0.357158378	0.965588	6.57E-06	hypermethylated
cg16154416	0.16233	0.316986486	0.965492	2.32E-05	hypermethylated
cg10052561	0.22635	0.441943784	0.965308	0.000174	hypermethylated
cg20707630	0.15762	0.307721622	0.965175	8.63E-05	hypermethylated
cg23093609	0.17037	0.332564324	0.964962	8.15E-06	hypermethylated
cg15105326	0.21959	0.428614595	0.964869	9.07E-06	hypermethylated
cg00715696	0.23772	0.46398	0.964799	1.84E-05	hypermethylated
cg15769184	0.15128	0.295194595	0.964445	8.13E-05	hypermethylated
cg21579274	0.1925	0.3756	0.964339	5.51E-05	hypermethylated
cg00569447	0.20163	0.393294595	0.9639	1.89E-05	hypermethylated
cg17019053	0.23178	0.452067568	0.963782	2.45E-05	hypermethylated
cg18811550	0.24005	0.468182703	0.963737	1.80E-05	hypermethylated
cg13486820	0.17999	0.351001081	0.963559	2.27E-05	hypermethylated
cg04228468	0.21126	0.411966486	0.963507	0.000104	hypermethylated
cg08049853	0.25718	0.501490811	0.963445	2.10E-05	hypermethylated
cg26581714	0.25813	0.503317297	0.96337	7.12E-06	hypermethylated
cg00178249	0.25198	0.491323784	0.963365	1.21E-05	hypermethylated
cg18160072	0.12507	0.243851351	0.963266	0.00018	hypermethylated
cg13736376	0.17192	0.335184324	0.963217	8.22E-05	hypermethylated

cg26668276	0.1921	0.374460541	0.962956	8.42E-05	hypermethylated
cg12563372	0.23997	0.467754054	0.962896	0.000112	hypermethylated
cg10625533	0.16398	0.319583243	0.962672	0.000242	hypermethylated
cg01824466	0.30422	0.592891351	0.962653	1.94E-05	hypermethylated
cg06760830	0.22259	0.433767027	0.962532	3.85E-05	hypermethylated
cg00481951	0.16505	0.321631892	0.962507	0.000741	hypermethylated
cg19969606	0.15152	0.295261622	0.962486	9.72E-05	hypermethylated
cg11112161	0.20301	0.395594595	0.962472	0.000283	hypermethylated
cg16857801	0.19195	0.373938378	0.96207	3.57E-05	hypermethylated
cg18184411	0.17947	0.349591351	0.961927	8.42E-05	hypermethylated
cg25297303	0.1501	0.292352432	0.961785	4.82E-05	hypermethylated
cg27013696	0.22452	0.437278378	0.961708	2.05E-06	hypermethylated
cg10568624	0.19895	0.387467027	0.961668	0.000459	hypermethylated
cg15400655	0.19681	0.383097297	0.960907	6.15E-05	hypermethylated
cg07072366	0.15562	0.30289027	0.960768	7.38E-05	hypermethylated
cg18242103	0.20804	0.404863243	0.960574	0.000166	hypermethylated
cg21483110	0.17371	0.337980541	0.960259	1.71E-05	hypermethylated
cg00321480	0.18003	0.350265946	0.960213	0.000399	hypermethylated
cg05828005	0.21168	0.411809189	0.960091	1.89E-05	hypermethylated
cg19395056	0.20815	0.40482973	0.959692	1.80E-05	hypermethylated
cg01354473	0.232	0.451173514	0.959558	2.16E-06	hypermethylated
cg02935338	0.21312	0.414441081	0.959501	3.49E-05	hypermethylated
cg23085662	0.20274	0.394168108	0.95918	3.32E-05	hypermethylated
cg10679688	0.21546	0.418867027	0.959072	5.72E-05	hypermethylated
cg26469608	0.1938	0.376718378	0.958918	4.26E-05	hypermethylated
cg24414788	0.19095	0.371048108	0.958411	2.92E-05	hypermethylated
cg11735997	0.19533	0.379528649	0.958295	8.95E-06	hypermethylated
cg07078225	0.19984	0.388194595	0.957935	0.001875	hypermethylated
cg05258489	0.09908	0.19246	0.957893	0.000165	hypermethylated
cg02065637	0.21672	0.420849189	0.957471	8.22E-05	hypermethylated
cg11282657	0.22042	0.427985405	0.957306	2.51E-05	hypermethylated
cg04188862	0.32775	0.636301622	0.957115	4.24E-06	hypermethylated
cg04674956	0.2502	0.485698919	0.95698	1.18E-05	hypermethylated
cg11827453	0.18615	0.361340541	0.956894	0.000178	hypermethylated
cg00157199	0.28628	0.555377838	0.956043	0.00018	hypermethylated
cg19385090	0.26979	0.523364324	0.955979	2.24E-05	hypermethylated
cg25930591	0.15406	0.298812432	0.955748	7.93E-05	hypermethylated
cg18815647	0.22663	0.439567027	0.955744	1.46E-05	hypermethylated
cg22455694	0.17771	0.344551892	0.955196	1.28E-05	hypermethylated
cg00468146	0.16079	0.311655135	0.954773	0.000271	hypermethylated
cg11174851	0.13814	0.267718378	0.954585	1.46E-05	hypermethylated
cg01257975	0.22015	0.426426486	0.95381	3.53E-05	hypermethylated
cg03107393	0.2388	0.462542162	0.953782	0.000207	hypermethylated
cg17630392	0.20395	0.395035135	0.953765	1.29E-06	hypermethylated
cg17669581	0.2002	0.387736757	0.953636	0.000195	hypermethylated
cg18987335	0.15956	0.309004324	0.953528	5.79E-05	hypermethylated
cg03692563	0.26214	0.507601622	0.953359	5.73E-06	hypermethylated
cg06596654	0.24221	0.468965405	0.953223	6.39E-06	hypermethylated
cg14127859	0.21448	0.415270811	0.953209	0.000135	hypermethylated
cg18560204	0.20985	0.406285405	0.953135	1.08E-05	hypermethylated
cg26299084	0.18233	0.35298	0.953034	0.000517	hypermethylated
cg04247603	0.17382	0.336503243	0.953026	3.36E-05	hypermethylated
cg18263365	0.20924	0.405048649	0.952937	1.99E-05	hypermethylated
cg04637478	0.2056	0.39796973	0.952818	3.00E-05	hypermethylated
cg04467618	0.2341	0.453134595	0.952815	5.28E-06	hypermethylated
cg04268950	0.21152	0.409403243	0.952728	1.06E-05	hypermethylated
cg02026608	0.14131	0.273504865	0.952703	8.73E-05	hypermethylated
cg16656895	0.21078	0.407958919	0.952686	2.39E-06	hypermethylated

cg09232478	0.23686	0.458422162	0.952642	7.47E-05	hypermethylated
cg20414186	0.17145	0.331718378	0.952171	0.000335	hypermethylated
cg13319711	0.23225	0.449301622	0.952006	1.25E-05	hypermethylated
cg26702958	0.1821	0.352268108	0.951943	0.000335	hypermethylated
cg24527098	0.18628	0.360322162	0.951815	6.31E-05	hypermethylated
cg08801795	0.15233	0.294645946	0.951782	1.21E-05	hypermethylated
cg19942495	0.22993	0.44473027	0.951736	6.75E-06	hypermethylated
cg22763680	0.21896	0.423480541	0.951628	2.13E-05	hypermethylated
cg15746583	0.12494	0.241640541	0.951627	0.000378	hypermethylated
cg23546474	0.23453	0.453555135	0.951505	0.000126	hypermethylated
cg00457913	0.1606	0.310545946	0.951335	0.000265	hypermethylated
cg15324651	0.18331	0.354420541	0.951177	1.54E-05	hypermethylated
cg04274288	0.17364	0.33562973	0.950771	3.57E-05	hypermethylated
cg16648632	0.196	0.378819459	0.950657	4.70E-05	hypermethylated
cg11811610	0.18693	0.361234595	0.950438	2.79E-06	hypermethylated
cg01150433	0.20211	0.390487568	0.950136	1.66E-05	hypermethylated
cg09799571	0.21051	0.406687027	0.95003	8.22E-05	hypermethylated
cg03408271	0.16585	0.320404865	0.950017	2.24E-05	hypermethylated
cg17603689	0.2299	0.444142703	0.950017	6.05E-06	hypermethylated
cg02490989	0.1956	0.37786973	0.949983	0.000216	hypermethylated
cg15288618	0.17505	0.338158378	0.949932	5.45E-05	hypermethylated
cg25378939	0.12115	0.234	0.949714	3.32E-05	hypermethylated
cg01157046	0.32883	0.635085405	0.949609	2.10E-06	hypermethylated
cg06738242	0.20261	0.391258919	0.949418	0.000166	hypermethylated
cg01178624	0.3343	0.645509189	0.949294	7.29E-05	hypermethylated
cg25334575	0.20815	0.401855676	0.949054	6.62E-05	hypermethylated
cg15936446	0.22902	0.442129189	0.948994	8.32E-05	hypermethylated
cg26913248	0.18619	0.359405946	0.948839	0.000306	hypermethylated
cg23093589	0.22451	0.433370811	0.948822	0.000107	hypermethylated
cg04981696	0.13531	0.261177297	0.948761	0.000152	hypermethylated
cg03093838	0.19863	0.383377297	0.948681	7.52E-06	hypermethylated
cg03726556	0.2182	0.421045946	0.948327	1.56E-05	hypermethylated
cg04635983	0.21434	0.41359027	0.948301	1.54E-06	hypermethylated
cg17524151	0.10196	0.196735676	0.948255	0.000111	hypermethylated
cg23429510	0.1894	0.365454054	0.948254	8.42E-05	hypermethylated
cg20372690	0.16495	0.318165405	0.947748	2.15E-05	hypermethylated
cg03215422	0.25146	0.485028108	0.947739	2.42E-06	hypermethylated
cg01152019	0.16365	0.315551351	0.947261	0.000327	hypermethylated
cg14713139	0.17766	0.342522162	0.947078	1.46E-05	hypermethylated
cg14631053	0.23875	0.460272432	0.946987	1.48E-05	hypermethylated
cg06792417	0.15696	0.302488108	0.946481	3.95E-05	hypermethylated
cg04044720	0.21814	0.420326486	0.946256	1.26E-05	hypermethylated
cg26333652	0.30152	0.580914054	0.946071	6.39E-06	hypermethylated
cg11972677	0.2263	0.435972432	0.946	4.24E-06	hypermethylated
cg01774704	0.19934	0.383932432	0.945621	2.64E-06	hypermethylated
cg19029181	0.27784	0.535122703	0.945615	3.21E-06	hypermethylated
cg15710198	0.16884	0.325183784	0.945599	0.000155	hypermethylated
cg01898698	0.25607	0.493172432	0.945554	4.48E-06	hypermethylated
cg27552679	0.25029	0.48197027	0.945344	2.78E-05	hypermethylated
cg22614891	0.13676	0.263315135	0.945144	6.01E-05	hypermethylated
cg00785941	0.19996	0.38494973	0.944959	1.94E-05	hypermethylated
cg00502190	0.19882	0.382738919	0.944898	2.10E-05	hypermethylated
cg23930711	0.17188	0.33086973	0.944862	9.49E-05	hypermethylated
cg22979783	0.23496	0.452273514	0.94478	2.07E-05	hypermethylated
cg03889479	0.15549	0.299300541	0.944773	5.86E-05	hypermethylated
cg05321361	0.21248	0.408955135	0.944616	1.15E-05	hypermethylated
cg23605843	0.23406	0.450445946	0.944476	1.06E-05	hypermethylated
cg07609788	0.21018	0.404481622	0.944449	1.75E-05	hypermethylated

cg09821185	0.28296	0.544524324	0.944398	0.000163	hypermethylated
cg05345286	0.19564	0.376451351	0.944262	2.89E-05	hypermethylated
cg01296360	0.15703	0.302034054	0.943671	1.87E-05	hypermethylated
cg21681109	0.17178	0.330331351	0.943352	5.28E-06	hypermethylated
cg14655855	0.18363	0.353081622	0.9432	0.001074	hypermethylated
cg01308258	0.18556	0.356738919	0.942983	4.82E-05	hypermethylated
cg23517116	0.20686	0.397545946	0.942467	2.57E-05	hypermethylated
cg11028201	0.23073	0.443344324	0.942222	0.000189	hypermethylated
cg24430580	0.15702	0.301705946	0.942195	9.49E-05	hypermethylated
cg02126051	0.17693	0.339954054	0.942161	7.03E-05	hypermethylated
cg00181834	0.17408	0.334477838	0.94216	2.15E-05	hypermethylated
cg00896347	0.17699	0.340014595	0.941929	0.0002	hypermethylated
cg02744113	0.17593	0.33791027	0.941639	3.07E-05	hypermethylated
cg22635491	0.26558	0.510071351	0.941553	3.32E-05	hypermethylated
cg16491960	0.27221	0.522782162	0.94149	2.64E-05	hypermethylated
cg22815110	0.16255	0.312178378	0.941487	9.83E-05	hypermethylated
cg06633061	0.17355	0.333119459	0.940688	4.58E-05	hypermethylated
cg25616547	0.24488	0.469963784	0.940475	8.22E-05	hypermethylated
cg06426416	0.24014	0.460775676	0.940189	6.95E-05	hypermethylated
cg25829425	0.16081	0.308418378	0.939532	1.31E-05	hypermethylated
cg07152755	0.19899	0.381625946	0.939463	4.42E-05	hypermethylated
cg12451099	0.20289	0.388968108	0.938954	7.12E-06	hypermethylated
cg27175093	0.22056	0.422716757	0.93852	6.31E-05	hypermethylated
cg14283783	0.19165	0.367301622	0.938491	0.000168	hypermethylated
cg04972566	0.17557	0.336465946	0.938414	5.06E-05	hypermethylated
cg01581018	0.18349	0.351621622	0.938322	6.95E-05	hypermethylated
cg23369632	0.28571	0.547383243	0.938	0.000174	hypermethylated
cg26216629	0.16501	0.316125946	0.937946	7.72E-06	hypermethylated
cg18032969	0.19893	0.381026486	0.93763	6.22E-06	hypermethylated
cg20807545	0.16907	0.323824865	0.937593	0.000464	hypermethylated
cg27567430	0.2002	0.383441622	0.937565	9.27E-05	hypermethylated
cg26525091	0.16914	0.323932973	0.937477	0.000143	hypermethylated
cg13899718	0.23281	0.445765946	0.937133	0.0003	hypermethylated
cg16219603	0.1704	0.326267568	0.93713	2.24E-05	hypermethylated
cg18395499	0.21692	0.41532973	0.937094	3.76E-05	hypermethylated
cg11123720	0.23107	0.442205946	0.936388	0.00012	hypermethylated
cg10417567	0.16211	0.310230811	0.936369	0.000306	hypermethylated
cg01236132	0.22317	0.427078378	0.936358	9.44E-06	hypermethylated
cg12615535	0.2282	0.436701622	0.936349	0.00013	hypermethylated
cg20291049	0.23955	0.458409189	0.936309	1.04E-05	hypermethylated
cg21852117	0.22793	0.436130811	0.93617	2.79E-06	hypermethylated
cg14627089	0.19291	0.369056757	0.935915	0.000148	hypermethylated
cg20219381	0.18525	0.354371892	0.935791	5.86E-05	hypermethylated
cg18843682	0.16657	0.318564324	0.935456	7.56E-05	hypermethylated
cg23563234	0.17682	0.338142703	0.935351	7.47E-05	hypermethylated
cg02964724	0.17191	0.328705946	0.935144	7.47E-05	hypermethylated
cg07105285	0.21277	0.406833514	0.935144	3.90E-06	hypermethylated
cg01629716	0.2026	0.387385946	0.935137	0.000157	hypermethylated
cg07211875	0.14459	0.276456757	0.935086	1.35E-05	hypermethylated
cg06763054	0.20905	0.399597297	0.934699	5.86E-05	hypermethylated
cg04686763	0.12184	0.232877838	0.934586	0.000287	hypermethylated
cg02700626	0.17274	0.33013027	0.934433	4.58E-05	hypermethylated
cg06908778	0.18593	0.355316757	0.934346	4.53E-05	hypermethylated
cg17974166	0.23241	0.444121081	0.934281	9.27E-05	hypermethylated
cg06202276	0.22804	0.435748108	0.934207	0.000112	hypermethylated
cg23999932	0.1779	0.339898919	0.934039	9.60E-05	hypermethylated
cg22505086	0.18201	0.347659459	0.933657	8.03E-05	hypermethylated
cg21610164	0.14322	0.273558919	0.933619	0.000287	hypermethylated

cg17408527	0.23267	0.444403784	0.933586	6.05E-06	hypermethylated
cg14547067	0.18244	0.348441622	0.933495	0.001912	hypermethylated
cg17626960	0.22199	0.423907027	0.933253	0.000148	hypermethylated
cg06602847	0.17586	0.335767568	0.933036	4.82E-05	hypermethylated
cg07323825	0.18763	0.358238378	0.933029	0.000306	hypermethylated
cg02472801	0.19478	0.371883784	0.933006	0.000575	hypermethylated
cg10687131	0.201	0.383650811	0.932598	0.000528	hypermethylated
cg04369341	0.18558	0.35418973	0.932481	0.000178	hypermethylated
cg26068283	0.17266	0.329525405	0.932456	0.000111	hypermethylated
cg04938315	0.21269	0.405864324	0.932245	1.01E-05	hypermethylated
cg12503267	0.13666	0.260743243	0.932039	0.000495	hypermethylated
cg14772925	0.22387	0.427087027	0.931869	7.72E-06	hypermethylated
cg13011388	0.20795	0.396706486	0.931835	0.00032	hypermethylated
cg06896987	0.23464	0.447564865	0.931648	2.27E-05	hypermethylated
cg19456622	0.17801	0.339537297	0.931612	0.000219	hypermethylated
cg18002116	0.25706	0.490274054	0.931483	2.45E-05	hypermethylated
cg00613752	0.18494	0.352702162	0.931393	1.46E-05	hypermethylated
cg14631165	0.14856	0.283254054	0.931051	1.58E-05	hypermethylated
cg27322846	0.18602	0.354652432	0.930948	2.36E-06	hypermethylated
cg03532026	0.27219	0.518885946	0.930803	5.89E-06	hypermethylated
cg11920122	0.17215	0.328161081	0.930738	1.99E-05	hypermethylated
cg05168977	0.18671	0.355905405	0.930695	1.71E-05	hypermethylated
cg19435284	0.17848	0.340035676	0.929924	5.28E-06	hypermethylated
cg01237333	0.24584	0.468326486	0.929795	5.73E-06	hypermethylated
cg14857764	0.238	0.453191351	0.929159	0.000138	hypermethylated
cg11612345	0.18174	0.346056216	0.92913	0.000426	hypermethylated
cg13434352	0.12966	0.246835135	0.928814	3.57E-05	hypermethylated
cg08003628	0.24947	0.474816216	0.928503	0.000234	hypermethylated
cg15996459	0.21114	0.401795676	0.928262	0.000408	hypermethylated
cg18175808	0.17922	0.340975676	0.927937	8.26E-06	hypermethylated
cg00425918	0.16791	0.319404324	0.927696	4.47E-05	hypermethylated
cg24466241	0.22759	0.432915676	0.927649	4.24E-06	hypermethylated
cg06784991	0.21965	0.41778	0.927537	8.37E-06	hypermethylated
cg24541426	0.21574	0.410308649	0.927416	2.18E-05	hypermethylated
cg16208657	0.17699	0.336608108	0.927402	2.39E-05	hypermethylated
cg13209441	0.20055	0.381400541	0.927345	8.03E-05	hypermethylated
cg00769810	0.12386	0.235553514	0.927345	1.42E-05	hypermethylated
cg00229754	0.23278	0.44268	0.927297	2.21E-05	hypermethylated
cg12457773	0.18785	0.357215676	0.927214	8.63E-05	hypermethylated
cg22324567	0.26609	0.505992432	0.927202	7.03E-05	hypermethylated
cg20963227	0.20022	0.380727027	0.927171	1.84E-05	hypermethylated
cg06785213	0.14609	0.277787027	0.927122	0.00013	hypermethylated
cg14056644	0.1819	0.345834595	0.926937	8.37E-06	hypermethylated
cg26398921	0.23402	0.444836757	0.926644	2.04E-05	hypermethylated
cg03284310	0.13344	0.253613514	0.92644	1.71E-05	hypermethylated
cg11848563	0.15183	0.288518919	0.926209	1.21E-05	hypermethylated
cg06049868	0.23983	0.455678378	0.926004	9.07E-06	hypermethylated
cg24951286	0.21571	0.409793514	0.925804	3.32E-05	hypermethylated
cg20996682	0.18227	0.346188649	0.925481	0.000112	hypermethylated
cg08288811	0.21165	0.401979459	0.925441	4.12E-06	hypermethylated
cg00873601	0.29107	0.552818378	0.925439	6.31E-05	hypermethylated
cg18149485	0.17841	0.338814054	0.925297	4.05E-05	hypermethylated
cg05940231	0.1974	0.374796216	0.924984	0.000335	hypermethylated
cg16818395	0.22747	0.431831892	0.924794	6.93E-06	hypermethylated
cg19338524	0.17022	0.323124324	0.924689	5.73E-06	hypermethylated
cg17686487	0.25007	0.474603243	0.92439	0.000108	hypermethylated
cg06656994	0.1511	0.286742162	0.92425	6.95E-05	hypermethylated
cg10737118	0.15053	0.285654595	0.924221	4.82E-05	hypermethylated

cg17198308	0.22458	0.426138919	0.924094	0.000214	hypermethylated
cg13864319	0.16682	0.316396216	0.92344	2.04E-05	hypermethylated
cg26149682	0.23166	0.439344865	0.923345	8.60E-06	hypermethylated
cg26458072	0.17189	0.325975676	0.923279	0.001063	hypermethylated
cg21836370	0.24118	0.457332973	0.923135	3.40E-05	hypermethylated
cg06911084	0.1844	0.349641081	0.923036	0.000117	hypermethylated
cg03469618	0.18961	0.359346486	0.922341	0.00024	hypermethylated
cg09775263	0.21152	0.400865405	0.922324	4.15E-05	hypermethylated
cg12215739	0.28018	0.530907027	0.922105	1.71E-05	hypermethylated
cg06368184	0.17906	0.339296216	0.922102	1.20E-05	hypermethylated
cg11184748	0.22448	0.425320541	0.921964	1.06E-05	hypermethylated
cg14633398	0.17363	0.328956757	0.921882	0.00039	hypermethylated
cg14312649	0.18555	0.351501081	0.921721	6.95E-05	hypermethylated
cg04623265	0.1391	0.263460541	0.921464	6.78E-05	hypermethylated
cg25999578	0.20494	0.388135135	0.921357	6.54E-05	hypermethylated
cg21147648	0.1826	0.345753514	0.921057	1.18E-05	hypermethylated
cg14614211	0.2142	0.405561622	0.920963	1.31E-05	hypermethylated
cg15961744	0.25979	0.491757838	0.920602	9.07E-06	hypermethylated
cg15557878	0.21576	0.408351892	0.920385	4.12E-06	hypermethylated
cg20025656	0.19967	0.377858378	0.920228	4.48E-06	hypermethylated
cg01891252	0.17529	0.331680541	0.920051	2.85E-05	hypermethylated
cg11970349	0.18804	0.355803784	0.920042	8.03E-05	hypermethylated
cg07462756	0.23602	0.446559459	0.919943	5.51E-05	hypermethylated
cg06488443	0.19517	0.369254054	0.919882	7.12E-06	hypermethylated
cg15869649	0.20442	0.386705946	0.919701	3.00E-05	hypermethylated
cg06991392	0.25439	0.481081622	0.91924	0.000358	hypermethylated
cg14742937	0.20885	0.394941622	0.919172	0.0017	hypermethylated
cg17943279	0.21427	0.405164865	0.919079	3.12E-06	hypermethylated
cg06873880	0.1747	0.330323784	0.919001	9.96E-06	hypermethylated
cg07929264	0.22998	0.43484	0.918976	2.36E-06	hypermethylated
cg17080423	0.16294	0.308078919	0.918959	5.35E-06	hypermethylated
cg06865612	0.17592	0.332606486	0.918897	5.73E-06	hypermethylated
cg16209664	0.19509	0.368808108	0.918731	1.05E-05	hypermethylated
cg07341624	0.24347	0.460264324	0.918719	6.05E-06	hypermethylated
cg10606834	0.20873	0.394523784	0.918474	3.40E-05	hypermethylated
cg05854217	0.19537	0.369249189	0.918386	1.99E-05	hypermethylated
cg04765277	0.19747	0.373192973	0.918288	1.62E-05	hypermethylated
cg04917446	0.20349	0.384566486	0.918275	0.001314	hypermethylated
cg16916914	0.24803	0.468717838	0.918205	0.000152	hypermethylated
cg02254574	0.21	0.396783243	0.917962	0.000187	hypermethylated
cg03858673	0.24271	0.458533514	0.917794	6.57E-06	hypermethylated
cg05801246	0.24001	0.453151892	0.9169	1.92E-05	hypermethylated
cg18755846	0.18588	0.350922162	0.91678	8.15E-06	hypermethylated
cg17495715	0.22268	0.420338919	0.916581	5.28E-06	hypermethylated
cg12821804	0.27778	0.524313514	0.916487	7.72E-06	hypermethylated
cg04697262	0.21139	0.39897027	0.916374	0.000168	hypermethylated
cg14051264	0.20493	0.386718378	0.916152	7.72E-06	hypermethylated
cg01609275	0.20167	0.380549189	0.916086	0.000495	hypermethylated
cg06917617	0.21904	0.413261622	0.915861	0.000474	hypermethylated
cg20982412	0.19221	0.362617297	0.915764	1.84E-05	hypermethylated
cg23963802	0.19378	0.365541622	0.915616	7.65E-05	hypermethylated
cg06635722	0.1811	0.341592973	0.915492	8.42E-05	hypermethylated
cg01650137	0.23527	0.443736757	0.915387	0.000354	hypermethylated
cg24442454	0.18192	0.343070811	0.915202	0.000108	hypermethylated
cg07333191	0.29374	0.553837297	0.914922	2.15E-05	hypermethylated
cg13239348	0.20822	0.392525946	0.914679	0.000148	hypermethylated
cg10192198	0.20782	0.391754595	0.914616	1.50E-05	hypermethylated
cg06462796	0.21522	0.405696216	0.914588	6.22E-06	hypermethylated

cg17740322	0.18187	0.34282973	0.914585	0.000575	hypermethylated
cg01120173	0.25171	0.474375135	0.914266	0.000214	hypermethylated
cg26938561	0.23947	0.451182703	0.913867	3.23E-05	hypermethylated
cg00592870	0.10654	0.200692973	0.913595	7.47E-05	hypermethylated
cg26186239	0.22931	0.431936757	0.913521	1.63E-06	hypermethylated
cg24394631	0.22531	0.424343784	0.913322	1.46E-05	hypermethylated
cg05928053	0.23819	0.448523784	0.913072	0.000107	hypermethylated
cg02683197	0.21725	0.409062162	0.912964	0.000195	hypermethylated
cg08681855	0.16497	0.310617838	0.912937	8.84E-05	hypermethylated
cg10171357	0.2728	0.513575676	0.912733	9.05E-05	hypermethylated
cg15222651	0.13574	0.255537838	0.912691	6.62E-05	hypermethylated
cg10425861	0.20961	0.394580541	0.912612	7.12E-05	hypermethylated
cg10803098	0.17464	0.32874973	0.912606	0.000575	hypermethylated
cg25734913	0.17485	0.32913027	0.912541	3.08E-06	hypermethylated
cg18461458	0.14311	0.26927027	0.91193	0.000106	hypermethylated
cg21840976	0.18012	0.338695135	0.911029	0.000256	hypermethylated
cg03656099	0.22983	0.432149189	0.910962	4.26E-05	hypermethylated
cg26495109	0.18424	0.346285405	0.910375	0.000191	hypermethylated
cg20979061	0.25727	0.483540541	0.910354	1.71E-05	hypermethylated
cg01597891	0.15461	0.290570811	0.910256	6.15E-05	hypermethylated
cg24658517	0.29163	0.547891892	0.909752	8.83E-06	hypermethylated
cg10608596	0.26465	0.497073514	0.909374	2.45E-05	hypermethylated
cg24525176	0.18324	0.344156757	0.909331	1.33E-06	hypermethylated
cg15506609	0.32441	0.609204324	0.909108	4.73E-06	hypermethylated
cg10515131	0.18534	0.34802973	0.909036	0.000287	hypermethylated
cg08362273	0.21989	0.412900541	0.909012	4.30E-06	hypermethylated
cg02783918	0.26431	0.496301622	0.908986	1.71E-05	hypermethylated
cg16142855	0.24562	0.461188649	0.908929	8.15E-06	hypermethylated
cg17481117	0.2475	0.46444973	0.908094	5.73E-06	hypermethylated
cg00058879	0.20066	0.376471892	0.907789	2.45E-05	hypermethylated
cg02710296	0.31905	0.598580541	0.907763	4.36E-05	hypermethylated
cg01528028	0.1637	0.307004324	0.907205	5.86E-05	hypermethylated
cg08489674	0.20956	0.392974054	0.907071	0.000152	hypermethylated
cg04865691	0.20295	0.380571892	0.907045	5.00E-06	hypermethylated
cg01530101	0.28786	0.539758919	0.906948	3.59E-06	hypermethylated
cg21123160	0.24468	0.458763784	0.906855	7.47E-05	hypermethylated
cg16555417	0.19038	0.35695027	0.906841	0.00035	hypermethylated
cg16019434	0.27137	0.5088	0.906837	2.71E-05	hypermethylated
cg21784383	0.21788	0.408402162	0.906457	0.000287	hypermethylated
cg05735384	0.24583	0.46078	0.906417	1.94E-05	hypermethylated
cg07918726	0.1948	0.365098378	0.906292	6.01E-05	hypermethylated
cg03724423	0.19582	0.367007027	0.90628	6.62E-05	hypermethylated
cg10675276	0.18885	0.353938378	0.906257	0.000296	hypermethylated
cg02318784	0.23619	0.442587027	0.906013	3.76E-05	hypermethylated
cg07115626	0.20551	0.385035676	0.905784	2.10E-05	hypermethylated
cg24066601	0.21814	0.408665405	0.905666	0.0003	hypermethylated
cg17744293	0.20314	0.380514054	0.905475	8.60E-06	hypermethylated
cg07639287	0.22886	0.428632432	0.905276	8.15E-06	hypermethylated
cg14636534	0.25855	0.48414973	0.90501	1.01E-05	hypermethylated
cg01382414	0.15653	0.293037297	0.904645	0.000191	hypermethylated
cg13578408	0.17106	0.320117838	0.904101	1.31E-05	hypermethylated
cg18049638	0.21386	0.400141622	0.903844	4.20E-05	hypermethylated
cg26843711	0.21542	0.403048649	0.903802	2.04E-05	hypermethylated
cg27649239	0.28898	0.540648649	0.903722	8.42E-05	hypermethylated
cg15134033	0.19892	0.372116216	0.903565	9.07E-06	hypermethylated
cg22479299	0.13895	0.259930811	0.903562	5.79E-05	hypermethylated
cg19767622	0.2123	0.397138378	0.903537	6.70E-05	hypermethylated
cg19352038	0.26291	0.491806486	0.903522	1.12E-05	hypermethylated

cg14354327	0.22767	0.425705946	0.902913	6.75E-06	hypermethylated
cg23768829	0.23698	0.44310973	0.902899	1.46E-05	hypermethylated
cg22309923	0.13058	0.244151351	0.902842	5.43E-06	hypermethylated
cg18826572	0.21595	0.403764324	0.902816	0.000112	hypermethylated
cg15233892	0.15621	0.292027568	0.902618	2.54E-05	hypermethylated
cg11128216	0.24161	0.45157027	0.90227	0.000613	hypermethylated
cg09061632	0.16302	0.304618378	0.901954	2.27E-05	hypermethylated
cg07273051	0.17653	0.329842703	0.901865	8.63E-05	hypermethylated
cg10774586	0.12374	0.23116973	0.901641	0.000148	hypermethylated
cg08944170	0.24126	0.450666486	0.901471	0.000178	hypermethylated
cg09936561	0.23882	0.446011892	0.901159	1.66E-05	hypermethylated
cg06900404	0.16576	0.309534054	0.901002	0.000152	hypermethylated
cg27252164	0.208	0.388332973	0.900711	2.78E-05	hypermethylated
cg24372299	0.16768	0.312978378	0.900352	0.000653	hypermethylated
cg14625581	0.12871	0.240230811	0.900297	0.002574	hypermethylated
cg23754320	0.19237	0.35892973	0.899818	0.000104	hypermethylated
cg00756032	0.18591	0.346836757	0.899652	0.000161	hypermethylated
cg04481096	0.26438	0.493195676	0.899547	7.93E-05	hypermethylated
cg16852704	0.16275	0.303589189	0.899463	1.44E-05	hypermethylated
cg27105390	0.22647	0.422396216	0.899277	2.10E-06	hypermethylated
cg00525823	0.19971	0.37246	0.899179	3.23E-05	hypermethylated
cg08692733	0.25123	0.468497838	0.899034	3.49E-05	hypermethylated
cg09191626	0.21396	0.398995135	0.89903	0.000117	hypermethylated
cg23413924	0.19973	0.372388108	0.898756	1.71E-05	hypermethylated
cg02008154	0.19337	0.360470811	0.898518	1.89E-05	hypermethylated
cg03206681	0.29035	0.541251351	0.898506	6.22E-06	hypermethylated
cg05864140	0.22067	0.411348108	0.898469	5.25E-05	hypermethylated
cg14443467	0.2184	0.40710973	0.898445	3.40E-05	hypermethylated
cg01302656	0.2429	0.452766486	0.898405	8.71E-06	hypermethylated
cg04647982	0.19936	0.371538919	0.898137	2.64E-05	hypermethylated
cg16697438	0.2519	0.469395676	0.897953	8.42E-05	hypermethylated
cg13202751	0.20312	0.378456216	0.897794	3.57E-05	hypermethylated
cg04598774	0.16352	0.304661081	0.897738	2.67E-05	hypermethylated
cg02381539	0.21464	0.399874595	0.897629	0.000274	hypermethylated
cg16036528	0.18026	0.335814595	0.897586	3.00E-05	hypermethylated
cg16458436	0.17884	0.33306	0.897113	0.000119	hypermethylated
cg06808467	0.132	0.24581027	0.897007	4.47E-05	hypermethylated
cg07483462	0.16813	0.313060541	0.896865	1.31E-05	hypermethylated
cg23508813	0.174	0.323872432	0.896338	4.47E-05	hypermethylated
cg01524278	0.22827	0.42482973	0.896143	1.75E-05	hypermethylated
cg04937144	0.20665	0.384558378	0.896013	4.58E-05	hypermethylated
cg12936121	0.21569	0.40134	0.895866	7.84E-05	hypermethylated
cg22280475	0.20775	0.386528649	0.895727	0.000283	hypermethylated
cg11655243	0.28903	0.537702703	0.895589	0.000102	hypermethylated
cg22843446	0.14036	0.261110811	0.89553	2.78E-05	hypermethylated
cg26821745	0.18043	0.33561027	0.895348	4.15E-05	hypermethylated
cg25396728	0.21698	0.403574054	0.895271	0.000152	hypermethylated
cg11229513	0.22666	0.421496216	0.89499	0.000306	hypermethylated
cg00273068	0.14894	0.276891351	0.894589	0.000117	hypermethylated
cg19856705	0.24666	0.458528108	0.894486	0.000551	hypermethylated
cg03320827	0.17035	0.316629189	0.894292	9.49E-05	hypermethylated
cg00551679	0.26334	0.489257838	0.893668	0.000122	hypermethylated
cg17805540	0.24207	0.449707027	0.893561	4.10E-05	hypermethylated
cg24184636	0.20501	0.380772973	0.893237	0.000126	hypermethylated
cg21570988	0.25415	0.472032973	0.893207	1.99E-05	hypermethylated
cg21109744	0.1947	0.361511892	0.89279	0.000234	hypermethylated
cg07930539	0.26913	0.499702703	0.892767	2.15E-05	hypermethylated
cg04050315	0.14393	0.267239459	0.892766	0.000132	hypermethylated

cg04792712	0.2246	0.416987027	0.892645	6.15E-05	hypermethylated
cg23207077	0.20842	0.386912973	0.892515	1.33E-05	hypermethylated
cg18546622	0.21766	0.404051892	0.892464	4.36E-05	hypermethylated
cg08832227	0.26018	0.482794595	0.891899	7.93E-06	hypermethylated
cg09803262	0.25228	0.467925405	0.891253	4.94E-05	hypermethylated
cg27317647	0.25489	0.472616216	0.890794	2.32E-05	hypermethylated
cg19115492	0.16827	0.311931351	0.890451	0.000102	hypermethylated
cg18054172	0.19714	0.365424865	0.890354	0.000209	hypermethylated
cg00547425	0.16708	0.309667568	0.890181	6.15E-05	hypermethylated
cg05262829	0.31067	0.575797838	0.890179	3.85E-05	hypermethylated
cg22622477	0.24435	0.452851892	0.89009	0.00024	hypermethylated
cg04991036	0.19057	0.353155135	0.889981	1.09E-05	hypermethylated
cg12097883	0.20706	0.383705946	0.889952	0.00064	hypermethylated
cg21081803	0.27635	0.511981081	0.889594	5.14E-06	hypermethylated
cg03553587	0.21061	0.390150811	0.889458	2.39E-05	hypermethylated
cg19568544	0.24111	0.446517838	0.889026	5.32E-05	hypermethylated
cg05222924	0.17769	0.329031892	0.888865	0.000495	hypermethylated
cg01649773	0.19586	0.362644324	0.888732	0.000174	hypermethylated
cg27350402	0.23478	0.434565946	0.888266	9.69E-06	hypermethylated
cg22175873	0.25846	0.478336216	0.888084	5.28E-06	hypermethylated
cg01187920	0.23812	0.440594054	0.887761	1.52E-05	hypermethylated
cg26144458	0.22809	0.421945405	0.887453	8.15E-06	hypermethylated
cg18345386	0.17661	0.326704865	0.887421	4.15E-05	hypermethylated
cg11199639	0.26128	0.483304324	0.887335	0.000822	hypermethylated
cg02927346	0.19237	0.355827568	0.887294	0.001619	hypermethylated
cg24576050	0.20407	0.377421622	0.887113	7.32E-06	hypermethylated
cg01071966	0.20382	0.376942703	0.88705	2.36E-06	hypermethylated
cg13711394	0.23758	0.439289189	0.886758	7.21E-05	hypermethylated
cg04031757	0.16204	0.299542162	0.886409	8.83E-06	hypermethylated
cg20047489	0.23943	0.442517297	0.88613	0.000109	hypermethylated
cg06313716	0.15116	0.279346486	0.885979	0.000224	hypermethylated
cg11220565	0.20648	0.381547568	0.885861	0.000212	hypermethylated
cg27604145	0.23094	0.42667027	0.885604	0.000296	hypermethylated
cg08638320	0.23563	0.435296216	0.885474	0.002409	hypermethylated
cg07137606	0.2928	0.540816757	0.885224	5.25E-05	hypermethylated
cg07040303	0.18418	0.34016	0.885097	2.27E-05	hypermethylated
cg22796509	0.1725	0.318551351	0.88493	1.35E-05	hypermethylated
cg13072625	0.19749	0.364458919	0.883977	4.47E-05	hypermethylated
cg10094078	0.17876	0.329825405	0.883679	2.85E-05	hypermethylated
cg23154059	0.21661	0.399619459	0.883527	0.000214	hypermethylated
cg19713947	0.23521	0.433921081	0.883483	0.00054	hypermethylated
cg05222995	0.22235	0.410112973	0.883189	0.000191	hypermethylated
cg16589830	0.25477	0.469895135	0.883143	0.00054	hypermethylated
cg14171414	0.19355	0.356895676	0.882796	1.31E-05	hypermethylated
cg16181396	0.25408	0.468485405	0.882721	1.54E-05	hypermethylated
cg15513743	0.20559	0.378965946	0.882298	2.13E-05	hypermethylated
cg04851169	0.18572	0.342318919	0.882212	1.42E-05	hypermethylated
cg23063647	0.27747	0.511391892	0.882097	0.00048	hypermethylated
cg01729977	0.2043	0.376445946	0.881754	6.62E-05	hypermethylated
cg07730329	0.30107	0.554671351	0.881534	1.04E-05	hypermethylated
cg06893273	0.2802	0.516201081	0.881476	0.000209	hypermethylated
cg26489513	0.22745	0.419001622	0.881406	4.47E-05	hypermethylated
cg00362285	0.25727	0.473904324	0.881313	0.000132	hypermethylated
cg04521004	0.22949	0.422721081	0.881275	3.36E-05	hypermethylated
cg26870126	0.16925	0.311736757	0.881172	1.54E-05	hypermethylated
cg19134568	0.23105	0.425544865	0.881106	4.36E-06	hypermethylated
cg11586570	0.26828	0.494075135	0.880991	8.84E-05	hypermethylated
cg21296230	0.20947	0.385630811	0.880477	0.000155	hypermethylated

cg02762440	0.17004	0.313004324	0.880308	0.000102	hypermethylated
cg26996818	0.23699	0.436162703	0.88004	1.18E-05	hypermethylated
cg07035659	0.20745	0.381725405	0.879772	3.76E-05	hypermethylated
cg16185834	0.20135	0.370494595	0.879747	3.23E-05	hypermethylated
cg01757312	0.24306	0.447237838	0.87973	6.22E-06	hypermethylated
cg26886462	0.2426	0.446368649	0.879656	6.05E-06	hypermethylated
cg01805732	0.16151	0.297164324	0.879637	0.000102	hypermethylated
cg23089840	0.24296	0.446975676	0.879478	0.000142	hypermethylated
cg17444738	0.19695	0.362282162	0.879284	9.82E-06	hypermethylated
cg17389519	0.19754	0.363363784	0.87927	3.57E-05	hypermethylated
cg15936066	0.20072	0.369161622	0.879068	0.003195	hypermethylated
cg05573844	0.29754	0.547121081	0.878777	0.000102	hypermethylated
cg16717122	0.17634	0.324253514	0.878762	6.78E-05	hypermethylated
cg03945800	0.17436	0.320575676	0.878596	0.00071	hypermethylated
cg07115767	0.23853	0.43844	0.878209	1.18E-05	hypermethylated
cg02498722	0.10939	0.201011351	0.877796	2.35E-05	hypermethylated
cg20470857	0.22194	0.407782162	0.877629	1.82E-05	hypermethylated
cg06365535	0.25736	0.472849189	0.877592	3.95E-05	hypermethylated
cg15916004	0.20059	0.368524324	0.87751	0.00012	hypermethylated
cg13062913	0.17513	0.321708649	0.877328	0.000135	hypermethylated
cg09520393	0.19885	0.365277838	0.877314	3.32E-05	hypermethylated
cg25428553	0.23012	0.422718378	0.87731	0.000313	hypermethylated
cg12230728	0.17229	0.31647027	0.877231	0.001041	hypermethylated
cg25152348	0.18871	0.346616216	0.877168	0.000245	hypermethylated
cg06291867	0.19115	0.351051892	0.876979	9.27E-05	hypermethylated
cg18533201	0.24346	0.447016757	0.876644	7.52E-06	hypermethylated
cg08891110	0.21894	0.401876757	0.876218	2.45E-05	hypermethylated
cg02267483	0.23671	0.434423243	0.875981	2.38E-05	hypermethylated
cg09173768	0.27388	0.502527568	0.875659	0.000107	hypermethylated
cg24366557	0.21819	0.400208108	0.875165	1.25E-05	hypermethylated
cg11236429	0.15994	0.29330973	0.874894	0.000251	hypermethylated
cg22584618	0.20557	0.376922162	0.874637	9.05E-05	hypermethylated
cg07862488	0.20189	0.37014973	0.87454	1.75E-05	hypermethylated
cg07986943	0.16518	0.302817297	0.874409	2.92E-05	hypermethylated
cg17645823	0.19807	0.36302973	0.874077	9.96E-06	hypermethylated
cg02461363	0.20106	0.368480541	0.873962	0.000135	hypermethylated
cg07502829	0.24838	0.454837297	0.872802	1.15E-05	hypermethylated
cg02778237	0.26566	0.486326486	0.872344	1.15E-05	hypermethylated
cg14827778	0.18926	0.346418919	0.872148	1.39E-05	hypermethylated
cg20284629	0.15293	0.279903243	0.872057	1.21E-05	hypermethylated
cg10344832	0.21	0.384239459	0.871616	1.18E-05	hypermethylated
cg23725321	0.22107	0.404478378	0.871559	0.000726	hypermethylated
cg16049690	0.23346	0.427103243	0.871409	3.85E-05	hypermethylated
cg08465346	0.28348	0.518596757	0.871366	1.80E-05	hypermethylated
cg24456002	0.29619	0.541845405	0.871358	1.84E-05	hypermethylated
cg12582959	0.19728	0.360876757	0.871262	0.000523	hypermethylated
cg17912835	0.30585	0.559425405	0.871121	1.06E-05	hypermethylated
cg24638849	0.15384	0.281361081	0.870992	2.04E-05	hypermethylated
cg27477419	0.18559	0.339422703	0.870964	0.000143	hypermethylated
cg01089319	0.21685	0.396593514	0.870964	1.75E-05	hypermethylated
cg23679471	0.178	0.325537297	0.870946	6.62E-05	hypermethylated
cg08436419	0.34085	0.62333027	0.87086	0.000102	hypermethylated
cg08536841	0.22868	0.418178919	0.87079	0.00039	hypermethylated
cg25800765	0.21925	0.400875676	0.870578	0.000166	hypermethylated
cg05979020	0.26267	0.480043784	0.869915	5.28E-06	hypermethylated
cg04191403	0.26545	0.48508973	0.869811	0.002231	hypermethylated
cg13369325	0.20607	0.376555676	0.869729	2.89E-05	hypermethylated
cg00646200	0.25335	0.46295027	0.869725	7.12E-05	hypermethylated

cg08510205	0.19166	0.350212432	0.869681	0.000839	hypermethylated
cg16563171	0.20754	0.379192973	0.869543	0.000152	hypermethylated
cg00483304	0.28439	0.519580541	0.869477	0.000102	hypermethylated
cg21971621	0.23329	0.426201081	0.86941	0.00024	hypermethylated
cg19691778	0.2206	0.40298973	0.86931	0.000182	hypermethylated
cg13435649	0.20287	0.370568108	0.869183	0.000365	hypermethylated
cg24454829	0.20905	0.38183027	0.869083	2.39E-05	hypermethylated
cg26858144	0.2062	0.376609189	0.869024	0.000506	hypermethylated
cg18675097	0.27135	0.495587568	0.868985	0.000187	hypermethylated
cg02525108	0.20029	0.365737838	0.868719	2.35E-05	hypermethylated
cg08153621	0.26849	0.490177838	0.868437	7.12E-06	hypermethylated
cg21512447	0.16564	0.302401081	0.868412	6.95E-05	hypermethylated
cg16263367	0.14299	0.260988108	0.86807	0.000113	hypermethylated
cg26375057	0.18625	0.339855676	0.867682	2.38E-05	hypermethylated
cg11245681	0.3301	0.602261081	0.867486	4.73E-06	hypermethylated
cg23097878	0.22944	0.418592973	0.867431	0.002856	hypermethylated
cg00605063	0.20329	0.370858378	0.867329	3.23E-05	hypermethylated
cg07778819	0.23472	0.428192432	0.867319	0.000152	hypermethylated
cg10111115	0.1751	0.319330811	0.866873	0.000245	hypermethylated
cg07851675	0.22559	0.411408649	0.866869	8.26E-06	hypermethylated
cg22827250	0.25757	0.469727027	0.866858	2.36E-06	hypermethylated
cg02488942	0.22482	0.409934595	0.866623	6.22E-06	hypermethylated
cg00752195	0.19859	0.362044865	0.866376	2.04E-05	hypermethylated
cg06970510	0.25484	0.464587027	0.866357	0.000696	hypermethylated
cg18920097	0.24414	0.445031892	0.8662	3.53E-05	hypermethylated
cg02919422	0.22626	0.412404865	0.86608	6.57E-06	hypermethylated
cg11368643	0.237	0.431948649	0.865973	1.21E-05	hypermethylated
cg27009703	0.2914	0.531090811	0.865958	5.58E-06	hypermethylated
cg21932416	0.26588	0.484557838	0.865894	0.00039	hypermethylated
cg03883502	0.15295	0.278703243	0.86567	1.04E-05	hypermethylated
cg03792162	0.25541	0.465265946	0.865241	0.000506	hypermethylated
cg14178794	0.19508	0.355358378	0.865209	0.000163	hypermethylated
cg22603275	0.1646	0.299828108	0.865171	4.47E-05	hypermethylated
cg05259508	0.16014	0.291684324	0.865074	0.000718	hypermethylated
cg18519308	0.2377	0.432952432	0.865067	0.000435	hypermethylated
cg00594408	0.18161	0.33065027	0.864462	1.21E-05	hypermethylated
cg18972885	0.24303	0.442458378	0.864407	0.000155	hypermethylated
cg02409177	0.17791	0.323851351	0.864184	2.32E-05	hypermethylated
cg15196197	0.24529	0.44635027	0.863688	0.000626	hypermethylated
cg00606739	0.20629	0.375297838	0.863362	4.05E-05	hypermethylated
cg06375949	0.23172	0.421494595	0.863132	4.88E-05	hypermethylated
cg05569742	0.21236	0.386237838	0.862977	9.72E-05	hypermethylated
cg19897071	0.24478	0.445048649	0.862477	2.85E-05	hypermethylated
cg10280347	0.17014	0.309261081	0.862103	1.06E-05	hypermethylated
cg13099330	0.17037	0.309604324	0.861754	0.000134	hypermethylated
cg12265623	0.21446	0.389656757	0.861495	1.01E-05	hypermethylated
cg16357224	0.19882	0.361202703	0.861346	0.000163	hypermethylated
cg03895392	0.1528	0.277590811	0.861315	5.06E-05	hypermethylated
cg04935449	0.21513	0.390699459	0.860851	0.000234	hypermethylated
cg04384626	0.19086	0.346610811	0.860802	0.000234	hypermethylated
cg20192747	0.2676	0.485941622	0.860705	9.82E-06	hypermethylated
cg25731359	0.19581	0.35555027	0.860599	0.000109	hypermethylated
cg13316854	0.19194	0.34846	0.860338	0.000242	hypermethylated
cg26278454	0.21191	0.384677297	0.860197	0.000166	hypermethylated
cg10614025	0.16403	0.297755676	0.860169	0.000159	hypermethylated
cg23326689	0.25378	0.46062	0.859999	7.84E-05	hypermethylated
cg10792302	0.3002	0.544758919	0.859694	0.00024	hypermethylated
cg03052128	0.20172	0.366046486	0.859673	5.51E-05	hypermethylated

cg13788027	0.16594	0.301091351	0.85954	9.57E-06	hypermethylated
cg24641186	0.24335	0.441501081	0.859384	8.37E-06	hypermethylated
cg21649089	0.30602	0.555062162	0.859023	4.47E-05	hypermethylated
cg09577391	0.29594	0.536764324	0.858984	0.001368	hypermethylated
cg24394172	0.25876	0.469301081	0.858899	1.12E-05	hypermethylated
cg22455450	0.28616	0.518882703	0.858586	0.000166	hypermethylated
cg03799283	0.25429	0.461084324	0.858556	5.45E-05	hypermethylated
cg05024939	0.22297	0.404286486	0.858528	5.00E-06	hypermethylated
cg09700085	0.09926	0.179946486	0.858284	0.000386	hypermethylated
cg18691434	0.22167	0.401818378	0.85813	5.72E-05	hypermethylated
cg02154252	0.16269	0.294901081	0.858106	6.08E-05	hypermethylated
cg05256483	0.19953	0.36162973	0.857908	0.000245	hypermethylated
cg16655791	0.17819	0.322925405	0.857785	1.87E-05	hypermethylated
cg06073351	0.17328	0.314014054	0.857724	0.000382	hypermethylated
cg02925039	0.22293	0.403984324	0.857709	3.00E-05	hypermethylated
cg24427992	0.196	0.355165946	0.85764	8.84E-05	hypermethylated
cg03909902	0.12114	0.219512432	0.857627	0.00028	hypermethylated
cg11704005	0.31347	0.568023243	0.857623	1.04E-05	hypermethylated
cg10244368	0.1816	0.329052973	0.857556	0.001188	hypermethylated
cg02841155	0.16158	0.292661081	0.856982	0.000445	hypermethylated
cg01261007	0.16914	0.306254595	0.856514	5.06E-05	hypermethylated
cg14356396	0.16149	0.292394054	0.856469	0.000145	hypermethylated
cg20248516	0.26264	0.475534054	0.856462	2.15E-05	hypermethylated
cg23983435	0.16852	0.305094054	0.856334	2.21E-05	hypermethylated
cg03547797	0.29895	0.541070811	0.855913	6.08E-05	hypermethylated
cg02501779	0.23241	0.420584324	0.855723	3.07E-05	hypermethylated
cg03039002	0.17799	0.322086486	0.855652	0.000741	hypermethylated
cg09651522	0.24317	0.440030811	0.855639	3.15E-05	hypermethylated
cg14345012	0.26507	0.479608108	0.855483	1.15E-05	hypermethylated
cg14068328	0.16621	0.300711892	0.855375	0.000408	hypermethylated
cg09775260	0.1553	0.280919459	0.855099	0.000189	hypermethylated
cg14934413	0.232	0.419603784	0.854903	3.40E-05	hypermethylated
cg23448348	0.23412	0.423417297	0.854832	0.000613	hypermethylated
cg12758687	0.21471	0.388302162	0.85479	1.31E-05	hypermethylated
cg06220449	0.17001	0.307441081	0.85469	0.000435	hypermethylated
cg17966560	0.33917	0.613337838	0.854673	9.66E-07	hypermethylated
cg00101712	0.20776	0.375695135	0.854645	0.0002	hypermethylated
cg06832449	0.22111	0.399739459	0.854296	0.000102	hypermethylated
cg05371578	0.27851	0.503452973	0.854128	3.26E-06	hypermethylated
cg04935434	0.2247	0.405991351	0.853449	3.76E-05	hypermethylated
cg23097402	0.24589	0.444276216	0.853444	0.000191	hypermethylated
cg01644927	0.15861	0.286564865	0.853378	2.15E-05	hypermethylated
cg25555059	0.26606	0.480633514	0.853186	0.000306	hypermethylated
cg19284211	0.21419	0.386858378	0.852914	2.71E-05	hypermethylated
cg01729491	0.22957	0.414607027	0.85281	3.67E-05	hypermethylated
cg14879760	0.27343	0.4938	0.852755	4.64E-05	hypermethylated
cg26142965	0.20809	0.375784865	0.852699	6.54E-05	hypermethylated
cg01073178	0.10998	0.19860973	0.852695	0.000185	hypermethylated
cg04730685	0.25126	0.453715135	0.852606	5.19E-05	hypermethylated
cg04455430	0.2069	0.373551892	0.852375	5.28E-06	hypermethylated
cg15929797	0.3825	0.690497838	0.852177	1.31E-05	hypermethylated
cg00320094	0.31649	0.571282703	0.852045	0.000365	hypermethylated
cg21141234	0.15571	0.281056216	0.851997	2.32E-05	hypermethylated
cg13154880	0.17917	0.323332432	0.851689	5.79E-05	hypermethylated
cg17931641	0.17029	0.307196757	0.851169	7.03E-05	hypermethylated
cg19639898	0.21786	0.392947027	0.850934	3.76E-05	hypermethylated
cg25950278	0.2179	0.393011351	0.850905	0.000142	hypermethylated
cg23262218	0.23882	0.430707027	0.850783	1.30E-05	hypermethylated

cg08258922	0.18513	0.333864865	0.850726	0.000138	hypermethylated
cg20370678	0.25106	0.452751351	0.850687	0.000104	hypermethylated
cg05444816	0.2226	0.401425405	0.850678	3.23E-05	hypermethylated
cg04554928	0.22645	0.408359459	0.850647	6.39E-06	hypermethylated
cg25600766	0.20815	0.375355676	0.850635	0.000191	hypermethylated
cg00998124	0.21189	0.382061622	0.85049	0.000142	hypermethylated
cg12787323	0.24596	0.443487568	0.85047	0.000293	hypermethylated
cg25952581	0.21631	0.389909189	0.850038	2.32E-05	hypermethylated
cg17137424	0.26132	0.470993514	0.84989	0.000163	hypermethylated
cg27343616	0.15306	0.275868108	0.849881	3.19E-05	hypermethylated
cg19909239	0.20062	0.361452973	0.849343	4.42E-05	hypermethylated
cg01727145	0.30641	0.55204973	0.849335	7.03E-06	hypermethylated
cg04961101	0.25664	0.462375676	0.849319	2.36E-06	hypermethylated
cg02829688	0.20622	0.371517838	0.849247	1.04E-05	hypermethylated
cg05637536	0.23388	0.421300541	0.849081	4.48E-06	hypermethylated
cg21769619	0.2107	0.379505405	0.84893	1.15E-05	hypermethylated
cg24700462	0.17387	0.313109189	0.848657	3.23E-05	hypermethylated
cg20233073	0.18864	0.339593514	0.848173	0.000157	hypermethylated
cg24122498	0.26737	0.481222703	0.847867	1.60E-05	hypermethylated
cg07362328	0.18475	0.332482703	0.847705	0.0002	hypermethylated
cg03514351	0.21268	0.382697838	0.847521	6.95E-05	hypermethylated
cg09559189	0.30174	0.542890811	0.847356	2.10E-05	hypermethylated
cg01382864	0.25227	0.453805405	0.847105	5.51E-05	hypermethylated
cg07250162	0.20084	0.361206486	0.846777	3.15E-05	hypermethylated
cg25317941	0.2179	0.391861081	0.846676	7.12E-05	hypermethylated
cg07271394	0.18635	0.33506973	0.846447	7.52E-06	hypermethylated
cg13347970	0.19675	0.353723243	0.846257	0.00017	hypermethylated
cg19005707	0.21004	0.377608649	0.846228	4.70E-05	hypermethylated
cg07260592	0.26731	0.480538919	0.846139	4.26E-05	hypermethylated
cg16969368	0.23101	0.415277297	0.84612	0.000129	hypermethylated
cg00422918	0.16759	0.301147568	0.845535	1.31E-05	hypermethylated
cg03906434	0.25263	0.453916216	0.8454	0.000613	hypermethylated
cg08589354	0.22745	0.408642162	0.845289	0.000132	hypermethylated
cg12969193	0.30022	0.539376216	0.845272	4.58E-05	hypermethylated
cg23519626	0.22928	0.411918378	0.845248	0.000191	hypermethylated
cg09544728	0.33446	0.600856757	0.845187	0.002502	hypermethylated
cg17884843	0.21005	0.377343243	0.845145	1.01E-05	hypermethylated
cg02892388	0.24275	0.436063243	0.845066	0.000132	hypermethylated
cg06082548	0.1468	0.263697838	0.845034	7.12E-05	hypermethylated
cg02646491	0.25523	0.458419459	0.84487	1.80E-05	hypermethylated
cg14473344	0.19842	0.356375135	0.844839	3.95E-05	hypermethylated
cg24114014	0.31619	0.567883243	0.844803	1.09E-05	hypermethylated
cg05000994	0.27736	0.498122162	0.84474	0.000115	hypermethylated
cg14241370	0.16816	0.301995135	0.844691	0.000104	hypermethylated
cg16235582	0.26977	0.484468108	0.844672	3.44E-05	hypermethylated
cg05577810	0.21538	0.38678973	0.844665	0.000551	hypermethylated
cg21011616	0.21194	0.380609189	0.844654	0.000148	hypermethylated
cg14611112	0.33516	0.601858919	0.844575	6.01E-05	hypermethylated
cg19537719	0.32536	0.58416973	0.844351	0.000152	hypermethylated
cg04460847	0.2105	0.377935676	0.84432	6.31E-05	hypermethylated
cg10265690	0.16043	0.288027568	0.844263	7.20E-05	hypermethylated
cg02598872	0.19565	0.351258919	0.84426	6.15E-05	hypermethylated
cg14355134	0.20955	0.376078378	0.843739	0.000138	hypermethylated
cg11668749	0.24038	0.431235135	0.843158	3.00E-05	hypermethylated
cg12011136	0.20212	0.362587568	0.843117	6.08E-05	hypermethylated
cg09408917	0.20663	0.370654054	0.843024	2.39E-05	hypermethylated
cg04175027	0.27024	0.484632432	0.84265	6.15E-05	hypermethylated
cg14891410	0.29183	0.523262162	0.842406	0.000117	hypermethylated

cg06675478	0.20619	0.369695676	0.842364	1.12E-05	hypermethylated
cg12606911	0.32171	0.576455676	0.841449	4.76E-05	hypermethylated
cg03239638	0.10523	0.188551892	0.841416	0.000274	hypermethylated
cg10848640	0.27241	0.488071892	0.841314	6.05E-06	hypermethylated
cg08463758	0.24546	0.439750811	0.841198	3.40E-05	hypermethylated
cg14427009	0.25882	0.463654595	0.841101	5.43E-06	hypermethylated
cg18000029	0.19977	0.357819459	0.840892	1.75E-05	hypermethylated
cg12590600	0.21215	0.379983243	0.840851	3.04E-05	hypermethylated
cg03602029	0.25496	0.456549189	0.840499	1.84E-05	hypermethylated
cg07335915	0.19062	0.341325405	0.840448	4.00E-05	hypermethylated
cg20969194	0.17831	0.319116757	0.839697	0.000104	hypermethylated
cg20038591	0.21455	0.38395027	0.839606	4.70E-05	hypermethylated
cg11479165	0.19082	0.341472432	0.839557	0.000101	hypermethylated
cg13564742	0.2183	0.390640541	0.83953	0.000178	hypermethylated
cg10543982	0.17744	0.317500541	0.839428	3.85E-05	hypermethylated
cg06707978	0.1899	0.339734054	0.839166	2.10E-05	hypermethylated
cg04839289	0.22596	0.404163784	0.838873	1.12E-05	hypermethylated
cg10923662	0.29953	0.535741622	0.838837	0.001949	hypermethylated
cg00419702	0.21271	0.380407568	0.838658	0.00028	hypermethylated
cg00034468	0.24651	0.4408	0.838478	6.22E-06	hypermethylated
cg04271218	0.17513	0.313109189	0.83824	9.38E-05	hypermethylated
cg08162222	0.17092	0.305549189	0.838083	0.000142	hypermethylated
cg02109484	0.252	0.450456757	0.837965	6.78E-05	hypermethylated
cg08346922	0.2477	0.44277027	0.837964	1.66E-05	hypermethylated
cg01962146	0.19306	0.344857838	0.836952	6.08E-05	hypermethylated
cg24818566	0.19819	0.354004865	0.836885	2.78E-05	hypermethylated
cg06755825	0.20224	0.361187027	0.836678	5.65E-05	hypermethylated
cg00187692	0.38616	0.689477297	0.836304	0.001164	hypermethylated
cg10472919	0.33376	0.595796757	0.836009	7.72E-06	hypermethylated
cg26479630	0.19432	0.346877838	0.835993	3.49E-05	hypermethylated
cg16458866	0.25612	0.457155135	0.835864	0.000594	hypermethylated
cg15439725	0.29389	0.524552973	0.835812	0.000613	hypermethylated
cg09473315	0.26108	0.465953514	0.835694	5.72E-05	hypermethylated
cg07822928	0.201	0.358643784	0.835356	0.000166	hypermethylated
cg15588215	0.20094	0.358503243	0.835221	9.49E-05	hypermethylated
cg09670535	0.24185	0.431462703	0.835123	1.12E-05	hypermethylated
cg18252039	0.23922	0.426706486	0.834906	1.28E-05	hypermethylated
cg10124710	0.26597	0.474028649	0.833711	3.57E-05	hypermethylated
cg19358442	0.18845	0.335861622	0.833685	0.000104	hypermethylated
cg15608397	0.21051	0.375100541	0.833389	6.86E-05	hypermethylated
cg00346208	0.26695	0.475428649	0.832659	2.51E-05	hypermethylated
cg03350439	0.19629	0.349583243	0.832649	2.51E-05	hypermethylated
cg05053440	0.25513	0.454295676	0.832399	0.000122	hypermethylated
cg03330485	0.21131	0.376255676	0.832352	9.05E-05	hypermethylated
cg04428163	0.28046	0.499376216	0.832332	1.82E-05	hypermethylated
cg15668533	0.19341	0.344376757	0.832325	8.83E-06	hypermethylated
cg00088797	0.34441	0.61322973	0.832301	3.49E-05	hypermethylated
cg00561194	0.31628	0.563014054	0.831969	2.95E-06	hypermethylated
cg26654798	0.2448	0.435742162	0.831871	3.40E-05	hypermethylated
cg14334441	0.17803	0.316886486	0.831846	7.38E-05	hypermethylated
cg15422005	0.207	0.36841027	0.831683	9.49E-05	hypermethylated
cg24340081	0.25929	0.461445405	0.831593	0.000107	hypermethylated
cg12078229	0.22765	0.405123784	0.831545	2.04E-05	hypermethylated
cg21389309	0.21693	0.386003784	0.831385	0.00097	hypermethylated
cg17160984	0.27613	0.491323784	0.831326	9.72E-05	hypermethylated
cg16953816	0.37604	0.669085405	0.831304	0.000772	hypermethylated
cg21621538	0.24442	0.434892973	0.831298	0.000245	hypermethylated
cg03598754	0.12681	0.225626486	0.831268	0.000749	hypermethylated

cg18482892	0.20585	0.366251351	0.831241	0.001635	hypermethylated
cg23642747	0.21047	0.374394054	0.830943	0.000224	hypermethylated
cg09619146	0.28326	0.503819459	0.83078	2.15E-05	hypermethylated
cg06668065	0.29794	0.529895135	0.830685	0.000262	hypermethylated
cg06896909	0.30994	0.551226486	0.830656	1.42E-05	hypermethylated
cg01927745	0.25442	0.452431351	0.830487	5.43E-06	hypermethylated
cg26123256	0.2513	0.446849189	0.830377	0.000107	hypermethylated
cg00992850	0.26152	0.46496	0.830185	6.46E-05	hypermethylated
cg25781755	0.27929	0.496503784	0.830041	0.00044	hypermethylated
cg11230940	0.28084	0.499225405	0.829943	2.45E-05	hypermethylated
cg14811102	0.18751	0.333284865	0.829788	0.001141	hypermethylated
cg19855470	0.20285	0.360543243	0.829759	2.49E-06	hypermethylated
cg03086707	0.27233	0.484022703	0.829719	0.00048	hypermethylated
cg10343742	0.20767	0.369099459	0.829717	2.49E-06	hypermethylated
cg12409982	0.21109	0.375147027	0.829598	7.12E-05	hypermethylated
cg00055986	0.2314	0.411220541	0.829523	0.000464	hypermethylated
cg05021643	0.25557	0.454156757	0.829472	1.11E-05	hypermethylated
cg18541254	0.24137	0.428894054	0.829375	5.73E-06	hypermethylated
cg20884298	0.18678	0.331854595	0.829211	0.000195	hypermethylated
cg05596328	0.2089	0.371081081	0.828922	8.42E-05	hypermethylated
cg27631817	0.25955	0.461009189	0.828783	0.000126	hypermethylated
cg26356061	0.24466	0.434547027	0.828734	0.000102	hypermethylated
cg21293934	0.23274	0.413374054	0.828729	0.000335	hypermethylated
cg00708598	0.22694	0.403058919	0.82868	5.31E-05	hypermethylated
cg07211044	0.25373	0.450585946	0.828508	0.000135	hypermethylated
cg02919960	0.20842	0.37008973	0.828381	1.04E-05	hypermethylated
cg25573227	0.26633	0.472842703	0.828145	0.000112	hypermethylated
cg12651914	0.27892	0.495102703	0.827876	0.000214	hypermethylated
cg23578346	0.25073	0.445032973	0.827778	3.11E-05	hypermethylated
cg27540865	0.26915	0.477645946	0.827531	0.000204	hypermethylated
cg22494858	0.17881	0.317300541	0.827423	0.000107	hypermethylated
cg01621054	0.15622	0.277207568	0.827387	3.62E-05	hypermethylated
cg20119106	0.20696	0.367223243	0.827305	9.49E-05	hypermethylated
cg14612428	0.21378	0.379282703	0.827147	3.32E-05	hypermethylated
cg02943604	0.24472	0.434095676	0.826881	2.64E-05	hypermethylated
cg22275864	0.27282	0.483878919	0.826697	1.84E-05	hypermethylated
cg11935147	0.20712	0.36733027	0.826611	7.74E-05	hypermethylated
cg03506028	0.30873	0.547471892	0.826439	9.31E-06	hypermethylated
cg19747232	0.17578	0.311697297	0.826375	0.00101	hypermethylated
cg06966811	0.27852	0.493821081	0.826207	2.79E-06	hypermethylated
cg15954353	0.30588	0.542329189	0.826203	0.003914	hypermethylated
cg04837832	0.26081	0.462354054	0.825999	6.48E-06	hypermethylated
cg01565438	0.25984	0.460611892	0.825928	5.19E-05	hypermethylated
cg03300268	0.14373	0.254721622	0.82556	9.69E-06	hypermethylated
cg21934311	0.17446	0.309178919	0.825546	3.07E-05	hypermethylated
cg02518135	0.23152	0.4102	0.825191	3.04E-05	hypermethylated
cg15726426	0.22375	0.39641027	0.825107	1.89E-05	hypermethylated
cg22668218	0.29126	0.515989189	0.825033	0.000166	hypermethylated
cg14563954	0.24314	0.430625946	0.824648	6.46E-05	hypermethylated
cg18534176	0.19007	0.336625946	0.824616	1.01E-05	hypermethylated
cg04787888	0.28097	0.497558378	0.82445	0.000166	hypermethylated
cg12031863	0.26863	0.475686486	0.824391	0.000219	hypermethylated
cg09325156	0.1641	0.290536216	0.824143	0.000214	hypermethylated
cg23032674	0.22243	0.393724865	0.823836	7.84E-05	hypermethylated
cg15812020	0.26986	0.477645946	0.82373	1.89E-05	hypermethylated
cg00784153	0.27483	0.486314054	0.823349	7.56E-05	hypermethylated
cg03950614	0.21091	0.373193514	0.823296	6.31E-05	hypermethylated
cg20347139	0.25013	0.442536757	0.823119	4.82E-05	hypermethylated

cg10511988	0.2763	0.488705405	0.822729	0.0006	hypermethylated
cg22212237	0.18532	0.327772432	0.822676	4.05E-05	hypermethylated
cg03244696	0.17275	0.305479459	0.82239	0.000154	hypermethylated
cg17526175	0.25404	0.449225405	0.822384	0.001288	hypermethylated
cg23676302	0.23292	0.411855676	0.822304	0.000342	hypermethylated
cg25452071	0.18638	0.329556216	0.822278	5.86E-05	hypermethylated
cg00507008	0.28297	0.500306486	0.822163	9.27E-05	hypermethylated
cg09528449	0.23402	0.413708108	0.821981	0.000109	hypermethylated
cg04624228	0.12852	0.227195135	0.821939	0.002777	hypermethylated
cg15991405	0.31858	0.563108649	0.821758	3.57E-05	hypermethylated
cg16798252	0.22538	0.398317838	0.821561	2.45E-05	hypermethylated
cg24250393	0.19547	0.345455135	0.821551	5.72E-05	hypermethylated
cg01658421	0.24632	0.43526	0.821344	3.67E-05	hypermethylated
cg21476673	0.23781	0.420058919	0.820782	1.48E-05	hypermethylated
cg22389137	0.19897	0.351442703	0.820739	5.79E-05	hypermethylated
cg07716052	0.24731	0.436744865	0.82047	5.28E-06	hypermethylated
cg08206318	0.24491	0.432404324	0.820129	1.84E-05	hypermethylated
cg12459904	0.23659	0.417628108	0.81983	0.000191	hypermethylated
cg24549912	0.26601	0.469558378	0.819824	0.000324	hypermethylated
cg24586758	0.2566	0.452938378	0.819794	0.000681	hypermethylated
cg03368099	0.26816	0.473305405	0.819677	0.000358	hypermethylated
cg04157263	0.23536	0.415209189	0.818969	3.67E-05	hypermethylated
cg18404374	0.31363	0.553224865	0.818802	2.92E-05	hypermethylated
cg00965023	0.21159	0.373210811	0.818719	2.32E-05	hypermethylated
cg14243778	0.28977	0.511032973	0.818508	8.03E-05	hypermethylated
cg14855519	0.30295	0.534267027	0.818481	2.48E-05	hypermethylated
cg15372603	0.25137	0.443261622	0.818346	0.000214	hypermethylated
cg10536898	0.39644	0.699073514	0.818342	2.79E-06	hypermethylated
cg08066129	0.22	0.387795135	0.817791	8.13E-05	hypermethylated
cg10806820	0.20521	0.361651351	0.817499	1.48E-05	hypermethylated
cg11796455	0.26254	0.462644865	0.817368	3.23E-05	hypermethylated
cg12864185	0.22624	0.398642703	0.817242	8.94E-05	hypermethylated
cg22227345	0.20165	0.355314054	0.817241	7.29E-05	hypermethylated
cg18777299	0.28489	0.501916216	0.817042	4.42E-05	hypermethylated
cg19702785	0.19497	0.343469189	0.816929	1.35E-05	hypermethylated
cg01304890	0.16363	0.288241081	0.816839	5.19E-05	hypermethylated
cg25221239	0.31477	0.554401081	0.816632	2.24E-05	hypermethylated
cg02282631	0.32248	0.567903784	0.816437	0.000653	hypermethylated
cg22331862	0.26111	0.45981027	0.816381	3.15E-05	hypermethylated
cg26682068	0.20713	0.364731351	0.816298	1.58E-05	hypermethylated
cg25365260	0.20171	0.355174595	0.816246	9.27E-05	hypermethylated
cg07520506	0.35901	0.632094054	0.816115	7.84E-05	hypermethylated
cg23026602	0.17754	0.312569189	0.816031	0.000313	hypermethylated
cg14950829	0.29801	0.524645946	0.815983	1.31E-05	hypermethylated
cg06809252	0.31015	0.546005946	0.815951	0.000454	hypermethylated
cg05236677	0.16835	0.29636	0.815887	0.000219	hypermethylated
cg21366688	0.38599	0.679324324	0.815537	0.000517	hypermethylated
cg26885268	0.28845	0.507559459	0.815256	5.00E-05	hypermethylated
cg06953773	0.17999	0.316683784	0.815126	0.000212	hypermethylated
cg19120389	0.16386	0.288246486	0.814839	0.000454	hypermethylated
cg12196232	0.21132	0.371694054	0.814686	2.10E-05	hypermethylated
cg04080041	0.222	0.390444324	0.814557	2.57E-05	hypermethylated
cg24113409	0.26942	0.473818378	0.814477	2.83E-06	hypermethylated
cg18500733	0.1985	0.349058378	0.814329	5.00E-05	hypermethylated
cg22490780	0.34605	0.608363784	0.813954	0.000262	hypermethylated
cg17310600	0.27055	0.475525946	0.813629	4.82E-05	hypermethylated
cg12571423	0.21152	0.371754054	0.813554	0.000134	hypermethylated
cg19049194	0.24281	0.426735676	0.813515	2.21E-05	hypermethylated

cg13113525	0.1626	0.285747027	0.813411	0.000848	hypermethylated
cg04982308	0.26939	0.473383784	0.813314	0.000187	hypermethylated
cg11665991	0.19827	0.348381622	0.813202	0.000403	hypermethylated
cg23705224	0.1877	0.329749189	0.81294	0.000313	hypermethylated
cg04302194	0.17996	0.316137838	0.812877	0.000107	hypermethylated
cg21255438	0.21938	0.385327568	0.812653	2.91E-06	hypermethylated
cg02676375	0.19618	0.344561081	0.812582	9.52E-07	hypermethylated
cg03236397	0.21848	0.383724324	0.812569	0.000256	hypermethylated
cg05043461	0.18512	0.32508973	0.812377	8.63E-05	hypermethylated
cg09917979	0.17928	0.314834054	0.812377	0.000221	hypermethylated
cg10101521	0.20012	0.35142973	0.812371	4.15E-05	hypermethylated
cg10850791	0.16234	0.285082162	0.812359	0.000674	hypermethylated
cg16196812	0.21704	0.381117838	0.812276	2.71E-05	hypermethylated
cg19165390	0.24121	0.423518919	0.812137	1.58E-05	hypermethylated
cg14509153	0.17848	0.313325405	0.811899	0.000435	hypermethylated
cg01028849	0.23831	0.418310811	0.811736	6.01E-05	hypermethylated
cg24715245	0.25068	0.44001027	0.81169	7.93E-06	hypermethylated
cg18120446	0.28209	0.495107568	0.811586	3.85E-05	hypermethylated
cg23331484	0.28585	0.501653514	0.811433	9.95E-05	hypermethylated
cg13492364	0.23723	0.416170811	0.810889	0.000166	hypermethylated
cg06776146	0.20836	0.365354595	0.810219	4.88E-05	hypermethylated
cg12539415	0.29595	0.518923243	0.810168	0.000138	hypermethylated
cg19335130	0.24756	0.434039459	0.810048	7.56E-05	hypermethylated
cg22488857	0.20691	0.362722162	0.809862	0.0003	hypermethylated
cg11256607	0.18715	0.328081622	0.80986	6.54E-05	hypermethylated
cg14548272	0.2215	0.388280541	0.809793	8.94E-05	hypermethylated
cg11618529	0.20463	0.358662703	0.80961	1.62E-05	hypermethylated
cg08793792	0.20842	0.365295676	0.809571	0.000202	hypermethylated
cg23677657	0.29195	0.511574595	0.809223	1.31E-05	hypermethylated
cg12074182	0.21179	0.371021622	0.808869	3.69E-06	hypermethylated
cg13242000	0.2343	0.410367568	0.80856	5.06E-05	hypermethylated
cg20016673	0.26296	0.460438919	0.808166	0.00012	hypermethylated
cg16667279	0.19643	0.343917297	0.808046	9.95E-05	hypermethylated
cg04915044	0.3033	0.530863784	0.807596	7.84E-05	hypermethylated
cg27596495	0.17356	0.303763784	0.807515	0.000166	hypermethylated
cg12517027	0.15448	0.270281622	0.807043	0.001751	hypermethylated
cg13193239	0.21079	0.368747027	0.806825	1.84E-05	hypermethylated
cg15235999	0.28541	0.499177838	0.806518	0.00039	hypermethylated
cg02646021	0.19288	0.33727027	0.806202	0.000107	hypermethylated
cg05290820	0.25799	0.451078919	0.806065	0.001803	hypermethylated
cg22686132	0.37154	0.6496	0.806034	0.00513	hypermethylated
cg13130031	0.22205	0.388161081	0.805771	2.96E-05	hypermethylated
cg05096751	0.20845	0.364385405	0.805764	6.78E-05	hypermethylated
cg05771369	0.26454	0.462318919	0.805403	3.44E-05	hypermethylated
cg27598107	0.29337	0.5127	0.805394	0.001301	hypermethylated
cg06232807	0.29562	0.516628108	0.805382	1.62E-05	hypermethylated
cg19777067	0.29033	0.507324865	0.805216	0.000464	hypermethylated
cg22729726	0.31779	0.555302162	0.805199	6.84E-06	hypermethylated
cg08715595	0.26585	0.464516216	0.805116	6.75E-06	hypermethylated
cg12869949	0.17737	0.309876216	0.80493	4.88E-05	hypermethylated
cg03970615	0.18015	0.314726486	0.8049	9.49E-05	hypermethylated
cg24864241	0.21839	0.381454595	0.804605	0.000143	hypermethylated
cg25782229	0.26577	0.464191351	0.804541	0.000126	hypermethylated
cg06012215	0.26946	0.470605946	0.804448	7.32E-06	hypermethylated
cg22920700	0.2516	0.43937027	0.804305	0.000187	hypermethylated
cg02037307	0.30544	0.533371892	0.804253	7.29E-05	hypermethylated
cg12133554	0.27619	0.482292973	0.804249	0.000115	hypermethylated
cg02400433	0.1781	0.311001081	0.804232	6.08E-05	hypermethylated

cg21776577	0.36345	0.634567027	0.804016	0.000528	hypermethylated
cg03807316	0.22666	0.39569027	0.803842	7.12E-05	hypermethylated
cg02228815	0.14476	0.252705946	0.803797	8.84E-05	hypermethylated
cg20348858	0.19885	0.347034054	0.803397	7.52E-06	hypermethylated
cg08202494	0.25845	0.450925946	0.803005	9.07E-06	hypermethylated
cg15126179	0.26137	0.455949189	0.80278	4.26E-05	hypermethylated
cg19988426	0.36159	0.630742703	0.802697	0.000417	hypermethylated
cg22719241	0.32834	0.572732432	0.802671	0.000382	hypermethylated
cg15499368	0.20587	0.359062162	0.8025	1.18E-05	hypermethylated
cg03925294	0.21364	0.372572973	0.802341	2.02E-05	hypermethylated
cg06688989	0.22831	0.398091892	0.802107	0.000209	hypermethylated
cg21501525	0.25051	0.43678	0.802039	0.000182	hypermethylated
cg23995914	0.278	0.484708649	0.802033	3.32E-05	hypermethylated
cg15641657	0.20413	0.355894054	0.80196	0.000274	hypermethylated
cg23509641	0.21875	0.381382703	0.801956	0.000523	hypermethylated
cg20643029	0.23731	0.413739459	0.80195	7.29E-05	hypermethylated
cg27024272	0.19505	0.340035135	0.80184	0.000563	hypermethylated
cg23993425	0.24054	0.419244324	0.801514	6.31E-05	hypermethylated
cg06645033	0.30089	0.524217297	0.800929	4.26E-05	hypermethylated
cg15607672	0.28341	0.493749189	0.800888	0.000256	hypermethylated
cg23548627	0.26295	0.458093514	0.800854	3.95E-05	hypermethylated
cg09829551	0.18632	0.324576216	0.800775	0.000382	hypermethylated
cg03151469	0.24127	0.420262162	0.800641	6.62E-05	hypermethylated
cg18555440	0.17785	0.309764324	0.80051	4.94E-05	hypermethylated
cg18194945	0.26626	0.463617297	0.800099	1.80E-05	hypermethylated
cg27246129	0.24867	0.43295027	0.799969	0.003316	hypermethylated
cg04622802	0.24942	0.434231351	0.799887	0.000134	hypermethylated
cg15801340	0.2079	0.36191027	0.799742	4.30E-06	hypermethylated
cg10890302	0.31496	0.54826973	0.799717	0.002599	hypermethylated
cg06429887	0.37488	0.652456757	0.799453	9.38E-05	hypermethylated
cg26094789	0.29674	0.516451351	0.799433	5.35E-06	hypermethylated
cg01310750	0.2493	0.433841622	0.799286	0.000107	hypermethylated
cg04194947	0.3763	0.654820541	0.799216	5.65E-05	hypermethylated
cg05213896	0.27482	0.478190811	0.799099	7.12E-05	hypermethylated
cg04941278	0.19454	0.338497838	0.79908	0.000111	hypermethylated
cg16387141	0.20502	0.356714595	0.799006	5.79E-05	hypermethylated
cg25648436	0.32266	0.561350811	0.798888	5.19E-05	hypermethylated
cg15736524	0.28703	0.499342703	0.798829	0.000138	hypermethylated
cg17442776	0.23132	0.40236973	0.798632	1.15E-05	hypermethylated
cg20426959	0.22328	0.388269189	0.798203	1.09E-05	hypermethylated
cg10423771	0.24891	0.432772973	0.797986	1.52E-05	hypermethylated
cg00703513	0.21635	0.376126486	0.797851	2.92E-05	hypermethylated
cg04452311	0.22531	0.391681081	0.797768	3.81E-05	hypermethylated
cg06870213	0.36439	0.633422162	0.797684	0.000117	hypermethylated
cg20080624	0.24274	0.421944324	0.797641	3.57E-05	hypermethylated
cg21948716	0.17218	0.299242703	0.797398	4.47E-05	hypermethylated
cg12765028	0.24283	0.421986486	0.79725	5.31E-05	hypermethylated
cg15049101	0.42251	0.734177297	0.797143	5.19E-05	hypermethylated
cg17054759	0.16766	0.291324324	0.797088	0.000178	hypermethylated
cg13574488	0.23782	0.413192432	0.796944	7.74E-05	hypermethylated
cg17928883	0.25336	0.44008973	0.796609	0.00071	hypermethylated
cg02853616	0.26229	0.455576216	0.79653	0.000129	hypermethylated
cg09818081	0.18692	0.324663243	0.796523	6.01E-05	hypermethylated
cg19203333	0.26058	0.452436757	0.79599	0.000193	hypermethylated
cg21542223	0.22581	0.392048108	0.795921	3.67E-05	hypermethylated
cg22486834	0.27572	0.478648649	0.795763	0.007667	hypermethylated
cg00011482	0.16761	0.290895135	0.795391	3.95E-05	hypermethylated
cg01544851	0.21446	0.372169189	0.79525	2.05E-06	hypermethylated

cg18583910	0.23921	0.41504	0.794973	0.000112	hypermethylated
cg15503722	0.28533	0.495028108	0.794879	1.80E-05	hypermethylated
cg20982735	0.31038	0.538475676	0.794846	8.52E-05	hypermethylated
cg19143209	0.19892	0.345007568	0.79444	0.000229	hypermethylated
cg26295328	0.17809	0.308820541	0.794162	8.42E-05	hypermethylated
cg00852924	0.26431	0.458315676	0.794111	0.000107	hypermethylated
cg04892836	0.18678	0.323837838	0.793932	7.12E-05	hypermethylated
cg05618934	0.34197	0.592817297	0.793718	4.86E-06	hypermethylated
cg20160127	0.23002	0.398727568	0.793644	2.04E-05	hypermethylated
cg03490331	0.23567	0.408512973	0.793614	2.46E-06	hypermethylated
cg04645150	0.30935	0.536071351	0.793185	5.45E-05	hypermethylated
cg07637213	0.22114	0.383184324	0.793079	2.56E-06	hypermethylated
cg17594256	0.33677	0.583519459	0.793017	0.000667	hypermethylated
cg07011913	0.21878	0.379045946	0.792892	0.000259	hypermethylated
cg04819775	0.20167	0.349385946	0.792825	7.93E-05	hypermethylated
cg02524946	0.18862	0.326751892	0.792713	0.000198	hypermethylated
cg06758591	0.25231	0.437007027	0.792459	2.45E-05	hypermethylated
cg06672469	0.15614	0.270380541	0.792151	0.000229	hypermethylated
cg23489627	0.18579	0.321712973	0.792101	0.000866	hypermethylated
cg15452970	0.30913	0.535223784	0.791929	2.56E-06	hypermethylated
cg02992546	0.26377	0.456608649	0.791678	7.84E-05	hypermethylated
cg03259243	0.21156	0.366187027	0.791514	0.000117	hypermethylated
cg03463411	0.24752	0.428428649	0.79151	0.000234	hypermethylated
cg01805282	0.22337	0.386556757	0.791245	0.00017	hypermethylated
cg08151623	0.34354	0.594496216	0.79119	9.05E-05	hypermethylated
cg00748589	0.23317	0.403482703	0.791125	8.22E-05	hypermethylated
cg26690075	0.2516	0.435357838	0.79107	0.000104	hypermethylated
cg11122944	0.2143	0.370678919	0.790538	0.000417	hypermethylated
cg05671386	0.21793	0.37693027	0.790433	4.26E-05	hypermethylated
cg00989365	0.25737	0.445005946	0.789981	5.12E-05	hypermethylated
cg11322252	0.21934	0.379101622	0.789416	1.37E-05	hypermethylated
cg00006081	0.22793	0.393901081	0.789243	1.54E-05	hypermethylated
cg00116092	0.25954	0.448456216	0.78901	0.000104	hypermethylated
cg00010742	0.16866	0.291377838	0.788773	5.93E-05	hypermethylated
cg06055873	0.26571	0.459029189	0.788733	2.21E-05	hypermethylated
cg18030587	0.18526	0.319985405	0.788455	6.57E-06	hypermethylated
cg09176539	0.21573	0.372612973	0.788451	1.18E-05	hypermethylated
cg00219282	0.20862	0.360327027	0.788429	7.38E-05	hypermethylated
cg14005211	0.2328	0.402003243	0.788116	0.000224	hypermethylated
cg17284168	0.41546	0.717417297	0.788103	0.000399	hypermethylated
cg21647257	0.21256	0.367045946	0.788091	5.86E-05	hypermethylated
cg09156097	0.28864	0.498374054	0.787958	0.000178	hypermethylated
cg00558702	0.31186	0.538434595	0.787873	1.99E-06	hypermethylated
cg23856413	0.19825	0.342248649	0.787724	7.65E-05	hypermethylated
cg01693350	0.33858	0.584334595	0.787298	6.62E-05	hypermethylated
cg14405924	0.23185	0.400108649	0.7872	7.20E-07	hypermethylated
cg12884605	0.19321	0.333399459	0.787082	0.00094	hypermethylated
cg01139861	0.22684	0.391378919	0.786891	0.00078	hypermethylated
cg22283925	0.25171	0.434236216	0.786718	1.28E-05	hypermethylated
cg06710927	0.19195	0.331051351	0.786324	0.000102	hypermethylated
cg04213384	0.40588	0.69992	0.786137	0.001839	hypermethylated
cg24411961	0.35514	0.612362703	0.785999	2.04E-05	hypermethylated
cg14410016	0.2968	0.511710811	0.785838	1.21E-05	hypermethylated
cg02932314	0.22293	0.384334054	0.78577	1.58E-05	hypermethylated
cg12060499	0.29922	0.515811351	0.785637	0.000234	hypermethylated
cg13649056	0.19015	0.327711351	0.785288	0.000163	hypermethylated
cg16072304	0.2716	0.468052973	0.785188	0.000103	hypermethylated
cg02823803	0.19362	0.333631351	0.785027	4.94E-05	hypermethylated

cg03321133	0.20476	0.352823243	0.785012	5.19E-05	hypermethylated
cg27047406	0.23655	0.407564865	0.784885	7.47E-05	hypermethylated
cg00905524	0.24164	0.416296757	0.784753	4.80E-06	hypermethylated
cg27142059	0.23658	0.407504324	0.784487	1.15E-05	hypermethylated
cg08010865	0.40448	0.696272973	0.783585	0.000361	hypermethylated
cg12111714	0.39096	0.672839459	0.783241	3.32E-05	hypermethylated
cg04961235	0.2694	0.463568649	0.783033	3.95E-05	hypermethylated
cg12619940	0.19762	0.339887568	0.782329	0.000137	hypermethylated
cg24079051	0.2208	0.379614595	0.781795	0.000187	hypermethylated
cg10509254	0.22869	0.393068649	0.781388	3.11E-05	hypermethylated
cg08658787	0.36566	0.628444865	0.781283	2.02E-05	hypermethylated
cg06934774	0.25978	0.446426486	0.781132	3.15E-05	hypermethylated
cg05544500	0.23824	0.409405946	0.781116	1.21E-05	hypermethylated
cg07645844	0.25258	0.434020541	0.781023	1.15E-05	hypermethylated
cg05316605	0.19642	0.337499459	0.780943	0.000262	hypermethylated
cg08809260	0.16724	0.287226486	0.780269	0.000107	hypermethylated
cg02327997	0.26199	0.449926486	0.780178	2.95E-06	hypermethylated
cg15641789	0.16226	0.278643784	0.780115	2.04E-05	hypermethylated
cg26293019	0.29619	0.50858973	0.779979	0.000426	hypermethylated
cg25286482	0.32147	0.551985946	0.779947	0.000174	hypermethylated
cg14472366	0.32553	0.558889189	0.779772	1.28E-05	hypermethylated
cg23289079	0.34131	0.585974054	0.779754	4.58E-05	hypermethylated
cg24713204	0.26326	0.451972973	0.779748	0.000135	hypermethylated
cg05999049	0.27925	0.479421622	0.779738	0.000126	hypermethylated
cg10297473	0.22893	0.393028649	0.779728	9.72E-05	hypermethylated
cg08767286	0.3024	0.519161622	0.779726	1.80E-05	hypermethylated
cg10635895	0.40565	0.696404324	0.77969	0.000161	hypermethylated
cg08206623	0.19416	0.333309189	0.779615	0.00035	hypermethylated
cg16546442	0.22	0.377602703	0.779366	1.46E-05	hypermethylated
cg18568589	0.24677	0.42347027	0.779094	9.49E-05	hypermethylated
cg07109453	0.3632	0.62318	0.778885	0.001768	hypermethylated
cg13544333	0.17272	0.296324865	0.778745	8.84E-05	hypermethylated
cg17809748	0.24666	0.42314973	0.778644	1.99E-05	hypermethylated
cg14351904	0.24597	0.421951892	0.778596	3.76E-05	hypermethylated
cg15998779	0.26112	0.447832432	0.778246	0.0002	hypermethylated
cg26056703	0.15492	0.265675135	0.77814	5.79E-05	hypermethylated
cg20945085	0.32697	0.560701622	0.778075	3.49E-05	hypermethylated
cg12883523	0.23413	0.401328649	0.777474	5.58E-05	hypermethylated
cg11029716	0.234	0.401104865	0.777471	0.00029	hypermethylated
cg24790628	0.21884	0.374941622	0.77679	4.30E-06	hypermethylated
cg15949805	0.25989	0.445238919	0.776679	0.001074	hypermethylated
cg10942078	0.20067	0.343742162	0.776502	0.000102	hypermethylated
cg00624617	0.2304	0.394603784	0.776264	2.71E-05	hypermethylated
cg15699085	0.30429	0.520927027	0.775634	1.21E-05	hypermethylated
cg25051331	0.18492	0.316526486	0.775425	4.53E-05	hypermethylated
cg13858139	0.20121	0.344379459	0.775297	7.29E-05	hypermethylated
cg12157673	0.30476	0.52150973	0.775021	0.000245	hypermethylated
cg27641532	0.33089	0.566176757	0.774901	3.49E-06	hypermethylated
cg01320433	0.29541	0.505452973	0.774858	0.000464	hypermethylated
cg15092219	0.24915	0.426295135	0.774838	2.57E-05	hypermethylated
cg21929183	0.36739	0.628598378	0.774826	0.000219	hypermethylated
cg14546076	0.27392	0.468665405	0.774804	4.82E-05	hypermethylated
cg07838483	0.43463	0.743592973	0.774725	1.09E-05	hypermethylated
cg24172509	0.36331	0.621551351	0.774673	0.000229	hypermethylated
cg14150378	0.29231	0.500057838	0.774596	0.000382	hypermethylated
cg12670347	0.28172	0.481838378	0.774287	6.15E-05	hypermethylated
cg18948125	0.22391	0.382880541	0.773975	4.53E-05	hypermethylated
cg17920479	0.20672	0.353466486	0.773895	0.000185	hypermethylated

cg14641472	0.218	0.372658919	0.773528	0.00044	hypermethylated
cg11666559	0.17935	0.306561081	0.773397	0.000303	hypermethylated
cg05073843	0.19635	0.335595676	0.773297	3.40E-05	hypermethylated
cg13570972	0.22692	0.387836216	0.773264	0.000464	hypermethylated
cg04094811	0.24025	0.410608108	0.773226	0.000157	hypermethylated
cg26069745	0.26137	0.446632973	0.772996	3.00E-05	hypermethylated
cg12354122	0.23147	0.395457838	0.772699	0.000232	hypermethylated
cg20185461	0.26434	0.451511892	0.772369	1.94E-05	hypermethylated
cg06894134	0.22429	0.383029189	0.772089	7.72E-06	hypermethylated
cg03512426	0.23224	0.396592973	0.772043	1.99E-05	hypermethylated
cg06032349	0.21628	0.369297297	0.771882	3.90E-06	hypermethylated
cg21349625	0.18201	0.310760541	0.771786	6.54E-05	hypermethylated
cg08486875	0.24516	0.418473514	0.771413	4.18E-06	hypermethylated
cg09540961	0.19353	0.33034	0.771394	0.000268	hypermethylated
cg14663451	0.26684	0.455430811	0.771257	5.19E-05	hypermethylated
cg06306198	0.2413	0.411827027	0.771211	8.60E-06	hypermethylated
cg13219590	0.22065	0.376504865	0.770909	0.001541	hypermethylated
cg20801476	0.26965	0.460056757	0.770724	6.57E-06	hypermethylated
cg09656363	0.21865	0.372843243	0.769946	1.94E-05	hypermethylated
cg14298200	0.30194	0.514791892	0.769727	0.000145	hypermethylated
cg10165801	0.24564	0.418729189	0.769472	0.000733	hypermethylated
cg14095959	0.29649	0.505348108	0.769294	2.39E-05	hypermethylated
cg08254359	0.30458	0.519078919	0.769133	0.000163	hypermethylated
cg27029821	0.26893	0.4583	0.769062	5.14E-06	hypermethylated
cg14144305	0.22515	0.383574054	0.768619	6.62E-05	hypermethylated
cg00400448	0.2985	0.508525946	0.76859	0.001262	hypermethylated
cg12198813	0.2914	0.496368649	0.768411	7.52E-06	hypermethylated
cg04503600	0.27877	0.474689189	0.767908	6.01E-05	hypermethylated
cg01085125	0.23778	0.404872973	0.767842	5.65E-05	hypermethylated
cg03776662	0.21101	0.359228108	0.767589	0.000313	hypermethylated
cg21945930	0.22299	0.379584865	0.767443	3.00E-05	hypermethylated
cg02380585	0.25496	0.433974595	0.76734	8.94E-05	hypermethylated
cg13585776	0.24885	0.423563243	0.767301	2.49E-06	hypermethylated
cg12057127	0.26739	0.455113514	0.767281	0.002965	hypermethylated
cg12782180	0.29978	0.510216216	0.767205	5.72E-05	hypermethylated
cg04562589	0.281	0.47824	0.767165	1.75E-05	hypermethylated
cg19508622	0.31497	0.536028649	0.767096	0.000435	hypermethylated
cg07805777	0.34922	0.594231351	0.766889	3.07E-05	hypermethylated
cg12810084	0.22664	0.385617297	0.766767	0.000195	hypermethylated
cg16101739	0.23374	0.397696757	0.766764	0.000142	hypermethylated
cg03337093	0.22025	0.37474	0.766748	2.21E-05	hypermethylated
cg05116382	0.26207	0.445857838	0.766632	4.76E-05	hypermethylated
cg21317965	0.25595	0.435403243	0.76649	0.000166	hypermethylated
cg26277754	0.33829	0.57536973	0.766229	7.93E-06	hypermethylated
cg13846270	0.29104	0.494867027	0.765823	0.000109	hypermethylated
cg08506931	0.23181	0.394115135	0.765674	6.15E-05	hypermethylated
cg20950167	0.36047	0.612787568	0.765508	3.19E-05	hypermethylated
cg11907797	0.27742	0.471596757	0.765482	4.00E-05	hypermethylated
cg22589628	0.1598	0.271627027	0.76536	0.000268	hypermethylated
cg24560494	0.18114	0.307895676	0.765336	8.94E-05	hypermethylated
cg00508975	0.23248	0.394992432	0.764718	0.000142	hypermethylated
cg15504461	0.31516	0.535444865	0.764654	6.66E-06	hypermethylated
cg24355907	0.21111	0.358562162	0.764228	7.62E-06	hypermethylated
cg04689048	0.19239	0.326678378	0.763837	0.001031	hypermethylated
cg05971894	0.19387	0.329182703	0.763799	0.000148	hypermethylated
cg22083053	0.19057	0.323575676	0.763782	7.65E-05	hypermethylated
cg18334915	0.25716	0.436631892	0.763751	5.58E-05	hypermethylated
cg25023596	0.34316	0.582624865	0.763686	0.001875	hypermethylated

cg19229402	0.18798	0.319122162	0.76353	0.000237	hypermethylated
cg21270847	0.45247	0.767970811	0.763229	0.001188	hypermethylated
cg07659054	0.3414	0.579288108	0.762818	0.00044	hypermethylated
cg00910715	0.2173	0.368675135	0.762662	2.79E-06	hypermethylated
cg06380356	0.19297	0.327316757	0.762311	4.47E-05	hypermethylated
cg08770761	0.21259	0.360501622	0.761932	3.00E-05	hypermethylated
cg01700290	0.19756	0.334896216	0.761423	3.95E-05	hypermethylated
cg19519747	0.2798	0.474254595	0.761266	8.37E-06	hypermethylated
cg00642359	0.2431	0.412007027	0.761119	9.72E-05	hypermethylated
cg22171539	0.22068	0.373992432	0.761053	2.78E-05	hypermethylated
cg18419271	0.30818	0.522255676	0.760983	0.00049	hypermethylated
cg06495131	0.207	0.350774595	0.760913	0.000245	hypermethylated
cg04779860	0.21025	0.356281622	0.760912	6.95E-05	hypermethylated
cg01416712	0.25668	0.434916757	0.760768	0.000283	hypermethylated
cg06020207	0.2415	0.409179459	0.760711	3.00E-05	hypermethylated
cg23361092	0.19534	0.330905405	0.760431	2.42E-05	hypermethylated
cg06654537	0.21966	0.372046486	0.760211	3.67E-05	hypermethylated
cg13836098	0.25406	0.430157838	0.759697	0.001511	hypermethylated
cg10806146	0.27663	0.46828973	0.759444	0.001912	hypermethylated
cg25560247	0.24225	0.410081081	0.759413	1.62E-05	hypermethylated
cg23767994	0.32448	0.549195135	0.759189	1.25E-05	hypermethylated
cg02612397	0.29678	0.502301081	0.759159	0.00039	hypermethylated
cg04611437	0.24089	0.407694054	0.759112	0.000517	hypermethylated
cg22856324	0.29632	0.501397297	0.758798	0.000104	hypermethylated
cg10490742	0.22972	0.388703243	0.758793	1.21E-05	hypermethylated
cg14428048	0.25197	0.426238378	0.758409	5.65E-05	hypermethylated
cg09442828	0.21908	0.370529189	0.758129	0.000163	hypermethylated
cg07170824	0.25854	0.437123784	0.757654	0.000245	hypermethylated
cg01289769	0.48118	0.813458919	0.757493	3.07E-05	hypermethylated
cg12878812	0.24148	0.408225405	0.757462	1.82E-05	hypermethylated
cg10205928	0.21794	0.368422703	0.757431	6.15E-05	hypermethylated
cg18689332	0.29169	0.493070811	0.757359	0.0003	hypermethylated
cg07015195	0.26772	0.452534595	0.757303	0.000189	hypermethylated
cg07240873	0.22724	0.384031351	0.757007	8.34E-07	hypermethylated
cg03196097	0.2637	0.445570811	0.756757	0.000142	hypermethylated
cg12149299	0.31482	0.531892432	0.756607	1.28E-05	hypermethylated
cg13702421	0.21929	0.370475676	0.756539	1.84E-05	hypermethylated
cg07150045	0.22995	0.38841027	0.756261	0.000797	hypermethylated
cg25372357	0.25103	0.423968108	0.756096	0.000501	hypermethylated
cg05457768	0.24252	0.409564324	0.755986	9.95E-05	hypermethylated
cg00629427	0.22954	0.38754	0.7556	4.26E-05	hypermethylated
cg16076997	0.15821	0.267096216	0.755519	4.70E-05	hypermethylated
cg26841967	0.22804	0.384984865	0.755515	4.36E-05	hypermethylated
cg03583111	0.18881	0.318738919	0.75544	0.000119	hypermethylated
cg03500056	0.17592	0.296955135	0.755325	0.001603	hypermethylated
cg15990972	0.28774	0.48566	0.755181	5.86E-05	hypermethylated
cg14975429	0.23073	0.389434595	0.755175	2.81E-05	hypermethylated
cg20557801	0.2244	0.378676216	0.754892	0.000324	hypermethylated
cg06632310	0.20933	0.353193514	0.75468	0.000112	hypermethylated
cg22300957	0.2251	0.379732432	0.754417	1.99E-05	hypermethylated
cg04470072	0.32286	0.544626486	0.754358	0.000102	hypermethylated
cg15852516	0.20796	0.350789189	0.754298	2.92E-05	hypermethylated
cg18609078	0.32326	0.545199459	0.754089	2.71E-05	hypermethylated
cg07895132	0.32565	0.549198378	0.754005	0.000108	hypermethylated
cg22727965	0.20157	0.339927568	0.753946	1.89E-05	hypermethylated
cg02803819	0.3118	0.525803243	0.753902	0.002318	hypermethylated
cg27366162	0.22341	0.37674	0.753875	0.002649	hypermethylated
cg04439218	0.27669	0.466552973	0.75377	1.09E-05	hypermethylated

cg23034362	0.2088	0.352041622	0.753624	0.000152	hypermethylated
cg04255201	0.18075	0.304674595	0.753274	0.000185	hypermethylated
cg08175609	0.23405	0.394485405	0.753155	0.000251	hypermethylated
cg05171584	0.20943	0.352972973	0.75309	1.50E-05	hypermethylated
cg08259413	0.21516	0.362609189	0.753006	5.58E-05	hypermethylated
cg14128735	0.29493	0.497025405	0.752947	2.32E-05	hypermethylated
cg23709172	0.22114	0.372562162	0.752521	5.43E-06	hypermethylated
cg15331781	0.17481	0.294495135	0.752456	7.65E-05	hypermethylated
cg05290058	0.27895	0.469923243	0.752419	6.62E-05	hypermethylated
cg01257889	0.30475	0.513291892	0.752153	0.000132	hypermethylated
cg01922095	0.24361	0.410284865	0.752053	0.000123	hypermethylated
cg14176752	0.18333	0.308696757	0.751747	0.000287	hypermethylated
cg01889237	0.24646	0.414911892	0.751451	1.75E-05	hypermethylated
cg15856829	0.24984	0.420590811	0.751413	0.001395	hypermethylated
cg06392753	0.40076	0.674499459	0.751079	0.003773	hypermethylated
cg07584066	0.29586	0.497904865	0.750955	0.000152	hypermethylated
cg14395444	0.27052	0.455182162	0.750709	0.000209	hypermethylated
cg02689863	0.24377	0.410133514	0.750573	1.44E-05	hypermethylated
cg05784193	0.44717	0.752287027	0.75046	0.000123	hypermethylated
cg17508941	0.36053	0.606471351	0.75032	1.94E-05	hypermethylated
cg13779907	0.2047	0.344302703	0.750166	4.36E-05	hypermethylated
cg05270938	0.23536	0.395781081	0.749833	9.95E-05	hypermethylated
cg05012697	0.19579	0.329238919	0.749828	7.38E-05	hypermethylated
cg19521279	0.30954	0.520507568	0.749793	0.004248	hypermethylated
cg04456238	0.33347	0.56068973	0.749646	5.12E-05	hypermethylated
cg18515031	0.33577	0.564542162	0.749608	0.000262	hypermethylated
cg18593317	0.31897	0.536276757	0.749557	8.84E-05	hypermethylated
cg06530563	0.20103	0.337985946	0.749552	0.001074	hypermethylated
cg05323533	0.28153	0.473268649	0.749371	0.000324	hypermethylated
cg26509328	0.19911	0.33469027	0.749261	5.86E-05	hypermethylated
cg05926722	0.32821	0.551684865	0.749225	1.62E-05	hypermethylated
cg14201424	0.31106	0.522843243	0.749186	0.000506	hypermethylated
cg25334928	0.20735	0.348521081	0.749178	0.000681	hypermethylated
cg26601317	0.25315	0.425486486	0.749121	7.12E-06	hypermethylated
cg17293161	0.28096	0.472154595	0.748895	0.00017	hypermethylated
cg22997113	0.38694	0.650198919	0.748771	8.60E-06	hypermethylated
cg09744068	0.25205	0.423519459	0.748718	7.29E-05	hypermethylated
cg10005230	0.21158	0.355493514	0.74862	0.000563	hypermethylated
cg18516557	0.37197	0.62472	0.748023	1.33E-05	hypermethylated
cg26618441	0.31862	0.53506	0.747864	0.00035	hypermethylated
cg01350680	0.22284	0.374189189	0.74776	8.03E-05	hypermethylated
cg18008247	0.3283	0.551104324	0.747311	0.000155	hypermethylated
cg03934782	0.25562	0.429061622	0.747184	0.000182	hypermethylated
cg06894334	0.23676	0.397378378	0.747088	4.36E-05	hypermethylated
cg08979895	0.30852	0.517818378	0.747082	4.26E-05	hypermethylated
cg18969232	0.26908	0.451519459	0.746753	5.72E-05	hypermethylated
cg21010475	0.17569	0.294805946	0.746734	0.000174	hypermethylated
cg09059319	0.25817	0.43318	0.746645	1.64E-05	hypermethylated
cg05748163	0.27441	0.460425405	0.746634	1.04E-05	hypermethylated
cg24718465	0.18422	0.309078378	0.746543	0.000681	hypermethylated
cg03349582	0.306	0.513368108	0.746462	0.000296	hypermethylated
cg10475970	0.23454	0.393474595	0.746436	1.15E-05	hypermethylated
cg10702770	0.29236	0.490467568	0.746412	0.00017	hypermethylated
cg04583232	0.2057	0.345078378	0.746382	3.04E-06	hypermethylated
cg06902607	0.23688	0.397261622	0.745933	8.04E-06	hypermethylated
cg20459495	0.3055	0.512301622	0.745821	0.000145	hypermethylated
cg17781669	0.26224	0.439752432	0.745804	7.12E-05	hypermethylated
cg00409684	0.23374	0.391930811	0.745694	7.12E-05	hypermethylated

cg18409732	0.26072	0.437143784	0.745607	0.000157	hypermethylated
cg17566175	0.32348	0.542346486	0.745538	6.15E-05	hypermethylated
cg12648074	0.28202	0.472801622	0.745438	4.26E-05	hypermethylated
cg02620013	0.23463	0.393318378	0.74531	4.26E-05	hypermethylated
cg16632715	0.19998	0.335212973	0.745222	9.07E-06	hypermethylated
cg12467933	0.19735	0.330765405	0.745052	0.000229	hypermethylated
cg08892899	0.21925	0.367438919	0.744928	0.000117	hypermethylated
cg08231493	0.28574	0.478718378	0.744474	1.99E-05	hypermethylated
cg20100445	0.20992	0.351677297	0.744413	0.000148	hypermethylated
cg00221382	0.2497	0.418302162	0.74435	0.000129	hypermethylated
cg16790291	0.23313	0.390528649	0.744294	0.000145	hypermethylated
cg04609576	0.22695	0.380171892	0.744277	0.000306	hypermethylated
cg02856338	0.24757	0.414672973	0.744138	0.000324	hypermethylated
cg15137566	0.2108	0.352983784	0.743727	0.000143	hypermethylated
cg08169778	0.27542	0.461155676	0.743621	0.000138	hypermethylated
cg15730491	0.34185	0.572357838	0.743554	0.001452	hypermethylated
cg16754788	0.42984	0.719610811	0.743417	0.000464	hypermethylated
cg26007189	0.3065	0.513112973	0.743389	0.000681	hypermethylated
cg01841306	0.20414	0.341734054	0.743315	0.00421	hypermethylated
cg07499553	0.21915	0.366830811	0.743196	0.00032	hypermethylated
cg07957294	0.31855	0.533209189	0.743182	0.000209	hypermethylated
cg22524061	0.35744	0.598255135	0.74306	0.000417	hypermethylated
cg03739573	0.23837	0.398964865	0.743059	0.000193	hypermethylated
cg01716975	0.29649	0.496040541	0.742475	0.00028	hypermethylated
cg04175111	0.30362	0.507854595	0.742149	0.000335	hypermethylated
cg05791173	0.28336	0.473920541	0.742009	1.87E-05	hypermethylated
cg25687358	0.34044	0.569370811	0.741968	0.001	hypermethylated
cg26770907	0.33849	0.566048108	0.741811	2.92E-05	hypermethylated
cg14497054	0.34465	0.576307027	0.741706	4.93E-06	hypermethylated
cg08654915	0.25142	0.420407027	0.741687	1.25E-05	hypermethylated
cg16808373	0.25284	0.422775135	0.741666	1.54E-05	hypermethylated
cg26709300	0.3128	0.523028108	0.741648	0.000163	hypermethylated
cg22478121	0.21452	0.358692973	0.741637	0.000102	hypermethylated
cg27615378	0.31277	0.522966486	0.741616	1.77E-06	hypermethylated
cg17177995	0.208	0.347764324	0.741526	0.000403	hypermethylated
cg06660395	0.24257	0.405544865	0.74146	0.000145	hypermethylated
cg07502389	0.22279	0.372442162	0.741332	0.000161	hypermethylated
cg13718539	0.25818	0.431470811	0.740886	3.81E-05	hypermethylated
cg02442900	0.25898	0.432794054	0.74084	0.00017	hypermethylated
cg00503840	0.26635	0.445101081	0.74081	2.23E-06	hypermethylated
cg25580656	0.46418	0.775546486	0.740529	0.000772	hypermethylated
cg16961816	0.28	0.467717838	0.740212	1.50E-05	hypermethylated
cg01255417	0.24806	0.414352973	0.740171	1.84E-05	hypermethylated
cg11260097	0.25182	0.420589189	0.740019	9.19E-06	hypermethylated
cg02952984	0.38154	0.637138378	0.739772	0.001164	hypermethylated
cg01080998	0.19373	0.32351027	0.739764	1.52E-05	hypermethylated
cg04321618	0.23815	0.397651351	0.739634	2.15E-05	hypermethylated
cg09010107	0.28453	0.475010811	0.73938	0.000324	hypermethylated
cg27346707	0.24627	0.411111351	0.739288	0.000245	hypermethylated
cg15748507	0.26839	0.447988108	0.73913	8.37E-06	hypermethylated
cg15812753	0.25597	0.427234054	0.739052	4.42E-05	hypermethylated
cg12127282	0.27903	0.465494595	0.738344	7.29E-05	hypermethylated
cg16523839	0.22264	0.371415135	0.73832	0.000395	hypermethylated
cg19935850	0.20958	0.34960973	0.738244	0.000165	hypermethylated
cg21215767	0.26042	0.434278378	0.73778	0.00035	hypermethylated
cg22284398	0.31127	0.519010811	0.737598	3.15E-05	hypermethylated
cg11540692	0.20208	0.336905405	0.737417	8.13E-05	hypermethylated
cg11711816	0.22074	0.367939459	0.73712	0.000127	hypermethylated

cg07706352	0.22497	0.374979459	0.737079	5.86E-05	hypermethylated
cg12268637	0.26478	0.441295676	0.736951	3.08E-06	hypermethylated
cg09516959	0.2038	0.339663243	0.736951	4.94E-05	hypermethylated
cg18133531	0.20276	0.337919459	0.736906	1.58E-05	hypermethylated
cg22381068	0.30598	0.509881081	0.736723	5.86E-05	hypermethylated
cg06200092	0.1995	0.332357297	0.736346	0.000474	hypermethylated
cg04471325	0.28461	0.474097297	0.736197	9.27E-05	hypermethylated
cg18049571	0.26167	0.435844865	0.736066	5.58E-05	hypermethylated
cg07077013	0.22538	0.375378378	0.735986	6.95E-05	hypermethylated
cg20624923	0.26939	0.448623243	0.735808	3.07E-05	hypermethylated
cg14094347	0.3494	0.581856216	0.735783	0.000681	hypermethylated
cg05272349	0.24239	0.403365946	0.734759	3.44E-05	hypermethylated
cg21199093	0.36739	0.611315135	0.734604	0.000132	hypermethylated
cg13245152	0.30294	0.504025946	0.734466	4.82E-05	hypermethylated
cg08830818	0.16091	0.267718919	0.734465	4.20E-05	hypermethylated
cg10759602	0.14995	0.249475676	0.734418	0.000399	hypermethylated
cg22119466	0.211	0.351037838	0.734384	0.002965	hypermethylated
cg07057579	0.26672	0.443691892	0.734232	1.56E-05	hypermethylated
cg01474257	0.22901	0.380924865	0.734096	1.66E-05	hypermethylated
cg08980715	0.2159	0.359115135	0.734083	2.74E-05	hypermethylated
cg10602757	0.2394	0.398140541	0.733855	0.000346	hypermethylated
cg14823851	0.25858	0.429977838	0.733652	2.78E-05	hypermethylated
cg21830368	0.41507	0.690124865	0.733503	0.00097	hypermethylated
cg18064824	0.2407	0.400174595	0.733393	9.72E-05	hypermethylated
cg22379472	0.37304	0.62017027	0.733334	0.008783	hypermethylated
cg26638505	0.28754	0.477963784	0.733139	5.31E-05	hypermethylated
cg08171351	0.25524	0.424142703	0.732695	7.52E-06	hypermethylated
cg19570924	0.43	0.714394595	0.732385	0.000204	hypermethylated
cg23415434	0.43969	0.730394054	0.732188	3.67E-05	hypermethylated
cg08067346	0.36712	0.609814054	0.732118	0.000875	hypermethylated
cg20028827	0.30118	0.500278919	0.732107	2.27E-05	hypermethylated
cg07861603	0.46067	0.765097838	0.731911	7.12E-06	hypermethylated
cg09601175	0.2297	0.381479459	0.731855	4.70E-05	hypermethylated
cg01024141	0.15008	0.249177838	0.731444	0.000229	hypermethylated
cg07799386	0.28005	0.464928649	0.731325	2.92E-05	hypermethylated
cg13223107	0.18545	0.307874595	0.731313	7.12E-05	hypermethylated
cg24035107	0.3939	0.653930811	0.731309	0.003671	hypermethylated
cg08616061	0.27671	0.4593	0.731062	6.22E-06	hypermethylated
cg13654588	0.27409	0.454932973	0.731004	1.39E-05	hypermethylated
cg26053840	0.45845	0.760801622	0.730756	1.31E-05	hypermethylated
cg05962092	0.34601	0.574184865	0.730702	0.000382	hypermethylated
cg15399466	0.28001	0.464602162	0.730518	0.000152	hypermethylated
cg27550060	0.20432	0.338943243	0.730213	0.000575	hypermethylated
cg27111250	0.31216	0.517742162	0.729948	0.002066	hypermethylated
cg04130163	0.19169	0.31792	0.729889	0.000772	hypermethylated
cg22852065	0.30031	0.497994595	0.729678	1.84E-05	hypermethylated
cg11436222	0.27309	0.452812432	0.729537	1.18E-05	hypermethylated
cg11647681	0.26026	0.431377297	0.728997	0.000115	hypermethylated
cg25718766	0.25728	0.426421081	0.728939	0.000229	hypermethylated
cg20439889	0.24611	0.407868108	0.728799	7.84E-05	hypermethylated
cg21960680	0.18259	0.302576757	0.728693	1.35E-05	hypermethylated
cg17806623	0.24222	0.401377838	0.728643	0.000219	hypermethylated
cg23051392	0.41725	0.691397838	0.728604	0.002502	hypermethylated
cg13773083	0.22497	0.372736757	0.728424	7.93E-06	hypermethylated
cg13059459	0.28908	0.478955676	0.728423	2.95E-06	hypermethylated
cg04654288	0.22192	0.367672432	0.728381	5.58E-06	hypermethylated
cg05496514	0.25531	0.422957297	0.728262	1.54E-05	hypermethylated
cg10791926	0.19348	0.320454054	0.727933	0.000335	hypermethylated

cg15852312	0.24314	0.402612973	0.727606	0.000154	hypermethylated
cg00459845	0.31811	0.526514054	0.726946	4.31E-05	hypermethylated
cg06533314	0.23274	0.385179459	0.726812	6.38E-05	hypermethylated
cg06093355	0.3242	0.536516216	0.726738	0.001734	hypermethylated
cg10823473	0.25787	0.426735135	0.726697	2.64E-05	hypermethylated
cg08750534	0.29423	0.486903784	0.726692	0.001288	hypermethylated
cg00097357	0.24914	0.412196757	0.726376	2.02E-05	hypermethylated
cg14832317	0.2286	0.378203784	0.726338	3.04E-06	hypermethylated
cg04680436	0.41055	0.679215135	0.726311	0.001587	hypermethylated
cg19624318	0.25183	0.416595676	0.726198	3.23E-05	hypermethylated
cg01817364	0.39216	0.648687027	0.72608	0.001481	hypermethylated
cg12865888	0.39064	0.646148108	0.726025	0.003808	hypermethylated
cg27517681	0.30826	0.509854595	0.725938	1.62E-05	hypermethylated
cg23245942	0.26198	0.433271892	0.725816	0.008783	hypermethylated
cg03016906	0.18644	0.308274054	0.725502	2.51E-05	hypermethylated
cg15508379	0.23863	0.394465405	0.725123	2.27E-05	hypermethylated
cg10901368	0.22657	0.374528108	0.725117	8.83E-06	hypermethylated
cg16020346	0.34729	0.573884865	0.72462	0.000124	hypermethylated
cg06746009	0.37501	0.61965027	0.724525	8.42E-05	hypermethylated
cg03312958	0.26841	0.44343027	0.724269	4.18E-06	hypermethylated
cg02584377	0.43433	0.717504865	0.724197	0.000229	hypermethylated
cg24908603	0.30727	0.507598919	0.724182	0.000875	hypermethylated
cg05767159	0.25751	0.425329189	0.723951	9.27E-05	hypermethylated
cg05256269	0.21627	0.357064324	0.72335	6.38E-05	hypermethylated
cg08726801	0.23472	0.387487568	0.723209	0.000485	hypermethylated
cg02642822	0.26652	0.439941081	0.723067	0.000382	hypermethylated
cg17107893	0.25534	0.421386486	0.722724	6.08E-05	hypermethylated
cg03388789	0.25274	0.417072973	0.722646	6.15E-05	hypermethylated
cg07820868	0.28937	0.47750973	0.722615	0.000127	hypermethylated
cg06528559	0.31371	0.517592973	0.722387	0.000193	hypermethylated
cg13907146	0.23155	0.382028649	0.722357	0.000454	hypermethylated
cg18395355	0.19855	0.327543784	0.722185	1.94E-05	hypermethylated
cg15925365	0.31463	0.519008649	0.722102	0.00513	hypermethylated
cg22958047	0.26397	0.4354	0.721967	1.73E-05	hypermethylated
cg03213289	0.23926	0.39464	0.721958	0.000726	hypermethylated
cg11836372	0.4727	0.779668649	0.721936	0.000115	hypermethylated
cg22347212	0.22555	0.371856216	0.721298	4.94E-05	hypermethylated
cg10742917	0.32079	0.528785405	0.721053	5.19E-05	hypermethylated
cg17463083	0.45459	0.749217838	0.720819	9.82E-06	hypermethylated
cg06116236	0.26708	0.44016973	0.720788	0.001063	hypermethylated
cg15706250	0.27183	0.447879459	0.720406	1.82E-05	hypermethylated
cg12504882	0.23499	0.387159459	0.720329	0.000148	hypermethylated
cg01089249	0.24501	0.403601081	0.720089	2.54E-05	hypermethylated
cg20981451	0.26164	0.430976757	0.720027	0.0003	hypermethylated
cg09641127	0.50882	0.838018919	0.719827	0.000189	hypermethylated
cg05735782	0.26738	0.440351892	0.719765	2.85E-05	hypermethylated
cg17650274	0.24443	0.402522703	0.719649	0.000176	hypermethylated
cg04503968	0.25066	0.412715135	0.719415	0.000563	hypermethylated
cg07640800	0.34283	0.564384865	0.719186	0.000108	hypermethylated
cg10779492	0.25615	0.421686486	0.719182	0.000195	hypermethylated
cg08663552	0.33779	0.556063784	0.719124	7.52E-06	hypermethylated
cg18311537	0.19892	0.32744973	0.719085	0.000155	hypermethylated
cg03760589	0.28176	0.463681622	0.718668	9.49E-05	hypermethylated
cg21733531	0.33146	0.545367027	0.718393	4.47E-05	hypermethylated
cg14267754	0.45408	0.747074595	0.718306	0.004096	hypermethylated
cg20637609	0.28142	0.462817838	0.71772	6.31E-05	hypermethylated
cg21657577	0.23949	0.393804865	0.717516	3.23E-05	hypermethylated
cg25008263	0.29502	0.485094054	0.717452	2.15E-05	hypermethylated

cg05872614	0.2118	0.34824973	0.71742	9.05E-05	hypermethylated
cg05227215	0.37236	0.612238378	0.717395	0.003316	hypermethylated
cg22234080	0.22741	0.373898378	0.717351	1.94E-05	hypermethylated
cg01016191	0.31643	0.520234054	0.717274	0.001541	hypermethylated
cg27090062	0.29181	0.479563784	0.716693	6.95E-05	hypermethylated
cg14197071	0.3672	0.603429189	0.716618	0.00064	hypermethylated
cg21092808	0.23417	0.384784324	0.716494	5.51E-05	hypermethylated
cg27642784	0.41893	0.688302162	0.716333	6.39E-06	hypermethylated
cg25304107	0.22688	0.372657838	0.715922	0.000234	hypermethylated
cg24375626	0.40456	0.664448108	0.715803	0.00097	hypermethylated
cg20636352	0.28588	0.469451892	0.715568	0.001052	hypermethylated
cg05717794	0.2394	0.39310973	0.715509	0.001409	hypermethylated
cg06402675	0.17715	0.290836757	0.715238	8.22E-05	hypermethylated
cg00278547	0.30954	0.508182703	0.715221	2.27E-05	hypermethylated
cg14770827	0.25374	0.416565405	0.715192	1.71E-05	hypermethylated
cg02741440	0.29713	0.487602162	0.71461	0.00044	hypermethylated
cg26010218	0.3636	0.596618378	0.714456	0.004776	hypermethylated
cg00585714	0.23369	0.383422703	0.71434	1.50E-05	hypermethylated
cg27521476	0.22369	0.366876757	0.713795	4.82E-05	hypermethylated
cg09906145	0.21962	0.360155135	0.713609	0.001031	hypermethylated
cg02305723	0.38251	0.627172432	0.713365	7.84E-05	hypermethylated
cg20807254	0.42059	0.689597838	0.713341	0.000386	hypermethylated
cg21865845	0.22472	0.368447027	0.713329	0.000361	hypermethylated
cg25603636	0.25171	0.412624324	0.713066	2.71E-05	hypermethylated
cg17838420	0.27974	0.458528108	0.712924	2.16E-06	hypermethylated
cg08607018	0.32469	0.532196757	0.712897	0.00035	hypermethylated
cg13928782	0.27931	0.457779459	0.712786	4.05E-05	hypermethylated
cg16225703	0.36907	0.604680541	0.712279	0.000115	hypermethylated
cg01998213	0.38205	0.625799459	0.711939	0.000209	hypermethylated
cg18552939	0.25328	0.414861081	0.711895	0.000805	hypermethylated
cg11429969	0.2176	0.356405946	0.711843	1.99E-05	hypermethylated
cg03940098	0.27793	0.455216757	0.711832	3.40E-05	hypermethylated
cg10824107	0.29629	0.485136216	0.71138	1.12E-06	hypermethylated
cg06615940	0.25553	0.418382162	0.711329	2.74E-05	hypermethylated
cg03772567	0.27734	0.45408973	0.711322	0.002574	hypermethylated
cg18336674	0.31464	0.515119459	0.711205	5.65E-06	hypermethylated
cg25516803	0.23784	0.389367568	0.711141	0.000202	hypermethylated
cg24169822	0.26534	0.434284324	0.710798	3.81E-05	hypermethylated
cg06070263	0.194	0.317444865	0.710449	3.81E-05	hypermethylated
cg26777456	0.30105	0.492585405	0.710371	3.71E-05	hypermethylated
cg11661914	0.32441	0.530464324	0.709437	0.002147	hypermethylated
cg02051771	0.32655	0.533876757	0.709203	0.00012	hypermethylated
cg23536681	0.19815	0.323938378	0.709126	1.50E-05	hypermethylated
cg10683929	0.22659	0.370395676	0.708983	0.000102	hypermethylated
cg16584393	0.30486	0.49825027	0.708724	0.000163	hypermethylated
cg20959460	0.25516	0.416921081	0.708372	0.000119	hypermethylated
cg18566177	0.49249	0.804624324	0.708221	0.000262	hypermethylated
cg26164879	0.23423	0.382673514	0.708188	0.000262	hypermethylated
cg12707135	0.24955	0.407694595	0.70816	0.000102	hypermethylated
cg10710468	0.1858	0.303528108	0.70808	0.000287	hypermethylated
cg25819441	0.21719	0.354724865	0.707743	0.00024	hypermethylated
cg04027548	0.35689	0.582857838	0.707665	9.07E-06	hypermethylated
cg08732526	0.28768	0.469687027	0.707235	9.82E-06	hypermethylated
cg02994956	0.26991	0.440541622	0.7068	2.32E-05	hypermethylated
cg07953201	0.31179	0.508870811	0.706725	0.000399	hypermethylated
cg01287000	0.35938	0.586524324	0.706681	1.68E-05	hypermethylated
cg09357276	0.2576	0.420331351	0.706394	3.95E-05	hypermethylated
cg01024458	0.48463	0.790773514	0.706381	0.00035	hypermethylated

cg04297067	0.29827	0.486678919	0.706351	5.19E-05	hypermethylated
cg01691827	0.32304	0.527083243	0.706318	2.15E-05	hypermethylated
cg07657465	0.36217	0.590787027	0.705971	4.73E-06	hypermethylated
cg06571387	0.26556	0.433178378	0.705923	1.21E-05	hypermethylated
cg02487331	0.28389	0.463024324	0.705756	0.000626	hypermethylated
cg20939084	0.25236	0.411582703	0.705699	7.65E-05	hypermethylated
cg14092276	0.32525	0.53045027	0.705668	0.00071	hypermethylated
cg06275853	0.2712	0.442241081	0.705476	9.49E-05	hypermethylated
cg19184415	0.21125	0.344437838	0.705292	1.14E-05	hypermethylated
cg05628616	0.27521	0.448697297	0.70521	0.000575	hypermethylated
cg12982322	0.31928	0.520306486	0.70454	5.73E-06	hypermethylated
cg23424407	0.30204	0.492152432	0.704366	0.000528	hypermethylated
cg00182727	0.1905	0.310385405	0.70427	5.72E-05	hypermethylated
cg03744763	0.30653	0.499434595	0.704267	8.03E-05	hypermethylated
cg02119134	0.23324	0.379927568	0.703909	3.59E-06	hypermethylated
cg01122241	0.23139	0.376790811	0.703437	6.62E-05	hypermethylated
cg02564291	0.2709	0.441069189	0.703245	0.000653	hypermethylated
cg15951870	0.27734	0.451484324	0.70302	0.0002	hypermethylated
cg16620392	0.26705	0.434583784	0.702524	6.38E-05	hypermethylated
cg20273670	0.27234	0.443128108	0.702315	0.002363	hypermethylated
cg26034341	0.47975	0.78040973	0.701949	0.00024	hypermethylated
cg20634266	0.29911	0.486489189	0.701732	4.60E-06	hypermethylated
cg12542255	0.35828	0.582626486	0.701484	0.00017	hypermethylated
cg25959472	0.33475	0.544245946	0.701175	0.00097	hypermethylated
cg19674091	0.50115	0.814781622	0.701171	3.95E-05	hypermethylated
cg23100720	0.22061	0.35862	0.700958	0.002318	hypermethylated
cg07892422	0.23843	0.387558378	0.700848	6.62E-05	hypermethylated
cg23766254	0.25937	0.42156	0.700726	0.000358	hypermethylated
cg16627346	0.36748	0.597198378	0.700545	8.52E-05	hypermethylated
cg05022105	0.26673	0.433438919	0.700449	0.00028	hypermethylated
cg11385338	0.3971	0.645277297	0.700417	0.000115	hypermethylated
cg23994112	0.35942	0.583901622	0.700055	0.004365	hypermethylated
cg00109703	0.20192	0.328026486	0.700028	0.00031	hypermethylated
cg04201253	0.18818	0.30565027	0.699769	0.000176	hypermethylated
cg26962778	0.23849	0.387301081	0.699527	0.000417	hypermethylated
cg10571104	0.26283	0.426790811	0.699399	0.001288	hypermethylated
cg17868307	0.49985	0.811627568	0.699323	9.72E-05	hypermethylated
cg23933241	0.31378	0.509442703	0.699166	0.00054	hypermethylated
cg14826456	0.25076	0.407124324	0.699162	6.46E-05	hypermethylated
cg19535672	0.20111	0.326482703	0.699022	8.73E-05	hypermethylated
cg03631455	0.39272	0.637507568	0.698941	0.000148	hypermethylated
cg09270346	0.26495	0.430024324	0.698698	4.94E-05	hypermethylated
cg23933044	0.27542	0.447008649	0.698669	0.001176	hypermethylated
cg20247911	0.32794	0.532246486	0.698663	0.000528	hypermethylated
cg18483289	0.21947	0.356184865	0.698602	6.57E-06	hypermethylated
cg02428056	0.20843	0.338258919	0.698565	8.42E-05	hypermethylated
cg18268547	0.35902	0.582628108	0.698511	6.22E-06	hypermethylated
cg16990168	0.31669	0.513771892	0.698057	5.19E-05	hypermethylated
cg13360823	0.26353	0.427457838	0.697815	0.000262	hypermethylated
cg09548780	0.2525	0.409563243	0.697803	0.00054	hypermethylated
cg13435718	0.24403	0.395747027	0.69752	0.000104	hypermethylated
cg01816425	0.22959	0.372315135	0.697464	5.19E-05	hypermethylated
cg00014998	0.36261	0.588003784	0.697407	0.000274	hypermethylated
cg00196407	0.22015	0.356990811	0.6974	0.001395	hypermethylated
cg21237591	0.2813	0.456077297	0.697169	7.47E-05	hypermethylated
cg04692403	0.21593	0.350052973	0.69701	1.12E-05	hypermethylated
cg12972233	0.25477	0.412948108	0.696765	7.93E-06	hypermethylated
cg14822490	0.2996	0.485517297	0.696485	9.72E-05	hypermethylated

cg01964852	0.26448	0.428511351	0.696175	0.003378	hypermethylated
cg07937271	0.31409	0.508828649	0.696002	1.21E-05	hypermethylated
cg12608145	0.23675	0.383529189	0.695972	0.000545	hypermethylated
cg05574357	0.33687	0.545682703	0.69587	9.27E-05	hypermethylated
cg09887220	0.32295	0.523085405	0.695736	9.82E-06	hypermethylated
cg24446586	0.28936	0.468626486	0.695573	0.000296	hypermethylated
cg17955729	0.30214	0.48930973	0.695531	1.44E-05	hypermethylated
cg18892212	0.29588	0.479122703	0.695383	0.000563	hypermethylated
cg00513205	0.27746	0.449223784	0.695155	4.76E-05	hypermethylated
cg05650810	0.21771	0.352447568	0.695001	0.000145	hypermethylated
cg19594666	0.31279	0.506191351	0.694488	0.000245	hypermethylated
cg13451356	0.2714	0.439139459	0.694258	0.003843	hypermethylated
cg10901633	0.3707	0.599795135	0.694218	0.000163	hypermethylated
cg20906291	0.35878	0.580375676	0.693888	2.29E-06	hypermethylated
cg10743390	0.34166	0.552663243	0.693839	9.07E-06	hypermethylated
cg07191594	0.21539	0.348403784	0.693809	0.000137	hypermethylated
cg01830023	0.25325	0.409579459	0.693581	6.75E-06	hypermethylated
cg24006721	0.23178	0.374803243	0.693377	0.010211	hypermethylated
cg03182688	0.25166	0.406898378	0.693193	0.000358	hypermethylated
cg01688688	0.26759	0.432651351	0.693181	4.05E-05	hypermethylated
cg10537450	0.31352	0.506885405	0.693102	0.000104	hypermethylated
cg12279419	0.26166	0.42300973	0.692997	5.28E-06	hypermethylated
cg02200717	0.37786	0.610787027	0.692818	0.00028	hypermethylated
cg22518433	0.52426	0.847396216	0.692754	4.05E-05	hypermethylated
cg05196366	0.22984	0.371440541	0.692501	0.000551	hypermethylated
cg26789064	0.35145	0.567955135	0.692458	0.001096	hypermethylated
cg03726817	0.34068	0.550510811	0.692354	0.000245	hypermethylated
cg02765731	0.37774	0.61028	0.692078	0.001751	hypermethylated
cg19025113	0.32533	0.525561081	0.691955	0.000327	hypermethylated
cg19701087	0.31185	0.503712432	0.691748	0.004059	hypermethylated
cg08317694	0.41171	0.664905405	0.691521	5.51E-05	hypermethylated
cg09841842	0.34719	0.560518378	0.691036	0.000101	hypermethylated
cg07214164	0.27536	0.44440973	0.690571	9.07E-06	hypermethylated
cg25817244	0.2512	0.40539027	0.690475	0.000464	hypermethylated
cg07791011	0.32454	0.523740541	0.690456	9.27E-05	hypermethylated
cg17818471	0.25393	0.409747027	0.690303	7.29E-05	hypermethylated
cg00059246	0.21824	0.352118378	0.690145	0.000135	hypermethylated
cg17104824	0.36281	0.585323243	0.690019	5.86E-05	hypermethylated
cg20831708	0.28457	0.459093514	0.690004	0.00099	hypermethylated
cg02148562	0.4588	0.740103784	0.689862	5.72E-05	hypermethylated
cg25586361	0.26283	0.423905405	0.689612	0.000152	hypermethylated
cg09435415	0.25974	0.418883243	0.68948	0.000111	hypermethylated
cg17214023	0.31174	0.50270973	0.689382	2.21E-05	hypermethylated
cg20974609	0.33053	0.532959459	0.689245	0.004059	hypermethylated
cg21304158	0.29248	0.471598378	0.689221	0.00012	hypermethylated
cg21412973	0.24185	0.389951351	0.689182	0.000101	hypermethylated
cg07471614	0.3461	0.557931892	0.6889	0.000464	hypermethylated
cg20588069	0.31155	0.502210811	0.688829	0.000182	hypermethylated
cg09151754	0.27444	0.44236973	0.688762	2.51E-05	hypermethylated
cg20176142	0.29319	0.472588649	0.688749	0.004776	hypermethylated
cg16752876	0.36707	0.591637838	0.688659	5.86E-05	hypermethylated
cg23853507	0.31323	0.504851351	0.688636	6.15E-05	hypermethylated
cg08424749	0.26515	0.427313514	0.688486	2.10E-05	hypermethylated
cg12924956	0.30199	0.486645946	0.688372	0.000653	hypermethylated
cg23865240	0.39715	0.639958378	0.688294	0.000417	hypermethylated
cg25012961	0.43022	0.693218378	0.688235	0.000749	hypermethylated
cg00145253	0.27047	0.435778919	0.688128	9.31E-06	hypermethylated
cg23906261	0.23077	0.371751892	0.687884	0.000822	hypermethylated

cg11173579	0.50646	0.81555027	0.687325	0.002599	hypermethylated
cg18849102	0.27743	0.446578378	0.68679	9.44E-06	hypermethylated
cg13891220	0.29997	0.482831351	0.686701	8.83E-06	hypermethylated
cg14128641	0.23421	0.376944324	0.686549	6.15E-05	hypermethylated
cg04098339	0.22636	0.364275135	0.686409	0.000474	hypermethylated
cg06391932	0.27136	0.43666973	0.686334	3.23E-05	hypermethylated
cg15821562	0.43625	0.701988649	0.686293	2.57E-05	hypermethylated
cg06942814	0.33258	0.535163784	0.686279	6.22E-06	hypermethylated
cg13246242	0.22059	0.354956757	0.686276	0.000112	hypermethylated
cg07050692	0.42476	0.683447568	0.686183	0.005606	hypermethylated
cg03074925	0.20948	0.337055676	0.686174	1.46E-05	hypermethylated
cg22244192	0.26115	0.420167568	0.686086	7.75E-05	hypermethylated
cg12526923	0.3559	0.572607027	0.686073	0.001031	hypermethylated
cg12141135	0.21457	0.345172973	0.685871	9.72E-05	hypermethylated
cg00601836	0.43273	0.696099459	0.685826	0.002455	hypermethylated
cg05655885	0.31128	0.500721622	0.685796	0.000166	hypermethylated
cg18984724	0.28719	0.461965946	0.685781	7.38E-05	hypermethylated
cg07605285	0.30134	0.484725405	0.685776	1.40E-05	hypermethylated
cg09399716	0.24741	0.397968649	0.685751	0.004445	hypermethylated
cg16001165	0.28683	0.461367027	0.685719	0.003195	hypermethylated
cg23547017	0.25421	0.408818378	0.685439	4.15E-05	hypermethylated
cg01196950	0.24886	0.400196757	0.685375	0.000382	hypermethylated
cg26457809	0.27431	0.440964865	0.684856	7.38E-05	hypermethylated
cg04565337	0.31248	0.502295135	0.684771	8.60E-06	hypermethylated
cg11564711	0.25376	0.407863784	0.684623	8.84E-05	hypermethylated
cg16458596	0.24967	0.401257297	0.684505	1.12E-05	hypermethylated
cg25614364	0.43019	0.691379459	0.684504	0.007035	hypermethylated
cg09890775	0.30767	0.494415135	0.684339	8.37E-06	hypermethylated
cg04880138	0.49171	0.790092432	0.684214	0.000327	hypermethylated
cg14270434	0.34257	0.550441081	0.684189	0.00017	hypermethylated
cg08845721	0.43944	0.70606	0.684125	0.000667	hypermethylated
cg09743950	0.30355	0.487697838	0.684053	0.004172	hypermethylated
cg18592365	0.19892	0.319494054	0.683601	0.000435	hypermethylated
cg18331466	0.22226	0.356938378	0.683427	7.84E-05	hypermethylated
cg13149245	0.30043	0.482278919	0.682839	0.004134	hypermethylated
cg10244666	0.40507	0.650241081	0.682803	0.014258	hypermethylated
cg16631083	0.40287	0.646706486	0.682797	0.001423	hypermethylated
cg08328513	0.29957	0.480748649	0.68239	0.000176	hypermethylated
cg27435498	0.31622	0.50728	0.681854	1.25E-05	hypermethylated
cg24471254	0.43697	0.70098	0.681839	7.93E-05	hypermethylated
cg09774917	0.23328	0.37421027	0.681787	5.07E-06	hypermethylated
cg09099830	0.25514	0.409164324	0.681391	0.00064	hypermethylated
cg02260587	0.2658	0.426200541	0.681191	7.38E-05	hypermethylated
cg16512661	0.29697	0.476152432	0.681106	1.54E-05	hypermethylated
cg21238818	0.20812	0.333641622	0.680884	0.000303	hypermethylated
cg01442953	0.24251	0.388676216	0.680525	0.000495	hypermethylated
cg14959580	0.27149	0.435099459	0.680446	0.000256	hypermethylated
cg22152229	0.37954	0.608254054	0.680422	5.45E-05	hypermethylated
cg12754571	0.33874	0.542847027	0.680367	0.00032	hypermethylated
cg00469856	0.22135	0.354718919	0.680347	6.38E-05	hypermethylated
cg26801014	0.20869	0.334430811	0.680346	0.000163	hypermethylated
cg10717869	0.32392	0.519048108	0.680231	0.003506	hypermethylated
cg06553058	0.35152	0.563114595	0.679822	0.001327	hypermethylated
cg18824549	0.28113	0.450268108	0.679547	0.017101	hypermethylated
cg00085013	0.332	0.531723243	0.679492	7.47E-05	hypermethylated
cg07630723	0.20471	0.327799459	0.679232	0.000365	hypermethylated
cg21893358	0.2878	0.460723243	0.678834	2.15E-05	hypermethylated
cg21648941	0.25423	0.406950811	0.67872	0.000182	hypermethylated

cg00650641	0.3449	0.552015676	0.678531	6.39E-06	hypermethylated
cg10107473	0.30091	0.481591351	0.678477	0.003473	hypermethylated
cg16134678	0.26685	0.427022703	0.678284	0.000382	hypermethylated
cg25824760	0.19311	0.309011351	0.678237	0.001635	hypermethylated
cg22832557	0.38764	0.620285405	0.678215	0.000152	hypermethylated
cg15074709	0.30296	0.484696757	0.677955	3.76E-05	hypermethylated
cg18836745	0.23993	0.383842162	0.6779	0.000187	hypermethylated
cg26158150	0.20762	0.332001081	0.677243	0.000202	hypermethylated
cg24999105	0.3587	0.573523784	0.677076	0.000214	hypermethylated
cg14456683	0.29753	0.475582162	0.676659	1.06E-05	hypermethylated
cg04344018	0.23198	0.370631351	0.675984	0.000207	hypermethylated
cg17894318	0.24468	0.390861081	0.67576	6.78E-05	hypermethylated
cg21006031	0.29727	0.47486973	0.675758	0.000212	hypermethylated
cg01361263	0.34149	0.545421622	0.675529	0.000417	hypermethylated
cg16134191	0.22924	0.366102703	0.67539	4.82E-05	hypermethylated
cg06757585	0.3266	0.521507568	0.675163	0.000327	hypermethylated
cg24900983	0.22191	0.354338919	0.675155	7.12E-05	hypermethylated
cg14325153	0.40034	0.639228108	0.675105	0.000528	hypermethylated
cg21566642	0.24948	0.398342703	0.675086	0.000626	hypermethylated
cg03032497	0.29904	0.477472973	0.675081	5.00E-05	hypermethylated
cg16909495	0.26931	0.430002162	0.675076	0.000251	hypermethylated
cg26279070	0.31545	0.503658378	0.675034	1.39E-05	hypermethylated
cg18498241	0.35628	0.568705946	0.674671	7.72E-06	hypermethylated
cg05266564	0.30038	0.479349189	0.674288	3.57E-05	hypermethylated
cg23251701	0.3179	0.507251351	0.674128	0.009713	hypermethylated
cg02770983	0.33044	0.527187027	0.673927	0.000142	hypermethylated
cg25621182	0.15762	0.251461622	0.673888	0.00017	hypermethylated
cg20046343	0.29736	0.474338378	0.673706	3.49E-05	hypermethylated
cg00210249	0.30557	0.487312432	0.673344	0.008208	hypermethylated
cg05107535	0.41068	0.654894054	0.673247	6.01E-05	hypermethylated
cg02434121	0.32674	0.521019459	0.673194	0.001839	hypermethylated
cg06373870	0.32315	0.515273514	0.673134	4.36E-05	hypermethylated
cg23449696	0.32342	0.515703243	0.673132	0.000166	hypermethylated
cg19698137	0.25374	0.404551892	0.672974	0.000123	hypermethylated
cg21002957	0.38184	0.608733514	0.672843	0.006395	hypermethylated
cg17508591	0.34456	0.549273514	0.672769	7.86E-07	hypermethylated
cg14872762	0.31122	0.496091351	0.672671	0.000313	hypermethylated
cg03714676	0.44891	0.715541622	0.672609	0.000358	hypermethylated
cg11356767	0.25878	0.412472432	0.672572	0.000306	hypermethylated
cg02213260	0.23716	0.377978378	0.672443	0.000789	hypermethylated
cg08640361	0.32259	0.514118378	0.672399	0.002856	hypermethylated
cg09057517	0.26972	0.42983027	0.672305	0.000216	hypermethylated
cg22022798	0.36734	0.585337838	0.672154	0.000145	hypermethylated
cg18806140	0.30031	0.478490811	0.672039	0.000248	hypermethylated
cg02051616	0.3068	0.488796757	0.671936	1.31E-05	hypermethylated
cg14001023	0.33657	0.536223784	0.671929	0.000506	hypermethylated
cg19389884	0.28675	0.456780541	0.671708	0.000382	hypermethylated
cg02183564	0.322	0.512896216	0.671606	0.000528	hypermethylated
cg08100944	0.17507	0.278836216	0.671486	0.000124	hypermethylated
cg04171235	0.31498	0.501655135	0.671436	3.27E-05	hypermethylated
cg03685886	0.30962	0.493103243	0.671391	0.000399	hypermethylated
cg27343836	0.25385	0.404265946	0.671328	4.00E-05	hypermethylated
cg22763649	0.27612	0.439723784	0.671302	4.31E-05	hypermethylated
cg04914126	0.31863	0.507415676	0.671286	0.001734	hypermethylated
cg06981948	0.3308	0.526740541	0.671133	4.82E-05	hypermethylated
cg03234702	0.45239	0.720178378	0.670787	0.000262	hypermethylated
cg16551520	0.3204	0.51	0.670623	0.003136	hypermethylated
cg19685491	0.34117	0.542987568	0.670428	4.36E-05	hypermethylated

cg17300723	0.24819	0.394993514	0.670384	0.000839	hypermethylated
cg03793270	0.30477	0.485032432	0.67036	4.64E-05	hypermethylated
cg03433597	0.2813	0.447675676	0.670344	9.27E-05	hypermethylated
cg06515771	0.24505	0.389951351	0.670218	0.000132	hypermethylated
cg18579879	0.28422	0.452244865	0.670096	3.32E-05	hypermethylated
cg16519487	0.28363	0.451268649	0.669976	1.65E-06	hypermethylated
cg13933080	0.31323	0.498284865	0.669748	2.39E-05	hypermethylated
cg27561818	0.50138	0.797568649	0.669704	0.003505	hypermethylated
cg05266537	0.24083	0.383099459	0.669704	1.73E-05	hypermethylated
cg02852670	0.30777	0.489562703	0.669641	0.001556	hypermethylated
cg25678088	0.24686	0.392668649	0.669619	1.46E-05	hypermethylated
cg26328180	0.34446	0.547756757	0.669199	1.38E-05	hypermethylated
cg14622069	0.26433	0.420312973	0.669124	2.26E-06	hypermethylated
cg09523275	0.25469	0.404975676	0.669093	8.22E-05	hypermethylated
cg04965050	0.25016	0.397755135	0.669029	0.000242	hypermethylated
cg00363813	0.27613	0.439038919	0.669001	0.000166	hypermethylated
cg13686751	0.32775	0.521037297	0.668791	8.22E-05	hypermethylated
cg21390624	0.33906	0.538897297	0.66847	0.000108	hypermethylated
cg00445443	0.2492	0.396072432	0.66846	0.001188	hypermethylated
cg21828951	0.19462	0.309265946	0.668188	0.00049	hypermethylated
cg13729816	0.3341	0.530851351	0.668028	0.000303	hypermethylated
cg05579030	0.30189	0.479649189	0.667957	8.63E-05	hypermethylated
cg25789916	0.24414	0.387888108	0.667932	0.000187	hypermethylated
cg20847580	0.29772	0.473012973	0.667924	3.23E-05	hypermethylated
cg14273502	0.31736	0.504198378	0.667871	1.80E-05	hypermethylated
cg24803719	0.27895	0.44311027	0.667659	0.000374	hypermethylated
cg07273003	0.45799	0.727375676	0.667385	0.001619	hypermethylated
cg00652908	0.26193	0.415869189	0.666948	5.72E-05	hypermethylated
cg24469464	0.40548	0.643672973	0.666697	7.93E-06	hypermethylated
cg08316825	0.26498	0.420576216	0.666484	1.14E-05	hypermethylated
cg22344631	0.24288	0.385475135	0.666394	0.000212	hypermethylated
cg04615460	0.25261	0.40086	0.666187	0.000511	hypermethylated
cg04701505	0.23342	0.370337838	0.665914	0.000112	hypermethylated
cg04027736	0.27825	0.441462703	0.66591	0.007536	hypermethylated
cg13911707	0.33932	0.538284865	0.665723	0.001734	hypermethylated
cg06993512	0.25995	0.412354054	0.665649	0.000408	hypermethylated
cg10172432	0.25956	0.411700541	0.665527	0.000214	hypermethylated
cg04847275	0.48724	0.772682162	0.665243	0.002409	hypermethylated
cg03794214	0.39447	0.625477297	0.665042	0.000613	hypermethylated
cg02034311	0.27061	0.429057838	0.664957	8.84E-05	hypermethylated
cg09595050	0.41904	0.664053514	0.664212	0.000224	hypermethylated
cg26578682	0.29133	0.461625405	0.664068	4.05E-05	hypermethylated
cg10383568	0.40891	0.64788973	0.663965	0.000126	hypermethylated
cg25215834	0.25356	0.401700541	0.663793	0.000725	hypermethylated
cg19304273	0.44661	0.707505405	0.663726	1.46E-05	hypermethylated
cg17238766	0.25143	0.398253514	0.66353	9.95E-05	hypermethylated
cg11235602	0.25146	0.398289189	0.663487	0.000229	hypermethylated
cg24154937	0.28346	0.448961622	0.663447	2.04E-05	hypermethylated
cg18921954	0.24439	0.38696	0.662999	0.000749	hypermethylated
cg06514855	0.2569	0.406510811	0.662087	7.47E-05	hypermethylated
cg04794430	0.34202	0.541175135	0.662015	0.000224	hypermethylated
cg05648614	0.44181	0.699047568	0.661965	6.15E-05	hypermethylated
cg05617469	0.26308	0.416237297	0.661905	3.40E-05	hypermethylated
cg23671082	0.18311	0.289702162	0.66186	4.15E-05	hypermethylated
cg09059267	0.3157	0.499444865	0.661771	0.000187	hypermethylated
cg05492170	0.28941	0.457685946	0.661243	1.66E-05	hypermethylated
cg08923271	0.31276	0.494515135	0.660959	7.47E-05	hypermethylated
cg04493169	0.44114	0.697484865	0.660925	0.001603	hypermethylated

cg22424284	0.5826	0.921115135	0.660876	0.000102	hypermethylated
cg22916586	0.36744	0.580767027	0.660451	0.000155	hypermethylated
cg25951210	0.30158	0.476565946	0.660135	2.21E-05	hypermethylated
cg21737226	0.31609	0.499488649	0.660116	7.12E-05	hypermethylated
cg02147194	0.29246	0.462083243	0.659913	1.94E-05	hypermethylated
cg00517787	0.29501	0.466085405	0.65983	8.95E-06	hypermethylated
cg13641903	0.32916	0.519982703	0.659675	4.36E-06	hypermethylated
cg13694927	0.35755	0.564807027	0.659613	7.65E-05	hypermethylated
cg10266211	0.35201	0.556050811	0.6596	7.32E-06	hypermethylated
cg13031611	0.29519	0.465954054	0.658544	0.000123	hypermethylated
cg08081407	0.18936	0.298862703	0.658351	0.000245	hypermethylated
cg08451582	0.18322	0.289145946	0.658221	0.000152	hypermethylated
cg22121557	0.31699	0.500067568	0.657686	5.73E-06	hypermethylated
cg05711980	0.21767	0.343372432	0.657632	9.31E-06	hypermethylated
cg27513935	0.21454	0.33838973	0.657439	0.000408	hypermethylated
cg00730561	0.2754	0.434378919	0.657426	0.000696	hypermethylated
cg17781710	0.2378	0.374973514	0.65704	4.15E-05	hypermethylated
cg09656389	0.30211	0.476317297	0.656849	0.000474	hypermethylated
cg12320039	0.2777	0.437805405	0.656763	5.00E-06	hypermethylated
cg17963840	0.27575	0.43473027	0.65676	3.49E-05	hypermethylated
cg20708961	0.25165	0.396721622	0.656708	7.93E-06	hypermethylated
cg16449219	0.43132	0.679673514	0.656083	0.004287	hypermethylated
cg21298174	0.28289	0.445741081	0.655965	1.26E-05	hypermethylated
cg00919411	0.27296	0.430002703	0.655656	6.22E-06	hypermethylated
cg12383699	0.26214	0.4129	0.655455	0.000126	hypermethylated
cg05700079	0.29765	0.468800541	0.655357	1.15E-05	hypermethylated
cg26670249	0.24628	0.387847568	0.65519	0.002046	hypermethylated
cg16855929	0.52279	0.823142162	0.65491	3.76E-05	hypermethylated
cg10377921	0.27694	0.435984865	0.654705	0.010042	hypermethylated
cg00599770	0.23733	0.373623243	0.65469	1.01E-05	hypermethylated
cg26194089	0.28482	0.448254054	0.654266	5.06E-05	hypermethylated
cg18632637	0.38393	0.604128649	0.654013	0.00097	hypermethylated
cg14216068	0.40405	0.635721622	0.653861	0.004096	hypermethylated
cg01218896	0.29162	0.458799459	0.653774	2.15E-05	hypermethylated
cg07698121	0.34663	0.545168108	0.653305	0.005316	hypermethylated
cg23666378	0.27563	0.433489189	0.653263	0.000134	hypermethylated
cg13842240	0.27095	0.426078378	0.653092	1.17E-05	hypermethylated
cg03811478	0.19775	0.310947027	0.652991	4.42E-06	hypermethylated
cg15743907	0.33469	0.526267568	0.652971	9.72E-05	hypermethylated
cg24211976	0.36177	0.568825405	0.652913	0.001096	hypermethylated
cg05768922	0.2115	0.332509189	0.652737	0.001288	hypermethylated
cg04927004	0.26071	0.40986973	0.65272	3.15E-05	hypermethylated
cg12006284	0.34889	0.548391351	0.652434	5.58E-05	hypermethylated
cg26248586	0.40662	0.638980541	0.652091	0.003986	hypermethylated
cg12591491	0.25685	0.403585405	0.651948	1.94E-05	hypermethylated
cg26530319	0.34854	0.547651351	0.651933	0.000268	hypermethylated
cg17242351	0.35084	0.551147027	0.651624	0.000374	hypermethylated
cg20308540	0.2429	0.381537838	0.651464	0.000912	hypermethylated
cg23986620	0.33505	0.526178919	0.651177	0.000161	hypermethylated
cg25647300	0.2596	0.407610811	0.650902	0.006015	hypermethylated
cg16744996	0.35768	0.561564324	0.650782	5.86E-05	hypermethylated
cg03398865	0.25507	0.400462162	0.650773	5.58E-06	hypermethylated
cg16046493	0.50609	0.794465405	0.65059	0.00095	hypermethylated
cg25217100	0.34262	0.537652432	0.650064	0.000287	hypermethylated
cg20632143	0.26563	0.416777838	0.64986	0.000485	hypermethylated
cg00096810	0.28346	0.444717297	0.649743	2.32E-05	hypermethylated
cg22970357	0.27816	0.436394054	0.649716	0.000191	hypermethylated
cg06675538	0.35083	0.550382162	0.649662	0.000262	hypermethylated

cg21216477	0.28073	0.44033027	0.649403	5.12E-05	hypermethylated
cg16582779	0.2114	0.331565405	0.649318	6.39E-06	hypermethylated
cg24170248	0.35594	0.558108649	0.648912	9.16E-05	hypermethylated
cg11496364	0.25547	0.400566486	0.648888	3.00E-05	hypermethylated
cg07208161	0.26137	0.409795135	0.648809	3.95E-05	hypermethylated
cg01748892	0.30119	0.472105946	0.648437	0.000115	hypermethylated
cg22797270	0.30588	0.479352432	0.648121	0.000195	hypermethylated
cg19516404	0.2474	0.387617838	0.647789	1.75E-05	hypermethylated
cg07996594	0.33635	0.526968108	0.647752	0.002409	hypermethylated
cg06926146	0.29253	0.45831027	0.64774	9.05E-05	hypermethylated
cg17548735	0.45879	0.718763243	0.647683	0.001212	hypermethylated
cg03468072	0.28306	0.443446486	0.647652	5.58E-05	hypermethylated
cg00998744	0.27468	0.43018	0.647189	0.000107	hypermethylated
cg03768916	0.24268	0.380063243	0.647184	2.64E-05	hypermethylated
cg00792513	0.31152	0.487831351	0.647058	8.22E-05	hypermethylated
cg04485799	0.25138	0.393646486	0.647031	0.000108	hypermethylated
cg03993263	0.18223	0.28534	0.646922	4.94E-05	hypermethylated
cg07823492	0.49116	0.769064865	0.646912	0.001452	hypermethylated
cg07266350	0.51843	0.811708108	0.646812	0.001052	hypermethylated
cg20596162	0.23602	0.369527027	0.646771	0.000152	hypermethylated
cg22022580	0.38364	0.600604865	0.646663	0.004445	hypermethylated
cg03190219	0.4589	0.718185946	0.646178	7.93E-06	hypermethylated
cg13400512	0.31013	0.485336216	0.646111	0.001074	hypermethylated
cg00871610	0.27748	0.434167568	0.645868	0.002751	hypermethylated
cg21415530	0.31102	0.486635676	0.645835	0.000232	hypermethylated
cg04498349	0.25367	0.396895676	0.645807	2.78E-05	hypermethylated
cg21664030	0.22423	0.350832432	0.645803	0.000224	hypermethylated
cg21951729	0.26657	0.417054595	0.645722	9.27E-05	hypermethylated
cg14573448	0.35349	0.553010811	0.645638	0.000789	hypermethylated
cg24224304	0.28612	0.447604865	0.645605	6.70E-05	hypermethylated
cg16703762	0.32054	0.501387568	0.645422	0.000107	hypermethylated
cg04436207	0.32073	0.501684324	0.645421	0.000256	hypermethylated
cg12694372	0.37814	0.591396216	0.645205	0.002502	hypermethylated
cg12727940	0.44121	0.69000973	0.645151	0.00044	hypermethylated
cg12017745	0.45762	0.715593514	0.64499	0.002066	hypermethylated
cg16786808	0.45581	0.712561622	0.644582	0.000159	hypermethylated
cg07824172	0.31731	0.495943784	0.644284	0.000563	hypermethylated
cg16638920	0.31985	0.499904324	0.644257	2.45E-05	hypermethylated
cg18255813	0.43185	0.674917838	0.644182	0.004692	hypermethylated
cg02718825	0.27724	0.433250811	0.644067	6.31E-05	hypermethylated
cg16834823	0.3994	0.624069189	0.643872	0.00291	hypermethylated
cg21915313	0.37252	0.582028649	0.643772	0.000271	hypermethylated
cg15638414	0.24349	0.380404865	0.643673	0.000132	hypermethylated
cg04858155	0.23825	0.372205946	0.643625	2.74E-05	hypermethylated
cg02058108	0.32163	0.50238	0.643377	0.000306	hypermethylated
cg25493589	0.26038	0.406697297	0.643337	3.32E-05	hypermethylated
cg10912240	0.23841	0.372362162	0.643262	1.56E-05	hypermethylated
cg04676482	0.28796	0.449702703	0.643103	1.67E-06	hypermethylated
cg07599881	0.28047	0.437997838	0.643077	8.94E-05	hypermethylated
cg09151762	0.22362	0.349191351	0.642969	2.32E-05	hypermethylated
cg09413529	0.37503	0.58562	0.642959	8.22E-05	hypermethylated
cg14324816	0.2666	0.416131351	0.642362	0.000109	hypermethylated
cg06942685	0.22	0.343359459	0.642216	3.85E-05	hypermethylated
cg10195169	0.29046	0.453282703	0.642072	0.004951	hypermethylated
cg12745203	0.30312	0.473016757	0.642002	3.07E-05	hypermethylated
cg10661615	0.24506	0.38238973	0.641909	8.13E-05	hypermethylated
cg26096837	0.33249	0.518791892	0.641845	0.000115	hypermethylated
cg07591229	0.23549	0.367401081	0.64169	0.004326	hypermethylated

cg15824707	0.31568	0.492452973	0.641523	5.14E-06	hypermethylated
cg08819431	0.40457	0.631014595	0.641284	0.00421	hypermethylated
cg09268963	0.40688	0.634499459	0.641016	0.00049	hypermethylated
cg17519645	0.33479	0.522025946	0.640865	0.00062	hypermethylated
cg22008851	0.29122	0.454079459	0.640835	3.62E-05	hypermethylated
cg26034658	0.42088	0.656205405	0.640739	1.35E-05	hypermethylated
cg02025583	0.2255	0.351564865	0.640663	0.000277	hypermethylated
cg02416510	0.30715	0.478600541	0.639879	4.64E-05	hypermethylated
cg17563034	0.25307	0.39427027	0.639648	0.000159	hypermethylated
cg15386434	0.25756	0.40122	0.639485	0.000613	hypermethylated
cg13337064	0.32502	0.506255135	0.639336	0.000142	hypermethylated
cg15798385	0.39225	0.610963243	0.639312	5.72E-05	hypermethylated
cg12484113	0.19225	0.299434054	0.639255	0.001212	hypermethylated
cg06211872	0.33003	0.514027568	0.639249	0.000112	hypermethylated
cg21464220	0.45599	0.71019027	0.639203	0.003843	hypermethylated
cg12508451	0.27981	0.435772973	0.639129	0.000469	hypermethylated
cg12835736	0.37862	0.589624324	0.639045	7.47E-05	hypermethylated
cg06388730	0.2775	0.432097838	0.63887	1.71E-05	hypermethylated
cg06268875	0.28547	0.444501622	0.63885	0.000358	hypermethylated
cg21980338	0.24284	0.378088108	0.638716	0.000122	hypermethylated
cg12426612	0.39196	0.610215135	0.638612	0.000365	hypermethylated
cg08525845	0.24398	0.379657297	0.637935	1.06E-05	hypermethylated
cg04744409	0.36449	0.567157297	0.63787	0.00097	hypermethylated
cg08598654	0.24811	0.386062703	0.637855	0.000178	hypermethylated
cg14500300	0.31783	0.49451027	0.637745	0.002624	hypermethylated
cg20586076	0.35396	0.550683784	0.637638	3.57E-05	hypermethylated
cg17611674	0.2854	0.443974595	0.637492	6.46E-05	hypermethylated
cg11625005	0.35883	0.558177297	0.637423	1.58E-05	hypermethylated
cg20289911	0.26757	0.416212432	0.637404	9.95E-05	hypermethylated
cg07547765	0.43724	0.680065405	0.637248	0.000569	hypermethylated
cg02655630	0.26652	0.414490811	0.637096	1.54E-05	hypermethylated
cg20474425	0.33196	0.516210811	0.636951	1.75E-05	hypermethylated
cg05764839	0.33513	0.521086486	0.636802	1.77E-06	hypermethylated
cg24391460	0.33362	0.518725405	0.636765	3.49E-05	hypermethylated
cg04268624	0.41571	0.646356757	0.636753	0.000374	hypermethylated
cg18280830	0.32992	0.512909189	0.636587	0.00013	hypermethylated
cg25590826	0.32368	0.503163243	0.636458	0.000216	hypermethylated
cg10068417	0.39939	0.62074973	0.636214	8.22E-05	hypermethylated
cg16752592	0.39587	0.615071892	0.635728	0.006507	hypermethylated
cg13521620	0.45331	0.704262162	0.635615	0.000271	hypermethylated
cg11696200	0.47955	0.74488	0.635327	0.005963	hypermethylated
cg18335796	0.38146	0.592481081	0.635237	0.000237	hypermethylated
cg04907173	0.32397	0.50316	0.635157	6.31E-05	hypermethylated
cg03758467	0.33733	0.523892973	0.635111	3.23E-05	hypermethylated
cg07562888	0.41949	0.651473514	0.63507	0.000296	hypermethylated
cg23015341	0.21451	0.333121081	0.635002	2.64E-05	hypermethylated
cg05574272	0.3026	0.469858919	0.634816	0.000112	hypermethylated
cg07462292	0.24245	0.376412432	0.634627	1.15E-05	hypermethylated
cg27151303	0.35659	0.553615135	0.634617	0.000358	hypermethylated
cg11382529	0.4212	0.653897838	0.63456	0.000102	hypermethylated
cg21707172	0.4881	0.757744865	0.634535	2.21E-05	hypermethylated
cg03501539	0.45951	0.713225405	0.634262	0.008208	hypermethylated
cg09247060	0.198	0.307303784	0.634165	7.29E-05	hypermethylated
cg04737133	0.26094	0.404937838	0.633982	1.54E-05	hypermethylated
cg06726820	0.36785	0.570823784	0.633928	0.000528	hypermethylated
cg14830601	0.29182	0.452814595	0.633842	5.58E-05	hypermethylated
cg25967031	0.46594	0.722991351	0.633834	0.000789	hypermethylated
cg03785076	0.3208	0.497706486	0.633621	0.004287	hypermethylated

cg04161526	0.26987	0.418663784	0.633528	0.00024	hypermethylated
cg14535980	0.27749	0.430422703	0.633318	2.92E-05	hypermethylated
cg07852825	0.26585	0.412367027	0.633317	4.36E-05	hypermethylated
cg17280346	0.35679	0.553412432	0.63328	0.000174	hypermethylated
cg27454064	0.32083	0.497540541	0.633005	7.84E-05	hypermethylated
cg05341781	0.42267	0.655358378	0.632752	0.000681	hypermethylated
cg01550348	0.45572	0.706574595	0.632694	0.002189	hypermethylated
cg25845985	0.27072	0.419700541	0.632559	6.54E-05	hypermethylated
cg20815297	0.24699	0.382862703	0.632374	2.42E-05	hypermethylated
cg04273148	0.48344	0.749378378	0.632358	0.000382	hypermethylated
cg12453631	0.28495	0.441684324	0.632307	3.26E-06	hypermethylated
cg20366906	0.2939	0.455551351	0.632288	3.85E-06	hypermethylated
cg02331883	0.32676	0.506464865	0.632231	0.000174	hypermethylated
cg26509965	0.27257	0.42247027	0.632223	0.000137	hypermethylated
cg23440004	0.23363	0.362096216	0.632148	0.00012	hypermethylated
cg13075537	0.4239	0.656514595	0.631103	0.003705	hypermethylated
cg01709312	0.48629	0.753116757	0.631057	2.39E-05	hypermethylated
cg24366314	0.29416	0.455554054	0.631021	0.000229	hypermethylated
cg19021738	0.19645	0.304232432	0.631012	0.000178	hypermethylated
cg05973337	0.20004	0.309769189	0.630905	2.18E-05	hypermethylated
cg03256198	0.5492	0.850363784	0.630749	0.00017	hypermethylated
cg12534008	0.30342	0.469793514	0.630711	9.49E-05	hypermethylated
cg26276327	0.32037	0.496017297	0.630651	5.58E-05	hypermethylated
cg14672084	0.48424	0.749666486	0.630527	0.000287	hypermethylated
cg04374393	0.27795	0.430158378	0.630043	3.30E-06	hypermethylated
cg21286967	0.26091	0.403730811	0.629841	0.001481	hypermethylated
cg22352078	0.29105	0.45034973	0.629779	4.82E-05	hypermethylated
cg09182138	0.3267	0.50550973	0.629772	5.28E-06	hypermethylated
cg13759172	0.25365	0.392448649	0.629665	1.15E-05	hypermethylated
cg19403534	0.29702	0.459518378	0.629562	0.000142	hypermethylated
cg09561125	0.33839	0.523495135	0.629489	2.32E-05	hypermethylated
cg07464571	0.27587	0.426768108	0.629464	0.000159	hypermethylated
cg03788227	0.39933	0.61768	0.629278	0.0006	hypermethylated
cg11739399	0.29871	0.461983243	0.629095	0.000117	hypermethylated
cg13680388	0.46896	0.725138919	0.628793	0.001511	hypermethylated
cg07816637	0.28063	0.433927568	0.628785	8.37E-06	hypermethylated
cg20710709	0.27739	0.428677297	0.627976	5.72E-05	hypermethylated
cg07284273	0.19353	0.299029189	0.627729	0.000912	hypermethylated
cg11388320	0.28928	0.446856216	0.627344	0.0002	hypermethylated
cg25725843	0.32189	0.497092432	0.626946	2.51E-05	hypermethylated
cg13381984	0.33429	0.516208649	0.626854	8.52E-05	hypermethylated
cg15802263	0.26875	0.41499027	0.626813	0.000132	hypermethylated
cg06908618	0.42748	0.66009027	0.626806	0.003195	hypermethylated
cg18356785	0.38159	0.589212973	0.626766	0.00291	hypermethylated
cg21395577	0.23728	0.366295135	0.626416	0.00032	hypermethylated
cg13475333	0.42454	0.65534973	0.626365	0.004365	hypermethylated
cg26206456	0.44872	0.692471892	0.62594	0.003506	hypermethylated
cg19547547	0.30999	0.478247027	0.625534	0.000106	hypermethylated
cg04492228	0.36905	0.569352432	0.625506	0.00024	hypermethylated
cg04996195	0.32829	0.506456757	0.625468	0.000613	hypermethylated
cg16790847	0.3088	0.476268108	0.625101	2.85E-05	hypermethylated
cg23372684	0.34039	0.524942162	0.62497	7.93E-06	hypermethylated
cg16890796	0.28327	0.436795135	0.624779	0.000219	hypermethylated
cg21544633	0.21048	0.324528649	0.624663	0.000534	hypermethylated
cg00713567	0.27055	0.417135135	0.62462	0.000234	hypermethylated
cg17038626	0.55718	0.859056757	0.62461	0.000557	hypermethylated
cg16406892	0.3741	0.576784865	0.624609	0.000342	hypermethylated
cg05977669	0.30791	0.47463027	0.624295	0.000174	hypermethylated

cg03169508	0.29861	0.460242162	0.624131	0.000148	hypermethylated
cg11108474	0.30393	0.468395676	0.623989	0.000104	hypermethylated
cg14118850	0.33051	0.509343243	0.623944	0.005223	hypermethylated
cg16361966	0.22003	0.339034595	0.623732	0.000113	hypermethylated
cg25649641	0.27598	0.425142162	0.623382	0.000143	hypermethylated
cg12299795	0.46159	0.711032432	0.623303	0.005508	hypermethylated
cg00699219	0.2759	0.424957838	0.623174	9.31E-06	hypermethylated
cg10640072	0.32816	0.505404865	0.62304	2.57E-05	hypermethylated
cg11762968	0.28796	0.443317838	0.622473	2.45E-05	hypermethylated
cg02851062	0.28087	0.432378378	0.622392	2.61E-05	hypermethylated
cg22583148	0.31666	0.487267027	0.621778	5.32E-05	hypermethylated
cg18998365	0.31124	0.478839459	0.621515	0.002599	hypermethylated
cg10116505	0.25342	0.389854595	0.621406	5.28E-06	hypermethylated
cg05967403	0.29987	0.461251351	0.621216	5.19E-05	hypermethylated
cg03322353	0.42869	0.659364324	0.621141	0.00134	hypermethylated
cg19810034	0.21798	0.335262703	0.621096	9.19E-06	hypermethylated
cg23082862	0.31128	0.478565405	0.620503	2.21E-05	hypermethylated
cg06488256	0.30379	0.467009189	0.620377	0.000102	hypermethylated
cg22400015	0.34056	0.523518378	0.620331	2.53E-06	hypermethylated
cg11739633	0.37018	0.568990811	0.620178	3.57E-05	hypermethylated
cg00287096	0.38117	0.585876216	0.620161	0.00028	hypermethylated
cg04096767	0.29271	0.449854054	0.619985	0.000155	hypermethylated
cg00805880	0.32837	0.504653514	0.619971	0.000374	hypermethylated
cg03032025	0.45185	0.694399459	0.619922	0.002965	hypermethylated
cg02154345	0.28992	0.445509189	0.6198	0.000229	hypermethylated
cg15265085	0.37272	0.572685946	0.619652	0.002066	hypermethylated
cg10257302	0.37236	0.572091892	0.619549	0.000501	hypermethylated
cg19514381	0.35081	0.538784324	0.619018	5.86E-05	hypermethylated
cg19718563	0.39918	0.613008649	0.618868	0.000219	hypermethylated
cg03613782	0.28547	0.438198919	0.618247	4.94E-05	hypermethylated
cg06897145	0.26351	0.404447027	0.618093	1.42E-05	hypermethylated
cg03522150	0.50572	0.776168108	0.61803	0.010211	hypermethylated
cg05543520	0.39981	0.61360973	0.618007	0.000741	hypermethylated
cg01462607	0.28375	0.435435676	0.617839	0.000399	hypermethylated
cg03431741	0.29179	0.447686486	0.617558	5.38E-05	hypermethylated
cg21436572	0.36097	0.553791351	0.617464	0.00102	hypermethylated
cg03877332	0.29961	0.459613514	0.617335	1.46E-05	hypermethylated
cg19274703	0.34244	0.525216216	0.61706	0.001164	hypermethylated
cg03405909	0.38063	0.583688108	0.616808	0.000256	hypermethylated
cg15461491	0.22364	0.342896757	0.616596	2.07E-05	hypermethylated
cg17371334	0.28249	0.433056216	0.616355	7.47E-05	hypermethylated
cg08146945	0.34779	0.533122703	0.616251	1.54E-05	hypermethylated
cg07459170	0.25916	0.397228649	0.616127	5.06E-05	hypermethylated
cg01105418	0.41532	0.636506486	0.615952	0.002274	hypermethylated
cg03707183	0.25678	0.393525405	0.615924	0.000426	hypermethylated
cg05524354	0.43879	0.672353514	0.615689	2.78E-05	hypermethylated
cg05258736	0.23929	0.366642703	0.615615	3.36E-05	hypermethylated
cg08750504	0.33431	0.512218919	0.615574	0.000107	hypermethylated
cg10038618	0.31109	0.476615135	0.615493	5.31E-05	hypermethylated
cg00766964	0.4166	0.638241081	0.615439	0.003571	hypermethylated
cg14277923	0.28126	0.430714595	0.614828	0.00102	hypermethylated
cg14640592	0.3362	0.514834054	0.614788	2.30E-05	hypermethylated
cg08732659	0.29897	0.45782	0.61478	4.01E-06	hypermethylated
cg25785397	0.28933	0.44294973	0.614427	0.000132	hypermethylated
cg16758800	0.25818	0.395138919	0.613983	2.10E-05	hypermethylated
cg24519147	0.36965	0.565684865	0.613839	8.03E-05	hypermethylated
cg13925360	0.24782	0.37921027	0.613705	9.95E-05	hypermethylated
cg23662142	0.36191	0.553756757	0.613621	0.000335	hypermethylated

cg13524919	0.33052	0.505715135	0.613587	5.06E-05	hypermethylated
cg13412754	0.37627	0.575659459	0.613447	0.021224	hypermethylated
cg13327545	0.36188	0.553595135	0.61332	1.99E-05	hypermethylated
cg09828714	0.32118	0.491331351	0.613314	0.000214	hypermethylated
cg10556005	0.51123	0.782015135	0.613224	0.001635	hypermethylated
cg13297560	0.3899	0.596416216	0.613215	0.010211	hypermethylated
cg23098195	0.26375	0.403382703	0.612978	2.45E-05	hypermethylated
cg23347399	0.35979	0.550263784	0.612968	0.00064	hypermethylated
cg00646347	0.34186	0.52264973	0.612439	4.15E-05	hypermethylated
cg18449120	0.34827	0.532387568	0.612271	1.58E-05	hypermethylated
cg02328239	0.23741	0.362894054	0.612168	3.57E-05	hypermethylated
cg23178550	0.36788	0.562085946	0.611555	3.23E-05	hypermethylated
cg22655696	0.3212	0.490728108	0.611452	1.80E-05	hypermethylated
cg04908960	0.55494	0.847675135	0.61118	0.000408	hypermethylated
cg04890851	0.29354	0.448373514	0.611144	5.45E-05	hypermethylated
cg23368787	0.34156	0.521542703	0.610646	2.38E-05	hypermethylated
cg01097180	0.28349	0.432860541	0.610604	0.000107	hypermethylated
cg18747039	0.24603	0.375662703	0.610604	5.58E-05	hypermethylated
cg27639662	0.31544	0.481574595	0.610394	0.000287	hypermethylated
cg16175941	0.51674	0.788689189	0.610018	0.005706	hypermethylated
cg11100804	0.38819	0.592462703	0.609961	3.07E-05	hypermethylated
cg01525538	0.38341	0.585121081	0.609847	6.39E-06	hypermethylated
cg15117739	0.24767	0.377898378	0.609579	0.002856	hypermethylated
cg01178680	0.36157	0.551670811	0.609533	7.62E-06	hypermethylated
cg01798261	0.31663	0.483044324	0.609358	4.36E-05	hypermethylated
cg22353551	0.33007	0.503534595	0.609319	2.27E-05	hypermethylated
cg08874609	0.26046	0.397325946	0.609261	0.000168	hypermethylated
cg09155881	0.37597	0.573392973	0.608907	0.002026	hypermethylated
cg09312576	0.27607	0.420939459	0.608579	1.54E-05	hypermethylated
cg21676440	0.53771	0.819871351	0.608569	0.00096	hypermethylated
cg16184803	0.27641	0.421434054	0.608497	4.15E-05	hypermethylated
cg17298751	0.44556	0.679183784	0.608182	3.21E-06	hypermethylated
cg19764418	0.44088	0.671877838	0.607813	0.000445	hypermethylated
cg19802138	0.27073	0.412541622	0.607685	4.82E-05	hypermethylated
cg12069267	0.39652	0.604205405	0.607645	2.89E-05	hypermethylated
cg01971160	0.28675	0.436836757	0.607301	0.000112	hypermethylated
cg16026922	0.27846	0.4242	0.607275	0.000129	hypermethylated
cg00816406	0.39449	0.600815135	0.606932	0.011649	hypermethylated
cg02105152	0.25856	0.393584324	0.606174	8.63E-05	hypermethylated
cg18452695	0.38891	0.591801081	0.605676	0.000191	hypermethylated
cg12490835	0.28939	0.440351351	0.60564	0.000129	hypermethylated
cg14476101	0.39913	0.607297297	0.605544	0.000501	hypermethylated
cg21102121	0.3449	0.524743784	0.605435	7.47E-05	hypermethylated
cg18757468	0.4691	0.713630811	0.605282	0.002253	hypermethylated
cg15895690	0.28156	0.428241081	0.604981	1.01E-05	hypermethylated
cg10297792	0.39135	0.595115676	0.604711	0.000358	hypermethylated
cg25936177	0.28127	0.427711351	0.604682	1.02E-05	hypermethylated
cg09606807	0.40653	0.618129189	0.604547	8.63E-05	hypermethylated
cg09183671	0.36447	0.554111892	0.604377	0.00071	hypermethylated
cg08082810	0.33479	0.508942162	0.604245	5.65E-05	hypermethylated
cg08650890	0.34835	0.529531892	0.60418	0.001949	hypermethylated
cg05238917	0.31758	0.482715676	0.604054	0.000178	hypermethylated
cg16822666	0.38723	0.588542703	0.603956	0.000893	hypermethylated
cg09254980	0.22418	0.340671892	0.603725	1.64E-05	hypermethylated
cg20702419	0.26951	0.409542162	0.603673	0.000449	hypermethylated
cg07742404	0.29444	0.447386486	0.603548	6.54E-05	hypermethylated
cg05046306	0.36609	0.55624	0.603509	0.004951	hypermethylated
cg21476494	0.33859	0.514435676	0.603451	0.000594	hypermethylated

cg05875410	0.31486	0.478378378	0.603442	0.00012	hypermethylated
cg00222341	0.24987	0.379633514	0.60343	0.000116	hypermethylated
cg22017213	0.2051	0.311547568	0.603125	0.002026	hypermethylated
cg07587893	0.24048	0.365248649	0.602962	5.25E-05	hypermethylated
cg19688403	0.25108	0.381281622	0.60271	5.06E-05	hypermethylated
cg03497399	0.38373	0.582714595	0.602698	4.94E-05	hypermethylated
cg07676002	0.29568	0.448976757	0.602604	2.45E-05	hypermethylated
cg25497399	0.28421	0.431474595	0.602318	0.00035	hypermethylated
cg22646995	0.26664	0.404667027	0.601842	0.000219	hypermethylated
cg12243007	0.26696	0.405064324	0.601527	0.000338	hypermethylated
cg02812891	0.37463	0.568385946	0.601404	0.000626	hypermethylated
cg04386759	0.55033	0.834857297	0.601233	0.001684	hypermethylated
cg19715094	0.40109	0.608456216	0.601227	0.004691	hypermethylated
cg14468658	0.28252	0.428574595	0.601193	7.47E-05	hypermethylated
cg05740045	0.3867	0.58644	0.600769	3.07E-05	hypermethylated
cg18633154	0.3369	0.510899459	0.600719	0.016315	hypermethylated
cg01278720	0.20652	0.313099459	0.60034	7.03E-05	hypermethylated
cg27034935	0.33329	0.505252973	0.600228	0.000142	hypermethylated
cg03472755	0.27393	0.415248649	0.600168	0.000191	hypermethylated
cg06480171	0.28392	0.430381081	0.60013	0.000219	hypermethylated
cg05349624	0.27481	0.416551892	0.600062	0.000191	hypermethylated
cg18313661	0.38602	0.585082162	0.599964	0.000116	hypermethylated
cg12052661	0.323	0.489488108	0.59974	1.31E-05	hypermethylated
cg20948486	0.27835	0.421809189	0.59969	0.000626	hypermethylated
cg19016652	0.34549	0.523538378	0.599651	3.23E-05	hypermethylated
cg05781417	0.25433	0.385314595	0.599335	0.001803	hypermethylated
cg23832237	0.30539	0.462604865	0.599128	5.72E-05	hypermethylated
cg08520746	0.24976	0.378334595	0.59912	5.65E-05	hypermethylated
cg05886546	0.29741	0.45051027	0.599107	0.000159	hypermethylated
cg27213509	0.24885	0.376943784	0.599073	1.04E-05	hypermethylated
cg26046467	0.37321	0.565124324	0.598581	1.54E-05	hypermethylated
cg00273449	0.26274	0.397840541	0.598554	4.70E-05	hypermethylated
cg00637144	0.35433	0.536492973	0.598466	0.00064	hypermethylated
cg25859099	0.33497	0.507049189	0.598094	3.57E-05	hypermethylated
cg25390440	0.32982	0.499128108	0.597731	7.29E-05	hypermethylated
cg02438611	0.43712	0.661394595	0.597482	0.000126	hypermethylated
cg15160709	0.47387	0.716936757	0.597355	0.000857	hypermethylated
cg21837669	0.27703	0.419103784	0.597265	0.000283	hypermethylated
cg19430694	0.22533	0.340887027	0.597254	0.005606	hypermethylated
cg20689135	0.30279	0.458043784	0.597168	0.0003	hypermethylated
cg17448336	0.37176	0.562343784	0.597081	3.23E-05	hypermethylated
cg14585371	0.27322	0.413273514	0.597034	2.95E-06	hypermethylated
cg04407305	0.26837	0.405925946	0.596993	7.29E-05	hypermethylated
cg18348836	0.46199	0.698692973	0.596797	0.01274	hypermethylated
cg25079691	0.249	0.376544324	0.596674	0.000142	hypermethylated
cg26562772	0.41013	0.620194595	0.59664	0.007408	hypermethylated
cg03283421	0.55052	0.832479459	0.59662	0.000445	hypermethylated
cg19527617	0.24584	0.371736216	0.59656	0.000296	hypermethylated
cg04854226	0.38415	0.580865405	0.596534	0.000435	hypermethylated
cg09998861	0.30831	0.466188108	0.596531	0.000145	hypermethylated
cg07018577	0.54103	0.817955135	0.596313	0.004649	hypermethylated
cg11793699	0.42501	0.642521081	0.596247	0.000772	hypermethylated
cg26516532	0.30551	0.461776757	0.595976	0.000113	hypermethylated
cg16376108	0.23996	0.362688649	0.595938	1.71E-05	hypermethylated
cg06275642	0.26357	0.398189189	0.595268	0.00032	hypermethylated
cg10595952	0.46281	0.699178919	0.595242	0.003078	hypermethylated
cg10261191	0.34839	0.526318378	0.595233	0.000229	hypermethylated
cg12534424	0.29287	0.442429189	0.595186	0.000189	hypermethylated

cg06794543	0.44925	0.678653514	0.595157	2.54E-05	hypermethylated
cg10989634	0.24407	0.368666486	0.595021	2.85E-05	hypermethylated
cg02331262	0.44228	0.66801027	0.59491	0.000313	hypermethylated
cg13301003	0.37487	0.566164324	0.59483	0.000138	hypermethylated
cg02229261	0.30832	0.465532432	0.594453	0.000741	hypermethylated
cg09692428	0.42411	0.640358378	0.594441	0.000563	hypermethylated
cg15959252	0.40528	0.611905946	0.594391	0.000607	hypermethylated
cg19696083	0.3081	0.465018378	0.593889	0.000176	hypermethylated
cg08682532	0.33913	0.511796757	0.593733	0.000132	hypermethylated
cg10848373	0.32366	0.488425405	0.593659	0.00071	hypermethylated
cg04845915	0.40152	0.605917838	0.59365	9.57E-06	hypermethylated
cg13419986	0.26832	0.404896216	0.593598	0.00054	hypermethylated
cg11468819	0.31014	0.467931892	0.593379	0.000232	hypermethylated
cg13578194	0.25158	0.379561081	0.593315	0.006339	hypermethylated
cg04570362	0.51495	0.776816757	0.593142	0.009394	hypermethylated
cg22830707	0.2617	0.39474973	0.593024	2.89E-05	hypermethylated
cg22108567	0.32341	0.487827568	0.593007	0.000756	hypermethylated
cg02047300	0.37653	0.567931892	0.592953	1.04E-05	hypermethylated
cg17432857	0.3872	0.584017297	0.592932	0.001052	hypermethylated
cg09578568	0.29758	0.448787568	0.592755	1.35E-05	hypermethylated
cg01261206	0.53101	0.800601622	0.592346	0.001839	hypermethylated
cg21123355	0.24211	0.365024865	0.592332	0.000112	hypermethylated
cg07546139	0.40787	0.614917297	0.592283	0.000495	hypermethylated
cg24427376	0.30168	0.454788649	0.592177	0.000327	hypermethylated
cg24024424	0.2838	0.427742703	0.591869	0.000148	hypermethylated
cg00342530	0.34035	0.512974595	0.591868	0.006122	hypermethylated
cg06368300	0.20284	0.305716757	0.591853	0.003021	hypermethylated
cg22902505	0.34822	0.524777838	0.591708	0.001041	hypermethylated
cg26006951	0.26783	0.403537297	0.591384	0.000756	hypermethylated
cg03700287	0.2951	0.444583243	0.59125	9.49E-05	hypermethylated
cg03769116	0.46552	0.701279459	0.591146	0.000115	hypermethylated
cg15751116	0.27668	0.41673027	0.590896	0.000501	hypermethylated
cg10026317	0.3514	0.529261622	0.590867	3.19E-05	hypermethylated
cg25066972	0.4792	0.721698378	0.590768	0.002007	hypermethylated
cg06547490	0.24259	0.365305405	0.590583	0.010468	hypermethylated
cg19956914	0.27372	0.412168649	0.590534	0.001603	hypermethylated
cg15123428	0.38311	0.576798919	0.59031	0.006975	hypermethylated
cg15084803	0.35596	0.535865405	0.590156	0.001734	hypermethylated
cg22185643	0.27698	0.416965405	0.590146	4.15E-05	hypermethylated
cg11751117	0.52217	0.785932973	0.589887	0.00099	hypermethylated
cg06882544	0.29683	0.446704865	0.589685	9.57E-06	hypermethylated
cg00595030	0.31878	0.479736216	0.58968	6.38E-05	hypermethylated
cg24092282	0.31782	0.478280541	0.589647	0.00032	hypermethylated
cg20450471	0.32898	0.495047568	0.589567	2.92E-05	hypermethylated
cg07892597	0.34201	0.514644865	0.589539	1.84E-05	hypermethylated
cg05722504	0.28057	0.422167568	0.589455	3.40E-05	hypermethylated
cg09271157	0.29207	0.439464865	0.589434	0.000112	hypermethylated
cg06193628	0.39414	0.593022703	0.589379	0.001875	hypermethylated
cg10169241	0.34212	0.514711892	0.589263	0.000626	hypermethylated
cg01159576	0.2461	0.370177297	0.588972	0.000268	hypermethylated
cg26811372	0.34663	0.52137027	0.588912	0.0006	hypermethylated
cg02311725	0.29733	0.447188108	0.588817	6.57E-06	hypermethylated
cg24932585	0.34721	0.522174054	0.588722	9.49E-05	hypermethylated
cg08178786	0.32325	0.48601027	0.588336	0.000253	hypermethylated
cg24330379	0.25587	0.384688108	0.588278	0.000839	hypermethylated
cg24459147	0.2343	0.352207568	0.588069	2.39E-05	hypermethylated
cg22927247	0.30853	0.463767568	0.587991	4.86E-06	hypermethylated
cg19633390	0.49925	0.75039027	0.587879	0.000399	hypermethylated

cg05239225	0.35561	0.53447027	0.587814	0.006737	hypermethylated
cg14042889	0.38189	0.573964865	0.587805	0.003255	hypermethylated
cg09416908	0.47385	0.712151351	0.587753	0.000358	hypermethylated
cg26819611	0.21219	0.318853514	0.587537	0.000417	hypermethylated
cg06898293	0.43659	0.656029189	0.587481	0.004059	hypermethylated
cg04191300	0.40447	0.607745405	0.587434	0.002409	hypermethylated
cg13661519	0.3691	0.554509189	0.5872	0.002699	hypermethylated
cg10432422	0.30061	0.45158973	0.58712	5.86E-05	hypermethylated
cg25027788	0.31552	0.473951351	0.587008	0.00513	hypermethylated
cg12334871	0.52811	0.793206486	0.586858	0.000626	hypermethylated
cg04578903	0.305	0.457903784	0.586235	0.000129	hypermethylated
cg00154357	0.35968	0.539942703	0.586092	1.71E-05	hypermethylated
cg06222162	0.25684	0.385537297	0.586001	0.002106	hypermethylated
cg15400629	0.3052	0.458052432	0.585758	4.58E-05	hypermethylated
cg06219103	0.4983	0.747834595	0.585705	1.09E-05	hypermethylated
cg08244443	0.32882	0.493255135	0.585036	2.85E-05	hypermethylated
cg17455088	0.32524	0.48784973	0.584932	8.42E-05	hypermethylated
cg14742809	0.3136	0.470376216	0.58489	0.000613	hypermethylated
cg19103219	0.35323	0.52978	0.584786	0.004951	hypermethylated
cg07539798	0.36469	0.546884865	0.584566	0.000204	hypermethylated
cg07815521	0.28936	0.433902162	0.584504	0.003195	hypermethylated
cg18839637	0.32995	0.494751351	0.584456	3.07E-05	hypermethylated
cg11375458	0.40727	0.610682703	0.584437	4.58E-05	hypermethylated
cg00651488	0.4018	0.602457297	0.584381	0.000274	hypermethylated
cg20016411	0.27643	0.414474054	0.584368	2.51E-05	hypermethylated
cg13452162	0.32884	0.492982703	0.584151	0.001768	hypermethylated
cg12836958	0.45662	0.684492973	0.584042	0.002856	hypermethylated
cg15677916	0.26088	0.390974054	0.583687	9.27E-05	hypermethylated
cg24483493	0.40472	0.606505405	0.583596	0.008348	hypermethylated
cg13826452	0.32957	0.493837297	0.583451	0.000132	hypermethylated
cg01603559	0.22398	0.335604865	0.583394	0.000474	hypermethylated
cg00558689	0.38871	0.582381081	0.583269	0.001571	hypermethylated
cg18141888	0.36736	0.55032973	0.583102	3.95E-05	hypermethylated
cg12417775	0.4202	0.629456216	0.58303	0.000335	hypermethylated
cg20996351	0.33807	0.506366486	0.58286	4.82E-05	hypermethylated
cg21542793	0.22785	0.341235676	0.582684	0.000104	hypermethylated
cg25664813	0.52712	0.789422703	0.582667	0.00134	hypermethylated
cg11555228	0.28951	0.433519459	0.582484	1.84E-05	hypermethylated
cg08823554	0.56997	0.853435676	0.582396	0.000262	hypermethylated
cg01814898	0.55089	0.824851892	0.582371	0.003136	hypermethylated
cg24076884	0.28702	0.429715135	0.582229	4.70E-05	hypermethylated
cg04603391	0.38321	0.573721081	0.582214	0.000166	hypermethylated
cg07766263	0.33406	0.500089189	0.582078	7.65E-05	hypermethylated
cg15260428	0.58818	0.880381081	0.58187	4.15E-05	hypermethylated
cg15792134	0.46889	0.701775135	0.581759	0.028186	hypermethylated
cg06971092	0.34091	0.510206486	0.58169	0.000293	hypermethylated
cg15291243	0.31278	0.468095135	0.581654	0.000101	hypermethylated
cg21826699	0.31278	0.468011892	0.581397	0.000224	hypermethylated
cg12530503	0.30212	0.452046486	0.581349	3.44E-05	hypermethylated
cg09601584	0.2451	0.366725405	0.58133	7.12E-05	hypermethylated
cg25902889	0.47109	0.704829189	0.581271	0.002106	hypermethylated
cg14914238	0.45056	0.673933514	0.580887	0.003506	hypermethylated
cg06816350	0.46199	0.690999459	0.580823	0.004691	hypermethylated
cg04726439	0.32702	0.489098919	0.580747	3.07E-05	hypermethylated
cg13333954	0.3574	0.534527568	0.580725	0.000256	hypermethylated
cg27344587	0.34747	0.519653514	0.580662	0.00018	hypermethylated
cg01295399	0.3387	0.506523243	0.58062	5.45E-05	hypermethylated
cg01794802	0.45141	0.67500973	0.58047	0.002189	hypermethylated

cg06536868	0.37863	0.566166486	0.580438	0.000511	hypermethylated
cg17489908	0.38751	0.579208108	0.579848	0.000338	hypermethylated
cg22134325	0.33336	0.498204324	0.579657	1.80E-05	hypermethylated
cg10612492	0.24802	0.370625405	0.579505	0.004526	hypermethylated
cg13313813	0.28779	0.430023243	0.579398	1.87E-05	hypermethylated
cg26708874	0.30668	0.458190811	0.579214	0.000107	hypermethylated
cg13102348	0.32357	0.48342	0.579199	0.009315	hypermethylated
cg16572603	0.31811	0.475246486	0.57915	2.71E-05	hypermethylated
cg08812889	0.36422	0.544122162	0.57912	7.38E-05	hypermethylated
cg14974749	0.44668	0.667150811	0.578771	0.005963	hypermethylated
cg14645017	0.38795	0.579152432	0.578072	0.004776	hypermethylated
cg13565138	0.35342	0.527590811	0.578036	0.001314	hypermethylated
cg22063654	0.33795	0.504495676	0.578032	0.000646	hypermethylated
cg05561193	0.35495	0.529853514	0.577978	0.002147	hypermethylated
cg15268244	0.2227	0.332383784	0.577748	0.001141	hypermethylated
cg09144707	0.29933	0.446737297	0.57769	7.12E-05	hypermethylated
cg05337753	0.50528	0.754098919	0.577671	0.000653	hypermethylated
cg16387467	0.33499	0.499917297	0.577571	3.90E-05	hypermethylated
cg18793404	0.33942	0.506417297	0.577255	8.63E-05	hypermethylated
cg18891762	0.28463	0.424635135	0.577136	0.000327	hypermethylated
cg07453407	0.54354	0.810556757	0.576527	0.002363	hypermethylated
cg01432663	0.34536	0.514952973	0.57634	0.003225	hypermethylated
cg00380860	0.25896	0.386081622	0.576177	0.000303	hypermethylated
cg19536929	0.35444	0.528391892	0.576067	7.84E-05	hypermethylated
cg01178099	0.37487	0.558815135	0.575981	0.000733	hypermethylated
cg03296797	0.32236	0.480498378	0.575859	8.84E-05	hypermethylated
cg21838979	0.29577	0.440811892	0.575687	2.21E-05	hypermethylated
cg10471743	0.46707	0.696073514	0.575601	0.000253	hypermethylated
cg16824643	0.49989	0.744957838	0.575548	0.000178	hypermethylated
cg00854273	0.38022	0.566612973	0.575529	0.000274	hypermethylated
cg21092551	0.53874	0.80283027	0.575506	0.004287	hypermethylated
cg24395452	0.34313	0.511275135	0.575345	1.28E-05	hypermethylated
cg01628509	0.24669	0.367563784	0.575295	0.000378	hypermethylated
cg12668523	0.48591	0.723918919	0.575139	0.00546	hypermethylated
cg22010052	0.30573	0.45534973	0.574717	8.22E-05	hypermethylated
cg04071270	0.47344	0.705131351	0.57471	5.72E-05	hypermethylated
cg27496615	0.30445	0.45336	0.574452	7.74E-05	hypermethylated
cg00969405	0.43534	0.648258378	0.574426	0.001452	hypermethylated
cg07547549	0.36497	0.54344	0.574343	0.000335	hypermethylated
cg01224715	0.4374	0.651207568	0.574164	0.000112	hypermethylated
cg16817992	0.35298	0.525508649	0.574128	3.49E-05	hypermethylated
cg17520027	0.34125	0.508007568	0.574021	0.001949	hypermethylated
cg13725172	0.37514	0.558443784	0.573983	0.0017	hypermethylated
cg00929376	0.33049	0.491916216	0.573806	0.003473	hypermethylated
cg20706778	0.37176	0.553343243	0.573803	0.002274	hypermethylated
cg25666233	0.36436	0.542198919	0.573458	0.000178	hypermethylated
cg14164099	0.33593	0.499871892	0.573398	0.000805	hypermethylated
cg03989260	0.26861	0.399652973	0.573235	2.21E-05	hypermethylated
cg21581504	0.37407	0.556514595	0.573111	0.000764	hypermethylated
cg04007726	0.40526	0.602915676	0.573108	0.0012	hypermethylated
cg05150023	0.29345	0.436502162	0.572874	8.22E-05	hypermethylated
cg02111951	0.34987	0.52033027	0.572609	9.07E-06	hypermethylated
cg07332002	0.25041	0.372391892	0.572529	0.00035	hypermethylated
cg00510437	0.39423	0.586067568	0.572029	3.67E-05	hypermethylated
cg24139639	0.32573	0.484131351	0.571722	2.42E-05	hypermethylated
cg19763863	0.32279	0.479754054	0.571699	0.001684	hypermethylated
cg02034887	0.37816	0.562023784	0.571634	0.01146	hypermethylated
cg01579600	0.36528	0.542776757	0.571356	0.005223	hypermethylated

cg02905900	0.20568	0.30562	0.571337	0.000857	hypermethylated
cg03970229	0.29416	0.437087027	0.571319	0.000123	hypermethylated
cg24622589	0.3239	0.481224324	0.571161	0.012333	hypermethylated
cg07482257	0.3702	0.550005946	0.571142	0.002189	hypermethylated
cg01340163	0.34217	0.508313514	0.571005	4.82E-05	hypermethylated
cg11728747	0.46984	0.697969189	0.570994	0.001327	hypermethylated
cg02120584	0.32025	0.475678919	0.57079	0.000168	hypermethylated
cg15986413	0.24866	0.369324865	0.570716	0.000172	hypermethylated
cg10586870	0.2933	0.435598919	0.570623	0.002274	hypermethylated
cg16926102	0.30572	0.453917838	0.57022	3.40E-05	hypermethylated
cg26282731	0.47789	0.709541081	0.570208	0.00095	hypermethylated
cg06493930	0.43929	0.652224865	0.570196	0.004951	hypermethylated
cg10149054	0.29798	0.442415676	0.570187	0.000163	hypermethylated
cg12686273	0.35691	0.529794595	0.569873	3.76E-05	hypermethylated
cg23095192	0.3056	0.453629189	0.569869	0.000342	hypermethylated
cg10501976	0.36174	0.536951351	0.569838	4.76E-05	hypermethylated
cg27051315	0.3027	0.449186486	0.569426	0.00035	hypermethylated
cg22481263	0.33478	0.496789189	0.56942	7.93E-06	hypermethylated
cg15011409	0.23735	0.352197297	0.569368	0.000426	hypermethylated
cg05465604	0.2955	0.438406486	0.569111	0.000575	hypermethylated
cg04043150	0.3093	0.458832973	0.568962	2.29E-06	hypermethylated
cg03980550	0.32647	0.484273514	0.568872	0.008069	hypermethylated
cg16463460	0.36105	0.535564865	0.568863	2.64E-05	hypermethylated
cg23497569	0.42862	0.635774054	0.568815	0.019361	hypermethylated
cg21099419	0.59108	0.876741622	0.568798	0.000256	hypermethylated
cg09643136	0.35403	0.525122703	0.568783	3.07E-05	hypermethylated
cg07503630	0.22764	0.337649189	0.568771	0.002478	hypermethylated
cg25705519	0.3406	0.505181622	0.568724	0.000107	hypermethylated
cg14509403	0.35722	0.529796216	0.568625	3.11E-05	hypermethylated
cg24691891	0.39367	0.583801081	0.56849	0.003538	hypermethylated
cg26092675	0.34356	0.509478378	0.568459	0.002699	hypermethylated
cg03162823	0.40854	0.605765405	0.568282	0.001481	hypermethylated
cg10531355	0.40693	0.603291351	0.568074	0.00024	hypermethylated
cg00298065	0.41332	0.612717297	0.567962	7.84E-05	hypermethylated
cg15887459	0.30646	0.454297297	0.567938	0.000221	hypermethylated
cg25457886	0.36576	0.542176757	0.567866	0.000129	hypermethylated
cg09764761	0.39691	0.58822	0.567544	0.0017	hypermethylated
cg01020758	0.27846	0.412654054	0.567463	0.00099	hypermethylated
cg12446629	0.26919	0.398839459	0.567183	7.12E-05	hypermethylated
cg26406176	0.29595	0.438466486	0.567113	0.000421	hypermethylated
cg11420883	0.28228	0.418201081	0.56707	0.000274	hypermethylated
cg07540084	0.52075	0.771404865	0.566897	0.003316	hypermethylated
cg11839291	0.28093	0.415988649	0.566333	5.31E-05	hypermethylated
cg10888790	0.43311	0.641137297	0.5659	3.00E-05	hypermethylated
cg14051662	0.34497	0.510623243	0.565788	0.012334	hypermethylated
cg02881274	0.30516	0.451668649	0.565699	0.000207	hypermethylated
cg01338347	0.25863	0.382714595	0.565379	1.71E-05	hypermethylated
cg06798008	0.4702	0.695775676	0.565348	0.00064	hypermethylated
cg16467015	0.5519	0.816639459	0.565292	5.72E-05	hypermethylated
cg06687091	0.40718	0.602447568	0.565169	0.006915	hypermethylated
cg04980849	0.41782	0.618020541	0.564773	0.018485	hypermethylated
cg21117734	0.36804	0.544339459	0.564644	0.000293	hypermethylated
cg04704294	0.35528	0.525386486	0.564423	0.000214	hypermethylated
cg10055566	0.31201	0.461387027	0.564385	2.51E-05	hypermethylated
cg13989664	0.45632	0.674693514	0.564186	0.001768	hypermethylated
cg25050332	0.32046	0.473807568	0.564157	0.001452	hypermethylated
cg14229207	0.60224	0.890234595	0.563847	0.000176	hypermethylated
cg16962683	0.55604	0.821907568	0.563787	0.006679	hypermethylated

cg03876032	0.34368	0.50799027	0.563735	5.19E-05	hypermethylated
cg18881778	0.35003	0.517370811	0.56372	0.001237	hypermethylated
cg14592399	0.31283	0.462359459	0.563636	1.12E-05	hypermethylated
cg19426522	0.39454	0.582963243	0.563233	6.78E-05	hypermethylated
cg25479732	0.33693	0.497808108	0.563141	0.00097	hypermethylated
cg15554023	0.42804	0.632421081	0.56314	0.000204	hypermethylated
cg07179981	0.5684	0.839604324	0.562803	0.000674	hypermethylated
cg03457195	0.32963	0.486842162	0.562607	0.000274	hypermethylated
cg01541178	0.32918	0.486164324	0.562567	0.000135	hypermethylated
cg09151131	0.44252	0.653507027	0.56246	5.93E-05	hypermethylated
cg03308399	0.37187	0.549102162	0.562276	0.003078	hypermethylated
cg27649348	0.3052	0.450606486	0.562113	9.05E-05	hypermethylated
cg13412545	0.32556	0.480659459	0.562092	0.000408	hypermethylated
cg19240319	0.38698	0.571316757	0.562032	2.42E-06	hypermethylated
cg22872857	0.46211	0.682212432	0.561985	0.010211	hypermethylated
cg22682811	0.38502	0.568283243	0.561677	0.000474	hypermethylated
cg04236178	0.35024	0.516939459	0.561651	6.01E-05	hypermethylated
cg17478228	0.37272	0.550093514	0.561585	4.26E-05	hypermethylated
cg12187394	0.32086	0.473532973	0.561521	7.56E-05	hypermethylated
cg06331333	0.28424	0.41946973	0.561457	2.54E-05	hypermethylated
cg25168494	0.28867	0.425945405	0.561247	0.000148	hypermethylated
cg15915658	0.42251	0.623397297	0.561166	3.67E-05	hypermethylated
cg05835726	0.34532	0.509401622	0.56087	0.003986	hypermethylated
cg14156650	0.31881	0.470276216	0.560811	0.001667	hypermethylated
cg25536014	0.38319	0.565177297	0.560644	0.000588	hypermethylated
cg12924936	0.50045	0.738094595	0.56058	0.006451	hypermethylated
cg03556669	0.36935	0.544681081	0.560423	0.00101	hypermethylated
cg08719486	0.37205	0.54863027	0.560338	0.00095	hypermethylated
cg26471674	0.29601	0.436498919	0.560332	2.02E-05	hypermethylated
cg23904955	0.31602	0.465984324	0.560266	0.003255	hypermethylated
cg13912311	0.35252	0.519801081	0.560255	0.000129	hypermethylated
cg06455422	0.44701	0.659048649	0.560078	0.017101	hypermethylated
cg07922843	0.39621	0.58413027	0.560025	0.006796	hypermethylated
cg20826576	0.32047	0.472455676	0.55999	0.000581	hypermethylated
cg02057782	0.32397	0.477605405	0.559959	6.75E-06	hypermethylated
cg02503117	0.22766	0.335598378	0.559855	0.000306	hypermethylated
cg15310162	0.56944	0.839364865	0.559754	0.00095	hypermethylated
cg09014626	0.5198	0.76618973	0.559745	0.006915	hypermethylated
cg12766736	0.36917	0.544138378	0.559688	3.76E-05	hypermethylated
cg19037007	0.31012	0.456931351	0.559151	0.000358	hypermethylated
cg00015319	0.32487	0.478661081	0.559142	3.00E-05	hypermethylated
cg20762480	0.40148	0.591472432	0.558983	1.44E-05	hypermethylated
cg23488198	0.40064	0.590204324	0.558908	0.001096	hypermethylated
cg06928993	0.47302	0.696755676	0.558752	0.007472	hypermethylated
cg24739407	0.42541	0.626597297	0.558685	0.000772	hypermethylated
cg24185357	0.31779	0.468067568	0.558643	0.000696	hypermethylated
cg06132727	0.39341	0.57924973	0.558152	0.000145	hypermethylated
cg26802026	0.45018	0.662676216	0.557802	0.010042	hypermethylated
cg02244431	0.47634	0.701131892	0.557694	0.002409	hypermethylated
cg21896766	0.37395	0.550393514	0.557618	0.000408	hypermethylated
cg10764068	0.30155	0.44382	0.557577	2.38E-05	hypermethylated
cg19131667	0.43546	0.64089027	0.557537	0.000287	hypermethylated
cg25360180	0.26791	0.394257838	0.557391	8.42E-05	hypermethylated
cg25252197	0.4514	0.664147027	0.557096	0.00101	hypermethylated
cg11416492	0.4077	0.599744324	0.55684	0.001437	hypermethylated
cg10235817	0.26608	0.391397297	0.556774	0.000155	hypermethylated
cg04902474	0.1778	0.261432973	0.556186	0.02255	hypermethylated
cg04716021	0.29843	0.438774054	0.556086	0.001262	hypermethylated

cg08379637	0.24578	0.361311351	0.555875	9.27E-05	hypermethylated
cg13882284	0.32913	0.48375027	0.555605	1.04E-05	hypermethylated
cg10402698	0.40537	0.595803243	0.555597	0.001734	hypermethylated
cg10175249	0.52948	0.778191351	0.555549	0.007933	hypermethylated
cg26272623	0.30904	0.454197297	0.555526	2.85E-05	hypermethylated
cg15004136	0.32913	0.483707568	0.555478	0.000271	hypermethylated
cg01713272	0.25488	0.374477297	0.55506	0.000354	hypermethylated
cg15180789	0.33225	0.488110811	0.55494	8.13E-05	hypermethylated
cg02371631	0.55448	0.81452973	0.554832	7.47E-05	hypermethylated
cg12883980	0.34667	0.509238919	0.55478	1.18E-05	hypermethylated
cg07330481	0.54166	0.795658378	0.554762	2.64E-05	hypermethylated
cg27305357	0.2739	0.402288108	0.55458	3.00E-05	hypermethylated
cg12363472	0.38108	0.559678378	0.554504	1.84E-05	hypermethylated
cg05935800	0.34897	0.512488108	0.554416	0.020903	hypermethylated
cg08915632	0.31655	0.464832432	0.554277	0.000214	hypermethylated
cg00823843	0.35065	0.514850811	0.554123	0.00035	hypermethylated
cg04090392	0.26884	0.394681081	0.55394	8.13E-05	hypermethylated
cg26951440	0.30754	0.451466486	0.553845	9.27E-05	hypermethylated
cg00540067	0.44159	0.648247027	0.553836	0.000681	hypermethylated
cg06051411	0.54019	0.792971351	0.553802	0.000749	hypermethylated
cg14507146	0.43976	0.645460541	0.553613	0.000653	hypermethylated
cg26464586	0.36731	0.53906	0.553448	0.000575	hypermethylated
cg13393830	0.36984	0.542697297	0.553246	0.001803	hypermethylated
cg24112562	0.31628	0.464096757	0.553223	2.85E-05	hypermethylated
cg09469610	0.26395	0.387261081	0.553042	5.72E-05	hypermethylated
cg07917609	0.31168	0.45728	0.553012	0.00017	hypermethylated
cg13918042	0.47433	0.695854595	0.552895	0.008419	hypermethylated
cg17873048	0.41867	0.61412973	0.55273	0.007536	hypermethylated
cg07194839	0.33575	0.492452973	0.552599	0.000204	hypermethylated
cg21542881	0.35199	0.516230811	0.552482	0.002231	hypermethylated
cg14160480	0.4477	0.656593514	0.552468	0.001603	hypermethylated
cg16705273	0.35648	0.52261027	0.551914	0.005807	hypermethylated
cg08267888	0.42933	0.629352973	0.551782	1.54E-05	hypermethylated
cg24315815	0.35615	0.521938378	0.551394	0.012637	hypermethylated
cg12186618	0.31956	0.468229189	0.551128	0.001164	hypermethylated
cg07019315	0.3878	0.568148108	0.550954	0.000756	hypermethylated
cg01750780	0.28263	0.413995676	0.550701	0.000256	hypermethylated
cg16784745	0.58043	0.850113514	0.550533	8.22E-05	hypermethylated
cg14078335	0.50366	0.737643243	0.550473	0.005757	hypermethylated
cg18573327	0.36635	0.536528108	0.550431	7.84E-05	hypermethylated
cg24259244	0.26916	0.394135135	0.550226	2.39E-05	hypermethylated
cg00755130	0.45286	0.663062703	0.55008	7.65E-05	hypermethylated
cg24909309	0.5005	0.732775676	0.550002	0.000327	hypermethylated
cg02113641	0.27514	0.402822703	0.549979	9.27E-05	hypermethylated
cg17482089	0.41198	0.603031892	0.54966	6.93E-06	hypermethylated
cg26698460	0.47816	0.699897838	0.549651	0.001052	hypermethylated
cg03389890	0.35594	0.520936757	0.549474	0.002478	hypermethylated
cg11484576	0.42007	0.614739459	0.549345	0.004567	hypermethylated
cg09121543	0.31837	0.465896757	0.549306	0.000875	hypermethylated
cg18597643	0.27674	0.404945946	0.549198	2.78E-05	hypermethylated
cg03335246	0.41155	0.601983784	0.548657	8.42E-05	hypermethylated
cg18947175	0.35738	0.522725946	0.548596	0.000399	hypermethylated
cg05348776	0.52167	0.762943243	0.548438	0.003378	hypermethylated
cg10155522	0.55133	0.806302703	0.548405	0.000212	hypermethylated
cg09832244	0.30449	0.445296216	0.548371	0.00017	hypermethylated
cg15442037	0.40783	0.596345946	0.548182	5.51E-05	hypermethylated
cg26644049	0.34391	0.502866486	0.548144	2.13E-05	hypermethylated
cg09304047	0.42017	0.614302703	0.547977	0.002026	hypermethylated

cg00716257	0.42502	0.621338378	0.547848	0.009552	hypermethylated
cg23513966	0.30507	0.44597027	0.547807	2.78E-05	hypermethylated
cg07048527	0.41427	0.605516216	0.547594	0.008491	hypermethylated
cg16767916	0.3919	0.572727568	0.547363	1.35E-05	hypermethylated
cg11246938	0.4709	0.688141622	0.547285	0.002409	hypermethylated
cg04439252	0.24531	0.358434595	0.547104	0.00221	hypermethylated
cg26207380	0.38216	0.558355135	0.547006	0.005606	hypermethylated
cg11165752	0.36663	0.535582162	0.546783	1.94E-05	hypermethylated
cg07493858	0.35011	0.511339459	0.546473	8.94E-05	hypermethylated
cg04427469	0.23917	0.349294595	0.546408	6.31E-05	hypermethylated
cg26649904	0.51443	0.751244324	0.546307	0.002649	hypermethylated
cg19051015	0.30574	0.446474054	0.546271	0.004691	hypermethylated
cg26196385	0.35165	0.513491892	0.546201	0.000588	hypermethylated
cg19671120	0.26707	0.389888649	0.545844	0.000112	hypermethylated
cg02339752	0.3064	0.447304324	0.54584	5.19E-05	hypermethylated
cg14073497	0.22155	0.323395135	0.545666	0.007933	hypermethylated
cg09619624	0.4604	0.671938378	0.545441	0.000667	hypermethylated
cg24656271	0.32883	0.479899459	0.54539	3.85E-05	hypermethylated
cg24343823	0.28506	0.415968649	0.545209	0.000214	hypermethylated
cg07727170	0.38684	0.564476216	0.545176	0.000426	hypermethylated
cg26456223	0.4028	0.587733514	0.545098	4.60E-06	hypermethylated
cg08645860	0.3764	0.549086486	0.544767	2.71E-05	hypermethylated
cg25611369	0.55266	0.806095135	0.544558	0.002026	hypermethylated
cg18140268	0.39088	0.570082162	0.544444	0.000667	hypermethylated
cg10804656	0.40182	0.586009189	0.544374	0.000182	hypermethylated
cg25016420	0.52744	0.769174595	0.544304	0.001839	hypermethylated
cg14504259	0.32361	0.471906486	0.544245	7.84E-05	hypermethylated
cg20550790	0.32889	0.479563243	0.544116	0.000399	hypermethylated
cg07416237	0.29365	0.428148108	0.544012	8.52E-05	hypermethylated
cg12852499	0.29816	0.434722162	0.544007	4.58E-05	hypermethylated
cg06773488	0.29715	0.43323027	0.543943	0.000342	hypermethylated
cg13536060	0.46904	0.683783243	0.543828	0.000126	hypermethylated
cg21548955	0.45139	0.657947027	0.543597	0.000313	hypermethylated
cg01167502	0.3549	0.517283784	0.543543	0.000313	hypermethylated
cg17029237	0.45828	0.667915676	0.543437	0.005508	hypermethylated
cg06564388	0.31154	0.454018919	0.543335	2.51E-05	hypermethylated
cg26814075	0.35473	0.516955135	0.543318	0.00028	hypermethylated
cg18105979	0.28598	0.416726486	0.543187	0.000569	hypermethylated
cg25015733	0.50895	0.741591351	0.5431	0.000626	hypermethylated
cg12236088	0.35802	0.521610811	0.542934	0.000902	hypermethylated
cg17642941	0.3718	0.54162	0.542754	0.000229	hypermethylated
cg12935350	0.54565	0.794865405	0.542735	0.000756	hypermethylated
cg07010948	0.29065	0.423314595	0.542447	1.62E-05	hypermethylated
cg11491407	0.25812	0.375851351	0.54212	0.000912	hypermethylated
cg24244500	0.35518	0.517175676	0.542104	4.58E-05	hypermethylated
cg07807395	0.37908	0.551928108	0.541978	0.005411	hypermethylated
cg04684114	0.29719	0.432697838	0.541974	2.85E-05	hypermethylated
cg25461801	0.44253	0.644280541	0.541914	0.000152	hypermethylated
cg06942701	0.31151	0.453503243	0.541834	1.75E-05	hypermethylated
cg21814870	0.46773	0.680723243	0.541392	0.000474	hypermethylated
cg20664313	0.36228	0.527212973	0.541281	0.001734	hypermethylated
cg20699586	0.43622	0.634743784	0.541118	0.002318	hypermethylated
cg20467957	0.32464	0.47236973	0.541076	3.81E-05	hypermethylated
cg14494620	0.39645	0.576728108	0.540752	0.000274	hypermethylated
cg15996534	0.3591	0.52228	0.540438	0.000195	hypermethylated
cg23906060	0.26887	0.391028108	0.540364	0.000174	hypermethylated
cg02658730	0.37014	0.538245405	0.540193	9.82E-06	hypermethylated
cg13808012	0.3505	0.509633514	0.540046	0.0003	hypermethylated

cg22894301	0.33947	0.493537297	0.539875	8.03E-05	hypermethylated
cg24524057	0.35674	0.518612973	0.539785	9.95E-05	hypermethylated
cg01603921	0.3376	0.490730811	0.539617	0.000306	hypermethylated
cg14716686	0.47139	0.685189189	0.539581	1.73E-05	hypermethylated
cg20148850	0.26383	0.383467568	0.539496	3.07E-05	hypermethylated
cg19526568	0.29015	0.421661622	0.539287	2.13E-05	hypermethylated
cg18470456	0.49115	0.713755676	0.539267	0.007799	hypermethylated
cg17162319	0.3574	0.519376216	0.53924	0.000569	hypermethylated
cg23675401	0.32864	0.477562162	0.53918	8.84E-05	hypermethylated
cg02441618	0.28022	0.407138378	0.538959	9.27E-05	hypermethylated
cg03729337	0.34364	0.499251351	0.538868	5.25E-05	hypermethylated
cg19392831	0.31747	0.46118973	0.53874	1.30E-05	hypermethylated
cg00055679	0.26757	0.38866	0.538592	9.27E-05	hypermethylated
cg18985828	0.28411	0.412667027	0.538529	2.32E-05	hypermethylated
cg04653800	0.51228	0.744069189	0.538504	0.002455	hypermethylated
cg15496335	0.30997	0.450203784	0.53845	0.00031	hypermethylated
cg18734433	0.33525	0.486814595	0.538135	0.001857	hypermethylated
cg09031823	0.4611	0.669556216	0.538126	0.002231	hypermethylated
cg16738453	0.5347	0.776380541	0.538034	0.000454	hypermethylated
cg06868946	0.27346	0.397047027	0.53798	0.000166	hypermethylated
cg04729913	0.31739	0.460765405	0.537776	1.25E-05	hypermethylated
cg15644756	0.5412	0.785629189	0.537687	0.002363	hypermethylated
cg12152384	0.42814	0.621491351	0.537652	0.02324	hypermethylated
cg00875989	0.30648	0.444882162	0.53763	8.84E-05	hypermethylated
cg14684434	0.4127	0.599027568	0.537529	0.000931	hypermethylated
cg05166820	0.36873	0.535108108	0.537266	6.95E-05	hypermethylated
cg07263322	0.42332	0.614322162	0.537247	0.000445	hypermethylated
cg26189021	0.28454	0.412855135	0.537004	0.004691	hypermethylated
cg10292376	0.28601	0.414986486	0.536999	2.04E-05	hypermethylated
cg01730258	0.41488	0.601962162	0.536979	0.006679	hypermethylated
cg00388812	0.35125	0.509591351	0.536843	1.26E-05	hypermethylated
cg10130374	0.62202	0.902389189	0.536789	4.00E-05	hypermethylated
cg03753391	0.29111	0.422317297	0.536763	8.22E-05	hypermethylated
cg04956511	0.2119	0.307384865	0.536663	0.006564	hypermethylated
cg25391117	0.42624	0.618226486	0.536469	0.000822	hypermethylated
cg26967100	0.35194	0.510414595	0.53634	0.000107	hypermethylated
cg11429111	0.40911	0.593240541	0.536128	0.002965	hypermethylated
cg04918696	0.43057	0.624284865	0.535957	0.001987	hypermethylated
cg13943333	0.43194	0.626225405	0.535851	0.000327	hypermethylated
cg11118422	0.34884	0.505740541	0.535832	0.000417	hypermethylated
cg18394340	0.29922	0.433785946	0.535777	0.000232	hypermethylated
cg11234328	0.25846	0.374694054	0.535772	0.000142	hypermethylated
cg27035480	0.47386	0.68692973	0.535702	0.000382	hypermethylated
cg00349061	0.43825	0.635282703	0.535645	8.63E-05	hypermethylated
cg03345448	0.34834	0.504915135	0.535545	7.72E-06	hypermethylated
cg21407117	0.31321	0.453988649	0.535526	0.007933	hypermethylated
cg03053290	0.45964	0.666151892	0.535347	0.003316	hypermethylated
cg17284553	0.24669	0.357518919	0.53532	0.000229	hypermethylated
cg03534031	0.41401	0.599895676	0.535046	5.19E-05	hypermethylated
cg10741478	0.50017	0.724702162	0.53497	0.010211	hypermethylated
cg15561613	0.40213	0.582642162	0.534948	0.000335	hypermethylated
cg16227623	0.39896	0.577992432	0.534806	1.39E-05	hypermethylated
cg21174375	0.5454	0.790128649	0.534773	0.000469	hypermethylated
cg14012310	0.32106	0.464927027	0.534161	3.76E-05	hypermethylated
cg12492087	0.45286	0.655765946	0.534116	0.000132	hypermethylated
cg04353438	0.52554	0.760941622	0.533985	0.002526	hypermethylated
cg18632634	0.49892	0.722380541	0.533951	0.000495	hypermethylated
cg00041989	0.40368	0.584397838	0.533739	0.000226	hypermethylated

cg03888033	0.36492	0.528276757	0.533714	6.31E-05	hypermethylated
cg05364567	0.30404	0.440142703	0.53371	0.000386	hypermethylated
cg03415617	0.49241	0.712567568	0.533167	0.001717	hypermethylated
cg13791971	0.50715	0.733883784	0.533139	0.001875	hypermethylated
cg03984866	0.44673	0.646351892	0.532917	0.007667	hypermethylated
cg15446391	0.43491	0.62913027	0.532642	1.50E-05	hypermethylated
cg18067859	0.42429	0.613748649	0.532597	1.80E-05	hypermethylated
cg14628260	0.37396	0.540926486	0.532549	2.85E-05	hypermethylated
cg23457588	0.30076	0.435012432	0.532444	3.57E-05	hypermethylated
cg04766061	0.21015	0.303921081	0.532277	0.006796	hypermethylated
cg26478297	0.36825	0.532533514	0.532187	0.008138	hypermethylated
cg05656364	0.20123	0.290980541	0.532077	0.006451	hypermethylated
cg07676709	0.37467	0.541697297	0.531866	0.002502	hypermethylated
cg24490133	0.54537	0.788261622	0.531439	0.000262	hypermethylated
cg25597138	0.25792	0.372781081	0.531405	8.63E-05	hypermethylated
cg04419610	0.29564	0.427223784	0.53115	0.005557	hypermethylated
cg00589850	0.36915	0.533353514	0.530885	0.000104	hypermethylated
cg06727067	0.47008	0.679107027	0.530733	0.001839	hypermethylated
cg12351768	0.51226	0.739995135	0.53064	0.000342	hypermethylated
cg03742724	0.4545	0.656499459	0.530514	0.002856	hypermethylated
cg25456849	0.33471	0.483377297	0.530238	1.18E-05	hypermethylated
cg27631389	0.52965	0.764899459	0.530231	0.000575	hypermethylated
cg10098523	0.32568	0.470263243	0.530013	0.007601	hypermethylated
cg04195774	0.42954	0.620202162	0.529946	0.000789	hypermethylated
cg17470674	0.4032	0.582158919	0.529917	0.007219	hypermethylated
cg10398682	0.30711	0.443409189	0.529883	0.001164	hypermethylated
cg11908057	0.41722	0.602343243	0.529778	0.003255	hypermethylated
cg05803237	0.31208	0.45052973	0.529706	3.32E-05	hypermethylated
cg12079548	0.31438	0.45376973	0.529451	4.31E-05	hypermethylated
cg08111158	0.34382	0.496182162	0.529216	0.0006	hypermethylated
cg21074347	0.41906	0.604754595	0.529193	0.000814	hypermethylated
cg00562553	0.42274	0.609953514	0.528929	0.00134	hypermethylated
cg21743182	0.46676	0.673419459	0.528824	0.00291	hypermethylated
cg23378722	0.34414	0.496252432	0.528079	0.007866	hypermethylated
cg03987660	0.35859	0.517072432	0.528031	0.000214	hypermethylated
cg02662828	0.36844	0.53126973	0.528015	0.000112	hypermethylated
cg14789818	0.43598	0.628627568	0.527944	0.002699	hypermethylated
cg04004590	0.34874	0.502788108	0.527799	0.000287	hypermethylated
cg14323204	0.37773	0.544518919	0.527627	8.84E-05	hypermethylated
cg14780466	0.33078	0.476831892	0.527609	0.000831	hypermethylated
cg19633004	0.49402	0.712072432	0.527455	0.01109	hypermethylated
cg17107017	0.5712	0.823147027	0.527154	0.00012	hypermethylated
cg00855990	0.32024	0.461427568	0.526951	5.86E-05	hypermethylated
cg02852421	0.53885	0.776342703	0.52681	0.003986	hypermethylated
cg16890121	0.60028	0.864831892	0.526784	0.000626	hypermethylated
cg10936057	0.57287	0.825285946	0.526686	0.010381	hypermethylated
cg19352726	0.55015	0.792446486	0.526488	0.000168	hypermethylated
cg00830492	0.46023	0.662861081	0.526352	0.004863	hypermethylated
cg15916646	0.29392	0.423304865	0.526274	0.000102	hypermethylated
cg16982087	0.57511	0.828235135	0.526202	0.001541	hypermethylated
cg14753432	0.28479	0.410035676	0.525851	3.23E-05	hypermethylated
cg05343808	0.49514	0.712836757	0.525735	0.002168	hypermethylated
cg07884222	0.47148	0.678752973	0.52569	4.10E-05	hypermethylated
cg14862806	0.26618	0.383167568	0.525573	9.60E-05	hypermethylated
cg08836199	0.49869	0.717724324	0.525287	0.012234	hypermethylated
cg13293535	0.45101	0.649077838	0.525232	0.002126	hypermethylated
cg11724970	0.34744	0.499875676	0.524805	0.004734	hypermethylated
cg27391357	0.55081	0.792392973	0.524661	0.001354	hypermethylated

cg03585823	0.53704	0.772571892	0.52464	0.006915	hypermethylated
cg03985452	0.40587	0.583808649	0.524478	0.002751	hypermethylated
cg17241310	0.43685	0.628245946	0.524191	1.46E-05	hypermethylated
cg21419585	0.58366	0.839368649	0.524176	0.000202	hypermethylated
cg12404831	0.37586	0.540475676	0.524034	0.000594	hypermethylated
cg19979853	0.5371	0.772328108	0.524023	0.000626	hypermethylated
cg22333259	0.31493	0.452816757	0.523896	0.002699	hypermethylated
cg07179872	0.36341	0.522495135	0.523819	4.94E-05	hypermethylated
cg21969566	0.49106	0.705856757	0.523476	5.50E-06	hypermethylated
cg13229857	0.33527	0.481840541	0.523232	0.006507	hypermethylated
cg10811426	0.3638	0.522804324	0.523126	0.009959	hypermethylated
cg00237475	0.26918	0.386776216	0.522928	5.31E-05	hypermethylated
cg17546247	0.38137	0.547807027	0.522476	5.19E-05	hypermethylated
cg15196806	0.46971	0.674633514	0.522334	0.001635	hypermethylated
cg22562383	0.56063	0.805066486	0.522059	0.00305	hypermethylated
cg01966891	0.40763	0.585322162	0.521971	0.00032	hypermethylated
cg08602008	0.37866	0.543701081	0.521911	5.12E-05	hypermethylated
cg00964997	0.33786	0.485109189	0.521884	3.23E-05	hypermethylated
cg08110693	0.47992	0.689017297	0.521746	0.038628	hypermethylated
cg24125828	0.57916	0.831424865	0.521624	2.29E-06	hypermethylated
cg26570233	0.5482	0.786973514	0.521613	0.013589	hypermethylated
cg01493727	0.33684	0.483514054	0.521494	1.01E-05	hypermethylated
cg01468220	0.41228	0.591778919	0.521434	8.32E-05	hypermethylated
cg17786697	0.36233	0.519998919	0.521204	2.21E-05	hypermethylated
cg11754318	0.31528	0.452342162	0.520781	1.18E-05	hypermethylated
cg06013632	0.37227	0.534104865	0.520774	3.40E-05	hypermethylated
cg09011231	0.38643	0.554352432	0.520596	0.001667	hypermethylated
cg07943461	0.35835	0.51405027	0.52054	0.000324	hypermethylated
cg01596674	0.39595	0.567878919	0.520265	0.001751	hypermethylated
cg03731131	0.37454	0.537003784	0.519812	0.000517	hypermethylated
cg15318546	0.47032	0.674202162	0.519539	0.00291	hypermethylated
cg26055747	0.46511	0.666727568	0.519525	0.011182	hypermethylated
cg27160395	0.40777	0.584508649	0.519469	0.000696	hypermethylated
cg00188627	0.39121	0.560682162	0.51924	0.000187	hypermethylated
cg19266635	0.57771	0.827859459	0.51904	0.008208	hypermethylated
cg09411999	0.3259	0.466976216	0.51892	0.000166	hypermethylated
cg11078084	0.44303	0.634805405	0.51891	0.000182	hypermethylated
cg21931419	0.45462	0.651210811	0.518463	3.57E-05	hypermethylated
cg22272713	0.40436	0.578948649	0.517795	0.007282	hypermethylated
cg12689205	0.38927	0.557340541	0.517788	0.00031	hypermethylated
cg06816651	0.28884	0.413516216	0.517673	0.000335	hypermethylated
cg26880961	0.22903	0.327860541	0.517546	0.003914	hypermethylated
cg14759565	0.55432	0.793462703	0.517443	6.46E-05	hypermethylated
cg07857469	0.34741	0.497257297	0.517353	7.84E-05	hypermethylated
cg11411203	0.30881	0.44198973	0.517293	1.54E-05	hypermethylated
cg07557903	0.51138	0.731901081	0.517253	3.23E-05	hypermethylated
cg00525277	0.42878	0.613574054	0.517	0.004649	hypermethylated
cg01323104	0.39967	0.571887568	0.516922	0.001118	hypermethylated
cg26390660	0.41462	0.593261081	0.516877	0.002829	hypermethylated
cg17766651	0.54319	0.777094595	0.516633	0.000839	hypermethylated
cg17353900	0.2516	0.359927027	0.516573	0.000875	hypermethylated
cg26514430	0.40799	0.583538378	0.516294	2.74E-05	hypermethylated
cg01306410	0.27573	0.394316757	0.516099	5.06E-05	hypermethylated
cg20798152	0.27852	0.398302162	0.516082	9.31E-06	hypermethylated
cg09075558	0.32066	0.458559459	0.516064	0.0006	hypermethylated
cg00848392	0.29915	0.427772432	0.515974	0.000528	hypermethylated
cg05984317	0.35684	0.510212973	0.515822	5.38E-05	hypermethylated
cg08701183	0.3701	0.529132432	0.515714	0.000115	hypermethylated

cg10211414	0.4222	0.603550811	0.515549	0.001667	hypermethylated
cg10366797	0.42399	0.605774054	0.51475	3.85E-06	hypermethylated
cg12448933	0.27958	0.399425946	0.514667	5.38E-05	hypermethylated
cg26885488	0.3473	0.496154054	0.514606	1.99E-05	hypermethylated
cg26047066	0.56418	0.805913514	0.51447	0.003914	hypermethylated
cg17404981	0.29182	0.416822703	0.514355	0.009473	hypermethylated
cg04272613	0.35328	0.504578919	0.514268	0.004951	hypermethylated
cg17233506	0.39409	0.562808649	0.514119	0.007282	hypermethylated
cg11334165	0.34503	0.492671351	0.513904	0.000893	hypermethylated
cg19267457	0.4112	0.587123784	0.513824	0.008491	hypermethylated
cg03048432	0.45038	0.643020541	0.513722	0.000209	hypermethylated
cg11323255	0.36403	0.519720541	0.513679	0.0017	hypermethylated
cg03995968	0.53029	0.756875676	0.513275	0.00255	hypermethylated
cg14238120	0.47729	0.681058919	0.512914	0.000327	hypermethylated
cg24678611	0.30956	0.441717297	0.512904	0.00012	hypermethylated
cg05426006	0.56083	0.800097838	0.512613	0.005459	hypermethylated
cg02357541	0.46934	0.669485946	0.51242	0.018628	hypermethylated
cg03854796	0.29985	0.427688649	0.51232	2.39E-05	hypermethylated
cg10655046	0.26632	0.379852973	0.51228	4.67E-06	hypermethylated
cg13777798	0.58807	0.838710811	0.512186	0.00151	hypermethylated
cg03829739	0.38378	0.547264324	0.511958	0.001452	hypermethylated
cg01572696	0.33483	0.477328108	0.511552	5.72E-05	hypermethylated
cg15844419	0.45916	0.654524865	0.511451	0.001395	hypermethylated
cg17036007	0.29649	0.422488649	0.510929	2.78E-05	hypermethylated
cg01568492	0.4499	0.641061622	0.510859	1.21E-05	hypermethylated
cg16664193	0.37459	0.533735135	0.510812	0.000148	hypermethylated
cg15022308	0.26709	0.380499459	0.510568	0.001031	hypermethylated
cg06664357	0.3681	0.524331351	0.510381	0.000581	hypermethylated
cg00040588	0.26725	0.380631351	0.510204	0.000454	hypermethylated
cg00260802	0.29517	0.420338378	0.510005	9.96E-06	hypermethylated
cg05934012	0.52717	0.750588108	0.509753	6.78E-05	hypermethylated
cg26772847	0.47482	0.675948108	0.509532	0.000557	hypermethylated
cg06727242	0.48769	0.694193514	0.509373	6.75E-06	hypermethylated
cg01121554	0.37147	0.528762162	0.509373	0.000126	hypermethylated
cg02568761	0.33515	0.476988649	0.509148	0.000271	hypermethylated
cg12689506	0.37035	0.527082162	0.509139	0.000195	hypermethylated
cg17741986	0.26743	0.3806	0.509114	9.72E-05	hypermethylated
cg22852830	0.35764	0.508956757	0.509035	0.000557	hypermethylated
cg17961200	0.55996	0.796872432	0.509025	0.002147	hypermethylated
cg09343092	0.44608	0.634793514	0.508985	0.001734	hypermethylated
cg15070710	0.44567	0.634208649	0.508982	0.018485	hypermethylated
cg23712458	0.33267	0.473402703	0.508976	0.003316	hypermethylated
cg03512076	0.37966	0.540225405	0.508853	0.000256	hypermethylated
cg12592691	0.3576	0.508731892	0.508559	0.002066	hypermethylated
cg04500377	0.3534	0.502739459	0.508509	0.006507	hypermethylated
cg20166027	0.4346	0.618124324	0.508209	0.003986	hypermethylated
cg10142436	0.40731	0.579244324	0.508045	0.000412	hypermethylated
cg20128928	0.34958	0.49710973	0.507942	0.000287	hypermethylated
cg14755690	0.55993	0.796178378	0.507845	0.004365	hypermethylated
cg19839825	0.43948	0.624845405	0.507702	0.006737	hypermethylated
cg08172947	0.48786	0.693355676	0.507128	0.02638	hypermethylated
cg02596940	0.31843	0.452557297	0.507124	0.000426	hypermethylated
cg01795955	0.35848	0.509461081	0.507079	0.004365	hypermethylated
cg24202131	0.38925	0.553033514	0.50667	0.000198	hypermethylated
cg26791307	0.43009	0.611047027	0.506645	0.006507	hypermethylated
cg18731024	0.5844	0.830197838	0.506499	0.003773	hypermethylated
cg03229561	0.38623	0.548672432	0.506485	7.47E-05	hypermethylated
cg01171212	0.25284	0.359116757	0.506228	0.000119	hypermethylated

cg21660130	0.49377	0.7013	0.506193	0.007408	hypermethylated
cg21236845	0.53038	0.753250811	0.506104	0.012637	hypermethylated
cg22287148	0.56975	0.809067027	0.50593	0.005411	hypermethylated
cg12600530	0.60247	0.855516216	0.505906	0.003225	hypermethylated
cg24702360	0.38045	0.540213514	0.505823	0.000132	hypermethylated
cg12389786	0.40782	0.579065405	0.505794	2.15E-05	hypermethylated
cg24953321	0.29394	0.417357838	0.505763	1.18E-05	hypermethylated
cg13782301	0.39007	0.55381027	0.505659	1.82E-05	hypermethylated
cg10469980	0.39621	0.562513514	0.505622	0.006737	hypermethylated
cg05141333	0.60589	0.860164324	0.505556	6.78E-05	hypermethylated
cg07053697	0.3951	0.560687568	0.504979	0.000674	hypermethylated
cg27516568	0.43922	0.623287027	0.504953	0.000575	hypermethylated
cg22154659	0.51707	0.733681081	0.504793	0.00028	hypermethylated
cg07428004	0.37042	0.52559027	0.504777	0.000464	hypermethylated
cg00473624	0.64782	0.919167568	0.504735	0.000296	hypermethylated
cg05109569	0.38308	0.543534054	0.504725	0.005411	hypermethylated
cg16607685	0.41872	0.594092432	0.504702	3.76E-05	hypermethylated
cg11728738	0.44422	0.630260541	0.504674	0.008208	hypermethylated
cg13707793	0.60765	0.862115676	0.504641	0.000216	hypermethylated
cg20492034	0.49582	0.703394054	0.504517	0.027371	hypermethylated
cg10201616	0.60124	0.852927568	0.504482	0.002502	hypermethylated
cg01386185	0.4957	0.703057838	0.504176	0.010468	hypermethylated
cg12594635	0.3824	0.542338378	0.504111	0.006915	hypermethylated
cg00895997	0.42974	0.60942973	0.503996	0.003843	hypermethylated
cg06593118	0.51176	0.725704324	0.503914	0.006339	hypermethylated
cg02363202	0.42954	0.608947027	0.503524	0.004863	hypermethylated
cg09326087	0.57141	0.809948108	0.503303	6.38E-05	hypermethylated
cg23679260	0.26463	0.375101081	0.503303	0.000195	hypermethylated
cg00474798	0.47587	0.674441622	0.503126	0.00193	hypermethylated
cg05886272	0.2799	0.396654595	0.502972	0.003705	hypermethylated
cg09257635	0.51707	0.732664865	0.502794	0.000399	hypermethylated
cg14384093	0.49272	0.698084865	0.502634	0.000485	hypermethylated
cg16501028	0.38127	0.540144865	0.502533	3.67E-05	hypermethylated
cg24101039	0.52109	0.738191892	0.502463	0.014838	hypermethylated
cg08395122	0.32556	0.461125946	0.502237	3.79E-06	hypermethylated
cg11315991	0.49101	0.695403784	0.502099	0.003021	hypermethylated
cg04997045	0.52318	0.74096	0.502088	0.001635	hypermethylated
cg06282247	0.35344	0.500537297	0.502012	5.45E-05	hypermethylated
cg00590029	0.34403	0.48714	0.501802	3.07E-05	hypermethylated
cg06614044	0.45763	0.647831351	0.501437	0.000207	hypermethylated
cg23556923	0.2761	0.390845405	0.501407	0.000733	hypermethylated
cg05114861	0.44025	0.623014054	0.500942	0.001031	hypermethylated
cg26808747	0.39351	0.556815676	0.5008	7.72E-06	hypermethylated
cg15087376	0.49369	0.698511351	0.500678	0.027776	hypermethylated
cg22375610	0.33716	0.47703027	0.500647	0.006229	hypermethylated
cg14610335	0.42316	0.598565405	0.500306	0.000399	hypermethylated
cg24964368	0.46272	0.654507568	0.50027	5.19E-05	hypermethylated
cg05900785	0.2888	0.408478919	0.500191	1.18E-05	hypermethylated
cg02206157	0.28597	0.404467027	0.500158	0.000132	hypermethylated
cg22805485	0.54993	0.777789189	0.500131	0.000588	hypermethylated
cg04093645	0.5596	0.791452973	0.500108	0.002432	hypermethylated
cg00370815	0.25731	0.363901081	0.500039	0.000358	hypermethylated
cg01911239	0.33677	0.476215676	0.499851	3.23E-05	hypermethylated
cg01165683	0.47005	0.664498378	0.499451	0.03076	hypermethylated
cg15817769	0.53257	0.752836216	0.499365	0.001262	hypermethylated
cg16923137	0.60161	0.850423784	0.499353	0.000165	hypermethylated
cg15842276	0.30658	0.433318378	0.499164	1.42E-05	hypermethylated
cg05767720	0.45889	0.648462162	0.498874	0.000187	hypermethylated

cg25643223	0.47802	0.675464865	0.49881	0.000741	hypermethylated
cg11166453	0.47101	0.66553027	0.498747	0.000931	hypermethylated
cg02323356	0.33649	0.475447568	0.498723	2.32E-05	hypermethylated
cg02215430	0.51783	0.731591892	0.498561	0.005316	hypermethylated
cg27054655	0.40613	0.573749189	0.498479	3.81E-05	hypermethylated
cg11681428	0.30158	0.426036757	0.498437	0.00071	hypermethylated
cg27038634	0.32364	0.457187027	0.498395	0.00048	hypermethylated
cg06807928	0.50404	0.711796757	0.497927	0.01073	hypermethylated
cg19846491	0.37415	0.528114595	0.497234	0.01274	hypermethylated
cg01819512	0.34051	0.480616216	0.497188	8.63E-05	hypermethylated
cg02726930	0.31517	0.444822703	0.4971	0.001875	hypermethylated
cg01146063	0.34374	0.485121081	0.497027	0.000152	hypermethylated
cg24617504	0.28958	0.408658919	0.496935	0.000126	hypermethylated
cg21871232	0.46889	0.661634054	0.496784	0.008069	hypermethylated
cg17953764	0.42378	0.597913514	0.496621	8.03E-05	hypermethylated
cg22494346	0.51992	0.733552432	0.49661	0.030983	hypermethylated
cg24017995	0.45553	0.642643784	0.496473	0.01109	hypermethylated
cg23092421	0.56547	0.797731351	0.496452	0.000135	hypermethylated
cg02268156	0.45622	0.643598919	0.496432	2.51E-05	hypermethylated
cg23691946	0.35049	0.494391351	0.49628	1.58E-05	hypermethylated
cg09574499	0.51181	0.721932432	0.496255	0.005223	hypermethylated
cg23527621	0.29993	0.422915135	0.495742	0.000474	hypermethylated
cg05638493	0.34725	0.489617838	0.495681	9.27E-05	hypermethylated
cg27173965	0.50158	0.707220541	0.49568	0.000327	hypermethylated
cg16544169	0.28642	0.403663243	0.49502	4.94E-05	hypermethylated
cg26612727	0.37719	0.531584865	0.495009	7.03E-05	hypermethylated
cg03828328	0.32751	0.461532432	0.494893	0.001301	hypermethylated
cg11795276	0.40998	0.577747027	0.494884	6.46E-05	hypermethylated
cg25152404	0.3614	0.509243243	0.494758	0.007282	hypermethylated
cg23570694	0.32456	0.45730973	0.494686	3.96E-06	hypermethylated
cg10295552	0.43522	0.613201622	0.494617	0.001212	hypermethylated
cg00962755	0.49145	0.692395135	0.494551	2.05E-06	hypermethylated
cg07631435	0.41487	0.584473514	0.494478	0.000358	hypermethylated
cg04223553	0.43093	0.607083243	0.494441	9.27E-05	hypermethylated
cg03679544	0.54866	0.772921622	0.49441	2.10E-05	hypermethylated
cg02337614	0.46884	0.660318378	0.494066	0.004951	hypermethylated
cg06990571	0.56258	0.792296757	0.493983	0.002725	hypermethylated
cg23666945	0.51619	0.726854595	0.493765	0.000772	hypermethylated
cg04735481	0.3399	0.478602703	0.493718	0.000839	hypermethylated
cg09477232	0.33731	0.474941081	0.493673	0.001734	hypermethylated
cg26345916	0.40063	0.564049189	0.493551	0.002026	hypermethylated
cg11237948	0.30342	0.427183243	0.493539	0.000112	hypermethylated
cg03827835	0.48657	0.684995676	0.493448	0.000163	hypermethylated
cg23596826	0.56208	0.791283784	0.49342	0.020586	hypermethylated
cg27269917	0.50481	0.710579459	0.493255	0.000884	hypermethylated
cg07944936	0.46705	0.657361622	0.49311	0.003195	hypermethylated
cg03276401	0.40846	0.574824865	0.492928	0.008491	hypermethylated
cg21493505	0.28993	0.407959459	0.492721	3.90E-05	hypermethylated
cg11457640	0.36671	0.515967027	0.492639	0.000408	hypermethylated
cg15865511	0.55986	0.787688108	0.492558	0.003878	hypermethylated
cg02122937	0.5064	0.712416757	0.492444	0.000226	hypermethylated
cg06761530	0.60688	0.853350811	0.491728	0.005411	hypermethylated
cg08352755	0.39063	0.549168108	0.491445	0.000187	hypermethylated
cg20014988	0.4298	0.604164324	0.491276	0.003225	hypermethylated
cg10024478	0.3813	0.535947568	0.491165	0.006451	hypermethylated
cg06570224	0.37207	0.522972973	0.491162	1.18E-05	hypermethylated
cg00600617	0.42248	0.593761622	0.491001	0.028602	hypermethylated
cg01134260	0.47867	0.67267027	0.490868	0.005808	hypermethylated

cg06888121	0.46682	0.656006486	0.490844	0.002066	hypermethylated
cg03979754	0.40642	0.571015676	0.490559	0.000335	hypermethylated
cg07429284	0.32426	0.45551027	0.490333	0.002363	hypermethylated
cg19360104	0.36772	0.51655027	0.490301	6.01E-05	hypermethylated
cg00702719	0.38046	0.534405405	0.49019	5.25E-05	hypermethylated
cg04813119	0.50013	0.702475135	0.490144	0.000152	hypermethylated
cg04051396	0.62744	0.881086486	0.489806	0.003255	hypermethylated
cg12758973	0.40559	0.569512432	0.489705	0.00054	hypermethylated
cg06850597	0.28454	0.399536757	0.489697	6.31E-05	hypermethylated
cg26381514	0.51704	0.725998919	0.489692	0.00097	hypermethylated
cg23266594	0.44842	0.629591351	0.489565	6.95E-05	hypermethylated
cg16311302	0.27319	0.383524865	0.489415	0.001912	hypermethylated
cg24652842	0.3272	0.459284324	0.489215	0.000163	hypermethylated
cg24288527	0.65754	0.922942703	0.489162	4.70E-05	hypermethylated
cg07790279	0.46229	0.648881622	0.489157	0.000805	hypermethylated
cg01568919	0.2814	0.394972432	0.48913	9.49E-05	hypermethylated
cg00548268	0.35791	0.502354595	0.489109	5.73E-06	hypermethylated
cg10055580	0.43902	0.616139459	0.48897	0.010731	hypermethylated
cg14371590	0.35247	0.494667027	0.488957	0.000182	hypermethylated
cg05262549	0.40527	0.568690811	0.488761	0.000296	hypermethylated
cg14520423	0.45225	0.634478919	0.488452	0.003441	hypermethylated
cg08980382	0.34838	0.488727027	0.488367	0.000229	hypermethylated
cg09619390	0.2964	0.4158	0.488344	9.27E-05	hypermethylated
cg15683166	0.59702	0.83737027	0.488086	0.001437	hypermethylated
cg26643754	0.30227	0.423903784	0.487899	0.000122	hypermethylated
cg18394552	0.46629	0.653596216	0.487172	0.005364	hypermethylated
cg11738976	0.31371	0.439712973	0.487131	2.32E-05	hypermethylated
cg16857771	0.39413	0.552396216	0.487032	0.003378	hypermethylated
cg21253742	0.48525	0.680043243	0.486898	0.000667	hypermethylated
cg26581886	0.38063	0.533352432	0.4867	4.64E-05	hypermethylated
cg14037728	0.41647	0.583530811	0.486596	0.00032	hypermethylated
cg27295850	0.28655	0.401481081	0.486545	0.001085	hypermethylated
cg11679455	0.58088	0.813797297	0.486429	0.003136	hypermethylated
cg25800638	0.48415	0.678276216	0.486419	0.001074	hypermethylated
cg10120856	0.42269	0.592157297	0.48638	0.006339	hypermethylated
cg07758738	0.37816	0.529705946	0.486195	2.54E-05	hypermethylated
cg09073799	0.37341	0.523042162	0.486167	0.001496	hypermethylated
cg06872138	0.47946	0.671580541	0.48615	0.027776	hypermethylated
cg15772157	0.41874	0.586414054	0.485865	2.32E-05	hypermethylated
cg15325373	0.43345	0.606981081	0.485786	0.000506	hypermethylated
cg22560214	0.36876	0.516389189	0.485777	2.27E-05	hypermethylated
cg14037652	0.62681	0.877452973	0.485294	0.000287	hypermethylated
cg02352240	0.35824	0.501452432	0.485186	6.05E-06	hypermethylated
cg01217984	0.35686	0.499512432	0.485162	0.001368	hypermethylated
cg21279601	0.4012	0.561549189	0.485091	0.000256	hypermethylated
cg14270725	0.30075	0.420948649	0.48508	0.000551	hypermethylated
cg18484189	0.39423	0.551775135	0.485043	0.003316	hypermethylated
cg27316626	0.46675	0.65319027	0.484853	9.49E-05	hypermethylated
cg04646186	0.44031	0.616182162	0.484837	0.001212	hypermethylated
cg27552378	0.61885	0.865898919	0.484609	3.32E-05	hypermethylated
cg14074305	0.53375	0.746804324	0.484566	0.003705	hypermethylated
cg16354688	0.48741	0.681882703	0.484388	0.006068	hypermethylated
cg23932873	0.48159	0.673694054	0.484288	0.019361	hypermethylated
cg15960924	0.53875	0.75358973	0.484163	0.000365	hypermethylated
cg05446471	0.52985	0.741088649	0.484062	0.003705	hypermethylated
cg21683069	0.45263	0.633022162	0.483924	0.003136	hypermethylated
cg18436984	0.34947	0.488662162	0.483669	9.16E-05	hypermethylated
cg04629595	0.35256	0.492968649	0.483627	0.000219	hypermethylated

cg02246609	0.36932	0.516311351	0.48337	8.22E-05	hypermethylated
cg09263516	0.36002	0.503298378	0.483337	6.70E-05	hypermethylated
cg24794052	0.46049	0.643684324	0.483184	0.009877	hypermethylated
cg11142514	0.50073	0.699882162	0.483079	0.037297	hypermethylated
cg09363891	0.52559	0.734466486	0.482759	0.000506	hypermethylated
cg12232463	0.53052	0.741315676	0.482681	7.65E-05	hypermethylated
cg14047339	0.23106	0.322845946	0.482578	0.000193	hypermethylated
cg04075738	0.3951	0.552011892	0.482482	0.000681	hypermethylated
cg13829680	0.53781	0.751337838	0.482365	0.016061	hypermethylated
cg00851532	0.63926	0.892874054	0.482054	0.014603	hypermethylated
cg03400785	0.3835	0.535559459	0.48182	9.72E-05	hypermethylated
cg24767131	0.37345	0.521507027	0.481772	0.004863	hypermethylated
cg17608570	0.37914	0.529421622	0.481686	0.003705	hypermethylated
cg18527574	0.52841	0.737754595	0.481483	4.94E-05	hypermethylated
cg17479280	0.50415	0.703778919	0.481269	7.65E-05	hypermethylated
cg16826777	0.40522	0.565574054	0.481011	4.70E-05	hypermethylated
cg18031134	0.27117	0.378468649	0.480976	0.0002	hypermethylated
cg24317217	0.41904	0.584835676	0.480943	0.0182	hypermethylated
cg10749822	0.31472	0.439227568	0.4809	1.15E-05	hypermethylated
cg25306277	0.46087	0.643119459	0.480727	0.000772	hypermethylated
cg02859129	0.43174	0.602436216	0.480646	8.42E-05	hypermethylated
cg05452645	0.4333	0.604602703	0.480621	3.40E-05	hypermethylated
cg02106682	0.37558	0.524022703	0.480509	0.001188	hypermethylated
cg25935985	0.40894	0.570475676	0.480276	5.50E-06	hypermethylated
cg21078322	0.48429	0.675502162	0.480089	0.029449	hypermethylated
cg10267867	0.37963	0.529511892	0.480069	0.00066	hypermethylated
cg12599168	0.47199	0.658293514	0.479975	0.003637	hypermethylated
cg03601372	0.42293	0.589804324	0.479818	0.01146	hypermethylated
cg01413790	0.28994	0.404325946	0.479764	0.000575	hypermethylated
cg11868485	0.49303	0.687385946	0.479445	0.006015	hypermethylated
cg27509347	0.42334	0.590153514	0.479273	0.00504	hypermethylated
cg03202564	0.46035	0.641685946	0.479136	2.18E-05	hypermethylated
cg22231400	0.39696	0.553290811	0.479044	0.000293	hypermethylated
cg23884241	0.34658	0.483056216	0.479003	0.000822	hypermethylated
cg14522034	0.38679	0.539096757	0.478994	0.001603	hypermethylated
cg23358886	0.40135	0.559374054	0.478952	0.0006	hypermethylated
cg12341314	0.36256	0.505282703	0.478871	0.000204	hypermethylated
cg01245224	0.55627	0.77518	0.478746	0.002147	hypermethylated
cg15639951	0.41496	0.578248649	0.478718	0.000848	hypermethylated
cg25007722	0.37868	0.527655676	0.478618	1.30E-05	hypermethylated
cg02074191	0.4228	0.589059459	0.478438	3.40E-05	hypermethylated
cg26377880	0.46119	0.642541081	0.478427	0.017101	hypermethylated
cg16021217	0.36324	0.506073514	0.478424	0.005269	hypermethylated
cg04674315	0.483	0.672838378	0.478237	1.15E-05	hypermethylated
cg25727671	0.52424	0.730053514	0.477775	0.009959	hypermethylated
cg08091601	0.49076	0.683408108	0.47773	0.041702	hypermethylated
cg16626580	0.38988	0.542892432	0.477636	0.000221	hypermethylated
cg20721467	0.51098	0.711477297	0.477551	0.006796	hypermethylated
cg07724623	0.4073	0.567024324	0.477319	0.000551	hypermethylated
cg18739887	0.41818	0.582097838	0.477138	0.003571	hypermethylated
cg02938172	0.42075	0.585674595	0.477136	0.001839	hypermethylated
cg08564636	0.48054	0.668857838	0.477043	0.034997	hypermethylated
cg13057576	0.5817	0.80965027	0.477024	0.000681	hypermethylated
cg23855818	0.5487	0.763615135	0.476828	0.001768	hypermethylated
cg20070618	0.5047	0.702267568	0.476595	0.019361	hypermethylated
cg27031484	0.53233	0.740614595	0.476402	0.00291	hypermethylated
cg27159583	0.50032	0.696060541	0.476362	0.036516	hypermethylated
cg26775079	0.42639	0.593134054	0.476185	0.000374	hypermethylated

cg16924061	0.56541	0.786481081	0.476115	0.003225	hypermethylated
cg04986781	0.37559	0.522416757	0.476043	0.000172	hypermethylated
cg07097876	0.40218	0.559338378	0.47588	0.000921	hypermethylated
cg16583088	0.63991	0.88991027	0.475791	0.00049	hypermethylated
cg02420027	0.42141	0.586002162	0.475681	0.005706	hypermethylated
cg11062417	0.45546	0.633240541	0.475429	0.002699	hypermethylated
cg15045292	0.32568	0.452774054	0.475336	0.001912	hypermethylated
cg13788819	0.47359	0.658272432	0.475046	0.000123	hypermethylated
cg27006947	0.38567	0.535972432	0.474792	0.00101	hypermethylated
cg23889730	0.36243	0.503425405	0.474076	5.31E-05	hypermethylated
cg25911551	0.41037	0.570002703	0.474043	0.018773	hypermethylated
cg03421195	0.5336	0.741162703	0.474032	0.001734	hypermethylated
cg20457275	0.31781	0.441356216	0.473779	0.001188	hypermethylated
cg17662941	0.46163	0.641042162	0.473682	0.000143	hypermethylated
cg05112120	0.38604	0.536030811	0.473566	0.003538	hypermethylated
cg01023808	0.63857	0.886666486	0.473547	0.000588	hypermethylated
cg26430027	0.49283	0.684123784	0.473167	3.95E-05	hypermethylated
cg12577942	0.6261	0.869109189	0.473144	0.002126	hypermethylated
cg01122167	0.4381	0.608054054	0.472939	0.044065	hypermethylated
cg20120512	0.37274	0.517263784	0.472731	0.000123	hypermethylated
cg03888064	0.54279	0.753181081	0.472603	0.000575	hypermethylated
cg17039428	0.53083	0.736582162	0.472597	0.001717	hypermethylated
cg07496545	0.58052	0.805447568	0.472445	0.000506	hypermethylated
cg19675142	0.41864	0.580839459	0.472429	0.000174	hypermethylated
cg00852921	0.45631	0.633002162	0.472196	1.94E-05	hypermethylated
cg03622666	0.40564	0.562701622	0.47217	0.000588	hypermethylated
cg26413683	0.35902	0.497961622	0.47197	5.86E-05	hypermethylated
cg06097077	0.53839	0.74673027	0.471936	0.000517	hypermethylated
cg06118571	0.42501	0.589424324	0.47181	0.000893	hypermethylated
cg13435326	0.48443	0.671815676	0.471777	0.001164	hypermethylated
cg20546778	0.57658	0.799391892	0.471382	0.001327	hypermethylated
cg03479211	0.45542	0.631375676	0.471301	0.005411	hypermethylated
cg02182210	0.43988	0.609766486	0.471147	0.000594	hypermethylated
cg21167761	0.45776	0.63443027	0.47087	0.002026	hypermethylated
cg00048759	0.46003	0.637456216	0.470598	1.06E-05	hypermethylated
cg24768365	0.42329	0.586501622	0.470489	0.000209	hypermethylated
cg07926618	0.5669	0.785297297	0.470145	0.002046	hypermethylated
cg02106466	0.24501	0.339394054	0.470121	0.003705	hypermethylated
cg06641366	0.50174	0.695008108	0.47009	0.018485	hypermethylated
cg24497732	0.37347	0.517261081	0.4699	7.38E-05	hypermethylated
cg08000731	0.42698	0.591305405	0.469735	0.005606	hypermethylated
cg00061039	0.51698	0.715931892	0.469714	0.019661	hypermethylated
cg22506453	0.59662	0.826047568	0.469413	0.000331	hypermethylated
cg21292909	0.60914	0.84332	0.469306	0.001987	hypermethylated
cg13048147	0.5949	0.823486486	0.469098	7.74E-05	hypermethylated
cg05991820	0.45479	0.629518919	0.469049	0.000613	hypermethylated
cg14970273	0.41663	0.576661622	0.468958	0.000797	hypermethylated
cg02711479	0.61089	0.845504865	0.4689	0.000474	hypermethylated
cg26206598	0.45335	0.627422703	0.468812	0.000152	hypermethylated
cg18087266	0.34277	0.474359459	0.46874	0.001839	hypermethylated
cg06878709	0.34825	0.48193027	0.468701	0.001275	hypermethylated
cg02611934	0.44629	0.617578378	0.468641	0.001587	hypermethylated
cg26363555	0.39411	0.545341622	0.468562	0.006564	hypermethylated
cg12354056	0.50983	0.705445946	0.468519	0.009552	hypermethylated
cg02907098	0.3166	0.438074054	0.468514	0.000219	hypermethylated
cg21569714	0.62845	0.86928973	0.468039	4.82E-05	hypermethylated
cg09504320	0.40942	0.566164324	0.467639	4.00E-05	hypermethylated
cg27338202	0.55699	0.770199459	0.467581	0.000839	hypermethylated

cg23831876	0.46562	0.643853514	0.467579	0.000563	hypermethylated
cg24377495	0.46976	0.649530811	0.467474	0.000857	hypermethylated
cg16748643	0.43305	0.59862	0.467107	0.003441	hypermethylated
cg03346415	0.60371	0.834516216	0.467084	0.006395	hypermethylated
cg08944086	0.58959	0.814771892	0.466684	0.014838	hypermethylated
cg09096555	0.47133	0.651268649	0.466515	4.94E-05	hypermethylated
cg25649039	0.47769	0.660046486	0.466493	0.024491	hypermethylated
cg06146977	0.50147	0.692818378	0.466314	0.001096	hypermethylated
cg20443451	0.40311	0.556827027	0.466056	0.001803	hypermethylated
cg09395612	0.48768	0.673351351	0.465425	5.28E-06	hypermethylated
cg05965387	0.43573	0.601586486	0.465338	0.000805	hypermethylated
cg04159420	0.35067	0.484074054	0.465114	0.000191	hypermethylated
cg12716639	0.49508	0.683358919	0.464982	0.003316	hypermethylated
cg17789809	0.55785	0.769907568	0.464808	0.003473	hypermethylated
cg07737063	0.66504	0.917615135	0.464448	0.000464	hypermethylated
cg24739457	0.57215	0.789442703	0.464441	0.029023	hypermethylated
cg05845376	0.57473	0.793002162	0.46444	0.001118	hypermethylated
cg26820209	0.54341	0.749780541	0.464427	0.004365	hypermethylated
cg23316599	0.40493	0.558707027	0.464419	9.05E-05	hypermethylated
cg18690385	0.60908	0.840207568	0.464114	2.85E-05	hypermethylated
cg16536855	0.50417	0.695465946	0.46407	0.000857	hypermethylated
cg26634219	0.52411	0.722901622	0.46393	0.007601	hypermethylated
cg12600201	0.43971	0.606479459	0.463906	0.043166	hypermethylated
cg21681320	0.45195	0.62335027	0.46388	0.000912	hypermethylated
cg25500028	0.44465	0.613257838	0.463824	6.62E-05	hypermethylated
cg18920088	0.61268	0.844969189	0.463765	0.001164	hypermethylated
cg20230340	0.61681	0.850661622	0.463759	0.005606	hypermethylated
cg04250451	0.38306	0.528288108	0.463755	0.005606	hypermethylated
cg24047810	0.36243	0.499803784	0.463659	0.000103	hypermethylated
cg03096785	0.51289	0.707263784	0.463599	0.011274	hypermethylated
cg18582342	0.33579	0.463040541	0.463579	0.000123	hypermethylated
cg00654888	0.32263	0.444866486	0.463492	6.78E-05	hypermethylated
cg13931725	0.33093	0.456300541	0.463458	4.31E-05	hypermethylated
cg11716380	0.36636	0.505085946	0.463267	0.000268	hypermethylated
cg23222169	0.58385	0.80486973	0.463158	0.008278	hypermethylated
cg04153551	0.45616	0.628835135	0.463142	0.020903	hypermethylated
cg19671395	0.64139	0.884138378	0.46307	0.006621	hypermethylated
cg26023912	0.38537	0.531216757	0.463056	0.0017	hypermethylated
cg21408915	0.43168	0.59500973	0.462951	1.30E-05	hypermethylated
cg20319604	0.55081	0.759122162	0.462777	0.000667	hypermethylated
cg22082800	0.24065	0.331610811	0.462555	0.003136	hypermethylated
cg14644846	0.40748	0.561455676	0.462443	0.049464	hypermethylated
cg07658280	0.52974	0.729892973	0.4624	0.016836	hypermethylated
cg19728382	0.42004	0.578587568	0.462009	0.000163	hypermethylated
cg19864468	0.57016	0.785228108	0.461745	0.000109	hypermethylated
cg14364472	0.65042	0.895607568	0.461495	8.22E-05	hypermethylated
cg02014853	0.35152	0.483942703	0.461229	3.76E-05	hypermethylated
cg06176824	0.43421	0.597771351	0.461201	0.000126	hypermethylated
cg02426178	0.36883	0.507758378	0.461186	0.003986	hypermethylated
cg14738670	0.28739	0.395616216	0.461092	0.009877	hypermethylated
cg13532463	0.49589	0.682401622	0.460601	0.031661	hypermethylated
cg18687914	0.39094	0.537963243	0.46056	0.001096	hypermethylated
cg10327980	0.48337	0.665137297	0.460524	0.000145	hypermethylated
cg02269978	0.44064	0.606337838	0.460521	0.000245	hypermethylated
cg05285244	0.41292	0.568167568	0.460454	0.001074	hypermethylated
cg04205744	0.435	0.598469189	0.460262	0.002066	hypermethylated
cg13424940	0.4504	0.619630811	0.460202	0.00032	hypermethylated
cg14417099	0.49986	0.687661081	0.460174	0.002829	hypermethylated

cg04891961	0.39199	0.539206486	0.460021	0.000187	hypermethylated
cg03225520	0.60346	0.829977838	0.459815	0.000342	hypermethylated
cg01835580	0.54806	0.753727568	0.459709	0.001603	hypermethylated
cg13983776	0.27313	0.375618378	0.45968	3.64E-06	hypermethylated
cg26720545	0.43606	0.599645405	0.459583	0.000408	hypermethylated
cg05983061	0.34754	0.477915135	0.459575	0.001481	hypermethylated
cg23679492	0.43437	0.597303243	0.459539	0.00099	hypermethylated
cg27100471	0.50533	0.694821081	0.459416	0.006339	hypermethylated
cg08285446	0.45783	0.629422162	0.459216	3.23E-05	hypermethylated
cg23204968	0.47738	0.656274054	0.45916	0.004365	hypermethylated
cg19821713	0.46005	0.632243243	0.458689	7.83E-06	hypermethylated
cg04720592	0.50463	0.69333027	0.458317	0.001314	hypermethylated
cg11943209	0.37323	0.512687568	0.458015	0.000111	hypermethylated
cg00444151	0.45768	0.628646486	0.45791	0.001526	hypermethylated
cg05738240	0.58089	0.797757297	0.457685	0.003378	hypermethylated
cg13803688	0.47274	0.649104865	0.457405	3.07E-05	hypermethylated
cg13537237	0.57401	0.788128108	0.457354	2.78E-05	hypermethylated
cg05325182	0.48316	0.663353514	0.457277	0.00064	hypermethylated
cg02964172	0.65699	0.901830811	0.456985	0.01951	hypermethylated
cg06144718	0.37751	0.518157297	0.456875	0.000667	hypermethylated
cg22701534	0.52594	0.721869189	0.456839	0.004365	hypermethylated
cg02374107	0.44717	0.613744865	0.456816	0.003843	hypermethylated
cg08657492	0.39348	0.539906486	0.456419	5.51E-05	hypermethylated
cg01210589	0.48229	0.661757838	0.456402	5.65E-05	hypermethylated
cg26937943	0.51768	0.710279459	0.456326	0.037035	hypermethylated
cg18197808	0.58392	0.800906486	0.455863	0.006451	hypermethylated
cg19256368	0.59013	0.809347568	0.455727	0.000386	hypermethylated
cg09576209	0.3754	0.514839459	0.455694	0.0012	hypermethylated
cg22911054	0.53266	0.730478919	0.455628	0.014373	hypermethylated
cg08731435	0.66023	0.905307027	0.455438	1.62E-05	hypermethylated
cg11452043	0.62076	0.851076757	0.455254	0.001327	hypermethylated
cg10089801	0.39816	0.545788108	0.454993	0.00012	hypermethylated
cg25588274	0.52129	0.714543243	0.454935	0.016187	hypermethylated
cg01410359	0.2994	0.410361081	0.45482	0.000129	hypermethylated
cg02950701	0.59131	0.810452973	0.454814	0.001262	hypermethylated
cg06878741	0.47571	0.65196	0.454701	0.00504	hypermethylated
cg17802213	0.36619	0.501825946	0.454595	0.000374	hypermethylated
cg02633036	0.55239	0.756918378	0.454451	0.01348	hypermethylated
cg05940452	0.38966	0.533933514	0.454444	0.000893	hypermethylated
cg18912160	0.54806	0.75097027	0.454422	0.03076	hypermethylated
cg10543634	0.61847	0.847311351	0.454189	0.014604	hypermethylated
cg18161956	0.41648	0.570543243	0.454089	0.000426	hypermethylated
cg23285825	0.36349	0.497921081	0.454001	5.25E-05	hypermethylated
cg08902698	0.36893	0.505314595	0.453835	0.002231	hypermethylated
cg05255351	0.45259	0.619876757	0.453777	0.000124	hypermethylated
cg26679785	0.35196	0.482043243	0.453751	1.58E-05	hypermethylated
cg01789499	0.53365	0.730864865	0.453711	0.000551	hypermethylated
cg07380026	0.43566	0.596637838	0.453653	0.000132	hypermethylated
cg24022656	0.5482	0.750615135	0.453371	0.002086	hypermethylated
cg06162185	0.52095	0.71328973	0.453343	0.00101	hypermethylated
cg04619046	0.54746	0.749576757	0.453323	5.93E-05	hypermethylated
cg12032648	0.5258	0.719802162	0.453086	0.005316	hypermethylated
cg01465769	0.45483	0.622491892	0.452728	7.84E-05	hypermethylated
cg26621770	0.44815	0.613262162	0.452522	0.0002	hypermethylated
cg14023291	0.57805	0.791011351	0.452504	8.83E-06	hypermethylated
cg14482712	0.54258	0.742423784	0.452407	0.000187	hypermethylated
cg24183575	0.49216	0.673318919	0.452163	0.002007	hypermethylated
cg18605377	0.28203	0.385816757	0.452067	0.007035	hypermethylated

cg03653317	0.56004	0.766095676	0.451995	0.00513	hypermethylated
cg01454947	0.59259	0.810524865	0.451822	0.001314	hypermethylated
cg22312354	0.44011	0.601876757	0.451604	0.001164	hypermethylated
cg18931036	0.36978	0.505671351	0.451533	5.86E-05	hypermethylated
cg05288253	0.34343	0.469560541	0.451295	0.001466	hypermethylated
cg05317851	0.38074	0.520479459	0.451035	6.01E-05	hypermethylated
cg25461249	0.49739	0.679909189	0.450965	0.009238	hypermethylated
cg22050611	0.48927	0.668631892	0.450581	0.005859	hypermethylated
cg23937382	0.33564	0.458632432	0.450424	0.0003	hypermethylated
cg24587297	0.44574	0.608719459	0.449575	0.000588	hypermethylated
cg11711057	0.45938	0.627326486	0.449528	0.002751	hypermethylated
cg06392574	0.47134	0.643636757	0.449479	0.011649	hypermethylated
cg01285926	0.4446	0.607092432	0.449408	1.99E-05	hypermethylated
cg15358549	0.55243	0.754295135	0.449337	0.008636	hypermethylated
cg06291314	0.63972	0.873388649	0.449183	0.001651	hypermethylated
cg23008562	0.35588	0.485815135	0.449017	1.80E-05	hypermethylated
cg02232089	0.62833	0.857072432	0.447895	0.001893	hypermethylated
cg02172492	0.38553	0.525815676	0.447714	7.65E-05	hypermethylated
cg22846776	0.52147	0.711147568	0.447565	0.004134	hypermethylated
cg20770435	0.52144	0.711094595	0.44754	4.26E-05	hypermethylated
cg02880877	0.56079	0.764744324	0.447517	0.000588	hypermethylated
cg18926450	0.62391	0.850802162	0.447486	0.002409	hypermethylated
cg22720790	0.49856	0.679837297	0.447422	0.002699	hypermethylated
cg04689058	0.47528	0.648068108	0.447368	0.00039	hypermethylated
cg12085698	0.60838	0.829498378	0.447266	0.00101	hypermethylated
cg10297617	0.56577	0.771317838	0.44711	0.005411	hypermethylated
cg01522592	0.45888	0.625560541	0.447033	0.00035	hypermethylated
cg22884541	0.59355	0.809094595	0.446939	0.005706	hypermethylated
cg25693769	0.46759	0.63736973	0.446886	2.10E-05	hypermethylated
cg15967709	0.32048	0.436841081	0.446874	1.75E-05	hypermethylated
cg25841625	0.46662	0.635875135	0.446495	0.003571	hypermethylated
cg05355757	0.49597	0.675796757	0.446337	0.00032	hypermethylated
cg06872981	0.34548	0.470681081	0.446148	0.000342	hypermethylated
cg03579904	0.54953	0.748622703	0.446041	0.004526	hypermethylated
cg24279017	0.42076	0.573171892	0.44597	0.000741	hypermethylated
cg16677112	0.48974	0.667023243	0.445721	0.017101	hypermethylated
cg17762554	0.50169	0.683234054	0.445584	0.000142	hypermethylated
cg10381113	0.63173	0.860268108	0.445478	3.85E-05	hypermethylated
cg07506916	0.43453	0.591724324	0.445469	0.001212	hypermethylated
cg25390165	0.42187	0.574261081	0.444908	0.001667	hypermethylated
cg01790083	0.3588	0.488404324	0.444896	0.007096	hypermethylated
cg11624345	0.34111	0.46428973	0.444788	0.002803	hypermethylated
cg04329125	0.46531	0.633287568	0.444669	0.001651	hypermethylated
cg14424407	0.30705	0.417848108	0.444505	0.000132	hypermethylated
cg26767593	0.5337	0.726248649	0.444435	0.002409	hypermethylated
cg10329579	0.49884	0.678755676	0.444315	0.019213	hypermethylated
cg08828036	0.51092	0.69516	0.444248	0.001409	hypermethylated
cg01631277	0.61998	0.843511351	0.444186	0.004863	hypermethylated
cg00347620	0.40081	0.545252973	0.444007	3.71E-05	hypermethylated
cg06306791	0.42067	0.572176757	0.443772	0.023769	hypermethylated
cg14547895	0.41491	0.564301622	0.443668	0.002231	hypermethylated
cg12635937	0.36971	0.502763784	0.443487	8.84E-05	hypermethylated
cg26540191	0.59089	0.803522703	0.443449	0.001496	hypermethylated
cg24442760	0.37035	0.503560541	0.443276	3.71E-05	hypermethylated
cg08292467	0.38451	0.522761081	0.443131	0.0002	hypermethylated
cg20087519	0.43656	0.593482703	0.443026	0.000262	hypermethylated
cg04865220	0.55393	0.752811351	0.442585	0.001571	hypermethylated
cg03630821	0.43408	0.589765946	0.442182	0.000789	hypermethylated

cg13512268	0.49888	0.677662703	0.441875	0.002751	hypermethylated
cg07231479	0.35414	0.481007568	0.44174	2.45E-05	hypermethylated
cg10909790	0.49095	0.666658919	0.441373	0.000268	hypermethylated
cg00324748	0.54001	0.732954054	0.440737	0.020903	hypermethylated
cg26132493	0.43313	0.58773027	0.440354	5.58E-05	hypermethylated
cg03695871	0.55449	0.752407027	0.440352	0.007035	hypermethylated
cg18656708	0.34646	0.470078919	0.440214	6.75E-06	hypermethylated
cg17120366	0.5755	0.780838919	0.440209	0.004059	hypermethylated
cg09974780	0.48015	0.651359459	0.439969	0.001452	hypermethylated
cg11574745	0.56097	0.760977297	0.43993	2.21E-05	hypermethylated
cg22671717	0.34495	0.46792973	0.439905	0.000248	hypermethylated
cg23224191	0.33953	0.460543243	0.439798	0.001395	hypermethylated
cg13857947	0.51813	0.702797297	0.439795	0.001164	hypermethylated
cg13663855	0.51724	0.701558378	0.439729	0.000287	hypermethylated
cg02281167	0.32066	0.434923243	0.439716	9.83E-05	hypermethylated
cg01281904	0.40494	0.549216757	0.439667	0.000575	hypermethylated
cg18080733	0.48858	0.662634595	0.439619	0.008208	hypermethylated
cg26782150	0.49512	0.671476757	0.439559	0.016061	hypermethylated
cg27019093	0.61928	0.83980973	0.439471	0.000148	hypermethylated
cg04856896	0.47446	0.643223784	0.439034	0.003195	hypermethylated
cg15689969	0.64255	0.870986486	0.438842	0.000469	hypermethylated
cg16294838	0.56173	0.761406486	0.43879	0.010211	hypermethylated
cg09178678	0.34556	0.468388108	0.438768	2.27E-05	hypermethylated
cg09099868	0.46592	0.631492973	0.438684	4.15E-05	hypermethylated
cg05579037	0.54563	0.739477297	0.438583	0.000688	hypermethylated
cg00274203	0.48225	0.653456216	0.438309	0.001	hypermethylated
cg08056229	0.61187	0.829077297	0.438281	0.000563	hypermethylated
cg27408345	0.63347	0.857786486	0.437342	0.001786	hypermethylated
cg09596722	0.62377	0.844574054	0.43721	0.005316	hypermethylated
cg25750507	0.55674	0.753805405	0.437188	0.007933	hypermethylated
cg15362957	0.4231	0.572774595	0.436969	0.000137	hypermethylated
cg14848450	0.54405	0.736511351	0.436969	0.029449	hypermethylated
cg04419883	0.34457	0.466410811	0.436804	0.012036	hypermethylated
cg23528791	0.3658	0.495098378	0.43666	5.45E-05	hypermethylated
cg20050826	0.33014	0.446798919	0.436548	0.000251	hypermethylated
cg14912034	0.48948	0.662376757	0.436402	0.004863	hypermethylated
cg21928406	0.46531	0.629621081	0.436292	1.73E-05	hypermethylated
cg02641288	0.34507	0.466873514	0.436143	2.42E-05	hypermethylated
cg25008858	0.34741	0.470031351	0.436118	0.000159	hypermethylated
cg09938049	0.34234	0.463143243	0.436029	0.003637	hypermethylated
cg17588578	0.4048	0.547641081	0.436021	0.000931	hypermethylated
cg17250160	0.47364	0.640763243	0.436	3.57E-05	hypermethylated
cg02205073	0.44806	0.606131351	0.435939	0.018773	hypermethylated
cg26382551	0.51526	0.697021081	0.435902	0.004134	hypermethylated
cg25981315	0.44068	0.596076757	0.435767	0.003473	hypermethylated
cg00449067	0.51394	0.695164865	0.435755	0.00028	hypermethylated
cg21795221	0.40094	0.542261622	0.435603	0.001949	hypermethylated
cg07592254	0.6014	0.813333514	0.435522	0.000875	hypermethylated
cg00144335	0.43357	0.586263243	0.435284	9.49E-05	hypermethylated
cg11562901	0.55006	0.743746486	0.435222	0.002147	hypermethylated
cg22367264	0.4816	0.651176757	0.435214	0.000696	hypermethylated
cg09194815	0.66282	0.896184324	0.435178	0.001096	hypermethylated
cg26735980	0.45397	0.613728649	0.435004	0.024675	hypermethylated
cg27293357	0.49764	0.672735676	0.434937	0.000417	hypermethylated
cg02775230	0.57624	0.778930811	0.434825	0.011181	hypermethylated
cg25773262	0.41126	0.555917838	0.434821	2.89E-05	hypermethylated
cg01514490	0.47442	0.641152973	0.434504	0.001368	hypermethylated
cg13210467	0.43564	0.588574054	0.434088	4.05E-05	hypermethylated

cg17517296	0.3725	0.503218378	0.433944	9.16E-05	hypermethylated
cg19847945	0.40996	0.553778378	0.433826	0.015935	hypermethylated
cg24553170	0.4152	0.560807568	0.433699	0.002318	hypermethylated
cg07703530	0.57363	0.774739459	0.433591	0.000875	hypermethylated
cg01417692	0.69787	0.942508649	0.433548	0.000695	hypermethylated
cg00902516	0.64051	0.864998378	0.433476	0.000696	hypermethylated
cg27635470	0.59845	0.808121081	0.433341	0.000212	hypermethylated
cg12497374	0.36528	0.493177838	0.433105	0.000324	hypermethylated
cg02022519	0.43239	0.583773514	0.433076	0.001452	hypermethylated
cg19023320	0.45311	0.611653514	0.432853	0.012844	hypermethylated
cg19095000	0.50835	0.686170811	0.432746	0.000108	hypermethylated
cg18466173	0.55719	0.752068108	0.432694	0.002599	hypermethylated
cg20852557	0.4564	0.616008649	0.432652	0.006451	hypermethylated
cg16179589	0.55613	0.750598378	0.432619	0.001164	hypermethylated
cg12948920	0.32368	0.436823784	0.432483	0.000113	hypermethylated
cg13187827	0.38912	0.525115135	0.432419	0.003473	hypermethylated
cg24874557	0.54197	0.73132	0.43229	0.004819	hypermethylated
cg14624314	0.55378	0.747227027	0.432234	0.002318	hypermethylated
cg05616010	0.46949	0.633307568	0.431812	0.000474	hypermethylated
cg17592231	0.40971	0.552640541	0.431738	8.60E-06	hypermethylated
cg09354454	0.3723	0.502135135	0.43161	0.0006	hypermethylated
cg22108374	0.439	0.592051351	0.431501	0.000182	hypermethylated
cg06539116	0.37117	0.500540541	0.431407	0.000789	hypermethylated
cg08295111	0.43636	0.588428108	0.431347	0.004691	hypermethylated
cg16830930	0.37526	0.50598	0.43119	3.32E-05	hypermethylated
cg16246661	0.59254	0.798907568	0.431116	0.001635	hypermethylated
cg16236779	0.61865	0.834075135	0.431054	0.00101	hypermethylated
cg22849665	0.46053	0.620812432	0.430862	6.86E-05	hypermethylated
cg23143313	0.39282	0.529494595	0.430748	9.05E-05	hypermethylated
cg04213746	0.54978	0.741057297	0.430731	0.004445	hypermethylated
cg01119875	0.46695	0.629336757	0.430564	0.00134	hypermethylated
cg08553601	0.44721	0.602720541	0.430537	0.001237	hypermethylated
cg03143486	0.57192	0.770647568	0.430258	0.000224	hypermethylated
cg17147317	0.37882	0.510343784	0.429957	0.000112	hypermethylated
cg17124583	0.33779	0.455061081	0.429934	0.00101	hypermethylated
cg05029822	0.33095	0.445836216	0.429901	3.67E-05	hypermethylated
cg09835408	0.48463	0.652858919	0.429888	1.66E-05	hypermethylated
cg02578944	0.51649	0.69575027	0.429829	0.000408	hypermethylated
cg24683222	0.51325	0.691167568	0.429374	5.86E-05	hypermethylated
cg05487664	0.34839	0.469151892	0.429352	7.65E-05	hypermethylated
cg17522727	0.5754	0.774828108	0.429311	0.007536	hypermethylated
cg21641458	0.43929	0.591537297	0.429295	0.0017	hypermethylated
cg20826165	0.55059	0.741403784	0.429281	0.006796	hypermethylated
cg06732684	0.37438	0.504093514	0.429188	3.67E-05	hypermethylated
cg18543991	0.57802	0.778283243	0.429176	0.004365	hypermethylated
cg27345762	0.54956	0.739962162	0.429174	0.009238	hypermethylated
cg08415977	0.38171	0.513878919	0.428951	7.84E-05	hypermethylated
cg08446824	0.6072	0.817355135	0.428791	0.001452	hypermethylated
cg10013501	0.48728	0.655924324	0.428778	0.01073	hypermethylated
cg01952185	0.3463	0.466107027	0.428639	0.000741	hypermethylated
cg04904318	0.67148	0.903712973	0.42852	0.001821	hypermethylated
cg15512534	0.49266	0.662869189	0.428132	0.002066	hypermethylated
cg17775765	0.40848	0.549601622	0.428121	8.83E-06	hypermethylated
cg15833565	0.5023	0.675808649	0.428066	0.000102	hypermethylated
cg09915729	0.56788	0.764016216	0.428017	0.020274	hypermethylated
cg04060571	0.34335	0.461917297	0.427955	0.000374	hypermethylated
cg11100481	0.43239	0.581676757	0.427884	0.000756	hypermethylated
cg00254306	0.38965	0.524144865	0.427787	0.000176	hypermethylated

cg06584433	0.58781	0.790682703	0.427749	0.000408	hypermethylated
cg01316476	0.49805	0.669940541	0.427742	0.000109	hypermethylated
cg25331919	0.60626	0.815491892	0.427734	0.000557	hypermethylated
cg12954234	0.43395	0.583632432	0.427531	0.008208	hypermethylated
cg01342411	0.68102	0.915878378	0.427459	7.29E-05	hypermethylated
cg11956467	0.53678	0.721869189	0.427407	0.005859	hypermethylated
cg20416874	0.47893	0.643998378	0.427242	0.013053	hypermethylated
cg01096617	0.61469	0.82654	0.427226	0.022893	hypermethylated
cg11041817	0.52372	0.704181081	0.427151	0.001912	hypermethylated
cg19596468	0.4861	0.653584865	0.427121	3.76E-05	hypermethylated
cg06055845	0.41261	0.554747568	0.427053	0.000378	hypermethylated
cg21618730	0.53363	0.71742973	0.426998	3.07E-05	hypermethylated
cg15868425	0.55383	0.744545946	0.426918	8.63E-05	hypermethylated
cg05339076	0.62801	0.844240541	0.426867	9.72E-05	hypermethylated
cg13007988	0.5664	0.761371892	0.42678	0.015076	hypermethylated
cg11847933	0.41336	0.555561081	0.426547	0.00048	hypermethylated
cg21870668	0.44734	0.601218919	0.426519	0.000224	hypermethylated
cg20917338	0.62784	0.843794595	0.426495	0.000445	hypermethylated
cg15966151	0.45945	0.617483784	0.426493	0.001734	hypermethylated
cg19294953	0.39447	0.530148649	0.426481	0.000117	hypermethylated
cg23350904	0.41086	0.552057838	0.426173	0.000106	hypermethylated
cg06733394	0.5315	0.713989189	0.425833	0.010296	hypermethylated
cg13599092	0.35576	0.477875135	0.425729	0.000193	hypermethylated
cg08274637	0.44159	0.593008649	0.425346	0.00125	hypermethylated
cg11085454	0.31824	0.427357838	0.425329	0.004567	hypermethylated
cg14882265	0.42677	0.57305027	0.425203	0.001912	hypermethylated
cg00031105	0.42357	0.568724324	0.425129	0.015809	hypermethylated
cg16257029	0.37001	0.496734054	0.424909	5.86E-05	hypermethylated
cg14440640	0.41707	0.559837838	0.424719	0.000726	hypermethylated
cg05713044	0.62418	0.837832973	0.424701	0.007408	hypermethylated
cg06036239	0.42514	0.570589189	0.424514	0.016704	hypermethylated
cg02141570	0.37287	0.500387568	0.424373	8.63E-05	hypermethylated
cg21639031	0.30116	0.404123243	0.424265	0.001368	hypermethylated
cg21615397	0.6216	0.834067027	0.424177	0.00305	hypermethylated
cg04521583	0.433	0.580980541	0.424123	0.024309	hypermethylated
cg24457118	0.58154	0.780222703	0.424008	0.000109	hypermethylated
cg03550129	0.40659	0.545443243	0.423854	0.001912	hypermethylated
cg15418593	0.468	0.627795676	0.423787	0.004134	hypermethylated
cg23337631	0.43854	0.588227027	0.423665	0.000696	hypermethylated
cg09636525	0.5119	0.686476216	0.423348	0.006284	hypermethylated
cg26353287	0.6425	0.861614054	0.423345	0.001212	hypermethylated
cg18597315	0.64708	0.867685405	0.423228	0.001118	hypermethylated
cg05333149	0.5158	0.691637838	0.423205	0.000219	hypermethylated
cg18266783	0.4867	0.652494054	0.422932	0.01274	hypermethylated
cg01143938	0.37851	0.507373514	0.422717	0.001667	hypermethylated
cg25592918	0.47975	0.643076216	0.422707	0.000274	hypermethylated
cg25103417	0.4353	0.583443784	0.422584	0.00032	hypermethylated
cg23722790	0.49787	0.667307568	0.422583	0.004819	hypermethylated
cg08802358	0.52413	0.702361081	0.422288	0.000495	hypermethylated
cg07255841	0.48148	0.645050811	0.421937	0.000485	hypermethylated
cg22302929	0.46937	0.628772432	0.421812	0.006122	hypermethylated
cg15037137	0.50941	0.682358378	0.421702	0.009238	hypermethylated
cg23121787	0.58537	0.784067568	0.421629	0.007601	hypermethylated
cg22517388	0.36332	0.486631351	0.421588	2.24E-05	hypermethylated
cg00495201	0.56688	0.759274054	0.421577	0.000902	hypermethylated
cg00144673	0.44477	0.595717838	0.42157	0.004405	hypermethylated
cg03908391	0.54025	0.723511892	0.42139	6.78E-05	hypermethylated
cg06511276	0.47496	0.636041622	0.421315	0.000148	hypermethylated

cg16122424	0.45165	0.604776216	0.421196	6.62E-05	hypermethylated
cg04536844	0.47455	0.635363784	0.421023	0.000234	hypermethylated
cg04489066	0.60478	0.809722162	0.421017	0.002126	hypermethylated
cg05697976	0.55491	0.742951351	0.421014	0.000822	hypermethylated
cg00513316	0.39127	0.523835135	0.420948	0.000126	hypermethylated
cg11232245	0.56323	0.75402973	0.420897	0.003286	hypermethylated
cg12075498	0.5473	0.732483784	0.420465	0.000485	hypermethylated
cg20839206	0.45865	0.61376973	0.420304	2.74E-05	hypermethylated
cg27129048	0.42888	0.573890811	0.420202	0.000417	hypermethylated
cg21158528	0.53892	0.721121081	0.42017	0.003166	hypermethylated
cg10534873	0.60138	0.804667027	0.420115	0.001839	hypermethylated
cg20763327	0.51617	0.690457297	0.419706	0.001074	hypermethylated
cg23290121	0.46281	0.619045946	0.419626	0.000123	hypermethylated
cg22805603	0.4688	0.626864324	0.419181	0.000772	hypermethylated
cg22352800	0.38617	0.516374054	0.41918	0.000575	hypermethylated
cg16770238	0.40572	0.542490811	0.419114	6.95E-05	hypermethylated
cg24399349	0.4089	0.546737297	0.4191	6.95E-05	hypermethylated
cg21206652	0.33844	0.452517297	0.419073	0.007799	hypermethylated
cg10856812	0.50988	0.681700541	0.41898	0.009959	hypermethylated
cg10553204	0.55397	0.740598919	0.418885	0.00024	hypermethylated
cg12268562	0.38719	0.517601081	0.418799	3.07E-05	hypermethylated
cg18466162	0.61922	0.827622162	0.41852	0.000822	hypermethylated
cg10274108	0.55823	0.746065946	0.418443	5.19E-05	hypermethylated
cg05478407	0.46647	0.623412973	0.418404	3.40E-05	hypermethylated
cg14935646	0.45253	0.604777297	0.418391	0.003378	hypermethylated
cg13399544	0.42336	0.565720541	0.418205	0.001587	hypermethylated
cg09287629	0.41213	0.55070973	0.418193	4.05E-05	hypermethylated
cg16583552	0.52126	0.696471892	0.418062	0.008348	hypermethylated
cg27269962	0.52021	0.694871892	0.417653	2.10E-05	hypermethylated
cg21649013	0.35917	0.479717297	0.417518	0.000274	hypermethylated
cg09598437	0.39908	0.532873514	0.417115	0.030317	hypermethylated
cg08485158	0.56396	0.753007568	0.417072	0.000245	hypermethylated
cg23149064	0.3338	0.445660541	0.416961	5.97E-06	hypermethylated
cg11696388	0.3996	0.533488108	0.4169	0.002231	hypermethylated
cg16535788	0.57299	0.764947568	0.416851	0.00099	hypermethylated
cg17437640	0.35376	0.472201081	0.41663	0.0003	hypermethylated
cg18087477	0.4739	0.63253027	0.416552	0.000185	hypermethylated
cg11054816	0.51943	0.693242703	0.416431	0.016836	hypermethylated
cg15662251	0.3729	0.497628649	0.416281	0.000464	hypermethylated
cg03200571	0.56287	0.75113027	0.416261	0.008069	hypermethylated
cg13336665	0.45622	0.608756216	0.416135	0.000893	hypermethylated
cg03585720	0.61621	0.821939459	0.41561	0.001987	hypermethylated
cg06898168	0.45748	0.610198919	0.415571	1.01E-05	hypermethylated
cg02556634	0.3841	0.512269189	0.41542	0.000523	hypermethylated
cg06475633	0.58625	0.781784865	0.415256	0.009877	hypermethylated
cg14078730	0.50165	0.668934595	0.415184	0.002274	hypermethylated
cg03295417	0.50561	0.67408973	0.414916	0.000242	hypermethylated
cg03321020	0.37472	0.499581081	0.414906	0.000187	hypermethylated
cg19895185	0.53452	0.712598378	0.414845	0.000313	hypermethylated
cg08422054	0.50477	0.67290973	0.414787	0.003255	hypermethylated
cg05768047	0.53417	0.712083784	0.414748	0.001603	hypermethylated
cg06837568	0.5122	0.682755676	0.414662	0.000209	hypermethylated
cg12699647	0.61921	0.825367568	0.414608	0.000277	hypermethylated
cg04214370	0.67207	0.895769189	0.414516	0.005706	hypermethylated
cg00366531	0.41479	0.552847568	0.414501	1.84E-05	hypermethylated
cg23992403	0.66433	0.885307568	0.414279	0.003637	hypermethylated
cg05476956	0.38055	0.507094595	0.414169	0.000226	hypermethylated
cg02306798	0.6114	0.81464973	0.414063	0.000209	hypermethylated

cg04913803	0.45167	0.601804865	0.414027	0.004287	hypermethylated
cg00162806	0.44744	0.596150811	0.413983	0.005223	hypermethylated
cg04682699	0.47357	0.630949189	0.413946	0.014604	hypermethylated
cg26345105	0.45631	0.607943784	0.413924	7.84E-05	hypermethylated
cg08615333	0.46938	0.625336216	0.413876	0.017781	hypermethylated
cg17527819	0.49861	0.664014595	0.413303	4.12E-06	hypermethylated
cg01615333	0.3445	0.458707027	0.413069	0.00017	hypermethylated
cg13840445	0.46028	0.612808649	0.412925	0.000327	hypermethylated
cg25985013	0.36933	0.491687027	0.41283	0.001768	hypermethylated
cg21855816	0.58923	0.784297838	0.412571	0.000875	hypermethylated
cg17074989	0.57764	0.768852432	0.412536	0.003136	hypermethylated
cg14737994	0.49437	0.657995676	0.412487	0.000495	hypermethylated
cg05785989	0.47018	0.625721081	0.412307	2.92E-05	hypermethylated
cg01618923	0.51695	0.687872973	0.412117	0.012638	hypermethylated
cg21221690	0.50692	0.674494595	0.412049	0.010211	hypermethylated
cg11685223	0.38908	0.517643243	0.411891	0.008636	hypermethylated
cg17181598	0.68561	0.912155135	0.411891	0.00193	hypermethylated
cg23889772	0.34323	0.456603784	0.411767	0.001085	hypermethylated
cg22506548	0.57494	0.764848108	0.411762	0.0017	hypermethylated
cg17804611	0.50504	0.671788649	0.41161	0.002274	hypermethylated
cg12524640	0.41721	0.554926486	0.411523	0.003021	hypermethylated
cg16170346	0.44258	0.588652973	0.411479	0.000756	hypermethylated
cg08498179	0.64478	0.857574595	0.411455	0.001275	hypermethylated
cg05134019	0.6103	0.811698919	0.411426	0.007666	hypermethylated
cg27338807	0.55665	0.740322703	0.411384	0.004526	hypermethylated
cg11197630	0.53139	0.706657838	0.411241	0.000557	hypermethylated
cg09895822	0.55981	0.744338919	0.411022	0.000327	hypermethylated
cg01881549	0.52837	0.70250973	0.41097	0.019361	hypermethylated
cg11438039	0.55623	0.739507027	0.410882	0.000469	hypermethylated
cg14691596	0.40743	0.541608108	0.410697	0.000132	hypermethylated
cg26109568	0.62498	0.830723243	0.410558	0.003316	hypermethylated
cg08149747	0.58167	0.773145405	0.410539	0.004566	hypermethylated
cg04910970	0.42461	0.564356216	0.410468	0.00012	hypermethylated
cg24401487	0.50085	0.665678919	0.410448	0.000104	hypermethylated
cg01684248	0.38772	0.515295135	0.410384	0.000575	hypermethylated
cg16805094	0.58142	0.772728108	0.41038	8.63E-05	hypermethylated
cg17166338	0.53127	0.706021081	0.410266	0.001511	hypermethylated
cg04330881	0.52931	0.703366486	0.410164	0.000464	hypermethylated
cg08238265	0.64308	0.854419459	0.409946	0.002478	hypermethylated
cg08200869	0.40726	0.54108	0.409892	0.00012	hypermethylated
cg11160362	0.32957	0.437805946	0.409707	2.61E-05	hypermethylated
cg18237616	0.57268	0.760645405	0.409495	0.035497	hypermethylated
cg03111114	0.36136	0.479963784	0.409489	0.000653	hypermethylated
cg08473752	0.59588	0.791416216	0.409415	0.000145	hypermethylated
cg15194163	0.35332	0.469255676	0.409399	3.39E-06	hypermethylated
cg15209808	0.45055	0.59834973	0.409302	9.49E-05	hypermethylated
cg24607283	0.5008	0.665045946	0.409219	0.000178	hypermethylated
cg13876553	0.60907	0.80877027	0.409122	0.017101	hypermethylated
cg19542346	0.44368	0.589128649	0.409063	0.000283	hypermethylated
cg27460824	0.63392	0.841680541	0.408972	0.000202	hypermethylated
cg00058923	0.632	0.839112973	0.408941	0.00098	hypermethylated
cg07022343	0.40644	0.539594595	0.408834	0.016836	hypermethylated
cg00744431	0.39456	0.523803784	0.408782	0.000653	hypermethylated
cg21251230	0.51879	0.688725405	0.408778	0.016444	hypermethylated
cg16685313	0.58436	0.775657838	0.408563	0.002086	hypermethylated
cg00985983	0.44139	0.58582	0.408404	0.00341	hypermethylated
cg14990413	0.47167	0.625902162	0.408159	0.008348	hypermethylated
cg20310894	0.44057	0.584601081	0.408081	0.000117	hypermethylated

cg19788941	0.41886	0.555759459	0.407992	0.00255	hypermethylated
cg16700555	0.59473	0.789023243	0.407833	0.007799	hypermethylated
cg13583664	0.46918	0.622390811	0.407679	0.02988	hypermethylated
cg12475507	0.40407	0.535958919	0.407517	9.95E-05	hypermethylated
cg23164076	0.51108	0.677847027	0.407411	0.000449	hypermethylated
cg17113147	0.59758	0.79252973	0.407333	0.009315	hypermethylated
cg03608520	0.38465	0.510131351	0.407322	0.002341	hypermethylated
cg17744883	0.45305	0.600835676	0.4073	0.00504	hypermethylated
cg17592667	0.60502	0.802361081	0.407269	0.004691	hypermethylated
cg24925945	0.63261	0.838914054	0.407207	0.000204	hypermethylated
cg07139350	0.62839	0.83331027	0.407194	0.001603	hypermethylated
cg21390082	0.51093	0.677530811	0.407161	0.00134	hypermethylated
cg21970789	0.36462	0.483384324	0.406777	6.62E-05	hypermethylated
cg00704780	0.48946	0.648803784	0.406591	0.000464	hypermethylated
cg13153708	0.54571	0.723296757	0.406453	0.008208	hypermethylated
cg10393227	0.29961	0.397105946	0.406438	0.000134	hypermethylated
cg21245492	0.66915	0.88688	0.406409	0.000268	hypermethylated
cg00676030	0.48575	0.643711351	0.4062	0.008491	hypermethylated
cg12859429	0.52276	0.692712973	0.406109	0.000123	hypermethylated
cg09491120	0.55306	0.732809189	0.406002	0.00017	hypermethylated
cg07861790	0.48874	0.647475676	0.405759	7.12E-05	hypermethylated
cg04453594	0.33053	0.437829189	0.405587	5.79E-05	hypermethylated
cg18171392	0.38659	0.512076216	0.405554	9.05E-05	hypermethylated
cg05529123	0.52694	0.697960541	0.405507	0.004445	hypermethylated
cg12339328	0.63567	0.841961622	0.405476	3.95E-05	hypermethylated
cg26587870	0.59124	0.783112432	0.405476	0.000884	hypermethylated
cg00318436	0.53627	0.710244324	0.405356	0.000797	hypermethylated
cg19252741	0.57469	0.761123243	0.405346	0.006451	hypermethylated
cg18374393	0.64525	0.854465405	0.405164	0.000365	hypermethylated
cg04617640	0.4623	0.612188108	0.405146	0.004863	hypermethylated
cg02041497	0.35897	0.475310811	0.405008	0.010126	hypermethylated
cg03624135	0.3508	0.46442973	0.404812	9.72E-05	hypermethylated
cg18543610	0.41612	0.550805946	0.404545	0.000148	hypermethylated
cg25886621	0.56746	0.750928649	0.404157	0.010642	hypermethylated
cg06072036	0.53128	0.702856757	0.403758	0.000256	hypermethylated
cg01942816	0.47232	0.624746486	0.403506	0.024491	hypermethylated
cg19884223	0.36303	0.480139459	0.403365	0.001262	hypermethylated
cg15073906	0.56043	0.741144865	0.403221	0.046225	hypermethylated
cg20100801	0.5767	0.762567027	0.403043	0.004691	hypermethylated
cg02478023	0.4879	0.64498973	0.402691	0.000119	hypermethylated
cg11076814	0.47001	0.621208108	0.402385	0.012134	hypermethylated
cg20704654	0.6057	0.800497838	0.402294	0.001031	hypermethylated
cg14925024	0.51931	0.68630973	0.402264	0.005606	hypermethylated
cg10857341	0.568	0.750655676	0.40226	0.002751	hypermethylated
cg04095339	0.40714	0.537923243	0.401875	8.84E-05	hypermethylated
cg01069965	0.5674	0.749641081	0.401834	0.00054	hypermethylated
cg01124922	0.4302	0.568374054	0.401833	0.003136	hypermethylated
cg24002183	0.46241	0.610927568	0.401829	0.014838	hypermethylated
cg17132535	0.59488	0.785853514	0.401662	0.000287	hypermethylated
cg09880291	0.56081	0.740821622	0.401614	0.009238	hypermethylated
cg01075852	0.44386	0.586285405	0.401498	0.008069	hypermethylated
cg16854606	0.53782	0.710358378	0.401424	0.000251	hypermethylated
cg11743000	0.53085	0.70115027	0.401419	0.001423	hypermethylated
cg08751950	0.59843	0.790352432	0.401314	0.001635	hypermethylated
cg21386766	0.59455	0.785224865	0.401308	0.000187	hypermethylated
cg04944853	0.58386	0.771044865	0.401192	0.005411	hypermethylated
cg20018563	0.50062	0.661012973	0.400963	0.00039	hypermethylated
cg05797615	0.58014	0.765968649	0.400884	0.000902	hypermethylated

cg12985235	0.6729	0.888359459	0.400751	8.42E-05	hypermethylated
cg06850159	0.52428	0.692144865	0.400737	0.000327	hypermethylated
cg18422423	0.69759	0.920626486	0.400237	0.000234	hypermethylated
cg17517480	0.63062	0.83224	0.400229	0.001987	hypermethylated
cg02501166	0.54983	0.725612973	0.400215	0.009394	hypermethylated
cg17419731	0.60197	0.794354054	0.400091	0.000306	hypermethylated
cg12230883	0.61589	0.812619459	0.399907	0.004059	hypermethylated
cg08500200	0.53073	0.700227568	0.399846	0.004134	hypermethylated
cg15364355	0.55018	0.725876216	0.39982	0.00095	hypermethylated
cg06504162	0.56509	0.745483784	0.399696	3.49E-05	hypermethylated
cg17494585	0.60753	0.801267027	0.399327	0.000756	hypermethylated
cg22885014	0.42638	0.562317297	0.399245	0.000358	hypermethylated
cg03345668	0.48181	0.635386486	0.39917	6.78E-05	hypermethylated
cg01923020	0.56501	0.745035135	0.399032	0.000551	hypermethylated
cg23663318	0.58514	0.771550811	0.398979	0.000626	hypermethylated
cg12657297	0.47168	0.621756757	0.398542	0.001556	hypermethylated
cg07922933	0.49589	0.65362973	0.398453	0.002699	hypermethylated
cg22700015	0.60276	0.79445027	0.398373	0.000756	hypermethylated
cg10303978	0.62759	0.827037838	0.398131	0.009552	hypermethylated
cg10120807	0.69357	0.913923243	0.398032	0.000969	hypermethylated
cg16137772	0.61218	0.806662703	0.39801	0.006121	hypermethylated
cg23390118	0.52454	0.691162162	0.397971	7.93E-05	hypermethylated
cg13718664	0.57547	0.758255676	0.397944	0.003637	hypermethylated
cg09717809	0.70078	0.923299459	0.397837	0.001262	hypermethylated
cg00786909	0.65132	0.858062703	0.397717	0.004171	hypermethylated
cg00733328	0.53715	0.707572432	0.397553	5.58E-05	hypermethylated
cg24729221	0.47536	0.62616	0.397511	0.000127	hypermethylated
cg06616014	0.61276	0.806848108	0.396975	0.001225	hypermethylated
cg00461236	0.5631	0.741339459	0.396743	0.000251	hypermethylated
cg23646375	0.55819	0.734859459	0.396712	0.002363	hypermethylated
cg21610839	0.57038	0.750850811	0.396603	5.86E-05	hypermethylated
cg26205751	0.68985	0.908097838	0.396565	0.001031	hypermethylated
cg18180407	0.57333	0.754707568	0.396552	0.007933	hypermethylated
cg07037852	0.66182	0.871188649	0.396546	0.002803	hypermethylated
cg17098850	0.49136	0.646679459	0.39627	0.000772	hypermethylated
cg02236949	0.66285	0.872343784	0.396214	0.003538	hypermethylated
cg13327384	0.54863	0.721903784	0.395973	0.004248	hypermethylated
cg02332936	0.60389	0.794567568	0.395884	0.010999	hypermethylated
cg08893109	0.5851	0.769807568	0.395815	0.021549	hypermethylated
cg11401784	0.45029	0.592423243	0.395774	0.002455	hypermethylated
cg22810684	0.43592	0.573487027	0.395697	0.004951	hypermethylated
cg00808170	0.38784	0.510207027	0.395621	0.000313	hypermethylated
cg07125166	0.63258	0.83211027	0.395527	0.001603	hypermethylated
cg08758887	0.62816	0.826259459	0.395463	0.000633	hypermethylated
cg18861112	0.54817	0.720978378	0.395333	0.021548	hypermethylated
cg03226844	0.59676	0.784817297	0.395206	0.002455	hypermethylated
cg24824686	0.56095	0.737680541	0.395124	9.27E-05	hypermethylated
cg04772968	0.56806	0.747028649	0.39512	0.025045	hypermethylated
cg05037270	0.65838	0.865362703	0.394384	0.000306	hypermethylated
cg14757311	0.53245	0.699792432	0.394281	0.001968	hypermethylated
cg12405139	0.52949	0.695867027	0.394208	0.001857	hypermethylated
cg25157472	0.50977	0.669838919	0.393968	4.31E-05	hypermethylated
cg03169170	0.62262	0.818071351	0.393875	0.000395	hypermethylated
cg16051228	0.37392	0.491272973	0.393795	0.02561	hypermethylated
cg13851904	0.4004	0.525980541	0.393567	1.30E-05	hypermethylated
cg04387952	0.57198	0.751108108	0.393056	0.000506	hypermethylated
cg16997642	0.59183	0.777114595	0.392945	0.006915	hypermethylated
cg26856475	0.46177	0.606179459	0.392571	0.000464	hypermethylated

cg03472349	0.60673	0.796321622	0.392297	0.01348	hypermethylated
cg07157230	0.53088	0.696688649	0.392128	0.002502	hypermethylated
cg06868026	0.5534	0.726068108	0.391782	0.002363	hypermethylated
cg00822361	0.50401	0.661249189	0.391742	0.030537	hypermethylated
cg14658493	0.55789	0.731807568	0.391484	0.010381	hypermethylated
cg20392615	0.48921	0.641627027	0.391281	0.005807	hypermethylated
cg22966391	0.58984	0.773538919	0.39115	7.74E-05	hypermethylated
cg11631547	0.60209	0.789554595	0.39106	0.019065	hypermethylated
cg24914884	0.59976	0.786493514	0.39105	0.002409	hypermethylated
cg21722795	0.48856	0.640600541	0.390889	0.002599	hypermethylated
cg18596689	0.57802	0.75786	0.390812	0.001164	hypermethylated
cg23251141	0.35885	0.470472973	0.390731	0.001188	hypermethylated
cg15738933	0.42251	0.553904865	0.390653	0.000613	hypermethylated
cg05670717	0.49464	0.648354595	0.390404	0.001651	hypermethylated
cg27434368	0.40236	0.527381081	0.390359	0.000335	hypermethylated
cg14550760	0.34506	0.45227027	0.390338	0.002026	hypermethylated
cg04034583	0.38557	0.505364865	0.390333	2.10E-05	hypermethylated
cg21625737	0.65705	0.861174595	0.390303	0.002066	hypermethylated
cg22564695	0.58112	0.761532432	0.390069	0.001096	hypermethylated
cg20673721	0.5835	0.764574054	0.389924	0.000155	hypermethylated
cg19628619	0.53547	0.70161027	0.389864	0.013481	hypermethylated
cg11028624	0.59399	0.77824973	0.389795	0.001188	hypermethylated
cg04566826	0.49673	0.650817297	0.389791	0.001875	hypermethylated
cg24004943	0.63062	0.826228108	0.389769	0.000234	hypermethylated
cg01025774	0.44397	0.581674595	0.38975	0.00102	hypermethylated
cg23524354	0.49874	0.653360541	0.389591	0.005316	hypermethylated
cg09268961	0.54293	0.711128108	0.389343	0.001786	hypermethylated
cg09173348	0.51924	0.68008	0.389303	0.002803	hypermethylated
cg18550381	0.59099	0.774035135	0.389265	0.003878	hypermethylated
cg16435923	0.44807	0.586838378	0.389239	2.71E-05	hypermethylated
cg05185738	0.67855	0.888518378	0.388946	0.00395	hypermethylated
cg09293718	0.49714	0.650968649	0.388936	0.006451	hypermethylated
cg05404005	0.37723	0.493941622	0.388896	0.00291	hypermethylated
cg27645345	0.4286	0.56118973	0.388857	0.000866	hypermethylated
cg25748357	0.60266	0.789085946	0.388838	0.000588	hypermethylated
cg16703135	0.49401	0.646818378	0.38882	0.000115	hypermethylated
cg16679297	0.58422	0.764905946	0.388771	0.00039	hypermethylated
cg03243768	0.6787	0.888594054	0.38875	0.00017	hypermethylated
cg21115346	0.63788	0.835051892	0.388581	0.003316	hypermethylated
cg18858331	0.37194	0.48688973	0.388525	8.63E-05	hypermethylated
cg17040483	0.68596	0.897924865	0.38847	0.002189	hypermethylated
cg21860560	0.3697	0.483916757	0.388404	6.46E-05	hypermethylated
cg16770832	0.57471	0.752251351	0.388381	0.001541	hypermethylated
cg06501366	0.42163	0.551848108	0.388294	0.000626	hypermethylated
cg24948406	0.60295	0.789097297	0.388165	0.002751	hypermethylated
cg05813673	0.54486	0.713053514	0.388125	5.65E-05	hypermethylated
cg11718868	0.63197	0.826990811	0.388015	0.001237	hypermethylated
cg05749855	0.71129	0.930754054	0.387962	0.000848	hypermethylated
cg26710891	0.56286	0.736401081	0.387716	0.019065	hypermethylated
cg20011134	0.51446	0.672890811	0.387314	0.001096	hypermethylated
cg09748975	0.53965	0.705828108	0.387293	3.15E-05	hypermethylated
cg25821781	0.62481	0.817194054	0.387261	0.002502	hypermethylated
cg02247178	0.46765	0.611566486	0.38708	0.001912	hypermethylated
cg20585830	0.59554	0.778794595	0.387044	0.000495	hypermethylated
cg05083202	0.63569	0.831103784	0.386705	0.002066	hypermethylated
cg17623793	0.4932	0.644770811	0.386614	0.008348	hypermethylated
cg09830455	0.58567	0.765638378	0.386575	0.001875	hypermethylated
cg00573504	0.6724	0.878824865	0.386256	0.00071	hypermethylated

cg21220462	0.57892	0.756596757	0.386161	0.001096	hypermethylated
cg12526197	0.58728	0.767484324	0.386089	0.000159	hypermethylated
cg03978579	0.39027	0.509987027	0.385988	3.71E-05	hypermethylated
cg07177011	0.60206	0.786614595	0.38575	0.000412	hypermethylated
cg06535156	0.41488	0.542031892	0.385684	0.000204	hypermethylated
cg02289754	0.62017	0.810199459	0.385613	9.95E-05	hypermethylated
cg07052737	0.58546	0.764745946	0.38541	9.95E-05	hypermethylated
cg04521333	0.37067	0.48416	0.385349	9.19E-06	hypermethylated
cg05453358	0.64913	0.847662703	0.384983	0.002341	hypermethylated
cg02569509	0.63317	0.826674054	0.384726	0.000104	hypermethylated
cg11693848	0.42261	0.551698378	0.384553	0.000123	hypermethylated
cg22033530	0.5004	0.653095135	0.384211	0.000575	hypermethylated
cg14833621	0.53531	0.698590811	0.384073	0.012234	hypermethylated
cg15215348	0.55649	0.726142703	0.383897	0.00032	hypermethylated
cg02345417	0.6151	0.802597838	0.383856	0.000805	hypermethylated
cg26393977	0.58712	0.766070811	0.383822	0.001074	hypermethylated
cg08307753	0.43543	0.56804	0.383552	0.002066	hypermethylated
cg05414442	0.39091	0.509960541	0.383549	0.000382	hypermethylated
cg18501001	0.60618	0.790727027	0.383433	0.000365	hypermethylated
cg04765483	0.43769	0.570931892	0.383409	0.009713	hypermethylated
cg08003887	0.56138	0.732187027	0.383235	0.009552	hypermethylated
cg23372544	0.40207	0.524392973	0.383202	0.000741	hypermethylated
cg03070262	0.4213	0.549455676	0.383155	0.000112	hypermethylated
cg12847240	0.39919	0.520522703	0.382886	3.67E-05	hypermethylated
cg14244013	0.45865	0.597928649	0.38258	0.001188	hypermethylated
cg14940444	0.5937	0.773941622	0.382491	0.027573	hypermethylated
cg15777781	0.51473	0.670968649	0.382429	0.009552	hypermethylated
cg10498703	0.61929	0.807240541	0.382383	0.002856	hypermethylated
cg08819647	0.33492	0.436512432	0.382206	0.000293	hypermethylated
cg07039560	0.4529	0.590234595	0.382096	0.001314	hypermethylated
cg23035024	0.54538	0.71074973	0.38208	0.011274	hypermethylated
cg20208394	0.54163	0.705784324	0.38192	0.004485	hypermethylated
cg01432945	0.72193	0.940701622	0.381878	0.006229	hypermethylated
cg12927617	0.53881	0.702052432	0.381802	0.010211	hypermethylated
cg26030804	0.65395	0.852020541	0.381708	0.004566	hypermethylated
cg05239811	0.49272	0.641915676	0.381616	0.003286	hypermethylated
cg25657261	0.40281	0.524765946	0.381575	9.82E-06	hypermethylated
cg07914200	0.57086	0.743675135	0.381536	0.00032	hypermethylated
cg16907527	0.53223	0.693318378	0.381468	0.02324	hypermethylated
cg06720544	0.61806	0.804906486	0.381074	0.012948	hypermethylated
cg00282426	0.61704	0.80354973	0.381023	0.012333	hypermethylated
cg07049592	0.45318	0.590122162	0.380929	0.005131	hypermethylated
cg23201938	0.62089	0.80838	0.380696	0.000454	hypermethylated
cg07096883	0.66051	0.85987027	0.380539	0.0003	hypermethylated
cg07827420	0.43215	0.562579459	0.380525	0.000209	hypermethylated
cg00165994	0.49903	0.64964	0.380514	0.003843	hypermethylated
cg17897626	0.51857	0.67502	0.380392	0.000182	hypermethylated
cg00581154	0.54929	0.714913514	0.380201	0.000454	hypermethylated
cg16318253	0.58263	0.758302162	0.380193	0.002502	hypermethylated
cg02030410	0.60205	0.783323784	0.379725	0.000209	hypermethylated
cg19223129	0.59657	0.776164324	0.379671	0.00504	hypermethylated
cg15685144	0.40956	0.532823243	0.379582	0.000166	hypermethylated
cg19748485	0.38227	0.497182703	0.379184	0.000174	hypermethylated
cg11858450	0.54015	0.702507027	0.379153	0.004776	hypermethylated
cg22953960	0.54456	0.708164324	0.378993	8.03E-05	hypermethylated
cg06679270	0.58021	0.754494595	0.378935	1.21E-05	hypermethylated
cg15788059	0.71307	0.927186486	0.378816	0.001525	hypermethylated
cg08769073	0.6634	0.862557297	0.378741	0.001556	hypermethylated

cg14908170	0.59701	0.77615027	0.378581	0.000342	hypermethylated
cg09823859	0.57295	0.74484	0.378521	0.000528	hypermethylated
cg12593515	0.48019	0.624234595	0.378483	0.00039	hypermethylated
cg16297171	0.52966	0.688482703	0.378354	0.000102	hypermethylated
cg04252204	0.60833	0.790695676	0.378268	0.000256	hypermethylated
cg24512644	0.5251	0.682378378	0.37798	0.016574	hypermethylated
cg11061946	0.56791	0.737923784	0.37781	0.043764	hypermethylated
cg06127801	0.55191	0.71711027	0.377762	0.007035	hypermethylated
cg09829869	0.50896	0.66128973	0.37773	0.005269	hypermethylated
cg03376871	0.49173	0.638871351	0.377659	0.002318	hypermethylated
cg06294376	0.61069	0.793374054	0.377561	0.002189	hypermethylated
cg23043937	0.52686	0.684452432	0.377531	0.010042	hypermethylated
cg26417985	0.62158	0.807464324	0.377458	0.000607	hypermethylated
cg10162251	0.66601	0.865157297	0.377419	0.002856	hypermethylated
cg27490391	0.5197	0.675014595	0.37724	0.001481	hypermethylated
cg27664496	0.45446	0.590171892	0.376982	0.000229	hypermethylated
cg04220881	0.66364	0.861748649	0.376866	0.000495	hypermethylated
cg02879159	0.50681	0.658054595	0.376762	0.00054	hypermethylated
cg19797304	0.69495	0.902313514	0.37672	0.001262	hypermethylated
cg09074625	0.40686	0.52824973	0.376688	0.000129	hypermethylated
cg04237666	0.53236	0.691164324	0.376627	0.019661	hypermethylated
cg07815836	0.4018	0.521652432	0.376611	0.001511	hypermethylated
cg04494197	0.63213	0.820655676	0.376556	0.000123	hypermethylated
cg07325001	0.41174	0.534527568	0.376531	0.014032	hypermethylated
cg05260031	0.68901	0.894399459	0.376394	0.00102	hypermethylated
cg12159995	0.61209	0.794456216	0.376224	0.001074	hypermethylated
cg14055887	0.46906	0.608712973	0.37599	0.001288	hypermethylated
cg11005628	0.41499	0.538518378	0.375919	0.000563	hypermethylated
cg19542707	0.43262	0.561321081	0.375726	4.53E-05	hypermethylated
cg13523818	0.3841	0.498361622	0.375711	5.65E-05	hypermethylated
cg03786842	0.60894	0.790050811	0.375645	0.001481	hypermethylated
cg15874877	0.47142	0.611604324	0.375586	0.000327	hypermethylated
cg10897223	0.47132	0.611456757	0.375544	0.000696	hypermethylated
cg20421482	0.55755	0.723124865	0.375144	0.007732	hypermethylated
cg20449726	0.47482	0.61582	0.375128	0.000805	hypermethylated
cg03199745	0.51259	0.664789189	0.375092	5.14E-06	hypermethylated
cg17311022	0.4893	0.634572432	0.375066	0.002253	hypermethylated
cg15159588	0.59021	0.765427568	0.375037	0.003739	hypermethylated
cg04522596	0.58522	0.758841622	0.37482	0.002026	hypermethylated
cg16508028	0.57356	0.743715676	0.374807	0.001395	hypermethylated
cg13884295	0.58928	0.764050811	0.374715	0.000306	hypermethylated
cg25690589	0.50156	0.650308108	0.374701	0.001857	hypermethylated
cg06724693	0.46303	0.600250811	0.37446	0.000756	hypermethylated
cg17676129	0.36118	0.46820973	0.374437	0.000545	hypermethylated
cg17838516	0.48227	0.625154595	0.374372	9.27E-05	hypermethylated
cg07735851	0.62726	0.813076216	0.374327	0.003136	hypermethylated
cg13799919	0.55639	0.721152432	0.374208	0.00062	hypermethylated
cg22959260	0.65467	0.848496757	0.374141	0.000912	hypermethylated
cg21853871	0.54948	0.712082703	0.373978	0.008348	hypermethylated
cg00748072	0.55437	0.718389189	0.373916	0.000155	hypermethylated
cg07806674	0.60408	0.782751892	0.373815	0.001409	hypermethylated
cg16517298	0.57882	0.749987568	0.373752	0.000189	hypermethylated
cg26529864	0.70812	0.91748	0.373683	0.000534	hypermethylated
cg25492363	0.72686	0.941707568	0.373602	0.001586	hypermethylated
cg09932747	0.65754	0.851871892	0.373558	0.00101	hypermethylated
cg03510605	0.44647	0.578418378	0.37355	0.000306	hypermethylated
cg24543937	0.41138	0.532928108	0.373469	0.00099	hypermethylated
cg17775899	0.39395	0.51034	0.373446	0.006737	hypermethylated

cg11903177	0.72891	0.944233514	0.373403	0.004566	hypermethylated
cg07022279	0.62262	0.806466486	0.373263	0.000495	hypermethylated
cg24304309	0.44432	0.575450811	0.373094	0.00054	hypermethylated
cg27373390	0.59564	0.771362703	0.372969	0.039172	hypermethylated
cg17391741	0.56405	0.730430811	0.372925	0.00095	hypermethylated
cg06120399	0.45604	0.590545946	0.372889	0.00094	hypermethylated
cg04107939	0.50099	0.648750811	0.372883	0.039723	hypermethylated
cg25522738	0.53025	0.686457297	0.372497	0.000613	hypermethylated
cg18368845	0.64431	0.834115135	0.372492	0.00039	hypermethylated
cg05423392	0.57904	0.749341081	0.37196	0.003078	hypermethylated
cg01596963	0.38774	0.501777838	0.371959	8.03E-05	hypermethylated
cg03379477	0.62701	0.811408649	0.37194	0.002026	hypermethylated
cg03467555	0.46266	0.5987	0.371881	0.000327	hypermethylated
cg11021940	0.35911	0.464698378	0.371869	0.000234	hypermethylated
cg17597901	0.41416	0.535932432	0.371863	5.51E-05	hypermethylated
cg09412882	0.53391	0.690884865	0.371849	0.004995	hypermethylated
cg03532040	0.40928	0.529482162	0.371494	0.001734	hypermethylated
cg17806437	0.72453	0.937314054	0.371487	0.001188	hypermethylated
cg04656171	0.51872	0.671048108	0.37146	0.0017	hypermethylated
cg03771840	0.48932	0.632986486	0.371396	0.000382	hypermethylated
cg20637688	0.59563	0.770447568	0.37128	0.0017	hypermethylated
cg12288076	0.43697	0.565176757	0.371168	0.000667	hypermethylated
cg00158122	0.4109	0.53144	0.371119	1.35E-05	hypermethylated
cg09083359	0.6285	0.812859459	0.371093	0.012036	hypermethylated
cg09370594	0.46024	0.595152973	0.370874	0.004951	hypermethylated
cg03392571	0.49665	0.642235135	0.370872	0.000588	hypermethylated
cg19628603	0.51925	0.671444865	0.37084	0.000124	hypermethylated
cg08761909	0.54715	0.707487568	0.370768	0.007035	hypermethylated
cg06876872	0.67763	0.876081081	0.370567	0.001423	hypermethylated
cg13928102	0.59856	0.773845405	0.37055	0.019065	hypermethylated
cg05672616	0.59504	0.769135676	0.370251	0.007408	hypermethylated
cg23204113	0.52095	0.673338919	0.370188	0.005223	hypermethylated
cg06711560	0.60838	0.786251892	0.370019	0.006175	hypermethylated
cg08396193	0.59501	0.768927027	0.369933	0.003571	hypermethylated
cg23467079	0.55181	0.712945405	0.36962	0.000117	hypermethylated
cg00920572	0.46698	0.603274054	0.369453	1.99E-05	hypermethylated
cg24053163	0.67983	0.878049189	0.369128	0.013158	hypermethylated
cg24751154	0.64654	0.834933514	0.368922	0.001262	hypermethylated
cg02918557	0.46956	0.606222162	0.368537	0.002066	hypermethylated
cg26297344	0.44722	0.577308649	0.368358	0.00035	hypermethylated
cg11914962	0.50719	0.654637838	0.368171	0.00504	hypermethylated
cg13914708	0.59504	0.767898378	0.367929	0.003538	hypermethylated
cg01511557	0.43493	0.561211351	0.367761	0.000103	hypermethylated
cg11045531	0.57372	0.74028	0.367724	0.023592	hypermethylated
cg23071286	0.66332	0.855845405	0.367645	0.00071	hypermethylated
cg20737554	0.65852	0.849317838	0.367077	0.00099	hypermethylated
cg24033471	0.50827	0.655498378	0.366997	0.00054	hypermethylated
cg18313790	0.4349	0.560847027	0.366924	2.64E-05	hypermethylated
cg14276286	0.62121	0.801108649	0.366917	0.02324	hypermethylated
cg24757553	0.47537	0.613010811	0.366862	0.000115	hypermethylated
cg13357416	0.6579	0.848311892	0.366726	0.003316	hypermethylated
cg27132535	0.45692	0.589067568	0.366492	0.0017	hypermethylated
cg14828404	0.60255	0.776781081	0.366427	0.007408	hypermethylated
cg10500503	0.69069	0.890346486	0.366329	0.005706	hypermethylated
cg10230442	0.61736	0.795805405	0.366304	0.005508	hypermethylated
cg08641514	0.64953	0.837270811	0.366298	0.001949	hypermethylated
cg06899044	0.4958	0.639100541	0.366285	9.49E-05	hypermethylated
cg01168289	0.55947	0.720984324	0.365907	0.006564	hypermethylated

cg13476204	0.63663	0.820322703	0.365736	0.004608	hypermethylated
cg21745307	0.69661	0.897574595	0.365681	0.00291	hypermethylated
cg00499237	0.48762	0.628226486	0.365527	0.004365	hypermethylated
cg02700360	0.60471	0.779050811	0.365474	0.01073	hypermethylated
cg25747190	0.62223	0.801440541	0.365148	0.000822	hypermethylated
cg17352045	0.54737	0.704806486	0.364711	0.000148	hypermethylated
cg07370496	0.47845	0.615998378	0.364558	0.000198	hypermethylated
cg02835462	0.62397	0.803316757	0.364492	0.000361	hypermethylated
cg05956452	0.61187	0.787723243	0.364464	0.000653	hypermethylated
cg26674966	0.67326	0.866735135	0.364427	0.005807	hypermethylated
cg20239391	0.51392	0.661541622	0.364288	0.002574	hypermethylated
cg14327228	0.43425	0.558846486	0.363926	0.004365	hypermethylated
cg04816311	0.65239	0.839563784	0.363905	0.000117	hypermethylated
cg14888296	0.60847	0.783030811	0.363883	0.011841	hypermethylated
cg02681842	0.59741	0.768795676	0.363879	0.003078	hypermethylated
cg05783455	0.3677	0.473163784	0.36381	8.32E-05	hypermethylated
cg23942526	0.72742	0.936048649	0.363795	0.000122	hypermethylated
cg24901662	0.59125	0.760807027	0.363762	0.003195	hypermethylated
cg16792230	0.65092	0.837556757	0.363707	0.000327	hypermethylated
cg23092040	0.43979	0.565829189	0.363552	0.000346	hypermethylated
cg03265074	0.41753	0.537174595	0.363511	0.000912	hypermethylated
cg22280820	0.37844	0.486835135	0.363369	9.49E-05	hypermethylated
cg07832006	0.54043	0.695218919	0.36336	0.003078	hypermethylated
cg09709600	0.62099	0.798835676	0.363329	3.23E-05	hypermethylated
cg00281664	0.57991	0.745955135	0.36326	0.002599	hypermethylated
cg21834684	0.6234	0.801803784	0.363091	0.001031	hypermethylated
cg27571196	0.43042	0.553555135	0.362982	3.85E-05	hypermethylated
cg02793099	0.47319	0.608556757	0.362972	6.84E-06	hypermethylated
cg00982535	0.70695	0.909161081	0.362928	0.001803	hypermethylated
cg06306260	0.63384	0.815087027	0.362835	0.009552	hypermethylated
cg18149207	0.51743	0.665006486	0.362005	0.004287	hypermethylated
cg06207120	0.52476	0.674364865	0.361872	0.000287	hypermethylated
cg25962358	0.48535	0.623557838	0.361498	8.84E-05	hypermethylated
cg17480641	0.52884	0.679402162	0.361435	0.00291	hypermethylated
cg26218977	0.37736	0.484767568	0.361352	4.70E-05	hypermethylated
cg14717656	0.70207	0.901834595	0.361248	0.004325	hypermethylated
cg01285862	0.65178	0.837028649	0.360892	0.008001	hypermethylated
cg27287438	0.70999	0.911756757	0.36085	0.000109	hypermethylated
cg02126424	0.62704	0.805185946	0.360765	0.002725	hypermethylated
cg09204108	0.56428	0.724582703	0.360739	0.001052	hypermethylated
cg01983105	0.62736	0.805555135	0.36069	0.016835	hypermethylated
cg17816173	0.59727	0.766912973	0.36068	0.003347	hypermethylated
cg07996393	0.65487	0.840789189	0.360536	0.002502	hypermethylated
cg15805451	0.59636	0.765667027	0.360534	0.001041	hypermethylated
cg04105760	0.36075	0.463138378	0.360444	3.85E-05	hypermethylated
cg22205276	0.58365	0.749228649	0.360303	0.016574	hypermethylated
cg06943925	0.53644	0.688494054	0.360027	0.042575	hypermethylated
cg01080927	0.68528	0.879431351	0.359877	0.003316	hypermethylated
cg07714276	0.4088	0.524612432	0.359857	0.000445	hypermethylated
cg24835238	0.53014	0.680292432	0.359782	0.01737	hypermethylated
cg12010198	0.4595	0.589643243	0.359777	7.74E-05	hypermethylated
cg23411518	0.42949	0.5509	0.359166	0.007799	hypermethylated
cg01038597	0.57125	0.732712432	0.359125	0.00151	hypermethylated
cg05985317	0.42042	0.539246486	0.359114	0.000152	hypermethylated
cg21222350	0.427	0.547664324	0.359056	0.026576	hypermethylated
cg17382918	0.52075	0.667870811	0.358978	0.000172	hypermethylated
cg07042546	0.52089	0.667987027	0.358841	0.010381	hypermethylated
cg23571839	0.69755	0.894523784	0.358823	0.004862	hypermethylated

cg07007754	0.63566	0.815141622	0.358795	0.004287	hypermethylated
cg16976499	0.60505	0.775794054	0.358619	0.000667	hypermethylated
cg04011497	0.6337	0.812506486	0.358579	0.002829	hypermethylated
cg25362652	0.46725	0.599071351	0.358533	0.000232	hypermethylated
cg25354617	0.65459	0.839235135	0.358484	0.002432	hypermethylated
cg17433998	0.62085	0.795959459	0.35845	0.006451	hypermethylated
cg24695890	0.43092	0.552323243	0.358093	0.000174	hypermethylated
cg04134748	0.68232	0.874392973	0.357833	0.000741	hypermethylated
cg25635000	0.64049	0.820784324	0.357827	0.004608	hypermethylated
cg02520281	0.47291	0.605997838	0.357747	0.002274	hypermethylated
cg09947844	0.6345	0.812989189	0.357616	0.003078	hypermethylated
cg03112869	0.69473	0.889880541	0.357159	0.003843	hypermethylated
cg15108047	0.6317	0.809116216	0.357107	0.000374	hypermethylated
cg27038101	0.53006	0.678912973	0.357071	0.002126	hypermethylated
cg16056219	0.52501	0.672378378	0.356928	0.005606	hypermethylated
cg12111137	0.54067	0.692434054	0.356928	0.002856	hypermethylated
cg18760534	0.65402	0.837577297	0.356888	0.002318	hypermethylated
cg20841748	0.47396	0.606941622	0.356792	0.000268	hypermethylated
cg20647257	0.48694	0.623457297	0.356547	0.001423	hypermethylated
cg16989443	0.68035	0.871023243	0.356434	0.001651	hypermethylated
cg09554406	0.63548	0.813561081	0.356404	3.57E-05	hypermethylated
cg06233593	0.57168	0.73184973	0.35634	0.019361	hypermethylated
cg26657675	0.46433	0.594397297	0.356277	0.001875	hypermethylated
cg25577090	0.59934	0.767188649	0.356207	0.00048	hypermethylated
cg24102242	0.62056	0.794286486	0.356089	0.000159	hypermethylated
cg00521703	0.41025	0.525091351	0.356065	7.47E-05	hypermethylated
cg01541600	0.50543	0.646891892	0.356013	0.000528	hypermethylated
cg25817701	0.6058	0.775342162	0.355992	0.002649	hypermethylated
cg01281231	0.58324	0.746396757	0.355853	0.002455	hypermethylated
cg21522988	0.60544	0.774718378	0.355688	0.001437	hypermethylated
cg09938408	0.57198	0.731891892	0.355666	0.003843	hypermethylated
cg14823825	0.63266	0.809494595	0.355591	0.001734	hypermethylated
cg06358218	0.5901	0.754956216	0.355434	0.005316	hypermethylated
cg07319253	0.68291	0.873529189	0.35516	0.003505	hypermethylated
cg04413148	0.61735	0.78962973	0.355088	0.007536	hypermethylated
cg00152948	0.65389	0.836217297	0.35483	0.000893	hypermethylated
cg24953703	0.55835	0.714015676	0.354786	0.006122	hypermethylated
cg00557959	0.66672	0.852527568	0.354665	0.000653	hypermethylated
cg00718444	0.67223	0.859417297	0.354404	0.000884	hypermethylated
cg20011248	0.51222	0.65484	0.354379	5.06E-05	hypermethylated
cg03442254	0.59073	0.755205405	0.35437	0.001041	hypermethylated
cg19256474	0.50829	0.649768649	0.354274	0.000293	hypermethylated
cg17509561	0.69793	0.892098378	0.35412	0.000163	hypermethylated
cg04183158	0.63615	0.813026486	0.353935	0.025992	hypermethylated
cg17980283	0.36923	0.47186973	0.353869	0.000115	hypermethylated
cg00992417	0.66262	0.846812973	0.353862	0.00591	hypermethylated
cg00431894	0.60534	0.773568108	0.353783	0.018485	hypermethylated
cg16110314	0.70513	0.901017838	0.353666	0.002363	hypermethylated
cg04228709	0.58609	0.748824865	0.353506	8.63E-05	hypermethylated
cg18074184	0.47736	0.609887568	0.353466	0.000122	hypermethylated
cg01281038	0.63171	0.807064324	0.353421	0.002318	hypermethylated
cg02625536	0.48756	0.622873514	0.353359	0.002274	hypermethylated
cg06015733	0.47224	0.603270811	0.353286	0.012036	hypermethylated
cg03275201	0.52463	0.670048108	0.352964	0.00097	hypermethylated
cg11898646	0.49957	0.638034054	0.352947	0.00513	hypermethylated
cg00955307	0.52262	0.667452973	0.352904	1.23E-05	hypermethylated
cg24937136	0.55619	0.710299459	0.35285	0.000464	hypermethylated
cg15313740	0.59003	0.753423243	0.352672	0.000495	hypermethylated

cg01328444	0.59807	0.763607568	0.352517	0.014145	hypermethylated
cg00853742	0.55562	0.709362162	0.352424	0.012535	hypermethylated
cg09053247	0.55201	0.704725946	0.352368	0.001096	hypermethylated
cg26978776	0.67628	0.863263243	0.35218	0.006679	hypermethylated
cg13809270	0.47454	0.605668649	0.351999	0.003773	hypermethylated
cg18225577	0.69062	0.881361081	0.351841	0.000445	hypermethylated
cg03988107	0.52607	0.671363243	0.351839	0.00134	hypermethylated
cg22241045	0.54474	0.695141081	0.351738	0.010211	hypermethylated
cg02350039	0.65441	0.834852432	0.351326	0.027371	hypermethylated
cg16234490	0.52878	0.674540541	0.351238	0.00193	hypermethylated
cg00199065	0.65339	0.833405405	0.351074	0.001667	hypermethylated
cg01033419	0.63558	0.810420541	0.350597	0.000408	hypermethylated
cg13458253	0.50396	0.642574595	0.350555	0.002147	hypermethylated
cg08679971	0.54087	0.689503784	0.350277	0.002478	hypermethylated
cg02765177	0.66543	0.848197297	0.350113	0.00221	hypermethylated
cg10548254	0.52695	0.671641622	0.350026	0.002502	hypermethylated
cg13606990	0.48737	0.621171351	0.349974	0.003378	hypermethylated
cg17954142	0.62837	0.800638378	0.349536	0.000293	hypermethylated
cg18524262	0.66627	0.848875135	0.349445	0.001153	hypermethylated
cg04507915	0.65131	0.8298	0.349419	0.000195	hypermethylated
cg14532376	0.61802	0.787230811	0.349133	0.000195	hypermethylated
cg05333146	0.52065	0.663125946	0.348969	0.00039	hypermethylated
cg25528121	0.65015	0.828051892	0.348949	0.001237	hypermethylated
cg09501959	0.53944	0.686986486	0.348819	0.00095	hypermethylated
cg07714085	0.50723	0.645884865	0.348637	0.000251	hypermethylated
cg13609939	0.59982	0.763775676	0.348619	0.009394	hypermethylated
cg17863312	0.56897	0.724404324	0.348443	0.002318	hypermethylated
cg12188410	0.57792	0.735723243	0.348293	0.036003	hypermethylated
cg16445423	0.53967	0.686972973	0.348176	0.003986	hypermethylated
cg12781568	0.59171	0.753174054	0.348093	0.000129	hypermethylated
cg08094235	0.69544	0.885191892	0.348064	0.000104	hypermethylated
cg23586440	0.64755	0.82414973	0.347915	0.03076	hypermethylated
cg24079790	0.67504	0.859104865	0.347861	0.003136	hypermethylated
cg11659652	0.65261	0.830531351	0.347814	4.53E-05	hypermethylated
cg03291548	0.60227	0.766325946	0.347548	4.47E-05	hypermethylated
cg22908000	0.66471	0.845684865	0.347395	0.004863	hypermethylated
cg21946698	0.5826	0.741175135	0.347309	0.009795	hypermethylated
cg07589235	0.56034	0.712825405	0.347246	0.004691	hypermethylated
cg23348372	0.67705	0.861272432	0.347207	0.00101	hypermethylated
cg07026560	0.60511	0.769727027	0.347149	0.002455	hypermethylated
cg01722932	0.40446	0.514454595	0.347047	1.01E-05	hypermethylated
cg09941143	0.57946	0.737041081	0.347036	0.010999	hypermethylated
cg11888359	0.72316	0.919696757	0.346843	0.001367	hypermethylated
cg14611258	0.55804	0.709681081	0.346802	0.034997	hypermethylated
cg01845451	0.5847	0.743548649	0.346731	0.00039	hypermethylated
cg01872947	0.55949	0.711458919	0.346668	0.000182	hypermethylated
cg23245711	0.56401	0.717146486	0.346547	0.003107	hypermethylated
cg20680163	0.59296	0.753938919	0.346513	0.001395	hypermethylated
cg08149459	0.65055	0.827124324	0.346444	0.00024	hypermethylated
cg15445725	0.57873	0.735776216	0.346377	0.007408	hypermethylated
cg15122993	0.61536	0.782318919	0.346326	6.62E-05	hypermethylated
cg20209499	0.64015	0.813795135	0.346256	0.000875	hypermethylated
cg04889069	0.46509	0.591245946	0.346248	0.000445	hypermethylated
cg23371436	0.50494	0.641889189	0.346212	0.000588	hypermethylated
cg19961522	0.57471	0.73056973	0.346188	0.008069	hypermethylated
cg27593537	0.5047	0.641561622	0.346162	0.000613	hypermethylated
cg18449426	0.62636	0.796082703	0.345926	0.006679	hypermethylated
cg23077820	0.55329	0.703197297	0.345894	4.26E-05	hypermethylated

cg11599526	0.5946	0.755674595	0.345846	0.000214	hypermethylated
cg02074714	0.38496	0.489219459	0.345773	0.003773	hypermethylated
cg10577819	0.52139	0.662583243	0.345739	0.008069	hypermethylated
cg02161292	0.54956	0.698358919	0.345692	0.000157	hypermethylated
cg18634516	0.58133	0.738722703	0.345676	0.007799	hypermethylated
cg20296493	0.46965	0.596721081	0.345471	0.000204	hypermethylated
cg24687655	0.6128	0.778574595	0.345419	0.000123	hypermethylated
cg19815574	0.42773	0.543395676	0.345303	9.05E-05	hypermethylated
cg09169516	0.65532	0.832139459	0.344626	0.01737	hypermethylated
cg14317414	0.56577	0.718374595	0.344521	0.003843	hypermethylated
cg26980034	0.55088	0.699441081	0.344464	0.00029	hypermethylated
cg16365445	0.54495	0.691865946	0.344369	0.010819	hypermethylated
cg20834681	0.44834	0.569164865	0.344253	0.000822	hypermethylated
cg12211161	0.55083	0.699255676	0.344213	0.004485	hypermethylated
cg01624637	0.54913	0.697085946	0.344189	0.008069	hypermethylated
cg06431953	0.67274	0.85392	0.344052	0.010819	hypermethylated
cg25722041	0.58264	0.73936973	0.343691	0.00012	hypermethylated
cg18105665	0.68402	0.867998919	0.343655	0.011554	hypermethylated
cg26920808	0.64791	0.822149189	0.343607	0.002126	hypermethylated
cg03894103	0.63667	0.8078	0.343452	0.005269	hypermethylated
cg27532754	0.62072	0.787476757	0.343295	0.007472	hypermethylated
cg15001843	0.64462	0.817783243	0.34327	0.000166	hypermethylated
cg13175830	0.62532	0.793154054	0.343006	0.00064	hypermethylated
cg21488279	0.50105	0.635518919	0.34298	1.92E-05	hypermethylated
cg04658707	0.60072	0.761932432	0.34297	0.00421	hypermethylated
cg06895767	0.47445	0.601757838	0.342927	1.52E-05	hypermethylated
cg18591945	0.46856	0.594235135	0.3428	0.00504	hypermethylated
cg25818697	0.48115	0.610188649	0.342769	0.00031	hypermethylated
cg10722444	0.51246	0.649884865	0.342745	0.001466	hypermethylated
cg19296354	0.46099	0.584571351	0.342644	0.000528	hypermethylated
cg05081497	0.39364	0.499126486	0.342529	3.23E-05	hypermethylated
cg21542643	0.52665	0.667758378	0.342482	0.004365	hypermethylated
cg07804728	0.64071	0.812314595	0.342367	0.016315	hypermethylated
cg05349077	0.73458	0.931202162	0.342175	0.001395	hypermethylated
cg24248680	0.6759	0.856636216	0.341873	0.005222	hypermethylated
cg18566727	0.38856	0.492426486	0.341771	0.00029	hypermethylated
cg26584983	0.54811	0.694581622	0.341679	0.010042	hypermethylated
cg05771459	0.58181	0.737282162	0.341669	0.000474	hypermethylated
cg03680996	0.57781	0.732185405	0.341614	0.01109	hypermethylated
cg25198599	0.55797	0.706972973	0.341468	0.002318	hypermethylated
cg22161784	0.56325	0.713631351	0.341404	0.007472	hypermethylated
cg04207632	0.67733	0.858111892	0.341307	0.008783	hypermethylated
cg24494114	0.5124	0.649111351	0.341196	0.007158	hypermethylated
cg06740372	0.62074	0.786288649	0.34107	0.002274	hypermethylated
cg03693099	0.56397	0.714332973	0.340978	0.01737	hypermethylated
cg05410198	0.42473	0.537935135	0.340886	0.000426	hypermethylated
cg00950706	0.64713	0.819588108	0.340843	0.00421	hypermethylated
cg02135859	0.39865	0.504881622	0.340822	0.003506	hypermethylated
cg05757474	0.73434	0.92998	0.340752	0.001875	hypermethylated
cg00747890	0.73653	0.932725946	0.340709	0.002086	hypermethylated
cg24899806	0.54672	0.69234973	0.340699	0.000756	hypermethylated
cg00810939	0.67395	0.85345027	0.340666	0.001381	hypermethylated
cg27172557	0.53749	0.680582162	0.340531	0.000454	hypermethylated
cg15538767	0.46673	0.59094973	0.340447	1.42E-05	hypermethylated
cg10164264	0.70253	0.889503784	0.340441	0.000588	hypermethylated
cg18792031	0.63563	0.804798378	0.34044	0.002147	hypermethylated
cg18091615	0.61708	0.781278378	0.340379	0.011649	hypermethylated
cg16586406	0.48315	0.611671892	0.340287	0.000115	hypermethylated

cg19505194	0.55952	0.70832973	0.340231	0.005656	hypermethylated
cg12475142	0.63441	0.803037838	0.340052	0.01392	hypermethylated
cg12180613	0.62223	0.787605946	0.340026	1.84E-05	hypermethylated
cg05062854	0.43219	0.547008649	0.339898	0.000789	hypermethylated
cg16540134	0.65167	0.824697838	0.339724	0.0017	hypermethylated
cg18493030	0.60162	0.761325946	0.339662	0.005411	hypermethylated
cg11823159	0.60486	0.765360541	0.339538	0.006796	hypermethylated
cg14834653	0.67442	0.853343784	0.33948	0.000185	hypermethylated
cg12494683	0.63515	0.803580541	0.339345	0.009713	hypermethylated
cg15911153	0.50524	0.639112973	0.339102	0.00275	hypermethylated
cg15089111	0.51595	0.65266	0.3391	0.000382	hypermethylated
cg01790438	0.55866	0.706622162	0.338968	0.001667	hypermethylated
cg04355077	0.70107	0.886704865	0.338895	0.003441	hypermethylated
cg26947831	0.46267	0.585115135	0.338737	7.03E-06	hypermethylated
cg17468616	0.63202	0.799241622	0.338662	0.000893	hypermethylated
cg22142922	0.50467	0.638170811	0.338602	0.000253	hypermethylated
cg01250563	0.51411	0.650055135	0.338485	0.00013	hypermethylated
cg14215786	0.67762	0.856768108	0.338428	0.000193	hypermethylated
cg13461482	0.53229	0.672942162	0.33827	0.003986	hypermethylated
cg04205655	0.56847	0.718583784	0.338072	0.005911	hypermethylated
cg09762115	0.69876	0.883272973	0.338062	0.000875	hypermethylated
cg15231989	0.5782	0.730851892	0.33801	0.007799	hypermethylated
cg21880222	0.62141	0.78543027	0.337938	0.007035	hypermethylated
cg01804278	0.73625	0.930554595	0.337895	0.000245	hypermethylated
cg02384251	0.55301	0.698948649	0.337881	0.006679	hypermethylated
cg17927493	0.71237	0.900324324	0.337818	0.004525	hypermethylated
cg16574154	0.64549	0.815793514	0.337809	0.015439	hypermethylated
cg01400884	0.43329	0.547607027	0.337808	3.67E-05	hypermethylated
cg08884611	0.45892	0.579963784	0.33772	0.00101	hypermethylated
cg08209711	0.64948	0.820718919	0.337603	0.004485	hypermethylated
cg20764033	0.58151	0.734797297	0.337542	0.017643	hypermethylated
cg16051656	0.62251	0.786594054	0.337522	0.002649	hypermethylated
cg00833479	0.55826	0.705392432	0.337489	0.000408	hypermethylated
cg19394047	0.62321	0.787446486	0.337464	0.030537	hypermethylated
cg27015161	0.70088	0.885575676	0.337448	0.000163	hypermethylated
cg10826733	0.53268	0.673030811	0.337403	0.001164	hypermethylated
cg27227797	0.49881	0.630156216	0.337219	0.00028	hypermethylated
cg13056222	0.51756	0.65381027	0.337146	0.00032	hypermethylated
cg05564412	0.53821	0.679855676	0.337059	0.000403	hypermethylated
cg26613361	0.50199	0.634071351	0.336987	0.000209	hypermethylated
cg08324703	0.62921	0.794612432	0.33671	0.006796	hypermethylated
cg26194092	0.62171	0.785130811	0.336691	0.001212	hypermethylated
cg13316720	0.58278	0.735852432	0.336465	0.001912	hypermethylated
cg07898582	0.63059	0.796095676	0.33624	0.001635	hypermethylated
cg00067600	0.56334	0.711114595	0.336076	0.003637	hypermethylated
cg17160666	0.63271	0.798532973	0.335808	0.000495	hypermethylated
cg01336878	0.65436	0.825835135	0.335769	0.003986	hypermethylated
cg10418237	0.45695	0.576678378	0.335731	0.000219	hypermethylated
cg08115732	0.66462	0.838607027	0.335465	0.002318	hypermethylated
cg01748082	0.42239	0.532937297	0.33539	0.000132	hypermethylated
cg01836567	0.43365	0.547122162	0.335332	0.000119	hypermethylated
cg27456185	0.66097	0.833896216	0.335283	0.046224	hypermethylated
cg16562776	0.51827	0.653861081	0.33528	0.000588	hypermethylated
cg21859594	0.63001	0.794807568	0.335231	0.002231	hypermethylated
cg13779795	0.40961	0.516687027	0.33504	7.65E-05	hypermethylated
cg27578381	0.59452	0.749912432	0.334997	0.001212	hypermethylated
cg20591728	0.6729	0.848775676	0.334991	0.003843	hypermethylated
cg08749917	0.42609	0.537418378	0.334887	0.000485	hypermethylated

cg23056539	0.61829	0.779793514	0.334808	0.001423	hypermethylated
cg17482963	0.62881	0.792947027	0.3346	0.012637	hypermethylated
cg07576714	0.64667	0.815460541	0.334585	0.000831	hypermethylated
cg13167518	0.54274	0.684356216	0.334486	0.000142	hypermethylated
cg14678084	0.654	0.82454	0.334299	0.01806	hypermethylated
cg20160996	0.59053	0.74449027	0.334243	5.51E-05	hypermethylated
cg14856679	0.53874	0.679155676	0.334153	0.002599	hypermethylated
cg22191277	0.7226	0.910869189	0.334047	0.000219	hypermethylated
cg22008075	0.65616	0.827004865	0.333848	0.000545	hypermethylated
cg04266901	0.67566	0.851445946	0.333617	0.026186	hypermethylated
cg05365320	0.57137	0.719871351	0.333314	0.034014	hypermethylated
cg06507244	0.66366	0.836081622	0.333199	0.001052	hypermethylated
cg03841638	0.49255	0.620512432	0.33319	0.001452	hypermethylated
cg16669607	0.50373	0.634534595	0.333048	0.001041	hypermethylated
cg00870778	0.62317	0.784891892	0.332868	0.037035	hypermethylated
cg15273575	0.71382	0.898916757	0.332627	0.002599	hypermethylated
cg01217876	0.59062	0.743766486	0.33262	0.00099	hypermethylated
cg14780068	0.54078	0.680998919	0.332611	0.001262	hypermethylated
cg20309677	0.67283	0.847243243	0.332534	0.000274	hypermethylated
cg25486824	0.63668	0.80170973	0.332512	0.002363	hypermethylated
cg01856384	0.48549	0.611284324	0.332402	0.00028	hypermethylated
cg26410635	0.54144	0.681675135	0.332283	0.000875	hypermethylated
cg14758757	0.66795	0.840928108	0.332242	0.001118	hypermethylated
cg05679686	0.6481	0.815864324	0.332113	0.001074	hypermethylated
cg25306480	0.59972	0.754899459	0.331995	9.27E-05	hypermethylated
cg26274166	0.63513	0.799397297	0.331861	0.0006	hypermethylated
cg12964697	0.55356	0.696721622	0.331843	0.000361	hypermethylated
cg06261595	0.62016	0.780531351	0.331816	0.007933	hypermethylated
cg22628240	0.62202	0.782808108	0.331698	0.00513	hypermethylated
cg06659380	0.67344	0.847397297	0.331489	0.001571	hypermethylated
cg21336373	0.48232	0.606862703	0.33138	0.007933	hypermethylated
cg00095859	0.51636	0.649435135	0.330808	0.000178	hypermethylated
cg07187971	0.67741	0.851907027	0.330667	0.000613	hypermethylated
cg01191566	0.67309	0.846470811	0.330661	0.003195	hypermethylated
cg07125412	0.45938	0.577696216	0.330623	0.000495	hypermethylated
cg04051335	0.61685	0.775720541	0.330617	0.00099	hypermethylated
cg04304509	0.52339	0.65808	0.330377	0.008636	hypermethylated
cg01878308	0.67812	0.852570811	0.330279	0.043166	hypermethylated
cg16197088	0.71743	0.901904324	0.330136	0.001481	hypermethylated
cg08961621	0.56377	0.708625405	0.329916	0.000426	hypermethylated
cg27340506	0.47291	0.594329189	0.329697	0.00017	hypermethylated
cg00183067	0.47744	0.599948108	0.329518	0.00095	hypermethylated
cg27180492	0.68794	0.864451892	0.329503	0.02717	hypermethylated
cg22245961	0.52224	0.656167027	0.32935	0.013481	hypermethylated
cg26252958	0.65546	0.823474054	0.329215	0.0182	hypermethylated
cg17375267	0.44416	0.558011351	0.329215	0.000814	hypermethylated
cg21664351	0.65385	0.821427568	0.329174	0.024308	hypermethylated
cg00324366	0.57975	0.728314054	0.32913	0.029023	hypermethylated
cg14462779	0.43201	0.542688108	0.329059	2.24E-05	hypermethylated
cg16457842	0.60154	0.755587568	0.328938	7.56E-05	hypermethylated
cg21096141	0.66607	0.836640541	0.328934	0.002599	hypermethylated
cg04999691	0.50326	0.632082703	0.328809	0.000875	hypermethylated
cg20569034	0.56449	0.708942162	0.32872	0.000408	hypermethylated
cg21584979	0.68027	0.854262703	0.328572	0.000839	hypermethylated
cg17133446	0.64615	0.811403243	0.32855	0.022044	hypermethylated
cg06757405	0.39224	0.492532973	0.328484	9.96E-06	hypermethylated
cg16459735	0.66618	0.836437297	0.328345	0.012036	hypermethylated
cg16851046	0.58617	0.735936757	0.328263	0.009394	hypermethylated

cg05506270	0.52249	0.655936216	0.328152	0.001949	hypermethylated
cg01880541	0.55939	0.702201081	0.32803	0.012637	hypermethylated
cg21945949	0.6718	0.843302703	0.328019	0.001893	hypermethylated
cg12636435	0.4126	0.517791892	0.327629	0.000417	hypermethylated
cg12307237	0.56226	0.705526486	0.327463	0.000789	hypermethylated
cg19769715	0.5767	0.723637297	0.327446	0.004776	hypermethylated
cg23237276	0.51892	0.651027568	0.327206	0.000123	hypermethylated
cg01733599	0.635	0.79658973	0.32708	0.008709	hypermethylated
cg08783253	0.4963	0.622574054	0.327033	0.001734	hypermethylated
cg27480241	0.72008	0.903277838	0.327013	0.001785	hypermethylated
cg19755314	0.5846	0.733321622	0.326996	0.006855	hypermethylated
cg27228433	0.47358	0.594048108	0.326972	0.000575	hypermethylated
cg15368455	0.53294	0.668429189	0.326802	0.000327	hypermethylated
cg14761227	0.56923	0.713865946	0.326641	0.002231	hypermethylated
cg24483767	0.57463	0.720626486	0.326618	0.009713	hypermethylated
cg24028260	0.60207	0.755004324	0.326554	0.002965	hypermethylated
cg21516162	0.6585	0.825686486	0.326411	0.001987	hypermethylated
cg06282952	0.47802	0.599370811	0.326378	0.002066	hypermethylated
cg07047280	0.6378	0.799679459	0.326318	0.010042	hypermethylated
cg00730820	0.4756	0.596237297	0.326138	0.000253	hypermethylated
cg24413339	0.47563	0.596271892	0.326131	0.001262	hypermethylated
cg03099728	0.50682	0.635325405	0.326022	0.004445	hypermethylated
cg21481937	0.60791	0.762003784	0.32594	0.001423	hypermethylated
cg08214455	0.52024	0.652071351	0.325853	0.00054	hypermethylated
cg24498500	0.70991	0.889804324	0.325852	0.01021	hypermethylated
cg04370634	0.59206	0.742058378	0.325789	0.001541	hypermethylated
cg11164639	0.58962	0.738919459	0.325632	0.000912	hypermethylated
cg08182501	0.60422	0.75714	0.325486	0.000129	hypermethylated
cg15332951	0.72281	0.905713514	0.325438	0.003637	hypermethylated
cg17317266	0.62954	0.788825946	0.325409	0.003473	hypermethylated
cg15456742	0.6832	0.856037297	0.325366	0.030537	hypermethylated
cg04807108	0.54559	0.683612432	0.325361	0.000335	hypermethylated
cg26247841	0.70598	0.884543784	0.325306	0.000417	hypermethylated
cg03489382	0.52417	0.65671027	0.325222	0.000575	hypermethylated
cg02149965	0.4989	0.625010811	0.32513	0.019361	hypermethylated
cg18119735	0.48482	0.607354595	0.32509	0.00013	hypermethylated
cg00966405	0.63637	0.797174054	0.325029	0.000191	hypermethylated
cg05269013	0.54102	0.677697297	0.324959	0.000374	hypermethylated
cg04834608	0.71612	0.896856216	0.324675	0.002318	hypermethylated
cg22704696	0.41018	0.51367027	0.324585	6.95E-05	hypermethylated
cg06097580	0.67737	0.848238919	0.324527	0.002409	hypermethylated
cg20585500	0.66787	0.836272973	0.324407	0.007667	hypermethylated
cg08712082	0.71747	0.898369189	0.32439	0.001949	hypermethylated
cg20054111	0.66862	0.837194595	0.324376	0.000725	hypermethylated
cg24797276	0.57074	0.714591351	0.324285	0.007158	hypermethylated
cg13772815	0.52182	0.653278919	0.324147	0.007282	hypermethylated
cg09694921	0.53169	0.665591351	0.324051	0.004485	hypermethylated
cg17311074	0.45262	0.566601622	0.324034	0.000733	hypermethylated
cg09138865	0.61586	0.770911892	0.323964	0.002363	hypermethylated
cg02311364	0.57985	0.72574973	0.323792	0.000112	hypermethylated
cg08244181	0.58469	0.731804324	0.323786	0.010819	hypermethylated
cg19041006	0.52408	0.655858919	0.323598	1.18E-05	hypermethylated
cg26348803	0.64574	0.808036757	0.323468	0.004022	hypermethylated
cg23259001	0.49969	0.625242703	0.323383	0.002318	hypermethylated
cg01058717	0.7414	0.927609189	0.323265	0.004525	hypermethylated
cg22737473	0.58959	0.737512432	0.322955	0.005706	hypermethylated
cg14905657	0.4944	0.618394054	0.322848	5.38E-05	hypermethylated
cg24903893	0.55992	0.700323784	0.322801	0.002502	hypermethylated

cg03808577	0.53174	0.665074054	0.322794	0.003773	hypermethylated
cg25492583	0.4203	0.525650811	0.322685	0.0003	hypermethylated
cg17376609	0.63919	0.79936	0.322601	0.002274	hypermethylated
cg21565421	0.49027	0.613058919	0.322449	0.000327	hypermethylated
cg00940260	0.71562	0.894831351	0.322422	0.000931	hypermethylated
cg04344749	0.47103	0.58890973	0.322228	0.000204	hypermethylated
cg13292542	0.75226	0.940489189	0.32218	6.30E-05	hypermethylated
cg12422704	0.49131	0.614221622	0.322126	1.25E-05	hypermethylated
cg12932195	0.68917	0.861457297	0.321919	0.002274	hypermethylated
cg19866325	0.73642	0.920509189	0.321903	0.000342	hypermethylated
cg08296263	0.64161	0.801981622	0.321873	0.005459	hypermethylated
cg14791193	0.66524	0.83147027	0.32179	0.000142	hypermethylated
cg15437741	0.46837	0.585398919	0.321771	0.003473	hypermethylated
cg02145310	0.50359	0.629417297	0.321767	7.56E-05	hypermethylated
cg17074353	0.51313	0.641258919	0.321583	0.00015	hypermethylated
cg19515186	0.67414	0.842437838	0.321522	0.01274	hypermethylated
cg11880367	0.57456	0.717952432	0.321431	0.000224	hypermethylated
cg16484042	0.582	0.727221622	0.321376	0.00035	hypermethylated
cg04254769	0.63838	0.797638919	0.32132	4.94E-05	hypermethylated
cg21719361	0.67629	0.845002162	0.321313	0.002502	hypermethylated
cg26846409	0.47581	0.5945	0.321291	0.001603	hypermethylated
cg26616258	0.74392	0.929482703	0.321281	5.86E-05	hypermethylated
cg20284239	0.48106	0.601051892	0.321273	0.002725	hypermethylated
cg13190608	0.59189	0.739378919	0.320985	0.001803	hypermethylated
cg12252039	0.713	0.890666486	0.320983	0.000178	hypermethylated
cg23287992	0.48076	0.600499459	0.320846	0.001481	hypermethylated
cg12410310	0.53371	0.666609189	0.320785	0.019813	hypermethylated
cg23398109	0.60244	0.752321081	0.320531	0.004022	hypermethylated
cg21815337	0.55207	0.68940973	0.32051	0.016444	hypermethylated
cg04835383	0.66022	0.82444	0.320468	0.006796	hypermethylated
cg14724925	0.4765	0.594983243	0.320373	0.001237	hypermethylated
cg17972789	0.67762	0.84607027	0.320301	0.022892	hypermethylated
cg03444587	0.57355	0.716078919	0.320199	0.010381	hypermethylated
cg10935498	0.56517	0.705595135	0.320156	0.001262	hypermethylated
cg02243276	0.69546	0.868217838	0.32009	0.001751	hypermethylated
cg24626310	0.49616	0.61940973	0.320089	0.013265	hypermethylated
cg25211622	0.75115	0.937667027	0.319975	0.004566	hypermethylated
cg26344513	0.63004	0.786474054	0.319956	0.001587	hypermethylated
cg19372975	0.72874	0.909671892	0.319942	0.008069	hypermethylated
cg05887092	0.55015	0.686735135	0.319929	7.65E-05	hypermethylated
cg24042452	0.63993	0.798718919	0.319774	0.000293	hypermethylated
cg23895963	0.50943	0.635761081	0.319601	0.000667	hypermethylated
cg12489915	0.71626	0.893868108	0.319579	0.00495	hypermethylated
cg06333307	0.62143	0.775498919	0.319533	0.006339	hypermethylated
cg00128386	0.64359	0.803067027	0.319378	0.006855	hypermethylated
cg04215359	0.67345	0.840322703	0.319373	0.000912	hypermethylated
cg21539229	0.6165	0.76914973	0.319164	0.012036	hypermethylated
cg11908453	0.62713	0.782388108	0.31912	0.010642	hypermethylated
cg13572771	0.65937	0.822522703	0.318967	8.84E-05	hypermethylated
cg24469013	0.57801	0.721030811	0.318966	0.006339	hypermethylated
cg19936016	0.71553	0.892539459	0.318904	8.22E-05	hypermethylated
cg13654884	0.6897	0.860264865	0.318812	0.000331	hypermethylated
cg18456140	0.73583	0.917777838	0.318772	0.000109	hypermethylated
cg12085501	0.63571	0.79286	0.318697	0.012948	hypermethylated
cg12357484	0.55461	0.691580541	0.318424	1.14E-05	hypermethylated
cg02203336	0.66426	0.8283	0.318405	0.000912	hypermethylated
cg14362113	0.62421	0.778358378	0.318403	8.42E-05	hypermethylated
cg24143766	0.59456	0.741363243	0.318358	0.031661	hypermethylated

cg01407254	0.64526	0.804568649	0.318335	0.006564	hypermethylated
cg08447992	0.70354	0.877216757	0.318301	0.000893	hypermethylated
cg04553307	0.62405	0.77799027	0.31809	0.000688	hypermethylated
cg26921611	0.69593	0.867595676	0.318081	0.000248	hypermethylated
cg11426800	0.47194	0.588344865	0.318059	0.001288	hypermethylated
cg17948913	0.68821	0.857912973	0.317982	0.00591	hypermethylated
cg19196335	0.60542	0.754639459	0.317851	0.00623	hypermethylated
cg03547757	0.61707	0.769155676	0.317841	1.97E-05	hypermethylated
cg05203809	0.59398	0.740344865	0.317783	0.011554	hypermethylated
cg03122674	0.54523	0.679524324	0.31766	0.001118	hypermethylated
cg11143671	0.58974	0.734969189	0.317605	5.45E-05	hypermethylated
cg26228014	0.60665	0.756002162	0.317526	0.000725	hypermethylated
cg05533539	0.56896	0.708965405	0.317388	1.52E-05	hypermethylated
cg07906331	0.59913	0.746425946	0.31713	0.016968	hypermethylated
cg14514120	0.6369	0.793476757	0.317121	0.000741	hypermethylated
cg02675546	0.69722	0.868451351	0.316831	0.001096	hypermethylated
cg26992415	0.69483	0.865463243	0.316813	8.94E-05	hypermethylated
cg21786381	0.4622	0.575703784	0.316809	0.002147	hypermethylated
cg12440350	0.54307	0.676397838	0.316734	0.000109	hypermethylated
cg22082456	0.57522	0.716413514	0.316679	0.026772	hypermethylated
cg08289056	0.68102	0.848041081	0.316437	0.002106	hypermethylated
cg11385933	0.47662	0.593491351	0.316388	7.84E-05	hypermethylated
cg21660452	0.71763	0.893554595	0.316316	0.001541	hypermethylated
cg14905600	0.75568	0.940886486	0.316245	0.001785	hypermethylated
cg20866932	0.73749	0.918230811	0.316233	0.000626	hypermethylated
cg11795921	0.63403	0.78927027	0.315968	0.001541	hypermethylated
cg03928367	0.72101	0.89745027	0.315813	0.004059	hypermethylated
cg10487418	0.60722	0.755742703	0.315676	0.002751	hypermethylated
cg02851397	0.46522	0.578954595	0.315537	0.000209	hypermethylated
cg21765730	0.63672	0.792361081	0.315499	0.010042	hypermethylated
cg05058846	0.63585	0.791174595	0.31531	0.000109	hypermethylated
cg27383174	0.60272	0.749934595	0.315277	0.025045	hypermethylated
cg00260012	0.61961	0.770875676	0.315138	0.001052	hypermethylated
cg01059818	0.68811	0.856039459	0.315038	8.42E-05	hypermethylated
cg14684068	0.57512	0.715471351	0.315031	0.006284	hypermethylated
cg11834658	0.68432	0.851158378	0.314756	0.004134	hypermethylated
cg12647587	0.70248	0.8737	0.314681	0.006855	hypermethylated
cg16757724	0.55315	0.687902162	0.314533	0.015439	hypermethylated
cg06641279	0.64669	0.804227568	0.314529	0.000152	hypermethylated
cg08042322	0.67012	0.833336757	0.31448	0.015076	hypermethylated
cg12407978	0.54793	0.681348649	0.314402	0.000681	hypermethylated
cg16929041	0.63442	0.788895676	0.314396	0.011554	hypermethylated
cg07777344	0.58638	0.729086486	0.314254	0.028602	hypermethylated
cg14978637	0.54544	0.678108649	0.314096	0.003107	hypermethylated
cg24202221	0.68366	0.849919459	0.314047	0.001327	hypermethylated
cg20501882	0.65367	0.812515676	0.313833	0.01274	hypermethylated
cg04011995	0.63547	0.789874595	0.3138	0.000293	hypermethylated
cg05825400	0.46668	0.579993514	0.313603	0.000104	hypermethylated
cg20475035	0.43467	0.540177838	0.313514	0.000485	hypermethylated
cg26010655	0.62691	0.778920541	0.313218	0.004526	hypermethylated
cg02996583	0.62226	0.773081081	0.313102	0.000104	hypermethylated
cg14570652	0.74767	0.928837838	0.313025	6.86E-05	hypermethylated
cg17509681	0.72183	0.896655676	0.312895	0.000474	hypermethylated
cg11664818	0.53665	0.666615135	0.312873	0.008348	hypermethylated
cg15773539	0.47419	0.589001081	0.312805	0.000251	hypermethylated
cg14416248	0.68976	0.856700541	0.312697	0.046224	hypermethylated
cg09917805	0.55169	0.685165405	0.312594	0.000365	hypermethylated
cg10656845	0.65151	0.809019459	0.312387	0.012036	hypermethylated

cg15827843	0.68031	0.844773514	0.312372	0.008491	hypermethylated
cg24219974	0.59584	0.739872432	0.312352	0.001587	hypermethylated
cg17967673	0.61689	0.765992432	0.312317	0.00032	hypermethylated
cg01760189	0.65389	0.811901081	0.312256	0.004445	hypermethylated
cg21930842	0.50534	0.627443784	0.312232	0.018773	hypermethylated
cg23201008	0.62204	0.772317838	0.312187	0.015317	hypermethylated
cg25306511	0.60095	0.745977297	0.311887	0.012535	hypermethylated
cg06009783	0.71061	0.881980541	0.311689	0.002086	hypermethylated
cg21768604	0.73142	0.907671892	0.311471	0.001619	hypermethylated
cg16510099	0.65624	0.814277838	0.311298	0.000293	hypermethylated
cg26681183	0.6267	0.777608108	0.311268	0.000165	hypermethylated
cg21706183	0.58556	0.726525405	0.311196	0.008857	hypermethylated
cg27201802	0.55115	0.683794595	0.311118	0.0002	hypermethylated
cg21049762	0.64341	0.798174054	0.310965	0.00151	hypermethylated
cg07865267	0.64453	0.799472973	0.310802	0.006175	hypermethylated
cg23007540	0.69515	0.862245405	0.310774	0.040844	hypermethylated
cg08513123	0.52938	0.656620541	0.310756	0.001288	hypermethylated
cg06050631	0.66236	0.821548649	0.31073	0.033532	hypermethylated
cg09685096	0.6211	0.770361622	0.31071	0.002106	hypermethylated
cg11661512	0.50148	0.62198	0.310676	0.00341	hypermethylated
cg27160348	0.60885	0.75496	0.310313	0.001164	hypermethylated
cg20645601	0.53619	0.664831892	0.310245	0.01392	hypermethylated
cg07379703	0.56034	0.694763784	0.31022	0.000875	hypermethylated
cg22879689	0.62622	0.776423243	0.310174	0.002649	hypermethylated
cg05278955	0.6751	0.836931892	0.310009	0.00504	hypermethylated
cg21550372	0.57835	0.716979459	0.309989	0.001262	hypermethylated
cg13305657	0.41616	0.515848108	0.309808	0.006229	hypermethylated
cg01860778	0.52802	0.654458378	0.309709	0.000696	hypermethylated
cg07190914	0.66988	0.830226486	0.309602	0.000831	hypermethylated
cg07985164	0.68491	0.848715676	0.309367	0.001381	hypermethylated
cg27205902	0.70385	0.872183243	0.309363	0.013809	hypermethylated
cg19699317	0.62558	0.775152973	0.309287	0.001275	hypermethylated
cg10004079	0.67353	0.834515676	0.309197	0.010381	hypermethylated
cg04248279	0.62852	0.778617297	0.308956	0.009238	hypermethylated
cg04955511	0.65357	0.809643784	0.308946	0.001541	hypermethylated
cg00775939	0.63368	0.784932432	0.308814	0.003255	hypermethylated
cg20723705	0.63698	0.788991892	0.308762	0.037035	hypermethylated
cg19878194	0.62677	0.77632973	0.308733	0.006451	hypermethylated
cg05520988	0.66091	0.818574054	0.308659	0.035497	hypermethylated
cg16236960	0.69994	0.866885405	0.30861	0.002965	hypermethylated
cg10155304	0.57614	0.713540541	0.308576	0.000262	hypermethylated
cg01068023	0.66618	0.825007027	0.308494	0.001619	hypermethylated
cg08097359	0.52698	0.652612432	0.308478	7.12E-05	hypermethylated
cg11229101	0.55568	0.688074595	0.308311	0.00017	hypermethylated
cg04214946	0.73011	0.903956216	0.308139	0.000741	hypermethylated
cg11878045	0.53685	0.664632432	0.308038	0.003441	hypermethylated
cg20847228	0.66309	0.820852432	0.307918	0.002409	hypermethylated
cg20656751	0.42767	0.529377838	0.3078	2.35E-05	hypermethylated
cg04819959	0.67822	0.839394595	0.307596	0.000262	hypermethylated
cg07421737	0.69793	0.863732432	0.307502	0.0017	hypermethylated
cg14789214	0.47574	0.588655676	0.307251	0.000857	hypermethylated
cg25952964	0.58903	0.728788108	0.307158	0.005364	hypermethylated
cg10811502	0.62042	0.767601622	0.307113	0.003195	hypermethylated
cg06741803	0.56743	0.702026486	0.307083	0.002066	hypermethylated
cg17403512	0.56518	0.699229189	0.307055	4.94E-05	hypermethylated
cg04760604	0.51943	0.642611351	0.307017	0.000138	hypermethylated
cg12446446	0.70098	0.867185405	0.306967	0.002751	hypermethylated
cg10032599	0.5375	0.664934595	0.306948	0.000358	hypermethylated

cg27276079	0.7618	0.942342162	0.306839	0.001466	hypermethylated
cg14609668	0.42451	0.525055135	0.30667	0.002751	hypermethylated
cg02451516	0.71273	0.881519459	0.306637	0.010042	hypermethylated
cg06638913	0.49244	0.608924324	0.306315	0.019661	hypermethylated
cg07799299	0.62138	0.768348649	0.306285	0.000117	hypermethylated
cg19094872	0.6617	0.818094054	0.306089	0.001085	hypermethylated
cg25262548	0.68389	0.845523243	0.30608	0.022892	hypermethylated
cg13649330	0.64358	0.795651351	0.306017	0.000495	hypermethylated
cg15864104	0.68694	0.849244865	0.305996	0.005508	hypermethylated
cg21787249	0.64685	0.799641081	0.305921	0.000696	hypermethylated
cg26349266	0.51849	0.640957297	0.305912	4.20E-05	hypermethylated
cg11401159	0.69772	0.862495135	0.305868	0.006339	hypermethylated
cg24216555	0.61941	0.765644865	0.305781	0.009084	hypermethylated
cg02952703	0.52933	0.654284865	0.305751	0.000317	hypermethylated
cg26328893	0.66135	0.81740973	0.305645	0.003506	hypermethylated
cg09591329	0.6847	0.846209189	0.305542	0.011554	hypermethylated
cg18034637	0.59856	0.739654595	0.305356	0.010467	hypermethylated
cg17373343	0.69322	0.856407027	0.304983	0.003136	hypermethylated
cg19795594	0.67298	0.831372432	0.304931	0.001	hypermethylated
cg12894814	0.66926	0.826595676	0.304615	0.004863	hypermethylated
cg05526731	0.7527	0.929649189	0.304611	0.000168	hypermethylated
cg00231300	0.45231	0.558634054	0.304592	0.005706	hypermethylated
cg10486098	0.49895	0.616163243	0.304417	0.000234	hypermethylated
cg14063129	0.65825	0.812824865	0.304309	0.001603	hypermethylated
cg01542143	0.65335	0.806567027	0.303938	0.009238	hypermethylated
cg10987503	0.57358	0.708023784	0.303803	0.002363	hypermethylated
cg13762320	0.5928	0.731626486	0.303562	6.31E-05	hypermethylated
cg16670307	0.67412	0.831891892	0.303391	0.001188	hypermethylated
cg07703790	0.62588	0.772352973	0.303374	0.004776	hypermethylated
cg18164305	0.53272	0.657156216	0.302859	0.004863	hypermethylated
cg24301352	0.60374	0.744754054	0.302837	0.003021	hypermethylated
cg08139729	0.64552	0.796150811	0.30258	0.004445	hypermethylated
cg19959917	0.52967	0.653244865	0.30253	4.36E-05	hypermethylated
cg05977021	0.65866	0.812157838	0.302226	0.006564	hypermethylated
cg23746628	0.68002	0.838456757	0.302159	0.011649	hypermethylated
cg16908948	0.68165	0.840453514	0.302137	0.000293	hypermethylated
cg20707409	0.62404	0.769374595	0.302048	0.000274	hypermethylated
cg12923203	0.72462	0.893194054	0.301749	0.000764	hypermethylated
cg08133669	0.69101	0.851718919	0.301671	0.013589	hypermethylated
cg19389397	0.51992	0.640810811	0.301609	0.005316	hypermethylated
cg13191508	0.72529	0.893904324	0.301562	2.46E-06	hypermethylated
cg05578673	0.66598	0.820791351	0.301537	0.040844	hypermethylated
cg02642565	0.62135	0.765708108	0.301388	0.002318	hypermethylated
cg19854437	0.62625	0.771721622	0.301342	0.004059	hypermethylated
cg14029170	0.65187	0.803264865	0.301291	0.002231	hypermethylated
cg14535746	0.70511	0.868763243	0.301115	0.002803	hypermethylated
cg02163943	0.70164	0.864404865	0.300976	0.022892	hypermethylated
cg16781502	0.56832	0.700136216	0.300932	0.013053	hypermethylated
cg04585435	0.68244	0.84057027	0.300666	0.001541	hypermethylated
cg01637244	0.6964	0.857758919	0.300656	0.002046	hypermethylated
cg15952725	0.70854	0.872681081	0.300605	0.002699	hypermethylated
cg17750109	0.52296	0.644076216	0.300531	0.000805	hypermethylated
cg06262497	0.7292	0.89801027	0.300417	0.0171	hypermethylated
cg10306035	0.65585	0.807632432	0.300333	0.00054	hypermethylated
cg06730678	0.67572	0.832083784	0.300303	0.003671	hypermethylated
cg13792694	0.74129	0.91281027	0.300277	0.003773	hypermethylated
cg13195955	0.51965	0.63986	0.300216	0.000528	hypermethylated
cg04147272	0.68831	0.84750973	0.300171	0.013589	hypermethylated

cg02733081	0.62293	0.766994595	0.300146	0.042869	hypermethylated
cg04509682	0.68886	0.848092973	0.300012	0.03189	hypermethylated
cg16763574	0.75292	0.926948649	0.299993	0.000115	hypermethylated
cg14168269	0.64735	0.796880541	0.299818	0.000137	hypermethylated
cg04607024	0.68511	0.843345946	0.299789	9.27E-05	hypermethylated
cg27639142	0.53299	0.655836757	0.299228	0.00066	hypermethylated
cg01009486	0.63373	0.779762162	0.299166	0.002699	hypermethylated
cg19689151	0.66773	0.821582162	0.29914	0.00421	hypermethylated
cg12251895	0.59828	0.735988108	0.298862	0.003506	hypermethylated
cg00502618	0.72381	0.890296757	0.298675	0.00645	hypermethylated
cg06813419	0.68461	0.842077838	0.298671	0.012234	hypermethylated
cg01239389	0.45097	0.554604865	0.298429	0.002318	hypermethylated
cg01973394	0.51438	0.632481622	0.298189	0.000338	hypermethylated
cg20345915	0.64141	0.788657838	0.298153	1.94E-05	hypermethylated
cg03654783	0.57651	0.708837297	0.298109	0.000224	hypermethylated
cg12729166	0.6508	0.800141622	0.298041	4.26E-05	hypermethylated
cg09226986	0.62674	0.770497838	0.297924	0.004326	hypermethylated
cg13912196	0.68316	0.839825405	0.297866	0.016835	hypermethylated
cg02657828	0.53265	0.654728649	0.297709	0.001052	hypermethylated
cg03704667	0.68136	0.837471892	0.297624	0.003378	hypermethylated
cg15944367	0.56561	0.695197838	0.297616	0.004567	hypermethylated
cg08688393	0.52787	0.648751892	0.297484	0.015561	hypermethylated
cg10845893	0.69667	0.856164865	0.297413	0.000797	hypermethylated
cg02387701	0.57607	0.707942162	0.297387	0.009084	hypermethylated
cg11071407	0.67775	0.832862162	0.297325	0.00064	hypermethylated
cg07514159	0.6114	0.751324324	0.297319	1.15E-05	hypermethylated
cg24313571	0.65752	0.807982162	0.297289	0.049797	hypermethylated
cg23538468	0.65772	0.808201622	0.297242	0.00039	hypermethylated
cg15527168	0.66123	0.812439459	0.297108	0.00101	hypermethylated
cg24186506	0.69736	0.85674973	0.29697	0.001052	hypermethylated
cg24530480	0.64443	0.791718378	0.296964	0.019965	hypermethylated
cg21313071	0.6426	0.789352973	0.29675	0.000109	hypermethylated
cg18147865	0.62481	0.767463784	0.296681	0.000588	hypermethylated
cg13618880	0.68476	0.841031351	0.296561	0.007732	hypermethylated
cg02732915	0.6614	0.812327027	0.296538	0.000459	hypermethylated
cg10523671	0.70333	0.863683784	0.296301	0.002231	hypermethylated
cg06948989	0.46499	0.570939459	0.296138	0.000354	hypermethylated
cg13983640	0.63014	0.773631351	0.295974	0.019965	hypermethylated
cg09632858	0.74347	0.912557838	0.295641	0.004209	hypermethylated
cg11311053	0.48426	0.594383784	0.295613	0.00071	hypermethylated
cg27392802	0.73424	0.900999459	0.295275	0.006505	hypermethylated
cg04146259	0.67707	0.830776216	0.295155	0.012234	hypermethylated
cg07926644	0.62389	0.765494054	0.295099	0.003195	hypermethylated
cg12381873	0.55	0.674801081	0.295031	0.000313	hypermethylated
cg06243084	0.71392	0.875761081	0.294775	0.00017	hypermethylated
cg04269530	0.50959	0.625012973	0.294549	0.000115	hypermethylated
cg02738641	0.56241	0.689774595	0.294503	0.002599	hypermethylated
cg10254035	0.65435	0.80253027	0.294493	0.014145	hypermethylated
cg09567180	0.63721	0.781411351	0.294313	0.008857	hypermethylated
cg07537750	0.58896	0.722239459	0.294308	0.020273	hypermethylated
cg02556042	0.45644	0.559722703	0.294287	0.001949	hypermethylated
cg18700428	0.5362	0.657473514	0.294162	7.12E-05	hypermethylated
cg13173552	0.57834	0.70913027	0.294133	0.004326	hypermethylated
cg14554244	0.5038	0.61758	0.293775	0.000551	hypermethylated
cg04446870	0.62696	0.768542703	0.293752	0.009959	hypermethylated
cg16454316	0.66126	0.810562162	0.293705	0.000107	hypermethylated
cg12786452	0.67328	0.825293514	0.293701	0.006679	hypermethylated
cg02856026	0.56484	0.692314054	0.293584	0.022549	hypermethylated

cg25113462	0.69728	0.854596216	0.293505	0.004059	hypermethylated
cg23134259	0.71752	0.879400541	0.293501	0.014956	hypermethylated
cg19919217	0.61767	0.756994595	0.293447	0.007933	hypermethylated
cg27445278	0.67181	0.82330973	0.293382	0.004995	hypermethylated
cg05221264	0.65676	0.804771351	0.293213	0.001141	hypermethylated
cg25657700	0.54191	0.664021081	0.293176	0.000374	hypermethylated
cg26105076	0.53084	0.650455676	0.293174	0.000551	hypermethylated
cg06043908	0.52021	0.637422162	0.293155	0.006015	hypermethylated
cg09274961	0.59842	0.73325027	0.293147	0.008636	hypermethylated
cg19816642	0.40434	0.4954	0.293025	0.000588	hypermethylated
cg17839324	0.57403	0.703295135	0.293004	0.00102	hypermethylated
cg07554664	0.48735	0.597067568	0.292936	0.034997	hypermethylated
cg03152428	0.56094	0.687095676	0.292665	0.003316	hypermethylated
cg08765199	0.65422	0.801339459	0.292638	0.025232	hypermethylated
cg03844894	0.51286	0.62815027	0.292545	0.000327	hypermethylated
cg00436254	0.48709	0.596574595	0.292514	0.001107	hypermethylated
cg19814174	0.74093	0.907458919	0.292495	0.000893	hypermethylated
cg03267742	0.5601	0.685978378	0.292479	0.041414	hypermethylated
cg00597931	0.66827	0.818425405	0.29242	0.023947	hypermethylated
cg16537483	0.61847	0.757408108	0.292367	0.000528	hypermethylated
cg05714552	0.41584	0.509241081	0.29232	4.05E-05	hypermethylated
cg26879339	0.66647	0.816147027	0.292289	0.01274	hypermethylated
cg18354248	0.7326	0.896937838	0.291982	0.008563	hypermethylated
cg03553613	0.6953	0.851268649	0.291979	7.29E-05	hypermethylated
cg05987548	0.57006	0.697857838	0.291819	0.001118	hypermethylated
cg10647547	0.68447	0.837894054	0.291781	0.00916	hypermethylated
cg13877715	0.57505	0.703918378	0.291721	0.012434	hypermethylated
cg00320608	0.63403	0.775937297	0.291389	0.009959	hypermethylated
cg03533058	0.54732	0.669817297	0.291383	0.003704	hypermethylated
cg16550651	0.64419	0.788303243	0.291264	0.000166	hypermethylated
cg24010859	0.54137	0.662399459	0.291087	0.023066	hypermethylated
cg09569989	0.71587	0.875888649	0.29105	1.60E-05	hypermethylated
cg14352682	0.47542	0.581624865	0.290886	0.002649	hypermethylated
cg13569987	0.68603	0.83928	0.290881	0.00291	hypermethylated
cg08535058	0.56411	0.68996973	0.290557	0.000204	hypermethylated
cg09427374	0.70957	0.867794054	0.290408	0.004287	hypermethylated
cg18130905	0.69549	0.850500541	0.290282	0.002409	hypermethylated
cg07220650	0.53768	0.657517838	0.290282	0.002699	hypermethylated
cg21190038	0.60327	0.737702162	0.290235	0.003021	hypermethylated
cg07297322	0.65777	0.80420973	0.289989	0.001541	hypermethylated
cg27109006	0.64033	0.782815676	0.289857	0.02324	hypermethylated
cg05075579	0.46374	0.566911351	0.289807	0.000209	hypermethylated
cg21198455	0.75452	0.922312432	0.289696	0.001684	hypermethylated
cg13898875	0.61156	0.747536757	0.28965	0.002231	hypermethylated
cg25732961	0.77793	0.950452432	0.288974	0.001716	hypermethylated
cg00028844	0.5231	0.639065946	0.288878	0.01146	hypermethylated
cg09571369	0.62645	0.765260541	0.288752	0.000152	hypermethylated
cg04969428	0.61769	0.754428649	0.288501	0.023948	hypermethylated
cg01819995	0.67585	0.825444865	0.288469	0.002253	hypermethylated
cg03473377	0.663	0.809744865	0.288459	0.000511	hypermethylated
cg01670896	0.71508	0.873318919	0.288404	0.01073	hypermethylated
cg11809668	0.5903	0.720840541	0.288232	0.004365	hypermethylated
cg19946376	0.53536	0.653708649	0.288138	0.003021	hypermethylated
cg13927454	0.63585	0.776337838	0.287998	0.004248	hypermethylated
cg13891539	0.69108	0.843665946	0.287819	0.000633	hypermethylated
cg26187962	0.59013	0.720426486	0.287818	0.000293	hypermethylated
cg08944029	0.59866	0.730824324	0.287788	0.004059	hypermethylated
cg20002958	0.71466	0.872407027	0.287744	0.005606	hypermethylated

cg10853431	0.62914	0.767982162	0.287692	0.002725	hypermethylated
cg26053291	0.62245	0.759798919	0.28766	0.000501	hypermethylated
cg04220930	0.64624	0.788836757	0.287657	0.001525	hypermethylated
cg14958141	0.65013	0.793522162	0.287542	0.007536	hypermethylated
cg08170911	0.45091	0.550335676	0.287472	0.000138	hypermethylated
cg09053536	0.64428	0.786256216	0.287312	0.008001	hypermethylated
cg21932672	0.68948	0.841308649	0.287126	0.00031	hypermethylated
cg26675485	0.58823	0.717748108	0.287097	0.005808	hypermethylated
cg03557916	0.69802	0.851697297	0.287072	0.001288	hypermethylated
cg14129589	0.68356	0.834045946	0.287059	0.000204	hypermethylated
cg06813782	0.65842	0.803295135	0.286922	0.011274	hypermethylated
cg00790847	0.71061	0.86692973	0.286857	0.001437	hypermethylated
cg09056876	0.69777	0.851197297	0.286742	0.001052	hypermethylated
cg02642962	0.74102	0.903930811	0.2867	0.001785	hypermethylated
cg21680729	0.58629	0.715032973	0.286395	0.001667	hypermethylated
cg22238209	0.49447	0.602996757	0.286267	0.003843	hypermethylated
cg12598606	0.56937	0.69415027	0.285882	0.003225	hypermethylated
cg07850896	0.70368	0.857877838	0.285853	0.013589	hypermethylated
cg22934200	0.64202	0.782678919	0.285802	0.003506	hypermethylated
cg21343223	0.42699	0.520535135	0.285793	1.50E-05	hypermethylated
cg26575637	0.48377	0.589721622	0.285713	8.84E-05	hypermethylated
cg25133533	0.48823	0.595134054	0.285654	6.78E-05	hypermethylated
cg09635533	0.48122	0.586439459	0.285286	0.0003	hypermethylated
cg11312087	0.65465	0.797608649	0.284957	0.024675	hypermethylated
cg05377041	0.57997	0.706588649	0.284892	0.004566	hypermethylated
cg21621104	0.6948	0.846438378	0.284807	0.020902	hypermethylated
cg02348119	0.62777	0.76476973	0.284789	0.00221	hypermethylated
cg15426006	0.57314	0.698161622	0.284673	3.15E-05	hypermethylated
cg19849478	0.66436	0.809207027	0.284544	0.021878	hypermethylated
cg11231143	0.57954	0.705773514	0.284297	0.000209	hypermethylated
cg16146718	0.74875	0.911792432	0.284221	0.001	hypermethylated
cg19528797	0.71881	0.87532973	0.284216	0.008491	hypermethylated
cg10595406	0.69944	0.851690811	0.284129	0.002126	hypermethylated
cg19938826	0.7216	0.87866	0.284106	0.010909	hypermethylated
cg18434588	0.65079	0.792415135	0.284064	0.003843	hypermethylated
cg26866142	0.7499	0.913072973	0.284032	0.047819	hypermethylated
cg05926586	0.67311	0.819537838	0.283968	0.001262	hypermethylated
cg12743419	0.53445	0.650709189	0.283958	0.001541	hypermethylated
cg05260714	0.64844	0.789485946	0.28394	0.011841	hypermethylated
cg14596987	0.71399	0.869185405	0.28376	0.000681	hypermethylated
cg12853539	0.72184	0.878585405	0.283503	0.001288	hypermethylated
cg19253850	0.60168	0.732271351	0.283382	0.002126	hypermethylated
cg06730250	0.4811	0.585462703	0.28324	0.008208	hypermethylated
cg14601053	0.56505	0.687555676	0.283098	0.003441	hypermethylated
cg17256234	0.72057	0.876714595	0.282969	0.001367	hypermethylated
cg07272353	0.5356	0.651656757	0.282956	0.011841	hypermethylated
cg07949011	0.65456	0.796160541	0.282534	0.003986	hypermethylated
cg17172761	0.617	0.750458919	0.282503	0.002066	hypermethylated
cg19261306	0.65581	0.797523784	0.28225	0.033532	hypermethylated
cg19539519	0.72391	0.880244324	0.282094	0.000287	hypermethylated
cg14841514	0.63221	0.768663243	0.281948	0.000361	hypermethylated
cg27616541	0.65283	0.793730811	0.281942	0.000248	hypermethylated
cg01068906	0.53359	0.648706486	0.281834	6.95E-05	hypermethylated
cg00892368	0.63624	0.773495135	0.281821	0.000126	hypermethylated
cg22328746	0.6463	0.785596757	0.281585	0.000182	hypermethylated
cg27462475	0.67926	0.825549189	0.28139	0.00395	hypermethylated
cg25364273	0.63616	0.773112973	0.28129	0.000346	hypermethylated
cg22290117	0.53736	0.653026486	0.281253	0.000245	hypermethylated

cg02198895	0.58885	0.71558973	0.281233	0.000563	hypermethylated
cg00905101	0.5446	0.661814054	0.281229	0.000137	hypermethylated
cg02485328	0.63511	0.77163027	0.280903	0.001717	hypermethylated
cg26557834	0.6387	0.775922162	0.280773	0.034258	hypermethylated
cg10505024	0.48584	0.590218378	0.280768	0.00513	hypermethylated
cg16475811	0.63137	0.767009189	0.280758	0.002106	hypermethylated
cg04170948	0.619	0.751897297	0.280596	0.000148	hypermethylated
cg23295647	0.52734	0.640525946	0.280524	8.13E-05	hypermethylated
cg02630854	0.68076	0.826854595	0.280487	2.51E-05	hypermethylated
cg04652943	0.65063	0.79014973	0.280289	0.000112	hypermethylated
cg00421335	0.57102	0.693466486	0.280285	0.001541	hypermethylated
cg20122586	0.56129	0.68162973	0.280242	0.045598	hypermethylated
cg19246065	0.53537	0.650102703	0.280131	0.016061	hypermethylated
cg21972431	0.70616	0.857491892	0.280128	0.039173	hypermethylated
cg06334484	0.53551	0.650258378	0.2801	0.000756	hypermethylated
cg11281005	0.75377	0.915280541	0.28009	0.014372	hypermethylated
cg15883603	0.616	0.747924324	0.279962	0.001423	hypermethylated
cg02377941	0.50622	0.614622703	0.279937	8.42E-05	hypermethylated
cg04812726	0.62757	0.761853514	0.279737	0.008491	hypermethylated
cg07872947	0.6367	0.77288	0.279631	0.003773	hypermethylated
cg01034631	0.59119	0.717501622	0.27936	0.013589	hypermethylated
cg03229033	0.41632	0.505247568	0.279298	0.000104	hypermethylated
cg25985263	0.51849	0.629187568	0.279174	0.001118	hypermethylated
cg06707356	0.69971	0.849077297	0.279139	0.015809	hypermethylated
cg24380053	0.61452	0.745564324	0.278873	0.010642	hypermethylated
cg00210133	0.65144	0.790240541	0.27866	0.006015	hypermethylated
cg04423294	0.61979	0.751806486	0.278582	0.007799	hypermethylated
cg19259125	0.74814	0.907472432	0.278546	0.016835	hypermethylated
cg04944090	0.72497	0.879333514	0.278489	0.001541	hypermethylated
cg20335293	0.63873	0.774612432	0.278268	0.003773	hypermethylated
cg23346969	0.52998	0.642696757	0.2782	0.002007	hypermethylated
cg19002907	0.66099	0.801556757	0.278176	0.000262	hypermethylated
cg12154110	0.54949	0.666332973	0.27815	0.001571	hypermethylated
cg00575672	0.63922	0.775096757	0.278064	0.003441	hypermethylated
cg20669572	0.56343	0.68319027	0.278051	2.32E-05	hypermethylated
cg17924901	0.60263	0.730628108	0.277865	0.000831	hypermethylated
cg02197303	0.64431	0.780943784	0.277464	0.001667	hypermethylated
cg00582941	0.55648	0.674378378	0.277228	0.02988	hypermethylated
cg04880278	0.59245	0.717957297	0.277205	0.003136	hypermethylated
cg15245556	0.63313	0.767214054	0.277127	0.0006	hypermethylated
cg00883123	0.61187	0.741421622	0.277069	0.002526	hypermethylated
cg02460314	0.6983	0.846132973	0.277037	0.004287	hypermethylated
cg04870949	0.7118	0.862450811	0.27697	0.001571	hypermethylated
cg02430183	0.68361	0.828274595	0.276936	0.00097	hypermethylated
cg01591037	0.69597	0.843206486	0.276861	0.000204	hypermethylated
cg11418681	0.63142	0.764944865	0.276756	0.002965	hypermethylated
cg03416228	0.54357	0.658504324	0.276727	0.002106	hypermethylated
cg05964918	0.69987	0.847699459	0.276466	0.002253	hypermethylated
cg27058882	0.53242	0.644718378	0.276104	0.000912	hypermethylated
cg11466131	0.69621	0.843033514	0.276067	0.005085	hypermethylated
cg06469895	0.5813	0.703838919	0.275962	0.00094	hypermethylated
cg23230158	0.68375	0.827534054	0.27535	0.003136	hypermethylated
cg21101386	0.60709	0.734676216	0.275198	0.006175	hypermethylated
cg08080923	0.74176	0.897647027	0.275196	0.001603	hypermethylated
cg09220563	0.68307	0.826618378	0.275188	0.000931	hypermethylated
cg26348521	0.67606	0.818126486	0.275173	0.005756	hypermethylated
cg11988604	0.60252	0.729120541	0.275148	0.016835	hypermethylated
cg11442877	0.62144	0.751957297	0.275036	0.007408	hypermethylated

cg00316312	0.49994	0.604862703	0.274853	0.003078	hypermethylated
cg12658374	0.63327	0.766082703	0.274679	6.31E-05	hypermethylated
cg00905220	0.70854	0.857119459	0.274647	0.006855	hypermethylated
cg10179363	0.67894	0.821302703	0.27463	0.02272	hypermethylated
cg17295053	0.62196	0.752355676	0.274593	0.006975	hypermethylated
cg04029933	0.71185	0.86097027	0.27439	0.013265	hypermethylated
cg08092050	0.71441	0.863992432	0.274266	0.002274	hypermethylated
cg05778278	0.65115	0.787451892	0.274202	0.000875	hypermethylated
cg25680629	0.65178	0.788203243	0.274183	0.011649	hypermethylated
cg26284985	0.66104	0.799247568	0.273905	0.012036	hypermethylated
cg17086686	0.62331	0.753605946	0.27386	0.000129	hypermethylated
cg02333960	0.67974	0.82176	0.273734	0.002253	hypermethylated
cg23224619	0.57864	0.699531892	0.273724	0.00064	hypermethylated
cg17192381	0.67965	0.821604865	0.273653	0.005606	hypermethylated
cg20227326	0.69085	0.835115676	0.273604	0.001768	hypermethylated
cg04732596	0.606	0.732515676	0.273542	0.014604	hypermethylated
cg13977835	0.58613	0.708208108	0.272953	0.000696	hypermethylated
cg21631754	0.67005	0.809464324	0.272699	0.010642	hypermethylated
cg16686174	0.68478	0.827203784	0.272602	0.001225	hypermethylated
cg00661855	0.57647	0.696336757	0.27254	0.024128	hypermethylated
cg26681523	0.72066	0.870268108	0.272141	0.004287	hypermethylated
cg10604373	0.69303	0.836763243	0.271902	0.000613	hypermethylated
cg07492143	0.67579	0.815917297	0.271848	0.001395	hypermethylated
cg22288657	0.68823	0.830926486	0.27183	0.015438	hypermethylated
cg14024893	0.73069	0.882178378	0.271811	0.000204	hypermethylated
cg22860848	0.57018	0.688381622	0.271791	0.02324	hypermethylated
cg06186808	0.67188	0.810687568	0.270942	0.000271	hypermethylated
cg05062620	0.60399	0.728762703	0.270924	0.008783	hypermethylated
cg15473155	0.59003	0.711902703	0.270892	7.93E-05	hypermethylated
cg03983058	0.679	0.819237838	0.270871	0.007732	hypermethylated
cg14029580	0.69929	0.843647027	0.270749	0.002253	hypermethylated
cg05375744	0.64205	0.774499459	0.270579	0.00078	hypermethylated
cg15107132	0.5326	0.642466486	0.270569	0.003195	hypermethylated
cg00352652	0.74121	0.894081081	0.270523	0.002699	hypermethylated
cg16557964	0.67508	0.814271351	0.270451	0.006451	hypermethylated
cg06582663	0.78671	0.948885405	0.270402	0.001073	hypermethylated
cg14014225	0.6492	0.782950811	0.270259	0.000335	hypermethylated
cg22918700	0.65818	0.793749189	0.270201	0.000152	hypermethylated
cg26804891	0.66006	0.795956216	0.270092	0.000733	hypermethylated
cg12666874	0.67711	0.816509189	0.270079	0.003506	hypermethylated
cg21408624	0.67934	0.819121622	0.269944	0.000506	hypermethylated
cg09399801	0.70515	0.850171351	0.269823	0.016835	hypermethylated
cg08888487	0.67031	0.808108649	0.269721	0.001288	hypermethylated
cg05380759	0.57111	0.688504324	0.269697	0.0003	hypermethylated
cg05265884	0.58763	0.708385405	0.269626	0.000688	hypermethylated
cg09823576	0.68368	0.824162162	0.269607	0.007799	hypermethylated
cg13149442	0.71152	0.857631351	0.269453	0.01021	hypermethylated
cg00382227	0.71988	0.867692432	0.269427	0.002189	hypermethylated
cg22519702	0.7326	0.883008649	0.269402	0.030759	hypermethylated
cg23740135	0.60428	0.728335676	0.269386	0.001803	hypermethylated
cg03924483	0.52817	0.636594054	0.269371	0.020119	hypermethylated
cg08313393	0.62437	0.752528108	0.269344	0.032586	hypermethylated
cg14614094	0.57379	0.691411892	0.269023	7.84E-05	hypermethylated
cg09263875	0.61162	0.736984324	0.268998	0.002455	hypermethylated
cg02036494	0.59135	0.712508108	0.268894	0.020586	hypermethylated
cg14038009	0.58386	0.703419459	0.268763	0.000789	hypermethylated
cg15600176	0.62604	0.754222703	0.268736	0.002478	hypermethylated
cg05257275	0.6426	0.77417027	0.26873	0.007158	hypermethylated

cg14975238	0.59883	0.721371892	0.268597	0.022212	hypermethylated
cg12013685	0.54102	0.651691351	0.268507	0.003506	hypermethylated
cg08351085	0.69506	0.837093514	0.268251	0.01073	hypermethylated
cg27080348	0.70239	0.845869189	0.268162	0.008208	hypermethylated
cg22245862	0.55274	0.665646486	0.268155	0.001	hypermethylated
cg01367097	0.69482	0.836727027	0.268118	0.001912	hypermethylated
cg01069808	0.71516	0.861126486	0.267959	0.001684	hypermethylated
cg00305996	0.68038	0.81919027	0.267858	0.02238	hypermethylated
cg04718883	0.75238	0.905442162	0.267161	0.00193	hypermethylated
cg06107104	0.56126	0.675344865	0.266955	0.048145	hypermethylated
cg24370974	0.68567	0.824989189	0.266861	0.004172	hypermethylated
cg07766743	0.64021	0.770255135	0.266791	0.001839	hypermethylated
cg20277356	0.72127	0.867742703	0.266728	0.00032	hypermethylated
cg14297867	0.73216	0.880645405	0.266402	0.001188	hypermethylated
cg10805220	0.46062	0.55402	0.266361	0.00043	hypermethylated
cg00709112	0.7063	0.849494054	0.266323	0.012233	hypermethylated
cg03075050	0.50804	0.610905405	0.266007	6.01E-05	hypermethylated
cg07238439	0.5874	0.706308649	0.265955	0.007667	hypermethylated
cg26661718	0.59199	0.711821081	0.265942	0.004906	hypermethylated
cg02392025	0.68976	0.829327027	0.265847	0.013159	hypermethylated
cg21165137	0.71283	0.857048108	0.265818	0.013265	hypermethylated
cg00767886	0.69449	0.834943784	0.265725	0.001768	hypermethylated
cg15428835	0.62482	0.751096216	0.265557	0.001423	hypermethylated
cg08705483	0.58485	0.702960541	0.265377	0.013699	hypermethylated
cg11030420	0.7149	0.859260541	0.265354	3.85E-05	hypermethylated
cg14904079	0.75349	0.905526486	0.265168	0.005039	hypermethylated
cg16307965	0.69979	0.840951892	0.265101	0.000187	hypermethylated
cg01255486	0.72525	0.871536216	0.265082	0.007345	hypermethylated
cg12531838	0.71589	0.86012973	0.264816	0.001839	hypermethylated
cg05490233	0.65541	0.787455135	0.2648	0.000789	hypermethylated
cg11776930	0.60623	0.728159459	0.264389	0.016835	hypermethylated
cg10314760	0.71943	0.864087027	0.264322	0.001096	hypermethylated
cg22624278	0.55409	0.665414595	0.264133	0.001839	hypermethylated
cg22795216	0.79725	0.957287027	0.263919	0.001618	hypermethylated
cg21296675	0.46504	0.558375135	0.26388	0.00012	hypermethylated
cg12589486	0.74643	0.895952432	0.263415	0.000399	hypermethylated
cg17924366	0.58141	0.697788649	0.263234	0.012233	hypermethylated
cg01093311	0.70682	0.848239459	0.263129	5.31E-05	hypermethylated
cg09034646	0.66273	0.795257297	0.263	0.024674	hypermethylated
cg06170425	0.73918	0.886912432	0.262866	0.000306	hypermethylated
cg09833545	0.66997	0.803756757	0.262662	0.008783	hypermethylated
cg04195548	0.66615	0.799072973	0.26248	0.002856	hypermethylated
cg02914667	0.69881	0.838234054	0.262453	0.01392	hypermethylated
cg04525408	0.58125	0.696971892	0.261942	0.018918	hypermethylated
cg06597095	0.71823	0.861162703	0.26184	0.001821	hypermethylated
cg10522818	0.48721	0.584114595	0.261708	0.000134	hypermethylated
cg06529761	0.56018	0.671595676	0.261702	0.001619	hypermethylated
cg23117727	0.66387	0.795781081	0.261471	0.005606	hypermethylated
cg03574652	0.62519	0.749395135	0.261432	0.000588	hypermethylated
cg03552025	0.56715	0.679602162	0.26096	0.02542	hypermethylated
cg08569517	0.66382	0.795396757	0.260883	0.000324	hypermethylated
cg03337430	0.74817	0.896221081	0.260489	0.005039	hypermethylated
cg11075176	0.74873	0.896833514	0.260395	0.014145	hypermethylated
cg10633051	0.64722	0.775194054	0.260301	0.002409	hypermethylated
cg26951614	0.72404	0.867141081	0.260197	0.000464	hypermethylated
cg13728069	0.75234	0.901024324	0.260181	0.01146	hypermethylated
cg04303439	0.65509	0.784506486	0.260092	0.008932	hypermethylated
cg15067606	0.6784	0.812318378	0.259909	0.000378	hypermethylated

cg23640615	0.70708	0.846496757	0.259631	0.023769	hypermethylated
cg02519879	0.61224	0.732838378	0.259398	0.03212	hypermethylated
cg01410314	0.66119	0.791379459	0.259305	0.039172	hypermethylated
cg02412684	0.68206	0.81625027	0.259113	0.004526	hypermethylated
cg27428744	0.74281	0.888797838	0.258862	0.006451	hypermethylated
cg01663563	0.65983	0.789395135	0.258653	0.003316	hypermethylated
cg14091372	0.61457	0.735228108	0.258615	0.000551	hypermethylated
cg17463655	0.62446	0.746971351	0.258444	0.010126	hypermethylated
cg23029851	0.58957	0.705109189	0.258184	0.034015	hypermethylated
cg12641165	0.67274	0.804576216	0.25818	0.006621	hypermethylated
cg25007447	0.6885	0.823213514	0.25781	0.001423	hypermethylated
cg24818418	0.68593	0.820124865	0.257782	0.021062	hypermethylated
cg09514524	0.74196	0.887038919	0.257656	0.015931	hypermethylated
cg11751213	0.64776	0.774383784	0.257589	0.001288	hypermethylated
cg07381141	0.62406	0.746038378	0.257565	0.039172	hypermethylated
cg15040499	0.73074	0.873560541	0.25755	0.01792	hypermethylated
cg16437211	0.66749	0.797892973	0.257449	0.000251	hypermethylated
cg13752649	0.64328	0.768886486	0.257324	0.007732	hypermethylated
cg09286367	0.77499	0.926136757	0.257048	0.00015	hypermethylated
cg24477315	0.60876	0.727470811	0.257016	0.003255	hypermethylated
cg03191794	0.70256	0.839504865	0.256917	0.008419	hypermethylated
cg23658709	0.73155	0.874034054	0.256733	0.029234	hypermethylated
cg21486233	0.71777	0.85754973	0.256699	0.00504	hypermethylated
cg04696969	0.73448	0.877471892	0.25663	6.86E-05	hypermethylated
cg03190911	0.59312	0.708535135	0.256515	0.004734	hypermethylated
cg01325188	0.62363	0.744846486	0.256253	0.004951	hypermethylated
cg11115431	0.63823	0.762281622	0.256248	0.000287	hypermethylated
cg25069596	0.63644	0.759840541	0.255672	0.009552	hypermethylated
cg02276973	0.73726	0.880165946	0.255602	0.000378	hypermethylated
cg16992627	0.64404	0.768744865	0.255355	0.000268	hypermethylated
cg25383605	0.58004	0.692	0.25462	0.000374	hypermethylated
cg10179300	0.63278	0.7549	0.254582	0.000875	hypermethylated
cg14757661	0.69602	0.830344324	0.254581	0.016444	hypermethylated
cg04281204	0.72871	0.869229189	0.254392	0.008783	hypermethylated
cg23551917	0.53231	0.634936216	0.254345	0.003225	hypermethylated
cg08549332	0.663	0.790736216	0.254188	0.000511	hypermethylated
cg01693830	0.62536	0.745767027	0.254038	0.009315	hypermethylated
cg17221945	0.73043	0.870948649	0.253842	0.012844	hypermethylated
cg27638288	0.71619	0.853942703	0.253797	0.000789	hypermethylated
cg20649716	0.46865	0.558784865	0.253782	0.000511	hypermethylated
cg07011231	0.72025	0.858720541	0.253691	0.000256	hypermethylated
cg25946538	0.56292	0.67108973	0.253576	0.004287	hypermethylated
cg10193069	0.72296	0.861838378	0.253502	0.019511	hypermethylated
cg25575937	0.679	0.809402703	0.253446	0.026575	hypermethylated
cg12347480	0.74542	0.888530811	0.253368	0.001619	hypermethylated
cg06449094	0.6776	0.807527568	0.253078	0.000741	hypermethylated
cg01280182	0.60857	0.725131892	0.25282	0.000219	hypermethylated
cg09357140	0.72496	0.863723243	0.252668	0.026575	hypermethylated
cg14552982	0.63741	0.759254054	0.252361	0.003637	hypermethylated
cg19983948	0.66347	0.790167027	0.252126	0.011745	hypermethylated
cg05328670	0.68811	0.819437297	0.251994	0.005316	hypermethylated
cg12465501	0.70925	0.844581622	0.251943	0.001496	hypermethylated
cg24408200	0.64424	0.767162703	0.251934	0.002386	hypermethylated
cg16472542	0.7169	0.853636216	0.251849	0.001063	hypermethylated
cg01110440	0.69096	0.822736757	0.251829	0.002599	hypermethylated
cg24046689	0.803	0.956019459	0.25164	0.000147	hypermethylated
cg01962750	0.65119	0.775279459	0.251638	4.42E-05	hypermethylated
cg07362258	0.72738	0.865844324	0.251398	0.000741	hypermethylated

cg08947167	0.63792	0.759244324	0.251189	0.000365	hypermethylated
cg18348494	0.65165	0.775576757	0.251172	0.009552	hypermethylated
cg08722638	0.7449	0.886554595	0.251163	0.003136	hypermethylated
cg09467345	0.64951	0.772964324	0.25105	0.008069	hypermethylated
cg09310065	0.65659	0.781289189	0.250864	0.001225	hypermethylated
cg21049487	0.71748	0.853687568	0.25077	0.001395	hypermethylated
cg26997966	0.72418	0.861535676	0.250562	9.31E-06	hypermethylated
cg02703822	0.77656	0.923845405	0.250554	0.002408	hypermethylated
cg05966235	0.74532	0.886581622	0.250393	0.000581	hypermethylated
cg02287325	0.66135	0.786665946	0.250337	0.003604	hypermethylated
cg08433095	0.68171	0.810874595	0.250321	0.012434	hypermethylated
cg17827208	0.56091	0.667168108	0.250281	0.000335	hypermethylated
cg11361442	0.71454	0.849794595	0.250099	0.011938	hypermethylated
cg18501647	0.57872	0.688145946	0.249849	7.03E-05	hypermethylated
cg16218477	0.68245	0.811471892	0.249818	0.006122	hypermethylated
cg06535102	0.73939	0.879154054	0.24978	0.003505	hypermethylated
cg05475485	0.70146	0.834015676	0.249714	0.015685	hypermethylated
cg19543317	0.62741	0.74597027	0.24971	4.64E-05	hypermethylated
cg25411239	0.71581	0.851008649	0.249597	0.002231	hypermethylated
cg13914024	0.6823	0.811151351	0.249565	0.008783	hypermethylated
cg23476802	0.601	0.714358378	0.249283	0.010211	hypermethylated
cg13859860	0.64228	0.76341027	0.249256	0.020902	hypermethylated
cg10478035	0.70416	0.836944324	0.249228	1.75E-05	hypermethylated
cg07710971	0.76761	0.912281081	0.249105	0.00101	hypermethylated
cg26389281	0.63139	0.750282162	0.248902	0.002066	hypermethylated
cg06528737	0.69614	0.827091351	0.248669	0.006175	hypermethylated
cg22188918	0.57138	0.678792432	0.24852	0.000168	hypermethylated
cg07148651	0.73756	0.876164865	0.248442	0.002803	hypermethylated
cg15271026	0.71341	0.847458378	0.248411	0.002318	hypermethylated
cg02976898	0.73331	0.871058378	0.248346	0.016573	hypermethylated
cg20333001	0.72122	0.8563	0.247677	0.016704	hypermethylated
cg23216434	0.72858	0.864984865	0.247587	0.003843	hypermethylated
cg08963581	0.73709	0.874952432	0.247364	5.00E-05	hypermethylated
cg12165223	0.68335	0.811137297	0.247321	0.029449	hypermethylated
cg24368408	0.74553	0.884735135	0.246979	0.001541	hypermethylated
cg21915998	0.73282	0.869411892	0.246581	0.001556	hypermethylated
cg25096740	0.66931	0.793914054	0.246308	0.005508	hypermethylated
cg16733813	0.59385	0.704397297	0.246291	0.001395	hypermethylated
cg11300341	0.69958	0.829786486	0.246251	0.009084	hypermethylated
cg04270085	0.70837	0.840173514	0.246184	3.67E-05	hypermethylated
cg20696432	0.57473	0.681664324	0.246177	0.006975	hypermethylated
cg27097660	0.6156	0.730054595	0.246011	0.019661	hypermethylated
cg27230984	0.56358	0.668354595	0.245993	0.004649	hypermethylated
cg25219728	0.74916	0.888427568	0.24598	0.001	hypermethylated
cg14571493	0.71292	0.845448108	0.245976	0.034014	hypermethylated
cg00163006	0.66435	0.787624324	0.245564	0.001968	hypermethylated
cg07584981	0.63842	0.756616757	0.245057	0.011554	hypermethylated
cg24782949	0.7355	0.871500541	0.244776	0.041413	hypermethylated
cg04539957	0.56691	0.671667027	0.244626	0.000166	hypermethylated
cg02478836	0.61006	0.722580541	0.244207	0.012948	hypermethylated
cg13597544	0.63792	0.755507027	0.24407	0.00039	hypermethylated
cg02927058	0.74423	0.881296216	0.243878	0.007282	hypermethylated
cg18774692	0.63672	0.753865405	0.243648	0.001381	hypermethylated
cg08511818	0.67829	0.802898919	0.243316	0.001153	hypermethylated
cg07326665	0.7362	0.87127027	0.243023	0.014488	hypermethylated
cg15973818	0.65271	0.772328108	0.242772	0.010126	hypermethylated
cg00733001	0.74103	0.876821622	0.242751	0.000378	hypermethylated
cg00783000	0.66436	0.786089189	0.242728	0.006796	hypermethylated

cg01869554	0.67694	0.800940541	0.242667	0.010909	hypermethylated
cg05471509	0.74964	0.886848649	0.24249	0.001452	hypermethylated
cg06023295	0.55913	0.661449189	0.242447	0.000306	hypermethylated
cg23679332	0.71189	0.842104324	0.242345	0.015317	hypermethylated
cg26624744	0.68637	0.811909189	0.242332	0.007035	hypermethylated
cg23246769	0.75189	0.889398378	0.242308	0.01146	hypermethylated
cg06625077	0.6724	0.795275676	0.242135	0.006621	hypermethylated
cg10639428	0.55002	0.650421622	0.241891	0.007158	hypermethylated
cg09441069	0.72204	0.853685946	0.241627	0.005411	hypermethylated
cg13496660	0.77157	0.912198378	0.241551	0.001838	hypermethylated
cg09537792	0.74448	0.880033514	0.241325	0.004287	hypermethylated
cg22959054	0.64256	0.759511892	0.241241	0.000265	hypermethylated
cg23563866	0.67261	0.795011351	0.241205	0.036003	hypermethylated
cg11081251	0.64224	0.759087568	0.241154	0.001768	hypermethylated
cg23303685	0.78404	0.926591892	0.241007	0.026969	hypermethylated
cg18988856	0.7473	0.883113514	0.240911	0.020273	hypermethylated
cg26090619	0.5276	0.62342973	0.240782	0.000268	hypermethylated
cg05729352	0.65597	0.775086486	0.240727	0.000839	hypermethylated
cg25241823	0.62916	0.743204324	0.240332	0.001381	hypermethylated
cg05012672	0.79204	0.935607568	0.24033	0.001423	hypermethylated
cg07271473	0.69786	0.824334595	0.240292	0.004248	hypermethylated
cg21597025	0.70089	0.827842162	0.240168	0.000674	hypermethylated
cg03885098	0.79607	0.940222162	0.240106	0.000626	hypermethylated
cg20617977	0.67518	0.797436757	0.240098	6.46E-05	hypermethylated
cg00022235	0.68967	0.814347568	0.239738	0.01021	hypermethylated
cg27279583	0.65801	0.776951351	0.239715	0.015196	hypermethylated
cg11067712	0.76165	0.899260541	0.239611	0.025232	hypermethylated
cg03261948	0.67071	0.791885946	0.239604	0.000256	hypermethylated
cg07618581	0.62204	0.734390811	0.239541	0.006679	hypermethylated
cg06567920	0.63516	0.749863784	0.239508	0.000575	hypermethylated
cg09850764	0.75132	0.886942162	0.239413	0.000756	hypermethylated
cg16308533	0.75646	0.892995135	0.239389	0.000224	hypermethylated
cg14472390	0.60971	0.719754595	0.239382	0.00546	hypermethylated
cg05038288	0.60903	0.718935135	0.239348	0.001667	hypermethylated
cg07906527	0.70192	0.828471892	0.239146	0.001541	hypermethylated
cg04860563	0.71838	0.847777297	0.238938	0.00012	hypermethylated
cg25225155	0.69506	0.820224324	0.238881	0.000681	hypermethylated
cg17902007	0.82205	0.969992973	0.238748	0.001602	hypermethylated
cg23508264	0.7928	0.935464865	0.238727	0.000725	hypermethylated
cg15080870	0.77239	0.911365405	0.2387	0.001667	hypermethylated
cg18060030	0.71727	0.846287568	0.238632	0.001381	hypermethylated
cg19018435	0.67663	0.798209189	0.2384	0.003078	hypermethylated
cg11423909	0.74974	0.884329189	0.238193	0.001188	hypermethylated
cg05140624	0.62347	0.73503027	0.237484	0.000358	hypermethylated
cg10319073	0.68249	0.804535135	0.237348	0.000594	hypermethylated
cg14146657	0.69282	0.816523784	0.237014	0.034014	hypermethylated
cg09562563	0.75977	0.895372973	0.236926	0.003473	hypermethylated
cg14709826	0.6808	0.802237838	0.236799	0.002363	hypermethylated
cg26921533	0.66451	0.782813514	0.236378	0.002189	hypermethylated
cg05554151	0.76826	0.904984324	0.236298	0.005085	hypermethylated
cg07314409	0.68769	0.809924324	0.236029	0.00099	hypermethylated
cg00330953	0.58322	0.686876757	0.236011	0.001893	hypermethylated
cg22399111	0.72151	0.84968	0.2359	0.001368	hypermethylated
cg22457637	0.65613	0.772661622	0.235855	0.039723	hypermethylated
cg11544647	0.47007	0.553462162	0.235609	0.006068	hypermethylated
cg19871722	0.69855	0.822405946	0.235487	0.005364	hypermethylated
cg16720807	0.70583	0.830878378	0.235317	0.008208	hypermethylated
cg07605229	0.7572	0.891291892	0.235224	0.009315	hypermethylated

cg15488143	0.68857	0.810453514	0.235126	0.00071	hypermethylated
cg10164795	0.72313	0.851059459	0.235005	0.002455	hypermethylated
cg01526355	0.6192	0.728694054	0.234908	0.000674	hypermethylated
cg18487598	0.71348	0.839615135	0.234855	0.000588	hypermethylated
cg27284034	0.68458	0.805434054	0.234547	0.002106	hypermethylated
cg10909506	0.74141	0.872277838	0.234516	0.011649	hypermethylated
cg09499844	0.81192	0.955216216	0.23449	0.002525	hypermethylated
cg26988853	0.69248	0.814686486	0.234473	0.001635	hypermethylated
cg04012282	0.63396	0.745793514	0.234384	8.03E-05	hypermethylated
cg15684162	0.7731	0.909443784	0.234329	0.000221	hypermethylated
cg05324407	0.62164	0.731198919	0.234185	0.000117	hypermethylated
cg18450832	0.73764	0.867592432	0.234101	0.000469	hypermethylated
cg20773956	0.74962	0.881534595	0.233858	0.00096	hypermethylated
cg01291590	0.60296	0.709038919	0.233803	0.000575	hypermethylated
cg18066946	0.73784	0.867602703	0.233727	0.002318	hypermethylated
cg22479226	0.60332	0.709402162	0.23368	5.06E-05	hypermethylated
cg19280364	0.6693	0.786882162	0.233495	0.007866	hypermethylated
cg06330593	0.71929	0.845645405	0.233479	0.005222	hypermethylated
cg20441606	0.76335	0.897424324	0.233446	0.001571	hypermethylated
cg04704085	0.80446	0.945701081	0.233364	0.001063	hypermethylated
cg22815766	0.61571	0.723811351	0.233363	0.000931	hypermethylated
cg18065874	0.71986	0.846097297	0.233107	0.000426	hypermethylated
cg05883442	0.54393	0.639304324	0.233082	0.009238	hypermethylated
cg02209075	0.76042	0.893724324	0.233033	0.000253	hypermethylated
cg08423575	0.65382	0.768432432	0.233025	0.015317	hypermethylated
cg24902995	0.81836	0.961711351	0.232868	8.52E-05	hypermethylated
cg20302957	0.6892	0.809895135	0.232812	0.008783	hypermethylated
cg21129181	0.59232	0.696027027	0.232767	0.001096	hypermethylated
cg26649504	0.72568	0.852711892	0.232725	0.001153	hypermethylated
cg24176760	0.73706	0.865943784	0.232491	0.010909	hypermethylated
cg17367596	0.65661	0.771363243	0.232374	0.019065	hypermethylated
cg09491542	0.71824	0.843754595	0.232357	0.000135	hypermethylated
cg23916284	0.69691	0.818661622	0.232295	0.002725	hypermethylated
cg10533159	0.75044	0.881402703	0.232065	0.043763	hypermethylated
cg19701264	0.48613	0.570891351	0.231874	0.006796	hypermethylated
cg06389888	0.68152	0.800306486	0.231797	0.010554	hypermethylated
cg01831743	0.62414	0.732847027	0.231642	0.005131	hypermethylated
cg06579738	0.70958	0.833123784	0.231566	0.000207	hypermethylated
cg06528626	0.72058	0.846005405	0.231508	0.000667	hypermethylated
cg23332586	0.71061	0.83427027	0.231457	0.005963	hypermethylated
cg24815973	0.72864	0.855425946	0.231437	0.000335	hypermethylated
cg18963171	0.68773	0.807246486	0.231167	0.01109	hypermethylated
cg08217589	0.68088	0.799174054	0.231109	0.010819	hypermethylated
cg19125584	0.64051	0.751602703	0.230749	0.001734	hypermethylated
cg25751484	0.71879	0.843304865	0.230484	0.00035	hypermethylated
cg02307288	0.61618	0.722887568	0.230419	0.000696	hypermethylated
cg12141056	0.66795	0.783467027	0.230132	0.005316	hypermethylated
cg03187713	0.75425	0.884572973	0.229938	0.006679	hypermethylated
cg02451691	0.72835	0.854145405	0.22985	0.004134	hypermethylated
cg12027899	0.64306	0.754065946	0.229737	0.003773	hypermethylated
cg04159077	0.69839	0.818823784	0.22952	0.00504	hypermethylated
cg05027554	0.72975	0.855577838	0.229497	0.000374	hypermethylated
cg10613332	0.69591	0.815648108	0.229046	0.001839	hypermethylated
cg05335030	0.80527	0.943114054	0.22796	0.00029	hypermethylated
cg22460443	0.7097	0.831152432	0.227904	0.014956	hypermethylated
cg05314639	0.78605	0.920468108	0.227747	0.007219	hypermethylated
cg00902417	0.61918	0.724844324	0.227312	9.72E-05	hypermethylated
cg04634427	0.64178	0.751274595	0.227261	0.001541	hypermethylated

cg05807722	0.7545	0.883095135	0.227048	0.01073	hypermethylated
cg21300719	0.80113	0.937365405	0.226575	0.007999	hypermethylated
cg08619515	0.59679	0.698243784	0.226507	0.004776	hypermethylated
cg17141972	0.76919	0.899883243	0.226398	0.003195	hypermethylated
cg13079837	0.66495	0.777918919	0.226374	0.02798	hypermethylated
cg05655106	0.67437	0.788912432	0.226325	0.002751	hypermethylated
cg15656769	0.62194	0.727483243	0.226139	0.001188	hypermethylated
cg07505680	0.73165	0.855666486	0.225895	0.024491	hypermethylated
cg14377152	0.65241	0.762896757	0.225709	0.00341	hypermethylated
cg14600384	0.6448	0.753948649	0.225615	0.003316	hypermethylated
cg02842158	0.71198	0.832463784	0.225551	0.003195	hypermethylated
cg12031031	0.72223	0.84437027	0.225417	1.42E-05	hypermethylated
cg25191725	0.71559	0.836583784	0.225377	0.010381	hypermethylated
cg24414344	0.60317	0.704942703	0.224941	0.003078	hypermethylated
cg02070077	0.75372	0.880787568	0.224765	0.000245	hypermethylated
cg00344801	0.58288	0.681078378	0.224622	0.001949	hypermethylated
cg23040011	0.73023	0.853220541	0.224568	0.005756	hypermethylated
cg05260959	0.563	0.657818378	0.224554	0.000517	hypermethylated
cg21250061	0.73758	0.861781081	0.224522	0.008348	hypermethylated
cg18493314	0.70722	0.826169189	0.224278	0.002965	hypermethylated
cg25555505	0.77218	0.901946486	0.224105	0.045597	hypermethylated
cg17643662	0.63202	0.738051892	0.223752	0.007158	hypermethylated
cg21966860	0.79963	0.933545405	0.223388	0.000224	hypermethylated
cg24681438	0.67152	0.783744865	0.222954	0.01606	hypermethylated
cg07291387	0.65098	0.759691351	0.2228	0.001619	hypermethylated
cg24711397	0.61296	0.715116216	0.222385	0.012036	hypermethylated
cg00376979	0.70164	0.81856973	0.222374	0.001262	hypermethylated
cg00542638	0.67013	0.781774595	0.222312	0.01792	hypermethylated
cg22617898	0.74676	0.871142703	0.222264	0.00044	hypermethylated
cg23335916	0.67895	0.791929189	0.222066	0.000408	hypermethylated
cg21768835	0.70188	0.818660541	0.222041	0.009713	hypermethylated
cg02508204	0.66055	0.770302162	0.221757	0.003378	hypermethylated
cg13092575	0.68777	0.802036757	0.221742	0.000551	hypermethylated
cg21360079	0.69248	0.807463784	0.221625	0.006229	hypermethylated
cg03422651	0.66673	0.777196216	0.221176	0.001	hypermethylated
cg19076587	0.71246	0.830465946	0.221112	0.000528	hypermethylated
cg18342866	0.67566	0.787515135	0.22101	0.014373	hypermethylated
cg13369939	0.59776	0.696711351	0.220995	7.56E-05	hypermethylated
cg27383651	0.69774	0.812954054	0.220484	0.007799	hypermethylated
cg25544164	0.76102	0.886668649	0.220461	0.004444	hypermethylated
cg06213317	0.64352	0.749612432	0.22016	0.003078	hypermethylated
cg04030444	0.80421	0.93654	0.219768	0.020742	hypermethylated
cg07169046	0.67966	0.791341081	0.219486	0.000464	hypermethylated
cg18901940	0.77167	0.898367568	0.219322	0.00039	hypermethylated
cg02394395	0.68606	0.798696757	0.219313	0.001301	hypermethylated
cg07830644	0.75345	0.877132973	0.219284	0.00591	hypermethylated
cg19003412	0.70869	0.824961622	0.219172	0.006451	hypermethylated
cg07356488	0.71041	0.826828108	0.218936	0.002046	hypermethylated
cg23848152	0.70622	0.821904324	0.218853	0.000116	hypermethylated
cg24709001	0.80938	0.941802162	0.218607	0.001164	hypermethylated
cg18404513	0.71156	0.827687027	0.2181	0.000271	hypermethylated
cg00283857	0.71065	0.82652	0.217911	0.001237	hypermethylated
cg18316735	0.74244	0.863480541	0.217889	0.000613	hypermethylated
cg01498700	0.65057	0.756618919	0.217863	0.00504	hypermethylated
cg08920252	0.78762	0.916008649	0.217861	8.84E-05	hypermethylated
cg06999084	0.72966	0.848494595	0.217681	0.020902	hypermethylated
cg08561825	0.78376	0.911271351	0.217469	0.015684	hypermethylated
cg06120492	0.72459	0.8424	0.217341	0.0006	hypermethylated

cg08709276	0.73906	0.859020541	0.217001	0.001839	hypermethylated
cg05753046	0.75948	0.882709189	0.216926	0.000875	hypermethylated
cg06891164	0.74279	0.863275676	0.216867	0.000145	hypermethylated
cg01826337	0.65892	0.765758919	0.216787	0.025045	hypermethylated
cg20062116	0.64789	0.752755135	0.216432	0.007408	hypermethylated
cg01820007	0.72659	0.844039459	0.216169	0.016835	hypermethylated
cg07106737	0.70761	0.821957838	0.21611	0.000805	hypermethylated
cg03762760	0.791	0.918617838	0.215787	0.000417	hypermethylated
cg05185038	0.71963	0.83573027	0.215782	0.006679	hypermethylated
cg27127645	0.77173	0.896198919	0.215723	0.012637	hypermethylated
cg12480375	0.72933	0.846843784	0.215524	0.006737	hypermethylated
cg07560587	0.76834	0.891952973	0.215223	0.002147	hypermethylated
cg25139636	0.69648	0.808437838	0.215055	8.22E-05	hypermethylated
cg07673807	0.68825	0.798865946	0.215021	0.000178	hypermethylated
cg05106231	0.78138	0.906906486	0.214929	0.009472	hypermethylated
cg02550218	0.74041	0.859223243	0.214709	0.036003	hypermethylated
cg08404546	0.71093	0.824899459	0.214511	0.000408	hypermethylated
cg08481873	0.78567	0.911558378	0.214412	0.018772	hypermethylated
cg09684066	0.68209	0.791336216	0.214329	0.001667	hypermethylated
cg05081395	0.74661	0.866146486	0.214256	0.00291	hypermethylated
cg00852414	0.73286	0.850094054	0.214085	0.005411	hypermethylated
cg10663408	0.80029	0.9281	0.213757	0.014486	hypermethylated
cg22925751	0.67011	0.777030811	0.213574	0.000219	hypermethylated
cg13837335	0.71449	0.828388108	0.213393	0.003195	hypermethylated
cg22615203	0.73842	0.856088108	0.213318	0.033772	hypermethylated
cg26822782	0.69511	0.805770811	0.213128	0.017643	hypermethylated
cg09318162	0.68702	0.796347568	0.213046	0.000306	hypermethylated
cg01369895	0.69316	0.803430811	0.212985	0.003637	hypermethylated
cg03385262	0.77929	0.903064865	0.212669	0.000613	hypermethylated
cg14700684	0.60973	0.706536216	0.212593	0.000718	hypermethylated
cg19600023	0.72857	0.844210811	0.212536	0.00028	hypermethylated
cg26050906	0.66616	0.771892973	0.212532	0.001987	hypermethylated
cg10017626	0.58785	0.681120541	0.212462	0.004022	hypermethylated
cg18298050	0.65701	0.761175676	0.212314	0.010042	hypermethylated
cg26839961	0.72686	0.841861622	0.211906	0.001141	hypermethylated
cg23239647	0.63172	0.731631892	0.211833	0.00064	hypermethylated
cg00324719	0.76267	0.883246486	0.211757	0.002432	hypermethylated
cg03109914	0.7527	0.871681622	0.211726	0.02238	hypermethylated
cg14153069	0.64158	0.742985946	0.211706	0.000104	hypermethylated
cg07444414	0.71427	0.827136216	0.211655	0.00054	hypermethylated
cg03233940	0.71898	0.832497838	0.211495	0.000875	hypermethylated
cg13537240	0.63795	0.738422162	0.211002	0.002086	hypermethylated
cg17415381	0.75201	0.870373514	0.210883	0.018772	hypermethylated
cg08862830	0.69356	0.802682162	0.210808	0.004906	hypermethylated
cg14014964	0.7596	0.878958919	0.210556	0.0017	hypermethylated
cg17960934	0.69421	0.803231351	0.210443	0.003705	hypermethylated
cg07740640	0.6845	0.791778378	0.210046	0.004445	hypermethylated
cg02219383	0.75949	0.878330811	0.209733	0.00048	hypermethylated
cg25363679	0.66879	0.773276216	0.209431	0.000234	hypermethylated
cg13197551	0.68195	0.788488108	0.209423	0.000426	hypermethylated
cg22177840	0.73748	0.852647027	0.209345	0.004022	hypermethylated
cg26795540	0.63982	0.739587027	0.209054	0.000875	hypermethylated
cg03413355	0.73275	0.846534054	0.208247	0.002965	hypermethylated
cg02396253	0.73104	0.844353514	0.207897	0.000725	hypermethylated
cg12578575	0.72887	0.841618378	0.207505	0.030537	hypermethylated
cg04825814	0.77814	0.898457838	0.207421	0.008782	hypermethylated
cg17011300	0.72316	0.834755676	0.207039	0.003843	hypermethylated
cg16730509	0.75233	0.868357297	0.206923	0.007345	hypermethylated

cg12085155	0.72403	0.835657297	0.206862	0.000756	hypermethylated
cg21389119	0.71374	0.823542162	0.206444	0.000921	hypermethylated
cg26917367	0.67463	0.778312432	0.206253	0.000126	hypermethylated
cg07575466	0.80331	0.92657027	0.205944	1.77E-05	hypermethylated
cg04127867	0.7718	0.889722162	0.205128	0.000875	hypermethylated
cg02201132	0.70843	0.816534595	0.204889	0.026575	hypermethylated
cg13613439	0.7368	0.848988649	0.204472	1.66E-05	hypermethylated
cg10271819	0.61435	0.707661622	0.203999	0.000152	hypermethylated
cg16424082	0.72937	0.840104324	0.203918	0.001481	hypermethylated
cg13408597	0.7493	0.863005405	0.203826	0.020586	hypermethylated
cg25447202	0.70449	0.81122	0.203514	0.001188	hypermethylated
cg10888242	0.74728	0.860337297	0.203253	0.00151	hypermethylated
cg13653963	0.69591	0.800732432	0.20242	0.002363	hypermethylated
cg10696504	0.72635	0.835587568	0.202126	0.004776	hypermethylated
cg26504274	0.76938	0.884828108	0.201701	0.021223	hypermethylated
cg23460809	0.68263	0.784924324	0.20145	0.002026	hypermethylated
cg05656566	0.6487	0.745810811	0.201258	0.000191	hypermethylated
cg10205431	0.76851	0.883516757	0.201193	0.000653	hypermethylated
cg16144331	0.59559	0.684697838	0.201148	0.003166	hypermethylated
cg02377021	0.7479	0.859707568	0.201001	0.01109	hypermethylated
cg00625351	0.69518	0.798924865	0.200673	0.00035	hypermethylated
cg15810304	0.76062	0.873940541	0.200359	0.001857	hypermethylated
cg11345811	0.70283	0.807437838	0.200175	0.009552	hypermethylated
cg02747390	0.77958	0.895557838	0.20009	0.006121	hypermethylated
cg20387304	0.74543	0.85631027	0.200061	0.001987	hypermethylated
cg02596427	0.73383	0.842863784	0.199854	2.10E-05	hypermethylated
cg11851382	0.72189	0.829071351	0.199717	0.000234	hypermethylated
cg07873155	0.73762	0.846892432	0.199301	0.012036	hypermethylated
cg16927416	0.65605	0.753004865	0.198853	0.001188	hypermethylated
cg18547333	0.74571	0.855819459	0.198692	0.002106	hypermethylated
cg21236414	0.78006	0.894851351	0.198063	0.00043	hypermethylated
cg04340928	0.74799	0.857348108	0.196862	0.001987	hypermethylated
cg26814650	0.74177	0.850183784	0.196803	0.020903	hypermethylated
cg14125530	0.70682	0.810084324	0.196729	0.000342	hypermethylated
cg08667600	0.74125	0.849008108	0.195818	0.014258	hypermethylated
cg25718223	0.66613	0.762887027	0.195666	0.002026	hypermethylated
cg08199563	0.71153	0.814873514	0.195652	0.004326	hypermethylated
cg11816229	0.67723	0.775461081	0.195408	0.015317	hypermethylated
cg15200412	0.7538	0.863079459	0.195312	0.001212	hypermethylated
cg20392764	0.69799	0.799104865	0.195178	0.024309	hypermethylated
cg27473355	0.63464	0.726505946	0.195036	0.004525	hypermethylated
cg02215776	0.75928	0.868714595	0.19425	0.001164	hypermethylated
cg25974626	0.79474	0.908997838	0.193794	0.000569	hypermethylated
cg25107522	0.61505	0.703190811	0.193213	0.00341	hypermethylated
cg01440556	0.77328	0.883989189	0.193038	0.008138	hypermethylated
cg16014725	0.69555	0.795038378	0.19287	0.023591	hypermethylated
cg19244312	0.77217	0.882429189	0.192562	0.000283	hypermethylated
cg16509192	0.77927	0.890368108	0.192279	0.005606	hypermethylated
cg19402371	0.67484	0.770962162	0.192115	0.000212	hypermethylated
cg16012489	0.78232	0.893644865	0.191943	0.0006	hypermethylated
cg10507231	0.8065	0.921261622	0.191936	0.000101	hypermethylated
cg04009342	0.7533	0.86042	0.191817	8.22E-05	hypermethylated
cg27017251	0.77894	0.889705946	0.191816	0.013698	hypermethylated
cg16437087	0.71467	0.816112432	0.191491	0.001041	hypermethylated
cg22599115	0.78431	0.895611351	0.191449	0.001395	hypermethylated
cg05583014	0.66824	0.763017297	0.191349	0.015196	hypermethylated
cg25356006	0.77545	0.885169189	0.190919	0.000501	hypermethylated
cg05620165	0.68879	0.78599027	0.190447	0.00014	hypermethylated

cg23250157	0.71072	0.81086973	0.190189	0.004951	hypermethylated
cg23723818	0.74871	0.854096757	0.189992	0.005656	hypermethylated
cg17869851	0.79143	0.902335135	0.189202	0.01021	hypermethylated
cg18818210	0.71348	0.813416216	0.189121	0.000741	hypermethylated
cg06740950	0.6923	0.789184865	0.188966	0.000893	hypermethylated
cg12121983	0.69377	0.790776757	0.188813	0.010042	hypermethylated
cg22633932	0.76507	0.871948108	0.188651	0.046857	hypermethylated
cg17881203	0.71983	0.820377297	0.188631	0.002432	hypermethylated
cg04006457	0.60762	0.69246	0.188561	0.00421	hypermethylated
cg17048000	0.60259	0.686514595	0.188114	0.00035	hypermethylated
cg08932381	0.80432	0.916252432	0.187976	0.003808	hypermethylated
cg12034847	0.73565	0.838011351	0.18795	0.006914	hypermethylated
cg19068479	0.79125	0.901178378	0.187679	0.000789	hypermethylated
cg16404259	0.72387	0.8244	0.187614	0.001063	hypermethylated
cg14800197	0.73979	0.842407027	0.187402	0.015809	hypermethylated
cg16719582	0.70039	0.797425946	0.187192	0.019064	hypermethylated
cg21585100	0.81124	0.923522162	0.187018	0.042869	hypermethylated
cg26714129	0.72599	0.826454595	0.186986	0.000741	hypermethylated
cg04217177	0.7159	0.814663784	0.186447	0.001526	hypermethylated
cg14802771	0.77667	0.883701622	0.186258	0.000696	hypermethylated
cg13187820	0.75548	0.85940973	0.185953	0.009632	hypermethylated
cg25279739	0.77169	0.877707568	0.185719	0.002147	hypermethylated
cg16234986	0.76393	0.868794054	0.185574	0.01606	hypermethylated
cg23900070	0.75078	0.853771351	0.18546	0.005269	hypermethylated
cg15880211	0.80423	0.914312973	0.18508	0.012134	hypermethylated
cg08192337	0.76462	0.869251351	0.18503	0.003021	hypermethylated
cg04074001	0.78937	0.897374054	0.185008	0.001603	hypermethylated
cg25762262	0.78393	0.891111351	0.184881	0.029448	hypermethylated
cg15722265	0.6325	0.718975135	0.184876	0.000772	hypermethylated
cg10613684	0.77292	0.878553514	0.184811	0.001949	hypermethylated
cg00177875	0.63337	0.719741081	0.184429	0.000613	hypermethylated
cg00886598	0.77061	0.875501622	0.184109	0.002231	hypermethylated
cg08186575	0.77248	0.877435676	0.183796	0.031207	hypermethylated
cg24232092	0.77778	0.883069189	0.183164	0.002318	hypermethylated
cg07315755	0.76179	0.864398919	0.182304	0.005508	hypermethylated
cg25136408	0.78966	0.895797297	0.181941	0.030537	hypermethylated
cg06371489	0.67238	0.762717838	0.181873	0.00151	hypermethylated
cg13322954	0.72262	0.819678919	0.181822	0.009794	hypermethylated
cg11507187	0.69995	0.793854595	0.181623	0.000797	hypermethylated
cg05520712	0.72481	0.82180973	0.181202	0.000653	hypermethylated
cg11539296	0.68005	0.770478378	0.180114	0.001667	hypermethylated
cg22724228	0.72017	0.815927027	0.180103	0.002066	hypermethylated
cg03860982	0.81878	0.927430811	0.179764	0.001571	hypermethylated
cg24693478	0.72398	0.819918919	0.179531	0.002007	hypermethylated
cg27225130	0.69421	0.786157838	0.179447	0.002026	hypermethylated
cg04295172	0.74461	0.842674595	0.178491	0.005459	hypermethylated
cg08580032	0.74095	0.838228649	0.177968	0.00255	hypermethylated
cg06832406	0.75927	0.858949189	0.17796	0.000293	hypermethylated
cg10567498	0.74462	0.841488108	0.176439	0.003378	hypermethylated
cg01636354	0.71514	0.808151351	0.1764	9.49E-05	hypermethylated
cg13837295	0.82641	0.933652973	0.176029	0.008562	hypermethylated
cg04206025	0.82294	0.929686486	0.175957	0.005962	hypermethylated
cg18330866	0.71062	0.802721081	0.17582	0.001987	hypermethylated
cg00021933	0.79329	0.895591351	0.174992	0.000674	hypermethylated
cg02722434	0.64263	0.725416216	0.174821	0.001821	hypermethylated
cg08996678	0.71827	0.810492973	0.174273	0.00291	hypermethylated
cg18127648	0.76163	0.859012432	0.173589	0.010554	hypermethylated
cg14711433	0.71004	0.800767027	0.173482	0.015561	hypermethylated

cg13707645	0.66907	0.754368108	0.173112	0.007933	hypermethylated
cg24938743	0.7398	0.833694054	0.172383	0.014488	hypermethylated
cg20677171	0.72365	0.81527027	0.171986	3.49E-05	hypermethylated
cg13739115	0.72157	0.812785405	0.171735	0.000789	hypermethylated
cg22941086	0.72771	0.819673514	0.171686	0.002318	hypermethylated
cg24219966	0.74769	0.841965405	0.171321	0.003107	hypermethylated
cg10888399	0.68538	0.771005405	0.169837	0.000857	hypermethylated
cg16190209	0.77828	0.875469189	0.169767	0.002318	hypermethylated
cg00329728	0.74807	0.84141027	0.169636	0.000545	hypermethylated
cg13672348	0.84198	0.947024865	0.169616	0.000137	hypermethylated
cg22512256	0.79649	0.895557297	0.169129	0.01073	hypermethylated
cg08643997	0.646	0.726294595	0.169021	0.022549	hypermethylated
cg18714398	0.76922	0.864443784	0.168376	0.014258	hypermethylated
cg09870004	0.81918	0.920345405	0.167995	0.001466	hypermethylated
cg11629889	0.77579	0.871346486	0.16758	0.001603	hypermethylated
cg04020211	0.83559	0.938154595	0.16703	0.004906	hypermethylated
cg00826626	0.84058	0.943525405	0.166676	0.00849	hypermethylated
cg13185177	0.76242	0.855787027	0.166666	0.00095	hypermethylated
cg17337672	0.79454	0.891784324	0.166575	0.000789	hypermethylated
cg05992600	0.72859	0.817729189	0.166516	0.003136	hypermethylated
cg22417566	0.80438	0.902441081	0.165956	0.014258	hypermethylated
cg25942940	0.78473	0.880128108	0.165517	2.10E-05	hypermethylated
cg13733008	0.75938	0.850571892	0.163611	0.000358	hypermethylated
cg12169568	0.73489	0.822937838	0.163255	0.001603	hypermethylated
cg02449727	0.73831	0.826610811	0.162982	0.004248	hypermethylated
cg07969308	0.75518	0.845145946	0.16238	0.001052	hypermethylated
cg27174778	0.80286	0.898505405	0.162379	0.001354	hypermethylated
cg21715599	0.74718	0.83597027	0.161996	0.016444	hypermethylated
cg14484885	0.75344	0.842708108	0.16154	0.008636	hypermethylated
cg19931529	0.76906	0.859936757	0.161134	0.002066	hypermethylated
cg14091497	0.68773	0.768292432	0.159813	0.000113	hypermethylated
cg24151755	0.72508	0.809991892	0.159767	0.007408	hypermethylated
cg14660788	0.77138	0.861360541	0.159175	4.58E-05	hypermethylated
cg07555125	0.78605	0.877654595	0.159032	0.000335	hypermethylated
cg01536744	0.73195	0.816095676	0.156993	0.003538	hypermethylated
cg02827340	0.7436	0.828938378	0.156738	0.03076	hypermethylated
cg27243166	0.78746	0.877772432	0.15664	0.000506	hypermethylated
cg17528559	0.77927	0.867731892	0.155126	0.00066	hypermethylated
cg26212135	0.79145	0.880895135	0.154472	0.001118	hypermethylated
cg19910231	0.7986	0.888408108	0.153749	0.001875	hypermethylated
cg04839897	0.76913	0.855472973	0.153495	0.003773	hypermethylated
cg15652169	0.73738	0.819522703	0.152376	0.001635	hypermethylated
cg15712323	0.82056	0.911962162	0.152365	0.003136	hypermethylated
cg10763247	0.78174	0.868413514	0.151693	0.001118	hypermethylated
cg16244648	0.75212	0.835221081	0.151195	0.001734	hypermethylated
cg19625388	0.83165	0.922772432	0.149998	0.000551	hypermethylated
cg17214456	0.72294	0.801788649	0.149346	0.002126	hypermethylated
cg11543229	0.81426	0.902107568	0.14781	0.001603	hypermethylated
cg10441640	0.64275	0.711769189	0.147152	0.002253	hypermethylated
cg08570574	0.77121	0.85378973	0.146757	0.015316	hypermethylated
cg12630983	0.80663	0.892402703	0.145788	0.003773	hypermethylated
cg22973172	0.78808	0.870494595	0.143493	0.008932	hypermethylated
cg01903185	0.82692	0.912927568	0.142753	0.001803	hypermethylated
cg23055499	0.79841	0.880959459	0.141946	0.033772	hypermethylated
cg09976876	0.78172	0.861217838	0.139726	0.005176	hypermethylated
cg01993818	0.82054	0.901425946	0.135635	0.00064	hypermethylated
cg08945642	0.81201	0.891944865	0.135457	0.003808	hypermethylated
cg04466022	0.84119	0.923025946	0.13394	0.000789	hypermethylated

cg14398860	0.87097	0.953742703	0.130977	0.033768	hypermethylated
cg27311970	0.80966	0.886197838	0.130313	0.007536	hypermethylated
cg10628201	0.86181	0.942787027	0.129562	0.000733	hypermethylated
cg11494841	0.79763	0.871854054	0.128367	0.004248	hypermethylated
cg24145007	0.82365	0.896972432	0.123032	0.003316	hypermethylated
cg07145834	0.87667	0.791924324	-0.146671	0.000528	hypomethylated
cg08293097	0.88572	0.798316216	-0.14989	0.000893	hypomethylated
cg01219670	0.86508	0.778758919	-0.151657	0.000921	hypomethylated
cg21840908	0.82512	0.742777297	-0.151674	0.001541	hypomethylated
cg18921306	0.87026	0.780291892	-0.157433	0.002649	hypomethylated
cg06920946	0.87873	0.787591351	-0.157973	0.0003	hypomethylated
cg25881591	0.88317	0.791051892	-0.158919	0.001052	hypomethylated
cg08169830	0.87671	0.784707568	-0.159945	0.00024	hypomethylated
cg23020514	0.84904	0.758850811	-0.162016	0.002699	hypomethylated
cg11699334	0.79787	0.712320541	-0.163627	0.006451	hypomethylated
cg27648270	0.83498	0.742847568	-0.168675	0.001188	hypomethylated
cg19156963	0.8418	0.748791351	-0.168914	0.000449	hypomethylated
cg14281092	0.88409	0.786220541	-0.169259	0.000839	hypomethylated
cg12221621	0.8642	0.768475135	-0.169367	0.001212	hypomethylated
cg08549536	0.88096	0.781161081	-0.173456	0.000374	hypomethylated
cg14751481	0.83381	0.739168108	-0.173816	0.000435	hypomethylated
cg12084235	0.86254	0.764333514	-0.174389	6.78E-05	hypomethylated
cg26978611	0.8494	0.75189027	-0.175922	0.001571	hypomethylated
cg11693986	0.87689	0.776127568	-0.176102	7.12E-05	hypomethylated
cg16799805	0.81058	0.715372973	-0.180259	0.00078	hypomethylated
cg18029039	0.82568	0.728424865	-0.180803	0.000474	hypomethylated
cg13753183	0.87604	0.772852973	-0.180803	6.05E-06	hypomethylated
cg10532384	0.83148	0.733341081	-0.181197	0.000893	hypomethylated
cg26639730	0.84916	0.748254054	-0.182508	0.000417	hypomethylated
cg25270574	0.92257	0.812825946	-0.182712	4.86E-06	hypomethylated
cg11631579	0.78488	0.690852973	-0.184093	0.019511	hypomethylated
cg20553938	0.87887	0.773031351	-0.185123	1.40E-05	hypomethylated
cg07173218	0.84188	0.739802162	-0.186475	0.000259	hypomethylated
cg15207055	0.82094	0.721316216	-0.186645	0.000551	hypomethylated
cg03001333	0.85518	0.751378378	-0.186689	0.001541	hypomethylated
cg17896636	0.9577	0.841444865	-0.186705	6.86E-05	hypomethylated
cg10626896	0.86276	0.757910811	-0.186931	0.000528	hypomethylated
cg10284115	0.88057	0.773460541	-0.18711	8.13E-05	hypomethylated
cg19016972	0.84824	0.744581081	-0.188044	0.000342	hypomethylated
cg22272100	0.86035	0.754530811	-0.189344	2.85E-05	hypomethylated
cg19392379	0.78352	0.686682703	-0.190326	0.002106	hypomethylated
cg18765405	0.87407	0.765993514	-0.190417	0.00032	hypomethylated
cg19110982	0.83322	0.729952973	-0.190894	1.84E-05	hypomethylated
cg14789529	0.85443	0.747952432	-0.192016	0.003705	hypomethylated
cg21138550	0.84527	0.739932432	-0.192019	9.27E-05	hypomethylated
cg22614327	0.8243	0.720999459	-0.193171	0.003136	hypomethylated
cg15012607	0.87583	0.765842703	-0.193603	2.32E-05	hypomethylated
cg21935113	0.84584	0.7395	-0.193835	0.000848	hypomethylated
cg13008935	0.85051	0.743305405	-0.194373	8.94E-05	hypomethylated
cg16241867	0.77666	0.678709189	-0.19449	0.003316	hypomethylated
cg22734451	0.73829	0.644924865	-0.195057	0.000893	hypomethylated
cg24003866	0.85132	0.743089189	-0.196166	0.0003	hypomethylated
cg24712244	0.85149	0.743203784	-0.196232	0.00049	hypomethylated
cg20385001	0.8439	0.735801622	-0.197755	0.000575	hypomethylated
cg24950003	0.89691	0.781878919	-0.198018	0.00014	hypomethylated
cg16612995	0.8658	0.754260541	-0.198971	1.68E-05	hypomethylated
cg19897979	0.86664	0.754956216	-0.19904	0.000191	hypomethylated
cg15993652	0.85014	0.740482162	-0.199235	3.40E-05	hypomethylated

cg10703692	0.74658	0.650135676	-0.199556	0.00305	hypomethylated
cg13965612	0.7723	0.672355676	-0.199937	0.002274	hypomethylated
cg04503093	0.87048	0.75773027	-0.200127	0.000435	hypomethylated
cg10109635	0.85869	0.747119459	-0.200798	0.00064	hypomethylated
cg14607208	0.77847	0.677067027	-0.201343	0.000921	hypomethylated
cg25059541	0.85178	0.740723784	-0.201545	6.66E-06	hypomethylated
cg02551234	0.86926	0.755868649	-0.201652	0.000224	hypomethylated
cg26686339	0.76019	0.661014054	-0.201679	0.000528	hypomethylated
cg05954614	0.77183	0.671038378	-0.201888	0.000403	hypomethylated
cg21763718	0.85027	0.738817838	-0.202702	1.04E-05	hypomethylated
cg22550815	0.88976	0.773099459	-0.202762	0.000198	hypomethylated
cg24854914	0.90427	0.785474054	-0.20319	3.81E-05	hypomethylated
cg15065821	0.88673	0.770095135	-0.203458	3.57E-05	hypomethylated
cg07528349	0.7315	0.634952432	-0.204209	0.000182	hypomethylated
cg05829046	0.76084	0.66028	-0.204515	0.000435	hypomethylated
cg23068797	0.77973	0.676114595	-0.205707	0.001541	hypomethylated
cg20837332	0.84164	0.729405946	-0.206481	6.23E-05	hypomethylated
cg19513004	0.88563	0.767348649	-0.206822	9.05E-05	hypomethylated
cg22348299	0.87725	0.759978919	-0.207029	1.58E-05	hypomethylated
cg06368978	0.90103	0.78056973	-0.207048	9.38E-05	hypomethylated
cg22519265	0.83993	0.727611351	-0.207101	0.000857	hypomethylated
cg09389280	0.93228	0.807602162	-0.207119	2.57E-05	hypomethylated
cg09990481	0.78476	0.679692973	-0.207368	0.000127	hypomethylated
cg16885779	0.76647	0.663848108	-0.207376	0.002026	hypomethylated
cg14184954	0.86492	0.748731892	-0.208117	0.00039	hypomethylated
cg21816849	0.74636	0.645857838	-0.208655	0.000454	hypomethylated
cg11443888	0.82665	0.715097297	-0.209137	0.000109	hypomethylated
cg07575086	0.76955	0.665642703	-0.209267	0.002007	hypomethylated
cg27134084	0.87052	0.752802703	-0.209606	0.000253	hypomethylated
cg08129761	0.88928	0.768822162	-0.209988	9.07E-06	hypomethylated
cg16300681	0.87911	0.759768108	-0.210485	4.05E-05	hypomethylated
cg19201009	0.8766	0.757246486	-0.211156	0.000204	hypomethylated
cg12360123	0.86156	0.744148108	-0.211361	9.05E-05	hypomethylated
cg18681028	0.79596	0.687202162	-0.211961	0.000426	hypomethylated
cg10855276	0.82288	0.710423784	-0.212002	0.000733	hypomethylated
cg27074355	0.87792	0.757886486	-0.212108	1.99E-05	hypomethylated
cg24350484	0.81637	0.704674595	-0.212266	2.81E-05	hypomethylated
cg08964435	0.93519	0.806840541	-0.212976	1.35E-05	hypomethylated
cg16139249	0.85627	0.738291351	-0.213876	0.0006	hypomethylated
cg16419124	0.95325	0.821805946	-0.214057	8.15E-06	hypomethylated
cg22626525	0.80106	0.690401622	-0.214474	0.000365	hypomethylated
cg14560571	0.8221	0.708452432	-0.214643	1.46E-05	hypomethylated
cg21482176	0.88649	0.763516216	-0.215446	4.88E-05	hypomethylated
cg09980626	0.85171	0.733511892	-0.215542	6.05E-06	hypomethylated
cg04839409	0.84303	0.725885946	-0.215841	4.76E-05	hypomethylated
cg21343189	0.63083	0.543070811	-0.216111	0.000374	hypomethylated
cg19725098	0.87914	0.756790811	-0.216198	2.85E-05	hypomethylated
cg07663765	0.80016	0.688798919	-0.216206	0.000129	hypomethylated
cg22644320	0.85731	0.737365946	-0.217436	0.001409	hypomethylated
cg19462022	0.87018	0.748429189	-0.217448	0.000588	hypomethylated
cg23587532	0.79183	0.680768108	-0.218027	0.00035	hypomethylated
cg01690119	0.67573	0.580924865	-0.218095	0.003441	hypomethylated
cg16092390	0.73223	0.629406486	-0.218305	0.000822	hypomethylated
cg21218883	0.88653	0.762023243	-0.218334	3.07E-05	hypomethylated
cg05908960	0.82191	0.706419459	-0.218455	3.23E-05	hypomethylated
cg07544392	0.75996	0.653171892	-0.218461	0.000756	hypomethylated
cg07132492	0.82669	0.710469189	-0.218574	0.000112	hypomethylated
cg13179915	0.8201	0.704799459	-0.218587	6.62E-05	hypomethylated

cg10100437	0.84894	0.729455135	-0.218843	0.000822	hypomethylated
cg07316730	0.82164	0.705973514	-0.218892	0.000569	hypomethylated
cg03833060	0.8608	0.739612432	-0.218909	0.000229	hypomethylated
cg10071602	0.89672	0.770324865	-0.219191	1.01E-05	hypomethylated
cg27503117	0.84066	0.722055135	-0.219413	3.95E-05	hypomethylated
cg11803660	0.70725	0.607321622	-0.21976	0.002574	hypomethylated
cg07821739	0.85496	0.734122703	-0.219836	0.000145	hypomethylated
cg02668822	0.8395	0.720798919	-0.219933	4.53E-05	hypomethylated
cg05926784	0.71027	0.609798378	-0.220035	0.001141	hypomethylated
cg13926833	0.80536	0.691432973	-0.220044	0.001423	hypomethylated
cg09077530	0.79364	0.681337297	-0.220116	0.000449	hypomethylated
cg19910930	0.88362	0.758434054	-0.220402	0.000147	hypomethylated
cg25701710	0.86285	0.740519459	-0.220572	0.000287	hypomethylated
cg05813616	0.84503	0.725203243	-0.220617	0.000129	hypomethylated
cg20870752	0.83284	0.714112973	-0.221887	5.65E-05	hypomethylated
cg05390927	0.85419	0.732394595	-0.221936	4.58E-05	hypomethylated
cg11444527	0.87692	0.751537838	-0.222599	1.58E-05	hypomethylated
cg13330005	0.80205	0.687075676	-0.223223	0.000248	hypomethylated
cg05041265	0.81791	0.700564324	-0.223425	0.00049	hypomethylated
cg02872930	0.77109	0.66036	-0.223647	2.78E-05	hypomethylated
cg04263391	0.90797	0.777408649	-0.223971	1.01E-05	hypomethylated
cg14756040	0.75077	0.642768108	-0.224073	2.96E-05	hypomethylated
cg08017409	0.78323	0.670397838	-0.224419	0.001423	hypomethylated
cg19771626	0.71215	0.609507568	-0.224537	0.003021	hypomethylated
cg13808936	0.748	0.640187568	-0.224544	0.005508	hypomethylated
cg07021447	0.79739	0.682443243	-0.224576	0.000893	hypomethylated
cg05258834	0.84422	0.722159459	-0.225302	7.84E-05	hypomethylated
cg01766943	0.80651	0.689804865	-0.225504	0.000138	hypomethylated
cg25459300	0.81822	0.699702703	-0.225747	0.002699	hypomethylated
cg00169930	0.83793	0.716468649	-0.225926	9.05E-05	hypomethylated
cg20619563	0.84321	0.720917838	-0.226057	0.000399	hypomethylated
cg21646084	0.78378	0.669981081	-0.226328	0.000154	hypomethylated
cg24825974	0.90336	0.772125405	-0.226466	1.75E-05	hypomethylated
cg02176725	0.90686	0.775078919	-0.226537	1.21E-05	hypomethylated
cg11592082	0.73771	0.630489189	-0.226582	5.65E-05	hypomethylated
cg01628737	0.89763	0.767151351	-0.22661	1.99E-06	hypomethylated
cg14186944	0.77497	0.662282703	-0.226693	0.000306	hypomethylated
cg01142096	0.88227	0.753978919	-0.226696	0.000214	hypomethylated
cg20614157	0.91777	0.784231892	-0.226852	0.000112	hypomethylated
cg06402330	0.83147	0.710316216	-0.227203	0.000155	hypomethylated
cg20642630	0.81652	0.697473514	-0.22735	0.000435	hypomethylated
cg09827902	0.88111	0.752550811	-0.227533	4.15E-05	hypomethylated
cg14342528	0.93681	0.799908649	-0.227921	1.15E-06	hypomethylated
cg27625401	0.75544	0.645018378	-0.227977	0.000696	hypomethylated
cg13601740	0.86315	0.736885405	-0.228171	8.73E-05	hypomethylated
cg13730392	0.81599	0.696605405	-0.22821	3.67E-05	hypomethylated
cg12208770	0.70049	0.59796973	-0.228292	0.000607	hypomethylated
cg11888738	0.76495	0.652936216	-0.228423	0.001875	hypomethylated
cg10766866	0.78219	0.667563243	-0.228615	0.000182	hypomethylated
cg01583021	0.75779	0.646252973	-0.229699	0.000145	hypomethylated
cg27017414	0.83455	0.711611892	-0.229908	9.49E-05	hypomethylated
cg14212953	0.83461	0.711621622	-0.229992	6.46E-05	hypomethylated
cg16523185	0.78489	0.669198919	-0.230055	0.000395	hypomethylated
cg08136313	0.77223	0.658385946	-0.230097	0.001437	hypomethylated
cg20478261	0.84768	0.722691892	-0.230139	0.000259	hypomethylated
cg07791418	0.85061	0.725014054	-0.230489	7.29E-05	hypomethylated
cg08851181	0.84891	0.723557838	-0.230503	4.70E-05	hypomethylated
cg22807241	0.86938	0.740918378	-0.230672	2.39E-05	hypomethylated

cg13997759	0.84939	0.723867568	-0.230701	0.00039	hypomethylated
cg17767099	0.67387	0.574262162	-0.230761	0.001288	hypomethylated
cg24163568	0.75485	0.643214054	-0.230891	0.000718	hypomethylated
cg19873496	0.81128	0.691145946	-0.23121	0.001452	hypomethylated
cg27573017	0.80317	0.684227568	-0.231229	0.000102	hypomethylated
cg14606082	0.74581	0.635331351	-0.231299	0.000725	hypomethylated
cg27186519	0.76293	0.6499	-0.231333	0.000534	hypomethylated
cg12084631	0.73038	0.622092432	-0.231518	0.000313	hypomethylated
cg24994755	0.85274	0.726271892	-0.231596	6.15E-05	hypomethylated
cg13383734	0.76657	0.652841081	-0.231686	0.000399	hypomethylated
cg19391697	0.78798	0.671022162	-0.231799	0.00094	hypomethylated
cg02004044	0.86682	0.738078378	-0.231958	8.42E-05	hypomethylated
cg26598918	0.94652	0.805661622	-0.232459	3.67E-05	hypomethylated
cg26636061	0.72111	0.613634595	-0.23284	0.000256	hypomethylated
cg27469606	0.88483	0.752823784	-0.233088	4.58E-05	hypomethylated
cg25202141	0.89799	0.763887027	-0.23334	4.64E-05	hypomethylated
cg16022580	0.78766	0.669975676	-0.233464	0.000327	hypomethylated
cg14777519	0.85537	0.727542162	-0.233518	6.01E-05	hypomethylated
cg26226167	0.9117	0.775162162	-0.234061	5.50E-06	hypomethylated
cg04824378	0.83706	0.711676216	-0.23411	2.71E-05	hypomethylated
cg22430700	0.77131	0.655772973	-0.234114	0.001452	hypomethylated
cg16716189	0.82033	0.697352432	-0.234316	0.000145	hypomethylated
cg24830241	0.75122	0.638548108	-0.23444	0.00017	hypomethylated
cg19003803	0.88998	0.756116757	-0.235164	4.31E-05	hypomethylated
cg07516958	0.89086	0.756777297	-0.23533	8.60E-06	hypomethylated
cg23000950	0.87639	0.744483243	-0.235334	4.82E-05	hypomethylated
cg01235983	0.90504	0.768768649	-0.235432	6.57E-06	hypomethylated
cg06219204	0.78859	0.669793514	-0.235559	0.001052	hypomethylated
cg26959827	0.78922	0.670291351	-0.235639	0.000262	hypomethylated
cg12562461	0.83988	0.713266486	-0.235742	6.84E-06	hypomethylated
cg02052536	0.83825	0.711865946	-0.235775	0.000268	hypomethylated
cg13941555	0.67948	0.576956216	-0.235969	0.000126	hypomethylated
cg06779421	0.75194	0.638358919	-0.23625	4.47E-05	hypomethylated
cg26027526	0.8546	0.725338919	-0.236594	3.49E-05	hypomethylated
cg24239195	0.93321	0.792055135	-0.236601	9.69E-06	hypomethylated
cg27072212	0.90601	0.768924324	-0.236685	4.36E-06	hypomethylated
cg10594543	0.9255	0.785461622	-0.236692	4.12E-06	hypomethylated
cg01792592	0.77937	0.661428108	-0.236724	4.01E-06	hypomethylated
cg02568619	0.82411	0.699356757	-0.236808	0.000358	hypomethylated
cg03510310	0.87	0.738211892	-0.23698	4.70E-05	hypomethylated
cg01682917	0.79101	0.671098919	-0.237171	0.00028	hypomethylated
cg20219172	0.87615	0.743237297	-0.237355	0.000412	hypomethylated
cg10013998	0.70473	0.59766973	-0.237722	0.000633	hypomethylated
cg06241812	0.8528	0.723192432	-0.237828	3.62E-05	hypomethylated
cg21291321	0.81247	0.688987027	-0.237838	0.0006	hypomethylated
cg03723715	0.72044	0.610941622	-0.237844	0.000408	hypomethylated
cg10997335	0.89104	0.755599459	-0.237869	8.04E-06	hypomethylated
cg26845817	0.86807	0.736014595	-0.238077	1.75E-05	hypomethylated
cg24800616	0.90332	0.76582	-0.238232	5.65E-06	hypomethylated
cg26270038	0.75858	0.643081081	-0.238301	0.0006	hypomethylated
cg22701672	0.81159	0.687932973	-0.238483	0.000613	hypomethylated
cg22498453	0.91269	0.773575676	-0.238582	5.73E-06	hypomethylated
cg23695131	0.80343	0.680857297	-0.23882	0.000435	hypomethylated
cg07782795	0.84473	0.715718919	-0.239097	0.000189	hypomethylated
cg08764509	0.81771	0.692776757	-0.239199	4.76E-05	hypomethylated
cg23048915	0.84386	0.714921622	-0.239219	4.58E-05	hypomethylated
cg07523470	0.87257	0.739212432	-0.239282	4.36E-05	hypomethylated
cg14517863	0.79166	0.670617838	-0.23939	0.000342	hypomethylated

cg14928452	0.84493	0.715556757	-0.239766	0.000293	hypomethylated
cg17852876	0.80941	0.685409189	-0.239905	0.000135	hypomethylated
cg05454635	0.78205	0.662184324	-0.240028	0.000324	hypomethylated
cg04259971	0.76215	0.645175676	-0.240383	4.94E-05	hypomethylated
cg15451045	0.78062	0.660678919	-0.240671	1.06E-05	hypomethylated
cg19766178	0.88082	0.745372973	-0.240885	6.78E-05	hypomethylated
cg15748271	0.93136	0.78809027	-0.240978	6.05E-06	hypomethylated
cg14989661	0.87624	0.741439459	-0.240997	0.000204	hypomethylated
cg23273209	0.72544	0.613818378	-0.241044	0.000189	hypomethylated
cg04863197	0.86784	0.73415027	-0.241354	0.000814	hypomethylated
cg04978471	0.75328	0.637134054	-0.241589	0.000174	hypomethylated
cg06301529	0.90779	0.767778378	-0.241669	1.09E-05	hypomethylated
cg02213045	0.91261	0.771777838	-0.241813	2.10E-05	hypomethylated
cg09129839	0.86391	0.730528649	-0.24194	4.26E-05	hypomethylated
cg12448539	0.90049	0.761411351	-0.242034	3.67E-05	hypomethylated
cg13803455	0.86467	0.731077297	-0.242126	5.79E-05	hypomethylated
cg14993430	0.86459	0.730946486	-0.24225	0.0002	hypomethylated
cg02410245	0.79663	0.673487568	-0.242258	0.000251	hypomethylated
cg05928656	0.8526	0.720744324	-0.242381	0.000187	hypomethylated
cg20488341	0.84377	0.713267568	-0.242406	0.000104	hypomethylated
cg09293559	0.92339	0.780515676	-0.242513	1.25E-05	hypomethylated
cg26405835	0.89851	0.759416757	-0.242643	2.56E-06	hypomethylated
cg04862289	0.83413	0.704983784	-0.242682	0.000399	hypomethylated
cg12940991	0.79542	0.67226	-0.242698	0.000168	hypomethylated
cg05708073	0.87107	0.736161081	-0.242767	1.94E-05	hypomethylated
cg17489562	0.84544	0.71449027	-0.242788	2.92E-05	hypomethylated
cg15085006	0.89397	0.755486486	-0.24282	6.11E-07	hypomethylated
cg03407594	0.78763	0.665615135	-0.24283	0.000485	hypomethylated
cg17695682	0.86838	0.733762162	-0.243014	4.36E-06	hypomethylated
cg19505659	0.74807	0.632089189	-0.243045	0.000626	hypomethylated
cg18790382	0.88246	0.745554054	-0.243218	1.97E-05	hypomethylated
cg09507215	0.77877	0.657945946	-0.243228	7.84E-05	hypomethylated
cg06411506	0.79682	0.673184324	-0.243252	2.85E-05	hypomethylated
cg03312532	0.84004	0.709547568	-0.243559	8.83E-06	hypomethylated
cg04874343	0.73355	0.619516757	-0.243752	1.42E-05	hypomethylated
cg05220280	0.7383	0.623522703	-0.243765	0.000107	hypomethylated
cg08658981	0.82966	0.700620541	-0.243887	6.48E-06	hypomethylated
cg25987102	0.86854	0.733442703	-0.243908	3.69E-06	hypomethylated
cg11088489	0.77229	0.652015135	-0.244237	0.003538	hypomethylated
cg19025034	0.9037	0.76295027	-0.244255	6.93E-06	hypomethylated
cg04337188	0.84916	0.716768649	-0.244529	5.86E-05	hypomethylated
cg04691264	0.87576	0.739201622	-0.244568	0.000127	hypomethylated
cg00277384	0.8087	0.682594595	-0.244576	9.95E-05	hypomethylated
cg03687379	0.7756	0.654620541	-0.244654	3.81E-05	hypomethylated
cg09872392	0.78889	0.665782162	-0.244774	9.49E-05	hypomethylated
cg08128007	0.83459	0.704324324	-0.244828	0.000147	hypomethylated
cg24287175	0.70322	0.593445405	-0.244861	0.000772	hypomethylated
cg13587725	0.80966	0.683224865	-0.244956	0.000109	hypomethylated
cg06603851	0.89103	0.751647027	-0.245419	1.93E-06	hypomethylated
cg25219246	0.7251	0.611571351	-0.245659	5.58E-05	hypomethylated
cg13448828	0.88324	0.744918378	-0.245723	1.44E-05	hypomethylated
cg04962005	0.79211	0.668024324	-0.2458	0.000417	hypomethylated
cg09914060	0.85168	0.718253514	-0.245818	9.19E-06	hypomethylated
cg15003737	0.92288	0.778284324	-0.245846	5.89E-06	hypomethylated
cg02330507	0.85007	0.716773514	-0.246064	1.08E-05	hypomethylated
cg22800743	0.82076	0.691905405	-0.246386	0.000111	hypomethylated
cg16085744	0.91296	0.769570811	-0.246498	3.30E-06	hypomethylated
cg11741753	0.77122	0.649915135	-0.246891	0.000517	hypomethylated

cg16671682	0.74757	0.629952973	-0.246965	0.000226	hypomethylated
cg01291665	0.83437	0.703043784	-0.247073	9.05E-05	hypomethylated
cg20584157	0.81419	0.686031892	-0.24709	0.001556	hypomethylated
cg08860443	0.87031	0.73318	-0.247362	9.95E-05	hypomethylated
cg09273059	0.76799	0.646852432	-0.247651	0.003136	hypomethylated
cg14418633	0.77898	0.656069189	-0.247738	0.003808	hypomethylated
cg19770715	0.89386	0.752811351	-0.247761	1.56E-06	hypomethylated
cg21005457	0.76195	0.641705946	-0.247784	0.000182	hypomethylated
cg14278300	0.75266	0.633873514	-0.247803	0.000857	hypomethylated
cg01651169	0.79527	0.669726486	-0.247873	7.12E-05	hypomethylated
cg06112923	0.9075	0.764148108	-0.248045	1.17E-05	hypomethylated
cg06284816	0.8772	0.738618378	-0.248077	8.63E-05	hypomethylated
cg22209441	0.72097	0.606945946	-0.248371	0.000296	hypomethylated
cg00013804	0.88486	0.744889189	-0.248423	7.29E-05	hypomethylated
cg13982220	0.60924	0.51283027	-0.248529	0.000178	hypomethylated
cg14582917	0.87036	0.732536216	-0.248712	0.000234	hypomethylated
cg01816186	0.85535	0.719849189	-0.24882	0.000109	hypomethylated
cg03445965	0.88881	0.747993514	-0.248849	1.25E-05	hypomethylated
cg03374561	0.63973	0.538349189	-0.248921	0.001667	hypomethylated
cg00850193	0.72096	0.606652432	-0.249049	0.000189	hypomethylated
cg20050877	0.78312	0.658927027	-0.249115	0.001667	hypomethylated
cg02144516	0.76663	0.644976757	-0.249283	8.03E-05	hypomethylated
cg01128109	0.77987	0.656065946	-0.249393	0.001031	hypomethylated
cg05272594	0.67762	0.570028108	-0.249443	0.000399	hypomethylated
cg21743867	0.8746	0.735655676	-0.249593	5.28E-06	hypomethylated
cg09227277	0.91916	0.773034595	-0.249783	2.68E-06	hypomethylated
cg13098960	0.63748	0.536111892	-0.249846	0.0003	hypomethylated
cg01985343	0.87919	0.739331351	-0.249954	2.32E-05	hypomethylated
cg02395127	0.81257	0.683213514	-0.250156	0.000256	hypomethylated
cg26309311	0.77792	0.653855135	-0.250651	0.000262	hypomethylated
cg19604641	0.92536	0.777720541	-0.250763	5.58E-05	hypomethylated
cg14277677	0.74526	0.626342703	-0.250792	0.0003	hypomethylated
cg14614490	0.70953	0.596217838	-0.251024	0.000209	hypomethylated
cg07620544	0.75734	0.636390811	-0.251028	0.000163	hypomethylated
cg20022869	0.68561	0.576081622	-0.251115	0.000495	hypomethylated
cg12683120	0.84871	0.713108649	-0.25115	0.000104	hypomethylated
cg11095658	0.84724	0.711739459	-0.251421	1.01E-05	hypomethylated
cg00792184	0.81423	0.683963243	-0.251518	7.84E-05	hypomethylated
cg11382394	0.90425	0.759505946	-0.25166	7.12E-06	hypomethylated
cg20735682	0.81235	0.68230973	-0.251675	0.000805	hypomethylated
cg15807143	0.82111	0.689622162	-0.251769	0.000613	hypomethylated
cg08866633	0.7457	0.626255676	-0.251844	5.45E-05	hypomethylated
cg19914238	0.89007	0.747496216	-0.251853	7.84E-05	hypomethylated
cg08222053	0.86203	0.723899459	-0.251949	9.60E-05	hypomethylated
cg12533206	0.74394	0.624719459	-0.251978	0.000495	hypomethylated
cg03724728	0.89022	0.747550811	-0.25199	1.50E-05	hypomethylated
cg10551058	0.73852	0.62016	-0.251997	2.89E-05	hypomethylated
cg02975953	0.77214	0.6483	-0.252201	0.000178	hypomethylated
cg11585942	0.8419	0.706854054	-0.252237	0.000209	hypomethylated
cg25884442	0.59861	0.502567568	-0.252299	0.000511	hypomethylated
cg02896757	0.8583	0.720561622	-0.25236	0.000268	hypomethylated
cg02663496	0.82341	0.691248108	-0.252407	2.39E-05	hypomethylated
cg23185751	0.87071	0.73089027	-0.252537	2.67E-05	hypomethylated
cg03023935	0.94284	0.791435135	-0.252542	4.12E-06	hypomethylated
cg03977174	0.86015	0.722011351	-0.252567	4.26E-05	hypomethylated
cg05746370	0.88221	0.740442703	-0.252734	9.31E-06	hypomethylated
cg20775109	0.81574	0.68462973	-0.252785	2.48E-05	hypomethylated
cg08017202	0.85509	0.717601081	-0.252894	1.46E-05	hypomethylated

cg10173075	0.88928	0.746272973	-0.252934	0.000126	hypomethylated
cg08578212	0.65463	0.549303784	-0.253075	0.000155	hypomethylated
cg16569813	0.79674	0.668512973	-0.253153	0.00032	hypomethylated
cg20894936	0.73673	0.618158378	-0.253159	0.001481	hypomethylated
cg15367000	0.82799	0.694725946	-0.253169	0.000155	hypomethylated
cg08549241	0.82109	0.688922162	-0.253199	0.001821	hypomethylated
cg22808839	0.84174	0.706200541	-0.253297	0.000342	hypomethylated
cg26777475	0.79154	0.664079459	-0.253306	0.00071	hypomethylated
cg23514211	0.63631	0.533781622	-0.25348	0.000271	hypomethylated
cg22941178	0.79584	0.667603243	-0.253487	3.07E-05	hypomethylated
cg18669789	0.77444	0.649552432	-0.253707	2.61E-05	hypomethylated
cg12015615	0.8347	0.700037838	-0.253825	3.57E-05	hypomethylated
cg23106115	0.62402	0.523274054	-0.254026	0.00024	hypomethylated
cg14228788	0.84985	0.712643243	-0.254028	2.07E-05	hypomethylated
cg09554596	0.81906	0.686788649	-0.254103	0.000148	hypomethylated
cg08737815	0.91275	0.765119459	-0.254535	2.45E-05	hypomethylated
cg14479301	0.83781	0.702258378	-0.254621	0.000109	hypomethylated
cg24859986	0.90617	0.759547027	-0.254642	2.32E-05	hypomethylated
cg06528773	0.69587	0.583264865	-0.254667	3.69E-06	hypomethylated
cg16243195	0.65632	0.550112432	-0.254673	0.000681	hypomethylated
cg25890936	0.80942	0.678412432	-0.254726	7.47E-05	hypomethylated
cg22760037	0.86483	0.724837297	-0.254759	1.37E-05	hypomethylated
cg03807919	0.77576	0.65012973	-0.254883	3.07E-05	hypomethylated
cg07694735	0.84373	0.707074054	-0.25492	0.000109	hypomethylated
cg03466598	0.87878	0.73638973	-0.255033	8.84E-05	hypomethylated
cg22524332	0.85792	0.718857297	-0.255138	9.72E-05	hypomethylated
cg03995533	0.76771	0.643244865	-0.255193	0.001541	hypomethylated
cg08776942	0.88268	0.739563784	-0.255216	1.42E-05	hypomethylated
cg02964434	0.76555	0.641391351	-0.255292	0.00039	hypomethylated
cg00437969	0.90296	0.756508649	-0.255306	6.75E-06	hypomethylated
cg04811534	0.8961	0.750629189	-0.255559	1.21E-05	hypomethylated
cg07536328	0.87757	0.735083243	-0.255607	4.15E-05	hypomethylated
cg11789371	0.85034	0.71218	-0.255798	0.000157	hypomethylated
cg07205883	0.84589	0.708446486	-0.255811	0.000117	hypomethylated
cg15132165	0.79687	0.667345405	-0.255911	0.00071	hypomethylated
cg24584161	0.87808	0.735265946	-0.256086	1.46E-05	hypomethylated
cg01870519	0.77921	0.652434054	-0.25618	0.00017	hypomethylated
cg07047963	0.66794	0.559234054	-0.256266	0.000204	hypomethylated
cg18917486	0.76613	0.641435135	-0.256286	0.001262	hypomethylated
cg18575863	0.80893	0.677190811	-0.256452	0.001803	hypomethylated
cg00972420	0.79164	0.66271027	-0.256466	0.000426	hypomethylated
cg22160735	0.78103	0.653824865	-0.256474	0.000159	hypomethylated
cg23042540	0.85002	0.711543243	-0.256545	1.89E-05	hypomethylated
cg15643000	0.77916	0.652217838	-0.256566	0.000109	hypomethylated
cg09951201	0.83233	0.696674595	-0.256671	0.000135	hypomethylated
cg19282443	0.85323	0.714156757	-0.256694	0.00013	hypomethylated
cg18767057	0.73422	0.614331892	-0.257194	0.000204	hypomethylated
cg04143805	0.84339	0.705661622	-0.257223	6.78E-05	hypomethylated
cg09230763	0.71721	0.600077297	-0.257247	0.000399	hypomethylated
cg22511889	0.74862	0.626356757	-0.257249	0.000112	hypomethylated
cg12618270	0.82783	0.692615676	-0.257279	0.000417	hypomethylated
cg25191304	0.92016	0.769799459	-0.257402	5.58E-06	hypomethylated
cg06807837	0.87898	0.735335135	-0.257428	2.45E-05	hypomethylated
cg08508777	0.7458	0.623905946	-0.25746	2.21E-05	hypomethylated
cg18509719	0.93324	0.780648108	-0.257576	2.74E-05	hypomethylated
cg05265143	0.83687	0.700017838	-0.257612	0.000115	hypomethylated
cg11502921	0.70701	0.591315135	-0.257803	0.000148	hypomethylated
cg25473115	0.87844	0.734672432	-0.257843	4.47E-05	hypomethylated

cg07596106	0.76673	0.641160541	-0.258033	0.000193	hypomethylated
cg02370644	0.85235	0.712754595	-0.258041	8.42E-05	hypomethylated
cg19204958	0.81945	0.68523027	-0.258067	0.001052	hypomethylated
cg12216688	0.75982	0.635341081	-0.258126	0.000111	hypomethylated
cg01410415	0.70607	0.590390811	-0.258141	3.95E-05	hypomethylated
cg20161227	0.88658	0.741318919	-0.258156	6.62E-05	hypomethylated
cg04390004	0.77532	0.648205946	-0.25834	0.000178	hypomethylated
cg19927741	0.72069	0.60238973	-0.258682	0.000607	hypomethylated
cg18885372	0.82207	0.687098378	-0.258745	0.000313	hypomethylated
cg07457213	0.71491	0.597531892	-0.258746	0.000245	hypomethylated
cg23313266	0.78007	0.651915676	-0.258918	0.000113	hypomethylated
cg10967176	0.89395	0.746987027	-0.259111	6.31E-05	hypomethylated
cg04142080	0.78539	0.656150811	-0.259382	1.30E-05	hypomethylated
cg07757885	0.72927	0.609243243	-0.259435	0.0002	hypomethylated
cg09586198	0.79969	0.668001081	-0.25959	0.000306	hypomethylated
cg18642576	0.89269	0.745584324	-0.259788	4.15E-05	hypomethylated
cg26869512	0.93942	0.784598378	-0.259816	1.39E-06	hypomethylated
cg12791245	0.81908	0.684076757	-0.259846	1.89E-05	hypomethylated
cg25980489	0.88162	0.736297838	-0.259867	1.18E-05	hypomethylated
cg20808693	0.72574	0.606074595	-0.259957	0.000207	hypomethylated
cg02135122	0.88003	0.73492	-0.259965	5.65E-05	hypomethylated
cg01297744	0.86471	0.722111351	-0.259995	0.00013	hypomethylated
cg10095654	0.69014	0.576256216	-0.260179	0.000219	hypomethylated
cg20012366	0.62255	0.519796757	-0.260242	0.001768	hypomethylated
cg06924873	0.81132	0.677407027	-0.260248	0.000245	hypomethylated
cg03033344	0.83478	0.696984865	-0.260269	1.50E-05	hypomethylated
cg20933326	0.81166	0.677667568	-0.260298	9.82E-06	hypomethylated
cg07708016	0.83267	0.695200541	-0.260316	4.82E-05	hypomethylated
cg01396138	0.84037	0.701609189	-0.260357	1.56E-05	hypomethylated
cg14011229	0.85385	0.712858919	-0.260366	0.000216	hypomethylated
cg08486432	0.8561	0.714582703	-0.260678	4.48E-06	hypomethylated
cg13298167	0.81225	0.677922703	-0.260803	0.000234	hypomethylated
cg09847652	0.85793	0.71602	-0.26086	3.04E-05	hypomethylated
cg22114568	0.85936	0.717212973	-0.260861	4.82E-05	hypomethylated
cg01931222	0.80299	0.670083243	-0.261042	0.000511	hypomethylated
cg18960218	0.76433	0.637803243	-0.261084	0.000365	hypomethylated
cg21911100	0.77894	0.649971351	-0.261136	3.95E-05	hypomethylated
cg11569478	0.88827	0.741164324	-0.261205	4.36E-05	hypomethylated
cg22331032	0.73811	0.615843784	-0.261271	0.000335	hypomethylated
cg13333107	0.8895	0.742142703	-0.261298	3.67E-05	hypomethylated
cg19290938	0.8275	0.690357838	-0.261415	7.75E-05	hypomethylated
cg09944616	0.75449	0.629446486	-0.261418	5.00E-05	hypomethylated
cg21836117	0.79001	0.659075676	-0.261427	0.000138	hypomethylated
cg22218316	0.81726	0.681802703	-0.261441	8.63E-05	hypomethylated
cg07546508	0.74186	0.618865946	-0.26152	2.42E-05	hypomethylated
cg11664544	0.77008	0.642398919	-0.261539	0.00044	hypomethylated
cg21545013	0.78897	0.658154054	-0.261545	0.000155	hypomethylated
cg09508189	0.79661	0.664391892	-0.261839	0.000112	hypomethylated
cg13000789	0.84194	0.702127027	-0.261985	0.000148	hypomethylated
cg06738379	0.79412	0.662169189	-0.262157	0.000219	hypomethylated
cg24109934	0.74987	0.625247568	-0.262213	1.84E-05	hypomethylated
cg14384283	0.77991	0.650288649	-0.262227	0.000265	hypomethylated
cg12871463	0.77168	0.643354595	-0.262389	0.000219	hypomethylated
cg21900928	0.8121	0.677000541	-0.2625	0.000168	hypomethylated
cg08629176	0.76248	0.635626486	-0.26252	0.0003	hypomethylated
cg01451604	0.801	0.667732432	-0.262532	0.000653	hypomethylated
cg00065226	0.92805	0.773593514	-0.262627	1.46E-05	hypomethylated
cg10997190	0.88916	0.741160541	-0.262657	3.95E-05	hypomethylated

cg02400595	0.89061	0.742292432	-0.262806	1.75E-05	hypomethylated
cg06720949	0.77702	0.647594054	-0.262862	0.000262	hypomethylated
cg21208107	0.89764	0.748115135	-0.262877	2.36E-06	hypomethylated
cg15763121	0.79761	0.664745946	-0.26288	3.85E-05	hypomethylated
cg26899496	0.88158	0.734695676	-0.262945	1.20E-05	hypomethylated
cg23402920	0.86748	0.722867568	-0.263099	2.51E-05	hypomethylated
cg14036828	0.90527	0.754343243	-0.263127	9.38E-05	hypomethylated
cg19072957	0.87031	0.725202703	-0.263145	0.000112	hypomethylated
cg22272492	0.82421	0.686776216	-0.263172	3.67E-05	hypomethylated
cg04225554	0.75726	0.630958378	-0.263244	7.12E-06	hypomethylated
cg21218627	0.85973	0.716257838	-0.263405	1.94E-05	hypomethylated
cg23882164	0.78176	0.651272973	-0.263463	0.000132	hypomethylated
cg27359629	0.86758	0.722747568	-0.263505	2.04E-05	hypomethylated
cg16678564	0.85409	0.711488649	-0.263547	0.000495	hypomethylated
cg00890145	0.67899	0.56558973	-0.263634	0.00044	hypomethylated
cg07244783	0.9082	0.756471351	-0.263725	2.53E-06	hypomethylated
cg22285671	0.70598	0.58802973	-0.263738	0.000741	hypomethylated
cg10627703	0.93249	0.776671351	-0.263784	2.74E-05	hypomethylated
cg07219425	0.81461	0.678411892	-0.263948	0.000232	hypomethylated
cg04508312	0.69482	0.578629189	-0.264	0.000174	hypomethylated
cg23795082	0.8601	0.716164865	-0.264213	0.000126	hypomethylated
cg05556202	0.79422	0.661236216	-0.264373	0.000178	hypomethylated
cg07444554	0.85967	0.715721081	-0.264385	8.60E-06	hypomethylated
cg12019893	0.84033	0.699574054	-0.264479	0.000789	hypomethylated
cg11547696	0.76332	0.635443243	-0.264525	0.000884	hypomethylated
cg27114782	0.77667	0.64654	-0.264562	0.002274	hypomethylated
cg15320059	0.81159	0.675596216	-0.26459	1.89E-05	hypomethylated
cg19364141	0.91109	0.758413514	-0.264609	8.15E-06	hypomethylated
cg21767759	0.55719	0.463796757	-0.264677	0.000581	hypomethylated
cg06106510	0.87525	0.728475135	-0.264815	3.76E-05	hypomethylated
cg06171971	0.64795	0.539272432	-0.264868	0.000234	hypomethylated
cg23731501	0.80726	0.671851351	-0.264891	0.000148	hypomethylated
cg00119314	0.68841	0.572876757	-0.265043	0.000161	hypomethylated
cg25316305	0.89934	0.748402703	-0.265052	2.42E-05	hypomethylated
cg14288399	0.79698	0.663172432	-0.265159	0.000166	hypomethylated
cg04607671	0.78783	0.655531892	-0.265218	0.000214	hypomethylated
cg10888811	0.74507	0.619892432	-0.265358	0.000202	hypomethylated
cg21646032	0.82888	0.689545946	-0.265517	0.000653	hypomethylated
cg14215515	0.79473	0.661023784	-0.265763	4.64E-05	hypomethylated
cg08423142	0.77751	0.646682162	-0.265804	0.001118	hypomethylated
cg07034563	0.87658	0.728984324	-0.265998	2.81E-05	hypomethylated
cg27093376	0.81368	0.676663243	-0.266024	5.00E-05	hypomethylated
cg02202742	0.78439	0.652296757	-0.266043	0.000338	hypomethylated
cg14409104	0.93048	0.773691351	-0.266217	2.10E-05	hypomethylated
cg20666186	0.81312	0.676051351	-0.266335	0.000135	hypomethylated
cg13481776	0.80158	0.666414595	-0.266427	0.000653	hypomethylated
cg06349754	0.88924	0.739274054	-0.266464	1.39E-06	hypomethylated
cg27460531	0.74446	0.618892973	-0.266504	0.000435	hypomethylated
cg10260711	0.82625	0.686878378	-0.266524	0.000187	hypomethylated
cg00328210	0.87561	0.727797297	-0.266752	8.84E-05	hypomethylated
cg10343327	0.89216	0.741531351	-0.266795	3.44E-05	hypomethylated
cg15988204	0.70355	0.584727027	-0.26689	0.000445	hypomethylated
cg15705203	0.7474	0.621152432	-0.266933	0.000123	hypomethylated
cg09354263	0.73814	0.613381622	-0.26711	0.000185	hypomethylated
cg05418495	0.81067	0.673600541	-0.267221	9.49E-05	hypomethylated
cg13223043	0.76925	0.639078919	-0.267458	0.000369	hypomethylated
cg16827257	0.88427	0.734583784	-0.26756	8.22E-05	hypomethylated
cg03251852	0.85864	0.71328	-0.267585	4.36E-05	hypomethylated

cg07324245	0.66722	0.554239459	-0.267653	0.000256	hypomethylated
cg26904179	0.82187	0.682663243	-0.267736	5.58E-06	hypomethylated
cg00130778	0.73543	0.610844865	-0.267782	0.000696	hypomethylated
cg13127574	0.87562	0.727253514	-0.267847	5.58E-06	hypomethylated
cg07290522	0.8122	0.674573514	-0.267859	0.000382	hypomethylated
cg14289738	0.85196	0.70757027	-0.267912	9.96E-06	hypomethylated
cg22785170	0.88368	0.733902162	-0.267936	1.18E-05	hypomethylated
cg02518338	0.90026	0.747646486	-0.267985	8.32E-05	hypomethylated
cg09162518	0.89081	0.739688108	-0.268201	7.52E-06	hypomethylated
cg21372107	0.7936	0.658903243	-0.268345	8.42E-05	hypomethylated
cg14436231	0.7812	0.648604324	-0.268353	0.000756	hypomethylated
cg25268100	0.73438	0.609718919	-0.268382	0.000674	hypomethylated
cg05739379	0.83877	0.696337838	-0.268488	0.000209	hypomethylated
cg03357952	0.74933	0.622074595	-0.268514	0.00097	hypomethylated
cg19823504	0.8513	0.706726486	-0.268516	1.66E-05	hypomethylated
cg03879971	0.86536	0.718372432	-0.268568	8.42E-05	hypomethylated
cg03302135	0.68559	0.569137297	-0.268569	0.000221	hypomethylated
cg08837481	0.88794	0.737060541	-0.268679	4.47E-05	hypomethylated
cg24730307	0.86942	0.721635676	-0.268783	2.92E-05	hypomethylated
cg09916599	0.79856	0.662772432	-0.268887	0.000102	hypomethylated
cg14590214	0.92471	0.767444324	-0.268939	1.01E-05	hypomethylated
cg18461436	0.86633	0.718887568	-0.269151	1.58E-05	hypomethylated
cg11705208	0.76084	0.631325946	-0.269208	0.000135	hypomethylated
cg10114235	0.83873	0.695948649	-0.269226	5.45E-05	hypomethylated
cg13250566	0.79274	0.65778	-0.269243	0.000219	hypomethylated
cg27485075	0.75569	0.626941081	-0.269465	0.001768	hypomethylated
cg09126794	0.87411	0.725128108	-0.269579	4.36E-05	hypomethylated
cg18269382	0.82684	0.685874595	-0.269663	0.000142	hypomethylated
cg18099423	0.8565	0.710471892	-0.269676	9.49E-05	hypomethylated
cg04519626	0.83179	0.689959459	-0.269708	5.93E-05	hypomethylated
cg09344098	0.66637	0.552724324	-0.269763	1.75E-05	hypomethylated
cg03025880	0.79013	0.655365946	-0.269789	0.000365	hypomethylated
cg03774957	0.85365	0.707981622	-0.269933	0.000681	hypomethylated
cg09218398	0.96437	0.799792973	-0.26996	3.69E-06	hypomethylated
cg22906553	0.87101	0.722350811	-0.26999	3.85E-05	hypomethylated
cg09873544	0.71679	0.59442973	-0.270044	0.000358	hypomethylated
cg07372450	0.89116	0.739004865	-0.270101	5.19E-05	hypomethylated
cg15095427	0.86436	0.716772432	-0.270117	6.22E-06	hypomethylated
cg15407213	0.82513	0.68422	-0.270161	4.36E-05	hypomethylated
cg07053162	0.85024	0.704951892	-0.270345	0.000117	hypomethylated
cg26193418	0.84409	0.699833514	-0.270385	4.82E-05	hypomethylated
cg10461261	0.83237	0.690088108	-0.270444	0.000132	hypomethylated
cg03862705	0.66389	0.550398919	-0.270467	0.005757	hypomethylated
cg22451100	0.88782	0.736031351	-0.2705	3.12E-06	hypomethylated
cg13466409	0.88873	0.736764865	-0.270541	3.15E-05	hypomethylated
cg15010372	0.8075	0.669404324	-0.270584	1.77E-05	hypomethylated
cg11690983	0.90154	0.747358378	-0.270591	1.99E-05	hypomethylated
cg12701088	0.51706	0.428628649	-0.270603	0.000581	hypomethylated
cg08290756	0.90379	0.749207027	-0.270623	4.36E-06	hypomethylated
cg01955025	0.85536	0.709057838	-0.270628	6.86E-05	hypomethylated
cg26303613	0.79681	0.660497838	-0.270682	0.000132	hypomethylated
cg26955287	0.89871	0.744907027	-0.270795	2.53E-06	hypomethylated
cg03403081	0.87535	0.725482162	-0.27092	9.07E-06	hypomethylated
cg10343364	0.78481	0.650437838	-0.270932	0.001368	hypomethylated
cg03750525	0.73186	0.60652973	-0.270989	0.00024	hypomethylated
cg02850715	0.75779	0.627943243	-0.271164	0.000123	hypomethylated
cg07681739	0.82999	0.687715676	-0.271282	5.31E-05	hypomethylated
cg00245890	0.79608	0.659616757	-0.271285	0.000253	hypomethylated

cg14157052	0.93652	0.775956216	-0.271335	1.82E-06	hypomethylated
cg23894948	0.66158	0.548148108	-0.27135	0.000594	hypomethylated
cg12164955	0.89828	0.744216216	-0.271443	4.70E-05	hypomethylated
cg24327877	0.87389	0.723996216	-0.27147	1.46E-05	hypomethylated
cg08963118	0.82499	0.683463784	-0.271512	4.05E-05	hypomethylated
cg21910673	0.71382	0.591321081	-0.271619	0.001803	hypomethylated
cg24685109	0.83056	0.688019459	-0.271635	4.54E-06	hypomethylated
cg20890440	0.83537	0.691982162	-0.27168	0.000277	hypomethylated
cg25567805	0.83726	0.693532432	-0.271712	0.000653	hypomethylated
cg08785524	0.88863	0.736039459	-0.2718	6.05E-06	hypomethylated
cg26300500	0.87369	0.723664324	-0.271801	4.48E-06	hypomethylated
cg07516712	0.91596	0.758476757	-0.27218	1.54E-05	hypomethylated
cg27050818	0.85295	0.706216757	-0.27235	7.32E-06	hypomethylated
cg20686001	0.75017	0.621091351	-0.272412	0.000101	hypomethylated
cg19716073	0.78678	0.651351351	-0.272524	0.0003	hypomethylated
cg25162533	0.74031	0.612788649	-0.27274	0.000159	hypomethylated
cg06263178	0.82538	0.683116757	-0.272926	0.0003	hypomethylated
cg18109186	0.87342	0.722847568	-0.272984	4.86E-06	hypomethylated
cg04870732	0.86983	0.719864324	-0.273008	3.49E-05	hypomethylated
cg17140191	0.75355	0.623621081	-0.273034	5.32E-05	hypomethylated
cg01171456	0.84479	0.699124324	-0.273044	2.38E-05	hypomethylated
cg27526892	0.88582	0.733044865	-0.273112	1.73E-05	hypomethylated
cg09368716	0.78836	0.652333514	-0.273245	0.000358	hypomethylated
cg16450654	0.79244	0.655660541	-0.273353	6.78E-05	hypomethylated
cg13580107	0.73124	0.604839459	-0.273793	6.01E-05	hypomethylated
cg17968946	0.90311	0.746948649	-0.273893	4.73E-06	hypomethylated
cg14647877	0.87049	0.719966486	-0.273898	0.000103	hypomethylated
cg27180974	0.8538	0.706147568	-0.273928	9.72E-05	hypomethylated
cg00283986	0.70622	0.584059459	-0.274002	0.000485	hypomethylated
cg10640398	0.79814	0.660062162	-0.27404	0.000399	hypomethylated
cg19022978	0.88104	0.728543784	-0.274192	5.06E-05	hypomethylated
cg20303033	0.62005	0.512724324	-0.274201	0.001176	hypomethylated
cg26275110	0.64563	0.533837297	-0.274308	0.000426	hypomethylated
cg22392793	0.76336	0.631112973	-0.274465	0.00049	hypomethylated
cg18815565	0.90812	0.750778378	-0.274496	5.43E-06	hypomethylated
cg01548456	0.87913	0.726807027	-0.274504	1.04E-05	hypomethylated
cg22276227	0.79078	0.653696757	-0.274655	3.57E-05	hypomethylated
cg12042787	0.62046	0.512883243	-0.274708	6.86E-05	hypomethylated
cg12734688	0.86204	0.712574595	-0.274714	4.80E-06	hypomethylated
cg10960354	0.84888	0.701636757	-0.274836	1.77E-05	hypomethylated
cg06071334	0.73911	0.610878919	-0.274903	3.90E-05	hypomethylated
cg05885462	0.88934	0.735007568	-0.274976	3.57E-05	hypomethylated
cg24619694	0.81199	0.671075676	-0.274986	1.87E-05	hypomethylated
cg08626653	0.78634	0.649856216	-0.275033	7.38E-05	hypomethylated
cg25380464	0.92957	0.768127027	-0.275219	4.30E-06	hypomethylated
cg14294658	0.68552	0.566447568	-0.275256	0.001031	hypomethylated
cg27478579	0.74526	0.615801081	-0.275279	0.000224	hypomethylated
cg08317252	0.5555	0.458999459	-0.275294	0.000124	hypomethylated
cg08155908	0.84088	0.6948	-0.275302	0.000251	hypomethylated
cg08940490	0.65689	0.542731892	-0.275412	0.006679	hypomethylated
cg14662210	0.86734	0.716429189	-0.275774	5.45E-05	hypomethylated
cg25520440	0.70216	0.579980541	-0.275795	0.00043	hypomethylated
cg01172082	0.76765	0.634064865	-0.275818	0.000138	hypomethylated
cg26516710	0.87805	0.725234595	-0.275855	0.0003	hypomethylated
cg08205236	0.66017	0.545202703	-0.276045	0.00039	hypomethylated
cg06782350	0.75805	0.626028108	-0.276066	0.000127	hypomethylated
cg20781516	0.81144	0.67011027	-0.276086	5.86E-05	hypomethylated
cg05131646	0.62683	0.517585946	-0.276276	0.001409	hypomethylated

cg00543840	0.89868	0.742042703	-0.276305	9.07E-06	hypomethylated
cg10572659	0.82328	0.679734054	-0.276413	5.65E-06	hypomethylated
cg12710538	0.65061	0.537163243	-0.276432	0.000237	hypomethylated
cg26687381	0.90834	0.749881081	-0.276571	1.62E-05	hypomethylated
cg14492992	0.78058	0.644352432	-0.276697	0.003441	hypomethylated
cg03528302	0.7938	0.655248108	-0.276734	0.00017	hypomethylated
cg07312312	0.89849	0.741644865	-0.276774	1.23E-05	hypomethylated
cg26710371	0.78842	0.650777838	-0.276799	0.00017	hypomethylated
cg10853533	0.78821	0.650574054	-0.276867	4.86E-06	hypomethylated
cg06872381	0.72213	0.596023784	-0.276889	0.00028	hypomethylated
cg24771152	0.97262	0.802739459	-0.276944	9.69E-06	hypomethylated
cg20215290	0.92403	0.762559459	-0.27709	1.24E-06	hypomethylated
cg15105060	0.81605	0.673428649	-0.277132	7.62E-06	hypomethylated
cg07600170	0.94779	0.782078378	-0.277254	6.13E-06	hypomethylated
cg18597991	0.86723	0.715578919	-0.277304	0.000132	hypomethylated
cg01856892	0.78094	0.644341081	-0.277387	0.001074	hypomethylated
cg01800297	0.79049	0.652102703	-0.277648	0.000157	hypomethylated
cg07005654	0.67426	0.556175135	-0.277766	0.002147	hypomethylated
cg15070897	0.7703	0.635355135	-0.277857	0.003441	hypomethylated
cg17821664	0.74013	0.610464324	-0.277872	0.000147	hypomethylated
cg06314202	0.6558	0.540898378	-0.277898	4.36E-05	hypomethylated
cg19381819	0.7261	0.598826486	-0.27803	2.04E-05	hypomethylated
cg19997245	0.87278	0.719748108	-0.278126	6.08E-05	hypomethylated
cg24616382	0.85869	0.708088108	-0.278209	4.94E-05	hypomethylated
cg23793599	0.90303	0.744604865	-0.278299	1.33E-05	hypomethylated
cg22224043	0.76553	0.631226486	-0.278301	0.000293	hypomethylated
cg24493649	0.90353	0.745002162	-0.278328	6.75E-06	hypomethylated
cg03549739	0.74671	0.615669189	-0.278393	0.000718	hypomethylated
cg14025883	0.83911	0.691796216	-0.278513	7.72E-06	hypomethylated
cg11093373	0.81072	0.668362162	-0.278574	4.64E-05	hypomethylated
cg14669515	0.73005	0.601735135	-0.278867	0.001368	hypomethylated
cg21853989	0.89196	0.735122703	-0.278994	4.05E-05	hypomethylated
cg22880933	0.70631	0.582055676	-0.279144	9.07E-06	hypomethylated
cg22235901	0.74083	0.610454595	-0.279259	2.78E-05	hypomethylated
cg24959938	0.84444	0.695807568	-0.279307	3.49E-05	hypomethylated
cg18834029	0.93356	0.769196757	-0.27939	1.54E-05	hypomethylated
cg14170793	0.84845	0.699016216	-0.279504	5.00E-05	hypomethylated
cg12443001	0.67643	0.557290811	-0.27951	0.000134	hypomethylated
cg06524377	0.71716	0.590843243	-0.27952	0.000262	hypomethylated
cg24836242	0.69404	0.571790811	-0.279531	0.000132	hypomethylated
cg06047778	0.85664	0.705730811	-0.279571	6.15E-05	hypomethylated
cg17536465	0.78486	0.646589189	-0.279586	0.000454	hypomethylated
cg23095729	0.7959	0.655572973	-0.279831	0.000152	hypomethylated
cg11464885	0.8015	0.660181622	-0.27984	0.000185	hypomethylated
cg26612409	0.84186	0.693374595	-0.279945	7.12E-05	hypomethylated
cg20435125	0.79235	0.652527568	-0.280099	1.42E-05	hypomethylated
cg19651003	0.87971	0.724468649	-0.280105	0.0002	hypomethylated
cg15208832	0.79491	0.654629189	-0.280114	0.000268	hypomethylated
cg00401456	0.91417	0.752818378	-0.280161	2.21E-05	hypomethylated
cg21581096	0.73614	0.606181622	-0.28023	9.38E-05	hypomethylated
cg15240733	0.60808	0.500722703	-0.280249	0.000182	hypomethylated
cg27214687	0.9429	0.776421081	-0.280265	3.95E-05	hypomethylated
cg27409401	0.92045	0.757917297	-0.280299	1.46E-05	hypomethylated
cg12931707	0.88958	0.732461081	-0.280372	1.75E-05	hypomethylated
cg17923377	0.62602	0.515401081	-0.280513	0.004365	hypomethylated
cg15578332	0.84875	0.698741622	-0.280581	0.000274	hypomethylated
cg03849851	0.85163	0.701102162	-0.280602	5.19E-05	hypomethylated
cg03469804	0.74078	0.609807027	-0.280692	0.000772	hypomethylated

cg01470535	0.81343	0.669597838	-0.280723	0.000102	hypomethylated
cg25531857	0.69835	0.574835135	-0.280802	0.00095	hypomethylated
cg17838754	0.82947	0.682757297	-0.280817	5.86E-05	hypomethylated
cg05150973	0.85143	0.700804865	-0.280875	6.23E-05	hypomethylated
cg24743301	0.83747	0.68929027	-0.280926	0.000725	hypomethylated
cg14257263	0.7361	0.605813514	-0.281028	9.16E-05	hypomethylated
cg16790560	0.6733	0.554101081	-0.2811	0.00043	hypomethylated
cg25068071	0.74919	0.616507027	-0.281214	0.000551	hypomethylated
cg19991086	0.80602	0.663246486	-0.281271	6.01E-05	hypomethylated
cg07592216	0.75587	0.621964865	-0.281305	0.000234	hypomethylated
cg21790344	0.86271	0.709815676	-0.281431	2.27E-05	hypomethylated
cg06002157	0.84973	0.699046486	-0.281616	1.65E-06	hypomethylated
cg09075149	0.78295	0.643980541	-0.281903	0.0006	hypomethylated
cg16536740	0.70813	0.582414054	-0.281969	0.000696	hypomethylated
cg17282466	0.84211	0.692607568	-0.281971	4.58E-05	hypomethylated
cg07572233	0.78277	0.643773514	-0.282035	0.000551	hypomethylated
cg06097423	0.90904	0.747605946	-0.282066	8.84E-07	hypomethylated
cg06330587	0.6789	0.558322703	-0.2821	0.000221	hypomethylated
cg05318361	0.6566	0.539934054	-0.282232	0.002674	hypomethylated
cg06724409	0.78488	0.645363784	-0.282359	0.000335	hypomethylated
cg15601452	0.84059	0.691137838	-0.282429	0.000265	hypomethylated
cg19126910	0.70708	0.581327027	-0.282523	0.003255	hypomethylated
cg03162506	0.7654	0.629271351	-0.282532	0.000331	hypomethylated
cg24911198	0.72622	0.597044865	-0.282567	0.001118	hypomethylated
cg05426700	0.62819	0.516445946	-0.282584	0.000306	hypomethylated
cg09556939	0.7961	0.654478919	-0.282603	0.000176	hypomethylated
cg00966255	0.82988	0.682240541	-0.282622	6.54E-05	hypomethylated
cg09858811	0.70461	0.579216216	-0.282723	0.000229	hypomethylated
cg12048331	0.84288	0.692860541	-0.282762	0.000142	hypomethylated
cg18245316	0.7377	0.606388649	-0.282791	0.000274	hypomethylated
cg03737424	0.76386	0.627883784	-0.282811	0.000268	hypomethylated
cg26159905	0.69172	0.568584324	-0.282814	0.000172	hypomethylated
cg17550708	0.82887	0.681313514	-0.282827	2.35E-05	hypomethylated
cg01130611	0.8884	0.730148649	-0.283019	1.02E-05	hypomethylated
cg04835091	0.90527	0.744012432	-0.283021	2.04E-05	hypomethylated
cg18688392	0.80059	0.657941622	-0.283104	0.000116	hypomethylated
cg26615188	0.83995	0.690240541	-0.283204	0.000306	hypomethylated
cg08043998	0.70729	0.581224865	-0.283205	0.002274	hypomethylated
cg18634560	0.87406	0.71826	-0.283226	2.32E-05	hypomethylated
cg22600832	0.89539	0.735787027	-0.283228	6.93E-06	hypomethylated
cg26708427	0.82266	0.676009189	-0.283253	0.000209	hypomethylated
cg16568084	0.89571	0.73598973	-0.283346	2.46E-06	hypomethylated
cg06967304	0.79653	0.654367568	-0.283627	8.22E-05	hypomethylated
cg20181312	0.92437	0.759380541	-0.283647	1.58E-05	hypomethylated
cg03260781	0.68511	0.562808108	-0.283693	0.001367	hypomethylated
cg05308645	0.72277	0.593699459	-0.283804	0.000626	hypomethylated
cg27475088	0.63722	0.523418378	-0.283827	3.79E-06	hypomethylated
cg20329303	0.86087	0.707014054	-0.284056	7.38E-05	hypomethylated
cg15801433	0.6487	0.532745946	-0.284104	0.000207	hypomethylated
cg10604040	0.80073	0.657599459	-0.284107	1.94E-05	hypomethylated
cg22795769	0.73476	0.603380541	-0.284205	0.000232	hypomethylated
cg24951989	0.80017	0.657021622	-0.284366	0.000152	hypomethylated
cg16421157	0.83612	0.686527027	-0.284393	0.000104	hypomethylated
cg00350478	0.6189	0.508151351	-0.284448	7.29E-05	hypomethylated
cg13614606	0.85446	0.701548649	-0.28447	0.000123	hypomethylated
cg22259097	0.84251	0.691700541	-0.284546	2.42E-05	hypomethylated
cg26249852	0.75169	0.617125405	-0.284574	0.000313	hypomethylated
cg18861547	0.72611	0.596114595	-0.284598	0.00032	hypomethylated

cg21294616	0.88814	0.729127027	-0.284617	2.92E-05	hypomethylated
cg21186263	0.68945	0.566005405	-0.28463	0.000234	hypomethylated
cg10669597	0.92386	0.758423243	-0.284671	1.18E-06	hypomethylated
cg00079598	0.8065	0.66206	-0.284713	0.001423	hypomethylated
cg16241649	0.74977	0.615474054	-0.28475	0.000287	hypomethylated
cg22961699	0.88904	0.729628108	-0.285087	1.04E-05	hypomethylated
cg07677570	0.7275	0.597048108	-0.2851	0.000207	hypomethylated
cg15859311	0.76983	0.63176	-0.285163	2.61E-05	hypomethylated
cg25618916	0.66497	0.545704865	-0.285168	0.000101	hypomethylated
cg02978544	0.8446	0.693082703	-0.285241	4.47E-05	hypomethylated
cg03005191	0.87771	0.720239459	-0.285268	5.06E-05	hypomethylated
cg16624521	0.7767	0.63735027	-0.285271	0.000154	hypomethylated
cg10584914	0.77438	0.635441622	-0.285282	2.32E-05	hypomethylated
cg01562813	0.82558	0.677389189	-0.285423	0.000495	hypomethylated
cg14849421	0.76904	0.630921622	-0.285598	0.00028	hypomethylated
cg09147400	0.80495	0.660311892	-0.285752	8.03E-05	hypomethylated
cg01165661	0.72262	0.592744324	-0.285827	0.000374	hypomethylated
cg00476608	0.78137	0.640891351	-0.285926	0.001188	hypomethylated
cg02074763	0.85225	0.698983243	-0.286019	9.38E-05	hypomethylated
cg17161266	0.81094	0.665083784	-0.286059	0.000454	hypomethylated
cg11045198	0.86618	0.710341622	-0.286154	1.12E-05	hypomethylated
cg26922451	0.82109	0.673344865	-0.286195	4.47E-05	hypomethylated
cg27307975	0.83961	0.688520541	-0.28622	0.000104	hypomethylated
cg04434994	0.89012	0.729924324	-0.286253	1.71E-05	hypomethylated
cg22731190	0.75763	0.62126	-0.286296	4.30E-06	hypomethylated
cg16362014	0.73347	0.601428108	-0.286346	0.001587	hypomethylated
cg06019763	0.85653	0.702323243	-0.286369	0.000112	hypomethylated
cg25605174	0.76634	0.628283784	-0.286568	1.39E-05	hypomethylated
cg14858504	0.79631	0.652848649	-0.286582	3.67E-05	hypomethylated
cg08206517	0.7032	0.57642	-0.286815	0.000142	hypomethylated
cg04387059	0.84462	0.692336757	-0.286828	1.06E-05	hypomethylated
cg23682310	0.81285	0.666288108	-0.286843	8.32E-05	hypomethylated
cg02290550	0.64149	0.525818919	-0.286861	0.000178	hypomethylated
cg10331989	0.79719	0.653416216	-0.286921	0.000163	hypomethylated
cg26292910	0.74233	0.608437838	-0.286951	0.000517	hypomethylated
cg16320427	0.90437	0.741206486	-0.287038	9.82E-06	hypomethylated
cg18551353	0.78542	0.643691892	-0.287094	0.000155	hypomethylated
cg11179625	0.85882	0.703845946	-0.287096	0.000122	hypomethylated
cg12688942	0.76442	0.626446486	-0.287174	0.000155	hypomethylated
cg21125112	0.80159	0.656894595	-0.287203	0.000145	hypomethylated
cg26137915	0.86516	0.708988649	-0.287204	0.000256	hypomethylated
cg00943124	0.79834	0.654176757	-0.287323	1.25E-05	hypomethylated
cg23067535	0.71922	0.589316757	-0.28739	0.000157	hypomethylated
cg09179691	0.76969	0.630662703	-0.287409	9.49E-05	hypomethylated
cg26565120	0.80609	0.660428108	-0.287539	5.72E-05	hypomethylated
cg20102955	0.80323	0.658067027	-0.287579	0.000163	hypomethylated
cg14939765	0.79345	0.649946486	-0.287818	0.000245	hypomethylated
cg21804531	0.75399	0.61759027	-0.287895	0.000168	hypomethylated
cg11378979	0.81839	0.670292432	-0.287998	4.05E-05	hypomethylated
cg08118034	0.78991	0.64696	-0.288012	1.50E-05	hypomethylated
cg13146553	0.86604	0.709295135	-0.288048	1.35E-05	hypomethylated
cg20016569	0.84067	0.688508108	-0.288066	0.000163	hypomethylated
cg08039587	0.77332	0.633316757	-0.288138	0.000216	hypomethylated
cg23369364	0.80913	0.662639459	-0.288147	8.73E-05	hypomethylated
cg10631854	0.68141	0.557932973	-0.288431	0.000667	hypomethylated
cg24191847	0.77583	0.635234595	-0.288451	9.05E-05	hypomethylated
cg00724839	0.7511	0.614893514	-0.288668	0.000399	hypomethylated
cg15447017	0.88816	0.727084865	-0.288696	4.67E-06	hypomethylated

cg24183098	0.76571	0.626758378	-0.288889	0.00017	hypomethylated
cg09190795	0.87222	0.713916757	-0.288936	3.67E-05	hypomethylated
cg23345977	0.88156	0.721552432	-0.288955	8.37E-06	hypomethylated
cg11177614	0.90319	0.739237297	-0.288992	1.39E-05	hypomethylated
cg26713109	0.80088	0.655490811	-0.289011	0.000135	hypomethylated
cg14193806	0.80192	0.656305405	-0.289091	1.14E-05	hypomethylated
cg10031769	0.75639	0.619036216	-0.289106	0.000191	hypomethylated
cg09444426	0.84529	0.691756216	-0.289183	1.62E-05	hypomethylated
cg17437218	0.65161	0.533244865	-0.289211	0.000277	hypomethylated
cg18736676	0.95263	0.779567027	-0.289243	3.12E-06	hypomethylated
cg24852883	0.90988	0.744574595	-0.28926	8.48E-06	hypomethylated
cg16111488	0.87532	0.716285405	-0.289276	5.81E-06	hypomethylated
cg15789106	0.49801	0.407519459	-0.289306	0.00028	hypomethylated
cg25948831	0.89823	0.734975135	-0.289389	6.48E-06	hypomethylated
cg22542139	0.77877	0.637203784	-0.289442	0.000116	hypomethylated
cg10574843	0.90511	0.74051027	-0.289573	5.86E-05	hypomethylated
cg04415689	0.90261	0.738363784	-0.289771	4.07E-06	hypomethylated
cg12395919	0.86274	0.705708649	-0.289853	3.23E-05	hypomethylated
cg23172057	0.80826	0.661064865	-0.290028	8.03E-05	hypomethylated
cg02803141	0.88925	0.727287568	-0.290063	9.49E-05	hypomethylated
cg18245230	0.72222	0.590673514	-0.290077	0.000893	hypomethylated
cg18355311	0.75332	0.61604973	-0.290216	0.000523	hypomethylated
cg03807330	0.59869	0.489563784	-0.290312	0.000306	hypomethylated
cg13538475	0.85988	0.703135676	-0.290332	5.19E-05	hypomethylated
cg00185413	0.87481	0.715326486	-0.290368	1.54E-05	hypomethylated
cg13935127	0.82509	0.674638919	-0.290436	0.000313	hypomethylated
cg08853571	0.83267	0.680717297	-0.290689	5.93E-05	hypomethylated
cg00261668	0.73907	0.604194054	-0.290699	0.000262	hypomethylated
cg20989454	0.77737	0.635503243	-0.290702	5.65E-06	hypomethylated
cg25609954	0.76589	0.626034595	-0.290895	0.000256	hypomethylated
cg03276982	0.83728	0.684382162	-0.290908	0.000237	hypomethylated
cg13184814	0.81622	0.66716	-0.290925	3.49E-05	hypomethylated
cg19273668	0.80756	0.660075676	-0.290938	7.38E-05	hypomethylated
cg10633827	0.71686	0.585859459	-0.291137	5.93E-05	hypomethylated
cg00368296	0.84747	0.692595135	-0.29115	1.35E-05	hypomethylated
cg18209489	0.70642	0.577312432	-0.291174	5.86E-05	hypomethylated
cg26703956	0.85581	0.699374054	-0.291226	0.000207	hypomethylated
cg27110491	0.82001	0.67008973	-0.291287	2.27E-05	hypomethylated
cg17443080	0.6752	0.551748649	-0.291304	0.000435	hypomethylated
cg07855221	0.70986	0.580054595	-0.291346	0.001541	hypomethylated
cg03927131	0.88798	0.725516757	-0.291518	2.64E-05	hypomethylated
cg00391031	0.8878	0.72522973	-0.291797	1.50E-05	hypomethylated
cg07572435	0.77237	0.630917297	-0.291841	0.000166	hypomethylated
cg22280238	0.69589	0.568441081	-0.291848	0.001074	hypomethylated
cg13736939	0.73857	0.603281622	-0.291903	0.001571	hypomethylated
cg08468772	0.85393	0.697471892	-0.291983	4.53E-05	hypomethylated
cg15556591	0.85304	0.696744865	-0.291983	0.0002	hypomethylated
cg02174203	0.60087	0.490739459	-0.292096	0.00017	hypomethylated
cg19023552	0.57256	0.467596757	-0.292162	0.000335	hypomethylated
cg07886914	0.81987	0.669568649	-0.292163	8.84E-05	hypomethylated
cg19924352	0.77082	0.629496216	-0.292196	2.21E-05	hypomethylated
cg14741870	0.76759	0.626847027	-0.292222	0.00017	hypomethylated
cg16575694	0.84692	0.691616757	-0.292253	0.000109	hypomethylated
cg23257859	0.64164	0.523979459	-0.292254	0.000317	hypomethylated
cg22674248	0.80106	0.654044865	-0.292521	8.60E-06	hypomethylated
cg25483596	0.91128	0.744008108	-0.292576	2.21E-05	hypomethylated
cg03245218	0.83885	0.68484973	-0.292625	2.96E-05	hypomethylated
cg01933240	0.70228	0.573265946	-0.292842	0.000157	hypomethylated

cg25485805	0.68463	0.558845946	-0.292874	0.000581	hypomethylated
cg14340131	0.86671	0.707400541	-0.293022	5.43E-06	hypomethylated
cg10108710	0.91201	0.744346486	-0.293075	1.09E-05	hypomethylated
cg12302526	0.83031	0.677635676	-0.29314	2.32E-05	hypomethylated
cg12637085	0.76884	0.627467027	-0.293144	3.07E-05	hypomethylated
cg14852394	0.84497	0.689567568	-0.293208	0.000148	hypomethylated
cg26932364	0.87801	0.71651027	-0.29325	9.72E-05	hypomethylated
cg06296169	0.85331	0.696314054	-0.293332	2.89E-05	hypomethylated
cg00381442	0.66243	0.540518919	-0.293423	8.03E-05	hypomethylated
cg00830086	0.8547	0.697387027	-0.293459	4.47E-05	hypomethylated
cg24867501	0.65488	0.534329189	-0.293502	0.000174	hypomethylated
cg00013660	0.76191	0.621611892	-0.293606	1.62E-05	hypomethylated
cg03887218	0.8501	0.693558919	-0.293614	6.95E-05	hypomethylated
cg14009451	0.74116	0.604665405	-0.293648	9.84E-05	hypomethylated
cg00512454	0.81491	0.664810811	-0.293697	1.97E-05	hypomethylated
cg16781189	0.84011	0.685355135	-0.293726	2.49E-06	hypomethylated
cg10219789	0.70059	0.571535676	-0.293727	0.000145	hypomethylated
cg19778647	0.87536	0.714055135	-0.293841	0.000142	hypomethylated
cg02821464	0.63174	0.515297297	-0.293926	0.000517	hypomethylated
cg13863204	0.86665	0.706841081	-0.294064	2.85E-05	hypomethylated
cg26558862	0.92223	0.752145946	-0.294114	1.06E-05	hypomethylated
cg02047803	0.82123	0.669737297	-0.294191	1.08E-05	hypomethylated
cg22349332	0.71737	0.585028649	-0.29421	0.00071	hypomethylated
cg06852243	0.76411	0.623131892	-0.294243	0.000495	hypomethylated
cg14534803	0.76674	0.625234595	-0.29434	0.000166	hypomethylated
cg04231701	0.80904	0.659699459	-0.294402	3.53E-05	hypomethylated
cg27451550	0.91549	0.746475135	-0.29445	3.59E-06	hypomethylated
cg10460350	0.62085	0.506174054	-0.294611	0.001684	hypomethylated
cg02486268	0.79971	0.651987568	-0.294632	5.58E-05	hypomethylated
cg26609398	0.77087	0.628467027	-0.294651	0.000145	hypomethylated
cg17338299	0.78328	0.638578378	-0.294664	5.72E-05	hypomethylated
cg09417640	0.9255	0.754503784	-0.294705	3.30E-06	hypomethylated
cg00411411	0.6515	0.531115676	-0.294739	0.000185	hypomethylated
cg09414557	0.83872	0.683731892	-0.294759	2.07E-05	hypomethylated
cg15712520	0.70942	0.578293514	-0.294838	0.00024	hypomethylated
cg01032675	0.76506	0.623640541	-0.294858	0.000848	hypomethylated
cg09936663	0.90925	0.741130811	-0.294949	1.18E-05	hypomethylated
cg03749697	0.88856	0.724207568	-0.295066	1.12E-05	hypomethylated
cg02532672	0.64366	0.524596757	-0.29509	0.000207	hypomethylated
cg17746130	0.73256	0.596996216	-0.295225	8.03E-05	hypomethylated
cg16545821	0.82757	0.674410811	-0.295254	0.000107	hypomethylated
cg17598552	0.63853	0.520344865	-0.295286	0.000528	hypomethylated
cg16713321	0.84461	0.688278378	-0.295293	8.22E-05	hypomethylated
cg24689061	0.92997	0.75782	-0.295329	0.00035	hypomethylated
cg07658646	0.8456	0.689039459	-0.295389	0.00017	hypomethylated
cg02319094	0.61767	0.503305946	-0.295401	0.000335	hypomethylated
cg16780771	0.75537	0.6155	-0.295425	2.64E-05	hypomethylated
cg08795515	0.80288	0.654152973	-0.295556	6.46E-05	hypomethylated
cg04213073	0.8545	0.696131351	-0.295721	0.000137	hypomethylated
cg19251500	0.79081	0.644161081	-0.29591	1.80E-05	hypomethylated
cg24121168	0.71156	0.579607027	-0.29591	0.00078	hypomethylated
cg01798385	0.75325	0.613563784	-0.295915	5.58E-05	hypomethylated
cg24236839	0.62411	0.508332973	-0.296027	0.002803	hypomethylated
cg04665930	0.78757	0.641357297	-0.29628	4.15E-05	hypomethylated
cg04018738	0.94346	0.768302703	-0.296287	2.39E-05	hypomethylated
cg19376858	0.86368	0.703328649	-0.296298	0.000187	hypomethylated
cg16681436	0.82117	0.668660541	-0.296407	7.41E-06	hypomethylated
cg22725222	0.70849	0.576901081	-0.296424	0.000107	hypomethylated

cg13169491	0.88128	0.717563243	-0.296494	2.15E-05	hypomethylated
cg04723137	0.85921	0.699573514	-0.296535	5.86E-05	hypomethylated
cg08298644	0.67284	0.547814595	-0.296576	0.00049	hypomethylated
cg10284310	0.91637	0.746053514	-0.296651	1.82E-05	hypomethylated
cg04894027	0.72952	0.593846486	-0.296857	0.000145	hypomethylated
cg03722472	0.80524	0.655439459	-0.296956	1.35E-05	hypomethylated
cg09526712	0.95995	0.781337297	-0.297014	3.90E-06	hypomethylated
cg15039415	0.85459	0.69558	-0.297016	5.58E-06	hypomethylated
cg18623836	0.81994	0.66736973	-0.297032	0.000245	hypomethylated
cg12466022	0.91607	0.74556	-0.297133	1.04E-05	hypomethylated
cg02650128	0.80959	0.658870811	-0.297196	4.58E-05	hypomethylated
cg01801643	0.82689	0.672938919	-0.29722	0.000517	hypomethylated
cg21473545	0.68091	0.554137297	-0.297221	0.002253	hypomethylated
cg22396498	0.80839	0.657835676	-0.297324	0.000224	hypomethylated
cg05168404	0.5895	0.479705405	-0.297343	0.000696	hypomethylated
cg05498539	0.81363	0.662088649	-0.297348	5.58E-06	hypomethylated
cg17234201	0.83798	0.681863784	-0.297432	1.46E-05	hypomethylated
cg11763800	0.79654	0.648137838	-0.297446	0.00017	hypomethylated
cg01243950	0.88848	0.722916216	-0.297511	1.77E-05	hypomethylated
cg19136371	0.84814	0.690051351	-0.297599	0.000268	hypomethylated
cg00115149	0.90358	0.7351	-0.297712	2.67E-05	hypomethylated
cg10844275	0.87823	0.714443243	-0.297779	2.57E-05	hypomethylated
cg04149930	0.83909	0.682588649	-0.297809	4.88E-05	hypomethylated
cg17150809	0.79844	0.649508649	-0.297835	0.000268	hypomethylated
cg11906607	0.76579	0.622906486	-0.297933	5.06E-05	hypomethylated
cg06877366	0.88238	0.717717838	-0.297983	7.20E-05	hypomethylated
cg19407392	0.8124	0.660753514	-0.298078	0.000135	hypomethylated
cg26774156	0.87882	0.714734054	-0.298161	9.72E-05	hypomethylated
cg03478444	0.82676	0.672381081	-0.298189	0.000176	hypomethylated
cg08830588	0.72683	0.591101622	-0.298212	0.000224	hypomethylated
cg13686739	0.74646	0.607045946	-0.298259	1.50E-05	hypomethylated
cg17747551	0.88797	0.722125946	-0.29826	6.01E-05	hypomethylated
cg05302441	0.67698	0.550482703	-0.298416	0.00113	hypomethylated
cg21620606	0.85914	0.698554054	-0.298521	5.06E-05	hypomethylated
cg01321174	0.68696	0.558518378	-0.298621	4.70E-05	hypomethylated
cg18759732	0.81714	0.664292432	-0.298765	0.000216	hypomethylated
cg17223698	0.83586	0.679318378	-0.299173	5.50E-06	hypomethylated
cg14592798	0.88142	0.716324865	-0.299216	2.32E-05	hypomethylated
cg14084826	0.74759	0.60752	-0.299315	0.000123	hypomethylated
cg10596692	0.89491	0.727195135	-0.2994	2.91E-06	hypomethylated
cg22092292	0.81343	0.660974595	-0.299423	2.04E-05	hypomethylated
cg13756898	0.79513	0.646063243	-0.299515	0.00014	hypomethylated
cg26921566	0.71454	0.580562162	-0.299564	0.000195	hypomethylated
cg12962167	0.87248	0.708860541	-0.29962	0.000174	hypomethylated
cg04804814	0.65974	0.536002162	-0.299659	4.47E-05	hypomethylated
cg05726758	0.7271	0.59071027	-0.299703	0.000189	hypomethylated
cg25737397	0.85867	0.697591351	-0.299722	3.32E-05	hypomethylated
cg07580831	0.77946	0.633183243	-0.299852	0.000324	hypomethylated
cg26893451	0.66972	0.543994054	-0.299967	9.38E-05	hypomethylated
cg01638529	0.76825	0.623956216	-0.300131	3.76E-05	hypomethylated
cg22158252	0.79428	0.645085946	-0.300156	1.66E-05	hypomethylated
cg26167010	0.83342	0.676853514	-0.3002	3.67E-05	hypomethylated
cg06595022	0.94208	0.765082162	-0.300235	5.06E-05	hypomethylated
cg02021180	0.85706	0.696010811	-0.300286	0.000229	hypomethylated
cg01418951	0.76793	0.623624865	-0.300296	0.001288	hypomethylated
cg04997124	0.85629	0.695378919	-0.3003	3.90E-06	hypomethylated
cg16153549	0.83337	0.676748649	-0.300337	0.000563	hypomethylated
cg23516121	0.90414	0.734089189	-0.300591	2.45E-05	hypomethylated

cg23720898	0.79517	0.645585946	-0.300654	8.52E-05	hypomethylated
cg12251145	0.76124	0.618008108	-0.300726	0.000338	hypomethylated
cg02223135	0.91101	0.739555676	-0.300808	1.56E-05	hypomethylated
cg25078908	0.74204	0.602349189	-0.300897	0.000129	hypomethylated
cg02202411	0.89938	0.73006	-0.300916	2.32E-05	hypomethylated
cg17974515	0.7281	0.59102	-0.30093	0.000159	hypomethylated
cg04055086	0.88103	0.715154595	-0.300936	1.01E-05	hypomethylated
cg22200744	0.77081	0.625663784	-0.300988	0.000229	hypomethylated
cg21201924	0.69368	0.563057297	-0.300989	5.72E-05	hypomethylated
cg08989290	0.69506	0.564160541	-0.301032	0.00099	hypomethylated
cg14624731	0.66915	0.54312973	-0.301033	0.000789	hypomethylated
cg20840174	0.75344	0.611501081	-0.301138	0.000822	hypomethylated
cg25023198	0.71663	0.581593514	-0.301217	0.003316	hypomethylated
cg05953316	0.70901	0.575405946	-0.301226	6.62E-05	hypomethylated
cg04789475	0.74293	0.602881081	-0.301353	0.001041	hypomethylated
cg07233952	0.72309	0.586777838	-0.301361	0.001395	hypomethylated
cg25456144	0.85179	0.691212973	-0.301367	4.82E-05	hypomethylated
cg20344448	0.73063	0.592883243	-0.301393	0.000335	hypomethylated
cg03339956	0.60893	0.494123784	-0.301404	9.49E-05	hypomethylated
cg07324752	0.694	0.563144324	-0.301431	0.000229	hypomethylated
cg07183691	0.89423	0.725611892	-0.301448	5.00E-06	hypomethylated
cg27438128	0.70748	0.574054054	-0.301503	0.000268	hypomethylated
cg26493306	0.92465	0.750234054	-0.301567	6.30E-06	hypomethylated
cg16117554	0.80188	0.650598378	-0.301619	0.000182	hypomethylated
cg09366118	0.66719	0.541318378	-0.30162	0.001225	hypomethylated
cg03947749	0.8154	0.661552432	-0.301652	6.62E-05	hypomethylated
cg11228624	0.71136	0.577068108	-0.301838	2.92E-05	hypomethylated
cg09169739	0.8246	0.66887027	-0.301968	0.000251	hypomethylated
cg18922620	0.82333	0.667815676	-0.302021	0.000575	hypomethylated
cg09620688	0.70335	0.57046	-0.302117	4.82E-05	hypomethylated
cg12895370	0.83198	0.674779459	-0.302133	0.00018	hypomethylated
cg15500259	0.75805	0.614784865	-0.302211	9.31E-06	hypomethylated
cg09508490	0.81157	0.658132973	-0.302336	1.39E-05	hypomethylated
cg07147614	0.58197	0.471936757	-0.302351	0.000342	hypomethylated
cg19140928	0.8876	0.719772432	-0.302369	1.80E-05	hypomethylated
cg18485872	0.8098	0.656676216	-0.302383	5.58E-05	hypomethylated
cg12711059	0.75794	0.614599459	-0.302437	0.000262	hypomethylated
cg07854113	0.83889	0.680232973	-0.302453	0.000187	hypomethylated
cg11833938	0.97237	0.78840973	-0.30256	1.15E-06	hypomethylated
cg03806812	0.63701	0.516477297	-0.302611	0.001118	hypomethylated
cg26435198	0.75286	0.610401081	-0.302624	7.20E-05	hypomethylated
cg05607079	0.8949	0.725553514	-0.302644	3.67E-05	hypomethylated
cg20424781	0.88941	0.721067568	-0.302714	5.21E-06	hypomethylated
cg08729600	0.88845	0.72025027	-0.302792	1.06E-05	hypomethylated
cg03666300	0.86301	0.699597297	-0.302853	2.02E-05	hypomethylated
cg12619162	0.637	0.51638	-0.30286	0.000174	hypomethylated
cg22658979	0.93812	0.76048	-0.302862	1.12E-06	hypomethylated
cg21885134	0.7494	0.607484865	-0.302888	5.00E-06	hypomethylated
cg00594191	0.79773	0.646597297	-0.303033	0.000667	hypomethylated
cg07236001	0.79445	0.643926486	-0.30306	0.000445	hypomethylated
cg26632171	0.79446	0.643877838	-0.303188	0.000152	hypomethylated
cg26029221	0.66214	0.536630811	-0.303206	0.000191	hypomethylated
cg09934892	0.78537	0.636488649	-0.303238	0.000594	hypomethylated
cg15003194	0.79348	0.64304973	-0.303264	5.00E-05	hypomethylated
cg27128311	0.87733	0.71098973	-0.303291	0.000382	hypomethylated
cg17305275	0.77774	0.630191351	-0.303498	0.000122	hypomethylated
cg25781868	0.7108	0.57595027	-0.303499	0.000104	hypomethylated
cg21875839	0.81546	0.660711892	-0.303593	0.000178	hypomethylated

cg13560058	0.6879	0.557357838	-0.303595	0.000358	hypomethylated
cg13697715	0.57823	0.468488108	-0.303631	0.000145	hypomethylated
cg05655190	0.60551	0.490561622	-0.303716	0.012637	hypomethylated
cg19925599	0.75839	0.614414054	-0.303729	7.84E-05	hypomethylated
cg01727585	0.73162	0.592694054	-0.303807	0.000182	hypomethylated
cg18758900	0.82559	0.668815676	-0.303817	0.000214	hypomethylated
cg21880020	0.77173	0.625159459	-0.303872	4.47E-05	hypomethylated
cg03515464	0.82442	0.667798919	-0.303966	1.12E-05	hypomethylated
cg05877661	0.89077	0.721515135	-0.304023	0.000191	hypomethylated
cg13958199	0.76821	0.622223243	-0.304068	0.000426	hypomethylated
cg23755074	0.86316	0.699124865	-0.304078	3.95E-05	hypomethylated
cg01188509	0.8182	0.662703784	-0.304089	2.39E-05	hypomethylated
cg00795584	0.76885	0.622731351	-0.304092	0.000626	hypomethylated
cg21194726	0.80063	0.648396757	-0.304259	0.000191	hypomethylated
cg11358199	0.74148	0.600491351	-0.304264	0.003255	hypomethylated
cg19145320	0.84594	0.685057297	-0.304331	0.000109	hypomethylated
cg09886009	0.76904	0.622703243	-0.304514	7.65E-05	hypomethylated
cg23371476	0.86038	0.696647027	-0.304546	0.000189	hypomethylated
cg08658895	0.78372	0.634562703	-0.304576	2.57E-05	hypomethylated
cg27579097	0.82884	0.67108973	-0.304588	0.000209	hypomethylated
cg04957903	0.75944	0.614892973	-0.304601	0.00134	hypomethylated
cg17827767	0.77062	0.623927027	-0.304642	0.000342	hypomethylated
cg01219549	0.97197	0.786945405	-0.304648	3.49E-06	hypomethylated
cg17800396	0.65155	0.527488649	-0.304736	0.000342	hypomethylated
cg21746275	0.83725	0.677810811	-0.304776	0.000256	hypomethylated
cg21562656	0.86204	0.697787568	-0.304967	1.09E-05	hypomethylated
cg01000553	0.79249	0.64147027	-0.30501	2.74E-05	hypomethylated
cg10964472	0.89709	0.726133514	-0.305018	0.000382	hypomethylated
cg03489712	0.70943	0.574230811	-0.30503	0.001164	hypomethylated
cg08699270	0.7894	0.638942703	-0.30507	0.001164	hypomethylated
cg02843667	0.78498	0.635363784	-0.305073	0.000106	hypomethylated
cg14138287	0.77971	0.631078378	-0.305118	1.84E-05	hypomethylated
cg09560549	0.75013	0.607106486	-0.305191	0.002502	hypomethylated
cg16606561	0.89927	0.727740541	-0.30533	7.03E-06	hypomethylated
cg13696706	0.62432	0.505235135	-0.305331	0.001987	hypomethylated
cg14338073	0.76971	0.622864865	-0.305396	0.000789	hypomethylated
cg18506018	0.78117	0.63212973	-0.305416	0.000256	hypomethylated
cg08134671	0.74034	0.599057838	-0.305493	0.002066	hypomethylated
cg03291825	0.6191	0.500911351	-0.305617	0.000193	hypomethylated
cg00733493	0.66162	0.535300541	-0.305654	0.000107	hypomethylated
cg15090036	0.87471	0.707691892	-0.305683	7.32E-06	hypomethylated
cg03078586	0.82435	0.666942162	-0.305695	3.07E-05	hypomethylated
cg08376089	0.826	0.668271892	-0.305707	3.49E-05	hypomethylated
cg17972162	0.71281	0.576695135	-0.305709	0.000408	hypomethylated
cg03502236	0.64713	0.523485405	-0.305906	0.0002	hypomethylated
cg13180252	0.5736	0.463994054	-0.305939	0.000283	hypomethylated
cg15708776	0.68757	0.55614	-0.306058	2.10E-05	hypomethylated
cg04822278	0.78937	0.638472973	-0.306076	0.00054	hypomethylated
cg05385282	0.69666	0.563477838	-0.306096	0.000251	hypomethylated
cg23452957	0.51555	0.416985405	-0.306115	0.000178	hypomethylated
cg04497752	0.8394	0.678894054	-0.306172	3.64E-06	hypomethylated
cg04727357	0.69978	0.565969189	-0.306178	0.001107	hypomethylated
cg08860639	0.75568	0.611174054	-0.306192	0.000116	hypomethylated
cg14271150	0.7816	0.632137297	-0.306193	1.30E-05	hypomethylated
cg19206437	0.79206	0.640555135	-0.306287	3.76E-05	hypomethylated
cg00994323	0.86132	0.696559459	-0.306303	7.56E-05	hypomethylated
cg09490523	0.84171	0.680665946	-0.306376	0.000216	hypomethylated
cg25786651	0.62084	0.502039459	-0.306421	0.000178	hypomethylated

cg26140366	0.78365	0.633687027	-0.306439	4.36E-05	hypomethylated
cg05076755	0.73007	0.590297838	-0.306592	0.004567	hypomethylated
cg23088157	0.78104	0.631503243	-0.306606	4.64E-05	hypomethylated
cg14112978	0.84986	0.687145946	-0.306609	3.15E-05	hypomethylated
cg26475094	0.91874	0.742808108	-0.306667	8.37E-06	hypomethylated
cg04260633	0.61308	0.495668108	-0.306701	5.72E-05	hypomethylated
cg04130356	0.76852	0.621283243	-0.306832	0.000145	hypomethylated
cg18182039	0.80826	0.653402703	-0.306847	0.000506	hypomethylated
cg27054847	0.70705	0.571422703	-0.307254	9.27E-05	hypomethylated
cg01166892	0.72686	0.587428108	-0.307265	0.000733	hypomethylated
cg22322679	0.75161	0.607366486	-0.307417	8.84E-05	hypomethylated
cg00484484	0.88749	0.717152973	-0.30745	2.10E-05	hypomethylated
cg25455811	0.79594	0.643112973	-0.307587	0.000112	hypomethylated
cg01846046	0.93452	0.755078378	-0.307599	8.52E-05	hypomethylated
cg23059700	0.81499	0.658496757	-0.307606	0.00013	hypomethylated
cg26328951	0.82299	0.664912432	-0.307711	0.000756	hypomethylated
cg17633681	0.8412	0.679604865	-0.307753	0.000126	hypomethylated
cg00691948	0.81343	0.657155135	-0.307784	0.000214	hypomethylated
cg06434709	0.87147	0.704043784	-0.307786	3.15E-05	hypomethylated
cg21720385	0.77911	0.629343243	-0.30798	0.000102	hypomethylated
cg26688993	0.84722	0.684353514	-0.307995	0.000126	hypomethylated
cg07285481	0.70747	0.571431892	-0.308087	0.00012	hypomethylated
cg12742178	0.81705	0.659940541	-0.308088	5.19E-05	hypomethylated
cg11970177	0.89587	0.723587568	-0.308122	1.28E-05	hypomethylated
cg15044181	0.85462	0.690255676	-0.308152	5.58E-05	hypomethylated
cg04436971	0.65218	0.526748649	-0.308155	6.70E-05	hypomethylated
cg21962760	0.68115	0.550143784	-0.308164	4.70E-05	hypomethylated
cg23476401	0.64082	0.51756	-0.308193	0.000506	hypomethylated
cg22971029	0.70681	0.570837838	-0.308241	0.000517	hypomethylated
cg08969922	0.77868	0.628807568	-0.308412	0.000152	hypomethylated
cg01849903	0.51291	0.414184324	-0.308433	8.42E-05	hypomethylated
cg17992670	0.88604	0.71548973	-0.308441	7.12E-06	hypomethylated
cg20683057	0.53909	0.435301081	-0.308513	0.000287	hypomethylated
cg19965300	0.75149	0.606803243	-0.308525	0.000417	hypomethylated
cg18831260	0.84193	0.67982	-0.308547	2.24E-05	hypomethylated
cg05501498	0.80165	0.647224324	-0.308707	0.000335	hypomethylated
cg08290013	0.65098	0.525571892	-0.308725	0.000262	hypomethylated
cg11698445	0.77514	0.625794595	-0.308768	0.000403	hypomethylated
cg19202886	0.83929	0.677473514	-0.309005	3.07E-05	hypomethylated
cg07833104	0.64956	0.524319459	-0.309017	0.000435	hypomethylated
cg16386293	0.73233	0.591116216	-0.309052	9.16E-05	hypomethylated
cg04903916	0.88496	0.7143	-0.309082	1.82E-05	hypomethylated
cg19130824	0.8105	0.654196757	-0.309088	3.49E-05	hypomethylated
cg10191283	0.79106	0.638503784	-0.309092	4.58E-05	hypomethylated
cg11872478	0.78215	0.631301081	-0.309117	9.72E-05	hypomethylated
cg18104015	0.60775	0.490494595	-0.309241	9.38E-05	hypomethylated
cg24145685	0.84373	0.680938378	-0.309257	2.24E-05	hypomethylated
cg05886261	0.79389	0.64068973	-0.309313	4.94E-05	hypomethylated
cg07908984	0.77051	0.621809189	-0.309342	0.000216	hypomethylated
cg13893707	0.80816	0.652172973	-0.309386	0.000148	hypomethylated
cg07037509	0.7971	0.643235676	-0.309413	7.56E-05	hypomethylated
cg09839204	0.86954	0.701691892	-0.309415	1.46E-05	hypomethylated
cg01534390	0.81387	0.656765405	-0.30942	5.72E-05	hypomethylated
cg13617376	0.86698	0.699561081	-0.309549	3.67E-05	hypomethylated
cg26684191	0.72951	0.588624865	-0.309579	2.78E-05	hypomethylated
cg02352181	0.83595	0.674491892	-0.309616	9.49E-05	hypomethylated
cg07757007	0.87894	0.709175135	-0.309623	2.27E-05	hypomethylated
cg00141925	0.89791	0.724478919	-0.309627	1.77E-06	hypomethylated

cg01345055	0.58239	0.469815135	-0.309892	0.002026	hypomethylated
cg10959672	0.7643	0.616560541	-0.309896	0.00095	hypomethylated
cg14165772	0.84107	0.678465405	-0.309951	0.000178	hypomethylated
cg21838773	0.65362	0.527249189	-0.309967	0.001717	hypomethylated
cg26088629	0.89504	0.721990811	-0.309972	0.000102	hypomethylated
cg12256856	0.92019	0.742258919	-0.310009	5.34E-07	hypomethylated
cg12543918	0.77074	0.621675676	-0.310082	0.000653	hypomethylated
cg18366480	0.85326	0.688230811	-0.310093	4.82E-05	hypomethylated
cg02171804	0.82369	0.664306486	-0.310252	3.39E-06	hypomethylated
cg00423068	0.86994	0.701605405	-0.310256	1.99E-05	hypomethylated
cg01148741	0.64859	0.523029189	-0.310415	0.001212	hypomethylated
cg22263591	0.72556	0.585092973	-0.310429	4.70E-05	hypomethylated
cg24965322	0.90126	0.72674	-0.310504	2.71E-06	hypomethylated
cg15260921	0.66686	0.53770973	-0.310556	0.00134	hypomethylated
cg02696561	0.85861	0.69226	-0.310689	9.57E-06	hypomethylated
cg13390372	0.86649	0.698565405	-0.310788	7.93E-06	hypomethylated
cg04903600	0.65833	0.530743784	-0.310795	5.31E-05	hypomethylated
cg26766064	0.69792	0.562618919	-0.310904	0.000109	hypomethylated
cg21515113	0.77339	0.623440541	-0.310944	9.05E-05	hypomethylated
cg00845319	0.80593	0.649670811	-0.310946	8.84E-05	hypomethylated
cg25312122	0.83403	0.672318919	-0.310954	9.83E-05	hypomethylated
cg05581394	0.87678	0.706730811	-0.311054	1.62E-05	hypomethylated
cg17540765	0.77827	0.627316757	-0.311077	6.62E-05	hypomethylated
cg14742187	0.83004	0.669011892	-0.311149	0.000148	hypomethylated
cg08840230	0.84352	0.679843784	-0.311219	4.94E-05	hypomethylated
cg11815984	0.82104	0.661725405	-0.31122	0.000209	hypomethylated
cg19307406	0.74267	0.598478919	-0.311421	9.72E-05	hypomethylated
cg15618336	0.90572	0.729847568	-0.31147	1.71E-05	hypomethylated
cg24134056	0.8229	0.663083243	-0.311527	1.25E-05	hypomethylated
cg13798885	0.85322	0.687480541	-0.311599	0.000152	hypomethylated
cg05935961	0.64551	0.520113514	-0.311613	0.000369	hypomethylated
cg03216884	0.93656	0.754604324	-0.311651	1.72E-06	hypomethylated
cg21048812	0.8184	0.659395676	-0.311662	0.000378	hypomethylated
cg25098478	0.61072	0.492035676	-0.311748	6.62E-05	hypomethylated
cg00919126	0.7232	0.582647027	-0.311773	0.00048	hypomethylated
cg19475067	0.78269	0.630558378	-0.311811	1.08E-05	hypomethylated
cg05308970	0.84621	0.681700541	-0.311878	2.23E-06	hypomethylated
cg11633264	0.60248	0.485287568	-0.312073	0.001212	hypomethylated
cg24729988	0.81522	0.656638378	-0.31209	2.24E-05	hypomethylated
cg11105610	0.68398	0.550919459	-0.312113	0.000271	hypomethylated
cg02950892	0.71484	0.575756757	-0.312161	0.00028	hypomethylated
cg15190149	0.86015	0.692714595	-0.312327	2.51E-05	hypomethylated
cg03150409	0.82869	0.667341081	-0.312408	8.84E-05	hypomethylated
cg00736104	0.73278	0.590049189	-0.312545	0.001314	hypomethylated
cg04509526	0.83514	0.672446486	-0.312599	3.49E-05	hypomethylated
cg03513893	0.83957	0.675993514	-0.312641	6.75E-06	hypomethylated
cg12600109	0.98113	0.789959459	-0.312666	8.26E-06	hypomethylated
cg12556823	0.92505	0.7448	-0.312678	2.10E-06	hypomethylated
cg04755561	0.75226	0.605645405	-0.312758	0.00028	hypomethylated
cg00082285	0.74369	0.598728649	-0.312799	1.82E-05	hypomethylated
cg08711320	0.87293	0.702689189	-0.312979	7.56E-05	hypomethylated
cg09785394	0.70539	0.567787027	-0.313071	0.00039	hypomethylated
cg17121205	0.84993	0.684128108	-0.313078	3.04E-05	hypomethylated
cg18770029	0.75993	0.611673514	-0.313105	0.000137	hypomethylated
cg00004562	0.78599	0.632641622	-0.313122	1.40E-05	hypomethylated
cg24889829	0.88587	0.712921081	-0.313353	6.57E-06	hypomethylated
cg27651070	0.66857	0.538021622	-0.313414	2.27E-05	hypomethylated
cg18049474	0.71038	0.571634054	-0.313499	6.38E-05	hypomethylated

cg14406501	0.82033	0.660105405	-0.313508	9.27E-05	hypomethylated
cg03457984	0.83258	0.669960541	-0.313513	4.70E-05	hypomethylated
cg03525467	0.72841	0.586117838	-0.31356	2.45E-05	hypomethylated
cg19293162	0.68072	0.54769027	-0.313701	0.000109	hypomethylated
cg04753836	0.71956	0.578938378	-0.313705	0.00024	hypomethylated
cg26117369	0.78822	0.634175676	-0.313716	0.00039	hypomethylated
cg00107241	0.85419	0.687224865	-0.313775	0.000191	hypomethylated
cg26560699	0.83257	0.669818378	-0.313802	5.32E-05	hypomethylated
cg04946315	0.73081	0.587936757	-0.313835	0.000523	hypomethylated
cg12978582	0.62487	0.502695135	-0.313872	0.000242	hypomethylated
cg17277248	0.88998	0.715950811	-0.313912	2.61E-05	hypomethylated
cg25591377	0.89607	0.72082	-0.313972	4.60E-06	hypomethylated
cg03130962	0.68292	0.549300541	-0.314121	0.000154	hypomethylated
cg17619755	0.7814	0.628486486	-0.31418	0.000154	hypomethylated
cg06516820	0.76022	0.611421622	-0.314249	9.31E-06	hypomethylated
cg09097916	0.77482	0.623161622	-0.314255	0.000191	hypomethylated
cg14220678	0.74065	0.595678919	-0.314257	0.001118	hypomethylated
cg11143092	0.76372	0.614228108	-0.314269	0.000338	hypomethylated
cg15851278	0.81184	0.652928108	-0.314271	5.00E-05	hypomethylated
cg16149437	0.74441	0.598676757	-0.31432	0.001063	hypomethylated
cg01928143	0.92767	0.746050811	-0.314338	1.58E-05	hypomethylated
cg03262802	0.73447	0.590609189	-0.3145	7.12E-05	hypomethylated
cg17767484	0.84502	0.679467568	-0.314581	0.000143	hypomethylated
cg06042156	0.63971	0.514372973	-0.314603	0.000195	hypomethylated
cg09709565	0.77766	0.625241081	-0.314727	0.000251	hypomethylated
cg16685586	0.86	0.691418378	-0.314778	4.05E-05	hypomethylated
cg02229288	0.80918	0.650558919	-0.314781	0.000653	hypomethylated
cg06204229	0.82239	0.661178378	-0.314783	7.72E-06	hypomethylated
cg13906098	0.82605	0.664098378	-0.314832	0.000204	hypomethylated
cg07481027	0.85076	0.683904324	-0.314958	0.000148	hypomethylated
cg09686390	0.70626	0.567731892	-0.31499	0.000229	hypomethylated
cg07813495	0.69165	0.555975135	-0.315022	0.002168	hypomethylated
cg01145653	0.87681	0.704792432	-0.315066	3.32E-05	hypomethylated
cg18045152	0.79569	0.639568649	-0.315107	2.78E-05	hypomethylated
cg06700061	0.90562	0.727928649	-0.315109	3.04E-05	hypomethylated
cg22939709	0.7865	0.632177838	-0.315116	8.84E-05	hypomethylated
cg10940251	0.64115	0.515303243	-0.31524	0.000265	hypomethylated
cg22478258	0.6461	0.519256216	-0.315311	0.000464	hypomethylated
cg23181035	0.64625	0.519338378	-0.315418	0.000251	hypomethylated
cg04388863	0.86348	0.693890811	-0.315454	3.19E-05	hypomethylated
cg09501282	0.82077	0.659552432	-0.315491	6.15E-05	hypomethylated
cg21407354	0.86203	0.692703784	-0.3155	5.86E-05	hypomethylated
cg10102736	0.78515	0.630922162	-0.315506	9.57E-06	hypomethylated
cg19979896	0.8144	0.654423784	-0.315512	0.000293	hypomethylated
cg10481560	0.85476	0.686826486	-0.315574	1.46E-05	hypomethylated
cg08666707	0.54107	0.434757838	-0.315603	0.000229	hypomethylated
cg00906908	0.82046	0.65924973	-0.315608	0.000174	hypomethylated
cg18128976	0.83207	0.668551351	-0.315667	2.92E-05	hypomethylated
cg26681123	0.79203	0.636327027	-0.315787	1.09E-05	hypomethylated
cg18061140	0.88335	0.709676216	-0.315824	1.05E-06	hypomethylated
cg10715147	0.90956	0.730695676	-0.315898	3.32E-05	hypomethylated
cg26155170	0.72927	0.585832973	-0.315964	0.000335	hypomethylated
cg09596131	0.77436	0.622022162	-0.316038	0.003255	hypomethylated
cg24312489	0.6152	0.494150811	-0.316104	0.000667	hypomethylated
cg09957933	0.8676	0.696858378	-0.316165	3.67E-05	hypomethylated
cg14029117	0.74224	0.596167568	-0.316168	8.13E-05	hypomethylated
cg24691965	0.86685	0.696248649	-0.31618	3.40E-05	hypomethylated
cg11067179	0.7512	0.603267027	-0.3164	0.000358	hypomethylated

cg00549601	0.74463	0.597974054	-0.316441	0.000256	hypomethylated
cg10963033	0.87212	0.700345946	-0.316459	2.27E-05	hypomethylated
cg08458733	0.6585	0.52877027	-0.316542	0.001041	hypomethylated
cg13705014	0.94062	0.755306486	-0.31655	3.53E-05	hypomethylated
cg22717593	0.79007	0.634392432	-0.316605	0.000224	hypomethylated
cg09205751	0.75413	0.605515135	-0.31665	0.000182	hypomethylated
cg03264209	0.58208	0.467352973	-0.316705	0.000117	hypomethylated
cg22919908	0.71568	0.5746	-0.316757	9.05E-05	hypomethylated
cg08378932	0.78605	0.631092432	-0.31677	1.71E-05	hypomethylated
cg18289306	0.76788	0.616503784	-0.316771	8.42E-05	hypomethylated
cg03550233	0.57565	0.462162703	-0.316791	0.001327	hypomethylated
cg25616918	0.8492	0.681773514	-0.316812	0.000117	hypomethylated
cg22584435	0.74292	0.596436216	-0.316839	5.86E-05	hypomethylated
cg16668903	0.8004	0.642541622	-0.316931	2.81E-05	hypomethylated
cg08245989	0.79317	0.636700541	-0.317015	2.51E-05	hypomethylated
cg02915739	0.64088	0.51445027	-0.317023	0.000165	hypomethylated
cg21399807	0.90786	0.728761622	-0.317023	1.68E-05	hypomethylated
cg09674500	0.89737	0.720333514	-0.317038	1.35E-05	hypomethylated
cg03750061	0.84153	0.67544973	-0.317166	0.000107	hypomethylated
cg00500770	0.78577	0.630688649	-0.317179	0.000226	hypomethylated
cg12795208	0.82909	0.665423243	-0.317256	4.36E-05	hypomethylated
cg15369784	0.86452	0.693806486	-0.317366	1.06E-05	hypomethylated
cg03126567	0.84597	0.678824324	-0.317568	7.65E-05	hypomethylated
cg13521045	0.83995	0.673981081	-0.317595	1.54E-05	hypomethylated
cg21242002	0.65695	0.5271	-0.317707	0.001225	hypomethylated
cg22208304	0.95361	0.765108649	-0.317735	1.04E-05	hypomethylated
cg14258078	0.86258	0.692022703	-0.317839	3.00E-05	hypomethylated
cg20731031	0.86366	0.692848649	-0.317923	1.48E-05	hypomethylated
cg02790458	0.67069	0.53802	-0.317986	0.00012	hypomethylated
cg17218041	0.75337	0.604278919	-0.318144	0.000251	hypomethylated
cg18741321	0.7946	0.637344324	-0.318156	7.12E-06	hypomethylated
cg13155549	0.63794	0.511655135	-0.318249	0.000148	hypomethylated
cg27437413	0.92089	0.738544865	-0.318343	4.73E-06	hypomethylated
cg22123459	0.54428	0.43648	-0.318433	0.00039	hypomethylated
cg21773820	0.84051	0.674011351	-0.318492	0.000106	hypomethylated
cg13970540	0.80451	0.645136216	-0.318507	3.07E-05	hypomethylated
cg23696864	0.91113	0.73063027	-0.318515	2.49E-06	hypomethylated
cg03003746	0.92001	0.737730811	-0.318555	1.93E-06	hypomethylated
cg00334507	0.79698	0.639073514	-0.318562	0.000331	hypomethylated
cg23059701	0.87479	0.70144973	-0.318597	1.12E-05	hypomethylated
cg00232105	0.6554	0.525500541	-0.318683	0.000122	hypomethylated
cg04896048	0.92147	0.738817297	-0.31872	0.000126	hypomethylated
cg13525397	0.66119	0.530123243	-0.318737	0.000421	hypomethylated
cg09025071	0.75696	0.606888649	-0.318785	0.000517	hypomethylated
cg00619263	0.77453	0.620972973	-0.318791	0.000517	hypomethylated
cg18645316	0.82384	0.660432973	-0.318952	6.31E-05	hypomethylated
cg17165158	0.94523	0.75771027	-0.319019	5.72E-05	hypomethylated
cg22007638	0.70885	0.568208108	-0.319061	1.14E-05	hypomethylated
cg16901862	0.80855	0.64810973	-0.319099	0.000517	hypomethylated
cg10599446	0.85848	0.688125946	-0.319112	2.10E-05	hypomethylated
cg02800711	0.92438	0.740876216	-0.319254	2.99E-06	hypomethylated
cg03318428	0.88592	0.710048649	-0.319259	8.73E-05	hypomethylated
cg24634577	0.82533	0.661473514	-0.319288	2.64E-05	hypomethylated
cg18009575	0.78119	0.626093514	-0.319295	0.000204	hypomethylated
cg27124512	0.71019	0.569189189	-0.319297	0.000274	hypomethylated
cg14699839	0.79671	0.638523784	-0.319314	3.19E-05	hypomethylated
cg23726483	0.77576	0.62171027	-0.319368	7.47E-05	hypomethylated
cg27583604	0.77306	0.619520541	-0.319428	0.000229	hypomethylated

cg18909903	0.7353	0.58924973	-0.319454	1.77E-05	hypomethylated
cg19382019	0.84646	0.67832973	-0.319455	4.36E-05	hypomethylated
cg15218995	0.78659	0.630342703	-0.319475	8.13E-05	hypomethylated
cg22170599	0.89182	0.714651351	-0.319513	5.00E-05	hypomethylated
cg08265495	0.77745	0.622971892	-0.319583	0.0002	hypomethylated
cg13124080	0.84667	0.678431351	-0.319597	1.71E-05	hypomethylated
cg18594482	0.89297	0.715489189	-0.319682	0.000117	hypomethylated
cg04774822	0.79809	0.639458378	-0.319701	0.000135	hypomethylated
cg08628580	0.88507	0.709121081	-0.31976	9.05E-05	hypomethylated
cg19100772	0.73933	0.592352432	-0.319763	0.000142	hypomethylated
cg18402166	0.71169	0.570132973	-0.319951	0.000145	hypomethylated
cg21860285	0.73623	0.589779459	-0.319981	5.25E-05	hypomethylated
cg02735733	0.84583	0.677532432	-0.320078	9.72E-05	hypomethylated
cg26227592	0.62396	0.499807568	-0.320081	2.48E-05	hypomethylated
cg10994870	0.63253	0.506665405	-0.320101	0.000369	hypomethylated
cg08051604	0.80974	0.64860973	-0.320108	0.0006	hypomethylated
cg24707640	0.84445	0.676331351	-0.320282	1.14E-05	hypomethylated
cg15554087	0.87961	0.704465405	-0.320335	9.66E-07	hypomethylated
cg25372195	0.80264	0.642819459	-0.320339	8.84E-05	hypomethylated
cg22329555	0.59124	0.473497838	-0.320386	2.92E-05	hypomethylated
cg17181043	0.88078	0.705268108	-0.32061	6.01E-05	hypomethylated
cg13201644	0.82244	0.658483784	-0.320763	0.000262	hypomethylated
cg14759277	0.94417	0.7559	-0.320851	0.000202	hypomethylated
cg07824081	0.60503	0.484380541	-0.320866	0.000112	hypomethylated
cg00390484	0.879	0.703688108	-0.320927	0.000251	hypomethylated
cg23908385	0.78	0.624417297	-0.320964	9.49E-05	hypomethylated
cg13680200	0.79719	0.638148108	-0.321032	0.000417	hypomethylated
cg22112110	0.86391	0.691512432	-0.321126	5.72E-05	hypomethylated
cg22380322	0.73137	0.585416216	-0.321139	0.000772	hypomethylated
cg16313944	0.66413	0.53158973	-0.321152	2.10E-05	hypomethylated
cg14737704	0.66726	0.534092432	-0.32116	0.00054	hypomethylated
cg13152690	0.76764	0.614389189	-0.321277	0.00291	hypomethylated
cg17890298	0.70459	0.563917297	-0.3213	0.000104	hypomethylated
cg05591210	0.8621	0.689938919	-0.321387	2.89E-05	hypomethylated
cg21308656	0.90519	0.724417838	-0.321399	2.57E-05	hypomethylated
cg18898209	0.82484	0.660014054	-0.321618	3.44E-05	hypomethylated
cg09950076	0.91567	0.732684324	-0.321636	1.40E-05	hypomethylated
cg15006681	0.69957	0.559711351	-0.321785	4.26E-05	hypomethylated
cg03702686	0.75999	0.608017297	-0.321868	0.000152	hypomethylated
cg13727473	0.74258	0.59406	-0.321938	0.000145	hypomethylated
cg10773266	0.76015	0.60811027	-0.321951	0.01146	hypomethylated
cg17031787	0.74424	0.59534973	-0.322031	0.000296	hypomethylated
cg16118839	0.77659	0.621197297	-0.322102	0.000893	hypomethylated
cg00912580	0.68513	0.548028108	-0.322128	0.001301	hypomethylated
cg10549071	0.82324	0.658496757	-0.322137	8.63E-05	hypomethylated
cg14520913	0.81481	0.651749189	-0.322147	0.000174	hypomethylated
cg25345746	0.85468	0.68359027	-0.322253	2.67E-05	hypomethylated
cg04602081	0.89205	0.713475135	-0.322261	2.67E-05	hypomethylated
cg20951054	0.7867	0.629156757	-0.322394	2.51E-05	hypomethylated
cg05295841	0.89814	0.718197297	-0.32256	9.27E-05	hypomethylated
cg13598881	0.79647	0.636883243	-0.322591	8.84E-05	hypomethylated
cg16239278	0.89537	0.715913514	-0.322699	5.45E-05	hypomethylated
cg12425700	0.81177	0.649050811	-0.32274	7.83E-06	hypomethylated
cg20212364	0.88432	0.707014595	-0.322829	2.38E-05	hypomethylated
cg15374518	0.7831	0.626076757	-0.322857	0.000506	hypomethylated
cg13487334	0.76378	0.61062973	-0.322859	2.64E-05	hypomethylated
cg23091122	0.7812	0.624527568	-0.322927	6.08E-05	hypomethylated
cg15450966	0.52506	0.419756757	-0.322929	0.0002	hypomethylated

cg03126633	0.66904	0.534853514	-0.322949	0.000155	hypomethylated
cg22075924	0.66246	0.529591892	-0.322952	0.000435	hypomethylated
cg15209376	0.83669	0.668825405	-0.323064	3.85E-05	hypomethylated
cg09924848	0.75628	0.604534595	-0.323096	0.000346	hypomethylated
cg24160371	0.85816	0.685959459	-0.323123	5.25E-05	hypomethylated
cg14975547	0.84072	0.672017838	-0.323126	7.84E-05	hypomethylated
cg04848426	0.88951	0.711007568	-0.323146	2.45E-05	hypomethylated
cg14779271	0.81178	0.648833514	-0.32324	5.38E-05	hypomethylated
cg23001905	0.87774	0.701543784	-0.323261	1.23E-05	hypomethylated
cg19488206	0.59693	0.477101081	-0.323267	7.47E-05	hypomethylated
cg14350257	0.78628	0.628410811	-0.323335	3.81E-05	hypomethylated
cg26070865	0.59967	0.479259459	-0.323362	0.000893	hypomethylated
cg19865472	0.92035	0.735545946	-0.323367	0.000147	hypomethylated
cg08917664	0.91465	0.730953514	-0.32344	3.26E-06	hypomethylated
cg09910207	0.8642	0.6906	-0.323515	1.28E-05	hypomethylated
cg12409874	0.89911	0.718481081	-0.323547	3.44E-05	hypomethylated
cg12038298	0.70994	0.567272432	-0.323655	0.000142	hypomethylated
cg17373759	0.85657	0.684414054	-0.323702	8.63E-05	hypomethylated
cg23205936	0.82607	0.660031892	-0.323728	0.00032	hypomethylated
cg09773473	0.90927	0.726485405	-0.323775	3.49E-06	hypomethylated
cg05580415	0.76232	0.609067027	-0.323796	4.47E-05	hypomethylated
cg22643193	0.8405	0.671522703	-0.323812	1.58E-05	hypomethylated
cg26252045	0.86528	0.691291892	-0.323872	3.00E-05	hypomethylated
cg09662304	0.80326	0.641731351	-0.323898	4.47E-05	hypomethylated
cg19038027	0.5978	0.477582703	-0.323912	0.000195	hypomethylated
cg20333727	0.7433	0.593769189	-0.324042	1.97E-05	hypomethylated
cg05233946	0.80759	0.645122162	-0.324051	0.00028	hypomethylated
cg23670188	0.77463	0.618782703	-0.324075	0.000155	hypomethylated
cg04756594	0.77267	0.61719027	-0.324137	0.000594	hypomethylated
cg22283058	0.77914	0.622295676	-0.324282	0.000142	hypomethylated
cg08186915	0.68355	0.545936216	-0.324314	7.29E-05	hypomethylated
cg24699699	0.87983	0.70258	-0.324562	1.66E-05	hypomethylated
cg08439439	0.87694	0.700243784	-0.324621	8.52E-05	hypomethylated
cg22330638	0.71358	0.569785946	-0.324655	0.000117	hypomethylated
cg19311448	0.69545	0.555302703	-0.324672	0.000551	hypomethylated
cg26123824	0.68721	0.548715676	-0.324692	0.00018	hypomethylated
cg14370520	0.77528	0.619025946	-0.324718	0.00099	hypomethylated
cg05814135	0.83216	0.66443027	-0.324743	6.01E-05	hypomethylated
cg03259292	0.69221	0.552684324	-0.324754	0.000293	hypomethylated
cg01902704	0.7513	0.599801622	-0.324904	0.000358	hypomethylated
cg17936783	0.70672	0.564204324	-0.324921	0.000224	hypomethylated
cg08809408	0.80534	0.642905405	-0.324992	4.15E-05	hypomethylated
cg09715545	0.73192	0.584282703	-0.325019	0.00066	hypomethylated
cg23484912	0.88343	0.70522	-0.325042	2.51E-05	hypomethylated
cg14548777	0.80629	0.643616757	-0.325097	2.45E-05	hypomethylated
cg02463513	0.88239	0.704361081	-0.325101	0.000107	hypomethylated
cg08915438	0.6843	0.546232973	-0.325113	0.000274	hypomethylated
cg09638264	0.8907	0.710971351	-0.325148	4.00E-05	hypomethylated
cg16902863	0.73844	0.589392973	-0.325251	0.00028	hypomethylated
cg22532155	0.88315	0.704863243	-0.325315	1.04E-05	hypomethylated
cg11173131	0.67872	0.541672973	-0.325394	0.001587	hypomethylated
cg05492657	0.85099	0.679147568	-0.325417	1.25E-05	hypomethylated
cg08344174	0.89078	0.710898378	-0.325426	2.78E-05	hypomethylated
cg01097897	0.82061	0.654896757	-0.325429	0.000342	hypomethylated
cg27345989	0.80608	0.643262703	-0.325515	3.95E-05	hypomethylated
cg05998333	0.85614	0.683198378	-0.325542	1.31E-05	hypomethylated
cg24737570	0.73808	0.588974054	-0.325573	0.000152	hypomethylated
cg25880347	0.72645	0.579651892	-0.325677	3.00E-05	hypomethylated

cg08832596	0.51267	0.409061622	-0.325712	0.000108	hypomethylated
cg12669088	0.86322	0.688759459	-0.325728	0.000155	hypomethylated
cg24137216	0.8928	0.712352973	-0.325745	0.000148	hypomethylated
cg03519180	0.82062	0.654684865	-0.325914	0.000242	hypomethylated
cg08251025	0.86473	0.689863243	-0.325939	8.60E-06	hypomethylated
cg06960356	0.9116	0.727209189	-0.32603	0.000163	hypomethylated
cg10334489	0.63902	0.50969027	-0.32624	0.000335	hypomethylated
cg12420383	0.47353	0.377688649	-0.326258	2.27E-05	hypomethylated
cg04544908	0.76359	0.609016216	-0.326318	0.000147	hypomethylated
cg07897069	0.74569	0.594705405	-0.326401	0.00039	hypomethylated
cg03453890	0.71659	0.571458919	-0.326498	2.96E-05	hypomethylated
cg18151262	0.7673	0.611843243	-0.326629	8.22E-05	hypomethylated
cg16584392	0.69943	0.557720541	-0.326637	5.79E-05	hypomethylated
cg08463231	0.68827	0.548804865	-0.326681	0.000262	hypomethylated
cg14575950	0.84204	0.671356216	-0.32681	0.000102	hypomethylated
cg02340818	0.86969	0.693383784	-0.326847	0.000202	hypomethylated
cg17333291	0.62863	0.501189189	-0.326856	4.26E-05	hypomethylated
cg05341549	0.63925	0.509625946	-0.326942	0.000202	hypomethylated
cg10244770	0.88527	0.705748108	-0.326964	1.06E-05	hypomethylated
cg08571304	0.89064	0.710002703	-0.327018	4.80E-06	hypomethylated
cg10009380	0.70411	0.561275676	-0.327091	0.00071	hypomethylated
cg18810691	0.69184	0.551480541	-0.327128	0.001803	hypomethylated
cg26568171	0.87215	0.695191892	-0.327165	4.42E-06	hypomethylated
cg22248286	0.7816	0.623005946	-0.327185	5.38E-05	hypomethylated
cg02618550	0.5638	0.449358378	-0.327317	0.000113	hypomethylated
cg01018864	0.81065	0.646095135	-0.327333	4.58E-05	hypomethylated
cg15423315	0.70648	0.563054595	-0.327374	0.001354	hypomethylated
cg02990598	0.64721	0.515814595	-0.327381	4.36E-05	hypomethylated
cg26515162	0.94716	0.754858919	-0.327401	6.46E-05	hypomethylated
cg20125501	0.7945	0.633190811	-0.327407	3.27E-05	hypomethylated
cg15339688	0.84186	0.670928649	-0.327421	2.81E-05	hypomethylated
cg00255699	0.82211	0.655185946	-0.327427	0.000667	hypomethylated
cg27127272	0.79095	0.630310811	-0.327523	0.000148	hypomethylated
cg21426065	0.97949	0.780543784	-0.327551	1.33E-06	hypomethylated
cg17818731	0.87036	0.693574595	-0.327561	1.42E-05	hypomethylated
cg01920398	0.70006	0.557843784	-0.327617	2.92E-05	hypomethylated
cg21439481	0.7979	0.635793514	-0.32765	8.22E-05	hypomethylated
cg12104698	0.6478	0.516185405	-0.327659	0.001603	hypomethylated
cg10175203	0.94338	0.751677297	-0.327726	2.95E-06	hypomethylated
cg06069457	0.97355	0.775671892	-0.327809	4.93E-06	hypomethylated
cg09671309	0.87098	0.693935135	-0.327839	7.12E-05	hypomethylated
cg06832359	0.62589	0.498654054	-0.32787	5.86E-05	hypomethylated
cg19087028	0.87905	0.700342703	-0.327884	6.01E-05	hypomethylated
cg08327038	0.78213	0.623056216	-0.328046	0.000857	hypomethylated
cg15890469	0.68551	0.546032973	-0.32819	0.001481	hypomethylated
cg11231434	0.966	0.769389189	-0.32831	1.23E-05	hypomethylated
cg08610987	0.72794	0.579687568	-0.328544	0.000575	hypomethylated
cg01487468	0.68942	0.548892973	-0.328858	0.000204	hypomethylated
cg27303933	0.72113	0.574124865	-0.328895	0.000112	hypomethylated
cg17949316	0.94557	0.752811351	-0.328896	9.57E-06	hypomethylated
cg13705810	0.73847	0.587927568	-0.328901	8.84E-05	hypomethylated
cg18537482	0.72083	0.573880541	-0.328909	0.000159	hypomethylated
cg21391373	0.87639	0.697725946	-0.328913	4.86E-06	hypomethylated
cg24069602	0.66178	0.526864865	-0.328919	0.000221	hypomethylated
cg18448426	0.85078	0.677286486	-0.32902	8.94E-05	hypomethylated
cg10914667	0.84797	0.675034595	-0.329052	2.10E-05	hypomethylated
cg01025720	0.82865	0.659653514	-0.329054	0.000382	hypomethylated
cg22491440	0.69276	0.551468108	-0.329078	8.84E-05	hypomethylated

cg01497527	0.80517	0.640931351	-0.329124	5.19E-05	hypomethylated
cg03660606	0.58865	0.46855027	-0.329206	7.32E-06	hypomethylated
cg03825810	0.73395	0.584202703	-0.329213	9.60E-05	hypomethylated
cg20701457	0.69882	0.556174054	-0.329384	0.003441	hypomethylated
cg17449759	0.92138	0.733247568	-0.329496	6.75E-06	hypomethylated
cg09167117	0.69105	0.549941081	-0.329513	0.000234	hypomethylated
cg13562353	0.80572	0.641182703	-0.329543	6.05E-06	hypomethylated
cg19820705	0.53382	0.424796216	-0.329582	0.000145	hypomethylated
cg27056501	0.89807	0.714647027	-0.329597	7.72E-06	hypomethylated
cg07600499	0.77435	0.616184324	-0.329624	0.000262	hypomethylated
cg10287113	0.54298	0.432072432	-0.329626	0.000104	hypomethylated
cg24242280	0.74792	0.595136216	-0.329664	7.72E-06	hypomethylated
cg16983541	0.51916	0.413105405	-0.329669	7.47E-05	hypomethylated
cg18183240	0.86316	0.686815135	-0.329706	3.44E-05	hypomethylated
cg27127887	0.66381	0.528167027	-0.329776	0.000464	hypomethylated
cg26786974	0.73988	0.588664865	-0.329845	0.000902	hypomethylated
cg04914198	0.7525	0.598658378	-0.329959	3.57E-05	hypomethylated
cg01647560	0.85307	0.678658378	-0.329979	3.95E-05	hypomethylated
cg24453438	0.81467	0.648095135	-0.33001	1.21E-05	hypomethylated
cg14793611	0.7544	0.600136757	-0.330038	0.000138	hypomethylated
cg04269747	0.55691	0.44302973	-0.330041	2.64E-05	hypomethylated
cg12681948	0.79359	0.631205946	-0.330283	0.000459	hypomethylated
cg08119325	0.62831	0.499741622	-0.330294	0.000259	hypomethylated
cg21566086	0.61754	0.491157838	-0.330346	0.000449	hypomethylated
cg23919924	0.79196	0.629862703	-0.33039	7.38E-05	hypomethylated
cg03322234	0.71455	0.568284324	-0.330422	0.00014	hypomethylated
cg14023573	0.86393	0.687083243	-0.33043	4.36E-05	hypomethylated
cg10479629	0.70342	0.559416757	-0.330463	0.00017	hypomethylated
cg20289854	0.83065	0.660588649	-0.330489	3.85E-05	hypomethylated
cg05136956	0.76226	0.606175676	-0.330547	1.68E-05	hypomethylated
cg11479568	0.69782	0.554911351	-0.330598	0.000115	hypomethylated
cg02266878	0.76649	0.609500541	-0.330639	4.70E-05	hypomethylated
cg13282068	0.93786	0.745731351	-0.330717	4.01E-06	hypomethylated
cg00300969	0.67133	0.533795135	-0.330736	0.001368	hypomethylated
cg20597143	0.70136	0.557662162	-0.330764	9.07E-06	hypomethylated
cg23594208	0.83488	0.663816757	-0.330784	8.63E-05	hypomethylated
cg26140483	0.85852	0.682597838	-0.330816	7.47E-05	hypomethylated
cg18927901	0.71097	0.565278919	-0.330826	5.06E-05	hypomethylated
cg06557580	0.84663	0.673118919	-0.33087	0.000115	hypomethylated
cg14689365	0.64461	0.51248	-0.330931	5.65E-05	hypomethylated
cg01727105	0.82212	0.653603243	-0.330934	1.35E-05	hypomethylated
cg08137852	0.7524	0.598152973	-0.330985	3.07E-05	hypomethylated
cg27003309	0.92992	0.739243784	-0.331056	6.15E-05	hypomethylated
cg03028769	0.72596	0.577054054	-0.331184	0.000251	hypomethylated
cg26013992	0.80972	0.643598919	-0.331261	0.000399	hypomethylated
cg01963059	0.85571	0.680128108	-0.331315	4.82E-05	hypomethylated
cg15117681	0.73892	0.58727027	-0.331394	0.00012	hypomethylated
cg08252585	0.91082	0.723883243	-0.331409	6.93E-06	hypomethylated
cg08617020	0.7948	0.631650811	-0.331465	1.31E-05	hypomethylated
cg25852472	0.61794	0.491094595	-0.331466	0.000174	hypomethylated
cg23935642	0.78173	0.621232973	-0.331536	0.000313	hypomethylated
cg12108798	0.52243	0.415167568	-0.331544	4.94E-05	hypomethylated
cg12965095	0.95275	0.757135676	-0.331546	5.43E-06	hypomethylated
cg17740140	0.76612	0.608817297	-0.331561	0.000306	hypomethylated
cg18464364	0.82693	0.657132973	-0.33158	0.000268	hypomethylated
cg25912009	0.76003	0.60396973	-0.33158	0.000102	hypomethylated
cg06466796	0.64297	0.510932973	-0.331617	0.00029	hypomethylated
cg09256716	0.93275	0.741193514	-0.33164	2.04E-06	hypomethylated

cg08099570	0.6344	0.504111892	-0.331649	7.47E-05	hypomethylated
cg07217063	0.64192	0.510083243	-0.331661	2.92E-05	hypomethylated
cg20983876	0.82338	0.654257838	-0.331699	0.000219	hypomethylated
cg21225563	0.68701	0.545896216	-0.331704	0.000313	hypomethylated
cg27425383	0.7003	0.556408108	-0.33183	5.00E-05	hypomethylated
cg25598376	0.84029	0.667590811	-0.331923	3.76E-05	hypomethylated
cg16769381	0.88202	0.700723243	-0.331967	1.99E-06	hypomethylated
cg17097293	0.76229	0.605595135	-0.331986	8.83E-06	hypomethylated
cg09843272	0.7651	0.607814595	-0.332017	1.25E-05	hypomethylated
cg00739471	0.74703	0.593407027	-0.332144	0.001987	hypomethylated
cg11797364	0.96535	0.766827027	-0.332151	6.57E-06	hypomethylated
cg27662789	0.70106	0.556880541	-0.33217	0.002502	hypomethylated
cg12424383	0.77201	0.613235676	-0.332178	1.89E-05	hypomethylated
cg15089806	0.72374	0.574886486	-0.332194	0.000435	hypomethylated
cg08454546	0.91856	0.729606486	-0.332255	1.18E-06	hypomethylated
cg22169874	0.7593	0.603104324	-0.332262	2.15E-05	hypomethylated
cg05796178	0.78526	0.623691892	-0.332337	0.000166	hypomethylated
cg25182621	0.67047	0.532515676	-0.332349	9.49E-05	hypomethylated
cg26274929	0.58636	0.465706486	-0.332366	0.000106	hypomethylated
cg02522367	0.86208	0.684648108	-0.332459	0.000142	hypomethylated
cg14585892	0.78776	0.625601081	-0.332513	3.49E-06	hypomethylated
cg20118577	0.80438	0.638798919	-0.332515	8.83E-06	hypomethylated
cg26343761	0.83721	0.664870811	-0.332516	1.15E-05	hypomethylated
cg18982477	0.92555	0.735011892	-0.332543	4.36E-06	hypomethylated
cg25658010	0.77962	0.619115135	-0.332563	1.04E-05	hypomethylated
cg11708690	0.78351	0.622197297	-0.33258	3.76E-05	hypomethylated
cg18018913	0.95371	0.757308649	-0.332669	1.96E-06	hypomethylated
cg14578311	0.87807	0.69720973	-0.332743	1.97E-05	hypomethylated
cg07781701	0.86688	0.688297838	-0.332799	5.06E-05	hypomethylated
cg05653887	0.77394	0.614459459	-0.332904	4.47E-05	hypomethylated
cg15608892	0.6852	0.543987027	-0.332953	2.96E-05	hypomethylated
cg14950134	0.66347	0.526725405	-0.33298	9.27E-05	hypomethylated
cg23743114	0.88803	0.704996757	-0.332992	4.05E-05	hypomethylated
cg01529404	0.75957	0.603004865	-0.333013	0.000454	hypomethylated
cg20537521	0.85739	0.680657297	-0.333023	2.10E-05	hypomethylated
cg14555350	0.66298	0.52632	-0.333025	0.00017	hypomethylated
cg22442617	0.81704	0.648548108	-0.333193	0.000161	hypomethylated
cg13026772	0.84067	0.667254595	-0.333302	1.71E-05	hypomethylated
cg08582840	0.73892	0.58647027	-0.33336	5.45E-05	hypomethylated
cg11218561	0.83118	0.659687568	-0.333378	8.22E-05	hypomethylated
cg14947846	0.77209	0.61274973	-0.333471	0.000155	hypomethylated
cg20445197	0.68691	0.545142162	-0.333489	0.000142	hypomethylated
cg25697881	0.73062	0.57983027	-0.333491	8.84E-05	hypomethylated
cg19698340	0.87025	0.690627027	-0.333523	2.45E-05	hypomethylated
cg07461273	0.58369	0.463211892	-0.33353	6.15E-05	hypomethylated
cg06292312	0.80566	0.639337838	-0.333593	1.20E-05	hypomethylated
cg11566152	0.8211	0.651571892	-0.333634	0.000109	hypomethylated
cg12145564	0.73998	0.587152432	-0.333751	0.000191	hypomethylated
cg05122040	0.86436	0.685835676	-0.333769	4.36E-05	hypomethylated
cg08382072	0.87472	0.694055676	-0.33377	1.17E-05	hypomethylated
cg21637761	0.70638	0.560452973	-0.333851	0.001164	hypomethylated
cg05261349	0.6841	0.542726486	-0.333982	3.95E-05	hypomethylated
cg18564167	0.88723	0.703857838	-0.334024	0.000107	hypomethylated
cg18644782	0.79901	0.63386	-0.334049	3.85E-05	hypomethylated
cg11160572	0.89303	0.708426486	-0.33409	8.03E-05	hypomethylated
cg22845912	0.76686	0.608316216	-0.334142	0.000245	hypomethylated
cg08479629	0.73346	0.581781081	-0.334242	1.77E-05	hypomethylated
cg22862003	0.612	0.485437297	-0.334247	0.001511	hypomethylated

cg00550340	0.68999	0.547283243	-0.334288	0.000317	hypomethylated
cg08018143	0.69825	0.55382	-0.334327	0.000454	hypomethylated
cg22107147	0.80614	0.639363784	-0.334393	8.22E-05	hypomethylated
cg07479843	0.83745	0.664096216	-0.334611	8.83E-06	hypomethylated
cg25556051	0.81073	0.642897297	-0.334633	5.14E-06	hypomethylated
cg15456144	0.86714	0.687614054	-0.334666	0.000234	hypomethylated
cg08273063	0.84418	0.669364324	-0.334759	0.000135	hypomethylated
cg17182270	0.88257	0.699782703	-0.334804	0.000112	hypomethylated
cg18234296	0.58443	0.463384865	-0.334819	0.000178	hypomethylated
cg23774016	0.69375	0.550060541	-0.334825	0.000148	hypomethylated
cg17920653	0.78024	0.618614595	-0.334877	1.44E-05	hypomethylated
cg12592321	0.60901	0.482797297	-0.335048	0.000204	hypomethylated
cg03903296	0.79542	0.630537297	-0.335135	5.45E-05	hypomethylated
cg05120428	0.60842	0.482287027	-0.335176	0.000195	hypomethylated
cg04133293	0.9153	0.725479459	-0.33531	7.72E-06	hypomethylated
cg11758345	0.83327	0.66046	-0.335313	3.04E-06	hypomethylated
cg14009561	0.78711	0.623865946	-0.335329	0.000667	hypomethylated
cg14696926	0.80648	0.639216757	-0.335334	0.000313	hypomethylated
cg17841440	0.65084	0.515837297	-0.335387	6.38E-05	hypomethylated
cg03220734	0.66256	0.525124324	-0.335392	7.56E-05	hypomethylated
cg19225602	0.71455	0.566299459	-0.33547	0.000435	hypomethylated
cg09360044	0.72595	0.575310811	-0.335529	0.00097	hypomethylated
cg04510209	0.64836	0.513812973	-0.335552	5.06E-05	hypomethylated
cg00420347	0.827	0.65538	-0.335556	1.25E-05	hypomethylated
cg26912314	0.80254	0.635975676	-0.335602	7.38E-05	hypomethylated
cg18864926	0.90646	0.718318378	-0.33562	3.85E-05	hypomethylated
cg13389652	0.7198	0.570394054	-0.335637	0.000163	hypomethylated
cg23219253	0.78791	0.62432973	-0.335723	8.52E-05	hypomethylated
cg13434764	0.75685	0.59970973	-0.335743	0.00017	hypomethylated
cg04742977	0.77647	0.615221081	-0.335825	0.000145	hypomethylated
cg09976157	0.87378	0.692317297	-0.335837	9.82E-06	hypomethylated
cg10129938	0.80872	0.640759459	-0.335857	0.000104	hypomethylated
cg18201505	0.83725	0.663349189	-0.33589	9.44E-06	hypomethylated
cg18399427	0.64749	0.513003243	-0.33589	3.67E-05	hypomethylated
cg11902180	0.76733	0.607917838	-0.335971	8.03E-05	hypomethylated
cg12410939	0.92946	0.736340541	-0.33602	4.36E-06	hypomethylated
cg01561861	0.94997	0.75256973	-0.336057	8.60E-06	hypomethylated
cg18446110	0.69311	0.549076757	-0.336076	0.0003	hypomethylated
cg27475923	0.7688	0.609033514	-0.336087	0.000107	hypomethylated
cg11629955	0.86514	0.685326486	-0.336142	3.44E-05	hypomethylated
cg11198851	0.87083	0.689827568	-0.336155	5.73E-06	hypomethylated
cg08123067	0.8594	0.680758378	-0.336187	4.86E-06	hypomethylated
cg19513727	0.71744	0.568272973	-0.336274	0.00039	hypomethylated
cg16559570	0.81956	0.649127027	-0.336349	3.53E-05	hypomethylated
cg17471836	0.85601	0.677990811	-0.336362	0.00035	hypomethylated
cg06099703	0.75711	0.59965027	-0.336382	0.000152	hypomethylated
cg06463142	0.61046	0.483480541	-0.336439	1.31E-05	hypomethylated
cg05990312	0.68001	0.538558919	-0.336452	0.001875	hypomethylated
cg20811788	0.53738	0.425576757	-0.336523	0.000145	hypomethylated
cg03955764	0.78813	0.624128108	-0.336591	0.000145	hypomethylated
cg04355801	0.79708	0.631203243	-0.33662	3.95E-05	hypomethylated
cg19995049	0.89416	0.708055676	-0.33667	5.45E-05	hypomethylated
cg16022876	0.9353	0.740631892	-0.336673	6.39E-06	hypomethylated
cg05306029	0.80022	0.63364973	-0.336711	0.000613	hypomethylated
cg21617903	0.79659	0.630767027	-0.33673	2.81E-05	hypomethylated
cg00112341	0.66764	0.52866	-0.33673	0.001367	hypomethylated
cg08745334	0.66987	0.530425405	-0.336731	0.00039	hypomethylated
cg06434451	0.73692	0.583512432	-0.336745	0.000374	hypomethylated

cg03555157	0.63615	0.503716757	-0.336754	0.001368	hypomethylated
cg13800802	0.86787	0.687194595	-0.33676	0.000107	hypomethylated
cg22172610	0.75547	0.598183243	-0.336787	0.000274	hypomethylated
cg19149463	0.76154	0.602952973	-0.336874	1.12E-05	hypomethylated
cg00848245	0.8272	0.654934054	-0.336887	0.000155	hypomethylated
cg18048071	0.75951	0.601318919	-0.336939	0.001141	hypomethylated
cg23789604	0.81977	0.648984865	-0.337034	0.000287	hypomethylated
cg15122358	0.89146	0.705733514	-0.337047	0.000232	hypomethylated
cg20543645	0.75685	0.599114054	-0.337177	0.000229	hypomethylated
cg04425624	0.5677	0.449377838	-0.3372	0.001237	hypomethylated
cg07471203	0.70823	0.560598378	-0.33725	0.000262	hypomethylated
cg23989110	0.80481	0.637034595	-0.337277	8.42E-05	hypomethylated
cg00071887	0.77508	0.613480541	-0.337328	3.36E-05	hypomethylated
cg24631065	0.85853	0.679513514	-0.337366	9.31E-06	hypomethylated
cg05634376	0.86887	0.687693514	-0.337375	3.19E-05	hypomethylated
cg11422156	0.63184	0.500076216	-0.337411	0.000408	hypomethylated
cg12020444	0.87318	0.691085946	-0.337414	3.15E-05	hypomethylated
cg08185255	0.68689	0.543635676	-0.337439	4.70E-05	hypomethylated
cg06813260	0.73395	0.58083027	-0.337565	0.000102	hypomethylated
cg21150839	0.91114	0.721019459	-0.337635	3.39E-06	hypomethylated
cg15864691	0.83407	0.660025405	-0.337647	0.000182	hypomethylated
cg00294267	0.63263	0.500612432	-0.337668	2.45E-05	hypomethylated
cg22509816	0.87619	0.693336757	-0.337688	1.71E-05	hypomethylated
cg06781213	0.68301	0.540468649	-0.337696	0.000166	hypomethylated
cg08011335	0.72217	0.571443784	-0.337727	3.62E-05	hypomethylated
cg11313780	0.79038	0.625385405	-0.337801	0.000219	hypomethylated
cg01274753	0.92802	0.734285405	-0.337815	0.000274	hypomethylated
cg22209573	0.81775	0.647003243	-0.337887	6.62E-05	hypomethylated
cg19954234	0.88059	0.696677838	-0.337979	1.62E-05	hypomethylated
cg26767897	0.92523	0.731936757	-0.338093	6.39E-06	hypomethylated
cg16511831	0.76375	0.604175676	-0.338132	6.15E-05	hypomethylated
cg14177084	0.65556	0.518587568	-0.33814	0.000132	hypomethylated
cg24524485	0.81175	0.642112432	-0.33821	1.92E-05	hypomethylated
cg06018514	0.77569	0.613568649	-0.338255	2.92E-05	hypomethylated
cg10787197	0.7215	0.570698919	-0.33827	0.002699	hypomethylated
cg01129238	0.79514	0.628935135	-0.338298	0.000262	hypomethylated
cg23535449	0.89549	0.708214595	-0.338491	7.93E-06	hypomethylated
cg04503319	0.68907	0.544960541	-0.338499	0.000342	hypomethylated
cg13535903	0.66269	0.524070811	-0.338572	0.00044	hypomethylated
cg19495444	0.7159	0.566142703	-0.338592	0.000214	hypomethylated
cg09895653	0.64	0.506115135	-0.338606	0.000111	hypomethylated
cg16744911	0.86114	0.680976216	-0.338643	3.40E-05	hypomethylated
cg16265859	0.79657	0.629914054	-0.338646	0.000374	hypomethylated
cg05363574	0.91981	0.727335135	-0.338716	6.48E-06	hypomethylated
cg15914863	0.66105	0.522644865	-0.338928	0.00054	hypomethylated
cg22435313	0.83419	0.659534054	-0.338929	1.39E-05	hypomethylated
cg07131540	0.80373	0.635439459	-0.338956	2.15E-05	hypomethylated
cg23160829	0.90404	0.714743784	-0.33896	6.22E-06	hypomethylated
cg14525390	0.88162	0.696993514	-0.339012	2.78E-05	hypomethylated
cg09886558	0.62484	0.493961081	-0.339089	0.000202	hypomethylated
cg14360139	0.74974	0.592674054	-0.339151	2.64E-05	hypomethylated
cg13588355	0.79702	0.630001081	-0.339262	4.05E-05	hypomethylated
cg14428628	0.83178	0.657462703	-0.339293	4.15E-05	hypomethylated
cg06865913	0.81033	0.640492432	-0.339328	1.62E-05	hypomethylated
cg17141902	0.57533	0.454736757	-0.339358	0.000342	hypomethylated
cg01594949	0.56649	0.447734595	-0.339407	0.000386	hypomethylated
cg26433582	0.72731	0.574832432	-0.339429	0.004059	hypomethylated
cg02652369	0.67547	0.533825946	-0.339522	0.000268	hypomethylated

cg26477856	0.692	0.546871892	-0.339569	0.00098	hypomethylated
cg24688837	0.81462	0.643774595	-0.339572	7.93E-06	hypomethylated
cg04492496	0.66799	0.527864324	-0.339659	6.86E-05	hypomethylated
cg19091930	0.82898	0.655074595	-0.339678	0.000646	hypomethylated
cg14360405	0.76439	0.604017297	-0.339719	4.47E-05	hypomethylated
cg26244616	0.7618	0.60196973	-0.339721	9.05E-05	hypomethylated
cg16293118	0.74512	0.588787027	-0.339727	2.57E-05	hypomethylated
cg19580930	0.88827	0.701897838	-0.339737	9.72E-05	hypomethylated
cg22601415	0.84742	0.669603243	-0.339771	0.000256	hypomethylated
cg09363128	0.62144	0.491009189	-0.339865	2.32E-05	hypomethylated
cg20002381	0.82791	0.654129189	-0.339898	1.35E-05	hypomethylated
cg21494132	0.88442	0.698775135	-0.339903	4.47E-05	hypomethylated
cg04057599	0.85319	0.674086486	-0.339933	6.78E-05	hypomethylated
cg22894276	0.74789	0.590867027	-0.339993	1.87E-05	hypomethylated
cg13261938	0.73806	0.583029189	-0.34017	0.000204	hypomethylated
cg17128256	0.78903	0.623287568	-0.340182	3.79E-06	hypomethylated
cg05892419	0.69037	0.54534973	-0.340188	0.000155	hypomethylated
cg09983546	0.77943	0.615701622	-0.340188	6.78E-05	hypomethylated
cg03071876	0.71248	0.562781081	-0.340276	0.000435	hypomethylated
cg00921973	0.80754	0.63770973	-0.340634	9.72E-05	hypomethylated
cg08556541	0.81014	0.639754595	-0.340653	5.25E-05	hypomethylated
cg18047920	0.72619	0.573439459	-0.340706	2.85E-05	hypomethylated
cg26976108	0.73903	0.583538378	-0.340805	1.80E-05	hypomethylated
cg04261607	0.64135	0.506404865	-0.340821	4.70E-05	hypomethylated
cg26552233	0.83719	0.661014595	-0.340873	0.000207	hypomethylated
cg00232996	0.7555	0.596514054	-0.340876	0.000132	hypomethylated
cg02473847	0.83978	0.663057838	-0.340877	0.000109	hypomethylated
cg01952537	0.6139	0.484710811	-0.340879	0.000126	hypomethylated
cg20359793	0.79152	0.624936216	-0.340917	1.30E-05	hypomethylated
cg08654262	0.85621	0.675942703	-0.341064	8.22E-05	hypomethylated
cg06862696	0.79893	0.630712432	-0.341087	5.58E-05	hypomethylated
cg17438457	0.83021	0.655395676	-0.34111	3.49E-05	hypomethylated
cg10914815	0.77117	0.608736757	-0.34123	0.000358	hypomethylated
cg26890181	0.69695	0.550135135	-0.341269	0.000138	hypomethylated
cg08096702	0.92256	0.728210811	-0.341287	4.58E-05	hypomethylated
cg04874562	0.65839	0.519648649	-0.341406	0.000214	hypomethylated
cg17947364	0.85175	0.672239459	-0.341455	7.12E-05	hypomethylated
cg00555085	0.60295	0.475835676	-0.341575	0.000313	hypomethylated
cg12468786	0.64964	0.512680541	-0.34158	8.42E-05	hypomethylated
cg11733958	0.72654	0.573323784	-0.341692	0.000681	hypomethylated
cg04482760	0.83876	0.661828649	-0.3418	0.00012	hypomethylated
cg00740389	0.9125	0.719996757	-0.341834	3.23E-05	hypomethylated
cg26545519	0.85229	0.672477297	-0.341859	1.84E-05	hypomethylated
cg00868476	0.8177	0.645168649	-0.341895	4.26E-05	hypomethylated
cg13010373	0.77757	0.613503784	-0.3419	9.49E-05	hypomethylated
cg27066295	0.88535	0.698533514	-0.341919	3.04E-05	hypomethylated
cg01300684	0.67669	0.533868108	-0.342012	0.000106	hypomethylated
cg15091123	0.80316	0.633627568	-0.342052	1.40E-05	hypomethylated
cg01071644	0.57593	0.454358378	-0.342063	0.000857	hypomethylated
cg27168858	0.75702	0.597195676	-0.342128	9.49E-05	hypomethylated
cg11099375	0.83003	0.654785405	-0.342141	0.00024	hypomethylated
cg12535402	0.78934	0.622662703	-0.342196	9.49E-05	hypomethylated
cg14370507	0.81808	0.645317838	-0.342232	0.000229	hypomethylated
cg09423651	0.81708	0.644514054	-0.342266	0.00031	hypomethylated
cg17929273	0.70603	0.556911351	-0.342282	7.65E-05	hypomethylated
cg11188031	0.92471	0.7294	-0.342291	5.58E-06	hypomethylated
cg02076624	0.71645	0.565101081	-0.342357	0.000159	hypomethylated
cg02995731	0.73699	0.581278378	-0.342416	1.97E-05	hypomethylated

cg18195228	0.81226	0.640641081	-0.342425	2.51E-05	hypomethylated
cg10105268	0.90211	0.7115	-0.34244	1.25E-05	hypomethylated
cg24647031	0.91792	0.723921622	-0.342535	3.90E-06	hypomethylated
cg06371582	0.78284	0.617382703	-0.342552	0.001409	hypomethylated
cg25203007	0.57294	0.451838378	-0.342577	0.000212	hypomethylated
cg24994360	0.64837	0.511305405	-0.342632	0.000382	hypomethylated
cg08089543	0.53007	0.417991351	-0.34271	6.15E-05	hypomethylated
cg23711059	0.66951	0.527942162	-0.342726	0.0006	hypomethylated
cg21765032	0.78938	0.622437838	-0.34279	4.70E-05	hypomethylated
cg22745143	0.80964	0.638398378	-0.342824	0.000224	hypomethylated
cg07229760	0.72694	0.573169189	-0.342875	5.19E-05	hypomethylated
cg10654948	0.90427	0.712933514	-0.342986	3.00E-05	hypomethylated
cg21531389	0.66086	0.521015676	-0.343018	0.007666	hypomethylated
cg01966732	0.76271	0.601272973	-0.343115	0.00028	hypomethylated
cg04840800	0.81915	0.645739459	-0.343175	1.15E-05	hypomethylated
cg18297705	0.74362	0.586165405	-0.343258	3.00E-05	hypomethylated
cg01944226	0.86488	0.68174	-0.343278	0.0002	hypomethylated
cg17346246	0.90227	0.711202703	-0.343298	8.84E-05	hypomethylated
cg09414620	0.58404	0.460353514	-0.343325	0.000303	hypomethylated
cg00201457	0.53386	0.420792973	-0.343351	0.000195	hypomethylated
cg04804094	0.58944	0.464594595	-0.343373	7.84E-05	hypomethylated
cg23088810	0.86835	0.684419459	-0.343396	5.73E-06	hypomethylated
cg24074448	0.67107	0.528923784	-0.343403	0.00101	hypomethylated
cg13708222	0.61107	0.481627027	-0.343421	0.000674	hypomethylated
cg12495853	0.85187	0.671383784	-0.343496	2.21E-05	hypomethylated
cg01662942	0.88142	0.694636216	-0.343572	2.57E-05	hypomethylated
cg10352418	0.87031	0.685878919	-0.343575	1.01E-06	hypomethylated
cg16726435	0.92644	0.7301	-0.343603	3.79E-06	hypomethylated
cg10219995	0.69455	0.547327568	-0.343674	0.000182	hypomethylated
cg14356530	0.66712	0.525701622	-0.343702	0.00013	hypomethylated
cg13512859	0.52385	0.412793514	-0.343733	0.000219	hypomethylated
cg05481579	0.77431	0.610122162	-0.343813	4.86E-06	hypomethylated
cg02574101	0.63972	0.504062703	-0.343837	0.000342	hypomethylated
cg08241082	0.86742	0.683467568	-0.343858	1.62E-05	hypomethylated
cg26342985	0.81688	0.643642703	-0.343864	0.000134	hypomethylated
cg14438695	0.80001	0.630349189	-0.343867	0.000155	hypomethylated
cg03562360	0.76817	0.605194595	-0.344027	0.000229	hypomethylated
cg05869417	0.61965	0.488182162	-0.344034	0.000857	hypomethylated
cg09060469	0.90962	0.716593514	-0.344109	4.60E-06	hypomethylated
cg03969763	0.77686	0.611987568	-0.344152	3.62E-05	hypomethylated
cg01012026	0.63267	0.498396216	-0.34416	7.29E-05	hypomethylated
cg08597802	0.79862	0.629098378	-0.344224	0.000113	hypomethylated
cg21643547	0.73376	0.577985946	-0.344274	9.72E-05	hypomethylated
cg01545079	0.81291	0.640331892	-0.344276	4.48E-06	hypomethylated
cg18784113	0.81274	0.64018	-0.344316	9.95E-05	hypomethylated
cg05790960	0.76904	0.60574973	-0.344337	9.27E-05	hypomethylated
cg08726863	0.7435	0.585623784	-0.344359	0.00054	hypomethylated
cg00682487	0.87658	0.690415135	-0.344422	5.14E-06	hypomethylated
cg25214346	0.62416	0.491596757	-0.344441	0.000214	hypomethylated
cg02871021	0.6097	0.480193514	-0.344484	0.000369	hypomethylated
cg04478698	0.84746	0.667338919	-0.344726	5.86E-05	hypomethylated
cg05444541	0.70596	0.555904324	-0.34475	0.00032	hypomethylated
cg00946479	0.80818	0.636363784	-0.344825	3.15E-05	hypomethylated
cg04854189	0.88707	0.698478919	-0.344831	2.87E-06	hypomethylated
cg13185702	0.80164	0.631204865	-0.344846	0.00017	hypomethylated
cg23154272	0.61113	0.481163243	-0.344953	5.06E-05	hypomethylated
cg09959684	0.82893	0.652642162	-0.344958	7.38E-05	hypomethylated
cg15440661	0.65441	0.515223243	-0.344997	9.83E-05	hypomethylated

cg23877497	0.84318	0.663793514	-0.345106	2.04E-05	hypomethylated
cg05625526	0.78575	0.618578378	-0.345114	9.82E-06	hypomethylated
cg04755857	0.83685	0.658769189	-0.345196	0.000174	hypomethylated
cg20206377	0.5229	0.411621081	-0.345218	0.001052	hypomethylated
cg03860038	0.87823	0.691331892	-0.34522	0.000342	hypomethylated
cg03476212	0.72232	0.56856	-0.345326	0.000245	hypomethylated
cg10960681	0.83906	0.660449189	-0.345326	1.28E-05	hypomethylated
cg03992830	0.84858	0.667938919	-0.345334	2.85E-05	hypomethylated
cg08789739	0.92475	0.727887027	-0.345349	4.73E-06	hypomethylated
cg04807739	0.84758	0.667135676	-0.345369	2.49E-06	hypomethylated
cg24506604	0.81194	0.639082703	-0.34537	2.92E-05	hypomethylated
cg11672772	0.84909	0.668312432	-0.345395	0.000154	hypomethylated
cg02757488	0.5266	0.41444	-0.345544	0.000703	hypomethylated
cg16238419	0.82052	0.645756216	-0.345549	7.52E-06	hypomethylated
cg20802826	0.72371	0.56953027	-0.345639	0.000251	hypomethylated
cg14195059	0.53956	0.424607027	-0.345655	0.000123	hypomethylated
cg09164206	0.85412	0.672141622	-0.345674	0.000187	hypomethylated
cg15450768	0.85507	0.672862703	-0.34573	4.36E-05	hypomethylated
cg02664479	0.73073	0.575017297	-0.345733	0.00054	hypomethylated
cg24032414	0.82318	0.647745405	-0.345781	5.58E-06	hypomethylated
cg01174240	0.56122	0.44159027	-0.345858	0.000209	hypomethylated
cg25315503	0.54986	0.432642162	-0.34589	0.000101	hypomethylated
cg03873049	0.75291	0.592387027	-0.345937	7.29E-05	hypomethylated
cg08264266	0.76413	0.601212973	-0.345942	0.000123	hypomethylated
cg06786600	0.77071	0.606378919	-0.345969	0.000124	hypomethylated
cg18202205	0.84071	0.661451351	-0.345973	4.82E-05	hypomethylated
cg02909097	0.75686	0.595474054	-0.345988	3.19E-05	hypomethylated
cg12305074	0.85292	0.67104	-0.346012	0.00029	hypomethylated
cg02034222	0.8787	0.691241081	-0.346182	6.31E-05	hypomethylated
cg07554496	0.92991	0.731524324	-0.346185	1.80E-05	hypomethylated
cg19128261	0.77001	0.605726486	-0.346211	3.57E-05	hypomethylated
cg14321936	0.75867	0.596798919	-0.346228	3.00E-05	hypomethylated
cg16611584	0.68135	0.535970811	-0.346242	0.002699	hypomethylated
cg24813588	0.66073	0.51974	-0.346271	0.000104	hypomethylated
cg16369987	0.57485	0.452168649	-0.346325	0.000104	hypomethylated
cg16658460	0.83548	0.657139459	-0.346406	0.000157	hypomethylated
cg14210454	0.83308	0.655245946	-0.346419	2.42E-05	hypomethylated
cg10959999	0.68057	0.53528973	-0.346424	4.47E-05	hypomethylated
cg00703843	0.83335	0.655447568	-0.346442	1.72E-06	hypomethylated
cg15646543	0.75172	0.591203784	-0.34654	0.000338	hypomethylated
cg00668515	0.66504	0.523010811	-0.3466	5.72E-05	hypomethylated
cg07285675	0.86115	0.677226486	-0.346626	3.67E-05	hypomethylated
cg13401658	0.8971	0.705415676	-0.346795	1.04E-05	hypomethylated
cg00966522	0.51307	0.403431892	-0.346831	0.000178	hypomethylated
cg21914526	0.75538	0.593915135	-0.346946	6.23E-05	hypomethylated
cg09480446	0.77727	0.611099459	-0.347009	0.000216	hypomethylated
cg16179720	0.75795	0.595895676	-0.347043	0.000251	hypomethylated
cg09213451	0.76211	0.599153514	-0.347074	0.00054	hypomethylated
cg01411912	0.70793	0.556557297	-0.347076	7.84E-05	hypomethylated
cg16042652	0.81072	0.637353514	-0.34711	6.15E-05	hypomethylated
cg25313204	0.64342	0.505815135	-0.347151	0.001839	hypomethylated
cg19927565	0.54539	0.42874	-0.347185	0.000191	hypomethylated
cg02179764	0.92947	0.730648649	-0.34723	7.03E-06	hypomethylated
cg16775939	0.95483	0.750578919	-0.34724	4.48E-06	hypomethylated
cg03491895	0.81044	0.637073514	-0.347246	0.000245	hypomethylated
cg07143559	0.73981	0.581535676	-0.347287	7.12E-05	hypomethylated
cg22249291	0.69051	0.542772973	-0.347313	2.85E-05	hypomethylated
cg26226988	0.84919	0.667468649	-0.347387	0.000613	hypomethylated

cg09617441	0.7728	0.607421081	-0.347398	9.72E-05	hypomethylated
cg04151220	0.83375	0.655285946	-0.34749	0.000234	hypomethylated
cg19272720	0.73902	0.580820541	-0.347521	0.000358	hypomethylated
cg17445212	0.73002	0.573742162	-0.347533	0.00054	hypomethylated
cg22976120	0.75971	0.597065946	-0.347559	0.000485	hypomethylated
cg08228672	0.708	0.556422703	-0.347568	4.15E-05	hypomethylated
cg18855621	0.86386	0.678911892	-0.347573	1.62E-05	hypomethylated
cg01801826	0.72716	0.571478378	-0.347574	0.000358	hypomethylated
cg16502747	0.58517	0.459884324	-0.347585	0.000464	hypomethylated
cg07198365	0.93964	0.738451892	-0.347604	0.000103	hypomethylated
cg11570656	0.53851	0.423207027	-0.34761	0.000229	hypomethylated
cg25840824	0.82639	0.649422162	-0.347666	0.000163	hypomethylated
cg02153041	0.66523	0.52276973	-0.347678	0.000204	hypomethylated
cg11435441	0.86021	0.675993514	-0.347679	0.00094	hypomethylated
cg12574576	0.72836	0.572361081	-0.347726	0.000187	hypomethylated
cg20462100	0.66821	0.525089189	-0.347739	0.000245	hypomethylated
cg02356435	0.81089	0.637205405	-0.347748	0.00098	hypomethylated
cg14663208	0.84676	0.665376757	-0.347782	7.47E-05	hypomethylated
cg15610437	0.68615	0.539151351	-0.347834	0.000382	hypomethylated
cg10319709	0.85496	0.671793514	-0.347839	9.72E-05	hypomethylated
cg17810211	0.78371	0.615764865	-0.34794	0.0003	hypomethylated
cg02678414	0.85266	0.66993027	-0.34796	3.07E-05	hypomethylated
cg13392029	0.66922	0.525799459	-0.347968	0.002318	hypomethylated
cg09115713	0.86468	0.679365405	-0.347979	0.000232	hypomethylated
cg09644157	0.6315	0.496139459	-0.348037	0.000232	hypomethylated
cg08940169	0.7435	0.584091351	-0.348139	0.000148	hypomethylated
cg22289115	0.66265	0.520557838	-0.348189	0.0002	hypomethylated
cg23679992	0.6987	0.548822703	-0.348333	0.000101	hypomethylated
cg00908813	0.87903	0.690457297	-0.34836	3.67E-05	hypomethylated
cg22493616	0.76493	0.600784865	-0.348479	0.000313	hypomethylated
cg22357379	0.58794	0.461752432	-0.348549	2.10E-05	hypomethylated
cg21584862	0.9044	0.710271892	-0.34859	2.46E-06	hypomethylated
cg23985077	0.85835	0.674053514	-0.348703	2.92E-05	hypomethylated
cg02554274	0.50552	0.396974054	-0.348723	0.003021	hypomethylated
cg19712659	0.57959	0.455092973	-0.348871	0.000277	hypomethylated
cg26876101	0.60124	0.472088108	-0.348885	0.000506	hypomethylated
cg04951638	0.88027	0.691177838	-0.348889	3.30E-06	hypomethylated
cg09892262	0.86577	0.679763243	-0.348951	1.01E-05	hypomethylated
cg20998127	0.85705	0.672885946	-0.349017	3.49E-05	hypomethylated
cg19974120	0.8145	0.639476757	-0.349023	7.12E-05	hypomethylated
cg23894587	0.80009	0.628148649	-0.349056	4.05E-05	hypomethylated
cg12493906	0.85875	0.674193514	-0.349075	0.000187	hypomethylated
cg21484512	0.7693	0.603961622	-0.349089	0.000111	hypomethylated
cg08302325	0.59535	0.46739027	-0.34911	0.000653	hypomethylated
cg00038857	0.81341	0.638513514	-0.349266	4.58E-05	hypomethylated
cg22379436	0.80558	0.632350811	-0.349303	0.000575	hypomethylated
cg13348574	0.80218	0.629672432	-0.349324	8.63E-05	hypomethylated
cg05164406	0.91233	0.716125946	-0.349342	5.81E-06	hypomethylated
cg09576223	0.70218	0.551157838	-0.349375	0.000454	hypomethylated
cg04884612	0.80154	0.629141081	-0.349391	1.94E-05	hypomethylated
cg03437675	0.64492	0.506197838	-0.349419	7.74E-05	hypomethylated
cg13901000	0.81282	0.637978919	-0.349427	0.0002	hypomethylated
cg03080043	0.90193	0.707894054	-0.349482	3.95E-05	hypomethylated
cg05673731	0.77178	0.605720541	-0.349537	0.001395	hypomethylated
cg22844623	0.74039	0.581077838	-0.349554	1.99E-05	hypomethylated
cg24659758	0.83955	0.658848649	-0.349669	1.75E-05	hypomethylated
cg27478879	0.60952	0.47831027	-0.349727	6.01E-05	hypomethylated
cg08091707	0.66624	0.522808649	-0.349759	0.002649	hypomethylated

cg00657094	0.78784	0.618229189	-0.349761	7.84E-05	hypomethylated
cg08202972	0.77883	0.611147568	-0.349788	0.000102	hypomethylated
cg26223797	0.88899	0.697578919	-0.349811	2.64E-05	hypomethylated
cg17028814	0.6963	0.546369189	-0.349833	0.000214	hypomethylated
cg02196147	0.79167	0.621182162	-0.349883	4.47E-05	hypomethylated
cg13511231	0.73186	0.574206486	-0.349998	0.000152	hypomethylated
cg06466839	0.82262	0.645385405	-0.350065	0.00039	hypomethylated
cg24866792	0.56772	0.445400541	-0.350076	8.03E-05	hypomethylated
cg27019158	0.8572	0.672461081	-0.350181	2.71E-05	hypomethylated
cg27535687	0.73358	0.575471351	-0.35021	1.58E-05	hypomethylated
cg26608199	0.84576	0.66345027	-0.35026	5.86E-05	hypomethylated
cg27472701	0.93125	0.730485405	-0.350313	1.09E-05	hypomethylated
cg23095988	0.56146	0.440412973	-0.350326	0.000251	hypomethylated
cg25714326	0.83525	0.655167568	-0.350344	2.51E-05	hypomethylated
cg20468081	0.79725	0.625268649	-0.350556	2.92E-05	hypomethylated
cg04338119	0.80542	0.631656216	-0.350602	2.64E-06	hypomethylated
cg01182863	0.97848	0.767367568	-0.350625	2.95E-06	hypomethylated
cg07679322	0.86603	0.679176216	-0.350631	1.23E-05	hypomethylated
cg10095801	0.74146	0.581469189	-0.350666	9.72E-05	hypomethylated
cg00052588	0.9211	0.722328108	-0.350703	2.68E-06	hypomethylated
cg18503234	0.61714	0.483938378	-0.350774	0.002478	hypomethylated
cg11100658	0.82327	0.645577297	-0.350776	9.95E-05	hypomethylated
cg24663419	0.81947	0.642560541	-0.350859	5.06E-05	hypomethylated
cg04509221	0.74778	0.586345405	-0.350863	0.000501	hypomethylated
cg15078958	0.56356	0.441887568	-0.35089	0.000262	hypomethylated
cg01761236	0.72474	0.568241622	-0.350959	8.03E-05	hypomethylated
cg25344194	0.59026	0.462776216	-0.351036	0.000182	hypomethylated
cg23119026	0.55128	0.432207568	-0.351061	3.40E-05	hypomethylated
cg17243957	0.8053	0.631261081	-0.35129	3.15E-05	hypomethylated
cg25648211	0.97048	0.760709189	-0.351353	1.93E-06	hypomethylated
cg26846864	0.77561	0.607908649	-0.351477	0.000224	hypomethylated
cg09410380	0.89384	0.700514054	-0.351603	1.48E-05	hypomethylated
cg13013841	0.67819	0.531496216	-0.35163	0.007035	hypomethylated
cg12056210	0.64594	0.506210811	-0.351662	0.000117	hypomethylated
cg16567216	0.74884	0.586844324	-0.35168	0.001031	hypomethylated
cg05856951	0.87037	0.682013514	-0.351829	3.15E-05	hypomethylated
cg09633579	0.54182	0.424553514	-0.351867	1.62E-05	hypomethylated
cg01277983	0.78307	0.613572973	-0.351906	4.26E-05	hypomethylated
cg20064006	0.86572	0.678322162	-0.35193	9.96E-06	hypomethylated
cg08378742	0.85031	0.666196757	-0.352041	4.70E-05	hypomethylated
cg11294312	0.77924	0.610509189	-0.352055	5.58E-06	hypomethylated
cg20146967	0.83343	0.652954054	-0.35208	1.28E-05	hypomethylated
cg16291069	0.84404	0.661260541	-0.352093	7.29E-05	hypomethylated
cg14384685	0.84204	0.659688649	-0.352103	1.77E-05	hypomethylated
cg07924154	0.59709	0.467776216	-0.35213	5.73E-06	hypomethylated
cg04968426	0.82871	0.649205405	-0.352192	1.99E-05	hypomethylated
cg10055235	0.78988	0.618784865	-0.352196	6.93E-06	hypomethylated
cg13948456	0.65643	0.51424	-0.352199	2.51E-05	hypomethylated
cg22581200	0.89874	0.704048108	-0.35223	8.37E-06	hypomethylated
cg08042273	0.74771	0.585717838	-0.352273	3.23E-05	hypomethylated
cg16928869	0.79876	0.625705405	-0.352279	2.15E-05	hypomethylated
cg25652751	0.85043	0.66617027	-0.352302	8.22E-05	hypomethylated
cg03462053	0.88993	0.697097838	-0.352331	8.37E-06	hypomethylated
cg02242572	0.687	0.538117297	-0.352389	0.00101	hypomethylated
cg07041720	0.74283	0.58184	-0.35241	0.000234	hypomethylated
cg14343414	0.88821	0.69571027	-0.352414	2.23E-06	hypomethylated
cg04531473	0.52519	0.411358378	-0.352444	0.000104	hypomethylated
cg24932628	0.82173	0.643603243	-0.352493	7.65E-05	hypomethylated

cg03619332	0.62669	0.490838378	-0.352504	6.95E-05	hypomethylated
cg05896714	0.84009	0.657958378	-0.352548	0.000581	hypomethylated
cg00249205	0.71582	0.56062973	-0.352549	9.27E-05	hypomethylated
cg10334976	0.8301	0.650128108	-0.352561	0.000107	hypomethylated
cg00665343	0.83378	0.652992432	-0.3526	2.57E-05	hypomethylated
cg10273135	0.75415	0.590621622	-0.352617	0.000667	hypomethylated
cg07556018	0.75011	0.587455135	-0.352624	0.001118	hypomethylated
cg19217463	0.71557	0.56035027	-0.352764	0.000822	hypomethylated
cg22678398	0.81139	0.635364865	-0.35281	0.000129	hypomethylated
cg25686905	0.82129	0.643093514	-0.352863	1.58E-05	hypomethylated
cg21331947	0.71562	0.560337838	-0.352897	0.001212	hypomethylated
cg21245092	0.76306	0.597479459	-0.352907	0.000132	hypomethylated
cg05599155	0.95349	0.74656973	-0.352941	2.51E-05	hypomethylated
cg19804071	0.89637	0.701839459	-0.352953	3.00E-05	hypomethylated
cg26259865	0.71969	0.56348	-0.353011	3.76E-05	hypomethylated
cg02526838	0.93712	0.733665946	-0.35311	1.09E-06	hypomethylated
cg11453400	0.70732	0.553751351	-0.353125	0.000259	hypomethylated
cg13223721	0.85566	0.66988	-0.353135	2.87E-06	hypomethylated
cg11214532	0.8783	0.687598919	-0.353147	2.10E-05	hypomethylated
cg21750887	0.78587	0.615237297	-0.353148	4.82E-05	hypomethylated
cg00679556	0.77604	0.607534595	-0.353164	0.000155	hypomethylated
cg13491490	0.81239	0.635950811	-0.353257	0.00024	hypomethylated
cg08495245	0.64957	0.508490811	-0.353263	0.001857	hypomethylated
cg15149205	0.85338	0.668034595	-0.353265	6.01E-05	hypomethylated
cg06113298	0.53771	0.420922162	-0.353275	0.000262	hypomethylated
cg01447854	0.82325	0.644395135	-0.353385	1.90E-06	hypomethylated
cg09247020	0.82857	0.648548649	-0.353409	5.65E-05	hypomethylated
cg12531542	0.90611	0.709240541	-0.353411	1.80E-05	hypomethylated
cg26515755	0.77904	0.609692973	-0.353614	0.000107	hypomethylated
cg20287434	0.68398	0.535285946	-0.353644	1.06E-05	hypomethylated
cg13827582	0.76529	0.598914595	-0.353656	0.00029	hypomethylated
cg00932063	0.57715	0.451655676	-0.353723	0.000219	hypomethylated
cg26482939	0.7232	0.565927027	-0.353779	0.001875	hypomethylated
cg16378003	0.80067	0.626474054	-0.353953	2.24E-05	hypomethylated
cg26236329	0.58958	0.461302162	-0.353976	0.014259	hypomethylated
cg01078048	0.55464	0.433961622	-0.353984	0.000417	hypomethylated
cg02005771	0.81334	0.636360541	-0.354014	0.000454	hypomethylated
cg05980111	0.75745	0.592620541	-0.354042	0.000112	hypomethylated
cg15729307	0.7937	0.620943243	-0.354132	0.000445	hypomethylated
cg15601361	0.78595	0.614860541	-0.354178	0.000187	hypomethylated
cg09113474	0.79175	0.619381622	-0.354216	7.29E-05	hypomethylated
cg23599026	0.43903	0.343433514	-0.354289	0.000219	hypomethylated
cg08536838	0.83338	0.651907568	-0.354307	1.25E-05	hypomethylated
cg10464130	0.62693	0.490412973	-0.354307	0.000931	hypomethylated
cg05629278	0.85083	0.665532432	-0.354362	1.12E-05	hypomethylated
cg22590522	0.737	0.576446486	-0.354478	1.12E-06	hypomethylated
cg01233518	0.96122	0.751772973	-0.35457	1.50E-05	hypomethylated
cg15601682	0.96676	0.756098919	-0.354583	3.11E-05	hypomethylated
cg07950397	0.45331	0.354527027	-0.354602	4.58E-05	hypomethylated
cg05601974	0.63869	0.499498378	-0.354636	0.000607	hypomethylated
cg24015522	0.85459	0.668333514	-0.354664	8.22E-05	hypomethylated
cg04615850	0.60386	0.47222973	-0.354725	7.12E-05	hypomethylated
cg04125273	0.65353	0.511058919	-0.354764	0.000189	hypomethylated
cg18019572	0.78954	0.617384865	-0.354842	0.000274	hypomethylated
cg01763884	0.75802	0.592711892	-0.354905	3.15E-05	hypomethylated
cg09264065	0.6002	0.469307027	-0.354911	0.000588	hypomethylated
cg13209113	0.78547	0.614166486	-0.354926	4.15E-05	hypomethylated
cg02100410	0.75226	0.588197838	-0.35493	8.13E-05	hypomethylated

cg10856296	0.89937	0.703179459	-0.355022	4.80E-06	hypomethylated
cg07013680	0.7046	0.550880541	-0.355065	2.81E-05	hypomethylated
cg17894779	0.79561	0.622034595	-0.355067	4.00E-05	hypomethylated
cg22576950	0.67627	0.528667027	-0.35524	0.000214	hypomethylated
cg08610968	0.87304	0.682480541	-0.35526	1.12E-05	hypomethylated
cg08703808	0.77726	0.607590811	-0.355297	7.65E-05	hypomethylated
cg00563566	0.79867	0.62432	-0.355314	4.36E-05	hypomethylated
cg15345396	0.87785	0.686182162	-0.355383	3.00E-05	hypomethylated
cg01279378	0.72928	0.570042162	-0.355404	1.35E-05	hypomethylated
cg08314176	0.63331	0.494988649	-0.355516	0.001667	hypomethylated
cg04012857	0.75869	0.592972973	-0.355544	0.000193	hypomethylated
cg19163939	0.85606	0.669073514	-0.355547	5.19E-05	hypomethylated
cg11739297	0.90667	0.708625946	-0.355553	1.01E-05	hypomethylated
cg16710099	0.67146	0.524788649	-0.355565	6.31E-05	hypomethylated
cg21503392	0.70894	0.554077838	-0.355575	3.40E-05	hypomethylated
cg18596043	0.7387	0.577329189	-0.355594	6.31E-05	hypomethylated
cg02667656	0.78186	0.610980541	-0.355784	5.65E-06	hypomethylated
cg09113530	0.5916	0.462298919	-0.355796	0.0002	hypomethylated
cg01905142	0.63262	0.494352973	-0.355798	2.96E-05	hypomethylated
cg22036057	0.8258	0.645264865	-0.355901	2.64E-05	hypomethylated
cg01154336	0.76342	0.596485405	-0.35599	7.65E-05	hypomethylated
cg12759387	0.59193	0.462489189	-0.356007	0.00035	hypomethylated
cg15808008	0.73052	0.570769189	-0.356016	2.38E-05	hypomethylated
cg11161550	0.51792	0.404631892	-0.356119	8.37E-06	hypomethylated
cg00816609	0.74427	0.58146973	-0.356122	0.000154	hypomethylated
cg07563033	0.82711	0.646156216	-0.356196	0.000107	hypomethylated
cg16215705	0.7757	0.605979459	-0.35623	0.000293	hypomethylated
cg27137836	0.81389	0.635807568	-0.356244	7.32E-06	hypomethylated
cg01109135	0.41591	0.324905405	-0.356252	0.000912	hypomethylated
cg13534999	0.87036	0.679915135	-0.356258	2.85E-05	hypomethylated
cg07287508	0.65176	0.509137838	-0.356285	0.000107	hypomethylated
cg15404019	0.65334	0.51033027	-0.356403	0.000408	hypomethylated
cg07759833	0.81391	0.635731351	-0.356452	1.84E-05	hypomethylated
cg01537494	0.62191	0.485737838	-0.356528	4.26E-05	hypomethylated
cg11860777	0.77787	0.607518378	-0.356601	0.000229	hypomethylated
cg01512532	0.826	0.645105946	-0.356606	0.000101	hypomethylated
cg21236500	0.87099	0.680238378	-0.356616	3.67E-05	hypomethylated
cg08336938	0.88175	0.688621622	-0.356658	3.95E-05	hypomethylated
cg20828084	0.82308	0.642758919	-0.356755	0.000256	hypomethylated
cg00004089	0.81077	0.633138378	-0.356772	7.29E-05	hypomethylated
cg08097086	0.70311	0.549063243	-0.356778	5.72E-05	hypomethylated
cg03967448	0.7748	0.605044865	-0.356782	0.000135	hypomethylated
cg09863950	0.7674	0.599265405	-0.356784	9.27E-05	hypomethylated
cg09696115	0.72994	0.569985946	-0.356852	5.19E-05	hypomethylated
cg24929181	0.86788	0.67769027	-0.35687	1.63E-06	hypomethylated
cg04607131	0.65726	0.513224865	-0.356873	0.000358	hypomethylated
cg07694621	0.68593	0.535596216	-0.356916	0.000756	hypomethylated
cg26296653	0.71473	0.558071892	-0.356947	2.32E-05	hypomethylated
cg23979832	0.70095	0.547306486	-0.356963	0.000912	hypomethylated
cg26286365	0.70416	0.549792973	-0.357015	0.000395	hypomethylated
cg27261397	0.87708	0.684785946	-0.357055	1.73E-05	hypomethylated
cg21166985	0.90019	0.702817838	-0.357079	6.95E-05	hypomethylated
cg13982366	0.74659	0.582886486	-0.357101	0.000313	hypomethylated
cg13411229	0.67883	0.529965946	-0.357151	0.000102	hypomethylated
cg09926488	0.70173	0.54783027	-0.357187	4.47E-05	hypomethylated
cg03607648	0.88806	0.69328973	-0.357199	1.94E-05	hypomethylated
cg08722675	0.63611	0.496582162	-0.357244	0.003843	hypomethylated
cg02483101	0.55603	0.433986486	-0.357513	0.000296	hypomethylated

cg17389427	0.66811	0.521460541	-0.357528	0.000109	hypomethylated
cg04309194	0.67567	0.527356757	-0.35754	0.00012	hypomethylated
cg15429888	0.82784	0.64608	-0.357639	2.27E-05	hypomethylated
cg07022048	0.48054	0.375030811	-0.357647	0.000159	hypomethylated
cg24998110	0.78925	0.615950811	-0.357667	0.000154	hypomethylated
cg25214310	0.66608	0.519772973	-0.357814	0.000245	hypomethylated
cg18163092	0.97272	0.75899027	-0.357943	1.41E-06	hypomethylated
cg01618151	0.66845	0.521567027	-0.357967	0.002856	hypomethylated
cg24597131	0.75254	0.587161622	-0.358011	1.46E-05	hypomethylated
cg00571809	0.86722	0.676637297	-0.358015	6.78E-05	hypomethylated
cg08670181	0.87296	0.681071351	-0.35811	1.12E-05	hypomethylated
cg11024522	0.63958	0.498975135	-0.358157	5.86E-05	hypomethylated
cg26338202	0.83716	0.653118378	-0.358159	0.00014	hypomethylated
cg02412803	0.70858	0.552771892	-0.358246	1.46E-05	hypomethylated
cg26234644	0.8868	0.691800541	-0.358253	3.76E-05	hypomethylated
cg17978103	0.85407	0.666261622	-0.358266	3.11E-05	hypomethylated
cg25928742	0.63663	0.496596757	-0.35838	1.75E-05	hypomethylated
cg04945153	0.6377	0.49742	-0.358413	8.03E-05	hypomethylated
cg00148223	0.63345	0.494097838	-0.358434	0.000209	hypomethylated
cg17046756	0.62837	0.490106486	-0.358519	0.00017	hypomethylated
cg21446172	0.62426	0.486900541	-0.35852	0.000187	hypomethylated
cg09995507	0.73528	0.573478919	-0.358553	5.65E-05	hypomethylated
cg22945666	0.81175	0.633118378	-0.35856	2.96E-05	hypomethylated
cg25597362	0.86567	0.675148649	-0.358612	3.15E-05	hypomethylated
cg04095776	0.57248	0.446485405	-0.358612	2.15E-05	hypomethylated
cg06255037	0.67035	0.522793514	-0.358673	8.63E-05	hypomethylated
cg04959298	0.68865	0.537056757	-0.358696	5.45E-05	hypomethylated
cg23258400	0.82115	0.640380541	-0.358716	0.000234	hypomethylated
cg08903389	0.84142	0.656165405	-0.358767	0.000262	hypomethylated
cg01121978	0.9495	0.740425405	-0.358814	6.66E-06	hypomethylated
cg07622079	0.82969	0.646992973	-0.358822	8.15E-06	hypomethylated
cg01330456	0.93503	0.729121622	-0.358853	2.46E-06	hypomethylated
cg17469934	0.58531	0.456380541	-0.358964	0.000168	hypomethylated
cg09600088	0.88218	0.687817297	-0.359048	2.27E-05	hypomethylated
cg19056664	0.6331	0.493610811	-0.359059	3.67E-05	hypomethylated
cg09964873	0.82353	0.642041622	-0.359154	1.50E-05	hypomethylated
cg11666857	0.82565	0.643683243	-0.359179	4.12E-06	hypomethylated
cg22211507	0.68278	0.5323	-0.359181	0.000187	hypomethylated
cg06111140	0.73277	0.57126	-0.359213	0.000277	hypomethylated
cg04368724	0.82836	0.645778378	-0.359219	8.37E-06	hypomethylated
cg18232841	0.6873	0.535802162	-0.35924	0.000187	hypomethylated
cg26531076	0.93912	0.732099459	-0.35927	1.18E-06	hypomethylated
cg10858677	0.68053	0.53051027	-0.359278	0.000653	hypomethylated
cg05897465	0.70689	0.551057838	-0.359282	6.86E-05	hypomethylated
cg06654510	0.80031	0.623875676	-0.3593	0.0003	hypomethylated
cg17009836	0.53265	0.415207027	-0.359357	6.46E-05	hypomethylated
cg02303801	0.59953	0.467334595	-0.359376	0.000805	hypomethylated
cg03234072	0.87572	0.682615676	-0.359396	5.86E-05	hypomethylated
cg05634879	0.81181	0.632727027	-0.359559	0.000147	hypomethylated
cg11862081	0.64472	0.502468649	-0.359639	9.72E-05	hypomethylated
cg10632966	0.80068	0.623985946	-0.359712	9.49E-05	hypomethylated
cg15016120	0.70601	0.550167568	-0.359818	0.000756	hypomethylated
cg09354294	0.52708	0.410723243	-0.359855	7.38E-05	hypomethylated
cg17386689	0.80364	0.626225946	-0.359866	0.000224	hypomethylated
cg14936266	0.75404	0.587569189	-0.359882	2.21E-05	hypomethylated
cg13853813	0.86227	0.671887027	-0.359921	8.71E-06	hypomethylated
cg18050903	0.68343	0.532525405	-0.359943	5.86E-05	hypomethylated
cg20805475	0.61399	0.478397838	-0.360004	0.004134	hypomethylated

cg13151664	0.81373	0.634002162	-0.360062	0.000209	hypomethylated
cg14530930	0.77669	0.605137297	-0.360076	1.71E-05	hypomethylated
cg23272369	0.89718	0.69895027	-0.360208	3.74E-06	hypomethylated
cg09489757	0.67352	0.524627568	-0.360427	0.000187	hypomethylated
cg16096836	0.7977	0.621322162	-0.360505	0.000912	hypomethylated
cg02785332	0.83218	0.648164865	-0.360535	7.12E-05	hypomethylated
cg21211321	0.83866	0.653206486	-0.360547	0.000195	hypomethylated
cg20458044	0.76437	0.595318378	-0.36061	0.000575	hypomethylated
cg00805360	0.75214	0.585756216	-0.360701	4.94E-05	hypomethylated
cg08222913	0.78619	0.612245946	-0.360767	2.48E-05	hypomethylated
cg17590003	0.60616	0.472030811	-0.360818	0.000789	hypomethylated
cg22044398	0.62159	0.484035135	-0.360852	7.20E-05	hypomethylated
cg04933530	0.74793	0.582347568	-0.361023	0.000251	hypomethylated
cg07812849	0.81613	0.635434595	-0.361055	9.72E-05	hypomethylated
cg10318148	0.6536	0.508867027	-0.361119	2.64E-06	hypomethylated
cg24174020	0.78093	0.607983243	-0.361162	0.000408	hypomethylated
cg22877504	0.85745	0.66753027	-0.361219	4.05E-05	hypomethylated
cg23517743	0.80339	0.62542973	-0.361253	0.000386	hypomethylated
cg07426848	0.5597	0.435711351	-0.361281	6.01E-05	hypomethylated
cg03979024	0.6441	0.501412432	-0.361287	0.000209	hypomethylated
cg12178904	0.73935	0.575535676	-0.361352	0.000426	hypomethylated
cg26230851	0.86779	0.675495676	-0.361399	1.06E-05	hypomethylated
cg21773633	0.78412	0.610360541	-0.361413	1.89E-05	hypomethylated
cg26532812	0.97967	0.76256973	-0.361427	4.18E-06	hypomethylated
cg18434354	0.57453	0.447209189	-0.361432	0.000445	hypomethylated
cg17738010	0.76508	0.595531351	-0.361433	0.000633	hypomethylated
cg23112821	0.86191	0.670888649	-0.361464	0.00013	hypomethylated
cg22475071	0.80767	0.628659459	-0.361487	0.000195	hypomethylated
cg14070039	0.78068	0.607637838	-0.36152	0.000408	hypomethylated
cg03841376	0.72551	0.564688649	-0.36154	3.07E-05	hypomethylated
cg24462992	0.73084	0.568772432	-0.361704	0.000115	hypomethylated
cg13742513	0.72555	0.564624865	-0.361782	0.000209	hypomethylated
cg23728189	0.75986	0.591322162	-0.361789	9.05E-05	hypomethylated
cg16561256	0.75432	0.587004865	-0.361804	9.16E-05	hypomethylated
cg03553819	0.90007	0.700422162	-0.361812	1.56E-05	hypomethylated
cg02691393	0.8445	0.657122162	-0.361936	8.84E-05	hypomethylated
cg16989784	0.55971	0.435500541	-0.362005	7.03E-05	hypomethylated
cg26595893	0.95219	0.740822703	-0.362121	5.43E-06	hypomethylated
cg27222669	0.74189	0.577194595	-0.362148	0.000117	hypomethylated
cg17357548	0.76077	0.591825946	-0.362287	0.000403	hypomethylated
cg10754941	0.73479	0.571613514	-0.362292	7.52E-06	hypomethylated
cg22027725	0.70758	0.550442162	-0.362302	0.000152	hypomethylated
cg27319971	0.8631	0.671423784	-0.362304	7.65E-05	hypomethylated
cg18167160	0.88745	0.690354595	-0.362328	6.05E-06	hypomethylated
cg15377933	0.90524	0.70416973	-0.362377	3.49E-06	hypomethylated
cg13410000	0.64282	0.500017838	-0.362435	8.13E-05	hypomethylated
cg01789576	0.61843	0.480998919	-0.362577	8.63E-05	hypomethylated
cg18932078	0.79168	0.615716757	-0.362651	1.04E-05	hypomethylated
cg16087432	0.72944	0.567308108	-0.362657	0.000123	hypomethylated
cg24576298	0.66034	0.513561081	-0.362673	0.000575	hypomethylated
cg13683626	0.77123	0.59978973	-0.362704	8.42E-05	hypomethylated
cg17972352	0.84255	0.655230811	-0.362759	1.18E-05	hypomethylated
cg13440206	0.79867	0.621065405	-0.362854	0.000191	hypomethylated
cg18232313	0.88048	0.684658919	-0.362905	0.000129	hypomethylated
cg19031085	0.77907	0.605787027	-0.362942	0.000313	hypomethylated
cg01618928	0.91422	0.710856216	-0.362984	3.90E-06	hypomethylated
cg00124095	0.49885	0.38788	-0.362996	0.000805	hypomethylated
cg05383531	0.77896	0.605668649	-0.363021	0.000365	hypomethylated

cg09658183	0.74347	0.578036757	-0.363113	5.45E-05	hypomethylated
cg01917209	0.81535	0.633884865	-0.363199	0.000219	hypomethylated
cg10962223	0.67205	0.522471351	-0.363217	9.27E-05	hypomethylated
cg26520396	0.80141	0.623038378	-0.363219	5.86E-05	hypomethylated
cg14599823	0.77313	0.601042703	-0.363244	0.000365	hypomethylated
cg26159860	0.72605	0.564436757	-0.363257	9.60E-05	hypomethylated
cg02239377	0.77429	0.601902703	-0.363344	0.001452	hypomethylated
cg20271985	0.7297	0.567236216	-0.363354	7.03E-06	hypomethylated
cg21068293	0.69484	0.540122703	-0.363394	2.89E-05	hypomethylated
cg15661536	0.76919	0.597891892	-0.363455	0.000166	hypomethylated
cg10930101	0.66574	0.51746973	-0.363484	8.03E-05	hypomethylated
cg04505023	0.77574	0.602935676	-0.363569	2.92E-05	hypomethylated
cg01938825	0.81033	0.629792432	-0.363633	0.000204	hypomethylated
cg20847292	0.56009	0.435282162	-0.363708	5.06E-05	hypomethylated
cg00864954	0.69716	0.541795676	-0.363741	0.000178	hypomethylated
cg04996020	0.87555	0.680428108	-0.363747	9.07E-06	hypomethylated
cg18868540	0.84348	0.655467568	-0.363829	8.15E-06	hypomethylated
cg26886066	0.75962	0.590294595	-0.363843	0.000342	hypomethylated
cg08165083	0.84576	0.657227027	-0.363857	0.000109	hypomethylated
cg17799563	0.66205	0.514459459	-0.363883	0.000185	hypomethylated
cg24633312	0.59112	0.459325946	-0.363933	0.000234	hypomethylated
cg13766329	0.8929	0.693802703	-0.363973	2.32E-05	hypomethylated
cg09530438	0.66727	0.51844	-0.364094	0.000417	hypomethylated
cg06043201	0.79039	0.614075135	-0.36415	0.000245	hypomethylated
cg18100564	0.84538	0.656761081	-0.364231	2.51E-05	hypomethylated
cg06760077	0.75096	0.583363243	-0.364342	0.000741	hypomethylated
cg15406343	0.80092	0.62216	-0.364373	2.35E-05	hypomethylated
cg09233429	0.60402	0.469195676	-0.364407	0.001096	hypomethylated
cg25607643	0.73282	0.569232973	-0.36444	0.000293	hypomethylated
cg01767270	0.93109	0.723243243	-0.36444	6.11E-07	hypomethylated
cg23359385	0.63941	0.496662703	-0.364475	0.00032	hypomethylated
cg16070740	0.69897	0.542852973	-0.364669	9.05E-05	hypomethylated
cg01584473	0.75454	0.586003784	-0.364687	0.000134	hypomethylated
cg24475598	0.68563	0.532476757	-0.364712	0.000557	hypomethylated
cg19577080	0.64332	0.499611892	-0.364729	0.000214	hypomethylated
cg07979348	0.70104	0.54440973	-0.364804	4.58E-05	hypomethylated
cg27617780	0.86714	0.673367568	-0.364871	2.35E-05	hypomethylated
cg19009132	0.72216	0.560775676	-0.364895	0.00012	hypomethylated
cg03065165	0.75287	0.584618378	-0.364906	4.47E-05	hypomethylated
cg21418854	0.6967	0.540997838	-0.364915	4.53E-05	hypomethylated
cg07014973	0.66684	0.517792973	-0.364965	0.000104	hypomethylated
cg17583844	0.83773	0.650474595	-0.364993	0.000214	hypomethylated
cg11580790	0.57507	0.446483243	-0.365132	0.000667	hypomethylated
cg10368641	0.66803	0.51864973	-0.365152	0.000613	hypomethylated
cg08006182	0.87977	0.683036216	-0.365164	2.21E-05	hypomethylated
cg22304285	0.86857	0.674321081	-0.365206	1.20E-05	hypomethylated
cg14656153	0.75308	0.584645946	-0.36524	0.000237	hypomethylated
cg01106572	0.74253	0.576445946	-0.365264	0.000893	hypomethylated
cg24145481	0.71093	0.551879459	-0.365354	0.000142	hypomethylated
cg25034766	0.93336	0.724532973	-0.365382	5.65E-05	hypomethylated
cg27370104	0.56982	0.442328108	-0.365389	0.003538	hypomethylated
cg27530053	0.86543	0.671787568	-0.365412	4.15E-05	hypomethylated
cg20467658	0.52623	0.408481081	-0.365424	0.000335	hypomethylated
cg27546012	0.74281	0.576590811	-0.365445	0.00032	hypomethylated
cg24964130	0.89364	0.693524865	-0.365746	5.14E-06	hypomethylated
cg25557280	0.84677	0.657140541	-0.365768	3.00E-05	hypomethylated
cg26538691	0.74551	0.578548649	-0.365789	0.00012	hypomethylated
cg12994591	0.7616	0.591020541	-0.365825	3.07E-05	hypomethylated

cg06219310	0.70448	0.546667027	-0.365896	0.000688	hypomethylated
cg25101291	0.72051	0.55908973	-0.365939	3.57E-05	hypomethylated
cg13911594	0.77456	0.6009	-0.366252	2.71E-05	hypomethylated
cg08387551	0.7703	0.597569189	-0.366315	6.46E-05	hypomethylated
cg22237425	0.67175	0.521083243	-0.366411	0.000607	hypomethylated
cg12083644	0.90671	0.703329189	-0.366441	1.04E-05	hypomethylated
cg14237567	0.76177	0.590876757	-0.366498	0.000119	hypomethylated
cg24904739	0.891	0.691108649	-0.366513	4.00E-05	hypomethylated
cg14303017	0.8077	0.626494595	-0.366517	8.26E-06	hypomethylated
cg25314624	0.86592	0.671652432	-0.366519	2.35E-05	hypomethylated
cg25165975	0.61905	0.480153514	-0.36656	2.71E-05	hypomethylated
cg17240454	0.72332	0.561024324	-0.366571	5.45E-05	hypomethylated
cg06318676	0.80367	0.623337838	-0.366589	1.25E-05	hypomethylated
cg15803671	0.86808	0.673228649	-0.366731	1.99E-05	hypomethylated
cg20268054	0.75773	0.587647568	-0.366733	9.07E-06	hypomethylated
cg25000900	0.80235	0.622197838	-0.366858	0.000187	hypomethylated
cg02577745	0.7586	0.588189189	-0.367059	5.86E-05	hypomethylated
cg08807496	0.8187	0.634781622	-0.367075	3.27E-05	hypomethylated
cg10020787	0.53613	0.415683243	-0.367098	4.05E-05	hypomethylated
cg19760965	0.86371	0.669592973	-0.367263	0.00014	hypomethylated
cg05396243	0.8842	0.68546973	-0.36728	1.17E-05	hypomethylated
cg17243654	0.72319	0.560642703	-0.367293	3.76E-05	hypomethylated
cg15938671	0.913	0.707779459	-0.367315	1.75E-06	hypomethylated
cg02992118	0.69448	0.538370811	-0.367333	1.73E-05	hypomethylated
cg19774788	0.70823	0.54896973	-0.367491	0.000256	hypomethylated
cg23678906	0.82851	0.642195135	-0.367507	5.58E-06	hypomethylated
cg24387800	0.69908	0.541865405	-0.367523	3.32E-05	hypomethylated
cg15519474	0.71235	0.552141081	-0.367549	0.000327	hypomethylated
cg12897502	0.77405	0.599944324	-0.367598	0.000145	hypomethylated
cg03340667	0.82277	0.637682162	-0.367652	8.15E-06	hypomethylated
cg15441656	0.77991	0.604449189	-0.367687	0.000191	hypomethylated
cg18930012	0.58298	0.451822703	-0.36769	7.29E-05	hypomethylated
cg01423643	0.93886	0.727587027	-0.36779	1.99E-06	hypomethylated
cg09244312	0.74984	0.581101081	-0.367794	0.000725	hypomethylated
cg10194536	0.77307	0.5991	-0.367802	0.000613	hypomethylated
cg10187927	0.64008	0.49601027	-0.367882	5.86E-05	hypomethylated
cg00182273	0.58976	0.457012973	-0.367893	4.15E-05	hypomethylated
cg03363633	0.62486	0.484204865	-0.367915	0.001667	hypomethylated
cg17585031	0.56642	0.438868649	-0.368083	0.000224	hypomethylated
cg14353508	0.60908	0.471918378	-0.368094	0.002502	hypomethylated
cg14947787	0.86977	0.673859459	-0.368186	9.27E-05	hypomethylated
cg11375547	0.63537	0.492254595	-0.368192	8.52E-05	hypomethylated
cg01619416	0.72544	0.561996216	-0.368296	0.000117	hypomethylated
cg02153286	0.89743	0.695231892	-0.368305	1.52E-05	hypomethylated
cg14918744	0.7206	0.558240541	-0.368312	3.23E-05	hypomethylated
cg26167249	0.71377	0.552938919	-0.368339	0.000112	hypomethylated
cg19915762	0.52698	0.408221081	-0.368398	2.21E-05	hypomethylated
cg09039163	0.67933	0.526222703	-0.368439	0.000646	hypomethylated
cg08104310	0.6628	0.513416216	-0.368445	0.000234	hypomethylated
cg16543027	0.55075	0.426605946	-0.368494	0.014032	hypomethylated
cg07128624	0.7165	0.554994054	-0.368494	0.000159	hypomethylated
cg10458734	0.87713	0.679377297	-0.368578	6.46E-05	hypomethylated
cg12589308	0.92861	0.719205946	-0.368668	6.54E-05	hypomethylated
cg04865929	0.7843	0.60742	-0.368711	7.84E-05	hypomethylated
cg25545917	0.71736	0.555548649	-0.368784	5.86E-05	hypomethylated
cg09610644	0.80473	0.623210811	-0.368785	5.32E-05	hypomethylated
cg00095629	0.68403	0.529730811	-0.3688	0.000207	hypomethylated
cg14359680	0.63555	0.492185405	-0.368804	0.00305	hypomethylated

cg05808709	0.72089	0.558274595	-0.368804	0.000137	hypomethylated
cg11958234	0.82008	0.635078919	-0.368829	8.94E-05	hypomethylated
cg27174420	0.87825	0.680120541	-0.368841	5.86E-05	hypomethylated
cg11150222	0.81083	0.627907027	-0.368849	0.000102	hypomethylated
cg26060817	0.8291	0.642048649	-0.368864	2.48E-05	hypomethylated
cg05460486	0.90602	0.701586486	-0.368922	6.57E-06	hypomethylated
cg17500202	0.81449	0.630645946	-0.369067	6.93E-06	hypomethylated
cg11736739	0.78925	0.611100541	-0.369073	0.000193	hypomethylated
cg15700850	0.72776	0.563481622	-0.369094	7.32E-06	hypomethylated
cg26948823	0.58022	0.449215676	-0.369192	0.000327	hypomethylated
cg20490031	0.85584	0.66258973	-0.369225	8.63E-05	hypomethylated
cg05684195	0.58315	0.451471892	-0.369231	1.71E-05	hypomethylated
cg06720084	0.86197	0.667324865	-0.369248	2.85E-05	hypomethylated
cg01756899	0.85307	0.66042973	-0.369259	4.47E-05	hypomethylated
cg09869811	0.81909	0.634122162	-0.369261	0.000268	hypomethylated
cg01362515	0.81776	0.633091892	-0.369263	7.93E-06	hypomethylated
cg20061378	0.95381	0.738385946	-0.369327	6.57E-06	hypomethylated
cg00316341	0.77669	0.601254054	-0.369364	0.000219	hypomethylated
cg15806880	0.73162	0.566265405	-0.369616	0.000293	hypomethylated
cg03499943	0.75074	0.581056757	-0.369634	0.000115	hypomethylated
cg07222243	0.57755	0.44700973	-0.36964	3.04E-05	hypomethylated
cg01125010	0.74736	0.578432432	-0.369655	0.000135	hypomethylated
cg02202664	0.83908	0.649407027	-0.369685	9.72E-05	hypomethylated
cg06639267	0.65319	0.505532432	-0.369699	8.13E-05	hypomethylated
cg24212268	0.6981	0.540283243	-0.369718	0.003843	hypomethylated
cg01182620	0.73133	0.565996757	-0.369729	0.000135	hypomethylated
cg03449125	0.74736	0.578361081	-0.369833	0.00012	hypomethylated
cg21810793	0.76906	0.59514973	-0.369843	0.000126	hypomethylated
cg16643542	0.75507	0.584294595	-0.369914	0.000138	hypomethylated
cg05735432	0.75821	0.586688649	-0.370002	6.15E-05	hypomethylated
cg24146288	0.80492	0.622778378	-0.370127	0.000459	hypomethylated
cg01430385	0.68814	0.532392432	-0.370212	5.12E-05	hypomethylated
cg08892386	0.79433	0.614523243	-0.370271	7.47E-05	hypomethylated
cg07875121	0.61095	0.472641622	-0.370308	0.00039	hypomethylated
cg05033749	0.81215	0.62822973	-0.370454	1.25E-05	hypomethylated
cg24461524	0.63178	0.488694054	-0.370491	4.64E-05	hypomethylated
cg07078452	0.81319	0.629012973	-0.370503	1.31E-05	hypomethylated
cg19104757	0.92503	0.715463243	-0.370623	9.57E-06	hypomethylated
cg01471036	0.76488	0.591572973	-0.370677	6.62E-05	hypomethylated
cg06355652	0.75478	0.583752973	-0.370698	0.000107	hypomethylated
cg09530156	0.77575	0.599965946	-0.370711	0.000142	hypomethylated
cg00479718	0.84985	0.657252432	-0.370761	3.15E-05	hypomethylated
cg20239912	0.63103	0.487965946	-0.370928	3.30E-06	hypomethylated
cg04757411	0.84535	0.65364973	-0.371031	7.65E-05	hypomethylated
cg12221970	0.92413	0.714546486	-0.371068	9.69E-06	hypomethylated
cg24382178	0.70868	0.547948649	-0.371094	1.40E-05	hypomethylated
cg00688979	0.76402	0.590728108	-0.371116	9.38E-05	hypomethylated
cg22092397	0.79062	0.61128973	-0.371128	9.95E-05	hypomethylated
cg06627043	0.67213	0.519672973	-0.371136	0.000232	hypomethylated
cg07005770	0.65391	0.505568108	-0.371187	2.21E-05	hypomethylated
cg07086380	0.79653	0.615822162	-0.371215	0.000875	hypomethylated
cg20373634	0.90209	0.697424324	-0.371235	5.81E-06	hypomethylated
cg26965877	0.89586	0.692604324	-0.371242	1.62E-05	hypomethylated
cg15257783	0.56654	0.437984324	-0.371299	0.000245	hypomethylated
cg06230303	0.79395	0.613771351	-0.371347	6.13E-06	hypomethylated
cg14630748	0.83548	0.645851351	-0.371403	1.80E-05	hypomethylated
cg19272754	0.74895	0.578942162	-0.37145	6.22E-06	hypomethylated
cg06748146	0.72874	0.563287568	-0.371533	0.000417	hypomethylated

cg19088634	0.58908	0.455333514	-0.37154	1.54E-05	hypomethylated
cg25790453	0.67074	0.518411892	-0.371655	0.001237	hypomethylated
cg14076161	0.60092	0.464417838	-0.37175	1.12E-05	hypomethylated
cg01287833	0.8712	0.673284324	-0.371788	9.72E-05	hypomethylated
cg03165356	0.72741	0.562082703	-0.371986	0.001188	hypomethylated
cg10628909	0.83396	0.644394595	-0.372034	2.15E-05	hypomethylated
cg06899522	0.8517	0.658053514	-0.37214	0.00014	hypomethylated
cg18040826	0.82118	0.63444973	-0.372193	4.26E-05	hypomethylated
cg27139805	0.76592	0.591748108	-0.372211	1.54E-05	hypomethylated
cg12039583	0.84585	0.6535	-0.372215	0.0002	hypomethylated
cg25836232	0.66309	0.512282162	-0.372266	0.000445	hypomethylated
cg03490200	0.87432	0.675460541	-0.37229	2.57E-05	hypomethylated
cg05490864	0.67276	0.519705405	-0.372398	4.47E-05	hypomethylated
cg06151718	0.85656	0.661685405	-0.372409	2.21E-05	hypomethylated
cg23396177	0.73032	0.564162162	-0.372419	7.29E-05	hypomethylated
cg18046311	0.83659	0.646245405	-0.372439	4.80E-06	hypomethylated
cg00628477	0.60547	0.467684865	-0.372519	8.15E-06	hypomethylated
cg20592317	0.74218	0.573234054	-0.372645	3.49E-06	hypomethylated
cg18009653	0.82004	0.633348649	-0.372694	1.66E-05	hypomethylated
cg26623753	0.56139	0.433581622	-0.3727	0.002231	hypomethylated
cg09503566	0.83197	0.642555676	-0.37271	4.67E-06	hypomethylated
cg09155025	0.76176	0.588235135	-0.372944	0.000163	hypomethylated
cg23873406	0.67313	0.519768108	-0.373017	3.15E-05	hypomethylated
cg08617645	0.61432	0.474340541	-0.373067	8.22E-05	hypomethylated
cg02357046	0.77588	0.599078919	-0.373087	7.93E-06	hypomethylated
cg00168694	0.78874	0.608995676	-0.373118	0.001164	hypomethylated
cg16001335	0.63836	0.492883784	-0.373123	0.000327	hypomethylated
cg18836689	0.71427	0.551490811	-0.373133	0.000219	hypomethylated
cg08387270	0.80443	0.621036216	-0.373289	2.64E-06	hypomethylated
cg11624993	0.75806	0.58523027	-0.373308	3.57E-05	hypomethylated
cg08140055	0.68379	0.527890811	-0.373314	6.62E-05	hypomethylated
cg09558069	0.73099	0.564308649	-0.373367	0.000229	hypomethylated
cg24083746	0.49331	0.380822703	-0.373375	0.000435	hypomethylated
cg10183885	0.83184	0.642157297	-0.373379	9.95E-05	hypomethylated
cg15829116	0.57902	0.446962162	-0.37346	3.67E-05	hypomethylated
cg16705300	0.89506	0.690913514	-0.373479	3.23E-05	hypomethylated
cg03882071	0.64589	0.498568649	-0.373496	0.000296	hypomethylated
cg14355939	0.81525	0.629297297	-0.373501	7.93E-06	hypomethylated
cg19772651	0.96044	0.741358919	-0.373523	3.69E-06	hypomethylated
cg24323726	0.7346	0.567022703	-0.373552	0.00101	hypomethylated
cg13166171	0.62318	0.481009189	-0.373584	7.84E-05	hypomethylated
cg12237269	0.70693	0.545648649	-0.373595	2.27E-05	hypomethylated
cg24544047	0.6892	0.531955135	-0.373618	0.001968	hypomethylated
cg02232529	0.82067	0.63342	-0.37364	1.14E-05	hypomethylated
cg06538426	0.74522	0.575166486	-0.373687	7.93E-05	hypomethylated
cg08464513	0.79639	0.614558919	-0.373924	0.00024	hypomethylated
cg10517281	0.6103	0.470952973	-0.373936	3.15E-05	hypomethylated
cg11093872	0.82209	0.634351351	-0.374014	8.83E-06	hypomethylated
cg01283141	0.78359	0.604634595	-0.374035	1.18E-05	hypomethylated
cg05751310	0.76277	0.58854973	-0.374084	1.99E-06	hypomethylated
cg03833948	0.82409	0.635847027	-0.374122	3.32E-05	hypomethylated
cg15882394	0.81689	0.630278919	-0.374151	0.000234	hypomethylated
cg07200421	0.86972	0.671001081	-0.374236	4.15E-05	hypomethylated
cg05044414	0.70997	0.547747568	-0.374247	0.000327	hypomethylated
cg26656884	0.78365	0.604547568	-0.374354	8.83E-06	hypomethylated
cg14893129	0.84597	0.652615676	-0.374373	1.18E-05	hypomethylated
cg09811127	0.68223	0.526288649	-0.374404	0.000335	hypomethylated
cg23441018	0.74488	0.574575676	-0.374511	0.00064	hypomethylated

cg23033158	0.85486	0.659341622	-0.374662	1.12E-05	hypomethylated
cg17029694	0.53825	0.415137838	-0.374686	2.85E-05	hypomethylated
cg15692837	0.5519	0.425661081	-0.374702	0.000354	hypomethylated
cg16444062	0.91042	0.702111351	-0.374832	9.95E-05	hypomethylated
cg25752514	0.74977	0.578205946	-0.374865	1.25E-05	hypomethylated
cg11505841	0.91045	0.70210973	-0.374883	8.95E-06	hypomethylated
cg18991321	0.62563	0.482458919	-0.374904	0.000191	hypomethylated
cg08011941	0.83306	0.642419459	-0.374905	1.04E-05	hypomethylated
cg13381679	0.8424	0.649571351	-0.375017	1.06E-05	hypomethylated
cg03497895	0.87729	0.676345946	-0.375292	2.45E-05	hypomethylated
cg06589239	0.78579	0.605751892	-0.375417	0.002455	hypomethylated
cg05771157	0.88945	0.685653514	-0.375434	1.67E-06	hypomethylated
cg04705318	0.80869	0.623395135	-0.37544	2.92E-05	hypomethylated
cg05659486	0.68781	0.530211351	-0.375443	0.000116	hypomethylated
cg14280181	0.78225	0.603011892	-0.375443	0.000399	hypomethylated
cg20377232	0.65937	0.508282703	-0.375457	0.000474	hypomethylated
cg00566164	0.92953	0.716534054	-0.375466	2.71E-06	hypomethylated
cg20698113	0.69406	0.535006486	-0.375504	0.000145	hypomethylated
cg16555466	0.81102	0.625146486	-0.375543	8.13E-05	hypomethylated
cg11245181	0.80013	0.616747568	-0.375554	0.000152	hypomethylated
cg26852857	0.88206	0.679896216	-0.375562	0.000123	hypomethylated
cg14446406	0.77794	0.599637297	-0.375569	1.94E-05	hypomethylated
cg13471735	0.81203	0.625907027	-0.375585	0.00017	hypomethylated
cg00392257	0.55985	0.431494054	-0.3757	4.00E-05	hypomethylated
cg13423274	0.85334	0.657693514	-0.375705	7.52E-06	hypomethylated
cg14176942	0.874	0.673605405	-0.37573	1.94E-05	hypomethylated
cg03226872	0.76986	0.593324865	-0.375774	2.78E-05	hypomethylated
cg08676510	0.64505	0.497124865	-0.375803	0.000588	hypomethylated
cg26808637	0.86228	0.664530811	-0.37582	2.30E-05	hypomethylated
cg18178189	0.81239	0.626072432	-0.375843	2.85E-05	hypomethylated
cg04007890	0.78229	0.602849189	-0.375906	1.12E-05	hypomethylated
cg11601375	0.77226	0.595119459	-0.375907	0.000166	hypomethylated
cg01390445	0.9137	0.704103243	-0.375934	2.89E-05	hypomethylated
cg09439722	0.66577	0.513007568	-0.376044	5.38E-05	hypomethylated
cg26159990	0.7218	0.556167027	-0.376081	0.000159	hypomethylated
cg11735008	0.84214	0.648891351	-0.376083	1.58E-05	hypomethylated
cg07475178	0.92836	0.715312973	-0.37611	0.000511	hypomethylated
cg21908886	0.7464	0.575105405	-0.376123	0.000182	hypomethylated
cg00243854	0.71409	0.5502	-0.37615	0.000313	hypomethylated
cg13963044	0.68157	0.525128649	-0.376191	0.00024	hypomethylated
cg01991934	0.5526	0.425758919	-0.376199	4.31E-05	hypomethylated
cg22591889	0.8035	0.619035676	-0.376275	3.85E-05	hypomethylated
cg06149671	0.88172	0.679290811	-0.376291	8.71E-06	hypomethylated
cg07959609	0.72532	0.558768108	-0.376368	0.000102	hypomethylated
cg24180759	0.78409	0.604041622	-0.376371	0.00028	hypomethylated
cg23805594	0.8587	0.661512432	-0.376386	1.75E-05	hypomethylated
cg00351750	0.80419	0.619430811	-0.376593	0.000234	hypomethylated
cg22646210	0.62623	0.482351892	-0.376607	0.000168	hypomethylated
cg05345310	0.83817	0.645590811	-0.376623	0.000129	hypomethylated
cg06144311	0.5984	0.460906486	-0.376636	7.12E-05	hypomethylated
cg18200075	0.66182	0.509736216	-0.376688	0.000358	hypomethylated
cg17836612	0.52396	0.403528108	-0.376788	0.000145	hypomethylated
cg00328720	0.85437	0.657966486	-0.376847	0.000212	hypomethylated
cg02104392	0.75102	0.578368649	-0.376862	0.000104	hypomethylated
cg09148608	0.77664	0.598076757	-0.376915	5.86E-05	hypomethylated
cg24768463	0.56738	0.43692	-0.376946	0.000126	hypomethylated
cg04208928	0.80478	0.619731351	-0.376951	0.000145	hypomethylated
cg16722931	0.73797	0.568238919	-0.377065	9.49E-05	hypomethylated

cg25221919	0.71874	0.553427027	-0.377077	4.26E-05	hypomethylated
cg25954705	0.75398	0.580547568	-0.377112	7.72E-06	hypomethylated
cg21582831	0.61901	0.476606486	-0.377164	0.002189	hypomethylated
cg17476101	0.86387	0.665031892	-0.377391	9.44E-06	hypomethylated
cg03224650	0.85958	0.661711892	-0.377429	1.18E-05	hypomethylated
cg10759972	0.74084	0.570294054	-0.377456	0.001031	hypomethylated
cg19867250	0.65293	0.502615676	-0.377473	0.000124	hypomethylated
cg18030943	0.70852	0.545406486	-0.377477	0.015685	hypomethylated
cg13753946	0.85532	0.658378919	-0.377546	4.82E-05	hypomethylated
cg00240178	0.73959	0.569295135	-0.377549	0.000613	hypomethylated
cg19600528	0.82512	0.635118378	-0.377578	1.39E-05	hypomethylated
cg13500113	0.75441	0.58069027	-0.37758	2.27E-05	hypomethylated
cg14815183	0.77989	0.600285405	-0.377622	1.39E-05	hypomethylated
cg22633936	0.73844	0.568380541	-0.377624	0.000219	hypomethylated
cg18195165	0.82372	0.634008108	-0.377653	9.82E-06	hypomethylated
cg22797169	0.72777	0.560155676	-0.377655	0.000469	hypomethylated
cg10063663	0.89496	0.688839459	-0.377655	7.12E-05	hypomethylated
cg11348165	0.70681	0.544011351	-0.377686	7.74E-05	hypomethylated
cg16628205	0.83724	0.644396757	-0.377692	1.21E-05	hypomethylated
cg01675238	0.60197	0.463302162	-0.377738	0.01109	hypomethylated
cg13689699	0.68043	0.523676216	-0.377772	0.00064	hypomethylated
cg14694075	0.58661	0.451460541	-0.377802	0.000202	hypomethylated
cg07317843	0.79966	0.615416216	-0.377824	0.00029	hypomethylated
cg13053992	0.69452	0.534491351	-0.37785	0.000168	hypomethylated
cg00818899	0.83262	0.640753514	-0.377889	5.06E-05	hypomethylated
cg24781699	0.7238	0.556990811	-0.377938	0.003078	hypomethylated
cg19819654	0.95656	0.736107568	-0.377939	7.93E-06	hypomethylated
cg12095505	0.58849	0.452858378	-0.377958	4.15E-05	hypomethylated
cg20066782	0.88592	0.681714595	-0.378009	1.66E-05	hypomethylated
cg13790576	0.60141	0.462775135	-0.378038	0.000287	hypomethylated
cg07661904	0.9154	0.704375135	-0.378058	0.000135	hypomethylated
cg07192961	0.85874	0.660775135	-0.378062	9.72E-05	hypomethylated
cg05996573	0.78757	0.606006486	-0.378075	1.92E-05	hypomethylated
cg23697467	0.76787	0.590844865	-0.378083	4.36E-05	hypomethylated
cg25287149	0.89011	0.684894595	-0.378102	9.57E-06	hypomethylated
cg15206445	0.60725	0.467246486	-0.378107	0.00094	hypomethylated
cg11152825	0.86205	0.663285946	-0.378141	1.28E-05	hypomethylated
cg14424963	0.64643	0.497331351	-0.378287	0.003986	hypomethylated
cg16900102	0.65126	0.501041081	-0.378305	0.000274	hypomethylated
cg22357390	0.78833	0.606467027	-0.37837	3.07E-05	hypomethylated
cg21083556	0.81642	0.628071351	-0.378383	1.40E-05	hypomethylated
cg06218344	0.6223	0.478731892	-0.378392	0.000528	hypomethylated
cg00034755	0.78679	0.605171892	-0.378634	0.000143	hypomethylated
cg18129996	0.84179	0.647466486	-0.378655	0.000142	hypomethylated
cg17017284	0.62432	0.480192432	-0.378673	8.22E-05	hypomethylated
cg16703312	0.69543	0.534878378	-0.378694	7.65E-05	hypomethylated
cg01808969	0.82992	0.638314054	-0.378706	8.03E-05	hypomethylated
cg22916017	0.90112	0.69307027	-0.378718	0.000569	hypomethylated
cg13820776	0.74978	0.576658378	-0.37875	1.18E-06	hypomethylated
cg25517015	0.88044	0.677134054	-0.378783	5.72E-05	hypomethylated
cg20230416	0.80242	0.617125405	-0.378794	3.54E-06	hypomethylated
cg26720010	0.87305	0.671441622	-0.378802	3.39E-06	hypomethylated
cg13100137	0.69939	0.537883784	-0.378803	4.76E-05	hypomethylated
cg10234693	0.51199	0.393747027	-0.378847	0.008069	hypomethylated
cg17921439	0.71715	0.551514595	-0.378876	0.000166	hypomethylated
cg09361748	0.58069	0.446552973	-0.378937	0.000277	hypomethylated
cg16357353	0.5179	0.398255135	-0.378981	0.000335	hypomethylated
cg20765441	0.62597	0.481358378	-0.378982	0.000741	hypomethylated

cg00250483	0.60839	0.467835676	-0.378995	0.000102	hypomethylated
cg06725798	0.79943	0.614721081	-0.37904	2.10E-05	hypomethylated
cg10727432	0.8252	0.634510811	-0.379099	6.13E-06	hypomethylated
cg07636761	0.66625	0.512230811	-0.37927	0.000283	hypomethylated
cg09690632	0.83661	0.643208108	-0.37927	2.36E-06	hypomethylated
cg15082824	0.71699	0.551223784	-0.379315	0.000209	hypomethylated
cg17422915	0.79411	0.610511351	-0.379321	8.63E-05	hypomethylated
cg18389931	0.73274	0.563285405	-0.379435	1.66E-05	hypomethylated
cg04629204	0.59053	0.453960541	-0.379443	0.000209	hypomethylated
cg13720262	0.81604	0.627307568	-0.379467	0.000123	hypomethylated
cg26462055	0.83293	0.640287568	-0.379475	0.000142	hypomethylated
cg18599081	0.67339	0.517635676	-0.379505	0.003195	hypomethylated
cg03752885	0.7118	0.547159459	-0.379511	0.00096	hypomethylated
cg22876402	0.82162	0.631574054	-0.379519	3.95E-05	hypomethylated
cg02821871	0.84071	0.646247568	-0.379521	0.000103	hypomethylated
cg16413327	0.85629	0.658217297	-0.379536	1.52E-05	hypomethylated
cg20793420	0.451	0.346672973	-0.379552	0.00134	hypomethylated
cg10407585	0.81354	0.625303784	-0.379656	3.15E-05	hypomethylated
cg13476123	0.72329	0.555923243	-0.379689	6.01E-05	hypomethylated
cg05255580	0.79638	0.612095676	-0.3797	3.04E-06	hypomethylated
cg04344000	0.75266	0.578491351	-0.379703	0.000122	hypomethylated
cg01532749	0.74398	0.571785405	-0.37979	7.38E-05	hypomethylated
cg27617775	0.81046	0.622868649	-0.379813	2.10E-05	hypomethylated
cg21545720	0.65885	0.506341622	-0.379839	1.35E-05	hypomethylated
cg07059469	0.64317	0.494281622	-0.379867	0.001354	hypomethylated
cg12533496	0.5151	0.395851351	-0.379894	6.62E-05	hypomethylated
cg22602105	0.62139	0.477524865	-0.379923	0.00049	hypomethylated
cg00409861	0.86638	0.665738919	-0.380043	2.15E-05	hypomethylated
cg12797987	0.83785	0.643798378	-0.380083	5.12E-05	hypomethylated
cg15068709	0.82871	0.636774054	-0.380086	2.18E-05	hypomethylated
cg10968815	0.79591	0.611551351	-0.380132	2.89E-05	hypomethylated
cg14612133	0.79973	0.614465946	-0.38018	9.31E-06	hypomethylated
cg26334507	0.8016	0.615867027	-0.380264	0.000138	hypomethylated
cg11816838	0.85498	0.656878378	-0.380264	5.43E-06	hypomethylated
cg25881535	0.66998	0.514727568	-0.380309	0.000189	hypomethylated
cg03637066	0.74036	0.568795676	-0.380316	6.62E-05	hypomethylated
cg26761744	0.85086	0.653688108	-0.380319	4.82E-05	hypomethylated
cg23051970	0.89061	0.68419027	-0.380396	9.96E-06	hypomethylated
cg09491901	0.78299	0.601475676	-0.380487	6.78E-05	hypomethylated
cg04903159	0.4906	0.376866486	-0.380494	0.000523	hypomethylated
cg00510330	0.81604	0.626848649	-0.380523	0.000142	hypomethylated
cg25343204	0.888	0.68212	-0.380534	3.00E-05	hypomethylated
cg16678159	0.71589	0.549911892	-0.380537	0.000293	hypomethylated
cg01806427	0.79243	0.608673514	-0.380615	0.000426	hypomethylated
cg17412005	0.62411	0.479384324	-0.380618	0.001031	hypomethylated
cg23322223	0.85186	0.654321081	-0.380618	3.04E-05	hypomethylated
cg27386326	0.85867	0.659511351	-0.380706	7.29E-05	hypomethylated
cg14373988	0.89667	0.688693514	-0.380715	5.06E-05	hypomethylated
cg09231675	0.87641	0.673131892	-0.380717	1.49E-06	hypomethylated
cg06534586	0.90101	0.692006486	-0.380758	7.12E-06	hypomethylated
cg01055129	0.82714	0.635271351	-0.380759	1.50E-05	hypomethylated
cg20124610	0.54541	0.418892432	-0.380761	1.52E-05	hypomethylated
cg00789198	0.77191	0.592831892	-0.38081	8.63E-05	hypomethylated
cg23087707	0.91676	0.704066486	-0.380832	4.48E-06	hypomethylated
cg05708497	0.62792	0.482228108	-0.380865	6.93E-06	hypomethylated
cg06927337	0.76317	0.58602973	-0.381031	7.12E-05	hypomethylated
cg09049451	0.9132	0.701210811	-0.381083	9.38E-07	hypomethylated
cg21449569	0.84091	0.645701081	-0.381085	4.26E-05	hypomethylated

cg26680675	0.84728	0.650571892	-0.38113	1.92E-05	hypomethylated
cg25215890	0.60423	0.463944865	-0.381144	0.000545	hypomethylated
cg03255379	0.83373	0.640144865	-0.381182	5.73E-06	hypomethylated
cg14485901	0.78678	0.604093514	-0.381188	3.07E-05	hypomethylated
cg24450508	0.71516	0.549077297	-0.381257	0.00097	hypomethylated
cg26034934	0.60636	0.465518378	-0.381336	8.42E-05	hypomethylated
cg11307623	0.65504	0.502883243	-0.38136	6.93E-06	hypomethylated
cg00578614	0.76326	0.585917838	-0.381476	0.001031	hypomethylated
cg26748297	0.84062	0.645218378	-0.381666	5.43E-06	hypomethylated
cg04528382	0.69775	0.535558378	-0.381666	1.71E-05	hypomethylated
cg05367967	0.89037	0.683381081	-0.381715	7.65E-05	hypomethylated
cg04450003	0.76404	0.586413514	-0.38173	0.000313	hypomethylated
cg15122966	0.61877	0.474904324	-0.381766	0.000191	hypomethylated
cg13275679	0.74002	0.567942703	-0.381819	1.46E-05	hypomethylated
cg25080973	0.71758	0.550712973	-0.381839	4.36E-05	hypomethylated
cg06765219	0.70114	0.538092973	-0.381847	0.0003	hypomethylated
cg09490007	0.70931	0.544342703	-0.381901	1.58E-05	hypomethylated
cg24080086	0.79842	0.612725946	-0.381906	1.15E-05	hypomethylated
cg09550697	0.94775	0.727318378	-0.38192	7.62E-06	hypomethylated
cg01999046	0.85122	0.653174595	-0.382063	0.000142	hypomethylated
cg09110274	0.79903	0.613124865	-0.382069	1.12E-05	hypomethylated
cg15114431	0.86407	0.663028649	-0.382077	1.46E-05	hypomethylated
cg18565795	0.90506	0.694465405	-0.382111	7.52E-06	hypomethylated
cg03678062	0.67998	0.521714054	-0.382233	0.001074	hypomethylated
cg27212428	0.75337	0.577972432	-0.382358	4.36E-05	hypomethylated
cg24314434	0.71132	0.545694054	-0.382406	5.58E-05	hypomethylated
cg20951444	0.70715	0.542478378	-0.382451	2.92E-05	hypomethylated
cg24282479	0.8361	0.641396757	-0.382458	5.86E-05	hypomethylated
cg00284420	0.97098	0.744845946	-0.3825	3.44E-06	hypomethylated
cg18983132	0.73815	0.566215135	-0.382564	0.000613	hypomethylated
cg08856033	0.57304	0.439548108	-0.382615	0.000163	hypomethylated
cg11401820	0.86699	0.664969189	-0.382728	7.22E-06	hypomethylated
cg11580615	0.86282	0.661757838	-0.382756	1.01E-05	hypomethylated
cg11056055	0.87769	0.673149189	-0.382785	1.89E-05	hypomethylated
cg01886524	0.66811	0.512405405	-0.3828	3.23E-05	hypomethylated
cg19902593	0.74088	0.568200541	-0.38284	0.000165	hypomethylated
cg02520399	0.89305	0.684868108	-0.382915	4.48E-06	hypomethylated
cg26433494	0.76222	0.584523243	-0.382947	1.35E-05	hypomethylated
cg08800670	0.56759	0.435255135	-0.382988	6.95E-05	hypomethylated
cg21669226	0.90321	0.692616216	-0.383005	3.21E-06	hypomethylated
cg27144368	0.72798	0.558229189	-0.383041	0.000528	hypomethylated
cg06768670	0.61128	0.468724865	-0.383092	0.001368	hypomethylated
cg11585357	0.91046	0.69813027	-0.383099	2.21E-05	hypomethylated
cg27050612	0.52582	0.403181081	-0.383141	0.001327	hypomethylated
cg19554281	0.79076	0.606315135	-0.383172	0.000195	hypomethylated
cg15206981	0.82169	0.630022703	-0.38319	2.24E-05	hypomethylated
cg06074534	0.9389	0.719881081	-0.383213	3.15E-05	hypomethylated
cg16650212	0.74218	0.569045946	-0.383224	4.20E-05	hypomethylated
cg27528222	0.74386	0.57033027	-0.383234	0.000262	hypomethylated
cg26588061	0.76449	0.586092973	-0.383368	0.000674	hypomethylated
cg12899248	0.85374	0.654482703	-0.383442	1.25E-05	hypomethylated
cg12821315	0.57557	0.441196216	-0.383571	3.49E-05	hypomethylated
cg01503938	0.69209	0.530510811	-0.383578	1.05E-05	hypomethylated
cg06047270	0.51136	0.391963784	-0.383619	0.006451	hypomethylated
cg13856405	0.69622	0.533645405	-0.383662	1.89E-05	hypomethylated
cg09578155	0.53507	0.41012	-0.383682	0.002231	hypomethylated
cg02066184	0.85216	0.653148108	-0.383714	7.93E-05	hypomethylated
cg26144734	0.75405	0.577905405	-0.383827	2.36E-06	hypomethylated

cg06966613	0.70304	0.53879027	-0.383883	1.75E-05	hypomethylated
cg13563390	0.81353	0.623438378	-0.383949	6.86E-05	hypomethylated
cg14711976	0.72474	0.555383784	-0.383978	0.000313	hypomethylated
cg27409154	0.69571	0.533133514	-0.383989	0.000174	hypomethylated
cg20443357	0.80455	0.616515135	-0.384046	4.24E-06	hypomethylated
cg16806195	0.57073	0.437341081	-0.38405	0.000104	hypomethylated
cg22469948	0.59139	0.453164324	-0.384076	8.22E-05	hypomethylated
cg06818582	0.72675	0.556857297	-0.384152	1.15E-05	hypomethylated
cg09282946	0.78954	0.604910811	-0.38429	4.36E-06	hypomethylated
cg03953069	0.51861	0.397311351	-0.38438	0.000262	hypomethylated
cg14200470	0.6632	0.508052432	-0.384467	1.56E-05	hypomethylated
cg02723964	0.75725	0.5801	-0.384468	3.40E-05	hypomethylated
cg14497203	0.78684	0.602758378	-0.384491	3.90E-05	hypomethylated
cg08411049	0.73595	0.56376973	-0.384502	2.45E-05	hypomethylated
cg15849872	0.50609	0.387679459	-0.38453	3.40E-05	hypomethylated
cg18593210	0.71655	0.548885946	-0.384561	3.79E-06	hypomethylated
cg14568203	0.82918	0.635144865	-0.3846	4.36E-06	hypomethylated
cg13027727	0.5443	0.416928108	-0.384603	4.53E-05	hypomethylated
cg11426170	0.72079	0.552117297	-0.384604	0.000378	hypomethylated
cg14976391	0.62126	0.475872973	-0.384621	0.000287	hypomethylated
cg11228744	0.75698	0.579825946	-0.384635	0.000132	hypomethylated
cg17742559	0.66217	0.507196216	-0.384658	0.000681	hypomethylated
cg25840318	0.84858	0.649967568	-0.384683	7.29E-05	hypomethylated
cg07795964	0.96115	0.736153514	-0.384755	3.54E-06	hypomethylated
cg16723982	0.64141	0.491260541	-0.384758	0.000435	hypomethylated
cg11261264	0.90732	0.69490973	-0.384786	1.09E-05	hypomethylated
cg25797454	0.70997	0.543758919	-0.384791	0.000129	hypomethylated
cg00411358	0.78016	0.597499459	-0.384833	2.04E-05	hypomethylated
cg09193477	0.87306	0.668642162	-0.384846	9.27E-05	hypomethylated
cg15647617	0.57257	0.438505405	-0.384857	0.00035	hypomethylated
cg23024102	0.63564	0.486785405	-0.384924	8.42E-05	hypomethylated
cg04802386	0.86875	0.665299459	-0.384937	6.31E-05	hypomethylated
cg18761763	0.82315	0.630354054	-0.384993	1.54E-05	hypomethylated
cg07064720	0.61985	0.474657838	-0.385031	0.00029	hypomethylated
cg08203210	0.7935	0.607614054	-0.385075	6.01E-05	hypomethylated
cg19244553	0.90194	0.690650811	-0.385075	3.40E-05	hypomethylated
cg07855572	0.5831	0.446494595	-0.385101	3.67E-05	hypomethylated
cg24570042	0.61697	0.472419459	-0.385132	0.002432	hypomethylated
cg14151317	0.91968	0.70416	-0.385229	5.00E-06	hypomethylated
cg04737185	0.89959	0.68876	-0.385266	2.10E-05	hypomethylated
cg00470341	0.79557	0.609056757	-0.385412	0.000626	hypomethylated
cg10635092	0.62624	0.479422703	-0.385417	0.000361	hypomethylated
cg11220043	0.893	0.683642703	-0.385418	8.60E-06	hypomethylated
cg12801391	0.82519	0.631722162	-0.385436	2.27E-05	hypomethylated
cg21518261	0.77331	0.591962162	-0.385542	8.95E-06	hypomethylated
cg12434312	0.48849	0.373927027	-0.385572	0.000417	hypomethylated
cg07759857	0.86613	0.66296973	-0.385641	2.27E-05	hypomethylated
cg01792640	0.90451	0.692343243	-0.385649	1.05E-05	hypomethylated
cg03612692	0.61432	0.470212432	-0.385678	4.58E-05	hypomethylated
cg26910384	0.61783	0.47288	-0.385736	4.26E-05	hypomethylated
cg01461211	0.60845	0.465683243	-0.38579	7.47E-05	hypomethylated
cg09644662	0.82477	0.631212973	-0.385865	9.95E-05	hypomethylated
cg12989498	0.78068	0.597439459	-0.385939	3.00E-05	hypomethylated
cg26528298	0.76215	0.583252432	-0.385955	2.27E-05	hypomethylated
cg12967723	0.6011	0.459995135	-0.385986	0.000369	hypomethylated
cg25844590	0.85402	0.653543243	-0.385987	2.71E-05	hypomethylated
cg14418857	0.71847	0.549793514	-0.386038	0.000718	hypomethylated
cg01054938	0.75473	0.577521081	-0.386087	0.000187	hypomethylated

cg13817395	0.58371	0.446627027	-0.386181	0.000123	hypomethylated
cg02512902	0.69972	0.535387027	-0.386196	0.00134	hypomethylated
cg08616234	0.84021	0.642877297	-0.386207	2.71E-05	hypomethylated
cg09142858	0.6259	0.478888649	-0.386242	2.87E-06	hypomethylated
cg03249047	0.64165	0.490937838	-0.386246	0.000106	hypomethylated
cg23382741	0.86075	0.658558378	-0.386283	8.37E-06	hypomethylated
cg15006627	0.62928	0.481456757	-0.386296	2.45E-05	hypomethylated
cg23517752	0.71341	0.545805405	-0.386345	2.71E-05	hypomethylated
cg21026469	0.91824	0.702500541	-0.386372	2.13E-05	hypomethylated
cg15863552	0.9164	0.70108973	-0.386378	0.000104	hypomethylated
cg11240431	0.69583	0.532327027	-0.386422	2.27E-05	hypomethylated
cg05626927	0.73204	0.560015135	-0.386457	0.000262	hypomethylated
cg16447311	0.81297	0.621885405	-0.386553	0.000132	hypomethylated
cg13168117	0.76451	0.584775676	-0.386652	9.05E-05	hypomethylated
cg04617606	0.76007	0.581377838	-0.386656	0.000135	hypomethylated
cg19911494	0.85516	0.654052973	-0.386787	1.99E-05	hypomethylated
cg01182154	0.87327	0.667901081	-0.386793	8.83E-06	hypomethylated
cg01629435	0.75251	0.575511892	-0.386865	0.000219	hypomethylated
cg01290136	0.76481	0.584904324	-0.386901	6.31E-05	hypomethylated
cg00302479	0.55501	0.424452973	-0.386909	0.001949	hypomethylated
cg10921168	0.69485	0.531354054	-0.387028	0.000148	hypomethylated
cg03723321	0.64811	0.495590811	-0.387089	2.85E-05	hypomethylated
cg15892280	0.61872	0.473112973	-0.387102	0.001395	hypomethylated
cg11684549	0.79061	0.604513514	-0.387192	5.19E-05	hypomethylated
cg22083047	0.75126	0.574420541	-0.387205	0.000234	hypomethylated
cg09123615	0.78032	0.59664	-0.387205	2.42E-06	hypomethylated
cg02901644	0.68587	0.524405405	-0.387253	0.000374	hypomethylated
cg02246725	0.7176	0.548656216	-0.387277	0.000138	hypomethylated
cg19554257	0.62061	0.474491351	-0.387305	0.003225	hypomethylated
cg11721177	0.5512	0.421384324	-0.387439	0.000262	hypomethylated
cg13167431	0.60631	0.463514054	-0.387443	0.000822	hypomethylated
cg12748639	0.58801	0.449511351	-0.387483	0.000395	hypomethylated
cg05468458	0.62752	0.479708649	-0.387503	0.000142	hypomethylated
cg14503815	0.81663	0.62426973	-0.387513	5.45E-05	hypomethylated
cg05997730	0.56702	0.433450811	-0.387531	0.000107	hypomethylated
cg23907852	0.58742	0.449031892	-0.387574	2.71E-05	hypomethylated
cg17315844	0.89941	0.687502703	-0.387614	2.38E-05	hypomethylated
cg19771372	0.37479	0.286484865	-0.387624	6.46E-05	hypomethylated
cg08644381	0.66301	0.506794595	-0.387629	3.15E-05	hypomethylated
cg00600454	0.6545	0.500271892	-0.387681	0.000116	hypomethylated
cg24446429	0.81201	0.620622703	-0.387781	0.000135	hypomethylated
cg07204711	0.75763	0.579055135	-0.387793	2.15E-05	hypomethylated
cg23473088	0.88632	0.677399459	-0.387821	5.58E-06	hypomethylated
cg21341520	0.53547	0.40922	-0.387929	0.000198	hypomethylated
cg00160478	0.62569	0.478158378	-0.387959	3.15E-05	hypomethylated
cg10018933	0.74786	0.571504865	-0.388002	0.000306	hypomethylated
cg18504989	0.68592	0.524160541	-0.388032	1.06E-05	hypomethylated
cg06209035	0.77987	0.595945405	-0.388053	3.95E-05	hypomethylated
cg25976170	0.80215	0.612961622	-0.388075	0.000106	hypomethylated
cg08466082	0.87233	0.666536757	-0.38819	3.85E-05	hypomethylated
cg04643397	0.82566	0.63084	-0.388274	6.46E-05	hypomethylated
cg05301124	0.85852	0.655939459	-0.388289	4.12E-06	hypomethylated
cg09985802	0.61488	0.469762162	-0.388374	9.27E-05	hypomethylated
cg14327296	0.5463	0.417334054	-0.388491	0.00099	hypomethylated
cg13917589	0.907	0.692875676	-0.388506	9.31E-06	hypomethylated
cg04976075	0.5642	0.430994595	-0.388537	5.31E-05	hypomethylated
cg01504030	0.83033	0.634287027	-0.388549	5.58E-05	hypomethylated
cg26337559	0.90593	0.692035676	-0.388553	5.93E-05	hypomethylated

cg21865249	0.65183	0.497907027	-0.388619	0.000274	hypomethylated
cg15827285	0.7461	0.569892432	-0.388679	0.000633	hypomethylated
cg09414673	0.64493	0.492611892	-0.388691	0.000219	hypomethylated
cg00264129	0.77391	0.591124324	-0.388704	0.000123	hypomethylated
cg22862656	0.75148	0.573982162	-0.388729	0.000189	hypomethylated
cg18345635	0.55664	0.425123243	-0.388863	0.00421	hypomethylated
cg10372302	0.79507	0.607200541	-0.388909	5.12E-05	hypomethylated
cg13204236	0.70867	0.541208108	-0.388931	0.000335	hypomethylated
cg16906765	0.54243	0.414243243	-0.388959	0.000138	hypomethylated
cg02169981	0.83857	0.640378919	-0.389005	6.93E-06	hypomethylated
cg16048817	0.74415	0.568252432	-0.389062	7.52E-06	hypomethylated
cg05757448	0.84134	0.642465405	-0.38907	6.15E-05	hypomethylated
cg02205193	0.85227	0.650805405	-0.389084	2.27E-05	hypomethylated
cg18699466	0.789	0.602471351	-0.389133	2.89E-05	hypomethylated
cg22830638	0.80531	0.61486973	-0.389263	5.14E-06	hypomethylated
cg07635132	0.6899	0.526733514	-0.389314	0.000435	hypomethylated
cg09373676	0.88062	0.672326486	-0.389358	6.62E-05	hypomethylated
cg06240652	0.80655	0.615736757	-0.38945	3.21E-06	hypomethylated
cg00917471	0.65092	0.496918919	-0.38947	0.00099	hypomethylated
cg13811955	0.65407	0.499320541	-0.389479	3.15E-05	hypomethylated
cg01742357	0.56617	0.432214054	-0.389489	0.000464	hypomethylated
cg11300809	0.43117	0.329154595	-0.389491	0.000283	hypomethylated
cg06900571	0.89141	0.680472973	-0.389551	1.50E-05	hypomethylated
cg21092021	0.69243	0.528555676	-0.389613	2.89E-05	hypomethylated
cg18010085	0.85824	0.655102703	-0.38966	1.99E-05	hypomethylated
cg19358608	0.73561	0.561495676	-0.389666	0.001839	hypomethylated
cg25042258	0.72467	0.55312973	-0.389706	1.56E-05	hypomethylated
cg26019472	0.79811	0.609175676	-0.389729	0.000726	hypomethylated
cg05302489	0.83712	0.638936216	-0.389763	6.84E-06	hypomethylated
cg19471433	0.72298	0.551817297	-0.389765	0.000313	hypomethylated
cg25574111	0.52704	0.402246486	-0.389833	0.000342	hypomethylated
cg10622536	0.83339	0.63605027	-0.389851	1.71E-05	hypomethylated
cg12768145	0.86459	0.659853514	-0.38987	4.48E-06	hypomethylated
cg15730481	0.71931	0.548968108	-0.389891	0.000155	hypomethylated
cg06415153	0.53945	0.411696216	-0.389909	0.000166	hypomethylated
cg11031737	0.76721	0.585517297	-0.38991	0.000251	hypomethylated
cg01544270	0.61857	0.472035135	-0.390043	0.000189	hypomethylated
cg10307345	0.54242	0.413918919	-0.390062	0.000135	hypomethylated
cg24773412	0.83417	0.636532432	-0.390107	1.71E-05	hypomethylated
cg05198969	0.7928	0.604962703	-0.390111	5.25E-05	hypomethylated
cg04294383	0.92099	0.702742703	-0.390189	1.88E-06	hypomethylated
cg22576236	0.54736	0.417642162	-0.390223	0.000517	hypomethylated
cg15902830	0.87918	0.670815135	-0.390243	2.27E-05	hypomethylated
cg25291404	0.60965	0.465145946	-0.390298	6.15E-05	hypomethylated
cg16281322	0.55316	0.422038378	-0.390323	9.72E-05	hypomethylated
cg03069731	0.78164	0.596341622	-0.390365	0.000445	hypomethylated
cg14663278	0.72817	0.555543243	-0.390376	8.37E-06	hypomethylated
cg05293365	0.60076	0.458287027	-0.390537	0.01146	hypomethylated
cg14478475	0.73657	0.561880541	-0.390559	4.20E-05	hypomethylated
cg12017722	0.85274	0.650485405	-0.390589	3.15E-05	hypomethylated
cg08555912	0.76357	0.58246	-0.390601	9.69E-06	hypomethylated
cg07249224	0.73975	0.564256757	-0.390686	0.000317	hypomethylated
cg03669367	0.74147	0.565567568	-0.390689	3.04E-05	hypomethylated
cg01001472	0.83939	0.640190811	-0.390839	3.00E-05	hypomethylated
cg24676612	0.7707	0.587778919	-0.390896	4.36E-05	hypomethylated
cg15280363	0.52961	0.403908108	-0.390903	0.000469	hypomethylated
cg11685391	0.62426	0.476084324	-0.39093	0.001734	hypomethylated
cg27640064	0.68518	0.522541081	-0.390939	0.007282	hypomethylated

cg07408148	0.73798	0.562785946	-0.390995	0.00043	hypomethylated
cg03012028	0.51931	0.396018378	-0.391029	6.08E-05	hypomethylated
cg13434570	0.87603	0.668022703	-0.391083	1.54E-05	hypomethylated
cg02607319	0.59886	0.456661081	-0.391095	0.000195	hypomethylated
cg10825234	0.81966	0.625027027	-0.391107	3.32E-05	hypomethylated
cg03883572	0.68678	0.5237	-0.391107	0.004365	hypomethylated
cg26861995	0.70211	0.535387568	-0.391113	0.004287	hypomethylated
cg08715226	0.47812	0.364575135	-0.391157	0.001587	hypomethylated
cg16136068	0.83591	0.637338378	-0.391288	8.42E-05	hypomethylated
cg23580024	0.96101	0.732714054	-0.391301	6.66E-06	hypomethylated
cg15323253	0.76908	0.586375135	-0.39131	0.000374	hypomethylated
cg27568165	0.6839	0.521416216	-0.39135	8.22E-05	hypomethylated
cg13956452	0.71285	0.543485946	-0.391356	1.04E-05	hypomethylated
cg26377677	0.7377	0.562422703	-0.391379	3.11E-05	hypomethylated
cg26948274	0.55203	0.420854054	-0.391427	8.15E-06	hypomethylated
cg09373727	0.86929	0.662715676	-0.391448	3.57E-05	hypomethylated
cg20806725	0.82588	0.62961027	-0.391473	5.06E-05	hypomethylated
cg24910161	0.68234	0.520155676	-0.391547	1.66E-05	hypomethylated
cg12584520	0.89335	0.681001622	-0.391567	2.54E-05	hypomethylated
cg17104766	0.84062	0.640791892	-0.391598	2.71E-05	hypomethylated
cg22623273	0.73986	0.563944324	-0.3917	0.000103	hypomethylated
cg24237576	0.79511	0.606037297	-0.391748	2.64E-06	hypomethylated
cg18119644	0.95785	0.730056757	-0.391791	6.05E-06	hypomethylated
cg10419556	0.62144	0.473632432	-0.391847	0.000271	hypomethylated
cg18919659	0.65418	0.498582162	-0.391856	1.15E-05	hypomethylated
cg09741917	0.60633	0.462094054	-0.391917	0.000202	hypomethylated
cg08455073	0.73059	0.556781081	-0.391952	0.000245	hypomethylated
cg22569154	0.79177	0.603392973	-0.391984	3.23E-05	hypomethylated
cg04033718	0.66663	0.508009189	-0.392032	0.000117	hypomethylated
cg21658153	0.84012	0.64020973	-0.392051	2.04E-05	hypomethylated
cg25366404	0.6194	0.471987568	-0.392123	3.57E-05	hypomethylated
cg21498490	0.90346	0.688432973	-0.392145	6.70E-05	hypomethylated
cg03023237	0.71622	0.545734595	-0.392203	1.35E-05	hypomethylated
cg14137548	0.66293	0.505124865	-0.392216	6.46E-05	hypomethylated
cg10325497	0.57961	0.441636757	-0.392222	1.54E-05	hypomethylated
cg03286735	0.87065	0.663363243	-0.392294	2.27E-05	hypomethylated
cg15246238	0.76962	0.586330811	-0.392431	0.000262	hypomethylated
cg16157895	0.78595	0.598664324	-0.39269	2.39E-05	hypomethylated
cg06852309	0.61665	0.469677297	-0.392782	2.35E-05	hypomethylated
cg04266474	0.79851	0.608097838	-0.393007	1.25E-05	hypomethylated
cg25963412	0.78745	0.59962973	-0.393116	9.31E-06	hypomethylated
cg06730183	0.89581	0.682138378	-0.393128	4.42E-06	hypomethylated
cg26346796	0.57069	0.434563243	-0.393141	8.22E-05	hypomethylated
cg21776813	0.78519	0.59788	-0.393186	2.27E-05	hypomethylated
cg25599065	0.73606	0.560436757	-0.393272	2.27E-05	hypomethylated
cg09639264	0.74267	0.565461622	-0.393292	5.89E-06	hypomethylated
cg13783238	0.79113	0.602356757	-0.393297	0.000506	hypomethylated
cg24526899	0.67423	0.513297838	-0.393445	0.00028	hypomethylated
cg11600991	0.66647	0.507385405	-0.393458	1.89E-05	hypomethylated
cg13413982	0.8317	0.633142703	-0.393533	1.54E-05	hypomethylated
cg24793722	0.5145	0.391660541	-0.393567	0.000214	hypomethylated
cg09837037	0.77969	0.593515676	-0.393614	0.000117	hypomethylated
cg27504861	0.7109	0.541124865	-0.393685	0.000805	hypomethylated
cg20418529	0.80947	0.616144865	-0.393708	0.000454	hypomethylated
cg14291900	0.87021	0.662367027	-0.393733	9.72E-05	hypomethylated
cg05134325	0.69208	0.52673027	-0.393874	0.000327	hypomethylated
cg04287684	0.86215	0.656150811	-0.393911	2.36E-06	hypomethylated
cg08244028	0.77159	0.587217297	-0.39394	2.45E-05	hypomethylated

cg21481966	0.58481	0.445047027	-0.39401	2.21E-05	hypomethylated
cg13249774	0.86901	0.661305405	-0.394056	1.50E-05	hypomethylated
cg25141995	0.65068	0.495128649	-0.394145	9.95E-05	hypomethylated
cg10549018	0.63107	0.480194595	-0.394181	0.001096	hypomethylated
cg16381059	0.86478	0.658015135	-0.394212	3.90E-06	hypomethylated
cg26130864	0.80605	0.613322162	-0.394224	1.46E-05	hypomethylated
cg27196194	0.95232	0.724611351	-0.394239	9.69E-06	hypomethylated
cg16087826	0.81334	0.618848108	-0.394273	2.45E-05	hypomethylated
cg22715761	0.6629	0.504339459	-0.394396	5.73E-06	hypomethylated
cg03421657	0.71791	0.546182703	-0.394419	0.000245	hypomethylated
cg00292447	0.69164	0.526177297	-0.394472	6.01E-05	hypomethylated
cg08599249	0.53867	0.409802162	-0.394474	0.000626	hypomethylated
cg10503007	0.60596	0.460987027	-0.394496	0.000132	hypomethylated
cg25555068	0.68348	0.519951892	-0.394521	1.92E-05	hypomethylated
cg07392702	0.58943	0.448397838	-0.394541	0.000152	hypomethylated
cg04034685	0.58433	0.444512432	-0.39456	0.001314	hypomethylated
cg17475774	0.71311	0.542473514	-0.394572	7.12E-06	hypomethylated
cg16747928	0.59519	0.45272973	-0.3947	0.001556	hypomethylated
cg20918682	0.46223	0.351592432	-0.394707	7.12E-05	hypomethylated
cg05372679	0.81653	0.621084865	-0.394715	8.52E-05	hypomethylated
cg23838943	0.79226	0.602621622	-0.394722	6.62E-05	hypomethylated
cg04912542	0.71042	0.540355676	-0.394763	1.28E-05	hypomethylated
cg14763096	0.69879	0.531504865	-0.394776	0.000178	hypomethylated
cg06646708	0.72915	0.554596757	-0.394776	0.000115	hypomethylated
cg05774698	0.87599	0.666279459	-0.394787	1.99E-05	hypomethylated
cg27582563	0.72372	0.55044	-0.394846	7.93E-05	hypomethylated
cg21790314	0.7829	0.595432973	-0.394889	5.14E-06	hypomethylated
cg07955887	0.90919	0.691470811	-0.394913	1.05E-05	hypomethylated
cg18254586	0.74255	0.564661081	-0.395103	0.000159	hypomethylated
cg16585619	0.87965	0.668904324	-0.39513	1.89E-05	hypomethylated
cg12182580	0.83428	0.63434	-0.395275	4.82E-05	hypomethylated
cg08687540	0.75639	0.575114054	-0.395282	0.002803	hypomethylated
cg07724905	0.76152	0.578983784	-0.395359	3.07E-05	hypomethylated
cg12339133	0.71814	0.545997297	-0.395371	0.000229	hypomethylated
cg17006413	0.95066	0.722780541	-0.395372	3.23E-05	hypomethylated
cg02495552	0.97082	0.738057297	-0.395471	3.04E-06	hypomethylated
cg05954120	0.65122	0.495024324	-0.395646	0.00012	hypomethylated
cg21140160	0.80369	0.610904865	-0.395691	2.74E-05	hypomethylated
cg02597894	0.77125	0.586192432	-0.395824	4.31E-05	hypomethylated
cg24562081	0.85493	0.649792432	-0.395827	1.06E-05	hypomethylated
cg05024961	0.792	0.601962162	-0.395828	1.68E-05	hypomethylated
cg12338657	0.71986	0.547120541	-0.395858	4.26E-05	hypomethylated
cg23172853	0.71871	0.546227568	-0.395908	0.000921	hypomethylated
cg21686213	0.74459	0.565822703	-0.396096	0.000681	hypomethylated
cg11278107	0.92971	0.706448108	-0.396197	7.86E-07	hypomethylated
cg20286882	0.56167	0.426788108	-0.396203	0.00031	hypomethylated
cg20596647	0.57	0.433108649	-0.396233	0.004059	hypomethylated
cg10631289	0.70081	0.532476757	-0.396305	0.000117	hypomethylated
cg04993130	0.48186	0.366094595	-0.396398	0.000464	hypomethylated
cg03421300	0.86055	0.653780541	-0.396453	3.21E-06	hypomethylated
cg04823219	0.86829	0.65964	-0.396498	1.87E-05	hypomethylated
cg24775180	0.92844	0.705318378	-0.396534	4.24E-06	hypomethylated
cg10131810	0.8007	0.608187027	-0.396747	7.22E-06	hypomethylated
cg01552731	0.78954	0.599705946	-0.396757	0.000268	hypomethylated
cg04348222	0.5963	0.452921622	-0.396777	0.000445	hypomethylated
cg24315710	0.83206	0.631972973	-0.396825	8.60E-06	hypomethylated
cg14495958	0.60821	0.461938919	-0.396867	3.27E-05	hypomethylated
cg05347216	0.62486	0.47458	-0.396882	0.00024	hypomethylated

cg05854892	0.78901	0.599225946	-0.396943	2.64E-05	hypomethylated
cg10447404	0.74093	0.562696216	-0.396981	0.000174	hypomethylated
cg09976369	0.56572	0.429627568	-0.397002	0.004485	hypomethylated
cg22636722	0.71791	0.545166486	-0.397106	0.00095	hypomethylated
cg03738656	0.78652	0.597196216	-0.397278	0.000104	hypomethylated
cg12970171	0.53813	0.408556757	-0.397418	0.001734	hypomethylated
cg21679730	0.84047	0.638066486	-0.39749	4.47E-05	hypomethylated
cg00992039	0.77481	0.588215676	-0.397497	3.62E-05	hypomethylated
cg02306654	0.9127	0.69282973	-0.39764	4.73E-06	hypomethylated
cg05556361	0.77955	0.59174	-0.397678	0.000221	hypomethylated
cg08343600	0.80645	0.61214	-0.397723	3.67E-05	hypomethylated
cg03590328	0.73998	0.561617838	-0.397898	1.31E-05	hypomethylated
cg03534410	0.65471	0.496898378	-0.397905	2.45E-05	hypomethylated
cg10557907	0.80096	0.607877297	-0.39795	2.45E-05	hypomethylated
cg04829830	0.80726	0.612645946	-0.39798	0.000155	hypomethylated
cg26992186	0.83914	0.636836757	-0.397988	2.15E-05	hypomethylated
cg05244766	0.85186	0.646438378	-0.398103	1.12E-05	hypomethylated
cg19907968	0.91357	0.693266486	-0.398105	7.93E-06	hypomethylated
cg08111922	0.61107	0.463702703	-0.398138	0.000191	hypomethylated
cg03544781	0.78415	0.595035135	-0.398155	0.000563	hypomethylated
cg15975171	0.80409	0.610151892	-0.398189	4.10E-05	hypomethylated
cg06801385	0.57708	0.437848108	-0.398341	1.99E-05	hypomethylated
cg21603144	0.71873	0.54530973	-0.398374	0.000696	hypomethylated
cg08917117	0.85007	0.644917838	-0.398466	2.42E-05	hypomethylated
cg03908688	0.80809	0.613062703	-0.398481	1.88E-06	hypomethylated
cg14139652	0.84413	0.64037027	-0.398559	4.48E-06	hypomethylated
cg01186980	0.67385	0.511188108	-0.398573	0.000256	hypomethylated
cg08836954	0.75252	0.570842703	-0.398637	0.000112	hypomethylated
cg27230882	0.72846	0.55258973	-0.398641	0.000528	hypomethylated
cg16762308	0.75884	0.57562	-0.398679	1.37E-05	hypomethylated
cg13420413	0.59609	0.452161622	-0.398692	1.48E-05	hypomethylated
cg15658306	0.54266	0.411613514	-0.398758	0.001	hypomethylated
cg21249659	0.75839	0.575242703	-0.398769	0.000119	hypomethylated
cg00633768	0.67507	0.512041622	-0.398776	0.0003	hypomethylated
cg21439129	0.76343	0.579051351	-0.398805	1.71E-05	hypomethylated
cg00941010	0.81602	0.618936757	-0.398813	3.76E-05	hypomethylated
cg24900654	0.55601	0.421708108	-0.398866	6.31E-05	hypomethylated
cg06269559	0.74806	0.567368108	-0.398869	2.78E-05	hypomethylated
cg03142741	0.82522	0.625885405	-0.39888	6.84E-06	hypomethylated
cg05450336	0.87294	0.662040541	-0.398963	8.71E-06	hypomethylated
cg01014224	0.68243	0.517527027	-0.399047	0.000306	hypomethylated
cg03511735	0.96337	0.730578378	-0.399051	7.32E-06	hypomethylated
cg02070740	0.83918	0.636372973	-0.399108	0.000155	hypomethylated
cg00460589	0.77042	0.584226486	-0.399117	0.001141	hypomethylated
cg22941294	0.73605	0.558162703	-0.399118	0.000178	hypomethylated
cg03016867	0.6031	0.45731027	-0.399224	7.47E-05	hypomethylated
cg10195726	0.71756	0.544077297	-0.399288	1.58E-05	hypomethylated
cg06161099	0.79622	0.60371027	-0.399311	3.11E-05	hypomethylated
cg13873263	0.66628	0.505183243	-0.399322	0.009084	hypomethylated
cg03440989	0.7689	0.582974595	-0.399363	4.70E-05	hypomethylated
cg21616935	0.72659	0.550883243	-0.399395	0.001314	hypomethylated
cg10683615	0.5017	0.380363784	-0.399445	0.000219	hypomethylated
cg20095398	0.62379	0.472922703	-0.399456	0.000101	hypomethylated
cg11530213	0.69066	0.523612973	-0.399475	0.002649	hypomethylated
cg10632507	0.72909	0.552743784	-0.399486	6.46E-05	hypomethylated
cg04404694	0.50051	0.379447027	-0.3995	0.000358	hypomethylated
cg13963210	0.74941	0.568128649	-0.399538	5.45E-05	hypomethylated
cg19260329	0.60918	0.461786486	-0.399643	2.32E-05	hypomethylated

cg14497545	0.58838	0.446017838	-0.399647	7.47E-05	hypomethylated
cg12798338	0.61197	0.463875135	-0.399724	0.002409	hypomethylated
cg11457534	0.70189	0.532020541	-0.399763	7.52E-06	hypomethylated
cg01643090	0.78757	0.596954595	-0.399787	4.01E-06	hypomethylated
cg12183875	0.87979	0.666839459	-0.39982	1.31E-05	hypomethylated
cg13777668	0.74531	0.564904324	-0.399834	2.51E-05	hypomethylated
cg02936493	0.91717	0.695142703	-0.39988	1.04E-05	hypomethylated
cg03234557	0.81136	0.614914595	-0.399956	0.000563	hypomethylated
cg14724298	0.8119	0.615281622	-0.400055	0.000126	hypomethylated
cg24032156	0.52985	0.401535676	-0.400056	2.21E-05	hypomethylated
cg01617018	0.67217	0.509385946	-0.400067	0.00054	hypomethylated
cg16971128	0.73016	0.553304865	-0.400138	0.000274	hypomethylated
cg01289421	0.76021	0.576075676	-0.40014	8.63E-05	hypomethylated
cg06807993	0.88709	0.672218378	-0.40015	6.95E-05	hypomethylated
cg05445326	0.88719	0.672224865	-0.400299	3.27E-05	hypomethylated
cg01242196	0.64507	0.488768649	-0.400304	2.02E-05	hypomethylated
cg15422147	0.76656	0.580817838	-0.400313	2.27E-05	hypomethylated
cg26853415	0.69672	0.527878919	-0.400372	0.000111	hypomethylated
cg02703235	0.71878	0.544583784	-0.400396	0.00017	hypomethylated
cg00864293	0.6447	0.488448649	-0.400421	0.00097	hypomethylated
cg12658400	0.89042	0.674588649	-0.400478	6.05E-06	hypomethylated
cg23746050	0.79127	0.599461081	-0.400504	0.000112	hypomethylated
cg19397176	0.80205	0.607618919	-0.400525	3.95E-05	hypomethylated
cg25535982	0.74346	0.563214054	-0.400572	0.001511	hypomethylated
cg25193885	0.80143	0.60712	-0.400595	1.28E-05	hypomethylated
cg04138756	0.75091	0.568847027	-0.400599	3.32E-05	hypomethylated
cg21368566	0.80274	0.608094595	-0.400637	0.000112	hypomethylated
cg15043318	0.55286	0.418788108	-0.400694	0.000107	hypomethylated
cg13491563	0.74158	0.561696757	-0.400811	0.000112	hypomethylated
cg01046798	0.6511	0.493151892	-0.400847	0.000117	hypomethylated
cg14894245	0.75745	0.573691892	-0.400875	1.02E-05	hypomethylated
cg00024046	0.83419	0.631794595	-0.40092	1.46E-05	hypomethylated
cg04058799	0.7731	0.585519459	-0.400938	0.000132	hypomethylated
cg01400671	0.71847	0.544118919	-0.401006	0.003571	hypomethylated
cg17396305	0.50628	0.383416216	-0.401024	6.95E-05	hypomethylated
cg24189917	0.84014	0.636236757	-0.401066	2.51E-05	hypomethylated
cg23418201	0.88335	0.668937297	-0.401114	9.31E-06	hypomethylated
cg02558132	0.70325	0.532520541	-0.4012	2.85E-05	hypomethylated
cg09099500	0.78298	0.592868649	-0.401263	5.19E-05	hypomethylated
cg24854861	0.90059	0.681916757	-0.401275	3.79E-06	hypomethylated
cg03312427	0.61047	0.462241081	-0.401275	1.82E-05	hypomethylated
cg21856334	0.96621	0.731598378	-0.401285	1.49E-06	hypomethylated
cg12357606	0.74203	0.56182973	-0.401345	0.000342	hypomethylated
cg18767278	0.71928	0.544601622	-0.401352	0.000163	hypomethylated
cg09024962	0.92077	0.697125405	-0.401423	2.36E-06	hypomethylated
cg26162295	0.55498	0.420172973	-0.401452	5.14E-06	hypomethylated
cg04302618	0.9258	0.700890811	-0.401511	1.12E-06	hypomethylated
cg11966998	0.71362	0.540235135	-0.401569	0.000805	hypomethylated
cg06358191	0.84438	0.639193514	-0.40164	5.65E-06	hypomethylated
cg09999348	0.70275	0.531968108	-0.401672	0.00018	hypomethylated
cg23551720	0.75517	0.571643784	-0.401685	0.000495	hypomethylated
cg15925478	0.749	0.566961622	-0.401715	0.004567	hypomethylated
cg00190795	0.73648	0.55746973	-0.401753	2.78E-05	hypomethylated
cg14603605	0.84923	0.642797838	-0.40179	1.28E-05	hypomethylated
cg06236559	0.68414	0.517831351	-0.401809	0.004172	hypomethylated
cg03049846	0.66371	0.502327568	-0.401925	7.93E-05	hypomethylated
cg02985293	0.72388	0.547845946	-0.40198	1.84E-05	hypomethylated
cg19747745	0.7402	0.560187568	-0.402005	2.39E-05	hypomethylated

cg06889086	0.769	0.581962162	-0.402058	0.000588	hypomethylated
cg04918770	0.82338	0.623087027	-0.402125	7.12E-05	hypomethylated
cg27049094	0.87024	0.658518919	-0.402188	4.94E-05	hypomethylated
cg24461242	0.84103	0.636392432	-0.402241	5.89E-06	hypomethylated
cg23120541	0.66208	0.500977297	-0.40226	0.000365	hypomethylated
cg09512406	0.7806	0.590616216	-0.402362	3.69E-06	hypomethylated
cg01808708	0.88262	0.667803784	-0.402368	2.29E-06	hypomethylated
cg18393172	0.53667	0.406043784	-0.4024	9.69E-06	hypomethylated
cg01149264	0.82775	0.626268649	-0.402413	0.000112	hypomethylated
cg11185991	0.82381	0.623265946	-0.402464	0.000115	hypomethylated
cg16999568	0.54122	0.409467027	-0.402468	3.57E-05	hypomethylated
cg00656881	0.54266	0.410545405	-0.402507	0.000104	hypomethylated
cg24736734	0.68061	0.514896757	-0.402545	0.002106	hypomethylated
cg17730764	0.86377	0.653392973	-0.402696	2.39E-05	hypomethylated
cg20201177	0.81695	0.617910811	-0.402849	1.66E-05	hypomethylated
cg14204619	0.95538	0.722614054	-0.402849	1.04E-05	hypomethylated
cg23732725	0.92895	0.702579459	-0.40294	6.22E-06	hypomethylated
cg09853936	0.67779	0.512614054	-0.402965	0.000358	hypomethylated
cg25471639	0.60733	0.459322162	-0.402974	0.000517	hypomethylated
cg00175150	0.80178	0.60637027	-0.403007	2.30E-05	hypomethylated
cg08049614	0.77717	0.587751892	-0.403023	2.10E-05	hypomethylated
cg25362050	0.57616	0.435690811	-0.403165	1.99E-05	hypomethylated
cg17457701	0.76019	0.574834054	-0.403215	9.31E-06	hypomethylated
cg14826683	0.86949	0.657475135	-0.403233	1.09E-05	hypomethylated
cg10755077	0.57709	0.436361622	-0.403272	0.000138	hypomethylated
cg07699300	0.51557	0.389822162	-0.403352	9.95E-05	hypomethylated
cg08670715	0.72599	0.548894054	-0.403422	5.19E-05	hypomethylated
cg25202407	0.83803	0.633603243	-0.403422	1.84E-05	hypomethylated
cg05214511	0.86196	0.65169027	-0.403434	1.08E-05	hypomethylated
cg11912215	0.54626	0.412979459	-0.403518	9.82E-06	hypomethylated
cg26923862	0.96626	0.730484324	-0.403558	7.72E-06	hypomethylated
cg10085326	0.82768	0.625699459	-0.403603	1.71E-05	hypomethylated
cg23599820	0.65593	0.495842162	-0.403661	0.001395	hypomethylated
cg22762189	0.82452	0.623275135	-0.403685	1.23E-05	hypomethylated
cg14037769	0.66995	0.506423243	-0.40371	5.38E-05	hypomethylated
cg25343661	0.664	0.501908108	-0.40376	0.000256	hypomethylated
cg12535109	0.8643	0.65330973	-0.403765	8.03E-05	hypomethylated
cg11580028	0.76541	0.578554595	-0.40378	1.31E-05	hypomethylated
cg12656272	0.63993	0.483687568	-0.403839	0.004863	hypomethylated
cg20154206	0.53698	0.405860541	-0.403884	6.31E-05	hypomethylated
cg15225810	0.64828	0.489970811	-0.403921	2.32E-05	hypomethylated
cg18565216	0.44795	0.338561081	-0.403922	0.002965	hypomethylated
cg12692386	0.72374	0.546939459	-0.40409	3.39E-06	hypomethylated
cg18892446	0.86979	0.657271351	-0.404178	5.43E-06	hypomethylated
cg23813681	0.78757	0.595107568	-0.404258	0.000104	hypomethylated
cg08720806	0.64929	0.490617297	-0.404265	0.001368	hypomethylated
cg10071493	0.71582	0.54088	-0.404288	5.72E-05	hypomethylated
cg26611070	0.91964	0.694874054	-0.404318	1.80E-06	hypomethylated
cg26493188	0.63727	0.481516757	-0.404319	9.31E-06	hypomethylated
cg08548559	0.61922	0.467848649	-0.40441	0.007536	hypomethylated
cg06139893	0.73284	0.553667568	-0.404478	1.46E-05	hypomethylated
cg02465859	0.85297	0.644416757	-0.404501	6.14E-06	hypomethylated
cg18567041	0.70793	0.534835135	-0.404512	0.000219	hypomethylated
cg11839681	0.71746	0.542024865	-0.404539	9.49E-05	hypomethylated
cg02696798	0.70109	0.529632432	-0.404608	0.0006	hypomethylated
cg02216951	0.8085	0.610757297	-0.404649	4.48E-06	hypomethylated
cg21157065	0.72694	0.549139459	-0.404664	0.00012	hypomethylated
cg24036039	0.76676	0.579215135	-0.404676	3.76E-05	hypomethylated

cg00677272	0.66261	0.500523243	-0.404723	0.000764	hypomethylated
cg13785779	0.74555	0.563141622	-0.404807	1.58E-05	hypomethylated
cg08977790	0.78585	0.593554054	-0.404875	9.49E-05	hypomethylated
cg06103086	0.70641	0.533508649	-0.404994	0.000667	hypomethylated
cg04848686	0.77242	0.583352973	-0.405016	6.46E-05	hypomethylated
cg18957070	0.83169	0.628108649	-0.405032	3.57E-05	hypomethylated
cg18037826	0.79769	0.602428649	-0.405038	0.000145	hypomethylated
cg22469870	0.90926	0.686685946	-0.405042	2.05E-06	hypomethylated
cg07776049	0.6496	0.490559459	-0.405124	0.000399	hypomethylated
cg14752965	0.76239	0.575734054	-0.405127	6.86E-05	hypomethylated
cg10057904	0.65855	0.497293514	-0.405195	0.000123	hypomethylated
cg24339704	0.80843	0.610456757	-0.405234	4.26E-05	hypomethylated
cg13690407	0.76631	0.578620541	-0.405311	0.000726	hypomethylated
cg03321553	0.69491	0.524678919	-0.405391	0.000256	hypomethylated
cg16603012	0.79761	0.602201622	-0.405437	0.000178	hypomethylated
cg02646779	0.75039	0.566543243	-0.405455	3.15E-05	hypomethylated
cg01090482	0.56874	0.429331351	-0.405678	0.001768	hypomethylated
cg17384323	0.77106	0.582055135	-0.405687	0.00012	hypomethylated
cg22054793	0.86255	0.651098919	-0.405731	8.26E-06	hypomethylated
cg00273863	0.60227	0.454620541	-0.405748	2.10E-05	hypomethylated
cg01378158	0.68759	0.518994054	-0.405831	0.000102	hypomethylated
cg05055844	0.73793	0.556978919	-0.405861	0.000119	hypomethylated
cg21138405	0.65177	0.491942703	-0.405873	0.001052	hypomethylated
cg16072754	0.62671	0.473016216	-0.405908	0.000185	hypomethylated
cg14852276	0.88169	0.665458378	-0.405923	1.84E-05	hypomethylated
cg06058576	0.88253	0.666074054	-0.405963	6.86E-05	hypomethylated
cg25875163	0.79074	0.596755676	-0.406063	1.66E-05	hypomethylated
cg01883662	0.74007	0.558512973	-0.406071	0.000132	hypomethylated
cg07820280	0.87531	0.660545946	-0.406135	1.54E-06	hypomethylated
cg17893669	0.75921	0.572931351	-0.406137	5.31E-05	hypomethylated
cg13646480	0.57695	0.435388108	-0.406144	2.67E-05	hypomethylated
cg13505608	0.76886	0.580195676	-0.406181	1.28E-05	hypomethylated
cg07800544	0.79083	0.596764324	-0.406206	0.000209	hypomethylated
cg21511203	0.92843	0.700595135	-0.406212	1.63E-06	hypomethylated
cg24704396	0.91273	0.688722162	-0.406266	8.03E-05	hypomethylated
cg11215644	0.44451	0.33539027	-0.406375	0.000245	hypomethylated
cg25098793	0.75136	0.566907568	-0.406391	0.000132	hypomethylated
cg19371339	0.7171	0.541043243	-0.40643	6.08E-05	hypomethylated
cg21423741	0.86436	0.652126486	-0.40648	1.93E-06	hypomethylated
cg15002761	0.95342	0.719280541	-0.406557	3.04E-05	hypomethylated
cg01762581	0.60131	0.453636216	-0.406573	0.000216	hypomethylated
cg27221631	0.88846	0.670218378	-0.406676	2.51E-05	hypomethylated
cg12587087	0.7104	0.535878378	-0.406726	0.00032	hypomethylated
cg02325250	0.47408	0.357612432	-0.406734	0.000115	hypomethylated
cg06737981	0.55443	0.41821027	-0.406777	0.001541	hypomethylated
cg21735384	0.82073	0.619044865	-0.406864	1.72E-06	hypomethylated
cg13882345	0.69722	0.525883784	-0.40687	2.79E-06	hypomethylated
cg06892907	0.6873	0.51838973	-0.406903	0.001409	hypomethylated
cg12559827	0.75819	0.571854595	-0.406911	0.000157	hypomethylated
cg01165355	0.70539	0.532018919	-0.406944	0.000166	hypomethylated
cg24924505	0.53729	0.405231892	-0.406953	4.70E-05	hypomethylated
cg26703511	0.80949	0.610484324	-0.407059	5.45E-05	hypomethylated
cg07953935	0.59867	0.451444865	-0.407211	0.000293	hypomethylated
cg20250250	0.71624	0.540094054	-0.407232	0.000159	hypomethylated
cg19807685	0.58858	0.443823784	-0.407252	0.000207	hypomethylated
cg15388107	0.72125	0.543846486	-0.4073	8.84E-05	hypomethylated
cg18024358	0.71978	0.542734595	-0.407309	3.00E-05	hypomethylated
cg02638567	0.8135	0.613361622	-0.407404	0.000178	hypomethylated

cg20376082	0.87317	0.658332973	-0.407445	3.15E-05	hypomethylated
cg07803218	0.83684	0.630920541	-0.407493	0.000107	hypomethylated
cg01727625	0.50663	0.381963784	-0.407497	5.72E-05	hypomethylated
cg15163151	0.78702	0.593357297	-0.407499	0.0002	hypomethylated
cg00386586	0.91075	0.686620541	-0.407542	3.96E-06	hypomethylated
cg24707200	0.53826	0.405779459	-0.407607	0.000152	hypomethylated
cg16037981	0.73626	0.555034595	-0.407638	0.001526	hypomethylated
cg10276016	0.77502	0.584237297	-0.407679	5.32E-05	hypomethylated
cg18176482	0.64982	0.489792973	-0.407868	0.000506	hypomethylated
cg14489794	0.56635	0.426871892	-0.407891	6.15E-05	hypomethylated
cg17756730	0.66428	0.500683784	-0.407892	0.000726	hypomethylated
cg14095048	0.56929	0.429064865	-0.407968	0.000204	hypomethylated
cg24480515	0.86631	0.652923243	-0.40797	1.05E-05	hypomethylated
cg23014759	0.81456	0.613898378	-0.408021	9.49E-05	hypomethylated
cg26936219	0.6883	0.518734054	-0.408042	0.001164	hypomethylated
cg06366345	0.87396	0.658644324	-0.408068	1.99E-05	hypomethylated
cg01811583	0.59218	0.446276216	-0.408099	6.15E-05	hypomethylated
cg12127811	0.69194	0.521454595	-0.408105	0.00064	hypomethylated
cg20731121	0.76256	0.574667027	-0.408125	0.000253	hypomethylated
cg23295636	0.79519	0.599237297	-0.408172	1.04E-05	hypomethylated
cg00295259	0.80003	0.602874054	-0.408197	2.78E-05	hypomethylated
cg01165442	0.84237	0.634762703	-0.408237	0.000107	hypomethylated
cg08974852	0.77939	0.58729027	-0.408272	1.06E-05	hypomethylated
cg13211559	0.84814	0.63904973	-0.408374	3.07E-05	hypomethylated
cg10251594	0.70461	0.530866486	-0.408476	0.000155	hypomethylated
cg14861497	0.82965	0.625071892	-0.408481	1.01E-05	hypomethylated
cg20170777	0.68533	0.516333514	-0.408496	1.44E-05	hypomethylated
cg19245120	0.83464	0.628746486	-0.408676	2.57E-05	hypomethylated
cg20401945	0.47161	0.355266486	-0.408693	0.000155	hypomethylated
cg23088126	0.78644	0.592402703	-0.408758	0.000191	hypomethylated
cg22243298	0.85646	0.645138919	-0.408776	0.000274	hypomethylated
cg19458497	0.59298	0.446666486	-0.408785	1.46E-05	hypomethylated
cg26548834	0.81584	0.614528108	-0.408807	0.000331	hypomethylated
cg22160267	0.4989	0.375792432	-0.408815	0.001212	hypomethylated
cg07169637	0.76281	0.574571892	-0.408836	7.72E-06	hypomethylated
cg11898431	0.65911	0.496448108	-0.408876	0.002026	hypomethylated
cg09915232	0.66525	0.501066486	-0.408895	0.0002	hypomethylated
cg04585669	0.78257	0.589402162	-0.408967	5.45E-05	hypomethylated
cg07306604	0.76374	0.575217838	-0.408973	2.26E-06	hypomethylated
cg00736681	0.85316	0.642551351	-0.409005	8.22E-05	hypomethylated
cg17126924	0.85544	0.644241622	-0.409065	0.000126	hypomethylated
cg24024661	0.6823	0.513832432	-0.409108	0.001541	hypomethylated
cg23699463	0.74909	0.564125946	-0.409122	0.000245	hypomethylated
cg09408902	0.59898	0.451071351	-0.409152	2.02E-05	hypomethylated
cg19030994	0.87662	0.660134054	-0.409193	1.84E-05	hypomethylated
cg08767838	0.87367	0.657862162	-0.409303	0.000202	hypomethylated
cg23419907	0.61083	0.459933514	-0.409346	6.15E-05	hypomethylated
cg16708938	0.54528	0.410574054	-0.409355	8.42E-05	hypomethylated
cg13004635	0.71059	0.535016216	-0.409435	2.78E-05	hypomethylated
cg09202851	0.63639	0.47912973	-0.409495	0.000245	hypomethylated
cg21193888	0.78268	0.589261081	-0.409516	0.000176	hypomethylated
cg23980760	0.7478	0.562981081	-0.409566	8.37E-06	hypomethylated
cg03719555	0.79031	0.594957838	-0.409631	6.95E-05	hypomethylated
cg04849850	0.66006	0.496863784	-0.409747	0.000221	hypomethylated
cg14212447	0.55651	0.418907568	-0.409776	0.000262	hypomethylated
cg10505902	0.83234	0.626526486	-0.409797	0.000191	hypomethylated
cg08248751	0.61493	0.462806486	-0.410013	1.89E-05	hypomethylated
cg03669698	0.76248	0.573818919	-0.410104	0.000365	hypomethylated

cg03372205	0.7724	0.581282162	-0.41011	3.76E-05	hypomethylated
cg24074594	0.8177	0.615308108	-0.410263	0.000126	hypomethylated
cg26219843	0.80652	0.606889189	-0.410277	1.18E-05	hypomethylated
cg01034754	0.84611	0.636679459	-0.410278	5.32E-05	hypomethylated
cg01722566	0.7752	0.583317297	-0.410288	7.83E-06	hypomethylated
cg18693051	0.74403	0.55981027	-0.410423	0.000163	hypomethylated
cg26553501	0.61767	0.464704865	-0.410522	0.000667	hypomethylated
cg06987292	0.82164	0.618128108	-0.410601	1.12E-05	hypomethylated
cg04457794	0.70364	0.529354054	-0.410605	2.10E-05	hypomethylated
cg10986946	0.47633	0.358336757	-0.410645	0.0002	hypomethylated
cg26488978	0.69372	0.52186973	-0.410664	0.000696	hypomethylated
cg04662594	0.53175	0.400016216	-0.41069	0.00017	hypomethylated
cg23410129	0.73826	0.555344324	-0.410746	4.47E-05	hypomethylated
cg18780489	0.67827	0.510202162	-0.410791	0.00039	hypomethylated
cg02165670	0.8051	0.605591892	-0.410822	0.000449	hypomethylated
cg03044344	0.58855	0.442696216	-0.410848	1.25E-05	hypomethylated
cg12764314	0.68127	0.512401622	-0.410952	1.99E-05	hypomethylated
cg18552520	0.77669	0.584117297	-0.411081	4.12E-06	hypomethylated
cg06968788	0.84647	0.636554595	-0.411175	2.21E-05	hypomethylated
cg09728393	0.80499	0.605347027	-0.411208	5.45E-05	hypomethylated
cg15252509	0.80382	0.604411351	-0.411342	9.44E-06	hypomethylated
cg11673687	0.78079	0.587071892	-0.411397	1.89E-05	hypomethylated
cg05250241	0.94362	0.709482703	-0.411438	1.46E-05	hypomethylated
cg04270264	0.84036	0.631841622	-0.411445	6.05E-06	hypomethylated
cg18208602	0.81379	0.611863243	-0.411447	0.000189	hypomethylated
cg19103704	0.4921	0.369972973	-0.411532	0.000449	hypomethylated
cg03398156	0.68111	0.512065405	-0.41156	4.47E-05	hypomethylated
cg01356872	0.84066	0.632008649	-0.411578	0.000135	hypomethylated
cg13576290	0.62096	0.466804865	-0.411681	0.000756	hypomethylated
cg00425213	0.70424	0.529408108	-0.411687	0.000287	hypomethylated
cg12551957	0.71199	0.535204865	-0.411766	9.95E-05	hypomethylated
cg21565415	0.7285	0.547611351	-0.411777	0.000317	hypomethylated
cg14121772	0.78746	0.591913514	-0.41182	3.39E-06	hypomethylated
cg05208178	0.51768	0.389125405	-0.411825	0.002993	hypomethylated
cg11147309	0.6876	0.516842703	-0.411844	8.83E-06	hypomethylated
cg18118198	0.79461	0.597264865	-0.411876	5.43E-06	hypomethylated
cg10828561	0.60885	0.457637297	-0.411882	1.62E-05	hypomethylated
cg27507254	0.78635	0.59102973	-0.411941	4.47E-05	hypomethylated
cg09033641	0.56514	0.424734054	-0.412049	9.95E-05	hypomethylated
cg21484834	0.72053	0.541498919	-0.4121	3.15E-05	hypomethylated
cg06601993	0.79121	0.59460973	-0.412118	0.000331	hypomethylated
cg25948982	0.45854	0.3446	-0.412125	0.000207	hypomethylated
cg05846044	0.86766	0.652056757	-0.412132	1.31E-05	hypomethylated
cg19251656	0.62958	0.473123243	-0.412174	1.58E-05	hypomethylated
cg18166959	0.69302	0.520764865	-0.412265	0.000626	hypomethylated
cg06082883	0.61576	0.462677838	-0.41236	0.000805	hypomethylated
cg08660562	0.8767	0.658742162	-0.412369	1.35E-05	hypomethylated
cg14766231	0.74544	0.560108108	-0.412387	0.000563	hypomethylated
cg09169014	0.78984	0.593468649	-0.412389	9.44E-06	hypomethylated
cg10880599	0.79431	0.596816216	-0.412415	0.000454	hypomethylated
cg18880986	0.80864	0.607565946	-0.412457	2.21E-05	hypomethylated
cg07181257	0.79956	0.600737838	-0.412471	1.42E-05	hypomethylated
cg23526087	0.60027	0.450994595	-0.412501	0.000342	hypomethylated
cg14069214	0.78332	0.588500541	-0.412558	0.000253	hypomethylated
cg26591162	0.8194	0.615572973	-0.412638	1.09E-05	hypomethylated
cg07028950	0.58633	0.44046	-0.412702	0.00028	hypomethylated
cg19795866	0.85136	0.639515676	-0.41279	8.83E-06	hypomethylated
cg16870215	0.58953	0.442831892	-0.412806	1.58E-05	hypomethylated

cg16354802	0.66583	0.500106486	-0.412919	0.000117	hypomethylated
cg07918814	0.92114	0.69186	-0.41294	3.69E-06	hypomethylated
cg26552650	0.79639	0.59812	-0.41304	0.000382	hypomethylated
cg18473733	0.57656	0.433013514	-0.413059	0.001141	hypomethylated
cg04421974	0.9271	0.696270811	-0.413076	3.85E-05	hypomethylated
cg01747591	0.81214	0.609925405	-0.413096	1.40E-05	hypomethylated
cg19928195	0.75926	0.570205405	-0.413112	7.84E-05	hypomethylated
cg17612535	0.55551	0.417187027	-0.413119	0.00012	hypomethylated
cg15010903	0.90535	0.679897838	-0.413158	4.42E-06	hypomethylated
cg03038262	0.53557	0.402156757	-0.413317	0.001803	hypomethylated
cg12404590	0.6417	0.481849189	-0.413317	0.000237	hypomethylated
cg11074968	0.73894	0.554831892	-0.413407	5.86E-05	hypomethylated
cg08670693	0.81889	0.614842162	-0.413454	0.000155	hypomethylated
cg19798881	0.83704	0.628398378	-0.413617	0.000107	hypomethylated
cg19055098	0.88892	0.667336216	-0.41364	3.44E-06	hypomethylated
cg13420075	0.59112	0.443755676	-0.413685	0.000417	hypomethylated
cg11075561	0.70561	0.529695676	-0.413707	0.000306	hypomethylated
cg10230591	0.70463	0.528938919	-0.413765	8.42E-05	hypomethylated
cg04983349	0.6843	0.513672432	-0.41378	0.000805	hypomethylated
cg27511289	0.87888	0.659703243	-0.413849	2.10E-05	hypomethylated
cg21553700	0.95087	0.713736757	-0.413856	3.04E-05	hypomethylated
cg02577580	0.68029	0.510632973	-0.413863	3.85E-05	hypomethylated
cg19551485	0.79002	0.592980541	-0.413904	0.00012	hypomethylated
cg14227911	0.73716	0.553298378	-0.41392	6.62E-05	hypomethylated
cg09780964	0.75006	0.562972973	-0.41394	1.75E-05	hypomethylated
cg02106180	0.87962	0.660201081	-0.413975	5.19E-05	hypomethylated
cg04756252	0.79213	0.594532973	-0.41398	4.58E-05	hypomethylated
cg24112628	0.8217	0.616721622	-0.413992	0.000242	hypomethylated
cg22621734	0.49764	0.373454595	-0.41417	0.000324	hypomethylated
cg22109795	0.67497	0.506521622	-0.4142	0.000195	hypomethylated
cg26357344	0.74623	0.559968108	-0.414276	0.000115	hypomethylated
cg20788021	0.72846	0.54663027	-0.414284	3.07E-05	hypomethylated
cg12277870	0.87375	0.655628108	-0.414343	8.04E-06	hypomethylated
cg06431514	0.78258	0.587177838	-0.414441	0.000163	hypomethylated
cg22749051	0.9579	0.718711351	-0.414463	2.78E-05	hypomethylated
cg25461389	0.4504	0.337930811	-0.414479	0.000176	hypomethylated
cg08770935	0.59769	0.448398378	-0.414616	8.84E-05	hypomethylated
cg23348723	0.66943	0.502178378	-0.414733	0.0003	hypomethylated
cg04104789	0.53844	0.403913514	-0.414739	0.000563	hypomethylated
cg12211091	0.84432	0.63336	-0.414764	7.65E-05	hypomethylated
cg01981417	0.6978	0.523436757	-0.414798	8.22E-05	hypomethylated
cg01929744	0.66621	0.499735135	-0.414813	7.41E-06	hypomethylated
cg17574812	0.75624	0.567252432	-0.414853	0.000667	hypomethylated
cg22716262	0.7909	0.59324973	-0.414856	0.000523	hypomethylated
cg03918703	0.6864	0.514862703	-0.414862	1.94E-05	hypomethylated
cg21733794	0.72064	0.540524865	-0.414918	1.80E-05	hypomethylated
cg22354175	0.72519	0.543936757	-0.41492	0.00066	hypomethylated
cg00033304	0.82965	0.622278378	-0.414943	1.40E-05	hypomethylated
cg22838050	0.60935	0.457019459	-0.415016	0.005316	hypomethylated
cg11692307	0.90495	0.67872	-0.415022	3.67E-05	hypomethylated
cg26334888	0.71552	0.536614595	-0.415106	3.23E-05	hypomethylated
cg22188603	0.87135	0.653466486	-0.415139	2.71E-06	hypomethylated
cg06366107	0.49611	0.372037838	-0.415211	0.004365	hypomethylated
cg19284726	0.82979	0.622242162	-0.41527	0.000159	hypomethylated
cg17340729	0.79115	0.593247027	-0.415318	0.00035	hypomethylated
cg03071245	0.83508	0.626183243	-0.41533	1.68E-05	hypomethylated
cg03561787	0.76545	0.573960541	-0.415357	1.62E-05	hypomethylated
cg07047589	0.56512	0.423743243	-0.415367	0.000667	hypomethylated

cg09508556	0.80382	0.602696216	-0.415441	1.41E-06	hypomethylated
cg05593510	0.81857	0.613746486	-0.415463	4.20E-05	hypomethylated
cg08568298	0.77613	0.581915135	-0.41549	2.21E-05	hypomethylated
cg18572353	0.88801	0.665777838	-0.415535	1.93E-06	hypomethylated
cg19753609	0.80088	0.600436216	-0.415575	2.61E-05	hypomethylated
cg05031931	0.89368	0.669979459	-0.415641	1.15E-05	hypomethylated
cg17987982	0.71793	0.538212973	-0.415666	0.00014	hypomethylated
cg25646708	0.8168	0.612295135	-0.415756	0.000613	hypomethylated
cg14471996	0.57816	0.433375676	-0.415851	0.000152	hypomethylated
cg06775420	0.94161	0.705789189	-0.415892	3.36E-05	hypomethylated
cg14664412	0.81809	0.613177297	-0.415955	0.000256	hypomethylated
cg17920789	0.79747	0.597721081	-0.415958	0.000195	hypomethylated
cg05845533	0.85015	0.637132973	-0.416123	7.12E-06	hypomethylated
cg04003903	0.54115	0.405553514	-0.416136	8.84E-05	hypomethylated
cg25070253	0.70206	0.526138919	-0.416151	0.000204	hypomethylated
cg18503829	0.90946	0.681562703	-0.416164	9.31E-06	hypomethylated
cg09916692	0.65004	0.487143243	-0.416182	0.000268	hypomethylated
cg06376426	0.67404	0.505088649	-0.416298	0.00098	hypomethylated
cg08500417	0.86634	0.649172973	-0.41633	5.45E-05	hypomethylated
cg06599949	0.59511	0.445889189	-0.416471	2.39E-05	hypomethylated
cg24960291	0.66016	0.494618919	-0.416498	0.000313	hypomethylated
cg21404063	0.76539	0.573444324	-0.416542	0.000575	hypomethylated
cg09298971	0.7727	0.578874054	-0.416659	1.23E-05	hypomethylated
cg05220069	0.81194	0.608263243	-0.416677	1.66E-05	hypomethylated
cg06907626	0.79082	0.592409189	-0.416755	5.58E-05	hypomethylated
cg11324467	0.72764	0.545075135	-0.41677	0.000111	hypomethylated
cg23837282	0.66823	0.500565946	-0.416785	7.52E-06	hypomethylated
cg09174162	0.74169	0.555592973	-0.416788	4.26E-05	hypomethylated
cg00640719	0.67863	0.508341081	-0.416828	3.19E-05	hypomethylated
cg17811994	0.88003	0.659192973	-0.416852	2.64E-05	hypomethylated
cg14199384	0.81908	0.61351027	-0.416917	4.73E-06	hypomethylated
cg10158715	0.6887	0.515844324	-0.41694	1.58E-05	hypomethylated
cg18155888	0.88239	0.660893514	-0.416999	1.94E-05	hypomethylated
cg10670430	0.66862	0.500776216	-0.41702	0.002007	hypomethylated
cg21776286	0.72957	0.546415676	-0.417048	0.0006	hypomethylated
cg05988358	0.68307	0.511566486	-0.417112	6.15E-05	hypomethylated
cg18045685	0.74998	0.561663784	-0.417145	0.000667	hypomethylated
cg15696058	0.64318	0.481679459	-0.417149	0.000511	hypomethylated
cg27533454	0.68263	0.511222703	-0.417152	4.58E-05	hypomethylated
cg09476347	0.91813	0.687587027	-0.417156	8.10E-07	hypomethylated
cg13213755	0.879	0.658245405	-0.417238	4.15E-05	hypomethylated
cg20283771	0.70074	0.524739459	-0.417278	2.71E-05	hypomethylated
cg18520925	0.87445	0.654818919	-0.41728	4.80E-06	hypomethylated
cg09539538	0.62127	0.465226486	-0.417287	0.000124	hypomethylated
cg13582028	0.62013	0.464371351	-0.417292	0.000219	hypomethylated
cg11114141	0.71215	0.53326973	-0.417316	3.15E-05	hypomethylated
cg12445424	0.86628	0.64866973	-0.417349	4.58E-05	hypomethylated
cg21177463	0.7588	0.568172432	-0.417391	9.27E-05	hypomethylated
cg09215282	0.8153	0.610438378	-0.417485	3.00E-05	hypomethylated
cg26120813	0.7304	0.546856757	-0.417524	0.000445	hypomethylated
cg23586996	0.78188	0.585394595	-0.417538	1.15E-05	hypomethylated
cg17338816	0.60336	0.451724865	-0.417575	0.008709	hypomethylated
cg23451221	0.52562	0.393520541	-0.417581	0.000166	hypomethylated
cg17753169	0.81119	0.607320541	-0.417582	4.26E-05	hypomethylated
cg27032813	0.75429	0.564718378	-0.417588	2.78E-05	hypomethylated
cg07098502	0.88387	0.66168973	-0.417679	2.39E-05	hypomethylated
cg01541846	0.85525	0.64024973	-0.417711	2.95E-06	hypomethylated
cg27615603	0.65076	0.487152973	-0.417751	8.13E-05	hypomethylated

cg23632393	0.58627	0.438867568	-0.41778	0.000256	hypomethylated
cg23730696	0.84532	0.632777297	-0.4178	3.23E-05	hypomethylated
cg02399008	0.77217	0.578005405	-0.417836	3.39E-06	hypomethylated
cg27042983	0.58818	0.440277838	-0.417844	2.89E-05	hypomethylated
cg08215954	0.78935	0.59085027	-0.417873	3.96E-06	hypomethylated
cg26986147	0.55748	0.417287027	-0.41788	0.004134	hypomethylated
cg04366994	0.79068	0.591829189	-0.417913	1.26E-05	hypomethylated
cg25276521	0.87663	0.656151892	-0.417938	5.89E-06	hypomethylated
cg25634141	0.49625	0.371434595	-0.417959	7.84E-05	hypomethylated
cg01806956	0.66804	0.500011892	-0.417972	0.002803	hypomethylated
cg15903562	0.88874	0.665196216	-0.417981	1.14E-05	hypomethylated
cg24833277	0.72476	0.542387027	-0.418181	6.23E-05	hypomethylated
cg05136724	0.82505	0.617428649	-0.418209	7.84E-05	hypomethylated
cg21492882	0.58553	0.438180541	-0.418218	4.82E-05	hypomethylated
cg26055488	0.79664	0.596156757	-0.418236	2.27E-05	hypomethylated
cg21334510	0.58119	0.434918378	-0.418265	1.33E-05	hypomethylated
cg00396699	0.59154	0.44266	-0.418277	7.72E-06	hypomethylated
cg17383272	0.71973	0.538567568	-0.418328	0.00017	hypomethylated
cg05125838	0.50936	0.381144865	-0.418346	0.000342	hypomethylated
cg19934294	0.62299	0.466169189	-0.418355	0.000224	hypomethylated
cg08587313	0.7862	0.588266486	-0.418427	0.000123	hypomethylated
cg24495350	0.76729	0.574108649	-0.418448	0.00032	hypomethylated
cg26784300	0.83116	0.621885405	-0.418477	0.000191	hypomethylated
cg22726373	0.73329	0.548653514	-0.418489	0.000138	hypomethylated
cg21614107	0.9331	0.698092432	-0.418614	1.25E-05	hypomethylated
cg22043788	0.95056	0.711135135	-0.418654	6.86E-05	hypomethylated
cg20340346	0.91926	0.687712973	-0.418666	0.000161	hypomethylated
cg09370867	0.61748	0.461943243	-0.418677	0.003021	hypomethylated
cg12907644	0.57728	0.431868108	-0.41868	0.000474	hypomethylated
cg04339692	0.92945	0.69521027	-0.418928	4.36E-05	hypomethylated
cg14812474	0.62005	0.463782703	-0.418936	0.000198	hypomethylated
cg00570628	0.72876	0.545094054	-0.418939	0.000178	hypomethylated
cg03364620	0.62719	0.469115135	-0.418961	9.05E-05	hypomethylated
cg27040708	0.80201	0.599859459	-0.418996	9.83E-05	hypomethylated
cg00368356	0.81556	0.609994054	-0.418996	4.36E-06	hypomethylated
cg03150378	0.85281	0.637845405	-0.419018	9.72E-05	hypomethylated
cg25689499	0.67598	0.505578378	-0.419046	6.46E-05	hypomethylated
cg16348003	0.59051	0.441647027	-0.419068	0.000132	hypomethylated
cg26804336	0.74302	0.555684324	-0.419136	6.48E-06	hypomethylated
cg25361506	0.65384	0.488982703	-0.419154	0.001262	hypomethylated
cg21283302	0.83652	0.625598919	-0.419162	2.68E-06	hypomethylated
cg13457496	0.92954	0.695143784	-0.419206	1.54E-05	hypomethylated
cg04334496	0.67522	0.504949189	-0.419219	0.00024	hypomethylated
cg00600477	0.59009	0.441284324	-0.419227	0.00024	hypomethylated
cg16361253	0.62312	0.465979459	-0.419244	7.12E-06	hypomethylated
cg08912860	0.80513	0.602080541	-0.419265	9.95E-05	hypomethylated
cg02524834	0.88097	0.658790811	-0.419272	1.66E-05	hypomethylated
cg24672624	0.67807	0.50705027	-0.419305	0.00255	hypomethylated
cg24335793	0.66173	0.49479027	-0.419426	2.15E-05	hypomethylated
cg08867471	0.7147	0.534389189	-0.419447	0.000421	hypomethylated
cg08288237	0.88252	0.659831892	-0.41953	4.30E-06	hypomethylated
cg03138287	0.86094	0.643656216	-0.419622	0.000191	hypomethylated
cg16410115	0.49339	0.368857297	-0.419666	0.000607	hypomethylated
cg01623485	0.91877	0.686860541	-0.419687	3.08E-06	hypomethylated
cg09314155	0.76502	0.571896757	-0.419743	3.76E-05	hypomethylated
cg04517258	0.70729	0.528732432	-0.419764	2.71E-05	hypomethylated
cg24607642	0.67439	0.504137297	-0.419766	0.000138	hypomethylated
cg07148024	0.87755	0.656001622	-0.419782	8.26E-06	hypomethylated

cg08048178	0.935	0.69892	-0.419839	2.79E-06	hypomethylated
cg02623114	0.55442	0.414415676	-0.419901	1.60E-05	hypomethylated
cg16879115	0.77389	0.578460541	-0.41991	0.000107	hypomethylated
cg23909079	0.81858	0.611864324	-0.419912	1.66E-05	hypomethylated
cg19484680	0.85262	0.637298378	-0.419934	5.28E-06	hypomethylated
cg23164850	0.65779	0.491655135	-0.41998	3.85E-05	hypomethylated
cg16692090	0.78986	0.590367027	-0.419985	0.000176	hypomethylated
cg15345388	0.78634	0.58772	-0.420024	5.38E-05	hypomethylated
cg21984645	0.83624	0.625014054	-0.420028	1.66E-05	hypomethylated
cg22956205	0.87314	0.652512973	-0.420206	1.46E-05	hypomethylated
cg23705155	0.79439	0.593641081	-0.420257	0.000417	hypomethylated
cg06447799	0.55109	0.411805405	-0.420325	7.65E-05	hypomethylated
cg26643476	0.81819	0.611387568	-0.420349	0.000204	hypomethylated
cg10182421	0.77851	0.581734054	-0.420356	0.00015	hypomethylated
cg07739927	0.77639	0.58012	-0.42043	0.000152	hypomethylated
cg26473110	0.6606	0.493597297	-0.420442	0.000102	hypomethylated
cg05885688	0.84632	0.632362162	-0.420452	4.70E-05	hypomethylated
cg07522403	0.90209	0.674032432	-0.420453	1.04E-05	hypomethylated
cg00356999	0.8952	0.668881081	-0.42046	2.87E-06	hypomethylated
cg11200568	0.86933	0.649527568	-0.420513	1.15E-05	hypomethylated
cg23418510	0.76214	0.569415676	-0.420574	4.26E-05	hypomethylated
cg24831391	0.96156	0.718360541	-0.420669	7.12E-06	hypomethylated
cg00007426	0.76015	0.567881622	-0.420694	3.57E-05	hypomethylated
cg11056766	0.66897	0.499762703	-0.420698	1.50E-05	hypomethylated
cg05793240	0.67209	0.502092973	-0.4207	5.79E-05	hypomethylated
cg01463644	0.70365	0.525666486	-0.42071	4.15E-05	hypomethylated
cg25428846	0.74024	0.552996216	-0.420723	0.000148	hypomethylated
cg02887458	0.62286	0.465305405	-0.42073	0.000145	hypomethylated
cg14571539	0.77195	0.576668649	-0.420765	9.95E-05	hypomethylated
cg16240816	0.7261	0.542384865	-0.420851	2.04E-05	hypomethylated
cg14771313	0.84931	0.634402703	-0.420892	5.89E-06	hypomethylated
cg09906448	0.71053	0.530731892	-0.420912	1.66E-05	hypomethylated
cg01331461	0.66001	0.492985405	-0.420943	1.99E-05	hypomethylated
cg03286387	0.52823	0.394552973	-0.420947	0.000245	hypomethylated
cg01119585	0.81716	0.610334595	-0.421018	3.26E-06	hypomethylated
cg01622018	0.51956	0.388055135	-0.421029	0.000109	hypomethylated
cg04662250	0.76929	0.574563784	-0.42106	1.75E-05	hypomethylated
cg00879790	0.71682	0.535304865	-0.42125	0.000369	hypomethylated
cg24497819	0.52921	0.395177838	-0.421338	0.002803	hypomethylated
cg09122223	0.82505	0.616071892	-0.421383	4.00E-05	hypomethylated
cg03343515	0.79878	0.596455135	-0.421385	1.39E-05	hypomethylated
cg01378182	0.52935	0.395267027	-0.421394	6.95E-05	hypomethylated
cg08319417	0.86896	0.648842162	-0.421422	8.03E-05	hypomethylated
cg16430819	0.78029	0.582588108	-0.421534	0.000245	hypomethylated
cg13996453	0.68815	0.513775135	-0.421586	0.000256	hypomethylated
cg01397912	0.68362	0.510345405	-0.421721	0.00043	hypomethylated
cg10665892	0.69253	0.516984865	-0.421755	0.000417	hypomethylated
cg19683073	0.84593	0.631482162	-0.421796	0.000112	hypomethylated
cg03164969	0.5196	0.387873514	-0.421815	6.39E-06	hypomethylated
cg14343701	0.75739	0.565370811	-0.421839	6.05E-06	hypomethylated
cg22989533	0.68703	0.512846486	-0.421846	0.00095	hypomethylated
cg08767936	0.81701	0.609847027	-0.421906	1.94E-05	hypomethylated
cg18010131	0.75867	0.56628	-0.421957	0.000365	hypomethylated
cg07056299	0.68228	0.509252973	-0.421981	0.001031	hypomethylated
cg15952840	0.76326	0.569670811	-0.422046	0.000129	hypomethylated
cg24842334	0.61696	0.460475676	-0.422052	0.001096	hypomethylated
cg21487099	0.63472	0.473714054	-0.422104	3.00E-05	hypomethylated
cg12073319	0.57059	0.425851351	-0.422105	0.000528	hypomethylated

cg16724070	0.85828	0.640558378	-0.422118	6.05E-06	hypomethylated
cg01832218	0.85114	0.635221622	-0.422136	4.01E-06	hypomethylated
cg08595656	0.55912	0.417237297	-0.42229	6.78E-05	hypomethylated
cg18709710	0.70903	0.529091351	-0.42233	0.00014	hypomethylated
cg09385936	0.83897	0.626031351	-0.422384	0.0002	hypomethylated
cg02812767	0.45586	0.340146486	-0.422435	0.000117	hypomethylated
cg24296397	0.6666	0.497375676	-0.422485	0.000306	hypomethylated
cg01941278	0.77767	0.580241081	-0.422506	0.000123	hypomethylated
cg14889070	0.81436	0.607600541	-0.422544	2.21E-05	hypomethylated
cg01825818	0.60882	0.454212432	-0.422649	0.002341	hypomethylated
cg24047802	0.57753	0.430862162	-0.422669	0.000172	hypomethylated
cg13876844	0.72552	0.541267027	-0.422675	0.00039	hypomethylated
cg02637086	0.79737	0.594845946	-0.422733	1.21E-05	hypomethylated
cg01795894	0.91316	0.681214054	-0.422759	2.87E-06	hypomethylated
cg00936935	0.70039	0.522487568	-0.422762	4.20E-05	hypomethylated
cg09528501	0.70226	0.523877297	-0.422776	0.000207	hypomethylated
cg01229943	0.7932	0.591708108	-0.422799	3.15E-05	hypomethylated
cg09226288	0.70221	0.523820541	-0.42283	1.64E-05	hypomethylated
cg11532182	0.63032	0.470178378	-0.422876	1.21E-05	hypomethylated
cg20531020	0.52032	0.38812	-0.422896	0.000271	hypomethylated
cg22676516	0.67584	0.504118919	-0.422918	0.000245	hypomethylated
cg10514593	0.8052	0.600591351	-0.422963	3.21E-06	hypomethylated
cg15318662	0.94888	0.707736757	-0.423013	1.22E-06	hypomethylated
cg02768671	0.53529	0.399252432	-0.42302	0.000129	hypomethylated
cg20443278	0.4747	0.354058378	-0.423029	1.84E-05	hypomethylated
cg15863841	0.74631	0.556630811	-0.423054	0.000182	hypomethylated
cg16031528	0.7335	0.547028649	-0.423181	2.48E-05	hypomethylated
cg01726448	0.84134	0.627444324	-0.423201	9.16E-05	hypomethylated
cg13567744	0.76941	0.573796757	-0.423213	5.19E-05	hypomethylated
cg08036899	0.81279	0.606142703	-0.423225	3.40E-05	hypomethylated
cg10874111	0.84066	0.626913514	-0.423256	3.95E-05	hypomethylated
cg18972123	0.54583	0.407046486	-0.423258	0.005316	hypomethylated
cg05275832	0.83925	0.625853514	-0.423276	0.000102	hypomethylated
cg17223520	0.67944	0.506678378	-0.423276	6.75E-06	hypomethylated
cg23278235	0.75734	0.56476973	-0.423278	9.19E-06	hypomethylated
cg25940485	0.86697	0.646512432	-0.423304	4.60E-06	hypomethylated
cg00961640	0.70555	0.526129189	-0.423331	0.0003	hypomethylated
cg02856688	0.59739	0.445467027	-0.423354	0.00504	hypomethylated
cg14099718	0.53401	0.398202703	-0.423364	2.57E-05	hypomethylated
cg23899779	0.62223	0.463971351	-0.423412	3.23E-05	hypomethylated
cg03398002	0.6042	0.450523784	-0.423423	0.000108	hypomethylated
cg17777603	0.71401	0.5324	-0.423434	0.00039	hypomethylated
cg19190762	0.65211	0.486241622	-0.423442	0.000138	hypomethylated
cg26857192	0.82023	0.611582162	-0.423482	2.23E-06	hypomethylated
cg00795830	0.73405	0.547295676	-0.423558	0.000148	hypomethylated
cg10847094	0.42996	0.320536757	-0.423713	0.001395	hypomethylated
cg19611163	0.65689	0.489702703	-0.423746	5.25E-05	hypomethylated
cg16659773	0.49616	0.369864324	-0.423809	0.000317	hypomethylated
cg08729279	0.8344	0.621992432	-0.423842	7.65E-05	hypomethylated
cg11928064	0.85323	0.636015676	-0.423872	3.71E-05	hypomethylated
cg15618646	0.66095	0.49268	-0.42389	0.000138	hypomethylated
cg11914650	0.91335	0.680795676	-0.423946	0.00028	hypomethylated
cg14305659	0.76485	0.570074054	-0.424027	3.69E-06	hypomethylated
cg06325540	0.75539	0.563021081	-0.424033	0.000214	hypomethylated
cg24815934	0.58366	0.435012432	-0.424072	0.000875	hypomethylated
cg11787522	0.50799	0.378606486	-0.424101	0.000159	hypomethylated
cg26066724	0.75853	0.565306486	-0.424173	6.38E-05	hypomethylated
cg23826980	0.70313	0.524015135	-0.424183	0.000256	hypomethylated

cg12252759	0.73798	0.549972432	-0.424222	5.31E-05	hypomethylated
cg25307724	0.91759	0.683810811	-0.424252	1.09E-05	hypomethylated
cg20646556	0.70928	0.528558919	-0.424291	6.95E-05	hypomethylated
cg03478610	0.85481	0.636991351	-0.42433	9.57E-06	hypomethylated
cg02654940	0.7069	0.526757838	-0.424366	0.001409	hypomethylated
cg12565635	0.81325	0.605997297	-0.424388	0.00024	hypomethylated
cg20660989	0.7282	0.542616216	-0.424403	0.000327	hypomethylated
cg11203990	0.63475	0.472974595	-0.424426	2.04E-05	hypomethylated
cg17221226	0.63004	0.469455135	-0.424456	0.000374	hypomethylated
cg03784611	0.95617	0.712453514	-0.424471	2.05E-06	hypomethylated
cg25046720	0.68427	0.509826486	-0.424559	0.00071	hypomethylated
cg13199127	0.73012	0.543977838	-0.424586	2.85E-05	hypomethylated
cg21137244	0.79158	0.589761081	-0.424605	2.04E-05	hypomethylated
cg14879645	0.75146	0.559862703	-0.424623	4.20E-05	hypomethylated
cg10057218	0.817	0.608687568	-0.424634	4.26E-05	hypomethylated
cg18131582	0.78844	0.587406486	-0.424642	0.000551	hypomethylated
cg12054171	0.93601	0.697302162	-0.42474	1.49E-06	hypomethylated
cg26469387	0.70974	0.528736216	-0.424742	0.001734	hypomethylated
cg09981198	0.74035	0.551524324	-0.424783	0.000123	hypomethylated
cg13908299	0.65616	0.488800541	-0.424802	1.01E-05	hypomethylated
cg11874976	0.83061	0.618749189	-0.424817	0.000262	hypomethylated
cg26109145	0.80245	0.597755676	-0.424856	4.05E-05	hypomethylated
cg03638937	0.93792	0.698627568	-0.424941	4.54E-06	hypomethylated
cg19735903	0.74089	0.551829189	-0.425038	0.000703	hypomethylated
cg26523175	0.71948	0.535870811	-0.425069	8.42E-05	hypomethylated
cg03630386	0.65432	0.487327568	-0.425105	5.12E-05	hypomethylated
cg22615730	0.80604	0.600321622	-0.425116	0.000166	hypomethylated
cg11772193	0.80972	0.603057297	-0.425128	3.21E-06	hypomethylated
cg10794973	0.59421	0.442542703	-0.425156	0.001912	hypomethylated
cg05622577	0.66579	0.495848649	-0.425167	0.000342	hypomethylated
cg04685253	0.78239	0.582679459	-0.425185	2.27E-05	hypomethylated
cg05631194	0.76334	0.568432432	-0.425337	0.000104	hypomethylated
cg15998505	0.63986	0.476473514	-0.42536	0.000135	hypomethylated
cg24662231	0.43251	0.322063784	-0.425387	5.32E-05	hypomethylated
cg25609480	0.90028	0.670364865	-0.425427	4.60E-06	hypomethylated
cg20293725	0.65605	0.488494595	-0.425463	0.000178	hypomethylated
cg08403419	0.60223	0.448402703	-0.42552	7.03E-05	hypomethylated
cg03918756	0.86751	0.645857838	-0.425664	7.52E-06	hypomethylated
cg10970500	0.85401	0.635736757	-0.425823	3.21E-06	hypomethylated
cg27499105	0.62278	0.463604324	-0.425829	0.000688	hypomethylated
cg11406388	0.95967	0.714386486	-0.425834	2.07E-06	hypomethylated
cg12793803	0.48591	0.361706486	-0.42587	0.002168	hypomethylated
cg06539091	0.66881	0.497830811	-0.425941	0.000129	hypomethylated
cg17981101	0.90517	0.673726486	-0.426026	2.42E-06	hypomethylated
cg10374402	0.81632	0.607571351	-0.426081	6.62E-05	hypomethylated
cg16519772	0.84586	0.629551892	-0.426094	1.99E-05	hypomethylated
cg09098720	0.66097	0.491942162	-0.426096	7.29E-05	hypomethylated
cg04884025	0.6048	0.450131351	-0.426112	0.000256	hypomethylated
cg03687765	0.64705	0.481568649	-0.426136	4.07E-06	hypomethylated
cg12960126	0.78276	0.582565405	-0.42615	5.35E-06	hypomethylated
cg05116443	0.92057	0.685109189	-0.426193	1.87E-05	hypomethylated
cg20698410	0.91429	0.680427568	-0.42621	1.15E-06	hypomethylated
cg05449217	0.46308	0.344629189	-0.426217	0.002502	hypomethylated
cg03051116	0.61838	0.460187568	-0.426272	0.000204	hypomethylated
cg24791666	0.54087	0.402471892	-0.426394	0.000789	hypomethylated
cg18497162	0.72751	0.541337297	-0.426439	7.29E-05	hypomethylated
cg14977069	0.72047	0.536088108	-0.426468	0.000251	hypomethylated
cg16731016	0.78869	0.586820541	-0.426539	0.00017	hypomethylated

cg17910564	0.75678	0.563070811	-0.426558	0.000435	hypomethylated
cg15718528	0.73109	0.543938919	-0.426604	7.84E-05	hypomethylated
cg07669969	0.66319	0.493415135	-0.42662	0.000104	hypomethylated
cg02283238	0.81281	0.604703243	-0.426691	0.000262	hypomethylated
cg22011888	0.85365	0.635078919	-0.426709	3.69E-06	hypomethylated
cg03970915	0.83832	0.623658919	-0.426744	2.39E-05	hypomethylated
cg00780520	0.87351	0.649826486	-0.42677	2.71E-05	hypomethylated
cg20987067	0.58527	0.43538	-0.426827	3.67E-05	hypomethylated
cg27219955	0.60992	0.453690811	-0.426911	3.15E-05	hypomethylated
cg16351441	0.89667	0.666961081	-0.426975	2.71E-05	hypomethylated
cg11132469	0.75498	0.561551892	-0.427019	4.82E-05	hypomethylated
cg08432509	0.66009	0.490967027	-0.427037	0.000256	hypomethylated
cg01859228	0.67302	0.500573514	-0.427067	2.85E-05	hypomethylated
cg15765889	0.87577	0.651348649	-0.427122	1.35E-05	hypomethylated
cg21576256	0.84342	0.627277838	-0.427147	1.47E-06	hypomethylated
cg25298754	0.75002	0.557797297	-0.427188	0.000626	hypomethylated
cg06564875	0.7686	0.571614054	-0.427192	0.00028	hypomethylated
cg12411858	0.65248	0.485242162	-0.427229	2.51E-05	hypomethylated
cg25210134	0.43211	0.321353514	-0.427237	0.002455	hypomethylated
cg26562921	0.6637	0.49356973	-0.427277	0.000726	hypomethylated
cg13553455	0.69858	0.519478378	-0.427362	0.000178	hypomethylated
cg22482278	0.96483	0.717463243	-0.42737	5.07E-06	hypomethylated
cg26646427	0.71428	0.531130811	-0.427423	2.71E-05	hypomethylated
cg19247032	0.69424	0.516204865	-0.427491	0.000109	hypomethylated
cg16098545	0.84303	0.626833514	-0.427502	2.10E-05	hypomethylated
cg13975093	0.88039	0.654597838	-0.427534	1.94E-05	hypomethylated
cg05364179	0.81538	0.606254595	-0.427549	6.15E-05	hypomethylated
cg03284102	0.79397	0.590332973	-0.427556	2.51E-05	hypomethylated
cg20445283	0.73049	0.543126486	-0.427576	2.64E-05	hypomethylated
cg01371233	0.92301	0.686255676	-0.4276	4.10E-05	hypomethylated
cg21007971	0.8253	0.613602703	-0.427614	1.99E-06	hypomethylated
cg20239639	0.67251	0.499992973	-0.427648	7.74E-05	hypomethylated
cg11218176	0.65225	0.484918919	-0.427681	2.64E-06	hypomethylated
cg11590932	0.84749	0.630064865	-0.427696	3.62E-05	hypomethylated
cg18818531	0.37318	0.277431892	-0.427738	6.08E-05	hypomethylated
cg19019345	0.66584	0.494985946	-0.427788	0.000296	hypomethylated
cg21079719	0.87244	0.648571892	-0.427789	2.13E-05	hypomethylated
cg15826891	0.87459	0.650124865	-0.42789	7.52E-06	hypomethylated
cg23193616	0.59804	0.444528649	-0.427966	4.05E-05	hypomethylated
cg17628894	0.46403	0.344911351	-0.427992	4.10E-05	hypomethylated
cg10151799	0.7611	0.565707568	-0.42803	6.31E-05	hypomethylated
cg13019844	0.47506	0.353068649	-0.428161	0.00018	hypomethylated
cg17431952	0.84527	0.628196216	-0.428197	2.51E-05	hypomethylated
cg14051181	0.67664	0.502868649	-0.428207	8.83E-06	hypomethylated
cg27174108	0.57385	0.426473514	-0.428218	9.27E-05	hypomethylated
cg15612947	0.76506	0.568551892	-0.428281	0.000528	hypomethylated
cg15450349	0.873	0.648754595	-0.428309	6.78E-05	hypomethylated
cg05347456	0.46437	0.345086486	-0.428317	0.001635	hypomethylated
cg23243343	0.7387	0.548924324	-0.428381	4.00E-05	hypomethylated
cg26049390	0.65017	0.483117297	-0.428443	0.00101	hypomethylated
cg07180475	0.6139	0.456157838	-0.428471	0.000182	hypomethylated
cg15329642	0.53045	0.394144865	-0.428491	9.72E-05	hypomethylated
cg26674445	0.6947	0.516180541	-0.428514	0.000159	hypomethylated
cg21251203	0.7386	0.548787027	-0.428547	5.06E-05	hypomethylated
cg03033543	0.8121	0.603366486	-0.428623	1.39E-05	hypomethylated
cg12603369	0.70893	0.526701081	-0.428659	0.000163	hypomethylated
cg07631533	0.61307	0.455470811	-0.428693	4.88E-05	hypomethylated
cg02753354	0.46926	0.34862	-0.428732	0.002126	hypomethylated

cg02334660	0.89817	0.667262162	-0.428735	1.88E-06	hypomethylated
cg01412886	0.56452	0.419363243	-0.428824	0.001949	hypomethylated
cg21900078	0.74728	0.555126486	-0.428832	8.15E-06	hypomethylated
cg27305009	0.66936	0.497231351	-0.428865	0.000454	hypomethylated
cg00942293	0.63836	0.474166486	-0.428977	2.64E-05	hypomethylated
cg12974832	0.6442	0.478502162	-0.428983	0.000875	hypomethylated
cg19240569	0.69879	0.51905027	-0.428985	0.000293	hypomethylated
cg15431934	0.4032	0.299488108	-0.428997	0.002856	hypomethylated
cg00259019	0.87615	0.650777838	-0.429013	2.15E-05	hypomethylated
cg18065174	0.93248	0.692617297	-0.429014	1.89E-05	hypomethylated
cg12496975	0.79201	0.588261081	-0.429062	0.000107	hypomethylated
cg13795689	0.65257	0.484678919	-0.429103	1.66E-05	hypomethylated
cg00181327	0.55273	0.410517297	-0.429132	0.000152	hypomethylated
cg11012543	0.77246	0.573711892	-0.429134	1.54E-05	hypomethylated
cg00731691	0.66168	0.491427568	-0.429155	1.82E-05	hypomethylated
cg21207450	0.77426	0.575031892	-0.429176	6.62E-05	hypomethylated
cg19642408	0.61734	0.458487027	-0.429184	0.000104	hypomethylated
cg04275506	0.77705	0.577097838	-0.429192	3.53E-05	hypomethylated
cg11247087	0.78691	0.584369189	-0.429319	1.84E-05	hypomethylated
cg06521149	0.85964	0.638368649	-0.429343	1.42E-05	hypomethylated
cg22531801	0.72077	0.535223243	-0.429398	0.001237	hypomethylated
cg14195117	0.55248	0.410233514	-0.429477	2.32E-05	hypomethylated
cg00187981	0.88479	0.656981622	-0.429482	2.78E-05	hypomethylated
cg11479035	0.93505	0.694287568	-0.42951	4.93E-06	hypomethylated
cg22954687	0.7158	0.53148973	-0.429515	0.001063	hypomethylated
cg09336922	0.73896	0.548684865	-0.429518	2.30E-05	hypomethylated
cg17399143	0.55848	0.414665405	-0.429558	0.000931	hypomethylated
cg17820025	0.58223	0.432287027	-0.4296	3.11E-05	hypomethylated
cg05114898	0.55096	0.409062703	-0.429626	1.58E-05	hypomethylated
cg19820609	0.61242	0.454688649	-0.429642	1.20E-05	hypomethylated
cg27648224	0.75947	0.563843784	-0.429697	3.15E-05	hypomethylated
cg06244306	0.89914	0.667509189	-0.429758	1.80E-05	hypomethylated
cg17488500	0.74424	0.552510811	-0.429765	6.46E-05	hypomethylated
cg11071912	0.66507	0.493711351	-0.429838	9.95E-05	hypomethylated
cg07352691	0.73952	0.548965946	-0.429873	2.39E-05	hypomethylated
cg27346988	0.62676	0.465250811	-0.429904	1.82E-05	hypomethylated
cg04476341	0.76493	0.567794595	-0.429959	7.93E-06	hypomethylated
cg06100324	0.84303	0.625755135	-0.429986	6.57E-06	hypomethylated
cg02331673	0.65776	0.488223784	-0.430019	1.71E-05	hypomethylated
cg18128495	0.74958	0.556349189	-0.430092	1.80E-05	hypomethylated
cg26949055	0.8639	0.641144324	-0.430215	5.93E-05	hypomethylated
cg13304638	0.80587	0.598074054	-0.430223	0.000256	hypomethylated
cg06080300	0.81768	0.606819459	-0.430269	1.25E-05	hypomethylated
cg18842310	0.59455	0.441207027	-0.430342	0.000172	hypomethylated
cg08711281	0.7357	0.545950811	-0.430347	6.57E-06	hypomethylated
cg01520454	0.69667	0.516977838	-0.430373	3.90E-06	hypomethylated
cg19196806	0.70976	0.526661622	-0.430455	2.39E-05	hypomethylated
cg03839782	0.81103	0.601766486	-0.430552	1.09E-05	hypomethylated
cg11158374	0.8173	0.606412432	-0.430566	4.73E-06	hypomethylated
cg16377880	0.70785	0.525193514	-0.430595	2.10E-05	hypomethylated
cg24806326	0.63591	0.471810811	-0.430614	0.000104	hypomethylated
cg20282814	0.76095	0.564581081	-0.430621	3.15E-05	hypomethylated
cg01120874	0.46742	0.346780541	-0.430697	0.001452	hypomethylated
cg04658243	0.71894	0.533361081	-0.430759	0.000399	hypomethylated
cg25424299	0.53398	0.396130811	-0.430809	5.79E-05	hypomethylated
cg00061635	0.65386	0.485063243	-0.430809	5.32E-05	hypomethylated
cg14244439	0.44795	0.332309189	-0.430812	0.001262	hypomethylated
cg08458711	0.72779	0.539904324	-0.430818	1.42E-05	hypomethylated

cg18373855	0.79976	0.593268649	-0.430882	0.00097	hypomethylated
cg00733115	0.42429	0.314730811	-0.430932	0.000293	hypomethylated
cg24119674	0.54159	0.401739459	-0.430941	0.000176	hypomethylated
cg26431550	0.60002	0.445065946	-0.430991	0.000159	hypomethylated
cg25251459	0.79897	0.592576757	-0.431139	9.95E-05	hypomethylated
cg12588880	0.48958	0.36309027	-0.431216	0.00054	hypomethylated
cg02863988	0.65832	0.48823027	-0.431227	0.00017	hypomethylated
cg09038599	0.74423	0.551942162	-0.431231	0.000495	hypomethylated
cg04930921	0.83909	0.622268108	-0.431289	4.47E-05	hypomethylated
cg17960629	0.50364	0.373492973	-0.431312	7.47E-05	hypomethylated
cg01057945	0.65608	0.48653027	-0.431342	3.85E-05	hypomethylated
cg05587926	0.89322	0.662368649	-0.431381	6.48E-06	hypomethylated
cg12807237	0.69615	0.516227568	-0.431391	9.57E-06	hypomethylated
cg25424279	0.61787	0.458176757	-0.431399	0.0006	hypomethylated
cg10907866	0.77158	0.572152432	-0.431416	0.000259	hypomethylated
cg14760020	0.67752	0.502401622	-0.431422	0.000104	hypomethylated
cg04299274	0.58562	0.434245405	-0.431454	6.31E-05	hypomethylated
cg03440799	0.86787	0.643535135	-0.43146	4.26E-05	hypomethylated
cg15663823	0.85466	0.633738919	-0.431462	7.72E-06	hypomethylated
cg22116041	0.59822	0.44356	-0.431547	0.00395	hypomethylated
cg08968034	0.59896	0.44409027	-0.431607	7.12E-05	hypomethylated
cg08949735	0.7407	0.549148108	-0.431694	3.21E-06	hypomethylated
cg19459434	0.78391	0.58117027	-0.431727	5.58E-06	hypomethylated
cg03698144	0.74208	0.550157297	-0.431731	3.67E-05	hypomethylated
cg25259564	0.95287	0.706425405	-0.431742	3.62E-05	hypomethylated
cg19710662	0.60324	0.447218919	-0.431751	1.84E-05	hypomethylated
cg15428904	0.80257	0.594944324	-0.431873	1.89E-05	hypomethylated
cg22065124	0.86637	0.642211892	-0.431934	1.31E-05	hypomethylated
cg01447427	0.50369	0.373368108	-0.431937	0.000202	hypomethylated
cg04295322	0.64958	0.481503784	-0.43196	4.58E-05	hypomethylated
cg01917266	0.80077	0.593569189	-0.431972	5.19E-05	hypomethylated
cg24478966	0.94812	0.702783784	-0.431989	5.28E-06	hypomethylated
cg15016481	0.74717	0.553795676	-0.432083	8.22E-05	hypomethylated
cg16105591	0.56098	0.415791351	-0.43209	0.000262	hypomethylated
cg24109894	0.73877	0.547549189	-0.432137	0.00012	hypomethylated
cg08429256	0.60997	0.452048649	-0.43226	0.000221	hypomethylated
cg13680337	0.5242	0.388481081	-0.432273	4.64E-05	hypomethylated
cg07843568	0.71158	0.527341622	-0.432288	2.32E-05	hypomethylated
cg10061384	0.65028	0.481902162	-0.432321	1.01E-05	hypomethylated
cg17156828	0.85597	0.634321622	-0.432346	3.57E-05	hypomethylated
cg04221650	0.7984	0.591648649	-0.432371	2.81E-05	hypomethylated
cg11702866	0.6368	0.471882703	-0.432412	0.000667	hypomethylated
cg00259234	0.95747	0.709503784	-0.432417	3.64E-06	hypomethylated
cg08687163	0.67048	0.496836757	-0.432422	1.99E-05	hypomethylated
cg14437634	0.84948	0.629472973	-0.432436	8.22E-05	hypomethylated
cg26578156	0.78706	0.583211892	-0.432453	3.40E-05	hypomethylated
cg26472036	0.82898	0.614272973	-0.432457	6.78E-05	hypomethylated
cg26305174	0.73492	0.544539459	-0.432551	0.000327	hypomethylated
cg01279632	0.71797	0.531958378	-0.43261	8.71E-06	hypomethylated
cg26567736	0.60734	0.449980541	-0.432642	0.000495	hypomethylated
cg01446731	0.80905	0.599404324	-0.432699	6.15E-05	hypomethylated
cg06726231	0.71251	0.527879459	-0.432702	2.78E-05	hypomethylated
cg23875404	0.52903	0.391922703	-0.43278	0.000501	hypomethylated
cg18330338	0.7175	0.531539459	-0.432802	5.72E-05	hypomethylated
cg15825304	0.50641	0.375147027	-0.43285	0.001541	hypomethylated
cg06875832	0.90979	0.673958919	-0.432873	8.37E-06	hypomethylated
cg19474571	0.49719	0.368302703	-0.432905	3.67E-05	hypomethylated
cg20911165	0.83829	0.620958378	-0.432953	4.36E-05	hypomethylated

cg02377915	0.73269	0.542720541	-0.432993	1.60E-05	hypomethylated
cg05488681	0.847	0.627392432	-0.432994	8.37E-06	hypomethylated
cg13316404	0.72131	0.534282703	-0.433016	1.94E-05	hypomethylated
cg25113767	0.48356	0.35817027	-0.433049	0.000143	hypomethylated
cg23881697	0.69348	0.51362	-0.433153	2.15E-05	hypomethylated
cg10025830	0.36556	0.270735135	-0.433226	0.000902	hypomethylated
cg13273136	0.50791	0.37614	-0.433303	0.002386	hypomethylated
cg00080125	0.6627	0.49077027	-0.433308	0.000696	hypomethylated
cg17866650	0.88175	0.652975135	-0.433342	1.12E-06	hypomethylated
cg03467825	0.60877	0.450814595	-0.433363	0.000187	hypomethylated
cg00599124	0.67266	0.498096216	-0.433453	0.000718	hypomethylated
cg21346966	0.50568	0.374408108	-0.433613	6.78E-05	hypomethylated
cg00525683	0.75358	0.557944865	-0.433638	0.00028	hypomethylated
cg02694576	0.91513	0.677518919	-0.433715	3.76E-05	hypomethylated
cg13375571	0.66595	0.493003784	-0.433815	0.00102	hypomethylated
cg14790115	0.70065	0.518686486	-0.433831	0.000464	hypomethylated
cg03717367	0.58984	0.436624324	-0.433931	0.00035	hypomethylated
cg11772804	0.65705	0.486367568	-0.433956	4.73E-06	hypomethylated
cg05570141	0.7693	0.56944	-0.434002	0.000528	hypomethylated
cg08412316	0.86926	0.643394595	-0.434084	4.86E-06	hypomethylated
cg03137629	0.80844	0.598345405	-0.434162	0.00013	hypomethylated
cg10696677	0.57472	0.425354595	-0.434193	0.000102	hypomethylated
cg16397638	0.92861	0.687238919	-0.434261	5.58E-06	hypomethylated
cg03758633	0.7696	0.569559459	-0.434262	0.00029	hypomethylated
cg14339650	0.57313	0.424156216	-0.434267	8.22E-05	hypomethylated
cg14397813	0.86059	0.636884324	-0.434295	2.27E-05	hypomethylated
cg08594606	0.95184	0.704335135	-0.434457	5.07E-06	hypomethylated
cg22524864	0.93301	0.690320541	-0.434626	1.15E-05	hypomethylated
cg05894719	0.86573	0.640517838	-0.434678	3.21E-06	hypomethylated
cg07828024	0.7564	0.55960973	-0.434728	0.000172	hypomethylated
cg01135165	0.88669	0.655969189	-0.434802	2.32E-05	hypomethylated
cg09043456	0.53073	0.392628108	-0.434815	8.84E-05	hypomethylated
cg25423174	0.62784	0.464461622	-0.434838	1.71E-05	hypomethylated
cg16388983	0.80744	0.597303243	-0.434892	0.000185	hypomethylated
cg09611620	0.737	0.54516973	-0.434959	0.003316	hypomethylated
cg25446191	0.90431	0.668926486	-0.43497	1.72E-06	hypomethylated
cg26609120	0.55088	0.407483243	-0.434997	0.007799	hypomethylated
cg06430753	0.68688	0.508038919	-0.435119	0.000408	hypomethylated
cg01766718	0.89128	0.659178378	-0.43521	1.06E-05	hypomethylated
cg05368724	0.7718	0.570782162	-0.435287	1.29E-06	hypomethylated
cg12439423	0.72175	0.533767568	-0.435288	2.71E-05	hypomethylated
cg24710671	0.83215	0.615396757	-0.435327	6.57E-06	hypomethylated
cg18767964	0.66922	0.494903243	-0.435334	2.81E-05	hypomethylated
cg03540589	0.59311	0.438611892	-0.435355	0.000296	hypomethylated
cg06720722	0.77467	0.572877297	-0.435356	0.000408	hypomethylated
cg02144647	0.82653	0.611227568	-0.435358	2.45E-05	hypomethylated
cg18642361	0.9066	0.670419459	-0.435402	1.70E-06	hypomethylated
cg11053466	0.72765	0.538062703	-0.43547	1.06E-05	hypomethylated
cg23012855	0.8747	0.646795135	-0.435479	1.58E-05	hypomethylated
cg01874334	0.71379	0.527807568	-0.435488	4.26E-05	hypomethylated
cg11043993	0.65906	0.487320541	-0.435539	7.12E-06	hypomethylated
cg24579589	0.55751	0.412230811	-0.435545	1.21E-05	hypomethylated
cg07167933	0.63467	0.469260541	-0.435617	4.10E-05	hypomethylated
cg01196079	0.58971	0.436013514	-0.435633	1.46E-05	hypomethylated
cg17837192	0.69888	0.516721622	-0.435658	0.001395	hypomethylated
cg04737124	0.79929	0.590934595	-0.435721	0.000245	hypomethylated
cg15923943	0.60388	0.446462162	-0.435724	0.00017	hypomethylated
cg26585416	0.71037	0.525152973	-0.435833	0.000464	hypomethylated

cg09981464	0.88932	0.657437297	-0.435849	1.46E-05	hypomethylated
cg24845165	0.73435	0.542862703	-0.43588	0.00012	hypomethylated
cg23981702	0.82251	0.608020541	-0.435913	0.00012	hypomethylated
cg21235678	0.75319	0.556768108	-0.435937	0.000209	hypomethylated
cg16703220	0.84682	0.625980541	-0.435938	4.15E-05	hypomethylated
cg00249974	0.78784	0.582341081	-0.436038	4.70E-05	hypomethylated
cg00172803	0.73514	0.543374595	-0.436072	1.18E-05	hypomethylated
cg15955277	0.96172	0.710828108	-0.436116	4.73E-06	hypomethylated
cg04177684	0.56592	0.418282703	-0.43612	0.000163	hypomethylated
cg19251326	0.67976	0.502417297	-0.436139	2.57E-05	hypomethylated
cg23250019	0.73987	0.546826486	-0.436189	2.38E-05	hypomethylated
cg17250770	0.78731	0.58187027	-0.436234	1.40E-05	hypomethylated
cg02137956	0.75365	0.556968108	-0.4363	7.84E-05	hypomethylated
cg25376593	0.8969	0.662827568	-0.436314	3.08E-06	hypomethylated
cg01930947	0.88027	0.650529189	-0.436332	8.60E-06	hypomethylated
cg15704408	0.80205	0.592715135	-0.436353	8.60E-06	hypomethylated
cg10721942	0.75593	0.558628649	-0.436363	9.07E-06	hypomethylated
cg00858400	0.69501	0.513601081	-0.436385	0.000464	hypomethylated
cg16746901	0.87538	0.646887027	-0.436396	0.000152	hypomethylated
cg11230112	0.87527	0.64678	-0.436453	4.26E-05	hypomethylated
cg08070476	0.80832	0.597283784	-0.43651	0.000126	hypomethylated
cg16675525	0.48674	0.359654595	-0.436539	0.000528	hypomethylated
cg13702185	0.86479	0.638992973	-0.43655	1.82E-06	hypomethylated
cg07560741	0.93001	0.687173514	-0.436572	1.94E-05	hypomethylated
cg18298997	0.86738	0.640883784	-0.436601	5.50E-06	hypomethylated
cg01314165	0.91039	0.672630811	-0.43667	6.57E-06	hypomethylated
cg13335567	0.75681	0.559159459	-0.436671	0.00064	hypomethylated
cg14285881	0.70459	0.52057027	-0.436691	3.85E-05	hypomethylated
cg02976843	0.66624	0.492231892	-0.436704	3.76E-05	hypomethylated
cg15512715	0.77211	0.570429189	-0.436759	1.25E-05	hypomethylated
cg20837735	0.8505	0.628333514	-0.436781	0.000123	hypomethylated
cg26389955	0.74689	0.551731892	-0.436928	3.85E-05	hypomethylated
cg16295461	0.86197	0.636675135	-0.43708	2.21E-05	hypomethylated
cg20331980	0.68557	0.506381081	-0.43708	5.06E-05	hypomethylated
cg00920938	0.5497	0.406013514	-0.437117	0.001803	hypomethylated
cg09207296	0.80847	0.597138378	-0.437129	2.85E-05	hypomethylated
cg08196138	0.45764	0.337988649	-0.437238	0.000135	hypomethylated
cg26175343	0.56516	0.417387568	-0.437272	0.000256	hypomethylated
cg17482237	0.89524	0.661144324	-0.437309	5.58E-06	hypomethylated
cg14254767	0.53442	0.394667568	-0.437336	7.41E-06	hypomethylated
cg03058163	0.81378	0.600945946	-0.437404	8.37E-06	hypomethylated
cg13285077	0.67945	0.501740541	-0.437426	1.46E-05	hypomethylated
cg15353603	0.6437	0.475323243	-0.43748	6.95E-05	hypomethylated
cg18642234	0.67961	0.501777297	-0.43766	0.000893	hypomethylated
cg10721755	0.77393	0.571414054	-0.437667	6.70E-05	hypomethylated
cg19512969	0.76803	0.567056216	-0.437671	0.000369	hypomethylated
cg02258534	0.72521	0.53542	-0.437728	6.31E-05	hypomethylated
cg21197796	0.75919	0.56048	-0.437798	3.85E-05	hypomethylated
cg08362283	0.69518	0.513208649	-0.437841	3.62E-05	hypomethylated
cg02653521	0.87145	0.643326486	-0.437867	8.63E-05	hypomethylated
cg22749855	0.69705	0.514557838	-0.437929	0.000271	hypomethylated
cg22583147	0.72985	0.538764865	-0.437944	9.72E-05	hypomethylated
cg09036996	0.57069	0.421266486	-0.437974	0.0003	hypomethylated
cg08696192	0.86246	0.636624865	-0.438014	4.18E-06	hypomethylated
cg25348105	0.59525	0.43938	-0.438027	0.000931	hypomethylated
cg16451803	0.63872	0.471454054	-0.438066	0.000293	hypomethylated
cg27292264	0.48407	0.357296757	-0.438093	5.25E-05	hypomethylated
cg09432154	0.63616	0.469536757	-0.438152	2.71E-05	hypomethylated

cg22887880	0.74095	0.546873514	-0.438169	2.57E-05	hypomethylated
cg16452248	0.95939	0.708062703	-0.43824	1.15E-06	hypomethylated
cg01670771	0.72835	0.537541081	-0.438257	5.19E-05	hypomethylated
cg08737189	0.77494	0.571904865	-0.438309	1.66E-05	hypomethylated
cg05903710	0.7157	0.52814973	-0.438408	0.000166	hypomethylated
cg00507135	0.84186	0.621236757	-0.438437	4.76E-05	hypomethylated
cg13705556	0.79838	0.589149189	-0.438443	3.62E-05	hypomethylated
cg14754494	0.8019	0.591676216	-0.438614	4.10E-05	hypomethylated
cg16073143	0.81714	0.602861622	-0.438756	9.72E-05	hypomethylated
cg18432572	0.74629	0.550578919	-0.438787	3.69E-06	hypomethylated
cg18161025	0.78486	0.579027568	-0.438803	9.57E-06	hypomethylated
cg06294416	0.82518	0.608728649	-0.43891	6.05E-06	hypomethylated
cg02928885	0.83334	0.614722703	-0.438969	5.93E-05	hypomethylated
cg26834627	0.86056	0.634751892	-0.439083	1.89E-05	hypomethylated
cg01927000	0.87337	0.644197838	-0.439089	1.01E-05	hypomethylated
cg11726507	0.82485	0.608387568	-0.439141	0.000262	hypomethylated
cg23778358	0.9688	0.714559459	-0.439145	9.24E-07	hypomethylated
cg17747879	0.55339	0.408157297	-0.439171	0.00028	hypomethylated
cg03795574	0.74295	0.547945946	-0.439232	0.000756	hypomethylated
cg10965749	0.63334	0.467105405	-0.439232	2.39E-05	hypomethylated
cg04519870	0.66086	0.487400541	-0.439237	1.84E-05	hypomethylated
cg08391380	0.92097	0.679225946	-0.439263	1.58E-06	hypomethylated
cg18174530	0.68231	0.503210811	-0.439264	4.01E-06	hypomethylated
cg05312962	0.64775	0.477719459	-0.439273	0.000174	hypomethylated
cg07443717	0.80106	0.590734595	-0.4394	2.64E-05	hypomethylated
cg25128781	0.5516	0.40676973	-0.43941	0.000324	hypomethylated
cg05695995	0.51287	0.378192432	-0.439473	2.96E-05	hypomethylated
cg20668765	0.61543	0.453817838	-0.439481	5.65E-05	hypomethylated
cg01473602	0.51851	0.382342162	-0.439508	0.000718	hypomethylated
cg22445712	0.54993	0.405497838	-0.439554	0.000293	hypomethylated
cg02724909	0.65267	0.481252432	-0.43956	1.75E-05	hypomethylated
cg13405631	0.5215	0.384531892	-0.439564	0.001571	hypomethylated
cg03015585	0.91168	0.672225405	-0.439582	2.29E-06	hypomethylated
cg23985447	0.69392	0.511647568	-0.439619	0.002066	hypomethylated
cg06221946	0.68043	0.501653514	-0.439755	0.000417	hypomethylated
cg01034813	0.7855	0.579105946	-0.439784	4.10E-05	hypomethylated
cg27404351	0.67718	0.499241622	-0.439801	6.78E-05	hypomethylated
cg09636245	0.61942	0.456654054	-0.439816	0.003078	hypomethylated
cg05138403	0.83166	0.613111351	-0.439845	8.42E-05	hypomethylated
cg01300852	0.65929	0.486034595	-0.439854	0.00039	hypomethylated
cg05504025	0.72563	0.534934054	-0.439873	6.39E-06	hypomethylated
cg08608523	0.60978	0.449529189	-0.439874	1.54E-05	hypomethylated
cg13158199	0.84441	0.622494054	-0.439884	3.04E-06	hypomethylated
cg16738984	0.64205	0.473287568	-0.439969	1.89E-05	hypomethylated
cg07701049	0.80138	0.590703784	-0.440052	2.71E-05	hypomethylated
cg24974982	0.68366	0.503901081	-0.440138	0.000262	hypomethylated
cg16527877	0.74751	0.550938919	-0.4402	0.00015	hypomethylated
cg11800672	0.78453	0.578207568	-0.440241	0.000313	hypomethylated
cg16090599	0.53488	0.394194054	-0.440309	7.84E-05	hypomethylated
cg01869342	0.88294	0.650703784	-0.440314	2.67E-05	hypomethylated
cg24097814	0.88479	0.652067027	-0.440315	1.52E-05	hypomethylated
cg11411884	0.9379	0.691191892	-0.440348	6.05E-06	hypomethylated
cg09146232	0.91202	0.672072432	-0.440449	2.49E-06	hypomethylated
cg19450056	0.79763	0.587747568	-0.440523	3.40E-05	hypomethylated
cg19573490	0.75642	0.557316757	-0.44069	0.000166	hypomethylated
cg11069071	0.74151	0.54630973	-0.440747	1.18E-05	hypomethylated
cg18819791	0.74477	0.548661622	-0.440878	0.000234	hypomethylated
cg14301030	0.83642	0.616159459	-0.440924	1.39E-05	hypomethylated

cg06295784	0.7763	0.571818919	-0.441056	2.87E-06	hypomethylated
cg18508425	0.63341	0.466558919	-0.44108	3.76E-05	hypomethylated
cg01252713	0.8653	0.637328649	-0.441163	5.50E-06	hypomethylated
cg23172911	0.70169	0.516801622	-0.441223	0.00035	hypomethylated
cg14575790	0.81062	0.597025946	-0.441232	1.39E-05	hypomethylated
cg02780210	0.50792	0.374077838	-0.441263	0.000454	hypomethylated
cg16294363	0.77909	0.573698919	-0.441496	4.15E-05	hypomethylated
cg23822312	0.81935	0.603321081	-0.441554	2.87E-06	hypomethylated
cg25907138	0.55493	0.408614595	-0.441565	3.85E-05	hypomethylated
cg08090621	0.74331	0.547303243	-0.441624	0.000129	hypomethylated
cg09678829	0.89251	0.657137838	-0.441672	4.86E-06	hypomethylated
cg08821747	0.75293	0.554358919	-0.441695	2.78E-05	hypomethylated
cg05412410	0.71557	0.526783243	-0.441883	8.84E-05	hypomethylated
cg25840173	0.76133	0.560454595	-0.441924	4.15E-05	hypomethylated
cg14673936	0.72046	0.530334054	-0.442017	5.65E-06	hypomethylated
cg20092936	0.93255	0.686437297	-0.442053	3.95E-05	hypomethylated
cg02639435	0.93575	0.68879027	-0.442058	1.23E-05	hypomethylated
cg25481157	0.61663	0.453877297	-0.442103	0.000613	hypomethylated
cg11801110	0.63906	0.470362703	-0.442178	7.74E-05	hypomethylated
cg16147544	0.71223	0.524200541	-0.442224	3.59E-06	hypomethylated
cg15677087	0.82877	0.609956216	-0.442266	0.000563	hypomethylated
cg16269010	0.52046	0.383037297	-0.442302	6.39E-06	hypomethylated
cg26351966	0.43606	0.320902162	-0.442393	0.002649	hypomethylated
cg00112314	0.56287	0.414223243	-0.442393	0.000126	hypomethylated
cg05765734	0.85721	0.630826486	-0.442405	2.21E-05	hypomethylated
cg27423177	0.64418	0.474043243	-0.442445	0.000469	hypomethylated
cg25668117	0.84586	0.622447568	-0.442467	4.48E-06	hypomethylated
cg00508965	0.77314	0.568925946	-0.442489	9.69E-06	hypomethylated
cg24764810	0.71502	0.526155676	-0.442494	2.71E-05	hypomethylated
cg00869330	0.80577	0.592921081	-0.442528	0.000102	hypomethylated
cg10891521	0.65002	0.47830973	-0.442539	4.88E-05	hypomethylated
cg14407179	0.82145	0.604353514	-0.44278	0.000866	hypomethylated
cg03981685	0.75072	0.552296216	-0.442833	0.000667	hypomethylated
cg03631656	0.62351	0.458706486	-0.442841	0.00095	hypomethylated
cg08866557	0.85512	0.629096757	-0.442845	2.85E-05	hypomethylated
cg00966920	0.83003	0.610625405	-0.442876	7.12E-05	hypomethylated
cg09539496	0.67293	0.495046486	-0.442892	0.000474	hypomethylated
cg15948030	0.60873	0.447773514	-0.443033	0.00035	hypomethylated
cg15736553	0.51906	0.38180973	-0.443047	9.27E-05	hypomethylated
cg01152374	0.80068	0.588957297	-0.443063	2.92E-05	hypomethylated
cg00077904	0.52756	0.388035135	-0.443148	3.49E-05	hypomethylated
cg13650740	0.55943	0.411472973	-0.44316	0.000342	hypomethylated
cg07945582	0.74782	0.550034595	-0.443169	0.000129	hypomethylated
cg16850150	0.4693	0.345177297	-0.443173	4.47E-05	hypomethylated
cg06761584	0.77057	0.566755135	-0.4432	1.84E-05	hypomethylated
cg13630114	0.80434	0.591556757	-0.443289	5.45E-05	hypomethylated
cg21147708	0.70393	0.517700541	-0.443314	0.000161	hypomethylated
cg10208113	0.87834	0.645951892	-0.443353	4.12E-06	hypomethylated
cg10449466	0.69703	0.5126	-0.443387	0.000174	hypomethylated
cg15726025	0.86817	0.638434054	-0.44344	7.32E-06	hypomethylated
cg01810863	0.44625	0.328107568	-0.443683	0.000123	hypomethylated
cg01974549	0.79714	0.586072973	-0.443753	2.78E-05	hypomethylated
cg25407876	0.6455	0.474582162	-0.443759	0.000469	hypomethylated
cg24278841	0.76836	0.564886486	-0.443821	4.12E-06	hypomethylated
cg22914057	0.87552	0.643657297	-0.443847	1.99E-06	hypomethylated
cg14065109	0.64214	0.472082703	-0.443848	0.001571	hypomethylated
cg14651650	0.80593	0.592491351	-0.44386	4.15E-05	hypomethylated
cg09180877	0.71151	0.523067568	-0.443887	4.60E-06	hypomethylated

cg12003463	0.7791	0.572756216	-0.443887	7.83E-06	hypomethylated
cg07978738	0.54338	0.399427568	-0.444028	0.000358	hypomethylated
cg00238570	0.79769	0.586363784	-0.444032	3.85E-05	hypomethylated
cg22057234	0.39531	0.290567568	-0.444111	8.94E-05	hypomethylated
cg26701815	0.7471	0.549085405	-0.444271	0.00017	hypomethylated
cg06341100	0.80803	0.593855135	-0.444298	7.20E-05	hypomethylated
cg21189611	0.87951	0.646358378	-0.444366	4.26E-05	hypomethylated
cg19904905	0.77243	0.567654595	-0.444391	0.000317	hypomethylated
cg01008256	0.59861	0.439858919	-0.444576	0.000365	hypomethylated
cg01699293	0.90172	0.662573514	-0.444599	3.30E-06	hypomethylated
cg12475590	0.88672	0.651504865	-0.444703	6.57E-06	hypomethylated
cg10758902	0.94593	0.695007568	-0.444705	3.39E-06	hypomethylated
cg04770370	0.53416	0.392434054	-0.444822	7.12E-06	hypomethylated
cg15353533	0.7786	0.572012973	-0.444834	1.37E-06	hypomethylated
cg01647204	0.60487	0.444355676	-0.44491	0.000229	hypomethylated
cg00515905	0.86089	0.632408108	-0.444973	5.00E-06	hypomethylated
cg24599065	0.49091	0.360614595	-0.445001	0.001635	hypomethylated
cg20327784	0.69184	0.508186486	-0.44508	1.75E-05	hypomethylated
cg15809077	0.87362	0.641707568	-0.44509	1.62E-05	hypomethylated
cg01767862	0.86062	0.632138919	-0.445135	3.39E-06	hypomethylated
cg26147935	0.86535	0.635598919	-0.445167	7.12E-05	hypomethylated
cg18526870	0.81648	0.599668108	-0.445253	1.75E-05	hypomethylated
cg24755459	0.71255	0.523329189	-0.445273	0.000176	hypomethylated
cg15259904	0.78503	0.576492432	-0.445446	0.000245	hypomethylated
cg14405137	0.60746	0.44604973	-0.445585	0.012637	hypomethylated
cg25546428	0.8136	0.597402162	-0.445617	9.07E-06	hypomethylated
cg00127781	0.64975	0.47702	-0.445835	1.15E-06	hypomethylated
cg24667756	0.76797	0.563806486	-0.44585	0.000237	hypomethylated
cg19867579	0.66792	0.490345946	-0.445875	0.003021	hypomethylated
cg19777001	0.86381	0.634148108	-0.445894	2.71E-05	hypomethylated
cg27255275	0.71351	0.523807027	-0.445898	0.000132	hypomethylated
cg04022194	0.62777	0.460844865	-0.445955	5.73E-06	hypomethylated
cg21606527	0.72608	0.532976216	-0.446057	7.12E-05	hypomethylated
cg09692449	0.64068	0.470286486	-0.446064	0.000163	hypomethylated
cg18995788	0.87662	0.643455135	-0.446112	0.000147	hypomethylated
cg09773238	0.70673	0.518724324	-0.446191	7.84E-05	hypomethylated
cg10133725	0.72428	0.531571892	-0.446283	0.000495	hypomethylated
cg07573872	0.73578	0.539998919	-0.446318	0.000224	hypomethylated
cg03769927	0.76167	0.558949189	-0.446449	0.000253	hypomethylated
cg18555142	0.79926	0.586481081	-0.44658	2.29E-06	hypomethylated
cg02558476	0.57978	0.425416757	-0.446629	0.00018	hypomethylated
cg02286809	0.81201	0.595806486	-0.446654	3.32E-05	hypomethylated
cg13168012	0.50711	0.372087568	-0.446657	6.54E-05	hypomethylated
cg10005565	0.79526	0.583508649	-0.446673	1.80E-05	hypomethylated
cg23424357	0.84745	0.621800541	-0.446676	2.42E-06	hypomethylated
cg26688315	0.80242	0.588740541	-0.446726	2.30E-05	hypomethylated
cg12188416	0.67922	0.498346486	-0.44673	0.002318	hypomethylated
cg02589576	0.60112	0.441034054	-0.446763	3.57E-05	hypomethylated
cg02285155	0.72918	0.534975135	-0.446803	0.000797	hypomethylated
cg14079243	0.5628	0.412885946	-0.446879	8.32E-05	hypomethylated
cg14361654	0.62669	0.45974973	-0.446903	2.32E-05	hypomethylated
cg08836861	0.69884	0.512677297	-0.446911	0.000129	hypomethylated
cg15036326	0.73197	0.536979459	-0.446918	0.00017	hypomethylated
cg20108855	0.80532	0.590761081	-0.446987	1.39E-05	hypomethylated
cg13357821	0.73127	0.536433514	-0.447005	9.31E-06	hypomethylated
cg08755026	0.85899	0.630120541	-0.447013	5.28E-06	hypomethylated
cg10995381	0.67853	0.497735676	-0.447033	2.27E-05	hypomethylated
cg24700219	0.68982	0.505986486	-0.447121	4.36E-05	hypomethylated

cg02001060	0.61912	0.454126486	-0.447125	6.62E-05	hypomethylated
cg01574788	0.58128	0.426364324	-0.447147	0.0002	hypomethylated
cg12800047	0.78092	0.57279027	-0.447168	0.000224	hypomethylated
cg07086226	0.76265	0.559340541	-0.447294	4.88E-05	hypomethylated
cg14778967	0.74563	0.546856757	-0.447297	0.000485	hypomethylated
cg00253228	0.50559	0.370805405	-0.447306	0.003808	hypomethylated
cg14505256	0.8338	0.611515135	-0.447313	1.09E-05	hypomethylated
cg18303608	0.66208	0.485558919	-0.447359	0.000135	hypomethylated
cg16754857	0.79912	0.586039459	-0.447414	1.09E-05	hypomethylated
cg18915450	0.86796	0.636521081	-0.44742	2.64E-05	hypomethylated
cg12743248	0.74535	0.546556757	-0.447547	0.000112	hypomethylated
cg15698244	0.76386	0.560111351	-0.447595	1.01E-05	hypomethylated
cg08498836	0.83079	0.609178378	-0.447619	2.18E-05	hypomethylated
cg24298696	0.88963	0.652316216	-0.447634	2.64E-06	hypomethylated
cg04510919	0.79432	0.582410811	-0.447683	3.95E-05	hypomethylated
cg18186672	0.66906	0.490563784	-0.447695	0.003571	hypomethylated
cg19414383	0.62361	0.457217297	-0.447764	0.001571	hypomethylated
cg21931317	0.92194	0.675930811	-0.447797	8.15E-06	hypomethylated
cg01452581	0.79176	0.580435676	-0.447927	1.18E-06	hypomethylated
cg21547649	0.50471	0.369985946	-0.447984	0.000189	hypomethylated
cg17481703	0.70538	0.51708	-0.448013	0.000152	hypomethylated
cg23330006	0.62601	0.458879459	-0.44807	0.000772	hypomethylated
cg14802951	0.63272	0.463795676	-0.448078	1.12E-05	hypomethylated
cg26557179	0.536	0.392894054	-0.448093	0.006339	hypomethylated
cg22384356	0.68942	0.505348649	-0.448104	1.01E-05	hypomethylated
cg12402318	0.74985	0.549641622	-0.448111	1.94E-05	hypomethylated
cg25863289	0.94679	0.693987568	-0.448135	5.35E-06	hypomethylated
cg24158844	0.49167	0.360388649	-0.448137	0.002147	hypomethylated
cg27302373	0.8418	0.617029189	-0.448139	3.95E-05	hypomethylated
cg13941508	0.91211	0.668535135	-0.448204	3.85E-06	hypomethylated
cg10075819	0.56525	0.414292432	-0.44824	0.000166	hypomethylated
cg15404785	0.51186	0.375152432	-0.448272	0.000117	hypomethylated
cg14313576	0.73483	0.538569189	-0.448279	4.15E-05	hypomethylated
cg18205883	0.94895	0.695473514	-0.448337	3.49E-06	hypomethylated
cg25531341	0.81412	0.596645946	-0.448366	1.21E-05	hypomethylated
cg25799589	0.656	0.480763243	-0.448369	0.001541	hypomethylated
cg06162324	0.80095	0.586971351	-0.448422	4.12E-06	hypomethylated
cg17397004	0.58179	0.42636	-0.448426	0.00012	hypomethylated
cg21345826	0.84458	0.618912973	-0.448498	4.36E-06	hypomethylated
cg15497006	0.61391	0.449874054	-0.448506	0.005606	hypomethylated
cg03049445	0.81113	0.594381622	-0.448544	4.48E-06	hypomethylated
cg07890553	0.58773	0.430654595	-0.448622	0.000365	hypomethylated
cg19257402	0.44102	0.32313027	-0.448728	0.000135	hypomethylated
cg26108621	0.73529	0.538724324	-0.448766	0.00017	hypomethylated
cg24180219	0.52563	0.385109189	-0.44878	3.67E-05	hypomethylated
cg02100150	0.74578	0.546392973	-0.448811	6.31E-05	hypomethylated
cg17629796	0.93654	0.68613027	-0.448858	3.15E-05	hypomethylated
cg26968025	0.8204	0.601040541	-0.448865	4.24E-06	hypomethylated
cg26586843	0.60582	0.443804324	-0.448965	0.000265	hypomethylated
cg06242449	0.4685	0.343205946	-0.448975	0.000191	hypomethylated
cg18866792	0.76632	0.561373514	-0.448986	1.99E-05	hypomethylated
cg00328219	0.67121	0.491687568	-0.449022	9.57E-06	hypomethylated
cg21487856	0.65089	0.476787568	-0.449067	0.000805	hypomethylated
cg08459746	0.82219	0.60222973	-0.449158	2.56E-06	hypomethylated
cg02958014	0.75764	0.554947027	-0.449162	1.37E-05	hypomethylated
cg11580676	0.88452	0.647864865	-0.449202	3.17E-06	hypomethylated
cg16356737	0.78643	0.576017297	-0.449206	5.86E-05	hypomethylated
cg21498475	0.56744	0.415618378	-0.449208	0.000116	hypomethylated

cg00857862	0.80597	0.590322162	-0.449224	0.000107	hypomethylated
cg02723617	0.53307	0.390426486	-0.449274	0.000209	hypomethylated
cg22789714	0.37485	0.274525405	-0.449374	0.000674	hypomethylated
cg19009305	0.60653	0.444195135	-0.449385	2.39E-05	hypomethylated
cg07348300	0.48675	0.356452973	-0.449469	7.12E-06	hypomethylated
cg10028434	0.66217	0.484905946	-0.449497	1.01E-05	hypomethylated
cg07143158	0.87418	0.640152973	-0.449514	1.46E-05	hypomethylated
cg04561791	0.57688	0.422436216	-0.449538	7.29E-05	hypomethylated
cg18665334	0.60098	0.440076757	-0.449562	0.000633	hypomethylated
cg10172675	0.55384	0.405520541	-0.449694	0.000358	hypomethylated
cg18317531	0.89249	0.653457297	-0.449743	3.08E-06	hypomethylated
cg26319827	0.83872	0.614077297	-0.449769	7.93E-06	hypomethylated
cg25526061	0.694	0.508090811	-0.449849	0.0017	hypomethylated
cg24544082	0.8124	0.594760541	-0.449881	0.000306	hypomethylated
cg10294820	0.65174	0.477094595	-0.450021	5.72E-05	hypomethylated
cg05609780	0.90986	0.666042162	-0.450031	2.10E-06	hypomethylated
cg12985065	0.57425	0.420349189	-0.450091	4.36E-06	hypomethylated
cg25783892	0.87318	0.639158378	-0.450106	5.28E-06	hypomethylated
cg01782097	0.42993	0.314684324	-0.450196	0.000245	hypomethylated
cg11476241	0.73376	0.537054595	-0.45024	0.000839	hypomethylated
cg05977376	0.56896	0.41642973	-0.450254	0.000268	hypomethylated
cg12562828	0.5657	0.413971892	-0.450504	0.000259	hypomethylated
cg21621782	0.68128	0.498547568	-0.450517	5.86E-05	hypomethylated
cg14871010	0.84339	0.617172432	-0.450526	4.01E-06	hypomethylated
cg02584867	0.70444	0.515489189	-0.450535	6.93E-06	hypomethylated
cg09359810	0.77522	0.567282703	-0.450538	0.000229	hypomethylated
cg07824422	0.63764	0.466597297	-0.450564	0.00035	hypomethylated
cg02042997	0.59493	0.435330811	-0.450608	0.003637	hypomethylated
cg01483139	0.80358	0.588003243	-0.450618	0.000756	hypomethylated
cg12170787	0.5983	0.437785405	-0.450645	0.000667	hypomethylated
cg04396850	0.79299	0.580233514	-0.450669	8.03E-05	hypomethylated
cg00966557	0.75125	0.54968	-0.450701	5.43E-06	hypomethylated
cg03651219	0.91518	0.669618378	-0.450716	1.66E-05	hypomethylated
cg26050975	0.66898	0.48947027	-0.450742	0.004287	hypomethylated
cg07397616	0.62646	0.458315676	-0.450881	7.93E-05	hypomethylated
cg07097725	0.77562	0.567429189	-0.45091	9.72E-05	hypomethylated
cg27639613	0.83819	0.613142703	-0.451054	0.00012	hypomethylated
cg13247117	0.69416	0.507771351	-0.451089	2.81E-05	hypomethylated
cg00967711	0.78505	0.574255676	-0.451091	8.03E-05	hypomethylated
cg02012483	0.7962	0.582349189	-0.451246	8.15E-06	hypomethylated
cg16536610	0.91172	0.666801622	-0.451333	1.49E-06	hypomethylated
cg05334655	0.63435	0.46391027	-0.451433	4.94E-05	hypomethylated
cg17259265	0.86864	0.635236757	-0.451464	4.42E-05	hypomethylated
cg21106695	0.88797	0.64936973	-0.451471	8.26E-06	hypomethylated
cg16672203	0.72007	0.526563243	-0.45153	4.26E-05	hypomethylated
cg12446045	0.77045	0.563359459	-0.451646	1.89E-05	hypomethylated
cg06602478	0.87652	0.640916757	-0.45165	6.57E-06	hypomethylated
cg26775695	0.81759	0.597798919	-0.451717	8.60E-06	hypomethylated
cg02280644	0.53837	0.393638378	-0.451727	3.85E-05	hypomethylated
cg03274800	0.87684	0.6411	-0.451764	9.31E-06	hypomethylated
cg26164488	0.7961	0.582065946	-0.451767	0.000426	hypomethylated
cg01020774	0.8169	0.597243784	-0.45184	1.04E-05	hypomethylated
cg27300194	0.36479	0.266700541	-0.451845	0.00032	hypomethylated
cg21103992	0.83989	0.614048649	-0.451847	0.000187	hypomethylated
cg18580385	0.87393	0.638890811	-0.451948	6.93E-06	hypomethylated
cg06935608	0.6298	0.460413514	-0.451964	0.000129	hypomethylated
cg15859390	0.72203	0.527827027	-0.451994	9.95E-05	hypomethylated
cg10661002	0.89461	0.653977297	-0.452018	4.73E-06	hypomethylated

cg13505393	0.5213	0.381076757	-0.452032	0.004863	hypomethylated
cg13791379	0.82566	0.603563243	-0.452043	7.12E-06	hypomethylated
cg03926640	0.66608	0.486894595	-0.452086	1.18E-05	hypomethylated
cg23017002	0.80355	0.587379459	-0.452095	3.07E-05	hypomethylated
cg09880665	0.63194	0.461891892	-0.452232	0.000107	hypomethylated
cg00563495	0.57752	0.422091351	-0.452316	8.73E-05	hypomethylated
cg14476212	0.7973	0.582717838	-0.452325	4.15E-05	hypomethylated
cg24278165	0.94122	0.687900541	-0.452332	8.15E-06	hypomethylated
cg10090591	0.85563	0.625334054	-0.45236	4.26E-05	hypomethylated
cg05199904	0.73692	0.538571892	-0.452369	0.000262	hypomethylated
cg04261496	0.69611	0.508739459	-0.452388	0.005316	hypomethylated
cg25986240	0.55595	0.406302162	-0.452402	6.54E-05	hypomethylated
cg04089788	0.39688	0.290035135	-0.452475	0.000107	hypomethylated
cg16600733	0.94983	0.694121081	-0.452482	1.40E-05	hypomethylated
cg22059438	0.74247	0.542543784	-0.452593	8.22E-05	hypomethylated
cg10560420	0.82322	0.601540541	-0.452616	4.86E-06	hypomethylated
cg07556261	0.63491	0.463938378	-0.452619	4.47E-05	hypomethylated
cg17079987	0.79454	0.580577297	-0.452632	0.000506	hypomethylated
cg16851482	0.64141	0.468681081	-0.45264	0.0003	hypomethylated
cg25619717	0.85984	0.628281622	-0.452657	2.29E-06	hypomethylated
cg24441324	0.8204	0.59944973	-0.452689	0.000214	hypomethylated
cg00264419	0.59872	0.437443243	-0.452786	0.00044	hypomethylated
cg03481488	0.82677	0.604045946	-0.452828	1.82E-06	hypomethylated
cg20424311	0.65166	0.476105946	-0.452837	8.84E-05	hypomethylated
cg11384014	0.64775	0.473248108	-0.45284	6.66E-06	hypomethylated
cg19036773	0.59233	0.432741622	-0.452895	5.19E-05	hypomethylated
cg07134252	0.76279	0.557236216	-0.452997	2.10E-05	hypomethylated
cg03602014	0.63103	0.460964324	-0.453053	0.000931	hypomethylated
cg22079077	0.56993	0.416269189	-0.453268	0.000274	hypomethylated
cg26496628	0.64849	0.473631351	-0.45332	0.000253	hypomethylated
cg07409471	0.85006	0.620843243	-0.453336	8.63E-05	hypomethylated
cg11206634	0.68917	0.503277297	-0.453506	0.000234	hypomethylated
cg01588581	0.6632	0.484304865	-0.453529	0.000148	hypomethylated
cg26107033	0.83237	0.60780973	-0.453605	4.58E-05	hypomethylated
cg00179940	0.69795	0.509638378	-0.45365	8.73E-05	hypomethylated
cg06705122	0.54991	0.401536757	-0.453663	0.000107	hypomethylated
cg15263682	0.78198	0.570980541	-0.45369	1.58E-06	hypomethylated
cg18410110	0.76489	0.558472973	-0.453765	5.00E-05	hypomethylated
cg15620905	0.69698	0.508872973	-0.453812	0.000117	hypomethylated
cg14997942	0.91294	0.666517838	-0.453877	3.21E-06	hypomethylated
cg12179658	0.60201	0.439487568	-0.453965	8.03E-05	hypomethylated
cg07892167	0.69655	0.508495676	-0.453991	0.002856	hypomethylated
cg00808492	0.91513	0.668059459	-0.454	2.16E-06	hypomethylated
cg08939394	0.67579	0.493325405	-0.454035	0.001667	hypomethylated
cg06438797	0.63856	0.466135676	-0.454072	1.75E-05	hypomethylated
cg00418071	0.65376	0.477230811	-0.454074	0.000287	hypomethylated
cg24599650	0.57633	0.420699459	-0.454105	0.000117	hypomethylated
cg05504759	0.48187	0.351747027	-0.454106	0.006015	hypomethylated
cg02516845	0.70565	0.515078919	-0.454159	5.45E-05	hypomethylated
cg14352298	0.49765	0.363247027	-0.45418	6.31E-05	hypomethylated
cg01738406	0.58394	0.426192973	-0.454313	0.000163	hypomethylated
cg26689934	0.73684	0.537774054	-0.454351	7.47E-05	hypomethylated
cg20518497	0.8358	0.609937838	-0.454496	1.49E-06	hypomethylated
cg19534021	0.66383	0.484427027	-0.454534	0.000119	hypomethylated
cg14664316	0.73169	0.533942703	-0.454548	6.01E-05	hypomethylated
cg14752525	0.64557	0.471094595	-0.454557	0.000214	hypomethylated
cg16922937	0.76982	0.561763784	-0.454558	0.000756	hypomethylated
cg13603551	0.70896	0.517347568	-0.45457	3.07E-05	hypomethylated

cg26325867	0.52432	0.382596216	-0.454625	2.27E-05	hypomethylated
cg12382557	0.92078	0.671891892	-0.454627	8.60E-06	hypomethylated
cg00741986	0.62572	0.456576216	-0.454662	0.000696	hypomethylated
cg27500292	0.6929	0.505595676	-0.454663	3.67E-05	hypomethylated
cg21244322	0.6221	0.453913514	-0.454729	0.003506	hypomethylated
cg08641990	0.76199	0.555943243	-0.454834	0.0003	hypomethylated
cg02862467	0.80493	0.58725027	-0.454888	0.00024	hypomethylated
cg14885762	0.55697	0.406344865	-0.454895	0.00012	hypomethylated
cg11746651	0.76062	0.554884324	-0.454989	5.86E-05	hypomethylated
cg23057567	0.57437	0.419011892	-0.454989	0.000189	hypomethylated
cg22136020	0.51981	0.379208108	-0.454995	0.002409	hypomethylated
cg19440734	0.7032	0.512970811	-0.455058	0.001262	hypomethylated
cg18188916	0.80561	0.587672973	-0.455068	0.000358	hypomethylated
cg06571687	0.52723	0.384583784	-0.455135	0.000374	hypomethylated
cg19064302	0.78847	0.575100541	-0.455242	9.72E-05	hypomethylated
cg17672850	0.39093	0.285136757	-0.455256	5.58E-05	hypomethylated
cg16753939	0.73833	0.538494595	-0.455334	4.94E-05	hypomethylated
cg09991092	0.66835	0.487435135	-0.455394	2.85E-05	hypomethylated
cg15885043	0.73072	0.532908649	-0.45543	3.59E-06	hypomethylated
cg16611234	0.39856	0.290631351	-0.455607	0.000145	hypomethylated
cg15944459	0.80276	0.585374595	-0.455609	1.15E-05	hypomethylated
cg10409560	0.88458	0.64502	-0.455649	9.82E-06	hypomethylated
cg24141382	0.44821	0.326825405	-0.455655	0.00028	hypomethylated
cg02145220	0.72265	0.526917838	-0.455719	0.000102	hypomethylated
cg11608390	0.78797	0.574511892	-0.455804	0.000117	hypomethylated
cg18698431	0.73098	0.532948649	-0.455835	0.000174	hypomethylated
cg09688127	0.78365	0.571328108	-0.45589	0.000335	hypomethylated
cg11165391	0.687	0.500864324	-0.45589	0.000145	hypomethylated
cg12322672	0.66146	0.482242703	-0.455895	8.22E-05	hypomethylated
cg02774282	0.68775	0.50135027	-0.456065	0.000148	hypomethylated
cg04494791	0.5448	0.397134054	-0.456101	8.42E-05	hypomethylated
cg07989851	0.64512	0.470231351	-0.456197	0.000517	hypomethylated
cg10091265	0.53603	0.390711892	-0.456209	6.01E-05	hypomethylated
cg24477401	0.61636	0.449254054	-0.456242	0.000912	hypomethylated
cg02147126	0.68765	0.501185946	-0.456328	0.000182	hypomethylated
cg14240304	0.70685	0.515178919	-0.456331	4.80E-06	hypomethylated
cg08012275	0.95521	0.696156757	-0.456406	2.29E-06	hypomethylated
cg14196507	0.86557	0.630825405	-0.45641	1.28E-05	hypomethylated
cg02980249	0.675	0.491874054	-0.456599	5.19E-05	hypomethylated
cg04169483	0.73726	0.537202162	-0.456708	0.00028	hypomethylated
cg17085710	0.6306	0.459474595	-0.45674	4.26E-05	hypomethylated
cg14598846	0.64071	0.46684	-0.456743	5.58E-05	hypomethylated
cg14046640	0.77635	0.565639459	-0.456824	8.60E-06	hypomethylated
cg27179693	0.58746	0.428013514	-0.456834	0.000495	hypomethylated
cg26169991	0.67817	0.494098378	-0.456849	0.000214	hypomethylated
cg27006169	0.86813	0.632480541	-0.45689	8.63E-05	hypomethylated
cg24618387	0.84488	0.615504324	-0.456977	9.57E-06	hypomethylated
cg12771187	0.71482	0.520751351	-0.456985	0.000681	hypomethylated
cg07069934	0.65213	0.475011892	-0.457196	0.00028	hypomethylated
cg11783497	0.59587	0.434014595	-0.457254	0.0003	hypomethylated
cg18950952	0.54115	0.394147568	-0.457293	0.000382	hypomethylated
cg08802944	0.72251	0.526236216	-0.457307	0.000293	hypomethylated
cg06706068	0.61372	0.446985946	-0.457351	0.001987	hypomethylated
cg17421241	0.7423	0.540616757	-0.457396	6.93E-06	hypomethylated
cg13520525	0.75661	0.551022162	-0.457439	2.57E-05	hypomethylated
cg03607644	0.91113	0.663536216	-0.457482	1.49E-06	hypomethylated
cg20821276	0.54219	0.394818919	-0.457607	0.000165	hypomethylated
cg14031473	0.53867	0.39224973	-0.457629	0.000232	hypomethylated

cg05457221	0.64325	0.468390811	-0.457667	0.000551	hypomethylated
cg11783815	0.77158	0.561804324	-0.457748	3.95E-06	hypomethylated
cg11469587	0.5988	0.435990811	-0.457776	8.42E-05	hypomethylated
cg01565584	0.88527	0.644543243	-0.45784	5.28E-06	hypomethylated
cg01442005	0.84241	0.613327027	-0.457866	4.47E-05	hypomethylated
cg16017979	0.87284	0.635456216	-0.457924	2.51E-05	hypomethylated
cg26955845	0.66224	0.482116216	-0.457973	0.001987	hypomethylated
cg15754878	0.86035	0.626324865	-0.458013	5.06E-05	hypomethylated
cg15726557	0.72258	0.526029189	-0.458014	1.99E-06	hypomethylated
cg19544434	0.54781	0.398785946	-0.458061	0.000338	hypomethylated
cg02571534	0.54144	0.394125946	-0.458145	2.45E-05	hypomethylated
cg21198586	0.51912	0.377838378	-0.458299	0.000464	hypomethylated
cg05541460	0.83975	0.611193514	-0.458331	1.40E-05	hypomethylated
cg26484108	0.68948	0.501813514	-0.458357	0.000256	hypomethylated
cg14341131	0.64856	0.472027568	-0.458369	0.001314	hypomethylated
cg20610950	0.87319	0.635487568	-0.458432	5.06E-05	hypomethylated
cg05476182	0.52615	0.382867027	-0.458631	5.06E-05	hypomethylated
cg10821261	0.54137	0.393936757	-0.458651	0.006122	hypomethylated
cg15668538	0.74601	0.542823243	-0.458712	4.05E-05	hypomethylated
cg01259329	0.90546	0.658836216	-0.458731	4.01E-06	hypomethylated
cg07819010	0.7982	0.580777297	-0.458765	1.39E-05	hypomethylated
cg09361094	0.67737	0.492859459	-0.458768	0.000207	hypomethylated
cg12583970	0.89064	0.648016216	-0.458812	2.83E-06	hypomethylated
cg14707834	0.59446	0.432492973	-0.458903	0.007408	hypomethylated
cg26853371	0.86238	0.627405405	-0.458926	1.39E-05	hypomethylated
cg02170478	0.42826	0.311540541	-0.459067	0.000221	hypomethylated
cg01638311	0.78284	0.569477838	-0.459078	0.000365	hypomethylated
cg23428143	0.74986	0.545451351	-0.459171	2.92E-05	hypomethylated
cg20373326	0.56652	0.412086486	-0.45918	3.32E-05	hypomethylated
cg18704527	0.71476	0.519882162	-0.459274	0.003316	hypomethylated
cg00583535	0.4531	0.329559459	-0.459291	0.00032	hypomethylated
cg22923514	0.47905	0.348377838	-0.459523	4.82E-05	hypomethylated
cg26971423	0.85475	0.621569189	-0.459588	3.40E-05	hypomethylated
cg19164997	0.83906	0.610152973	-0.459603	1.09E-06	hypomethylated
cg13393785	0.57006	0.414515135	-0.459689	9.82E-06	hypomethylated
cg03907390	0.59666	0.433841622	-0.459741	0.001651	hypomethylated
cg13625026	0.49219	0.357860541	-0.459818	0.0006	hypomethylated
cg03334213	0.68409	0.497384324	-0.459825	3.53E-05	hypomethylated
cg13892257	0.79658	0.57914	-0.459907	4.94E-05	hypomethylated
cg20346503	0.78905	0.573655135	-0.459933	3.57E-05	hypomethylated
cg11785183	0.73223	0.53233027	-0.459975	4.12E-06	hypomethylated
cg12400025	0.88893	0.646241081	-0.459997	9.07E-06	hypomethylated
cg23465685	0.82513	0.599853514	-0.460011	9.27E-05	hypomethylated
cg01461514	0.64239	0.467004324	-0.460014	5.06E-05	hypomethylated
cg27178677	0.7238	0.526140541	-0.460143	6.46E-05	hypomethylated
cg17861277	0.72391	0.526217297	-0.460152	0.000667	hypomethylated
cg10275770	0.52428	0.381098919	-0.460172	0.001734	hypomethylated
cg13884879	0.84428	0.613650811	-0.460304	6.75E-06	hypomethylated
cg12103951	0.534	0.388089189	-0.460451	0.00078	hypomethylated
cg09010372	0.83206	0.604661081	-0.460561	6.30E-07	hypomethylated
cg22341848	0.59625	0.433288108	-0.460591	2.10E-06	hypomethylated
cg02770724	0.6446	0.468379459	-0.460726	0.001314	hypomethylated
cg24797508	0.58413	0.424414595	-0.460815	0.001875	hypomethylated
cg00892792	0.84217	0.611896216	-0.460825	0.00024	hypomethylated
cg13042250	0.87924	0.638822162	-0.460843	7.72E-06	hypomethylated
cg15379858	0.78213	0.568265405	-0.460844	2.39E-05	hypomethylated
cg00412772	0.62336	0.452905946	-0.460854	2.61E-05	hypomethylated
cg23327896	0.85774	0.623193514	-0.46086	0.000111	hypomethylated

cg16594468	0.60274	0.437888649	-0.460972	8.63E-05	hypomethylated
cg19556284	0.84202	0.611697838	-0.461035	3.49E-05	hypomethylated
cg19609923	0.64293	0.467051351	-0.46108	1.21E-05	hypomethylated
cg03512414	0.63215	0.459207568	-0.461121	0.00054	hypomethylated
cg09518969	0.80393	0.583973514	-0.461167	2.78E-05	hypomethylated
cg25750901	0.55581	0.403704865	-0.461291	0.000138	hypomethylated
cg01878963	0.81136	0.589316757	-0.461299	5.72E-05	hypomethylated
cg18964590	0.69515	0.504902162	-0.46132	5.58E-05	hypomethylated
cg04321126	0.58168	0.422481622	-0.461337	6.15E-05	hypomethylated
cg00039463	0.73738	0.535556216	-0.46137	1.84E-05	hypomethylated
cg08158518	0.80372	0.583701081	-0.461463	3.40E-05	hypomethylated
cg18310639	0.63134	0.45848973	-0.461528	0.001603	hypomethylated
cg09171931	0.84402	0.612905946	-0.461611	1.58E-05	hypomethylated
cg02057755	0.53851	0.391047027	-0.461631	0.000129	hypomethylated
cg07943832	0.84747	0.61536	-0.461732	3.32E-05	hypomethylated
cg14408823	0.73849	0.536210811	-0.461778	3.39E-06	hypomethylated
cg07545081	0.76011	0.551886486	-0.461837	3.95E-05	hypomethylated
cg10094191	0.67794	0.492215676	-0.461867	1.82E-05	hypomethylated
cg03246914	0.68681	0.498648108	-0.461889	0.000313	hypomethylated
cg18763536	0.6005	0.435981622	-0.461897	0.000789	hypomethylated
cg02732164	0.7004	0.508507568	-0.46191	3.85E-06	hypomethylated
cg08894532	0.57722	0.419069189	-0.461933	0.010042	hypomethylated
cg05396156	0.89031	0.646371892	-0.461943	3.90E-06	hypomethylated
cg08418872	0.80087	0.581437297	-0.461944	0.000696	hypomethylated
cg25637966	0.64857	0.47081027	-0.462117	5.45E-05	hypomethylated
cg03956353	0.79242	0.575226486	-0.462135	3.69E-06	hypomethylated
cg03950253	0.72019	0.522787568	-0.462153	0.00028	hypomethylated
cg01335980	0.81528	0.591804324	-0.462175	5.19E-05	hypomethylated
cg02737399	0.83074	0.603023243	-0.462183	2.05E-06	hypomethylated
cg04327247	0.67771	0.491940541	-0.462184	0.00018	hypomethylated
cg26184501	0.74656	0.541900541	-0.46223	5.89E-06	hypomethylated
cg17087907	0.76136	0.552618919	-0.462294	7.72E-06	hypomethylated
cg00049616	0.88426	0.64182	-0.462302	6.39E-06	hypomethylated
cg21253043	0.71477	0.518798378	-0.462305	1.71E-05	hypomethylated
cg15302379	0.72803	0.52840973	-0.462341	5.58E-05	hypomethylated
cg21226965	0.82338	0.597615135	-0.462342	6.78E-05	hypomethylated
cg13328209	0.86151	0.625243243	-0.46245	1.94E-05	hypomethylated
cg15133098	0.7498	0.544167027	-0.462456	0.000485	hypomethylated
cg12251803	0.63072	0.457730811	-0.4625	0.000193	hypomethylated
cg25068347	0.67649	0.490918919	-0.462584	0.000822	hypomethylated
cg04833731	0.84853	0.615742162	-0.462639	2.78E-05	hypomethylated
cg03704061	0.67261	0.488064324	-0.462699	1.35E-05	hypomethylated
cg25392995	0.75493	0.547783784	-0.462736	8.42E-05	hypomethylated
cg06895640	0.78317	0.568252432	-0.462794	0.000251	hypomethylated
cg08133629	0.60233	0.437013514	-0.462876	0.00024	hypomethylated
cg19654061	0.78787	0.571617297	-0.462908	3.85E-05	hypomethylated
cg09084279	0.69744	0.505964865	-0.463032	2.32E-05	hypomethylated
cg03116016	0.60326	0.437628649	-0.463073	0.000459	hypomethylated
cg13742752	0.8498	0.616478378	-0.463073	8.60E-06	hypomethylated
cg21988950	0.87099	0.631824324	-0.463133	4.01E-06	hypomethylated
cg21772953	0.81575	0.59172	-0.463212	2.36E-06	hypomethylated
cg12925881	0.82939	0.601607027	-0.463229	2.21E-05	hypomethylated
cg10399269	0.83507	0.605668108	-0.46337	4.60E-06	hypomethylated
cg25627341	0.68896	0.499683784	-0.463405	0.000214	hypomethylated
cg27600804	0.56909	0.412743784	-0.46341	0.000112	hypomethylated
cg16482761	0.81853	0.593651351	-0.463419	1.01E-05	hypomethylated
cg24951886	0.81474	0.590901081	-0.463423	8.63E-05	hypomethylated
cg06639923	0.80544	0.584155135	-0.463426	1.80E-05	hypomethylated

cg02939781	0.85237	0.618173514	-0.463468	3.95E-05	hypomethylated
cg16018921	0.84771	0.614793514	-0.463469	3.67E-05	hypomethylated
cg08047629	0.80194	0.581557838	-0.463572	2.79E-06	hypomethylated
cg20226122	0.88699	0.643226486	-0.463591	7.52E-06	hypomethylated
cg04987071	0.81139	0.588376216	-0.463657	7.84E-05	hypomethylated
cg07669403	0.6136	0.444944324	-0.463674	0.002026	hypomethylated
cg08122151	0.60042	0.435376757	-0.463708	0.000259	hypomethylated
cg17015522	0.69926	0.507031892	-0.463752	0.000187	hypomethylated
cg05173058	0.74283	0.538621081	-0.463761	9.72E-05	hypomethylated
cg14793844	0.77854	0.564486486	-0.463832	2.71E-05	hypomethylated
cg12919119	0.43427	0.31487027	-0.463835	0.000163	hypomethylated
cg25499746	0.85853	0.622472973	-0.463857	6.75E-06	hypomethylated
cg02012159	0.68541	0.496916216	-0.463965	3.85E-05	hypomethylated
cg14583406	0.69389	0.503031892	-0.464057	3.32E-05	hypomethylated
cg20792436	0.92539	0.670851351	-0.464068	7.12E-06	hypomethylated
cg06729381	0.62643	0.454090811	-0.464172	0.00078	hypomethylated
cg10598776	0.7724	0.559898919	-0.464182	9.96E-06	hypomethylated
cg15430294	0.55209	0.400198919	-0.464186	0.000138	hypomethylated
cg06784218	0.50008	0.362463243	-0.464324	5.00E-06	hypomethylated
cg01638792	0.91284	0.661603243	-0.464396	2.49E-06	hypomethylated
cg16405308	0.71896	0.521078378	-0.464411	6.75E-06	hypomethylated
cg19909117	0.67786	0.491281622	-0.464437	0.000123	hypomethylated
cg15679747	0.91156	0.660618378	-0.464521	4.36E-06	hypomethylated
cg08314781	0.73017	0.529115135	-0.464651	6.62E-05	hypomethylated
cg25255988	0.75127	0.544399459	-0.464666	7.32E-06	hypomethylated
cg02443967	0.92147	0.667704324	-0.464728	5.81E-06	hypomethylated
cg05984508	0.51034	0.369788108	-0.46476	0.003773	hypomethylated
cg01542423	0.81715	0.592095135	-0.464772	4.48E-06	hypomethylated
cg10648763	0.52225	0.378396757	-0.464841	0.000378	hypomethylated
cg20689294	0.53145	0.385045405	-0.464905	0.001734	hypomethylated
cg14089474	0.84204	0.610065946	-0.464924	8.15E-06	hypomethylated
cg09104660	0.45709	0.331163784	-0.464933	0.007282	hypomethylated
cg04466743	0.64034	0.463928649	-0.464935	8.42E-05	hypomethylated
cg11778714	0.82547	0.598036757	-0.464982	1.37E-06	hypomethylated
cg24782497	0.6021	0.436206486	-0.464992	0.00012	hypomethylated
cg08719608	0.5079	0.367955135	-0.465015	0.003441	hypomethylated
cg19169342	0.5194	0.376284324	-0.465023	7.65E-05	hypomethylated
cg21331510	0.75071	0.543814595	-0.465141	6.22E-06	hypomethylated
cg10712476	0.83092	0.601875135	-0.465245	3.49E-05	hypomethylated
cg00264133	0.77683	0.562672973	-0.465302	5.45E-05	hypomethylated
cg25554496	0.8352	0.604921622	-0.465373	5.45E-05	hypomethylated
cg17571266	0.51262	0.37126973	-0.465422	0.000545	hypomethylated
cg21531126	0.82554	0.597851892	-0.46555	1.66E-05	hypomethylated
cg06768939	0.7802	0.564991351	-0.465615	7.22E-06	hypomethylated
cg22233974	0.68844	0.498541622	-0.465617	0.000857	hypomethylated
cg04150954	0.71763	0.519662703	-0.465665	6.62E-05	hypomethylated
cg24804768	0.56893	0.411983243	-0.465665	0.000857	hypomethylated
cg08455089	0.85264	0.617415135	-0.465696	2.36E-06	hypomethylated
cg02560744	0.80456	0.582596757	-0.465702	4.58E-05	hypomethylated
cg00414041	0.92862	0.672423243	-0.465719	2.13E-06	hypomethylated
cg01312394	0.7293	0.528092432	-0.465722	5.58E-05	hypomethylated
cg06383241	0.6255	0.452923243	-0.465743	0.005316	hypomethylated
cg14329285	0.83794	0.606744324	-0.465758	3.81E-05	hypomethylated
cg16235748	0.52503	0.380152973	-0.46582	0.000667	hypomethylated
cg08053935	0.51903	0.375804324	-0.465836	0.000122	hypomethylated
cg03224627	0.81613	0.590909189	-0.465863	2.21E-05	hypomethylated
cg13131015	0.78389	0.567556757	-0.465887	1.11E-05	hypomethylated
cg14281591	0.77663	0.562263243	-0.465982	0.000271	hypomethylated

cg27228272	0.77335	0.559881622	-0.466	0.000224	hypomethylated
cg07867517	0.80314	0.581440541	-0.46602	0.000191	hypomethylated
cg16289449	0.43866	0.317550811	-0.466116	0.003378	hypomethylated
cg23527468	0.77869	0.563677838	-0.466178	2.45E-05	hypomethylated
cg20415517	0.90482	0.654962703	-0.466218	9.49E-05	hypomethylated
cg19614643	0.76757	0.5556	-0.466252	0.000386	hypomethylated
cg10254082	0.70571	0.510810811	-0.466286	1.33E-05	hypomethylated
cg14557690	0.77714	0.562492973	-0.466339	1.18E-05	hypomethylated
cg00514486	0.55022	0.398230811	-0.466404	6.46E-05	hypomethylated
cg21201572	0.80603	0.583374054	-0.466412	0.000382	hypomethylated
cg24021846	0.61349	0.444005946	-0.466461	0.0003	hypomethylated
cg01342115	0.74169	0.536765405	-0.466525	0.000931	hypomethylated
cg20769856	0.7111	0.514607568	-0.46658	0.000386	hypomethylated
cg11421768	0.92513	0.669487568	-0.466599	7.84E-05	hypomethylated
cg20567368	0.61978	0.448475135	-0.466728	0.000317	hypomethylated
cg04799329	0.79759	0.577135676	-0.466737	1.09E-05	hypomethylated
cg03333699	0.73227	0.529862703	-0.466757	1.42E-05	hypomethylated
cg00546897	0.62687	0.453584865	-0.466794	5.72E-05	hypomethylated
cg16073408	0.72613	0.525385946	-0.46685	0.000123	hypomethylated
cg04153489	0.55641	0.402580541	-0.466871	6.01E-05	hypomethylated
cg24125648	0.30091	0.217709189	-0.46693	0.000866	hypomethylated
cg08827700	0.80075	0.579342162	-0.466936	6.39E-06	hypomethylated
cg04689606	0.83831	0.606507568	-0.466958	0.000214	hypomethylated
cg23697406	0.65624	0.474767568	-0.467002	0.00012	hypomethylated
cg09871057	0.61457	0.444616757	-0.467015	0.000681	hypomethylated
cg15484375	0.52006	0.376238919	-0.467029	0.00054	hypomethylated
cg16736889	0.78292	0.566381081	-0.467092	0.000839	hypomethylated
cg01207473	0.74444	0.538523243	-0.467147	0.000119	hypomethylated
cg02241145	0.61795	0.446996757	-0.467226	2.32E-05	hypomethylated
cg00154455	0.788	0.569986486	-0.467268	2.10E-05	hypomethylated
cg11644973	0.84992	0.614723243	-0.46739	9.31E-06	hypomethylated
cg14473904	0.82334	0.595491351	-0.467408	1.28E-05	hypomethylated
cg15636519	0.69544	0.502978378	-0.46743	0.001176	hypomethylated
cg01081096	0.66773	0.482912432	-0.467503	1.56E-05	hypomethylated
cg10639435	0.66679	0.48222	-0.467541	0.000174	hypomethylated
cg04895166	0.70563	0.510275676	-0.467635	4.18E-06	hypomethylated
cg13887966	0.9224	0.666947568	-0.467819	3.21E-06	hypomethylated
cg01982835	0.72244	0.522351351	-0.467857	1.01E-05	hypomethylated
cg05975710	0.49152	0.355383784	-0.467872	4.26E-05	hypomethylated
cg13823169	0.59284	0.428639459	-0.467878	0.000191	hypomethylated
cg00110832	0.68221	0.493252432	-0.46789	0.000107	hypomethylated
cg11061343	0.62595	0.45257027	-0.467906	0.001987	hypomethylated
cg02745494	0.7463	0.539540541	-0.468024	2.04E-05	hypomethylated
cg26458557	0.86536	0.625598378	-0.468064	3.25E-07	hypomethylated
cg26683025	0.94978	0.686627027	-0.468067	5.50E-06	hypomethylated
cg02968918	0.63623	0.45994973	-0.468072	6.15E-05	hypomethylated
cg10949576	0.46782	0.3382	-0.468077	5.19E-05	hypomethylated
cg15540723	0.45251	0.327123243	-0.468115	4.58E-05	hypomethylated
cg18912103	0.75201	0.543619459	-0.468155	0.00014	hypomethylated
cg02332117	0.4255	0.307586486	-0.468167	9.83E-05	hypomethylated
cg11583041	0.85485	0.617860541	-0.46839	9.11E-07	hypomethylated
cg05070273	0.85586	0.618586486	-0.4684	7.03E-06	hypomethylated
cg01511480	0.73321	0.529925946	-0.468436	3.95E-05	hypomethylated
cg00292107	0.62527	0.451887568	-0.468515	0.00017	hypomethylated
cg02314348	0.74884	0.541178919	-0.468552	1.08E-05	hypomethylated
cg02671204	0.83238	0.601548649	-0.468561	8.15E-06	hypomethylated
cg21187068	0.72442	0.523492973	-0.468656	2.27E-05	hypomethylated
cg18015534	0.77468	0.559795676	-0.4687	1.31E-05	hypomethylated

cg14151158	0.90366	0.652968649	-0.468766	1.71E-05	hypomethylated
cg02079584	0.5244	0.378920541	-0.468772	4.94E-05	hypomethylated
cg01053766	0.85847	0.620275676	-0.468858	4.48E-06	hypomethylated
cg07468956	0.87307	0.63082	-0.468869	4.93E-06	hypomethylated
cg09956592	0.54467	0.393524865	-0.468928	8.52E-05	hypomethylated
cg19872681	0.69165	0.49968	-0.469038	4.70E-05	hypomethylated
cg22909609	0.63707	0.460232973	-0.469088	0.000219	hypomethylated
cg17588294	0.68172	0.492444324	-0.469219	1.68E-05	hypomethylated
cg21721825	0.74007	0.534587568	-0.469235	4.36E-05	hypomethylated
cg05986067	0.64318	0.464586486	-0.469275	6.95E-05	hypomethylated
cg19687075	0.63212	0.456581622	-0.469326	0.001041	hypomethylated
cg23508201	0.67652	0.488575676	-0.469551	0.000104	hypomethylated
cg27005118	0.65061	0.469848108	-0.469599	0.000426	hypomethylated
cg06574229	0.58467	0.422207027	-0.469672	7.56E-05	hypomethylated
cg05664039	0.97133	0.701400541	-0.469723	8.34E-07	hypomethylated
cg12707926	0.44178	0.31899027	-0.469816	1.26E-05	hypomethylated
cg07699978	0.83738	0.604610811	-0.469876	2.32E-05	hypomethylated
cg10091408	0.64434	0.465222162	-0.469902	3.95E-05	hypomethylated
cg05091736	0.78044	0.56348	-0.469923	1.06E-05	hypomethylated
cg06704631	0.7244	0.522977297	-0.470038	7.29E-05	hypomethylated
cg08802482	0.62146	0.448633514	-0.470124	6.08E-05	hypomethylated
cg25136988	0.84684	0.61133027	-0.470137	0.000138	hypomethylated
cg21088438	0.75639	0.546021081	-0.470174	0.000193	hypomethylated
cg00934322	0.51942	0.374956757	-0.470177	6.95E-05	hypomethylated
cg08792976	0.50268	0.362838378	-0.470313	3.67E-05	hypomethylated
cg00390511	0.82615	0.596306486	-0.47035	3.44E-05	hypomethylated
cg21617357	0.87971	0.634961622	-0.470359	1.02E-06	hypomethylated
cg07336987	0.66082	0.476947568	-0.470427	0.000207	hypomethylated
cg25810857	0.68452	0.494042703	-0.470457	4.58E-05	hypomethylated
cg18721397	0.78225	0.564555135	-0.470515	0.000221	hypomethylated
cg16680451	0.4581	0.330610811	-0.470529	2.39E-05	hypomethylated
cg08889480	0.80829	0.583307568	-0.470616	2.21E-05	hypomethylated
cg10930290	0.73893	0.533251351	-0.470622	3.40E-05	hypomethylated
cg12081267	0.88057	0.635463243	-0.470629	5.58E-06	hypomethylated
cg03167883	0.57687	0.416296757	-0.470634	5.79E-05	hypomethylated
cg06850099	0.72957	0.52649027	-0.47064	0.000506	hypomethylated
cg18222590	0.63368	0.457286486	-0.470656	8.60E-06	hypomethylated
cg07594247	0.74445	0.537212432	-0.470682	0.000426	hypomethylated
cg12127162	0.69811	0.50376	-0.470718	7.72E-06	hypomethylated
cg09364122	0.94681	0.683196757	-0.470774	3.90E-06	hypomethylated
cg09303236	0.65484	0.472506486	-0.470808	2.71E-05	hypomethylated
cg04130572	0.51011	0.368064324	-0.47085	1.99E-05	hypomethylated
cg15979932	0.81045	0.584731892	-0.470948	2.27E-05	hypomethylated
cg04766043	0.88734	0.640194054	-0.470978	5.43E-06	hypomethylated
cg13560619	0.86345	0.622956757	-0.470981	2.36E-06	hypomethylated
cg25078649	0.94501	0.681795135	-0.470991	8.48E-06	hypomethylated
cg04833713	0.89666	0.646826486	-0.471182	2.42E-06	hypomethylated
cg11147155	0.54937	0.39629027	-0.471221	0.004365	hypomethylated
cg19981409	0.62553	0.451220541	-0.471246	0.001511	hypomethylated
cg05338167	0.791	0.570579459	-0.47125	3.15E-05	hypomethylated
cg22992966	0.64746	0.467037838	-0.471252	1.99E-05	hypomethylated
cg07810884	0.63277	0.456438378	-0.471261	0.000268	hypomethylated
cg18995031	0.80153	0.578164865	-0.471276	9.57E-06	hypomethylated
cg15534755	0.61985	0.4471	-0.471322	0.000313	hypomethylated
cg00075967	0.77706	0.560488108	-0.471342	9.27E-05	hypomethylated
cg12009872	0.46303	0.333977297	-0.471356	0.00134	hypomethylated
cg25271892	0.4879	0.351896757	-0.471433	0.000251	hypomethylated
cg26912131	0.58674	0.42317027	-0.471483	2.57E-05	hypomethylated

cg15877233	0.75753	0.546332432	-0.471524	2.85E-05	hypomethylated
cg03532013	0.88805	0.640454054	-0.471546	2.32E-05	hypomethylated
cg05545454	0.80241	0.578664324	-0.471613	0.000187	hypomethylated
cg10396171	0.85651	0.617676757	-0.471618	0.000182	hypomethylated
cg18961788	0.73231	0.528072432	-0.471719	2.85E-05	hypomethylated
cg09272225	0.759	0.54731027	-0.471741	0.000588	hypomethylated
cg07097722	0.75467	0.544173514	-0.471779	0.000474	hypomethylated
cg27145347	0.83608	0.602864865	-0.471806	3.95E-05	hypomethylated
cg10583319	0.52527	0.378748108	-0.471821	0.000417	hypomethylated
cg14642259	0.95863	0.691205946	-0.471858	6.93E-06	hypomethylated
cg07227049	0.82901	0.597743784	-0.471862	3.40E-05	hypomethylated
cg00608661	0.77503	0.558814595	-0.471882	7.93E-05	hypomethylated
cg23268102	0.71514	0.51560973	-0.471946	4.58E-05	hypomethylated
cg18788725	0.74968	0.540508108	-0.471959	0.000157	hypomethylated
cg00622170	0.87489	0.630699459	-0.472149	7.32E-06	hypomethylated
cg24766327	0.57272	0.412862703	-0.472168	0.000342	hypomethylated
cg26073844	0.8244	0.594286486	-0.472186	3.85E-05	hypomethylated
cg13180566	0.81873	0.59019027	-0.472208	4.14E-07	hypomethylated
cg06061240	0.81772	0.589454595	-0.472226	1.12E-05	hypomethylated
cg01922891	0.48575	0.350131892	-0.472316	0.000155	hypomethylated
cg12611897	0.7338	0.528916757	-0.472346	0.000426	hypomethylated
cg24710179	0.7627	0.549697838	-0.472477	2.64E-05	hypomethylated
cg05235884	0.86451	0.623052973	-0.472528	1.72E-06	hypomethylated
cg02683307	0.62676	0.451678919	-0.472616	5.89E-06	hypomethylated
cg10734044	0.83302	0.600315676	-0.47263	1.64E-05	hypomethylated
cg01359236	0.55274	0.398327027	-0.472648	3.23E-05	hypomethylated
cg05075308	0.84118	0.606151892	-0.472735	8.22E-05	hypomethylated
cg16663033	0.52107	0.375470811	-0.472776	0.000245	hypomethylated
cg21307155	0.66077	0.476082703	-0.472936	1.01E-05	hypomethylated
cg08932727	0.43836	0.315835676	-0.472942	0.000408	hypomethylated
cg11693709	0.76907	0.554096216	-0.472978	3.00E-05	hypomethylated
cg24756966	0.73842	0.532000541	-0.473014	2.51E-05	hypomethylated
cg24542821	0.79147	0.570208108	-0.473046	1.39E-05	hypomethylated
cg10731020	0.79863	0.575362162	-0.473057	2.42E-06	hypomethylated
cg08611549	0.69059	0.497518919	-0.473078	7.65E-05	hypomethylated
cg07802625	0.45254	0.325996216	-0.47319	0.000106	hypomethylated
cg22321808	0.8135	0.586004865	-0.47323	9.57E-06	hypomethylated
cg20014974	0.60467	0.435569189	-0.473246	0.000454	hypomethylated
cg14174336	0.4583	0.330131892	-0.47325	0.016061	hypomethylated
cg27178401	0.83757	0.603329189	-0.473264	3.95E-05	hypomethylated
cg13112524	0.70299	0.506385946	-0.473267	3.85E-05	hypomethylated
cg25515997	0.84063	0.605518919	-0.473299	7.29E-05	hypomethylated
cg22376262	0.70973	0.511216216	-0.473337	0.000545	hypomethylated
cg15634747	0.60252	0.433981622	-0.473375	0.000262	hypomethylated
cg07261186	0.84347	0.607514595	-0.473418	4.58E-05	hypomethylated
cg04977528	0.75029	0.540394054	-0.473437	0.000361	hypomethylated
cg22996170	0.73953	0.532641081	-0.473445	9.72E-05	hypomethylated
cg08205396	0.70523	0.507921081	-0.473489	0.00012	hypomethylated
cg26522708	0.64996	0.468108108	-0.473509	0.000102	hypomethylated
cg22525069	0.84667	0.609775676	-0.473521	9.38E-07	hypomethylated
cg13431028	0.8777	0.632103243	-0.473568	2.71E-05	hypomethylated
cg20856545	0.68981	0.496782703	-0.473584	6.95E-05	hypomethylated
cg12685560	0.42067	0.302942162	-0.473647	0.001571	hypomethylated
cg26366616	0.76869	0.553518919	-0.473769	4.47E-05	hypomethylated
cg24593464	0.68208	0.491128649	-0.47384	3.12E-06	hypomethylated
cg09439204	0.64413	0.463800541	-0.473847	0.000718	hypomethylated
cg17132079	0.47723	0.343599459	-0.473957	5.86E-05	hypomethylated
cg25616514	0.55374	0.398674595	-0.473997	7.65E-05	hypomethylated

cg21915337	0.76208	0.548638919	-0.474085	0.000112	hypomethylated
cg25428297	0.43582	0.313747027	-0.474131	0.00031	hypomethylated
cg04435807	0.82719	0.59544973	-0.474239	1.26E-05	hypomethylated
cg18760360	0.69217	0.498230811	-0.474312	3.07E-05	hypomethylated
cg15600430	0.4989	0.359110811	-0.474322	0.000245	hypomethylated
cg07107130	0.52869	0.380553514	-0.474323	0.007601	hypomethylated
cg09650189	0.80994	0.58299027	-0.474343	2.85E-05	hypomethylated
cg04435320	0.84487	0.608117838	-0.474378	2.10E-05	hypomethylated
cg25755057	0.78444	0.564611892	-0.474404	2.15E-05	hypomethylated
cg19450816	0.6495	0.467479459	-0.474427	0.000207	hypomethylated
cg02353048	0.86709	0.624060541	-0.474496	5.86E-05	hypomethylated
cg07271261	0.65914	0.474370811	-0.47457	3.81E-05	hypomethylated
cg00152878	0.80755	0.581153514	-0.474632	0.000112	hypomethylated
cg08529744	0.44061	0.317070811	-0.474697	1.80E-05	hypomethylated
cg06700234	0.70563	0.507779459	-0.47471	0.000412	hypomethylated
cg27278470	0.73608	0.529677297	-0.474749	3.85E-06	hypomethylated
cg14911766	0.56142	0.403990811	-0.474758	0.000293	hypomethylated
cg10525372	0.85172	0.61285027	-0.474845	4.48E-06	hypomethylated
cg13442606	0.41134	0.295969189	-0.474884	0.000174	hypomethylated
cg03567939	0.83591	0.601455676	-0.474889	1.25E-05	hypomethylated
cg06707993	0.87374	0.628668649	-0.474904	3.12E-06	hypomethylated
cg17588094	0.91329	0.657122703	-0.47491	1.15E-05	hypomethylated
cg18883317	0.78215	0.562737297	-0.474984	2.71E-06	hypomethylated
cg07531549	0.74622	0.536833514	-0.475126	9.27E-05	hypomethylated
cg13821785	0.76044	0.547035676	-0.475199	5.72E-05	hypomethylated
cg08399733	0.92349	0.664282703	-0.475299	1.15E-05	hypomethylated
cg07018107	0.78448	0.564265405	-0.475363	5.19E-05	hypomethylated
cg02073525	0.79936	0.574967027	-0.475366	2.56E-06	hypomethylated
cg25928474	0.75742	0.544764865	-0.47546	3.49E-05	hypomethylated
cg27152299	0.77175	0.55506973	-0.475465	1.49E-06	hypomethylated
cg11344950	0.62685	0.45084973	-0.475474	8.37E-06	hypomethylated
cg21572000	0.85106	0.612105405	-0.475481	5.25E-05	hypomethylated
cg00687714	0.68946	0.495827027	-0.47563	0.000369	hypomethylated
cg01337514	0.6374	0.458377838	-0.475662	4.12E-06	hypomethylated
cg04813834	0.69018	0.496312432	-0.475724	4.70E-05	hypomethylated
cg17454094	0.50015	0.359645405	-0.475786	0.001381	hypomethylated
cg07172334	0.80616	0.579642162	-0.475904	6.08E-05	hypomethylated
cg06813100	0.44739	0.321668649	-0.475958	2.48E-05	hypomethylated
cg01845036	0.79406	0.570895676	-0.476021	3.11E-05	hypomethylated
cg10385692	0.60468	0.434683784	-0.476206	6.86E-05	hypomethylated
cg17208748	0.43557	0.313108108	-0.476244	0.000207	hypomethylated
cg21545548	0.89869	0.645994054	-0.476303	4.12E-06	hypomethylated
cg13703437	0.65546	0.471139459	-0.476354	0.000506	hypomethylated
cg21118780	0.77557	0.557425946	-0.476477	2.39E-05	hypomethylated
cg07944862	0.67927	0.488206486	-0.476494	0.000626	hypomethylated
cg15928742	0.82382	0.592093514	-0.476504	9.49E-05	hypomethylated
cg23765332	0.9068	0.651722703	-0.476526	9.11E-07	hypomethylated
cg00686132	0.67743	0.486872973	-0.476526	0.000696	hypomethylated
cg07991100	0.79433	0.570868108	-0.476581	1.67E-06	hypomethylated
cg04741094	0.83955	0.603342703	-0.476639	1.50E-05	hypomethylated
cg16676676	0.94872	0.681764324	-0.476709	3.39E-06	hypomethylated
cg21272843	0.79284	0.569702703	-0.476821	2.39E-05	hypomethylated
cg00475131	0.84711	0.608682162	-0.47686	6.39E-06	hypomethylated
cg08515811	0.93533	0.672070811	-0.476862	4.01E-06	hypomethylated
cg18914751	0.82166	0.590379459	-0.476899	1.12E-05	hypomethylated
cg06996254	0.82228	0.590793514	-0.476976	0.00035	hypomethylated
cg23552820	0.67673	0.486205405	-0.477014	1.99E-05	hypomethylated
cg02719634	0.81282	0.583955135	-0.477078	1.48E-05	hypomethylated

cg14252164	0.80688	0.579638919	-0.4772	4.36E-06	hypomethylated
cg13856674	0.66866	0.480344324	-0.477204	9.16E-05	hypomethylated
cg23366752	0.82309	0.591276216	-0.477218	3.79E-06	hypomethylated
cg14074174	0.46065	0.330905946	-0.47725	0.000523	hypomethylated
cg03420978	0.56653	0.406963243	-0.477254	0.00054	hypomethylated
cg06580770	0.69893	0.502029189	-0.477377	3.53E-05	hypomethylated
cg02258414	0.69	0.495614595	-0.477378	0.000324	hypomethylated
cg18114294	0.58086	0.417208649	-0.477421	0.000931	hypomethylated
cg07974367	0.79059	0.567846486	-0.477429	3.21E-06	hypomethylated
cg02666638	0.29496	0.211845405	-0.477507	5.38E-05	hypomethylated
cg06285439	0.60937	0.437658378	-0.477513	1.62E-05	hypomethylated
cg23378074	0.73045	0.524618919	-0.477516	0.000271	hypomethylated
cg21769117	0.66035	0.474267568	-0.47753	0.000335	hypomethylated
cg25299319	0.72657	0.521807568	-0.477584	6.22E-06	hypomethylated
cg06814287	0.68805	0.494141622	-0.477589	0.000626	hypomethylated
cg05042708	0.44606	0.320340541	-0.477631	0.00064	hypomethylated
cg08320359	0.88301	0.634136757	-0.477636	8.15E-06	hypomethylated
cg05447343	0.70968	0.509652973	-0.477653	3.23E-05	hypomethylated
cg25758828	0.71113	0.510685946	-0.477677	0.000417	hypomethylated
cg13693256	0.70431	0.505769189	-0.477731	5.58E-05	hypomethylated
cg26799474	0.78141	0.561135135	-0.477731	0.000626	hypomethylated
cg13512951	0.57346	0.411797297	-0.477758	6.62E-05	hypomethylated
cg10773972	0.80625	0.578891892	-0.477933	5.31E-05	hypomethylated
cg21604970	0.84184	0.604428649	-0.477974	1.44E-05	hypomethylated
cg07126115	0.53318	0.382807027	-0.478005	0.003571	hypomethylated
cg10272520	0.93832	0.673681622	-0.478013	1.88E-06	hypomethylated
cg24170085	0.5628	0.404047568	-0.478097	0.000805	hypomethylated
cg19988367	0.52241	0.375042162	-0.47813	0.000214	hypomethylated
cg06348773	0.47877	0.343710811	-0.478138	0.000335	hypomethylated
cg26110900	0.84746	0.608374595	-0.478185	4.47E-05	hypomethylated
cg13772414	0.77979	0.559794054	-0.478189	0.000681	hypomethylated
cg18975130	0.85052	0.610566486	-0.478197	9.19E-06	hypomethylated
cg06819373	0.73563	0.528086486	-0.478206	0.000132	hypomethylated
cg13829089	0.7029	0.504576216	-0.478247	0.000741	hypomethylated
cg05361415	0.76963	0.552406486	-0.478435	1.50E-05	hypomethylated
cg22388417	0.8585	0.616128649	-0.478587	0.000117	hypomethylated
cg04014685	0.87271	0.626321622	-0.478599	3.64E-06	hypomethylated
cg08985530	0.82914	0.595043243	-0.478621	2.32E-05	hypomethylated
cg06621452	0.76243	0.547167027	-0.478624	5.45E-05	hypomethylated
cg06549238	0.89764	0.64417027	-0.478695	0.000155	hypomethylated
cg18949415	0.52758	0.378595676	-0.478732	4.26E-05	hypomethylated
cg01577029	0.78849	0.565754595	-0.478916	3.40E-05	hypomethylated
cg13488851	0.86674	0.621894595	-0.478929	4.24E-06	hypomethylated
cg06267075	0.71943	0.516175135	-0.478994	0.000287	hypomethylated
cg19373545	0.87874	0.630428649	-0.479103	9.16E-05	hypomethylated
cg22089361	0.79422	0.569735676	-0.479246	3.49E-05	hypomethylated
cg00840694	0.66737	0.478732973	-0.479266	1.18E-05	hypomethylated
cg02499614	0.43639	0.313024865	-0.479341	1.28E-05	hypomethylated
cg13295878	0.83266	0.597255676	-0.479379	1.44E-05	hypomethylated
cg13697968	0.87083	0.62463027	-0.479389	4.73E-06	hypomethylated
cg03885055	0.55661	0.399244324	-0.479395	0.000313	hypomethylated
cg12941931	0.55978	0.401502162	-0.479452	0.001423	hypomethylated
cg16549596	0.73801	0.529303243	-0.479546	4.60E-06	hypomethylated
cg11362820	0.49249	0.353212432	-0.479558	0.001511	hypomethylated
cg11201177	0.79109	0.567307027	-0.479712	7.52E-06	hypomethylated
cg05845141	0.76225	0.546605946	-0.479763	3.59E-06	hypomethylated
cg04761746	0.51712	0.370813514	-0.479805	0.000237	hypomethylated
cg05461841	0.42829	0.3071	-0.479879	7.65E-05	hypomethylated

cg24299913	0.73998	0.530591351	-0.479885	2.71E-05	hypomethylated
cg05663278	0.75184	0.539084865	-0.479913	0.000374	hypomethylated
cg23971987	0.74187	0.531921081	-0.479954	6.38E-05	hypomethylated
cg22907295	0.61087	0.437954054	-0.480086	0.000154	hypomethylated
cg07740599	0.74465	0.533854054	-0.480117	0.000182	hypomethylated
cg17056676	0.52697	0.377794595	-0.480119	6.46E-05	hypomethylated
cg19375403	0.6231	0.446704324	-0.480143	0.006915	hypomethylated
cg20035679	0.7442	0.533502162	-0.480196	3.67E-05	hypomethylated
cg05105919	0.50209	0.359924865	-0.48025	0.000893	hypomethylated
cg18665384	0.707	0.506797297	-0.480301	1.46E-05	hypomethylated
cg20909017	0.56219	0.40296973	-0.480386	0.003021	hypomethylated
cg16523490	0.51387	0.368323784	-0.480429	4.76E-05	hypomethylated
cg04547002	0.60437	0.43318973	-0.480433	0.000193	hypomethylated
cg09911316	0.63626	0.456015676	-0.480533	4.15E-05	hypomethylated
cg25497530	0.75323	0.539839459	-0.48056	8.52E-05	hypomethylated
cg04560093	0.84183	0.603338378	-0.480562	0.000117	hypomethylated
cg09541248	0.70544	0.505582703	-0.480576	1.26E-05	hypomethylated
cg01905967	0.73724	0.528307027	-0.480758	0.000256	hypomethylated
cg22166883	0.55289	0.396185946	-0.480815	6.78E-05	hypomethylated
cg10060191	0.66235	0.474614595	-0.480837	0.000839	hypomethylated
cg21580456	0.81283	0.582413514	-0.48091	4.73E-06	hypomethylated
cg16015285	0.77032	0.551943784	-0.480937	9.57E-06	hypomethylated
cg15100947	0.89425	0.640740541	-0.480938	3.04E-06	hypomethylated
cg10923018	0.81856	0.586501622	-0.480953	1.77E-06	hypomethylated
cg19861914	0.89239	0.639370811	-0.481021	7.32E-06	hypomethylated
cg01033642	0.85087	0.609607568	-0.481058	3.67E-05	hypomethylated
cg20441130	0.72091	0.516483784	-0.481096	2.95E-06	hypomethylated
cg17311132	0.55857	0.400169189	-0.481128	0.000331	hypomethylated
cg19580263	0.87858	0.629415676	-0.481161	1.54E-05	hypomethylated
cg27031754	0.52235	0.374210811	-0.481166	0.001212	hypomethylated
cg23303108	0.91509	0.655555676	-0.481195	2.42E-05	hypomethylated
cg23414330	0.45677	0.327214054	-0.481233	3.71E-05	hypomethylated
cg00661399	0.68477	0.490491351	-0.481392	2.57E-05	hypomethylated
cg01997884	0.70217	0.50294973	-0.481406	0.000557	hypomethylated
cg12705693	0.72957	0.522575676	-0.481406	0.000145	hypomethylated
cg20488756	0.6961	0.498545405	-0.48157	5.00E-06	hypomethylated
cg18614510	0.60082	0.430297838	-0.481597	9.27E-05	hypomethylated
cg23969274	0.77471	0.554784324	-0.481729	1.39E-05	hypomethylated
cg00868074	0.63409	0.454074054	-0.48176	1.31E-05	hypomethylated
cg25743482	0.82392	0.59000973	-0.481766	7.93E-06	hypomethylated
cg07060794	0.76886	0.550561081	-0.481818	4.94E-05	hypomethylated
cg03666309	0.61268	0.438696757	-0.48191	3.15E-05	hypomethylated
cg16701133	0.82623	0.591571892	-0.48199	1.89E-05	hypomethylated
cg16459276	0.60656	0.434282162	-0.482018	0.002296	hypomethylated
cg04724556	0.76368	0.546771351	-0.482031	0.00015	hypomethylated
cg06772221	0.53324	0.381777838	-0.482052	0.000772	hypomethylated
cg06901893	0.72911	0.52196973	-0.48217	1.58E-05	hypomethylated
cg26508164	0.82549	0.590956757	-0.482198	4.76E-05	hypomethylated
cg11862144	0.93212	0.667286486	-0.482209	1.39E-05	hypomethylated
cg18608610	0.66159	0.473591351	-0.482295	7.52E-06	hypomethylated
cg09260048	0.88568	0.633982162	-0.482343	8.37E-06	hypomethylated
cg11297227	0.59945	0.429089189	-0.482362	2.96E-05	hypomethylated
cg19451021	0.75393	0.539666486	-0.482362	3.44E-05	hypomethylated
cg14161399	0.84171	0.602447568	-0.482488	1.28E-05	hypomethylated
cg00621646	0.83211	0.595561081	-0.482525	1.82E-05	hypomethylated
cg02131870	0.89778	0.642523243	-0.482613	7.62E-06	hypomethylated
cg18564686	0.49182	0.351975135	-0.482657	0.001314	hypomethylated
cg08091666	0.6896	0.493474054	-0.482786	1.54E-05	hypomethylated

cg19531536	0.59393	0.42501027	-0.482795	5.31E-05	hypomethylated
cg17316966	0.7839	0.56095027	-0.482797	9.16E-05	hypomethylated
cg09040942	0.82231	0.588405405	-0.482872	0.000166	hypomethylated
cg20521198	0.57885	0.414183243	-0.48292	0.000395	hypomethylated
cg09861057	0.49863	0.35678	-0.482935	0.00071	hypomethylated
cg18640660	0.74034	0.529721081	-0.482955	3.90E-06	hypomethylated
cg02217022	0.68397	0.489385405	-0.482962	3.95E-05	hypomethylated
cg05316065	0.54418	0.389363243	-0.482967	9.72E-05	hypomethylated
cg10743102	0.77464	0.554243243	-0.483007	0.000474	hypomethylated
cg13172432	0.67773	0.484895135	-0.483038	5.72E-05	hypomethylated
cg25403488	0.64578	0.462021622	-0.483082	9.49E-05	hypomethylated
cg13374701	0.93634	0.66989027	-0.483108	7.72E-06	hypomethylated
cg00093433	0.59812	0.427896757	-0.483172	0.00048	hypomethylated
cg14325112	0.77872	0.557082162	-0.483215	2.67E-05	hypomethylated
cg14413177	0.60862	0.435392973	-0.483224	1.25E-05	hypomethylated
cg05363382	0.66912	0.478639459	-0.483326	6.31E-05	hypomethylated
cg01862897	0.62411	0.446435135	-0.48335	0.000126	hypomethylated
cg06869641	0.64422	0.460818919	-0.483354	1.38E-05	hypomethylated
cg05991902	0.76689	0.548521081	-0.483473	2.39E-05	hypomethylated
cg13714378	0.77804	0.556485405	-0.4835	5.45E-05	hypomethylated
cg08942191	0.66036	0.472302162	-0.483543	0.000342	hypomethylated
cg21491443	0.89785	0.642137297	-0.483593	1.93E-06	hypomethylated
cg05728596	0.86384	0.617782703	-0.483665	1.62E-05	hypomethylated
cg00543474	0.5183	0.370661622	-0.483685	0.00095	hypomethylated
cg22421417	0.71779	0.513322703	-0.483696	6.62E-05	hypomethylated
cg15774065	0.56729	0.405692973	-0.483698	3.32E-05	hypomethylated
cg04285710	0.69216	0.49497027	-0.483764	8.63E-05	hypomethylated
cg18293437	0.70425	0.503607568	-0.483788	3.07E-05	hypomethylated
cg08468599	0.74851	0.535246486	-0.483818	0.000101	hypomethylated
cg15579389	0.52229	0.373443784	-0.48396	0.003506	hypomethylated
cg06884882	0.44765	0.320072973	-0.48397	0.000588	hypomethylated
cg25440893	0.40131	0.286923784	-0.48405	0.000123	hypomethylated
cg16337430	0.55879	0.399507027	-0.484085	0.002026	hypomethylated
cg24537836	0.64122	0.458392432	-0.484236	0.000772	hypomethylated
cg09071093	0.54125	0.386921081	-0.484256	0.000219	hypomethylated
cg19161251	0.87233	0.623594595	-0.484266	7.52E-06	hypomethylated
cg11844042	0.87333	0.624294054	-0.484301	4.12E-06	hypomethylated
cg08715862	0.52763	0.377167568	-0.484321	0.000354	hypomethylated
cg26282283	0.55689	0.398076216	-0.484348	0.000374	hypomethylated
cg22732126	0.5863	0.419088649	-0.484384	1.80E-05	hypomethylated
cg05295023	0.78196	0.558934595	-0.484415	6.02E-07	hypomethylated
cg06310712	0.85276	0.609526486	-0.484451	2.87E-06	hypomethylated
cg05447102	0.82564	0.590101081	-0.484551	1.99E-05	hypomethylated
cg09030187	0.56343	0.402688649	-0.484572	0.000445	hypomethylated
cg26689077	0.53818	0.384615135	-0.484673	0.004365	hypomethylated
cg03096107	0.40322	0.288161081	-0.484692	0.00012	hypomethylated
cg06647928	0.49948	0.35694973	-0.484706	6.38E-05	hypomethylated
cg00325139	0.67049	0.47916	-0.484708	0.000893	hypomethylated
cg01769428	0.49897	0.356583243	-0.484714	9.72E-05	hypomethylated
cg04843461	0.73113	0.52248973	-0.484725	2.71E-05	hypomethylated
cg15050051	0.71044	0.507686486	-0.484775	0.000523	hypomethylated
cg04961225	0.7315	0.522735676	-0.484776	0.00095	hypomethylated
cg20932150	0.41885	0.299297297	-0.484854	1.01E-05	hypomethylated
cg01759628	0.53582	0.382869189	-0.484897	0.000306	hypomethylated
cg02643667	0.84692	0.605089189	-0.485078	4.36E-06	hypomethylated
cg03453870	0.76855	0.549069189	-0.485151	7.12E-05	hypomethylated
cg19106326	0.71861	0.513381622	-0.485177	0.00028	hypomethylated
cg16120196	0.67838	0.484636216	-0.485191	3.49E-05	hypomethylated

cg04189320	0.74999	0.53576973	-0.485258	6.39E-06	hypomethylated
cg04626878	0.47109	0.336531892	-0.485259	0.001481	hypomethylated
cg18844900	0.79485	0.567753514	-0.485418	5.73E-06	hypomethylated
cg26551026	0.85701	0.61214973	-0.485427	1.46E-05	hypomethylated
cg15664152	0.41881	0.299140541	-0.485472	0.005085	hypomethylated
cg17384457	0.58962	0.421139459	-0.485487	1.75E-05	hypomethylated
cg06743312	0.44266	0.316165405	-0.485519	5.72E-05	hypomethylated
cg16856722	0.89476	0.639050811	-0.48557	2.95E-06	hypomethylated
cg15692360	0.6086	0.434668649	-0.485578	5.38E-05	hypomethylated
cg09968361	0.67243	0.480244324	-0.485616	2.45E-05	hypomethylated
cg23670779	0.78263	0.558944324	-0.485626	5.19E-05	hypomethylated
cg02495767	0.82061	0.585986486	-0.485829	2.27E-05	hypomethylated
cg06873564	0.84195	0.601189189	-0.485915	1.58E-05	hypomethylated
cg24067652	0.79779	0.569656216	-0.485918	2.78E-05	hypomethylated
cg25310700	0.46405	0.331325946	-0.486029	0.000251	hypomethylated
cg15159104	0.5015	0.358059459	-0.486051	0.003225	hypomethylated
cg09214398	0.88962	0.635167027	-0.486053	1.89E-05	hypomethylated
cg19464524	0.521	0.371924324	-0.486274	0.004059	hypomethylated
cg26081974	0.90317	0.644651892	-0.486477	2.04E-05	hypomethylated
cg16661143	0.72616	0.518302703	-0.486493	1.71E-05	hypomethylated
cg03029752	0.68328	0.487693514	-0.486502	1.09E-05	hypomethylated
cg09080444	0.67727	0.483389189	-0.486546	2.64E-05	hypomethylated
cg22708944	0.79369	0.566445946	-0.486637	3.57E-05	hypomethylated
cg14120703	0.69084	0.493036216	-0.486658	0.000193	hypomethylated
cg02006134	0.57492	0.410292432	-0.486709	0.001481	hypomethylated
cg19573230	0.85115	0.607422703	-0.486713	7.32E-06	hypomethylated
cg18198461	0.48253	0.344342703	-0.486774	9.27E-05	hypomethylated
cg16919680	0.72736	0.519043243	-0.486815	0.000797	hypomethylated
cg00375608	0.89913	0.641541081	-0.486988	5.00E-06	hypomethylated
cg25407528	0.63791	0.455155135	-0.486995	0.000262	hypomethylated
cg17379860	0.50491	0.36022	-0.487148	1.06E-05	hypomethylated
cg25271976	0.78076	0.556998919	-0.487205	8.37E-06	hypomethylated
cg00748226	0.66463	0.474144324	-0.487225	1.80E-05	hypomethylated
cg07492281	0.85692	0.611273514	-0.487342	1.29E-06	hypomethylated
cg14839558	0.93572	0.667484324	-0.487343	6.57E-06	hypomethylated
cg06431681	0.82581	0.589077297	-0.487353	1.60E-05	hypomethylated
cg07095737	0.68014	0.485164324	-0.487358	5.65E-05	hypomethylated
cg12911952	0.80803	0.576384324	-0.487378	1.15E-05	hypomethylated
cg05036153	0.48674	0.347200541	-0.487382	0.000137	hypomethylated
cg24696728	0.81417	0.580727568	-0.487469	8.95E-06	hypomethylated
cg27409771	0.39872	0.284396216	-0.487474	0.000123	hypomethylated
cg08210297	0.79179	0.564751351	-0.487502	4.60E-06	hypomethylated
cg03469682	0.80655	0.575269189	-0.487527	4.47E-05	hypomethylated
cg15132169	0.52488	0.374331892	-0.48767	0.001118	hypomethylated
cg08009669	0.44674	0.318596757	-0.487704	0.006451	hypomethylated
cg11097954	0.61511	0.438641622	-0.487802	0.001153	hypomethylated
cg16306870	0.74536	0.531468649	-0.487953	0.000667	hypomethylated
cg08306303	0.89639	0.639131351	-0.488014	8.83E-06	hypomethylated
cg24925163	0.65338	0.465818919	-0.488153	4.60E-06	hypomethylated
cg17941109	0.74104	0.528268649	-0.48828	7.42E-06	hypomethylated
cg23568538	0.60672	0.43248973	-0.488365	5.72E-05	hypomethylated
cg20156116	0.48575	0.346235676	-0.48846	0.000857	hypomethylated
cg22352499	0.81786	0.582851351	-0.488726	2.87E-06	hypomethylated
cg12970937	0.72249	0.514861622	-0.488793	0.000224	hypomethylated
cg08094614	0.85161	0.606785946	-0.489005	6.75E-06	hypomethylated
cg13340959	0.53026	0.377803243	-0.489065	1.80E-05	hypomethylated
cg03283080	0.73894	0.526439459	-0.48919	6.15E-05	hypomethylated
cg26596307	0.56906	0.40541027	-0.489198	0.000191	hypomethylated

cg26566898	0.52599	0.374711351	-0.489256	0.000435	hypomethylated
cg15319824	0.90712	0.646222162	-0.489263	2.10E-05	hypomethylated
cg03893663	0.90448	0.644321081	-0.489309	4.12E-06	hypomethylated
cg21303745	0.87111	0.620543243	-0.489323	6.66E-06	hypomethylated
cg06196689	0.76099	0.542096216	-0.489329	7.74E-05	hypomethylated
cg11577329	0.83363	0.593841081	-0.48933	4.01E-06	hypomethylated
cg09057412	0.79356	0.565276757	-0.489382	8.37E-06	hypomethylated
cg26495711	0.60178	0.428663784	-0.48939	1.87E-05	hypomethylated
cg18818949	0.73823	0.525851892	-0.489414	5.06E-05	hypomethylated
cg26530341	0.80203	0.571295135	-0.48942	9.49E-05	hypomethylated
cg21156590	0.82867	0.590247568	-0.489478	1.80E-05	hypomethylated
cg15073164	0.7677	0.546812432	-0.489497	2.05E-06	hypomethylated
cg15059474	0.64819	0.46166	-0.489586	0.000109	hypomethylated
cg07696099	0.70627	0.503019459	-0.489606	0.00012	hypomethylated
cg06688014	0.3975	0.283090811	-0.48969	4.31E-05	hypomethylated
cg00599530	0.51078	0.363742703	-0.489784	0.00024	hypomethylated
cg01880463	0.54982	0.391541622	-0.489794	0.000193	hypomethylated
cg02977443	0.72904	0.519168108	-0.489796	5.86E-05	hypomethylated
cg12217954	0.8153	0.58059027	-0.489811	8.42E-05	hypomethylated
cg12157156	0.62299	0.443629189	-0.489855	1.60E-05	hypomethylated
cg03678566	0.78213	0.556949189	-0.489863	1.66E-05	hypomethylated
cg08454125	0.53349	0.379894054	-0.489864	3.95E-05	hypomethylated
cg16755251	0.92794	0.660771892	-0.489879	2.71E-06	hypomethylated
cg04250837	0.80716	0.574758919	-0.489898	9.95E-05	hypomethylated
cg18801579	0.82314	0.586131351	-0.489914	1.46E-05	hypomethylated
cg25737218	0.77093	0.548919459	-0.490005	5.58E-06	hypomethylated
cg15026767	0.72567	0.516687568	-0.490021	1.31E-05	hypomethylated
cg14036402	0.44694	0.318210811	-0.490098	0.001164	hypomethylated
cg08919846	0.90251	0.642564324	-0.490102	2.54E-05	hypomethylated
cg21690505	0.72858	0.518725405	-0.490116	6.75E-06	hypomethylated
cg04388548	0.61359	0.436850811	-0.490134	1.42E-05	hypomethylated
cg07161179	0.6365	0.453143784	-0.490192	0.000147	hypomethylated
cg12019109	0.84484	0.601455135	-0.490221	4.24E-06	hypomethylated
cg09485853	0.6315	0.449556757	-0.490279	0.000187	hypomethylated
cg08804626	0.93055	0.662432973	-0.490309	1.26E-06	hypomethylated
cg05405389	0.70628	0.50274	-0.490428	0.000126	hypomethylated
cg08827454	0.65015	0.462776757	-0.490456	0.000575	hypomethylated
cg13093389	0.68043	0.484317838	-0.490493	5.58E-05	hypomethylated
cg18449135	0.90593	0.644803784	-0.490539	2.23E-06	hypomethylated
cg20636714	0.74976	0.533632432	-0.490583	1.52E-05	hypomethylated
cg13845858	0.77981	0.55501027	-0.490608	1.93E-06	hypomethylated
cg03761477	0.52486	0.373537838	-0.490678	0.001875	hypomethylated
cg13882377	0.53546	0.381058378	-0.490767	0.000148	hypomethylated
cg21022906	0.8006	0.56974	-0.490778	1.28E-05	hypomethylated
cg15261730	0.55902	0.397818919	-0.490788	0.000116	hypomethylated
cg14760797	0.7607	0.541315676	-0.490857	0.000454	hypomethylated
cg12018888	0.61764	0.439505946	-0.490884	2.71E-05	hypomethylated
cg26907768	0.29537	0.210178919	-0.490905	0.00064	hypomethylated
cg04811592	0.81966	0.583245405	-0.490923	6.01E-05	hypomethylated
cg05996671	0.7028	0.500061081	-0.49101	3.81E-05	hypomethylated
cg11839815	0.82008	0.583506486	-0.491016	5.06E-05	hypomethylated
cg18147048	0.87135	0.619985405	-0.491018	6.78E-05	hypomethylated
cg18126802	0.73963	0.52625027	-0.491055	0.0002	hypomethylated
cg00732383	0.55963	0.398155135	-0.491143	0.000681	hypomethylated
cg26470696	0.74883	0.532745946	-0.491191	5.89E-06	hypomethylated
cg14459021	0.94904	0.675181622	-0.491193	9.82E-06	hypomethylated
cg09649347	0.64231	0.456945946	-0.491246	0.000147	hypomethylated
cg07369606	0.77846	0.553783243	-0.491302	1.42E-05	hypomethylated

cg24554151	0.90474	0.643616216	-0.491303	2.36E-06	hypomethylated
cg14003265	0.44126	0.313868649	-0.491468	0.000551	hypomethylated
cg14059257	0.65572	0.466405946	-0.491494	5.58E-05	hypomethylated
cg10499172	0.46766	0.332634054	-0.491524	0.00035	hypomethylated
cg07186199	0.67813	0.48232973	-0.491542	0.00013	hypomethylated
cg06339706	0.64938	0.461871892	-0.49157	0.000191	hypomethylated
cg21756208	0.52965	0.376705946	-0.491601	9.16E-05	hypomethylated
cg18246298	0.74519	0.530004324	-0.491604	2.81E-05	hypomethylated
cg16218715	0.48018	0.341498919	-0.491694	1.94E-05	hypomethylated
cg03956820	0.5028	0.35756973	-0.49176	0.00097	hypomethylated
cg24078695	0.84424	0.600384865	-0.491766	2.87E-06	hypomethylated
cg20326647	0.9087	0.646191351	-0.491843	0.000124	hypomethylated
cg07100542	0.49868	0.354618919	-0.491845	0.001212	hypomethylated
cg20625334	0.81477	0.579346486	-0.491966	2.32E-05	hypomethylated
cg02997796	0.67471	0.47975027	-0.491984	8.63E-05	hypomethylated
cg10950111	0.80548	0.572722162	-0.492013	3.49E-05	hypomethylated
cg02560081	0.67863	0.482517297	-0.492045	0.000287	hypomethylated
cg02389859	0.61389	0.436484865	-0.492049	7.12E-06	hypomethylated
cg21519058	0.59755	0.424797297	-0.492285	6.31E-05	hypomethylated
cg07012178	0.77327	0.549712432	-0.492295	3.79E-06	hypomethylated
cg13365753	0.71701	0.509714054	-0.492305	6.93E-06	hypomethylated
cg17274742	0.72447	0.514953514	-0.492484	0.000107	hypomethylated
cg16332435	0.84109	0.597776216	-0.492655	4.70E-05	hypomethylated
cg03628441	0.7934	0.563862162	-0.492706	4.12E-06	hypomethylated
cg18322822	0.84388	0.599723784	-0.49274	8.59E-07	hypomethylated
cg08782899	0.71986	0.511584324	-0.492744	1.93E-06	hypomethylated
cg03129324	0.41642	0.295933514	-0.492766	0.000293	hypomethylated
cg22121647	0.72054	0.512012432	-0.4929	0.00014	hypomethylated
cg25541968	0.58558	0.416095676	-0.492951	0.000122	hypomethylated
cg04717143	0.59587	0.423399459	-0.492978	0.000445	hypomethylated
cg02854937	0.36333	0.258158919	-0.493021	0.000454	hypomethylated
cg02650512	0.71325	0.506773514	-0.493067	3.07E-05	hypomethylated
cg25475538	0.69295	0.492332973	-0.493117	1.11E-05	hypomethylated
cg07771160	0.67606	0.480323784	-0.493144	0.000485	hypomethylated
cg03607573	0.6674	0.474157297	-0.493186	0.000138	hypomethylated
cg17435683	0.6032	0.428538919	-0.49321	0.000154	hypomethylated
cg19885306	0.44384	0.315322162	-0.493213	0.00028	hypomethylated
cg18728264	0.66678	0.473698378	-0.493242	0.000224	hypomethylated
cg04157272	0.85581	0.607918919	-0.493412	4.15E-05	hypomethylated
cg16325826	0.93777	0.666123784	-0.493444	2.30E-05	hypomethylated
cg06624951	0.65823	0.467540541	-0.4935	4.47E-05	hypomethylated
cg24425149	0.4374	0.310664865	-0.493594	0.000772	hypomethylated
cg18615133	0.65163	0.462820541	-0.4936	0.000501	hypomethylated
cg10663897	0.73834	0.524393514	-0.493635	0.000256	hypomethylated
cg13242944	0.82071	0.582858378	-0.493727	2.16E-06	hypomethylated
cg00373436	0.5957	0.423058378	-0.493729	3.32E-05	hypomethylated
cg21157904	0.55167	0.391768108	-0.493806	0.000129	hypomethylated
cg17638841	0.55863	0.396699459	-0.493847	1.84E-05	hypomethylated
cg12983285	0.55558	0.394516216	-0.49391	0.000485	hypomethylated
cg10687219	0.90296	0.64117027	-0.493955	2.61E-05	hypomethylated
cg00238283	0.72568	0.515265946	-0.494016	3.85E-05	hypomethylated
cg14761989	0.535	0.379864865	-0.494053	8.42E-05	hypomethylated
cg05235525	0.57351	0.407207027	-0.494056	1.92E-05	hypomethylated
cg26206185	0.54897	0.389777838	-0.494075	5.06E-05	hypomethylated
cg21058511	0.72442	0.514314595	-0.494175	0.000112	hypomethylated
cg03531754	0.74966	0.532227027	-0.494195	8.15E-06	hypomethylated
cg04483701	0.64563	0.458367568	-0.494203	3.00E-05	hypomethylated
cg25004838	0.86459	0.613816757	-0.494208	4.05E-05	hypomethylated

cg14691971	0.80741	0.573221622	-0.494208	0.000161	hypomethylated
cg06641593	0.87662	0.622355135	-0.494214	7.72E-06	hypomethylated
cg02493905	0.70291	0.498975135	-0.494372	2.24E-05	hypomethylated
cg18356190	0.53742	0.381495135	-0.494385	0.001107	hypomethylated
cg10548968	0.74263	0.52712973	-0.494486	2.64E-06	hypomethylated
cg14924160	0.85662	0.60802973	-0.494513	2.32E-05	hypomethylated
cg05260877	0.68164	0.483808649	-0.494573	1.42E-05	hypomethylated
cg05798627	0.71066	0.504392432	-0.494613	6.93E-06	hypomethylated
cg16567056	0.54369	0.385878919	-0.494636	0.007667	hypomethylated
cg20708135	0.74669	0.529956216	-0.494636	3.00E-05	hypomethylated
cg13865610	0.77201	0.54788	-0.49476	0.000191	hypomethylated
cg19194645	0.72812	0.51673027	-0.494765	2.05E-06	hypomethylated
cg12313969	0.87682	0.622257297	-0.494769	3.23E-05	hypomethylated
cg09695261	0.95519	0.677872432	-0.494774	2.42E-06	hypomethylated
cg22329743	0.80175	0.568976216	-0.494784	3.49E-06	hypomethylated
cg08710911	0.47343	0.33596973	-0.49482	0.000506	hypomethylated
cg03884543	0.75186	0.533552973	-0.494833	8.37E-06	hypomethylated
cg16764778	0.54234	0.384858919	-0.494868	0.000426	hypomethylated
cg02899870	0.66779	0.473857838	-0.49494	9.27E-05	hypomethylated
cg25338818	0.84419	0.59901027	-0.494987	4.24E-06	hypomethylated
cg04577212	0.44986	0.319203243	-0.495001	0.002147	hypomethylated
cg04115307	0.61531	0.436558919	-0.495137	5.43E-06	hypomethylated
cg22014579	0.88718	0.62942973	-0.495182	6.39E-06	hypomethylated
cg04650653	0.82267	0.583626486	-0.495268	2.89E-05	hypomethylated
cg15705746	0.57675	0.409156216	-0.495294	0.00024	hypomethylated
cg07177437	0.82532	0.585473514	-0.49535	4.36E-06	hypomethylated
cg01591152	0.57739	0.409562162	-0.495464	3.07E-05	hypomethylated
cg13888509	0.73074	0.518321081	-0.495512	3.69E-06	hypomethylated
cg08621168	0.87261	0.618941081	-0.495535	9.82E-06	hypomethylated
cg26204417	0.90766	0.6438	-0.495539	2.95E-06	hypomethylated
cg15067015	0.6319	0.44818	-0.495618	4.94E-05	hypomethylated
cg00355019	0.85346	0.605243784	-0.495807	1.18E-05	hypomethylated
cg23506979	0.67634	0.479613514	-0.495876	2.45E-05	hypomethylated
cg20715809	0.63442	0.449876216	-0.49591	2.64E-06	hypomethylated
cg02623200	0.48565	0.344380541	-0.495913	0.00125	hypomethylated
cg04714466	0.80252	0.569063243	-0.495948	2.64E-05	hypomethylated
cg04463607	0.73671	0.522396757	-0.495951	5.45E-05	hypomethylated
cg03748603	0.76981	0.545861622	-0.495967	6.78E-05	hypomethylated
cg16348668	0.82866	0.58759027	-0.49597	2.71E-05	hypomethylated
cg24881334	0.7067	0.501106486	-0.495981	0.000528	hypomethylated
cg26037936	0.73117	0.518448108	-0.496007	4.36E-05	hypomethylated
cg15697852	0.75865	0.537919459	-0.496044	0.000426	hypomethylated
cg15975980	0.84915	0.602078919	-0.496067	3.04E-06	hypomethylated
cg21010701	0.84788	0.601177838	-0.496068	1.54E-06	hypomethylated
cg14017402	0.56102	0.397754054	-0.496176	0.000417	hypomethylated
cg15952370	0.69426	0.492179459	-0.496292	0.000163	hypomethylated
cg20667796	0.65993	0.467802703	-0.496413	4.80E-06	hypomethylated
cg05644480	0.55994	0.396911892	-0.496453	3.23E-05	hypomethylated
cg17344040	0.60592	0.429478919	-0.49654	0.00049	hypomethylated
cg07363131	0.51597	0.365672432	-0.496735	0.000214	hypomethylated
cg01796223	0.71965	0.51001027	-0.496769	0.000262	hypomethylated
cg27488807	0.81443	0.577167027	-0.496802	1.42E-05	hypomethylated
cg02570493	0.74987	0.5314	-0.496842	1.31E-05	hypomethylated
cg03980366	0.87546	0.620398919	-0.496845	2.42E-06	hypomethylated
cg23507761	0.75984	0.538458919	-0.496859	6.05E-06	hypomethylated
cg16718760	0.79876	0.56603027	-0.496883	6.95E-05	hypomethylated
cg07866909	0.6325	0.448170811	-0.497017	2.71E-05	hypomethylated
cg01099300	0.73818	0.523021081	-0.497104	3.95E-05	hypomethylated

cg19234983	0.85184	0.603498378	-0.497233	1.28E-05	hypomethylated
cg22433862	0.88443	0.626467027	-0.497509	2.07E-05	hypomethylated
cg20994118	0.52899	0.374684865	-0.497563	1.75E-05	hypomethylated
cg23843180	0.83264	0.589755135	-0.497577	2.27E-05	hypomethylated
cg22704780	0.62724	0.444266486	-0.497592	2.45E-05	hypomethylated
cg04370247	0.73722	0.52209027	-0.497796	0.000358	hypomethylated
cg11342789	0.83951	0.594514595	-0.497835	1.58E-06	hypomethylated
cg25040562	0.77609	0.549577838	-0.4979	1.35E-05	hypomethylated
cg16615508	0.73189	0.518271351	-0.497919	5.50E-06	hypomethylated
cg21650243	0.56894	0.402847568	-0.498042	0.000115	hypomethylated
cg15197218	0.82726	0.585723243	-0.498122	4.01E-06	hypomethylated
cg19279257	0.73723	0.521979459	-0.498122	0.000262	hypomethylated
cg01702055	0.70343	0.498044865	-0.498131	0.003021	hypomethylated
cg26625897	0.75013	0.531071351	-0.498235	9.07E-06	hypomethylated
cg20450318	0.62788	0.444503243	-0.498295	0.000159	hypomethylated
cg04865113	0.51694	0.365945946	-0.498366	0.000229	hypomethylated
cg23392390	0.75874	0.537117838	-0.498367	2.35E-05	hypomethylated
cg26256521	0.66079	0.467759459	-0.498425	0.000102	hypomethylated
cg12684668	0.66596	0.471391892	-0.498509	0.000772	hypomethylated
cg17654660	0.56293	0.398441622	-0.498587	0.000797	hypomethylated
cg15519786	0.60608	0.42894973	-0.4987	4.05E-05	hypomethylated
cg00660272	0.2914	0.206235676	-0.498707	0.001786	hypomethylated
cg03014589	0.88716	0.627696216	-0.499128	5.00E-06	hypomethylated
cg11592230	0.72677	0.514211351	-0.499137	1.17E-05	hypomethylated
cg08759026	0.4572	0.323467568	-0.499204	0.000613	hypomethylated
cg03433986	0.64389	0.45554973	-0.499206	0.000814	hypomethylated
cg02447879	0.55909	0.395550811	-0.499218	0.000374	hypomethylated
cg08553950	0.7496	0.53031027	-0.499284	1.62E-05	hypomethylated
cg15686949	0.50382	0.356415135	-0.49935	0.000313	hypomethylated
cg13419087	0.56573	0.400185405	-0.499445	7.20E-05	hypomethylated
cg14935626	0.44511	0.314860541	-0.499449	0.003316	hypomethylated
cg23149881	0.74374	0.526085405	-0.499501	0.001262	hypomethylated
cg04171808	0.72839	0.515224865	-0.499509	0.000134	hypomethylated
cg16486109	0.81277	0.574885405	-0.499573	1.99E-05	hypomethylated
cg08999895	0.66958	0.473592432	-0.49961	1.21E-05	hypomethylated
cg15565576	0.64956	0.459431892	-0.499612	4.05E-05	hypomethylated
cg22276685	0.76761	0.542905405	-0.499673	1.25E-05	hypomethylated
cg09558425	0.67478	0.477241622	-0.499697	0.000117	hypomethylated
cg15453708	0.64366	0.455205405	-0.499781	0.000168	hypomethylated
cg02000275	0.77334	0.546914054	-0.499789	0.00035	hypomethylated
cg02290880	0.72483	0.512566486	-0.499904	4.26E-05	hypomethylated
cg07611933	0.90134	0.637374595	-0.49993	3.39E-06	hypomethylated
cg15350899	0.53672	0.379527027	-0.499967	9.82E-06	hypomethylated
cg12537003	0.70742	0.500226486	-0.499986	5.45E-05	hypomethylated
cg25318211	0.65395	0.462392432	-0.500063	0.000148	hypomethylated
cg05503433	0.74194	0.524603784	-0.500074	3.90E-06	hypomethylated
cg24416660	0.67611	0.478050811	-0.500094	3.81E-05	hypomethylated
cg02895699	0.80893	0.571909189	-0.500229	0.000161	hypomethylated
cg26648579	0.72346	0.511478919	-0.500238	1.94E-05	hypomethylated
cg10322254	0.55081	0.389416216	-0.500242	0.012333	hypomethylated
cg02780269	0.70777	0.500349189	-0.500345	0.000374	hypomethylated
cg04213854	0.60918	0.430614054	-0.500473	4.86E-06	hypomethylated
cg06128161	0.55634	0.39322973	-0.500594	4.26E-05	hypomethylated
cg08629043	0.72893	0.515216216	-0.500602	6.62E-05	hypomethylated
cg21809161	0.57108	0.403630811	-0.500657	1.87E-05	hypomethylated
cg17804071	0.73625	0.520365946	-0.500669	0.000417	hypomethylated
cg06521247	0.81058	0.572895676	-0.500682	1.02E-05	hypomethylated
cg15221831	0.81219	0.573977838	-0.500822	7.93E-06	hypomethylated

cg10589330	0.77307	0.546329189	-0.500829	6.22E-06	hypomethylated
cg23570810	0.60368	0.426604865	-0.500884	0.000107	hypomethylated
cg13261536	0.93697	0.662104324	-0.500944	8.15E-06	hypomethylated
cg14950321	0.64686	0.457083243	-0.500997	0.0006	hypomethylated
cg23538064	0.57051	0.403129189	-0.50101	6.31E-05	hypomethylated
cg26543150	0.66295	0.468441081	-0.501032	0.000626	hypomethylated
cg25292663	0.61874	0.437197297	-0.501049	0.000511	hypomethylated
cg04625914	0.94989	0.67116	-0.501104	2.68E-06	hypomethylated
cg21018997	0.8132	0.574572432	-0.501121	8.60E-06	hypomethylated
cg03082060	0.68363	0.483010811	-0.50116	1.37E-05	hypomethylated
cg23253961	0.74669	0.527551351	-0.501198	0.000168	hypomethylated
cg11680590	0.79915	0.564587568	-0.501269	5.43E-06	hypomethylated
cg16200531	0.752	0.531253514	-0.501332	0.000726	hypomethylated
cg22721827	0.65369	0.461788108	-0.501376	2.85E-05	hypomethylated
cg27059354	0.81833	0.578051351	-0.501485	2.15E-05	hypomethylated
cg06788764	0.87109	0.615303243	-0.501524	1.06E-05	hypomethylated
cg23459424	0.67697	0.478139459	-0.50166	5.31E-05	hypomethylated
cg20820107	0.52336	0.369622162	-0.501752	0.001912	hypomethylated
cg09990169	0.49287	0.348078919	-0.501793	1.73E-05	hypomethylated
cg13061767	0.91157	0.643760541	-0.501829	1.99E-06	hypomethylated
cg10131026	0.57395	0.405327568	-0.501837	0.000214	hypomethylated
cg00573857	0.67948	0.479841081	-0.501874	0.000454	hypomethylated
cg24847621	0.50321	0.355352973	-0.501908	0.000408	hypomethylated
cg02974085	0.72769	0.513857838	-0.501955	0.000575	hypomethylated
cg10423842	0.55796	0.393982703	-0.502029	0.000772	hypomethylated
cg13551074	0.62805	0.443471892	-0.502037	0.00029	hypomethylated
cg16974832	0.69475	0.490563784	-0.502053	0.000178	hypomethylated
cg15542639	0.8903	0.628621622	-0.5021	3.39E-06	hypomethylated
cg15690542	0.40351	0.284898378	-0.502157	6.95E-05	hypomethylated
cg05704183	0.74356	0.524957838	-0.502248	3.95E-05	hypomethylated
cg14024579	0.51672	0.3648	-0.502277	0.000287	hypomethylated
cg20305578	0.66617	0.470300541	-0.502307	6.78E-05	hypomethylated
cg17865555	0.80907	0.571174054	-0.502334	6.15E-05	hypomethylated
cg06375539	0.85681	0.604864324	-0.502364	2.79E-06	hypomethylated
cg15332871	0.69158	0.488189189	-0.502456	1.99E-06	hypomethylated
cg24136690	0.74269	0.524258378	-0.502482	6.46E-05	hypomethylated
cg05328461	0.75654	0.534021081	-0.50252	1.01E-05	hypomethylated
cg08106792	0.52772	0.372502162	-0.502524	0.000112	hypomethylated
cg02301528	0.57295	0.404422162	-0.502547	0.000102	hypomethylated
cg04713108	0.76005	0.536447568	-0.502657	1.68E-05	hypomethylated
cg25446789	0.71077	0.501648108	-0.502707	0.000805	hypomethylated
cg07359183	0.84624	0.597221081	-0.502802	6.22E-06	hypomethylated
cg19735151	0.90727	0.640281081	-0.502827	3.04E-06	hypomethylated
cg02520707	0.84029	0.592994595	-0.502868	1.09E-05	hypomethylated
cg00743081	0.67257	0.47463027	-0.50288	0.000517	hypomethylated
cg04801085	0.83508	0.589281622	-0.502957	3.19E-05	hypomethylated
cg09575442	0.76219	0.537837297	-0.502981	3.85E-05	hypomethylated
cg20938170	0.66738	0.470924865	-0.503012	7.72E-06	hypomethylated
cg13936846	0.83482	0.589067027	-0.503033	4.05E-05	hypomethylated
cg26590603	0.84693	0.597584865	-0.503099	7.93E-06	hypomethylated
cg09567048	0.78506	0.553904865	-0.503165	2.71E-05	hypomethylated
cg26531231	0.8735	0.616267027	-0.503252	7.32E-06	hypomethylated
cg16384957	0.92194	0.650425946	-0.503288	1.14E-05	hypomethylated
cg14884828	0.71904	0.507274054	-0.503307	0.000613	hypomethylated
cg01540203	0.70564	0.49782	-0.503308	1.05E-06	hypomethylated
cg21497082	0.68274	0.481615135	-0.503456	1.99E-05	hypomethylated
cg24465329	0.79228	0.558826486	-0.50361	5.45E-05	hypomethylated
cg07369374	0.74199	0.52335027	-0.503623	1.45E-06	hypomethylated

cg02314896	0.51188	0.361042162	-0.503638	7.12E-06	hypomethylated
cg09716807	0.55058	0.388337838	-0.50364	0.000163	hypomethylated
cg26089541	0.5551	0.3915	-0.503735	3.67E-05	hypomethylated
cg10853949	0.78496	0.5536	-0.503775	6.05E-06	hypomethylated
cg16897462	0.50407	0.355492432	-0.503805	0.004526	hypomethylated
cg05437648	0.59059	0.416438378	-0.504054	3.62E-05	hypomethylated
cg20414364	0.54113	0.381559459	-0.504067	0.002409	hypomethylated
cg14174221	0.47172	0.332607568	-0.50411	0.000174	hypomethylated
cg06173216	0.83638	0.589723784	-0.504119	1.28E-05	hypomethylated
cg06176471	0.62606	0.441410811	-0.504179	2.35E-05	hypomethylated
cg24756403	0.89338	0.629874054	-0.504211	4.24E-06	hypomethylated
cg14055655	0.79509	0.560565946	-0.504234	1.94E-05	hypomethylated
cg02149376	0.74866	0.527808649	-0.504296	1.31E-05	hypomethylated
cg04804772	0.73378	0.517265405	-0.504443	2.56E-06	hypomethylated
cg11549874	0.77217	0.544307027	-0.504498	7.72E-06	hypomethylated
cg22800830	0.64268	0.453025946	-0.504507	0.000219	hypomethylated
cg14323984	0.8884	0.626227027	-0.504524	1.33E-05	hypomethylated
cg03171478	0.58267	0.410701081	-0.50459	0.001	hypomethylated
cg17023034	0.66712	0.470224324	-0.504597	0.000506	hypomethylated
cg19878076	0.70763	0.498726486	-0.504746	1.28E-05	hypomethylated
cg02452732	0.55587	0.391751892	-0.504807	0.000365	hypomethylated
cg19161424	0.44863	0.316123243	-0.505039	8.73E-05	hypomethylated
cg02427576	0.79889	0.562899459	-0.50512	0.000103	hypomethylated
cg08864375	0.72199	0.508714054	-0.505124	0.000893	hypomethylated
cg04080022	0.51191	0.360682162	-0.505162	0.000122	hypomethylated
cg07455815	0.56175	0.395793514	-0.50518	7.29E-05	hypomethylated
cg03458316	0.83176	0.586014054	-0.505232	6.48E-06	hypomethylated
cg26514492	0.47584	0.335240541	-0.50528	0.000575	hypomethylated
cg20300541	0.71083	0.500751892	-0.505409	1.46E-05	hypomethylated
cg25085755	0.50599	0.356444865	-0.50543	1.30E-05	hypomethylated
cg02931464	0.4344	0.306011351	-0.505439	0.00048	hypomethylated
cg13772742	0.51613	0.363584865	-0.505442	3.23E-05	hypomethylated
cg14702787	0.81669	0.575304865	-0.505462	1.50E-05	hypomethylated
cg04055345	0.65137	0.458841081	-0.505483	0.000563	hypomethylated
cg06345909	0.56179	0.395728649	-0.505519	0.000764	hypomethylated
cg00883689	0.80228	0.565077838	-0.505656	6.31E-05	hypomethylated
cg21078330	0.6798	0.478761622	-0.505803	0.000159	hypomethylated
cg17220545	0.7702	0.542409189	-0.505852	6.31E-05	hypomethylated
cg25424194	0.60038	0.422786486	-0.505947	3.00E-05	hypomethylated
cg10833393	0.46916	0.330374595	-0.505977	8.52E-05	hypomethylated
cg12232118	0.69736	0.491067568	-0.505982	7.63E-07	hypomethylated
cg14574951	0.77463	0.545366486	-0.506281	2.71E-05	hypomethylated
cg14228177	0.91835	0.646545946	-0.506291	2.16E-06	hypomethylated
cg06623778	0.4791	0.337291351	-0.506331	0.002363	hypomethylated
cg08535779	0.67141	0.472678919	-0.506334	2.56E-06	hypomethylated
cg20389678	0.4624	0.325492432	-0.506517	6.15E-05	hypomethylated
cg02637031	0.54407	0.382969189	-0.506564	0.000382	hypomethylated
cg19389370	0.76239	0.536618919	-0.506631	0.000147	hypomethylated
cg18271897	0.62445	0.439522162	-0.50665	0.0003	hypomethylated
cg09387382	0.39111	0.275267568	-0.50674	0.000313	hypomethylated
cg05103064	0.74606	0.525081081	-0.506751	3.04E-05	hypomethylated
cg03344767	0.61649	0.433878919	-0.506785	0.000626	hypomethylated
cg03292675	0.64506	0.453979459	-0.506806	0.002751	hypomethylated
cg07665060	0.41813	0.294215135	-0.50708	0.000174	hypomethylated
cg23218363	0.8311	0.584776216	-0.507137	2.78E-05	hypomethylated
cg06735243	0.68495	0.481942162	-0.507139	3.59E-06	hypomethylated
cg00387357	0.82866	0.582998378	-0.507288	2.49E-06	hypomethylated
cg26330809	0.71019	0.49959027	-0.50746	1.80E-05	hypomethylated

cg11808677	0.69166	0.486539459	-0.507506	4.10E-05	hypomethylated
cg02004632	0.45776	0.321978919	-0.507625	0.000119	hypomethylated
cg23547157	0.54836	0.385681622	-0.507713	7.84E-05	hypomethylated
cg20992002	0.70415	0.495253514	-0.507716	0.000195	hypomethylated
cg21808406	0.45631	0.320931351	-0.50775	0.00054	hypomethylated
cg04165508	0.85069	0.59820973	-0.507982	2.04E-05	hypomethylated
cg22380178	0.55198	0.388135135	-0.508057	1.80E-05	hypomethylated
cg02023138	0.88501	0.622285405	-0.508117	8.83E-06	hypomethylated
cg00708486	0.78254	0.550187027	-0.508242	6.30E-06	hypomethylated
cg24156261	0.6503	0.457187568	-0.508319	0.000251	hypomethylated
cg08817867	0.62216	0.437398378	-0.508338	0.000178	hypomethylated
cg12888521	0.90171	0.633918378	-0.508366	1.72E-06	hypomethylated
cg09362047	0.84471	0.593833514	-0.508398	2.39E-05	hypomethylated
cg03875678	0.42853	0.30124973	-0.508436	0.000335	hypomethylated
cg20550154	0.63478	0.446238378	-0.508442	0.00099	hypomethylated
cg23217940	0.83913	0.589817838	-0.508625	3.90E-06	hypomethylated
cg02697427	0.78239	0.549921622	-0.508662	7.74E-05	hypomethylated
cg22779330	0.73682	0.517890811	-0.508664	4.12E-06	hypomethylated
cg24710503	0.78677	0.552983784	-0.508705	2.04E-05	hypomethylated
cg04772575	0.65484	0.460254595	-0.50871	0.000147	hypomethylated
cg09043214	0.80392	0.565026486	-0.508733	0.000789	hypomethylated
cg07826255	0.43183	0.303497838	-0.508777	0.000129	hypomethylated
cg05246522	0.6928	0.486805405	-0.509094	7.29E-05	hypomethylated
cg16378015	0.58015	0.407638919	-0.509134	3.32E-05	hypomethylated
cg13423554	0.66744	0.468966486	-0.509153	0.000667	hypomethylated
cg07102937	0.70476	0.495147027	-0.509275	1.43E-06	hypomethylated
cg13885205	0.66811	0.469350811	-0.509419	1.71E-05	hypomethylated
cg11819702	0.86629	0.608545405	-0.509485	1.04E-05	hypomethylated
cg06609496	0.76709	0.538848649	-0.509516	1.75E-05	hypomethylated
cg03074188	0.70356	0.494143784	-0.509743	0.002649	hypomethylated
cg22512768	0.8369	0.587780541	-0.509778	1.54E-06	hypomethylated
cg18026955	0.3933	0.276221622	-0.509804	0.000365	hypomethylated
cg09283548	0.64646	0.454011351	-0.509833	0.000884	hypomethylated
cg15152595	0.7635	0.536197297	-0.509864	3.69E-06	hypomethylated
cg18951352	0.61641	0.432895676	-0.509871	0.001603	hypomethylated
cg11821439	0.59309	0.416482703	-0.509994	6.62E-05	hypomethylated
cg27514336	0.87302	0.612989189	-0.510153	1.39E-05	hypomethylated
cg05898618	0.91181	0.640210811	-0.510186	2.42E-05	hypomethylated
cg23404711	0.69473	0.487777297	-0.51023	0.000104	hypomethylated
cg06582411	0.66417	0.46631027	-0.510262	1.39E-05	hypomethylated
cg07777652	0.70722	0.496531892	-0.510273	0.001096	hypomethylated
cg00334976	0.69664	0.489087568	-0.510321	2.79E-06	hypomethylated
cg21332304	0.82181	0.576963243	-0.510325	3.32E-05	hypomethylated
cg02999385	0.70354	0.493927568	-0.510333	2.15E-05	hypomethylated
cg13997068	0.57362	0.40271027	-0.510353	5.73E-06	hypomethylated
cg00290023	0.45303	0.318032432	-0.510433	0.000667	hypomethylated
cg17549878	0.60876	0.427353514	-0.510444	0.00014	hypomethylated
cg15745401	0.79701	0.559478919	-0.510514	2.64E-05	hypomethylated
cg17142931	0.63401	0.445050811	-0.510536	1.12E-05	hypomethylated
cg17193961	0.78705	0.552474595	-0.510547	0.000109	hypomethylated
cg01303236	0.66376	0.46593027	-0.510548	7.84E-05	hypomethylated
cg10097651	0.65701	0.461191892	-0.510548	0.000346	hypomethylated
cg00591333	0.6495	0.455889189	-0.510646	4.05E-05	hypomethylated
cg17168630	0.81862	0.574521622	-0.510833	4.64E-05	hypomethylated
cg21058973	0.94467	0.662945405	-0.51092	4.73E-06	hypomethylated
cg23461926	0.68578	0.481252432	-0.510952	4.48E-06	hypomethylated
cg08170519	0.59401	0.416820541	-0.511061	7.83E-06	hypomethylated
cg21488289	0.60305	0.423160541	-0.511073	2.45E-05	hypomethylated

cg16286735	0.69471	0.487475135	-0.511082	0.000148	hypomethylated
cg10606923	0.49737	0.348954595	-0.51128	2.04E-05	hypomethylated
cg10622174	0.5532	0.388112432	-0.511327	0.0003	hypomethylated
cg10130564	0.68848	0.483021081	-0.511329	0.00054	hypomethylated
cg21210630	0.7521	0.527541081	-0.511641	2.46E-06	hypomethylated
cg09287328	0.69632	0.488412432	-0.511651	0.000365	hypomethylated
cg08966188	0.81093	0.568765946	-0.511742	9.31E-06	hypomethylated
cg15581944	0.62707	0.439791351	-0.511807	0.000563	hypomethylated
cg06798115	0.51475	0.361003243	-0.51186	4.58E-05	hypomethylated
cg07278181	0.79899	0.560336216	-0.511885	2.16E-06	hypomethylated
cg23316449	0.50621	0.354995135	-0.511937	0.004326	hypomethylated
cg14818960	0.64898	0.455096216	-0.512002	0.00017	hypomethylated
cg05818394	0.54267	0.380542703	-0.512017	0.000403	hypomethylated
cg02907402	0.66039	0.463085405	-0.51204	0.000358	hypomethylated
cg09613192	0.63663	0.446415676	-0.512067	0.000756	hypomethylated
cg08675364	0.72534	0.508605946	-0.512109	8.60E-06	hypomethylated
cg04335293	0.39601	0.277677838	-0.512125	0.000126	hypomethylated
cg15824323	0.79854	0.559925405	-0.51213	5.58E-06	hypomethylated
cg11249486	0.62098	0.435395135	-0.512222	3.76E-05	hypomethylated
cg10028983	0.76104	0.533510811	-0.512455	9.38E-05	hypomethylated
cg13817802	0.71878	0.503874595	-0.512486	5.32E-05	hypomethylated
cg00377915	0.5959	0.417715135	-0.512551	5.86E-05	hypomethylated
cg12894711	0.57163	0.400697297	-0.512569	0.000204	hypomethylated
cg01629749	0.88527	0.620548108	-0.512574	3.04E-06	hypomethylated
cg22055891	0.89641	0.628322162	-0.512654	1.88E-06	hypomethylated
cg24069962	0.82741	0.57994	-0.512699	3.04E-06	hypomethylated
cg04722643	0.83348	0.584178378	-0.512739	1.54E-06	hypomethylated
cg19817165	0.59437	0.416568108	-0.512809	4.10E-05	hypomethylated
cg10299383	0.63028	0.441731892	-0.512822	6.01E-05	hypomethylated
cg25152942	0.89742	0.628919459	-0.512908	5.65E-06	hypomethylated
cg18879828	0.53881	0.377583784	-0.51298	1.14E-05	hypomethylated
cg08565003	0.86933	0.609185405	-0.513023	1.94E-05	hypomethylated
cg23290313	0.80421	0.563545405	-0.51304	7.12E-05	hypomethylated
cg23061795	0.73798	0.517106486	-0.51312	4.05E-05	hypomethylated
cg10857905	0.50444	0.353457297	-0.513147	6.15E-05	hypomethylated
cg21012061	0.63724	0.446495676	-0.513191	0.000378	hypomethylated
cg15695155	0.573	0.401474054	-0.513228	0.000224	hypomethylated
cg14131220	0.49405	0.346061081	-0.51363	0.005508	hypomethylated
cg21126943	0.37966	0.265929189	-0.513666	0.000528	hypomethylated
cg02392228	0.47329	0.331483243	-0.513788	0.000338	hypomethylated
cg02074956	0.69756	0.488528649	-0.513874	0.000135	hypomethylated
cg23536138	0.6591	0.461581081	-0.513913	5.32E-05	hypomethylated
cg20699549	0.53545	0.374984324	-0.513922	0.000839	hypomethylated
cg02499214	0.68402	0.479019459	-0.513954	0.001301	hypomethylated
cg20737812	0.66584	0.466271351	-0.514006	5.65E-06	hypomethylated
cg02304370	0.69228	0.484768108	-0.514061	0.000178	hypomethylated
cg01065161	0.95113	0.666004324	-0.514111	2.64E-06	hypomethylated
cg05678658	0.7181	0.50283027	-0.514113	7.41E-07	hypomethylated
cg10900049	0.91695	0.642040541	-0.514179	0.000327	hypomethylated
cg18958693	0.89999	0.630128649	-0.514263	1.01E-05	hypomethylated
cg16794579	0.63786	0.446588108	-0.514295	1.62E-05	hypomethylated
cg22758846	0.64149	0.449100541	-0.514388	0.000104	hypomethylated
cg23577033	0.82117	0.574880541	-0.514419	5.28E-06	hypomethylated
cg25198007	0.34196	0.239392973	-0.514447	0.003136	hypomethylated
cg16258854	0.69501	0.486541622	-0.514471	4.26E-05	hypomethylated
cg06710328	0.61102	0.427743784	-0.514473	0.000575	hypomethylated
cg16167060	0.68431	0.47905027	-0.514473	0.000313	hypomethylated
cg14244402	0.78034	0.546263784	-0.514505	2.15E-05	hypomethylated

cg05548488	0.41036	0.287244324	-0.514612	0.002318	hypomethylated
cg03547062	0.84972	0.594785946	-0.514617	1.39E-05	hypomethylated
cg17069533	0.85364	0.597526486	-0.514625	2.95E-06	hypomethylated
cg03509949	0.6335	0.443425946	-0.514651	0.000511	hypomethylated
cg24289314	0.77021	0.539096757	-0.514708	2.57E-05	hypomethylated
cg10529845	0.592	0.414346486	-0.514759	1.38E-05	hypomethylated
cg22502856	0.80563	0.5638	-0.514934	4.15E-05	hypomethylated
cg03339817	0.81624	0.571178378	-0.515052	6.31E-05	hypomethylated
cg15994604	0.49431	0.34588	-0.515144	0.000399	hypomethylated
cg27121309	0.83236	0.582391351	-0.515219	6.31E-05	hypomethylated
cg13728308	0.71494	0.500184865	-0.515361	1.06E-05	hypomethylated
cg06230674	0.46559	0.325712432	-0.515461	0.000805	hypomethylated
cg15470102	0.69187	0.483990811	-0.515521	0.00039	hypomethylated
cg05350293	0.76149	0.532614595	-0.515733	1.30E-05	hypomethylated
cg00302979	0.85453	0.597664865	-0.515794	3.79E-06	hypomethylated
cg23282441	0.60381	0.422291892	-0.515854	0.000251	hypomethylated
cg11953749	0.42365	0.296269189	-0.515964	0.004134	hypomethylated
cg21764708	0.79507	0.556002162	-0.515991	4.82E-05	hypomethylated
cg16530128	0.809	0.565714054	-0.516067	5.28E-06	hypomethylated
cg23823000	0.57907	0.404924865	-0.516084	0.000741	hypomethylated
cg04751761	0.60147	0.420531351	-0.516279	0.001031	hypomethylated
cg26118131	0.68941	0.481967027	-0.516428	8.03E-05	hypomethylated
cg23093090	0.7655	0.535135135	-0.516499	2.54E-05	hypomethylated
cg18997901	0.79998	0.559229189	-0.516524	3.85E-06	hypomethylated
cg05626664	0.71012	0.496394595	-0.516575	0.000506	hypomethylated
cg13235717	0.71743	0.501466486	-0.516685	7.93E-06	hypomethylated
cg11326510	0.93372	0.652643243	-0.516695	1.12E-06	hypomethylated
cg25178683	0.45426	0.317505946	-0.516735	9.16E-05	hypomethylated
cg23750514	0.70655	0.493812432	-0.516828	4.58E-05	hypomethylated
cg07441152	0.72305	0.505341081	-0.516838	5.58E-05	hypomethylated
cg15594444	0.86688	0.605857297	-0.516854	1.71E-05	hypomethylated
cg12182124	0.62746	0.438491892	-0.516973	0.000426	hypomethylated
cg25951288	0.56401	0.394137838	-0.51702	0.000445	hypomethylated
cg00440980	0.79472	0.555342703	-0.517068	1.12E-05	hypomethylated
cg05865746	0.50978	0.356226486	-0.51708	0.004776	hypomethylated
cg12027068	0.51173	0.35754	-0.517278	4.82E-05	hypomethylated
cg24302752	0.63397	0.442947568	-0.517279	0.000789	hypomethylated
cg08930413	0.7472	0.522027027	-0.51737	1.46E-05	hypomethylated
cg24159636	0.54708	0.382213514	-0.517373	5.43E-06	hypomethylated
cg07041428	0.7301	0.510051351	-0.517452	0.000129	hypomethylated
cg26844633	0.79372	0.554467027	-0.517528	2.32E-05	hypomethylated
cg09852221	0.43535	0.30412	-0.517535	0.001423	hypomethylated
cg00249584	0.66389	0.463760541	-0.517564	5.12E-05	hypomethylated
cg05982460	0.93563	0.653558919	-0.517621	4.58E-05	hypomethylated
cg07127410	0.65032	0.45424973	-0.517664	0.000148	hypomethylated
cg26392737	0.67974	0.474798378	-0.517668	0.000104	hypomethylated
cg19324997	0.77221	0.539384865	-0.517678	2.64E-05	hypomethylated
cg02847588	0.67157	0.469035135	-0.517842	0.000126	hypomethylated
cg02038492	0.81512	0.569263784	-0.517915	2.16E-06	hypomethylated
cg05701418	0.51869	0.362229189	-0.51797	0.0002	hypomethylated
cg09476006	0.87225	0.60912973	-0.517992	3.39E-06	hypomethylated
cg11267802	0.66134	0.461842162	-0.517992	2.38E-05	hypomethylated
cg21769093	0.77505	0.541221622	-0.51807	0.000132	hypomethylated
cg10796749	0.66896	0.467120541	-0.518125	0.001571	hypomethylated
cg10336193	0.51709	0.361065946	-0.518153	0.00064	hypomethylated
cg09338148	0.40504	0.282818919	-0.518186	9.72E-05	hypomethylated
cg21275690	0.42129	0.294143243	-0.518295	9.72E-05	hypomethylated
cg15913671	0.65072	0.454314595	-0.518345	3.17E-06	hypomethylated

cg06367321	0.87603	0.611616216	-0.518354	1.15E-05	hypomethylated
cg10991475	0.58251	0.406663243	-0.518448	0.000187	hypomethylated
cg07858848	0.7917	0.552605405	-0.518704	5.72E-05	hypomethylated
cg07118000	0.42174	0.294368649	-0.51873	0.005085	hypomethylated
cg22603450	0.4812	0.33586973	-0.518735	8.42E-05	hypomethylated
cg00736299	0.73658	0.514114595	-0.518752	0.000112	hypomethylated
cg04332442	0.57572	0.401824324	-0.518802	0.001667	hypomethylated
cg11202887	0.76989	0.53734	-0.518817	1.28E-05	hypomethylated
cg18706476	0.95686	0.667828108	-0.518831	2.10E-06	hypomethylated
cg03006527	0.46985	0.327900541	-0.518942	7.12E-05	hypomethylated
cg12616177	0.69362	0.48404	-0.519019	1.75E-05	hypomethylated
cg17316718	0.69221	0.483055676	-0.51902	0.000185	hypomethylated
cg07591515	0.79718	0.556302162	-0.519037	9.57E-06	hypomethylated
cg06601581	0.64575	0.45059027	-0.51916	0.00017	hypomethylated
cg12886406	0.75696	0.528163784	-0.519232	1.52E-05	hypomethylated
cg12123019	0.57723	0.402734595	-0.519317	0.001949	hypomethylated
cg03928887	0.7506	0.523681081	-0.519356	3.04E-05	hypomethylated
cg10547761	0.66587	0.464541622	-0.519433	3.15E-05	hypomethylated
cg21097788	0.65758	0.458751892	-0.519452	0.000182	hypomethylated
cg18241555	0.48994	0.341796216	-0.519469	0.000696	hypomethylated
cg06602545	0.5807	0.405103784	-0.519501	3.36E-05	hypomethylated
cg07807470	0.59834	0.417393514	-0.519557	0.000588	hypomethylated
cg07992484	0.47511	0.331427568	-0.519568	4.53E-05	hypomethylated
cg18127823	0.83995	0.585929189	-0.519577	1.54E-05	hypomethylated
cg19756068	0.77226	0.538695135	-0.519618	3.39E-06	hypomethylated
cg06837799	0.56931	0.397114054	-0.519661	0.000528	hypomethylated
cg10322876	0.80878	0.564133514	-0.519711	1.72E-06	hypomethylated
cg20539142	0.66971	0.467079459	-0.519869	0.00017	hypomethylated
cg02666257	0.44798	0.312436757	-0.51987	0.001107	hypomethylated
cg27391982	0.66024	0.460439459	-0.519979	0.000293	hypomethylated
cg20333292	0.61804	0.43100973	-0.51998	0.000435	hypomethylated
cg27658026	0.59804	0.417053514	-0.520009	7.56E-05	hypomethylated
cg14832378	0.69381	0.483832973	-0.520032	2.07E-06	hypomethylated
cg12640305	0.52771	0.367955135	-0.520215	4.05E-05	hypomethylated
cg05001389	0.47964	0.334411351	-0.520328	0.001786	hypomethylated
cg27409991	0.75753	0.528151351	-0.520352	3.69E-06	hypomethylated
cg02999711	0.87313	0.608742162	-0.520365	1.58E-05	hypomethylated
cg17184165	0.65882	0.459311892	-0.52041	2.42E-06	hypomethylated
cg14819242	0.88237	0.615156216	-0.520431	3.04E-06	hypomethylated
cg19997384	0.34816	0.242714054	-0.520493	8.73E-05	hypomethylated
cg14147151	0.47916	0.333975135	-0.520767	0.002026	hypomethylated
cg14453145	0.60328	0.420482703	-0.520781	0.000358	hypomethylated
cg20074340	0.70998	0.494848649	-0.520791	4.48E-06	hypomethylated
cg27239243	0.6299	0.438994054	-0.520921	0.000219	hypomethylated
cg08565197	0.56054	0.390654054	-0.520926	6.31E-05	hypomethylated
cg19607021	0.568	0.395849189	-0.52094	1.80E-05	hypomethylated
cg14285788	0.62522	0.435721081	-0.520959	0.000581	hypomethylated
cg18928221	0.63329	0.441344324	-0.520962	3.40E-05	hypomethylated
cg04717699	0.61244	0.426780541	-0.521074	0.000454	hypomethylated
cg10290728	0.63415	0.441905946	-0.521085	1.75E-05	hypomethylated
cg16426215	0.57223	0.398754595	-0.521094	1.15E-05	hypomethylated
cg27292099	0.68914	0.480222162	-0.521095	3.74E-06	hypomethylated
cg12821702	0.6702	0.466948649	-0.521328	5.19E-05	hypomethylated
cg09903452	0.48348	0.336789189	-0.52161	3.81E-05	hypomethylated
cg02685680	0.81033	0.564402703	-0.521785	8.22E-07	hypomethylated
cg20528787	0.56168	0.391191892	-0.521872	0.000234	hypomethylated
cg15599832	0.81454	0.567278378	-0.521929	6.57E-06	hypomethylated
cg17888259	0.70106	0.488245405	-0.521931	2.32E-05	hypomethylated

cg18787420	0.53884	0.375244324	-0.522027	7.56E-05	hypomethylated
cg06911744	0.94735	0.659717838	-0.522048	2.71E-06	hypomethylated
cg12395299	0.56908	0.396275676	-0.522127	1.04E-05	hypomethylated
cg23291048	0.66554	0.463441622	-0.522138	0.000262	hypomethylated
cg19396666	0.56552	0.393772432	-0.522216	1.25E-05	hypomethylated
cg23620049	0.66508	0.463083243	-0.522256	5.72E-05	hypomethylated
cg04205616	0.88179	0.613897838	-0.522437	1.28E-05	hypomethylated
cg10274029	0.68259	0.47518973	-0.522516	0.000588	hypomethylated
cg13725826	0.77371	0.538616216	-0.522535	6.78E-05	hypomethylated
cg11021222	0.65902	0.458771892	-0.522545	5.32E-05	hypomethylated
cg05754905	0.92483	0.643805405	-0.522564	8.34E-07	hypomethylated
cg21002223	0.72304	0.503325405	-0.522584	1.18E-05	hypomethylated
cg06818159	0.63729	0.443583784	-0.522743	9.49E-05	hypomethylated
cg13512948	0.67868	0.47236	-0.522845	1.58E-05	hypomethylated
cg24052475	0.59585	0.414708649	-0.522851	1.66E-05	hypomethylated
cg26292918	0.71607	0.498335676	-0.522983	7.52E-06	hypomethylated
cg01310482	0.81104	0.564398378	-0.523059	9.82E-06	hypomethylated
cg16854533	0.75651	0.526444865	-0.523077	1.58E-05	hypomethylated
cg26078976	0.72355	0.503496216	-0.523112	1.44E-05	hypomethylated
cg20051772	0.39473	0.274671892	-0.523157	0.000331	hypomethylated
cg11839020	0.65708	0.457225405	-0.523163	0.000382	hypomethylated
cg08579420	0.76279	0.530771892	-0.523194	0.000119	hypomethylated
cg17669033	0.68648	0.477614595	-0.523371	1.04E-05	hypomethylated
cg24063912	0.61625	0.428750811	-0.523376	2.21E-05	hypomethylated
cg22219089	0.59228	0.412071351	-0.523385	0.000563	hypomethylated
cg14361132	0.64852	0.451197297	-0.523393	6.78E-05	hypomethylated
cg03611265	0.73502	0.511333514	-0.523519	0.000229	hypomethylated
cg02744705	0.76754	0.533947568	-0.523544	1.87E-05	hypomethylated
cg23519637	0.7024	0.488618378	-0.523585	0.001452	hypomethylated
cg20623503	0.85277	0.593195135	-0.52365	2.95E-06	hypomethylated
cg17474524	0.48924	0.340299459	-0.523738	0.001395	hypomethylated
cg05985821	0.59212	0.411855676	-0.523751	0.00395	hypomethylated
cg12720921	0.626	0.435419459	-0.523757	0.001511	hypomethylated
cg14921326	0.61582	0.428315135	-0.523836	6.23E-05	hypomethylated
cg19766988	0.83701	0.582067027	-0.52406	8.60E-06	hypomethylated
cg08639339	0.55509	0.386004324	-0.524105	0.000135	hypomethylated
cg00618155	0.89473	0.622183243	-0.524113	1.80E-06	hypomethylated
cg19035908	0.71615	0.497988108	-0.524151	2.27E-05	hypomethylated
cg23299254	0.88712	0.616864865	-0.524175	1.09E-05	hypomethylated
cg23401796	0.89616	0.623120541	-0.524245	6.31E-05	hypomethylated
cg15487251	0.63145	0.439053514	-0.524272	0.000399	hypomethylated
cg21447717	0.61187	0.425401081	-0.524401	1.46E-05	hypomethylated
cg14080050	0.63297	0.440051892	-0.524463	0.001452	hypomethylated
cg01200021	0.73301	0.509582703	-0.524517	4.26E-05	hypomethylated
cg05357209	0.76296	0.530379459	-0.524583	1.09E-05	hypomethylated
cg17297634	0.64834	0.450662162	-0.524704	0.001368	hypomethylated
cg26247168	0.90631	0.629971892	-0.524717	1.04E-05	hypomethylated
cg25318189	0.47031	0.326891892	-0.524798	7.12E-05	hypomethylated
cg10124440	0.53183	0.369641081	-0.52484	1.62E-05	hypomethylated
cg27235315	0.89793	0.624062162	-0.524913	3.17E-06	hypomethylated
cg01767053	0.9237	0.641918378	-0.525035	2.29E-06	hypomethylated
cg03495053	0.74497	0.517662162	-0.525171	1.62E-05	hypomethylated
cg12907876	0.63503	0.441224324	-0.525312	7.72E-06	hypomethylated
cg23721083	0.43973	0.305503784	-0.525428	0.003705	hypomethylated
cg01359822	0.75676	0.525748108	-0.525464	8.63E-05	hypomethylated
cg12050175	0.49492	0.343835135	-0.525478	5.45E-05	hypomethylated
cg09346775	0.47598	0.330667027	-0.525522	1.28E-05	hypomethylated
cg27466235	0.73163	0.508263784	-0.525537	6.95E-05	hypomethylated

cg04892766	0.62642	0.43517027	-0.52555	6.05E-06	hypomethylated
cg15160746	0.90882	0.631345946	-0.525564	3.15E-05	hypomethylated
cg02153681	0.82042	0.569928649	-0.525581	0.000193	hypomethylated
cg21197219	0.39636	0.275336216	-0.525617	0.002274	hypomethylated
cg16236228	0.65463	0.454741622	-0.525633	1.94E-05	hypomethylated
cg16822095	0.74257	0.515728108	-0.525916	4.86E-06	hypomethylated
cg05371506	0.65937	0.457922703	-0.525984	0.000756	hypomethylated
cg09621603	0.88719	0.616126486	-0.526017	1.58E-06	hypomethylated
cg04697265	0.4582	0.318200541	-0.526041	0.000327	hypomethylated
cg18919720	0.59301	0.411816216	-0.526056	6.31E-05	hypomethylated
cg13314965	0.74625	0.518229189	-0.526069	4.26E-05	hypomethylated
cg27019645	0.87319	0.606375676	-0.526084	7.32E-06	hypomethylated
cg02641941	0.80543	0.559316216	-0.526095	5.65E-06	hypomethylated
cg21791017	0.42461	0.294858378	-0.526116	0.000148	hypomethylated
cg21095280	0.66846	0.464172973	-0.526179	0.000107	hypomethylated
cg22143064	0.57156	0.396883784	-0.526188	8.83E-06	hypomethylated
cg02675173	0.79689	0.553258378	-0.526427	2.48E-05	hypomethylated
cg11084474	0.601	0.41724973	-0.526454	1.08E-05	hypomethylated
cg19738281	0.60304	0.418664865	-0.526458	0.000474	hypomethylated
cg25036779	0.38881	0.269931892	-0.52647	0.003107	hypomethylated
cg27215916	0.79399	0.551219459	-0.526494	7.32E-06	hypomethylated
cg11978890	0.60931	0.422982703	-0.526578	1.25E-05	hypomethylated
cg16681421	0.69936	0.485476757	-0.526633	8.37E-06	hypomethylated
cg07005353	0.86611	0.601159459	-0.526803	1.22E-06	hypomethylated
cg06042148	0.66921	0.464467568	-0.526881	0.000653	hypomethylated
cg09784932	0.84169	0.584169189	-0.526903	2.49E-06	hypomethylated
cg00406211	0.7165	0.49726973	-0.526938	5.72E-05	hypomethylated
cg25087341	0.71631	0.497116216	-0.527001	8.22E-05	hypomethylated
cg03548857	0.57495	0.39900973	-0.527013	0.000313	hypomethylated
cg07779313	0.57716	0.400523243	-0.527085	0.000123	hypomethylated
cg13499318	0.60475	0.419649189	-0.527155	0.000102	hypomethylated
cg03340408	0.53732	0.372848108	-0.527194	5.86E-05	hypomethylated
cg03752977	0.65467	0.45426	-0.52725	2.02E-05	hypomethylated
cg01239651	0.49996	0.346882162	-0.527367	0.000741	hypomethylated
cg12413156	0.69207	0.480149189	-0.527435	0.000212	hypomethylated
cg14294321	0.62459	0.433306486	-0.527522	1.31E-05	hypomethylated
cg01932361	0.78544	0.54486973	-0.52759	5.32E-05	hypomethylated
cg13820205	0.66903	0.46410973	-0.527605	1.87E-05	hypomethylated
cg20315150	0.61122	0.424002162	-0.52762	0.000115	hypomethylated
cg11150074	0.76571	0.531155676	-0.527663	3.71E-05	hypomethylated
cg22050157	0.54476	0.37786973	-0.527732	1.99E-05	hypomethylated
cg26588076	0.61895	0.429282162	-0.527897	0.00095	hypomethylated
cg04447128	0.55299	0.383513514	-0.527976	2.38E-05	hypomethylated
cg15803845	0.57807	0.400872973	-0.528099	0.000107	hypomethylated
cg09528494	0.63333	0.439162703	-0.528202	1.12E-05	hypomethylated
cg21658968	0.71923	0.498687568	-0.528317	7.29E-05	hypomethylated
cg26336935	0.48945	0.339359459	-0.528347	0.000219	hypomethylated
cg17668756	0.64182	0.445003784	-0.528351	8.60E-06	hypomethylated
cg04767756	0.68574	0.475431351	-0.528425	7.47E-05	hypomethylated
cg15350661	0.54755	0.379622162	-0.528426	4.64E-05	hypomethylated
cg08701429	0.77322	0.536014595	-0.528607	3.85E-05	hypomethylated
cg10764891	0.77104	0.534496757	-0.528625	5.06E-05	hypomethylated
cg06991495	0.34301	0.237774595	-0.528656	0.001118	hypomethylated
cg13071185	0.65667	0.455197297	-0.528677	0.000374	hypomethylated
cg26758396	0.62611	0.43401027	-0.528687	5.06E-05	hypomethylated
cg13219080	0.72126	0.499945946	-0.528747	1.15E-05	hypomethylated
cg26874999	0.84435	0.585262162	-0.528758	8.83E-06	hypomethylated
cg21293464	0.5033	0.348852432	-0.528802	0.007933	hypomethylated

cg04301614	0.63273	0.438563784	-0.528803	0.00028	hypomethylated
cg25522867	0.55485	0.384557838	-0.528897	9.05E-05	hypomethylated
cg05155047	0.57784	0.400485946	-0.528918	0.000104	hypomethylated
cg02998240	0.55455	0.384336757	-0.528947	9.38E-05	hypomethylated
cg21890646	0.74627	0.517199459	-0.528977	0.00054	hypomethylated
cg01930756	0.58544	0.405714595	-0.529056	6.54E-05	hypomethylated
cg09462806	0.53162	0.368401622	-0.529116	0.00099	hypomethylated
cg26215727	0.47039	0.325962703	-0.52915	0.001237	hypomethylated
cg13313836	0.71543	0.495738919	-0.52923	0.000893	hypomethylated
cg06932535	0.73165	0.506973514	-0.529243	0.000563	hypomethylated
cg08179817	0.51178	0.354601622	-0.529325	0.000195	hypomethylated
cg12274898	0.79088	0.547977297	-0.529343	3.21E-06	hypomethylated
cg02096790	0.8063	0.558636757	-0.529406	5.14E-06	hypomethylated
cg02550119	0.67674	0.468862162	-0.529438	4.70E-05	hypomethylated
cg11203211	0.83793	0.580444865	-0.529671	2.05E-06	hypomethylated
cg05713242	0.54139	0.375023784	-0.529686	0.009238	hypomethylated
cg07002540	0.83667	0.579560541	-0.529699	4.10E-05	hypomethylated
cg18894552	0.75665	0.524107027	-0.529765	6.75E-06	hypomethylated
cg08772789	0.5934	0.411014595	-0.529815	0.0006	hypomethylated
cg23536675	0.88245	0.611132432	-0.530029	8.15E-06	hypomethylated
cg09864345	0.82395	0.5706	-0.530077	1.66E-05	hypomethylated
cg23976388	0.66401	0.45983027	-0.530104	0.000191	hypomethylated
cg12065616	0.53822	0.372704324	-0.530164	9.83E-05	hypomethylated
cg21376090	0.73037	0.505747568	-0.53021	0.00017	hypomethylated
cg25376491	0.62439	0.432342703	-0.530272	0.000143	hypomethylated
cg04593696	0.52724	0.365025405	-0.530463	0.000229	hypomethylated
cg24310786	0.41328	0.286125946	-0.530469	0.000839	hypomethylated
cg14055004	0.60523	0.419018378	-0.53047	5.19E-05	hypomethylated
cg08831077	0.6899	0.477618919	-0.530527	0.000165	hypomethylated
cg00357958	0.43951	0.30427027	-0.530543	0.00098	hypomethylated
cg01458495	0.80993	0.560687568	-0.5306	1.58E-06	hypomethylated
cg03708045	0.74897	0.518482703	-0.530612	2.89E-05	hypomethylated
cg11346718	0.57519	0.398177297	-0.530628	7.72E-06	hypomethylated
cg27338302	0.69885	0.483754595	-0.530707	9.69E-06	hypomethylated
cg11136562	0.62689	0.43394	-0.530717	2.15E-05	hypomethylated
cg14076239	0.67934	0.470240541	-0.530735	0.000474	hypomethylated
cg21839856	0.69967	0.484308649	-0.530748	2.21E-05	hypomethylated
cg10960266	0.78962	0.546559459	-0.53078	1.66E-05	hypomethylated
cg12177087	0.88798	0.614588649	-0.530906	8.15E-06	hypomethylated
cg23752828	0.63292	0.438038378	-0.530966	0.001395	hypomethylated
cg03112033	0.7715	0.533948108	-0.530967	1.64E-05	hypomethylated
cg13178170	0.78051	0.540183784	-0.530967	1.84E-05	hypomethylated
cg00328227	0.59213	0.409807027	-0.530969	0.000214	hypomethylated
cg21293455	0.81469	0.563831351	-0.530987	1.80E-05	hypomethylated
cg03079132	0.55164	0.381769189	-0.531026	2.35E-05	hypomethylated
cg00089814	0.68985	0.47741027	-0.531053	4.47E-05	hypomethylated
cg25839663	0.73682	0.509907027	-0.531078	4.42E-06	hypomethylated
cg08196512	0.7646	0.529122162	-0.531104	3.76E-05	hypomethylated
cg18541087	0.77071	0.533325405	-0.531172	1.04E-05	hypomethylated
cg04465154	0.49427	0.342016757	-0.531232	0.000495	hypomethylated
cg21560076	0.68879	0.476615135	-0.531239	0.000123	hypomethylated
cg07412254	0.66023	0.456839459	-0.531281	2.51E-05	hypomethylated
cg22481448	0.90801	0.628224865	-0.531427	6.15E-05	hypomethylated
cg08324925	0.71254	0.492955676	-0.531513	4.01E-06	hypomethylated
cg16861209	0.82515	0.570823784	-0.531611	4.24E-06	hypomethylated
cg03049863	0.45017	0.311332432	-0.532014	0.000135	hypomethylated
cg07925415	0.47614	0.329289189	-0.532031	0.000212	hypomethylated
cg11181587	0.72472	0.501200541	-0.532036	5.73E-06	hypomethylated

cg19704102	0.79953	0.552904865	-0.532121	1.35E-05	hypomethylated
cg25745642	0.56376	0.389818378	-0.532279	4.60E-06	hypomethylated
cg25957300	0.86478	0.597907568	-0.532411	1.84E-05	hypomethylated
cg12381074	0.8602	0.594728649	-0.532441	6.39E-06	hypomethylated
cg26644674	0.79165	0.547324324	-0.532467	3.07E-05	hypomethylated
cg07474269	0.83058	0.574229189	-0.532492	1.05E-06	hypomethylated
cg25556008	0.82015	0.566997838	-0.532545	9.57E-06	hypomethylated
cg23897302	0.69847	0.482855676	-0.532606	1.89E-05	hypomethylated
cg23740652	0.69854	0.482892432	-0.532641	0.000126	hypomethylated
cg11556164	0.83285	0.575726486	-0.532673	1.63E-06	hypomethylated
cg21794767	0.83171	0.574895676	-0.53278	8.15E-06	hypomethylated
cg09874659	0.89488	0.618525946	-0.53286	8.15E-06	hypomethylated
cg05035061	0.58951	0.407454595	-0.532877	3.32E-05	hypomethylated
cg03710889	0.60897	0.420901081	-0.53289	9.49E-05	hypomethylated
cg02049833	0.85199	0.588841081	-0.532958	2.32E-05	hypomethylated
cg04164584	0.78058	0.539468108	-0.533009	2.10E-06	hypomethylated
cg20788020	0.74597	0.515486486	-0.533183	4.94E-05	hypomethylated
cg00460916	0.74308	0.513489189	-0.533184	5.86E-05	hypomethylated
cg16960396	0.79246	0.547583784	-0.533258	4.60E-06	hypomethylated
cg20029881	0.59223	0.409208649	-0.533321	4.31E-05	hypomethylated
cg07010486	0.60411	0.417411892	-0.53334	1.01E-05	hypomethylated
cg14105536	0.81536	0.56335027	-0.533405	3.85E-06	hypomethylated
cg01604401	0.72272	0.499284324	-0.533575	8.83E-06	hypomethylated
cg03879160	0.8123	0.56116973	-0.533575	4.24E-06	hypomethylated
cg25254739	0.46076	0.318305405	-0.533604	3.57E-05	hypomethylated
cg13569051	0.55739	0.385042703	-0.533669	0.0006	hypomethylated
cg21723559	0.574	0.396512973	-0.533683	6.01E-05	hypomethylated
cg04147642	0.31015	0.214247568	-0.533687	0.002432	hypomethylated
cg22517356	0.53265	0.367940541	-0.533715	8.03E-05	hypomethylated
cg01609268	0.90001	0.621699459	-0.533724	7.32E-06	hypomethylated
cg13630878	0.85068	0.587621622	-0.533729	6.75E-06	hypomethylated
cg07052524	0.85527	0.590746486	-0.533841	0.000126	hypomethylated
cg09103187	0.90375	0.624223243	-0.533862	1.63E-06	hypomethylated
cg01854676	0.87134	0.601827568	-0.533886	1.04E-05	hypomethylated
cg19901403	0.57348	0.39606973	-0.533989	0.00013	hypomethylated
cg07473553	0.80068	0.552953514	-0.534068	8.84E-05	hypomethylated
cg05226729	0.80685	0.557187027	-0.534139	8.83E-06	hypomethylated
cg00117599	0.45383	0.313392432	-0.534182	3.32E-05	hypomethylated
cg17462560	0.61828	0.426944865	-0.534211	0.000306	hypomethylated
cg27588093	0.67983	0.469447027	-0.534212	6.30E-06	hypomethylated
cg01569083	0.55283	0.381738378	-0.534252	0.001041	hypomethylated
cg24631735	0.81691	0.564082703	-0.53427	3.69E-06	hypomethylated
cg25574175	0.79985	0.552286486	-0.534313	6.39E-06	hypomethylated
cg15962197	0.5959	0.411414595	-0.534477	8.94E-05	hypomethylated
cg16683508	0.84322	0.582074595	-0.534705	9.82E-06	hypomethylated
cg14900031	0.79903	0.551548108	-0.534763	5.28E-06	hypomethylated
cg07572930	0.71316	0.492219459	-0.534924	6.22E-06	hypomethylated
cg24730705	0.65617	0.452882703	-0.534932	1.04E-05	hypomethylated
cg25073708	0.61405	0.423803784	-0.53496	0.00024	hypomethylated
cg17030820	0.83136	0.573781622	-0.534972	3.59E-06	hypomethylated
cg23512558	0.45207	0.311979459	-0.535095	0.000426	hypomethylated
cg05802386	0.74113	0.511463243	-0.535096	7.62E-06	hypomethylated
cg14796755	0.78579	0.542261622	-0.535155	7.62E-06	hypomethylated
cg00665114	0.79932	0.551588108	-0.535182	3.67E-05	hypomethylated
cg01761729	0.59822	0.412812973	-0.535188	2.15E-05	hypomethylated
cg25054816	0.70442	0.486069189	-0.535274	3.39E-06	hypomethylated
cg15244778	0.73984	0.510475135	-0.535373	0.000191	hypomethylated
cg19676502	0.61623	0.425159459	-0.535465	0.004059	hypomethylated

cg27356115	0.71785	0.495259459	-0.535498	0.0003	hypomethylated
cg18186771	0.86428	0.596283243	-0.535501	3.12E-06	hypomethylated
cg02198988	0.62342	0.430091351	-0.535561	5.06E-05	hypomethylated
cg20721738	0.43281	0.298577297	-0.535629	0.0017	hypomethylated
cg27185793	0.5607	0.386778378	-0.535722	0.000161	hypomethylated
cg16960046	0.74067	0.510922162	-0.535727	2.99E-06	hypomethylated
cg02124184	0.64569	0.445378919	-0.535808	1.40E-05	hypomethylated
cg13587915	0.73772	0.508805946	-0.535958	6.15E-05	hypomethylated
cg13829849	0.81584	0.562668649	-0.536001	1.15E-05	hypomethylated
cg09651136	0.6077	0.41908973	-0.5361	4.26E-05	hypomethylated
cg26300517	0.72599	0.500627027	-0.536213	0.0006	hypomethylated
cg25649765	0.70067	0.483163784	-0.536223	7.65E-05	hypomethylated
cg24056365	0.64477	0.444605405	-0.536259	0.000159	hypomethylated
cg24520862	0.7109	0.4902	-0.536276	0.000123	hypomethylated
cg12537329	0.7679	0.529492973	-0.536307	2.18E-05	hypomethylated
cg19524238	0.75128	0.518025946	-0.536326	0.000123	hypomethylated
cg18763629	0.68863	0.474821622	-0.536343	2.64E-05	hypomethylated
cg27207756	0.68278	0.470775676	-0.536381	5.45E-05	hypomethylated
cg26232247	0.56581	0.390075135	-0.536566	0.005085	hypomethylated
cg25637972	0.77353	0.533240541	-0.536671	0.000129	hypomethylated
cg17179881	0.31041	0.213981081	-0.536692	3.49E-05	hypomethylated
cg13043815	0.76905	0.530139459	-0.536705	8.15E-06	hypomethylated
cg11895596	0.74755	0.515314595	-0.536717	2.21E-05	hypomethylated
cg08374494	0.61973	0.427166486	-0.536841	5.43E-06	hypomethylated
cg17934470	0.6206	0.427713514	-0.537019	0.000358	hypomethylated
cg00805193	0.57948	0.399341081	-0.537137	5.06E-05	hypomethylated
cg22046201	0.71957	0.49588	-0.537144	0.000726	hypomethylated
cg01182455	0.65072	0.448431351	-0.53715	0.001481	hypomethylated
cg05673892	0.84587	0.582914054	-0.537153	3.90E-06	hypomethylated
cg05871756	0.65743	0.45302973	-0.537232	0.000115	hypomethylated
cg16113793	0.51325	0.353665405	-0.537277	0.001667	hypomethylated
cg14736458	0.54992	0.378911351	-0.537361	0.000506	hypomethylated
cg24911891	0.8706	0.599828108	-0.537461	0.000129	hypomethylated
cg14327359	0.85363	0.588098378	-0.537553	1.56E-06	hypomethylated
cg03222834	0.66088	0.45529027	-0.537602	0.000117	hypomethylated
cg20602843	0.63828	0.439715135	-0.53762	8.32E-05	hypomethylated
cg24124145	0.41055	0.282826486	-0.537641	3.32E-05	hypomethylated
cg21454362	0.61459	0.423355135	-0.537756	8.84E-05	hypomethylated
cg11092836	0.66574	0.458520541	-0.537972	1.42E-05	hypomethylated
cg07632771	0.7555	0.520295676	-0.5381	4.05E-05	hypomethylated
cg06048910	0.90002	0.619756757	-0.538255	1.28E-05	hypomethylated
cg00832644	0.78113	0.537876216	-0.538288	1.99E-06	hypomethylated
cg07520919	0.62795	0.432363243	-0.538406	3.79E-06	hypomethylated
cg10979364	0.77683	0.534865946	-0.538422	4.64E-05	hypomethylated
cg24561305	0.50381	0.346876757	-0.538457	3.49E-05	hypomethylated
cg04737759	0.80373	0.553364324	-0.538481	1.80E-05	hypomethylated
cg18149745	0.73686	0.507321081	-0.538491	1.94E-05	hypomethylated
cg08795557	0.75223	0.517893514	-0.538518	7.52E-06	hypomethylated
cg09438113	0.49282	0.339269189	-0.53863	1.01E-05	hypomethylated
cg21008969	0.5235	0.360373514	-0.538697	0.000245	hypomethylated
cg23385208	0.58776	0.404598919	-0.538735	0.000104	hypomethylated
cg12147622	0.56636	0.389821081	-0.538907	0.00035	hypomethylated
cg03961030	0.87903	0.605021081	-0.538927	2.13E-06	hypomethylated
cg21817750	0.82499	0.567817297	-0.53895	3.30E-06	hypomethylated
cg11641006	0.50907	0.350369189	-0.538988	0.000178	hypomethylated
cg14082893	0.69656	0.479407568	-0.538995	0.000893	hypomethylated
cg04175872	0.51316	0.353176216	-0.539021	0.002803	hypomethylated
cg12186771	0.69155	0.475947027	-0.539033	0.001768	hypomethylated

cg07421969	0.71363	0.491121622	-0.539096	9.66E-07	hypomethylated
cg24599709	0.87899	0.604874054	-0.539212	6.22E-06	hypomethylated
cg08410533	0.51465	0.354145405	-0.53925	0.000306	hypomethylated
cg18332838	0.7495	0.515685946	-0.539436	0.000335	hypomethylated
cg14133064	0.44834	0.308458919	-0.539515	0.000741	hypomethylated
cg14935206	0.57825	0.397815676	-0.539593	0.004608	hypomethylated
cg06495763	0.75583	0.519945405	-0.539702	9.82E-06	hypomethylated
cg05302095	0.75692	0.520677838	-0.53975	6.39E-06	hypomethylated
cg17544584	0.77207	0.531016757	-0.539974	6.05E-06	hypomethylated
cg06473276	0.54931	0.377784865	-0.540056	0.001821	hypomethylated
cg04505972	0.78379	0.539009189	-0.540157	5.73E-06	hypomethylated
cg16576160	0.67337	0.463052432	-0.540224	4.36E-05	hypomethylated
cg00417147	0.78731	0.541254595	-0.540624	1.42E-05	hypomethylated
cg09966309	0.65587	0.450879459	-0.540668	0.0003	hypomethylated
cg26280911	0.52855	0.363318378	-0.540806	0.003078	hypomethylated
cg19554457	0.60969	0.419056216	-0.540932	0.000126	hypomethylated
cg26992245	0.67955	0.467038378	-0.541039	0.000109	hypomethylated
cg07679836	0.34639	0.238064324	-0.541046	0.000195	hypomethylated
cg04042248	0.4989	0.342874595	-0.54107	0.000107	hypomethylated
cg19683417	0.84456	0.580430811	-0.541076	3.59E-06	hypomethylated
cg19707677	0.84008	0.577319459	-0.541157	8.04E-06	hypomethylated
cg05573767	0.55403	0.380727027	-0.541207	0.001275	hypomethylated
cg08686879	0.76441	0.525275135	-0.541273	1.49E-06	hypomethylated
cg25612480	0.59778	0.410665946	-0.541649	8.15E-06	hypomethylated
cg08777211	0.83489	0.573548108	-0.541672	2.71E-06	hypomethylated
cg24681963	0.63749	0.437908108	-0.541775	3.23E-05	hypomethylated
cg22900681	0.4999	0.343378378	-0.54184	0.000342	hypomethylated
cg16909914	0.38545	0.264762162	-0.541847	0.000159	hypomethylated
cg09566023	0.7261	0.498734054	-0.541898	3.32E-05	hypomethylated
cg11861562	0.61635	0.423337297	-0.541942	0.00039	hypomethylated
cg10492657	0.74567	0.512146486	-0.541981	2.79E-06	hypomethylated
cg07734975	0.7958	0.54654	-0.542079	0.000517	hypomethylated
cg06966245	0.64335	0.441806486	-0.542189	6.15E-05	hypomethylated
cg24744557	0.4703	0.322953514	-0.542255	0.000224	hypomethylated
cg16152136	0.66617	0.457439459	-0.54231	0.00039	hypomethylated
cg25677394	0.70003	0.480681081	-0.542337	2.45E-05	hypomethylated
cg21211730	0.64986	0.446223243	-0.542363	3.07E-05	hypomethylated
cg03604774	0.75426	0.517867027	-0.54248	7.29E-05	hypomethylated
cg27139457	0.74574	0.512015135	-0.542486	9.31E-06	hypomethylated
cg14679668	0.80012	0.549338378	-0.542521	1.35E-05	hypomethylated
cg25843426	0.54713	0.375627027	-0.542583	3.85E-05	hypomethylated
cg07402003	0.86401	0.593167027	-0.54261	1.93E-06	hypomethylated
cg03203287	0.86137	0.591349189	-0.542623	9.57E-06	hypomethylated
cg02402368	0.60345	0.41424	-0.542767	0.000464	hypomethylated
cg03778909	0.81602	0.560157297	-0.542773	3.23E-05	hypomethylated
cg13002506	0.82259	0.564663243	-0.542783	4.70E-05	hypomethylated
cg24724917	0.79413	0.545104865	-0.542841	1.06E-05	hypomethylated
cg22229039	0.7049	0.483836757	-0.542898	0.00054	hypomethylated
cg03266686	0.50073	0.343682703	-0.542956	4.26E-05	hypomethylated
cg16949584	0.70658	0.484888649	-0.543199	8.59E-07	hypomethylated
cg24375627	0.51341	0.352321081	-0.543221	0.000161	hypomethylated
cg20124376	0.83927	0.575935676	-0.543227	6.39E-06	hypomethylated
cg14003231	0.45028	0.308983243	-0.543294	9.72E-05	hypomethylated
cg18891604	0.77317	0.53050973	-0.543406	1.58E-05	hypomethylated
cg13300246	0.55254	0.379086486	-0.543552	5.72E-05	hypomethylated
cg04646987	0.88168	0.604877838	-0.543611	0.000216	hypomethylated
cg25738786	0.56572	0.388096216	-0.543674	0.00095	hypomethylated
cg08514385	0.66776	0.458088649	-0.543703	0.000187	hypomethylated

cg24289912	0.59881	0.410757838	-0.54381	0.000797	hypomethylated
cg14240790	0.62357	0.427664865	-0.544071	0.000176	hypomethylated
cg19547192	0.85512	0.586416216	-0.544202	9.49E-05	hypomethylated
cg11146034	0.63436	0.435023784	-0.544208	0.000772	hypomethylated
cg09435411	0.81656	0.559955676	-0.544246	4.60E-06	hypomethylated
cg06123346	0.61735	0.423347027	-0.544248	1.39E-05	hypomethylated
cg03985360	0.64181	0.440091892	-0.544341	0.000667	hypomethylated
cg09715009	0.7154	0.490531351	-0.544405	3.19E-05	hypomethylated
cg20530056	0.52298	0.358572973	-0.544489	1.31E-05	hypomethylated
cg00491404	0.73782	0.505867568	-0.544509	1.26E-06	hypomethylated
cg09225287	0.74714	0.512238378	-0.544563	0.000726	hypomethylated
cg19729744	0.65852	0.451454595	-0.544646	9.27E-05	hypomethylated
cg19092396	0.44607	0.305784324	-0.544756	0.00024	hypomethylated
cg23083049	0.79989	0.548329189	-0.544759	2.87E-06	hypomethylated
cg20835708	0.38936	0.266897838	-0.544817	0.002274	hypomethylated
cg17215278	0.62047	0.425310811	-0.544844	0.000474	hypomethylated
cg06912282	0.67787	0.464649189	-0.544867	3.23E-05	hypomethylated
cg21640654	0.75896	0.520165946	-0.545052	3.59E-06	hypomethylated
cg14487665	0.93426	0.640283784	-0.545113	1.01E-05	hypomethylated
cg20268689	0.73717	0.505187027	-0.54518	2.71E-05	hypomethylated
cg11019923	0.4791	0.32828973	-0.545357	2.87E-06	hypomethylated
cg07191839	0.64988	0.445291892	-0.545422	8.42E-05	hypomethylated
cg25030266	0.7089	0.485657297	-0.545643	3.59E-06	hypomethylated
cg07125112	0.37445	0.256526486	-0.545665	5.58E-05	hypomethylated
cg24343430	0.64593	0.442508649	-0.545672	6.78E-05	hypomethylated
cg12850892	0.4425	0.303121081	-0.545783	0.000178	hypomethylated
cg12407070	0.77661	0.531945946	-0.545911	1.80E-05	hypomethylated
cg15428620	0.42349	0.290063784	-0.545958	0.0017	hypomethylated
cg22834542	0.86291	0.590985405	-0.546088	1.80E-05	hypomethylated
cg27314422	0.57646	0.39474	-0.546318	3.49E-06	hypomethylated
cg03085949	0.81669	0.559203784	-0.546414	9.07E-06	hypomethylated
cg20770006	0.51263	0.350976757	-0.546542	0.000191	hypomethylated
cg23048494	0.5942	0.406803784	-0.546616	3.15E-05	hypomethylated
cg27299047	0.62711	0.429322703	-0.546656	0.000293	hypomethylated
cg08478685	0.61685	0.422287568	-0.546694	0.000875	hypomethylated
cg26126806	0.49051	0.335773514	-0.546794	0.001031	hypomethylated
cg20329220	0.44632	0.305485405	-0.546975	0.000132	hypomethylated
cg15401418	0.68873	0.471392973	-0.547008	0.000454	hypomethylated
cg07068980	0.63473	0.434416757	-0.547063	4.10E-05	hypomethylated
cg03140135	0.42335	0.289738919	-0.547097	0.001603	hypomethylated
cg25203286	0.64682	0.442678378	-0.547105	0.000191	hypomethylated
cg18014547	0.76325	0.522342162	-0.547161	1.15E-05	hypomethylated
cg09186101	0.74543	0.510135135	-0.547193	2.10E-06	hypomethylated
cg23830540	0.70001	0.478995135	-0.547365	4.36E-05	hypomethylated
cg08142884	0.84518	0.578305405	-0.547427	3.11E-05	hypomethylated
cg25840926	0.49103	0.335972432	-0.547468	5.19E-05	hypomethylated
cg03875819	0.73923	0.505779459	-0.547515	0.000417	hypomethylated
cg07059925	0.56141	0.384107027	-0.547546	4.58E-05	hypomethylated
cg06344474	0.7831	0.535728108	-0.547696	1.72E-06	hypomethylated
cg07348845	0.54984	0.376131892	-0.547773	0.000152	hypomethylated
cg04431346	0.58118	0.397554054	-0.547834	3.40E-05	hypomethylated
cg10655396	0.44573	0.304895676	-0.547854	0.003441	hypomethylated
cg21214743	0.60625	0.414567027	-0.548307	8.60E-06	hypomethylated
cg21591452	0.78892	0.539464324	-0.548351	2.75E-06	hypomethylated
cg26107476	0.63099	0.431407568	-0.548566	5.19E-05	hypomethylated
cg11737334	0.51224	0.350201081	-0.548636	0.00095	hypomethylated
cg10433327	0.78377	0.535828649	-0.548659	1.80E-05	hypomethylated
cg10103635	0.55107	0.376731351	-0.548699	0.000268	hypomethylated

cg26211060	0.80334	0.549165946	-0.548769	2.05E-06	hypomethylated
cg00182893	0.69487	0.475	-0.548816	0.000107	hypomethylated
cg19113920	0.58457	0.399574595	-0.548911	0.000191	hypomethylated
cg12615982	0.53039	0.362535676	-0.548931	0.000262	hypomethylated
cg25898076	0.45623	0.311838919	-0.54896	0.000107	hypomethylated
cg03030267	0.69306	0.473712432	-0.548969	5.72E-05	hypomethylated
cg05655953	0.94671	0.647074054	-0.548992	2.99E-06	hypomethylated
cg20827128	0.6391	0.436807027	-0.549046	0.000667	hypomethylated
cg09182678	0.53543	0.365936216	-0.549106	0.002231	hypomethylated
cg00948520	0.70446	0.481368649	-0.549376	4.94E-05	hypomethylated
cg01612247	0.78811	0.538526486	-0.54938	0.00024	hypomethylated
cg12580943	0.71394	0.487794054	-0.549531	7.38E-05	hypomethylated
cg13329242	0.55069	0.376244865	-0.549569	4.47E-05	hypomethylated
cg20772593	0.78139	0.53383027	-0.549662	8.83E-06	hypomethylated
cg22509504	0.6322	0.431895135	-0.5497	9.95E-05	hypomethylated
cg09461545	0.5009	0.34214973	-0.549895	0.000174	hypomethylated
cg14526021	0.84256	0.575505946	-0.549949	6.54E-05	hypomethylated
cg13473184	0.86818	0.592992973	-0.549979	1.20E-05	hypomethylated
cg14996583	0.55887	0.381689189	-0.550114	0.000229	hypomethylated
cg10869531	0.7373	0.503522703	-0.550195	9.07E-06	hypomethylated
cg17021798	0.76826	0.524664865	-0.550198	3.49E-06	hypomethylated
cg18326022	0.87926	0.600446486	-0.550254	8.15E-06	hypomethylated
cg19846154	0.79886	0.545538919	-0.550261	5.86E-05	hypomethylated
cg17371070	0.81245	0.554783243	-0.550355	1.12E-05	hypomethylated
cg10262032	0.56813	0.387937297	-0.550398	0.000176	hypomethylated
cg05606455	0.76448	0.521977297	-0.550492	5.43E-06	hypomethylated
cg03079497	0.56519	0.385889189	-0.550549	0.002649	hypomethylated
cg13156207	0.79213	0.540829189	-0.550564	7.65E-05	hypomethylated
cg17213713	0.56193	0.383638919	-0.550641	0.000117	hypomethylated
cg13424302	0.61937	0.422850811	-0.550653	0.000511	hypomethylated
cg17735531	0.61966	0.423048649	-0.550653	0.000152	hypomethylated
cg09938443	0.75645	0.516413514	-0.550718	2.32E-05	hypomethylated
cg16512144	0.53688	0.366491351	-0.550821	0.000287	hypomethylated
cg03573390	0.76427	0.521698378	-0.550866	0.000107	hypomethylated
cg22153994	0.81144	0.553881081	-0.550908	2.15E-05	hypomethylated
cg05860566	0.54601	0.372689189	-0.550954	7.83E-06	hypomethylated
cg17143179	0.66262	0.452257838	-0.551036	5.19E-05	hypomethylated
cg06099014	0.78142	0.533332432	-0.551063	2.29E-06	hypomethylated
cg01777586	0.72088	0.491997297	-0.551109	4.48E-06	hypomethylated
cg18292904	0.93457	0.637766486	-0.551274	6.39E-06	hypomethylated
cg10122877	0.70572	0.481571892	-0.551345	7.32E-06	hypomethylated
cg22972055	0.73426	0.500996216	-0.551491	3.23E-05	hypomethylated
cg03977831	0.741	0.505554054	-0.551608	2.57E-05	hypomethylated
cg14491362	0.75932	0.518001081	-0.551753	3.21E-06	hypomethylated
cg14957186	0.26607	0.181508108	-0.551772	0.001085	hypomethylated
cg00028135	0.60734	0.414295676	-0.551844	0.000209	hypomethylated
cg10590925	0.56225	0.383521081	-0.551906	0.000293	hypomethylated
cg04257163	0.62599	0.426942162	-0.552099	5.86E-05	hypomethylated
cg19267205	0.48725	0.332311892	-0.552124	9.49E-05	hypomethylated
cg02331808	0.8094	0.551956757	-0.552298	2.64E-06	hypomethylated
cg21689902	0.71949	0.490638378	-0.552315	0.000159	hypomethylated
cg19803952	0.42374	0.288955676	-0.552331	7.20E-05	hypomethylated
cg01487910	0.66502	0.453471892	-0.552385	1.58E-05	hypomethylated
cg24996440	0.48501	0.330715676	-0.552423	0.000155	hypomethylated
cg27227804	0.66863	0.455888649	-0.552527	7.29E-05	hypomethylated
cg12743031	0.65486	0.446464865	-0.55264	8.03E-05	hypomethylated
cg10546210	0.64514	0.439795135	-0.552781	0.000109	hypomethylated
cg05427381	0.66718	0.454764865	-0.552955	9.95E-05	hypomethylated

cg02687883	0.61762	0.420978919	-0.552971	7.12E-06	hypomethylated
cg25507121	0.66366	0.452321081	-0.553097	3.59E-06	hypomethylated
cg08429815	0.85669	0.583875135	-0.553113	2.79E-06	hypomethylated
cg24704287	0.51755	0.352715676	-0.553193	0.00032	hypomethylated
cg23516451	0.72264	0.492482162	-0.553206	5.45E-05	hypomethylated
cg17244340	0.45027	0.306855135	-0.553233	0.002231	hypomethylated
cg16708465	0.64658	0.440635676	-0.553243	0.000506	hypomethylated
cg24768595	0.85476	0.58242	-0.553459	3.49E-06	hypomethylated
cg24363020	0.59342	0.404326486	-0.553533	8.32E-05	hypomethylated
cg18114811	0.81067	0.552345405	-0.553544	3.59E-06	hypomethylated
cg21519654	0.79345	0.540586486	-0.553614	2.92E-05	hypomethylated
cg19180828	0.5521	0.376138919	-0.553664	0.00039	hypomethylated
cg02872491	0.58706	0.399925946	-0.553775	1.15E-05	hypomethylated
cg19368016	0.54133	0.368765946	-0.553803	0.001734	hypomethylated
cg11534215	0.56903	0.387627568	-0.553834	5.45E-05	hypomethylated
cg26274662	0.31027	0.211353514	-0.553866	0.000931	hypomethylated
cg24189696	0.41126	0.280140541	-0.5539	0.001052	hypomethylated
cg15247093	0.90267	0.614872973	-0.55391	1.39E-05	hypomethylated
cg12581682	0.8662	0.590016757	-0.553944	1.12E-05	hypomethylated
cg01221209	0.94164	0.641385405	-0.553984	2.23E-06	hypomethylated
cg03520938	0.8283	0.564142162	-0.554095	2.21E-05	hypomethylated
cg26403171	0.72475	0.493590811	-0.554168	5.51E-05	hypomethylated
cg20998539	0.61317	0.417587027	-0.55421	0.000528	hypomethylated
cg05895618	0.6331	0.43107027	-0.55451	0.000129	hypomethylated
cg04508114	0.48599	0.330874595	-0.554642	0.001496	hypomethylated
cg06427571	0.52447	0.357063784	-0.554678	0.000445	hypomethylated
cg02749784	0.85267	0.580496216	-0.554701	9.82E-06	hypomethylated
cg04600000	0.61469	0.418444324	-0.554823	0.00012	hypomethylated
cg01772439	0.80157	0.545617297	-0.554939	1.39E-05	hypomethylated
cg08844698	0.5589	0.380407027	-0.555046	0.000132	hypomethylated
cg11954355	0.48249	0.328398919	-0.55505	0.000229	hypomethylated
cg08035351	0.62321	0.424175676	-0.555056	1.54E-06	hypomethylated
cg27105123	0.45976	0.312907027	-0.555147	8.42E-05	hypomethylated
cg25161912	0.57191	0.389216757	-0.555214	0.000426	hypomethylated
cg16273051	0.64313	0.437680541	-0.555232	3.76E-05	hypomethylated
cg15354065	0.61082	0.415682703	-0.555265	6.62E-05	hypomethylated
cg03648020	0.5115	0.348071351	-0.555351	0.000613	hypomethylated
cg20140201	0.76758	0.522275135	-0.555507	9.44E-06	hypomethylated
cg03527077	0.75409	0.513055135	-0.555623	1.05E-06	hypomethylated
cg05695699	0.87524	0.59548	-0.555626	5.73E-06	hypomethylated
cg00577202	0.35407	0.240894054	-0.555636	0.003078	hypomethylated
cg00651016	0.85133	0.579203243	-0.555649	7.62E-06	hypomethylated
cg22478317	0.4339	0.295198919	-0.555675	9.95E-05	hypomethylated
cg26631454	0.78154	0.531684865	-0.555748	1.42E-05	hypomethylated
cg18092351	0.9435	0.641828108	-0.555836	2.42E-06	hypomethylated
cg07167185	0.59213	0.402793514	-0.555873	4.73E-06	hypomethylated
cg13237068	0.77725	0.528680541	-0.555982	2.39E-05	hypomethylated
cg19249749	0.90453	0.615180541	-0.556158	1.67E-06	hypomethylated
cg07132183	0.83269	0.566250811	-0.556338	2.05E-06	hypomethylated
cg14205632	0.3925	0.266908108	-0.55635	6.70E-05	hypomethylated
cg19673376	0.79077	0.537730811	-0.556374	8.83E-06	hypomethylated
cg10752406	0.60301	0.41002	-0.556488	0.000551	hypomethylated
cg07678594	0.61635	0.419055676	-0.556608	8.37E-06	hypomethylated
cg16365424	0.73454	0.499391892	-0.556669	0.000563	hypomethylated
cg20446824	0.55729	0.378855135	-0.556782	0.000426	hypomethylated
cg10173586	0.52123	0.354314054	-0.556891	0.000417	hypomethylated
cg10373607	0.79387	0.539474595	-0.557348	6.62E-05	hypomethylated
cg10395101	0.62504	0.42474	-0.557369	0.000517	hypomethylated

cg15095917	0.32449	0.220501081	-0.557388	0.000386	hypomethylated
cg15729154	0.77039	0.523465946	-0.557493	1.33E-05	hypomethylated
cg27505627	0.9412	0.639483784	-0.557594	1.82E-06	hypomethylated
cg10214581	0.50867	0.345607027	-0.557597	0.000154	hypomethylated
cg07842521	0.60228	0.409206486	-0.557605	3.23E-05	hypomethylated
cg07511317	0.90032	0.611701622	-0.55761	4.24E-06	hypomethylated
cg20366110	0.65453	0.444675676	-0.557706	0.000674	hypomethylated
cg08134678	0.63451	0.431068649	-0.557725	0.003843	hypomethylated
cg18050804	0.62213	0.42259027	-0.557957	0.000313	hypomethylated
cg01749904	0.6737	0.457604865	-0.558004	1.94E-05	hypomethylated
cg06214007	0.59515	0.404228649	-0.558082	0.000805	hypomethylated
cg16489259	0.41301	0.280501081	-0.55817	8.03E-05	hypomethylated
cg06145435	0.59453	0.403782703	-0.558171	5.31E-05	hypomethylated
cg08607108	0.84381	0.573080541	-0.55818	4.36E-05	hypomethylated
cg12787571	0.77579	0.52684	-0.558301	2.07E-05	hypomethylated
cg25002294	0.83335	0.565896757	-0.558384	3.49E-06	hypomethylated
cg06513470	0.82334	0.559083784	-0.558424	5.03E-07	hypomethylated
cg10625247	0.84548	0.574092973	-0.558486	6.05E-06	hypomethylated
cg16405432	0.57642	0.391395676	-0.558493	0.000132	hypomethylated
cg01116477	0.84151	0.571363784	-0.558571	8.15E-06	hypomethylated
cg01919768	0.64215	0.435983784	-0.558636	1.39E-05	hypomethylated
cg24413842	0.55257	0.375158919	-0.558655	0.001875	hypomethylated
cg04469998	0.68086	0.462258378	-0.558659	2.57E-06	hypomethylated
cg07319315	0.89456	0.607302703	-0.558762	9.82E-06	hypomethylated
cg23482746	0.73315	0.497721081	-0.558771	1.04E-05	hypomethylated
cg25899154	0.57736	0.391895676	-0.559002	6.31E-05	hypomethylated
cg06565975	0.78898	0.535505405	-0.559088	0.000145	hypomethylated
cg13984330	0.84758	0.575269189	-0.559112	9.07E-06	hypomethylated
cg10064922	0.84249	0.571777838	-0.559205	8.63E-05	hypomethylated
cg08850243	0.72709	0.493436757	-0.559269	0.000104	hypomethylated
cg10211456	0.5529	0.375184865	-0.559417	0.000226	hypomethylated
cg26703507	0.68116	0.462203243	-0.559466	0.000528	hypomethylated
cg10564001	0.77285	0.524412973	-0.559485	4.05E-05	hypomethylated
cg23091777	0.87966	0.596877838	-0.55951	7.32E-06	hypomethylated
cg08610982	0.40384	0.274012973	-0.55954	0.00095	hypomethylated
cg06655216	0.59346	0.402671892	-0.559546	0.000408	hypomethylated
cg01824603	0.77997	0.529155135	-0.559728	1.18E-05	hypomethylated
cg24749089	0.69459	0.47122973	-0.559731	7.84E-05	hypomethylated
cg18882156	0.50811	0.344716216	-0.559732	0.000142	hypomethylated
cg13725899	0.81593	0.553518378	-0.559814	6.57E-06	hypomethylated
cg05784862	0.70976	0.481485405	-0.559839	6.15E-05	hypomethylated
cg02704570	0.46813	0.317529189	-0.56002	0.00291	hypomethylated
cg23342625	0.80943	0.54902973	-0.560022	1.52E-05	hypomethylated
cg09633240	0.55711	0.377876216	-0.560048	0.000138	hypomethylated
cg27461542	0.8772	0.594970811	-0.560087	2.75E-06	hypomethylated
cg26283879	0.41185	0.279335676	-0.560119	0.001949	hypomethylated
cg01464703	0.67941	0.460788649	-0.560177	0.000313	hypomethylated
cg07804749	0.57418	0.389395676	-0.560266	8.13E-05	hypomethylated
cg19107296	0.66062	0.448011351	-0.560285	3.23E-05	hypomethylated
cg10122294	0.71422	0.484357297	-0.560297	0.000182	hypomethylated
cg12924899	0.76421	0.518242703	-0.560341	1.75E-05	hypomethylated
cg14778437	0.7894	0.535320541	-0.560354	4.94E-05	hypomethylated
cg27187375	0.85369	0.578907568	-0.560379	3.04E-06	hypomethylated
cg02733351	0.84576	0.57349027	-0.560479	3.21E-06	hypomethylated
cg25591451	0.51946	0.352224865	-0.560516	0.000327	hypomethylated
cg06030535	0.81983	0.555858378	-0.560607	8.03E-05	hypomethylated
cg25349729	0.92522	0.627296216	-0.56065	2.13E-06	hypomethylated
cg16777510	0.67389	0.456891351	-0.560662	0.001949	hypomethylated

cg09120267	0.73613	0.499084865	-0.560675	3.59E-06	hypomethylated
cg03653026	0.55135	0.373794595	-0.560723	0.001875	hypomethylated
cg14424070	0.656	0.444692432	-0.560888	0.001052	hypomethylated
cg13712023	0.65903	0.4467	-0.561038	0.00024	hypomethylated
cg22566740	0.63696	0.431685405	-0.561222	2.57E-05	hypomethylated
cg01775802	0.65606	0.444612432	-0.561279	0.000209	hypomethylated
cg12011299	0.77837	0.527492432	-0.561306	8.22E-05	hypomethylated
cg02271139	0.72331	0.490141622	-0.561415	1.02E-06	hypomethylated
cg09113070	0.47845	0.324208649	-0.561446	0.001571	hypomethylated
cg13578134	0.69966	0.474042703	-0.561637	0.001481	hypomethylated
cg16466870	0.7297	0.494377297	-0.561691	1.41E-06	hypomethylated
cg10805880	0.50822	0.344311892	-0.561737	0.007799	hypomethylated
cg05514909	0.64565	0.437399459	-0.561801	6.78E-05	hypomethylated
cg00934987	0.40755	0.276096216	-0.561806	0.000293	hypomethylated
cg09839874	0.84739	0.574054595	-0.561838	1.50E-05	hypomethylated
cg03301165	0.82833	0.561113514	-0.561913	2.21E-05	hypomethylated
cg11370814	0.67254	0.455577297	-0.561924	0.000399	hypomethylated
cg18278424	0.46408	0.31435027	-0.562001	4.05E-05	hypomethylated
cg18688062	0.54388	0.368398919	-0.56202	1.25E-05	hypomethylated
cg15403283	0.90828	0.61520973	-0.562059	1.12E-05	hypomethylated
cg07215695	0.67617	0.457957838	-0.562171	2.38E-05	hypomethylated
cg25855001	0.78564	0.532056757	-0.562288	2.92E-05	hypomethylated
cg19242984	0.73581	0.498307568	-0.562297	8.63E-05	hypomethylated
cg07090714	0.46046	0.311805405	-0.56243	0.000365	hypomethylated
cg00232535	0.83013	0.56206973	-0.562588	8.15E-06	hypomethylated
cg25655096	0.50698	0.343202703	-0.562868	0.001188	hypomethylated
cg26753302	0.78565	0.531849189	-0.56287	5.89E-06	hypomethylated
cg09318763	0.64632	0.437528108	-0.562873	8.84E-05	hypomethylated
cg25332717	0.45631	0.308884324	-0.562948	8.63E-05	hypomethylated
cg24629380	0.92766	0.627934595	-0.562982	1.90E-06	hypomethylated
cg19402314	0.56078	0.379576757	-0.563043	9.07E-06	hypomethylated
cg21204860	0.41134	0.278423243	-0.563052	0.000123	hypomethylated
cg19272348	0.75727	0.512572432	-0.563052	2.78E-05	hypomethylated
cg24222580	0.57292	0.387765405	-0.56315	0.000382	hypomethylated
cg03925642	0.71335	0.482687027	-0.563522	4.47E-05	hypomethylated
cg00018181	0.44292	0.299700541	-0.563524	0.000242	hypomethylated
cg19906672	0.51775	0.350331892	-0.563533	5.19E-05	hypomethylated
cg06048750	0.60108	0.406704865	-0.563575	0.000772	hypomethylated
cg22700848	0.43925	0.297205405	-0.563582	0.000129	hypomethylated
cg09508668	0.6181	0.418185405	-0.563698	0.000262	hypomethylated
cg06136702	0.40742	0.275630811	-0.56378	5.86E-05	hypomethylated
cg19015997	0.66431	0.449421081	-0.563789	9.31E-06	hypomethylated
cg02082342	0.70452	0.476620541	-0.5638	1.50E-05	hypomethylated
cg23623251	0.77526	0.524420541	-0.563956	1.35E-05	hypomethylated
cg20663219	0.75994	0.514026486	-0.564043	5.14E-06	hypomethylated
cg16204717	0.42667	0.288575135	-0.564174	0.000681	hypomethylated
cg17416644	0.66736	0.451326486	-0.564294	0.000166	hypomethylated
cg11438560	0.37135	0.251138378	-0.564297	0.00032	hypomethylated
cg20703003	0.82395	0.557204324	-0.56435	8.37E-06	hypomethylated
cg21063296	0.40212	0.271924865	-0.564418	3.44E-05	hypomethylated
cg26189213	0.74603	0.504452973	-0.564514	7.47E-05	hypomethylated
cg11982525	0.60691	0.410377838	-0.56453	0.00255	hypomethylated
cg00738772	0.645	0.436112973	-0.564597	0.000112	hypomethylated
cg01037823	0.6661	0.450364324	-0.564646	4.82E-05	hypomethylated
cg12385643	0.8032	0.543056757	-0.564656	1.82E-06	hypomethylated
cg04276058	0.55082	0.372404324	-0.564711	0.001571	hypomethylated
cg16526732	0.73695	0.498082703	-0.565181	1.48E-05	hypomethylated
cg09350411	0.64002	0.43256973	-0.565184	0.000417	hypomethylated

cg13332142	0.83236	0.562542703	-0.565245	2.32E-05	hypomethylated
cg13443911	0.72877	0.492527027	-0.565261	0.000135	hypomethylated
cg10904867	0.61664	0.416723784	-0.565337	1.58E-06	hypomethylated
cg03158310	0.62674	0.423546486	-0.565347	3.11E-05	hypomethylated
cg01022670	0.77127	0.521212973	-0.565363	4.05E-05	hypomethylated
cg01015899	0.69317	0.468355135	-0.565606	0.000126	hypomethylated
cg26494916	0.80481	0.54378	-0.565625	6.93E-06	hypomethylated
cg15049549	0.62024	0.419057297	-0.565679	0.000195	hypomethylated
cg13823919	0.76171	0.514603784	-0.56578	3.04E-06	hypomethylated
cg24897320	0.70073	0.473394054	-0.565817	6.93E-06	hypomethylated
cg24296478	0.2787	0.188251892	-0.566049	0.000581	hypomethylated
cg25292309	0.68701	0.46392	-0.566455	2.15E-05	hypomethylated
cg09924669	0.5811	0.392394595	-0.566481	0.000148	hypomethylated
cg26178163	0.69313	0.468018919	-0.566559	0.000274	hypomethylated
cg13058710	0.3687	0.248954595	-0.566565	8.32E-05	hypomethylated
cg03018256	0.73336	0.495154595	-0.566643	2.15E-05	hypomethylated
cg03290213	0.86894	0.586640541	-0.56678	1.70E-06	hypomethylated
cg01174811	0.83993	0.566984865	-0.566959	1.40E-05	hypomethylated
cg20749059	0.48918	0.330214054	-0.566964	0.000382	hypomethylated
cg21965980	0.66027	0.445677297	-0.567057	2.85E-05	hypomethylated
cg26446832	0.81927	0.552960541	-0.567162	9.57E-06	hypomethylated
cg07177395	0.70206	0.473810811	-0.567283	0.000575	hypomethylated
cg23866916	0.68071	0.459366486	-0.567395	3.76E-05	hypomethylated
cg23957643	0.78207	0.527755676	-0.567428	2.46E-06	hypomethylated
cg17114584	0.52343	0.353218378	-0.567436	1.77E-06	hypomethylated
cg16009407	0.63094	0.425738378	-0.567536	0.000741	hypomethylated
cg01569082	0.57633	0.388888649	-0.567538	3.21E-06	hypomethylated
cg08710564	0.75665	0.510538919	-0.567605	3.40E-05	hypomethylated
cg13466284	0.756	0.510097838	-0.567612	8.26E-06	hypomethylated
cg26800893	0.49909	0.33675027	-0.567621	0.000335	hypomethylated
cg18563987	0.74751	0.504355676	-0.567651	1.15E-05	hypomethylated
cg05467458	0.74578	0.503188108	-0.567652	1.40E-05	hypomethylated
cg26210250	0.65403	0.441253514	-0.567749	0.001	hypomethylated
cg20972199	0.53926	0.363818919	-0.56776	0.001803	hypomethylated
cg08837215	0.75124	0.506832973	-0.567763	3.85E-05	hypomethylated
cg12229082	0.67503	0.455407027	-0.567795	1.93E-06	hypomethylated
cg20220678	0.7712	0.520251351	-0.567896	3.32E-05	hypomethylated
cg03417788	0.91555	0.61754973	-0.568083	8.60E-06	hypomethylated
cg20716202	0.8043	0.542503784	-0.568101	3.90E-06	hypomethylated
cg03304763	0.7717	0.520498919	-0.568145	8.03E-05	hypomethylated
cg26596719	0.65236	0.439997838	-0.568172	0.00094	hypomethylated
cg10064525	0.62266	0.419922703	-0.568321	1.31E-05	hypomethylated
cg24587080	0.46879	0.316150811	-0.568329	0.000129	hypomethylated
cg23381232	0.64109	0.432341622	-0.568355	9.95E-05	hypomethylated
cg22969914	0.60903	0.410716757	-0.568369	0.000756	hypomethylated
cg27388703	0.62663	0.42258	-0.568389	0.001912	hypomethylated
cg20778199	0.4991	0.336557297	-0.568477	0.00013	hypomethylated
cg08396863	0.25584	0.172512973	-0.568537	0.000749	hypomethylated
cg19838087	0.44707	0.301436757	-0.568645	0.001118	hypomethylated
cg21189909	0.43711	0.294706486	-0.568718	0.000102	hypomethylated
cg24647820	0.64239	0.433105946	-0.568729	9.49E-05	hypomethylated
cg09223940	0.80864	0.545167027	-0.568799	1.84E-05	hypomethylated
cg24199006	0.56973	0.384060541	-0.568945	0.005411	hypomethylated
cg25546651	0.83099	0.560155135	-0.569005	4.18E-06	hypomethylated
cg09769113	0.94628	0.637796216	-0.569172	1.20E-06	hypomethylated
cg05576245	0.52196	0.351794054	-0.569208	8.52E-05	hypomethylated
cg25908283	0.52789	0.355785946	-0.569228	4.82E-05	hypomethylated
cg23534216	0.66801	0.450179459	-0.569369	0.000412	hypomethylated

cg14976447	0.65986	0.444684865	-0.569377	0.000789	hypomethylated
cg00974944	0.85334	0.575020541	-0.569507	1.31E-05	hypomethylated
cg10837404	0.90955	0.61288	-0.569548	3.21E-06	hypomethylated
cg03651054	0.62583	0.421678378	-0.569628	3.07E-05	hypomethylated
cg17383842	0.50176	0.338067568	-0.569686	0.003571	hypomethylated
cg23946364	0.74442	0.501560541	-0.569693	8.60E-06	hypomethylated
cg23903301	0.68899	0.464191892	-0.569762	0.000135	hypomethylated
cg21268653	0.31065	0.209268108	-0.569938	0.0006	hypomethylated
cg02820413	0.59361	0.399858919	-0.570024	6.22E-06	hypomethylated
cg08580187	0.65083	0.438377297	-0.570108	0.001803	hypomethylated
cg19028462	0.80732	0.543754595	-0.570185	4.05E-05	hypomethylated
cg03189821	0.78798	0.530724324	-0.570196	1.58E-05	hypomethylated
cg26250086	0.41641	0.28044	-0.570313	7.93E-05	hypomethylated
cg21406967	0.38104	0.256612973	-0.570348	0.000214	hypomethylated
cg08326468	0.69737	0.469625946	-0.570412	2.87E-06	hypomethylated
cg13443733	0.48186	0.324494054	-0.570422	0.004951	hypomethylated
cg09749669	0.68024	0.458069189	-0.570478	5.45E-05	hypomethylated
cg26752663	0.72205	0.486215135	-0.570504	7.65E-05	hypomethylated
cg08401725	0.5595	0.376756757	-0.570505	8.22E-05	hypomethylated
cg02186444	0.73374	0.494075135	-0.570538	1.04E-05	hypomethylated
cg20475082	0.73782	0.49679027	-0.570632	0.000195	hypomethylated
cg22937172	0.91509	0.616147568	-0.570638	1.82E-06	hypomethylated
cg22214889	0.63016	0.424278919	-0.570705	0.002624	hypomethylated
cg15078284	0.33355	0.224574054	-0.570712	0.001667	hypomethylated
cg09709457	0.73465	0.494619459	-0.570738	7.38E-05	hypomethylated
cg07392307	0.72326	0.486934595	-0.570786	1.09E-06	hypomethylated
cg05155319	0.45143	0.30388973	-0.570954	3.15E-05	hypomethylated
cg18158670	0.83359	0.561138919	-0.57098	7.93E-06	hypomethylated
cg24693436	0.89788	0.60435027	-0.571138	6.20E-07	hypomethylated
cg13758054	0.45779	0.308107568	-0.571252	0.006915	hypomethylated
cg12491114	0.67396	0.453589189	-0.571277	0.000142	hypomethylated
cg11060604	0.83781	0.563855676	-0.571297	7.32E-06	hypomethylated
cg27076536	0.81884	0.551031892	-0.571446	8.60E-06	hypomethylated
cg10061532	0.58124	0.391134054	-0.571471	0.000107	hypomethylated
cg02486332	0.60256	0.405474054	-0.571495	5.65E-06	hypomethylated
cg10075976	0.58727	0.395179459	-0.571516	0.003441	hypomethylated
cg21531300	0.58855	0.396027568	-0.571564	0.000857	hypomethylated
cg22001807	0.51632	0.347374595	-0.571773	0.003378	hypomethylated
cg12413579	0.51112	0.34384973	-0.571884	0.000112	hypomethylated
cg03661324	0.7434	0.500086486	-0.571961	0.000174	hypomethylated
cg08227353	0.71282	0.479452432	-0.57215	0.00013	hypomethylated
cg23828876	0.79764	0.536492973	-0.572178	0.000232	hypomethylated
cg17322774	0.58162	0.391190811	-0.572204	2.87E-06	hypomethylated
cg19763108	0.64885	0.436404865	-0.572218	0.000274	hypomethylated
cg09802186	0.79251	0.533026486	-0.572222	3.23E-05	hypomethylated
cg06137072	0.53854	0.362206486	-0.572241	0.000224	hypomethylated
cg24724689	0.5211	0.350452973	-0.572339	0.000485	hypomethylated
cg19907725	0.60361	0.405902703	-0.572483	4.94E-05	hypomethylated
cg14340336	0.41522	0.279188108	-0.572638	0.001423	hypomethylated
cg03175417	0.75618	0.508435135	-0.572666	3.12E-06	hypomethylated
cg16888565	0.75927	0.510485405	-0.572743	1.84E-05	hypomethylated
cg05827190	0.69696	0.46857027	-0.57281	5.86E-05	hypomethylated
cg20477160	0.82903	0.557270811	-0.573046	6.95E-05	hypomethylated
cg25865120	0.63353	0.425821081	-0.573166	0.000378	hypomethylated
cg18461347	0.62855	0.422445405	-0.573263	7.32E-06	hypomethylated
cg03982897	0.4936	0.331744865	-0.573268	5.86E-05	hypomethylated
cg22052143	0.71073	0.47763027	-0.573407	0.000517	hypomethylated
cg13443768	0.67582	0.454165946	-0.57342	2.42E-05	hypomethylated

cg11755201	0.38321	0.257516216	-0.573472	0.000303	hypomethylated
cg11029367	0.81047	0.544597838	-0.573568	1.42E-05	hypomethylated
cg14785527	0.64871	0.435892973	-0.5736	0.001262	hypomethylated
cg07427475	0.67249	0.451841622	-0.573696	0.00039	hypomethylated
cg02915920	0.62508	0.419959459	-0.573791	5.19E-05	hypomethylated
cg17662083	0.69851	0.469221081	-0.574013	9.31E-06	hypomethylated
cg17656763	0.63518	0.42667027	-0.574044	0.000132	hypomethylated
cg05355436	0.7157	0.480744865	-0.574084	0.000127	hypomethylated
cg07168232	0.45875	0.308101081	-0.574304	0.001314	hypomethylated
cg16442312	0.74221	0.498458378	-0.574354	7.42E-06	hypomethylated
cg05373251	0.76398	0.513064865	-0.574394	0.00015	hypomethylated
cg05470166	0.484	0.325037838	-0.574399	1.62E-05	hypomethylated
cg27143703	0.91921	0.617292973	-0.574439	2.87E-06	hypomethylated
cg06595154	0.55472	0.372478378	-0.574603	0.000626	hypomethylated
cg13857678	0.61117	0.410358378	-0.574689	0.004951	hypomethylated
cg07064544	0.92584	0.621627568	-0.574712	6.84E-06	hypomethylated
cg03596178	0.69631	0.467481622	-0.57482	7.22E-06	hypomethylated
cg22449980	0.86619	0.581527568	-0.574836	3.79E-06	hypomethylated
cg18427091	0.48888	0.328212432	-0.57485	6.70E-05	hypomethylated
cg05662664	0.8115	0.544784324	-0.574906	1.42E-05	hypomethylated
cg06121808	0.56908	0.382021622	-0.574977	0.001314	hypomethylated
cg16100355	0.92824	0.623078919	-0.575083	5.89E-06	hypomethylated
cg18449021	0.51028	0.342497838	-0.575194	0.000445	hypomethylated
cg05413061	0.82035	0.550608649	-0.575212	5.14E-06	hypomethylated
cg19628739	0.57613	0.386689189	-0.57522	0.000102	hypomethylated
cg13883671	0.92403	0.620181081	-0.57525	3.79E-06	hypomethylated
cg04423820	0.80376	0.539396216	-0.575419	1.18E-06	hypomethylated
cg25202503	0.8983	0.602823784	-0.575461	6.68E-07	hypomethylated
cg09274344	0.5225	0.350621622	-0.575516	0.000741	hypomethylated
cg20587236	0.55919	0.37520973	-0.575641	0.000931	hypomethylated
cg04948438	0.71894	0.482387027	-0.57568	2.10E-05	hypomethylated
cg14794041	0.41863	0.280875676	-0.575744	8.22E-05	hypomethylated
cg10593028	0.7941	0.532783784	-0.575771	1.71E-05	hypomethylated
cg11306361	0.3171	0.212733514	-0.575891	0.000219	hypomethylated
cg15552491	0.7084	0.475221622	-0.575964	1.80E-05	hypomethylated
cg23939096	0.60781	0.407654054	-0.576275	0.000313	hypomethylated
cg09644356	0.74245	0.497949189	-0.576295	3.79E-06	hypomethylated
cg00598204	0.79592	0.533791351	-0.576347	2.85E-05	hypomethylated
cg26449680	0.33719	0.226097297	-0.576618	0.000408	hypomethylated
cg15377758	0.79395	0.532311892	-0.576776	1.99E-06	hypomethylated
cg14270581	0.50606	0.339261081	-0.576913	0.003316	hypomethylated
cg18002126	0.70374	0.471674595	-0.577251	0.000287	hypomethylated
cg02473237	0.74994	0.502631351	-0.577275	1.05E-06	hypomethylated
cg06694040	0.47622	0.319172432	-0.577292	0.002455	hypomethylated
cg00085790	0.78171	0.523917838	-0.577293	2.42E-06	hypomethylated
cg03753813	0.55291	0.37056973	-0.5773	8.63E-05	hypomethylated
cg05212892	0.88752	0.594777838	-0.577429	4.24E-06	hypomethylated
cg08371852	0.77773	0.521115676	-0.577666	4.48E-06	hypomethylated
cg04818594	0.77992	0.522576216	-0.577685	8.13E-05	hypomethylated
cg22417613	0.67642	0.453224865	-0.577692	0.000234	hypomethylated
cg20430773	0.61332	0.410907568	-0.577826	0.001481	hypomethylated
cg17702736	0.79004	0.529284865	-0.577881	4.94E-05	hypomethylated
cg08528486	0.86863	0.581911351	-0.577942	1.68E-05	hypomethylated
cg15549637	0.70762	0.474015676	-0.57804	0.000102	hypomethylated
cg11293828	0.40592	0.271910811	-0.578062	4.94E-05	hypomethylated
cg03301498	0.72003	0.482319459	-0.578068	5.58E-05	hypomethylated
cg03326606	0.83619	0.56011027	-0.57812	1.99E-05	hypomethylated
cg10328047	0.72559	0.485981081	-0.578254	4.01E-06	hypomethylated

cg07139495	0.73156	0.489974595	-0.578269	3.90E-06	hypomethylated
cg03139377	0.59179	0.396351892	-0.578303	0.000145	hypomethylated
cg24391989	0.7487	0.501425405	-0.578353	2.15E-05	hypomethylated
cg15378486	0.48601	0.325489189	-0.578376	5.45E-05	hypomethylated
cg06851151	0.59253	0.396796216	-0.57849	0.000313	hypomethylated
cg12506165	0.55493	0.371616757	-0.57849	0.000839	hypomethylated
cg01509243	0.55984	0.374892973	-0.578536	0.002699	hypomethylated
cg19303524	0.81478	0.545608108	-0.578545	4.36E-05	hypomethylated
cg20446334	0.90154	0.603670811	-0.578629	3.62E-05	hypomethylated
cg03634833	0.71592	0.479341622	-0.578744	4.15E-05	hypomethylated
cg07927984	0.52679	0.352681622	-0.578862	7.47E-05	hypomethylated
cg04342594	0.50018	0.334864324	-0.578871	0.004445	hypomethylated
cg10371155	0.69179	0.463076757	-0.579083	0.000374	hypomethylated
cg02590088	0.5443	0.364347568	-0.579087	8.42E-05	hypomethylated
cg09716613	0.53693	0.359371892	-0.579256	0.002803	hypomethylated
cg18318006	0.7222	0.483328108	-0.579396	0.000178	hypomethylated
cg06481431	0.48037	0.321468108	-0.57947	0.000262	hypomethylated
cg26883251	0.37047	0.247915135	-0.57951	0.000805	hypomethylated
cg23633330	0.39853	0.266691892	-0.579514	0.000454	hypomethylated
cg00206063	0.87524	0.585662703	-0.579609	6.93E-06	hypomethylated
cg05781996	0.52319	0.350065405	-0.57971	0.000506	hypomethylated
cg03502601	0.64607	0.432271892	-0.579751	0.000219	hypomethylated
cg20768506	0.61109	0.408803243	-0.579978	1.15E-05	hypomethylated
cg13639881	0.42562	0.284726486	-0.579989	0.0003	hypomethylated
cg21127079	0.74666	0.499454054	-0.580099	0.000495	hypomethylated
cg15903956	0.64282	0.429991351	-0.580107	0.000159	hypomethylated
cg26763394	0.68727	0.459723784	-0.58011	3.95E-05	hypomethylated
cg02988698	0.36143	0.241763784	-0.580118	0.006395	hypomethylated
cg01834856	0.60936	0.407592973	-0.580166	0.000268	hypomethylated
cg05132999	0.76396	0.510987027	-0.58021	3.44E-06	hypomethylated
cg01965380	0.7047	0.471343784	-0.58023	0.000251	hypomethylated
cg10492240	0.81848	0.547416216	-0.580309	7.20E-07	hypomethylated
cg24088438	0.50887	0.340337297	-0.580332	0.000725	hypomethylated
cg00838397	0.86255	0.576876757	-0.580345	9.31E-06	hypomethylated
cg26645709	0.56939	0.38078	-0.580459	0.000224	hypomethylated
cg08026735	0.37377	0.249954054	-0.580488	5.72E-05	hypomethylated
cg21330727	0.72876	0.487341622	-0.58051	2.05E-06	hypomethylated
cg15342087	0.56142	0.37542	-0.580575	0.000193	hypomethylated
cg00374672	0.54062	0.361446486	-0.580833	0.00028	hypomethylated
cg16353318	0.80427	0.53765027	-0.581012	4.60E-06	hypomethylated
cg01733438	0.72885	0.487221081	-0.581045	0.000166	hypomethylated
cg08564522	0.6591	0.440576757	-0.581104	2.64E-05	hypomethylated
cg07241909	0.51463	0.343963784	-0.581279	9.72E-05	hypomethylated
cg00414077	0.6902	0.461308649	-0.581282	2.79E-06	hypomethylated
cg05277122	0.89795	0.600089189	-0.581458	2.10E-05	hypomethylated
cg14112601	0.59698	0.398947027	-0.581485	0.001188	hypomethylated
cg05649391	0.91825	0.613595676	-0.581599	1.80E-05	hypomethylated
cg23622047	0.69677	0.465493514	-0.581921	5.51E-05	hypomethylated
cg15044270	0.44955	0.30032	-0.581981	7.65E-05	hypomethylated
cg04540406	0.46617	0.311418378	-0.582002	0.001734	hypomethylated
cg17884383	0.59215	0.395566486	-0.582042	3.21E-06	hypomethylated
cg23191354	0.74229	0.49578973	-0.582255	3.57E-05	hypomethylated
cg04948649	0.73962	0.493995135	-0.582287	0.000214	hypomethylated
cg24924502	0.82872	0.553493514	-0.582318	1.99E-05	hypomethylated
cg11847636	0.66473	0.443944324	-0.58239	0.00028	hypomethylated
cg16058274	0.73422	0.490328649	-0.582463	1.62E-05	hypomethylated
cg23400169	0.68483	0.457337838	-0.582486	7.29E-05	hypomethylated
cg23080427	0.45681	0.305059459	-0.582504	4.47E-05	hypomethylated

cg00204512	0.53421	0.356741081	-0.58253	0.000166	hypomethylated
cg10321973	0.39566	0.264208649	-0.582584	0.002231	hypomethylated
cg17330303	0.90339	0.603221081	-0.582662	1.31E-05	hypomethylated
cg03283990	0.685	0.457385946	-0.582692	5.00E-06	hypomethylated
cg19017142	0.63482	0.423876757	-0.582703	0.002803	hypomethylated
cg21538684	0.70043	0.467681081	-0.582716	0.001511	hypomethylated
cg11454459	0.71854	0.479752432	-0.582778	2.27E-05	hypomethylated
cg27326306	0.5505	0.367533514	-0.582867	0.000265	hypomethylated
cg12308965	0.52994	0.353776216	-0.582992	1.77E-06	hypomethylated
cg23030853	0.83352	0.556437838	-0.582996	5.14E-06	hypomethylated
cg26467269	0.82694	0.552039459	-0.583011	7.20E-07	hypomethylated
cg21086551	0.79452	0.530372432	-0.583078	1.50E-05	hypomethylated
cg17596359	0.64207	0.428575135	-0.583182	4.58E-05	hypomethylated
cg19648552	0.75171	0.50170973	-0.583323	9.31E-06	hypomethylated
cg18923740	0.34481	0.230132432	-0.583337	0.002066	hypomethylated
cg18006637	0.54712	0.365129189	-0.58345	0.002409	hypomethylated
cg16213217	0.78296	0.522482162	-0.583557	7.56E-05	hypomethylated
cg10275917	0.64239	0.428631351	-0.583712	2.85E-05	hypomethylated
cg20305489	0.76519	0.510547027	-0.583774	0.000234	hypomethylated
cg25747670	0.67	0.446958378	-0.584021	1.04E-05	hypomethylated
cg19497767	0.72406	0.483008649	-0.58406	6.39E-06	hypomethylated
cg18372930	0.44845	0.299135676	-0.584147	0.001541	hypomethylated
cg09082823	0.79351	0.529262162	-0.584266	1.37E-06	hypomethylated
cg18717167	0.57789	0.385432973	-0.584315	0.000185	hypomethylated
cg04003327	0.79223	0.52838	-0.584343	1.44E-05	hypomethylated
cg05045027	0.47318	0.315585946	-0.584356	0.00031	hypomethylated
cg16319142	0.86794	0.578837838	-0.584436	2.71E-06	hypomethylated
cg19097880	0.66469	0.443285946	-0.584444	3.40E-05	hypomethylated
cg24084564	0.6041	0.402847027	-0.584555	1.93E-06	hypomethylated
cg05582690	0.84894	0.566048649	-0.584737	1.18E-05	hypomethylated
cg26071033	0.41955	0.279702703	-0.584949	0.00095	hypomethylated
cg01033299	0.48236	0.321574595	-0.584957	0.000374	hypomethylated
cg03944072	0.80431	0.536162703	-0.585081	2.16E-06	hypomethylated
cg07915516	0.40572	0.270452432	-0.58511	3.23E-05	hypomethylated
cg03078767	0.86421	0.576032973	-0.585231	1.15E-05	hypomethylated
cg21845080	0.6984	0.465495676	-0.585286	1.11E-05	hypomethylated
cg19445335	0.89548	0.596792973	-0.585431	9.38E-07	hypomethylated
cg15597364	0.59128	0.39405027	-0.585462	7.84E-05	hypomethylated
cg17172331	0.6648	0.443001081	-0.58561	8.42E-05	hypomethylated
cg24643393	0.46203	0.307848649	-0.585765	0.000148	hypomethylated
cg20468415	0.55151	0.367462162	-0.585791	0.000161	hypomethylated
cg06586046	0.66005	0.439723784	-0.585978	6.22E-06	hypomethylated
cg08386009	0.54258	0.361431351	-0.586114	2.05E-06	hypomethylated
cg20954533	0.50655	0.337418919	-0.586164	0.002751	hypomethylated
cg16015593	0.58735	0.391231351	-0.586198	0.000142	hypomethylated
cg23166988	0.26693	0.177781081	-0.58636	0.000386	hypomethylated
cg10927461	0.54589	0.363520541	-0.586573	0.000262	hypomethylated
cg15849060	0.83036	0.552952432	-0.586582	9.31E-06	hypomethylated
cg12951282	0.39459	0.262723784	-0.586808	0.000417	hypomethylated
cg18776616	0.77481	0.515831892	-0.586942	8.83E-06	hypomethylated
cg07403899	0.57164	0.380544324	-0.587042	0.000485	hypomethylated
cg05652225	0.58049	0.38642973	-0.587065	6.93E-06	hypomethylated
cg14986890	0.79313	0.52796973	-0.587102	8.03E-05	hypomethylated
cg23534766	0.65469	0.435803784	-0.587133	1.56E-05	hypomethylated
cg18628646	0.73385	0.488461081	-0.587242	8.13E-05	hypomethylated
cg10785793	0.66723	0.444115135	-0.58725	0.001074	hypomethylated
cg03292149	0.84428	0.56192	-0.587357	5.28E-06	hypomethylated
cg23668195	0.57516	0.382770811	-0.587483	0.000129	hypomethylated

cg19255608	0.57735	0.384185405	-0.587643	4.36E-05	hypomethylated
cg14010805	0.28446	0.189283784	-0.587675	5.58E-05	hypomethylated
cg09063683	0.67613	0.449898919	-0.5877	1.09E-05	hypomethylated
cg14113958	0.684	0.45512	-0.587749	0.000306	hypomethylated
cg00479463	0.67058	0.446124324	-0.587964	0.00043	hypomethylated
cg04396112	0.7408	0.492837297	-0.587973	0.000148	hypomethylated
cg26396492	0.45571	0.303135676	-0.588152	0.00101	hypomethylated
cg01652514	0.66049	0.439272432	-0.588421	0.000138	hypomethylated
cg17333042	0.48788	0.324458378	-0.588493	9.95E-05	hypomethylated
cg25466245	0.50521	0.335955676	-0.588612	0.000107	hypomethylated
cg00211115	0.50183	0.333642703	-0.588895	0.000306	hypomethylated
cg22878582	0.7737	0.514389189	-0.588914	5.00E-06	hypomethylated
cg13652008	0.64977	0.431975676	-0.588979	0.00032	hypomethylated
cg08498833	0.69477	0.461889189	-0.588989	3.95E-05	hypomethylated
cg21370522	0.57992	0.385516216	-0.589062	0.010555	hypomethylated
cg02766259	0.8988	0.597411351	-0.589275	1.12E-05	hypomethylated
cg23407151	0.65934	0.438206486	-0.589412	1.80E-05	hypomethylated
cg00919591	0.5557	0.369291351	-0.589547	0.000725	hypomethylated
cg11001085	0.85092	0.565479459	-0.589549	1.93E-06	hypomethylated
cg16580616	0.66077	0.439042162	-0.589789	0.000115	hypomethylated
cg15497834	0.51066	0.339301081	-0.589797	0.001423	hypomethylated
cg20000107	0.58036	0.385595676	-0.589859	0.000209	hypomethylated
cg11386011	0.61221	0.406742162	-0.589912	2.57E-05	hypomethylated
cg23270841	0.59291	0.393891892	-0.590013	8.84E-05	hypomethylated
cg20041612	0.74831	0.497126486	-0.590023	3.12E-06	hypomethylated
cg01385669	0.7851	0.521561622	-0.590039	4.07E-06	hypomethylated
cg06709297	0.86059	0.571672432	-0.590137	2.29E-06	hypomethylated
cg11012153	0.78922	0.524172973	-0.590385	7.12E-05	hypomethylated
cg02144874	0.74571	0.495265405	-0.590413	5.72E-05	hypomethylated
cg10097313	0.75768	0.503214595	-0.590415	2.71E-05	hypomethylated
cg13401301	0.44998	0.298844865	-0.590464	0.006796	hypomethylated
cg21938894	0.66865	0.444043243	-0.590551	3.32E-05	hypomethylated
cg10438282	0.63431	0.421137297	-0.590897	1.37E-06	hypomethylated
cg15029037	0.82123	0.545218378	-0.590952	1.80E-05	hypomethylated
cg00066270	0.61413	0.407614595	-0.591338	3.44E-05	hypomethylated
cg23670794	0.85234	0.565717838	-0.591346	9.82E-06	hypomethylated
cg09262230	0.61635	0.409078378	-0.591373	3.95E-05	hypomethylated
cg04188920	0.72794	0.483132432	-0.591401	8.52E-05	hypomethylated
cg14528056	0.85303	0.566139459	-0.591439	2.57E-05	hypomethylated
cg26579550	0.62735	0.416318919	-0.591581	0.00028	hypomethylated
cg20237595	0.53992	0.358262703	-0.591728	0.005508	hypomethylated
cg13461178	0.47846	0.317466486	-0.591794	0.00048	hypomethylated
cg17650028	0.70417	0.46722	-0.591822	5.43E-06	hypomethylated
cg03125765	0.44783	0.297136216	-0.591827	0.000262	hypomethylated
cg01604946	0.5762	0.382238378	-0.592097	2.27E-05	hypomethylated
cg20991420	0.62843	0.416883243	-0.592109	0.000232	hypomethylated
cg18325192	0.6653	0.441321622	-0.592175	5.45E-05	hypomethylated
cg04647835	0.79413	0.526710811	-0.592364	6.66E-06	hypomethylated
cg04064032	0.89246	0.591923243	-0.592377	3.08E-06	hypomethylated
cg04212846	0.67465	0.447410811	-0.592539	3.76E-05	hypomethylated
cg07199862	0.73315	0.486176757	-0.592627	6.05E-06	hypomethylated
cg12885484	0.82731	0.548617297	-0.592628	3.85E-05	hypomethylated
cg25769469	0.78195	0.51853027	-0.592648	0.000374	hypomethylated
cg07697770	0.6171	0.409204865	-0.592681	2.07E-05	hypomethylated
cg26000536	0.80136	0.531384324	-0.592695	1.15E-06	hypomethylated
cg17213154	0.54719	0.362802703	-0.592857	2.51E-05	hypomethylated
cg27608102	0.60212	0.399207027	-0.592914	3.23E-05	hypomethylated
cg27022827	0.67136	0.445082703	-0.593013	1.01E-05	hypomethylated

cg23338993	0.72099	0.477984324	-0.593016	9.96E-06	hypomethylated
cg14029912	0.65823	0.436302703	-0.593262	3.53E-05	hypomethylated
cg09291474	0.71467	0.473711351	-0.593269	1.58E-06	hypomethylated
cg23689428	0.56911	0.377220541	-0.593299	0.00097	hypomethylated
cg14066757	0.39175	0.259656216	-0.593331	5.38E-05	hypomethylated
cg05129081	0.49798	0.330059459	-0.593362	2.92E-05	hypomethylated
cg13226232	0.86196	0.571265946	-0.593458	1.04E-05	hypomethylated
cg05004855	0.3575	0.236922162	-0.59353	0.000306	hypomethylated
cg07424400	0.72799	0.482436216	-0.59358	1.93E-06	hypomethylated
cg08140114	0.54935	0.364042703	-0.593618	4.58E-05	hypomethylated
cg06468454	0.41399	0.274325405	-0.593708	0.000274	hypomethylated
cg22412747	0.60698	0.402186486	-0.593784	7.84E-05	hypomethylated
cg15688324	0.81221	0.538162162	-0.593812	9.82E-06	hypomethylated
cg06495586	0.69515	0.460571892	-0.593898	0.000142	hypomethylated
cg14837598	0.65657	0.434964324	-0.594052	2.51E-05	hypomethylated
cg23386895	0.66037	0.437446486	-0.594168	0.000123	hypomethylated
cg11888982	0.77959	0.516359459	-0.59434	0.00035	hypomethylated
cg26165286	0.55582	0.368143243	-0.594351	0.000588	hypomethylated
cg11095099	0.7264	0.481115135	-0.594382	1.88E-06	hypomethylated
cg08854834	0.49683	0.329060541	-0.594399	0.000204	hypomethylated
cg05673966	0.71516	0.473635676	-0.594488	7.12E-06	hypomethylated
cg10104290	0.44748	0.29634	-0.59457	0.000474	hypomethylated
cg09551613	0.28993	0.192001081	-0.59459	0.001096	hypomethylated
cg00025357	0.35211	0.233175135	-0.594612	0.00151	hypomethylated
cg13120756	0.77364	0.512305405	-0.594658	1.29E-06	hypomethylated
cg00221794	0.62048	0.410812973	-0.594903	0.002274	hypomethylated
cg17079034	0.39644	0.262476216	-0.594916	0.000449	hypomethylated
cg09247695	0.51695	0.342254054	-0.594957	0.000551	hypomethylated
cg23581088	0.62839	0.416012973	-0.595032	3.15E-05	hypomethylated
cg27002325	0.88891	0.588477838	-0.595049	3.79E-06	hypomethylated
cg10334750	0.81373	0.538662162	-0.595169	6.62E-05	hypomethylated
cg06627532	0.63452	0.419944865	-0.595466	2.81E-05	hypomethylated
cg10954944	0.68576	0.453827027	-0.595561	6.22E-06	hypomethylated
cg14030904	0.64991	0.430042703	-0.59576	0.000342	hypomethylated
cg21402096	0.73826	0.488449189	-0.59592	2.15E-05	hypomethylated
cg19051042	0.77506	0.51278	-0.595968	6.93E-06	hypomethylated
cg08418670	0.80511	0.532579459	-0.596189	4.94E-05	hypomethylated
cg20152088	0.58107	0.384365946	-0.596231	2.42E-06	hypomethylated
cg03211098	0.49219	0.32557027	-0.596246	6.38E-05	hypomethylated
cg20427318	0.80618	0.533261622	-0.596258	4.36E-05	hypomethylated
cg24896460	0.75945	0.502345946	-0.596274	8.83E-06	hypomethylated
cg14787880	0.71359	0.472001622	-0.596304	3.85E-05	hypomethylated
cg10601624	0.49997	0.330692973	-0.596349	3.23E-05	hypomethylated
cg16966139	0.43775	0.289458919	-0.596749	2.32E-05	hypomethylated
cg04881228	0.37407	0.247348649	-0.596762	4.82E-05	hypomethylated
cg25463742	0.7555	0.49955027	-0.596802	0.000123	hypomethylated
cg05407338	0.55349	0.365965946	-0.596848	0.00024	hypomethylated
cg01311222	0.73868	0.488363243	-0.596995	9.05E-05	hypomethylated
cg01662869	0.59434	0.392935676	-0.596995	8.42E-05	hypomethylated
cg22688802	0.88243	0.583390811	-0.597019	9.31E-06	hypomethylated
cg05492904	0.72062	0.476400541	-0.597064	6.22E-06	hypomethylated
cg09010707	0.47565	0.314442162	-0.597106	0.000408	hypomethylated
cg19727499	0.66723	0.441077297	-0.597153	4.82E-05	hypomethylated
cg13023014	0.56833	0.375696757	-0.59716	1.92E-05	hypomethylated
cg24107163	0.58731	0.388218378	-0.597254	0.001395	hypomethylated
cg13015534	0.68401	0.452101081	-0.597372	0.000187	hypomethylated
cg17272838	0.73958	0.488810811	-0.59743	5.89E-06	hypomethylated
cg02047661	0.62126	0.410581622	-0.597528	5.43E-06	hypomethylated

cg00305071	0.42306	0.279574054	-0.597632	0.000797	hypomethylated
cg24031764	0.76972	0.508594054	-0.597819	1.84E-05	hypomethylated
cg09093656	0.62138	0.410576216	-0.597826	0.000756	hypomethylated
cg12403162	0.5697	0.376423243	-0.597847	0.00134	hypomethylated
cg04864538	0.70198	0.463798378	-0.597932	9.44E-06	hypomethylated
cg06458557	0.63991	0.422738919	-0.598102	5.86E-05	hypomethylated
cg01963702	0.90413	0.597267027	-0.598154	1.67E-06	hypomethylated
cg14454094	0.87429	0.577543784	-0.598182	1.43E-06	hypomethylated
cg07010146	0.77439	0.51150973	-0.598299	1.93E-06	hypomethylated
cg04540882	0.67158	0.443571892	-0.598391	8.15E-06	hypomethylated
cg20651995	0.45306	0.299206486	-0.598561	8.63E-05	hypomethylated
cg16564525	0.39193	0.258835135	-0.598563	3.59E-06	hypomethylated
cg20893936	0.71634	0.473073514	-0.59858	0.000116	hypomethylated
cg26130669	0.80922	0.534365405	-0.598705	2.32E-05	hypomethylated
cg27027375	0.74332	0.490832973	-0.598751	1.25E-05	hypomethylated
cg11385938	0.85151	0.562258919	-0.598789	4.94E-05	hypomethylated
cg15835664	0.81861	0.540525946	-0.598812	6.05E-06	hypomethylated
cg22208536	0.36473	0.240828108	-0.598825	4.82E-05	hypomethylated
cg22270364	0.74434	0.491479459	-0.598831	1.99E-05	hypomethylated
cg06805925	0.56808	0.375094054	-0.598842	0.000256	hypomethylated
cg00092383	0.93551	0.617597838	-0.599085	6.57E-06	hypomethylated
cg14905634	0.71725	0.473470811	-0.599201	0.000159	hypomethylated
cg13687274	0.87899	0.580231351	-0.599219	2.49E-06	hypomethylated
cg02660643	0.66409	0.438334054	-0.599348	4.15E-05	hypomethylated
cg24429533	0.56809	0.374967568	-0.599354	1.12E-06	hypomethylated
cg26856289	0.73341	0.484074595	-0.599391	0.000741	hypomethylated
cg25936381	0.833	0.549754054	-0.59953	2.95E-06	hypomethylated
cg08500112	0.62523	0.412595676	-0.599658	0.001526	hypomethylated
cg18400079	0.79663	0.525645405	-0.59982	0.000104	hypomethylated
cg01621390	0.6688	0.441295135	-0.599831	1.89E-05	hypomethylated
cg18452973	0.49347	0.325597838	-0.599871	9.05E-05	hypomethylated
cg06748147	0.29165	0.192430811	-0.599898	0.001118	hypomethylated
cg05462761	0.47092	0.310686486	-0.600023	0.003286	hypomethylated
cg23010507	0.69781	0.46036973	-0.600041	0.000187	hypomethylated
cg14140379	0.78681	0.519063784	-0.600103	1.40E-05	hypomethylated
cg11737941	0.54801	0.361379459	-0.600688	0.001619	hypomethylated
cg25626472	0.74996	0.494552973	-0.600689	1.46E-05	hypomethylated
cg20077028	0.71265	0.469929189	-0.60075	0.000191	hypomethylated
cg02010852	0.56797	0.374505405	-0.600828	0.000129	hypomethylated
cg14332120	0.72468	0.477812432	-0.6009	1.12E-05	hypomethylated
cg20000220	0.9127	0.60172	-0.601048	2.42E-06	hypomethylated
cg18376773	0.61772	0.407192432	-0.601242	9.95E-05	hypomethylated
cg19301114	0.49289	0.324904865	-0.601248	0.001288	hypomethylated
cg21560697	0.84106	0.554365946	-0.60137	9.31E-06	hypomethylated
cg04793096	0.63772	0.420315135	-0.601452	0.000435	hypomethylated
cg15128679	0.58812	0.387617297	-0.601478	0.000159	hypomethylated
cg09893465	0.80713	0.531932973	-0.601557	0.000733	hypomethylated
cg00039489	0.62992	0.415114595	-0.601659	0.000166	hypomethylated
cg16587838	0.33178	0.218632973	-0.601716	0.000749	hypomethylated
cg19627034	0.48068	0.316739459	-0.60178	0.003441	hypomethylated
cg13146484	0.38614	0.254407568	-0.601982	4.10E-05	hypomethylated
cg13362546	0.66332	0.437012432	-0.602031	6.57E-06	hypomethylated
cg12554573	0.71437	0.47064	-0.602048	2.10E-06	hypomethylated
cg26673980	0.59487	0.391905946	-0.602067	0.000138	hypomethylated
cg26489497	0.48961	0.322518919	-0.602249	8.13E-05	hypomethylated
cg20025658	0.73764	0.485772432	-0.602636	0.000204	hypomethylated
cg15580052	0.78876	0.519436757	-0.602638	2.15E-05	hypomethylated
cg24686551	0.74513	0.490674054	-0.602727	0.000369	hypomethylated

cg16608498	0.59079	0.389031351	-0.602759	0.000875	hypomethylated
cg08642285	0.56005	0.368776757	-0.602808	0.000148	hypomethylated
cg13747967	0.62012	0.408311351	-0.602878	0.001839	hypomethylated
cg12281620	0.65436	0.430838378	-0.602938	1.18E-05	hypomethylated
cg08994082	0.79814	0.525501081	-0.602948	1.15E-05	hypomethylated
cg06873343	0.77425	0.509745405	-0.603023	0.00017	hypomethylated
cg16421411	0.90793	0.597688108	-0.603188	2.83E-06	hypomethylated
cg11378101	0.77384	0.509404324	-0.603224	1.04E-05	hypomethylated
cg13393327	0.76212	0.501642162	-0.60336	2.78E-05	hypomethylated
cg07469815	0.75986	0.500137838	-0.603408	2.85E-05	hypomethylated
cg18808929	0.57308	0.377192973	-0.603434	0.000517	hypomethylated
cg11531272	0.56783	0.373728108	-0.60347	0.001768	hypomethylated
cg19286437	0.66586	0.438223243	-0.603553	1.37E-05	hypomethylated
cg27601906	0.67751	0.445872432	-0.603611	3.57E-05	hypomethylated
cg11957248	0.63474	0.417714595	-0.603648	8.26E-06	hypomethylated
cg27583815	0.81443	0.535909189	-0.603802	4.88E-07	hypomethylated
cg10649130	0.77567	0.510367027	-0.603908	3.76E-05	hypomethylated
cg15135286	0.61735	0.406184865	-0.603952	0.000142	hypomethylated
cg02591564	0.86212	0.567172432	-0.604101	1.87E-05	hypomethylated
cg07235218	0.44272	0.291229189	-0.60424	0.001541	hypomethylated
cg18170229	0.50358	0.331237297	-0.604356	0.000122	hypomethylated
cg01281040	0.91575	0.602338919	-0.604378	3.21E-06	hypomethylated
cg00517261	0.43864	0.288496216	-0.604485	0.000214	hypomethylated
cg24296761	0.77353	0.508654595	-0.604771	1.75E-05	hypomethylated
cg05256179	0.79565	0.523170811	-0.604852	5.03E-07	hypomethylated
cg14398113	0.57646	0.379037838	-0.604879	0.004691	hypomethylated
cg23898497	0.49155	0.323194595	-0.604935	0.00017	hypomethylated
cg09678939	0.6613	0.4348	-0.604953	0.000875	hypomethylated
cg21734707	0.31329	0.205984324	-0.604964	0.001452	hypomethylated
cg26737223	0.71203	0.468131892	-0.605023	0.000109	hypomethylated
cg14753094	0.85174	0.559918378	-0.605197	8.83E-06	hypomethylated
cg21721681	0.48259	0.317245405	-0.605199	0.000464	hypomethylated
cg16429927	0.29901	0.196555135	-0.60526	3.00E-05	hypomethylated
cg09171112	0.69473	0.456682162	-0.605262	8.42E-05	hypomethylated
cg02566391	0.66283	0.435707027	-0.605281	0.000435	hypomethylated
cg20738274	0.64195	0.421974595	-0.605305	6.84E-06	hypomethylated
cg07999953	0.8312	0.546272973	-0.605574	3.90E-06	hypomethylated
cg16450097	0.81334	0.534503784	-0.605658	4.26E-05	hypomethylated
cg25749254	0.63596	0.417901081	-0.605775	7.47E-05	hypomethylated
cg10009968	0.9451	0.621017297	-0.605834	3.54E-06	hypomethylated
cg07528209	0.78928	0.518601622	-0.60591	1.71E-05	hypomethylated
cg23807924	0.65359	0.429428649	-0.605967	9.05E-05	hypomethylated
cg16609966	0.69797	0.458583784	-0.60598	1.80E-05	hypomethylated
cg21605781	0.44224	0.290561622	-0.605985	4.47E-05	hypomethylated
cg10310616	0.79668	0.523430811	-0.606002	1.06E-05	hypomethylated
cg15172966	0.50313	0.330547027	-0.606076	0.000417	hypomethylated
cg26298737	0.57092	0.375082703	-0.60608	0.000557	hypomethylated
cg06827256	0.80087	0.526154054	-0.606083	1.01E-05	hypomethylated
cg00275828	0.63235	0.415427568	-0.606126	0.000293	hypomethylated
cg09939948	0.26509	0.174113514	-0.606454	0.000209	hypomethylated
cg09406107	0.3323	0.218232973	-0.606617	0.000831	hypomethylated
cg07451762	0.57702	0.378942162	-0.606644	0.00012	hypomethylated
cg01455178	0.76314	0.501105946	-0.606832	3.79E-06	hypomethylated
cg18787437	0.58177	0.381941081	-0.607099	0.000408	hypomethylated
cg21450381	0.76756	0.503843784	-0.607303	1.54E-05	hypomethylated
cg16773067	0.53025	0.348056757	-0.60735	6.46E-05	hypomethylated
cg02893550	0.55767	0.36605027	-0.60737	0.000134	hypomethylated
cg23258611	0.59246	0.388881081	-0.607389	0.000135	hypomethylated

cg00220225	0.70782	0.464596757	-0.607403	4.82E-05	hypomethylated
cg03652336	0.63796	0.418732973	-0.607435	0.005911	hypomethylated
cg19319037	0.68744	0.451168649	-0.607567	0.003773	hypomethylated
cg12068124	0.41111	0.269798919	-0.60764	0.007799	hypomethylated
cg20170271	0.53257	0.349503243	-0.607665	0.00099	hypomethylated
cg11938718	0.66943	0.439300541	-0.607725	2.64E-05	hypomethylated
cg06186245	0.55872	0.366633514	-0.607787	0.000195	hypomethylated
cg18497238	0.48371	0.317374054	-0.607958	0.000607	hypomethylated
cg19049754	0.76996	0.505114595	-0.608173	4.15E-05	hypomethylated
cg13050357	0.76884	0.504299459	-0.608403	2.79E-06	hypomethylated
cg20292636	0.55105	0.361431892	-0.608459	2.71E-05	hypomethylated
cg06521827	0.55388	0.363281622	-0.608485	7.47E-05	hypomethylated
cg10188797	0.67337	0.44165027	-0.608495	2.15E-05	hypomethylated
cg12865818	0.78113	0.512315135	-0.608531	8.34E-07	hypomethylated
cg16978398	0.84375	0.55336973	-0.608572	3.04E-06	hypomethylated
cg03978041	0.89415	0.586396757	-0.60864	1.18E-06	hypomethylated
cg06513247	0.34229	0.224475676	-0.60866	0.000517	hypomethylated
cg00886780	0.41637	0.273026486	-0.608825	8.84E-05	hypomethylated
cg00974523	0.63377	0.415542162	-0.608965	2.13E-05	hypomethylated
cg12358050	0.53204	0.348818378	-0.609059	7.32E-06	hypomethylated
cg23976431	0.3973	0.26046	-0.609167	0.000214	hypomethylated
cg04363470	0.61214	0.401285946	-0.609231	1.18E-05	hypomethylated
cg25025992	0.71756	0.470337838	-0.609402	3.72E-07	hypomethylated
cg12711760	0.3101	0.203247568	-0.609495	4.47E-05	hypomethylated
cg01617071	0.66769	0.437588108	-0.609605	4.94E-05	hypomethylated
cg02238461	0.49871	0.326821622	-0.609698	0.002938	hypomethylated
cg04819580	0.42185	0.276438919	-0.609769	0.000129	hypomethylated
cg17319198	0.68078	0.446105405	-0.609804	1.31E-05	hypomethylated
cg00134295	0.59087	0.387184865	-0.609818	0.007035	hypomethylated
cg26457700	0.78106	0.511811351	-0.609821	4.01E-06	hypomethylated
cg04362790	0.90617	0.593773514	-0.609869	4.82E-05	hypomethylated
cg25772221	0.48839	0.320018919	-0.609876	0.003705	hypomethylated
cg19377250	0.77115	0.505287027	-0.609908	7.93E-06	hypomethylated
cg03958308	0.56531	0.370400541	-0.609956	1.84E-05	hypomethylated
cg13445177	0.69372	0.454533514	-0.609967	3.49E-05	hypomethylated
cg15250073	0.48538	0.317984324	-0.610159	0.0006	hypomethylated
cg21547763	0.8354	0.547258919	-0.610244	1.72E-06	hypomethylated
cg03654169	0.70009	0.458592973	-0.610326	3.79E-06	hypomethylated
cg20127859	0.59818	0.39181027	-0.610424	5.58E-05	hypomethylated
cg10854819	0.7254	0.475137297	-0.610432	0.000313	hypomethylated
cg19654743	0.49021	0.321068649	-0.610518	2.21E-05	hypomethylated
cg15899738	0.86471	0.566295676	-0.610661	1.21E-05	hypomethylated
cg11542165	0.88039	0.576521081	-0.610769	7.32E-06	hypomethylated
cg14757107	0.82108	0.537663243	-0.61082	3.59E-06	hypomethylated
cg22202381	0.76621	0.50166973	-0.611002	2.45E-05	hypomethylated
cg13488011	0.7325	0.479572432	-0.61108	6.62E-05	hypomethylated
cg12441694	0.73439	0.480797297	-0.611118	3.59E-06	hypomethylated
cg26474043	0.54771	0.358541622	-0.611272	0.000154	hypomethylated
cg23664708	0.41617	0.272354595	-0.611687	0.00028	hypomethylated
cg02532341	0.52186	0.341521081	-0.611688	0.003021	hypomethylated
cg19238349	0.48492	0.317340541	-0.611715	8.22E-05	hypomethylated
cg16650073	0.70719	0.462737297	-0.611904	3.15E-05	hypomethylated
cg06799735	0.67665	0.442683784	-0.612133	2.10E-05	hypomethylated
cg22198603	0.67922	0.444354595	-0.612168	7.47E-05	hypomethylated
cg14241894	0.84512	0.55286	-0.612242	5.58E-05	hypomethylated
cg07774251	0.73861	0.483172973	-0.612273	4.70E-05	hypomethylated
cg01703780	0.7171	0.469038919	-0.612467	7.42E-06	hypomethylated
cg14966613	0.82978	0.542732432	-0.612488	2.16E-06	hypomethylated

cg18270378	0.83682	0.547275676	-0.61265	1.66E-05	hypomethylated
cg00101629	0.62603	0.409395676	-0.612736	0.000313	hypomethylated
cg27051318	0.68637	0.448853514	-0.612742	0.000342	hypomethylated
cg14396214	0.46319	0.302877838	-0.612868	0.0003	hypomethylated
cg03499675	0.6539	0.427566486	-0.612921	9.82E-06	hypomethylated
cg23068558	0.84272	0.550950811	-0.61313	0.00017	hypomethylated
cg03585053	0.73152	0.478235135	-0.613177	4.12E-06	hypomethylated
cg10472651	0.65427	0.427705405	-0.613269	0.00018	hypomethylated
cg26283496	0.77685	0.507804865	-0.613362	0.000107	hypomethylated
cg12011522	0.53956	0.352635676	-0.613605	5.06E-05	hypomethylated
cg24788090	0.76579	0.500454595	-0.61371	1.52E-05	hypomethylated
cg02579140	0.9104	0.594899459	-0.613855	5.19E-05	hypomethylated
cg17552357	0.86907	0.567869189	-0.613914	1.39E-05	hypomethylated
cg17333973	0.39643	0.258995676	-0.614138	0.000445	hypomethylated
cg19473623	0.74927	0.489511351	-0.614143	2.27E-05	hypomethylated
cg26826183	0.68951	0.450467027	-0.61415	5.28E-06	hypomethylated
cg19640821	0.6677	0.436203784	-0.614198	0.000613	hypomethylated
cg05416223	0.75692	0.494400541	-0.614461	5.43E-06	hypomethylated
cg23249922	0.65994	0.431037297	-0.614522	0.000374	hypomethylated
cg16387532	0.50819	0.331922162	-0.614523	0.001603	hypomethylated
cg16497293	0.76803	0.501596757	-0.614635	5.07E-06	hypomethylated
cg19132462	0.63217	0.412818378	-0.614805	0.000187	hypomethylated
cg10022788	0.59211	0.386641622	-0.614868	0.00028	hypomethylated
cg21642988	0.80193	0.523585405	-0.615051	5.43E-06	hypomethylated
cg14825555	0.57752	0.377022162	-0.615222	2.32E-05	hypomethylated
cg27661394	0.78585	0.513022162	-0.615233	1.50E-05	hypomethylated
cg26651303	0.67398	0.439982162	-0.615261	0.000474	hypomethylated
cg13269555	0.66969	0.437140541	-0.615396	6.70E-05	hypomethylated
cg24968629	0.93814	0.612346486	-0.615455	9.07E-06	hypomethylated
cg00478421	0.50254	0.328018378	-0.615462	1.44E-05	hypomethylated
cg18729298	0.52912	0.345324324	-0.615643	0.001327	hypomethylated
cg11790673	0.46236	0.301745405	-0.615685	8.60E-06	hypomethylated
cg07306253	0.66737	0.435384324	-0.616197	7.12E-05	hypomethylated
cg06762457	0.51374	0.335134595	-0.616298	4.70E-05	hypomethylated
cg04555780	0.81508	0.531707027	-0.61631	8.71E-06	hypomethylated
cg11639815	0.7573	0.494001622	-0.616349	0.00044	hypomethylated
cg17756105	0.67987	0.443461622	-0.61645	1.66E-05	hypomethylated
cg09633081	0.46901	0.30592	-0.616464	3.32E-05	hypomethylated
cg03226203	0.681	0.444096757	-0.616781	2.05E-06	hypomethylated
cg06738887	0.4937	0.321938378	-0.61685	0.000681	hypomethylated
cg05762671	0.67499	0.440099459	-0.617037	9.05E-05	hypomethylated
cg20947259	0.77953	0.508255135	-0.617052	2.13E-05	hypomethylated
cg23051926	0.59157	0.385699459	-0.617072	3.76E-05	hypomethylated
cg02196805	0.72797	0.47463027	-0.617075	1.41E-06	hypomethylated
cg18773597	0.6677	0.435320541	-0.617122	0.001912	hypomethylated
cg10620680	0.6319	0.411945405	-0.617243	6.31E-05	hypomethylated
cg20413202	0.83281	0.542723784	-0.617769	3.07E-05	hypomethylated
cg15326755	0.96926	0.631604324	-0.617863	2.42E-06	hypomethylated
cg11963006	0.52884	0.344600541	-0.617906	0.0006	hypomethylated
cg27499185	0.41684	0.271609189	-0.617961	9.49E-05	hypomethylated
cg09477895	0.7138	0.465075135	-0.618056	2.57E-05	hypomethylated
cg07615493	0.75177	0.489765946	-0.618199	3.49E-05	hypomethylated
cg17115419	0.81316	0.529718378	-0.618314	3.00E-05	hypomethylated
cg27523087	0.50632	0.329817297	-0.618382	0.000495	hypomethylated
cg24143287	0.87005	0.566696757	-0.618521	1.39E-06	hypomethylated
cg00339913	0.54997	0.358209189	-0.618551	6.78E-05	hypomethylated
cg02987249	0.71642	0.466607027	-0.618598	6.84E-06	hypomethylated
cg22104744	0.4906	0.319513514	-0.61867	0.000569	hypomethylated

cg24527751	0.85536	0.557068108	-0.618678	7.12E-06	hypomethylated
cg14439774	0.63296	0.412180541	-0.618838	2.15E-05	hypomethylated
cg05372727	0.85583	0.557276757	-0.61893	2.16E-06	hypomethylated
cg10714639	0.54977	0.357969189	-0.618993	0.002455	hypomethylated
cg03109660	0.73284	0.477144865	-0.619071	0.0002	hypomethylated
cg15694987	0.67293	0.43810973	-0.619164	1.42E-05	hypomethylated
cg24587185	0.46319	0.301537297	-0.619268	0.000875	hypomethylated
cg01284289	0.57462	0.373981622	-0.619641	0.000511	hypomethylated
cg05220092	0.44826	0.291731351	-0.619695	0.001511	hypomethylated
cg14659814	0.73385	0.477544324	-0.619851	8.83E-06	hypomethylated
cg22054191	0.39918	0.259742703	-0.619956	0.002066	hypomethylated
cg14165142	0.69675	0.453321081	-0.620108	0.000145	hypomethylated
cg23792934	0.4291	0.279127568	-0.620389	1.06E-05	hypomethylated
cg01099024	0.64212	0.417685946	-0.620424	5.58E-06	hypomethylated
cg11818555	0.88671	0.576729189	-0.620568	2.71E-06	hypomethylated
cg25205321	0.59688	0.388201622	-0.620635	9.72E-05	hypomethylated
cg11668419	0.69939	0.454872432	-0.620635	4.48E-06	hypomethylated
cg23889010	0.54667	0.35550973	-0.620781	0.000342	hypomethylated
cg22453826	0.85887	0.558467568	-0.620966	2.32E-05	hypomethylated
cg06815976	0.39528	0.257	-0.621107	0.000417	hypomethylated
cg03661110	0.68691	0.446573514	-0.621223	0.000152	hypomethylated
cg00537837	0.76143	0.495005946	-0.621266	2.45E-05	hypomethylated
cg11597902	0.77026	0.500745946	-0.621267	0.000245	hypomethylated
cg10450692	0.54121	0.351836757	-0.621282	0.00096	hypomethylated
cg23645885	0.30508	0.19832	-0.621357	0.00255	hypomethylated
cg24809011	0.68062	0.442438919	-0.621371	1.06E-05	hypomethylated
cg08302480	0.60721	0.394697297	-0.621449	0.000528	hypomethylated
cg10777887	0.54039	0.35124973	-0.621504	0.037559	hypomethylated
cg17560015	0.55544	0.361019459	-0.621554	0.002993	hypomethylated
cg17128799	0.71258	0.463127568	-0.621642	6.01E-05	hypomethylated
cg22434923	0.54277	0.352756757	-0.621667	0.001511	hypomethylated
cg05979170	0.77712	0.505022162	-0.621791	1.31E-05	hypomethylated
cg17245726	0.80355	0.522126486	-0.621988	3.95E-05	hypomethylated
cg04618171	0.87518	0.568647027	-0.622046	4.86E-06	hypomethylated
cg14148156	0.77293	0.502192432	-0.622097	9.44E-06	hypomethylated
cg08422793	0.53085	0.344902162	-0.622117	0.00623	hypomethylated
cg23518541	0.56294	0.365741622	-0.622156	0.0002	hypomethylated
cg17150651	0.4833	0.313965405	-0.622313	0.002026	hypomethylated
cg20697025	0.62786	0.407864865	-0.622352	0.002803	hypomethylated
cg08380391	0.6222	0.404171892	-0.622409	2.87E-06	hypomethylated
cg02947498	0.77115	0.500862162	-0.622598	3.79E-06	hypomethylated
cg23667602	0.63974	0.41547027	-0.62274	2.21E-05	hypomethylated
cg00674220	0.74039	0.480815135	-0.622803	2.81E-05	hypomethylated
cg22734236	0.51417	0.333901081	-0.622825	0.000374	hypomethylated
cg25982657	0.50971	0.330972973	-0.622963	0.000382	hypomethylated
cg26712743	0.69181	0.449215676	-0.622968	1.84E-05	hypomethylated
cg14533523	0.71665	0.46534	-0.622983	4.82E-05	hypomethylated
cg16704703	0.55593	0.360979459	-0.622986	0.000374	hypomethylated
cg15816503	0.6275	0.407429189	-0.623066	8.63E-05	hypomethylated
cg14643264	0.70765	0.459450811	-0.623126	0.000132	hypomethylated
cg02185007	0.66955	0.434661081	-0.623301	0.000147	hypomethylated
cg06547285	0.67364	0.437267568	-0.623461	5.79E-05	hypomethylated
cg11911769	0.77045	0.500077838	-0.623549	1.42E-05	hypomethylated
cg02713162	0.79601	0.516623784	-0.623672	0.000129	hypomethylated
cg13389508	0.56158	0.364425405	-0.623868	1.94E-05	hypomethylated
cg10279922	0.66446	0.431175676	-0.623907	4.31E-05	hypomethylated
cg12184624	0.76648	0.497221622	-0.624359	8.83E-06	hypomethylated
cg01692279	0.38109	0.247170811	-0.624623	0.002965	hypomethylated

cg27596415	0.43878	0.284539459	-0.624869	0.005223	hypomethylated
cg09723635	0.81804	0.530438919	-0.624985	9.82E-06	hypomethylated
cg26719625	0.71562	0.463955676	-0.625207	4.64E-05	hypomethylated
cg00019654	0.64612	0.418872432	-0.625291	0.000229	hypomethylated
cg21814178	0.51656	0.334872973	-0.625322	0.002189	hypomethylated
cg19106968	0.78404	0.508222703	-0.625466	1.80E-05	hypomethylated
cg05360714	0.60058	0.389301081	-0.62547	0.000163	hypomethylated
cg14918359	0.65843	0.42676973	-0.625572	6.62E-05	hypomethylated
cg24492058	0.40975	0.265582162	-0.625586	0.000449	hypomethylated
cg07745671	0.79579	0.515757297	-0.625695	0.000207	hypomethylated
cg00305740	0.78142	0.506437838	-0.625713	6.01E-05	hypomethylated
cg21175685	0.71043	0.460338919	-0.625996	1.17E-05	hypomethylated
cg27290215	0.48473	0.314071892	-0.626087	4.47E-05	hypomethylated
cg02372702	0.43319	0.280660541	-0.626174	0.000797	hypomethylated
cg05761882	0.46532	0.301474595	-0.626187	4.94E-05	hypomethylated
cg01194336	0.72691	0.470863243	-0.626469	1.71E-05	hypomethylated
cg00086488	0.82705	0.535725405	-0.626481	6.57E-06	hypomethylated
cg11902863	0.50636	0.327989189	-0.626515	0.000931	hypomethylated
cg13134297	0.68875	0.446102162	-0.626606	0.00054	hypomethylated
cg02152631	0.61559	0.398693514	-0.62669	9.72E-05	hypomethylated
cg20636248	0.70617	0.45730973	-0.626844	8.84E-05	hypomethylated
cg16339434	0.55249	0.357687027	-0.627251	0.001262	hypomethylated
cg21579399	0.59394	0.384505946	-0.627311	0.001839	hypomethylated
cg15269394	0.61218	0.396301622	-0.627357	0.00032	hypomethylated
cg16558770	0.72169	0.467193514	-0.627359	0.00017	hypomethylated
cg01607932	0.80259	0.519519459	-0.627485	2.85E-05	hypomethylated
cg03611487	0.49269	0.318861081	-0.627752	0.000575	hypomethylated
cg13773247	0.65751	0.42548	-0.627922	0.00012	hypomethylated
cg17619020	0.41422	0.268035135	-0.627975	0.000741	hypomethylated
cg20450123	0.51211	0.331347568	-0.628108	0.003021	hypomethylated
cg17278453	0.32978	0.213364865	-0.628181	0.002803	hypomethylated
cg07103618	0.58053	0.375581622	-0.628244	7.47E-05	hypomethylated
cg23043143	0.39611	0.256258919	-0.628299	0.0003	hypomethylated
cg09502149	0.53096	0.343497838	-0.628302	5.06E-05	hypomethylated
cg21766667	0.48357	0.31280973	-0.628439	1.46E-05	hypomethylated
cg17220055	0.55499	0.358965946	-0.628615	0.000195	hypomethylated
cg10143731	0.73919	0.478104865	-0.628618	4.80E-06	hypomethylated
cg03727673	0.62462	0.403996216	-0.628637	1.25E-05	hypomethylated
cg23950233	0.59876	0.387263243	-0.628663	0.004287	hypomethylated
cg15626405	0.55681	0.360107027	-0.628759	1.04E-05	hypomethylated
cg02394249	0.68473	0.442761081	-0.629007	1.80E-05	hypomethylated
cg09232358	0.7201	0.46563027	-0.629012	3.40E-05	hypomethylated
cg11586448	0.39855	0.257708649	-0.62902	0.000575	hypomethylated
cg05646575	0.73978	0.478205405	-0.629466	9.57E-06	hypomethylated
cg02698806	0.42864	0.277071351	-0.629509	0.009713	hypomethylated
cg11130630	0.68732	0.444204324	-0.629759	0.000857	hypomethylated
cg04379703	0.56116	0.362622703	-0.629943	0.000454	hypomethylated
cg21323106	0.83511	0.539591892	-0.630098	3.59E-06	hypomethylated
cg01189072	0.87435	0.564891351	-0.630237	6.46E-05	hypomethylated
cg07243202	0.60608	0.391545405	-0.630329	6.54E-05	hypomethylated
cg05113410	0.70438	0.455031351	-0.630388	2.27E-05	hypomethylated
cg22816294	0.35933	0.232111892	-0.630489	0.000138	hypomethylated
cg25711558	0.73389	0.474045405	-0.630539	4.82E-05	hypomethylated
cg03979241	0.74773	0.482982703	-0.630546	0.000132	hypomethylated
cg17419597	0.35914	0.231963784	-0.630647	0.000138	hypomethylated
cg20388732	0.55973	0.361456216	-0.63091	0.009877	hypomethylated
cg07372824	0.31055	0.200450811	-0.631577	0.000132	hypomethylated
cg22401939	0.39391	0.254232432	-0.631718	0.001395	hypomethylated

cg16041798	0.72708	0.469238919	-0.631791	0.000109	hypomethylated
cg07016556	0.53615	0.345992973	-0.631894	0.000435	hypomethylated
cg01999701	0.38181	0.246387027	-0.631929	1.58E-05	hypomethylated
cg27305460	0.52625	0.339568649	-0.632045	0.000129	hypomethylated
cg04892643	0.64055	0.413321081	-0.632048	0.000224	hypomethylated
cg08141959	0.86347	0.55716	-0.632054	7.12E-06	hypomethylated
cg16326421	0.77022	0.496982162	-0.632077	1.17E-05	hypomethylated
cg25738326	0.49602	0.320048108	-0.63211	0.001803	hypomethylated
cg08380379	0.8511	0.549145405	-0.63214	5.89E-06	hypomethylated
cg15755348	0.73603	0.474874595	-0.632218	4.70E-05	hypomethylated
cg02709321	0.4394	0.28348	-0.632288	0.001949	hypomethylated
cg25164589	0.71572	0.461711351	-0.632404	6.95E-05	hypomethylated
cg11696165	0.52125	0.336256216	-0.632415	0.000287	hypomethylated
cg03349134	0.68732	0.443331892	-0.632595	0.000741	hypomethylated
cg27172175	0.59677	0.384897297	-0.632701	7.12E-05	hypomethylated
cg16171484	0.67482	0.435216216	-0.63277	1.01E-05	hypomethylated
cg20358011	0.47213	0.304455135	-0.632955	0.001481	hypomethylated
cg22829164	0.62854	0.405245405	-0.633209	5.45E-05	hypomethylated
cg06000878	0.56746	0.365842703	-0.633295	0.00095	hypomethylated
cg25340711	0.9239	0.595630811	-0.633318	5.81E-06	hypomethylated
cg16426764	0.53687	0.346067568	-0.633519	5.45E-05	hypomethylated
cg09244071	0.73965	0.476771351	-0.633545	1.89E-05	hypomethylated
cg10225951	0.79197	0.510464865	-0.633634	6.31E-05	hypomethylated
cg17958516	0.65767	0.423868649	-0.633747	7.12E-06	hypomethylated
cg15514918	0.83313	0.536844865	-0.634036	5.28E-06	hypomethylated
cg26717066	0.87856	0.566082703	-0.634128	2.36E-06	hypomethylated
cg14695751	0.50575	0.325856757	-0.634187	0.000327	hypomethylated
cg27085869	0.30339	0.195442703	-0.634428	0.000214	hypomethylated
cg03072665	0.53868	0.347013514	-0.634437	1.31E-05	hypomethylated
cg26279261	0.44125	0.284244324	-0.634465	0.002274	hypomethylated
cg05363955	0.76057	0.489924865	-0.634521	5.72E-05	hypomethylated
cg14483244	0.46419	0.298996216	-0.634588	0.007933	hypomethylated
cg18477949	0.54378	0.350254054	-0.634621	1.33E-06	hypomethylated
cg02355558	0.61168	0.393897297	-0.634958	3.62E-05	hypomethylated
cg16247269	0.55905	0.359955676	-0.635158	1.15E-05	hypomethylated
cg23007325	0.74391	0.478857297	-0.635532	1.18E-05	hypomethylated
cg10410146	0.75184	0.483932432	-0.63562	1.05E-06	hypomethylated
cg15393702	0.61922	0.398510811	-0.635833	0.000157	hypomethylated
cg17568962	0.42191	0.271525946	-0.635845	0.00015	hypomethylated
cg24624505	0.6618	0.425905946	-0.63586	0.000174	hypomethylated
cg15224459	0.64933	0.417855676	-0.635947	0.002318	hypomethylated
cg22743884	0.83425	0.536834054	-0.636004	9.82E-06	hypomethylated
cg04070188	0.77019	0.495597297	-0.636046	1.01E-05	hypomethylated
cg27436118	0.71274	0.458600541	-0.636138	0.000104	hypomethylated
cg23005797	0.89621	0.576625405	-0.636202	3.59E-06	hypomethylated
cg12671632	0.47064	0.302735135	-0.636568	0.000399	hypomethylated
cg20701183	0.73651	0.473607568	-0.637013	9.38E-05	hypomethylated
cg14026238	0.73062	0.469805405	-0.637058	2.95E-06	hypomethylated
cg00852846	0.3554	0.228528108	-0.637072	0.002883	hypomethylated
cg11048797	0.88698	0.570332432	-0.637098	3.49E-06	hypomethylated
cg00891886	0.35614	0.228958378	-0.637359	0.000306	hypomethylated
cg04910082	0.84297	0.541878919	-0.637511	1.58E-06	hypomethylated
cg03787603	0.90129	0.57934	-0.637581	2.75E-06	hypomethylated
cg03957109	0.80465	0.517211351	-0.637607	6.54E-05	hypomethylated
cg05574870	0.76431	0.491277838	-0.637619	2.39E-05	hypomethylated
cg21519787	0.70818	0.455176216	-0.637691	2.71E-05	hypomethylated
cg01017395	0.4928	0.316742162	-0.637693	0.002274	hypomethylated
cg26476820	0.72159	0.463759459	-0.637803	2.57E-05	hypomethylated

cg17643598	0.41995	0.269887027	-0.637862	0.007601	hypomethylated
cg06942111	0.71402	0.458827027	-0.638014	2.89E-05	hypomethylated
cg16296417	0.73765	0.473999459	-0.638051	3.07E-05	hypomethylated
cg20709530	0.56444	0.362688649	-0.638089	0.000857	hypomethylated
cg04019522	0.50345	0.323454054	-0.638288	4.94E-05	hypomethylated
cg13610455	0.6615	0.424938378	-0.638488	4.05E-05	hypomethylated
cg26426690	0.66714	0.42850973	-0.638662	0.000459	hypomethylated
cg02925248	0.71448	0.458908108	-0.638688	2.95E-06	hypomethylated
cg22492271	0.75817	0.486913514	-0.638856	6.22E-06	hypomethylated
cg22223119	0.6435	0.413264324	-0.638875	0.00028	hypomethylated
cg01872593	0.47239	0.303372432	-0.638888	0.00099	hypomethylated
cg20208009	0.44132	0.283363243	-0.639172	0.000399	hypomethylated
cg07546943	0.69176	0.444158378	-0.639197	7.12E-05	hypomethylated
cg24207068	0.55295	0.355013514	-0.639275	0.000772	hypomethylated
cg11807280	0.61538	0.395068649	-0.639374	2.38E-05	hypomethylated
cg20899781	0.61498	0.39473027	-0.639672	0.000187	hypomethylated
cg08136020	0.64516	0.414034595	-0.639906	0.005223	hypomethylated
cg07303577	0.87925	0.564259459	-0.639915	8.83E-06	hypomethylated
cg01870834	0.55198	0.354204324	-0.640034	0.006068	hypomethylated
cg22184944	0.63816	0.409465405	-0.640177	1.71E-05	hypomethylated
cg12972064	0.49719	0.31896	-0.640422	0.000822	hypomethylated
cg07442907	0.49943	0.320360541	-0.640586	0.006564	hypomethylated
cg11583392	0.52461	0.336463243	-0.640796	1.71E-05	hypomethylated
cg01084566	0.59235	0.379895135	-0.640849	0.000581	hypomethylated
cg27312979	0.62312	0.399623784	-0.640868	0.000138	hypomethylated
cg05261596	0.36415	0.233531351	-0.640917	1.56E-05	hypomethylated
cg06398236	0.5572	0.357329189	-0.640941	0.000327	hypomethylated
cg00912277	0.85844	0.550488649	-0.641004	1.29E-06	hypomethylated
cg04100337	0.63966	0.410165405	-0.641099	3.85E-05	hypomethylated
cg19695507	0.73455	0.470991351	-0.64116	0.000256	hypomethylated
cg00645755	0.62308	0.399456757	-0.641378	6.01E-05	hypomethylated
cg23361127	0.69393	0.444831351	-0.641532	0.000534	hypomethylated
cg09522147	0.54563	0.349724865	-0.641703	0.000148	hypomethylated
cg04898939	0.45309	0.290396216	-0.641775	4.26E-05	hypomethylated
cg16978268	0.57596	0.369125946	-0.641855	9.95E-05	hypomethylated
cg24169486	0.73754	0.472676216	-0.641869	1.21E-05	hypomethylated
cg07533239	0.43395	0.278106486	-0.641891	0.000155	hypomethylated
cg08800893	0.47116	0.301944865	-0.641932	0.004951	hypomethylated
cg22672067	0.59176	0.379215135	-0.641996	0.000106	hypomethylated
cg09316347	0.56402	0.361433514	-0.642016	0.000251	hypomethylated
cg21544219	0.60952	0.390532973	-0.642229	8.63E-05	hypomethylated
cg07405121	0.57225	0.366651351	-0.642237	0.0003	hypomethylated
cg26504110	0.56058	0.359163243	-0.642281	0.000214	hypomethylated
cg22786472	0.827	0.529802703	-0.642432	4.26E-05	hypomethylated
cg14646653	0.49763	0.318795676	-0.642441	0.00071	hypomethylated
cg05339037	0.63515	0.406873514	-0.642517	4.94E-05	hypomethylated
cg16569650	0.62436	0.399958378	-0.642528	0.000191	hypomethylated
cg27153327	0.7386	0.473137838	-0.642533	3.85E-05	hypomethylated
cg23920016	0.84051	0.538387027	-0.642621	7.93E-06	hypomethylated
cg09218076	0.6109	0.391304865	-0.642643	0.000112	hypomethylated
cg10211776	0.66643	0.426849189	-0.642727	2.51E-05	hypomethylated
cg17942750	0.87804	0.56238	-0.642741	1.12E-05	hypomethylated
cg02627216	0.53474	0.342472973	-0.642847	0.000365	hypomethylated
cg07781445	0.56369	0.361012432	-0.642853	4.60E-06	hypomethylated
cg03889013	0.66231	0.424154054	-0.642918	0.002599	hypomethylated
cg05349016	0.71287	0.45652973	-0.64293	0.000104	hypomethylated
cg18859033	0.66976	0.428891892	-0.64303	4.05E-05	hypomethylated
cg21049397	0.60958	0.390351892	-0.64304	0.000866	hypomethylated

cg13940444	0.48888	0.313026486	-0.643196	0.001314	hypomethylated
cg24822529	0.61749	0.395363784	-0.643235	0.000126	hypomethylated
cg25630380	0.5949	0.38086	-0.643386	3.95E-05	hypomethylated
cg13579752	0.73009	0.4674	-0.643417	1.37E-06	hypomethylated
cg08476984	0.66379	0.424930811	-0.643499	4.36E-06	hypomethylated
cg16205058	0.26299	0.168338919	-0.643639	0.000814	hypomethylated
cg04642300	0.69498	0.444821622	-0.643745	3.85E-05	hypomethylated
cg11162104	0.70921	0.453901622	-0.643833	1.28E-05	hypomethylated
cg06834507	0.65659	0.420205405	-0.643898	4.05E-05	hypomethylated
cg16677448	0.58561	0.374771892	-0.643927	5.51E-05	hypomethylated
cg23537419	0.80052	0.512290811	-0.643974	2.42E-06	hypomethylated
cg25714865	0.86932	0.556298378	-0.644028	4.82E-05	hypomethylated
cg04094838	0.69298	0.443405946	-0.644186	5.07E-06	hypomethylated
cg14713217	0.42428	0.271473514	-0.644205	0.00035	hypomethylated
cg19859193	0.6249	0.399811892	-0.644304	1.26E-05	hypomethylated
cg17328839	0.62252	0.398265405	-0.64439	2.71E-05	hypomethylated
cg14985891	0.75109	0.480502703	-0.644441	0.000313	hypomethylated
cg16734433	0.91678	0.586498919	-0.644447	6.48E-06	hypomethylated
cg09648727	0.46654	0.298461081	-0.644458	0.000528	hypomethylated
cg03597159	0.83048	0.531238919	-0.644585	1.72E-06	hypomethylated
cg16001384	0.74489	0.476462703	-0.644664	1.87E-05	hypomethylated
cg25741118	0.69358	0.443592973	-0.644826	2.79E-06	hypomethylated
cg17233819	0.56131	0.358972432	-0.644925	0.00017	hypomethylated
cg15503752	0.72118	0.461196757	-0.644977	1.66E-05	hypomethylated
cg08564487	0.69646	0.445305405	-0.645245	2.64E-05	hypomethylated
cg13655341	0.43884	0.280560541	-0.645383	0.000224	hypomethylated
cg11138227	0.7179	0.458916757	-0.64555	2.64E-06	hypomethylated
cg20777796	0.66577	0.42556973	-0.645628	0.00097	hypomethylated
cg25885803	0.41428	0.26471027	-0.646192	0.000277	hypomethylated
cg11072119	0.3633	0.232117838	-0.646304	0.000464	hypomethylated
cg06974428	0.75164	0.480224324	-0.646333	7.12E-06	hypomethylated
cg14145074	0.36995	0.236357297	-0.646361	0.002253	hypomethylated
cg27240158	0.54897	0.350694595	-0.646512	0.000117	hypomethylated
cg09887955	0.51141	0.326658378	-0.646698	0.003473	hypomethylated
cg23173466	0.29376	0.187607027	-0.646924	0.011274	hypomethylated
cg13552026	0.56068	0.358027568	-0.647107	1.48E-05	hypomethylated
cg16461134	0.84261	0.537998919	-0.647262	1.02E-06	hypomethylated
cg27199976	0.41869	0.267316216	-0.647335	0.001635	hypomethylated
cg09412707	0.61019	0.389577297	-0.647349	4.88E-05	hypomethylated
cg03562120	0.70841	0.452269189	-0.647403	3.23E-05	hypomethylated
cg18749617	0.51972	0.331803784	-0.647404	0.001875	hypomethylated
cg12340503	0.65775	0.419876216	-0.647575	6.54E-05	hypomethylated
cg19319487	0.63716	0.406713514	-0.647643	0.001041	hypomethylated
cg19373199	0.41444	0.264545405	-0.647648	4.88E-05	hypomethylated
cg10264003	0.70527	0.450181081	-0.64767	0.000224	hypomethylated
cg07735586	0.55314	0.353072973	-0.647678	0.00071	hypomethylated
cg16789230	0.54328	0.346714054	-0.64795	1.31E-05	hypomethylated
cg27000534	0.5691	0.363142162	-0.648148	0.002674	hypomethylated
cg01768814	0.47901	0.305635135	-0.648245	0.019065	hypomethylated
cg06913345	0.93802	0.598475135	-0.648327	2.57E-06	hypomethylated
cg16934235	0.76632	0.488884324	-0.648454	1.12E-05	hypomethylated
cg24843609	0.71162	0.453960541	-0.64854	7.84E-05	hypomethylated
cg20155035	0.5593	0.356672973	-0.64902	0.001803	hypomethylated
cg26230275	0.39805	0.253802703	-0.649242	0.000148	hypomethylated
cg02888518	0.34796	0.221788649	-0.649736	0.00064	hypomethylated
cg08360599	0.36185	0.23062973	-0.649813	0.00031	hypomethylated
cg02109507	0.66719	0.425180541	-0.650022	3.40E-05	hypomethylated
cg13410002	0.3002	0.191299459	-0.650091	4.05E-05	hypomethylated

cg00131261	0.85261	0.543306486	-0.65012	2.39E-05	hypomethylated
cg09657114	0.83622	0.532854595	-0.650141	1.18E-05	hypomethylated
cg21052932	0.64458	0.410710811	-0.650237	0.000857	hypomethylated
cg10855978	0.41256	0.262870811	-0.65025	0.000155	hypomethylated
cg14698665	0.6613	0.421282703	-0.650516	0.000251	hypomethylated
cg25742326	0.56411	0.359365946	-0.650523	0.00035	hypomethylated
cg17125585	0.54019	0.344123784	-0.650539	8.15E-06	hypomethylated
cg02524099	0.37871	0.241244865	-0.650595	0.000725	hypomethylated
cg23560676	0.85955	0.547517297	-0.650677	4.93E-06	hypomethylated
cg01291854	0.88237	0.561948108	-0.650947	2.46E-06	hypomethylated
cg17670650	0.45011	0.286656216	-0.650956	0.000161	hypomethylated
cg19229692	0.83353	0.530818378	-0.651016	4.73E-06	hypomethylated
cg25212701	0.87571	0.557544865	-0.651365	5.51E-07	hypomethylated
cg07332757	0.42402	0.269961081	-0.651381	0.000198	hypomethylated
cg20992708	0.76828	0.48912973	-0.651415	8.32E-05	hypomethylated
cg17541002	0.73699	0.46916973	-0.651535	0.000365	hypomethylated
cg21130113	0.62064	0.395001622	-0.651898	0.002168	hypomethylated
cg06817454	0.69779	0.444072973	-0.651996	1.60E-05	hypomethylated
cg25676471	0.54496	0.346809189	-0.652008	0.000921	hypomethylated
cg20216309	0.87932	0.559588649	-0.652022	3.59E-06	hypomethylated
cg10857221	0.44399	0.282544324	-0.65205	0.000117	hypomethylated
cg16711332	0.86895	0.552961622	-0.652094	1.12E-05	hypomethylated
cg02912371	0.56733	0.361019459	-0.652112	1.07E-06	hypomethylated
cg21527078	0.78892	0.501961622	-0.652302	2.21E-05	hypomethylated
cg02049243	0.60638	0.385785405	-0.652424	9.19E-06	hypomethylated
cg19696794	0.65474	0.416490811	-0.652637	0.000107	hypomethylated
cg07313701	0.6305	0.401052432	-0.652706	0.000117	hypomethylated
cg26252794	0.3805	0.24202973	-0.652712	0.001803	hypomethylated
cg09612099	0.6632	0.421798919	-0.652889	2.07E-05	hypomethylated
cg17819732	0.57215	0.363885405	-0.652909	1.31E-05	hypomethylated
cg07910075	0.70353	0.447405946	-0.653028	1.92E-05	hypomethylated
cg16362949	0.48129	0.306067027	-0.653059	0.00098	hypomethylated
cg03902450	0.46259	0.294143243	-0.653215	0.000822	hypomethylated
cg23817637	0.75519	0.48018	-0.653264	0.000102	hypomethylated
cg27504369	0.6356	0.404130811	-0.653297	3.57E-05	hypomethylated
cg25568114	0.90269	0.573890811	-0.653454	7.12E-06	hypomethylated
cg11820824	0.78059	0.49622	-0.653585	1.58E-05	hypomethylated
cg14420953	0.73658	0.468196757	-0.653727	4.76E-05	hypomethylated
cg09631880	0.59505	0.37814	-0.65409	2.39E-05	hypomethylated
cg13919908	0.73325	0.465928108	-0.654198	2.61E-05	hypomethylated
cg24890964	0.61502	0.390780541	-0.654275	4.94E-05	hypomethylated
cg02920604	0.63678	0.404592432	-0.654326	6.05E-06	hypomethylated
cg23323158	0.72864	0.462938378	-0.654386	1.28E-05	hypomethylated
cg03887438	0.62095	0.394495135	-0.65447	3.96E-06	hypomethylated
cg18860310	0.57784	0.367092432	-0.654527	4.76E-05	hypomethylated
cg06360703	0.39316	0.249766486	-0.654537	0.013481	hypomethylated
cg10113526	0.64304	0.408507027	-0.654548	0.0006	hypomethylated
cg05573829	0.79489	0.504932432	-0.654665	5.43E-06	hypomethylated
cg15999356	0.73542	0.467155676	-0.654665	2.16E-06	hypomethylated
cg00355699	0.78815	0.500647027	-0.654676	8.83E-06	hypomethylated
cg08147181	0.60736	0.385796757	-0.654711	0.0006	hypomethylated
cg11468193	0.40276	0.255829189	-0.65474	0.000234	hypomethylated
cg24659037	0.91204	0.579284865	-0.654824	7.52E-06	hypomethylated
cg09989037	0.74246	0.471574054	-0.654829	0.000191	hypomethylated
cg04687437	0.31778	0.201818378	-0.654971	0.001395	hypomethylated
cg15248935	0.31607	0.200704324	-0.655172	0.002526	hypomethylated
cg17209188	0.77948	0.494951351	-0.655225	4.70E-05	hypomethylated
cg20299802	0.65576	0.416372973	-0.655291	1.66E-05	hypomethylated

cg04244970	0.71873	0.456273514	-0.655551	0.000115	hypomethylated
cg23750206	0.67424	0.427959459	-0.655788	0.002106	hypomethylated
cg01392656	0.79065	0.501791351	-0.655952	4.12E-06	hypomethylated
cg10965581	0.6624	0.420384865	-0.655992	2.85E-05	hypomethylated
cg02598079	0.56568	0.358987027	-0.656054	0.002455	hypomethylated
cg16341979	0.76653	0.486412973	-0.656161	8.83E-06	hypomethylated
cg11118235	0.46341	0.294023243	-0.656359	0.006015	hypomethylated
cg19348484	0.62031	0.393443243	-0.656834	0.000283	hypomethylated
cg17600393	0.64599	0.409726486	-0.656851	3.81E-05	hypomethylated
cg12905085	0.40825	0.258922162	-0.656934	0.002751	hypomethylated
cg09134497	0.71622	0.454222162	-0.657005	1.65E-06	hypomethylated
cg01285783	0.64994	0.412165946	-0.657081	9.69E-06	hypomethylated
cg06011086	0.61732	0.391471892	-0.65711	0.00039	hypomethylated
cg17563773	0.47849	0.303372432	-0.657399	2.27E-05	hypomethylated
cg06783197	0.60472	0.383376216	-0.657506	0.000104	hypomethylated
cg00337466	0.39829	0.252487027	-0.65761	0.001188	hypomethylated
cg17660833	0.76583	0.485436757	-0.657741	0.000132	hypomethylated
cg21562208	0.83795	0.53110973	-0.657854	4.07E-06	hypomethylated
cg02577773	0.59304	0.375854595	-0.657955	0.000101	hypomethylated
cg00910893	0.25517	0.161707568	-0.658072	0.000902	hypomethylated
cg22168796	0.54739	0.346856216	-0.658231	0.001803	hypomethylated
cg18921475	0.75036	0.475413514	-0.6584	7.72E-06	hypomethylated
cg01797450	0.46271	0.29315027	-0.658468	0.000107	hypomethylated
cg15387123	0.51772	0.32799027	-0.658519	0.0003	hypomethylated
cg21614638	0.65421	0.41444973	-0.658557	0.001262	hypomethylated
cg23437479	0.86057	0.545152973	-0.658631	1.45E-06	hypomethylated
cg20068496	0.42285	0.267857297	-0.658681	7.75E-05	hypomethylated
cg12913097	0.19863	0.125818919	-0.658735	0.008069	hypomethylated
cg01412762	0.56245	0.356222703	-0.658945	0.005508	hypomethylated
cg08912801	0.47092	0.298252432	-0.658948	0.000893	hypomethylated
cg21069965	0.71242	0.451192432	-0.658985	1.50E-05	hypomethylated
cg08071719	0.88582	0.560948108	-0.659146	4.01E-06	hypomethylated
cg07593977	0.688	0.435651892	-0.659233	0.00102	hypomethylated
cg22926842	0.61313	0.388182162	-0.659459	0.0002	hypomethylated
cg13708645	0.46984	0.297447568	-0.659534	0.000725	hypomethylated
cg26182253	0.84466	0.534683243	-0.659686	3.21E-06	hypomethylated
cg07945618	0.72533	0.459046486	-0.659997	1.31E-05	hypomethylated
cg07497941	0.67846	0.429345405	-0.660125	0.000224	hypomethylated
cg13670306	0.26789	0.169520541	-0.660181	0.005756	hypomethylated
cg26052586	0.69047	0.436911351	-0.660238	5.06E-05	hypomethylated
cg25734089	0.7585	0.479905946	-0.660397	5.06E-05	hypomethylated
cg16728539	0.69925	0.442313514	-0.660739	0.000517	hypomethylated
cg15161854	0.47564	0.300832973	-0.660907	0.000214	hypomethylated
cg03316864	0.28682	0.181407027	-0.660915	4.05E-05	hypomethylated
cg24405567	0.51422	0.325196216	-0.661075	0.000653	hypomethylated
cg22794304	0.39755	0.251399459	-0.661155	9.49E-05	hypomethylated
cg23965720	0.46457	0.293747027	-0.661322	0.001541	hypomethylated
cg15044760	0.35869	0.226766486	-0.66153	0.0017	hypomethylated
cg00294534	0.68609	0.433725946	-0.661614	6.39E-06	hypomethylated
cg19955173	0.89084	0.563081622	-0.661822	3.69E-06	hypomethylated
cg04589210	0.60053	0.379575135	-0.661851	2.71E-05	hypomethylated
cg01860897	0.28201	0.178232973	-0.661982	0.001541	hypomethylated
cg10196558	0.44903	0.283778378	-0.662047	0.000112	hypomethylated
cg02710534	0.7231	0.456921622	-0.662248	1.09E-05	hypomethylated
cg21537187	0.72048	0.455160541	-0.662583	1.21E-05	hypomethylated
cg18007641	0.58991	0.372664324	-0.662618	2.35E-05	hypomethylated
cg16622061	0.36943	0.233369189	-0.662687	0.000166	hypomethylated
cg23230478	0.9176	0.579497297	-0.663063	1.28E-05	hypomethylated

cg08976687	0.82911	0.523607027	-0.663079	4.36E-06	hypomethylated
cg27494897	0.5844	0.369044865	-0.66316	0.0002	hypomethylated
cg00782811	0.58296	0.368112973	-0.663248	0.0012	hypomethylated
cg14050824	0.92932	0.586797297	-0.663313	6.13E-06	hypomethylated
cg08977611	0.44899	0.283494595	-0.663362	2.10E-05	hypomethylated
cg04956471	0.52325	0.330303243	-0.663709	0.000633	hypomethylated
cg19372602	0.39637	0.250202703	-0.66375	0.000517	hypomethylated
cg12894524	0.53742	0.339158378	-0.664091	0.00101	hypomethylated
cg16137928	0.52467	0.331104324	-0.664124	0.000214	hypomethylated
cg17152989	0.30828	0.194537838	-0.664191	2.30E-05	hypomethylated
cg10800346	0.73982	0.466832973	-0.664268	0.000109	hypomethylated
cg24859433	0.54267	0.34236	-0.664561	0.000115	hypomethylated
cg11768167	0.2612	0.164781081	-0.664604	0.00421	hypomethylated
cg19244029	0.60991	0.384743784	-0.664698	0.000234	hypomethylated
cg16366686	0.45754	0.288591892	-0.664867	0.000109	hypomethylated
cg05394294	0.84118	0.530394054	-0.66535	0.000172	hypomethylated
cg02989244	0.58537	0.369088649	-0.665381	0.006915	hypomethylated
cg22261694	0.62486	0.393948108	-0.665527	0.00421	hypomethylated
cg04208434	0.46946	0.295962703	-0.665587	0.00071	hypomethylated
cg24773560	0.49628	0.312854595	-0.665662	0.002231	hypomethylated
cg27062243	0.56373	0.355324324	-0.665868	3.85E-05	hypomethylated
cg26369382	0.76109	0.479721081	-0.665871	3.69E-06	hypomethylated
cg06388099	0.30723	0.193630811	-0.666011	0.002296	hypomethylated
cg07157333	0.61688	0.388755676	-0.666126	1.75E-05	hypomethylated
cg21981144	0.50714	0.319595676	-0.666136	3.76E-05	hypomethylated
cg20945055	0.75179	0.473756216	-0.666185	4.15E-05	hypomethylated
cg13283635	0.47752	0.300867568	-0.666433	0.001395	hypomethylated
cg19008097	0.4518	0.284658378	-0.666453	3.07E-05	hypomethylated
cg06978117	0.78566	0.494943243	-0.666642	7.29E-05	hypomethylated
cg18633230	0.72963	0.459644324	-0.666647	1.15E-05	hypomethylated
cg01066157	0.66271	0.417476216	-0.666684	6.86E-05	hypomethylated
cg08437570	0.33612	0.211715135	-0.666852	0.008563	hypomethylated
cg23015138	0.37505	0.236220541	-0.666949	0.008636	hypomethylated
cg13100118	0.80739	0.508516216	-0.666972	4.64E-05	hypomethylated
cg23564178	0.39176	0.246741081	-0.666972	0.000327	hypomethylated
cg08119452	0.74878	0.471587568	-0.667016	1.71E-05	hypomethylated
cg03813377	0.35558	0.223936757	-0.667083	0.000575	hypomethylated
cg25378181	0.59133	0.372406486	-0.667085	1.92E-05	hypomethylated
cg03265268	0.83111	0.523393514	-0.667143	3.79E-06	hypomethylated
cg14733031	0.51958	0.327154595	-0.667373	0.000485	hypomethylated
cg03919488	0.67662	0.426032973	-0.667381	5.19E-05	hypomethylated
cg02184226	0.80865	0.509112973	-0.66753	6.75E-06	hypomethylated
cg27583534	0.45036	0.283537297	-0.66754	4.94E-05	hypomethylated
cg07745373	0.44626	0.280943784	-0.667603	0.000412	hypomethylated
cg21868063	0.68907	0.433786486	-0.667665	1.31E-05	hypomethylated
cg21196201	0.46836	0.294820541	-0.667781	4.70E-05	hypomethylated
cg03193328	0.57282	0.360568108	-0.66781	0.001667	hypomethylated
cg14091840	0.79774	0.502105405	-0.667928	1.99E-06	hypomethylated
cg04134818	0.28139	0.177091892	-0.668073	0.000161	hypomethylated
cg01751245	0.76372	0.480619459	-0.668149	8.94E-05	hypomethylated
cg19263548	0.75708	0.476400541	-0.668271	4.12E-06	hypomethylated
cg12415569	0.53785	0.338434054	-0.668329	6.95E-05	hypomethylated
cg14646244	0.7412	0.46616	-0.669038	0.000229	hypomethylated
cg22708150	0.78763	0.495341081	-0.669096	0.000187	hypomethylated
cg07906484	0.73188	0.460278378	-0.6691	3.39E-06	hypomethylated
cg10505873	0.22579	0.141998919	-0.669102	0.00035	hypomethylated
cg17250262	0.44169	0.277749189	-0.669251	2.24E-05	hypomethylated
cg04246167	0.72119	0.453468649	-0.669377	9.16E-05	hypomethylated

cg02659568	0.79437	0.499398919	-0.669618	9.57E-06	hypomethylated
cg05146922	0.62731	0.394241081	-0.6701	1.99E-06	hypomethylated
cg22681001	0.32494	0.204207568	-0.670137	0.000395	hypomethylated
cg10342016	0.5689	0.357472432	-0.670343	1.28E-05	hypomethylated
cg23315892	0.66972	0.420794595	-0.670442	2.10E-06	hypomethylated
cg24894531	0.86569	0.543903784	-0.670499	4.01E-06	hypomethylated
cg08916374	0.51	0.320402162	-0.670613	2.27E-05	hypomethylated
cg13745870	0.54571	0.342657838	-0.671366	0.001262	hypomethylated
cg14106234	0.88267	0.554147568	-0.671604	1.12E-05	hypomethylated
cg27408541	0.54011	0.339048649	-0.671761	0.003225	hypomethylated
cg19040266	0.58284	0.365855676	-0.671825	0.000115	hypomethylated
cg21937377	0.76884	0.482452973	-0.672295	0.001096	hypomethylated
cg12072001	0.47002	0.294921622	-0.672391	0.00032	hypomethylated
cg26035201	0.83253	0.522360541	-0.672456	1.33E-06	hypomethylated
cg17112426	0.71084	0.445918378	-0.672745	1.08E-05	hypomethylated
cg18720687	0.37986	0.238288108	-0.672761	0.000979	hypomethylated
cg10523140	0.43932	0.275573514	-0.672835	0.004059	hypomethylated
cg18114313	0.65759	0.412473514	-0.672887	8.42E-05	hypomethylated
cg27173717	0.55937	0.350848649	-0.672954	0.000287	hypomethylated
cg08043565	0.57454	0.360342703	-0.673038	1.50E-05	hypomethylated
cg16002660	0.56195	0.352391351	-0.673263	0.00012	hypomethylated
cg04563996	0.59566	0.37353027	-0.673264	0.0006	hypomethylated
cg02502358	0.5097	0.319534595	-0.673676	0.000256	hypomethylated
cg09517873	0.68755	0.430964324	-0.673896	0.000756	hypomethylated
cg25594736	0.7141	0.447598378	-0.673921	0.000912	hypomethylated
cg10081994	0.76405	0.478834054	-0.674141	7.93E-06	hypomethylated
cg26484813	0.65182	0.408497838	-0.674145	0.000204	hypomethylated
cg10897459	0.57489	0.360245946	-0.674304	3.57E-05	hypomethylated
cg07082267	0.4015	0.251590811	-0.674321	0.000138	hypomethylated
cg13522882	0.35383	0.221719459	-0.674321	0.000528	hypomethylated
cg00752263	0.36695	0.229931892	-0.674377	8.22E-05	hypomethylated
cg24152264	0.59733	0.374271892	-0.674441	3.67E-05	hypomethylated
cg27404606	0.26019	0.163017838	-0.674536	0.003021	hypomethylated
cg09981407	0.44756	0.280384324	-0.674675	0.00134	hypomethylated
cg07157107	0.53852	0.337361081	-0.674706	0.000145	hypomethylated
cg02101833	0.56724	0.355282162	-0.674994	0.000681	hypomethylated
cg00939627	0.51017	0.319520541	-0.675069	3.49E-05	hypomethylated
cg26113233	0.6294	0.394184865	-0.675105	2.04E-05	hypomethylated
cg10340048	0.71018	0.444758919	-0.675161	3.62E-05	hypomethylated
cg06342072	0.67972	0.425679459	-0.675173	2.85E-05	hypomethylated
cg00207428	0.61931	0.387845946	-0.675178	1.35E-05	hypomethylated
cg23199335	0.43057	0.269631351	-0.67526	0.046858	hypomethylated
cg26853536	0.4501	0.281824324	-0.675449	2.10E-05	hypomethylated
cg27298940	0.84374	0.528238919	-0.675608	5.89E-06	hypomethylated
cg22147446	0.61027	0.382052973	-0.675675	7.84E-05	hypomethylated
cg01306183	0.81857	0.512432973	-0.675742	5.89E-06	hypomethylated
cg02661728	0.84004	0.525671892	-0.676295	6.05E-06	hypomethylated
cg27410595	0.6493	0.406296757	-0.676351	1.28E-05	hypomethylated
cg07207490	0.53087	0.332118919	-0.676659	0.000138	hypomethylated
cg04462662	0.83291	0.520992973	-0.676897	6.05E-06	hypomethylated
cg09127107	0.63276	0.39576973	-0.676997	1.18E-06	hypomethylated
cg14537247	0.56024	0.350405405	-0.67702	4.00E-05	hypomethylated
cg27165884	0.42837	0.267924865	-0.677029	0.000741	hypomethylated
cg13568106	0.75203	0.470354054	-0.677043	3.49E-05	hypomethylated
cg27181349	0.57973	0.362544324	-0.677224	0.000789	hypomethylated
cg14096207	0.46795	0.292632973	-0.677262	0.000103	hypomethylated
cg14688342	0.69723	0.435977838	-0.67738	1.29E-06	hypomethylated
cg13136655	0.5823	0.364110811	-0.677385	0.001667	hypomethylated

cg14904363	0.27696	0.173177297	-0.677428	0.006015	hypomethylated
cg14284257	0.83815	0.524047568	-0.677511	2.16E-06	hypomethylated
cg19930352	0.56429	0.352695676	-0.678013	0.000403	hypomethylated
cg07754940	0.60151	0.375897838	-0.678248	0.00255	hypomethylated
cg23856611	0.70984	0.443427568	-0.678795	0.000293	hypomethylated
cg06218079	0.82571	0.515758919	-0.678938	6.78E-05	hypomethylated
cg23731595	0.44177	0.275924324	-0.679023	9.16E-05	hypomethylated
cg27549186	0.5746	0.358877297	-0.679067	0.002189	hypomethylated
cg01513157	0.6816	0.425658919	-0.679227	0.000313	hypomethylated
cg07495704	0.52722	0.329225946	-0.679327	0.000251	hypomethylated
cg21335012	0.69829	0.436017838	-0.679439	3.15E-05	hypomethylated
cg18842107	0.76979	0.480572432	-0.679711	2.10E-05	hypomethylated
cg13477614	0.51689	0.322669189	-0.679801	0.000581	hypomethylated
cg22760287	0.75076	0.468656757	-0.67982	1.15E-05	hypomethylated
cg01546820	0.42808	0.267220541	-0.67985	4.73E-06	hypomethylated
cg07786675	0.85392	0.532981622	-0.680015	7.51E-06	hypomethylated
cg27262041	0.54309	0.338952973	-0.680106	0.000317	hypomethylated
cg03330678	0.54868	0.342438919	-0.680118	0.000198	hypomethylated
cg02782895	0.36065	0.225086486	-0.68012	3.15E-05	hypomethylated
cg27461254	0.62411	0.389510811	-0.680137	0.000178	hypomethylated
cg12586347	0.34648	0.216235676	-0.680168	0.000109	hypomethylated
cg25063165	0.39354	0.245601622	-0.68019	0.000132	hypomethylated
cg14064268	0.47671	0.297492432	-0.680259	1.08E-05	hypomethylated
cg14644871	0.40512	0.252807027	-0.680313	0.001237	hypomethylated
cg01753788	0.4783	0.298423243	-0.680556	2.78E-05	hypomethylated
cg13252152	0.36222	0.225985405	-0.680637	0.000454	hypomethylated
cg08159594	0.40688	0.253833514	-0.680721	6.48E-06	hypomethylated
cg05255994	0.83133	0.518614054	-0.68076	1.99E-05	hypomethylated
cg19255783	0.50472	0.314842703	-0.680852	0.0002	hypomethylated
cg07911961	0.54382	0.339063784	-0.681573	0.000172	hypomethylated
cg01543184	0.52621	0.328072973	-0.681622	0.000464	hypomethylated
cg12494529	0.78956	0.492257297	-0.681636	8.15E-06	hypomethylated
cg13804575	0.52189	0.325375676	-0.681639	0.000839	hypomethylated
cg14780070	0.69232	0.431627568	-0.681652	8.03E-05	hypomethylated
cg19781870	0.33228	0.207158919	-0.681662	0.000234	hypomethylated
cg23904247	0.44783	0.279197838	-0.681663	0.012535	hypomethylated
cg12542656	0.66504	0.414528108	-0.681971	0.000187	hypomethylated
cg01637090	0.52368	0.326405405	-0.682021	5.86E-05	hypomethylated
cg13514772	0.49724	0.309892432	-0.682175	0.000109	hypomethylated
cg19483007	0.31875	0.198631892	-0.682328	0.000174	hypomethylated
cg19352830	0.50569	0.315036757	-0.682733	2.79E-06	hypomethylated
cg04188351	0.41699	0.259771351	-0.68277	0.000646	hypomethylated
cg18668449	0.76416	0.476046486	-0.682772	3.04E-06	hypomethylated
cg27262821	0.72895	0.45406973	-0.682906	1.35E-05	hypomethylated
cg19478820	0.69287	0.43152973	-0.683125	5.73E-06	hypomethylated
cg24603113	0.45436	0.282971892	-0.683177	0.001803	hypomethylated
cg21108085	0.56742	0.353266486	-0.68366	0.003773	hypomethylated
cg07081477	0.61047	0.380017838	-0.683853	0.001262	hypomethylated
cg08913523	0.53925	0.335671351	-0.683905	0.004485	hypomethylated
cg03547355	0.41023	0.255296757	-0.684258	2.42E-05	hypomethylated
cg20561830	0.30289	0.18847027	-0.684457	0.015809	hypomethylated
cg10816760	0.34859	0.216855676	-0.684796	0.0003	hypomethylated
cg02068356	0.54886	0.341434054	-0.684831	0.000195	hypomethylated
cg23116540	0.53835	0.334864324	-0.684968	0.000102	hypomethylated
cg12999267	0.43487	0.270496757	-0.684973	7.32E-06	hypomethylated
cg02816525	0.78699	0.489498919	-0.68504	9.27E-05	hypomethylated
cg21493727	0.73746	0.458657297	-0.685148	1.84E-05	hypomethylated
cg13177275	0.70083	0.435852973	-0.685223	1.94E-05	hypomethylated

cg26451345	0.82249	0.5115	-0.685264	4.73E-06	hypomethylated
cg01610979	0.64386	0.400336216	-0.685535	0.000365	hypomethylated
cg03221483	0.55312	0.343857838	-0.68578	0.00017	hypomethylated
cg04987122	0.87365	0.543083243	-0.685882	4.01E-06	hypomethylated
cg25198049	0.6547	0.406947568	-0.685991	0.001107	hypomethylated
cg13992911	0.28875	0.179464324	-0.686124	1.42E-05	hypomethylated
cg01674147	0.60582	0.376510811	-0.686198	2.95E-06	hypomethylated
cg16409409	0.62061	0.385698919	-0.686212	0.000382	hypomethylated
cg17349632	0.83867	0.521178378	-0.686326	3.30E-06	hypomethylated
cg18110015	0.70835	0.440171351	-0.686397	9.82E-06	hypomethylated
cg06762332	0.76601	0.475988108	-0.686438	2.21E-05	hypomethylated
cg24505687	0.31551	0.196018919	-0.686693	8.84E-05	hypomethylated
cg10083464	0.44985	0.279441622	-0.686897	0.000214	hypomethylated
cg08220120	0.63056	0.391667568	-0.687004	6.39E-06	hypomethylated
cg27149973	0.44959	0.279258378	-0.687009	0.00071	hypomethylated
cg10976218	0.58022	0.360395676	-0.687018	3.54E-06	hypomethylated
cg14671011	0.74448	0.462405405	-0.687075	1.15E-05	hypomethylated
cg11769476	0.54761	0.340122162	-0.687096	0.00099	hypomethylated
cg06264679	0.34465	0.214047027	-0.687204	0.000293	hypomethylated
cg27083087	0.82097	0.509777297	-0.687462	1.80E-05	hypomethylated
cg22795471	0.3843	0.238618919	-0.687525	0.00064	hypomethylated
cg14131038	0.44496	0.276261081	-0.687643	0.0002	hypomethylated
cg10825315	0.48319	0.299956216	-0.687839	0.000459	hypomethylated
cg04178634	0.71008	0.440793514	-0.687879	1.87E-05	hypomethylated
cg25065131	0.66995	0.415855676	-0.687971	1.42E-05	hypomethylated
cg02604730	0.74303	0.461171351	-0.688118	3.59E-06	hypomethylated
cg23839571	0.37075	0.230086486	-0.68827	0.000342	hypomethylated
cg20617957	0.41135	0.255261081	-0.688393	0.004951	hypomethylated
cg23542902	0.58673	0.364065405	-0.688499	0.000588	hypomethylated
cg00416710	0.71754	0.445207027	-0.688583	4.15E-05	hypomethylated
cg07837078	0.77476	0.480637297	-0.688801	4.73E-06	hypomethylated
cg08284233	0.68473	0.424784865	-0.688803	0.00012	hypomethylated
cg26705724	0.52043	0.32281027	-0.689018	0.000485	hypomethylated
cg19061696	0.74156	0.45996973	-0.689025	1.31E-05	hypomethylated
cg05209306	0.51803	0.321310811	-0.689066	0.000159	hypomethylated
cg01708377	0.8257	0.512123784	-0.689125	5.58E-06	hypomethylated
cg19836199	0.67476	0.418464865	-0.689268	0.001511	hypomethylated
cg10005270	0.76107	0.471984324	-0.68929	4.12E-06	hypomethylated
cg24348240	0.39335	0.243932973	-0.689329	0.001571	hypomethylated
cg15617950	0.38009	0.235709189	-0.689333	0.001212	hypomethylated
cg22990967	0.53256	0.330227568	-0.689484	0.001734	hypomethylated
cg01426968	0.62722	0.388905405	-0.689552	2.45E-05	hypomethylated
cg23102014	0.62958	0.390368649	-0.689553	4.94E-05	hypomethylated
cg05592245	0.57369	0.355699459	-0.689613	2.21E-05	hypomethylated
cg15282973	0.64133	0.397549189	-0.689934	1.80E-05	hypomethylated
cg19893585	0.5856	0.362983243	-0.690013	6.46E-05	hypomethylated
cg06092869	0.47554	0.294761081	-0.690021	5.31E-05	hypomethylated
cg13026629	0.75481	0.467719459	-0.69047	8.83E-06	hypomethylated
cg05711445	0.53171	0.329407027	-0.690768	0.000342	hypomethylated
cg08550523	0.83906	0.5198	-0.690817	1.18E-05	hypomethylated
cg13794404	0.50991	0.315863243	-0.690943	7.84E-05	hypomethylated
cg08765100	0.55978	0.346694054	-0.691197	8.42E-05	hypomethylated
cg09776772	0.84726	0.524695676	-0.691324	3.67E-05	hypomethylated
cg26605164	0.74644	0.462164324	-0.69162	2.15E-05	hypomethylated
cg20938802	0.73103	0.452612973	-0.691653	2.27E-05	hypomethylated
cg18887230	0.33484	0.207311892	-0.691669	0.000403	hypomethylated
cg06399735	0.50601	0.313254595	-0.69183	0.001381	hypomethylated
cg19512473	0.49897	0.308831351	-0.692134	0.000358	hypomethylated

cg02088785	0.55631	0.344312973	-0.692168	2.45E-05	hypomethylated
cg15765546	0.6784	0.419867027	-0.692204	9.31E-06	hypomethylated
cg24060040	0.36458	0.225627027	-0.692296	0.003843	hypomethylated
cg17262810	0.67855	0.419922162	-0.692333	0.000667	hypomethylated
cg22681709	0.70775	0.437956757	-0.692451	3.00E-05	hypomethylated
cg01657995	0.3956	0.244762162	-0.692662	0.000931	hypomethylated
cg00433917	0.62949	0.38937027	-0.693041	4.58E-05	hypomethylated
cg12265130	0.4319	0.267134054	-0.693133	0.000313	hypomethylated
cg08033031	0.28065	0.173530811	-0.69358	0.002478	hypomethylated
ch.6.113866684R	0.26693	0.165038919	-0.693655	0.001074	hypomethylated
cg07835482	0.65758	0.406504865	-0.693894	5.28E-06	hypomethylated
cg26546113	0.54368	0.336068649	-0.694002	0.000148	hypomethylated
cg15754112	0.69811	0.431479459	-0.694162	1.71E-05	hypomethylated
cg16477879	0.77052	0.476215676	-0.694217	2.15E-05	hypomethylated
cg10880902	0.39845	0.246251892	-0.694264	0.000506	hypomethylated
cg04057956	0.54981	0.339739459	-0.694504	0.000426	hypomethylated
cg01903799	0.57573	0.355701081	-0.694727	8.63E-05	hypomethylated
cg01723892	0.45372	0.280317838	-0.694739	0.000454	hypomethylated
cg20533982	0.75105	0.463981081	-0.694843	4.36E-06	hypomethylated
cg14506696	0.6179	0.381685405	-0.694989	0.000857	hypomethylated
cg13385210	0.53364	0.329624865	-0.695042	7.29E-05	hypomethylated
cg15774391	0.46141	0.285001622	-0.695079	0.003571	hypomethylated
cg05901357	0.35462	0.218992432	-0.695393	0.000445	hypomethylated
cg06958636	0.47069	0.290655676	-0.695466	2.51E-05	hypomethylated
cg05973398	0.4298	0.265378378	-0.695615	0.000454	hypomethylated
cg00041401	0.6092	0.376101622	-0.695793	0.004776	hypomethylated
cg18221226	0.38125	0.235363784	-0.695845	0.000569	hypomethylated
cg07507418	0.55825	0.344567568	-0.696124	0.000152	hypomethylated
cg08015107	0.5167	0.318916757	-0.696147	0.000224	hypomethylated
cg10051588	0.71869	0.443579459	-0.696177	2.18E-05	hypomethylated
cg27226320	0.51931	0.320470811	-0.696403	0.002574	hypomethylated
cg20327845	0.621	0.383212973	-0.696447	9.95E-05	hypomethylated
cg09890077	0.53207	0.328321081	-0.696509	8.15E-06	hypomethylated
cg26711638	0.55464	0.342247568	-0.696511	8.83E-06	hypomethylated
cg27407935	0.30449	0.187876757	-0.696608	6.95E-05	hypomethylated
cg00324097	0.3001	0.185143784	-0.696797	0.001164	hypomethylated
cg13435834	0.72553	0.447503784	-0.697135	9.95E-05	hypomethylated
cg21027526	0.33536	0.206761622	-0.697742	0.000703	hypomethylated
cg16764274	0.29272	0.180464865	-0.697803	2.78E-05	hypomethylated
cg19284277	0.35624	0.219607568	-0.697922	0.001423	hypomethylated
cg10094886	0.8453	0.520984865	-0.698222	3.30E-06	hypomethylated
cg08521995	0.60258	0.371378919	-0.698261	1.75E-05	hypomethylated
cg09183450	0.35969	0.221638919	-0.698543	0.00255	hypomethylated
cg11321083	0.50343	0.310159459	-0.698781	0.000399	hypomethylated
cg20244273	0.60415	0.372187568	-0.698877	4.05E-05	hypomethylated
cg17436656	0.40206	0.247622703	-0.699267	5.86E-05	hypomethylated
cg06939851	0.52094	0.320838378	-0.69927	5.45E-05	hypomethylated
cg09462956	0.63032	0.388175135	-0.699377	4.05E-05	hypomethylated
cg21946631	0.74266	0.457268649	-0.69966	2.51E-05	hypomethylated
cg04744624	0.4889	0.300959459	-0.69997	0.000805	hypomethylated
cg03475190	0.39316	0.242019459	-0.699993	0.001571	hypomethylated
cg05725404	0.5052	0.310976757	-0.700048	0.000382	hypomethylated
cg08899105	0.49134	0.302421622	-0.70016	1.80E-05	hypomethylated
cg16067628	0.80597	0.496064324	-0.700199	3.49E-06	hypomethylated
cg23251746	0.61452	0.378189189	-0.700352	0.00035	hypomethylated
cg07972458	0.40364	0.248396757	-0.700423	2.64E-05	hypomethylated
cg11539857	0.87949	0.541198919	-0.700508	1.06E-05	hypomethylated
cg23283335	0.5136	0.316011351	-0.700669	6.46E-05	hypomethylated

cg23250795	0.87404	0.537658378	-0.70101	2.15E-05	hypomethylated
cg08601673	0.45315	0.278721081	-0.701167	0.0006	hypomethylated
cg12232146	0.69448	0.427112432	-0.701317	9.27E-05	hypomethylated
cg20955688	0.49821	0.306372432	-0.701467	0.011274	hypomethylated
cg25278144	0.66519	0.409055135	-0.701471	4.86E-06	hypomethylated
cg10453071	0.49881	0.306733514	-0.701505	0.000117	hypomethylated
cg00471159	0.48473	0.298037297	-0.701688	0.001212	hypomethylated
cg05623526	0.54006	0.332017297	-0.701861	0.00043	hypomethylated
cg04810745	0.79976	0.491652973	-0.701927	1.50E-05	hypomethylated
cg17440044	0.33852	0.208097297	-0.701983	0.001237	hypomethylated
cg08912666	0.77627	0.47714973	-0.702116	1.50E-05	hypomethylated
cg27280535	0.46947	0.288534595	-0.702289	0.000117	hypomethylated
cg16861964	0.58861	0.361703784	-0.702503	0.000219	hypomethylated
cg16291048	0.79558	0.488887027	-0.702506	3.30E-06	hypomethylated
cg08754149	0.73536	0.451854054	-0.702594	3.15E-05	hypomethylated
cg04084348	0.41006	0.251967568	-0.702597	0.000361	hypomethylated
cg18808466	0.73025	0.448697297	-0.702648	8.71E-06	hypomethylated
cg18388547	0.52962	0.325381622	-0.702825	0.00035	hypomethylated
cg05447023	0.83739	0.514447568	-0.702876	9.82E-06	hypomethylated
cg08983279	0.64405	0.395663243	-0.7029	2.24E-05	hypomethylated
cg04956949	0.64816	0.398176216	-0.702943	3.04E-06	hypomethylated
cg13447566	0.7606	0.467201622	-0.703093	4.82E-05	hypomethylated
cg19913118	0.52066	0.319804865	-0.70315	4.01E-06	hypomethylated
cg12126243	0.82431	0.506290811	-0.703221	1.28E-05	hypomethylated
cg05729490	0.54635	0.335567027	-0.703224	3.07E-05	hypomethylated
cg19889066	0.60492	0.371454595	-0.703558	0.000563	hypomethylated
cg03537386	0.80711	0.495572973	-0.703668	2.57E-05	hypomethylated
cg00950497	0.50384	0.309301622	-0.703951	0.002455	hypomethylated
cg06329491	0.32528	0.199674054	-0.704035	0.000245	hypomethylated
cg12176793	0.67639	0.415181081	-0.704115	1.90E-06	hypomethylated
cg27559399	0.33451	0.205308649	-0.704255	6.08E-05	hypomethylated
cg00792251	0.75536	0.463557297	-0.704417	4.24E-06	hypomethylated
cg06834177	0.925	0.567566486	-0.704664	3.21E-06	hypomethylated
cg23090603	0.38337	0.235224324	-0.7047	0.002599	hypomethylated
cg09043518	0.77327	0.47441027	-0.704837	1.82E-06	hypomethylated
cg20457051	0.84879	0.520714054	-0.704916	6.05E-06	hypomethylated
cg23314364	0.69374	0.425591892	-0.704924	0.000274	hypomethylated
cg17680767	0.72495	0.44472973	-0.704953	5.58E-05	hypomethylated
cg07965110	0.64107	0.393258378	-0.705004	0.000382	hypomethylated
cg05506147	0.57758	0.354300541	-0.705047	1.50E-05	hypomethylated
cg08426898	0.75342	0.462003784	-0.70555	2.92E-05	hypomethylated
cg09888283	0.464	0.284490811	-0.705743	0.000159	hypomethylated
cg03891318	0.63321	0.388237297	-0.705745	8.60E-06	hypomethylated
cg14177914	0.48355	0.296436757	-0.705941	8.63E-05	hypomethylated
cg26601073	0.53623	0.328723243	-0.705978	6.95E-05	hypomethylated
cg11134430	0.36246	0.222181622	-0.706082	0.00078	hypomethylated
cg05501617	0.70446	0.431677297	-0.706565	8.42E-05	hypomethylated
cg21983531	0.67529	0.413791351	-0.706604	7.47E-05	hypomethylated
cg21761639	0.35545	0.217793514	-0.706686	0.000399	hypomethylated
cg08887327	0.42702	0.261638919	-0.706726	0.000256	hypomethylated
cg09187338	0.65662	0.402316216	-0.706729	5.72E-05	hypomethylated
cg26250989	0.76662	0.469679459	-0.706835	1.67E-06	hypomethylated
cg07948480	0.70286	0.430486486	-0.707269	3.15E-05	hypomethylated
cg00502597	0.55798	0.341675135	-0.707588	6.31E-05	hypomethylated
cg02965295	0.70394	0.431045405	-0.707613	0.000112	hypomethylated
cg01550445	0.63734	0.39024	-0.707702	1.35E-05	hypomethylated
cg09555736	0.56873	0.348189189	-0.707872	0.000195	hypomethylated
cg08428188	0.80158	0.490694595	-0.708021	1.09E-05	hypomethylated

cg23903588	0.68285	0.41782973	-0.708654	1.22E-06	hypomethylated
cg26534425	0.47422	0.290132432	-0.708845	4.15E-05	hypomethylated
cg04756223	0.62736	0.383814595	-0.708884	0.000115	hypomethylated
cg23512231	0.77417	0.473576757	-0.709052	5.72E-05	hypomethylated
cg11637968	0.62586	0.382824324	-0.709157	0.000358	hypomethylated
cg14439572	0.37721	0.230711351	-0.709279	0.001481	hypomethylated
cg21860675	0.74975	0.458544865	-0.709347	6.05E-06	hypomethylated
cg04177251	0.56813	0.34742973	-0.7095	0.000365	hypomethylated
cg21462844	0.52586	0.321555135	-0.709613	0.000135	hypomethylated
cg25133376	0.51841	0.316991892	-0.709648	0.001603	hypomethylated
cg13257690	0.75263	0.460207568	-0.709656	5.32E-05	hypomethylated
cg07277633	0.3028	0.185126486	-0.709854	0.000681	hypomethylated
cg00316800	0.76254	0.466078378	-0.71024	9.07E-06	hypomethylated
cg24425972	0.73788	0.450959459	-0.710388	1.21E-05	hypomethylated
cg09942743	0.51373	0.313963243	-0.710415	5.31E-05	hypomethylated
cg13966557	0.55336	0.338134595	-0.710621	1.12E-05	hypomethylated
cg11288831	0.60501	0.369671892	-0.710714	2.21E-05	hypomethylated
cg22482700	0.57653	0.352260541	-0.710753	2.95E-06	hypomethylated
cg06333233	0.70563	0.431132973	-0.710779	6.15E-05	hypomethylated
cg22764374	0.52619	0.321442162	-0.711025	0.002965	hypomethylated
cg26870745	0.44375	0.271080541	-0.711026	0.000931	hypomethylated
cg06250720	0.77429	0.472955135	-0.711171	1.62E-05	hypomethylated
cg19017553	0.64787	0.395722162	-0.711216	1.89E-05	hypomethylated
cg08349497	0.58689	0.358447027	-0.71133	0.001839	hypomethylated
cg13859639	0.42875	0.261825405	-0.711532	0.000403	hypomethylated
cg23516310	0.5481	0.334649189	-0.71179	0.000696	hypomethylated
cg01152624	0.41176	0.251374054	-0.711968	0.000551	hypomethylated
cg00685614	0.91455	0.558279459	-0.712075	4.36E-06	hypomethylated
cg25109721	0.5624	0.343219459	-0.712465	0.004326	hypomethylated
cg18324583	0.72162	0.440287027	-0.712795	7.93E-06	hypomethylated
cg18021623	0.67482	0.411696216	-0.712923	1.15E-05	hypomethylated
cg08079052	0.51635	0.314962703	-0.713168	0.000805	hypomethylated
cg01858698	0.53917	0.328881622	-0.713172	0.002106	hypomethylated
cg08494738	0.72699	0.443399459	-0.713329	1.62E-05	hypomethylated
cg02398682	0.36822	0.224567568	-0.713418	0.001603	hypomethylated
cg12279294	0.3764	0.229552432	-0.713443	0.001107	hypomethylated
cg18413830	0.68809	0.419575135	-0.713668	1.18E-05	hypomethylated
cg01910639	0.54167	0.33020973	-0.714032	1.62E-05	hypomethylated
cg19111408	0.45018	0.274433514	-0.714045	0.001423	hypomethylated
cg08376992	0.42278	0.257717297	-0.714118	0.000287	hypomethylated
cg03501666	0.60123	0.366460541	-0.714259	0.000912	hypomethylated
cg07775417	0.61685	0.375977838	-0.714272	8.83E-06	hypomethylated
cg03354616	0.83282	0.507609189	-0.714287	6.75E-06	hypomethylated
cg13817046	0.66223	0.403622162	-0.714327	1.89E-05	hypomethylated
cg27484412	0.5395	0.328813514	-0.714353	0.003705	hypomethylated
cg14032261	0.71932	0.438397297	-0.714395	2.27E-05	hypomethylated
cg03108070	0.57443	0.350067568	-0.714498	0.006915	hypomethylated
cg16929739	0.67493	0.411306486	-0.714524	5.19E-05	hypomethylated
cg12846656	0.42751	0.260517838	-0.714576	0.005131	hypomethylated
cg07588216	0.83977	0.511680541	-0.714751	2.23E-06	hypomethylated
cg24816455	0.52548	0.320178378	-0.71476	0.000417	hypomethylated
cg22905866	0.69004	0.420318919	-0.715196	9.57E-06	hypomethylated
cg26978064	0.46863	0.285451351	-0.715204	5.50E-06	hypomethylated
cg00160981	0.54596	0.332502162	-0.715432	0.000214	hypomethylated
cg02824980	0.41594	0.253310811	-0.715467	0.000152	hypomethylated
cg15013617	0.56661	0.34506973	-0.715468	0.000588	hypomethylated
cg07875068	0.45165	0.275045405	-0.715535	4.26E-05	hypomethylated
cg10250177	0.43955	0.267671892	-0.715562	3.62E-05	hypomethylated

cg24836281	0.59579	0.362765946	-0.715765	3.07E-05	hypomethylated
cg27282397	0.6914	0.420964324	-0.715823	2.78E-05	hypomethylated
cg25744552	0.42846	0.260855676	-0.715909	2.71E-05	hypomethylated
cg07080653	0.72571	0.441800541	-0.715998	3.67E-05	hypomethylated
cg13784312	0.59097	0.359746486	-0.716104	0.000426	hypomethylated
cg07763231	0.4681	0.284935135	-0.716183	8.42E-05	hypomethylated
cg11316887	0.55314	0.336669189	-0.716313	6.75E-06	hypomethylated
cg03213374	0.51273	0.31205027	-0.716421	0.003878	hypomethylated
cg16989719	0.68016	0.413916216	-0.716535	5.32E-05	hypomethylated
cg05312104	0.51291	0.312131892	-0.71655	1.21E-05	hypomethylated
cg00761125	0.73649	0.448158378	-0.716657	0.000178	hypomethylated
cg10134799	0.71975	0.437907027	-0.716871	5.58E-06	hypomethylated
cg17696194	0.43323	0.263461081	-0.717543	0.000195	hypomethylated
cg26546105	0.23231	0.141264324	-0.717654	0.0017	hypomethylated
cg25581222	0.6731	0.409283243	-0.717721	0.000445	hypomethylated
cg02149069	0.48689	0.296052432	-0.717743	7.56E-05	hypomethylated
cg11493223	0.49566	0.301375676	-0.717788	0.002106	hypomethylated
cg16714154	0.51621	0.313777297	-0.718217	0.000626	hypomethylated
cg20658466	0.31576	0.191914054	-0.718368	2.21E-05	hypomethylated
cg26314722	0.45277	0.275172973	-0.71844	0.001734	hypomethylated
cg24988684	0.47177	0.286692432	-0.71858	0.000517	hypomethylated
cg08779649	0.68998	0.419258919	-0.718713	8.84E-05	hypomethylated
cg17758048	0.74542	0.452886486	-0.718904	4.42E-05	hypomethylated
cg04910041	0.57121	0.34700973	-0.719045	0.000464	hypomethylated
cg17494199	0.65021	0.394956216	-0.719213	2.85E-05	hypomethylated
cg08599635	0.65128	0.395573514	-0.719332	0.000912	hypomethylated
cg14695663	0.44417	0.26973027	-0.719595	0.001803	hypomethylated
cg03916189	0.42764	0.259628649	-0.719947	0.000195	hypomethylated
cg12392104	0.56453	0.342719459	-0.720022	0.00099	hypomethylated
cg00516092	0.58467	0.354905405	-0.720188	3.76E-05	hypomethylated
cg24367957	0.61582	0.373762703	-0.720386	0.00064	hypomethylated
cg10037049	0.53015	0.321744865	-0.720483	0.000459	hypomethylated
cg17318719	0.6091	0.369634054	-0.720581	3.23E-05	hypomethylated
cg21994818	0.63891	0.387652973	-0.720847	0.000107	hypomethylated
cg08374799	0.46319	0.281032432	-0.720867	0.00095	hypomethylated
cg19009471	0.64956	0.394024324	-0.721178	0.000772	hypomethylated
cg12251111	0.75085	0.455326486	-0.721623	3.69E-06	hypomethylated
cg08908264	0.41963	0.254465405	-0.721648	0.001717	hypomethylated
cg16677191	0.63867	0.387224324	-0.721901	0.000674	hypomethylated
cg11316517	0.7386	0.447737838	-0.722139	4.26E-05	hypomethylated
cg16417118	0.47294	0.286658378	-0.722325	0.000142	hypomethylated
cg26311501	0.56245	0.340897297	-0.722388	8.32E-05	hypomethylated
cg00763594	0.59716	0.361933514	-0.722393	1.12E-05	hypomethylated
cg03930313	0.63447	0.384545405	-0.722398	0.000234	hypomethylated
cg05778494	0.60054	0.363975135	-0.72242	2.04E-05	hypomethylated
cg11918450	0.76785	0.465374054	-0.722434	1.80E-05	hypomethylated
cg11667061	0.69169	0.419209189	-0.722455	0.000256	hypomethylated
cg15551096	0.55736	0.337716757	-0.722796	0.012844	hypomethylated
cg27341866	0.92735	0.561813514	-0.723023	5.72E-05	hypomethylated
cg07329251	0.58306	0.353226486	-0.723051	0.000262	hypomethylated
cg02341503	0.36329	0.219990811	-0.723678	0.000772	hypomethylated
cg21226735	0.69398	0.420204324	-0.723803	3.39E-06	hypomethylated
cg26165146	0.7699	0.466052973	-0.724177	4.82E-05	hypomethylated
cg10100887	0.62086	0.375812973	-0.724253	3.49E-06	hypomethylated
cg18773937	0.47585	0.288023784	-0.724319	0.005656	hypomethylated
cg04830629	0.44396	0.268663784	-0.724628	0.00012	hypomethylated
cg09232805	0.49953	0.302192432	-0.725104	9.05E-05	hypomethylated
cg07507251	0.4585	0.277368649	-0.725117	0.000875	hypomethylated

cg02601475	0.67498	0.408258378	-0.725362	0.000306	hypomethylated
cg13005202	0.60516	0.366001081	-0.725469	0.000268	hypomethylated
cg08275025	0.4457	0.269482703	-0.72588	0.004059	hypomethylated
cg01635061	0.24639	0.148956216	-0.726055	0.002856	hypomethylated
cg25632577	0.86217	0.521176216	-0.726201	2.57E-06	hypomethylated
cg03604067	0.63762	0.385432973	-0.726217	0.001237	hypomethylated
cg22670759	0.47256	0.285648108	-0.726259	0.002478	hypomethylated
cg27236629	0.46679	0.282114054	-0.726495	8.13E-05	hypomethylated
cg27092248	0.62999	0.380739459	-0.726525	2.54E-05	hypomethylated
cg19982230	0.45535	0.275181622	-0.726592	0.000667	hypomethylated
cg18098065	0.47682	0.288080541	-0.726973	0.004485	hypomethylated
cg02064267	0.42919	0.259224324	-0.727415	0.000313	hypomethylated
cg12491643	0.77435	0.46767027	-0.727494	3.40E-05	hypomethylated
cg15208197	0.34796	0.210121081	-0.727701	3.04E-06	hypomethylated
cg11807006	0.48	0.289790811	-0.728023	0.001301	hypomethylated
cg17232357	0.55256	0.333595135	-0.728033	1.21E-05	hypomethylated
cg00952054	0.78773	0.475522162	-0.728189	0.000109	hypomethylated
cg26962618	0.63606	0.383955676	-0.728223	0.00039	hypomethylated
cg02511456	0.35375	0.21352	-0.728359	0.000166	hypomethylated
cg16755214	0.61144	0.369054054	-0.728379	3.85E-05	hypomethylated
cg12622958	0.31151	0.188021622	-0.72838	0.000234	hypomethylated
cg05167973	0.47513	0.286776757	-0.728394	0.001074	hypomethylated
cg24333189	0.4446	0.268282162	-0.728757	0.000152	hypomethylated
cg11784887	0.44522	0.268655135	-0.728763	0.000726	hypomethylated
cg17346857	0.42579	0.25692973	-0.728768	0.006015	hypomethylated
cg14416623	0.59442	0.358641622	-0.72894	0.00134	hypomethylated
cg06160973	0.4782	0.288516216	-0.728962	0.000354	hypomethylated
cg13800005	0.69926	0.421888649	-0.728967	0.000195	hypomethylated
cg01815912	0.52612	0.317409189	-0.729048	1.35E-05	hypomethylated
cg22066894	0.56964	0.343662703	-0.729057	0.002599	hypomethylated
cg25818214	0.48833	0.294525946	-0.729462	0.000116	hypomethylated
cg17298925	0.5606	0.338070811	-0.729646	0.003914	hypomethylated
cg01513078	0.69999	0.422083243	-0.729807	1.75E-05	hypomethylated
cg00036347	0.45171	0.272364324	-0.729859	0.001875	hypomethylated
cg09896544	0.44313	0.267179459	-0.729921	6.62E-05	hypomethylated
cg13279476	0.5165	0.311394054	-0.730027	0.000653	hypomethylated
cg26575450	0.56346	0.339636216	-0.730323	0.005706	hypomethylated
cg15033552	0.7748	0.467009189	-0.730373	1.84E-05	hypomethylated
cg19145082	0.60696	0.365724865	-0.730843	0.000166	hypomethylated
cg14212038	0.50833	0.306287027	-0.730881	6.15E-05	hypomethylated
cg23704195	0.57806	0.348251892	-0.731088	1.89E-05	hypomethylated
cg16270485	0.50733	0.305628649	-0.731145	0.000287	hypomethylated
cg19773937	0.75476	0.454615676	-0.731371	1.94E-05	hypomethylated
cg16163382	0.62345	0.375522162	-0.731376	0.000306	hypomethylated
cg11961138	0.40599	0.24436973	-0.732379	0.00099	hypomethylated
cg09298313	0.427	0.256959459	-0.732695	0.000306	hypomethylated
cg16429725	0.59005	0.35507027	-0.732733	2.07E-05	hypomethylated
cg04510459	0.49489	0.297796216	-0.732782	0.000112	hypomethylated
cg20289045	0.70495	0.424115676	-0.733063	2.92E-05	hypomethylated
cg08928408	0.55074	0.331301081	-0.733228	0.000303	hypomethylated
cg14241836	0.7597	0.456985946	-0.73328	0.000123	hypomethylated
cg22691256	0.66421	0.399488108	-0.733487	1.80E-05	hypomethylated
cg25968076	0.32721	0.196755135	-0.733816	0.000242	hypomethylated
cg26690592	0.23029	0.138434595	-0.734247	0.000382	hypomethylated
cg19861260	0.49536	0.297767568	-0.734291	0.000696	hypomethylated
cg06808585	0.38915	0.233922162	-0.734298	0.000127	hypomethylated
cg00184457	0.59771	0.359278378	-0.734344	0.001571	hypomethylated
cg16209064	0.81738	0.491315135	-0.734358	4.48E-06	hypomethylated

cg00073460	0.57267	0.344213514	-0.7344	0.0002	hypomethylated
cg21513352	0.35568	0.213786486	-0.734409	0.001526	hypomethylated
cg04468741	0.76598	0.460364865	-0.734529	1.80E-05	hypomethylated
cg04212150	0.3379	0.20308	-0.734548	0.001635	hypomethylated
cg00424451	0.46921	0.281964324	-0.734721	0.000115	hypomethylated
cg03821121	0.68133	0.409308108	-0.735166	9.49E-05	hypomethylated
cg16434331	0.60322	0.362383243	-0.735168	0.000268	hypomethylated
cg20488673	0.59383	0.356684324	-0.735402	0.000287	hypomethylated
ch.2.20642108R	0.31529	0.18936	-0.735548	0.001041	hypomethylated
cg05279738	0.62405	0.374760541	-0.735693	1.80E-05	hypomethylated
cg20911180	0.65408	0.392763784	-0.735805	0.001301	hypomethylated
cg01781725	0.32426	0.194703243	-0.735874	6.01E-05	hypomethylated
cg17799287	0.49424	0.296587568	-0.736754	0.000317	hypomethylated
cg08698997	0.45587	0.27354	-0.736871	0.000893	hypomethylated
cg12377816	0.41443	0.248673514	-0.736876	6.78E-05	hypomethylated
cg00339782	0.64268	0.385578919	-0.737074	6.75E-06	hypomethylated
cg11778563	0.39977	0.239841622	-0.737088	0.003506	hypomethylated
cg04324821	0.38692	0.232073514	-0.737453	0.00291	hypomethylated
cg14939821	0.64344	0.385872973	-0.73768	0.0006	hypomethylated
cg25336892	0.72083	0.43218973	-0.737994	8.15E-06	hypomethylated
cg17260706	0.36423	0.218289189	-0.738609	3.67E-05	hypomethylated
cg26550194	0.60207	0.360779459	-0.738814	0.000129	hypomethylated
cg20629315	0.6943	0.415944324	-0.739169	0.000191	hypomethylated
cg04322486	0.45917	0.275064324	-0.739259	0.001212	hypomethylated
cg27543334	0.31202	0.186894595	-0.739414	0.000733	hypomethylated
cg07340025	0.50426	0.302020541	-0.739521	0.003441	hypomethylated
cg16308270	0.66035	0.395494595	-0.739573	8.15E-06	hypomethylated
cg26203572	0.84902	0.508487568	-0.739586	1.01E-05	hypomethylated
cg19791637	0.69269	0.414766486	-0.739911	1.15E-05	hypomethylated
cg02747950	0.60126	0.360016216	-0.739927	0.000209	hypomethylated
cg12422154	0.7098	0.424997297	-0.739959	3.30E-06	hypomethylated
cg20359363	0.46454	0.278139459	-0.739994	2.30E-05	hypomethylated
cg04031093	0.71908	0.430523243	-0.740061	6.95E-05	hypomethylated
cg19676285	0.54935	0.328886486	-0.740136	2.04E-05	hypomethylated
cg22813430	0.44832	0.268366486	-0.740324	0.00028	hypomethylated
cg14370448	0.38736	0.231857838	-0.740434	0.002007	hypomethylated
cg14854503	0.71687	0.429085405	-0.740447	0.000137	hypomethylated
cg15876968	0.35479	0.212357297	-0.740472	0.000426	hypomethylated
cg15756114	0.59342	0.355183243	-0.74049	8.42E-05	hypomethylated
cg08035555	0.56286	0.336779459	-0.740972	0.000506	hypomethylated
cg26158270	0.69828	0.417723243	-0.741258	1.54E-05	hypomethylated
cg08554554	0.57354	0.343083784	-0.741333	0.000178	hypomethylated
cg24174557	0.53868	0.322136757	-0.741755	0.002699	hypomethylated
cg12549411	0.43567	0.260525946	-0.741809	0.000667	hypomethylated
cg08428292	0.57633	0.34463027	-0.741846	0.0017	hypomethylated
cg18458509	0.60643	0.362613514	-0.741908	0.00096	hypomethylated
cg20515580	0.5057	0.302382162	-0.741909	0.000327	hypomethylated
cg07830472	0.73254	0.438004865	-0.741961	1.82E-05	hypomethylated
cg00159243	0.57949	0.346458378	-0.742102	0.002455	hypomethylated
cg24995678	0.74567	0.445716216	-0.742412	0.000142	hypomethylated
cg20545458	0.5369	0.320877297	-0.742632	0.000293	hypomethylated
cg11761483	0.63488	0.379403784	-0.74275	0.000653	hypomethylated
cg23954655	0.40985	0.244878378	-0.743031	0.000224	hypomethylated
cg01300291	0.32637	0.19498	-0.743182	0.000182	hypomethylated
cg26687579	0.40602	0.242523784	-0.743425	5.07E-06	hypomethylated
cg02505956	0.84981	0.507604865	-0.743434	2.57E-06	hypomethylated
cg16542322	0.3837	0.229183784	-0.743474	0.000464	hypomethylated
cg18608055	0.59391	0.354726486	-0.743537	0.000256	hypomethylated

cg18661868	0.34979	0.208901081	-0.743669	0.00134	hypomethylated
cg14074117	0.61233	0.365574054	-0.744146	3.00E-05	hypomethylated
cg24618514	0.42528	0.253879459	-0.744269	0.002026	hypomethylated
cg10324998	0.2773	0.165535135	-0.74431	5.65E-05	hypomethylated
cg20445727	0.78259	0.467030811	-0.744739	5.00E-06	hypomethylated
cg04312209	0.38263	0.228343784	-0.744742	0.000204	hypomethylated
cg25247520	0.77893	0.464826486	-0.744801	8.42E-05	hypomethylated
cg27326514	0.58923	0.351574054	-0.745002	0.000185	hypomethylated
cg12840818	0.44462	0.265247568	-0.745233	1.26E-05	hypomethylated
cg02142926	0.44928	0.268001081	-0.745376	4.70E-05	hypomethylated
cg11998200	0.54537	0.325268108	-0.745606	0.00094	hypomethylated
cg14607660	0.62984	0.375627568	-0.745682	1.18E-05	hypomethylated
cg17375585	0.50177	0.29921027	-0.745867	0.000399	hypomethylated
cg00590817	0.56875	0.339115676	-0.746017	0.000268	hypomethylated
cg16411857	0.37591	0.224098919	-0.746252	0.006395	hypomethylated
cg25296938	0.54742	0.326334595	-0.746296	0.00513	hypomethylated
cg19050555	0.80435	0.479476757	-0.746363	4.58E-05	hypomethylated
cg02107844	0.50309	0.299868108	-0.746488	0.000207	hypomethylated
cg09920804	0.56635	0.33752973	-0.746679	3.12E-06	hypomethylated
cg17052170	0.63357	0.377397297	-0.74742	9.95E-05	hypomethylated
cg24820936	0.54036	0.321758919	-0.747941	0.001949	hypomethylated
cg26271591	0.44044	0.262239459	-0.748061	0.003225	hypomethylated
cg23350385	0.732	0.435764324	-0.748296	6.95E-05	hypomethylated
cg01441777	0.43659	0.259877838	-0.748445	0.000129	hypomethylated
cg18842353	0.30474	0.181345946	-0.748834	0.000117	hypomethylated
cg21650866	0.46915	0.279176216	-0.748873	0.00095	hypomethylated
cg18082515	0.44832	0.266760541	-0.748984	0.000245	hypomethylated
cg06422467	0.48389	0.287863784	-0.749293	0.000142	hypomethylated
cg05117982	0.41875	0.249111351	-0.749298	0.001314	hypomethylated
cg00058887	0.60137	0.357747568	-0.749311	2.89E-05	hypomethylated
cg13942186	0.38028	0.226203243	-0.749442	0.001368	hypomethylated
cg09229893	0.63069	0.375137297	-0.749512	0.000327	hypomethylated
cg10950358	0.31741	0.188795676	-0.749522	0.000209	hypomethylated
cg08824847	0.59047	0.35115027	-0.749775	5.86E-05	hypomethylated
cg14550985	0.51097	0.303861081	-0.749827	0.000123	hypomethylated
cg26541218	0.54682	0.325159459	-0.749919	0.00024	hypomethylated
cg17583957	0.86938	0.516926486	-0.750028	9.38E-07	hypomethylated
cg06126421	0.58529	0.347977838	-0.750156	6.15E-05	hypomethylated
cg06278108	0.37479	0.22282	-0.750204	0.000209	hypomethylated
cg05152300	0.48477	0.288187027	-0.750295	0.000756	hypomethylated
cg09958560	0.65562	0.389696216	-0.75051	0.000191	hypomethylated
cg15298059	0.30519	0.181368108	-0.750787	0.000209	hypomethylated
cg09108532	0.38533	0.228975135	-0.750904	3.59E-06	hypomethylated
cg08866794	0.60152	0.357423784	-0.750977	0.000166	hypomethylated
cg00523604	0.52814	0.313815135	-0.751005	0.000408	hypomethylated
cg07773593	0.42821	0.254435676	-0.751018	0.001031	hypomethylated
cg02595280	0.41385	0.245891892	-0.751084	0.008783	hypomethylated
cg17631150	0.45369	0.269557297	-0.751115	4.36E-05	hypomethylated
cg06606386	0.55397	0.329116216	-0.751211	0.0017	hypomethylated
cg03504039	0.4772	0.283472432	-0.751386	2.61E-05	hypomethylated
cg10142520	0.64854	0.385103784	-0.751948	0.003773	hypomethylated
cg02349468	0.81517	0.484048649	-0.751949	5.21E-06	hypomethylated
cg18798289	0.53765	0.319204865	-0.752185	0.003136	hypomethylated
cg02632362	0.50302	0.29863027	-0.752255	0.003195	hypomethylated
cg00044372	0.78868	0.468162703	-0.75243	1.72E-06	hypomethylated
cg23466749	0.52865	0.313807568	-0.752433	0.000216	hypomethylated
cg25124300	0.70752	0.419891892	-0.752753	5.58E-05	hypomethylated
cg26839512	0.35816	0.212502703	-0.753123	0.001667	hypomethylated

cg19543867	0.80091	0.475062162	-0.753524	4.36E-06	hypomethylated
cg24687805	0.42034	0.249306486	-0.753636	0.007035	hypomethylated
cg17290213	0.49189	0.291732973	-0.753687	0.001118	hypomethylated
cg19767205	0.54367	0.322422703	-0.753778	6.78E-05	hypomethylated
cg19867107	0.57113	0.338687568	-0.753864	0.006339	hypomethylated
cg05191397	0.42885	0.254277838	-0.754067	0.001288	hypomethylated
cg19821297	0.59342	0.351804324	-0.75428	0.00012	hypomethylated
cg11278749	0.73287	0.434447027	-0.754377	2.45E-05	hypomethylated
cg02854902	0.81665	0.484079459	-0.754474	7.72E-06	hypomethylated
cg01421539	0.23295	0.138062162	-0.754702	0.000335	hypomethylated
cg19845249	0.34648	0.205338378	-0.754769	0.001511	hypomethylated
cg10437931	0.59841	0.354617838	-0.754869	0.000219	hypomethylated
cg09740450	0.43937	0.260326486	-0.755114	5.58E-06	hypomethylated
cg11838152	0.49536	0.293495676	-0.755138	0.00255	hypomethylated
cg01786048	0.59963	0.355203784	-0.755426	0.000653	hypomethylated
cg15858483	0.49852	0.295308108	-0.75543	0.001511	hypomethylated
cg25628433	0.41912	0.248255676	-0.755537	4.31E-05	hypomethylated
cg27171194	0.37401	0.221526486	-0.755598	0.000313	hypomethylated
cg02789515	0.82485	0.488504865	-0.755759	8.83E-06	hypomethylated
cg19169932	0.58108	0.344107568	-0.755877	8.63E-05	hypomethylated
cg24127989	0.26877	0.159135676	-0.756115	0.002274	hypomethylated
cg20627046	0.55727	0.329865405	-0.756499	4.15E-05	hypomethylated
cg21490635	0.55106	0.326175135	-0.756563	1.12E-05	hypomethylated
cg07011538	0.56948	0.336991351	-0.756934	0.000115	hypomethylated
cg15033653	0.26265	0.155410811	-0.757055	0.000226	hypomethylated
cg18098839	0.5033	0.297774054	-0.757201	0.003571	hypomethylated
cg11854392	0.56653	0.335165946	-0.757277	0.00421	hypomethylated
cg24439686	0.60612	0.35856	-0.757389	0.000674	hypomethylated
cg14329059	0.35087	0.207535676	-0.757577	0.002147	hypomethylated
cg07863022	0.48426	0.286418919	-0.757655	0.000653	hypomethylated
cg25962699	0.46947	0.277627027	-0.757885	4.05E-05	hypomethylated
cg12138695	0.2447	0.144704865	-0.757901	1.31E-05	hypomethylated
cg15887927	0.62748	0.370992432	-0.75818	3.95E-05	hypomethylated
cg24402880	0.64197	0.379548649	-0.758221	0.000262	hypomethylated
cg16172099	0.49185	0.29077027	-0.758339	0.000613	hypomethylated
cg24450239	0.72892	0.430751892	-0.758903	7.47E-05	hypomethylated
cg00622799	0.48202	0.284727568	-0.759511	0.000159	hypomethylated
cg20045394	0.42764	0.252594054	-0.759576	0.001875	hypomethylated
cg17117459	0.67562	0.399058378	-0.759612	0.0002	hypomethylated
cg03851496	0.29498	0.17423027	-0.759622	0.000449	hypomethylated
cg07447769	0.62583	0.369631351	-0.759684	2.39E-05	hypomethylated
cg13632630	0.63122	0.372783243	-0.759806	2.71E-05	hypomethylated
cg24904788	0.64361	0.379886486	-0.760618	0.000435	hypomethylated
cg25432336	0.62679	0.36994973	-0.760653	9.72E-05	hypomethylated
cg06733329	0.28238	0.166654595	-0.760777	0.000613	hypomethylated
cg03078551	0.28615	0.168874595	-0.760819	0.003773	hypomethylated
cg05783290	0.44324	0.261555676	-0.76097	0.006122	hypomethylated
cg23367358	0.67166	0.396322703	-0.761056	7.65E-05	hypomethylated
cg20311002	0.78115	0.460903784	-0.761134	9.82E-06	hypomethylated
cg27285720	0.6437	0.37979027	-0.761186	0.001395	hypomethylated
cg18758433	0.7161	0.422430811	-0.761446	0.000182	hypomethylated
cg11610614	0.4003	0.236120541	-0.761558	0.00591	hypomethylated
cg09449150	0.57339	0.338204865	-0.761619	9.49E-05	hypomethylated
cg02641539	0.45074	0.265854595	-0.761658	0.000382	hypomethylated
cg08243465	0.56564	0.333623243	-0.761664	4.93E-06	hypomethylated
cg23934955	0.59811	0.352675135	-0.762071	2.10E-05	hypomethylated
cg04270489	0.52877	0.311734054	-0.762325	5.72E-05	hypomethylated
cg06879394	0.67753	0.399432973	-0.762331	0.002599	hypomethylated

cg07956751	0.48115	0.283641622	-0.762417	0.007158	hypomethylated
cg10778971	0.6401	0.377302703	-0.762575	2.45E-05	hypomethylated
cg04504205	0.45351	0.267264865	-0.762864	0.00032	hypomethylated
cg07480754	0.30262	0.178340541	-0.762873	0.000696	hypomethylated
cg06780032	0.48684	0.286850811	-0.763147	0.00064	hypomethylated
cg19945931	0.76515	0.450711351	-0.763539	4.15E-05	hypomethylated
cg24310959	0.55534	0.327051892	-0.763852	3.23E-05	hypomethylated
cg15132013	0.71684	0.422103784	-0.764053	1.80E-05	hypomethylated
cg05696969	0.77605	0.456884865	-0.764319	1.75E-05	hypomethylated
cg18702012	0.48867	0.287666486	-0.764463	1.64E-05	hypomethylated
cg19267254	0.56128	0.330327027	-0.764826	0.000159	hypomethylated
cg19236431	0.36483	0.21470973	-0.764837	0.003166	hypomethylated
cg08056069	0.5992	0.352626486	-0.764897	0.000293	hypomethylated
cg08409642	0.51063	0.300425946	-0.765269	0.007158	hypomethylated
cg00375457	0.29345	0.172648108	-0.76528	0.000884	hypomethylated
cg15916554	0.5211	0.30652973	-0.765533	0.000166	hypomethylated
cg20513976	0.70895	0.416983784	-0.765693	0.0003	hypomethylated
cg09315887	0.44708	0.262938919	-0.765805	0.000653	hypomethylated
cg01714284	0.51148	0.300804865	-0.76585	0.001141	hypomethylated
cg13427748	0.51181	0.300929189	-0.766184	0.000626	hypomethylated
cg12386646	0.33465	0.196743784	-0.766335	0.00031	hypomethylated
cg25565203	0.59824	0.351708649	-0.766344	1.12E-05	hypomethylated
cg16567330	0.47923	0.28169027	-0.766608	0.000435	hypomethylated
cg27116114	0.76579	0.450102703	-0.766695	8.60E-06	hypomethylated
cg13787850	0.45212	0.265716216	-0.766819	0.00035	hypomethylated
cg19627006	0.65358	0.384035676	-0.767123	1.80E-05	hypomethylated
cg27149179	0.44042	0.258784865	-0.767127	0.000176	hypomethylated
cg12761965	0.51151	0.300534054	-0.767234	0.000789	hypomethylated
cg01484075	0.75513	0.44367027	-0.767237	2.15E-05	hypomethylated
cg11868461	0.40809	0.239761081	-0.76729	0.000848	hypomethylated
cg16588163	0.55551	0.326361081	-0.767344	0.001395	hypomethylated
cg00778190	0.60434	0.354984324	-0.767605	0.000104	hypomethylated
cg10459087	0.54398	0.31952	-0.767647	0.000346	hypomethylated
cg24540678	0.30823	0.181037297	-0.76772	0.000303	hypomethylated
cg22635096	0.60075	0.352843243	-0.767737	0.000399	hypomethylated
cg13065206	0.43471	0.25516973	-0.768596	0.000245	hypomethylated
cg14152591	0.59363	0.348331351	-0.769104	9.49E-05	hypomethylated
cg14171514	0.37648	0.220859459	-0.769445	0.007158	hypomethylated
cg24939196	0.50388	0.295561081	-0.769624	0.0002	hypomethylated
cg01282174	0.65309	0.383069189	-0.769677	3.23E-05	hypomethylated
cg06354695	0.75682	0.443868649	-0.769817	2.71E-06	hypomethylated
cg21052873	0.41378	0.242668108	-0.769879	0.000626	hypomethylated
cg25132536	0.49249	0.288761622	-0.770215	9.60E-05	hypomethylated
cg10184289	0.56343	0.330336216	-0.770301	5.32E-05	hypomethylated
cg27411547	0.87279	0.511704865	-0.770323	2.45E-05	hypomethylated
cg25773259	0.75883	0.444869189	-0.770396	1.15E-05	hypomethylated
cg10191240	0.60791	0.35630973	-0.770726	0.001987	hypomethylated
cg21145248	0.6987	0.40949027	-0.770844	1.42E-05	hypomethylated
cg07589202	0.68413	0.400928108	-0.770927	9.82E-06	hypomethylated
cg26192309	0.75273	0.441115135	-0.770977	8.37E-06	hypomethylated
cg04961042	0.84523	0.495321081	-0.77098	1.54E-06	hypomethylated
cg23680451	0.60273	0.353104865	-0.771415	0.001452	hypomethylated
cg23841186	0.60121	0.352182703	-0.771545	0.000129	hypomethylated
cg19405484	0.50563	0.296131351	-0.771845	0.000474	hypomethylated
cg24709951	0.63319	0.370838378	-0.771848	0.000191	hypomethylated
cg14969094	0.38373	0.224732973	-0.77188	0.000132	hypomethylated
cg07480640	0.50349	0.294716216	-0.772637	0.000893	hypomethylated
cg22018051	0.43811	0.256425405	-0.772754	0.000495	hypomethylated

cg25953130	0.41059	0.240264324	-0.773076	9.27E-05	hypomethylated
cg25310081	0.47193	0.276154595	-0.773097	0.004059	hypomethylated
cg14189116	0.78793	0.461032973	-0.773198	1.99E-05	hypomethylated
cg21005416	0.57005	0.333513514	-0.773343	0.001734	hypomethylated
cg25015038	0.45192	0.264364324	-0.77354	0.000374	hypomethylated
cg26318265	0.38047	0.222505405	-0.773942	0.000772	hypomethylated
cg06822193	0.62208	0.363800541	-0.773952	5.89E-06	hypomethylated
cg26580869	0.5668	0.331462162	-0.773996	8.13E-05	hypomethylated
cg07086918	0.44539	0.260426486	-0.774193	0.000741	hypomethylated
cg04890576	0.53003	0.309859459	-0.77446	0.000163	hypomethylated
cg06578276	0.46601	0.272356216	-0.774866	1.18E-05	hypomethylated
cg03447547	0.66744	0.390045405	-0.774996	1.54E-05	hypomethylated
cg16849268	0.43073	0.251680541	-0.77519	0.007933	hypomethylated
cg10732611	0.31718	0.185251351	-0.775818	0.000528	hypomethylated
cg14189441	0.54692	0.319390811	-0.776007	3.76E-05	hypomethylated
cg05033401	0.25094	0.146535135	-0.776096	0.0017	hypomethylated
cg24435571	0.60615	0.353951351	-0.776124	0.000464	hypomethylated
cg08697732	0.63894	0.373072432	-0.776225	2.51E-05	hypomethylated
cg05088820	0.81237	0.474278378	-0.776403	1.35E-05	hypomethylated
cg21282907	0.45752	0.267074054	-0.776595	0.000229	hypomethylated
cg26680608	0.72193	0.421402162	-0.776661	0.0003	hypomethylated
cg04749549	0.2905	0.169522703	-0.77706	5.79E-05	hypomethylated
cg25423573	0.50864	0.296767568	-0.777311	3.57E-05	hypomethylated
cg21449597	0.53577	0.312595676	-0.777316	3.39E-06	hypomethylated
cg03171003	0.47666	0.278037297	-0.777682	0.002363	hypomethylated
cg13066703	0.6541	0.381468649	-0.777947	0.00099	hypomethylated
cg18081863	0.59955	0.349557297	-0.778351	0.000106	hypomethylated
cg06438559	0.66634	0.388377838	-0.778798	1.09E-05	hypomethylated
cg20890989	0.42415	0.247207027	-0.778855	0.003843	hypomethylated
cg13563026	0.37469	0.218364324	-0.77896	0.000408	hypomethylated
cg22365276	0.56708	0.330464324	-0.779058	0.00095	hypomethylated
cg14397690	0.49033	0.285658919	-0.779459	0.000306	hypomethylated
cg02832512	0.65529	0.381760541	-0.779466	8.03E-05	hypomethylated
cg15867307	0.71963	0.419123243	-0.779881	4.10E-05	hypomethylated
cg06728055	0.60493	0.352292432	-0.779995	0.000283	hypomethylated
cg25588844	0.672	0.391323243	-0.7801	0.000435	hypomethylated
cg07006935	0.74361	0.432904324	-0.780498	1.75E-05	hypomethylated
cg20042662	0.80363	0.467737838	-0.780831	1.42E-05	hypomethylated
cg27109748	0.76167	0.443285405	-0.78093	5.45E-05	hypomethylated
cg08900316	0.81343	0.473406486	-0.780939	1.88E-06	hypomethylated
cg03128029	0.5167	0.300690811	-0.781046	0.000102	hypomethylated
cg18460175	0.38396	0.223414054	-0.781236	0.001587	hypomethylated
cg26232553	0.71064	0.413463784	-0.781358	0.000232	hypomethylated
cg22367631	0.60114	0.349743784	-0.781403	0.00099	hypomethylated
cg11825899	0.52234	0.303887568	-0.781452	0.000528	hypomethylated
cg08928958	0.42958	0.249918919	-0.781467	5.45E-05	hypomethylated
cg08951051	0.34631	0.201418378	-0.781869	9.72E-05	hypomethylated
cg04121938	0.51288	0.298259459	-0.782053	0.003255	hypomethylated
cg21314318	0.52302	0.30414973	-0.782084	0.000145	hypomethylated
cg02468320	0.56814	0.330346486	-0.782266	0.000772	hypomethylated
cg22007110	0.57633	0.335102703	-0.782292	0.000506	hypomethylated
cg25072336	0.49702	0.28894973	-0.782485	0.004863	hypomethylated
cg07210669	0.56537	0.328572432	-0.782984	0.000382	hypomethylated
cg04416111	0.64867	0.376914054	-0.783249	9.31E-06	hypomethylated
cg21474062	0.42518	0.247042703	-0.783313	0.000103	hypomethylated
cg27022584	0.41684	0.242147568	-0.783607	1.80E-05	hypomethylated
cg00522451	0.65156	0.378496216	-0.783619	6.62E-05	hypomethylated
cg24178897	0.65369	0.379667568	-0.78387	9.27E-05	hypomethylated

cg12674357	0.39913	0.231794054	-0.784015	0.000805	hypomethylated
cg24283921	0.3158	0.183355135	-0.78437	0.00035	hypomethylated
cg22610620	0.43038	0.249815135	-0.78475	0.000245	hypomethylated
cg12656085	0.66913	0.388338378	-0.784972	0.00032	hypomethylated
cg27592794	0.47002	0.272772973	-0.785021	0.000109	hypomethylated
cg17738521	0.63073	0.366014595	-0.785121	0.000495	hypomethylated
cg23261443	0.68214	0.39578973	-0.785334	5.45E-05	hypomethylated
cg14457284	0.39723	0.230440541	-0.78558	4.47E-05	hypomethylated
cg05406088	0.53393	0.309668108	-0.785928	0.000789	hypomethylated
cg03984209	0.46891	0.271842703	-0.786539	0.000495	hypomethylated
cg05750047	0.58062	0.336587027	-0.786615	1.54E-05	hypomethylated
cg18146074	0.57522	0.333437297	-0.786698	9.69E-06	hypomethylated
cg20656868	0.45873	0.265902703	-0.786747	0.000219	hypomethylated
cg02931058	0.64758	0.375263784	-0.787153	7.47E-05	hypomethylated
cg04627863	0.65556	0.379830811	-0.787371	2.78E-05	hypomethylated
cg04914221	0.79068	0.458071892	-0.78752	2.68E-06	hypomethylated
cg15833534	0.31396	0.181870811	-0.787667	0.000696	hypomethylated
cg16210447	0.47651	0.275989189	-0.787895	0.00421	hypomethylated
cg02582848	0.61505	0.3562	-0.788016	6.95E-05	hypomethylated
cg18643093	0.48096	0.278489189	-0.788296	0.001734	hypomethylated
cg03168497	0.47585	0.275473514	-0.788593	0.002147	hypomethylated
cg21452188	0.65064	0.376633514	-0.788698	2.85E-05	hypomethylated
cg22888181	0.54262	0.3141	-0.788718	0.004445	hypomethylated
cg18538668	0.42315	0.244938378	-0.78875	0.000107	hypomethylated
cg16912957	0.45969	0.266074054	-0.788833	0.000122	hypomethylated
cg05695106	0.76556	0.442903784	-0.789522	7.72E-06	hypomethylated
cg01117384	0.51561	0.298265405	-0.789684	3.85E-06	hypomethylated
cg01464985	0.27184	0.157244865	-0.789745	0.000772	hypomethylated
cg26995224	0.47829	0.276636757	-0.789893	0.000142	hypomethylated
cg15826479	0.4807	0.278012432	-0.789987	0.000342	hypomethylated
cg16888658	0.69029	0.399193514	-0.790114	0.000229	hypomethylated
cg14434062	0.50774	0.293595135	-0.790262	0.000303	hypomethylated
cg26052635	0.40682	0.235229189	-0.790324	0.000386	hypomethylated
cg24181174	0.41828	0.241735135	-0.791042	1.89E-05	hypomethylated
cg13298859	0.52122	0.301225405	-0.791049	4.00E-05	hypomethylated
cg19160624	0.45883	0.265147027	-0.791167	1.38E-05	hypomethylated
cg09432376	0.58495	0.337973514	-0.791403	0.000327	hypomethylated
cg04962621	0.50472	0.291597838	-0.791503	3.19E-05	hypomethylated
cg11724602	0.7639	0.441312973	-0.791582	1.71E-05	hypomethylated
cg17453456	0.54514	0.314909189	-0.791691	2.21E-05	hypomethylated
cg24408469	0.43865	0.253303243	-0.792205	0.003255	hypomethylated
cg26361436	0.59033	0.340853514	-0.79237	0.000214	hypomethylated
cg12591668	0.61875	0.357235676	-0.79248	0.001875	hypomethylated
cg20092122	0.51524	0.297463784	-0.792531	0.003195	hypomethylated
cg16849024	0.42372	0.244507027	-0.793235	0.001875	hypomethylated
cg18749404	0.57988	0.334572432	-0.793436	5.28E-06	hypomethylated
cg26775866	0.39183	0.226062703	-0.793505	0.000219	hypomethylated
cg26854588	0.65414	0.377396757	-0.793517	2.85E-05	hypomethylated
cg15026265	0.77999	0.449974054	-0.793614	3.95E-05	hypomethylated
cg03818307	0.63164	0.364367027	-0.79371	3.57E-05	hypomethylated
cg09451427	0.57534	0.331681081	-0.794618	0.006015	hypomethylated
cg19852660	0.42934	0.247476757	-0.794828	0.000551	hypomethylated
cg04255391	0.56479	0.325454595	-0.795258	0.000365	hypomethylated
cg15985132	0.77581	0.447051351	-0.795263	1.54E-05	hypomethylated
cg05033369	0.48996	0.282330811	-0.795277	0.000142	hypomethylated
cg23288838	0.51079	0.294309189	-0.795398	0.000123	hypomethylated
cg13181928	0.27046	0.155816216	-0.79557	0.00071	hypomethylated
cg06469726	0.63097	0.363508649	-0.795582	5.93E-05	hypomethylated

cg04349084	0.55168	0.317791351	-0.795752	0.000435	hypomethylated
cg10825839	0.40269	0.231923243	-0.796022	0.004608	hypomethylated
cg03000596	0.51953	0.299191351	-0.796139	0.000912	hypomethylated
cg02920600	0.71813	0.41348	-0.796427	4.36E-05	hypomethylated
cg11798406	0.70446	0.405567027	-0.796577	1.99E-05	hypomethylated
cg03310376	0.6123	0.352432973	-0.79689	0.001857	hypomethylated
cg10344477	0.64921	0.373597297	-0.797201	8.22E-05	hypomethylated
cg02397720	0.53131	0.305704324	-0.797417	0.004485	hypomethylated
cg17654419	0.71818	0.413155135	-0.797662	2.36E-06	hypomethylated
cg11636127	0.33428	0.192272432	-0.797905	0.000365	hypomethylated
cg27183818	0.70706	0.406662703	-0.798	5.32E-05	hypomethylated
cg09220326	0.59456	0.341955135	-0.798015	0.000335	hypomethylated
cg11857093	0.39628	0.227909189	-0.798061	0.00032	hypomethylated
cg03962527	0.46608	0.268042703	-0.798115	1.01E-05	hypomethylated
cg12146829	0.51131	0.29404	-0.798186	0.000417	hypomethylated
cg25885280	0.55205	0.317374054	-0.798615	0.000271	hypomethylated
cg13410764	0.64239	0.369272973	-0.798762	0.0003	hypomethylated
cg16409012	0.57231	0.328981081	-0.798792	0.000563	hypomethylated
cg24251035	0.35422	0.203602703	-0.798889	3.53E-05	hypomethylated
cg06828335	0.76478	0.439416216	-0.799457	1.45E-06	hypomethylated
cg25946059	0.65221	0.374731351	-0.79948	0.001912	hypomethylated
cg09238801	0.57427	0.329902703	-0.799689	0.000805	hypomethylated
cg20168495	0.57261	0.328888649	-0.799954	2.45E-05	hypomethylated
cg25347526	0.56367	0.323727027	-0.800073	5.19E-05	hypomethylated
cg03257179	0.28624	0.164382703	-0.800167	0.00032	hypomethylated
cg10558233	0.49024	0.281488108	-0.800414	0.004526	hypomethylated
cg24522654	0.55241	0.317171351	-0.800477	5.72E-05	hypomethylated
cg16071681	0.51685	0.296749189	-0.800502	0.002046	hypomethylated
cg27594683	0.42361	0.243205405	-0.800561	4.15E-05	hypomethylated
cg22772930	0.63934	0.367017297	-0.800735	2.79E-06	hypomethylated
cg19291696	0.72887	0.418387027	-0.800823	2.57E-05	hypomethylated
cg17571559	0.72325	0.415108649	-0.801005	1.99E-05	hypomethylated
cg08382534	0.8367	0.480212973	-0.801036	9.82E-06	hypomethylated
cg00294382	0.42109	0.241671892	-0.801079	0.001176	hypomethylated
cg15878619	0.31088	0.178374595	-0.801448	0.002253	hypomethylated
cg21289919	0.39613	0.227265946	-0.801592	0.000224	hypomethylated
cg03055671	0.56539	0.324372973	-0.801593	0.001452	hypomethylated
cg06545761	0.73454	0.421415135	-0.801599	7.47E-05	hypomethylated
cg21330896	0.58121	0.333444865	-0.801611	0.005606	hypomethylated
cg06783423	0.36445	0.209087027	-0.801617	0.001096	hypomethylated
cg04396998	0.36531	0.209573514	-0.801665	0.001893	hypomethylated
cg21699330	0.52316	0.300097838	-0.801819	0.000696	hypomethylated
cg00108282	0.73113	0.419136757	-0.802707	1.25E-05	hypomethylated
cg14163444	0.37408	0.214395135	-0.803075	0.002829	hypomethylated
cg10321156	0.41668	0.238793514	-0.803176	0.000517	hypomethylated
cg23289024	0.46943	0.26892973	-0.803681	0.003571	hypomethylated
cg15474318	0.31477	0.180325405	-0.803695	0.000191	hypomethylated
cg09240693	0.65405	0.374591351	-0.804083	1.31E-05	hypomethylated
cg13464117	0.62428	0.357437297	-0.804503	5.43E-06	hypomethylated
cg17416730	0.40581	0.232177838	-0.805574	0.005223	hypomethylated
cg05307752	0.55336	0.316576757	-0.805663	0.000224	hypomethylated
cg12810800	0.55107	0.315265405	-0.805669	2.15E-05	hypomethylated
cg10397389	0.63208	0.361453514	-0.806297	0.000575	hypomethylated
cg20031845	0.59094	0.337882162	-0.806491	0.000718	hypomethylated
cg08371391	0.35424	0.202503784	-0.806778	0.000875	hypomethylated
cg14140118	0.79446	0.454155135	-0.806789	6.39E-06	hypomethylated
cg03731202	0.52813	0.301876216	-0.806936	9.95E-05	hypomethylated
cg14870271	0.3087	0.176404324	-0.80732	1.50E-05	hypomethylated

cg07621749	0.38796	0.221692432	-0.807348	0.001141	hypomethylated
cg22734058	0.68252	0.389999459	-0.807399	5.65E-06	hypomethylated
cg14611816	0.44143	0.252227027	-0.807462	5.07E-06	hypomethylated
cg04555941	0.48655	0.277965405	-0.807683	0.000365	hypomethylated
cg08272572	0.48624	0.277776757	-0.807743	0.001368	hypomethylated
cg24796998	0.6968	0.397934595	-0.808213	3.57E-05	hypomethylated
cg04424621	0.46121	0.263352432	-0.808429	0.000756	hypomethylated
cg24503796	0.29877	0.170588649	-0.808514	0.001212	hypomethylated
cg27067618	0.43659	0.249238919	-0.80875	2.78E-05	hypomethylated
cg17605604	0.61923	0.353477297	-0.808858	5.72E-05	hypomethylated
cg12492380	0.81241	0.463645405	-0.809186	2.71E-06	hypomethylated
cg05956608	0.5863	0.334579459	-0.80929	0.001074	hypomethylated
cg26188212	0.37982	0.216679459	-0.809753	2.10E-05	hypomethylated
cg09213124	0.28036	0.159841622	-0.810637	0.00071	hypomethylated
cg15564590	0.17572	0.100170811	-0.810816	0.000805	hypomethylated
cg15543045	0.46894	0.267316216	-0.810856	0.010042	hypomethylated
cg10011232	0.23758	0.135414054	-0.811036	0.001452	hypomethylated
cg26099834	0.43964	0.250490811	-0.811565	0.0012	hypomethylated
cg03240301	0.64523	0.367597838	-0.811685	9.27E-05	hypomethylated
cg21398275	0.29042	0.16544	-0.811833	0.004248	hypomethylated
cg10460033	0.41474	0.236220541	-0.812073	0.000417	hypomethylated
cg04147593	0.61247	0.348793514	-0.812266	6.78E-05	hypomethylated
cg16799831	0.44272	0.252057838	-0.81264	4.36E-05	hypomethylated
cg23138413	0.58929	0.335470811	-0.812791	1.31E-05	hypomethylated
cg14660676	0.70444	0.400918919	-0.813166	3.90E-06	hypomethylated
cg10943263	0.58276	0.331645946	-0.813258	0.000506	hypomethylated
cg19828516	0.3286	0.186976757	-0.813474	0.000581	hypomethylated
cg02462661	0.49453	0.28138973	-0.813488	5.72E-05	hypomethylated
cg09077096	0.89132	0.50714973	-0.813532	1.72E-06	hypomethylated
cg02099474	0.5035	0.286446486	-0.813726	9.95E-05	hypomethylated
cg06971773	0.34448	0.195977297	-0.813734	0.000551	hypomethylated
cg21936552	0.64664	0.367828649	-0.813929	2.04E-05	hypomethylated
cg15536663	0.43747	0.248736216	-0.814568	0.000445	hypomethylated
cg17515024	0.46546	0.264617297	-0.81475	0.000667	hypomethylated
cg18670236	0.56637	0.321958378	-0.814871	0.003316	hypomethylated
cg18778196	0.52545	0.298668108	-0.81501	0.000182	hypomethylated
cg04499701	0.6564	0.373003784	-0.815385	2.96E-05	hypomethylated
cg17143518	0.28659	0.162837297	-0.815557	0.000626	hypomethylated
cg16242615	0.5309	0.301633514	-0.815643	9.95E-05	hypomethylated
cg14072016	0.81639	0.463685405	-0.816112	8.04E-06	hypomethylated
cg01329577	0.3389	0.192455676	-0.816333	0.011649	hypomethylated
cg20904475	0.42334	0.24037027	-0.816558	9.95E-05	hypomethylated
cg22191981	0.59748	0.33918	-0.816839	0.002649	hypomethylated
cg15972148	0.74427	0.422503243	-0.816864	3.07E-05	hypomethylated
cg01511901	0.49846	0.282870811	-0.817334	0.000485	hypomethylated
cg09293816	0.61556	0.349316216	-0.817366	8.84E-05	hypomethylated
cg03906115	0.4189	0.237678919	-0.817592	0.000232	hypomethylated
cg21684021	0.52664	0.298782162	-0.817723	0.000563	hypomethylated
cg00430895	0.46865	0.265880541	-0.817733	0.000857	hypomethylated
cg23222604	0.32235	0.182848108	-0.817982	0.000135	hypomethylated
cg12848614	0.23449	0.133007568	-0.818018	0.000417	hypomethylated
cg02523270	0.48626	0.275797838	-0.818117	0.001314	hypomethylated
cg00806644	0.6755	0.38312973	-0.818123	0.001031	hypomethylated
cg09851528	0.31855	0.180644865	-0.818364	1.35E-05	hypomethylated
cg11916054	0.35601	0.201884324	-0.818389	0.002856	hypomethylated
cg03254336	0.51041	0.289377297	-0.818705	0.000358	hypomethylated
cg03059420	0.2935	0.166365405	-0.819005	0.001188	hypomethylated
cg04927537	0.3563	0.201951892	-0.819081	9.57E-06	hypomethylated

cg21837069	0.42065	0.238251351	-0.820136	0.003571	hypomethylated
cg25731261	0.57116	0.323477297	-0.82023	7.84E-05	hypomethylated
cg25856090	0.69848	0.395433514	-0.820784	2.04E-05	hypomethylated
cg10820936	0.55541	0.314415676	-0.82088	0.003136	hypomethylated
cg11767757	0.41375	0.234002162	-0.822237	0.000182	hypomethylated
cg14255824	0.77384	0.437604865	-0.822407	2.27E-05	hypomethylated
cg08698943	0.49313	0.278850811	-0.822475	0.0002	hypomethylated
cg12255995	0.47356	0.267756216	-0.822627	0.003571	hypomethylated
cg06653796	0.55277	0.31249027	-0.822868	0.000195	hypomethylated
cg22357164	0.43788	0.247540541	-0.822871	0.000293	hypomethylated
cg03076037	0.4067	0.229872973	-0.823128	1.35E-05	hypomethylated
cg10180415	0.57971	0.327555676	-0.823591	0.000839	hypomethylated
cg06889339	0.37835	0.213605405	-0.824773	0.007035	hypomethylated
cg20151476	0.89491	0.505197297	-0.824896	1.18E-05	hypomethylated
cg11972305	0.31038	0.175204324	-0.824997	0.000342	hypomethylated
cg13413719	0.60198	0.339804324	-0.825011	0.000696	hypomethylated
cg01135464	0.64683	0.365074595	-0.825195	0.000155	hypomethylated
cg17942763	0.48598	0.274275135	-0.825273	5.65E-05	hypomethylated
cg03441171	0.46513	0.262431892	-0.825691	0.000408	hypomethylated
cg03339910	0.58125	0.327789189	-0.826391	0.000219	hypomethylated
cg21661379	0.48267	0.272176216	-0.826496	0.000399	hypomethylated
cg02917867	0.38723	0.218343784	-0.826589	7.12E-05	hypomethylated
cg13292703	0.48532	0.273557838	-0.82709	0.00017	hypomethylated
cg19951006	0.57723	0.325339459	-0.8272	2.38E-05	hypomethylated
cg16282892	0.59535	0.335495676	-0.827444	0.000551	hypomethylated
cg26082814	0.70267	0.395938919	-0.827569	5.28E-06	hypomethylated
cg08527124	0.58528	0.329382162	-0.829365	0.00291	hypomethylated
cg09046979	0.44906	0.252685946	-0.829563	1.48E-05	hypomethylated
cg24935896	0.65831	0.370416216	-0.82962	1.06E-05	hypomethylated
cg06028808	0.33422	0.188004324	-0.830032	0.001176	hypomethylated
cg00755063	0.31046	0.174631351	-0.830095	0.003255	hypomethylated
cg10615591	0.57063	0.320931892	-0.830288	0.000667	hypomethylated
cg13058457	0.61843	0.347788108	-0.830402	0.000588	hypomethylated
cg23108232	0.48546	0.272926486	-0.83084	0.000306	hypomethylated
cg17076667	0.48722	0.273803243	-0.831434	1.18E-05	hypomethylated
cg08922729	0.37563	0.211021081	-0.831925	3.95E-05	hypomethylated
cg06914505	0.44466	0.249743243	-0.832257	9.95E-05	hypomethylated
cg03867607	0.42721	0.239923784	-0.832369	0.006679	hypomethylated
cg05214748	0.43819	0.245921622	-0.833358	0.002599	hypomethylated
cg20337996	0.51731	0.290288649	-0.833541	0.001987	hypomethylated
cg11093142	0.68425	0.383904865	-0.833775	9.49E-05	hypomethylated
cg04148285	0.37694	0.211475676	-0.833843	0.00035	hypomethylated
cg02399371	0.67562	0.379009189	-0.833979	3.90E-06	hypomethylated
cg05907835	0.43031	0.241376757	-0.83409	0.003255	hypomethylated
cg04275695	0.47578	0.266856757	-0.834229	0.000417	hypomethylated
cg06521357	0.41129	0.230635676	-0.83454	0.000756	hypomethylated
cg03434029	0.22647	0.126971351	-0.834817	0.000195	hypomethylated
cg01980793	0.52226	0.292794595	-0.834879	0.00048	hypomethylated
cg13985234	0.54045	0.302981622	-0.834931	0.004445	hypomethylated
cg24328095	0.44466	0.249212432	-0.835327	0.000142	hypomethylated
cg19266329	0.51909	0.290897297	-0.835475	4.10E-05	hypomethylated
cg24495643	0.52259	0.292644324	-0.836531	0.000293	hypomethylated
cg09715059	0.51581	0.288836757	-0.836585	0.000107	hypomethylated
cg02200207	0.63047	0.352978378	-0.836848	0.000358	hypomethylated
cg24033633	0.63376	0.354800541	-0.836928	0.001	hypomethylated
cg15454698	0.59936	0.33548973	-0.837154	0.00024	hypomethylated
cg00446123	0.77272	0.432516757	-0.83719	0.000198	hypomethylated
cg23679344	0.71694	0.401257838	-0.837323	2.02E-05	hypomethylated

cg03495084	0.51565	0.288565946	-0.837491	0.00035	hypomethylated
cg02088237	0.45398	0.253991351	-0.837849	0.002502	hypomethylated
cg20785674	0.51485	0.287941081	-0.838379	0.015196	hypomethylated
cg13602461	0.6027	0.336963784	-0.838847	0.000224	hypomethylated
cg09473745	0.29973	0.167497297	-0.839526	0.000517	hypomethylated
cg11229273	0.56892	0.317876216	-0.839761	0.00071	hypomethylated
cg16547186	0.48834	0.272774595	-0.840177	0.00101	hypomethylated
cg22378252	0.41096	0.229531351	-0.840307	0.000176	hypomethylated
cg19972648	0.66001	0.368608649	-0.840398	3.85E-05	hypomethylated
cg08482837	0.70124	0.391620541	-0.840452	4.00E-05	hypomethylated
cg15934095	0.73268	0.409111351	-0.84069	1.77E-05	hypomethylated
cg02898159	0.48512	0.270785946	-0.841189	0.0002	hypomethylated
cg00480331	0.38764	0.216323243	-0.841529	0.002455	hypomethylated
cg02805890	0.51674	0.28835027	-0.841616	0.003705	hypomethylated
cg06968859	0.57009	0.3181	-0.841709	9.31E-06	hypomethylated
cg04943879	0.54758	0.305537838	-0.841719	2.39E-05	hypomethylated
cg26954174	0.43845	0.244631351	-0.841803	0.000703	hypomethylated
cg24028809	0.61444	0.3428	-0.841905	0.000517	hypomethylated
cg03620376	0.33819	0.188662703	-0.842025	0.000741	hypomethylated
cg15176413	0.42331	0.236145946	-0.842036	0.0006	hypomethylated
cg26392367	0.49394	0.275520541	-0.842176	0.000726	hypomethylated
cg07727594	0.58298	0.32514	-0.842385	2.04E-05	hypomethylated
cg16801374	0.27996	0.156118919	-0.842575	0.005411	hypomethylated
cg25461508	0.47106	0.262675135	-0.842631	0.000772	hypomethylated
cg21410633	0.35243	0.196514054	-0.842704	0.001635	hypomethylated
cg10142237	0.42357	0.236100541	-0.843199	0.000138	hypomethylated
cg16233797	0.82493	0.459600541	-0.843891	3.44E-06	hypomethylated
cg08884752	0.42802	0.238442703	-0.844036	8.22E-05	hypomethylated
cg24996979	0.4346	0.242071351	-0.844256	0.001912	hypomethylated
cg06613738	0.7697	0.4287	-0.844328	1.80E-05	hypomethylated
cg22161562	0.43557	0.242529189	-0.844746	0.00054	hypomethylated
cg11304234	0.38419	0.213908108	-0.844829	1.46E-05	hypomethylated
cg04921315	0.68891	0.383496757	-0.845101	0.000182	hypomethylated
cg17345741	0.35335	0.196687027	-0.845196	2.78E-05	hypomethylated
cg26733301	0.54782	0.304914054	-0.845299	0.003506	hypomethylated
cg02488653	0.38256	0.212917297	-0.845393	0.005808	hypomethylated
cg03254465	0.29128	0.162081622	-0.845686	0.01274	hypomethylated
cg17186066	0.59458	0.330831351	-0.845775	0.001164	hypomethylated
cg26402417	0.70594	0.392751892	-0.845927	0.000251	hypomethylated
cg13197560	0.7679	0.42722	-0.845939	2.51E-05	hypomethylated
cg01553231	0.79419	0.441766486	-0.8462	1.99E-05	hypomethylated
cg25298161	0.46417	0.258179459	-0.846279	0.000382	hypomethylated
cg01538305	0.49332	0.274371892	-0.846391	6.01E-05	hypomethylated
cg18975416	0.56923	0.316529189	-0.846673	0.000831	hypomethylated
cg14302214	0.83177	0.462413514	-0.847001	5.28E-06	hypomethylated
cg19919590	0.49813	0.276900541	-0.847154	0.001603	hypomethylated
cg10575547	0.7485	0.416037838	-0.847288	1.31E-05	hypomethylated
cg23021584	0.68543	0.380954595	-0.84739	0.000129	hypomethylated
cg21370924	0.40289	0.223865946	-0.847751	0.000159	hypomethylated
cg04470557	0.40217	0.223425946	-0.848009	0.001875	hypomethylated
cg13579607	0.45379	0.252084324	-0.848118	0.000575	hypomethylated
cg08003402	0.63121	0.350610811	-0.84825	0.000174	hypomethylated
cg12510708	0.61799	0.343244324	-0.848348	0.000126	hypomethylated
cg17266282	0.56144	0.311803243	-0.848496	0.001096	hypomethylated
cg07271561	0.28947	0.16076	-0.848505	0.00095	hypomethylated
cg15331705	0.55154	0.306300541	-0.848518	4.47E-05	hypomethylated
cg11170179	0.27126	0.150627568	-0.84869	0.000454	hypomethylated
cg20940675	0.47641	0.264538919	-0.848724	0.000474	hypomethylated

cg11970797	0.73491	0.407974054	-0.84909	3.76E-05	hypomethylated
cg04510874	0.32698	0.181512973	-0.84913	0.000474	hypomethylated
cg26781466	0.35023	0.19438973	-0.849351	0.000248	hypomethylated
cg22254463	0.79434	0.440848649	-0.849473	3.07E-05	hypomethylated
cg02772121	0.84626	0.469417838	-0.850228	4.24E-06	hypomethylated
cg02693002	0.20369	0.112968108	-0.85046	0.00097	hypomethylated
cg15321306	0.73667	0.40847027	-0.850787	4.48E-06	hypomethylated
cg10437265	0.48687	0.26993027	-0.85095	1.66E-05	hypomethylated
cg26177041	0.67076	0.371716757	-0.851593	2.04E-05	hypomethylated
cg14283454	0.39733	0.220141622	-0.851906	0.000374	hypomethylated
cg10611580	0.47265	0.26172	-0.852748	0.00097	hypomethylated
cg08614871	0.42877	0.237416757	-0.852782	0.0017	hypomethylated
cg23958704	0.65467	0.36238	-0.853265	0.000117	hypomethylated
cg04760448	0.66763	0.369543784	-0.853303	3.39E-06	hypomethylated
cg25383479	0.23829	0.131896216	-0.853315	0.004365	hypomethylated
cg12902896	0.43166	0.238873514	-0.853649	7.47E-05	hypomethylated
cg14204586	0.47394	0.262197297	-0.854052	0.000421	hypomethylated
cg19095568	0.61225	0.33871027	-0.854069	0.000127	hypomethylated
cg11421182	0.5857	0.323927568	-0.854491	1.72E-06	hypomethylated
cg10185478	0.57545	0.318223243	-0.854651	0.000893	hypomethylated
cg15355111	0.466	0.257642162	-0.854961	0.000805	hypomethylated
cg02771117	0.635	0.351064324	-0.855021	8.84E-05	hypomethylated
cg16715129	0.4523	0.250027027	-0.855196	0.000653	hypomethylated
cg17641252	0.61917	0.342236216	-0.855343	3.40E-05	hypomethylated
cg17472111	0.45374	0.250778919	-0.85545	0.000195	hypomethylated
cg05417332	0.36088	0.19942	-0.855709	0.001354	hypomethylated
cg06362313	0.50332	0.278124324	-0.855746	1.11E-05	hypomethylated
cg24036791	0.85261	0.470942162	-0.856336	4.24E-06	hypomethylated
cg24327132	0.50349	0.278062703	-0.856553	0.000176	hypomethylated
cg19531130	0.38553	0.212851351	-0.856997	0.00078	hypomethylated
cg24671734	0.53227	0.293795676	-0.857345	0.001074	hypomethylated
cg07078958	0.63635	0.351235135	-0.857383	0.000435	hypomethylated
cg07277549	0.52277	0.288527027	-0.85747	0.000474	hypomethylated
cg17327067	0.33382	0.184195135	-0.857835	3.49E-05	hypomethylated
cg13782615	0.51222	0.282605946	-0.857972	0.008491	hypomethylated
cg19274341	0.51592	0.284371892	-0.859369	0.001667	hypomethylated
cg20388580	0.59429	0.327557838	-0.859417	0.000921	hypomethylated
cg21380183	0.31296	0.172462703	-0.859694	0.003255	hypomethylated
cg06112171	0.67545	0.372185946	-0.859825	6.57E-06	hypomethylated
cg07162085	0.55178	0.30403027	-0.859878	1.31E-05	hypomethylated
cg16800111	0.2218	0.122193514	-0.860092	0.000725	hypomethylated
cg09817016	0.33774	0.186051892	-0.860208	0.000245	hypomethylated
cg17980404	0.55851	0.30751027	-0.860949	0.0002	hypomethylated
cg05951609	0.37429	0.206079459	-0.860956	0.001212	hypomethylated
cg03063658	0.50452	0.277662162	-0.861581	0.000142	hypomethylated
cg25888386	0.61284	0.337237297	-0.861746	0.00134	hypomethylated
cg07912922	0.62804	0.345384865	-0.862652	0.001768	hypomethylated
cg22027433	0.52311	0.287597838	-0.863062	0.002231	hypomethylated
cg22786667	0.56951	0.312979459	-0.863653	8.63E-05	hypomethylated
cg10466548	0.3971	0.218203784	-0.863826	0.002599	hypomethylated
cg06191357	0.20519	0.112738378	-0.863982	0.000756	hypomethylated
cg10364630	0.29263	0.160762162	-0.86415	0.010819	hypomethylated
cg25258233	0.25908	0.142294595	-0.864517	0.003637	hypomethylated
cg13876325	0.63063	0.346141622	-0.865431	6.15E-05	hypomethylated
cg14065857	0.64611	0.354543784	-0.865816	6.46E-05	hypomethylated
cg04214938	0.71593	0.392841622	-0.865871	7.12E-05	hypomethylated
cg26020069	0.49662	0.272500541	-0.865883	1.35E-05	hypomethylated
cg04262471	0.31704	0.173881622	-0.866559	0.005706	hypomethylated

cg09845604	0.39279	0.215412973	-0.866653	0.000696	hypomethylated
cg05257291	0.33213	0.182053514	-0.867385	0.009877	hypomethylated
cg09507567	0.5237	0.286988108	-0.86775	1.09E-05	hypomethylated
cg17411016	0.45002	0.246592432	-0.867861	0.0002	hypomethylated
cg11811510	0.53747	0.294482703	-0.868001	0.000495	hypomethylated
cg10241809	0.33446	0.183187568	-0.868512	1.84E-05	hypomethylated
cg02747253	0.37017	0.202728649	-0.868638	0.001409	hypomethylated
cg23328404	0.49415	0.270625405	-0.868652	0.000245	hypomethylated
cg24956391	0.77765	0.425816757	-0.868888	6.46E-05	hypomethylated
cg11123744	0.60626	0.331888649	-0.869237	2.74E-05	hypomethylated
cg06616245	0.32893	0.180048649	-0.869394	0.000469	hypomethylated
cg23391288	0.77737	0.425445946	-0.869626	1.35E-05	hypomethylated
cg07709148	0.25198	0.137903784	-0.869647	0.004608	hypomethylated
cg13446584	0.82614	0.451894054	-0.870402	4.24E-06	hypomethylated
cg05191839	0.60408	0.330397838	-0.870535	5.00E-06	hypomethylated
cg08824384	0.43698	0.23896	-0.870798	5.58E-05	hypomethylated
cg03498081	0.47546	0.25996973	-0.87098	0.000382	hypomethylated
cg22110158	0.57063	0.311999459	-0.871012	6.22E-06	hypomethylated
cg06457736	0.75493	0.412724865	-0.871163	3.15E-05	hypomethylated
cg02538248	0.37783	0.206508108	-0.871539	3.23E-05	hypomethylated
cg22737241	0.59768	0.326544324	-0.872094	0.000204	hypomethylated
cg04894537	0.6273	0.342676216	-0.872309	7.03E-05	hypomethylated
cg12048965	0.66095	0.361055676	-0.87232	0.000182	hypomethylated
cg22382836	0.49171	0.268585946	-0.872424	0.001237	hypomethylated
cg16541026	0.4054	0.221438919	-0.872437	2.04E-05	hypomethylated
cg00085448	0.27059	0.147788649	-0.872573	0.003195	hypomethylated
cg21574271	0.59021	0.322322703	-0.872723	7.29E-05	hypomethylated
cg22918360	0.37009	0.202089189	-0.872884	0.007472	hypomethylated
cg08528170	0.39189	0.213955676	-0.873137	0.00291	hypomethylated
cg27115863	0.49047	0.2677	-0.873548	0.000633	hypomethylated
cg17367616	0.4825	0.263261622	-0.874032	9.49E-05	hypomethylated
cg21851534	0.37399	0.204	-0.874431	0.0017	hypomethylated
cg22728904	0.50657	0.276298378	-0.874535	0.000365	hypomethylated
cg12126038	0.59528	0.324568649	-0.875045	6.22E-06	hypomethylated
cg21461300	0.31188	0.170044324	-0.87508	0.000185	hypomethylated
cg15653254	0.66219	0.360997297	-0.875257	3.21E-06	hypomethylated
cg17501395	0.55805	0.304215676	-0.8753	0.000575	hypomethylated
cg25123566	0.52516	0.28627027	-0.875379	0.003538	hypomethylated
cg10025586	0.6205	0.338170811	-0.875679	0.000142	hypomethylated
cg04998634	0.4406	0.240082162	-0.875941	0.003473	hypomethylated
cg16820615	0.42174	0.229762162	-0.876213	0.000551	hypomethylated
cg10576245	0.62696	0.34156	-0.876234	0.000274	hypomethylated
cg13997435	0.42603	0.232041081	-0.876575	2.15E-05	hypomethylated
cg08412188	0.27555	0.150068649	-0.876692	0.001857	hypomethylated
cg09889479	0.38598	0.210188649	-0.876841	0.000138	hypomethylated
cg20561863	0.4882	0.265795135	-0.877158	9.27E-05	hypomethylated
cg10732871	0.2846	0.154941622	-0.877211	9.83E-05	hypomethylated
cg13022977	0.28701	0.156248108	-0.877262	0.000157	hypomethylated
cg17951713	0.45043	0.245083784	-0.878028	0.000107	hypomethylated
cg22354646	0.41294	0.224649189	-0.878258	0.000132	hypomethylated
cg12458966	0.50689	0.275738378	-0.878373	1.28E-05	hypomethylated
cg23656110	0.36617	0.199075135	-0.879201	0.000335	hypomethylated
cg14339466	0.68999	0.375103243	-0.879288	3.30E-06	hypomethylated
cg15127702	0.55154	0.299678378	-0.880051	0.001466	hypomethylated
cg00301239	0.24428	0.132718378	-0.880168	0.000342	hypomethylated
cg07551060	0.73009	0.396592973	-0.880415	4.47E-05	hypomethylated
cg02331830	0.51599	0.280287568	-0.880435	0.000214	hypomethylated
cg16933922	0.23614	0.12816	-0.881696	8.42E-05	hypomethylated

cg20266715	0.63756	0.345987568	-0.881841	6.39E-06	hypomethylated
cg14731462	0.69023	0.374447027	-0.882316	0.000135	hypomethylated
cg04719574	0.34474	0.187014595	-0.882358	0.004691	hypomethylated
cg27113419	0.48927	0.265408649	-0.882415	8.94E-05	hypomethylated
cg01535567	0.21229	0.115144324	-0.882593	0.000445	hypomethylated
cg01303372	0.67922	0.368371351	-0.882718	0.000575	hypomethylated
cg10795659	0.5642	0.305801081	-0.883613	0.003255	hypomethylated
cg00146645	0.32353	0.175337838	-0.883762	0.000718	hypomethylated
cg07267600	0.36044	0.195286486	-0.884167	0.000293	hypomethylated
cg01612140	0.61915	0.335443784	-0.884218	0.000122	hypomethylated
cg09516200	0.64152	0.347543243	-0.884302	8.03E-05	hypomethylated
cg16033151	0.42803	0.231680541	-0.885575	5.06E-05	hypomethylated
cg13480493	0.56199	0.30396	-0.886663	0.002007	hypomethylated
cg09501687	0.60451	0.326931892	-0.886776	0.001314	hypomethylated
cg25816127	0.32146	0.173809189	-0.887135	0.008348	hypomethylated
cg18041642	0.59564	0.321971892	-0.887506	0.000127	hypomethylated
cg23172671	0.70803	0.382565946	-0.888102	9.82E-06	hypomethylated
cg06834637	0.34876	0.188404324	-0.888403	0.000219	hypomethylated
cg14982472	0.4096	0.221242703	-0.888586	0.005411	hypomethylated
cg02836864	0.31635	0.170813514	-0.889099	0.0003	hypomethylated
cg14032089	0.45186	0.243965405	-0.889199	0.000159	hypomethylated
cg04339837	0.31747	0.171365405	-0.889544	0.004059	hypomethylated
cg04654167	0.44143	0.238162162	-0.89024	5.45E-05	hypomethylated
cg04770088	0.45107	0.243107568	-0.891757	0.002574	hypomethylated
cg07220903	0.56974	0.307027027	-0.891938	0.000506	hypomethylated
cg08918658	0.37449	0.201797297	-0.89202	0.000523	hypomethylated
cg04223548	0.53853	0.29019027	-0.892028	0.000135	hypomethylated
cg21201401	0.72607	0.391238919	-0.892059	0.000408	hypomethylated
cg15186638	0.28078	0.151296757	-0.892059	0.000287	hypomethylated
cg09159452	0.78264	0.421700541	-0.89213	2.27E-05	hypomethylated
cg02276807	0.65042	0.350438919	-0.892209	5.32E-05	hypomethylated
cg14129735	0.37848	0.203914054	-0.892256	0.004326	hypomethylated
cg23729881	0.46501	0.250522162	-0.892324	0.002965	hypomethylated
cg00691874	0.6102	0.328678378	-0.892606	5.19E-05	hypomethylated
cg03048889	0.30319	0.163268649	-0.892974	0.018773	hypomethylated
cg10462187	0.57325	0.308663243	-0.893131	3.49E-06	hypomethylated
cg14753356	0.50395	0.271201081	-0.893918	0.000174	hypomethylated
cg24862514	0.26497	0.142576757	-0.89409	0.000848	hypomethylated
cg19257864	0.22872	0.123022703	-0.894658	0.000189	hypomethylated
cg09668030	0.50732	0.272856216	-0.894755	0.006796	hypomethylated
cg04186657	0.20356	0.10948	-0.894787	0.008208	hypomethylated
cg05133205	0.46346	0.249172973	-0.895297	0.000126	hypomethylated
cg25755851	0.41095	0.220929189	-0.895379	7.12E-05	hypomethylated
cg25067702	0.50036	0.268943784	-0.895662	0.00049	hypomethylated
cg13709639	0.23715	0.127467568	-0.89567	0.000148	hypomethylated
cg15646741	0.57946	0.311285405	-0.896471	0.000163	hypomethylated
cg17967818	0.30668	0.164747568	-0.896477	0.000126	hypomethylated
cg14606858	0.53449	0.28712	-0.896509	0.000464	hypomethylated
cg04324276	0.35228	0.189220541	-0.896654	0.000148	hypomethylated
cg03611598	0.19728	0.105960541	-0.896718	0.002599	hypomethylated
cg14396008	0.40253	0.216129189	-0.897202	0.000681	hypomethylated
cg00073090	0.42996	0.230826486	-0.897394	0.000104	hypomethylated
cg19069346	0.22346	0.119958378	-0.897483	0.000501	hypomethylated
cg01904393	0.30602	0.164243243	-0.897792	7.12E-05	hypomethylated
cg18582260	0.69237	0.37139027	-0.898607	0.000342	hypomethylated
cg10416784	0.65395	0.350662162	-0.899099	8.60E-06	hypomethylated
cg13921921	0.47152	0.252797838	-0.899335	0.000145	hypomethylated
cg27628707	0.38867	0.208362162	-0.899452	0.006068	hypomethylated

cg20881888	0.36127	0.193604324	-0.899966	0.00071	hypomethylated
cg15420926	0.36921	0.197825405	-0.900214	7.29E-05	hypomethylated
cg13581922	0.43669	0.233950811	-0.900404	0.000756	hypomethylated
cg03037030	0.51555	0.276081622	-0.901017	0.000485	hypomethylated
cg17534029	0.55493	0.297040541	-0.901646	0.001052	hypomethylated
cg11606261	0.63814	0.341551892	-0.901768	0.000104	hypomethylated
cg11993160	0.3172	0.169761081	-0.901887	8.03E-05	hypomethylated
cg14102437	0.49792	0.266474054	-0.901919	0.000187	hypomethylated
cg11640208	0.46017	0.246247568	-0.902057	0.00134	hypomethylated
cg06105296	0.37707	0.201709189	-0.902556	0.002274	hypomethylated
cg26395331	0.37186	0.19892	-0.902571	0.00032	hypomethylated
cg01928076	0.30232	0.161695676	-0.902795	0.000501	hypomethylated
cg20365336	0.43034	0.230005946	-0.903806	0.005706	hypomethylated
cg10020892	0.56376	0.301204324	-0.904339	0.000306	hypomethylated
cg03554573	0.57558	0.307481622	-0.904516	0.000931	hypomethylated
cg06665622	0.6624	0.353856757	-0.904537	1.51E-06	hypomethylated
cg14815361	0.74807	0.399607568	-0.904589	8.60E-06	hypomethylated
cg07313319	0.42284	0.225855676	-0.904711	0.000134	hypomethylated
cg00667789	0.26113	0.139446486	-0.905057	0.000523	hypomethylated
cg21873674	0.29979	0.160087027	-0.905096	0.001288	hypomethylated
cg06051311	0.63997	0.341459459	-0.90629	3.32E-05	hypomethylated
cg23451678	0.36998	0.197386486	-0.906424	0.002649	hypomethylated
cg23340505	0.38011	0.202778378	-0.906513	0.00101	hypomethylated
cg24146125	0.41939	0.223707568	-0.906678	0.000342	hypomethylated
cg11334730	0.53653	0.286097838	-0.90715	2.32E-05	hypomethylated
cg27183030	0.24082	0.128408649	-0.907213	0.002599	hypomethylated
cg12679308	0.36654	0.195376757	-0.907712	0.003316	hypomethylated
cg23425762	0.47962	0.25554973	-0.908288	0.008636	hypomethylated
cg16647844	0.37599	0.200295676	-0.908563	0.000875	hypomethylated
cg06085204	0.4595	0.244762703	-0.908681	0.002189	hypomethylated
cg21625464	0.2064	0.109904324	-0.909195	0.004608	hypomethylated
cg07739369	0.50667	0.269790811	-0.909205	5.65E-05	hypomethylated
cg02793828	0.63333	0.33709027	-0.909822	4.05E-05	hypomethylated
cg02319354	0.37163	0.197781622	-0.909959	0.001603	hypomethylated
cg06401414	0.46749	0.248791892	-0.909996	4.73E-06	hypomethylated
cg03590237	0.40771	0.21696973	-0.91005	0.000551	hypomethylated
cg11462533	0.29904	0.159087568	-0.910517	0.001176	hypomethylated
cg08730348	0.41706	0.221857297	-0.910623	0.002699	hypomethylated
cg03364486	0.6526	0.347111351	-0.9108	0.000251	hypomethylated
cg17490196	0.53485	0.284427027	-0.911076	0.000148	hypomethylated
cg07563611	0.7832	0.416296216	-0.91177	6.22E-06	hypomethylated
cg08752433	0.72062	0.382999459	-0.911896	5.58E-06	hypomethylated
cg26835302	0.52641	0.279721081	-0.912198	0.000626	hypomethylated
cg03852144	0.57279	0.304164324	-0.913155	0.003506	hypomethylated
cg20970886	0.80219	0.425944865	-0.913277	6.39E-06	hypomethylated
cg14602087	0.59317	0.314904865	-0.91353	0.000123	hypomethylated
cg26400954	0.79345	0.421168649	-0.913741	8.60E-06	hypomethylated
cg22301854	0.37537	0.199216757	-0.913974	0.000501	hypomethylated
cg24910675	0.29226	0.155092432	-0.914124	0.005656	hypomethylated
cg26940518	0.23712	0.125757838	-0.914969	0.003986	hypomethylated
cg12787405	0.3266	0.173139459	-0.91559	0.000607	hypomethylated
cg10574566	0.32389	0.171699459	-0.915618	0.000163	hypomethylated
cg26856257	0.42841	0.227019459	-0.916176	0.00044	hypomethylated
cg00980058	0.4838	0.256332432	-0.916395	0.000195	hypomethylated
cg13427361	0.64035	0.339264324	-0.916451	0.000365	hypomethylated
cg26788216	0.50502	0.267547027	-0.916548	0.000112	hypomethylated
cg07962303	0.37372	0.197957838	-0.916765	0.0003	hypomethylated
cg00733978	0.70423	0.373024865	-0.916775	1.25E-05	hypomethylated

cg16472904	0.57064	0.302248108	-0.916848	2.64E-05	hypomethylated
cg12737497	0.29359	0.155488649	-0.916994	0.000365	hypomethylated
cg05514299	0.65767	0.348246486	-0.917255	3.49E-05	hypomethylated
cg23739746	0.52987	0.280573514	-0.91726	0.001651	hypomethylated
cg11108890	0.33287	0.176133514	-0.918289	8.94E-05	hypomethylated
cg03081478	0.44365	0.234725405	-0.918448	0.000435	hypomethylated
cg01120308	0.33251	0.175907027	-0.918585	0.000209	hypomethylated
cg10316899	0.70346	0.372095676	-0.918795	5.43E-06	hypomethylated
cg09033006	0.46271	0.244669189	-0.919276	9.38E-05	hypomethylated
cg09183124	0.61096	0.323008649	-0.919505	0.000274	hypomethylated
cg04990202	0.67923	0.35877027	-0.92084	4.94E-05	hypomethylated
cg11201447	0.74133	0.391380541	-0.921544	4.82E-05	hypomethylated
cg01738022	0.49738	0.262496216	-0.922052	0.023591	hypomethylated
cg04293307	0.4839	0.255283243	-0.92261	2.39E-05	hypomethylated
cg14130039	0.34882	0.18401027	-0.922696	9.83E-05	hypomethylated
cg08892236	0.26918	0.141868649	-0.924015	0.004526	hypomethylated
cg19769182	0.19697	0.103810811	-0.924019	0.000408	hypomethylated
cg02097203	0.50624	0.266742162	-0.924376	0.000569	hypomethylated
cg14901243	0.63765	0.335975676	-0.924408	5.19E-05	hypomethylated
cg14511923	0.64989	0.342372432	-0.924629	0.00024	hypomethylated
cg14174912	0.45313	0.238692973	-0.924769	0.001667	hypomethylated
cg13266096	0.31166	0.164157838	-0.924889	0.002751	hypomethylated
cg14106046	0.57115	0.300775676	-0.925182	0.000703	hypomethylated
cg04276626	0.56452	0.297282703	-0.925189	1.84E-05	hypomethylated
cg15601264	0.49972	0.263136216	-0.92531	0.000374	hypomethylated
cg20034617	0.36904	0.19424973	-0.925865	0.003136	hypomethylated
cg11219400	0.28993	0.152581622	-0.926123	0.002599	hypomethylated
cg18650367	0.38067	0.200332432	-0.926145	0.000226	hypomethylated
cg05726195	0.39632	0.208541081	-0.926334	0.007282	hypomethylated
cg14679780	0.52204	0.274629189	-0.926675	0.00028	hypomethylated
cg05288172	0.56375	0.296415135	-0.927436	0.000101	hypomethylated
cg09552548	0.60174	0.316370811	-0.927524	0.000107	hypomethylated
cg23526474	0.31501	0.165608108	-0.927624	0.0017	hypomethylated
cg24876187	0.50505	0.265511351	-0.927653	0.000893	hypomethylated
cg24605325	0.26525	0.139434595	-0.927764	0.00066	hypomethylated
cg19061690	0.35218	0.185115676	-0.927886	0.000374	hypomethylated
cg12686441	0.63394	0.333214054	-0.927897	8.63E-05	hypomethylated
cg02849956	0.59424	0.312176216	-0.928685	5.19E-05	hypomethylated
cg19131731	0.40468	0.212515676	-0.929212	0.000528	hypomethylated
cg12799029	0.22351	0.117363784	-0.929352	0.001556	hypomethylated
cg27268352	0.59509	0.312430811	-0.929571	1.31E-05	hypomethylated
cg02822788	0.40973	0.21511027	-0.929597	0.000365	hypomethylated
cg21241823	0.51137	0.268454595	-0.929689	0.00013	hypomethylated
cg07638935	0.45233	0.237445946	-0.929777	0.000454	hypomethylated
cg23684410	0.41572	0.218095676	-0.930651	3.27E-05	hypomethylated
cg11505417	0.34495	0.180959459	-0.930721	0.003255	hypomethylated
cg10784519	0.25084	0.131551892	-0.931135	0.00341	hypomethylated
cg17415265	0.56271	0.295072432	-0.931322	0.001949	hypomethylated
cg05906166	0.47647	0.249808108	-0.931565	0.000857	hypomethylated
cg26579713	0.61268	0.321203243	-0.931647	0.00113	hypomethylated
cg16355945	0.24318	0.127455135	-0.932035	0.00044	hypomethylated
cg03850117	0.42177	0.220833514	-0.933497	8.03E-05	hypomethylated
cg05870586	0.59245	0.310141081	-0.933769	2.10E-05	hypomethylated
cg01556706	0.58501	0.306008649	-0.934889	0.000126	hypomethylated
cg20852851	0.88723	0.464057838	-0.935004	3.12E-06	hypomethylated
cg13606889	0.44246	0.231347027	-0.935488	7.65E-05	hypomethylated
cg24680129	0.33668	0.176014595	-0.935683	0.000174	hypomethylated
cg10503655	0.28909	0.151067027	-0.93633	1.58E-05	hypomethylated

cg14369648	0.41478	0.216627027	-0.937133	0.002189	hypomethylated
cg07165167	0.39772	0.207644865	-0.937635	0.000749	hypomethylated
cg14289469	0.32827	0.171265946	-0.938645	4.94E-05	hypomethylated
cg11429044	0.54006	0.281742703	-0.938741	0.000789	hypomethylated
cg15068733	0.3206	0.167239459	-0.938859	0.001212	hypomethylated
cg13058623	0.45768	0.238655676	-0.939409	4.05E-05	hypomethylated
cg23252902	0.28869	0.150502703	-0.939732	0.004526	hypomethylated
cg17252884	0.53243	0.277556757	-0.939809	0.001052	hypomethylated
cg08123444	0.68993	0.359574054	-0.940161	0.000155	hypomethylated
cg15628518	0.56791	0.295975676	-0.940184	6.78E-05	hypomethylated
cg23202253	0.61349	0.319725405	-0.940206	1.01E-05	hypomethylated
cg17178175	0.56023	0.291904324	-0.940524	0.000435	hypomethylated
cg11118962	0.42131	0.219514595	-0.940565	0.000221	hypomethylated
cg06075311	0.46822	0.243856216	-0.941156	0.00095	hypomethylated
cg18956547	0.48757	0.253932432	-0.941165	0.0003	hypomethylated
cg23792308	0.39084	0.203552432	-0.941178	0.000426	hypomethylated
cg16783186	0.60404	0.314534595	-0.941425	8.63E-05	hypomethylated
cg22908423	0.4631	0.241135135	-0.941482	3.23E-05	hypomethylated
cg23452752	0.31511	0.163976757	-0.942364	0.000117	hypomethylated
cg08169827	0.39733	0.206736216	-0.942547	0.000839	hypomethylated
cg16983159	0.50921	0.264859459	-0.943034	0.002106	hypomethylated
cg10255237	0.61759	0.321206486	-0.943148	0.000426	hypomethylated
cg12526997	0.64885	0.337438919	-0.943259	0.000459	hypomethylated
cg10987840	0.60805	0.315989189	-0.944315	1.75E-05	hypomethylated
cg16316624	0.40929	0.212569189	-0.945191	0.000358	hypomethylated
cg11549025	0.52796	0.274130811	-0.945564	0.000256	hypomethylated
cg16809762	0.57055	0.296062703	-0.946451	6.57E-06	hypomethylated
cg23662097	0.52721	0.273480541	-0.94694	0.002455	hypomethylated
cg00293245	0.22493	0.116657838	-0.947193	0.001288	hypomethylated
cg08461352	0.52028	0.269819459	-0.947294	2.95E-06	hypomethylated
cg19770281	0.33576	0.174025946	-0.948128	0.000581	hypomethylated
cg12363375	0.22273	0.115401622	-0.948632	0.000417	hypomethylated
cg24722577	0.64167	0.332420541	-0.948822	0.000653	hypomethylated
cg02273436	0.5459	0.282788108	-0.948915	0.000287	hypomethylated
cg05376228	0.49194	0.254726486	-0.949533	7.12E-05	hypomethylated
cg22367310	0.44199	0.228845946	-0.949637	0.000528	hypomethylated
cg06142351	0.41463	0.214622162	-0.950025	0.003637	hypomethylated
cg09853702	0.60662	0.313662703	-0.951579	0.000115	hypomethylated
cg26203879	0.35768	0.184863243	-0.952211	0.000435	hypomethylated
cg08823209	0.52206	0.269770811	-0.952481	1.08E-05	hypomethylated
cg26928531	0.31931	0.164983243	-0.952638	0.000588	hypomethylated
cg00542992	0.52722	0.272338919	-0.953002	2.45E-05	hypomethylated
cg03568017	0.30047	0.155042703	-0.954555	0.008069	hypomethylated
cg03454663	0.42836	0.22102973	-0.954583	4.15E-05	hypomethylated
cg24082121	0.26531	0.136771351	-0.955913	0.001031	hypomethylated
cg04691781	0.31212	0.160761081	-0.957183	0.007035	hypomethylated
cg13029400	0.45365	0.233653514	-0.957209	0.000268	hypomethylated
cg08305942	0.70218	0.361592973	-0.957474	6.22E-06	hypomethylated
cg27413008	0.43542	0.224017297	-0.958798	0.000931	hypomethylated
cg16719560	0.37204	0.191285405	-0.959731	0.000163	hypomethylated
cg04155862	0.22788	0.117158919	-0.959808	0.000219	hypomethylated
cg16094145	0.31932	0.164111892	-0.960323	0.000454	hypomethylated
cg16596317	0.75024	0.385563243	-0.960385	1.09E-05	hypomethylated
cg18693822	0.66515	0.341631892	-0.961237	2.07E-05	hypomethylated
cg16072777	0.40168	0.206237297	-0.961741	0.001481	hypomethylated
cg14115597	0.69804	0.358390811	-0.961776	7.12E-06	hypomethylated
cg17922998	0.61639	0.316406486	-0.962064	0.000129	hypomethylated
cg04452095	0.38146	0.195801081	-0.962143	8.84E-05	hypomethylated

cg10338787	0.21622	0.110963784	-0.962411	0.000126	hypomethylated
cg17972655	0.31434	0.161309189	-0.962497	0.000143	hypomethylated
cg03532331	0.39471	0.202535676	-0.962617	0.00032	hypomethylated
cg14856585	0.21484	0.110235676	-0.962671	0.000921	hypomethylated
cg17614506	0.24464	0.125511351	-0.962842	3.67E-05	hypomethylated
cg21196487	0.29984	0.153722703	-0.963863	0.000193	hypomethylated
cg09072560	0.38804	0.198892432	-0.964217	0.001074	hypomethylated
cg03402605	0.32633	0.167196757	-0.964785	0.005223	hypomethylated
cg16396284	0.43461	0.222663784	-0.964854	0.012036	hypomethylated
cg13417862	0.3909	0.200248108	-0.965011	0.001237	hypomethylated
cg21243631	0.48741	0.249535676	-0.96589	5.72E-05	hypomethylated
cg02072495	0.28485	0.145816216	-0.966051	0.001096	hypomethylated
cg09730500	0.43898	0.224661081	-0.966405	0.000123	hypomethylated
cg16736018	0.42949	0.219673514	-0.967264	0.000399	hypomethylated
cg20606255	0.22452	0.114775135	-0.968034	0.001481	hypomethylated
cg12903224	0.37631	0.192344865	-0.968226	0.000219	hypomethylated
cg02578087	0.71173	0.363767568	-0.968313	2.71E-05	hypomethylated
cg09988805	0.6966	0.356023784	-0.968357	2.64E-06	hypomethylated
cg19918734	0.42962	0.219520541	-0.968705	0.001141	hypomethylated
cg26348487	0.54942	0.280727027	-0.968741	0.00099	hypomethylated
cg14552947	0.61649	0.314767027	-0.969793	0.001052	hypomethylated
cg01246622	0.52018	0.265468108	-0.970472	0.000726	hypomethylated
cg03598112	0.55306	0.282244865	-0.970489	0.000594	hypomethylated
cg05903736	0.56934	0.290368108	-0.971407	3.81E-05	hypomethylated
cg09535960	0.40427	0.206082162	-0.9721	0.005706	hypomethylated
cg24067911	0.68755	0.350412973	-0.972408	2.87E-06	hypomethylated
cg27144670	0.73061	0.372303784	-0.972621	3.79E-06	hypomethylated
cg17329164	0.31142	0.158680541	-0.972736	0.000262	hypomethylated
cg17745097	0.38788	0.197631892	-0.972795	0.001893	hypomethylated
cg17715243	0.55062	0.280545946	-0.97282	5.32E-05	hypomethylated
cg13036352	0.36563	0.186258378	-0.973079	0.018773	hypomethylated
cg04888360	0.46982	0.239331351	-0.973099	5.81E-06	hypomethylated
cg02225720	0.64513	0.328456216	-0.973889	0.000772	hypomethylated
cg21130374	0.57006	0.290192973	-0.974101	0.00421	hypomethylated
cg01777397	0.30411	0.154801622	-0.974173	0.001556	hypomethylated
cg03189210	0.42107	0.214321081	-0.974286	0.006015	hypomethylated
cg13052638	0.63321	0.32220973	-0.974684	0.000126	hypomethylated
ch.6.2925136R	0.25122	0.127789189	-0.975186	0.000358	hypomethylated
cg11496630	0.38741	0.197003784	-0.975638	0.007933	hypomethylated
cg02858512	0.46548	0.236697838	-0.975673	0.004776	hypomethylated
cg27005749	0.64234	0.326511892	-0.976202	0.000365	hypomethylated
cg27001715	0.53465	0.271695135	-0.976606	8.22E-05	hypomethylated
cg13432339	0.47962	0.243717838	-0.97668	0.000145	hypomethylated
cg03624316	0.55189	0.280361081	-0.977095	1.94E-05	hypomethylated
cg07186138	0.35724	0.181447568	-0.977341	0.00421	hypomethylated
cg10574006	0.75049	0.381048108	-0.97786	2.87E-06	hypomethylated
cg06946797	0.53513	0.271657297	-0.978102	0.000646	hypomethylated
cg01174264	0.41172	0.208987027	-0.97825	5.65E-05	hypomethylated
cg05675514	0.3516	0.178463243	-0.978308	0.002147	hypomethylated
cg26666580	0.30372	0.154006486	-0.979751	0.000633	hypomethylated
cg15819128	0.58525	0.296625405	-0.980411	0.001141	hypomethylated
cg15391590	0.65602	0.332447027	-0.980615	0.00017	hypomethylated
cg05429895	0.34017	0.17224973	-0.981754	0.001237	hypomethylated
cg03096126	0.39832	0.201663243	-0.98198	0.002106	hypomethylated
cg01077100	0.68944	0.348863784	-0.982761	3.85E-05	hypomethylated
cg09791621	0.57417	0.290507568	-0.982902	0.000857	hypomethylated
cg18335931	0.31278	0.158245946	-0.98298	0.000126	hypomethylated
cg14251009	0.58411	0.295415676	-0.983494	0.00255	hypomethylated

cg21110456	0.43082	0.217836216	-0.983841	0.002777	hypomethylated
cg14654171	0.40111	0.202795135	-0.983975	0.000234	hypomethylated
cg12560128	0.46838	0.236712432	-0.984544	0.001368	hypomethylated
cg14276379	0.51253	0.25898973	-0.984742	0.00032	hypomethylated
cg22207139	0.61731	0.311917838	-0.984829	5.00E-06	hypomethylated
cg23066280	0.70362	0.355437297	-0.985201	0.000667	hypomethylated
cg01826354	0.47797	0.241436757	-0.985275	0.00099	hypomethylated
cg03667317	0.3228	0.163034595	-0.985462	0.000495	hypomethylated
cg21323720	0.56026	0.282911892	-0.985744	0.000138	hypomethylated
cg02923485	0.57519	0.290399459	-0.986	2.36E-06	hypomethylated
cg02227496	0.40032	0.202049189	-0.986447	0.001052	hypomethylated
cg14292522	0.46867	0.236517838	-0.986623	5.06E-05	hypomethylated
cg02482460	0.22705	0.114556757	-0.986947	0.001096	hypomethylated
cg08357990	0.29665	0.149616757	-0.98749	0.000145	hypomethylated
cg04465078	0.46926	0.236655135	-0.987601	0.000563	hypomethylated
cg11303839	0.6876	0.346749189	-0.987677	0.000219	hypomethylated
cg05471169	0.45527	0.229347027	-0.98919	0.003705	hypomethylated
cg04633683	0.54675	0.275278919	-0.989987	7.65E-05	hypomethylated
cg14415629	0.33531	0.168791892	-0.99025	9.49E-05	hypomethylated
cg01238435	0.70792	0.356296216	-0.990509	2.89E-05	hypomethylated
cg04821520	0.47674	0.239844324	-0.991104	0.000129	hypomethylated
cg15832662	0.28905	0.145168649	-0.993589	0.000204	hypomethylated
cg01862311	0.69322	0.347955135	-0.994412	2.92E-05	hypomethylated
cg23238834	0.34219	0.171745405	-0.994526	0.001786	hypomethylated
cg19308375	0.50649	0.254175676	-0.994708	0.006564	hypomethylated
cg11533712	0.51514	0.25849027	-0.994855	2.04E-05	hypomethylated
cg09255732	0.40063	0.200923784	-0.995622	0.005706	hypomethylated
cg21197336	0.70059	0.351291892	-0.9959	4.88E-05	hypomethylated
cg16407699	0.60488	0.303265405	-0.996068	0.000123	hypomethylated
cg08063160	0.27031	0.135495676	-0.996368	4.24E-06	hypomethylated
cg06485603	0.2574	0.128915676	-0.997584	0.000613	hypomethylated
cg25753473	0.54838	0.274527568	-0.998225	0.000772	hypomethylated
cg27139419	0.55304	0.276775135	-0.998669	3.95E-05	hypomethylated
cg13414270	0.52476	0.26248973	-0.999397	0.000262	hypomethylated
cg07248223	0.66147	0.330798919	-0.999721	0.001141	hypomethylated
cg04111435	0.2243	0.112140541	-1.000122	0.000374	hypomethylated
cg11906021	0.61767	0.30876	-1.00035	8.60E-06	hypomethylated
cg25979108	0.22653	0.113216757	-1.000615	0.000253	hypomethylated
cg13780718	0.61671	0.308212432	-1.000667	0.000399	hypomethylated
cg20518446	0.73593	0.367522703	-1.001735	0.000109	hypomethylated
cg17837069	0.28854	0.144084865	-1.001853	0.000857	hypomethylated
cg09287864	0.67989	0.339475676	-1.001993	0.000182	hypomethylated
cg20003368	0.43901	0.219099459	-1.002668	0.003347	hypomethylated
cg17644208	0.33372	0.166518378	-1.002957	0.000613	hypomethylated
cg14661886	0.22513	0.112334054	-1.002963	0.003914	hypomethylated
cg12394201	0.53057	0.264716216	-1.003097	4.05E-05	hypomethylated
cg21568661	0.45958	0.229156216	-1.003985	6.15E-05	hypomethylated
cg09960641	0.52947	0.263909189	-1.004507	1.66E-05	hypomethylated
cg22177308	0.75473	0.375996757	-1.00524	1.88E-06	hypomethylated
cg01141339	0.39576	0.196996757	-1.006454	0.003195	hypomethylated
cg25388882	0.57033	0.283872973	-1.006551	5.14E-06	hypomethylated
cg13942922	0.27921	0.138891892	-1.007388	0.00064	hypomethylated
cg00362657	0.46374	0.230660541	-1.007545	0.011274	hypomethylated
cg14356919	0.6447	0.320614595	-1.007788	1.54E-06	hypomethylated
cg23323669	0.3801	0.188797838	-1.009537	0.000681	hypomethylated
cg04937184	0.55453	0.275401622	-1.009728	0.000152	hypomethylated
cg26758857	0.24239	0.120351892	-1.010071	0.001768	hypomethylated
cg09782560	0.51372	0.255032432	-1.010302	6.70E-05	hypomethylated

cg24438354	0.50152	0.248904865	-1.010713	0.000287	hypomethylated
cg07493562	0.38648	0.191757297	-1.011112	3.40E-05	hypomethylated
cg11935248	0.33797	0.167648108	-1.011459	0.001164	hypomethylated
cg04291025	0.28261	0.140124324	-1.012105	0.000435	hypomethylated
cg06021088	0.4897	0.24274973	-1.012429	0.000174	hypomethylated
cg23937993	0.32992	0.163537838	-1.012492	0.00028	hypomethylated
cg05398036	0.50539	0.250471892	-1.012748	0.000214	hypomethylated
cg12453905	0.29866	0.147955676	-1.013339	0.004022	hypomethylated
cg20803910	0.41783	0.206975135	-1.013459	0.003986	hypomethylated
cg01165043	0.3142	0.155626486	-1.013596	0.00078	hypomethylated
cg01261503	0.20266	0.100355135	-1.013947	0.000123	hypomethylated
cg19894975	0.19791	0.097952973	-1.014683	0.001327	hypomethylated
cg01425762	0.55211	0.273121081	-1.015415	2.92E-05	hypomethylated
cg06853894	0.59754	0.295575135	-1.01551	5.06E-05	hypomethylated
cg05151395	0.28408	0.14048	-1.015933	0.003604	hypomethylated
cg02544257	0.40494	0.200149189	-1.016632	4.00E-05	hypomethylated
cg19174643	0.55737	0.275483784	-1.016668	0.000358	hypomethylated
cg12592365	0.71661	0.354045405	-1.017254	8.15E-06	hypomethylated
cg02892153	0.33287	0.164395676	-1.017787	9.83E-05	hypomethylated
cg13067634	0.25039	0.123656757	-1.017836	0.000224	hypomethylated
cg06261066	0.47813	0.236105946	-1.017969	0.001912	hypomethylated
cg09884146	0.64142	0.316676216	-1.018261	6.95E-05	hypomethylated
cg05832051	0.32407	0.159995135	-1.018277	0.001949	hypomethylated
cg21365602	0.1515	0.074771351	-1.01876	0.001074	hypomethylated
cg15101245	0.32364	0.159704865	-1.018982	0.000178	hypomethylated
cg23271915	0.61957	0.305616216	-1.019546	1.80E-05	hypomethylated
cg16271437	0.19307	0.095013514	-1.022919	0.001096	hypomethylated
cg07541559	0.49749	0.24477027	-1.023239	0.000148	hypomethylated
cg23305899	0.69649	0.342628649	-1.023457	9.31E-06	hypomethylated
cg25450266	0.4407	0.216571892	-1.024951	0.001603	hypomethylated
cg25053413	0.31191	0.153231892	-1.025413	0.004365	hypomethylated
cg13454226	0.45967	0.225664324	-1.02642	0.005131	hypomethylated
cg17951878	0.62537	0.306931892	-1.026791	0.000102	hypomethylated
cg20618695	0.39967	0.196115135	-1.027108	0.000374	hypomethylated
cg13601855	0.30728	0.150754054	-1.027357	0.000733	hypomethylated
cg23720331	0.37906	0.185934595	-1.027631	0.000358	hypomethylated
cg04495354	0.70356	0.345047568	-1.027878	4.05E-05	hypomethylated
cg09451215	0.47799	0.234312432	-1.028547	0.000287	hypomethylated
cg13749927	0.26425	0.129524865	-1.028674	0.0006	hypomethylated
cg20300175	0.23681	0.116065405	-1.028792	0.003316	hypomethylated
cg27147785	0.33669	0.165000541	-1.02895	0.001875	hypomethylated
cg16997203	0.28754	0.140885405	-1.02924	0.000523	hypomethylated
cg03553715	0.3759	0.184074595	-1.030058	0.003166	hypomethylated
cg11348442	0.22528	0.110301081	-1.030272	0.001751	hypomethylated
cg16001422	0.42748	0.209296757	-1.030307	8.03E-05	hypomethylated
cg07153010	0.49273	0.241211351	-1.0305	0.000166	hypomethylated
cg19358397	0.36565	0.178925405	-1.031105	0.000219	hypomethylated
cg10373891	0.20061	0.098164324	-1.031123	3.15E-05	hypomethylated
cg07101841	0.36912	0.180575135	-1.031491	0.002803	hypomethylated
cg03178838	0.50917	0.249075676	-1.031563	0.00504	hypomethylated
cg03064100	0.5166	0.252623784	-1.032057	0.000202	hypomethylated
cg06858555	0.54875	0.268344865	-1.032061	0.000182	hypomethylated
cg05467676	0.4487	0.219322162	-1.0327	0.002147	hypomethylated
cg23694492	0.34226	0.16719027	-1.033602	0.001496	hypomethylated
cg19131313	0.21881	0.106871351	-1.033804	0.002883	hypomethylated
cg12750917	0.40929	0.199812973	-1.034473	5.31E-05	hypomethylated
cg23261919	0.52479	0.256178378	-1.034592	0.002599	hypomethylated
cg21525032	0.31512	0.153820541	-1.034653	0.001651	hypomethylated

cg06114334	0.53061	0.258885946	-1.035335	0.005085	hypomethylated
cg24366211	0.48119	0.234748649	-1.03549	0.006015	hypomethylated
cg25952192	0.48882	0.238470811	-1.035491	4.18E-06	hypomethylated
cg03628719	0.22268	0.108589189	-1.036092	0.000449	hypomethylated
cg13585930	0.56812	0.277014054	-1.036237	9.72E-05	hypomethylated
cg17001034	0.30079	0.146596216	-1.036909	0.001141	hypomethylated
cg07635227	0.61777	0.301048108	-1.037076	4.70E-05	hypomethylated
cg15058210	0.5371	0.261590811	-1.037879	1.66E-05	hypomethylated
cg11756029	0.30651	0.149268108	-1.038028	0.010126	hypomethylated
cg13275603	0.44319	0.21582	-1.038097	1.12E-05	hypomethylated
cg15034300	0.40754	0.198370811	-1.038742	0.003441	hypomethylated
cg20979153	0.3254	0.158349189	-1.039105	0.00071	hypomethylated
cg26612165	0.25087	0.122058919	-1.039362	0.002938	hypomethylated
cg08446038	0.54429	0.264748108	-1.039755	0.000449	hypomethylated
cg08467103	0.60752	0.295453514	-1.040001	0.000274	hypomethylated
cg04202892	0.42426	0.206198919	-1.040912	0.000805	hypomethylated
cg02518245	0.33047	0.16052	-1.041766	0.001875	hypomethylated
cg05579652	0.32826	0.159283784	-1.04324	0.008069	hypomethylated
cg22009908	0.43448	0.210806486	-1.043371	0.000224	hypomethylated
cg21052608	0.31199	0.151341081	-1.043696	0.002526	hypomethylated
cg27477067	0.26301	0.127517297	-1.044425	0.015809	hypomethylated
cg27657983	0.39916	0.193048108	-1.048007	0.000495	hypomethylated
cg19391247	0.47257	0.228485946	-1.048423	0.001354	hypomethylated
cg05309989	0.32518	0.157175676	-1.048861	0.007866	hypomethylated
cg08060857	0.22876	0.11056	-1.049005	0.000534	hypomethylated
cg07382998	0.34973	0.169003784	-1.049186	0.000408	hypomethylated
cg05926314	0.85477	0.413050811	-1.049217	3.59E-06	hypomethylated
cg06590173	0.38031	0.183655676	-1.050172	0.006339	hypomethylated
cg21669326	0.48193	0.232484324	-1.05169	3.40E-05	hypomethylated
cg26110130	0.37526	0.181010811	-1.051815	0.001839	hypomethylated
cg08415592	0.5858	0.282554054	-1.051881	0.000178	hypomethylated
cg15771683	0.36319	0.175094054	-1.052594	0.000187	hypomethylated
cg22417733	0.39997	0.192665405	-1.053794	7.29E-05	hypomethylated
cg03297783	0.30819	0.148363243	-1.054686	0.003773	hypomethylated
cg06788267	0.5743	0.276346486	-1.055326	6.95E-05	hypomethylated
cg24921221	0.40314	0.193896757	-1.055992	5.19E-05	hypomethylated
cg22790839	0.55455	0.266600541	-1.056638	2.13E-05	hypomethylated
cg07474842	0.49693	0.238852432	-1.056923	0.000772	hypomethylated
cg10077672	0.19006	0.091341081	-1.057119	0.000912	hypomethylated
cg16688437	0.27572	0.132421081	-1.058071	0.000148	hypomethylated
cg04951797	0.43643	0.209582703	-1.058231	0.000426	hypomethylated
cg00494337	0.38937	0.186921622	-1.058708	0.000772	hypomethylated
cg09495643	0.49167	0.236	-1.058903	0.004951	hypomethylated
cg10377245	0.25524	0.122484324	-1.059257	0.001367	hypomethylated
cg13323489	0.54278	0.260404865	-1.059611	0.000839	hypomethylated
cg19241689	0.38051	0.182523784	-1.05985	0.014838	hypomethylated
cg08782022	0.64848	0.310971892	-1.060278	0.000109	hypomethylated
cg25225070	0.27521	0.131974054	-1.060279	0.000232	hypomethylated
cg05602356	0.24656	0.118137838	-1.061468	0.00623	hypomethylated
cg07733481	0.34826	0.166857297	-1.06155	0.000399	hypomethylated
cg27359566	0.59848	0.286654054	-1.061992	0.000805	hypomethylated
cg03354554	0.51917	0.248629189	-1.062211	0.00039	hypomethylated
cg21205978	0.39183	0.187625405	-1.062373	0.001968	hypomethylated
cg03280622	0.45913	0.219785946	-1.062804	5.79E-05	hypomethylated
cg12278705	0.35885	0.171667027	-1.063768	0.000703	hypomethylated
cg22579075	0.44121	0.211023784	-1.06406	3.00E-05	hypomethylated
cg20805133	0.50047	0.239342703	-1.064206	0.00113	hypomethylated
cg16561266	0.20884	0.099866486	-1.064326	0.010555	hypomethylated

cg24500959	0.44298	0.211794054	-1.064579	0.000313	hypomethylated
cg23408558	0.65038	0.31076	-1.065482	2.10E-06	hypomethylated
cg03466587	0.53629	0.256090811	-1.066358	0.000931	hypomethylated
cg01254505	0.29105	0.138746486	-1.068816	0.005223	hypomethylated
cg23072383	0.2794	0.133161081	-1.06916	0.006339	hypomethylated
cg03966751	0.47014	0.223757297	-1.071156	8.63E-05	hypomethylated
cg13668314	0.34488	0.164133514	-1.071225	0.001188	hypomethylated
cg04926881	0.56139	0.267135676	-1.071431	0.000123	hypomethylated
cg25799109	0.52741	0.250950811	-1.07152	0.000495	hypomethylated
cg16788286	0.42456	0.201872432	-1.072525	0.006564	hypomethylated
cg03800922	0.33948	0.161378919	-1.072874	0.002106	hypomethylated
cg13905238	0.62717	0.298120541	-1.072961	4.94E-05	hypomethylated
cg21430685	0.45378	0.215575676	-1.073799	0.002409	hypomethylated
cg14019757	0.42499	0.201772973	-1.074696	0.004445	hypomethylated
cg27132471	0.48527	0.230311892	-1.075199	0.000293	hypomethylated
cg20893203	0.51102	0.242425946	-1.075836	0.003637	hypomethylated
cg15017278	0.55182	0.261755676	-1.075977	0.000135	hypomethylated
cg21180572	0.38286	0.181585405	-1.076169	3.67E-05	hypomethylated
cg23309670	0.55834	0.264794054	-1.076273	0.000148	hypomethylated
cg05315321	0.4016	0.190432432	-1.07648	0.000374	hypomethylated
cg12918213	0.36895	0.174937297	-1.076587	0.00255	hypomethylated
cg13985198	0.17322	0.082112973	-1.076923	0.001107	hypomethylated
cg04625338	0.55569	0.263214595	-1.078041	3.23E-05	hypomethylated
cg26389380	0.55574	0.262968108	-1.079522	0.001074	hypomethylated
cg24039081	0.48538	0.229515676	-1.080522	0.000426	hypomethylated
cg27306787	0.23895	0.112984865	-1.080579	0.020743	hypomethylated
cg25343618	0.43365	0.205028108	-1.080709	0.000148	hypomethylated
cg13765004	0.24842	0.117444324	-1.080804	0.005363	hypomethylated
cg00602295	0.6586	0.311347027	-1.080879	1.15E-05	hypomethylated
cg27107970	0.27464	0.129822162	-1.081005	7.38E-05	hypomethylated
cg07146104	0.38731	0.183052973	-1.081228	0.001052	hypomethylated
cg09657615	0.33079	0.15630973	-1.081508	0.021548	hypomethylated
cg02629106	0.28702	0.135601622	-1.081777	0.0006	hypomethylated
cg10949007	0.53376	0.252114595	-1.082112	0.00151	hypomethylated
cg03140412	0.60027	0.283525946	-1.082131	0.002189	hypomethylated
cg22202558	0.39477	0.186257297	-1.083715	0.003986	hypomethylated
cg26191447	0.37454	0.176646486	-1.084255	0.002386	hypomethylated
cg21499869	0.39888	0.187628108	-1.088079	0.011274	hypomethylated
cg07713361	0.23883	0.112325405	-1.0883	0.00591	hypomethylated
cg19416417	0.30435	0.143117297	-1.088533	0.00044	hypomethylated
cg16973527	0.34909	0.163911351	-1.090683	0.000857	hypomethylated
cg14351425	0.29719	0.139342162	-1.092754	0.000123	hypomethylated
cg26015888	0.21466	0.100565946	-1.093912	0.000224	hypomethylated
cg10773016	0.24861	0.116466486	-1.093969	0.001734	hypomethylated
cg03577157	0.59996	0.281004324	-1.094274	9.72E-05	hypomethylated
cg21703606	0.22034	0.103157297	-1.094886	0.000667	hypomethylated
cg05026393	0.19603	0.091732432	-1.095571	0.004059	hypomethylated
cg23777956	0.30994	0.145001081	-1.095925	9.05E-05	hypomethylated
cg02640104	0.2939	0.137383784	-1.097114	0.001571	hypomethylated
cg15787146	0.47761	0.223203243	-1.097475	0.00024	hypomethylated
cg22525688	0.49392	0.230823784	-1.097486	0.000626	hypomethylated
cg23962483	0.28377	0.132578378	-1.097877	0.002253	hypomethylated
cg03157040	0.18357	0.085743243	-1.098235	0.00013	hypomethylated
cg23595451	0.39383	0.183943784	-1.098308	0.000129	hypomethylated
cg08549335	0.63782	0.297854595	-1.098541	5.93E-05	hypomethylated
cg25054907	0.32959	0.153887568	-1.098796	0.002624	hypomethylated
cg27471192	0.43857	0.204632432	-1.099772	4.47E-05	hypomethylated
cg06942649	0.46913	0.218877838	-1.099862	0.000182	hypomethylated

cg23344780	0.38732	0.180601081	-1.100719	0.000152	hypomethylated
cg22689909	0.5218	0.242757838	-1.103979	0.002883	hypomethylated
cg07805542	0.66536	0.309494595	-1.104221	6.31E-05	hypomethylated
cg07388969	0.66828	0.310680541	-1.105021	9.27E-05	hypomethylated
cg16474725	0.59046	0.274460541	-1.105241	0.000445	hypomethylated
cg09564133	0.28075	0.130454054	-1.105744	0.001839	hypomethylated
cg24003508	0.36024	0.167328108	-1.106279	0.000517	hypomethylated
cg14118226	0.31983	0.148472432	-1.10711	0.00102	hypomethylated
cg11847468	0.29379	0.136363243	-1.10733	0.005859	hypomethylated
cg05471495	0.3622	0.168079459	-1.107643	0.002965	hypomethylated
cg09126279	0.5293	0.245553514	-1.108048	0.000485	hypomethylated
cg19672546	0.43827	0.203296216	-1.108237	0.000143	hypomethylated
cg03707168	0.28407	0.131732973	-1.10863	0.000495	hypomethylated
cg03005261	0.2966	0.137515676	-1.108923	0.000613	hypomethylated
cg03518729	0.59274	0.274765405	-1.109199	0.000107	hypomethylated
cg22700686	0.45473	0.210750811	-1.109472	0.000159	hypomethylated
cg09550909	0.25589	0.118575135	-1.109722	0.002478	hypomethylated
cg01156249	0.58664	0.271438919	-1.111848	0.000112	hypomethylated
cg15244327	0.35034	0.162045405	-1.112358	0.002624	hypomethylated
cg06594281	0.25837	0.119491892	-1.112526	0.00275	hypomethylated
cg09803959	0.48972	0.226036757	-1.1154	6.95E-05	hypomethylated
cg25272655	0.26695	0.123124324	-1.116454	0.008932	hypomethylated
cg00834924	0.34873	0.160838378	-1.116499	0.002126	hypomethylated
cg25874782	0.51734	0.238508108	-1.117074	0.001052	hypomethylated
cg01303055	0.28637	0.132009189	-1.117242	0.000893	hypomethylated
cg17694130	0.31671	0.145927568	-1.11791	0.000756	hypomethylated
cg00664697	0.43964	0.202510811	-1.118324	0.000733	hypomethylated
cg27619475	0.2818	0.129777838	-1.118628	0.002147	hypomethylated
cg17988310	0.5843	0.26895027	-1.11937	7.47E-05	hypomethylated
cg02101203	0.32593	0.15	-1.1196	0.001368	hypomethylated
cg04029159	0.55024	0.253061622	-1.120572	0.002106	hypomethylated
cg26704293	0.51356	0.236183243	-1.120626	0.000534	hypomethylated
cg13485320	0.32236	0.148199459	-1.121133	0.000857	hypomethylated
cg18368411	0.41326	0.189913514	-1.121707	0.001164	hypomethylated
cg00539347	0.29931	0.137498919	-1.12222	0.001153	hypomethylated
cg14770102	0.38091	0.174819459	-1.123584	0.000342	hypomethylated
cg26140475	0.25562	0.117313514	-1.123632	0.00012	hypomethylated
cg25508319	0.53267	0.244424324	-1.123854	0.000112	hypomethylated
cg15782228	0.50826	0.233003243	-1.125217	1.26E-05	hypomethylated
cg18672030	0.39562	0.181350811	-1.125332	0.001164	hypomethylated
cg07756483	0.37235	0.17054973	-1.126467	3.85E-05	hypomethylated
cg12406559	0.57304	0.262363784	-1.127067	0.00028	hypomethylated
cg20873416	0.31912	0.146084865	-1.127292	0.000667	hypomethylated
cg14515996	0.40903	0.187196757	-1.127651	0.0006	hypomethylated
cg18082788	0.55718	0.254905405	-1.128182	0.001212	hypomethylated
cg17576316	0.30748	0.140637297	-1.128513	0.002106	hypomethylated
cg27179101	0.42471	0.194062162	-1.129959	7.29E-05	hypomethylated
cg23575688	0.48963	0.223667568	-1.130336	0.000155	hypomethylated
cg03761750	0.20557	0.093788108	-1.132153	0.006855	hypomethylated
cg05060704	0.2417	0.110247568	-1.132471	0.00125	hypomethylated
cg00544436	0.31372	0.142992973	-1.133533	0.002253	hypomethylated
cg05792169	0.54155	0.246825946	-1.1336	0.000185	hypomethylated
cg25349820	0.35325	0.16085027	-1.134971	0.000839	hypomethylated
cg14480249	0.37661	0.171448108	-1.135299	0.002856	hypomethylated
cg12209075	0.47984	0.218440541	-1.135313	0.000138	hypomethylated
cg03477080	0.35111	0.159825405	-1.135426	0.004365	hypomethylated
cg17881007	0.31949	0.145370811	-1.136033	0.024309	hypomethylated
cg05211768	0.3013	0.137089189	-1.136086	0.001141	hypomethylated

cg13786863	0.50862	0.231394054	-1.136236	0.002965	hypomethylated
cg00557308	0.233	0.105956216	-1.136862	0.000639	hypomethylated
cg08824350	0.34316	0.155917838	-1.138095	6.62E-05	hypomethylated
cg17471425	0.48075	0.218352432	-1.138628	0.000534	hypomethylated
cg12060422	0.60193	0.273158919	-1.139855	0.000435	hypomethylated
cg12944183	0.27728	0.125796757	-1.140249	0.003505	hypomethylated
cg14665413	0.37164	0.16852973	-1.140903	4.05E-05	hypomethylated
cg14823429	0.43577	0.197405405	-1.142405	0.00305	hypomethylated
cg21815063	0.47504	0.215174595	-1.142541	0.001176	hypomethylated
cg25518868	0.44925	0.203421081	-1.143049	0.000296	hypomethylated
cg16476991	0.60596	0.274363243	-1.143135	6.62E-05	hypomethylated
cg12273284	0.58098	0.263045946	-1.143174	0.000588	hypomethylated
cg20389635	0.56194	0.254241622	-1.144216	1.99E-05	hypomethylated
cg15063366	0.23637	0.106941622	-1.144223	0.000271	hypomethylated
cg23731089	0.59861	0.270807568	-1.144348	1.21E-05	hypomethylated
cg19262282	0.39119	0.176718919	-1.146413	0.000772	hypomethylated
cg12126901	0.35836	0.161867568	-1.146596	3.40E-05	hypomethylated
cg11122255	0.4421	0.199472432	-1.148183	0.000772	hypomethylated
cg16865138	0.26181	0.118102703	-1.148478	7.74E-05	hypomethylated
cg05971148	0.28189	0.127030811	-1.149954	0.004059	hypomethylated
cg00506866	0.35073	0.157995676	-1.150476	0.000506	hypomethylated
cg26994377	0.517	0.232741081	-1.151438	0.000626	hypomethylated
cg13957558	0.37299	0.167906486	-1.151479	0.00097	hypomethylated
cg02297063	0.4956	0.222967568	-1.152342	0.004776	hypomethylated
cg05981038	0.4263	0.191771892	-1.152478	0.0006	hypomethylated
cg26683425	0.34345	0.154438378	-1.153069	0.002296	hypomethylated
cg26376241	0.53478	0.240381622	-1.153619	6.62E-05	hypomethylated
cg05624478	0.3456	0.155079459	-1.156096	0.002409	hypomethylated
cg02633924	0.37468	0.168062703	-1.156659	0.003378	hypomethylated
cg00950718	0.32993	0.147912973	-1.157411	0.000123	hypomethylated
cg09474442	0.43424	0.194662703	-1.157516	6.08E-05	hypomethylated
cg25433316	0.48496	0.217258919	-1.15845	3.40E-05	hypomethylated
cg15054077	0.46655	0.208957838	-1.15882	0.003286	hypomethylated
cg05673882	0.46871	0.209918919	-1.158863	0.000262	hypomethylated
cg16238993	0.48599	0.217651351	-1.158908	0.003078	hypomethylated
cg01959516	0.3751	0.16797027	-1.159069	0.000129	hypomethylated
cg07937427	0.45053	0.201596757	-1.160151	0.002965	hypomethylated
cg25690715	0.36658	0.163883784	-1.161455	0.002386	hypomethylated
cg19162768	0.4019	0.179671351	-1.161476	0.000575	hypomethylated
cg02791973	0.50999	0.22792973	-1.16188	0.003347	hypomethylated
cg27129922	0.36263	0.162013514	-1.162384	0.003878	hypomethylated
cg12897164	0.45507	0.203215676	-1.163077	9.07E-06	hypomethylated
cg06023661	0.40586	0.181152973	-1.163774	0.000274	hypomethylated
cg13877315	0.37454	0.167141622	-1.164049	0.005557	hypomethylated
cg00973309	0.42232	0.188416216	-1.164413	0.000756	hypomethylated
cg23885932	0.41232	0.183824865	-1.165433	0.00275	hypomethylated
cg13972124	0.4405	0.196188108	-1.166904	0.000204	hypomethylated
cg10890829	0.23843	0.106187027	-1.166958	0.004287	hypomethylated
cg23807071	0.55997	0.249386486	-1.166966	0.002409	hypomethylated
cg24158259	0.47914	0.213235135	-1.168002	0.000772	hypomethylated
cg20218460	0.27596	0.122758378	-1.168638	0.001153	hypomethylated
cg10024587	0.29863	0.132805946	-1.169039	0.003136	hypomethylated
cg16251079	0.25106	0.111545405	-1.170401	0.000588	hypomethylated
cg07482372	0.31278	0.138916757	-1.170928	2.32E-05	hypomethylated
cg16651780	0.25567	0.113525405	-1.171268	0.001968	hypomethylated
cg03921696	0.62557	0.277642703	-1.171942	1.15E-05	hypomethylated
cg21328779	0.34003	0.150827568	-1.172762	7.84E-05	hypomethylated
cg11318133	0.26786	0.118770811	-1.173299	0.005606	hypomethylated

cg14295924	0.38958	0.172657838	-1.174004	4.58E-05	hypomethylated
cg26248066	0.45178	0.200163243	-1.174443	0.000102	hypomethylated
cg04663932	0.32579	0.144235676	-1.175514	0.003604	hypomethylated
cg01288184	0.59928	0.264925405	-1.177644	0.00035	hypomethylated
cg13759905	0.5079	0.224147568	-1.180096	6.31E-05	hypomethylated
cg16047279	0.5464	0.241101622	-1.180316	0.000274	hypomethylated
cg16210842	0.35294	0.155560541	-1.181947	0.001118	hypomethylated
cg18879590	0.29101	0.128181622	-1.182879	0.001466	hypomethylated
cg11116086	0.49457	0.21778973	-1.183239	0.00097	hypomethylated
cg23019886	0.53041	0.233382703	-1.18441	0.000219	hypomethylated
cg16783204	0.50098	0.220395135	-1.184661	4.58E-05	hypomethylated
cg01784327	0.26621	0.117102703	-1.18479	0.00013	hypomethylated
cg20665774	0.27569	0.121242703	-1.185149	0.00024	hypomethylated
cg19184897	0.36362	0.159884865	-1.185398	4.93E-06	hypomethylated
cg20272979	0.49927	0.219435676	-1.186022	0.002147	hypomethylated
cg17714703	0.22062	0.096902162	-1.186963	0.000138	hypomethylated
cg18361815	0.32885	0.144432973	-1.18703	0.005911	hypomethylated
cg19090437	0.55084	0.241770811	-1.187993	7.93E-06	hypomethylated
cg25969122	0.5799	0.254498378	-1.188148	6.01E-05	hypomethylated
cg07902749	0.24771	0.108531892	-1.190533	0.000253	hypomethylated
cg20765408	0.65071	0.285053514	-1.190782	0.000159	hypomethylated
cg04356381	0.35562	0.155759459	-1.191017	0.000756	hypomethylated
cg05675373	0.24531	0.107352973	-1.192244	0.000639	hypomethylated
cg25388800	0.55826	0.243835135	-1.195031	0.000626	hypomethylated
cg04456219	0.56351	0.246088649	-1.195263	0.000245	hypomethylated
cg20867963	0.48307	0.210923243	-1.195514	0.012637	hypomethylated
cg07220152	0.26955	0.117662703	-1.195896	0.001987	hypomethylated
cg01318557	0.50261	0.219342703	-1.196253	0.000588	hypomethylated
cg26221105	0.28044	0.122318378	-1.197051	0.002231	hypomethylated
cg24800655	0.33802	0.147428108	-1.197097	0.005316	hypomethylated
cg06487082	0.44155	0.19238	-1.198618	0.000274	hypomethylated
cg00672333	0.38795	0.169025405	-1.198631	0.003914	hypomethylated
cg16755500	0.30424	0.132403243	-1.200271	0.003773	hypomethylated
cg20928945	0.80003	0.348021081	-1.200879	5.73E-06	hypomethylated
cg24227481	0.47627	0.207158919	-1.201042	0.001314	hypomethylated
cg24287110	0.38058	0.165395676	-1.202278	0.001839	hypomethylated
cg08282819	0.35251	0.152913514	-1.204948	0.000563	hypomethylated
cg06400428	0.33981	0.147401081	-1.204981	0.000902	hypomethylated
cg27093918	0.40152	0.174057838	-1.205905	0.000317	hypomethylated
cg21208682	0.5583	0.241934054	-1.206427	0.000116	hypomethylated
cg07499182	0.43971	0.19050973	-1.206688	0.002502	hypomethylated
cg26577252	0.35236	0.152549189	-1.207776	0.000185	hypomethylated
cg14834675	0.40484	0.175212973	-1.208242	0.002409	hypomethylated
cg05343811	0.45582	0.197228649	-1.208595	0.000111	hypomethylated
cg22827210	0.45553	0.196997297	-1.20937	0.000741	hypomethylated
cg13775050	0.50556	0.21858	-1.209721	0.001987	hypomethylated
cg09940032	0.41042	0.177432432	-1.209831	0.001188	hypomethylated
cg06472476	0.24628	0.106456216	-1.210039	0.001225	hypomethylated
cg27454102	0.3313	0.143144865	-1.210662	0.001452	hypomethylated
cg02054792	0.2978	0.128638378	-1.211023	0.000214	hypomethylated
cg03033176	0.22294	0.096218378	-1.212271	0.001074	hypomethylated
cg16865965	0.47754	0.206069189	-1.212493	2.32E-05	hypomethylated
cg12280317	0.38999	0.168276757	-1.212601	3.32E-05	hypomethylated
cg11152384	0.49295	0.212375135	-1.214826	0.00102	hypomethylated
cg04263436	0.45003	0.193834054	-1.215199	0.00504	hypomethylated
cg08452061	0.3217	0.138366486	-1.217221	0.000395	hypomethylated
cg13860281	0.66831	0.287182703	-1.218549	9.07E-06	hypomethylated
cg03741862	0.24122	0.103505946	-1.220636	0.000912	hypomethylated

cg27481708	0.29014	0.124483784	-1.220791	0.002856	hypomethylated
cg26270195	0.34413	0.147584324	-1.221414	0.003021	hypomethylated
cg18781966	0.30802	0.132085405	-1.221553	4.53E-05	hypomethylated
cg18442362	0.62642	0.268583243	-1.221761	5.45E-05	hypomethylated
cg01899130	0.29526	0.126579459	-1.221943	0.002086	hypomethylated
cg15564619	0.54886	0.235198919	-1.222557	0.000106	hypomethylated
cg15934804	0.32499	0.139227027	-1.222956	0.000112	hypomethylated
cg09288894	0.25188	0.107878378	-1.223331	0.000331	hypomethylated
cg13074866	0.28451	0.121751351	-1.224542	3.95E-05	hypomethylated
cg21213593	0.45105	0.192956757	-1.22501	0.000132	hypomethylated
cg19163395	0.65222	0.278825405	-1.225997	3.12E-06	hypomethylated
cg08679238	0.28114	0.120163243	-1.226293	0.000342	hypomethylated
cg18943383	0.22731	0.097116757	-1.226869	0.00049	hypomethylated
cg21151432	0.52272	0.223250811	-1.227373	0.001212	hypomethylated
cg26361533	0.42602	0.18191027	-1.227694	8.42E-05	hypomethylated
cg06679087	0.54797	0.233925946	-1.228045	0.000166	hypomethylated
cg20175702	0.54054	0.230740541	-1.22813	0.000399	hypomethylated
cg07126235	0.31467	0.134275676	-1.228642	0.010211	hypomethylated
cg16371229	0.4778	0.203861081	-1.22882	0.000296	hypomethylated
cg11626629	0.28004	0.119354054	-1.230385	0.001603	hypomethylated
cg04194674	0.25548	0.108881081	-1.230457	0.003878	hypomethylated
cg12666727	0.40104	0.170613514	-1.233014	0.000931	hypomethylated
cg00068038	0.2623	0.111522703	-1.23388	0.001968	hypomethylated
cg23521140	0.44166	0.187753514	-1.234096	0.009315	hypomethylated
cg03851835	0.5091	0.21628973	-1.234984	8.42E-05	hypomethylated
cg02147797	0.34207	0.145267568	-1.235579	0.000182	hypomethylated
cg14409810	0.39701	0.168506486	-1.236371	0.000667	hypomethylated
cg23072161	0.60983	0.258117297	-1.24038	0.000191	hypomethylated
cg08206881	0.25403	0.107432973	-1.241562	0.001153	hypomethylated
cg23811057	0.46548	0.196624324	-1.243277	9.07E-06	hypomethylated
cg21672292	0.4925	0.207745405	-1.245307	0.000157	hypomethylated
cg24638828	0.44461	0.187310811	-1.247106	8.83E-06	hypomethylated
cg15311822	0.29689	0.124878378	-1.249405	0.000126	hypomethylated
cg10981580	0.26755	0.112465405	-1.250327	0.002856	hypomethylated
cg15229275	0.35085	0.147466486	-1.250467	0.00221	hypomethylated
cg26998850	0.49584	0.208159459	-1.252186	0.00098	hypomethylated
cg21435684	0.54101	0.227104324	-1.2523	0.000551	hypomethylated
cg05209483	0.41798	0.175313514	-1.253497	0.000155	hypomethylated
cg14563196	0.19397	0.08127027	-1.255034	0.000857	hypomethylated
cg03762081	0.31897	0.133594054	-1.255565	0.006015	hypomethylated
cg03989617	0.40809	0.170805946	-1.256529	2.78E-05	hypomethylated
cg05068848	0.51683	0.216278378	-1.2568	0.000178	hypomethylated
cg23889684	0.28334	0.118456216	-1.25818	0.0182	hypomethylated
cg05259836	0.25206	0.105350811	-1.258566	0.002386	hypomethylated
cg08443563	0.57546	0.239945946	-1.262006	1.92E-05	hypomethylated
cg21324456	0.42587	0.177557838	-1.262124	0.002883	hypomethylated
cg07537821	0.39069	0.162859459	-1.262397	6.46E-05	hypomethylated
cg01799671	0.45343	0.189004324	-1.262461	0.000464	hypomethylated
cg01413054	0.5555	0.231197838	-1.264659	0.000135	hypomethylated
cg07118796	0.30952	0.128681622	-1.266227	0.000485	hypomethylated
cg09129067	0.59208	0.245887027	-1.267797	0.000102	hypomethylated
cg23683800	0.34976	0.145037297	-1.269941	0.00035	hypomethylated
cg04645070	0.34393	0.142518378	-1.270967	4.82E-05	hypomethylated
cg10156217	0.35028	0.144843784	-1.274011	0.000725	hypomethylated
cg24593688	0.34175	0.141283784	-1.274345	0.001314	hypomethylated
cg11409101	0.33243	0.137158919	-1.277202	0.001667	hypomethylated
cg21477985	0.45769	0.188797297	-1.277533	0.00012	hypomethylated
cg26168881	0.30411	0.125341081	-1.278734	0.001118	hypomethylated

cg20740711	0.34645	0.142674595	-1.279919	5.72E-05	hypomethylated
cg05614013	0.28782	0.118485946	-1.280451	7.38E-05	hypomethylated
cg26502852	0.38648	0.158958378	-1.281745	0.004691	hypomethylated
cg04772025	0.40348	0.165761622	-1.283387	0.000772	hypomethylated
cg09015280	0.47687	0.195879459	-1.28363	0.000271	hypomethylated
cg13324103	0.41333	0.169743243	-1.28394	0.001164	hypomethylated
cg19516647	0.16043	0.065834595	-1.285026	0.000893	hypomethylated
cg15275625	0.38085	0.156133514	-1.286443	0.000143	hypomethylated
cg07688604	0.34806	0.142508649	-1.288287	0.003773	hypomethylated
cg14290616	0.16225	0.066417838	-1.288576	0.002599	hypomethylated
cg06530983	0.23698	0.097002703	-1.288668	6.54E-05	hypomethylated
cg12534147	0.50086	0.204602162	-1.291586	0.002046	hypomethylated
cg12026563	0.31963	0.130312973	-1.294422	0.00421	hypomethylated
cg15128801	0.25728	0.104845946	-1.295068	0.010042	hypomethylated
cg21571658	0.46162	0.188066486	-1.295463	0.001481	hypomethylated
cg06061086	0.5255	0.213625946	-1.298604	0.001949	hypomethylated
cg04794141	0.45332	0.184143784	-1.299697	8.63E-05	hypomethylated
cg19310148	0.23369	0.094924324	-1.299746	0.001667	hypomethylated
cg07576409	0.23802	0.09660973	-1.300842	0.004733	hypomethylated
cg27181375	0.38257	0.155235135	-1.301269	0.000159	hypomethylated
cg13278795	0.29142	0.118139459	-1.302609	0.000646	hypomethylated
cg13326508	0.43081	0.174619459	-1.302837	0.000506	hypomethylated
cg14516948	0.27447	0.111081081	-1.305035	0.002066	hypomethylated
cg03317505	0.287	0.116091892	-1.305784	0.00101	hypomethylated
cg16518772	0.3353	0.135549189	-1.306636	0.000331	hypomethylated
cg25416067	0.38	0.153474054	-1.308005	0.001074	hypomethylated
cg22274117	0.53154	0.214634054	-1.308299	0.000293	hypomethylated
cg20645040	0.36996	0.149235135	-1.309782	0.000209	hypomethylated
cg00446414	0.34519	0.13916973	-1.310545	0.004906	hypomethylated
cg15677957	0.42355	0.170691892	-1.311138	0.000822	hypomethylated
cg26051755	0.27293	0.109911351	-1.312191	0.000134	hypomethylated
cg10893986	0.37466	0.150584865	-1.315005	0.002502	hypomethylated
cg01827726	0.27068	0.108787568	-1.315075	0.001987	hypomethylated
cg08692423	0.26111	0.10493027	-1.315227	0.003637	hypomethylated
cg00082497	0.21819	0.087597838	-1.316618	0.001541	hypomethylated
cg14753321	0.35049	0.140632432	-1.317444	0.003225	hypomethylated
cg04403415	0.2848	0.114256216	-1.317676	9.05E-05	hypomethylated
cg22047295	0.55582	0.222937838	-1.317976	0.001141	hypomethylated
cg14204735	0.38239	0.15336	-1.318123	0.001262	hypomethylated
cg05336982	0.39373	0.157843243	-1.318714	0.001409	hypomethylated
cg10087036	0.49803	0.199565405	-1.319371	0.000789	hypomethylated
cg06747543	0.29279	0.11721027	-1.320767	0.002938	hypomethylated
cg14972576	0.53137	0.212382162	-1.323054	4.58E-05	hypomethylated
cg16066354	0.41753	0.166775135	-1.323976	0.002599	hypomethylated
cg26928858	0.33043	0.131767027	-1.326355	0.017643	hypomethylated
cg19869746	0.32156	0.127988649	-1.329072	0.000303	hypomethylated
cg20448053	0.2349	0.093482703	-1.329275	0.004133	hypomethylated
cg23274561	0.30257	0.120376216	-1.329719	0.000417	hypomethylated
cg19595750	0.348	0.138376216	-1.330491	0.005962	hypomethylated
cg18572413	0.28182	0.111921622	-1.332285	0.000358	hypomethylated
cg14248553	0.28235	0.112089189	-1.332837	0.001096	hypomethylated
cg13656752	0.4773	0.189457838	-1.33302	0.001031	hypomethylated
cg06433467	0.41578	0.165014054	-1.333231	0.000653	hypomethylated
cg12521167	0.28129	0.111585946	-1.333903	0.000626	hypomethylated
cg09956615	0.33914	0.134347568	-1.335911	6.78E-05	hypomethylated
cg16389901	0.55342	0.219157838	-1.336405	0.000495	hypomethylated
cg24713529	0.18425	0.072905946	-1.337556	0.002231	hypomethylated
cg07716663	0.28733	0.113648649	-1.338128	0.004209	hypomethylated

cg22473727	0.33565	0.132743243	-1.338319	0.000839	hypomethylated
cg21956614	0.32582	0.128467568	-1.342671	0.000111	hypomethylated
cg25181684	0.37153	0.146017838	-1.347334	0.000696	hypomethylated
cg23175074	0.36734	0.144322703	-1.347818	0.000674	hypomethylated
cg07348922	0.31619	0.124197838	-1.348152	0.003473	hypomethylated
cg15840891	0.31722	0.12433027	-1.351306	0.000688	hypomethylated
cg02585906	0.49177	0.192584865	-1.352489	0.001987	hypomethylated
cg06552563	0.33451	0.130765405	-1.355068	0.00513	hypomethylated
cg06210447	0.30208	0.118041622	-1.355635	0.001838	hypomethylated
cg07030727	0.4587	0.179125946	-1.356577	1.66E-05	hypomethylated
cg05664072	0.23505	0.091556757	-1.360229	0.001556	hypomethylated
cg02131967	0.31445	0.122377297	-1.361495	0.000639	hypomethylated
cg05337743	0.24782	0.096442703	-1.361549	0.002167	hypomethylated
cg15978561	0.54157	0.210692432	-1.362009	2.57E-05	hypomethylated
cg12435551	0.41025	0.159498919	-1.362957	0.001466	hypomethylated
cg06976598	0.55031	0.21390973	-1.363242	0.000135	hypomethylated
cg00506168	0.33656	0.130801622	-1.363483	0.001176	hypomethylated
cg23654821	0.25792	0.100125405	-1.365116	0.000153	hypomethylated
cg23322242	0.26314	0.102031351	-1.366818	0.000626	hypomethylated
cg20834178	0.49692	0.19226973	-1.369882	0.00024	hypomethylated
cg01616225	0.42802	0.165581081	-1.37014	5.19E-05	hypomethylated
cg21853021	0.40574	0.156842162	-1.371242	0.002649	hypomethylated
cg23305567	0.28882	0.111499459	-1.373134	0.003049	hypomethylated
cg19084031	0.3325	0.128343784	-1.373341	0.00291	hypomethylated
cg04098194	0.21495	0.082851351	-1.375404	0.000153	hypomethylated
cg26388816	0.25445	0.097961622	-1.377094	0.000857	hypomethylated
cg11006453	0.62106	0.239079459	-1.377242	6.93E-06	hypomethylated
cg21526750	0.49173	0.189207027	-1.377901	0.00035	hypomethylated
cg19671553	0.42071	0.161858378	-1.378094	0.00094	hypomethylated
cg25095994	0.3977	0.152988108	-1.378261	0.002363	hypomethylated
cg20799268	0.33443	0.128557838	-1.379287	0.000588	hypomethylated
cg22234930	0.43433	0.166805405	-1.380626	0.00049	hypomethylated
cg09417692	0.45066	0.172918919	-1.381944	0.00032	hypomethylated
cg14150907	0.31824	0.122083243	-1.38225	0.000772	hypomethylated
cg01224366	0.34403	0.131812432	-1.384048	0.000354	hypomethylated
cg01787285	0.27735	0.106237297	-1.384417	0.000979	hypomethylated
cg16957313	0.29331	0.112312973	-1.384902	0.000551	hypomethylated
cg23037321	0.33764	0.129280541	-1.384981	0.000931	hypomethylated
cg20968743	0.43682	0.167228649	-1.385217	0.00017	hypomethylated
cg23894287	0.30671	0.117320541	-1.38642	0.000839	hypomethylated
cg18020065	0.48264	0.184547027	-1.386959	0.000674	hypomethylated
cg24631526	0.47716	0.182208108	-1.388886	0.000528	hypomethylated
cg20557104	0.35044	0.133767027	-1.389445	0.000313	hypomethylated
cg09621958	0.30602	0.116368108	-1.39493	2.57E-05	hypomethylated
cg04466840	0.51803	0.196767568	-1.396543	0.000866	hypomethylated
cg19516340	0.48623	0.184518919	-1.39787	0.000501	hypomethylated
cg15862165	0.3831	0.145117838	-1.400496	0.000166	hypomethylated
cg16367976	0.15281	0.057882703	-1.400535	9.05E-05	hypomethylated
cg19950069	0.28085	0.106287568	-1.401827	0.001912	hypomethylated
cg04387396	0.30417	0.115079459	-1.402248	0.000346	hypomethylated
cg06197966	0.38038	0.14356	-1.405788	0.001751	hypomethylated
cg25338563	0.3503	0.132062162	-1.407374	3.81E-05	hypomethylated
cg02954562	0.48306	0.181703243	-1.410618	4.26E-05	hypomethylated
cg08412913	0.35543	0.133654595	-1.411056	0.00017	hypomethylated
cg18178715	0.47878	0.179994054	-1.411414	0.000756	hypomethylated
cg19250790	0.14857	0.055743784	-1.41426	0.001301	hypomethylated
cg20942867	0.45634	0.171180541	-1.41459	0.000145	hypomethylated
cg00971050	0.35385	0.132482703	-1.417334	0.001821	hypomethylated

cg01484686	0.2751	0.102776757	-1.420442	0.000358	hypomethylated
cg07339236	0.39706	0.148200541	-1.421806	1.54E-05	hypomethylated
cg11228682	0.49989	0.185818378	-1.427717	0.009959	hypomethylated
cg09825309	0.36508	0.135582162	-1.429045	1.35E-05	hypomethylated
cg04935109	0.35424	0.131192432	-1.433043	0.000805	hypomethylated
cg12221087	0.29581	0.109506486	-1.433654	0.00504	hypomethylated
cg24789596	0.26244	0.097112973	-1.434252	0.002363	hypomethylated
cg23047271	0.34642	0.127812973	-1.438488	0.000327	hypomethylated
cg26520930	0.27304	0.100014595	-1.448902	0.000756	hypomethylated
cg03663556	0.50213	0.183554054	-1.451856	2.71E-05	hypomethylated
cg17683908	0.22311	0.081463243	-1.453534	0.000148	hypomethylated
cg23558337	0.22697	0.082815676	-1.454526	0.000163	hypomethylated
cg14692854	0.19751	0.072045405	-1.454947	0.000166	hypomethylated
cg18886444	0.21563	0.078584324	-1.456244	0.000581	hypomethylated
cg08995609	0.31033	0.112615676	-1.462396	0.000681	hypomethylated
cg01573472	0.2112	0.076628649	-1.462654	0.00234	hypomethylated
cg15701237	0.24601	0.089224865	-1.463199	0.005858	hypomethylated
cg02983090	0.40005	0.145087027	-1.463262	0.000725	hypomethylated
cg13070763	0.37301	0.135205946	-1.464056	0.000594	hypomethylated
cg09889848	0.44749	0.16213027	-1.464702	0.000374	hypomethylated
cg08076266	0.3042	0.110152973	-1.465512	0.000902	hypomethylated
cg23844623	0.15069	0.054525405	-1.466583	0.000522	hypomethylated
cg23708763	0.33762	0.121708649	-1.471969	0.000789	hypomethylated
cg26650359	0.38383	0.138315676	-1.472503	3.40E-05	hypomethylated
cg13157980	0.3938	0.141658919	-1.475042	3.15E-05	hypomethylated
cg21708130	0.54069	0.194205946	-1.477214	3.85E-05	hypomethylated
cg17130457	0.47143	0.169265405	-1.477756	0.000126	hypomethylated
cg06665109	0.34282	0.123037297	-1.478356	0.011744	hypomethylated
cg09055236	0.39654	0.141980541	-1.481773	0.000408	hypomethylated
cg08226590	0.31589	0.113071892	-1.482182	0.001212	hypomethylated
cg06530961	0.33956	0.12149027	-1.482826	0.000214	hypomethylated
cg08989979	0.15203	0.054387027	-1.483022	0.000435	hypomethylated
cg03367387	0.47085	0.168265405	-1.484529	0.00054	hypomethylated
cg26084949	0.34228	0.122308649	-1.484651	8.63E-05	hypomethylated
cg10536999	0.39709	0.141356216	-1.490131	0.003878	hypomethylated
cg12457415	0.34735	0.123588649	-1.490844	0.000135	hypomethylated
cg20517697	0.27577	0.097716757	-1.496788	0.001212	hypomethylated
cg12864389	0.45399	0.160695676	-1.498329	7.03E-05	hypomethylated
cg23145794	0.34057	0.120539459	-1.498446	0.000931	hypomethylated
cg25250968	0.44142	0.155999459	-1.500611	8.84E-05	hypomethylated
cg25694349	0.31111	0.109675135	-1.504188	0.006973	hypomethylated
cg04650676	0.34774	0.122527568	-1.504903	0.002776	hypomethylated
cg23804620	0.29326	0.103175135	-1.507085	0.00101	hypomethylated
cg26365553	0.36779	0.129297297	-1.50819	0.000256	hypomethylated
cg07190763	0.3382	0.11884	-1.508856	0.00275	hypomethylated
cg08640824	0.33359	0.117131351	-1.509949	0.00097	hypomethylated
cg06616710	0.30878	0.108357838	-1.510776	0.000271	hypomethylated
cg26824216	0.19811	0.069415135	-1.51298	0.001911	hypomethylated
cg09418984	0.35724	0.124947027	-1.515577	0.010126	hypomethylated
cg18166947	0.18193	0.063364324	-1.521641	0.000517	hypomethylated
cg24965479	0.44636	0.155341622	-1.522763	0.000563	hypomethylated
cg26353296	0.2578	0.089624324	-1.52429	0.001949	hypomethylated
cg13600477	0.27965	0.097126486	-1.525686	0.002937	hypomethylated
cg21912556	0.52164	0.180945946	-1.527496	0.000123	hypomethylated
cg02853152	0.45817	0.157748108	-1.53826	0.000214	hypomethylated
cg24851858	0.31358	0.107637838	-1.542648	0.00094	hypomethylated
cg13393476	0.2836	0.096863784	-1.549828	0.000421	hypomethylated
cg00474080	0.2643	0.090267027	-1.549905	0.00305	hypomethylated

cg01802545	0.22551	0.076854595	-1.552988	6.15E-05	hypomethylated
cg09410512	0.45426	0.154003243	-1.560558	6.01E-05	hypomethylated
cg09920557	0.24831	0.084156216	-1.561001	0.002937	hypomethylated
cg15896084	0.32731	0.110770811	-1.56308	0.003107	hypomethylated
cg15754548	0.23954	0.080981622	-1.5646	0.006678	hypomethylated
cg08055087	0.36128	0.121446486	-1.572797	0.000517	hypomethylated
cg17766007	0.18288	0.061032432	-1.583249	0.000674	hypomethylated
cg02413040	0.25288	0.084307027	-1.584728	0.0017	hypomethylated
cg05876246	0.30198	0.100658378	-1.584986	0.000581	hypomethylated
cg00091044	0.19754	0.065611892	-1.590116	0.000511	hypomethylated
cg18002814	0.22602	0.075058919	-1.590355	0.004286	hypomethylated
cg05143530	0.5036	0.166589189	-1.595984	0.000182	hypomethylated
cg19513321	0.24818	0.081388649	-1.608487	0.000219	hypomethylated
cg09923107	0.2013	0.065951351	-1.609873	0.000788	hypomethylated
cg12641569	0.20623	0.067477838	-1.611769	0.016058	hypomethylated
cg25131452	0.24271	0.079289189	-1.614037	0.004134	hypomethylated
cg04786142	0.54892	0.178791351	-1.618319	6.46E-05	hypomethylated
cg01973676	0.53295	0.173366486	-1.620175	0.00014	hypomethylated
cg15281283	0.3382	0.109937297	-1.621196	0.000588	hypomethylated
cg21333861	0.34945	0.113287568	-1.625097	0.001451	hypomethylated
cg10633838	0.29269	0.09481027	-1.626258	0.001314	hypomethylated
cg00498024	0.30462	0.098300541	-1.631739	0.004134	hypomethylated
cg14043253	0.36036	0.115964865	-1.635751	0.00151	hypomethylated
cg12405258	0.30979	0.099512432	-1.638342	0.006229	hypomethylated
cg05656374	0.28004	0.089910811	-1.639066	0.000262	hypomethylated
cg20289346	0.21807	0.069532973	-1.649022	0.000949	hypomethylated
cg21961202	0.2033	0.063971351	-1.668112	0.000224	hypomethylated
cg18422268	0.26394	0.08236	-1.680194	0.000346	hypomethylated
cg01889574	0.34526	0.106043784	-1.703023	0.002883	hypomethylated
cg02231066	0.36088	0.109879459	-1.715597	0.005411	hypomethylated
cg18437710	0.32802	0.0996	-1.719566	0.00028	hypomethylated
cg16730737	0.20579	0.062085946	-1.728834	0.00093	hypomethylated
cg01308827	0.3547	0.106971892	-1.729368	0.001635	hypomethylated
cg09887589	0.23849	0.071081622	-1.74638	0.000234	hypomethylated
cg04987499	0.26885	0.079844865	-1.75153	0.000178	hypomethylated
cg22291084	0.26972	0.078570811	-1.779397	0.0002	hypomethylated
cg06936290	0.51503	0.149116216	-1.788219	2.64E-05	hypomethylated
cg05101437	0.3326	0.096129189	-1.790742	0.000545	hypomethylated
cg13365340	0.32069	0.09170973	-1.806033	0.00095	hypomethylated
cg18516619	0.29976	0.084620541	-1.824728	0.00095	hypomethylated
cg04560810	0.27667	0.077191351	-1.841655	0.002296	hypomethylated
cg08105396	0.29109	0.079989189	-1.863588	0.009083	hypomethylated
cg04111789	0.4779	0.127848649	-1.902272	0.001409	hypomethylated
cg24500441	0.21687	0.057278378	-1.920768	0.000129	hypomethylated
cg11997708	0.27353	0.072076757	-1.924093	0.012034	hypomethylated
cg25360181	0.24968	0.065677838	-1.926602	0.000142	hypomethylated
cg02386420	0.24931	0.06532973	-1.932129	0.000884	hypomethylated
cg11385003	0.2498	0.065010811	-1.942022	0.000346	hypomethylated
cg22627826	0.19731	0.051007027	-1.951696	0.000168	hypomethylated
cg10959711	0.24777	0.064001622	-1.952821	4.05E-05	hypomethylated
cg06075789	0.29797	0.07566	-1.977564	6.01E-05	hypomethylated
cg03130910	0.34757	0.08823027	-1.977958	0.000224	hypomethylated
cg02229757	0.29028	0.073394595	-1.983699	0.00049	hypomethylated
cg19664267	0.35898	0.090607027	-1.986209	0.001785	hypomethylated
cg27373972	0.26243	0.066191351	-1.987218	0.000893	hypomethylated
cg13118906	0.23707	0.058794595	-2.011558	0.00495	hypomethylated
cg15543281	0.31775	0.078367568	-2.019564	0.002209	hypomethylated
cg04659689	0.41628	0.101029189	-2.042782	0.00078	hypomethylated

cg27098900	0.34451	0.082672973	-2.059058	0.000653	hypomethylated
cg11772919	0.33696	0.077853514	-2.113743	0.000979	hypomethylated
cg01383799	0.32893	0.074508108	-2.142311	0.0003	hypomethylated
cg07675334	0.27025	0.060473514	-2.159919	0.015316	hypomethylated
cg00813746	0.24997	0.053176757	-2.232887	0.003049	hypomethylated
cg04588455	0.27194	0.055867568	-2.283205	0.000357	hypomethylated
