# Supplementary material for: CpG Island Methylator Phenotype Modulates the Immune Response of the Tumor Microenvironment and Influences the Prognosis of Pancreatic Cancer Patients
Source: J Oncol. 2021 Nov 28;2021:2715694. doi: 10.1155/2021/2715694 (PMC8645373; doi:10.1155/2021/2715694)
Supplement: Supplementary Materials — Supplementary Figure 1: the most significant 25 hypermethylated CpGs and the most 25 hypomethylated CpGs between 184 PC patients and 10 normal controls by the Wilcoxon test. Supplementary Figure 2: the most significant 25 worse overall survival-related CpGs and the most 25 better overall survival-related CpGs in PC patients by univariate Cox analysis. The hazard ratio (HR) and the 95% confidence interval of the HR were log10-transformed. Supplementary Material 1: it summarizes the differential CpGs between 184 PC patients and 10 normal controls by the Wilcoxon test. In total, 22,450 differential CpGs were identified between 184 PC patients and 10 normal controls (P < 0.05). Among these CpGs, 12,937 were hypermethylated CpGs (log2FC > 0), while 9,513 were hypomethylated CpGs (log2FC < 0). Supplementary Material 2: it summarizes the overall survival- (OS-) related CpGs in PC patients by univariate Cox analysis. In total, 3102 CpGs were found to be related with OS in PC patients (P < 0.05). Among these CpGs, 2858 CpGs were found to be associated with worse OS of PC patients (HR > 1), while 244 were found to be associated with better OS of PC patients (HR < 1). Supplementary Material 3: it summarizes the most OS-related CpGs in PC patients by multivariate Cox analysis. In order to identify the most OS-related CpGs, only 1073 out of 3102 OS-related CpGs with P < 0.01 were used for multivariate Cox analysis, and 72 CpGs were found to be the most OS-related CpGs and they were finally selected for unsupervised consensus clustering analysis. [file 2715694.f1.zip › 2715694.f1/Supplementary material 2 (1).pdf]

Overall survival-related CpGs in 184 pancreatic cancer patients identified by univariate Cox analysis

CpG	HR	HR.95L	HR.95H	pvalue
cg05026393	164.3354	3.331608	8106.033	0.010317
cg13512069	62.65259	8.162247	480.9149	6.92E-05
cg20132590	55.40887	9.918279	309.5439	4.79E-06
cg05812269	45.81789	4.210697	498.5586	0.001687
cg14290616	37.04429	3.475027	394.8975	0.002775
cg16701059	34.72601	5.433231	221.9482	0.000178
cg08039116	29.14275	4.951655	171.5184	0.000192
cg08061040	28.60071	2.575058	317.6631	0.006334
cg20218460	27.8	1.832204	421.8088	0.016559
cg17683908	26.33651	1.331196	521.0439	0.031729
cg09017434	24.40644	4.208447	141.5425	0.000368
cg13188098	23.17646	3.463103	155.106	0.001192
cg07609862	23.13177	3.698055	144.6919	0.000785
cg16399049	21.43512	4.401576	104.3863	0.000148
cg07867924	21.13815	1.54847	288.5568	0.022146
cg07143083	20.41666	3.747701	111.2256	0.000488
cg20019985	19.81928	3.763366	104.3757	0.000426
cg20779964	19.42531	3.843747	98.17058	0.000332
cg05903444	19.00844	3.842795	94.02547	0.000306
cg00498024	17.62235	2.166653	143.3303	0.007297
cg22374057	17.52056	2.881188	106.5429	0.001878
cg13371839	17.26727	2.396839	124.3966	0.00469
cg14661886	16.97419	1.47254	195.664	0.023194
cg12431879	16.93039	3.37296	84.98114	0.000588
cg23572163	16.31347	1.673065	159.0669	0.016266
cg12180703	16.0849	2.716004	95.25907	0.002206
cg14754787	15.98741	3.47206	73.6155	0.000374
cg23479922	15.91963	3.4648	73.14549	0.000375
cg03140968	15.86603	3.834097	65.65584	0.000136
cg04222358	15.76529	3.033946	81.9212	0.001038
cg20667845	15.61761	1.631751	149.4772	0.017086
cg22627826	15.60231	1.071755	227.1341	0.044359
cg10659886	15.17274	2.698686	85.30526	0.002023
cg11473001	15.11224	2.815381	81.11863	0.001539
cg06854015	14.9935	1.231785	182.5036	0.033716
cg08858649	14.94305	3.099793	72.03536	0.000753
cg09907509	14.81361	2.872765	76.38745	0.001278
cg24720580	14.76104	1.387255	157.0645	0.025663
cg09124223	14.71519	3.137651	69.01238	0.000649
cg15549927	14.56896	3.205985	66.20571	0.000524
cg22392666	14.56171	4.577742	46.32049	5.72E-06
cg06383163	14.17756	3.066938	65.53875	0.000687
cg09469566	13.61536	2.625878	70.59658	0.001873
cg20049415	13.20286	3.073189	56.72135	0.000521
cg06659727	13.00331	2.698416	62.66125	0.001388
cg25415246	12.91111	3.07673	54.17985	0.000473
cg11936688	12.91069	2.410094	69.16155	0.002815
cg19054524	12.70046	4.018014	40.14461	1.50E-05
cg21694941	12.58618	2.314592	68.44052	0.003375
cg18940047	12.50794	3.236711	48.33563	0.000249
cg02623400	12.37871	2.51921	60.82558	0.001952
cg20289346	12.34167	1.607346	94.76294	0.01568

cg06360427	12.32398	3.241276	46.85825	0.000228
cg01100175	12.30344	2.779854	54.45416	0.000943
cg00924143	12.28278	1.609105	93.75812	0.015578
cg25307168	12.2585	2.466609	60.92204	0.002187
cg06906472	12.19953	3.256491	45.70214	0.000206
cg00872726	12.14166	2.60248	56.64591	0.001487
cg07531228	12.06477	1.836402	79.26294	0.00952
cg13338350	12.04521	1.537883	94.34209	0.017797
cg26249873	11.84105	2.98682	46.94308	0.000436
cg19161124	11.71943	2.622124	52.37934	0.001274
cg01371799	11.5903	2.717466	49.43396	0.00093
cg11325267	11.58274	2.325809	57.68306	0.002786
cg20260127	11.36614	2.516443	51.33803	0.00158
cg02842227	11.135	2.330716	53.1975	0.002524
cg19107469	11.11184	2.415369	51.11968	0.001985
cg16956426	11.10066	2.653977	46.43021	0.000978
cg25090514	11.05143	2.387874	51.14762	0.002116
cg06304097	10.99895	3.379942	35.79258	6.81E-05
cg04317399	10.84121	2.978205	39.46402	0.0003
cg04764012	10.73596	2.147038	53.6836	0.003847
cg10132208	10.62216	1.79984	62.6891	0.009086
cg06462347	10.45683	3.414797	32.021	3.94E-05
cg24054190	10.45175	2.944262	37.10237	0.000283
cg25397945	10.31078	2.403969	44.22362	0.001686
cg02547394	10.30984	2.329762	45.62386	0.002109
cg09936426	10.3054	2.589782	41.00784	0.000932
cg20557104	10.29152	1.652136	64.10821	0.012493
cg14096889	10.28472	2.463967	42.92889	0.001389
cg04900080	10.2771	2.510868	42.06463	0.001194
cg05502701	10.2737	2.711777	38.92242	0.000608
cg17495912	10.17919	2.515813	41.18588	0.001139
cg01150683	10.16976	2.12853	48.58944	0.003653
cg08204280	10.15946	2.854973	36.15255	0.000344
cg03730428	10.15479	2.560167	40.27857	0.000977
cg06469345	10.13883	2.274921	45.18658	0.002382
cg00297721	10.12488	1.9127	53.59607	0.006475
cg01454487	10.12463	2.215642	46.26568	0.002825
cg15754548	10.07993	2.177718	46.65665	0.003122
cg07382920	9.943472	2.793684	35.39149	0.000391
cg07502439	9.899923	2.194512	44.66073	0.002859
cg03012170	9.895563	2.066002	47.39693	0.004133
cg03840594	9.868868	1.844493	52.80289	0.007464
cg17322443	9.857262	2.133303	45.54702	0.003387
cg21609339	9.811775	2.4058	40.01617	0.001453
cg22762091	9.765841	2.106403	45.27701	0.003593
cg16123202	9.746279	2.725347	34.85426	0.000462
cg24740218	9.689054	1.984748	47.29958	0.004995
cg25403721	9.663907	2.547045	36.66645	0.000856
cg10443187	9.646807	1.651886	56.33616	0.011822
cg00397673	9.641701	2.168169	42.87599	0.002916
cg23668184	9.610612	2.640532	34.97926	0.000597
cg02485566	9.59426	1.741524	52.85591	0.0094
cg23016129	9.589959	2.100753	43.77826	0.003522
cg19922137	9.572453	2.295376	39.9202	0.001933

cg05337743	9.542493	2.013035	45.23477	0.004495
cg17391928	9.515969	2.495765	36.28292	0.000969
cg04510788	9.505973	2.271471	39.78193	0.002047
cg04339613	9.499117	1.948187	46.31652	0.005353
cg15989068	9.446021	1.940477	45.98214	0.00542
cg02039267	9.39332	2.237838	39.42843	0.002209
cg16732616	9.391446	2.602531	33.8898	0.000624
cg26351229	9.303509	1.837274	47.1107	0.00704
cg25562664	9.251193	1.922814	44.51007	0.005509
cg03574723	9.242032	2.010109	42.4928	0.004277
cg10158541	9.238533	2.20145	38.77013	0.002379
cg00811065	9.210719	2.643643	32.09108	0.000489
cg17555373	9.156444	1.745507	48.03216	0.008827
cg26894523	9.147476	1.857632	45.04461	0.006501
cg00945234	9.126364	2.438983	34.14969	0.001023
cg04711162	9.074028	2.152454	38.25308	0.002662
cg25916711	9.063135	2.123557	38.68058	0.00291
cg10721834	9.052266	2.416061	33.91617	0.00108
cg05353133	9.015279	1.644836	49.41237	0.0113
cg12699371	8.992239	2.475695	32.66168	0.000845
cg14714046	8.958078	2.021713	39.69267	0.003892
cg18247055	8.946127	2.104771	38.02466	0.002998
cg26452056	8.939692	2.274671	35.1339	0.001708
cg01003961	8.924792	1.6042	49.65211	0.012429
cg06511917	8.923299	1.522526	52.29813	0.01527
cg09471659	8.894789	1.419463	55.73744	0.019593
cg22620221	8.831105	2.444564	31.9028	0.000887
cg09685182	8.816853	2.062584	37.68908	0.003317
cg02187214	8.78501	1.974167	39.09315	0.004332
cg22131234	8.759318	2.45749	31.22115	0.000818
cg04077662	8.730641	2.256393	33.7814	0.001697
cg24398479	8.729826	2.710728	28.11417	0.000282
cg05184456	8.709857	2.281989	33.24364	0.001539
cg20449685	8.702066	2.129844	35.55469	0.002589
cg12460133	8.685485	2.417959	31.1989	0.000922
cg21300373	8.680302	2.057079	36.62847	0.003263
cg13688769	8.647529	2.08299	35.90021	0.002975
cg14353137	8.642516	2.673168	27.94178	0.000315
cg07682600	8.608055	2.375423	31.19386	0.001049
cg18217632	8.574782	2.237221	32.86528	0.001721
cg26931862	8.566532	1.883493	38.96243	0.005449
cg17713613	8.556322	2.276223	32.16322	0.001486
cg06000994	8.554329	1.846631	39.62705	0.006067
cg11344566	8.544372	2.27773	32.05221	0.001471
cg05076914	8.537907	2.3314	31.26699	0.001203
cg22638505	8.531575	2.143559	33.9565	0.002351
cg25756635	8.519157	2.208998	32.85474	0.001866
cg22752533	8.447472	1.879766	37.96206	0.005383
cg25701114	8.441352	2.228434	31.97601	0.001694
cg08795964	8.42968	2.216526	32.05895	0.001761
cg27376182	8.407778	2.73524	25.84443	0.000202
cg20332645	8.38072	1.975165	35.55979	0.003939
cg19081437	8.37877	2.209684	31.77097	0.001773
cg02101203	8.364843	1.699479	41.1718	0.008997

cg03817667	8.311164	2.977488	23.19923	5.27E-05
cg13944175	8.306246	2.293623	30.08068	0.001263
cg08279008	8.29061	2.933331	23.43214	6.61E-05
cg11293190	8.288094	1.584378	43.35614	0.012242
cg03593833	8.285417	1.440907	47.64231	0.017825
cg06719900	8.282579	2.031111	33.77517	0.003198
cg03544320	8.279706	2.047073	33.48856	0.003029
cg04018622	8.258248	2.390498	28.52906	0.000844
cg24563570	8.240195	1.825662	37.19244	0.006092
cg22203219	8.220588	2.144265	31.51572	0.002123
cg15683295	8.214596	1.981114	34.06143	0.003707
cg17861230	8.199871	2.491865	26.98296	0.000535
cg05945059	8.193576	2.280679	29.43627	0.001266
cg23989963	8.159832	2.108104	31.58423	0.002366
cg27595860	8.139619	2.082611	31.81267	0.002571
cg15490703	8.125477	1.679583	39.30939	0.009197
cg26822097	8.121243	1.92518	34.25892	0.004347
cg12355110	8.096817	1.834677	35.73297	0.00576
cg09874822	8.083738	2.44908	26.6822	0.000603
cg10647513	8.082644	1.817735	35.93986	0.006053
cg02318926	8.076371	2.205374	29.57673	0.00161
cg07552803	8.055084	1.86337	34.82099	0.005218
cg20300343	8.044589	2.126598	30.43143	0.00213
cg00413617	8.040267	1.851527	34.91491	0.0054
cg18724565	8.026494	1.342478	47.98931	0.022444
cg14810013	8.020703	1.656091	38.84549	0.00969
cg14473102	8.000705	2.1398	29.91461	0.001998
cg19741167	7.992727	2.642107	24.17907	0.000233
cg08313939	7.986607	2.214722	28.80086	0.001498
cg20930366	7.978396	1.734021	36.70936	0.007658
cg04415176	7.970297	1.960563	32.40172	0.003722
cg18764577	7.968925	2.280588	27.84535	0.001148
cg10722226	7.956372	2.07414	30.52053	0.002498
cg23839136	7.942481	2.0254	31.14595	0.002956
cg05477514	7.921811	1.785789	35.14138	0.006472
cg24136205	7.920746	1.481571	42.34574	0.015539
cg19754554	7.893138	2.070717	30.08699	0.002477
cg11390378	7.883891	2.164719	28.71308	0.001742
cg08448701	7.876971	2.433465	25.49725	0.000573
cg13304274	7.874899	2.004892	30.93135	0.003112
cg26013553	7.860955	2.326093	26.56584	0.000904
cg03972398	7.849328	1.827131	33.7206	0.005599
cg14720763	7.849112	1.871577	32.91799	0.004849
cg14359292	7.843411	2.493291	24.67385	0.000428
cg05900347	7.831145	1.379801	44.44613	0.020158
cg05992357	7.819024	1.66975	36.61455	0.009033
cg13794993	7.811256	1.536989	39.69821	0.013206
cg13822303	7.806473	1.856876	32.81911	0.005037
cg03109827	7.804735	2.360562	25.80482	0.000758
cg20803857	7.793998	2.702639	22.4767	0.000145
cg08005774	7.789168	2.039965	29.74126	0.002674
cg17078686	7.788904	2.2389	27.0968	0.001251
cg05678749	7.774971	1.914274	31.57865	0.004131
cg19258062	7.753609	1.947964	30.86219	0.00366

cg27062795	7.740554	1.966443	30.46933	0.00342
cg23035602	7.726607	1.646571	36.25744	0.009536
cg05965288	7.725404	2.030597	29.3913	0.002709
cg12756396	7.724683	1.75768	33.94857	0.006797
cg15475851	7.721551	1.647672	36.18581	0.009498
cg19672997	7.709165	2.097582	28.33321	0.002102
cg14658067	7.705576	2.272873	26.12373	0.001045
cg05433039	7.691018	1.463046	40.43056	0.01598
cg13796823	7.685307	2.186229	27.01636	0.001476
cg02332525	7.68241	1.693002	34.86082	0.008235
cg19267861	7.661827	2.114819	27.75821	0.001933
cg13324546	7.65452	1.965681	29.80731	0.003343
cg26813646	7.597171	1.711579	33.72149	0.00766
cg03671597	7.58686	2.407553	23.90828	0.00054
cg02885007	7.586245	1.600898	35.94925	0.010687
cg15874092	7.562647	1.942231	29.44739	0.003533
cg07268058	7.559863	2.100589	27.20738	0.001962
cg22562942	7.549597	1.745907	32.64574	0.006812
cg07317062	7.512354	2.359379	23.91962	0.000643
cg12560987	7.510809	2.094169	26.93777	0.001973
cg07549278	7.500403	2.21133	25.43991	0.001223
cg12520549	7.492728	1.522957	36.86313	0.013233
cg04971534	7.465959	2.353523	23.68388	0.000642
cg19707040	7.463987	1.857462	29.99313	0.004618
cg25818402	7.453727	1.206149	46.06233	0.030644
cg16913789	7.447492	1.979345	28.02196	0.00298
cg12380743	7.440799	2.430886	22.77585	0.000438
cg20448053	7.436323	1.386633	39.88	0.01921
cg22989103	7.421068	2.078889	26.49119	0.002021
cg04745161	7.419085	1.609546	34.19773	0.010157
cg12212311	7.403288	2.558481	21.42235	0.000222
cg15491461	7.379616	1.647359	33.05821	0.008991
cg00939495	7.3557	2.190286	24.70286	0.001245
cg05376374	7.353642	2.299754	23.51384	0.000768
cg19717586	7.349344	2.420708	22.31284	0.000431
cg02222728	7.346407	1.507699	35.79607	0.013582
cg10298052	7.34359	2.375297	22.70382	0.000536
cg12668482	7.336339	2.100313	25.62565	0.001791
cg10384919	7.333191	1.810945	29.69482	0.005235
cg19786627	7.322349	1.792593	29.91019	0.005557
cg08235864	7.321722	2.418919	22.16181	0.000426
cg15060599	7.318022	2.213427	24.19481	0.001105
cg09155219	7.293331	1.38606	38.3769	0.019011
cg08159291	7.285428	1.899977	27.93585	0.00378
cg10420161	7.285283	1.548411	34.2773	0.01196
cg10836101	7.246827	1.663422	31.57137	0.008348
cg08013557	7.245466	1.983502	26.46671	0.002735
cg18206027	7.245107	2.178065	24.1001	0.001241
cg20635409	7.232056	1.775011	29.46609	0.00577
cg07103493	7.229379	1.967085	26.56923	0.002895
cg05151395	7.224457	1.285593	40.59822	0.024756
cg25024074	7.223443	2.205141	23.66204	0.00109
cg16880856	7.222272	1.046018	49.86644	0.044899
cg11651237	7.213338	2.000458	26.01016	0.002531

cg12527175	7.209505	1.619721	32.09006	0.009515
cg00182639	7.206044	1.103412	47.06044	0.039137
cg10997479	7.17081	1.610397	31.93032	0.009731
cg05143633	7.163093	1.831407	28.01666	0.004662
cg24721899	7.14139	1.767007	28.86205	0.0058
cg14015441	7.138289	1.77729	28.67016	0.005595
cg15916399	7.126185	2.266334	22.40735	0.00078
cg16762735	7.12517	1.466279	34.62373	0.014914
cg23217126	7.124758	2.082989	24.36988	0.001751
cg17007640	7.122075	1.83215	27.68548	0.004596
cg08305436	7.111586	1.765209	28.65081	0.005793
cg18343437	7.09329	1.928521	26.08982	0.003195
cg03712816	7.08506	1.885164	26.62797	0.003749
cg25486757	7.081428	2.03639	24.62525	0.002081
cg11708136	7.079917	1.49865	33.44693	0.013487
cg07244354	7.077819	1.510064	33.17445	0.013033
cg03570636	7.069585	1.857054	26.91307	0.004137
cg00816224	7.058795	1.68509	29.5691	0.007496
cg07203423	7.050264	1.717074	28.94821	0.006725
cg12591770	7.040325	1.734652	28.57413	0.006322
cg21573200	7.03033	2.026054	24.39498	0.002124
cg02657836	7.005865	1.633105	30.05449	0.00879
cg00333226	6.995383	2.044097	23.93985	0.001942
cg05697849	6.995377	2.221552	22.02753	0.000888
cg08141424	6.987305	2.019551	24.17489	0.002142
cg14530304	6.982243	1.734846	28.10146	0.00623
cg18023096	6.981107	1.665303	29.26546	0.007874
cg02578368	6.976461	2.639595	18.43882	8.95E-05
cg15032142	6.968835	1.64315	29.55583	0.008448
cg20585869	6.946644	1.648236	29.27727	0.008271
cg09451215	6.94302	1.451763	33.20482	0.015231
cg12595013	6.937402	1.723479	27.92464	0.006409
cg16418810	6.911356	2.326456	20.53202	0.000502
cg06636427	6.910598	1.801249	26.51293	0.004836
cg03881775	6.906787	1.851486	25.76509	0.004015
cg19677607	6.904361	1.717029	27.76319	0.006501
cg11410718	6.87621	2.109045	22.41881	0.001386
cg05937737	6.842823	2.011544	23.27775	0.002078
cg14209299	6.836098	1.988671	23.49923	0.002279
cg10056132	6.833863	1.949569	23.95488	0.002672
cg20146541	6.828546	1.75674	26.54293	0.005547
cg18556676	6.827136	1.99829	23.32483	0.002181
cg00127167	6.82622	1.788545	26.05318	0.004942
cg15431544	6.82194	1.694615	27.46279	0.006887
cg16651126	6.8216	1.511457	30.78765	0.012518
cg05756220	6.810231	1.744386	26.58772	0.005769
cg14384532	6.810147	1.843232	25.1613	0.004014
cg24089600	6.792563	2.020105	22.83986	0.001959
cg13891978	6.785197	2.005986	22.95076	0.002073
cg25684999	6.776437	1.680781	27.32069	0.007146
cg16764637	6.766969	1.750403	26.16076	0.005581
cg23929344	6.760715	2.141238	21.34618	0.001122
cg23883696	6.758934	1.965769	23.23935	0.002424
cg12483476	6.747952	1.964514	23.17868	0.002426

cg25136495	6.74742	2.356457	19.32039	0.000375
cg02509825	6.739433	1.393413	32.59619	0.017669
cg11530960	6.7384	1.538945	29.50465	0.011337
cg05313771	6.730896	1.701784	26.62204	0.006571
cg20405017	6.717252	1.682321	26.82097	0.007011
cg17070988	6.703126	1.630755	27.55282	0.008338
cg14492800	6.692857	2.148679	20.84738	0.00104
cg21077559	6.682001	1.94973	22.90017	0.002508
cg16494192	6.681429	1.62882	27.40726	0.008355
cg03609960	6.671302	2.067045	21.53135	0.0015
cg17078116	6.666058	1.841967	24.12439	0.003843
cg22476848	6.661378	1.411067	31.44708	0.016628
cg00986824	6.656285	1.776708	24.9372	0.00491
cg26521404	6.655506	1.92451	23.01664	0.002753
cg00070680	6.655177	1.998562	22.16162	0.002014
cg16619425	6.654398	1.975727	22.41252	0.002221
cg04324666	6.638194	1.580551	27.8799	0.009733
cg27434509	6.636164	1.856533	23.72092	0.003592
cg18044383	6.631771	1.70549	25.78754	0.006325
cg20270188	6.63036	1.600291	27.47105	0.0091
cg07960450	6.602834	1.881776	23.16823	0.003208
cg09371439	6.598252	1.119159	38.90148	0.03713
cg25901381	6.597014	1.822061	23.88537	0.004054
cg19828791	6.577902	1.251277	34.57972	0.026101
cg03209854	6.567192	1.77468	24.30186	0.004814
cg10739556	6.558235	2.408502	17.85776	0.000233
cg00688962	6.554938	1.457077	29.48863	0.014262
cg15191648	6.543098	1.668246	25.66296	0.007062
cg01283246	6.542491	2.116533	20.22373	0.001106
cg07015911	6.540873	1.909338	22.40725	0.002795
cg03532926	6.536444	2.174533	19.64794	0.000828
cg01856162	6.533433	1.949306	21.89792	0.002353
cg24000873	6.531052	1.972972	21.61948	0.002122
cg22491141	6.528825	1.414714	30.13017	0.016191
cg18952796	6.524249	2.172366	19.59422	0.00083
cg11148130	6.520119	2.057417	20.66278	0.001443
cg06724588	6.504344	1.786363	23.68304	0.004513
cg08146323	6.502171	1.556448	27.16328	0.010275
cg01305913	6.489782	1.511317	27.86791	0.011889
cg25602684	6.487701	1.97491	21.3125	0.00206
cg24129390	6.486106	2.056591	20.45597	0.001421
cg25903072	6.485031	1.034122	40.66797	0.045958
cg26492368	6.480499	1.96289	21.39542	0.002165
cg05749717	6.479488	1.759084	23.86683	0.00497
cg10767141	6.477035	1.900149	22.07826	0.002827
cg09549813	6.469791	2.102124	19.91233	0.001133
cg20289688	6.451706	1.84082	22.61194	0.003573
cg22674699	6.442786	2.059604	20.15412	0.001366
cg01429449	6.441563	1.513044	27.42401	0.011727
cg13567542	6.439078	1.740462	23.82226	0.005268
cg23130131	6.437161	1.510944	27.4246	0.011799
cg11097541	6.434453	1.477459	28.02257	0.013142
cg21100077	6.429663	1.800456	22.96116	0.004165
cg26257822	6.42782	1.485033	27.82219	0.012813

cg10320659	6.418912	2.014033	20.45767	0.001667
cg19594305	6.415953	1.84332	22.33169	0.003489
cg22686881	6.41477	1.880774	21.8789	0.002987
cg10806639	6.413175	2.299525	17.88579	0.000383
cg02249074	6.390451	1.557725	26.21636	0.010013
cg13755795	6.387228	1.884864	21.64436	0.002902
cg15149095	6.359609	1.75179	23.0876	0.00492
cg06786064	6.357206	1.703855	23.71919	0.005902
cg26789779	6.356079	2.043236	19.77243	0.001403
cg14473924	6.353399	2.154806	18.73285	0.000804
cg04613834	6.349548	1.452167	27.76316	0.014066
cg01210554	6.344458	1.923228	20.92947	0.002414
cg06980387	6.334618	1.488163	26.96438	0.012494
cg02919615	6.333285	1.680148	23.8732	0.006403
cg04534765	6.331932	2.096729	19.12186	0.001064
cg15965134	6.331868	1.361157	29.45477	0.018619
cg16317181	6.330959	1.688091	23.74341	0.006213
cg18181607	6.330613	1.553446	25.79856	0.01004
cg08566455	6.32746	1.891443	21.16731	0.00275
cg16939364	6.327456	1.576992	25.38801	0.009253
cg19119032	6.325596	1.54299	25.93223	0.010393
cg07405021	6.315796	1.269823	31.41325	0.024334
cg14994060	6.314142	1.543283	25.83349	0.010359
cg14035771	6.311226	1.90195	20.94249	0.002608
cg12325536	6.301389	1.564416	25.38169	0.009612
cg23254031	6.295495	1.424001	27.83232	0.015264
cg16151151	6.278115	1.42889	27.58415	0.014993
cg01886514	6.275324	1.650567	23.85828	0.00703
cg09086835	6.27494	1.602106	24.57695	0.008374
cg00854817	6.253999	1.646981	23.748	0.007084
cg27391267	6.246579	1.341825	29.07961	0.019561
cg12904880	6.244061	1.89349	20.5907	0.002624
cg01956420	6.238969	1.960469	19.8548	0.001937
cg14047094	6.219609	1.495435	25.86775	0.011959
cg20634442	6.219461	1.923585	20.10916	0.002269
cg27416372	6.218657	1.605927	24.0806	0.008151
cg19237294	6.209857	1.747867	22.0625	0.004754
cg03634479	6.189521	1.268757	30.19505	0.024174
cg24880701	6.187931	1.472242	26.00829	0.012848
cg24642065	6.184048	1.836755	20.82066	0.003265
cg03181248	6.181259	1.862609	20.51314	0.002918
cg05050657	6.175855	2.312897	16.49065	0.00028
cg24621437	6.172459	2.01422	18.91514	0.001445
cg23331421	6.171442	1.784561	21.34233	0.004042
cg01577751	6.168895	1.641326	23.18569	0.007071
cg24319902	6.166925	1.625765	23.39266	0.007486
cg20311863	6.166433	1.931907	19.68257	0.002126
cg11750165	6.163425	1.337868	28.39428	0.019625
cg15237923	6.158319	1.672313	22.67811	0.006275
cg05506365	6.141723	1.736786	21.71871	0.004854
cg14005246	6.139633	1.616568	23.31797	0.00769
cg04950301	6.134635	1.375654	27.357	0.017403
cg01783070	6.128319	2.131799	17.61718	0.000766
cg06717850	6.126692	1.598069	23.48857	0.008201

cg04669574	6.119737	2.114666	17.71021	0.000834
cg19595505	6.105331	1.77128	21.04415	0.004164
cg25720804	6.101261	1.898219	19.61069	0.002399
cg25359908	6.099053	1.298927	28.63783	0.02194
cg01281157	6.096894	1.959853	18.96679	0.001796
cg01574663	6.09185	1.331386	27.87369	0.019867
cg27490380	6.061167	1.477351	24.86731	0.012357
cg22333960	6.05384	1.912465	19.16321	0.002193
cg00332021	6.053201	1.069026	34.27534	0.041809
cg07440398	6.048425	1.46704	24.9369	0.012766
cg22125805	6.043632	1.82386	20.02648	0.003249
cg13954457	6.027235	2.134413	17.01993	0.000695
cg23683254	6.026783	1.774404	20.47004	0.003987
cg21231400	6.02296	1.721186	21.0762	0.00496
cg10502121	6.015885	1.241873	29.14216	0.02581
cg14459130	6.012845	1.665542	21.70724	0.006166
cg02730156	6.011587	1.537666	23.50262	0.009923
cg16783744	6.007449	1.449487	24.89808	0.013448
cg04227922	6.003108	1.756638	20.51493	0.004256
cg26014391	5.997508	1.94453	18.49809	0.001826
cg15007156	5.992728	1.632786	21.99479	0.006955
cg00505045	5.99134	1.363922	26.31834	0.01774
cg06410537	5.989094	1.30041	27.58303	0.021615
cg07072722	5.98108	1.497401	23.89027	0.011362
cg15565065	5.980028	2.18627	16.35696	0.000495
cg05336395	5.97886	1.234011	28.96795	0.026342
cg03758150	5.978322	1.847345	19.34687	0.002843
cg18621091	5.977999	1.917936	18.63278	0.002051
cg01366419	5.9667	1.555898	22.88164	0.0092
cg10978753	5.965378	1.707938	20.83549	0.005129
cg01874697	5.964076	1.346004	26.42652	0.018713
cg22095604	5.963974	2.067772	17.2016	0.000953
cg13473356	5.957617	1.682507	21.09542	0.005667
cg12918457	5.956723	1.82599	19.43196	0.003096
cg24328125	5.94686	1.455814	24.29235	0.013027
cg06425919	5.941675	1.58294	22.30249	0.008278
cg01610605	5.936948	1.587485	22.20327	0.008129
cg14098847	5.923067	1.933681	18.14297	0.001842
cg02914422	5.91565	1.948156	17.9631	0.001708
cg12432010	5.892992	1.618911	21.45106	0.007129
cg26055770	5.892556	1.934587	17.94813	0.001801
cg09123431	5.89162	1.33473	26.00616	0.019227
cg15310492	5.878424	1.661088	20.80315	0.006015
cg01866606	5.873429	1.403787	24.57436	0.015333
cg20896728	5.859968	1.061856	32.33886	0.042475
cg20168230	5.856084	1.717089	19.97201	0.004748
cg12610471	5.8527	1.649741	20.76333	0.006241
cg22546168	5.846625	1.625053	21.03502	0.006867
cg14712186	5.846565	1.712406	19.96158	0.004825
cg06550340	5.846293	1.513721	22.57955	0.010428
cg06256858	5.842607	1.567378	21.77908	0.008554
cg10807260	5.842077	1.148711	29.71145	0.033417
cg01486814	5.835517	1.340406	25.40518	0.018757
cg24127719	5.831624	1.876202	18.12589	0.002308

cg03171770	5.822307	1.757613	19.28711	0.003941
cg05663573	5.814293	2.074024	16.29972	0.000817
cg11739758	5.813757	1.610266	20.99017	0.007204
cg04020079	5.810838	1.764877	19.13212	0.0038
cg20892990	5.80372	1.557695	21.62373	0.008783
cg18752880	5.801562	1.149648	29.27689	0.033269
cg19030607	5.795138	1.109862	30.25928	0.037199
cg08347047	5.791368	1.639809	20.45357	0.006368
cg05581451	5.787188	1.539495	21.75489	0.009361
cg16650901	5.787177	1.639223	20.43128	0.006374
cg23842255	5.779722	1.307954	25.54003	0.020663
cg03169018	5.770678	1.471335	22.633	0.011944
cg10127275	5.768152	1.642808	20.25286	0.006245
cg14451382	5.754591	1.424053	23.25427	0.014045
cg12940822	5.753325	2.0577	16.08629	0.000852
cg15871127	5.751844	1.317525	25.11049	0.019982
cg13912117	5.749628	2.143656	15.42142	0.000511
cg13352750	5.746333	1.642907	20.09873	0.006198
cg04670857	5.743389	1.759049	18.75246	0.003786
cg11667258	5.742837	1.596704	20.65516	0.00744
cg00836482	5.738503	2.047297	16.08483	0.000892
cg14691529	5.737359	1.804745	18.2393	0.003071
cg24645221	5.735837	1.267461	25.95726	0.02335
cg15402529	5.735282	1.715849	19.17037	0.004556
cg26335633	5.73178	1.379558	23.81436	0.016272
cg08475379	5.730143	1.223808	26.82981	0.026665
cg23623622	5.72921	1.606895	20.42688	0.007119
cg16848624	5.724285	1.68975	19.39189	0.005069
cg26673012	5.721624	1.425514	22.96504	0.013895
cg05107500	5.719681	1.383885	23.6398	0.016009
cg07160746	5.714611	1.629466	20.0414	0.006477
cg09522056	5.705293	1.68533	19.31395	0.005128
cg06674731	5.703074	1.635886	19.88223	0.006287
cg27190946	5.702414	1.53877	21.13215	0.009192
cg04974913	5.702272	1.079327	30.12611	0.040379
cg19430967	5.698553	1.540583	21.07871	0.00912
cg10689404	5.692729	1.655816	19.57172	0.005774
cg21475402	5.686965	1.610366	20.08336	0.006932
cg17398677	5.669486	1.227053	26.19534	0.026283
cg03156893	5.66056	1.597924	20.05223	0.007225
cg22418909	5.659949	1.514356	21.15421	0.009969
cg19628148	5.650887	1.494478	21.36701	0.01071
cg24199834	5.648579	1.335929	23.88333	0.018588
cg11301556	5.645375	1.281885	24.86202	0.022122
cg21546671	5.643562	2.256095	14.11722	0.000216
cg11464895	5.642251	1.488744	21.3838	0.010917
cg04344565	5.636984	1.718279	18.49269	0.004331
cg22660147	5.635352	1.832571	17.32931	0.002555
cg01228134	5.624983	1.492676	21.19713	0.010718
cg02438781	5.624415	1.4646	21.59911	0.011877
cg18347642	5.621146	1.289342	24.50651	0.021548
cg22873539	5.618556	1.308256	24.12997	0.02027
cg04931256	5.610331	1.75417	17.94342	0.003645
cg13610307	5.609869	1.854239	16.97226	0.002264

cg07442479	5.605698	1.517692	20.70502	0.009716
cg06629130	5.596744	1.795288	17.44764	0.002991
cg17294725	5.596063	1.76162	17.77677	0.003499
cg08251037	5.593738	1.278401	24.47581	0.022248
cg09302895	5.592992	1.590399	19.66901	0.007294
cg08226590	5.592749	1.235537	25.316	0.02545
cg11308643	5.590333	1.910385	16.35891	0.001681
cg17236169	5.584283	1.469433	21.22195	0.01157
cg18948722	5.576992	1.625527	19.13401	0.006288
cg21000072	5.575854	1.358556	22.88471	0.017065
cg13929328	5.572147	1.44949	21.42052	0.01241
cg21647227	5.565806	1.724773	17.96074	0.00408
cg03111498	5.565718	1.595354	19.41715	0.007089
cg06472476	5.564009	1.103881	28.04487	0.037551
cg27034819	5.558494	1.475003	20.94698	0.011272
cg20792062	5.557932	1.374705	22.47072	0.016108
cg04704053	5.557489	1.527605	20.21837	0.009241
cg15889913	5.549573	1.623819	18.96625	0.006274
cg12510458	5.549313	1.373975	22.41299	0.016127
cg15841063	5.544641	1.53699	20.00211	0.008882
cg15448975	5.539948	1.357459	22.60917	0.017038
cg17834752	5.539206	1.466751	20.91888	0.011571
cg23201513	5.537041	1.362069	22.509	0.016766
cg10824063	5.533665	1.621667	18.8827	0.006296
cg12160317	5.530538	1.375211	22.24157	0.016011
cg00571033	5.529883	1.572693	19.44411	0.007681
cg14654886	5.529828	1.915841	15.96114	0.001566
cg08272731	5.526357	1.772719	17.22813	0.00321
cg02582387	5.52409	1.748414	17.45328	0.003593
cg11727383	5.520569	1.612957	18.89491	0.006499
cg04598121	5.514759	1.306907	23.27065	0.020107
cg13678973	5.513018	1.835783	16.55607	0.002345
cg06218338	5.511447	1.377909	22.04503	0.015813
cg27659903	5.509497	1.725134	17.59548	0.003972
cg25191628	5.50775	1.515951	20.01075	0.009541
cg06668555	5.507413	1.479612	20.4997	0.010953
cg26844246	5.507351	1.205121	25.16835	0.027762
cg19047707	5.50376	1.711549	17.69822	0.004213
cg16599703	5.502008	1.556048	19.45447	0.008142
cg08572611	5.492309	1.746844	17.26854	0.003564
cg26836233	5.490607	1.600735	18.83308	0.006768
cg19414741	5.487573	1.433391	21.00854	0.012932
cg13063344	5.483067	1.874982	16.0343	0.001883
cg22335876	5.482618	1.538111	19.54287	0.008693
cg10552126	5.479159	1.259758	23.83091	0.023339
cg00091285	5.467761	1.485982	20.11896	0.010593
cg15762032	5.464811	1.645611	18.14776	0.005548
cg18525486	5.463598	1.660456	17.97754	0.005199
cg03964958	5.463492	1.382987	21.58353	0.015412
cg03711485	5.462618	1.792621	16.64613	0.00282
cg22813950	5.462309	1.354791	22.0232	0.016995
cg03757784	5.456634	1.660412	17.93221	0.005185
cg20051292	5.454283	1.583985	18.78125	0.007166
cg03411507	5.453489	1.054372	28.20689	0.043062

cg21697851	5.441301	1.827389	16.20222	0.002343
cg11342468	5.439627	1.797712	16.45956	0.002716
cg05470523	5.436761	1.464171	20.18779	0.011419
cg14568830	5.432712	1.28198	23.02247	0.021612
cg01532168	5.425256	1.682855	17.49016	0.004634
cg06598836	5.421058	1.989365	14.77249	0.000951
cg17051321	5.420362	1.169051	25.13176	0.030809
cg12377139	5.416422	1.588776	18.46555	0.006938
cg18443359	5.413785	1.385162	21.15931	0.015164
cg16768018	5.413722	1.609498	18.20965	0.006354
cg18026588	5.413718	1.705583	17.18376	0.004158
cg17741689	5.412037	1.556582	18.81696	0.007909
cg06936155	5.409524	1.37155	21.33568	0.015899
cg06179765	5.401254	1.428757	20.41883	0.012925
cg27403265	5.399596	1.701141	17.13888	0.004216
cg01392544	5.387615	1.963422	14.78357	0.001075
cg18092028	5.38743	1.686256	17.21234	0.004488
cg24431667	5.374572	1.4603	19.78089	0.011423
cg22418829	5.372634	1.082755	26.65904	0.039662
cg27328797	5.367221	2.175976	13.23868	0.000265
cg07060551	5.362797	1.634736	17.59281	0.005592
cg08152546	5.361896	1.378288	20.85916	0.015399
cg00446413	5.361208	1.532824	18.75137	0.008575
cg13365752	5.357224	1.513396	18.96387	0.009257
cg11969556	5.350823	1.109579	25.80376	0.036663
cg12296772	5.349023	2.164324	13.21985	0.000281
cg03502002	5.348052	2.110704	13.55077	0.000408
cg21091227	5.345854	1.41591	20.1836	0.013398
cg01997272	5.326753	1.640393	17.29726	0.005376
cg09194159	5.325995	1.808773	15.68258	0.002401
cg03524083	5.322968	1.501914	18.86525	0.009597
cg00922781	5.31641	1.371946	20.60156	0.015626
cg26128092	5.313479	1.615374	17.47773	0.005971
cg11874762	5.307375	1.663258	16.93557	0.004812
cg05446629	5.299696	1.727197	16.26148	0.003553
cg20717123	5.297069	1.622886	17.28953	0.005741
cg10996058	5.293957	1.294056	21.65747	0.020417
cg24693551	5.287395	1.801019	15.52263	0.00244
cg16428251	5.287218	1.668598	16.7534	0.004654
cg17098147	5.286232	1.554339	17.97822	0.007672
cg08720517	5.282948	1.483851	18.80885	0.010197
cg08582701	5.275388	1.339615	20.77441	0.017404
cg17403609	5.274807	1.553746	17.90742	0.007662
cg26053480	5.273713	1.953587	14.2364	0.001032
cg21884231	5.272375	1.80009	15.44253	0.002429
cg20302133	5.271847	1.79254	15.50446	0.002525
cg10527010	5.269554	1.460427	19.01376	0.011135
cg11412853	5.26865	1.6869	16.45543	0.004239
cg09617993	5.268099	1.359805	20.40945	0.016184
cg19806642	5.263782	1.53411	18.0609	0.008284
cg03700449	5.263435	1.727063	16.04096	0.003489
cg21802055	5.262184	1.712888	16.16602	0.003734
cg15600488	5.261785	1.606267	17.23647	0.006092
cg21012296	5.258662	1.754484	15.76163	0.003039

cg19416570	5.253833	1.373082	20.10278	0.015391
cg20462512	5.253465	1.459195	18.91378	0.011144
cg15731655	5.251412	1.737691	15.8701	0.00329
cg08091192	5.251175	1.653614	16.6755	0.004907
cg21701379	5.246818	1.408313	19.54758	0.013504
cg20277905	5.239845	1.306165	21.02029	0.01945
cg18578405	5.237866	1.577099	17.39602	0.006854
cg08278741	5.235404	1.771129	15.4757	0.002757
cg02109405	5.234367	1.503233	18.22645	0.009314
cg00910518	5.229997	1.728035	15.82888	0.003411
cg10241484	5.229518	1.250573	21.86827	0.023434
cg07770968	5.22689	1.847172	14.79038	0.001832
cg09660365	5.225318	1.234201	22.12277	0.02472
cg11199046	5.222057	1.028133	26.52369	0.046215
cg18380175	5.22138	1.399565	19.47949	0.013879
cg22643811	5.220163	1.541831	17.67386	0.007912
cg25888561	5.220021	1.528503	17.827	0.008363
cg10023272	5.218182	1.19737	22.74103	0.027821
cg20892260	5.216724	1.627389	16.72263	0.005447
cg14486338	5.216623	1.590241	17.11261	0.006424
cg24464397	5.216388	1.304095	20.86558	0.019525
cg09972259	5.215026	1.4603	18.6239	0.010991
cg14345497	5.212659	1.568995	17.31797	0.007033
cg08263708	5.207869	1.365607	19.86069	0.015683
cg25865467	5.205346	1.327531	20.41055	0.017964
cg14785479	5.203919	1.086278	24.92988	0.039065
cg20599066	5.203703	1.139383	23.76596	0.033309
cg23208513	5.199017	1.591562	16.98318	0.006345
cg24019371	5.198291	1.582312	17.07769	0.006605
cg09968620	5.197325	1.554186	17.38027	0.007453
cg25185173	5.197264	1.866044	14.4753	0.001613
cg02172150	5.196285	1.699051	15.89203	0.003861
cg07281879	5.191893	1.16742	23.09002	0.03052
cg23003783	5.19122	1.524504	17.67707	0.008427
cg15950068	5.191075	1.78527	15.09422	0.002493
cg14699728	5.185188	1.891148	14.21685	0.001383
cg10904109	5.178504	1.052743	25.47336	0.043053
cg17361203	5.174714	1.517428	17.64674	0.008634
cg02457680	5.174391	1.236178	21.65895	0.024435
cg00152008	5.174256	1.674221	15.99128	0.004302
cg16435571	5.173547	1.531271	17.47933	0.008147
cg24811864	5.17299	1.506881	17.75842	0.009013
cg02583418	5.170573	1.274325	20.97959	0.021493
cg10097295	5.165188	1.04083	25.63258	0.044545
cg20076842	5.160299	1.316084	20.23326	0.018575
cg11747183	5.158331	1.616171	16.46384	0.005594
cg10176110	5.154932	1.593431	16.6768	0.006187
cg26990587	5.154357	1.586603	16.74483	0.006376
cg12552771	5.153951	1.80439	14.72143	0.002197
cg17777676	5.153707	1.59469	16.65571	0.006149
cg09426834	5.14998	1.818631	14.58366	0.002028
cg13566648	5.149625	1.364855	19.42963	0.01556
cg21639114	5.146208	1.384997	19.12167	0.014433
cg16823083	5.142169	1.600432	16.52173	0.005966

cg12306195	5.139301	1.31563	20.07586	0.018546
cg22605415	5.135864	1.604184	16.44269	0.005851
cg18988498	5.134024	1.405538	18.7531	0.013324
cg09683350	5.132592	1.454566	18.11089	0.011009
cg16001495	5.132454	1.608476	16.37704	0.005731
cg26675654	5.132132	1.607868	16.38119	0.005746
cg17864046	5.131865	1.477047	17.83019	0.010059
cg25188395	5.131344	1.768895	14.88539	0.002616
cg15556502	5.129885	1.897033	13.87204	0.001275
cg17486097	5.128201	1.534822	17.13453	0.007907
cg15837212	5.124993	1.396365	18.80995	0.013769
cg19304150	5.124154	1.671515	15.70847	0.004253
cg16086559	5.122194	1.801691	14.56236	0.002182
cg01243371	5.122117	1.907229	13.75613	0.001192
cg02621287	5.119013	1.91325	13.69622	0.001146
cg04694035	5.114345	1.272555	20.55433	0.021472
cg00497967	5.111241	1.395628	18.71902	0.013768
cg00792424	5.108945	1.776727	14.69068	0.002474
cg23054883	5.108456	1.383135	18.86751	0.014424
cg10591652	5.108064	1.330981	19.60382	0.017471
cg15386964	5.107657	2.211501	11.79658	0.000134
cg00043788	5.100447	1.550978	16.77301	0.007306
cg14565725	5.098341	1.680596	15.46659	0.004017
cg03893271	5.09429	1.509679	17.19027	0.008697
cg25203481	5.092946	1.077351	24.07581	0.039978
cg24740868	5.091452	1.357771	19.09223	0.0158
cg01638213	5.086617	1.784235	14.50126	0.002341
cg17911318	5.084339	1.741768	14.84153	0.002928
cg00027083	5.083951	1.608441	16.06932	0.005616
cg05212802	5.080806	1.504955	17.15307	0.008834
cg25733708	5.080695	1.47189	17.53763	0.010126
cg16902509	5.078421	1.395554	18.48037	0.013675
cg26115633	5.077586	1.852203	13.91958	0.001589
cg14230666	5.075982	1.756125	14.67185	0.002702
cg21431690	5.071858	1.036422	24.81976	0.045057
cg06825039	5.066726	1.322525	19.41114	0.01789
cg18326021	5.065762	1.551912	16.5357	0.007186
cg23665381	5.060235	1.464516	17.48426	0.010375
cg04316624	5.060157	1.200403	21.33049	0.027189
cg11439596	5.059288	1.671601	15.3125	0.004114
cg06495961	5.055013	1.742787	14.66223	0.00286
cg20055426	5.054636	1.368217	18.67346	0.015092
cg23618440	5.054554	1.474723	17.32429	0.009936
cg18115040	5.050913	1.39041	18.34834	0.013865
cg00589371	5.04684	1.630336	15.62292	0.004989
cg08165221	5.046489	1.294359	19.67541	0.019721
cg06066137	5.042309	1.163745	21.84747	0.030566
cg20981086	5.041477	1.972675	12.88428	0.000727
cg00534492	5.038311	1.312677	19.33803	0.018452
cg18716164	5.034502	1.50566	16.83396	0.008679
cg02658693	5.0345	1.331533	19.03535	0.017223
cg24472231	5.033668	1.48431	17.07043	0.009491
cg09311683	5.03156	1.805314	14.02338	0.002005
cg03867465	5.029198	1.625508	15.55995	0.005063

cg08528626	5.028488	1.742896	14.50786	0.002812
cg17555825	5.027627	1.424515	17.74431	0.012078
cg03131298	5.019476	1.124619	22.40327	0.034529
cg08857994	5.01924	1.071704	23.50721	0.040573
cg02456226	5.016967	1.713202	14.69176	0.003261
cg16907558	5.015558	1.656018	15.19054	0.004343
cg22191326	5.014017	1.581745	15.89407	0.006164
cg12744722	5.010488	1.443883	17.38714	0.011129
cg25993718	5.010411	1.382691	18.15606	0.014157
cg27630311	5.008304	1.274482	19.68103	0.021037
cg03383508	5.006723	1.500574	16.70513	0.00879
cg02757432	5.002849	1.147375	21.8137	0.032117
cg09614415	5.002189	1.337879	18.70266	0.016731
cg05832051	5.001107	1.145681	21.83075	0.032287
cg23305567	4.999517	1.269073	19.69561	0.021414
cg26720125	4.994819	1.536167	16.24057	0.007505
cg20266316	4.994163	1.556499	16.02421	0.006856
cg26504021	4.990515	1.681089	14.81495	0.003784
cg06482428	4.988774	1.428545	17.42183	0.01177
cg26503073	4.988139	1.310809	18.98181	0.018429
cg19079845	4.986809	1.625174	15.30191	0.004971
cg14944647	4.985897	1.791576	13.87559	0.002094
cg01715455	4.985593	1.676118	14.82958	0.00387
cg05956679	4.982557	1.21714	20.39689	0.025534
cg02523640	4.982535	1.651487	15.0323	0.004367
cg08089301	4.98227	1.603439	15.48111	0.0055
cg09388605	4.980102	1.432629	17.31182	0.011553
cg11097433	4.979838	1.853477	13.37961	0.001454
cg18279094	4.978619	1.625067	15.25269	0.004955
cg02792792	4.975106	1.558805	15.87863	0.006735
cg22167515	4.974996	1.747484	14.16355	0.00265
cg05732750	4.971221	1.315991	18.77903	0.018035
cg03193589	4.968824	1.719329	14.3598	0.003068
cg03044249	4.967704	1.861922	13.2541	0.001367
cg02279719	4.964823	1.616092	15.25251	0.005139
cg06675190	4.961361	1.396039	17.6321	0.013299
cg21926708	4.959811	1.313032	18.73505	0.018197
cg25306611	4.959585	1.435896	17.13041	0.01134
cg16419235	4.958842	1.271574	19.33833	0.021112
cg22634378	4.95693	1.475204	16.6561	0.009634
cg11626496	4.955683	1.422224	17.26788	0.011971
cg14383828	4.955238	1.087482	22.57911	0.038607
cg19407095	4.953535	1.013225	24.21724	0.048133
cg03323462	4.953251	1.685467	14.55662	0.003625
cg22685409	4.950974	1.331097	18.415	0.017
cg00831710	4.949897	1.027534	23.84494	0.046171
cg15554678	4.949727	1.120177	21.87135	0.034887
cg06228507	4.949553	1.330258	18.41603	0.017049
cg07033372	4.947722	1.333736	18.35442	0.016824
cg19326876	4.933152	1.500804	16.2153	0.008572
cg01567634	4.93236	1.342572	18.12057	0.016231
cg01452847	4.931083	1.783283	13.63529	0.002107
cg18365865	4.929849	1.43845	16.89556	0.011134
cg22605303	4.929372	1.28434	18.91921	0.020091

cg11229862	4.92898	1.283147	18.9338	0.020176
cg03816707	4.9246	1.167571	20.77105	0.029936
cg15829006	4.923091	1.222359	19.82792	0.024933
cg25234732	4.92011	1.672522	14.47364	0.003801
cg13702996	4.917846	1.210915	19.97268	0.025907
cg01423964	4.917571	1.566508	15.4372	0.006353
cg23291301	4.909149	1.406347	17.13641	0.01261
cg04549162	4.906935	1.36969	17.57917	0.01456
cg00619126	4.90556	1.512012	15.91556	0.008086
cg16334314	4.894988	1.687701	14.19737	0.003464
cg14008883	4.890565	1.125794	21.24513	0.034169
cg20673829	4.886393	1.569797	15.21014	0.006175
cg10373086	4.88639	1.021547	23.37319	0.04696
cg08676249	4.885882	1.567271	15.23148	0.006247
cg01574673	4.881466	1.431301	16.64829	0.011315
cg26809635	4.878871	1.978963	12.02821	0.000576
cg10671668	4.869705	1.468717	16.14608	0.00964
cg11554266	4.868549	1.218653	19.44997	0.025104
cg03909500	4.867804	1.114947	21.25259	0.035321
cg22931725	4.866391	1.094061	21.64573	0.037707
cg11763094	4.866109	1.500826	15.77733	0.008377
cg06873316	4.863383	1.477905	16.00407	0.009248
cg04198308	4.862682	1.66848	14.17198	0.003756
cg03794801	4.862637	1.3519	17.49037	0.015452
cg15086113	4.861214	1.467914	16.09863	0.009647
cg03442378	4.859255	1.534924	15.38341	0.007173
cg00814733	4.853955	1.739914	13.54141	0.002545
cg27050153	4.85261	1.255291	18.75886	0.022048
cg23397578	4.850684	1.022848	23.00355	0.046766
cg21779611	4.850579	1.867111	12.60135	0.001188
cg20695433	4.849366	1.398308	16.81772	0.012833
cg11835068	4.849061	1.36057	17.28202	0.014899
cg19224201	4.844501	1.602735	14.64321	0.005177
cg16795307	4.841194	1.638233	14.30636	0.004333
cg02825211	4.841191	1.557326	15.0496	0.006421
cg00467420	4.831312	1.590835	14.67254	0.005451
cg00030508	4.831039	1.211379	19.26643	0.025637
cg18097850	4.82812	1.115067	20.90525	0.035237
cg00046625	4.82793	1.379705	16.89412	0.013754
cg22130262	4.825896	1.389635	16.75928	0.013213
cg19651694	4.824918	1.367161	17.02787	0.014444
cg15484532	4.820633	1.30149	17.8553	0.018553
cg23758305	4.819866	1.592682	14.58616	0.005373
cg26682580	4.818875	1.513445	15.3435	0.007785
cg13139972	4.815646	1.619686	14.31786	0.004693
cg18454863	4.813265	1.089508	21.26421	0.038167
cg09022422	4.812059	1.397897	16.56482	0.012736
cg14794043	4.810902	1.300094	17.80239	0.018619
cg06966113	4.809365	1.256088	18.4143	0.021859
cg15461335	4.807626	1.386109	16.67493	0.013342
cg22868282	4.805229	1.790821	12.89366	0.001827
cg17466857	4.803323	1.803211	12.7949	0.001693
cg15617814	4.802032	1.824923	12.63588	0.00148
cg04958794	4.80134	1.364755	16.89158	0.014506

cg03175653	4.799235	1.575087	14.6231	0.005795
cg02864757	4.795093	1.487758	15.45475	0.008658
cg00157668	4.790878	1.408796	16.29229	0.012115
cg14323675	4.789762	1.299772	17.65064	0.018575
cg23990012	4.788295	1.429456	16.03951	0.011109
cg04842426	4.787671	1.496639	15.31551	0.0083
cg13909534	4.78751	1.314355	17.43841	0.017577
cg00336320	4.787308	1.262157	18.15806	0.021321
cg07489048	4.786863	1.253056	18.28654	0.02203
cg04778012	4.785569	1.473837	15.53881	0.009175
cg02237470	4.784258	1.604885	14.26216	0.004973
cg02347074	4.783713	1.390291	16.4598	0.013042
cg04217778	4.780677	1.784196	12.80962	0.001863
cg21995919	4.778473	1.698557	13.44306	0.003038
cg17252960	4.775944	1.655638	13.77695	0.003819
cg09619271	4.773415	1.795948	12.68717	0.001725
cg13939859	4.772263	1.4968	15.21546	0.008248
cg08813062	4.772203	1.328289	17.1453	0.016619
cg06652199	4.770333	1.572552	14.47079	0.005789
cg18417954	4.768777	1.296291	17.54331	0.018751
cg19142026	4.762846	1.978141	11.46769	0.000499
cg22757824	4.759775	1.716081	13.20186	0.002722
cg24575234	4.755596	1.544091	14.64661	0.006589
cg00017437	4.754422	1.401711	16.12638	0.012354
cg10177394	4.746567	1.137946	19.79873	0.032573
cg27423760	4.743199	1.495935	15.03939	0.008193
cg14562712	4.743117	1.969807	11.42099	0.000517
cg16234978	4.740663	1.856491	12.10557	0.00114
cg03696599	4.739954	1.218685	18.43557	0.024746
cg18297437	4.735104	1.419674	15.7932	0.011402
cg27237300	4.734983	1.495758	14.9891	0.008174
cg14552379	4.732367	1.343306	16.67178	0.01555
cg03307893	4.729673	1.434126	15.59822	0.010706
cg03700462	4.729539	1.148968	19.46838	0.031373
cg11793269	4.72717	1.436843	15.55224	0.010573
cg00933210	4.71961	1.065737	20.90077	0.040971
cg24496475	4.715031	1.016871	21.86266	0.047553
cg18734428	4.714234	1.678413	13.24108	0.003253
cg10286380	4.714221	1.240056	17.92168	0.022861
cg21816308	4.711792	1.227611	18.08471	0.023896
cg09230996	4.70647	1.55304	14.2629	0.006178
cg08938584	4.702213	1.069602	20.672	0.04046
cg04606210	4.700902	1.424068	15.51785	0.01108
cg13519035	4.700895	1.56475	14.12265	0.005821
cg02011374	4.698437	1.378462	16.01444	0.013399
cg13464448	4.696484	1.676232	13.15866	0.003254
cg10908460	4.696482	1.649872	13.36888	0.003755
cg09816507	4.696414	1.203312	18.32965	0.025991
cg14859460	4.694412	1.54913	14.22573	0.006262
cg26198463	4.691382	1.117258	19.69917	0.034736
cg22746058	4.686845	1.211335	18.13415	0.025241
cg23615741	4.686199	1.861305	11.79842	0.001043
cg20539752	4.684488	1.149489	19.0906	0.031215
cg22153181	4.682937	1.808834	12.12378	0.001467

cg02150988	4.68137	1.13848	19.24955	0.032375
cg16712637	4.680553	1.213031	18.0602	0.025073
cg02409815	4.679913	1.257178	17.42122	0.021378
cg09434193	4.677584	1.491851	14.66621	0.008144
cg03951603	4.676372	1.687878	12.95619	0.003009
cg18172877	4.675571	1.540908	14.18706	0.006461
cg26980244	4.674612	1.444168	15.1312	0.010075
cg07860213	4.674106	1.477007	14.79158	0.008703
cg20890210	4.668854	1.536877	14.18344	0.006568
cg05622465	4.663932	1.347732	16.13991	0.015053
cg12164282	4.661196	1.313652	16.53919	0.017211
cg19870512	4.66092	1.723866	12.60201	0.002421
cg27454136	4.657654	1.695895	12.79191	0.002839
cg27316886	4.653836	1.212658	17.86009	0.025028
cg09234616	4.653562	1.514234	14.30138	0.007269
cg08432727	4.651296	1.380695	15.66932	0.013119
cg03755123	4.651219	1.196695	18.07798	0.026473
cg19727439	4.649881	1.255344	17.22348	0.021428
cg25189564	4.64819	1.702178	12.69295	0.00272
cg02662277	4.647076	1.631689	13.23495	0.004017
cg20403938	4.638568	1.587426	13.55422	0.005037
cg19632594	4.638112	1.285286	16.7372	0.019115
cg06962177	4.637318	1.768477	12.16002	0.001814
cg00596508	4.634021	1.446249	14.84817	0.009851
cg21135135	4.633704	1.621427	13.24217	0.004209
cg03462380	4.632614	1.38861	15.45511	0.01263
cg16424078	4.63081	1.324858	16.18619	0.016371
cg03401738	4.630225	1.428472	15.00833	0.01064
cg00213479	4.626051	1.727754	12.38622	0.002302
cg26988423	4.62369	1.322092	16.17021	0.016527
cg06685968	4.619345	1.797834	11.86892	0.001482
cg21106486	4.616782	1.251397	17.03271	0.021638
cg02093732	4.616704	1.223703	17.41759	0.023948
cg07811198	4.614193	1.379134	15.43779	0.013077
cg18485844	4.614055	1.68465	12.63734	0.002934
cg24556441	4.613989	1.544603	13.78276	0.006169
cg06594404	4.613853	1.409309	15.10502	0.011505
cg19193956	4.613717	1.540513	13.81772	0.006294
cg24061141	4.61268	1.210945	17.57042	0.025061
cg11073773	4.61212	1.494318	14.23502	0.007849
cg17241776	4.61157	1.597509	13.31234	0.004713
cg07780543	4.611569	1.507303	14.10902	0.007381
cg25482900	4.611299	1.378297	15.42779	0.013113
cg00579520	4.60956	1.158969	18.33357	0.030052
cg01381846	4.607318	1.50569	14.09811	0.007425
cg05044185	4.606762	1.19168	17.80869	0.026818
cg19064258	4.605102	1.442994	14.69651	0.009899
cg16981024	4.60461	1.406222	15.07759	0.011627
cg14273116	4.60187	1.010372	20.95981	0.048461
cg22489321	4.599846	1.411756	14.98742	0.011336
cg19505136	4.599794	1.79699	11.77419	0.001462
cg10175795	4.597993	1.469894	14.38303	0.008743
cg18657094	4.593642	1.390625	15.17414	0.01239
cg18777119	4.587633	1.577951	13.33779	0.005148

cg12782992	4.584989	1.24801	16.84452	0.021809
cg13358636	4.577832	1.310309	15.99359	0.017153
cg12877723	4.576787	1.359002	15.4135	0.014084
cg19125370	4.569365	1.460127	14.2995	0.009047
cg25121513	4.566506	1.482657	14.0646	0.008141
cg02344833	4.562818	1.517643	13.71818	0.006877
cg21183256	4.562746	1.532322	13.58634	0.0064
cg01870995	4.56247	1.571001	13.25023	0.005265
cg27473895	4.558076	1.563314	13.28975	0.005464
cg21649258	4.557478	1.696267	12.2449	0.002631
cg17554604	4.556012	1.55935	13.31147	0.00557
cg09022943	4.555055	1.363529	15.21679	0.013746
cg15638709	4.55501	1.512884	13.71428	0.007014
cg15634398	4.553629	1.253017	16.54849	0.021304
cg04043571	4.552591	1.021217	20.29547	0.046868
cg03369269	4.549658	1.384923	14.94623	0.01254
cg03659519	4.549269	1.474028	14.04034	0.00842
cg09624466	4.549163	1.77452	11.66224	0.00161
cg02720618	4.548797	1.237118	16.72561	0.022592
cg08806408	4.547791	1.609814	12.84769	0.004256
cg21480165	4.545725	1.199784	17.22279	0.025883
cg26857670	4.545112	1.477562	13.98117	0.008268
cg04330449	4.544859	1.568745	13.16706	0.005277
cg08475953	4.539681	1.308443	15.75055	0.017148
cg03804213	4.536081	1.322719	15.55586	0.016182
cg14146100	4.535558	1.280157	16.06935	0.019148
cg16696270	4.532507	1.168063	17.58776	0.028924
cg12159189	4.530483	1.33784	15.3421	0.015197
cg18961681	4.529186	1.499052	13.68433	0.007416
cg09227138	4.526554	1.260749	16.252	0.0206
cg22717227	4.524926	1.133914	18.05689	0.032521
cg12040830	4.524829	1.698563	12.05376	0.00253
cg17694795	4.521237	1.400003	14.60111	0.011652
cg03365311	4.52033	1.386156	14.74104	0.012371
cg12718339	4.5185	1.116687	18.2834	0.034454
cg27501878	4.517441	1.790739	11.39601	0.001403
cg00983904	4.5172	1.777992	11.47648	0.001526
cg12776966	4.516179	1.394668	14.62417	0.011908
cg24224020	4.51393	1.432822	14.22058	0.010046
cg01200640	4.512492	1.478996	13.76785	0.008106
cg23720732	4.511591	1.746695	11.65312	0.001859
cg20631014	4.510945	1.32891	15.31227	0.015692
cg03091551	4.510422	1.402602	14.50441	0.011482
cg04606861	4.510003	1.15623	17.59177	0.030083
cg03599078	4.507863	1.237531	16.42046	0.022425
cg21858380	4.507601	1.474041	13.78419	0.008283
cg05771261	4.504397	1.086565	18.67315	0.038043
cg00803827	4.50349	1.369142	14.81323	0.013244
cg08620606	4.503171	1.080943	18.76006	0.038746
cg16415058	4.501301	1.332189	15.20934	0.015449
cg04894619	4.500149	1.269622	15.95068	0.019821
cg05206884	4.497476	1.40005	14.44755	0.011566
cg14866863	4.496121	1.505643	13.42623	0.007079
cg20611911	4.495279	1.5205	13.29006	0.006575

cg14752336	4.495126	1.158628	17.43973	0.029794
cg02370605	4.49171	1.420097	14.2071	0.01056
cg07557260	4.489292	1.27864	15.76186	0.019101
cg06159486	4.486745	1.284293	15.67469	0.018673
cg18335607	4.485099	1.118341	17.98745	0.034192
cg09251429	4.484447	1.54556	13.01163	0.005762
cg04738965	4.481631	1.413439	14.21003	0.010844
cg09887059	4.4813	1.527483	13.14715	0.006307
cg02721665	4.480749	1.294114	15.51417	0.017941
cg07871947	4.479658	1.115128	17.99554	0.034554
cg04138185	4.475523	1.656238	12.09385	0.003129
cg14001664	4.475519	1.005499	19.92073	0.049165
cg05736768	4.475479	1.408465	14.22109	0.011066
cg01054478	4.474692	1.270252	15.76292	0.019685
cg07035961	4.474663	1.240546	16.14016	0.022063
cg15044957	4.473588	1.563177	12.80277	0.005228
cg10723962	4.473496	1.198784	16.69372	0.02576
cg27032232	4.471597	1.653419	12.09323	0.003172
cg16021909	4.471411	1.343033	14.88683	0.014664
cg00699993	4.470387	1.576342	12.67768	0.004867
cg09430118	4.465502	1.139718	17.49618	0.03174
cg02739346	4.462249	1.25374	15.88182	0.020939
cg23156916	4.462235	1.379674	14.43206	0.012512
cg13272644	4.460884	1.19861	16.60214	0.025738
cg24842733	4.459074	1.354042	14.68443	0.013956
cg23771603	4.458298	1.703563	11.66756	0.002325
cg20977794	4.4578	1.29952	15.29178	0.017476
cg09175749	4.457584	1.068511	18.59601	0.040277
cg27659787	4.455311	1.13519	17.48588	0.032215
cg14768785	4.454135	1.692611	11.72114	0.002478
cg02300154	4.453217	1.729971	11.46328	0.001961
cg22802813	4.452783	1.42913	13.87367	0.010002
cg24221648	4.452271	1.559417	12.71162	0.00527
cg10242602	4.450857	1.527583	12.96828	0.00621
cg21899596	4.447271	1.49433	13.23551	0.007322
cg11346837	4.446532	1.400422	14.11835	0.011365
cg11214140	4.44582	1.438492	13.74031	0.009555
cg06243400	4.43958	1.168777	16.86366	0.028597
cg07846220	4.439121	1.548778	12.72345	0.005533
cg11183632	4.439031	1.352589	14.56836	0.013969
cg22650617	4.436036	1.464797	13.43423	0.00841
cg07963234	4.431752	1.452576	13.5211	0.008898
cg05209584	4.430407	1.451201	13.5257	0.008951
cg17541528	4.427745	1.523052	12.87213	0.006283
cg11601252	4.427687	1.573298	12.46071	0.004827
cg13298841	4.422342	1.704396	11.47451	0.002243
cg24072202	4.41814	1.063364	18.3568	0.040903
cg11893763	4.411539	1.33254	14.60495	0.015099
cg24934063	4.411373	1.271975	15.29921	0.01933
cg11907729	4.409312	1.605093	12.11272	0.004006
cg08286181	4.408299	1.467653	13.24094	0.008201
cg19484420	4.405982	1.285297	15.10365	0.018311
cg08189801	4.404466	1.20769	16.06315	0.024716
cg23676682	4.404161	1.424178	13.61953	0.010058

cg19623360	4.403046	1.013037	19.13731	0.048014
cg25181651	4.40229	1.44644	13.39852	0.009056
cg14134497	4.401637	1.603197	12.08485	0.004028
cg18096722	4.400374	1.545072	12.53229	0.005525
cg21229268	4.397616	1.383046	13.98292	0.012093
cg07027430	4.397202	1.304087	14.82676	0.016936
cg23642130	4.395859	1.517326	12.73528	0.006368
cg17653824	4.394064	1.07151	18.01924	0.039793
cg16407471	4.391631	1.45897	13.2192	0.008493
cg26348902	4.391282	1.228986	15.69047	0.022767
cg08490115	4.390629	1.507527	12.78758	0.006677
cg00100012	4.390421	1.086394	17.74292	0.03787
cg00332937	4.389973	1.534763	12.5569	0.0058
cg13576883	4.389334	1.440928	13.37072	0.009249
cg05057720	4.388218	1.601179	12.02643	0.004039
cg25335557	4.388065	1.113136	17.29808	0.034591
cg17093995	4.387903	1.634113	11.78235	0.003342
cg24937747	4.385168	1.121279	17.14979	0.03363
cg03530754	4.383386	1.491922	12.87874	0.007199
cg16086620	4.38338	1.606778	11.9581	0.0039
cg12640000	4.381821	1.040433	18.45419	0.04401
cg09686443	4.381727	1.322242	14.52043	0.015653
cg05081498	4.380765	1.447189	13.26095	0.008948
cg14289542	4.37853	1.272558	15.06534	0.019167
cg11850773	4.378144	1.397652	13.71454	0.011256
cg15590989	4.376327	1.60108	11.96207	0.00401
cg23207990	4.376203	1.500583	12.76248	0.006868
cg09969277	4.372325	1.428536	13.38239	0.009742
cg20997792	4.372308	1.041288	18.35907	0.043881
cg06335867	4.36947	1.489636	12.81673	0.007235
cg24613080	4.368614	1.066601	17.89309	0.040404
cg16767801	4.368598	1.450688	13.15559	0.008757
cg01718742	4.367728	1.208172	15.79001	0.024552
cg01805540	4.366332	1.664722	11.45228	0.002736
cg10239098	4.365352	1.673874	11.38455	0.002584
cg19884262	4.363784	1.424159	13.37113	0.009913
cg26425256	4.362113	1.247161	15.25707	0.021127
cg10731700	4.360932	1.733706	10.96941	0.001753
cg23712342	4.360908	1.104223	17.22253	0.035603
cg24101492	4.358991	1.367433	13.89523	0.012809
cg22536150	4.355281	1.239959	15.29767	0.021704
cg00582971	4.354564	1.538795	12.32278	0.005571
cg09559950	4.35418	1.022338	18.54464	0.046608
cg00489401	4.352571	1.331673	14.22637	0.014933
cg21266559	4.350405	1.299212	14.56731	0.017103
cg20253468	4.350184	1.223321	15.46946	0.023124
cg07919443	4.345194	1.594846	11.83858	0.004069
cg22356339	4.343008	1.328301	14.19988	0.015113
cg03078269	4.342669	1.531738	12.31201	0.005746
cg17011276	4.341637	1.19008	15.83912	0.026182
cg26492446	4.341021	1.707334	11.03737	0.002046
cg07665387	4.339748	1.305267	14.42879	0.01664
cg16675507	4.338678	1.428315	13.17926	0.00963
cg06329574	4.337569	1.487709	12.64663	0.007198

cg07438617	4.336789	1.744426	10.78162	0.001591
cg15107670	4.336461	1.226857	15.3277	0.022766
cg06110297	4.333495	1.157542	16.22333	0.029467
cg14265823	4.333192	1.232604	15.23324	0.022254
cg06947913	4.331541	1.675541	11.19773	0.002486
cg21169914	4.331469	1.357271	13.82305	0.01329
cg02119363	4.329902	1.265017	14.8204	0.019574
cg18375860	4.32514	1.314841	14.22745	0.01593
cg18646365	4.323913	1.267601	14.74929	0.019349
cg17537177	4.323458	1.279614	14.60775	0.018429
cg16580499	4.32111	1.702387	10.96812	0.002074
cg13161658	4.318083	1.151798	16.18847	0.03004
cg00891278	4.316553	1.159505	16.06947	0.029211
cg24809973	4.31533	1.181502	15.76136	0.026945
cg05589845	4.313992	1.173262	15.86221	0.027772
cg08260406	4.31381	1.437436	12.94594	0.009131
cg07175883	4.313143	1.26379	14.72017	0.019608
cg10851698	4.311968	1.188523	15.64384	0.02624
cg10676084	4.31159	1.274758	14.583	0.018752
cg10530883	4.310892	1.385348	13.41452	0.011645
cg16065021	4.31075	1.357247	13.69136	0.013212
cg11220663	4.310694	1.109325	16.75081	0.034878
cg19384289	4.310163	1.339032	13.87383	0.014308
cg02087954	4.310026	1.200734	15.4708	0.025058
cg23383871	4.30805	1.270556	14.60722	0.019061
cg21127068	4.307512	1.426902	13.00346	0.00958
cg22290744	4.30693	1.445593	12.83186	0.008752
cg16590237	4.301836	1.262471	14.65839	0.01967
cg26608174	4.3018	1.401369	13.20529	0.010783
cg17003293	4.298687	1.485054	12.44313	0.007162
cg00043819	4.298124	1.192681	15.48936	0.02579
cg22488797	4.295955	1.371219	13.45899	0.012356
cg06518628	4.295869	1.338824	13.78411	0.014266
cg04024095	4.295253	1.186937	15.54353	0.026342
cg04612444	4.291833	1.52424	12.0846	0.005816
cg24676817	4.289828	1.922652	9.571479	0.000376
cg25567594	4.288633	1.714145	10.72977	0.00186
cg11220245	4.287909	1.080815	17.01138	0.038406
cg12880658	4.286934	1.367155	13.44237	0.01255
cg09417809	4.282578	1.203792	15.23559	0.024678
cg14930075	4.280537	1.441852	12.70796	0.008817
cg00630958	4.277788	1.222796	14.96526	0.02292
cg08862035	4.274695	1.532614	11.92278	0.005506
cg01190024	4.27443	1.484635	12.30657	0.007094
cg03748376	4.273164	1.633691	11.1771	0.003071
cg01447112	4.270967	1.407884	12.95643	0.010343
cg24407243	4.270463	1.105782	16.49227	0.03522
cg02362103	4.268485	1.506701	12.09262	0.006305
cg17076890	4.265612	1.109291	16.40276	0.034781
cg05640128	4.264854	1.343508	13.53842	0.013855
cg25645064	4.262942	1.466606	12.39097	0.007736
cg24845274	4.260571	1.060684	17.11392	0.041052
cg12285988	4.260211	1.371644	13.23186	0.012194
cg23877720	4.259944	1.231451	14.73638	0.022094

cg23596233	4.258989	1.568919	11.56145	0.004456
cg13400168	4.257765	1.132799	16.00334	0.031989
cg18081940	4.25706	1.245123	14.55484	0.020916
cg09168808	4.254331	1.248959	14.49154	0.020587
cg13586599	4.252563	1.325119	13.6473	0.014968
cg00472801	4.250731	1.200814	15.04705	0.024851
cg17214381	4.25045	1.749621	10.32585	0.001397
cg26984343	4.243145	1.176395	15.30462	0.027233
cg23618344	4.242819	1.349752	13.3369	0.01339
cg15092343	4.241538	1.726275	10.42165	0.001631
cg10732215	4.241192	1.739484	10.34083	0.001486
cg20750832	4.238743	1.422888	12.62709	0.009508
cg12393318	4.23824	1.417554	12.6716	0.009755
cg13791254	4.238185	1.460249	12.30079	0.007898
cg00963169	4.235384	1.481731	12.10643	0.007065
cg16851417	4.234726	1.394834	12.85666	0.010857
cg17525406	4.233763	1.538845	11.64818	0.005195
cg26118906	4.227783	1.21576	14.70204	0.023378
cg11213369	4.226812	1.451141	12.31165	0.008227
cg25746778	4.226703	1.592126	11.22086	0.003809
cg07857243	4.225843	1.53196	11.6568	0.005371
cg14458834	4.219452	1.776374	10.02254	0.001108
cg27353143	4.217351	1.332851	13.34436	0.014331
cg26429655	4.217166	1.394556	12.7528	0.010803
cg26078977	4.214437	1.303213	13.62899	0.016296
cg24509810	4.211701	1.212815	14.62583	0.023591
cg12600018	4.21022	1.10806	15.99728	0.034805
cg22795586	4.209523	1.436089	12.33912	0.008804
cg25164624	4.208751	1.759001	10.07025	0.001243
cg15343119	4.207606	1.405954	12.59213	0.010194
cg14508508	4.206542	1.276666	13.86031	0.018204
cg04475027	4.205029	1.589343	11.12552	0.003812
cg08875705	4.200844	1.739554	10.14461	0.001419
cg08790440	4.200665	1.243429	14.19107	0.020847
cg16945186	4.200606	1.33946	13.17329	0.013849
cg02473540	4.197417	1.232391	14.29604	0.021782
cg17891759	4.19737	1.094773	16.09275	0.036437
cg09336320	4.197106	1.43289	12.29382	0.008898
cg23205387	4.193302	1.422646	12.35991	0.009346
cg04145134	4.192984	1.222879	14.37682	0.022607
cg19827780	4.191584	1.145529	15.33735	0.030369
cg19098710	4.190108	1.436702	12.22036	0.008704
cg01535698	4.189091	1.380454	12.71212	0.011432
cg07035165	4.188956	1.244249	14.10277	0.020734
cg12433277	4.185649	1.227348	14.27441	0.022182
cg14835443	4.185085	1.103909	15.86629	0.035261
cg12166610	4.183803	1.294455	13.52246	0.016795
cg19459876	4.182297	1.160485	15.07267	0.028705
cg00945238	4.181309	1.275162	13.71069	0.018219
cg11260046	4.17983	1.450763	12.04261	0.008069
cg00956199	4.17951	1.429743	12.21779	0.008971
cg06273376	4.177699	1.520317	11.47995	0.005567
cg00480389	4.177235	1.26498	13.79412	0.018995
cg05863502	4.177198	1.35523	12.87529	0.012802

cg13962186	4.176919	1.205943	14.46723	0.024108
cg03340466	4.17587	1.359345	12.82815	0.012557
cg02167020	4.173982	1.053821	16.53233	0.04189
cg24881420	4.17377	1.175244	14.82275	0.027127
cg07103129	4.173497	1.298265	13.41643	0.016481
cg07198552	4.173184	1.3468	12.93099	0.013289
cg07850418	4.171855	1.111994	15.6515	0.034233
cg23690166	4.170224	1.693995	10.26613	0.001892
cg07664198	4.170196	1.313822	13.2366	0.015388
cg14626309	4.169589	1.351638	12.86252	0.012984
cg07498624	4.166748	1.196598	14.50929	0.024967
cg03287574	4.164335	1.089621	15.91534	0.03703
cg17862113	4.159822	1.050358	16.47449	0.042364
cg05380019	4.157944	1.298364	13.31561	0.016411
cg14380586	4.157241	1.188253	14.54458	0.025754
cg08445263	4.155834	1.376092	12.55073	0.011534
cg04897683	4.155141	1.180993	14.61922	0.026477
cg19329160	4.152383	1.177883	14.63837	0.026785
cg07463541	4.151214	1.121423	15.36671	0.033042
cg15843567	4.150212	1.651833	10.42736	0.002464
cg05314394	4.149424	1.355426	12.70281	0.012678
cg25761626	4.148315	1.028271	16.73539	0.045593
cg03306374	4.146529	1.500529	11.45842	0.006098
cg04525757	4.146183	1.28946	13.3318	0.017006
cg08066943	4.145733	1.223865	14.0433	0.022343
cg10981651	4.14467	1.215179	14.13643	0.023129
cg06757662	4.14465	1.376999	12.47504	0.011439
cg18521914	4.143459	1.083248	15.84886	0.037821
cg08079580	4.14255	1.267374	13.54037	0.018669
cg12122146	4.14128	1.650763	10.38926	0.002461
cg01944655	4.140513	1.229942	13.93874	0.021782
cg20585038	4.139014	1.184276	14.46574	0.02609
cg00847277	4.138709	1.095118	15.64115	0.036268
cg10333808	4.138119	1.485507	11.5274	0.006586
cg16707405	4.137152	1.456922	11.74807	0.00766
cg17312004	4.13673	1.499703	11.41062	0.006092
cg26220673	4.134543	1.257629	13.5926	0.019415
cg26295057	4.131789	1.779428	9.593915	0.000964
cg14045872	4.131601	1.516048	11.25962	0.005547
cg03694713	4.131512	1.325818	12.87461	0.014433
cg10058779	4.130325	1.392373	12.25216	0.01057
cg16827475	4.129846	1.440856	11.83715	0.008296
cg05658491	4.128577	1.15424	14.76742	0.029216
cg05295006	4.125026	1.383122	12.30248	0.011031
cg05137358	4.12417	1.412418	12.04231	0.009555
cg11502555	4.121852	1.467825	11.57472	0.007178
cg03496122	4.120635	1.245851	13.62894	0.020333
cg01614020	4.119114	1.407396	12.05567	0.009775
cg12071328	4.119041	1.56409	10.84752	0.004165
cg08453021	4.116107	1.240653	13.65598	0.020757
cg10290276	4.113891	1.207193	14.01938	0.023761
cg04188273	4.111991	1.393375	12.1349	0.010444
cg25898550	4.109991	1.076056	15.69809	0.038718
cg02519751	4.109953	1.415003	11.93758	0.009376

cg27420520	4.109317	1.257885	13.42451	0.019293
cg02631462	4.105162	1.273492	13.23318	0.01804
cg01083633	4.102754	1.251596	13.4489	0.019782
cg24454144	4.101097	1.549598	10.85378	0.004483
cg26385172	4.097889	1.442233	11.64354	0.008115
cg03469054	4.095138	1.312131	12.78085	0.015192
cg16896079	4.094851	1.516564	11.05644	0.005407
cg12091642	4.094525	1.448556	11.57369	0.007839
cg25684640	4.092638	1.347501	12.43018	0.012913
cg15985191	4.091811	1.382032	12.11472	0.010953
cg25081106	4.09045	1.708996	9.790418	0.001559
cg23495279	4.086115	1.211734	13.77887	0.02323
cg13231921	4.085645	1.3576	12.29559	0.012286
cg14373923	4.084561	1.142973	14.5967	0.03034
cg18143296	4.082402	1.310985	12.71258	0.015217
cg02798576	4.081746	1.531249	10.88043	0.004928
cg06577205	4.081714	1.371784	12.14505	0.011466
cg11657203	4.081206	1.262896	13.18893	0.018775
cg00505277	4.078718	1.297432	12.8222	0.016149
cg27037551	4.077359	1.168349	14.22936	0.027528
cg12754421	4.075482	1.489588	11.15043	0.00622
cg27578811	4.074431	1.209079	13.73027	0.023435
cg16023545	4.074185	1.205039	13.77464	0.023818
cg20987924	4.072093	1.225199	13.53408	0.02194
cg09551147	4.07189	1.18409	14.00256	0.025874
cg20404387	4.070678	1.161128	14.27096	0.02828
cg18236477	4.070102	1.363499	12.14942	0.011882
cg25537434	4.06907	1.182049	14.00732	0.026072
cg24073122	4.067628	1.393038	11.87735	0.01028
cg11277087	4.06749	1.189632	13.90722	0.025299
cg23619365	4.067402	1.33633	12.38	0.013493
cg20337384	4.065704	1.55368	10.63922	0.004267
cg04328762	4.065357	1.401819	11.78977	0.009831
cg17714025	4.064532	1.116666	14.79442	0.03339
cg10084644	4.062443	1.325095	12.45454	0.01419
cg21854408	4.058864	1.120538	14.7022	0.032903
cg01802258	4.058377	1.420032	11.59862	0.008936
cg25958283	4.057738	1.645228	10.00787	0.002359
cg20475322	4.057248	1.646631	9.996936	0.002335
cg10652393	4.05209	1.340762	12.24634	0.013152
cg07104209	4.052014	1.449962	11.32362	0.007618
cg09229912	4.048673	1.213348	13.50952	0.022936
cg05031521	4.048019	1.245625	13.15521	0.02006
cg22687748	4.046642	1.173663	13.95231	0.026861
cg08553284	4.043738	1.308608	12.49557	0.015215
cg00090261	4.042759	1.348297	12.12188	0.012654
cg06419761	4.041644	1.414934	11.54463	0.009104
cg19509393	4.04046	1.14262	14.28762	0.030246
cg16242629	4.039004	1.240089	13.15515	0.020496
cg11994674	4.038947	1.281771	12.72699	0.017131
cg19792599	4.038497	1.076287	15.15344	0.038553
cg04200224	4.037143	1.24786	13.06118	0.019827
cg10108296	4.036255	1.037305	15.70547	0.044136
cg07212778	4.035027	1.206349	13.49647	0.023544

cg09554951	4.03266	1.340905	12.12789	0.01306
cg05627639	4.030036	1.478897	10.98196	0.00643
cg19760241	4.028561	1.371477	11.83345	0.011259
cg02317400	4.028276	1.235075	13.13848	0.020888
cg17774559	4.027761	1.446196	11.21761	0.007678
cg09799983	4.02708	1.637929	9.901147	0.002405
cg07315858	4.026994	1.12264	14.44512	0.03256
cg02034102	4.026863	1.101517	14.72117	0.035191
cg01718447	4.022102	1.552358	10.42112	0.004166
cg24483247	4.021424	1.411732	11.45533	0.009172
cg09524455	4.019146	1.492476	10.82331	0.005919
cg03638905	4.015734	1.017577	15.84757	0.047162
cg27200446	4.015359	1.552845	10.38295	0.004132
cg04550052	4.014519	1.49606	10.77254	0.005783
cg20732478	4.014484	1.017788	15.83442	0.047128
cg23244488	4.014437	1.416027	11.38093	0.008943
cg27072996	4.014411	1.538282	10.4763	0.004512
cg17816908	4.014201	1.286393	12.52635	0.016679
cg26381364	4.012584	1.44312	11.15696	0.007745
cg00800512	4.011668	1.059849	15.18469	0.0408
cg12921750	4.005569	1.093056	14.67865	0.036238
cg11563844	4.004078	1.11291	14.40605	0.033693
cg23266743	4.003165	1.240721	12.91615	0.020294
cg01796166	4.002195	1.212458	13.21083	0.022837
cg19947104	3.999638	1.3427	11.91413	0.012807
cg13266435	3.997567	1.389129	11.50401	0.010187
cg11071231	3.996215	1.442323	11.07223	0.007713
cg20377305	3.99537	1.328356	12.01709	0.013688
cg07533148	3.995	1.247269	12.79598	0.019701
cg00842325	3.994294	1.424274	11.20176	0.008485
cg27122536	3.993457	1.127478	14.14457	0.03188
cg21836358	3.992728	1.311377	12.15659	0.014803
cg15473084	3.989522	1.052373	15.12419	0.041847
cg14060496	3.988119	1.1754	13.53165	0.026471
cg26014036	3.985337	1.561273	10.17305	0.003831
cg25976257	3.981602	1.219335	13.00148	0.022114
cg25317585	3.981026	1.645976	9.628674	0.002171
cg18110483	3.980923	1.42849	11.09406	0.008243
cg20339230	3.980821	1.109333	14.2851	0.03408
cg05941108	3.980376	1.255157	12.62264	0.018981
cg07204550	3.980161	1.23114	12.86749	0.021038
cg13924432	3.978945	1.181615	13.39861	0.02579
cg23699196	3.978318	1.388577	11.39801	0.010134
cg12799689	3.977239	1.578673	10.02008	0.003407
cg03323696	3.976801	1.351614	11.70078	0.01217
cg19937061	3.976129	1.217417	12.98619	0.022269
cg18938204	3.974281	1.194259	13.2257	0.02449
cg04922810	3.973938	1.136105	13.90028	0.030796
cg19206040	3.973426	1.281056	12.3243	0.016902
cg16404157	3.973271	1.314987	12.00535	0.014472
cg06022179	3.972494	1.090054	14.477	0.036559
cg20139706	3.972487	1.156465	13.64559	0.028463
cg01159623	3.972214	1.245787	12.66547	0.019731
cg03357798	3.970517	1.437893	10.96397	0.007796

cg23189410	3.970063	1.603489	9.829439	0.002875
cg19793376	3.969258	1.280838	12.30055	0.0169
cg25975712	3.96916	1.420552	11.09022	0.008549
cg24129382	3.966332	1.50124	10.4792	0.005443
cg15984718	3.963704	1.053622	14.91137	0.041627
cg00637687	3.963393	1.140505	13.77328	0.030248
cg06528306	3.961482	1.49914	10.46823	0.005493
cg15174552	3.960527	1.32974	11.79612	0.013445
cg06838985	3.958608	1.033554	15.16184	0.044629
cg00247557	3.955504	1.207542	12.95691	0.023118
cg00812438	3.95338	1.400874	11.15675	0.00941
cg12573849	3.953271	1.078111	14.49605	0.038134
cg12973118	3.952394	1.113966	14.02325	0.03342
cg19502744	3.950984	1.113705	14.01652	0.033449
cg13012916	3.948824	1.332281	11.70415	0.013231
cg03970036	3.948513	1.651717	9.43912	0.002012
cg13640145	3.948397	1.320927	11.80219	0.013965
cg11002404	3.946707	1.233839	12.62442	0.020659
cg18331515	3.946113	1.288876	12.0817	0.016196
cg27615388	3.945183	1.247024	12.4813	0.01951
cg12382153	3.944541	1.496199	10.39928	0.005527
cg10943359	3.941837	1.028769	15.10356	0.045355
cg27100227	3.938429	1.569324	9.884017	0.003502
cg17029062	3.938329	1.22428	12.66902	0.021482
cg13677149	3.935721	1.382436	11.20479	0.010269
cg23244289	3.935569	1.298931	11.92419	0.015419
cg04812556	3.934117	1.437556	10.76638	0.007663
cg24507762	3.933083	1.114099	13.8849	0.03335
cg13601435	3.931595	1.704045	9.071028	0.00133
cg09477453	3.931065	1.095433	14.107	0.035748
cg24837370	3.928515	1.484641	10.39526	0.005853
cg24708471	3.928144	1.571663	9.817825	0.003419
cg03002848	3.927154	1.162235	13.26972	0.027667
cg25825488	3.926498	1.372034	11.23688	0.010786
cg13300273	3.922267	1.373346	11.20197	0.010696
cg00860808	3.921641	1.381634	11.13122	0.01025
cg23353952	3.92093	1.211376	12.6911	0.022611
cg14564076	3.920514	1.384969	11.09803	0.01007
cg21370856	3.918414	1.135797	13.51823	0.030657
cg07116997	3.918292	1.08729	14.12045	0.036806
cg16081281	3.918108	1.151957	13.32651	0.028782
cg21885046	3.917742	1.359087	11.29339	0.011473
cg24060451	3.917292	1.179277	13.01236	0.025801
cg11142389	3.915841	1.463275	10.4791	0.006569
cg09578028	3.91574	1.522773	10.06915	0.004616
cg21609106	3.914457	1.44625	10.59497	0.007226
cg20872937	3.913947	1.574106	9.731858	0.003323
cg14416371	3.912171	1.693771	9.036098	0.001405
cg09338032	3.911663	1.120405	13.65677	0.032501
cg03112087	3.908233	1.367363	11.17062	0.010963
cg04434339	3.907727	1.377439	11.08603	0.010411
cg12118269	3.904664	1.459359	10.44732	0.006673
cg20066716	3.904086	1.274907	11.9553	0.017065
cg23279117	3.90134	1.029842	14.77941	0.045152

cg03997176	3.900326	1.040203	14.62459	0.043548
cg09839170	3.896961	1.263677	12.01755	0.01792
cg23243867	3.896842	1.356483	11.19467	0.011529
cg27199820	3.893126	1.3963	10.85471	0.009375
cg23756251	3.892664	1.11023	13.64837	0.033726
cg22459630	3.892539	1.412071	10.73024	0.008616
cg26485937	3.890957	1.112473	13.60891	0.033436
cg23292160	3.888276	1.058429	14.28409	0.040806
cg18813020	3.887267	1.233872	12.24669	0.0204
cg27618240	3.887256	1.348564	11.20507	0.011951
cg21883598	3.887226	1.126699	13.41132	0.031653
cg14271531	3.886771	1.308466	11.54557	0.014527
cg10721149	3.886234	1.248252	12.09917	0.019148
cg01874877	3.884892	1.038753	14.52933	0.043752
cg24830738	3.884453	1.04812	14.39623	0.042328
cg04227961	3.882844	1.536962	9.809271	0.004118
cg23689712	3.882178	1.126116	13.38344	0.031709
cg09090724	3.881734	1.190241	12.65951	0.024531
cg06062984	3.88042	1.234477	12.1976	0.020317
cg23715830	3.879855	1.536067	9.799884	0.004132
cg26735846	3.879252	1.485019	10.1336	0.005656
cg10303487	3.878761	1.22009	12.33088	0.021615
cg14523847	3.878312	1.578989	9.525906	0.003114
cg15382538	3.876702	1.278954	11.75087	0.016628
cg13544006	3.875407	1.464226	10.25715	0.006375
cg08621473	3.873545	1.205054	12.45118	0.023023
cg14780632	3.871974	1.622628	9.239447	0.002282
cg00663077	3.871927	1.55936	9.614085	0.00353
cg19099050	3.871454	1.47452	10.16477	0.005988
cg01130127	3.869611	1.245382	12.02353	0.019318
cg22793136	3.865709	1.342934	11.12765	0.012191
cg16142306	3.865671	1.266038	11.8033	0.017589
cg11595545	3.863977	1.488015	10.03372	0.005498
cg24767540	3.862568	1.222003	12.209	0.021368
cg27606499	3.860867	1.391284	10.71406	0.009484
cg07681938	3.859681	1.158776	12.85593	0.027806
cg08431563	3.858406	1.490254	9.989769	0.005404
cg14510359	3.858204	1.424976	10.4463	0.007887
cg16190732	3.857118	1.443852	10.30394	0.007089
cg20507276	3.855903	1.309657	11.35258	0.014301
cg01307939	3.855797	1.108656	13.41008	0.033824
cg11700800	3.853159	1.106235	13.42105	0.03413
cg15186181	3.853035	1.448007	10.25263	0.006906
cg19134945	3.849593	1.418331	10.44846	0.008146
cg12175729	3.848518	1.3584	10.90334	0.011198
cg13933262	3.848211	1.61347	9.178184	0.002376
cg00174428	3.844708	1.406882	10.50677	0.008652
cg00339682	3.844487	1.240008	11.91934	0.01967
cg22345692	3.842733	1.014987	14.54856	0.047494
cg27058257	3.842042	1.35318	10.90859	0.01147
cg02745211	3.84014	1.468933	10.03904	0.006065
cg05379302	3.839843	1.26081	11.69438	0.017893
cg01610632	3.839708	1.341077	10.99367	0.012184
cg11548303	3.839532	1.193226	12.35475	0.024056

cg02765859	3.839085	1.195119	12.33231	0.023863
cg16332256	3.838484	1.363437	10.80648	0.010866
cg21956337	3.836182	1.427011	10.31267	0.007705
cg03775422	3.834711	1.420655	10.35086	0.007978
cg13172637	3.834104	1.326664	11.08069	0.013065
cg14381623	3.833632	1.036361	14.1811	0.044064
cg21545862	3.831729	1.283524	11.43894	0.016072
cg05842855	3.827994	1.249611	11.72648	0.018769
cg13777292	3.826481	1.402346	10.44105	0.008788
cg11615755	3.825263	1.141945	12.81378	0.029618
cg15520279	3.824225	1.103527	13.25268	0.034403
cg23572908	3.82306	1.559172	9.374066	0.003383
cg12196573	3.820358	1.054554	13.8401	0.041266
cg14750948	3.817258	1.255414	11.60689	0.018233
cg06952671	3.816039	1.537334	9.472344	0.003889
cg22837767	3.815952	1.4671	9.925355	0.006035
cg11381539	3.8154	1.229642	11.83863	0.020461
cg25260137	3.814329	1.088822	13.36224	0.036349
cg04774711	3.812986	1.184631	12.2729	0.02483
cg12768681	3.812203	1.281422	11.34122	0.016139
cg00741624	3.811953	1.282279	11.33216	0.016073
cg21154627	3.811874	1.193087	12.17881	0.023955
cg09147777	3.808439	1.348105	10.75896	0.011613
cg24430140	3.807636	1.179021	12.29672	0.025398
cg06460717	3.807384	1.098825	13.19243	0.034979
cg19949955	3.805389	1.129506	12.82064	0.031047
cg25953239	3.805319	1.295762	11.17524	0.015043
cg05983315	3.804948	1.388332	10.42808	0.009382
cg12307484	3.803386	1.333769	10.84577	0.012467
cg02630214	3.802619	1.308207	11.05323	0.01415
cg10445315	3.80259	1.369332	10.55967	0.010373
cg17877704	3.802404	1.11247	12.99655	0.033177
cg22534145	3.801791	1.174745	12.30362	0.025831
cg15928093	3.801677	1.174074	12.30991	0.025903
cg18425434	3.801061	1.221295	11.83012	0.021163
cg22849427	3.798422	1.214457	11.88022	0.021795
cg07073391	3.794569	1.389196	10.36481	0.009291
cg04945331	3.794475	1.506791	9.555436	0.004655
cg05571970	3.794407	1.050845	13.70089	0.041783
cg00745725	3.79313	1.310406	10.97968	0.013953
cg11178863	3.792779	1.374228	10.46782	0.010062
cg23302682	3.792537	1.256585	11.44638	0.01802
cg17336584	3.791086	1.174358	12.23846	0.02583
cg07703401	3.790666	1.125497	12.76694	0.031493
cg21424940	3.790648	1.088857	13.19642	0.036285
cg14970666	3.789776	1.153793	12.44799	0.028111
cg17657179	3.78815	1.248862	11.49052	0.018648
cg17316316	3.785016	1.169324	12.25182	0.026353
cg20463526	3.784588	1.535427	9.328417	0.003833
cg05227131	3.782073	1.34719	10.61772	0.011543
cg08046044	3.781862	1.102696	12.97046	0.034393
cg12478381	3.781339	1.286709	11.11248	0.015593
cg08426444	3.780077	1.223219	11.68146	0.020889
cg01800521	3.779789	1.247613	11.45131	0.018715

cg04127342	3.779424	1.232303	11.59134	0.020057
cg02483484	3.779416	1.284818	11.11752	0.015726
cg24856726	3.778105	1.160899	12.29571	0.02726
cg12612118	3.777941	1.197096	11.92289	0.023405
cg13721404	3.775817	1.215676	11.72746	0.021578
cg04021697	3.774394	1.567274	9.089698	0.003057
cg24637364	3.773959	1.262382	11.28245	0.017456
cg24838010	3.773595	1.004135	14.18137	0.04929
cg06151165	3.773549	1.474156	9.659539	0.005619
cg13051450	3.772579	1.260149	11.29418	0.017631
cg13706544	3.772461	1.067099	13.33659	0.039326
cg02250846	3.770624	1.311192	10.84327	0.01379
cg20495645	3.768933	1.397687	10.16312	0.008754
cg09797577	3.768137	1.230141	11.54247	0.0202
cg13491462	3.768126	1.085724	13.0777	0.036662
cg18451814	3.767974	1.343261	10.56953	0.011711
cg17074396	3.765381	1.045022	13.56727	0.042632
cg06094615	3.764726	1.29464	10.94757	0.014928
cg04028570	3.764158	1.27249	11.13477	0.0166
cg02119792	3.757439	1.497196	9.429859	0.004808
cg05442408	3.757281	1.023171	13.79746	0.0461
cg13628577	3.752212	1.470392	9.575059	0.005665
cg00384539	3.752055	1.257499	11.19517	0.017751
cg03506640	3.750444	1.266716	11.10417	0.016992
cg26422458	3.750277	1.345521	10.45289	0.01149
cg11021995	3.749443	1.121219	12.53842	0.031895
cg25517810	3.749336	1.309086	10.73843	0.013831
cg01024444	3.749132	1.462049	9.613897	0.00595
cg02760031	3.744718	1.089232	12.87413	0.036116
cg16113681	3.743211	1.272336	11.01253	0.01651
cg22633280	3.742659	1.133261	12.36034	0.030373
cg22769941	3.742458	1.31306	10.66668	0.013525
cg13344169	3.741853	1.099733	12.7317	0.034675
cg06444755	3.741373	1.076821	12.99925	0.037853
cg00252282	3.741046	1.357522	10.30954	0.010743
cg05534710	3.740374	1.191541	11.74143	0.023807
cg20585530	3.739197	1.176119	11.8879	0.025427
cg26186727	3.738776	1.163852	12.0105	0.026774
cg00966398	3.738562	1.029388	13.57782	0.045072
cg19841369	3.73842	1.396665	10.00654	0.008664
cg15014975	3.738027	1.214346	11.50648	0.021533
cg22346124	3.737589	1.386675	10.07415	0.009156
cg01354961	3.737146	1.283326	10.88286	0.015632
cg06704093	3.736914	1.109787	12.58307	0.033327
cg18652900	3.736854	1.433205	9.74325	0.007017
cg08551532	3.735736	1.138419	12.25886	0.029721
cg20279673	3.73459	1.213756	11.49091	0.021574
cg06535308	3.734385	1.026616	13.58408	0.045518
cg17285663	3.731948	1.321881	10.53608	0.012885
cg25809841	3.73147	1.123608	12.39211	0.031533
cg03391684	3.730963	1.1595	12.00525	0.027234
cg24198558	3.728563	1.33702	10.39788	0.011902
cg11120927	3.72744	1.454916	9.549564	0.006123
cg01610231	3.725244	1.132285	12.25614	0.030431

cg24066316	3.723155	1.113935	12.44407	0.032743
cg09403666	3.722638	1.298464	10.67263	0.014445
cg14730445	3.722148	1.160768	11.93553	0.027055
cg20023231	3.721767	1.252439	11.05966	0.018028
cg13208922	3.72054	1.204587	11.49143	0.022403
cg12161228	3.720183	1.314785	10.52626	0.013299
cg19411025	3.716411	1.106021	12.48774	0.03376
cg26667205	3.716248	1.320678	10.45713	0.012886
cg04671611	3.715887	1.240295	11.13269	0.019046
cg10224098	3.714411	1.426677	9.670623	0.007192
cg11789740	3.705798	1.080185	12.7135	0.037288
cg19443075	3.705095	1.073922	12.78279	0.038188
cg03281661	3.7045	1.111022	12.35198	0.033064
cg21548155	3.704249	1.28582	10.67137	0.015281
cg23638252	3.703645	1.084342	12.65006	0.036693
cg06831576	3.702866	1.220461	11.23446	0.020789
cg24643282	3.702111	1.022657	13.40197	0.046142
cg04245645	3.701357	1.172522	11.68425	0.02566
cg01464835	3.700323	1.156803	11.83641	0.02742
cg17305436	3.700049	1.346348	10.16852	0.011196
cg09252495	3.699771	1.387749	9.863677	0.008925
cg20129213	3.699401	1.607549	8.51331	0.002096
cg14007067	3.697932	1.145698	11.9357	0.028708
cg14638883	3.697618	1.270077	10.765	0.016464
cg23130254	3.695522	1.015464	13.4489	0.04734
cg00745389	3.695109	1.166607	11.70388	0.026288
cg06399302	3.694186	1.35503	10.07137	0.010658
cg18921771	3.691973	1.525653	8.934313	0.00377
cg00175709	3.689723	1.090521	12.484	0.03579
cg16256390	3.689262	1.398708	9.730878	0.008338
cg19656282	3.688258	1.288741	10.55545	0.014983
cg18795809	3.686716	1.223447	11.10949	0.020433
cg03949391	3.686653	1.074537	12.64862	0.038056
cg05142982	3.686434	1.617465	8.401909	0.001909
cg19497031	3.685393	1.34663	10.08601	0.011106
cg16945312	3.684252	1.429557	9.495045	0.006938
cg26872137	3.683473	1.119426	12.12048	0.031904
cg23746497	3.682267	1.230669	11.01766	0.019746
cg03506489	3.681344	1.053955	12.85851	0.041121
cg08492619	3.679118	1.455066	9.302606	0.005916
cg07687119	3.677812	1.142127	11.84308	0.029058
cg19810598	3.677542	1.163044	11.62837	0.026615
cg08217024	3.676716	1.52605	8.858317	0.003707
cg16664405	3.675357	1.328121	10.17095	0.012198
cg17384380	3.674418	1.127303	11.97668	0.030871
cg11475550	3.672272	1.257442	10.72462	0.017364
cg22978087	3.668347	1.110122	12.12189	0.033067
cg09582952	3.665445	1.096475	12.25334	0.034898
cg10617909	3.664007	1.34225	10.00182	0.011262
cg23180938	3.663426	1.417172	9.470052	0.007373
cg25645268	3.66335	1.302224	10.30555	0.013879
cg01009664	3.661161	1.285155	10.42994	0.015113
cg05099508	3.657908	1.261626	10.60559	0.016946
cg06617456	3.656753	1.366511	9.785386	0.00983

cg01625087	3.656384	1.211301	11.03702	0.021446
cg03217795	3.656016	1.328949	10.05791	0.012047
cg06158650	3.655775	1.0946	12.20966	0.035129
cg17985646	3.655642	1.165111	11.4699	0.02629
cg18685561	3.651528	1.227726	10.86044	0.019865
cg14985989	3.648048	1.541158	8.635231	0.003242
cg16697214	3.647826	1.27655	10.4239	0.015703
cg02232208	3.645157	1.251193	10.6196	0.017753
cg11229185	3.64413	1.183824	11.21761	0.024188
cg04167903	3.642691	1.092421	12.1466	0.035394
cg01645753	3.642302	1.14922	11.5438	0.028072
cg24129977	3.642282	1.269361	10.4511	0.016241
cg21145524	3.642223	1.391268	9.535038	0.008477
cg24801123	3.641922	1.01058	13.12474	0.048149
cg12974388	3.640505	1.342026	9.875573	0.011157
cg00257047	3.640137	1.114318	11.89121	0.032421
cg18267049	3.639496	1.409565	9.397179	0.007602
cg26170569	3.639226	1.232104	10.74907	0.019404
cg09493505	3.63831	1.413852	9.36258	0.007404
cg27019126	3.637836	1.210945	10.92853	0.021391
cg13267931	3.637339	1.354898	9.764744	0.010384
cg11073558	3.635825	1.09394	12.08405	0.035162
cg13842237	3.634108	1.034398	12.76756	0.044145
cg08812189	3.631879	1.192653	11.05983	0.023204
cg00379720	3.631618	1.210807	10.89244	0.021375
cg18877285	3.63086	1.041181	12.66172	0.043044
cg09275869	3.628687	1.279118	10.2941	0.015406
cg04267526	3.626878	1.015789	12.94978	0.047246
cg03250019	3.625803	1.262541	10.41269	0.016707
cg02091605	3.625496	1.029488	12.76772	0.044941
cg09813525	3.625092	1.255467	10.46726	0.01729
cg15104644	3.623777	1.314075	9.993158	0.012857
cg00177388	3.622744	1.102125	11.90816	0.033996
cg02622052	3.622199	1.196003	10.97015	0.022813
cg10038009	3.621185	1.408907	9.307199	0.007546
cg25886284	3.62068	1.271679	10.30867	0.015945
cg02320740	3.618989	1.171977	11.1752	0.025363
cg26727693	3.618657	1.302567	10.05298	0.013624
cg10216615	3.618153	1.139522	11.48817	0.029144
cg15428435	3.617712	1.28459	10.18833	0.014932
cg10541517	3.616662	1.201646	10.88527	0.022213
cg19542816	3.616332	1.29092	10.13065	0.014452
cg24390913	3.615731	1.258367	10.38926	0.017
cg05048377	3.613689	1.286556	10.15016	0.014763
cg24913868	3.611805	1.210965	10.77251	0.021263
cg15854847	3.611403	1.118081	11.66483	0.03183
cg13563725	3.608228	1.093677	11.90417	0.035118
cg03045635	3.606354	1.195575	10.87828	0.022782
cg05661282	3.604532	1.297444	10.01404	0.013915
cg01495122	3.604506	1.080948	12.01951	0.036921
cg14627172	3.602411	1.536638	8.445301	0.003196
cg16705627	3.601136	1.077502	12.0354	0.037414
cg21185289	3.601105	1.360852	9.529295	0.009865
cg03437186	3.600057	1.380568	9.387736	0.008807

cg08044097	3.599317	1.093705	11.84513	0.035088
cg16587616	3.597019	1.19604	10.81783	0.02269
cg08575537	3.596219	1.301325	9.938168	0.013594
cg04562217	3.595135	1.139222	11.34546	0.02909
cg17793621	3.594757	1.102429	11.72164	0.033866
cg25655234	3.594497	1.144647	11.28768	0.028426
cg27234067	3.592585	1.040093	12.40914	0.043164
cg11831238	3.591797	1.095514	11.77621	0.034812
cg20174066	3.591722	1.348084	9.569487	0.010547
cg17924936	3.591599	1.523587	8.466587	0.003474
cg08857144	3.590502	1.139158	11.31687	0.02908
cg06737561	3.588774	1.116222	11.5383	0.031994
cg07217924	3.587346	1.06781	12.05181	0.038821
cg02441747	3.587334	1.212048	10.61753	0.021037
cg00319168	3.586233	1.004925	12.79804	0.049122
cg02012703	3.58596	1.308874	9.824561	0.013013
cg05134015	3.584231	1.170608	10.97439	0.025361
cg13794530	3.58357	1.258156	10.20698	0.016849
cg09355771	3.583408	1.059218	12.12292	0.040123
cg02009585	3.582437	1.210667	10.60065	0.021148
cg24945701	3.581698	1.217551	10.53636	0.020475
cg26332560	3.581448	1.023721	12.52955	0.045863
cg19851909	3.580711	1.163672	11.01813	0.026129
cg12860391	3.580008	1.137566	11.26656	0.029234
cg18384778	3.579463	1.183078	10.82985	0.023971
cg09853371	3.577056	1.197457	10.68542	0.022448
cg00840960	3.576949	1.276707	10.02153	0.015321
cg05311410	3.576003	1.535416	8.328557	0.003136
cg14370314	3.575039	1.094714	11.67511	0.034873
cg22954449	3.574025	1.445329	8.837889	0.005827
cg03963198	3.573178	1.086733	11.74862	0.036
cg17689721	3.57271	1.212113	10.53059	0.020957
cg24190603	3.570712	1.401648	9.096424	0.007638
cg02657401	3.570566	1.308098	9.746162	0.012985
cg10325478	3.570286	1.09223	11.67057	0.035209
cg09980058	3.570217	1.295222	9.841128	0.013894
cg12684209	3.56838	1.054312	12.07739	0.040856
cg02886408	3.566106	1.16374	10.9278	0.026057
cg15698842	3.564901	1.092757	11.62978	0.035118
cg18382893	3.563678	1.122298	11.31589	0.031108
cg14714797	3.563671	1.269631	10.00271	0.015808
cg11362010	3.563616	1.345313	9.439703	0.010565
cg18755296	3.563362	1.354281	9.375859	0.010042
cg24727311	3.562345	1.24978	10.15402	0.017446
cg19939997	3.561817	1.059376	11.97549	0.040054
cg25999442	3.561674	1.37172	9.247896	0.009075
cg14448169	3.560195	1.333631	9.504122	0.011256
cg18101138	3.55996	1.055914	12.00223	0.040589
cg03146949	3.559848	1.193752	10.6157	0.022747
cg14885748	3.559079	1.25704	10.07688	0.016813
cg03425110	3.557462	1.240024	10.20588	0.018273
cg07565505	3.555723	1.107393	11.41705	0.03306
cg05756489	3.554831	1.161644	10.8784	0.026247
cg21041775	3.552857	1.332186	9.475246	0.011307

cg04963514	3.552456	1.309518	9.637088	0.01279
cg07788369	3.551358	1.209615	10.42657	0.021095
cg05229355	3.550369	1.012309	12.45185	0.047808
cg15054725	3.548335	1.33648	9.420775	0.011017
cg07252731	3.546145	1.211089	10.38334	0.020924
cg17498773	3.5461	1.335992	9.412355	0.011036
cg00310215	3.545843	1.172096	10.72693	0.025019
cg03815358	3.543436	1.104489	11.3681	0.033415
cg27393010	3.541105	1.309287	9.57729	0.012745
cg11248413	3.538219	1.078808	11.60448	0.037057
cg15708153	3.538166	1.411309	8.870219	0.007046
cg06272611	3.537637	1.162504	10.76545	0.026071
cg04223420	3.537599	1.158838	10.79928	0.026496
cg18161327	3.536152	1.384286	9.033082	0.008302
cg26309134	3.536079	1.342546	9.31354	0.010585
cg23346462	3.530454	1.310435	9.511427	0.012609
cg02350677	3.529771	1.057671	11.77992	0.040252
cg06164660	3.529506	1.218701	10.22188	0.020098
cg12840502	3.525257	1.263683	9.834295	0.016081
cg00481629	3.524972	1.139799	10.90141	0.028734
cg03882242	3.524298	1.372992	9.046436	0.008818
cg18115215	3.52303	1.188	10.44759	0.023173
cg11823511	3.522825	1.106891	11.21185	0.033016
cg17509967	3.522606	1.212266	10.236	0.020687
cg12993163	3.52171	1.27627	9.717728	0.015057
cg11597131	3.521418	1.429927	8.672041	0.006187
cg01586959	3.518731	1.240278	9.982813	0.018044
cg00090936	3.518387	1.227638	10.08363	0.019194
cg03058660	3.518293	1.281791	9.657099	0.014612
cg06496344	3.517936	1.337579	9.252442	0.010788
cg08120263	3.516585	1.204805	10.2642	0.021399
cg13031171	3.514724	1.032872	11.96013	0.044249
cg06549216	3.514556	1.019387	12.11718	0.04655
cg04347874	3.514379	1.427738	8.650647	0.006242
cg16987305	3.514186	1.420955	8.690987	0.00652
cg15272362	3.513322	1.349017	9.149947	0.010083
cg18325622	3.512991	1.141376	10.81248	0.028488
cg04733537	3.511863	1.175921	10.4881	0.024432
cg11552072	3.511232	1.194904	10.31777	0.022387
cg05036656	3.509114	1.202955	10.23636	0.021548
cg10567810	3.507134	1.023547	12.01703	0.045825
cg12250896	3.506514	1.261927	9.743547	0.016123
cg22340508	3.5054	1.271011	9.66776	0.015381
cg17847723	3.50123	1.095379	11.19121	0.034548
cg19492047	3.500469	1.292908	9.477306	0.013683
cg18787401	3.497192	1.287043	9.502679	0.014098
cg17910121	3.494387	1.027987	11.8783	0.045051
cg26692294	3.494277	1.098596	11.11416	0.03407
cg13702053	3.490819	1.028412	11.84916	0.044974
cg07291445	3.490814	1.308544	9.312474	0.012521
cg24415208	3.489583	1.186409	10.26391	0.023177
cg26343001	3.489288	1.214493	10.02486	0.020295
cg17722675	3.488508	1.061405	11.46564	0.039578
cg00840332	3.486459	1.212629	10.024	0.020463

cg21543987	3.486394	1.209962	10.04572	0.020726
cg00028935	3.486341	1.09607	11.08923	0.034401
cg04784475	3.482688	1.011692	11.98894	0.047884
cg03237606	3.47972	1.00345	12.06682	0.049369
cg11784623	3.479194	1.051676	11.51	0.041101
cg05522774	3.47864	1.091056	11.09103	0.035094
cg08516516	3.478046	1.243317	9.72946	0.017553
cg18276638	3.477959	1.334993	9.060869	0.01073
cg08586541	3.477067	1.102651	10.96448	0.033443
cg04510512	3.476093	1.237309	9.765723	0.018079
cg25442600	3.47585	1.018767	11.85897	0.046629
cg13982529	3.475072	1.201284	10.05268	0.021542
cg04542030	3.474555	1.029794	11.72324	0.04472
cg15811515	3.472809	1.240858	9.719408	0.017743
cg04996873	3.472692	1.34496	8.966506	0.010102
cg23906738	3.471323	1.29568	9.300201	0.013319
cg04005969	3.469264	1.169623	10.29032	0.024935
cg22428147	3.464175	1.133017	10.59164	0.029333
cg13458645	3.463793	1.039295	11.54423	0.043102
cg07099331	3.46346	1.280864	9.365205	0.014377
cg12764034	3.459404	1.040009	11.5071	0.042977
cg08441170	3.456952	1.448966	8.24762	0.005176
cg02401454	3.455879	1.138766	10.48775	0.028569
cg05923687	3.454883	1.070527	11.14985	0.038082
cg19096825	3.452531	1.349342	8.833914	0.009737
cg08784129	3.451835	1.144937	10.40683	0.027782
cg18325439	3.450572	1.126367	10.57066	0.030137
cg01350077	3.450502	1.263369	9.423983	0.015692
cg02318567	3.448896	1.181701	10.0659	0.023484
cg27553667	3.448752	1.175474	10.11838	0.024174
cg26371320	3.447064	1.21325	9.793735	0.020191
cg21124497	3.44611	1.296943	9.156669	0.013085
cg21745612	3.44526	1.000232	11.86706	0.049957
cg02428119	3.445215	1.13436	10.46362	0.029081
cg07915921	3.445188	1.233606	9.621641	0.018245
cg06653699	3.444486	1.103541	10.75128	0.033203
cg04698114	3.443919	1.291063	9.186681	0.0135
cg23714408	3.443731	1.056604	11.22396	0.040238
cg19610529	3.442511	1.289734	9.188626	0.01359
cg23290344	3.439161	1.071256	11.04108	0.037928
cg00378510	3.437874	1.15205	10.25908	0.026849
cg04557544	3.437325	1.321382	8.94155	0.011364
cg17046577	3.437164	1.025573	11.51951	0.045405
cg09894698	3.436871	1.104957	10.69008	0.032978
cg22851691	3.436827	1.271521	9.289489	0.014955
cg13246235	3.436435	1.250503	9.443471	0.016694
cg07477792	3.435651	1.09809	10.74931	0.033943
cg04131583	3.435492	1.247094	9.464082	0.016984
cg17892178	3.434023	1.132096	10.41653	0.029324
cg23676439	3.433242	1.005721	11.7201	0.048945
cg19288904	3.432754	1.295898	9.093154	0.013084
cg23421023	3.431963	1.071384	10.9936	0.03789
cg26195356	3.431362	1.16818	10.07914	0.024915
cg14258623	3.430969	1.068973	11.01201	0.038259

cg26460092	3.43033	1.258774	9.348111	0.015957
cg15408073	3.429539	1.011496	11.62807	0.047894
cg14062050	3.429019	1.160841	10.12901	0.025758
cg16420199	3.428627	1.113044	10.56157	0.03183
cg01739725	3.428174	1.23272	9.533694	0.018231
cg09233651	3.428095	1.163031	10.10449	0.025496
cg03224572	3.425281	1.311872	8.943365	0.011926
cg01878345	3.424606	1.500951	7.813666	0.003446
cg19560710	3.424397	1.141725	10.27086	0.028058
cg18082337	3.424258	1.277291	9.180012	0.01443
cg10416527	3.423827	1.326123	8.839746	0.010984
cg10486998	3.421076	1.174114	9.968163	0.024188
cg18372607	3.420553	1.277375	9.159554	0.014402
cg15312264	3.419208	1.235097	9.465641	0.017963
cg14131755	3.418392	1.107721	10.54905	0.032525
cg06946708	3.417313	1.09748	10.64077	0.033966
cg07113642	3.415726	1.276652	9.138891	0.01443
cg18774195	3.411291	1.089264	10.68328	0.035138
cg10410142	3.410973	1.386596	8.39086	0.007548
cg25088758	3.409652	1.201362	9.677117	0.021186
cg17542408	3.408254	1.37475	8.44968	0.00812
cg19472098	3.407523	1.324723	8.765011	0.01098
cg27042584	3.40692	1.091699	10.63215	0.034767
cg11723848	3.406647	1.136916	10.20766	0.028587
cg10358533	3.406196	1.215409	9.5459	0.019754
cg03304610	3.405683	1.378309	8.415152	0.007927
cg03289872	3.40506	1.288384	8.999205	0.013475
cg09885735	3.401582	1.160752	9.968327	0.025634
cg02589791	3.400716	1.304639	8.864421	0.01228
cg21656751	3.400533	1.028826	11.23964	0.044797
cg20884887	3.400027	1.504145	7.685554	0.003271
cg04917226	3.399808	1.13457	10.18773	0.028857
cg05039548	3.398707	1.050756	10.99324	0.041089
cg01240056	3.398641	1.225507	9.425293	0.018738
cg02978421	3.397685	1.226466	9.412622	0.018642
cg07696699	3.396197	1.330746	8.667435	0.010536
cg19084589	3.393626	1.196827	9.622695	0.02157
cg01939477	3.392431	1.33934	8.592728	0.009991
cg02743222	3.389962	1.106006	10.3904	0.032657
cg20927661	3.389729	1.379484	8.32939	0.007784
cg16422098	3.389306	1.221194	9.406693	0.019096
cg03032214	3.389239	1.223383	9.389489	0.018886
cg25339566	3.385551	1.337904	8.567102	0.010038
cg08526074	3.384833	1.309035	8.752319	0.011885
cg10659805	3.384297	1.206352	9.494302	0.020536
cg10521851	3.382636	1.073544	10.65837	0.03742
cg04853843	3.382191	1.029813	11.10805	0.044603
cg04248446	3.381412	1.232562	9.276569	0.017979
cg07466705	3.378804	1.006974	11.33725	0.048699
cg04035209	3.376745	1.133107	10.06295	0.028943
cg01995480	3.37642	1.199356	9.505276	0.021212
cg03940684	3.375504	1.170537	9.734023	0.024362
cg05686497	3.375243	1.25514	9.076491	0.015943
cg20567847	3.372101	1.50448	7.558136	0.003159

cg01559663	3.371453	1.143745	9.938137	0.027562
cg11334771	3.369889	1.146437	9.905605	0.027218
cg15959715	3.369711	1.380737	8.223831	0.007615
cg16357388	3.368581	1.185617	9.570831	0.022635
cg10603275	3.367903	1.356155	8.363921	0.008886
cg05085500	3.365905	1.106676	10.23725	0.032471
cg21806015	3.365729	1.065181	10.63494	0.038684
cg17504999	3.365209	1.069188	10.59181	0.03805
cg13874817	3.363904	1.134858	9.971156	0.028658
cg00926400	3.360593	1.176251	9.60134	0.023633
cg17460447	3.360098	1.514926	7.45268	0.002864
cg09548051	3.360085	1.255688	8.991221	0.015807
cg15384598	3.359499	1.396717	8.080548	0.006808
cg24723331	3.359395	1.239358	9.105949	0.01723
cg06750832	3.357037	1.245186	9.050614	0.016697
cg05991454	3.356819	1.167025	9.655519	0.024672
cg23263937	3.355969	1.293537	8.706766	0.012807
cg24842086	3.355453	1.090943	10.32049	0.034703
cg05619892	3.354721	1.154388	9.749017	0.026166
cg25652029	3.354469	1.319798	8.5259	0.010991
cg08196032	3.353862	1.077414	10.44018	0.036738
cg03813164	3.353712	1.301479	8.642	0.012225
cg06749053	3.353556	1.256066	8.953615	0.015736
cg10250663	3.353469	1.203573	9.343645	0.020647
cg20631104	3.352407	1.157244	9.711547	0.025809
cg08610862	3.35207	1.438677	7.81021	0.005067
cg00651523	3.351291	1.088614	10.31693	0.035035
cg14186641	3.350883	1.339984	8.379515	0.009716
cg21477176	3.349924	1.157467	9.695299	0.02577
cg16670554	3.349705	1.260124	8.904303	0.015373
cg18147485	3.349651	1.5056	7.452286	0.003048
cg18863595	3.348392	1.277847	8.773921	0.01394
cg07949597	3.346884	1.298827	8.624423	0.012372
cg03401357	3.346043	1.276302	8.772217	0.014046
cg01582980	3.345868	1.043502	10.72813	0.042195
cg13486532	3.345697	1.236594	9.052028	0.0174
cg13352836	3.345167	1.22702	9.119766	0.018286
cg23322933	3.344532	1.208507	9.255964	0.020093
cg03338754	3.342473	1.084524	10.3014	0.035618
cg12786548	3.342397	1.130103	9.885485	0.029181
cg08538258	3.339173	1.219785	9.14102	0.018944
cg13438893	3.338303	1.234856	9.024752	0.017515
cg23082454	3.337158	1.147405	9.705927	0.026939
cg09835225	3.33562	1.309369	8.497498	0.011572
cg09615505	3.334661	1.007855	11.03329	0.048521
cg10406295	3.334404	1.171075	9.494054	0.024085
cg07900968	3.334371	1.302889	8.533365	0.012011
cg13434308	3.333954	1.116344	9.956835	0.030996
cg24896649	3.333772	1.166808	9.525165	0.024578
cg05336698	3.333746	1.243364	8.938543	0.016719
cg05523056	3.331573	1.031258	10.76295	0.044282
cg09474331	3.331358	1.438104	7.71707	0.00499
cg24347663	3.33057	1.056115	10.5033	0.04006
cg12300353	3.330489	1.269909	8.734609	0.014458

cg03738025	3.330146	1.283552	8.639987	0.013393
cg00970396	3.329672	1.218585	9.098026	0.019005
cg23420260	3.328919	1.273916	8.698923	0.01413
cg10648197	3.328822	1.11845	9.907515	0.030685
cg09142313	3.326867	1.308682	8.457399	0.011567
cg23500059	3.326717	1.240341	8.922581	0.016947
cg25936054	3.325741	1.122903	9.84996	0.030067
cg17795158	3.324553	1.12686	9.808362	0.02953
cg21678445	3.324552	1.44272	7.660976	0.004795
cg13843613	3.323071	1.039929	10.6188	0.042763
cg01729827	3.321005	1.190283	9.265931	0.021866
cg23474501	3.320645	1.124286	9.807725	0.029858
cg03182917	3.317178	1.052339	10.45639	0.040653
cg07450037	3.316201	1.158408	9.493361	0.025484
cg06544111	3.315645	1.26247	8.707929	0.014972
cg07039180	3.313793	1.194619	9.192237	0.021359
cg22747746	3.311463	1.195812	9.17016	0.021219
cg02700891	3.31018	1.235614	8.867892	0.017277
cg23054189	3.309008	1.157482	9.459784	0.025559
cg19267252	3.307261	1.102829	9.918106	0.03279
cg10126205	3.307023	1.286756	8.499208	0.013011
cg17422774	3.30702	1.09485	9.988934	0.033953
cg19469447	3.30332	1.000744	10.90381	0.049858
cg00348762	3.303076	1.17219	9.30763	0.023788
cg02681442	3.302875	1.221517	8.930687	0.018561
cg05186311	3.3019	1.051967	10.36396	0.04068
cg21545390	3.301068	1.075671	10.13047	0.036846
cg23737737	3.299658	1.39039	7.830711	0.006781
cg10831607	3.296581	1.327187	8.188331	0.010177
cg10386483	3.296269	1.135206	9.571294	0.028298
cg08276295	3.295058	1.296088	8.377063	0.012254
cg18897632	3.294764	1.324753	8.194332	0.01032
cg17087640	3.28991	1.018115	10.63093	0.046595
cg24439256	3.289905	1.158432	9.34321	0.025344
cg24292761	3.289169	1.148288	9.421533	0.02659
cg14659193	3.287984	1.123317	9.624031	0.029842
cg05043349	3.287475	1.242806	8.696041	0.016488
cg19712603	3.286769	1.188841	9.086877	0.021828
cg05588496	3.286036	1.028998	10.49373	0.04462
cg23068913	3.284711	1.137699	9.483467	0.027918
cg19761848	3.283886	1.175281	9.175598	0.023327
cg09016242	3.283564	1.316486	8.189822	0.010784
cg06201642	3.28299	1.049323	10.2714	0.041083
cg25744613	3.281217	1.142074	9.427042	0.027337
cg08939095	3.281195	1.080771	9.961629	0.035989
cg18921980	3.279714	1.092871	9.842451	0.034145
cg22047387	3.278982	1.212355	8.868456	0.01932
cg06715976	3.278543	1.285787	8.359741	0.012907
cg04138502	3.276383	1.07709	9.966381	0.036546
cg00294096	3.276341	1.02408	10.482	0.045493
cg18705773	3.275239	1.210339	8.862963	0.019501
cg14002960	3.274828	1.218931	8.798281	0.018643
cg11224582	3.274362	1.438725	7.452046	0.0047
cg25527090	3.2727	1.075275	9.960765	0.036819

cg15617155	3.271393	1.232613	8.682374	0.017317
cg24213719	3.270803	1.202757	8.894696	0.020252
cg04597433	3.270771	1.137582	9.404106	0.027865
cg16138150	3.270709	1.161515	9.209985	0.02487
cg00741609	3.270422	1.025119	10.43357	0.045298
cg05292954	3.268588	1.266602	8.434902	0.014342
cg09422450	3.26807	1.138823	9.378354	0.02769
cg13092806	3.267865	1.348705	7.917923	0.008729
cg11064034	3.266508	1.103502	9.669285	0.03253
cg25104105	3.266383	1.323309	8.062556	0.01024
cg01485075	3.266295	1.295358	8.236089	0.012129
cg06392169	3.265496	1.185433	8.995414	0.02208
cg16269733	3.263754	1.234526	8.628484	0.017092
cg05157140	3.263712	1.459746	7.297036	0.003959
cg00614182	3.262587	1.108226	9.604966	0.031834
cg18599790	3.261524	1.181672	9.002106	0.022476
cg01815671	3.260637	1.132354	9.389071	0.028502
cg17023770	3.260341	1.033881	10.28148	0.043714
cg14695492	3.25999	1.105035	9.617375	0.032281
cg19149681	3.256265	1.075004	9.863458	0.036809
cg10453719	3.256177	1.112236	9.532771	0.031236
cg21547690	3.256079	1.283814	8.258245	0.012915
cg23900203	3.255806	1.01363	10.45773	0.047401
cg23234640	3.255256	1.105939	9.581627	0.032131
cg03781266	3.25399	1.080789	9.796961	0.035895
cg14441976	3.252198	1.209983	8.741274	0.019397
cg13875518	3.250637	1.110048	9.51909	0.031523
cg03442425	3.25021	1.077987	9.799623	0.03632
cg10034890	3.248798	1.473578	7.162624	0.003488
cg10959353	3.247978	1.1984	8.802872	0.020572
cg11842610	3.246783	1.325354	7.953797	0.009991
cg25775322	3.244659	1.129327	9.322204	0.028828
cg14781189	3.23825	1.11594	9.396798	0.030635
cg00824018	3.236052	1.089587	9.611008	0.034477
cg22875872	3.234829	1.302257	8.035371	0.011443
cg06785999	3.23306	1.212289	8.62227	0.019047
cg18497508	3.232703	1.039021	10.0579	0.042758
cg15146859	3.231631	1.28845	8.105427	0.012414
cg18468354	3.230108	1.258978	8.287355	0.014727
cg21751684	3.22988	1.003572	10.39499	0.049305
cg27288226	3.229306	1.119684	9.313711	0.030072
cg07519816	3.229136	1.042577	10.00148	0.042128
cg16509851	3.228711	1.026612	10.15434	0.044975
cg08829841	3.226686	1.082969	9.613849	0.035461
cg04281464	3.22659	1.324875	7.858012	0.009897
cg26814276	3.22622	1.352001	7.698585	0.0083
cg21859781	3.22603	1.192688	8.725895	0.021052
cg24369185	3.225668	1.251185	8.316063	0.015362
cg16935065	3.223685	1.318698	7.880608	0.010272
cg14738806	3.221564	1.219451	8.510778	0.018263
cg03577655	3.221185	1.249658	8.303095	0.015466
cg01603847	3.219454	1.241123	8.351214	0.016211
cg21776417	3.217203	1.202787	8.605345	0.019923
cg07212852	3.216853	1.027166	10.07446	0.044858

cg11254700	3.216306	1.104923	9.3623	0.032114
cg05178576	3.215938	1.096448	9.432516	0.033364
cg02172312	3.215492	1.025687	10.08045	0.045127
cg14231297	3.214495	1.011904	10.21142	0.0477
cg07920503	3.214107	1.375344	7.511197	0.007021
cg21606928	3.213536	1.21142	8.524554	0.019013
cg25116216	3.210589	1.19731	8.609206	0.020461
cg06428620	3.208992	1.343256	7.666168	0.008688
cg11885396	3.20899	1.074123	9.587004	0.036796
cg26104297	3.207264	1.06723	9.638546	0.037907
cg14470895	3.206936	1.198522	8.580934	0.020309
cg00812833	3.206842	1.122566	9.161005	0.029567
cg17603132	3.206774	1.109993	9.264381	0.031338
cg21517947	3.206041	1.233659	8.331879	0.016807
cg10569606	3.205626	1.064699	9.65159	0.038317
cg10615414	3.205386	1.146077	8.964926	0.026433
cg02828023	3.203319	1.272257	8.065395	0.013471
cg17971015	3.202225	1.056669	9.70431	0.039647
cg09772661	3.2012	1.105171	9.272484	0.032013
cg02767771	3.200019	1.318791	7.764782	0.010117
cg08086720	3.198642	1.319057	7.756533	0.010092
cg25201047	3.197905	1.122002	9.114593	0.029602
cg13407456	3.196756	1.246001	8.201638	0.015628
cg14072515	3.195063	1.324914	7.704971	0.009698
cg13834623	3.194917	1.055299	9.672609	0.03986
cg16230141	3.194209	1.128178	9.043761	0.028736
cg05733135	3.193658	1.039818	9.80888	0.042544
cg05825073	3.192569	1.136463	8.968609	0.027616
cg14625113	3.190951	1.236619	8.233879	0.016436
cg21331088	3.189845	1.119267	9.090868	0.029944
cg05680531	3.189841	1.143384	8.899098	0.026696
cg20764887	3.189106	1.119798	9.082346	0.029866
cg23905216	3.188518	1.044033	9.737859	0.041789
cg08776356	3.187071	1.142061	8.89394	0.026853
cg26272220	3.186471	1.289588	7.873521	0.012039
cg04748834	3.186062	1.135086	8.94293	0.027765
cg03810428	3.185625	1.06912	9.492113	0.037531
cg21114773	3.183723	1.109109	9.138953	0.031362
cg20100910	3.182009	1.212746	8.348967	0.018679
cg24469980	3.181841	1.024532	9.881696	0.045298
cg26059468	3.181365	1.14751	8.820039	0.02612
cg24657817	3.180466	1.076313	9.398167	0.03635
cg14783814	3.179753	1.026186	9.852825	0.044989
cg02467990	3.179688	1.348792	7.495905	0.008198
cg06610849	3.177937	1.176643	8.583136	0.022558
cg20095233	3.176617	1.170597	8.620298	0.023256
cg24863335	3.175778	1.116538	9.032889	0.030262
cg20718350	3.175259	1.152817	8.745768	0.025413
cg00400832	3.172631	1.088547	9.246815	0.034394
cg06159352	3.172242	1.301205	7.733693	0.011116
cg14230397	3.171575	1.078491	9.326821	0.03597
cg04545136	3.171261	1.093888	9.193715	0.033569
cg06558014	3.171056	1.201739	8.367541	0.019744
cg24080247	3.170133	1.269821	7.914298	0.013447

cg15672437	3.17002	1.131708	8.87952	0.028135
cg23316253	3.169681	1.009665	9.950705	0.048105
cg09618102	3.167516	1.181447	8.492261	0.021944
cg23500122	3.167021	1.154497	8.687787	0.025156
cg16589299	3.16677	1.195626	8.387597	0.020369
cg02587316	3.162565	1.139516	8.777248	0.027054
cg27111463	3.161277	1.008497	9.909478	0.048327
cg24604013	3.160136	1.144827	8.723119	0.026348
cg10649903	3.15955	1.032016	9.673057	0.043887
cg00875511	3.15939	1.000578	9.97598	0.049885
cg06845853	3.159074	1.304336	7.651212	0.010814
cg22702328	3.158837	1.035672	9.634568	0.043221
cg17307479	3.155672	1.08769	9.155427	0.034461
cg14304469	3.154508	1.128093	8.821011	0.028547
cg26437826	3.154468	1.017455	9.779965	0.046598
cg10366093	3.152093	1.078669	9.211064	0.035872
cg00919971	3.150583	1.099633	9.026809	0.032613
cg10601582	3.150526	1.224799	8.104032	0.017283
cg09971314	3.149816	1.188597	8.347103	0.021031
cg03072621	3.149655	1.199037	8.273576	0.019894
cg06660530	3.147245	1.063952	9.309778	0.038266
cg27621340	3.146293	1.080974	9.157629	0.035483
cg24031355	3.145667	1.046103	9.459126	0.04133
cg08053686	3.145084	1.012812	9.766422	0.047481
cg10864319	3.144791	1.297603	7.621523	0.011188
cg04819499	3.144044	1.173501	8.423522	0.022718
cg26777883	3.14226	1.188334	8.308944	0.021012
cg23676551	3.141554	1.205886	8.184323	0.01912
cg09019936	3.140971	1.128551	8.741917	0.028414
cg26116950	3.140431	1.314364	7.50348	0.010022
cg12919006	3.139022	1.276373	7.71989	0.012722
cg24812837	3.138379	1.195736	8.23712	0.020176
cg22920300	3.137395	1.052638	9.351031	0.040167
cg21210758	3.137288	1.210423	8.131515	0.018624
cg04514249	3.137117	1.069886	9.198651	0.037248
cg04293733	3.136805	1.173838	8.382376	0.022634
cg11205072	3.131651	1.341502	7.310639	0.008311
cg27360326	3.131611	1.141331	8.592592	0.026646
cg01245966	3.128851	1.079164	9.071566	0.035708
cg01921432	3.127292	1.235007	7.918945	0.016162
cg13940693	3.126426	1.077295	9.073219	0.036
cg23317501	3.124095	1.055945	9.242875	0.039559
cg24978630	3.123478	1.333959	7.313651	0.008696
cg24980653	3.123223	1.297647	7.517082	0.011041
cg16857858	3.122977	1.403182	6.950623	0.005274
cg26705960	3.12151	1.030626	9.454277	0.044081
cg19630629	3.12099	1.016863	9.579043	0.04668
cg11566394	3.119853	1.208974	8.051031	0.018657
cg18063312	3.117572	1.232074	7.888533	0.016369
cg15425280	3.117507	1.111294	8.745528	0.030736
cg01685883	3.117269	1.088008	8.931339	0.034258
cg05484788	3.114898	1.093743	8.870999	0.033356
cg25732462	3.11258	1.161487	8.341163	0.02397
cg21282549	3.112147	1.333441	7.263506	0.008654

cg02164046	3.111096	1.010966	9.573932	0.047818
cg01610488	3.111025	1.100693	8.793071	0.032279
cg10024876	3.110766	1.008683	9.593561	0.048267
cg18349911	3.110429	1.03573	9.341015	0.043121
cg16391955	3.109875	1.09458	8.835646	0.033206
cg04658772	3.108966	1.072421	9.012944	0.036733
cg17800654	3.108375	1.042718	9.266162	0.041848
cg11659501	3.107636	1.00254	9.63293	0.049488
cg14872657	3.107368	1.027419	9.398047	0.044657
cg04141813	3.10612	1.069748	9.01893	0.037166
cg15564098	3.105578	1.319027	7.311917	0.009494
cg08004425	3.104997	1.238736	7.782936	0.015666
cg17741501	3.104791	1.147655	8.399498	0.025669
cg01482645	3.103597	1.043268	9.232832	0.041739
cg17617843	3.103339	1.042297	9.2399	0.041913
cg03692651	3.102689	1.300128	7.404409	0.01073
cg12453014	3.102211	1.221578	7.878097	0.017272
cg04495670	3.101793	1.26704	7.593382	0.013208
cg24848035	3.100584	1.088934	8.828475	0.034044
cg22891500	3.099698	1.047119	9.175772	0.041041
cg04851268	3.098483	1.146124	8.376573	0.025831
cg07448060	3.098454	1.016766	9.442112	0.046678
cg15585794	3.097946	1.118085	8.583664	0.029658
cg17062109	3.097822	1.093247	8.77798	0.03336
cg05542338	3.096203	1.062875	9.019377	0.038289
cg03994318	3.096065	1.234132	7.767094	0.016029
cg20058043	3.094048	1.23616	7.744249	0.015827
cg24109980	3.093221	1.049663	9.115323	0.040573
cg03181478	3.090927	1.030568	9.270445	0.044042
cg09118932	3.090085	1.082546	8.820523	0.035016
cg00991875	3.089233	1.280408	7.453375	0.012072
cg08708684	3.087568	1.212436	7.862749	0.018085
cg18173058	3.087557	1.099555	8.66988	0.032345
cg16508480	3.086347	1.117954	8.520512	0.029618
cg04599297	3.085696	1.124852	8.464686	0.028635
cg17586860	3.084727	1.015753	9.367963	0.046862
cg14442421	3.084359	1.034616	9.194974	0.043277
cg13611347	3.084041	1.015978	9.361729	0.046818
cg19109538	3.081899	1.181061	8.042006	0.021447
cg22557091	3.081023	1.030246	9.214021	0.044086
cg00181125	3.080849	1.046847	9.066876	0.041043
cg21383810	3.080321	1.382838	6.861526	0.005902
cg03602280	3.079872	1.175681	8.068187	0.022059
cg24946597	3.079512	1.266133	7.490047	0.013127
cg22717014	3.077922	1.099623	8.615316	0.03229
cg00582524	3.076931	1.146904	8.254832	0.025603
cg07926691	3.076559	1.177361	8.03935	0.021841
cg11311843	3.076194	1.209517	7.823759	0.018306
cg03985727	3.075964	1.269124	7.455183	0.012861
cg21511365	3.075722	1.104897	8.561939	0.031482
cg24425021	3.075135	1.011233	9.351413	0.047743
cg20800509	3.075125	1.408179	6.715334	0.004818
cg03063639	3.074298	1.276033	7.406789	0.012305
cg18652346	3.074134	1.115713	8.470191	0.029878

cg03847373	3.073485	1.065587	8.864892	0.037755
cg05994094	3.073345	1.159184	8.148363	0.024015
cg08721908	3.07308	1.202698	7.852194	0.018998
cg15031661	3.073068	1.36452	6.920928	0.006723
cg24416513	3.072304	1.304268	7.237047	0.010239
cg02943578	3.070838	1.020127	9.243996	0.045999
cg01405040	3.070416	1.175983	8.016661	0.021962
cg05471296	3.070128	1.194842	7.888642	0.019823
cg05576262	3.069629	1.08865	8.655327	0.033959
cg08711858	3.068697	1.213938	7.757315	0.017803
cg23348270	3.064666	1.071177	8.768091	0.036783
cg18328894	3.062509	1.239079	7.5693	0.015338
cg12892506	3.062184	1.21743	7.702271	0.017406
cg04389897	3.06137	1.340812	6.989785	0.007903
cg17087479	3.061354	1.216809	7.702023	0.017462
cg20978694	3.061069	1.200935	7.802371	0.019103
cg04543008	3.060518	1.073579	8.724807	0.036367
cg22809871	3.060122	1.194343	7.840581	0.01981
cg00618450	3.060068	1.052791	8.894471	0.039931
cg15301316	3.058395	1.088477	8.593451	0.033938
cg04970570	3.057144	1.348595	6.930275	0.007447
cg00355281	3.056201	1.05335	8.867294	0.039821
cg14750277	3.055317	1.130459	8.25767	0.027687
cg23318063	3.054828	1.115487	8.36583	0.029811
cg05369857	3.052281	1.402741	6.641581	0.004906
cg27403635	3.052043	1.218744	7.64309	0.017204
cg14394692	3.051261	1.082735	8.598776	0.034829
cg26091021	3.051248	1.121414	8.302119	0.028937
cg02081266	3.050582	1.104821	8.423132	0.031371
cg02631468	3.050255	1.162985	8.000153	0.023398
cg13923530	3.050069	1.065129	8.734079	0.037755
cg14123923	3.049469	1.239069	7.505039	0.015247
cg07009376	3.047873	1.186274	7.830844	0.020626
cg17398252	3.047274	1.251575	7.419357	0.014119
cg03697918	3.046354	1.084945	8.553686	0.034452
cg12751432	3.045739	1.103977	8.402823	0.031475
cg01942962	3.045603	1.14276	8.116925	0.025962
cg03450948	3.045327	1.183683	7.834886	0.020904
cg23736843	3.044736	1.271264	7.292284	0.01247
cg15347189	3.044039	1.038727	8.920704	0.042435
cg03053579	3.042995	1.005132	9.212538	0.048951
cg27058486	3.042394	1.0375	8.921602	0.042659
cg23684973	3.039363	1.14672	8.055782	0.025401
cg23989821	3.039325	1.354448	6.820119	0.007024
cg05398903	3.038519	1.288356	7.166186	0.011125
cg21230745	3.037893	1.145345	8.057654	0.025573
cg00609966	3.037518	1.03304	8.931421	0.043484
cg27655158	3.037508	1.166185	7.911659	0.022922
cg25623768	3.033979	1.200207	7.669535	0.018994
cg18856581	3.033957	1.209386	7.611213	0.018026
cg23916167	3.03251	1.029149	8.935653	0.044212
cg22784954	3.029825	1.323979	6.933525	0.008681
cg04773818	3.028113	1.04733	8.755089	0.040822
cg24408656	3.027614	1.036646	8.842413	0.042787

cg21039708	3.027039	1.356464	6.755034	0.006843
cg05143887	3.02684	1.081947	8.467844	0.034857
cg02155398	3.026689	1.12122	8.170431	0.028831
cg04961466	3.025144	1.025833	8.921045	0.044836
cg14011639	3.024962	1.117156	8.190789	0.02941
cg18324707	3.023982	1.16303	7.86262	0.023223
cg11571585	3.022814	1.019965	8.958553	0.045975
cg27041794	3.020896	1.005594	9.075048	0.048849
cg19619405	3.020789	1.335044	6.835104	0.007965
cg03764518	3.019526	1.050504	8.679198	0.040226
cg07636117	3.019397	1.069576	8.523708	0.036889
cg25247290	3.018911	1.19339	7.63692	0.019631
cg11016563	3.01781	1.10226	8.262278	0.0316
cg02583633	3.01702	1.055265	8.625709	0.039367
cg09321400	3.016403	1.057532	8.603698	0.038964
cg23294090	3.016188	1.093608	8.318696	0.032938
cg19267596	3.016075	1.000364	9.093396	0.049924
cg15611413	3.015878	1.099244	8.274343	0.032056
cg02463418	3.014221	1.225294	7.414981	0.01629
cg08499046	3.012963	1.112896	8.157043	0.029972
cg15929698	3.012428	1.027005	8.83611	0.044591
cg26530498	3.011862	1.002949	9.044638	0.04939
cg02387803	3.011314	1.140254	7.95263	0.026091
cg01500945	3.011051	1.26393	7.173208	0.012817
cg05349062	3.010485	1.086657	8.340281	0.034022
cg08149193	3.010087	1.259972	7.191133	0.013137
cg12973591	3.009966	1.246635	7.26748	0.014281
cg10168149	3.009235	1.17436	7.711003	0.021748
cg00017221	3.009199	1.207371	7.5	0.018059
cg18722841	3.009191	1.004103	9.01823	0.049151
cg19875547	3.007971	1.151393	7.858211	0.024596
cg07411620	3.007553	1.212369	7.460911	0.01753
cg23632875	3.007274	1.196751	7.556875	0.01918
cg15949044	3.006201	1.142375	7.910931	0.025774
cg15228928	3.004733	1.047607	8.618137	0.04071
cg26476852	3.004317	1.074053	8.403606	0.036074
cg19714279	3.003379	1.049939	8.591246	0.040282
cg03020554	3.002906	1.012858	8.902969	0.047367
cg11389172	3.000475	1.007622	8.934749	0.048427
cg01851378	3.000274	1.133092	7.944315	0.027004
cg24663256	2.999702	1.036514	8.681229	0.042754
cg25683325	2.999697	1.097047	8.202183	0.032319
cg22660578	2.999682	1.00066	8.992161	0.049863
cg01775414	2.997323	1.107436	8.112379	0.030707
cg15621260	2.997071	1.089225	8.24663	0.033548
cg09851951	2.996153	1.0721	8.373227	0.036373
cg24724633	2.995283	1.277457	7.02311	0.01163
cg04398581	2.994398	1.213379	7.389624	0.017331
cg00290506	2.993886	1.002231	8.943396	0.049535
cg15051226	2.993604	1.075813	8.330127	0.035736
cg13163930	2.992587	1.1897	7.52759	0.019857
cg17400476	2.99186	1.280112	6.992534	0.011403
cg24610236	2.990388	1.263925	7.075116	0.012666
cg15594205	2.990314	1.26417	7.073399	0.012645

cg06401021	2.989733	1.337972	6.680637	0.007592
cg12926938	2.989647	1.183729	7.55071	0.020516
cg12602633	2.988883	1.124948	7.941188	0.028084
cg24524352	2.986579	1.08376	8.230287	0.034388
cg06183338	2.985778	1.07929	8.259935	0.035123
cg02253760	2.982351	1.345665	6.609683	0.007121
cg18249173	2.981323	1.094736	8.119114	0.032595
cg21161253	2.980272	1.097665	8.091743	0.032128
cg00549566	2.980044	1.155731	7.684025	0.023856
cg25778535	2.977711	1.074781	8.249832	0.035846
cg24911113	2.977608	1.034574	8.569854	0.043075
cg24280540	2.976117	1.11332	7.955725	0.02971
cg18627360	2.97571	1.121957	7.892328	0.028438
cg19923650	2.974133	1.040388	8.502083	0.041967
cg17218713	2.973431	1.178571	7.501707	0.021002
cg26667946	2.97215	1.25429	7.042769	0.013335
cg11985360	2.971894	1.068601	8.265153	0.036879
cg01570424	2.971115	1.105906	7.982165	0.030803
cg03243226	2.971056	1.173092	7.524707	0.021637
cg01743841	2.970929	1.212447	7.279839	0.017254
cg03679755	2.970799	1.190184	7.415367	0.019647
cg01181415	2.970009	1.221245	7.222919	0.016361
cg17290701	2.9691	1.169533	7.537673	0.022055
cg11873482	2.968642	1.23882	7.113897	0.014677
cg26328510	2.968481	1.215327	7.250625	0.016942
cg07401230	2.966002	1.133961	7.757914	0.026676
cg08236767	2.965002	1.305242	6.73533	0.009423
cg00661970	2.964964	1.284785	6.842396	0.010857
cg05438320	2.964884	1.064137	8.260724	0.037629
cg02315940	2.964502	1.132674	7.758872	0.026846
cg16896847	2.963956	1.19059	7.378722	0.019552
cg08151857	2.96349	1.219662	7.200581	0.016469
cg17448335	2.962433	1.001812	8.760135	0.049619
cg19679633	2.960349	1.218613	7.191506	0.01655
cg05694245	2.958981	1.081126	8.098566	0.034703
cg05783139	2.958728	1.094487	7.998331	0.032525
cg16415870	2.955404	1.041435	8.386899	0.041725
cg06410057	2.954076	1.028917	8.481309	0.044121
cg08287265	2.953894	1.138708	7.662625	0.025944
cg15508809	2.953701	1.023119	8.527207	0.045261
cg21277995	2.9532	1.262417	6.908484	0.012512
cg00295794	2.952853	1.21958	7.149465	0.016397
cg07180307	2.950993	1.106408	7.870839	0.030619
cg08448589	2.950592	1.05457	8.25549	0.039286
cg15775138	2.950468	1.085302	8.021048	0.033973
cg03782202	2.949339	1.008104	8.628676	0.048302
cg04150495	2.948806	1.196759	7.265833	0.018756
cg25875213	2.948441	1.161197	7.4865	0.022947
cg03225817	2.945132	1.138662	7.617537	0.025895
cg03483150	2.943192	1.023878	8.460362	0.045095
cg11755405	2.943056	1.077523	8.038413	0.035238
cg25963041	2.94151	1.267986	6.823797	0.011972
cg03585912	2.939781	1.023119	8.447021	0.045241
cg05223720	2.939063	1.082617	7.978894	0.034366

cg18106312	2.937952	1.147112	7.524604	0.024704
cg24346905	2.937634	1.066562	8.091134	0.037103
cg22758454	2.937263	1.036537	8.323403	0.042612
cg05495949	2.936739	1.012896	8.514625	0.047305
cg16007456	2.935599	1.132733	7.607922	0.026659
cg05513806	2.935028	1.127433	7.640708	0.027407
cg22531183	2.934373	1.236641	6.962849	0.014617
cg14118515	2.932791	1.398704	6.149453	0.004397
cg02681173	2.932088	1.045354	8.224145	0.040928
cg01893212	2.928982	1.322501	6.486907	0.008074
cg23827572	2.927572	1.172826	7.307717	0.021362
cg03595755	2.927411	1.033981	8.288099	0.043084
cg23891360	2.926671	1.176911	7.277866	0.020864
cg24761507	2.925799	1.29211	6.625054	0.010037
cg05525743	2.925588	1.13577	7.535915	0.02617
cg25266629	2.924635	1.163006	7.354636	0.022553
cg27298506	2.922791	1.007224	8.481432	0.048472
cg18582824	2.921011	1.052647	8.105573	0.039543
cg19001226	2.920086	1.20974	7.048538	0.017151
cg14861089	2.919755	1.389488	6.135334	0.004681
cg02970836	2.919484	1.185764	7.188099	0.019774
cg21322436	2.919317	1.142271	7.460935	0.025233
cg05593641	2.917716	1.084744	7.847998	0.033914
cg13879483	2.916581	1.40441	6.056953	0.004094
cg26858704	2.916354	1.0953	7.76511	0.032182
cg15873149	2.915897	1.109238	7.66513	0.029991
cg26649384	2.913769	1.171198	7.249032	0.02146
cg16234557	2.91375	1.242323	6.83392	0.013938
cg21484228	2.913676	1.055669	8.041827	0.038966
cg25984671	2.912481	1.081491	7.843381	0.034434
cg04904331	2.912254	1.301717	6.51541	0.009274
cg08602190	2.911531	1.070296	7.920253	0.036348
cg05167251	2.909967	1.027692	8.239732	0.044283
cg26162582	2.907436	1.260376	6.706875	0.012329
cg00690148	2.906746	1.089537	7.754825	0.033069
cg25051341	2.904278	1.107432	7.616568	0.030204
cg17787134	2.902892	1.174994	7.171764	0.02092
cg15562220	2.902769	1.132037	7.443277	0.026548
cg20312205	2.902758	1.09357	7.705045	0.032391
cg01995743	2.902166	1.118186	7.532349	0.028559
cg23881278	2.9004	1.13837	7.389794	0.025644
cg09354309	2.899997	1.131743	7.430995	0.026572
cg20099830	2.899348	1.023726	8.211394	0.045058
cg07676859	2.898829	1.137519	7.387318	0.025752
cg20185017	2.898204	1.114046	7.539708	0.029157
cg05161082	2.896867	1.096024	7.656617	0.031964
cg10196720	2.896491	1.203521	6.970926	0.017626
cg10358981	2.893833	1.109418	7.548349	0.029838
cg26354128	2.893081	1.258313	6.651701	0.012388
cg21200408	2.892516	1.238237	6.756906	0.014143
cg04541368	2.892383	1.229713	6.803119	0.014941
cg00765783	2.891249	1.145645	7.296608	0.024586
cg04574459	2.890218	1.085419	7.695977	0.03367
cg02623991	2.890017	1.179482	7.081246	0.020288

cg27319123	2.889956	1.14215	7.312389	0.025053
cg27291304	2.889508	1.025286	8.143347	0.044728
cg14872952	2.887278	1.050993	7.931907	0.039742
cg07651242	2.887091	1.012465	8.232677	0.047352
cg06338562	2.887047	1.113187	7.487546	0.029221
cg07849944	2.886421	1.050066	7.934198	0.039912
cg11717507	2.885837	1.037641	8.025953	0.042279
cg10636403	2.885817	1.185811	7.022991	0.019515
cg24226973	2.885638	1.018484	8.17579	0.046105
cg17300736	2.882896	1.124177	7.393041	0.027554
cg01615424	2.882646	1.060451	7.835958	0.037986
cg21097881	2.882398	1.091846	7.609328	0.032568
cg10520887	2.881359	1.041925	7.968164	0.04144
cg12118843	2.878943	1.103156	7.51327	0.030729
cg08276289	2.878812	1.044118	7.937376	0.041013
cg14073722	2.878805	1.055376	7.852672	0.038901
cg13222752	2.878744	1.147541	7.221674	0.024244
cg24005685	2.876732	1.164064	7.109223	0.022075
cg21587238	2.876576	1.091124	7.583637	0.032656
cg14817655	2.875477	1.049941	7.875077	0.039901
cg27443071	2.873061	1.008062	8.188464	0.048269
cg05575614	2.872844	1.156006	7.139437	0.023081
cg12374721	2.869453	1.247844	6.59839	0.013097
cg07746943	2.869096	1.121767	7.338162	0.027822
cg25340966	2.868229	1.169709	7.03315	0.021307
cg22572159	2.867927	1.123594	7.320265	0.027545
cg13252583	2.866876	1.08074	7.604951	0.034348
cg00903099	2.866384	1.008682	8.145441	0.048134
cg03278146	2.864989	1.253905	6.546082	0.012537
cg12477716	2.863342	1.160808	7.062948	0.022391
cg07124117	2.86285	1.02599	7.988298	0.04454
cg14667871	2.860977	1.037867	7.886552	0.042173
cg17045801	2.860109	1.067454	7.663299	0.036638
cg07068756	2.859957	1.142842	7.15703	0.024752
cg10293925	2.859957	1.126499	7.260864	0.027068
cg00334063	2.858958	1.215214	6.726095	0.016106
cg10300684	2.858495	1.147005	7.123764	0.024174
cg06008912	2.857029	1.114191	7.326045	0.028887
cg01708273	2.855727	1.068682	7.631062	0.036401
cg11841394	2.854388	1.041413	7.823535	0.041466
cg10811045	2.851979	1.312746	6.196006	0.008112
cg01581084	2.851571	1.094412	7.429978	0.031984
cg15662768	2.850068	1.126482	7.210844	0.027005
cg04763554	2.849852	1.211008	6.706526	0.016466
cg17926940	2.849852	1.055119	7.697387	0.038848
cg05663341	2.848106	1.324215	6.125673	0.007392
cg06808751	2.84712	1.030771	7.864107	0.043547
cg26567423	2.846563	1.067268	7.592206	0.036615
cg10397765	2.845764	1.15846	6.990636	0.022564
cg17059658	2.844728	1.139911	7.099215	0.025051
cg16783279	2.844228	1.064894	7.596653	0.037033
cg08361126	2.8434	1.031248	7.839944	0.043443
cg09754845	2.842032	1.000032	8.076883	0.049993
cg19063061	2.841825	1.103147	7.320846	0.030519

cg16426537	2.840542	1.006035	8.02028	0.048687
cg11642106	2.840333	1.116942	7.222837	0.028364
cg00287312	2.83945	1.017661	7.922552	0.046217
cg10384245	2.835991	1.001683	8.029331	0.049631
cg07918545	2.834505	1.064003	7.551128	0.037155
cg07573209	2.834421	1.103684	7.279208	0.03039
cg00904966	2.833952	1.294942	6.202042	0.00914
cg19098763	2.832326	1.088531	7.369629	0.032857
cg05241461	2.832272	1.053057	7.617598	0.039172
cg06187770	2.831613	1.00562	7.973221	0.048773
cg22759823	2.82959	1.160186	6.901116	0.022219
cg27136844	2.829233	1.04113	7.688341	0.041451
cg03227184	2.828269	1.054965	7.582342	0.038799
cg07150062	2.828011	1.102836	7.251892	0.030488
cg25636665	2.825323	1.107361	7.208534	0.029753
cg18229521	2.821435	1.04941	7.585687	0.039827
cg12824796	2.821027	1.207232	6.592096	0.016627
cg00040312	2.82054	1.117228	7.1207	0.028195
cg18406033	2.817298	1.036704	7.656154	0.042292
cg19945554	2.809581	1.066397	7.402262	0.036616
cg15393937	2.808902	1.056259	7.469692	0.038486
cg16206460	2.808016	1.183668	6.661454	0.019153
cg04317962	2.807742	1.059595	7.440028	0.037858
cg21426003	2.807371	1.119191	7.041989	0.02781
cg21319323	2.806417	1.007056	7.82079	0.048449
cg15808943	2.805548	1.072598	7.338352	0.035481
cg22797031	2.804625	1.170049	6.722729	0.020775
cg03943218	2.804607	1.105662	7.114127	0.029896
cg13128937	2.803707	1.007191	7.804647	0.048418
cg08146483	2.802999	1.130942	6.947134	0.026036
cg17302155	2.802231	1.049837	7.479733	0.039681
cg02478448	2.801435	1.157556	6.779831	0.022347
cg08534653	2.800716	1.080714	7.258169	0.034029
cg06274159	2.798259	1.179557	6.638297	0.019562
cg07302069	2.797464	1.347156	5.809131	0.005793
cg18009496	2.79717	1.011249	7.737126	0.047534
cg19579167	2.794826	1.06559	7.330265	0.036699
cg10143811	2.792771	1.327126	5.877037	0.00682
cg11668923	2.79223	1.000987	7.788857	0.04978
cg13314145	2.788203	1.033357	7.523124	0.042892
cg26595278	2.78738	1.058028	7.343364	0.038071
cg00548708	2.786818	1.031288	7.530732	0.04331
cg16024950	2.786177	1.269177	6.116391	0.010645
cg19406736	2.784573	1.008356	7.689593	0.048152
cg10362591	2.784053	1.026615	7.55001	0.044266
cg20340508	2.784046	1.133601	6.837429	0.025516
cg10364040	2.783938	1.092367	7.094971	0.031948
cg04556126	2.783616	1.060197	7.308562	0.037649
cg13142700	2.783094	1.160857	6.672323	0.021773
cg07972135	2.782864	1.105533	7.00507	0.029783
cg01672943	2.782293	1.11216	6.960469	0.028729
cg10863741	2.775933	1.028664	7.491081	0.043825
cg04059773	2.775751	1.048205	7.35046	0.039906
cg15042811	2.775693	1.043413	7.383917	0.040846

cg01047586	2.774144	1.014787	7.583731	0.046748
cg14038391	2.772902	1.065796	7.214314	0.036566
cg25915838	2.768087	1.046402	7.322526	0.040233
cg07080358	2.767056	1.10848	6.907294	0.029211
cg23895340	2.765847	1.017736	7.516599	0.046105
cg24680632	2.765335	1.063114	7.193092	0.037029
cg12997720	2.765232	1.061146	7.2059	0.037396
cg01705052	2.764108	1.134592	6.733954	0.025227
cg26132774	2.763815	1.178318	6.482691	0.019429
cg10922935	2.763267	1.129367	6.760996	0.025984
cg01618130	2.763091	1.006867	7.582598	0.048467
cg27347269	2.762248	1.090303	6.99807	0.032174
cg20398486	2.759931	1.006779	7.565934	0.048485
cg01873311	2.75975	1.008587	7.551374	0.048085
cg07142797	2.759151	1.052246	7.234918	0.039064
cg08893692	2.758272	1.055976	7.204767	0.038345
cg18348647	2.756168	1.131759	6.712081	0.025581
cg02345991	2.754774	1.019037	7.447009	0.045811
cg16301890	2.7523	1.095752	6.913205	0.031196
cg09907936	2.751475	1.11216	6.807128	0.028526
cg10507508	2.749988	1.024981	7.378121	0.044541
cg27467929	2.749701	1.064083	7.105514	0.03678
cg12506930	2.74905	1.024957	7.373262	0.044544
cg13242070	2.746672	1.004063	7.51368	0.049084
cg09513990	2.745318	1.076816	6.999127	0.034434
cg15679813	2.743202	1.239435	6.071442	0.012792
cg22001496	2.741223	1.215504	6.182047	0.015086
cg25640822	2.74084	1.079123	6.961398	0.033999
cg16370491	2.739496	1.014979	7.394083	0.046667
cg26410450	2.736789	1.013991	7.386668	0.046879
cg24569447	2.736523	1.095742	6.834239	0.031103
cg19803052	2.73512	1.073483	6.968792	0.034982
cg07197785	2.73444	1.04508	7.154635	0.040383
cg23089825	2.733316	1.057387	7.065546	0.037975
cg18070676	2.732379	1.053591	7.086146	0.038703
cg01287975	2.730579	1.013044	7.360057	0.04708
cg15603568	2.729644	1.174735	6.342672	0.019579
cg05571581	2.727926	1.04161	7.144304	0.041057
cg22861116	2.726337	1.113826	6.673315	0.028092
cg14669274	2.725011	1.047365	7.089872	0.039896
cg18034737	2.724652	1.058855	7.011095	0.037658
cg15175143	2.724339	1.023045	7.254834	0.044904
cg23797411	2.724127	1.114624	6.657733	0.027951
cg14510812	2.723107	1.020259	7.268073	0.0455
cg17840719	2.721136	1.027109	7.209148	0.044033
cg00778995	2.72013	1.146241	6.455107	0.023237
cg04184232	2.714937	1.056018	6.979888	0.038163
cg19147218	2.71368	1.177123	6.255979	0.019148
cg06288251	2.707065	1.07066	6.844563	0.035359
cg13445796	2.706771	1.161589	6.307406	0.021055
cg04431946	2.705855	1.110161	6.595126	0.028534
cg10188823	2.704151	1.104788	6.618854	0.029394
cg04370314	2.703479	1.02215	7.150411	0.045058
cg19570244	2.703121	1.014362	7.203408	0.046759

cg02230017	2.702708	1.004096	7.274834	0.049062
cg01670677	2.700532	1.026226	7.106498	0.044177
cg05196969	2.69895	1.05579	6.899414	0.038141
cg01070209	2.698491	1.000772	7.276236	0.049822
cg05475524	2.698253	1.050759	6.928869	0.039126
cg11891393	2.696374	1.105013	6.579498	0.029305
cg00516513	2.696331	1.044512	6.960382	0.040367
cg23121993	2.695336	1.159297	6.266588	0.02126
cg09238180	2.692782	1.067015	6.795666	0.035967
cg05099387	2.691103	1.092563	6.628482	0.031362
cg04454951	2.688708	1.074574	6.727456	0.034544
cg04846243	2.686136	1.011123	7.135952	0.047462
cg21158633	2.685463	1.197488	6.02237	0.016515
cg24934400	2.681871	1.117161	6.438133	0.027249
cg26860935	2.680721	1.038704	6.918492	0.041504
cg26236177	2.677889	1.032976	6.942166	0.042691
cg17459204	2.677448	1.090858	6.571641	0.031572
cg19461621	2.677406	1.004925	7.133368	0.048862
cg14324370	2.677067	1.019154	7.031992	0.045666
cg20945566	2.676334	1.191454	6.011779	0.017116
cg25084878	2.675888	1.017544	7.036919	0.046019
cg10528576	2.675344	1.062263	6.737943	0.036786
cg08495115	2.674274	1.092447	6.546534	0.031276
cg05020604	2.674228	1.133701	6.308097	0.024668
cg10512875	2.67337	1.084976	6.587157	0.03258
cg09221867	2.673342	1.107384	6.453734	0.028757
cg16791619	2.671766	1.086475	6.570176	0.032305
cg18630667	2.671272	1.079597	6.60959	0.033532
cg09354241	2.667572	1.01228	7.029613	0.047183
cg26074603	2.666625	1.152724	6.168771	0.021899
cg11414560	2.661546	1.114249	6.35749	0.027561
cg26067203	2.660517	1.055915	6.703522	0.037953
cg16729415	2.659942	1.000923	7.068764	0.049784
cg04575395	2.657687	1.018193	6.937093	0.045847
cg03505117	2.656889	1.120652	6.299064	0.026514
cg23536473	2.656284	1.062225	6.642517	0.036703
cg14979301	2.65586	1.19784	5.888592	0.016203
cg18805066	2.655425	1.027245	6.864264	0.043859
cg00711088	2.654932	1.015735	6.939471	0.046392
cg08322102	2.653114	1.122069	6.273245	0.026264
cg23954465	2.646428	1.024783	6.834207	0.044374
cg25763036	2.646121	1.005899	6.960895	0.048623
cg11600596	2.645597	1.050527	6.662542	0.038964
cg14874750	2.645228	1.050583	6.660336	0.038951
cg05214690	2.644903	1.085515	6.444419	0.032311
cg07990546	2.641704	1.012734	6.890856	0.047052
cg22499720	2.638305	1.051292	6.621049	0.03878
cg03900143	2.638243	1.019862	6.824768	0.045444
cg11074814	2.637172	1.016417	6.842349	0.046213
cg00418216	2.636767	1.054645	6.592304	0.038101
cg07095230	2.635629	1.050285	6.613959	0.038972
cg17629929	2.635524	1.026268	6.7682	0.044026
cg08545287	2.634722	1.047589	6.626411	0.039517
cg05415020	2.633489	1.043659	6.645145	0.04032

cg08190858	2.632919	1.119086	6.194574	0.026575
cg10784386	2.63217	1.047198	6.616057	0.039587
cg17371081	2.629455	1.146813	6.028909	0.022399
cg16304215	2.628288	1.084044	6.372344	0.032472
cg06241792	2.627132	1.111323	6.210455	0.027777
cg24032666	2.625881	1.17208	5.882919	0.018988
cg01328892	2.625569	1.008037	6.83865	0.048115
cg24150172	2.6229	1.073107	6.410923	0.034455
cg00177451	2.614645	1.081732	6.319837	0.032807
cg22286978	2.613806	1.060159	6.444298	0.036902
cg23424003	2.60868	1.080464	6.298415	0.033003
cg22898797	2.608207	1.127799	6.031875	0.025018
cg16166796	2.606852	1.041473	6.52506	0.040681
cg06060135	2.605662	1.075443	6.313187	0.033918
cg18130044	2.604877	1.183233	5.734615	0.017414
cg05151154	2.602651	1.151807	5.881013	0.021462
cg15313459	2.601234	1.063251	6.363894	0.036231
cg15230781	2.600542	1.01193	6.683087	0.04719
cg09017619	2.598596	1.054294	6.40495	0.038002
cg03232620	2.598416	1.047527	6.445433	0.039385
cg25209842	2.597921	1.026396	6.575626	0.04391
cg17373442	2.596368	1.132061	5.95474	0.024269
cg10770742	2.596049	1.005065	6.705502	0.048793
cg26122980	2.594335	1.186314	5.67352	0.016944
cg05888917	2.594146	1.017558	6.613475	0.045889
cg02895639	2.591989	1.074379	6.253295	0.034037
cg21938148	2.591202	1.05712	6.35153	0.037398
cg23235241	2.589296	1.021644	6.562413	0.044953
cg10448808	2.586095	1.068998	6.256222	0.035032
cg12505170	2.585539	1.006303	6.643142	0.048494
cg27510182	2.584178	1.002218	6.663199	0.049467
cg19115393	2.583999	1.026263	6.506176	0.043906
cg25397922	2.583051	1.055757	6.319782	0.037634
cg26963797	2.582391	1.110557	6.004865	0.027558
cg12356890	2.581533	1.202007	5.544318	0.015027
cg07821427	2.581068	1.186588	5.614344	0.016782
cg08249988	2.580663	1.009562	6.596741	0.047722
cg26195829	2.57919	1.003551	6.628678	0.049146
cg02340083	2.579126	1.031829	6.4467	0.042663
cg06122871	2.578506	1.029019	6.461194	0.04328
cg27170782	2.577569	1.09987	6.04059	0.029329
cg17437939	2.577328	1.070296	6.206341	0.034732
cg25078444	2.575724	1.064385	6.233038	0.035874
cg17320707	2.569837	1.086631	6.077556	0.031623
cg05221057	2.566466	1.044086	6.308625	0.039978
cg19871940	2.565977	1.030592	6.388794	0.042897
cg12060744	2.56199	1.073336	6.115318	0.034057
cg13539545	2.559963	1.02393	6.400256	0.044374
cg21229570	2.558773	1.033031	6.337965	0.042338
cg19944763	2.556644	1.005688	6.499457	0.048623
cg11573679	2.556129	1.10064	5.936359	0.029035
cg08464190	2.554282	1.076752	6.059292	0.033358
cg07034660	2.553868	1.051425	6.203242	0.038386
cg19074340	2.553796	1.089067	5.988497	0.03107

cg15132565	2.552541	1.113191	5.852966	0.026882
cg16703956	2.551476	1.02416	6.35646	0.044302
cg22029275	2.55011	1.151088	5.649493	0.021073
cg24531255	2.54698	1.029916	6.298672	0.042993
cg09341793	2.546406	1.029422	6.298856	0.043102
cg07143532	2.544114	1.040494	6.220615	0.04066
cg23524195	2.544069	1.026998	6.302143	0.043641
cg21045464	2.543938	1.047536	6.177947	0.039155
cg22471726	2.543614	1.130457	5.723326	0.024051
cg06223767	2.54296	1.079257	5.99176	0.032811
cg13021333	2.542035	1.126163	5.738018	0.024704
cg24847685	2.541433	1.071257	6.029256	0.034334
cg09671258	2.539614	1.068689	6.035099	0.034825
cg09420439	2.539556	1.088534	5.924796	0.031066
cg16787600	2.538034	1.018015	6.327625	0.045687
cg20443254	2.534108	1.022807	6.278513	0.044571
cg21350575	2.531573	1.034407	6.195687	0.041947
cg15133351	2.529942	1.124148	5.69374	0.024915
cg22976218	2.529607	1.03541	6.180076	0.041719
cg14192957	2.527588	1.119516	5.706665	0.025637
cg13699355	2.525856	1.07701	5.923759	0.033126
cg03059131	2.521496	1.037222	6.129777	0.041291
cg08248516	2.520074	1.018204	6.237229	0.04561
cg25738714	2.519038	1.039842	6.10242	0.040707
cg27600205	2.518927	1.073098	5.912777	0.033836
cg12162138	2.517627	1.093578	5.796065	0.02999
cg04546041	2.512674	1.01172	6.240391	0.047137
cg08575330	2.510074	1.008503	6.247349	0.04791
cg00459623	2.508323	1.106884	5.684144	0.027576
cg05417127	2.501856	1.099749	5.691554	0.028765
cg23697546	2.499508	1.074282	5.815546	0.033481
cg06740600	2.494608	1.021708	6.090853	0.044737
cg01578017	2.490821	1.111011	5.584272	0.026724
cg11267630	2.487108	1.054458	5.866241	0.037426
cg07152216	2.487105	1.002149	6.172426	0.049461
cg27223047	2.485287	1.012155	6.102473	0.046997
cg24150623	2.484844	1.131263	5.458015	0.02338
cg03779241	2.482413	1.057461	5.827518	0.036772
cg13267264	2.482154	1.016144	6.063204	0.046031
cg11359133	2.481222	1.131189	5.442471	0.023357
cg23262036	2.479947	1.056232	5.822712	0.037016
cg13062406	2.479303	1.01074	6.081628	0.047334
cg13661740	2.479265	1.014238	6.060465	0.046485
cg24686074	2.474875	1.0171	6.022029	0.045789
cg15506157	2.469343	1.207283	5.050725	0.01329
cg24403845	2.467141	1.080182	5.634961	0.032113
cg11818631	2.46654	1.081595	5.624855	0.031837
cg13356896	2.465065	1.138118	5.339117	0.022134
cg18479593	2.462337	1.087473	5.575407	0.03069
cg10362542	2.460027	1.102992	5.486652	0.027844
cg05224741	2.459344	1.099796	5.499544	0.028406
cg02043159	2.457982	1.033764	5.844348	0.04184
cg25285090	2.456156	1.046514	5.764566	0.038978
cg16288089	2.454401	1.059337	5.686656	0.036222

cg05375728	2.451899	1.003783	5.989151	0.049039
cg27464184	2.449405	1.047248	5.728907	0.038786
cg23080354	2.449037	1.005065	5.967558	0.048715
cg16504626	2.448398	1.052384	5.696262	0.037665
cg21393713	2.442253	1.098056	5.431963	0.028574
cg17712694	2.437572	1.037246	5.728397	0.040969
cg09780241	2.435355	1.059159	5.599683	0.036148
cg10903903	2.429065	1.153881	5.113491	0.019448
cg23858040	2.426484	1.026155	5.737755	0.043512
cg23806894	2.413559	1.058011	5.505865	0.036262
cg26134895	2.413228	1.041306	5.592659	0.039942
cg00532157	2.412536	1.020028	5.706051	0.044951
cg17016394	2.406719	1.068137	5.422804	0.03409
cg22455914	2.405354	1.085224	5.331363	0.030667
cg06204735	2.402808	1.151737	5.012852	0.019466
cg19715410	2.401403	1.014888	5.68214	0.046196
cg23528400	2.398261	1.023897	5.617417	0.043973
cg18991611	2.396597	1.117974	5.137579	0.024665
cg18856388	2.396229	1.032867	5.559199	0.041823
cg04307561	2.395059	1.008367	5.688713	0.047834
cg02081006	2.391857	1.021047	5.603051	0.044652
cg18323912	2.386879	1.009083	5.645908	0.047642
cg19513834	2.376116	1.040489	5.426225	0.039959
cg11372090	2.370876	1.049445	5.356213	0.037892
cg24675150	2.353983	1.078365	5.138551	0.031604
cg10297491	2.339224	1.002374	5.459006	0.049362
cg00343633	2.332518	1.037012	5.24646	0.040576
cg05864326	2.327984	1.017693	5.325288	0.045338
cg06463958	2.320623	1.058947	5.085513	0.035462
cg11328303	2.316948	1.000073	5.367856	0.04998
cg11014373	2.311638	1.047138	5.10312	0.038083
cg14763548	2.30989	1.015571	5.253785	0.045845
cg00674365	2.302307	1.026119	5.165697	0.043125
cg18448949	2.296052	1.042159	5.058591	0.039167
cg14487131	2.284858	1.057769	4.935458	0.035475
cg11638181	2.284623	1.02706	5.081982	0.042824
cg11965976	2.270683	1.064752	4.842443	0.033809
cg18680788	2.265354	1.001752	5.122853	0.04951
cg23695133	2.250674	1.028968	4.922928	0.042207
cg19019537	2.244744	1.055325	4.774711	0.035746
cg22604316	2.23929	1.048273	4.783507	0.03737
cg16801887	2.234174	1.03113	4.840837	0.041583
cg27154343	2.228462	1.048126	4.73802	0.037334
cg02471897	2.227307	1.072225	4.62673	0.031799
cg03048654	2.223486	1.014212	4.874614	0.046021
cg27555582	2.20718	1.019991	4.776164	0.044408
cg06445348	2.200819	1.004905	4.819961	0.048587
cg26654934	2.195361	1.007239	4.78497	0.047916
cg08768852	2.191642	1.051447	4.568269	0.036275
cg08018585	2.190683	1.006083	4.770077	0.048241
cg11017065	2.111797	1.001052	4.455	0.049678
cg22830113	2.098807	1.021899	4.310592	0.043492
cg18243760	0.418559	0.185728	0.943268	0.035654
cg05649108	0.414928	0.17454	0.986394	0.046484

cg12248614	0.399237	0.170491	0.934886	0.034425
cg08409113	0.376683	0.14969	0.947896	0.038115
cg03140412	0.355848	0.144906	0.873863	0.024189
cg03733219	0.34198	0.118909	0.983531	0.046506
cg20712263	0.34134	0.119273	0.976857	0.045112
cg14457782	0.338084	0.129746	0.880956	0.026463
cg17768491	0.337016	0.141592	0.802161	0.013963
cg20518446	0.330841	0.146136	0.748999	0.007973
cg04754315	0.330651	0.113242	0.965452	0.042942
cg00602295	0.325646	0.111453	0.951483	0.040279
cg09287864	0.320067	0.113474	0.902789	0.031298
cg02604503	0.317601	0.102876	0.980506	0.046131
cg08683938	0.309644	0.096602	0.992521	0.048542
cg15108727	0.302646	0.107149	0.854831	0.024068
cg03518729	0.300692	0.097287	0.929369	0.036871
cg09225457	0.298046	0.113874	0.780085	0.013668
cg10949007	0.293691	0.09505	0.907464	0.033282
cg16389901	0.288054	0.111236	0.745937	0.010355
cg24366211	0.286699	0.097102	0.846496	0.023719
cg08549335	0.285342	0.087657	0.928851	0.037294
cg07388969	0.283669	0.104284	0.771623	0.013596
cg08782022	0.280796	0.108944	0.723737	0.008557
cg27359566	0.277109	0.10591	0.725044	0.008919
cg16206813	0.273324	0.096664	0.772839	0.01445
cg03612357	0.272018	0.089556	0.826223	0.021635
cg15039102	0.271965	0.079612	0.92907	0.03777
cg23309670	0.270459	0.077161	0.94799	0.04101
cg22202558	0.263802	0.076796	0.906189	0.034309
cg16476991	0.263484	0.085741	0.809693	0.019885
cg19101547	0.260206	0.073822	0.917171	0.036218
cg04029159	0.257687	0.092992	0.714063	0.009118
cg19174643	0.256724	0.073842	0.892546	0.032454
cg23807071	0.253917	0.091679	0.703256	0.008358
cg22047295	0.253479	0.072225	0.889601	0.032146
cg21151432	0.250917	0.067964	0.926362	0.03801
cg07474842	0.249459	0.074086	0.839958	0.024993
cg13775050	0.249325	0.089158	0.697224	0.008113
cg05467676	0.248513	0.08088	0.763582	0.01506
cg04495354	0.243401	0.078897	0.750907	0.013957
cg04937184	0.241035	0.081249	0.715062	0.010334
cg02006615	0.238329	0.07564	0.750939	0.01432
cg12273284	0.238185	0.086314	0.657273	0.005601
cg03354554	0.237435	0.065715	0.857877	0.028246
cg06976598	0.233579	0.060928	0.895468	0.033922
cg21708130	0.233415	0.05918	0.920621	0.037701
cg06114334	0.23096	0.087241	0.611439	0.003174
cg20765408	0.230306	0.083414	0.635878	0.004601
cg15787146	0.226126	0.055049	0.928864	0.039176
cg05981038	0.226107	0.052752	0.969146	0.045267
cg15017278	0.224168	0.065616	0.765842	0.017053
cg12534147	0.223857	0.067129	0.746504	0.014862
cg05792169	0.223012	0.055799	0.891308	0.033775
cg26647617	0.22145	0.055934	0.876754	0.031769
cg13905238	0.221014	0.068926	0.70869	0.011112

cg22790839	0.219588	0.048306	0.998194	0.049727
cg13906811	0.219557	0.065947	0.730968	0.013487
cg12060422	0.219107	0.08102	0.592543	0.002781
cg00971050	0.218313	0.050505	0.943675	0.041592
cg25508319	0.218173	0.065462	0.727129	0.013184
cg10900455	0.21794	0.063975	0.742446	0.014844
cg21035875	0.217646	0.060924	0.777521	0.018908
cg01288184	0.217256	0.074557	0.633074	0.005146
cg18020065	0.217248	0.055267	0.853966	0.028816
cg23019886	0.217113	0.062731	0.751425	0.015904
cg26389380	0.216434	0.071946	0.651098	0.006458
cg25450266	0.216091	0.056763	0.82264	0.024691
cg12052765	0.213401	0.059126	0.77022	0.018342
cg14823429	0.211604	0.056601	0.79108	0.020983
cg02585906	0.211108	0.062549	0.712507	0.012206
cg03577157	0.210284	0.064818	0.682205	0.009408
cg08446038	0.210179	0.062	0.712504	0.012274
cg27549878	0.210067	0.05827	0.75731	0.017087
cg06552563	0.200066	0.042067	0.951502	0.04313
cg24631526	0.200006	0.040709	0.982645	0.047533
cg18442362	0.199337	0.064336	0.617622	0.005188
cg25874782	0.199327	0.039798	0.998337	0.049764
cg08415592	0.197883	0.054746	0.715256	0.013469
cg26998850	0.196215	0.058213	0.661374	0.008618
cg26620710	0.195662	0.050847	0.752918	0.017657
cg21568661	0.194693	0.040589	0.933885	0.040809
cg09129067	0.19443	0.061913	0.610582	0.005033
cg09782560	0.193236	0.045725	0.81662	0.025388
cg18178715	0.190272	0.042263	0.856615	0.030651
cg20834178	0.187629	0.047434	0.742177	0.017082
cg24965479	0.187471	0.04374	0.803512	0.02416
cg22525688	0.187069	0.049608	0.705429	0.013315
cg11122255	0.185821	0.039969	0.863905	0.031829
cg24921221	0.1832	0.034061	0.985355	0.048025
cg04794141	0.182609	0.03751	0.888983	0.035231
cg20175702	0.181894	0.057291	0.577496	0.003835
cg12394201	0.181596	0.045837	0.719441	0.015151
cg15564619	0.179317	0.053389	0.602267	0.005432
cg03297783	0.178659	0.037426	0.852859	0.030809
cg13656752	0.178488	0.050442	0.63158	0.007525
cg06487082	0.17769	0.031778	0.993578	0.049149
cg19391247	0.177227	0.045689	0.687461	0.012356
cg01973676	0.176644	0.049521	0.630104	0.007545
cg20803910	0.17603	0.052079	0.594993	0.005182
cg24287110	0.172898	0.038807	0.770321	0.02132
cg00664697	0.169483	0.043565	0.659356	0.010442
cg05336982	0.167543	0.032571	0.861832	0.032524
cg24039081	0.166635	0.040966	0.677816	0.012308
cg13972124	0.165867	0.033402	0.823648	0.028003
cg02853152	0.165743	0.042682	0.643617	0.009416
cg23323669	0.163678	0.032134	0.833698	0.029336
cg01141339	0.163177	0.040959	0.650088	0.010152
cg22009908	0.163116	0.026931	0.98796	0.048479
cg13780718	0.161769	0.049384	0.529908	0.002621

cg14019757	0.161554	0.037498	0.696038	0.014437
cg24227481	0.159955	0.045846	0.558071	0.004043
cg01425762	0.159595	0.03707	0.687098	0.013747
cg05315321	0.159414	0.034797	0.730315	0.018045
cg10893986	0.157013	0.043026	0.572976	0.005061
cg06261066	0.156539	0.043409	0.564498	0.004601
cg16371229	0.155932	0.033374	0.728568	0.018149
cg06788267	0.15489	0.044792	0.53561	0.003216
cg04202892	0.154871	0.040327	0.594765	0.006592
cg24500959	0.152781	0.042219	0.552881	0.004196
cg15244327	0.151806	0.024314	0.947808	0.043661
cg15688551	0.147762	0.031785	0.686922	0.01473
cg02518245	0.147691	0.02592	0.841526	0.031216
cg18672030	0.147253	0.031108	0.69704	0.015737
cg22700686	0.146527	0.036322	0.591106	0.00696
cg07146104	0.144911	0.027348	0.767849	0.02318
cg06433467	0.144457	0.026406	0.790272	0.025653
cg13454226	0.144306	0.040788	0.510546	0.002675
cg06853894	0.143757	0.041908	0.49313	0.002042
cg25181684	0.14373	0.02665	0.77517	0.02406
cg04951797	0.14272	0.024875	0.818842	0.028948
cg07541559	0.14189	0.035852	0.56156	0.005401
cg26994377	0.14138	0.036509	0.547485	0.004625
cg03064100	0.140189	0.031947	0.615171	0.009218
cg21571658	0.140055	0.039862	0.492082	0.00217
cg04663932	0.139577	0.020265	0.961366	0.045502
cg27132471	0.138503	0.039019	0.491627	0.002225
cg09417692	0.137812	0.029707	0.639308	0.011362
cg12126901	0.137167	0.022002	0.855138	0.033373
cg24158259	0.135206	0.04106	0.445222	0.000999
cg23521140	0.133677	0.04041	0.442206	0.000978
cg21430685	0.133114	0.031625	0.560293	0.005961
cg03466587	0.132732	0.041269	0.426897	0.000704
cg07937427	0.132214	0.034948	0.500183	0.002878
cg20968743	0.131137	0.030923	0.556123	0.005852
cg02892153	0.13066	0.017844	0.956762	0.045126
cg01413054	0.128175	0.035371	0.464464	0.001764
cg09550909	0.127642	0.017019	0.957313	0.045242
cg03553715	0.124662	0.0265	0.586434	0.008402
cg09126279	0.124613	0.035341	0.439386	0.001199
cg06590173	0.123575	0.030257	0.504705	0.003587
cg05398036	0.119938	0.032913	0.437071	0.001307
cg10087036	0.118102	0.034475	0.404587	0.000673
cg25799109	0.116804	0.030313	0.450074	0.001809
cg05673882	0.115781	0.027994	0.478867	0.002916
cg06858555	0.114523	0.029765	0.440631	0.001621
cg17130457	0.114093	0.023257	0.559703	0.007469
cg26502852	0.113627	0.027536	0.468881	0.002636
cg26612165	0.113603	0.014091	0.915901	0.041106
cg09803959	0.112433	0.023068	0.547992	0.006845
cg15101245	0.112321	0.015341	0.822353	0.031356
cg19358397	0.111851	0.017361	0.720603	0.021183
cg06197966	0.108374	0.02149	0.546543	0.007107
cg26577252	0.10801	0.019504	0.598139	0.01082

cg03800922	0.107011	0.021008	0.54509	0.007135
cg14515996	0.106793	0.020465	0.557285	0.007965
cg24003508	0.105279	0.018635	0.594763	0.010831
cg23804620	0.104987	0.01551	0.71066	0.020887
cg07220152	0.103793	0.012751	0.844882	0.034216
cg00446414	0.102896	0.017741	0.596774	0.011227
cg12435551	0.102363	0.018414	0.569036	0.00921
cg16066354	0.101143	0.021774	0.469824	0.003456
cg13585930	0.099213	0.02806	0.350788	0.000336
cg17576316	0.099065	0.013774	0.712485	0.021635
cg11847468	0.098488	0.016585	0.584867	0.01077
cg12280317	0.098148	0.018344	0.525121	0.006674
cg13749927	0.097648	0.009889	0.964219	0.046464
cg00973309	0.097395	0.0186	0.509984	0.005831
cg05926314	0.096996	0.028896	0.325591	0.000159
cg13324103	0.096995	0.022529	0.417593	0.001734
cg14834675	0.096105	0.022509	0.410341	0.001563
cg27181375	0.09437	0.017806	0.500164	0.005534
cg10536999	0.093196	0.019862	0.437289	0.002624
cg01799671	0.092053	0.020553	0.412282	0.001819
cg22234930	0.091012	0.017821	0.464783	0.003965
cg04926881	0.087451	0.024133	0.316898	0.000208
cg08824350	0.08737	0.013044	0.585204	0.012
cg27619475	0.085715	0.011354	0.647107	0.017221
cg25518868	0.08504	0.019446	0.371887	0.001061
cg10773016	0.084874	0.007636	0.943348	0.044704
cg13600477	0.084079	0.00875	0.807866	0.03197
cg26361533	0.083012	0.016131	0.427186	0.002906
cg21324456	0.081697	0.016222	0.411441	0.002392
cg23344780	0.08062	0.012095	0.537381	0.009278
cg01827726	0.080017	0.006799	0.941732	0.044676
cg20618695	0.079962	0.013904	0.459846	0.00465
cg03851835	0.079661	0.018633	0.340564	0.000642
cg05259836	0.079465	0.009657	0.653863	0.01852
cg15034300	0.078846	0.014783	0.42052	0.002937
cg23683800	0.077433	0.01088	0.551094	0.010617
cg03966751	0.076879	0.01709	0.345838	0.000826
cg16210842	0.076751	0.013724	0.429229	0.003468
cg25095994	0.075903	0.014307	0.402698	0.00246
cg24438354	0.07439	0.018771	0.29481	0.000217
cg14356919	0.073797	0.015158	0.359287	0.001249
cg11935248	0.073012	0.010179	0.523699	0.00923
cg05309989	0.070882	0.011342	0.442996	0.004644
cg07756483	0.068371	0.008807	0.53077	0.010295
cg23072383	0.068117	0.009683	0.479192	0.006954
cg25053413	0.06801	0.011457	0.40373	0.003096
cg00068038	0.066	0.005886	0.740077	0.027522
cg20873416	0.065952	0.008117	0.535869	0.010971
cg00834924	0.064339	0.008129	0.509223	0.00934
cg26191447	0.06282	0.010302	0.383079	0.002699
cg15771683	0.061134	0.007713	0.484542	0.008146
cg13942922	0.060699	0.006721	0.548203	0.012585
cg22827210	0.059847	0.012043	0.297396	0.000576
cg19262282	0.057516	0.009982	0.331408	0.001394

cg04387396	0.054068	0.005429	0.538454	0.012852
cg00950718	0.049359	0.005811	0.419252	0.005845
cg13485320	0.044996	0.004301	0.470716	0.009625
cg14150907	0.044148	0.004903	0.397535	0.005392
cg02640104	0.043167	0.005019	0.371227	0.004202
cg13765004	0.042726	0.00371	0.492055	0.011448
cg26051755	0.042076	0.002394	0.739405	0.03028
cg05471495	0.041364	0.005435	0.314839	0.002098
cg21052608	0.040909	0.005532	0.302496	0.00174
cg21853021	0.040506	0.006384	0.257023	0.000671
cg00539347	0.039656	0.00509	0.308958	0.002061
cg20979153	0.03939	0.005913	0.262419	0.00083
cg26168881	0.039359	0.003927	0.394432	0.005939
cg27471192	0.038763	0.00331	0.453893	0.00962
cg11318133	0.036105	0.004047	0.322115	0.002934
cg10156217	0.033254	0.002669	0.41428	0.008176
cg06594281	0.031628	0.002265	0.441696	0.010246
cg16973527	0.030947	0.004855	0.197251	0.000235
cg27129922	0.030045	0.002529	0.356924	0.005506
cg15063366	0.029384	0.000902	0.956984	0.047177
cg19310148	0.027474	0.001186	0.636354	0.02497
cg04111435	0.025927	0.001396	0.481679	0.014288
cg07713361	0.022529	0.000888	0.571578	0.021505
cg20799268	0.017981	0.001327	0.243586	0.002511
cg25116269	0.016912	0.00062	0.461636	0.015601
cg21365602	0.002422	3.66E-05	0.160134	0.004855
