# Supplementary material for: CpG Island Methylator Phenotype Modulates the Immune Response of the Tumor Microenvironment and Influences the Prognosis of Pancreatic Cancer Patients
Source: J Oncol. 2021 Nov 28;2021:2715694. doi: 10.1155/2021/2715694 (PMC8645373; doi:10.1155/2021/2715694)
Supplement: Supplementary Materials — Supplementary Figure 1: the most significant 25 hypermethylated CpGs and the most 25 hypomethylated CpGs between 184 PC patients and 10 normal controls by the Wilcoxon test. Supplementary Figure 2: the most significant 25 worse overall survival-related CpGs and the most 25 better overall survival-related CpGs in PC patients by univariate Cox analysis. The hazard ratio (HR) and the 95% confidence interval of the HR were log10-transformed. Supplementary Material 1: it summarizes the differential CpGs between 184 PC patients and 10 normal controls by the Wilcoxon test. In total, 22,450 differential CpGs were identified between 184 PC patients and 10 normal controls (P < 0.05). Among these CpGs, 12,937 were hypermethylated CpGs (log2FC > 0), while 9,513 were hypomethylated CpGs (log2FC < 0). Supplementary Material 2: it summarizes the overall survival- (OS-) related CpGs in PC patients by univariate Cox analysis. In total, 3102 CpGs were found to be related with OS in PC patients (P < 0.05). Among these CpGs, 2858 CpGs were found to be associated with worse OS of PC patients (HR > 1), while 244 were found to be associated with better OS of PC patients (HR < 1). Supplementary Material 3: it summarizes the most OS-related CpGs in PC patients by multivariate Cox analysis. In order to identify the most OS-related CpGs, only 1073 out of 3102 OS-related CpGs with P < 0.01 were used for multivariate Cox analysis, and 72 CpGs were found to be the most OS-related CpGs and they were finally selected for unsupervised consensus clustering analysis. [file 2715694.f1.zip › 2715694.f1/Supplementary material 3 (1).pdf]

The 72 most overall survival-related CpGs identified by multivariate Cox analysis

CpG	Gene	HR	pvalue
cg14356919	COL18A1	1.98E-45	1.02E-06
cg05398036	MIR548F1	2.92E+179	2.15E-07
cg06788267	SLC44A3	9.82E-85	3.08E-06
cg04926881	LOC100506497	4.54E-303	1.32E-07
cg27471192	MPEG1	Infinite	1.94E-07
cg13454226	KLF6	1.10E+184	4.36E-07
cg10536999	NFE2L3	2.99E-28	0.000300232
cg07541559	ABTB2	Infinite	2.08E-07
cg25799109	ARHGEF3	1.02E-42	4.10E-06
cg07317062	HOXA4	7.17E+128	1.04E-06
cg16945312	FOXF2	2.26E+15	1.41E-08
cg22392666	FXYD7	9.57E+29	2.94E-06
cg10659886	ZSCAN18	8.85E+43	0.001576161
cg13371839	ZNF98	6.74E+52	9.66E-05
cg07160746	KCNS1	4.17E-105	2.43E-07
cg08305436	ZNF492	2.20E+276	1.31E-07
cg19125370	TFAP2A	2.36E-223	2.48E-07
cg22605415	FBXO8	2.10E-13	7.62E-05
cg11073773	RNU6-16P	1.15E-92	2.62E-07
cg13944175	HAS1	8.03E-23	0.002209581
cg05678749	GABRG3	1.02E+30	0.000383257
cg11308643	GRIA4	1.30E+111	3.64E-07
cg22131234	VSTM2A	8.17E-102	9.80E-07
cg20266316	NPTX2	4.19E-165	1.52E-07
cg21135135	WBSCR17	6.53E+168	3.40E-07
cg24127719	SIM2	4.21E-30	4.31E-08
cg10175795	TCERG1L	Infinite	2.99E-07
cg15060599	FGD5P1	1.64E-79	1.86E-06
cg17098147	SPAG6	2.91E+39	0.00033391
cg13891978	KCNH5	5.59E+50	1.40E-06
cg18026588	GABRB3	9.44E-42	4.51E-07
cg00498024	B3GNT8	2.09E+302	3.55E-07
cg14290616	FAM83F	Infinite	2.31E-07
cg11936688	LOC401242	2.13E-35	0.000103731
cg07268058	ISL2	3.34E-72	4.72E-07
cg26115633	FOXI2	3.92E+209	2.91E-07
cg20611911	TP73	5.14E+21	2.22E-06
cg00854817	RPH3A	1.19E+225	2.47E-07
cg07104209	EVX1	3.82E-80	4.35E-07
cg16001495	SIM2	1.29E+40	7.67E-06
cg16210842	IGIP	Infinite	3.90E-07
cg20311863	BARHL2	9.52E+139	2.31E-07
cg01381846	HOXA10-HOXA9	1.03E-18	0.000258049
cg03574723	GPR26	Infinite	4.76E-07
cg10824063	NELL1	1.60E-36	7.38E-08
cg10143811	LMO3	2.21E+153	1.75E-07
cg15889913	EBF3	1.69E+94	5.35E-07
cg03111498	VSX1	1.01E-114	8.43E-07
cg21012296	NKX2-2	4.40E+69	7.13E-08
cg03609960	ANKS1B	6.68E+41	1.22E-09
cg17713613	ZNF738	1.27E+26	5.07E-06
cg19326876	ATP10A	2.30E+23	6.41E-06
cg26249873	GPR158	5.29E+92	1.67E-07
cg04415176	HOXD13	1.49E-21	5.18E-06
cg18961681	LOC100130673	1.45E-146	1.52E-07
cg15754548	SLC25A35	5.72E+188	2.57E-07

cg06629130	SKOR2	1.03E+61	1.36E-06
cg06197966	SVIL	2.08E+224	1.06E-06
cg10811045	LOC654342	2.50E-11	0.001135047
cg04475027	TMEM132C	1.07E-48	5.38E-07
cg14473102	HOXD8	1.24E-43	0.000277086
cg25191628	DMRTA2	1.75E-147	2.00E-07
cg02300154	WBSCR17	5.44E+122	2.75E-07
cg14712186	BARHL2	1.19E+49	8.00E-09
cg23217126	DOK6	2.82E+176	7.97E-07
cg06304097	TCERG1L	Infinite	1.53E-07
cg00596508	CNTN1	7.14E-112	7.00E-08
cg24454144	SLC32A1	8.32E+55	1.25E-07
cg26013553	KCNA3	1.42E-81	6.26E-08
cg04904331	VWC2	2.81E-40	9.70E-06
cg01532168	KCNK9	1.72E-136	6.33E-07
cg14353137	TCF24	5.32E+60	1.72E-07
