# Supplementary figures and images for: CpG Island Methylator Phenotype Modulates the Immune Response of the Tumor Microenvironment and Influences the Prognosis of Pancreatic Cancer Patients
Source: J Oncol. 2021 Nov 28;2021:2715694. doi: 10.1155/2021/2715694 (PMC8645373; doi:10.1155/2021/2715694)

Supplementary Figure 1

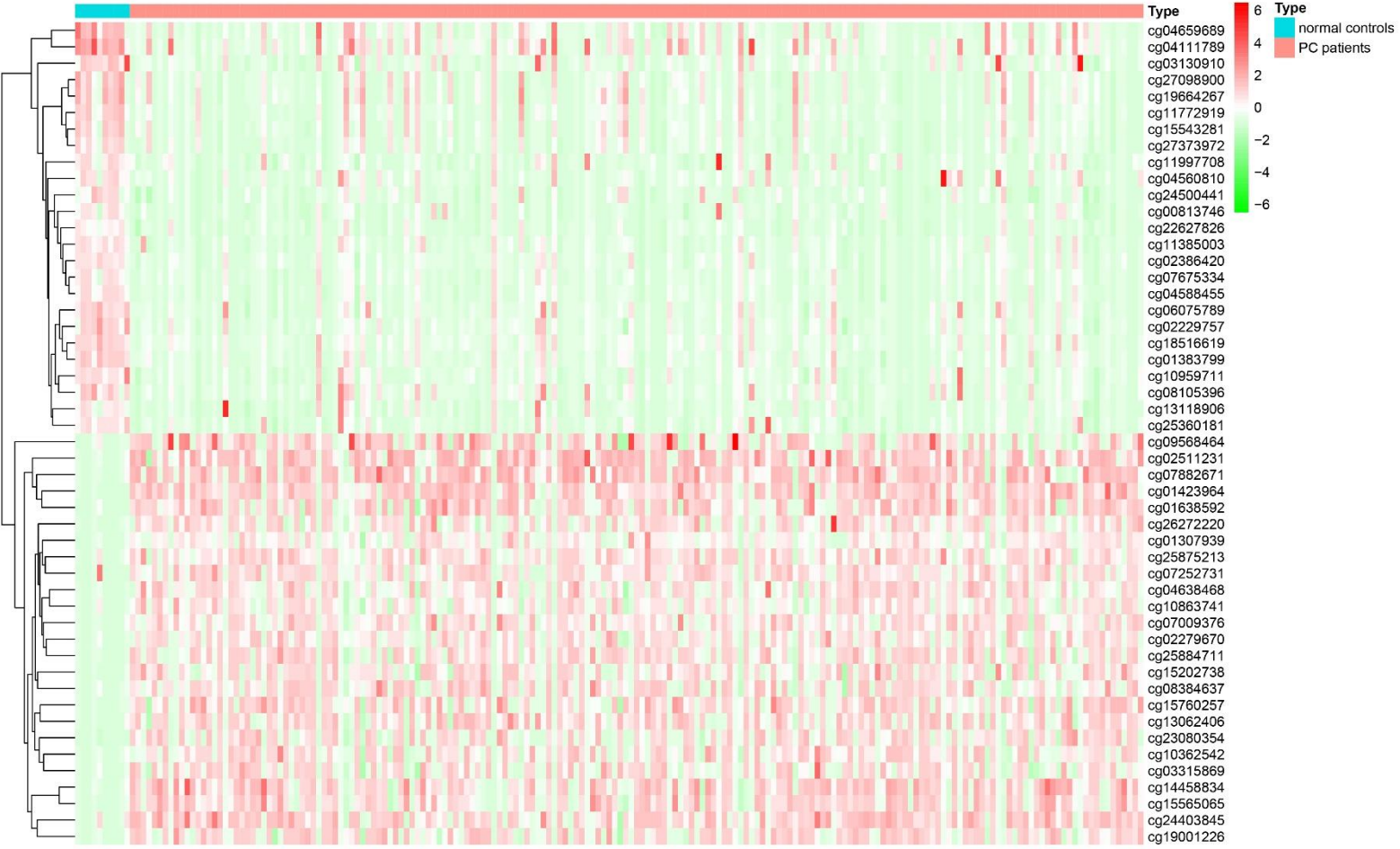

Supplement: Supplementary Materials — Supplementary Figure 1: the most significant 25 hypermethylated CpGs and the most 25 hypomethylated CpGs between 184 PC patients and 10 normal controls by the Wilcoxon test. Supplementary Figure 2: the most significant 25 worse overall survival-related CpGs and the most 25 better overall survival-related CpGs in PC patients by univariate Cox analysis. The hazard ratio (HR) and the 95% confidence interval of the HR were log10-transformed. Supplementary Material 1: it summarizes the differential CpGs between 184 PC patients and 10 normal controls by the Wilcoxon test. In total, 22,450 differential CpGs were identified between 184 PC patients and 10 normal controls (P < 0.05). Among these CpGs, 12,937 were hypermethylated CpGs (log2FC > 0), while 9,513 were hypomethylated CpGs (log2FC < 0). Supplementary Material 2: it summarizes the overall survival- (OS-) related CpGs in PC patients by univariate Cox analysis. In total, 3102 CpGs were found to be related with OS in PC patients (P < 0.05). Among these CpGs, 2858 CpGs were found to be associated with worse OS of PC patients (HR > 1), while 244 were found to be associated with better OS of PC patients (HR < 1). Supplementary Material 3: it summarizes the most OS-related CpGs in PC patients by multivariate Cox analysis. In order to identify the most OS-related CpGs, only 1073 out of 3102 OS-related CpGs with P < 0.01 were used for multivariate Cox analysis, and 72 CpGs were found to be the most OS-related CpGs and they were finally selected for unsupervised consensus clustering analysis. [file 2715694.f1.zip › 2715694.f1/Supplementary Figure 1.pdf]

Supplementary Figure 2

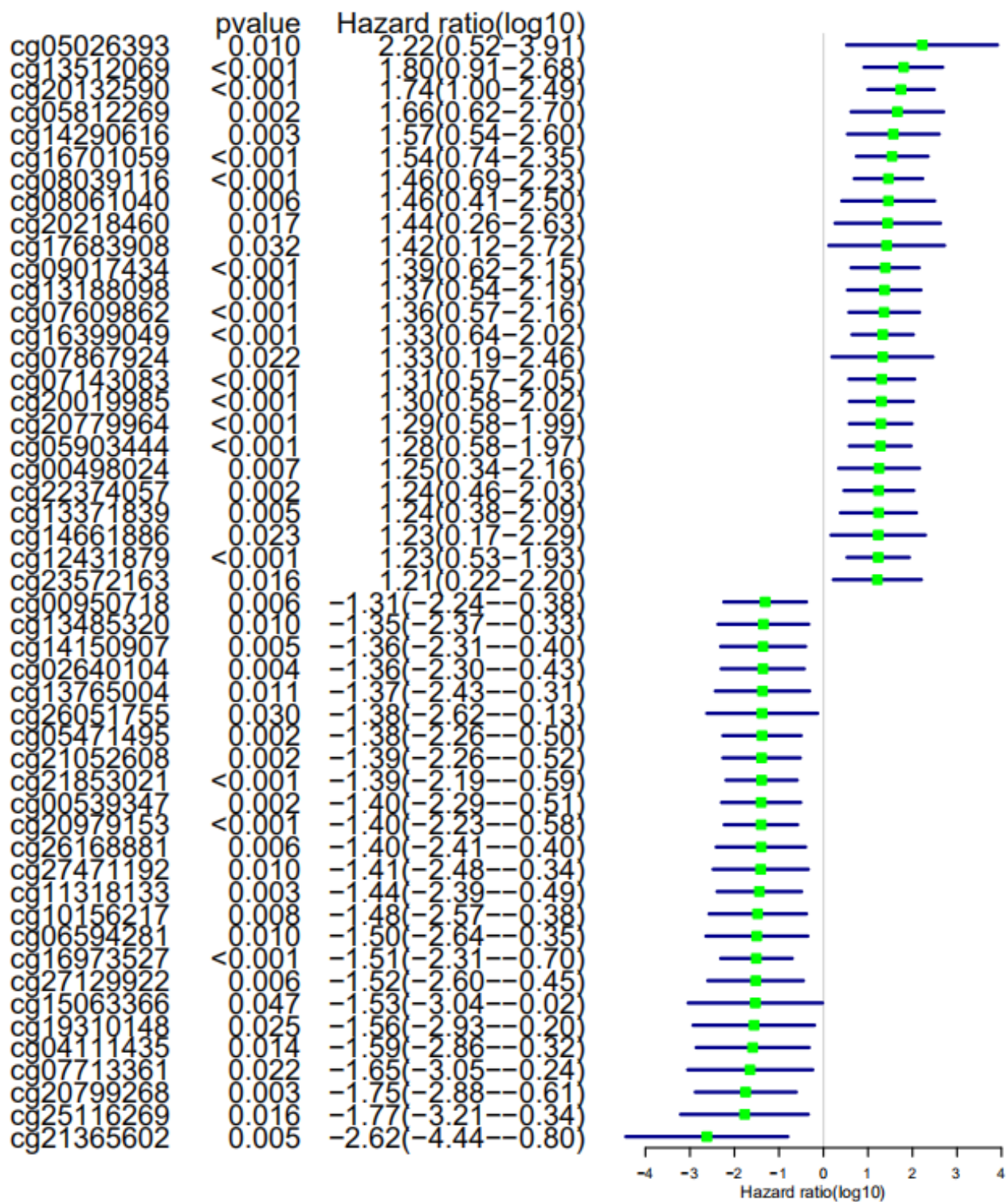

Supplement: Supplementary Materials — Supplementary Figure 1: the most significant 25 hypermethylated CpGs and the most 25 hypomethylated CpGs between 184 PC patients and 10 normal controls by the Wilcoxon test. Supplementary Figure 2: the most significant 25 worse overall survival-related CpGs and the most 25 better overall survival-related CpGs in PC patients by univariate Cox analysis. The hazard ratio (HR) and the 95% confidence interval of the HR were log10-transformed. Supplementary Material 1: it summarizes the differential CpGs between 184 PC patients and 10 normal controls by the Wilcoxon test. In total, 22,450 differential CpGs were identified between 184 PC patients and 10 normal controls (P < 0.05). Among these CpGs, 12,937 were hypermethylated CpGs (log2FC > 0), while 9,513 were hypomethylated CpGs (log2FC < 0). Supplementary Material 2: it summarizes the overall survival- (OS-) related CpGs in PC patients by univariate Cox analysis. In total, 3102 CpGs were found to be related with OS in PC patients (P < 0.05). Among these CpGs, 2858 CpGs were found to be associated with worse OS of PC patients (HR > 1), while 244 were found to be associated with better OS of PC patients (HR < 1). Supplementary Material 3: it summarizes the most OS-related CpGs in PC patients by multivariate Cox analysis. In order to identify the most OS-related CpGs, only 1073 out of 3102 OS-related CpGs with P < 0.01 were used for multivariate Cox analysis, and 72 CpGs were found to be the most OS-related CpGs and they were finally selected for unsupervised consensus clustering analysis. [file 2715694.f1.zip › 2715694.f1/Supplementary Figure 2.pdf]
